# Development and evaluation of a custom bait design based on 469 single-copy protein-coding genes for exon capture of isopods (Philosciidae: *Haloniscus*)

**DOI:** 10.1371/journal.pone.0256861

**Published:** 2021-09-17

**Authors:** Danielle N. Stringer, Terry Bertozzi, Karen Meusemann, Steven Delean, Michelle T. Guzik, Simon M. Tierney, Christoph Mayer, Steven J. B. Cooper, Mohammad Javidkar, Andreas Zwick, Andrew D. Austin

**Affiliations:** 1 Australian Centre for Evolutionary Biology and Biodiversity, School of Biological Sciences, The University of Adelaide, Adelaide, South Australia, Australia; 2 South Australian Museum, Adelaide, South Australia, Australia; 3 Evolutionary Biology and Ecology, Institute for Biology I, University of Freiburg, Freiburg, Germany; 4 Australian National Insect Collection, CSIRO National Research Collections Australia, Acton, Australian Capital Territory, Australia; 5 Center for Molecular Biodiversity Research, Zoological Research Museum Alexander Koenig, Bonn, Germany; 6 School of Biological Sciences and the Environment Institute, The University of Adelaide, Adelaide, South Australia, Australia; 7 Hawkesbury Institute for the Environment, Western Sydney University, Richmond, New South Wales, Australia; Nanjing Agricultural University, CHINA

## Abstract

Transcriptome-based exon capture approaches, along with next-generation sequencing, are allowing for the rapid and cost-effective production of extensive and informative phylogenomic datasets from non-model organisms for phylogenetics and population genetics research. These approaches generally employ a reference genome to infer the intron-exon structure of targeted loci and preferentially select longer exons. However, in the absence of an existing and well-annotated genome, we applied this exon capture method directly, without initially identifying intron-exon boundaries for bait design, to a group of highly diverse *Haloniscus* (Philosciidae), paraplatyarthrid and armadillid isopods, and examined the performance of our methods and bait design for phylogenetic inference. Here, we identified an isopod-specific set of single-copy protein-coding loci, and a custom bait design to capture targeted regions from 469 genes, and analysed the resulting sequence data with a mapping approach and newly-created post-processing scripts. We effectively recovered a large and informative dataset comprising both short (<100 bp) and longer (>300 bp) exons, with high uniformity in sequencing depth. We were also able to successfully capture exon data from up to 16-year-old museum specimens along with more distantly related outgroup taxa, and efficiently pool multiple samples prior to capture. Our well-resolved phylogenies highlight the overall utility of this methodological approach and custom bait design, which offer enormous potential for application to future isopod, as well as broader crustacean, molecular studies.

## Introduction

Phylogenetic and population genetic research on non-model organisms has largely relied on a limited selection of readily available genetic markers to address fundamental, and often difficult, evolutionary questions. Recent molecular studies have, nonetheless, highlighted that a large number of independently evolving loci are often required to produce robust, well-resolved phylogenies, and explore complex phylogenetic and biogeographic scenarios [1–3]. Continual advances and improvements in high-throughput next-generation sequencing (NGS) technologies are now helping to alleviate this issue by enabling the rapid and cost-effective production of substantial molecular datasets for phylogenetic, systematics, and population genetic investigations [4–6]. A variety of approaches are now available to help generate these datasets, with the majority classed as reduced representation sequencing, where sets of preferably orthologous loci (or clusters of orthologous groups) from a subset of the genome are obtained across taxa of interest [7]. Reduced representation approaches include RAD sequencing that targets unspecified loci associated with restriction enzyme sites [8], and those targeting highly specific loci with designed DNA or RNA baits (also termed probes), which are complementary to targeted DNA regions, including ultra-conserved element (UCE) sequencing [9], anchored hybrid enrichment (AHE) [10, 11], and transcriptome-based exon capture [12].

Transcriptome-based exon capture, in particular, uses the transcript sequences assigned to clusters of orthologous groups (OGs) to infer custom baits, which target protein-coding exons across taxa, and is especially useful for generating sequence data from non-model organisms lacking reference genomes [7, 12–18]. This method can further be employed to obtain genomic data from historical museum specimens, which may be critical for phylogenetics and taxonomic research, but typically contain degraded DNA, making it difficult to produce meaningful data using traditional Sanger sequencing techniques [19–22]. Established methods commonly employ a closely related genome to identify intron-exon boundaries and to preferentially distinguish long exon regions (>120 bp) during bait design [7, 12]. Boundary identification, nonetheless, becomes difficult when references are too divergent from the taxon of interest due to issues associated with aligning exons and since intron-exon structure may not be preserved in distantly related species [23].

In these instances, transcriptome sequences can be used directly to infer orthologous loci and design baits, precluding the need to differentiate exons using a genome reference *a priori*. A study by Portik et al. [24] effectively employed this transcriptome-based exon capture method to generate a large and informative phylogenomic dataset across divergent frog lineages. This approach allowed for exons of various lengths to be captured (because bait tiling may span multiple short exons), together with highly variable non-coding flanking sequences. Nevertheless, very few studies have used this direct approach to target exons without a reference genome, and further baseline information and empirical data, as well as detailed and reproducible bioinformatic methods, are fundamental for the successful design of future capture experiments [25]. In this study, we examine the performance and efficiency of transcriptome-based exon capture for the non-model isopod genus *Haloniscus* Chilton, 1920 [26], and the application of our bait design across more distantly related outgroup isopod taxa for phylogenetic inference. Orthologous gene and bait sets with broad taxonomic applicability are of major interest in phylogenetics, particularly as this promotes consistency and comparison across multiple studies [11, 18, 27].

Earlier phylogenetic studies on *Haloniscus* from Australian groundwater-dependent ecosystems have revealed extensive species diversity and short-range endemism, and have further proposed that this genus, which contains epigean and subterranean lineages, represents a relictual group with a complex evolutionary history [28–30]. However, molecular datasets have been limited to either a single mitochondrial (*cytochrome c oxidase subunit I*: *COI*) or two genes (*COI* and *18S rRNA*), resulting in poor topological resolution. Therefore, questions regarding the origins and biogeographic history of the genus remain unresolved and require further investigation with additional independent markers. The selection of a larger orthologous gene set and development of a custom bait design targeting *Haloniscus*, as well as more distantly related isopods, will allow for a better understanding of their evolution and the relationships among species.

We, therefore, aimed to produce an effective and thorough methodological phylogenomic framework for: inferring an isopod-specific set of single-copy protein-coding OGs from sequenced transcriptomes, developing a custom bait design to capture exons from a large number of loci (>400), conducting all laboratory-based protocols, and for processing the resulting sequence data using a mapping approach. Due to the absence of an existing closely related, and well-annotated reference genome, we used RNA transcript sequences for bait design without first predicting exon boundaries. The overall success and efficacy of our bait design was evaluated by determining the: i) number and length of exons, ii) sequencing depth (or coverage per base) for targeted exons, iii) percentage of missing data across exons, and iv) utility of the baits across more distantly related outgroup taxa, comparing the results of analyses using the mapping approach to those generated with an assembly pipeline. We further aimed to assess the influence of preserved specimen age and pool sizes (prior to capture and sequencing) on depth of coverage as well as the effect of pooling sizes on raw sequencing yield and PCR duplication. The successful inclusion of older or poorly preserved museum specimens in molecular phylogenetic analyses is of significant interest, particularly since these taxa may be rare, difficult to collect or now extinct [22]. Pooling multiple samples may, furthermore, help to reduce costs without decreasing capture efficiency [24]. Finally, we provide our transcriptome assemblies, final OGs, bait design, concatenated alignments, and post-processing scripts for a completely reproducible framework, without the need for outsourcing to external providers.

## Materials and methods

**Fig 1:**
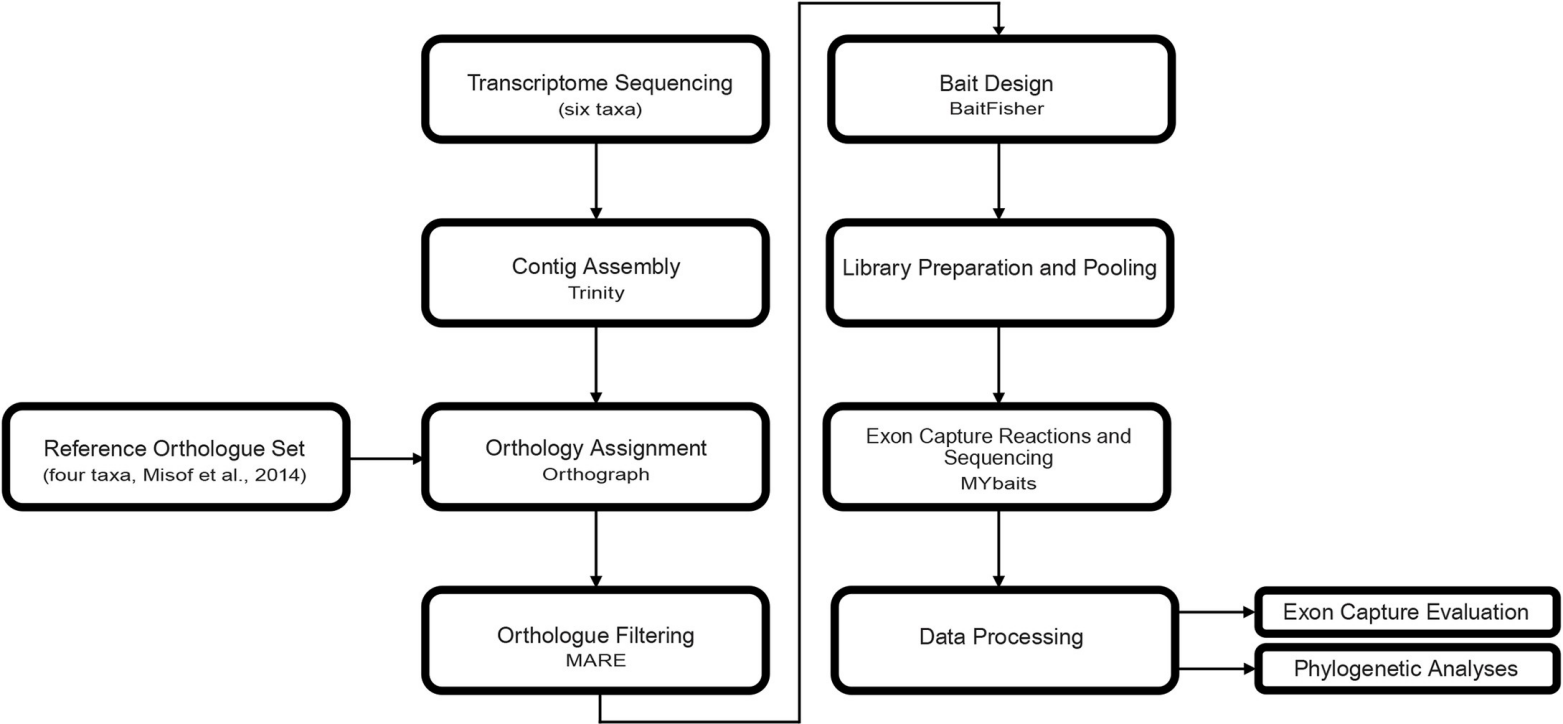
Schematic overview detailing the methodological framework used for orthology assignment, bait design and sequence data generation.

The methodological pipeline for this study comprised eight steps (Fig 1): (1) transcriptome sequencing; (2) contig assembly; (3) transcript assignment to OGs and verification of their orthology; (4) assessment of putative phylogenetic informativeness of OGs and final selection of OGs for downstream analyses; (5) bait design; (6) DNA extraction of preserved specimens, library preparation, and pooling; (7) exon capture reactions and sequencing; and (8) data processing, data evaluation, and phylogenetic analyses. Custom Perl and Linux shell scripts were created for the data processing, and are available on Bitbucket (https://bitbucket.org/tbertozzi/scripts) and Figshare (doi: 10.25909/5ef570329cbdd).

### Transcriptome sequencing and contig assembly

Six isopod species were selected for transcriptome sequencing, including one undescribed *Haloniscus* species (Philosciidae), and five additional species: *Porcellionides pruinosus* (Brandt, 1833) (Porcellionidae), *Paraplatyarthrus* sp. (Paraplatyarthridae), *Paraplatyarthrus subterraneus* Javidkar & King, 2015, *Ceratothoa* sp. (Cymothoidae), and *Armadillidium vulgare* (Latreille, 1804) (Armadillidiidae) (see S1 Table for details). Specimens were preserved in RNAlater (Qiagen) and total RNA was extracted from whole isopod bodies with an RNeasy Plus Micro or Mini Kit (Qiagen) according to the standard protocol for tissue extraction. RNA (pooled from multiple specimens in some cases due to very small individuals, S1 Table) was quantified using a Quantus Fluorometer (Promega). Double-stranded cDNA was synthesised and subsequently PCR-amplified using SMARTer cDNA Synthesis and Advantage 2 PCR Kits (Clontech), with PCR optimisation procedures verified using agarose gels. cDNA libraries were sent to the Australian Genome Research Facility (AGRF) in Adelaide, South Australia for *Paraplatyarthrus* sp., *P*. *subterraneus*, *P*. *pruinosus*, and *Haloniscus* sp., and to GATC (Eurofins Genomics) in Constance, Germany for *A*. *vulgare* and *Ceratothoa* sp. to be sequenced on the Illumina HiSeq2000 platform with TruSeq adapters, generating 100 bp paired-end reads.

For the *Haloniscus* sp. assembly (conducted by DNS), raw RNA-seq reads were quality controlled with FastQC v0.11.4 [31], and transcripts were filtered and trimmed in Cutadapt v1.1 [32] to remove low quality reads (Phred scores <30), Illumina TruSeq barcoded adapters, SMARTer adapters, poly-A tails, and sequences less than 25 bp following trimming. Reads were then *de novo* assembled using Trinity v2013-08-14 [33, 34] with default settings. Assembled contigs were quality assessed using Bowtie [35], which aligned contigs back against raw reads to specify the proportion of proper paired reads obtained. The five remaining isopod transcriptomes were contributed to this project as part of a collaboration and were processed by authors TB and AZ using the methods detailed in S1 File.

### Orthology assignment

We targeted single-copy protein-coding OGs, as recognised by The Hierarchical Catalogue of Orthologs (OrthoDB: http://www.orthodb.org/), from an earlier published orthologue set based on 12 reference arthropod species [36]. This reference set, built on OrthoDB v5.0 [37], consisted of 1,478 OGs from the crustacean *Daphnia pulex* Leydig, 1860, the arachnid *Ixodes scapularis* Say, 1821, and 10 hexapod taxa where complete genomes and official gene sets were readily available. We optimised this reference set by reducing the number of species to four (*D*. *pulex*, *I*. *scapularis*, the red flour beetle *Tribolium castaneum* (Herbst, 1797), and the termite *Zootermopsis nevadensis* Hagen, 1853), removing the remaining hexapods to lessen the bias towards insect taxa.

We inferred gene orthology in our six *de novo* sequenced and assembled isopod transcriptome libraries with a pre-release version of Orthograph (beta4.1 available at https://mptrsen.github.io/Orthograph/) [38] that assigned transcripts to OGs based on the reference set of four species detailed above. We used default parameters except for the following: maximum number of blast searches and blast hits = 50, minimum transcript length = 25, and also enabled the extension of the open reading frame (ORF) with a minimum overlap of 30% (extend-orf = 1 and orf-overlap-minimum = 0.3). Transcript sequences, where orthologue criteria were fulfilled, were summarised into individual files for each distinct OG, and two reference species, *T*. *castaneum* and *I*. *scapularis*, were removed since the majority of best reciprocal hits were to *D*. *pulex* and *Z*. *nevadensis*. Internal stop codons, along with Selenocysteine “U”, were masked with “X” (and “NNN” on a nucleotide level) using a custom Perl script (provided with the Orthograph package). For downstream analyses, we only included OGs that contained hits to all six transcriptomes, totalling 531 single-copy protein-coding genes. Amino acid sequences for each OG were aligned with MAFFT v.7.220 [39], and equivalent nucleotide multiple sequence alignments were inferred using a modified version of PAL2NAL v14.1 [40] (see [36] for further details on the modification) with the amino acid alignments as blueprints.

### Assessing phylogenetic informativeness of OGs

We applied the matrix reduction program MARE v0.1.2-rc [41, 42] to determine putative phylogenetic informativeness of all 531 OGs, and then assessed which OGs showed the highest information content (IC). MARE uses extended geometry quartet mapping to infer informativeness (or “tree-likeness”: the number of resolved quartets divided by the number of quartets drawn for each partition, i.e. for each OG in this study) from amino acid alignments within a user-provided supermatrix. The analysis yielded a reduced (optimised) matrix, but with an increased overall IC. Here, we retained OGs with an IC >0.5 (479 OGs in total) for use in downstream analyses.

### Bait design

The dataset, comprising 479 aligned OGs each with all eight reference species (the six transcriptomes, along with *D*. *pulex* and *Z*. *nevadensis*) on a nucleotide level, was used to design baits for targeted exon capture with the software BaitFisher v1.2.7 [27]. We specified a bait length of 120 bp and tiling design of seven baits spanning a total region of 300 bp, with a bait offset every 30 bp. The clustering threshold was set to 0.15. BaitFisher removed 10 OGs that were not suitable for bait design since the sequences were either too short (<300 bp) or an appropriate bait region could not be detected as the region likely consisted of too many gaps or Ns and the software could not place a full bait within the OG alignment. BaitFisher permits inclusion of a reference genome and annotation file to infer known exons; however, due to the absence of a related isopod genome at the time of design, we generated the baits directly from our multiple sequence alignments. The bait design was then optimised with BaitFilter v1.0.4 [27], following the method that maximises the number of sequences baits are inferred from. This process resulted in 15,053 baits for 24,258 sequences (37.95% of baits saved, with respect to a bait design where baits are constructed for each gene and species of interest, rather than for each cluster of similar gene sequences). Custom RNA baits were manufactured by Arbor Biosciences (Ann Arbor, MI, U.S.A.) for use with a MYbaits v3 kit.

### Library preparation and pooling

Genomic DNA was extracted from 36 whole specimens using the Gentra Puregene DNA Purification protocol (Gentra Systems Inc.) according to the manufacturer’s protocol. The specimens included 31 *Haloniscus* taxa, representing the majority of known lineages as inferred by *COI* mtDNA data [28, 30] with *H*. *anophthalmus* unlikely to belong to the genus, and five outgroup isopod species (S2 Table). An additional *Haloniscus* sample from Windimurra (Western Australia) was included using pooled DNA extracts from three different individuals (see S2 Table). DNA was quantified by Quantus Fluorometer (Promega) with the QuantiFlour dsDNA System Kit (Promega), and each sample was diluted to 1–10 ng/μL (reliant on initial DNA concentration) in 100 μL of molecular grade water. A Bioruptor Pico (Diagenode) was utilised to shear the DNA for 1–4 min using 30 s on/30 s off cycling. Each sonicated sample was analysed by electrophoresis on an Agilent 2200 TapeStation (Agilent Technologies) to determine whether fragments were appropriately sized (average size of 300–500 bp) for later sequencing. For samples exhibiting a broad fragment size distribution, a size selection step was completed with the SPRI bead method and polyethylene glycol to remove fragments less than 150 bp (protocol outlined by Li et al. [43]).

Genomic libraries were then prepared following Meyer and Kircher [44], with some modifications to the indexing PCR reaction. A unique combination of i7 and i5 indexes (1–10 from Meyer and Kircher [44], and 1–23 from Glenn et al. [45]) was added to each library in 25 μL reactions consisting of 1X KAPA HiFi Taq Ready Mix, 0.2 μM of each indexing primer, and 10 μL of library (the remaining library retained as a back-up). Thermal cycling conditions involved an initial denaturation step at 98 ˚C for 45 s, then 18 cycles at 98 ˚C for 15 s, an annealing temperature of 65 ˚C for 30 s, and an elongation of 72 ˚C for 60 s, followed by a final elongation phase at 72 ˚C for 10 min. Libraries were purified and the concentrations were measured with a Qubit Fluorometer (Life Technologies) and qPCR amplification (KAPA Library Quantification Kit, Illumina). The resulting 25 μL of amplified library had a concentration of at least 10 ng/μL, but the final results varied extensively due to starting concentration and sample quality.

These library preparation steps were completed across three rounds. In the first round, we optimised the number of libraries that could be pooled together in a single in-solution capture reaction by testing four different pooling strategies, with capture pools containing either one, two, three or four libraries (totalling 10 libraries). Three separate libraries, each with distinct dual indexes, were created using one sample (ID: BES18659, libraries: 27813, 27814, and 27815) and allocated across three pools of different sizes (see results for further details) to determine whether increasing the number of libraries that were pooled would negatively influence the final number of reads obtained. It was found that pooling four libraries prior to capture did not influence the number of reads and, as such, we created two pools of four libraries for the second round of exon capture (with one sample GAB00764.1 repeated across two libraries), and four pools of four libraries and two pools with three libraries for the third. This equated to 40 libraries in total across the three distinct rounds of library preparation prior to exon capture.

### Exon capture reactions and sequencing

Pooled libraries were concentrated down to 7 μL using a CentriVap DNA Concentrator (Labconco) for sequence capture. MYbaits capture reactions were performed following the v3.01 manual, with heat-denatured concentrated library pools combined with the designed baits and universal blocking oligos (included in the MYbaits kit), and hybridised for approximately 16–20 hrs at 65 ˚C. Reactions were then purified using Dynabeads MyOne Streptavidin C1 beads (Life Technologies) and post-capture products were amplified using KAPA HiFi DNA Polymerase (Kapa Biosystems) with the following protocol: 98 ˚C for 2 min, followed by 12 cycles at 98 ˚C for 20 s, 60 ˚C for 30 s, and 72 ˚C for 30 s, and a final extension of 5 min at 72 ˚C. Pools were purified with 90 μL of AMPure XP beads (Agencourt), resuspended in 30 μL of elution buffer, and quantified with the Qubit Fluorometer (Life Technologies) and/or a standard quantitative PCR run with the LightCycler 96 Real-Time PCR System (Roche Diagnostics) for equimolar pooling. The fragment size distribution for each pool was, additionally, visualised on the TapeStation. Following the first round of capture, the four pools were combined in equimolar ratios and sequenced on the Illumina MiSeq platform with 300 bp paired-end reads. For the second and third capture rounds, equimolar pools were also sequenced on the Illumina MiSeq platform, but instead with 150 bp paired-end reads due to the low average fragment size (<300 bp) in the final pools. Illumina sequencing of the captured DNA libraries was conducted by AGRF (Adelaide, South Australia).

### Exon capture data processing

Raw sequence reads were quality tested using FastQC v0.11.4 [31], and filtered with the BBDuk v35.92 software package, BBTools (https://sourceforge.net/projects/bbmap/files), by trimming adapters and removing low quality reads. Overlapping paired reads were merged using PEAR v0.9.10 [46] to avoid inflated coverage estimates. For each sequenced library, cleaned reads were mapped to the *Haloniscus* transcript OGs used in bait design with BWA v.0.7.15 [47] and SAMtools v1.3.1 [48]. The *Haloniscus* targeted OGs were concatenated into one continuous sequence of all 469 targets, each separated by a string of 1000 Ns using a custom script: catFasta.pl. The script provides the option of simultaneously creating a BED4 file, which defines the start and end position of each target, the target sequence length, and the name of each target. Output BAM files from the mapping step were assessed with the Integrative Genomics Viewer (IGV) [49]. Since an annotated reference genome was not used during bait design to separate intron-exon boundaries, the *Haloniscus* reference OGs were split into exon targets manually based on the BAM alignments (see S1 Fig for example) to reflect the boundaries. Reads were then mapped once again, but this time to the revised reference, similarly generated with the custom concatenation script, and duplicate reads were removed with Picard tools v2.2.4 (http://broadinstitute.github.io/picard/). Sequencing depth (or coverage per base) files were produced with BEDTools v2.25.0 [50].

Variant calling was performed using FreeBayes v1.0.2 [51] after initially trialling SAMtools v1.3.1 with BCFtools v1.4.1 [48] and HaplotypeCaller in GATK v.3.7 [52]. BCFtools frequently reported lower values for variant sequencing depth than expected, i.e. differing from those calculated with BEDTools and as seen in alignments with IGV, which led to issues in later steps when filtering based on depth. Whereas, the GATK variant caller provided higher depth results, but the VCF file contained much fewer variants, even after adjusting parameters. Overall, VCF files produced by FreeBayes included expected variant numbers and sequencing depth values to those within the BAM alignments. Complex polymorphisms in FreeBayes output files were decomposed to individual SNP and indel calls using vcfallelicprimitives in vcflib (https://github.com/vcflib/vcflib) and variants with <10x coverage (or sequencing depth) were removed with vcffilter (vcflib). Heterozygous calls were filtered with filterVCF.pl based on a minimum minor allele frequency of 0.2. Consensus sequences were generated with the script, applyVariants.pl. The script produced consensus sequences inferred using the mapped reads by applying variants in a VCF file to the *Haloniscus* reference sequence used in mapping. The script also masked bases with <10x (user-defined) coverage based on the ‘per base’ coverage files generated with BEDTools, and included IUPAC ambiguity codes for heterozygous sites.

A custom Perl script, groupTargets.pl, then used the fasta files created with applyVariants.pl to group the same target sequence from different isopod sequenced libraries into individual files ready for alignment. The Bash script, runMuscle.sh, and MUSCLE v3.8.31 [53] were used to align the sequences in each target file. Potential paralogous loci and messy alignments were identified and removed based on an elevated proportion of heterozygous sites (>3%) with the custom script, FilterMergedLoci.pl, and also checked manually. With this script, sequences with an excess of variable sites were replaced with a string of Ns equal in length to the original sequence.

### Exon capture evaluation

To examine capture efficiency, sequencing depth (coverage per base) values were calculated for each position along the mapped transcripts for all sequenced libraries with BEDTools (discussed above). The per base pair coverage estimates corresponding to each of the 469 target OGs were plotted for all 40 sequenced libraries, with intron-exon boundaries delineated by vertical lines. Using these outputs, the median sequencing depth values were calculated across the sequenced libraries for all exons within each OG, and separated according to sequencing batch (runs 1–3). Results were summarised for the ‘targeted’ exons, chosen from exons with the highest median sequencing depths from 50 randomly-selected OGs. Average differences in median sequencing depth between runs were estimated using a generalised linear mixed model fitted to the data on a log link scale and negative binomial variance distribution. Individual exon and sequencing identifiers were integrated as random effects to account for average differences in sequencing depth values among each of these factors. Marginal mean sequencing depth (with 95% confidence intervals) was estimated for each run, and contrasts were used to infer differences in sequencing depth across the runs.

Differences in median sequencing depth values for exons of a random subset of 50 targeted OGs were examined. Pool sizes (with 1–4 libraries) prior to exon capture, specimen preservation age (number of years since collected and preserved in 100% ethanol), raw paired-end data yield, and the percentage of missing data across exons within the concatenated alignment (dataset B, see below for details) were used as additional covariates. The percentage of PCR duplication amongst reads for each sequenced library was similarly examined against pool size prior to capture, with the amount of duplication calculated by dividing the number of duplicate reads (obtained with Picard tools as described previously) by the total number of raw paired-end reads (as filtered reads were merged using PEAR). We did not formally test for the relationships with specimen preservation age or pooling sizes prior to capture. The distribution of specimen preservation ages varied considerably between runs, sometimes over a short range, and, for some runs, a single sample differed substantially in age from the remaining samples in that run. For pool sizes, some runs consisted of merely one or two different pooling selections, while additional runs comprised pools with a larger range of sizes.

The exon sequencing depth uniformity across sequenced libraries, and among sequencing runs, was assessed by calculating the median absolute deviation and robust coefficient of dispersion (the median absolute deviation divided by the median) for targeted exons from the 50 randomly-selected OGs, and plotting the results. This calculation examines the amount of variation in sequencing depth across bases within exons. All calculations and analyses for capture evaluation detailed in this section were conducted using R v3.6.0 [54] (script available on Figshare at doi:10.25909/5ef570329cbdd).

### Phylogenetic analyses

The target alignments discussed previously were concatenated using a custom script, catAlignedLoci.pl, for phylogenetic analysis. The concatenation order was based on the BED4 candidate file, which was produced with the artificial reference prior to mapping. The script created a “candidate partition” file, including the target/exon boundaries, and further allowed for a threshold to be indicated to filter out targets that contained too many missing sequences (i.e. all Ns). Three distinct datasets were produced, each with differing thresholds for missing data: the first with a threshold of 25 (dataset A), which only included exon alignments with up to 25% of sequences missing entirely, the second with a threshold of 50 (dataset B), and the third dataset with a threshold of 75 (dataset C), which included exons from taxa with up to 75% of sequences missing. We employed PartitionFinder v2.1.1 [55] to determine suitable partitioning schemes for the three different datasets with the rcluster algorithm [56] (parameters: models = all, model_selection = aicc, branchlengths = linked, rcluster-percent = 10.0, rcluster-max = 1000, raxml), and the exon partition files discussed above. Maximum likelihood (ML) phylogenies were then inferred with RAxML v8.2.10 [57] using the deduced partitions, the GTRGAMMA model using the standard four rate categories, and 1,000 rapid bootstrap inferences (all remaining settings as default). The phylogenies were rooted using the six outgroup taxa, and Figtree v1.4.2 [58] was used to visualise the phylogenies. PartitionFinder and RAxML were run on the CIPRES Science Gateway v3.3 [59].

### HybPiper assembly comparison

To assess the efficacy of our mapping approach (see the ‘Exon capture data processing’ section above), we ran our capture data through a pre-existing assembly pipeline, HybPiper v1.3.1 [60], which uses a suite of Python scripts to wrap and connect bioinformatics tools to extract target sequences from high-throughput sequencing reads. HybPiper assigned our cleaned sequencing reads to target OGs within a target file, which contained all 469 OGs with sequences from the eight reference taxa used in orthology prediction (contrasting the use of only the *Haloniscus* reference OGs in the mapping approach detailed above), using BLASTx v2.7.1 [61]. HybPiper subsequently assembled OGs with SPAdes v3.12.0 [62] and used Exonerate v2.4.0 [63] to align contigs to the target file sequences. Sequences for all 40 sequenced libraries were then retrieved for each target, with HybPiper generating an unaligned fasta file for each OG. Potential paralogs, as indicated by the presence of multiple long contigs, were identified by using HybPiper scripts, and removed from the analysis. The Bash script, runMuscle.sh, together with MUSCLE v3.8.31 [53] were used to align the sequences in each OG file, and HybPiper was used to concatenate the targets into a single long sequence for each sequenced library (inserting gaps for missing OGs) for phylogenetic inference. Summary statistics were obtained with HybPiper scripts. Phylogenetic analysis of the output concatenated dataset (D) was then conducted with PartitionFinder and RAxML using the methods detailed above.

### Ethics statement

Specimen collection in Australian National Parks was completed under: Northern Territory Permit No. 54946 (‘Permit to Take Wildlife for Commercial Purposes’, approved by Parks and Wildlife Commission Northern Territory), South Australian Permit No. Z25519 (‘Permit to Undertake Scientific Research’, approved by The Government of South Australia, The Department for Environment and Heritage), and Western Australian Permit No. SF0009792 (‘Licence to Take Fauna for Scientific Purposes’, approved by The Government of Western Australia, Department of Parks and Wildlife). Additional approval was obtained for sample collection across sites in the Northern Territory at Newhaven Sanctuary from the Australian Wildlife Conservancy National Science and Conservation Manager, John Kanowski, and Central Mt Wedge from the Central Land Council and Traditional Owners (‘Special Purposes Permit’, with involvement from Jeff Hulcombe and the Aṉangu Luritjiku Rangers). For field collection at South Australian Great Artesian Basin springs, general permission was obtained to conduct fieldwork across private land from Greg Campbell (Chief Executive) of S. Kidman & Co Pty Ltd, and culturally sensitive land from Traditional Owner representatives, Reg Dodd and Dean Ah Chee. Additionally, many station managers and mining officers permitted access to perform fieldwork on private property. None of the fieldwork conducted for the purpose of this study involved collecting protected species.

## Results

### Transcriptomes, orthology and bait design

An average of 24.1 million (M) (21.3–27.9M) paired-end reads were sequenced for each transcriptome library, which assembled into approximately 6.2 × 10^4^ contigs (4.0 x 10^4^–1.37 x 10^5^) (Table 1). The larger contig values acquired for *A*. *vulgare* and *Ceratothoa* sp. (Table 1), despite the comparable number of paired-end raw reads to the other transcriptomes, can be explained by the inclusion of many short contigs by IDBA-Tran, which remain ambiguous and are unlikely to be true transcripts. Searching for 1,478 single-copy OGs, similar numbers were inferred amongst our isopod transcriptomes (806–1272, see Table 1). The six assembled transcriptomes, along with the results from the MARE filtering analysis, our 469 OG alignments, and the bait design are available on Figshare at doi:10.25909/5ef570329cbdd.

**Table 1:** Summary statistics for sequencing and *de novo* assembly of the isopod transcriptomes used in orthology assignment.

Isopod species	Pairs of raw reads	*De novo* assembled transcripts	Number of identified OGs
*Haloniscus* sp.	21,354,576	43,455	942
*Paraplatyarthrus* sp.	24,795,047	40,461	895
*Paraplatyarthrus subterraneus*	21,896,830	46,114	1,011
*Porcellionides pruinosus*	23,941,206	37,368	806
*Armadillidium vulgare*	27,943,392	66,407	1,272
*Ceratothoa* sp.	24,786,465	137,713	1,260

### Exon capture data

An average of 833,844 (73,241–2,898,504) paired-end reads were sequenced for all 40 libraries, with run 1 (10 pooled libraries) averaging 1,656,743 (764,956–2,898,504) paired-end reads, run 2 (8 pooled libraries) averaging 1,261,684 (747,169–2,058,751) paired-end reads, and run 3 (22 pooled libraries, including outgroup taxa) averaging 304,221 (73,241–717,722) paired-end reads (see Table 2). Following the removal of low-quality reads and adapters, the percentage of retained clean paired-end reads ranged from ~92–98.5% (Table 2). Mapping the cleaned reads directly to the *Haloniscus* sp. transcript OGs employed for bait design revealed many exon sequences of various lengths, and their associated non-coding flanking sequences (e.g. S2 Fig). Since the bait region was constrained to a length of 300 bp, the complete coding sequence for each target OG was not generally captured (see S3 Fig). Nevertheless, approximately 1,150 exons (median: 798 exons across libraries) were captured across all targeted OGs and sequenced libraries, with a median of two exons captured per target OG (range: 1–4 exons). Only nine OGs were not captured across any sequenced libraries. The length of exons captured varied substantially, ranging from 15–2,013 bp, with a median length of 153 bp for individual captured exons (S4 Fig).

**Table 2:** Exon capture sample, mapping and sequencing run statistics.

Sample ID	Sequencing library ID	Run	Ingroup or outgroup	Raw paired-end reads	Clean paired-end reads retained (%)	Duplication (%)	Missing data (%)	Preservation age (years)	Pool size/capture	Libraries/run
BES18775	27809	1	Ingroup	1,152,943	95.4	11.2	4.6	0.5	3	10
BES18774	27810	1	Ingroup	820,103	95.8	7.1	4.4	0.5	4	10
BES6573	27811	1	Ingroup	764,956	95.8	6.5	15.5	15.0	2	10
BES18645	27812	1	Ingroup	2,267,263	95.6	18.8	2.7	1.9	4	10
BES18659	27813	1	Ingroup	2,898,504	95.6	17.8	2.8	1.9	2	10
BES18659	27814	1	Ingroup	2,305,287	95.7	17.4	2.8	1.9	4	10
BES18659	27815	1	Ingroup	1,285,005	95.6	9.8	3.7	1.9	1	10
BES18601	27816	1	Ingroup	2,678,874	95.8	19.4	2.8	1.9	3	10
BES18754	27817	1	Ingroup	963,373	95.7	3.5	7.4	0.5	3	10
BES18644	27818	1	Ingroup	1,431,124	96.1	15.6	3.6	1.9	4	10
BES16434	28076	2	Ingroup	747,169	97.0	9.1	5.9	4.8	4	8
GAB01433	28077	2	Ingroup	875,801	97.3	20.0	7.9	7.1	4	8
GAB01616	28078	2	Ingroup	897,834	97.7	20.2	5.3	7.1	4	8
GAB00736	28079	2	Ingroup	2,058,751	94.3	29.0	4.5	8.8	4	8
GAB00764.1	28080	2	Ingroup	1,574,884	97.2	17.7	2.8	8.8	4	8
BES17062	28081	2	Ingroup	1,207,963	97.4	9.9	3.9	4.2	4	8
GAB01007.1	28082	2	Ingroup	1,696,723	97.3	24.0	3.7	8.0	4	8
GAB00764.1	28083	2	Ingroup	1,034,347	97.6	14.0	3.8	8.8	4	8
BES18773	1	3	Ingroup	282,187	96.9	0.8	37.5	1.9	4	22
BES18759.3	2	3	Ingroup	73,241	91.9	0.5	86.1	1.9	4	22
BES6655	3	3	Ingroup	135,325	94.2	1.4	83.7	16.2	4	22
BES16348	4	3	Ingroup	246,978	98.1	4.8	29.0	5.8	3	22
BES8623.1	5	3	Ingroup	230,473	95.2	0.6	70.0	15.9	4	22
BES16400.2	6	3	Outgroup	374,913	96.3	0.1	71.8	5.8	4	22
BES13246	7	3	Ingroup	620,284	95.9	0.7	19.4	10.2	4	22
BES13396	8	3	Ingroup	271,685	93.7	1.2	19.0	10.2	4	22
BES14385	9	3	Ingroup	109,362	98.4	0.7	52.8	10.2	4	22
BES13314	10	3	Ingroup	280,826	98.5	1.2	21.2	10.2	4	22
GAB00795	11	3	Ingroup	391,058	98.0	2.9	12.4	9.8	4	22
GAB00765	12	3	Ingroup	332,292	97.7	4.3	11.2	9.8	3	22
BES10201	13	3	Outgroup	89,157	98.4	0.1	99.4	14.2	4	22
BES6601.2	15	3	Ingroup	376,860	94.5	0.6	98.1	16.2	4	22
BES10410	16	3	Ingroup	509,729	92.1	1.8	40.5	13.2	4	22
BES6667.2	17	3	Ingroup	310,648	96.5	0.2	97.1	16.1	4	22
BES13452	18	3	Ingroup	382,589	97.8	0.2	44.6	10.2	4	22
BES8956, BES13133.1, BES13133.2	19	3	Ingroup	122,173	97.8	3.8	70.4	15.9	3	22
Ja243	20	3	Outgroup	346,847	98.3	0.6	66.8	6.4	3	22
B002	21	3	Outgroup	249,046	96.6	0.1	89.0	1.0	3	22
BES15525.10	22	3	Outgroup	239,462	98.4	1.3	38.8	7.0	4	22
BES15537.2	23	3	Outgroup	717,722	98.5	2.4	48.6	7.1	3	22

Note: sequencing of samples BES18659 and GAB00764.1 was repeated using multiple libraries prior to exon capture.

### Sequencing depth, duplication and missing data

**Fig 2:**
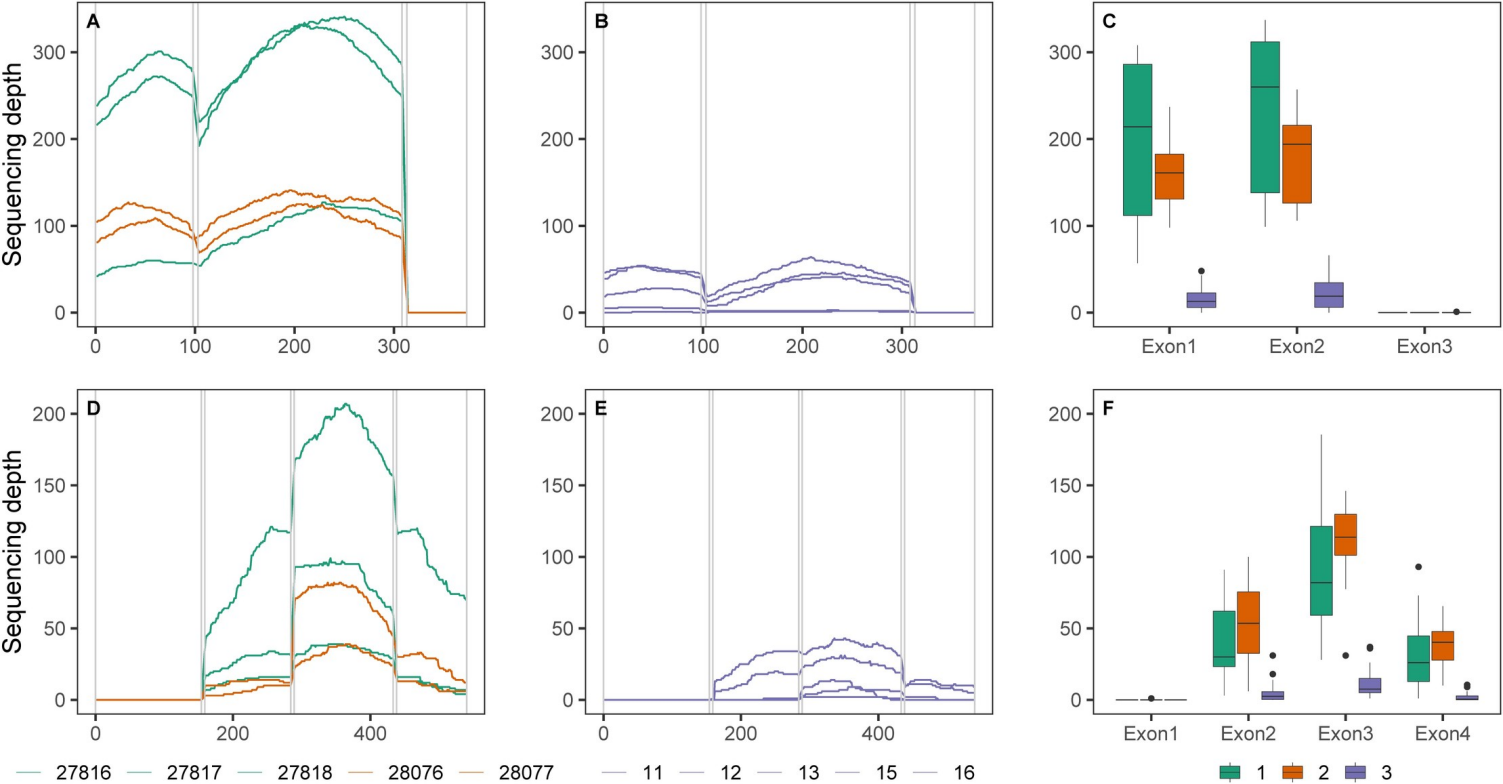
Example plots of sequencing depth per base across orthologous groups (OGs): EOG54MW8B (3 exons; upper row A–C) and EOG54MW7V (4 exons; lower row D–F). Examples from run 1 (includes sequenced libraries: 27816, 27817, 27818) and run 2 (sequenced libraries: 28076, 28077) are given in A and D, and from run 3 (sequenced libraries: 11, 12, 13, 15, 16) in B and E. Introns are delineated by vertical lines in A, B, D and E. Boxplots in C and F highlight the distribution of sequencing depth for each exon within the OG grouped by the three different sequencing runs. Run 1 is indicated in green, run 2 in orange, and run 3 in purple. Horizontal lines in C and F are median sequencing depths, vertical lines show boxplot whiskers, and solid points represent outliers.

Sequencing depth (coverage per base) summary plots (Fig 2A–2E for examples, and S3 Fig) revealed variation between exons, OGs, and across individual sequenced libraries. The most substantial differences in exon sequencing depth occurred amongst sequenced libraries from the three different sequencing runs (χ^2^ = 99.7, df = 2, p < 0.0001; Fig 2C and 2F, and S5 Fig). The third run comprised sequenced libraries with an 11.6 fold lower median coverage (95% CI = 7.2, 18.9) across exons than the average median coverage values of runs 1 and 2 (which have equivalent median sequencing depth; ratio of coverage = 1.1, 95% CI = 0.5, 2.2). In addition, partitioning random variation in sequencing depth among target OGs versus among sequenced libraries in the analysis (after accounting for differences resulting from sequencing run) revealed that 60% of the variation can be explained by individual sample differences, while only 15% is caused by gene to gene variation. Certain sequenced libraries, including 27813, 27816 and 12, consistently encompassed the highest sequencing depth values across exons within their respective runs, whereas sequenced libraries, such as 27809, 27811, 28076 and 15, repeatedly revealed some of the lowest values across exons (see S3 and S5 Figs). Sequenced libraries from run 1 further appeared to contain greater variation in sequencing depth values within distinct exons than runs 2 and 3 (see Fig 2C and 2F for examples).

**Fig 3:**
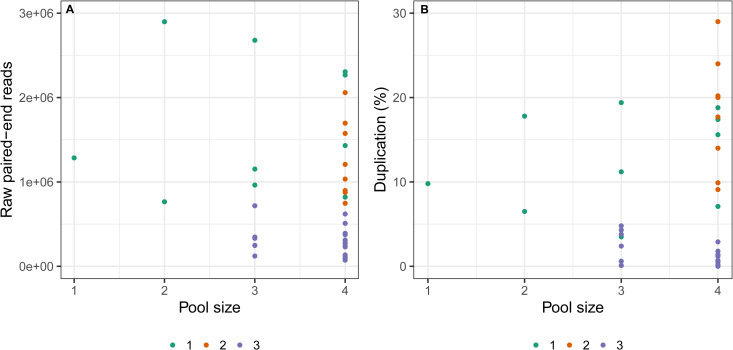
Plots of pooling sizes (1–4) prior to capture experiments against (A) raw paired-end reads and (B) duplication levels (%). Points are colour-coded by sequencing run, with run 1 indicated in green, run 2 in orange, and run 3 in purple.

Assessment of pooling selections prior to exon capture reactions (pool size/capture, Table 2) and their impact on median sequencing depth revealed no evident differences across particular pool sizes (S6 Fig). Although sample sizes for smaller pools were low and, as such, not rigorously tested here, a pool size of 2 revealed distinctly different results for sequencing depth across the two sequenced libraries included, with sequencing depth values constantly higher for one sequenced library over the other (S6 Fig). Furthermore, the replicate samples of BES18659 (27813, 27814 and 27815; S2 Table) had consistently high depth values for 50 randomly-selected exons irrespective of pool size (S6 Fig). Nevertheless, the sequencing depth values were persistently lower across exons for the single sequenced library, 27815 (pooled alone), which is consistent with the number of raw paired-end reads acquired for each of these libraries (Table 2). A comparison of the median sequencing depth values across exons compared with the number of raw paired-end reads obtained for each sequenced library revealed a largely positive linear relationship for the first two runs, but highlighted a less prominent pattern for run 3 (S7 Fig). Furthermore, the number of raw paired-end reads obtained was not correlated with pool sizes prior to capture (Fig 3A).

Levels of PCR duplication were reasonably low within runs, ranging from 3.5–19.4% in run 1, 9.1–29% in run 2 and 0.1–4.8% in run 3, and did not differ substantially amongst ingroup and outgroup species (Table 2). An assessment of the relationship between percentage duplication levels versus pool size per capture revealed no correlation, with similar values and no apparent pattern across pools 1–4 (Fig 3B). The amount of missing data in terms of coverage across exons (calculated based on the dataset B concatenated alignment) differed considerably between sequencing run 3 and the batches with fewer pooled libraries, ranging from 2.7–15.5% for run 1, 2.8–7.9% for run 2 and 11.2–99.4% for sequencing run 3 (Table 2). The raw paired-end data yield for sequenced libraries in run 3 did not appear to directly correspond to the amount of data acquired in the final alignment, with sequenced libraries 11 and 12 comprising 391,058 and 332,292 raw paired-end reads and 12.4 and 11.2% missing data, respectively, and sequenced libraries 15 and 17 consisting of a similar raw data yield, but a substantially larger amount of missing data (98.1 and 97.1%, respectively) (Table 2). The six outgroup taxa, which are more distantly related to the reference used for mapping, revealed similar levels of missing data to some of the ingroup sequenced libraries included in run 3 (Table 2).

**Fig 4:**
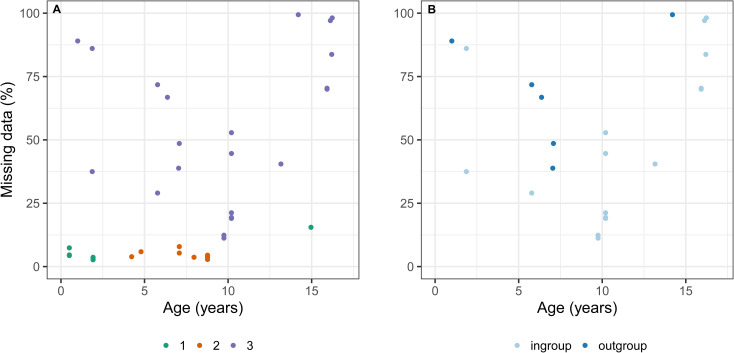
Preservation age of specimens (years) included in the exon captures against (A) missing data (%), and (B) missing data (%) for run 3 sequenced libraries only. Points in (A) are colour-coded by sequencing run, with run 1 indicated in green, run 2 in orange, and run 3 in purple. Points in (B) are coloured by ingroup (light blue) and outgroup (dark blue) status.

We assessed whether the differences in missing data within sequencing runs (particularly run 3) were related to the preservation age of specimens. For run 1, the majority of samples were collected 0.5–2 years prior and were characterised by a similarly low percentage of missing data. However, one older sample, which was collected around 15 years prior, consisted of the highest level of missing data for the run at 15.5% (Fig 4A and Table 2). For run 2, specimens ranged from 4–8.8 years old, and the level of missing sequence data for all sequenced libraries corresponded to that of the more freshly collected and preserved specimens from run 1 (Fig 4A and Table 2). For the third run, the percentage of missing data varied and did not appear to correlate with specimen preservation age; nevertheless, sequencing libraries with the oldest samples (14–16 years old) all comprised a large degree of missing data (Fig 4A and Table 2). Run 3 sequenced libraries were further compared on the basis of ingroup and outgroup status (Fig 4B), with most outgroup taxa comprising a reasonably large amount of missing data (38.8–99.4%) independent of preservation age. Furthermore, sequencing depth against age was examined for exons from 50 randomly-selected OGs, with no apparent relationship (S8 Fig). This lack of a correlation was particularly evident for run 1, which exhibited a large degree of variation in median sequencing depth among the sequenced libraries despite similar preservation ages, and highlighted the individual specimen effect on variation (S8 Fig).

### Exon sequencing depth uniformity

Coefficient of dispersion (CD) across sequencing runs for the targeted (regions of the target OG where overlapping baits were placed) exons for 50 randomly selected OGs was consistently higher and more variable in run 3 for sequencing depth (S9 Fig). The primarily low CD values of 10–15% for sequenced libraries in runs 1 and 2, and 20–30% for those in run 3, nevertheless, revealed largely consistent sequencing depth along positions within the majority of exons examined, indicating high uniformity (S9 Fig). These exons were typically short, between 94–234 bp long; however, CD values for the smaller number of long exons (EOG5FXPQ3: 852 bp and EOG505QG8: 876 bp, S9 Fig) were much higher (>60%), signifying greater levels of variation and lower uniformity.

### Phylogenetic analyses

**Fig 5:**
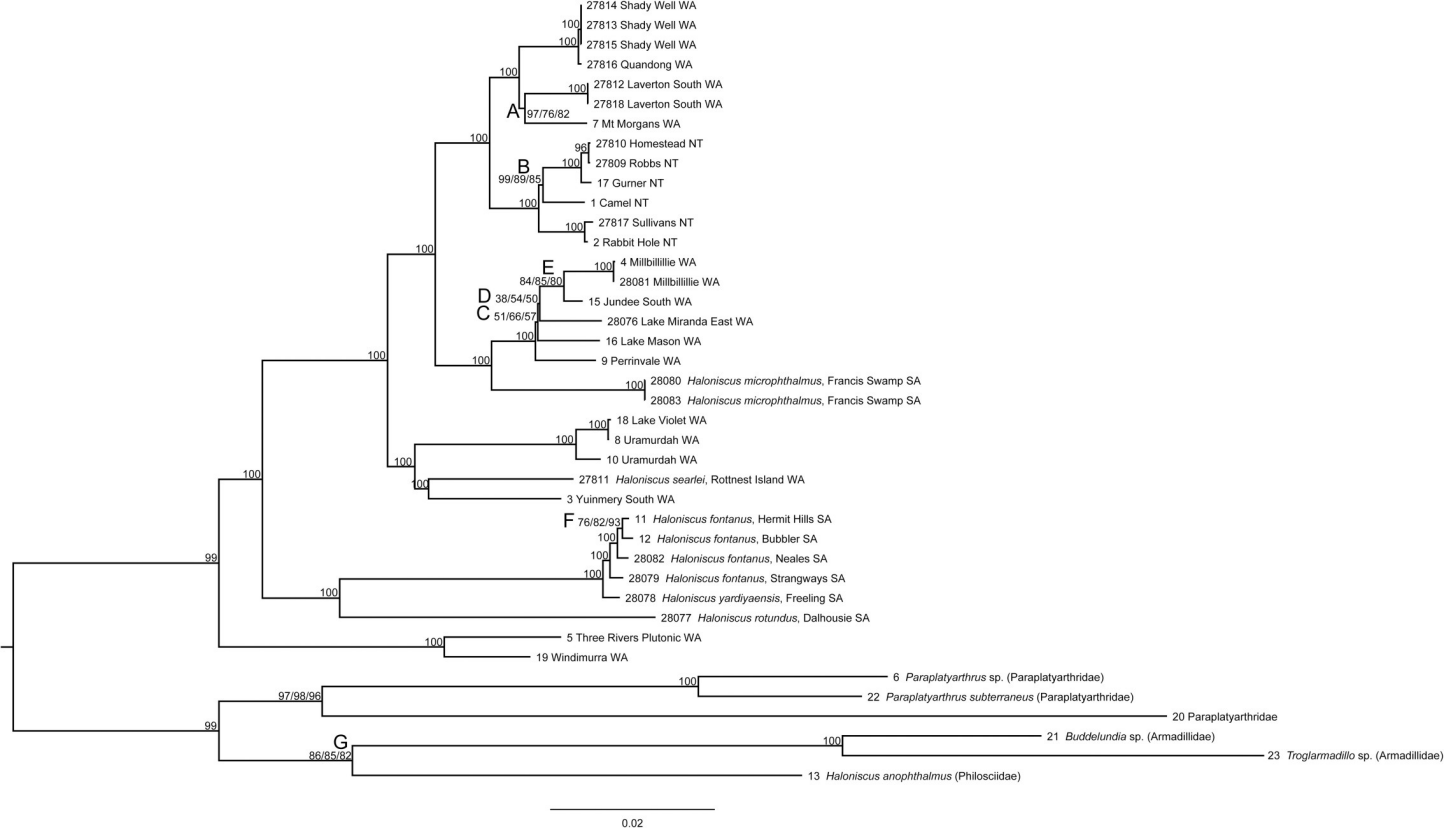
Maximum likelihood (ML) phylogeny of *Haloniscus* and outgroup isopod taxa inferred using RAxML. The phylogeny represents trees reconstructed using three datasets (datasets A–C) for which the resultant topologies were identical (see S10 Fig for all trees). ML bootstrap values are summarised on branches (i.e. datasets A, B and C, respectively, with each value separated by a forward slash unless all values are identical). Letters A–G represent branches with support values that differ significantly across the phylogenies and/or are referred to in the text. Isopod taxa are listed by species name and/or collection locality. Included abbreviations are as follows: WA = Western Australia, SA = South Australia, and NT = Northern Territory.

The three final alignments (containing up to 25% (A), 50% (B), and 75% (C) missing sequences) included sequence data for all 40 sequenced libraries, and consisted of 420, 807, and 1026 exons, respectively (alignments available on Figshare at doi:10.25909/5ef570329cbdd). For dataset A, these exons were included from 335 of the 469 targeted OGs, constituting 88,402 bp of DNA sequence data. Datasets B and C consisted of exons from 440 (143,445 bp) and 451 (174,006 bp) OGs, respectively. The inferred ML trees revealed identical topologies (Fig 5 and S10 Fig), with the majority of branches showing bootstrap support of 100%. Support values were largely consistent across the three phylogenies, discounting some of the more recent splits (see Fig 5A–5F), where bootstrap support varied between trees and did not necessarily increase with the addition of further exon data. Support for clades A and B (Fig 5) decreased noticeably between dataset A and the remaining two datasets (B and C), whereas support for clade F increased. The phylogenetic positions of the six outgroup taxa were principally well-resolved, except for clade G (Fig 5), which is likely due to the large amount of missing data for *H*. *anophthalmus* (BES10201 (library 13), Table 2).

### HybPiper assembly comparison

Sequence data assembly using HybPiper, which involved an initial mapping step where cleaned reads were assigned to target OGs from the eight reference species (see S1 Table), revealed that sequencing runs 1 and 2 generated a larger amount of data for sequenced libraries compared to run 3. The number of mapped reads and correspondingly the number of OGs recovered was typically higher for sequenced libraries of runs 1 and 2 (S3 Table). Results for libraries included in run 3 were more varied, with some sequenced libraries, including 4, 10, 11 and 12, containing over 400 captured genes, while others, such as 2, 13, 15 and 17, were found to comprise the largest amount of missing data (S3 Table). Sequenced libraries for outgroup species revealed variable results following the HybPiper pipeline, with between 12 (sequencing ID: 13) and 441 (sequencing ID: 22) total OGs captured. The final alignment (dataset D) consisted of sequences for all 40 isopod sequenced libraries, and comprised sequences from 454 (after removal of 11 OGs with paralogous sequences) concatenated OG files, totalling 253,786 bp (alignment available on Figshare at doi:10.25909/5ef570329cbdd). The inferred ML phylogeny (S11 Fig) revealed a consistent topology to the trees reconstructed using the mapping approach (Fig 5 and S10 Fig), with the majority of branches exhibiting bootstrap support values of 100%. Lower support values tended to be associated with clades containing taxa with a large amount of missing data.

## Discussion

We employed a custom transcriptome-based exon capture approach without a reference genome to effectively generate an informative phylogenomic dataset for the genus *Haloniscus* and more distantly related paraplatyarthrid and armadillid isopods. Transcriptome sequences were used without initially distinguishing intron-exon boundaries prior to bait design, which resulted in the recovery of numerous coding exons of various lengths, together with a considerable amount of non-coding (intron) flanking data. The inferred bait set represents a significant step forward from earlier molecular datasets used to explore *Haloniscus* evolution and systematics, which utilised one mitochondrial (*COI* [28, 29]) or two genes (*COI* and *18S rRNA* [30]), with bootstrap support values generally poor for internal splits within *Haloniscus* phylogenetic trees. The well-resolved phylogenies produced here, therefore, indicate that this custom bait set provides considerable potential for application in future isopod molecular studies, particularly in light of the broad taxonomic applicability of our baits [27]. We examine the overall efficacy of our bait design and the performance of this transcriptome-based exon capture approach in more detail below.

This transcriptome-based exon capture approach has several key advantages, most notably helping to overcome the lack of an available reference genome to recognise intron-exon boundaries prior to bait design. Tiling across these boundaries has allowed for the recovery of many short (<100 bp) and longer (>300 bp) exons, with high uniformity in sequencing depth (S9 Fig) and a large amount of variable non-coding flanking (intron) data. We recovered a significant quantity of short exons (median length: 153 bp), which would not typically be targeted in exon capture studies that use a reference genome and larger exons for bait design [7], but that dominate the majority of arthropod genes. Moreover, calculations for the coefficient of dispersion have highlighted that the bait design (which spans intron boundaries) promoted consistent sequencing depth values across, primarily short, exons. Portik et al. [24] reported comparable findings, contrasting with studies that utilised a reference genome in bait design to define and tile across exons, which have uncovered an ‘edge effect’, where fewer tiled baits towards the ends of exons leads to a decrease in sequencing depth at the exon edges [7, 15]. The pipeline outlined here, however, differs from that used by Portik et al. [24] by using: BaitFisher, which permits a large number of loci to be targeted for multiple reference species with a smaller number of baits (reducing overall costs) [27], and Orthograph, which reliably infers OGs using a best reciprocal hit approach [38]. Lastly, the intron sequence data, while not examined here, is likely to be valuable for population genetics, species delimitation, and phylogenetic analyses.

For our exon capture approach, sequencing depth was consistently high across targeted exons for all libraries incorporated within the first and second sequencing runs, but was significantly lower for the third. While the general coverage levels for the first two runs considerably outweighed the amount needed per exon for inclusion in the final alignments, the overall amount of missing data (across exons in the dataset B alignment) for the majority of sequenced libraries included in the third run was considerably larger than for libraries within the previous sequencing rounds. These differences in sequencing depth and missing data recovered in the third run likely resulted from the higher number of pooled libraries prior to sequencing and, consequently, we recommend either a more conservative pooling selection or an alternative high-throughput platform, such as the Illumina HiSeq or NovaSeq, especially when the amount of intron data being sequenced is unknown. The third run, however, also consisted of many isopod specimens stored for a long period of time (collected and preserved in ethanol >14 years prior) with likely degraded DNA as well as more divergent outgroup taxa, which may have further influenced the ultimate success of this exon capture run [7, 20, 24].

Analyses of specimen storage time (age) uncovered no apparent relationship with either the level of sequencing depth across exons or missing data (Fig 4A and S8 Fig). Overall, the percentage of missing data varied considerably across sequenced libraries within the third sequencing run, with data from some of the more recently collected specimens (i.e. sequencing ID: 2 (BES18759.3), which contained a corresponding low number of raw paired-end reads) consisting of few recovered exons (Fig 4). Nevertheless, the oldest specimens in the first and third runs all contained the highest proportion of missing data and, therefore, it is probable that specimen preservation age (likely combined with storage conditions [20], although not examined here) played a role in the success of these capture experiments. Overall, the bait design and this capture protocol successfully enriched exon data from up to 16-year-old isopod specimens, which is comparable to findings from previous phylogenomics studies [19, 64, 65].

The outgroup isopods exhibited an expected higher percentage of missing data (see [7, 24]), especially for library 21 (B002, Armadillidae). However, we acknowledge that this level of missing data may be a result of the mapping approach used (mapped to *Haloniscus* reference OGs) in data processing rather than to the efficacy of the baits, since similar numbers of raw paired-end reads were obtained for these outgroups and the *Haloniscus* sequenced libraries included in the run, which suggests that assembly methods may have been preferential to mapping for these outgroup taxa. Further analyses using the assembly pipeline, HybPiper, yielded similar results to those produced with the *Haloniscus* reference mapping approach, with capture runs 1 and 2 performing better overall (i.e. with greater numbers of OGs recovered) compared to run 3, and *Haloniscus* sequenced libraries revealing generally consistent results across the analyses. The results for the outgroups, however, were improved using the assembly method, with comparable results revealed to those for the *Haloniscus* sequenced libraries within that run (but with sequenced libraries 13 (*Haloniscus anophthalmus*) and 21 (Armadillidae) still recording the lowest number of recovered OGs). The increased number of OGs recovered is likely due to the inclusion of the eight reference species, with broad taxonomic depth, within the pipeline rather than only a single *Haloniscus* reference taxon in mapping. The designed baits were, therefore, able to enrich exon capture data from a range of oniscidean isopod families, including Philosciidae, Paraplatyarthridae, and Armadillidae (not represented in the bait design), and are suitable for high-level phylogenetic practice. The output trees (based on datasets Fig 5A–5D and S11 Fig), furthermore, were largely identical topologically, confirming the utility of the *Haloniscus* mapping approach, particularly when the reference is closely related to the target species.

We further provide additional empirical data on the question surrounding how many libraries may be pooled in a single reaction prior to capture without overly impacting the quality of the sequence data obtained (as in [24]). We tested pools consisting of 1–4 libraries (12 pooled reactions total), and considered potential effects on sequencing depth, raw paired-end data yield and PCR duplication levels. While low sample sizes precluded rigorous testing, we uncovered no discernible patterns in the exon sequencing depth or raw sequencing yield across sequenced libraries from different pool sizes (Fig 3A and S6 Fig). However, unlike Portik et al. [24], we uncovered no trend in duplication levels across pooling sizes (Fig 3B), but instead, duplication was predominantly lower across sequenced libraries in the third run, which included a lower number of raw paired-end reads for distinct libraries. Therefore, our results suggest that at least four libraries (or potentially more) may be pooled in a single capture reaction, which has important implications for reducing the costs of a study by improving the efficiency of experiments and increasing the number of samples that can be included in a project [12]. However, the limits of this pooling strategy have not been tested here and should be examined in future studies. Rather than library pooling selections or the preservation age of specimens, our results have indicated that undetermined characteristics of the samples included in the capture runs, such as field handling or storage conditions, likely accounted for the variation (after excluding sequencing run) in general sequence data quality.

Overall, the exon capture methods and bioinformatics data processing approach used here have been effective in obtaining a large set of single-copy orthologous groups, successful bait design that enriches targeted loci from diverse *Haloniscus* and other more distantly related isopod species, and generating a large and informative phylogenomic dataset. While the final alignments employed for ML tree inference contained differing levels of missing data, the phylogenies revealed identical topologies and were largely consistent with previous taxonomic and phylogenetic research [28,30,66]. In contrast to previous studies, however, most of the phylogenetic relationships inferred here were fully resolved, showing maximal bootstrap support, particularly for internal branches, which provides further confidence in this approach. Lower node support for some of the recently diverged isopod taxa (Fig 5 and S11 Fig) may have been influenced by methods used to initially select OGs (as per [67]); however, the issue may be equally attributed to samples of poorer quality and, thus, requires further investigation. Additional phylogenetic analyses, as well as the implications of these results for the systematics and biogeographic history of *Haloniscus*, will be examined in a subsequent paper. By providing our transcriptomes, filtered OGs, bait design, and custom bioinformatics scripts with automated post-processing steps, we make our approach transparent and, therefore, useful and adaptable for future studies. While detecting and separating out the many exons manually is a time-consuming process, automated scripts have recently been published [15] that infer intron-exon boundaries from alignments. Finally, our methodological outline permits these target capture methods to be carried out completely in-house, without the need for outsourcing, whereas previous protocols have not always disclosed complete workflows.

## Supporting information

S1 Fig A short read alignment (in integrative genomics viewer) highlighting the position of an intron-exon boundary. Each bar represents a single read. Bases matching the reference sequence are shown in grey, while soft-clipped bases (mismatched bases) are coloured. The *Haloniscus* reference sequence is indicated above the blue bar at the bottom of the figure.
(PDF)

S2 Fig Alignments of short reads (in integrative genomics viewer) to three putative exons (blue bars) after inferring intron-exon boundaries from the *Haloniscus* reference sequence used in bait design. (A) Bases matching the reference sequence are shown in grey, while mismatched bases or bases beyond the reference are coloured, representing introns and single nucleotide polymorphisms, (B) position of baits regions. The reads and the reference are from different species, but both are from the *Haloniscus* genus.
(PDF)

S3 Fig Sequencing depth summaries for all 469 targeted orthologous groups (OGs), and isopod sequenced libraries. Each page shows one OG and the sequencing depth results for all libraries. Vertical lines indicate intron positions.
(PDF)

S4 Fig A frequency distribution for the length of exons (bp) captured across the three sequencing runs. Run 1 is indicated in green, run 2 in orange, and run 3 in purple.
(TIF)

S5 Fig Distribution of sequencing depth across isopod sequenced libraries at each exon within all orthologous groups (OGs), grouped by the three sequencing runs. Run 1 is depicted in green, run 2 in orange, and run 3 in purple. The ID of each OG is specified above plots. Horizontal lines are median sequencing depths, vertical lines depict boxplot whiskers, and solid points represent outliers.
(PDF)

S6 Fig Plots of pooling sizes (1–4) prior to capture against median sequencing depth across isopod sequenced libraries for exons of 50 randomly targeted orthologous groups (OGs), separated by sequencing run (1–3). The ID of each OG is specified above plots, and points are coloured according to sequencing ID.
(PDF)

S7 Fig Plots of raw paired-end reads against median sequencing depth across isopod sequenced libraries for exons of 50 randomly targeted orthologous groups (OGs), separated by sequencing run (1–3). The ID of each OG is specified above plots, and points are coloured according to sequencing ID.
(PDF)

S8 Fig Plots of specimen preservation age (time in years) against median sequencing depth across isopod sequenced libraries for exons of 50 randomly targeted orthologous groups (OGs), separated by sequencing run (1–3). The ID of each OG is specified above plots, and points are coloured according to sequencing ID.
(PDF)

S9 Fig Distribution of the robust coefficient of dispersion (CD) across isopod sequenced libraries at each exon for 50 randomly targeted orthologous groups (OGs), separated by sequencing run. Run 1 is depicted in green, run 2 in orange, and run 3 in purple. The ID of each OG is specified above plots. Horizontal lines show median CD, vertical lines depict boxplot whiskers, and solid points represent outliers.
(PDF)

S10 Fig Maximum likelihood (ML) phylogenies of *Haloniscus* and outgroup isopods inferred using RAxML and varying levels of missing data. Dataset A has a 25% missing sequence threshold, dataset B a 50% threshold, and dataset C a 75% threshold. ML bootstrap values are indicated on branches, with letters A–G representing support values that differ significantly across the phylogenies and/or are referred to in the text. Isopod taxa are listed by species name and/or collection locality. Included abbreviations are as follows: WA = Western Australia, SA = South Australia, and NT = Northern Territory.
(PDF)

S11 Fig Maximum likelihood (ML) phylogeny of *Haloniscus* and outgroup isopod taxa inferred using RAxML and dataset D (HybPiper). ML bootstrap values are indicated on branches and isopod taxa are listed by species name and/or collection locality. Included abbreviations are as follows: WA = Western Australia, SA = South Australia, and NT = Northern Territory.
(PDF)

S1 File Continued details for transcriptome sequencing and contig assembly methods. (DOCX)

S1 Table Taxon sampling for transcriptome sequencing, and the number of individuals pooled for each library. (XLSX)

S2 Table Taxon sampling for exon capture with detailed collection data. This information includes Western Australian Museum Biospeleology Collection (BES) and Great Artesian Basin (GAB) sample identifiers. Note: the sample from Windimurra consists of pooled DNA extracts from three different individuals. Included abbreviations are as follows: WA = Western Australia, SA = South Australia, and NT = Northern Territory.
(XLSX)

S3 Table Summary statistics for HybPiper data assembly. Note: sequencing of samples BES18659 and GAB00764.1 was repeated using multiple libraries prior to exon capture.
(XLSX)
