# Supplementary material for: Development and evaluation of a custom bait design based on 469 single-copy protein-coding genes for exon capture of isopods (Philosciidae: Haloniscus)
Source: PLoS One. 2021 Sep 17;16(9):e0256861. doi: 10.1371/journal.pone.0256861 (PMC8448321; doi:10.1371/journal.pone.0256861)
Supplement: S1 Fig — Each bar represents a single read. Bases matching the reference sequence are shown in grey, while soft-clipped bases (mismatched bases) are coloured. The Haloniscus reference sequence is indicated above the blue bar at the bottom of the figure. (PDF) [file pone.0256861.s001.pdf]

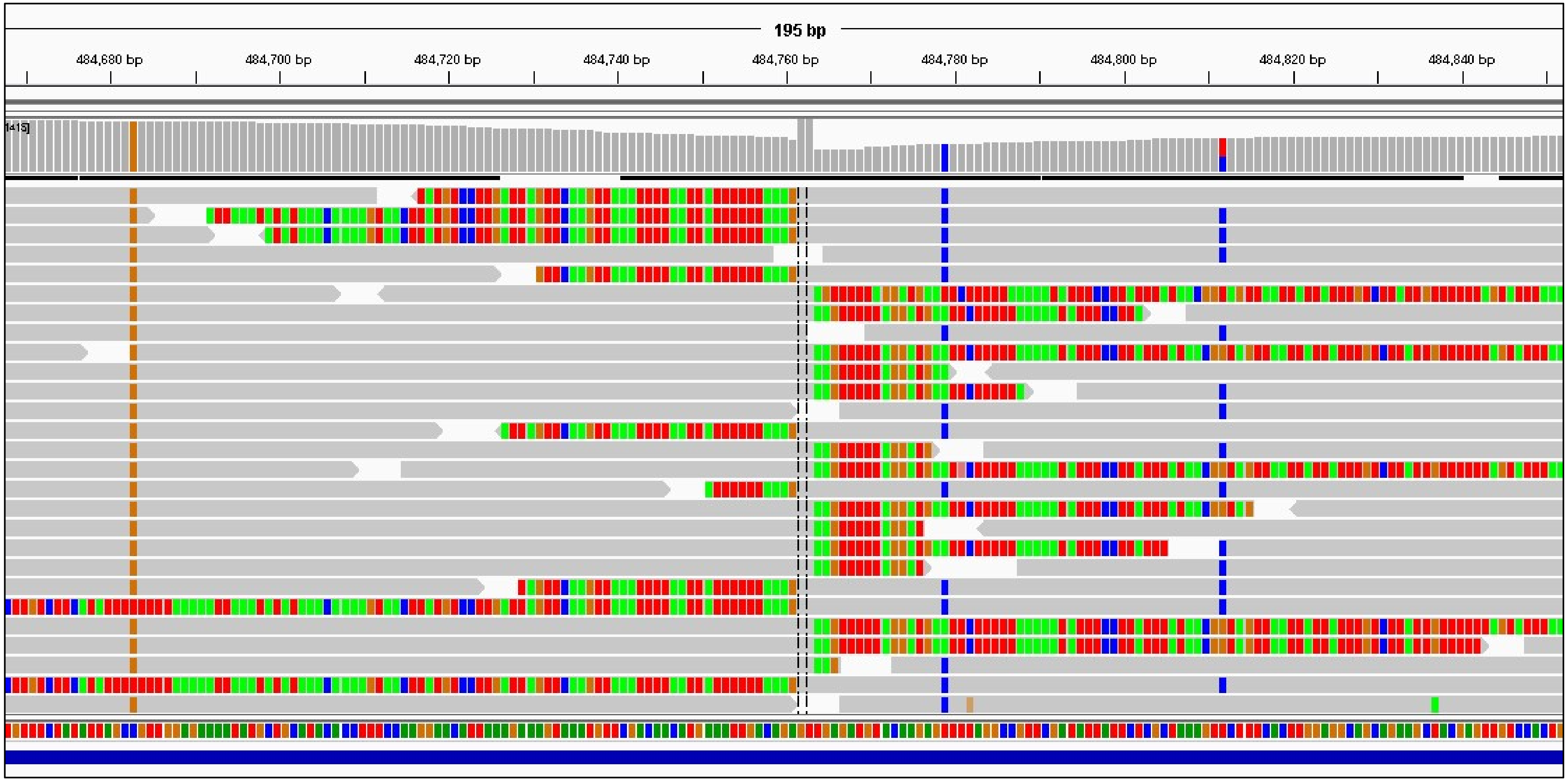


**S1 Fig. A short read alignment (in Integrative Genomics Viewer) highlighting the position of an intron-exon boundary.** Each bar represents a single read. Bases matching the reference sequence are shown in grey, while soft-clipped bases (mismatched bases) are coloured. The *Haloniscus* reference sequence is indicated above the blue bar at the bottom of the figure.
