# Supplementary material for: Development and evaluation of a custom bait design based on 469 single-copy protein-coding genes for exon capture of isopods (Philosciidae: Haloniscus)
Source: PLoS One. 2021 Sep 17;16(9):e0256861. doi: 10.1371/journal.pone.0256861 (PMC8448321; doi:10.1371/journal.pone.0256861)
Supplement: S2 Fig — (A) Bases matching the reference sequence are shown in grey, while mismatched bases or bases beyond the reference are coloured, representing introns and single nucleotide polymorphisms, (B) position of baits regions. The reads and the reference are from different species, but both are from the Haloniscus genus. (PDF) [file pone.0256861.s002.pdf]

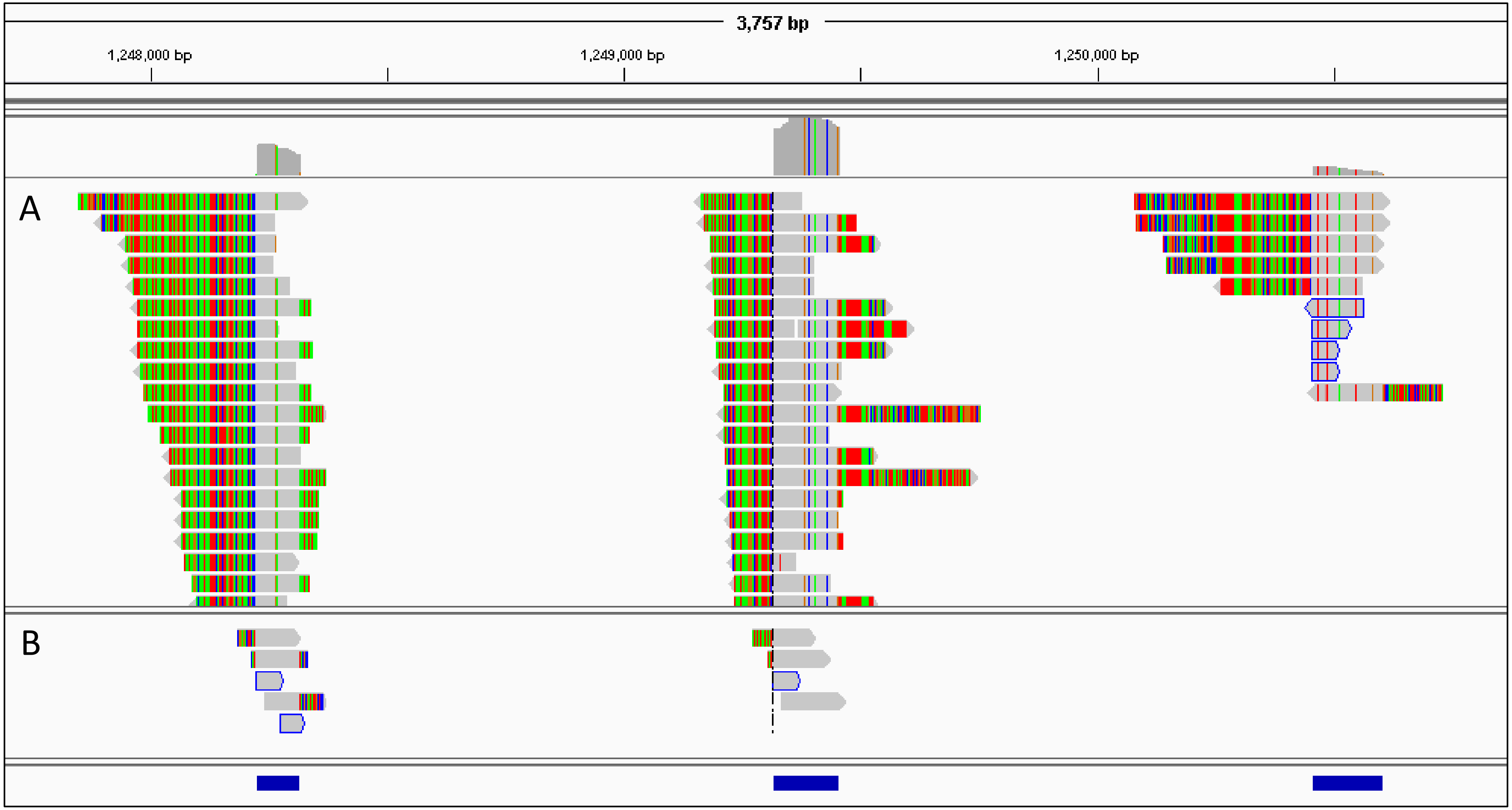

S2 Fig. Alignments of short reads (in Integrative Genomics Viewer) to three putative exons (blue bars) after inferring intron-exon boundaries from the *Haloniscus* reference sequence used in bait design. (A) Bases matching the reference sequence are shown in grey, while mismatched bases or bases beyond the reference are coloured, representing introns and single nucleotide polymorphisms, (B) position of baits regions. The reads and the reference are from different species, but both are from the *Haloniscus* genus.
