# Supplementary material for: Development and evaluation of a custom bait design based on 469 single-copy protein-coding genes for exon capture of isopods (Philosciidae: Haloniscus)
Source: PLoS One. 2021 Sep 17;16(9):e0256861. doi: 10.1371/journal.pone.0256861 (PMC8448321; doi:10.1371/journal.pone.0256861)
Supplement: S3 Fig — Each page shows one OG and the sequencing depth results for all libraries. Vertical lines indicate intron positions. (PDF) [file pone.0256861.s003.pdf]

EOG5QV9TR

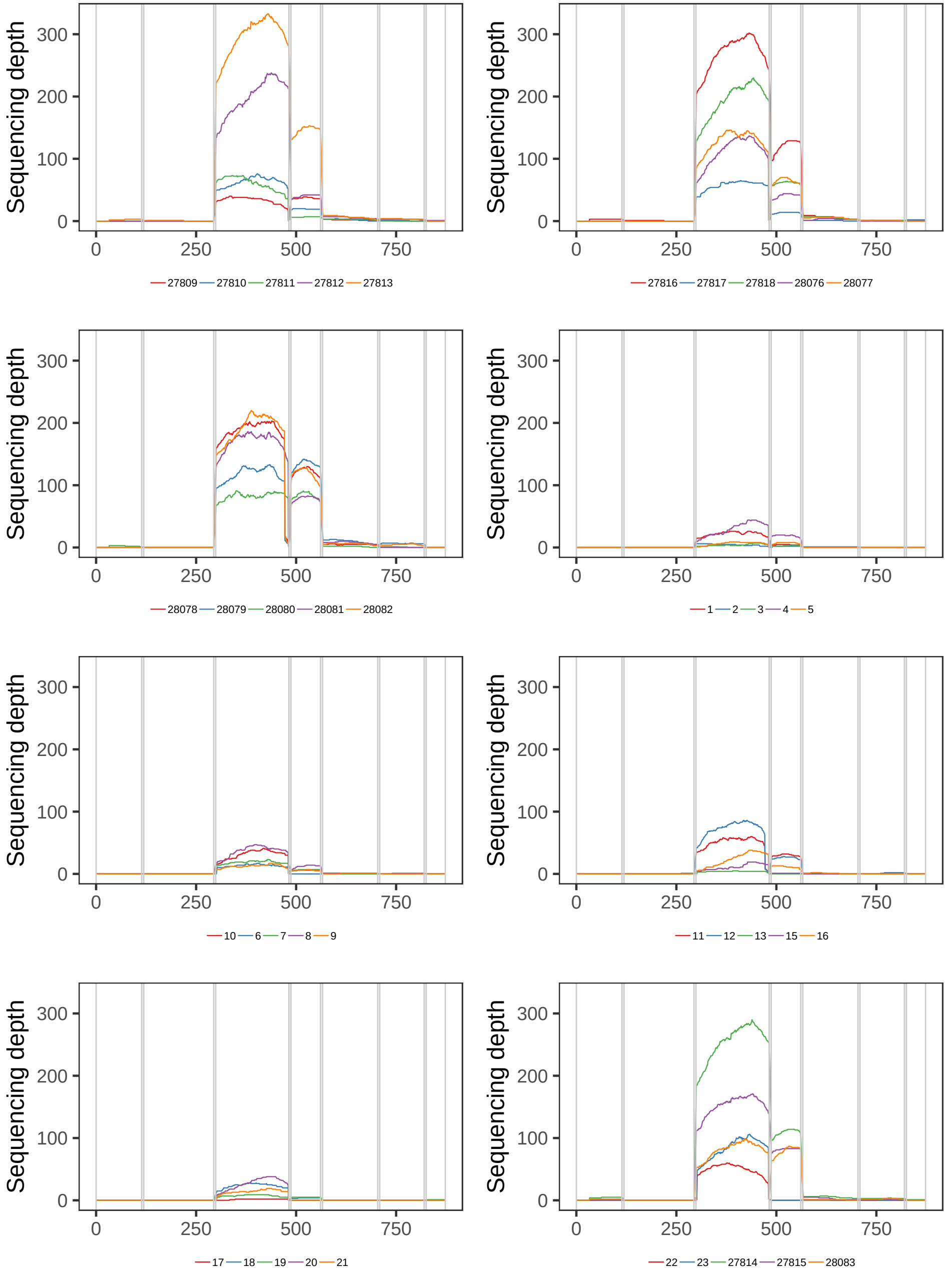
Sequencing depth
0
100
200
300
0
250
500
750
27809
27810
27811
27812
27813
27816
27817
27818
28076
28077
28078
28079
28080
28081
28082
1
2
3
4
5
10
6
7
8
9
11
12
13
15
16
17
18
19
20
21
22
23
27814
27815
28083

EOG5T4BBD

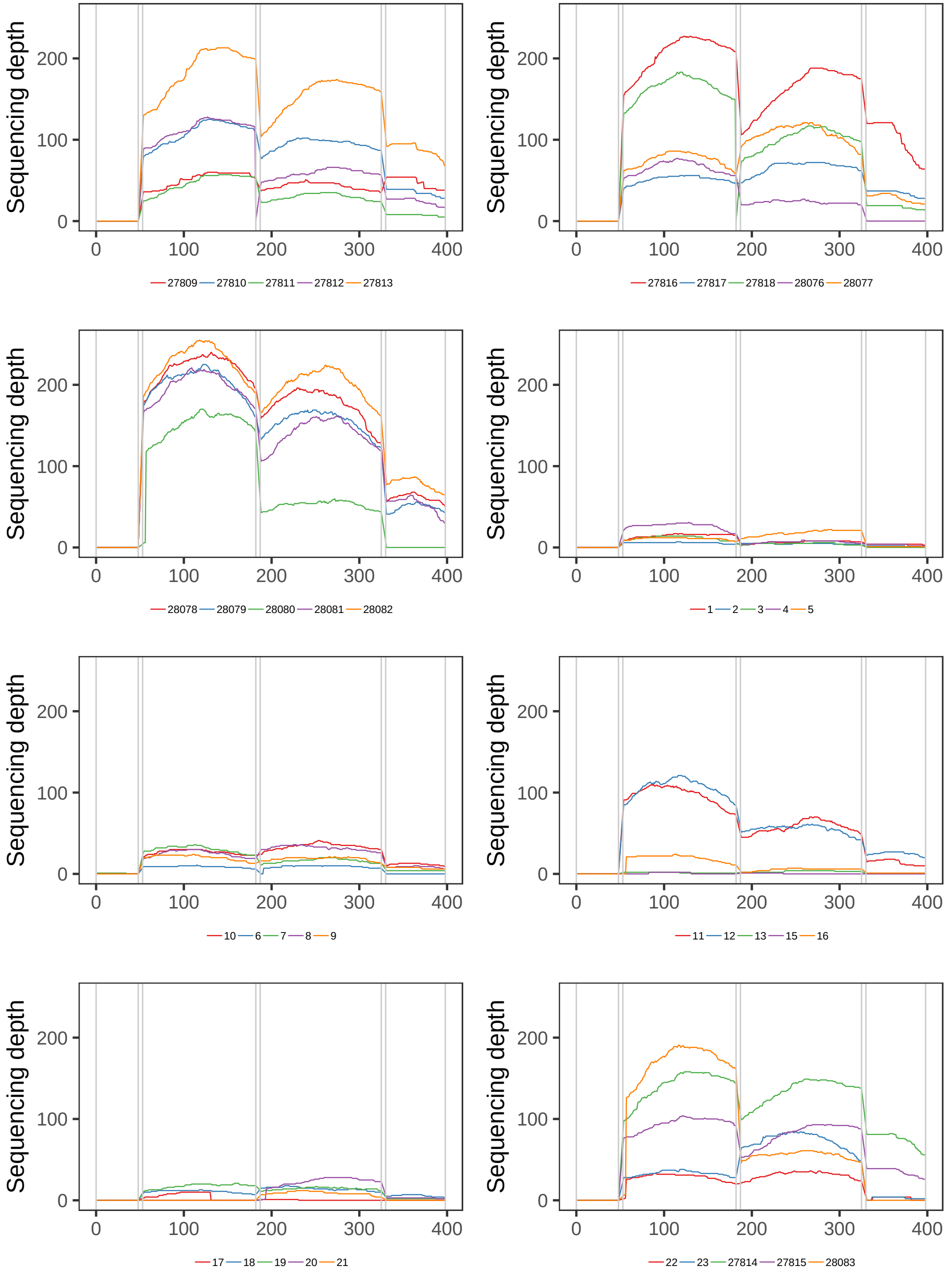
Sequencing depth
0
100
200
300
400
27809
27810
27811
27812
27813
27816
27817
27818
28076
28077
28078
28079
28080
28081
28082
1
2
3
4
5
10
6
7
8
9
11
12
13
15
16
17
18
19
20
21
22
23
27814
27815
28083

EOG5W3R4P

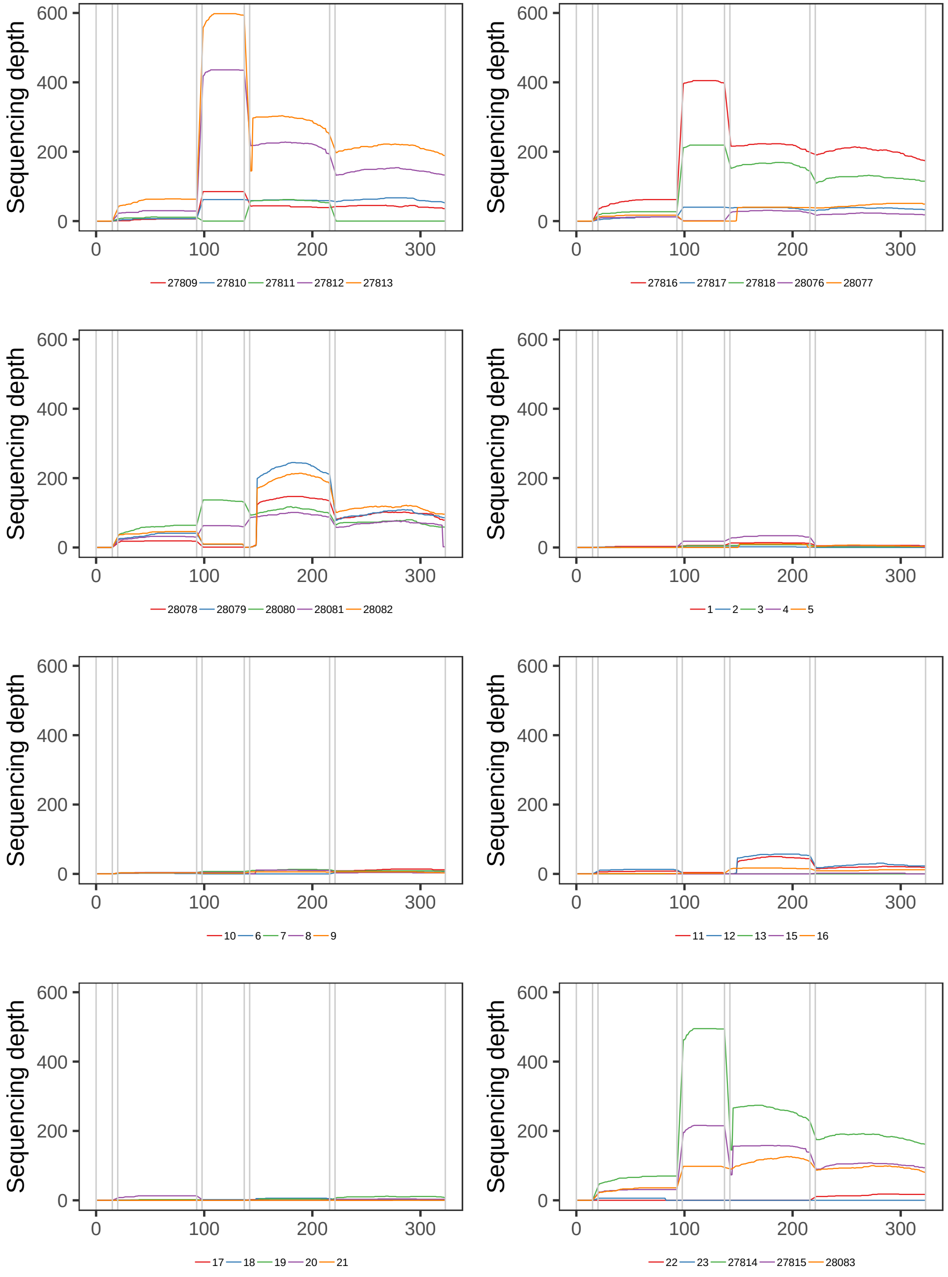
Sequencing depth
600
400
200
0
100
200
300
27809 27810 27811 27812 27813
27816 27817 27818 28076 28077
28078 28079 28080 28081 28082
1 2 3 4 5
10 6 7 8 9
11 12 13 15 16
17 18 19 20 21
22 23 27814 27815 28083

EOG544J20

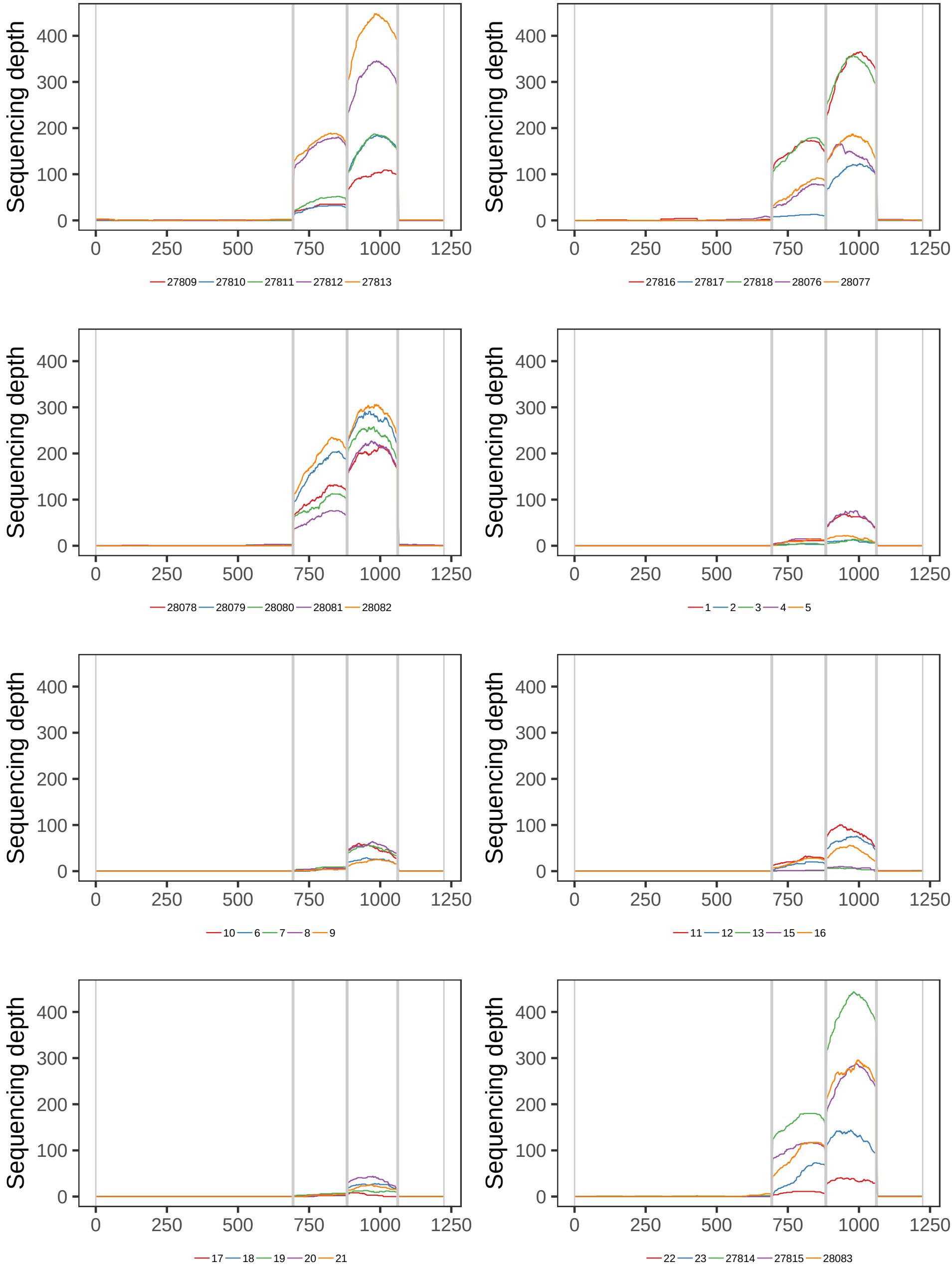
Sequencing depth
0
100
200
300
400
0
250
500
750
1000
1250
27809 27810 27811 27812 27813
27816 27817 27818 28076 28077
28078 28079 28080 28081 28082
1 2 3 4 5
10 6 7 8 9
11 12 13 15 16
17 18 19 20 21
22 23 27814 27815 28083

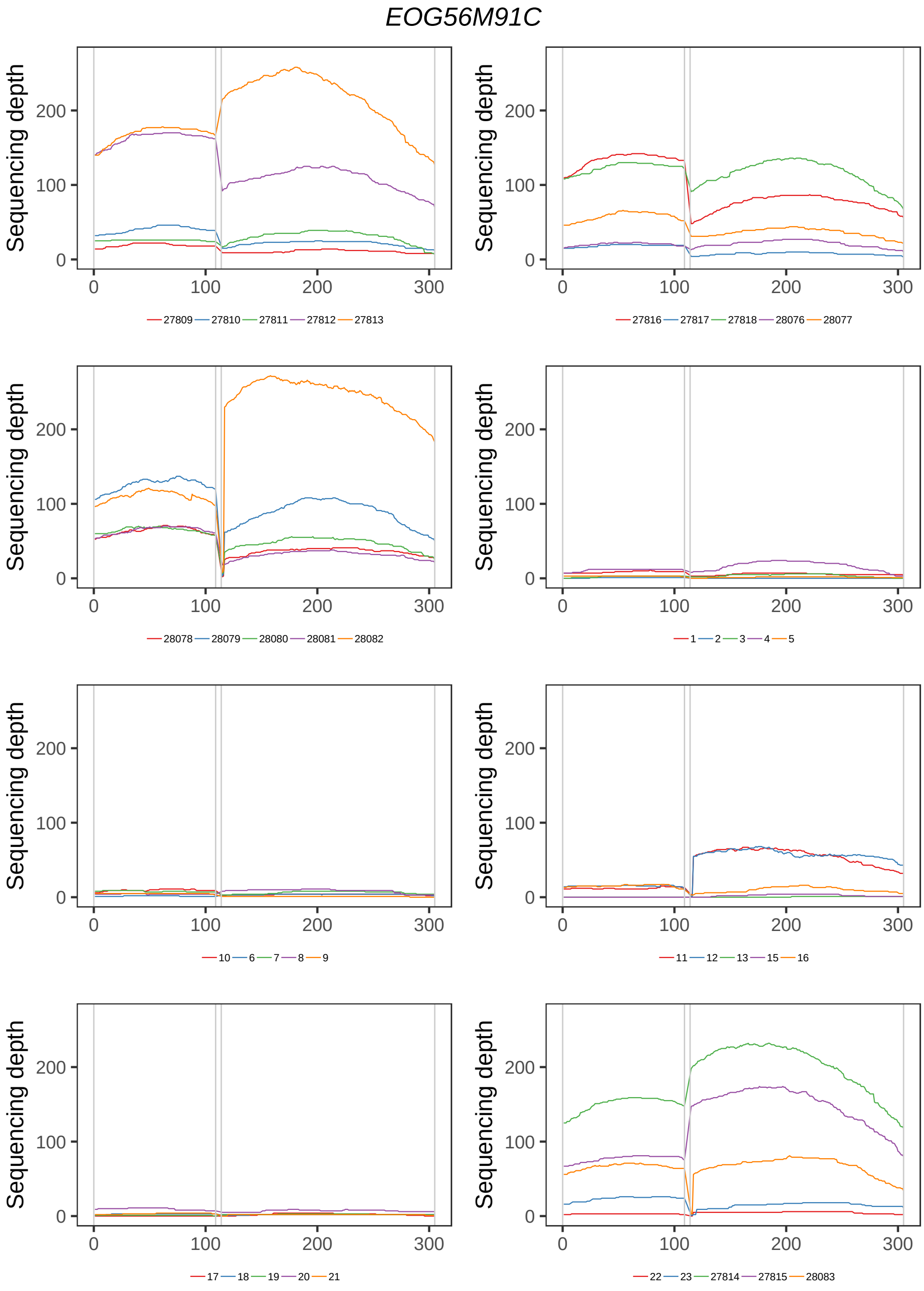
EOG56M91C
Sequencing depth
27809 27810 27811 27812 27813
27816 27817 27818 28076 28077
28078 28079 28080 28081 28082
1 2 3 4 5
10 6 7 8 9
11 12 13 15 16
17 18 19 20 21
22 23 27814 27815 28083

EOG576HGC

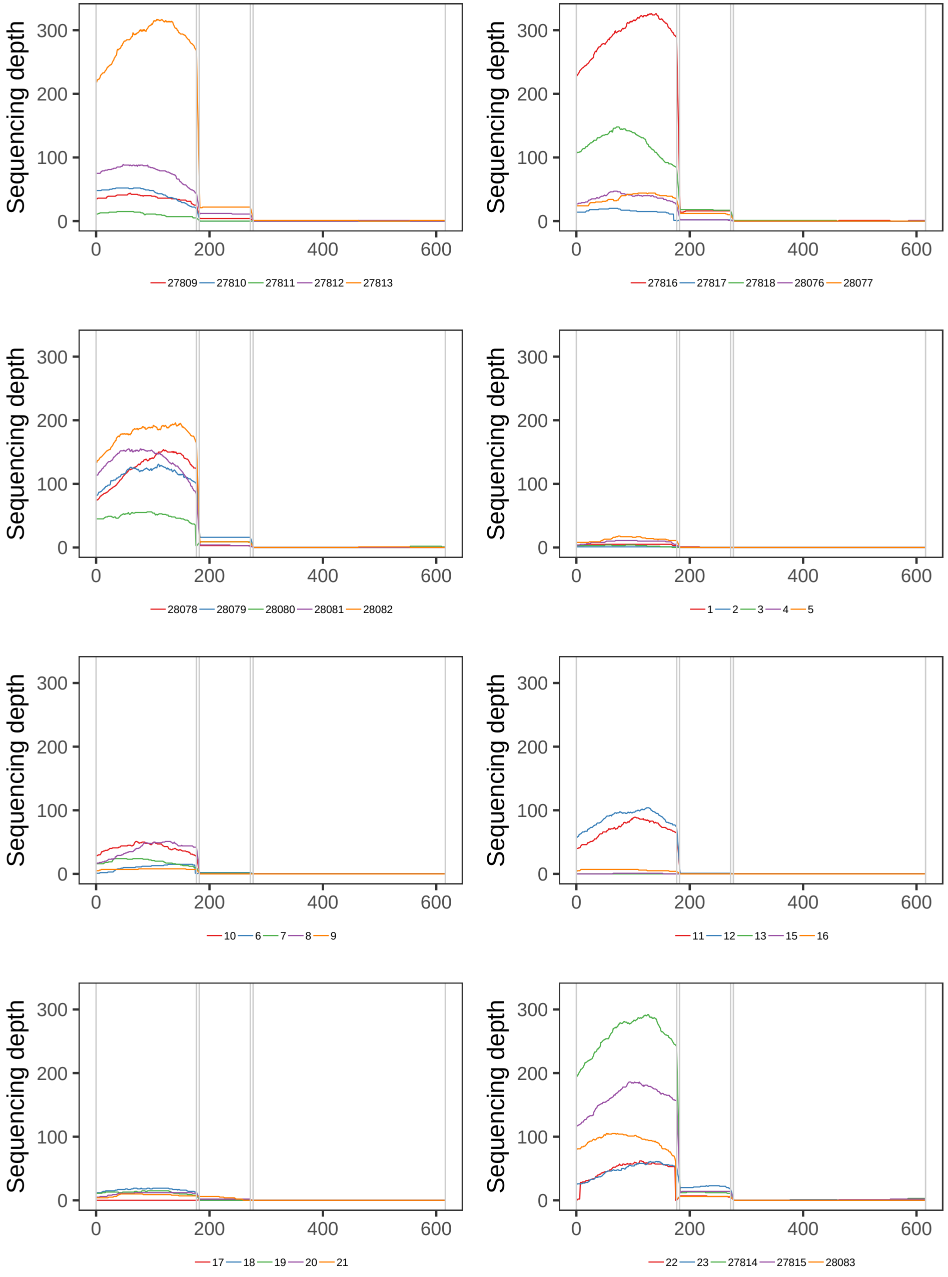
Sequencing depth
0
100
200
300
0
200
400
600
27809 27810 27811 27812 27813
27816 27817 27818 28076 28077
28078 28079 28080 28081 28082
1 2 3 4 5
10 6 7 8 9
11 12 13 15 16
17 18 19 20 21
22 23 27814 27815 28083

EOG57SQWH

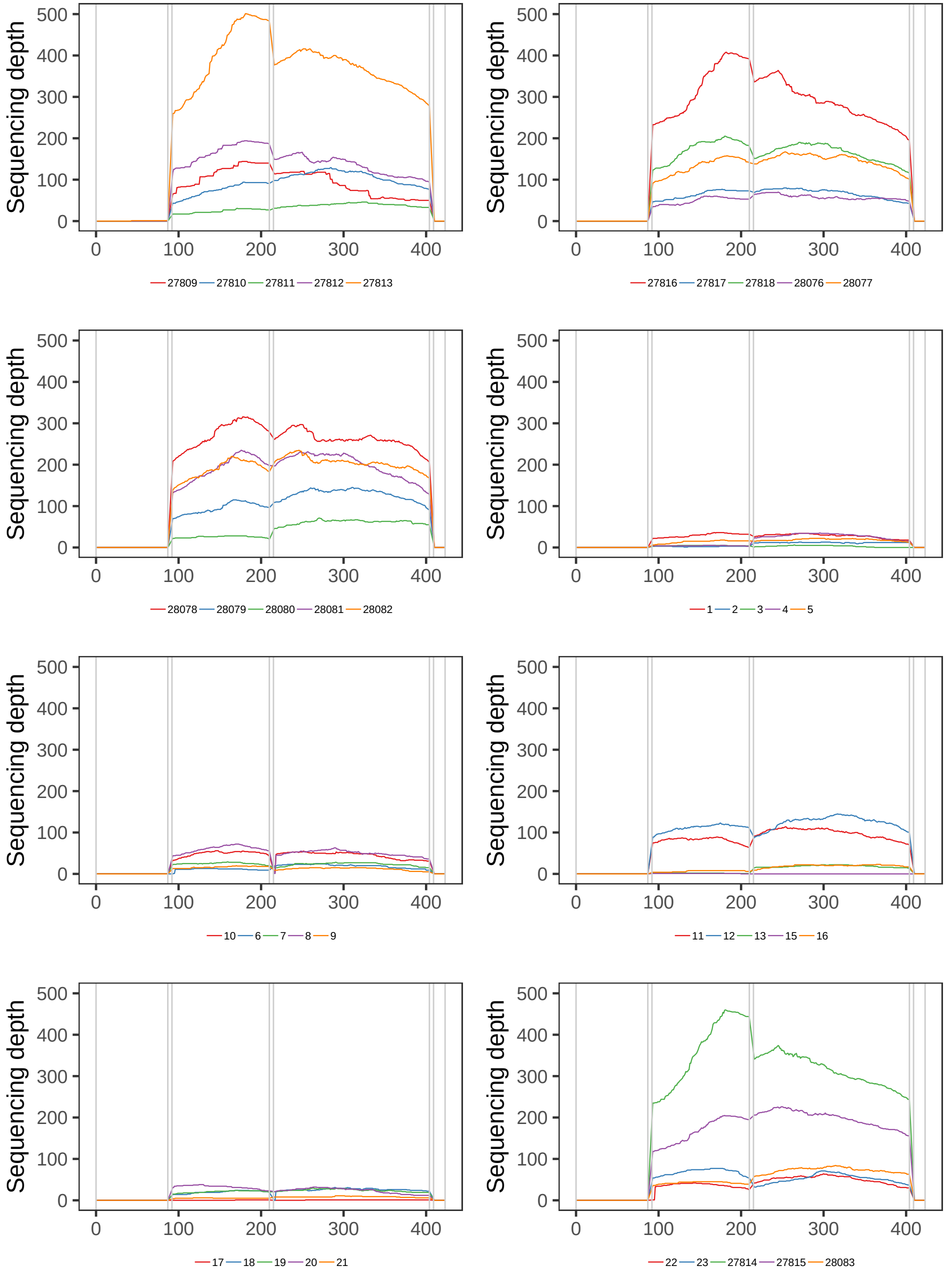
Sequencing depth
0
100
200
300
400
500
27809 27810 27811 27812 27813
27816 27817 27818 28076 28077
28078 28079 28080 28081 28082
1 2 3 4 5
10 6 7 8 9
11 12 13 15 16
17 18 19 20 21
22 23 27814 27815 28083

EOG59CNQM

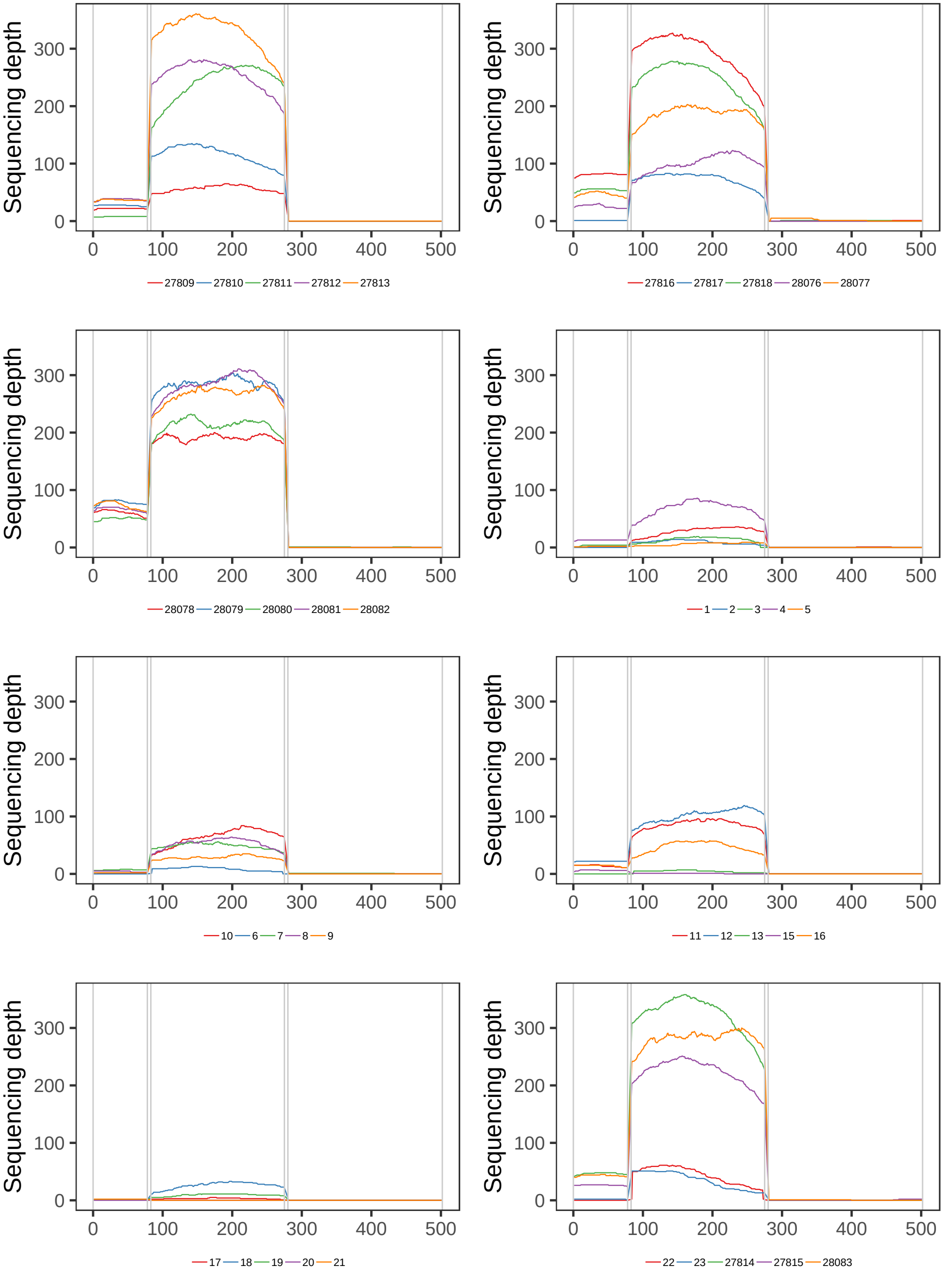
Sequencing depth
0
100
200
300
0
100
200
300
400
500
27809
27810
27811
27812
27813
27816
27817
27818
28076
28077
28078
28079
28080
28081
28082
1
2
3
4
5
10
6
7
8
9
11
12
13
15
16
17
18
19
20
21
22
23
27814
27815
28083

EOG5NP5M5

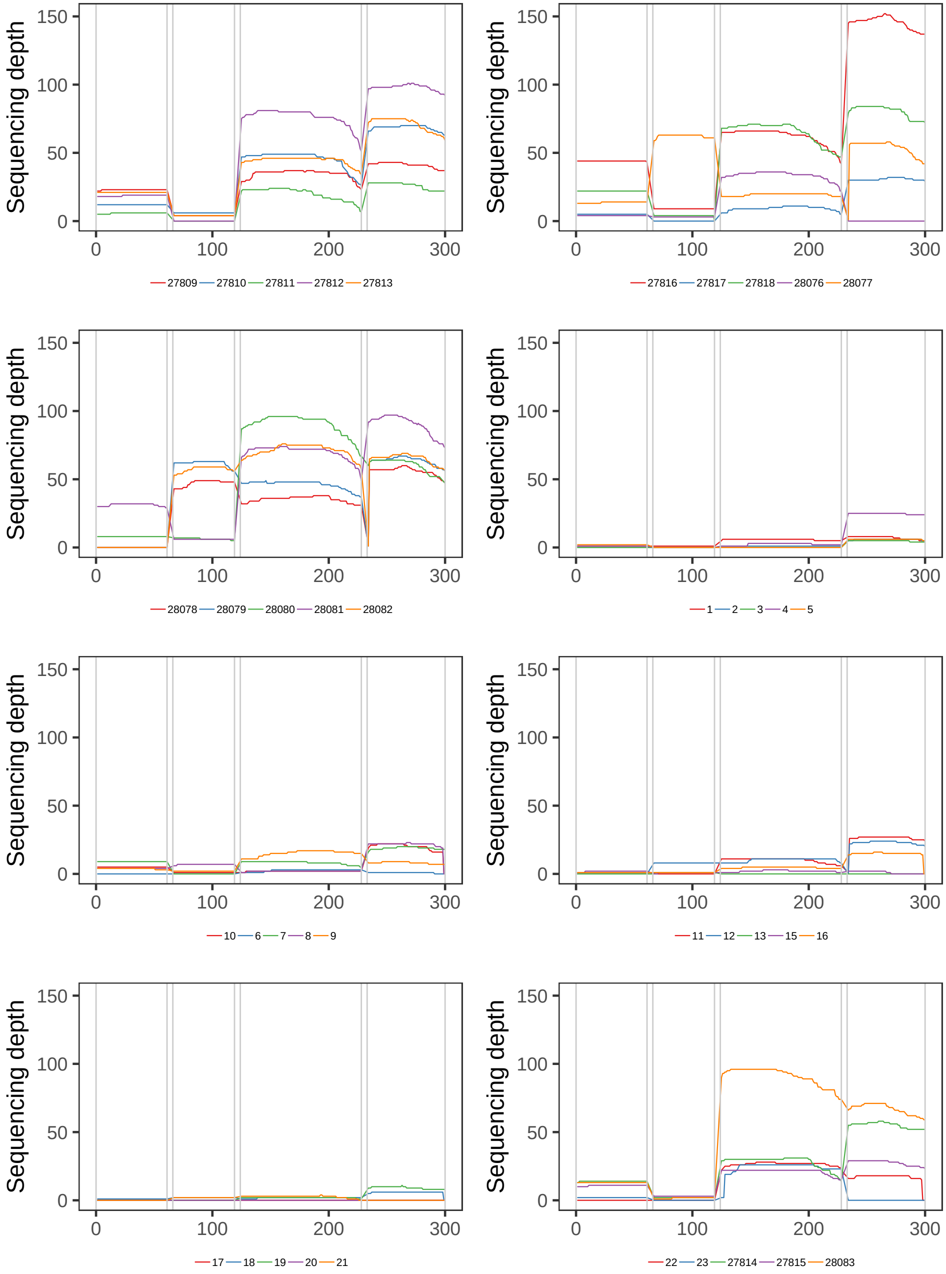
Sequencing depth
0
50
100
150
0
100
200
300
27809 27810 27811 27812 27813
27816 27817 27818 28076 28077
28078 28079 28080 28081 28082
1 2 3 4 5
10 6 7 8 9
11 12 13 15 16
17 18 19 20 21
22 23 27814 27815 28083

EOG5PNVZM

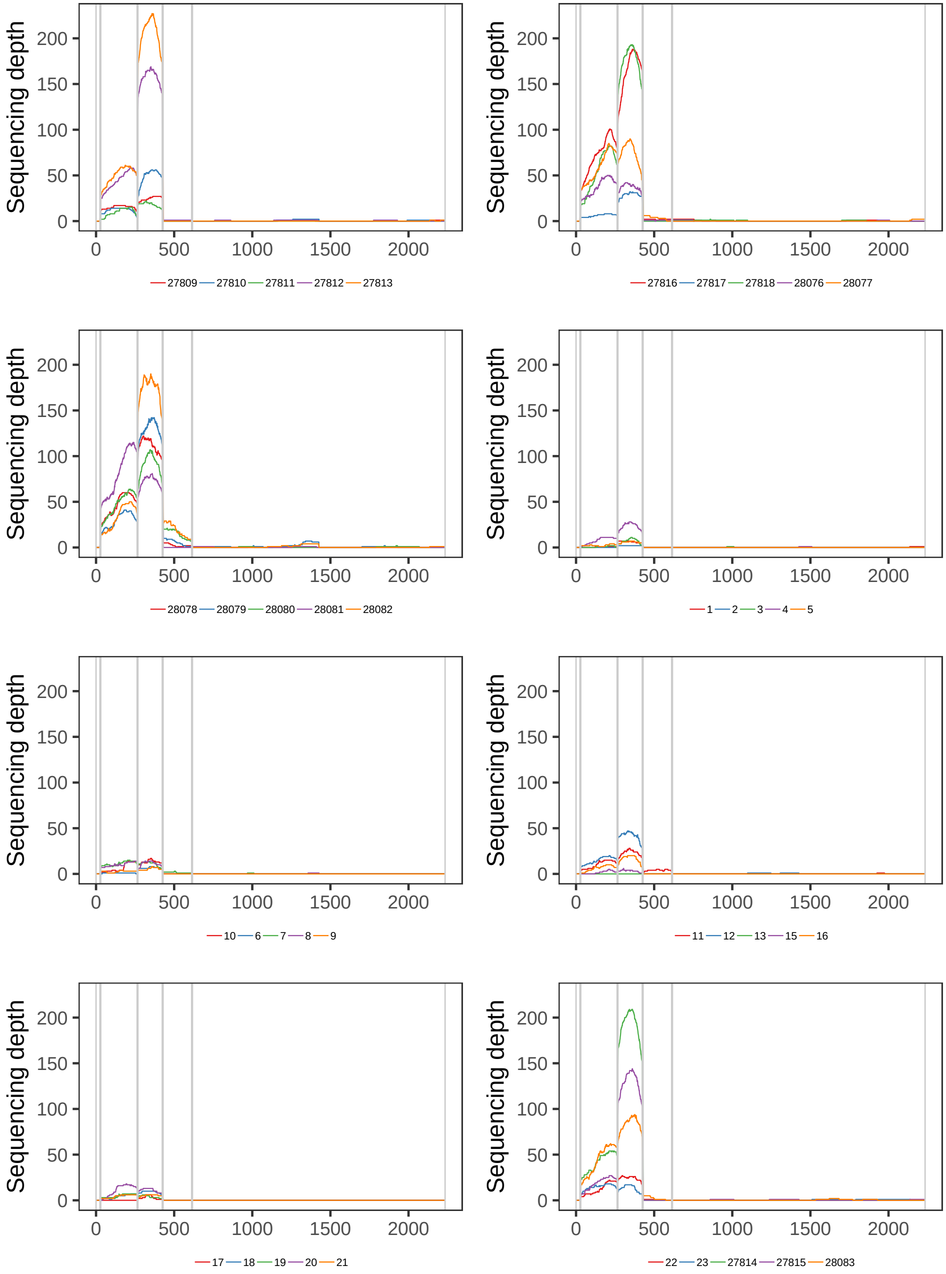

Sequencing depth
0
50
100
150
200
0
500
1000
1500
2000
27809 27810 27811 27812 27813
27816 27817 27818 28076 28077
28078 28079 28080 28081 28082
1 2 3 4 5
10 6 7 8 9
11 12 13 15 16
17 18 19 20 21
22 23 27814 27815 28083

EOG5QBZNK

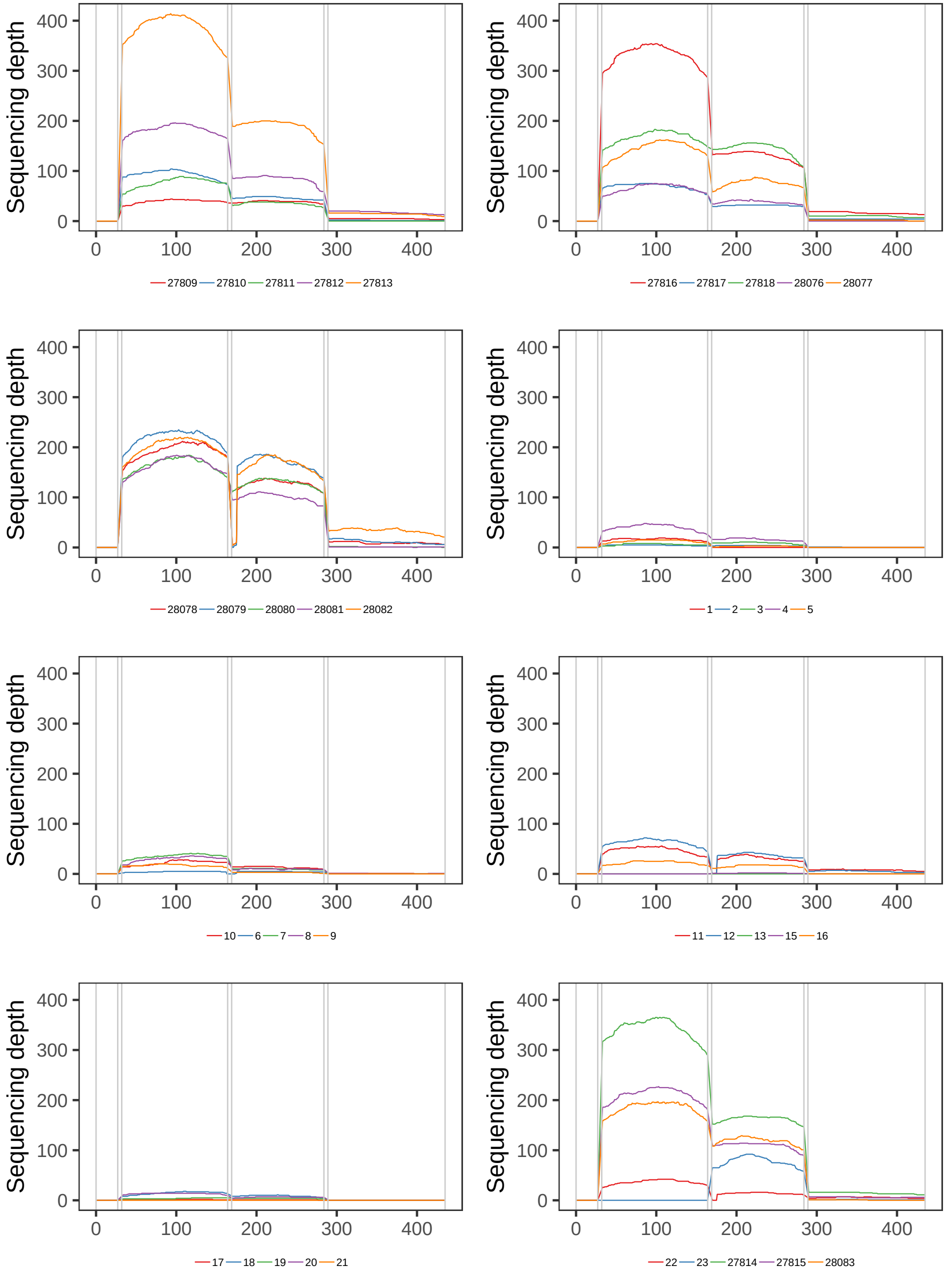

Sequencing depth
0
100
200
300
400
27809 27810 27811 27812 27813
27816 27817 27818 28076 28077
28078 28079 28080 28081 28082
1 2 3 4 5
10 6 7 8 9
11 12 13 15 16
17 18 19 20 21
22 23 27814 27815 28083

EOG52JM7K

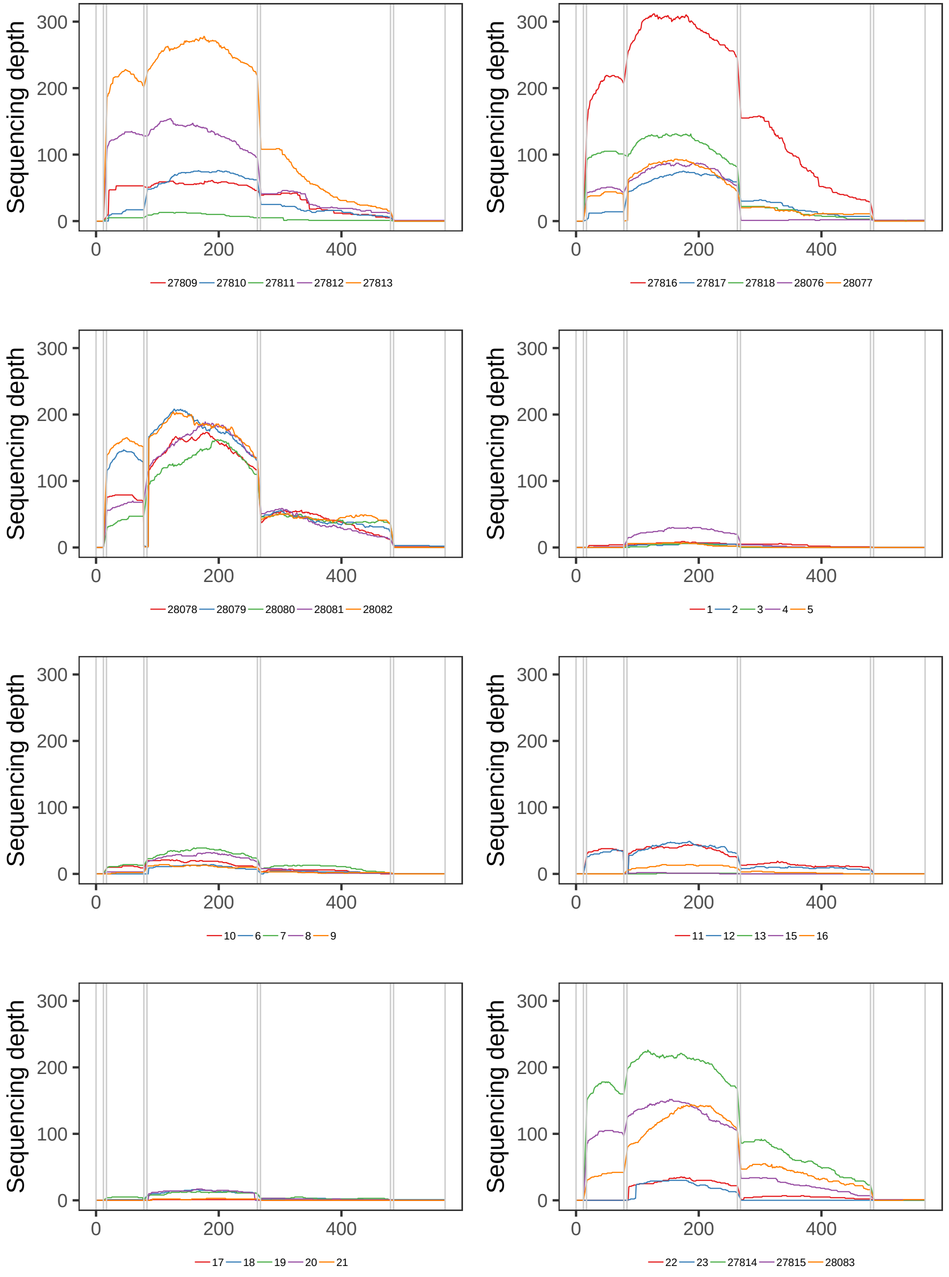
Sequencing depth
0
100
200
300
0
200
400
27809 27810 27811 27812 27813
27816 27817 27818 28076 28077
28078 28079 28080 28081 28082
1 2 3 4 5
10 6 7 8 9
11 12 13 15 16
17 18 19 20 21
22 23 27814 27815 28083

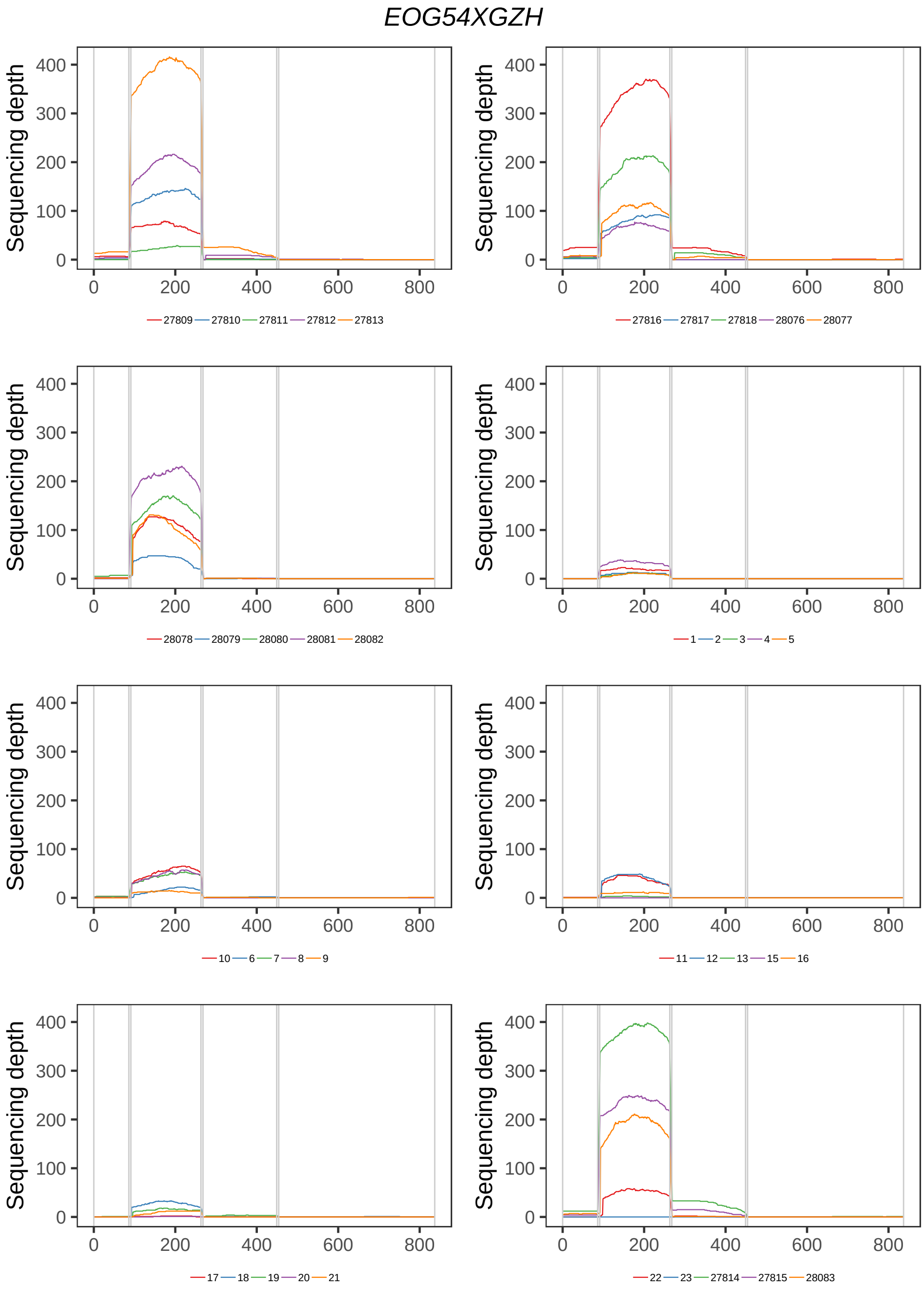
EOG54XGZH
Sequencing depth
27809 27810 27811 27812 27813
27816 27817 27818 28076 28077
28078 28079 28080 28081 28082
1 2 3 4 5
10 6 7 8 9
11 12 13 15 16
17 18 19 20 21
22 23 27814 27815 28083

EOG57D7Z3

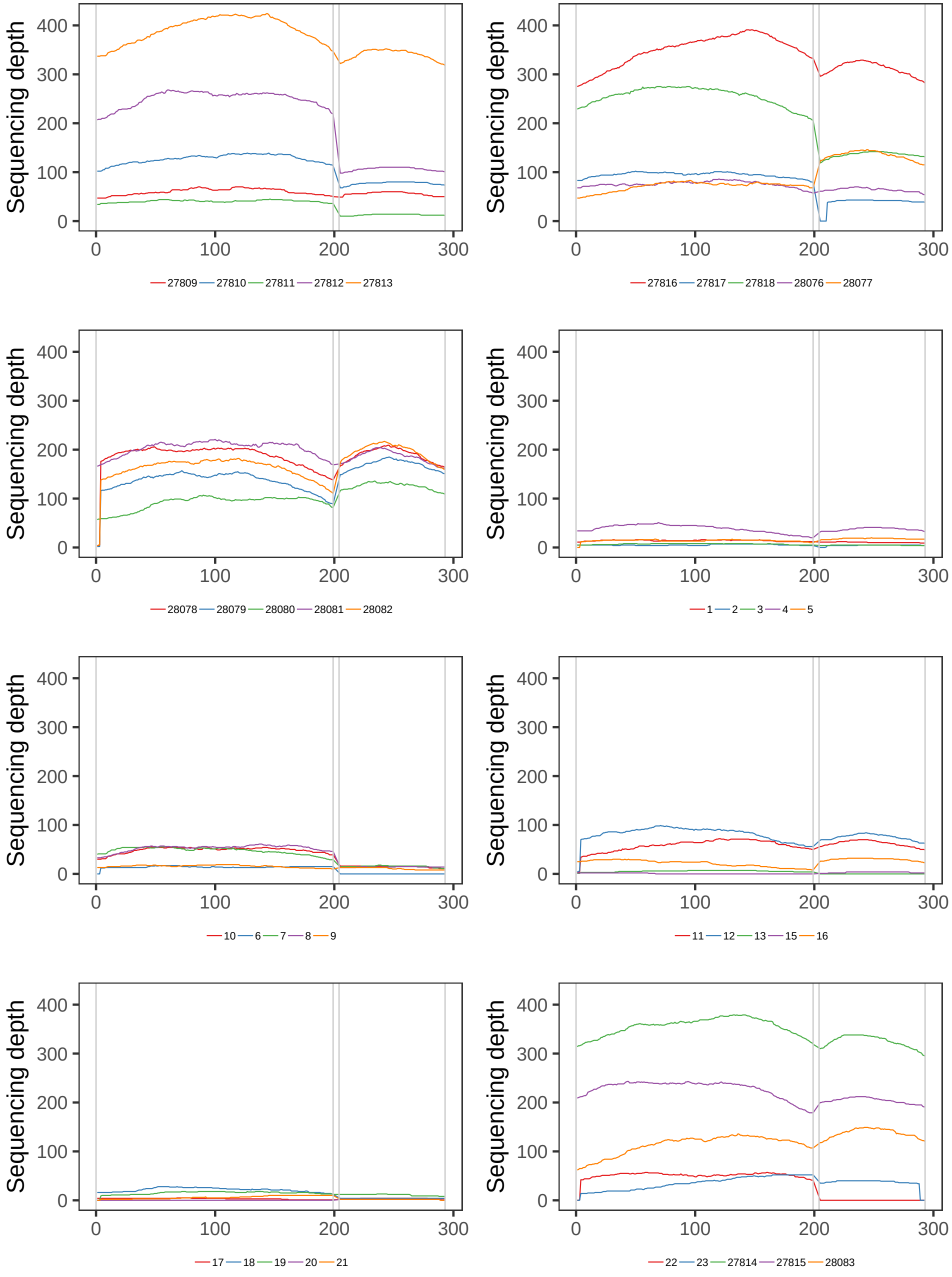
Sequencing depth
0
100
200
300
400
27809 27810 27811 27812 27813
27816 27817 27818 28076 28077
28078 28079 28080 28081 28082
1 2 3 4 5
10 6 7 8 9
11 12 13 15 16
17 18 19 20 21
22 23 27814 27815 28083

EOG59GHZP

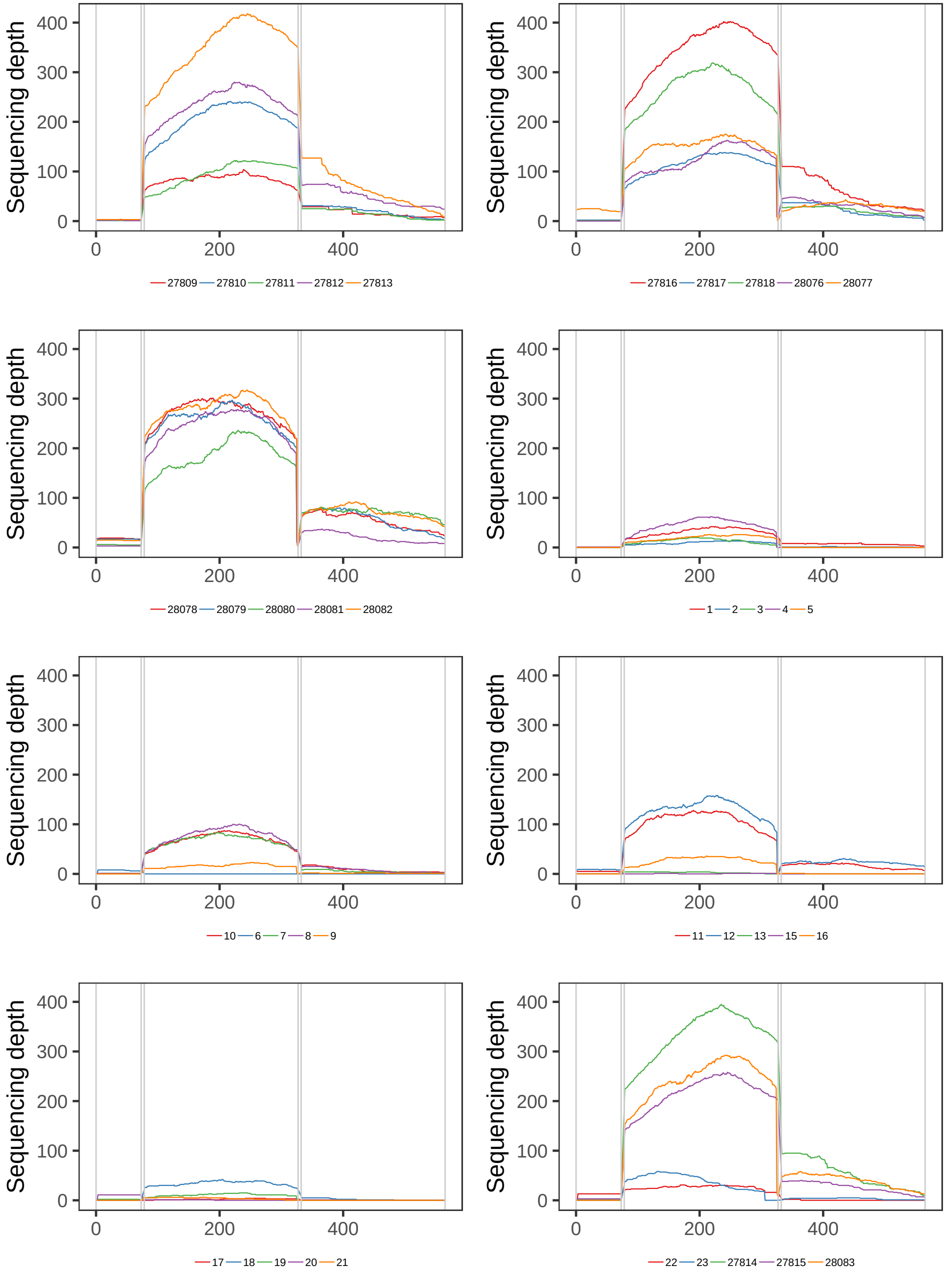

Sequencing depth
0
100
200
300
400
0
200
400
27809
27810
27811
27812
27813
27816
27817
27818
28076
28077
28078
28079
28080
28081
28082
1
2
3
4
5
10
6
7
8
9
11
12
13
15
16
17
18
19
20
21
22
23
27814
27815
28083

EOG5CVDP8

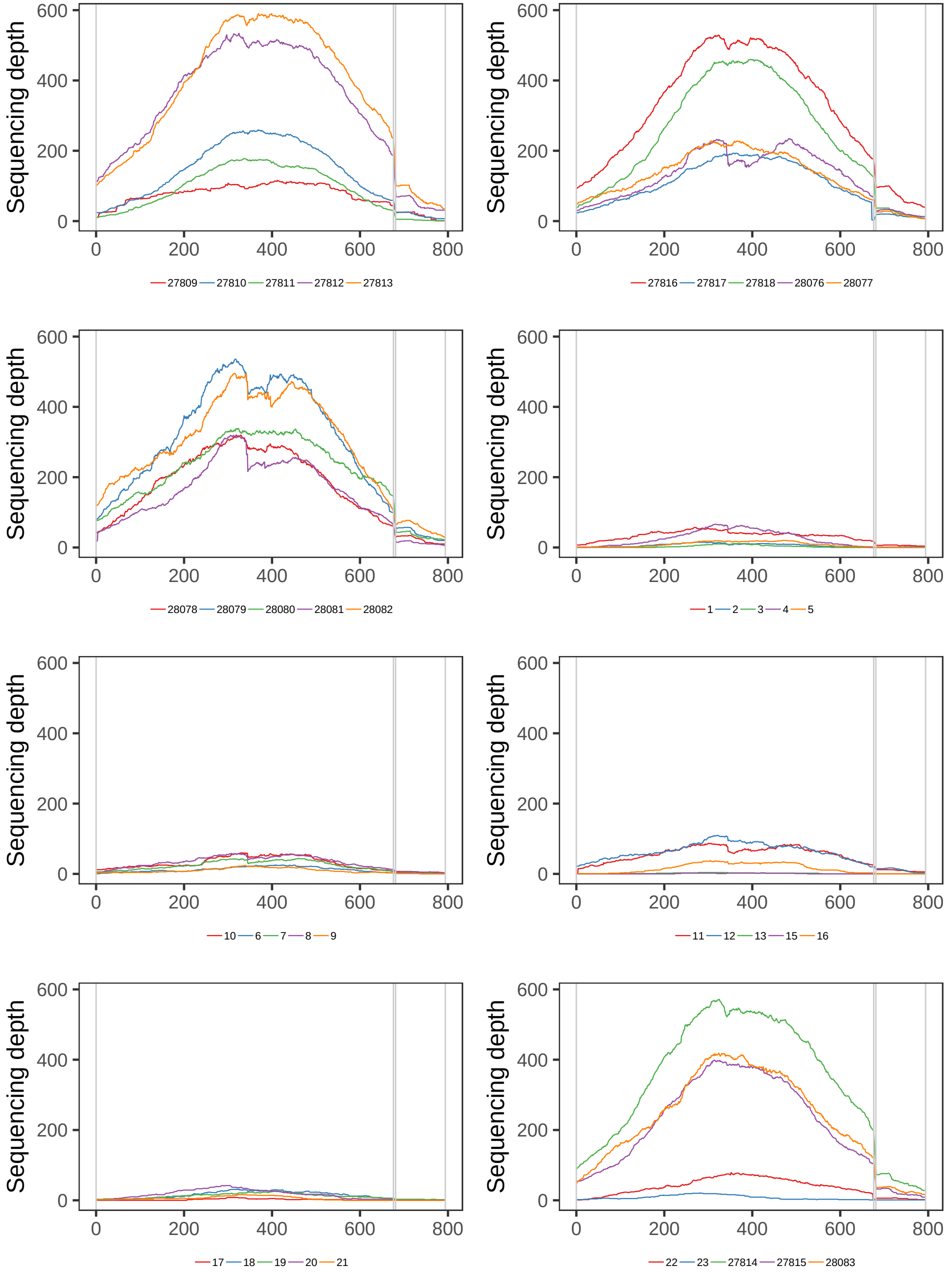
Sequencing depth
600
400
200
0
0
200
400
600
800
27809 27810 27811 27812 27813
27816 27817 27818 28076 28077
28078 28079 28080 28081 28082
1 2 3 4 5
10 6 7 8 9
11 12 13 15 16
17 18 19 20 21
22 23 27814 27815 28083

EOG5D254W

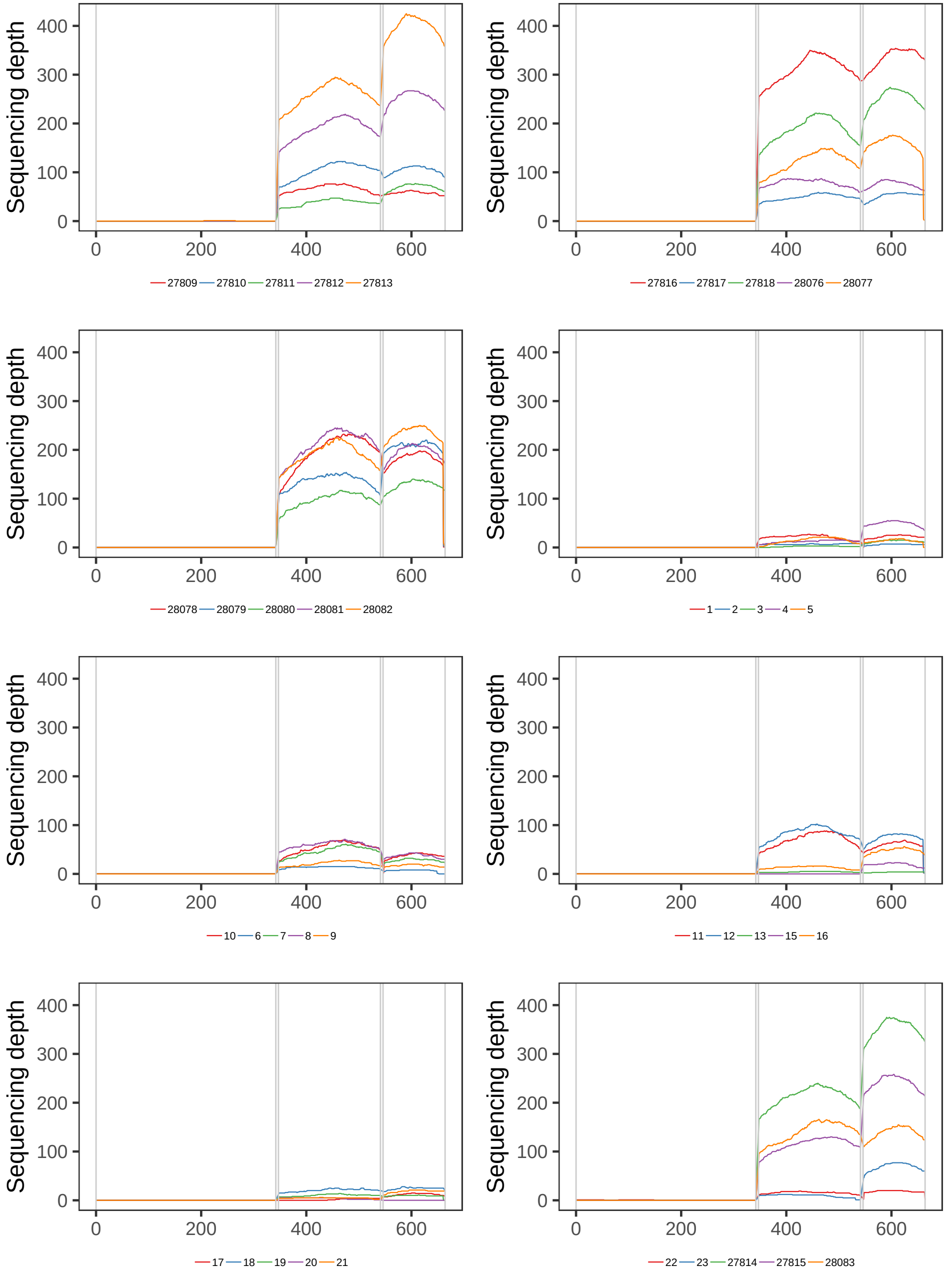
Sequencing depth
0
100
200
300
400
0
200
400
600
27809 27810 27811 27812 27813
27816 27817 27818 28076 28077
28078 28079 28080 28081 28082
1 2 3 4 5
10 6 7 8 9
11 12 13 15 16
17 18 19 20 21
22 23 27814 27815 28083

EOG5J9KFZ

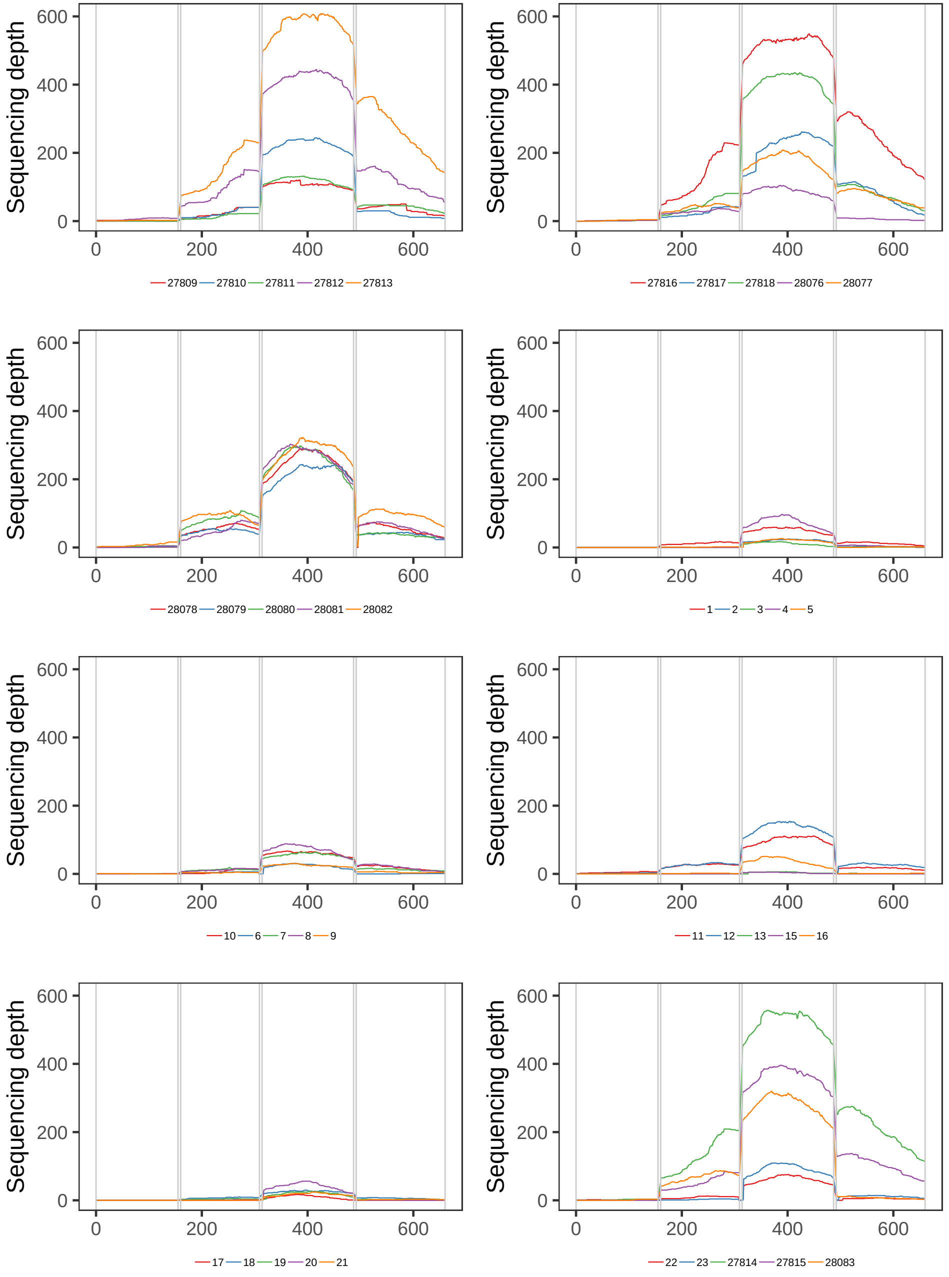
Sequencing depth
0
200
400
600
27809 27810 27811 27812 27813
27816 27817 27818 28076 28077
28078 28079 28080 28081 28082
1 2 3 4 5
10 6 7 8 9
11 12 13 15 16
17 18 19 20 21
22 23 27814 27815 28083

EOG5RV17Z

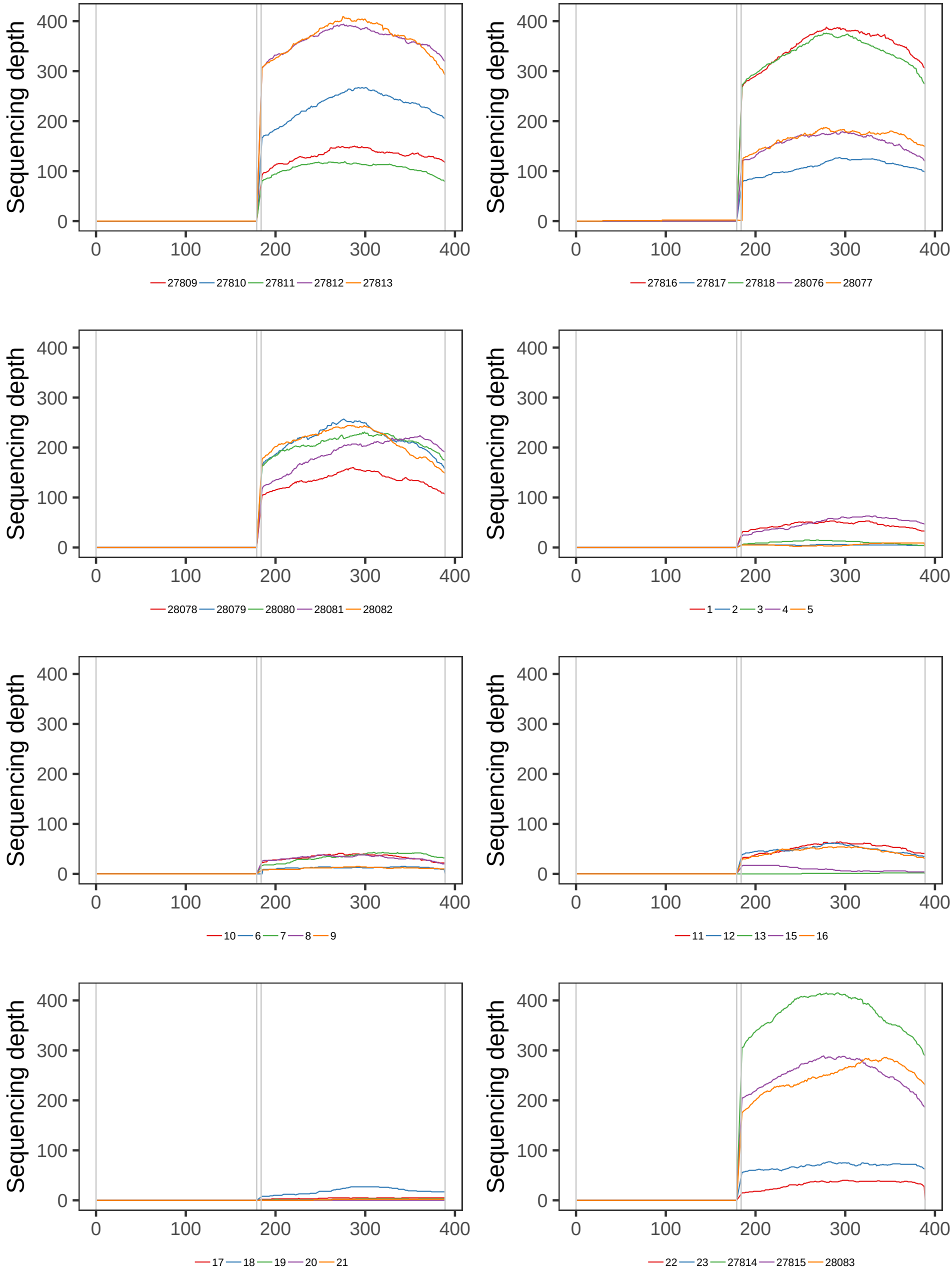

Sequencing depth
0
100
200
300
400
27809 27810 27811 27812 27813
27816 27817 27818 28076 28077
28078 28079 28080 28081 28082
1 2 3 4 5
10 6 7 8 9
11 12 13 15 16
17 18 19 20 21
22 23 27814 27815 28083

EOG5RXWFT

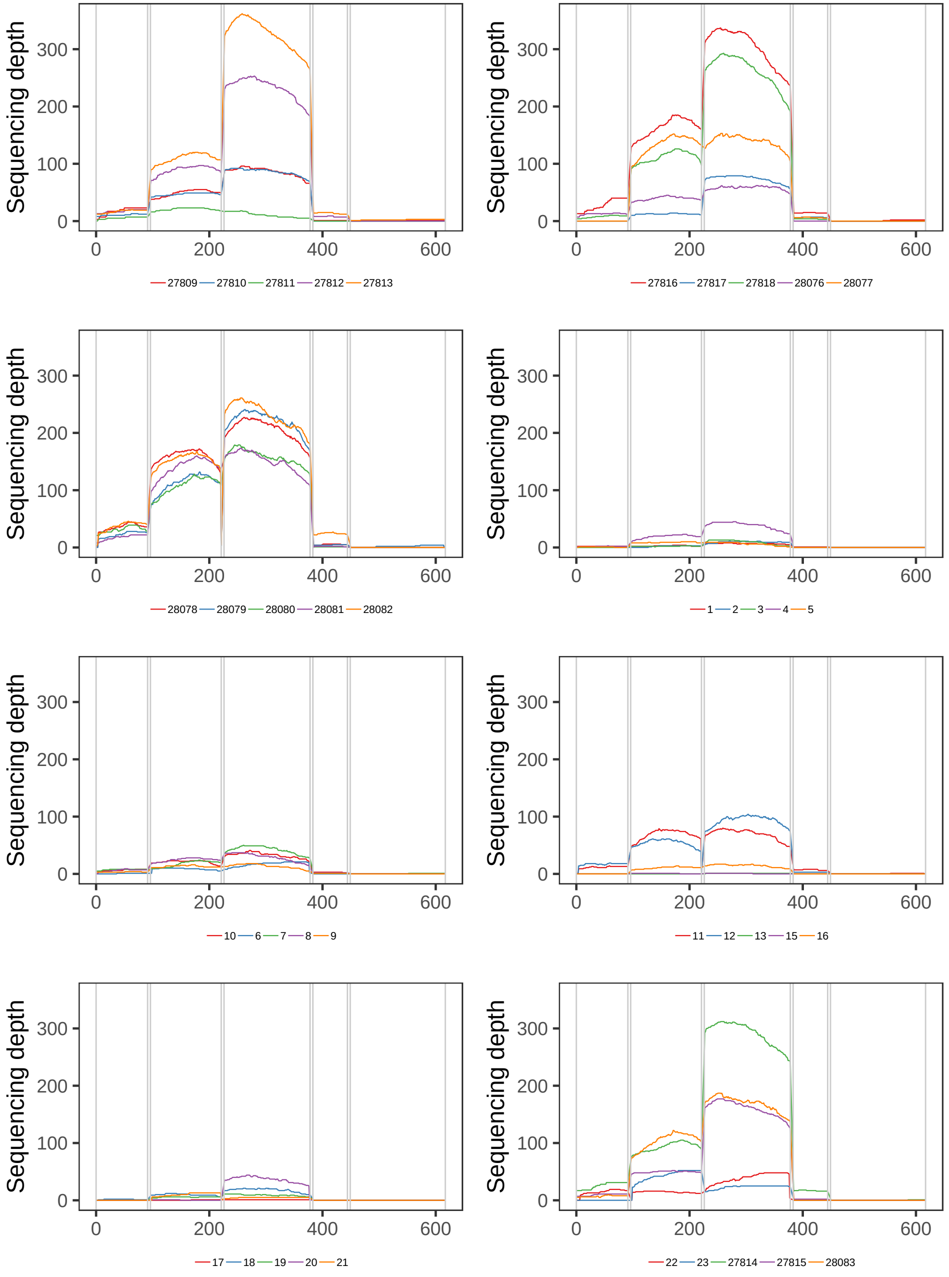
Sequencing depth
300
200
100
0
0
200
400
600
27809 27810 27811 27812 27813
27816 27817 27818 28076 28077
28078 28079 28080 28081 28082
1 2 3 4 5
10 6 7 8 9
11 12 13 15 16
17 18 19 20 21
22 23 27814 27815 28083

EOG5XD27P

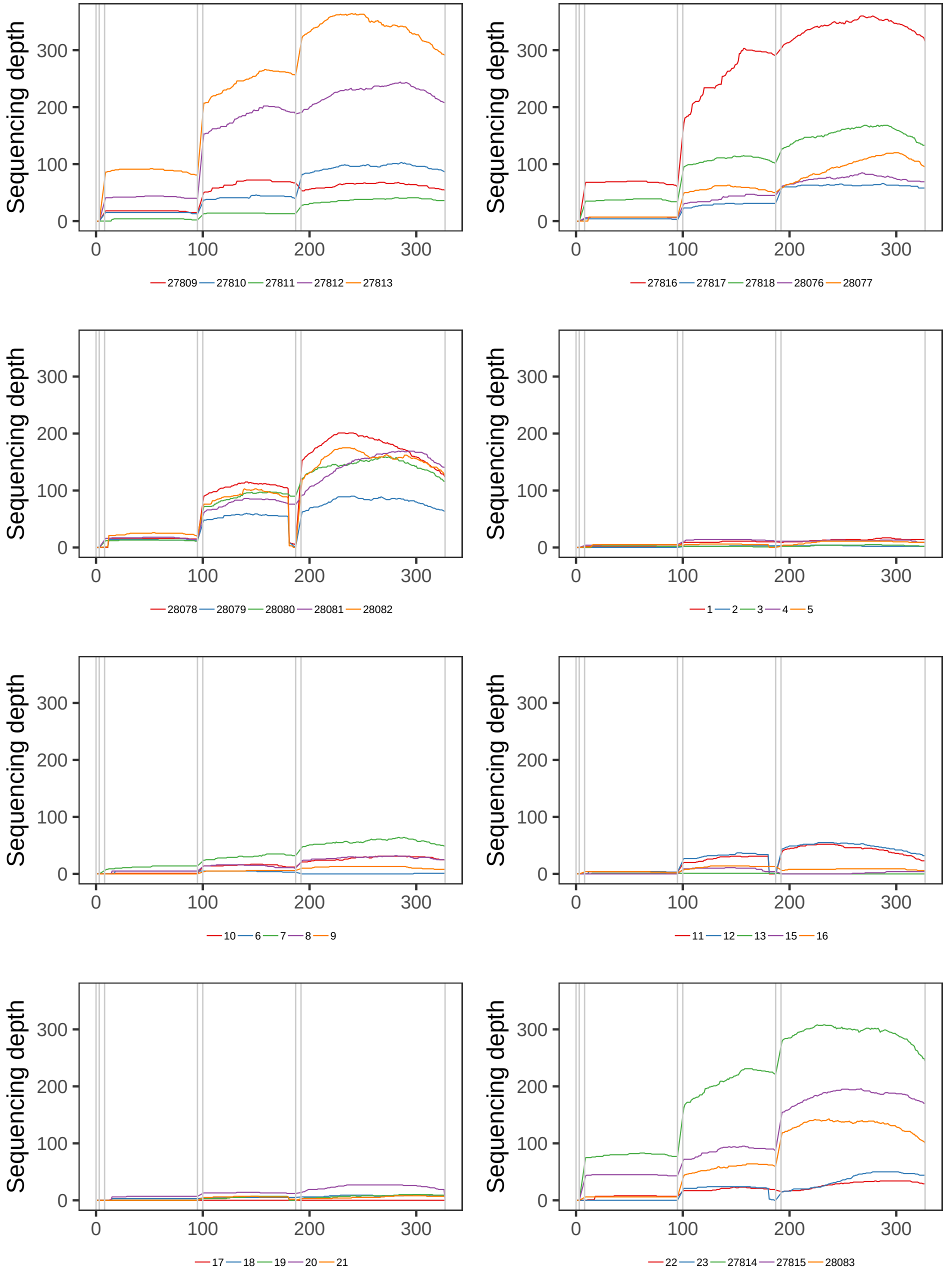

Sequencing depth
0
100
200
300
27809 27810 27811 27812 27813
27816 27817 27818 28076 28077
28078 28079 28080 28081 28082
1 2 3 4 5
10 6 7 8 9
11 12 13 15 16
17 18 19 20 21
22 23 27814 27815 28083

EOG5ZGMV9

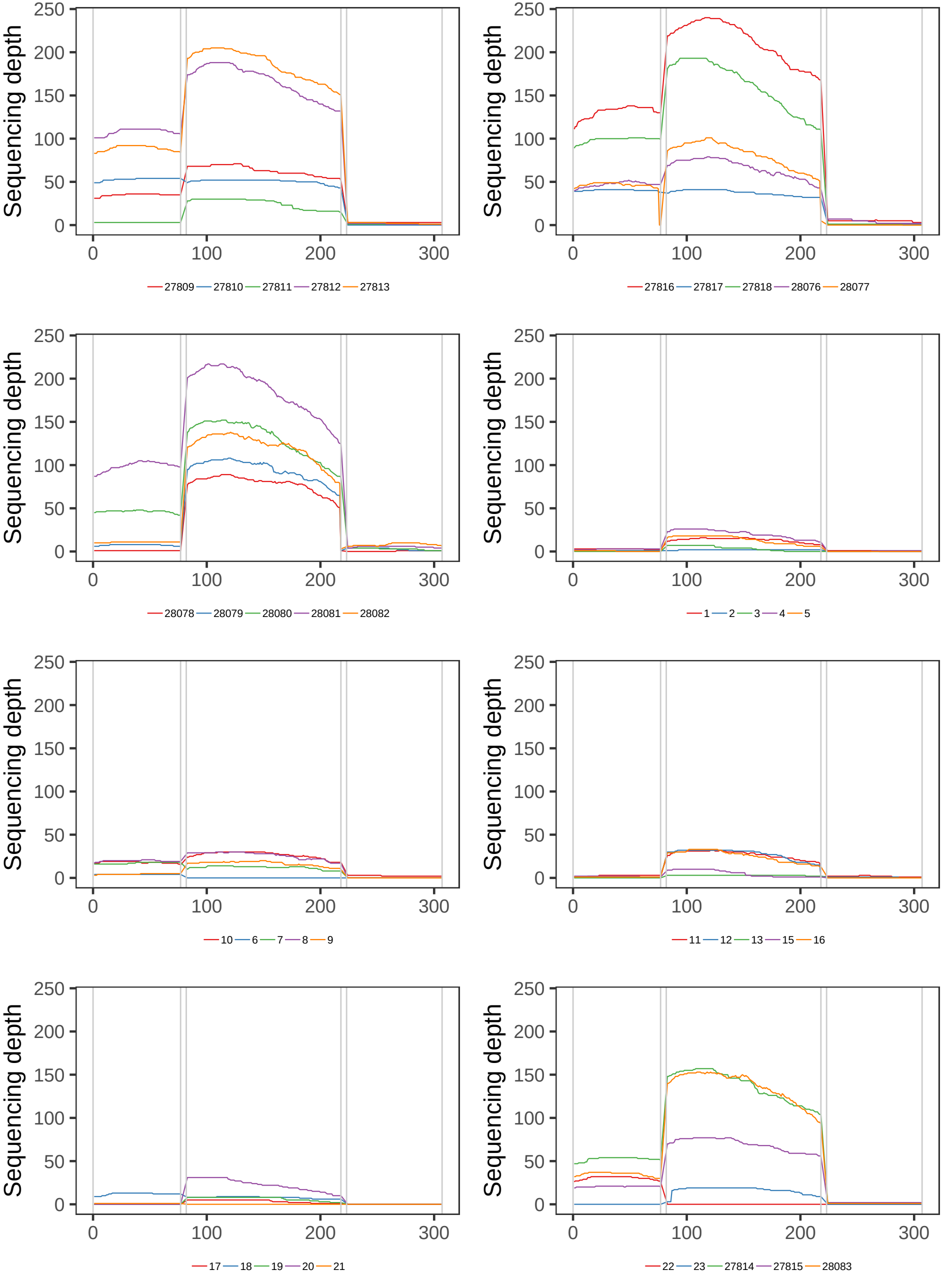
Sequencing depth
0
50
100
150
200
250
0
100
200
300
27809 27810 27811 27812 27813
27816 27817 27818 28076 28077
28078 28079 28080 28081 28082
1 2 3 4 5
10 6 7 8 9
11 12 13 15 16
17 18 19 20 21
22 23 27814 27815 28083

EOG54TMRB

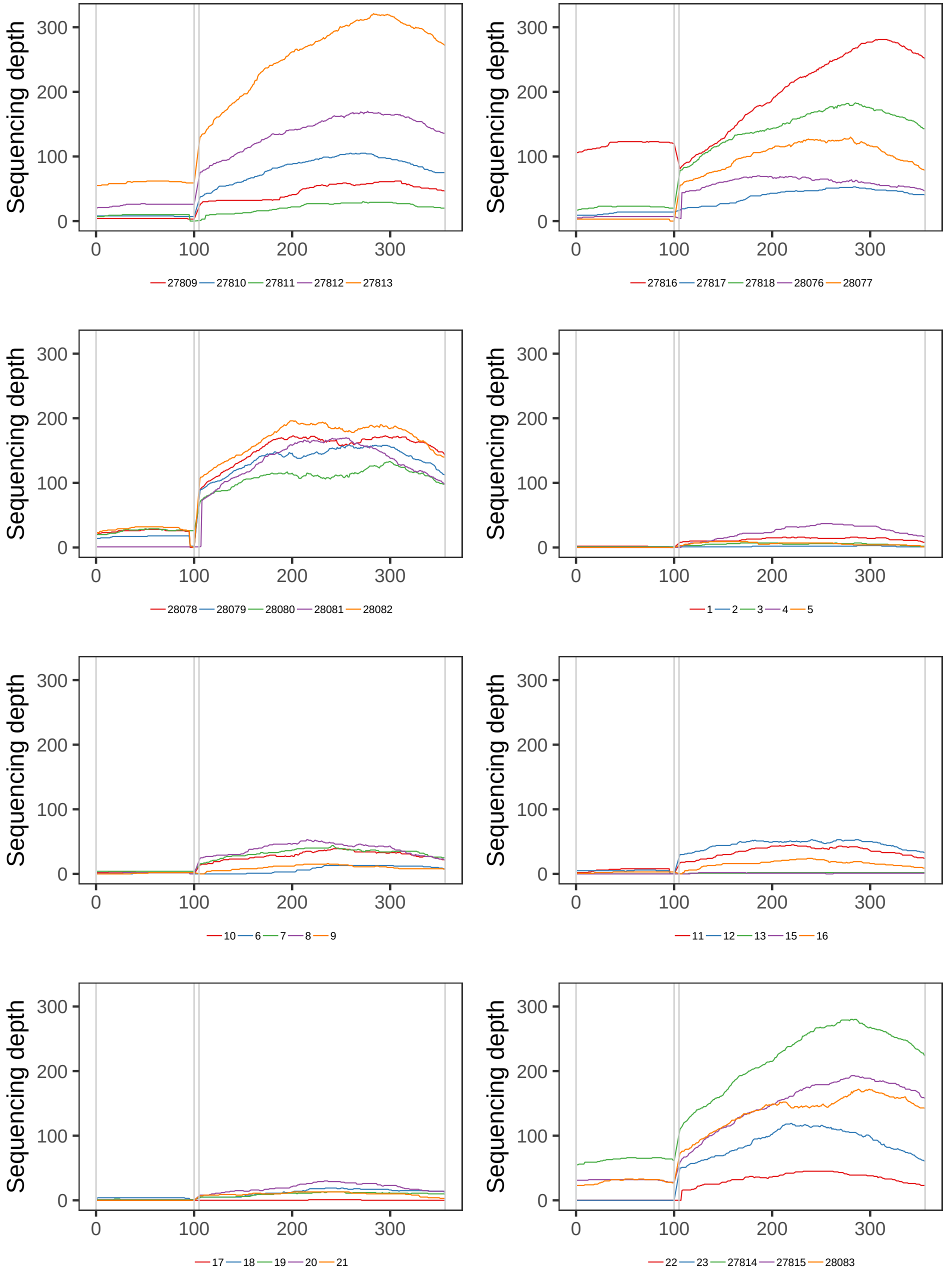
Sequencing depth
0
100
200
300
27809 27810 27811 27812 27813
27816 27817 27818 28076 28077
28078 28079 28080 28081 28082
1 2 3 4 5
10 6 7 8 9
11 12 13 15 16
17 18 19 20 21
22 23 27814 27815 28083

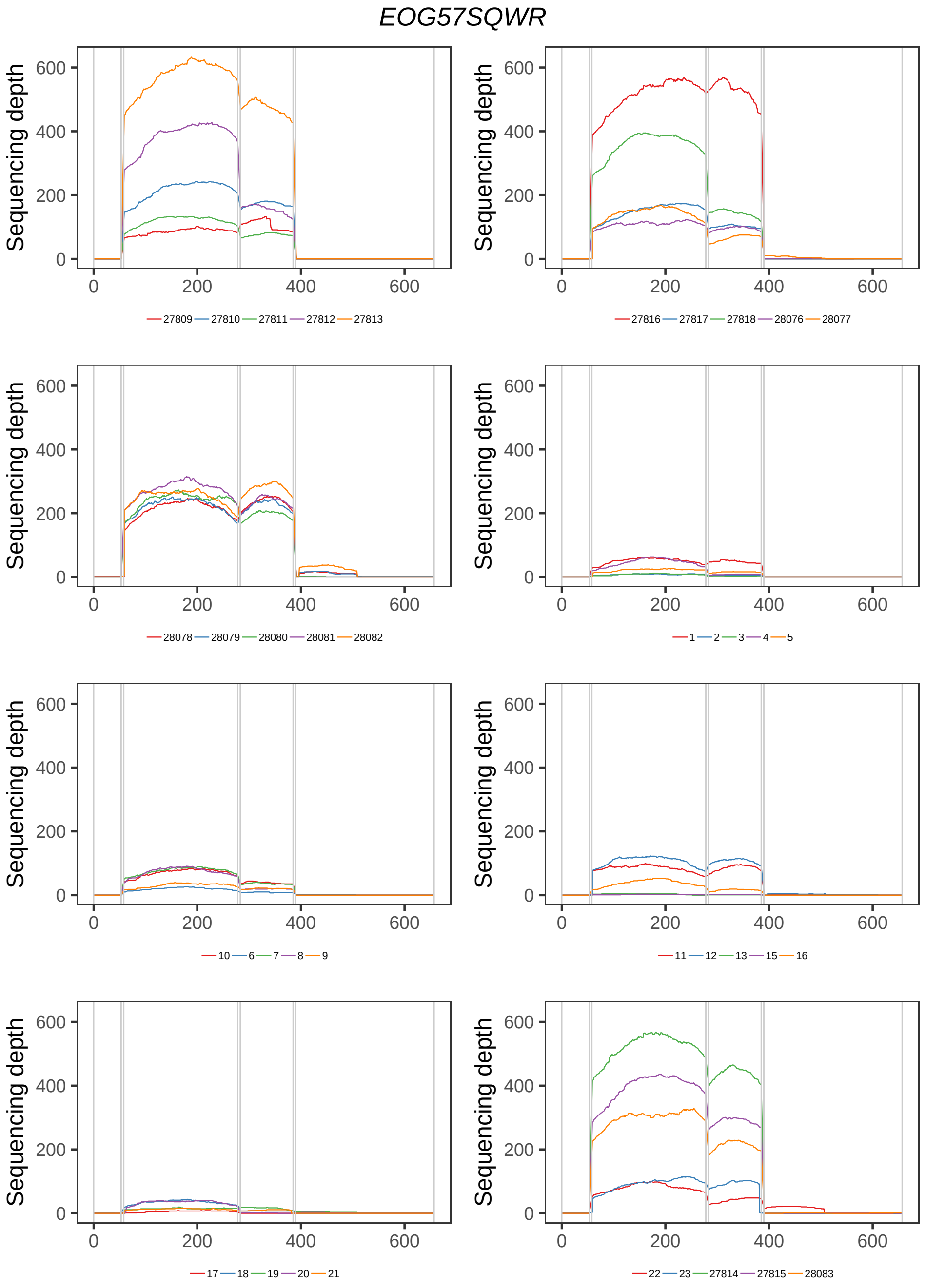

EOG57SQWR
Sequencing depth
0
200
400
600
27809 27810 27811 27812 27813
27816 27817 27818 28076 28077
28078 28079 28080 28081 28082
1 2 3 4 5
10 6 7 8 9
11 12 13 15 16
17 18 19 20 21
22 23 27814 27815 28083

EOG58PK1Z

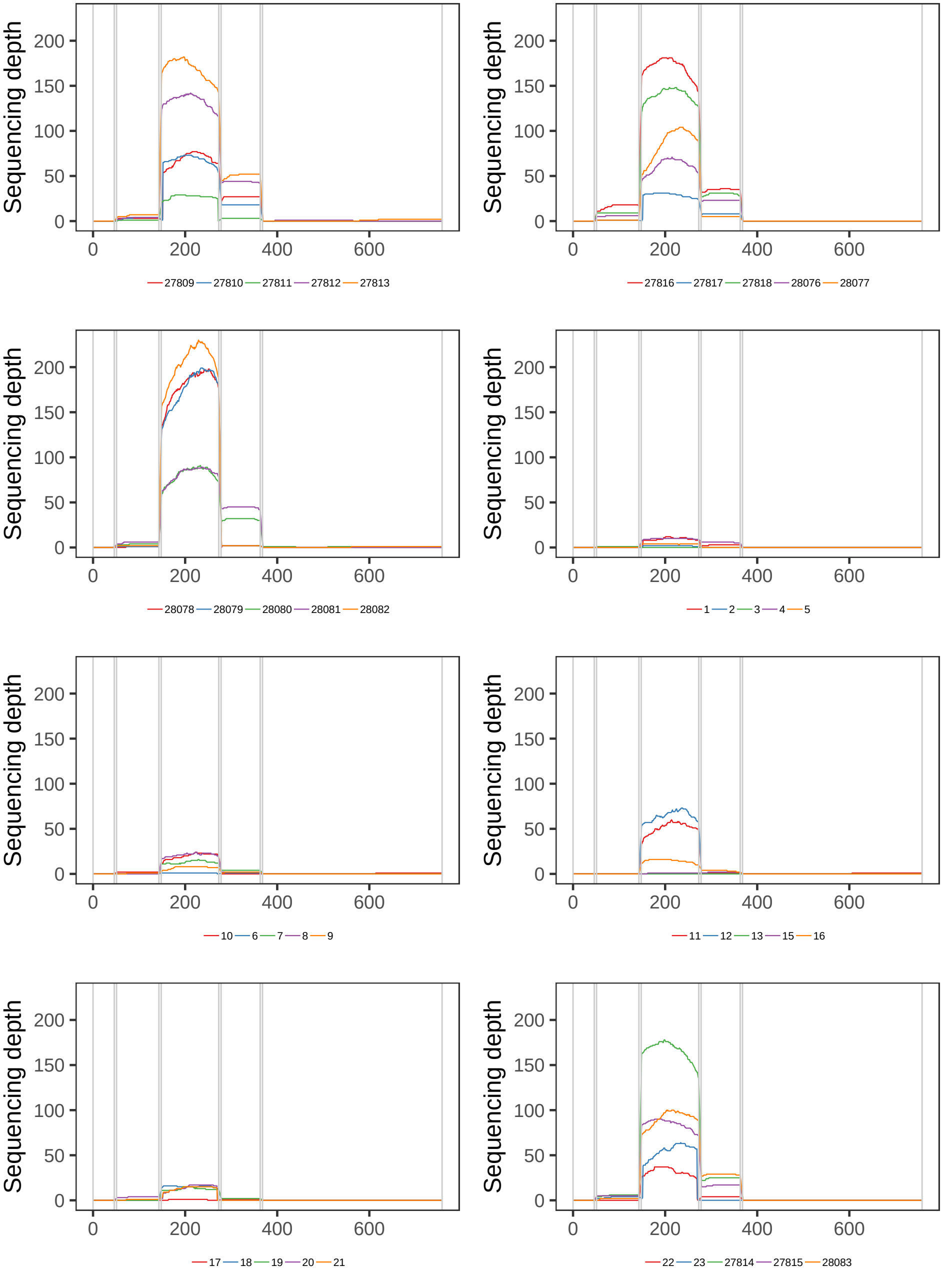
Sequencing depth
0
50
100
150
200
0
200
400
600
27809 27810 27811 27812 27813
27816 27817 27818 28076 28077
28078 28079 28080 28081 28082
1 2 3 4 5
10 6 7 8 9
11 12 13 15 16
17 18 19 20 21
22 23 27814 27815 28083

EOG5HMGS1

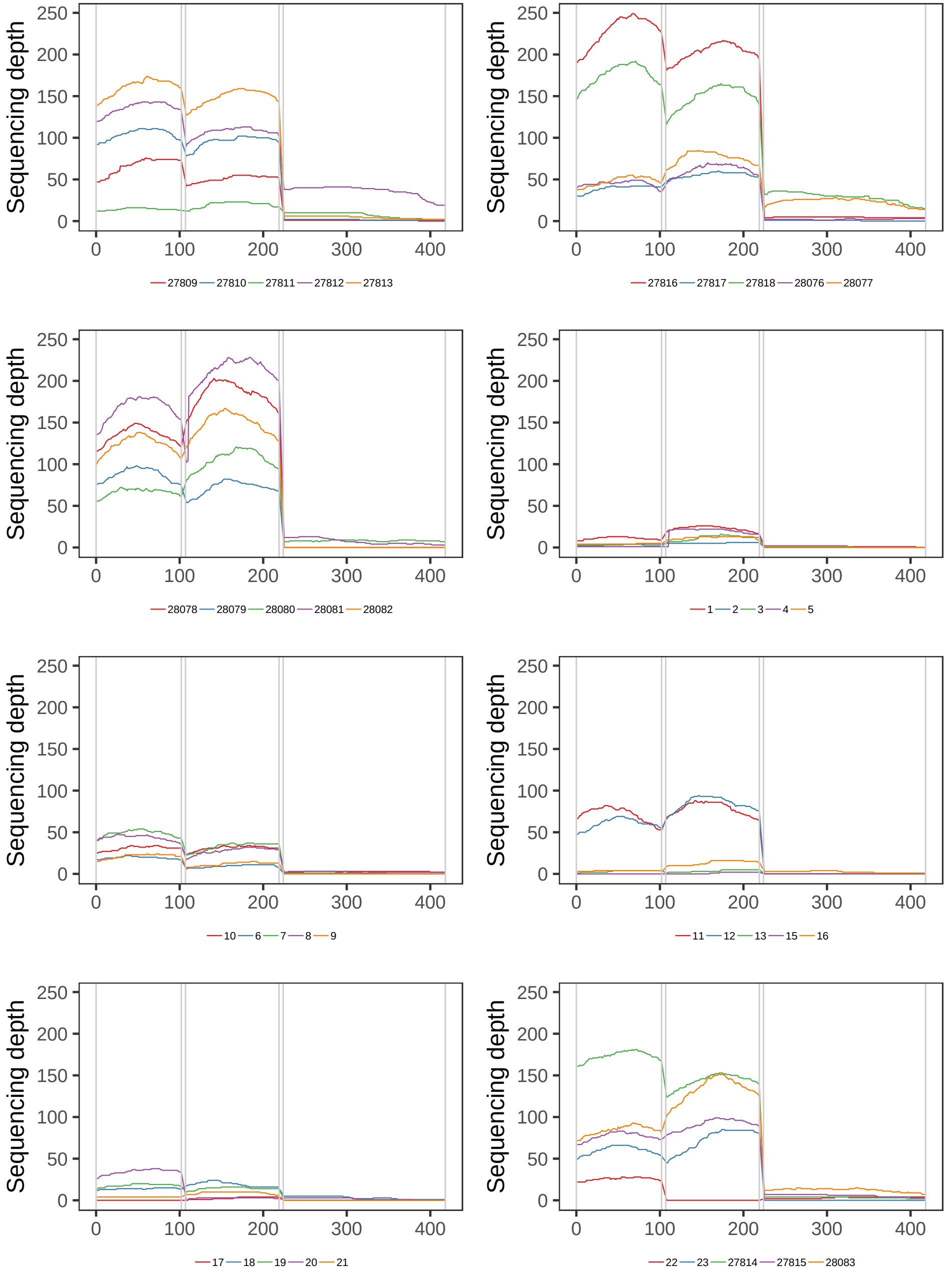
Sequencing depth
250
200
150
100
50
0
0
100
200
300
400
27809 27810 27811 27812 27813
27816 27817 27818 28076 28077
28078 28079 28080 28081 28082
1 2 3 4 5
10 6 7 8 9
11 12 13 15 16
17 18 19 20 21
22 23 27814 27815 28083

EOG5QBZNS

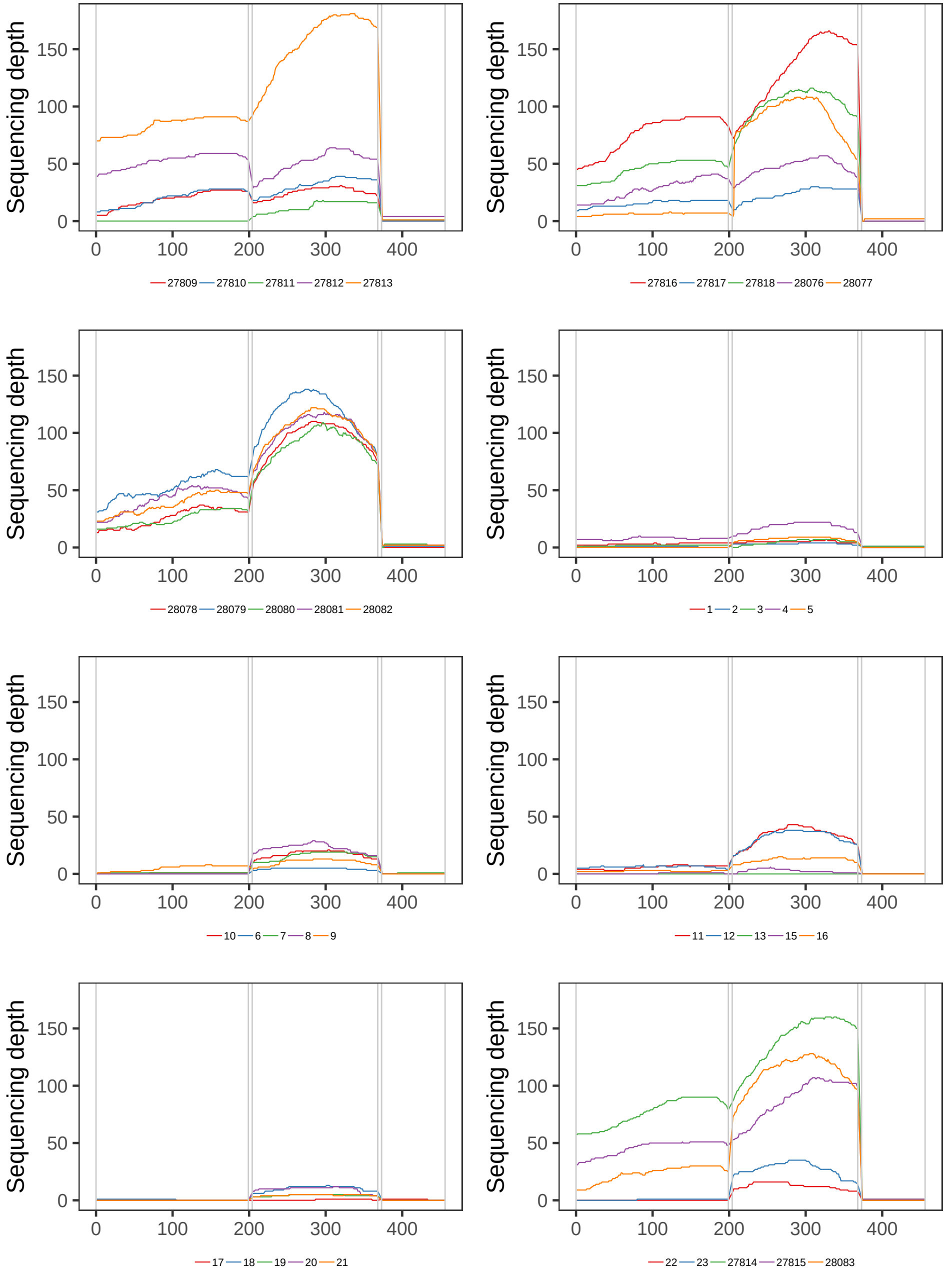
Sequencing depth
0
50
100
150
0
100
200
300
400
27809 27810 27811 27812 27813
27816 27817 27818 28076 28077
28078 28079 28080 28081 28082
1 2 3 4 5
10 6 7 8 9
11 12 13 15 16
17 18 19 20 21
22 23 27814 27815 28083

EOG5QJQ42

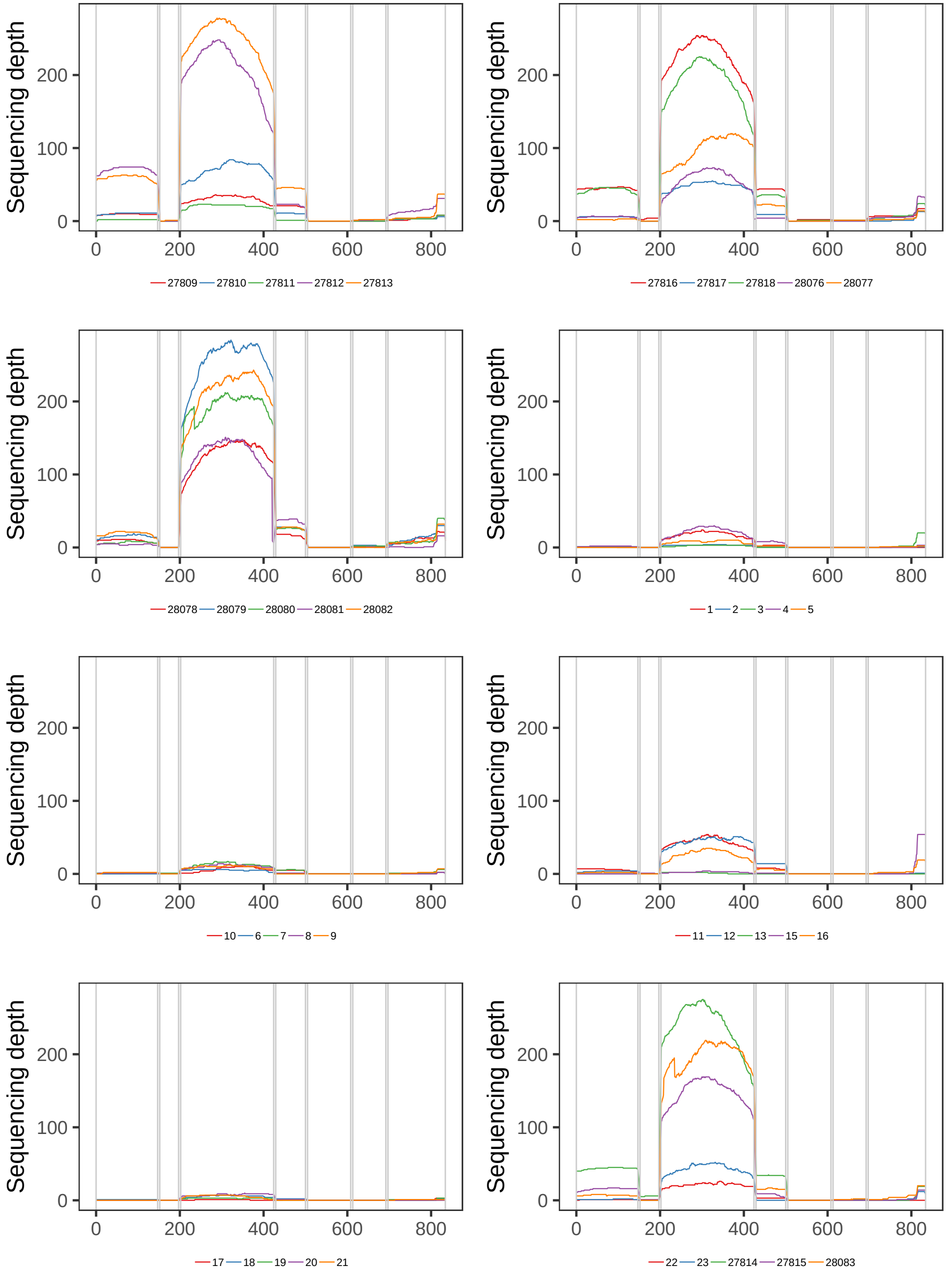
Sequencing depth
0
100
200
0
200
400
600
800
27809
27810
27811
27812
27813
27816
27817
27818
28076
28077
28078
28079
28080
28081
28082
1
2
3
4
5
10
6
7
8
9
11
12
13
15
16
17
18
19
20
21
22
23
27814
27815
28083

EOG5RN8RX

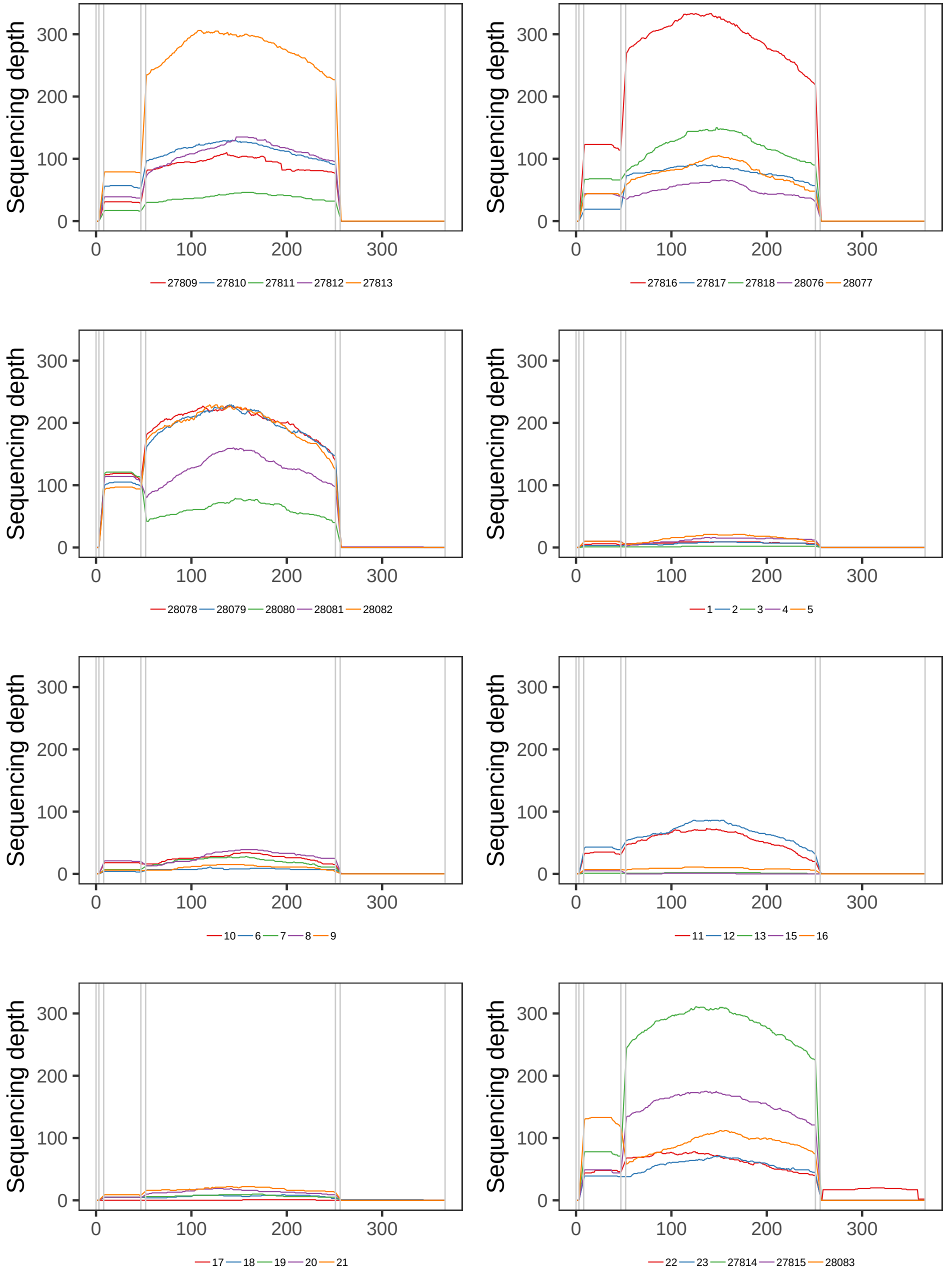

Sequencing depth
0
100
200
300
27809
27810
27811
27812
27813
27816
27817
27818
28076
28077
28078
28079
28080
28081
28082
1
2
3
4
5
10
6
7
8
9
11
12
13
15
16
17
18
19
20
21
22
23
27814
27815
28083

EOG5VX0N3

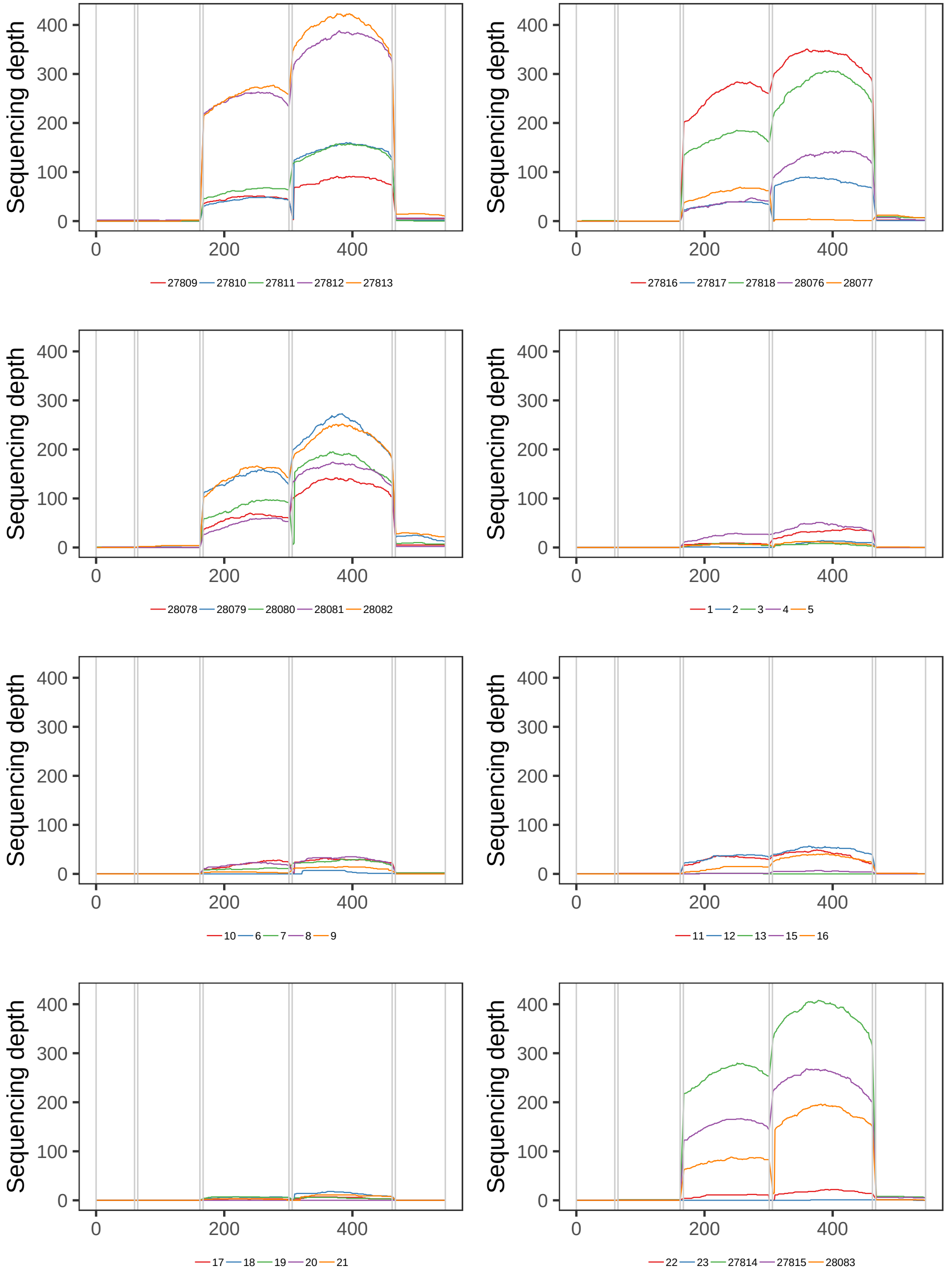
Sequencing depth
0
100
200
300
400
27809 27810 27811 27812 27813
27816 27817 27818 28076 28077
28078 28079 28080 28081 28082
1 2 3 4 5
10 6 7 8 9
11 12 13 15 16
17 18 19 20 21
22 23 27814 27815 28083

EOG5W3R3D

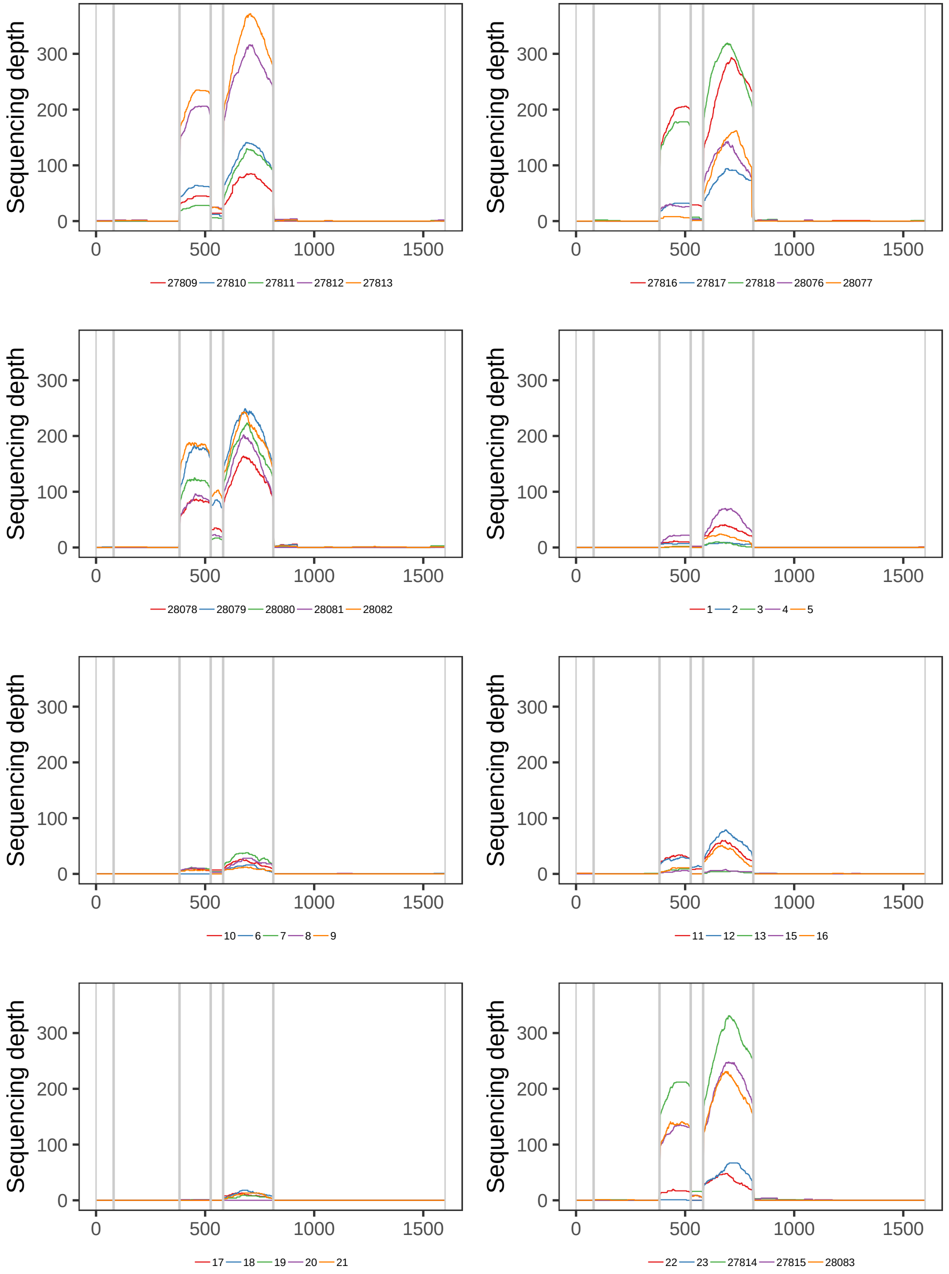
Sequencing depth
0
100
200
300
0
500
1000
1500
27809 27810 27811 27812 27813
27816 27817 27818 28076 28077
28078 28079 28080 28081 28082
1 2 3 4 5
10 6 7 8 9
11 12 13 15 16
17 18 19 20 21
22 23 27814 27815 28083

EOG5WWQ0Z

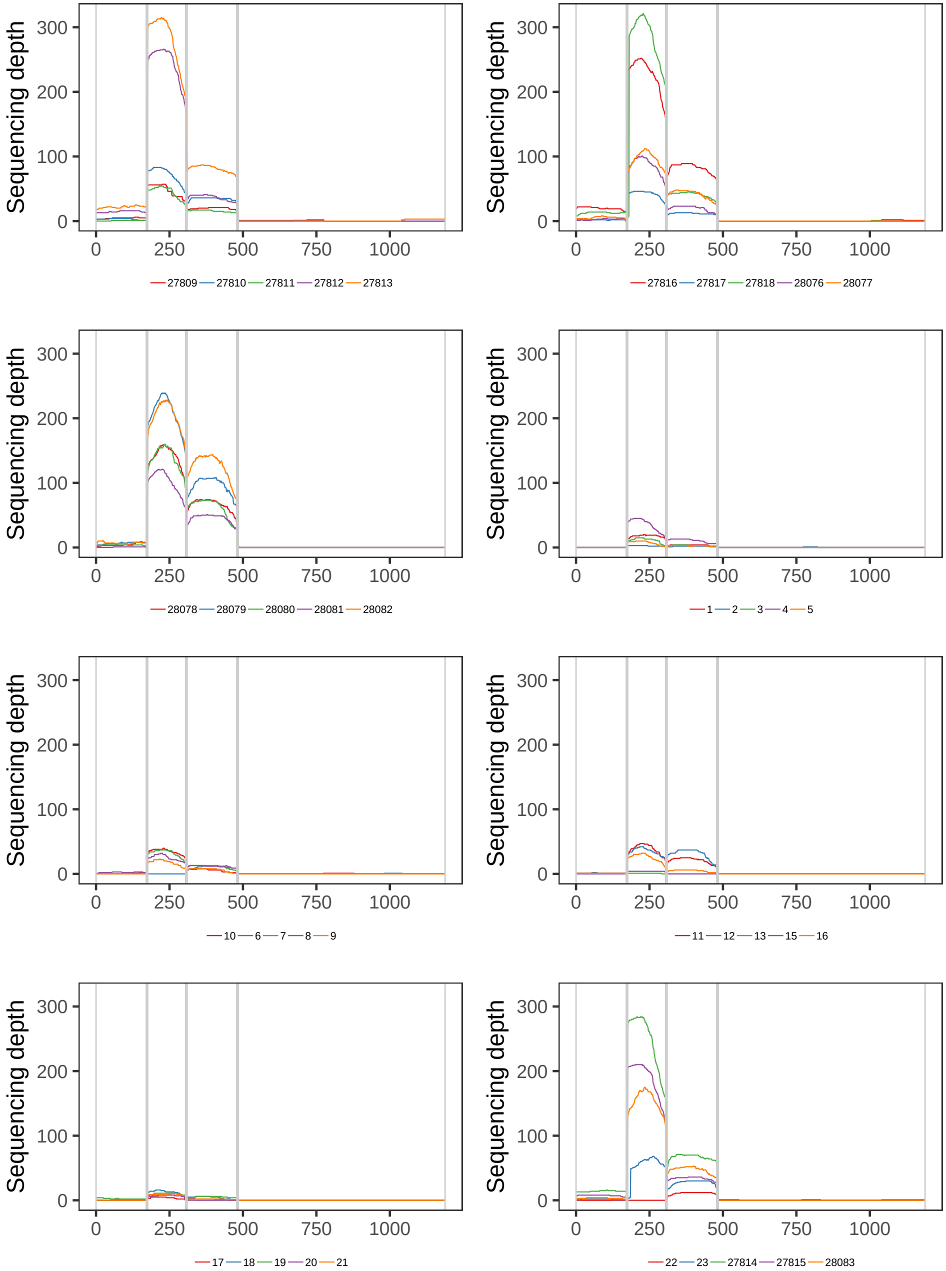
Sequencing depth
300
200
100
0
0
250
500
750
1000
27809
27810
27811
27812
27813
27816
27817
27818
28076
28077
28078
28079
28080
28081
28082
1
2
3
4
5
10
6
7
8
9
11
12
13
15
16
17
18
19
20
21
22
23
27814
27815
28083

EOG5X0K77

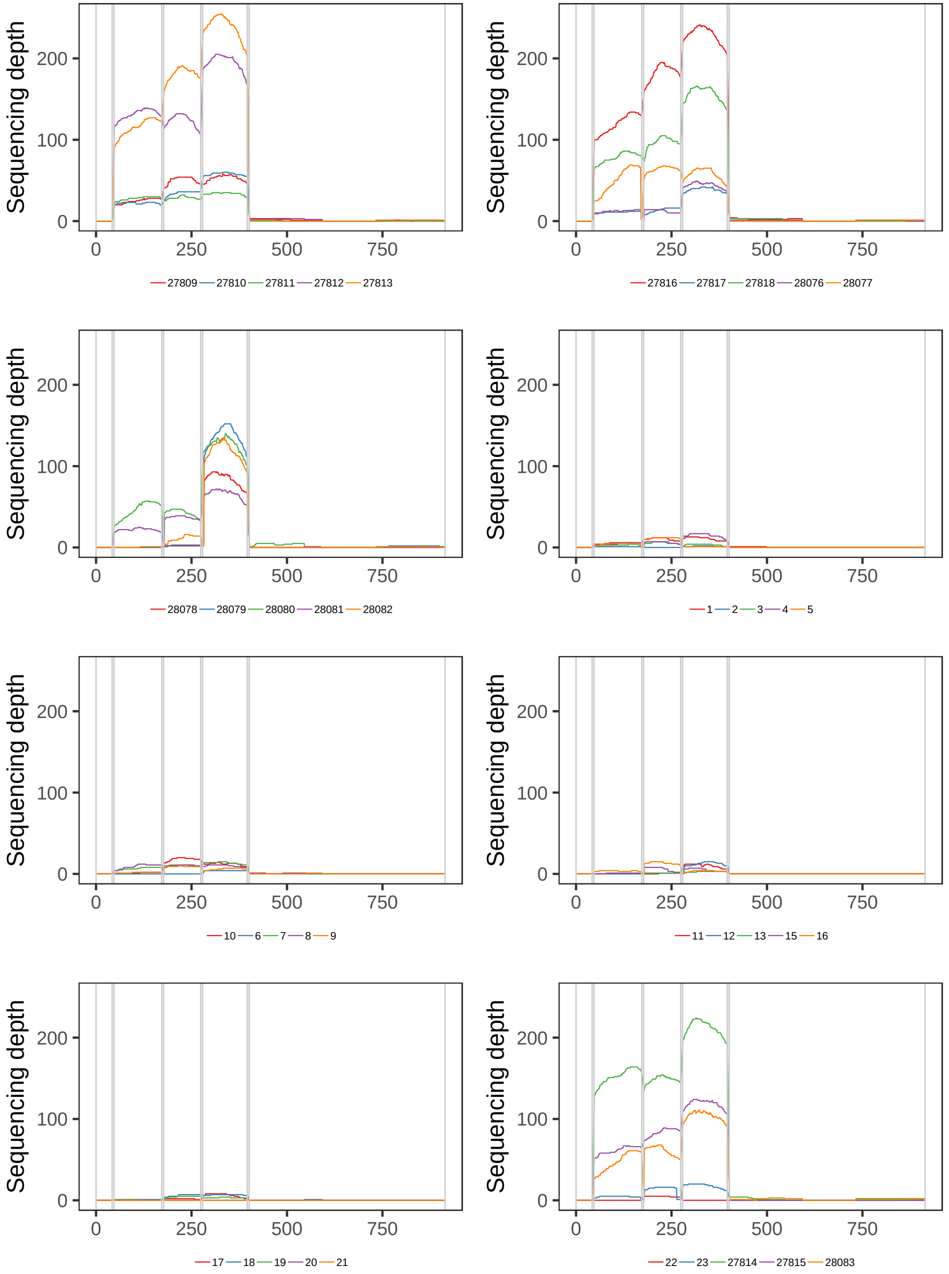

Sequencing depth
0
100
200
0
250
500
750
27809
27810
27811
27812
27813
27816
27817
27818
28076
28077
28078
28079
28080
28081
28082
1
2
3
4
5
10
6
7
8
9
11
12
13
15
16
17
18
19
20
21
22
23
27814
27815
28083

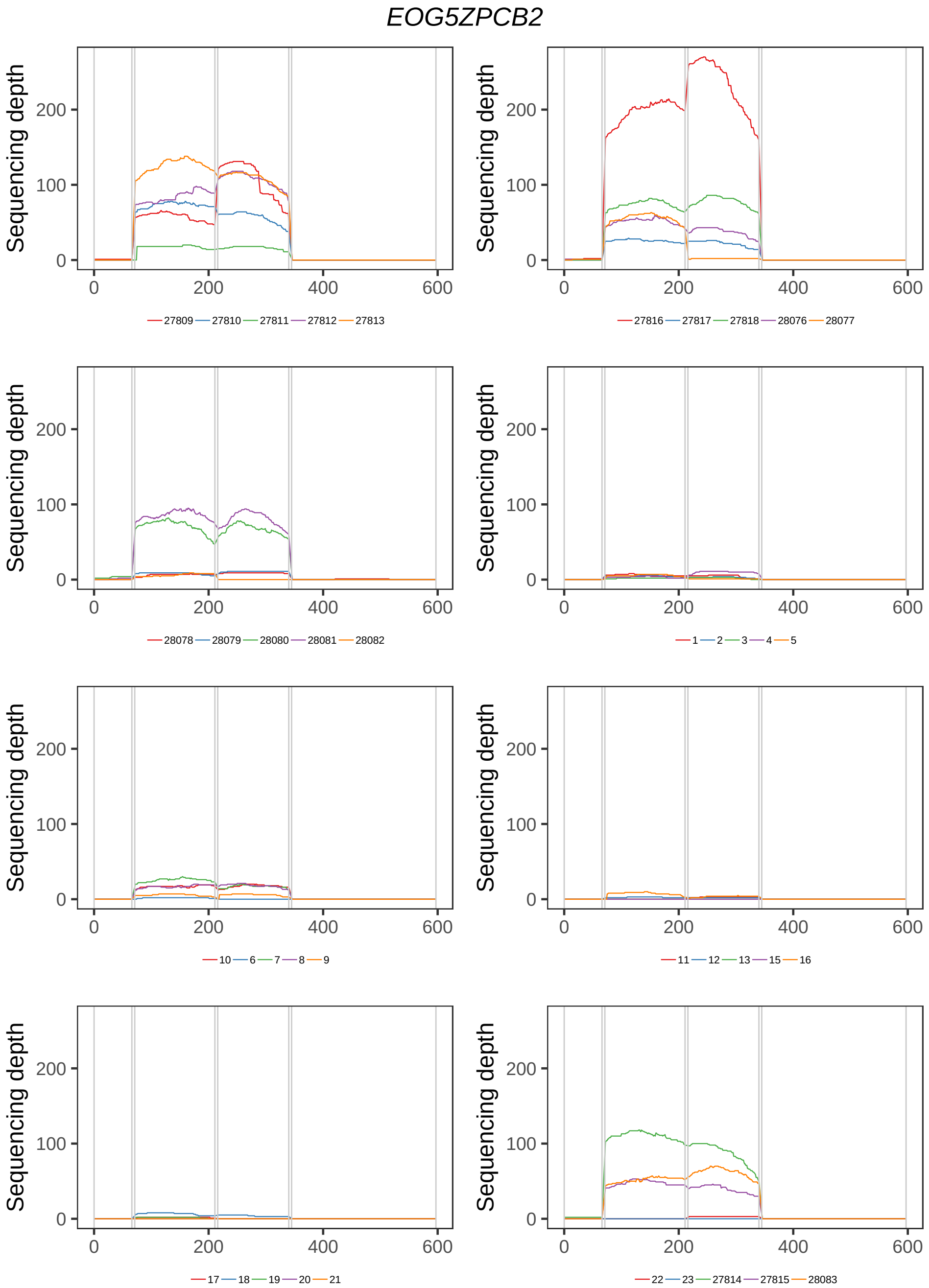
EOG5ZPCB2
Sequencing depth
0
100
200
400
600
27809
27810
27811
27812
27813
27816
27817
27818
28076
28077
28078
28079
28080
28081
28082
1
2
3
4
5
10
6
7
8
9
11
12
13
15
16
17
18
19
20
21
22
23
27814
27815
28083

EOG52BVRN

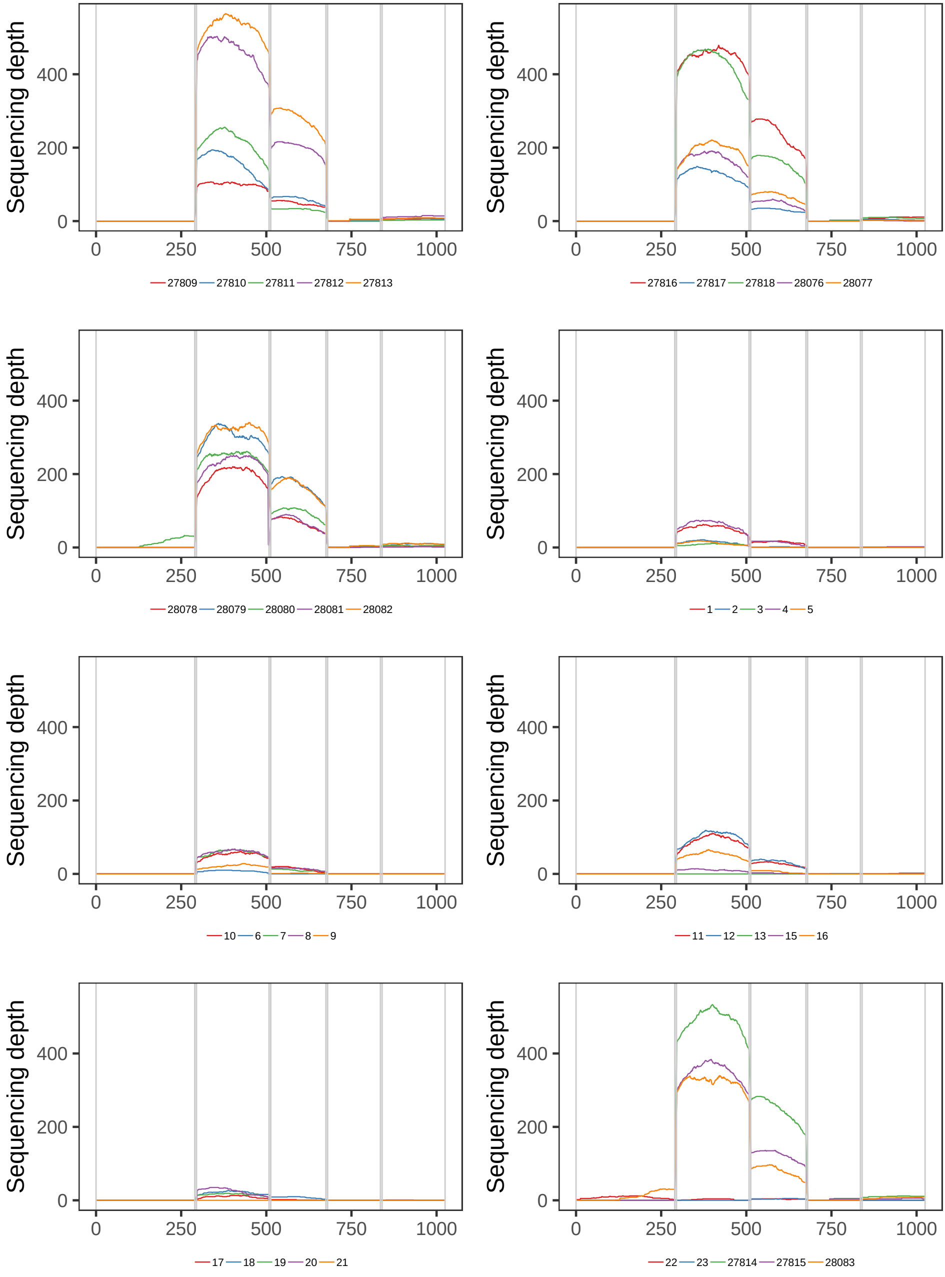
Sequencing depth
0
200
400
0
250
500
750
1000
27809 27810 27811 27812 27813
27816 27817 27818 28076 28077
28078 28079 28080 28081 28082
1 2 3 4 5
10 6 7 8 9
11 12 13 15 16
17 18 19 20 21
22 23 27814 27815 28083

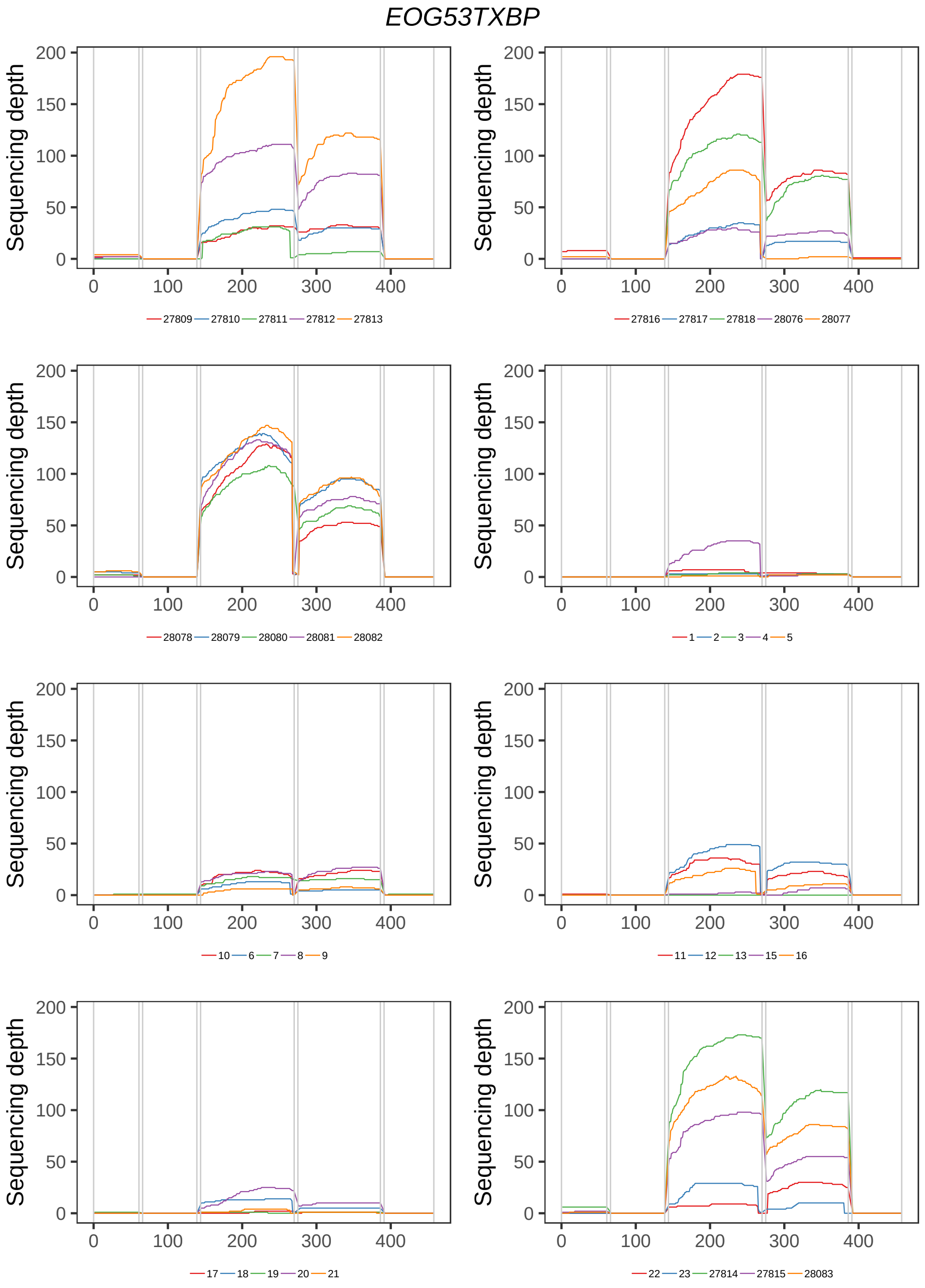
EOG53TXBP
Sequencing depth
27809 27810 27811 27812 27813
27816 27817 27818 28076 28077
28078 28079 28080 28081 28082
1 2 3 4 5
10 6 7 8 9
11 12 13 15 16
17 18 19 20 21
22 23 27814 27815 28083

EOG55QFW6

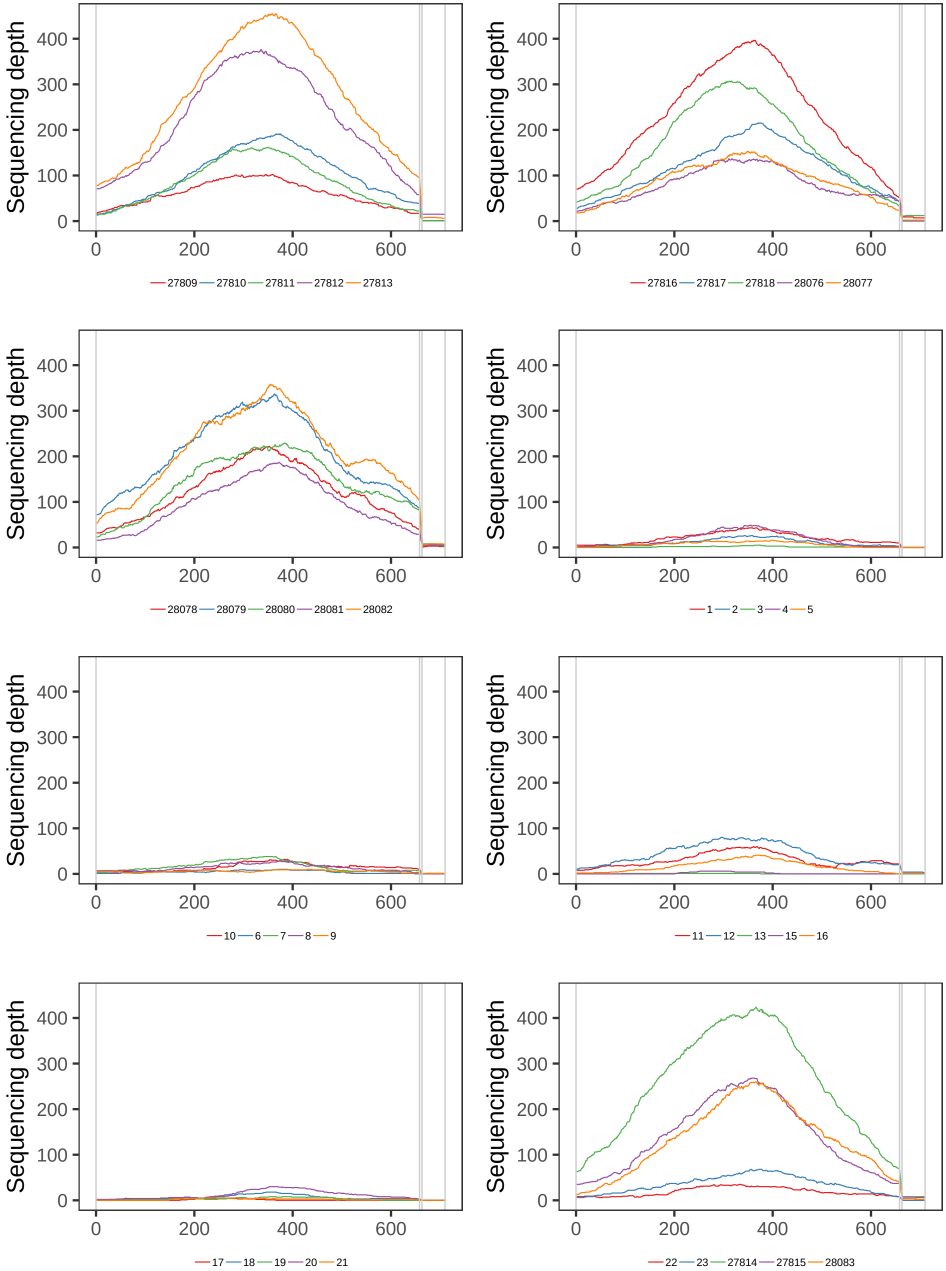

Sequencing depth
400
300
200
100
0
0
200
400
600
27809
27810
27811
27812
27813
27816
27817
27818
28076
28077
28078
28079
28080
28081
28082
1
2
3
4
5
10
6
7
8
9
11
12
13
15
16
17
18
19
20
21
22
23
27814
27815
28083

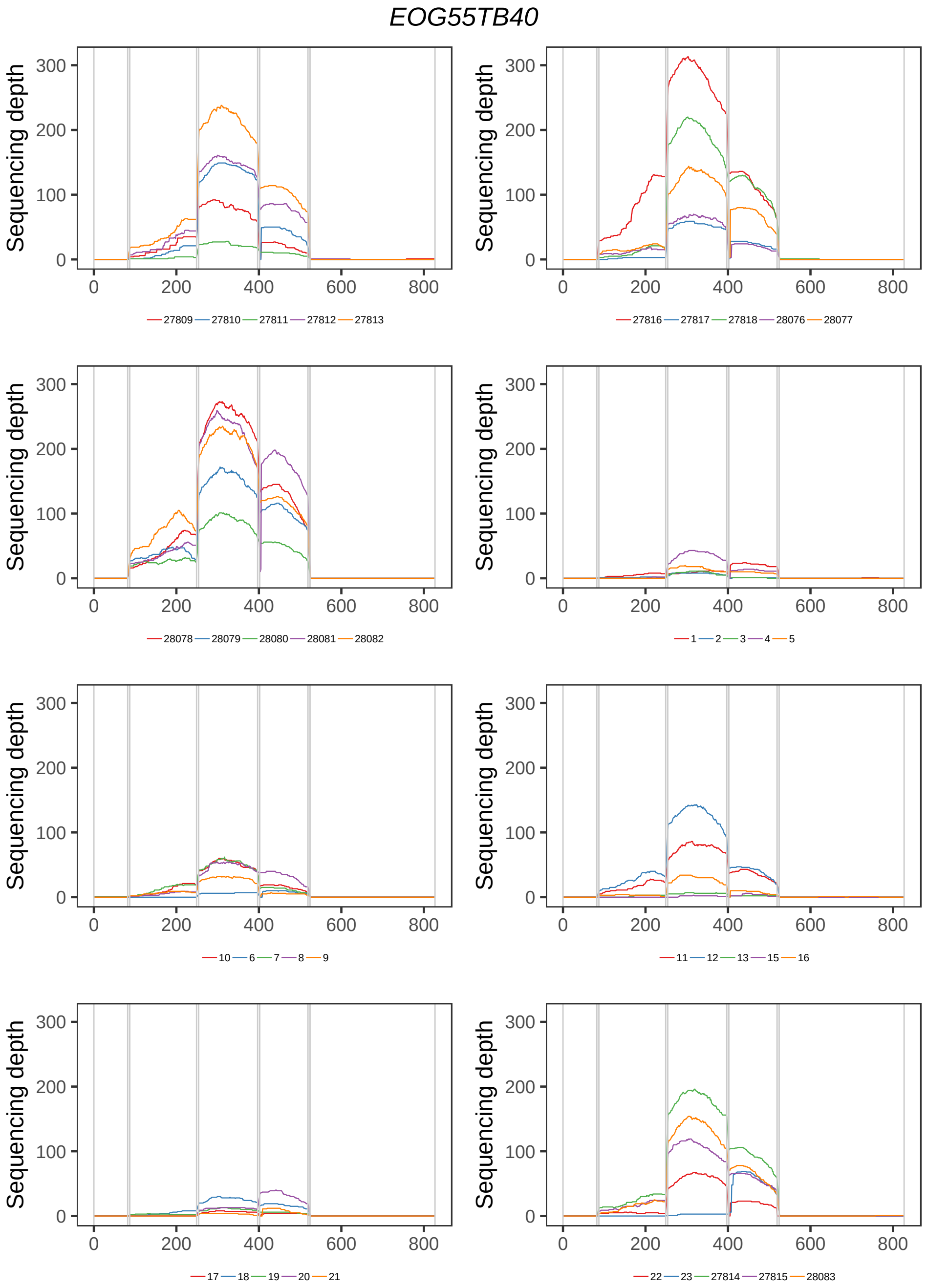
EOG55TB40
Sequencing depth
0
100
200
300
0
200
400
600
800
27809 27810 27811 27812 27813
27816 27817 27818 28076 28077
28078 28079 28080 28081 28082
1 2 3 4 5
10 6 7 8 9
11 12 13 15 16
17 18 19 20 21
22 23 27814 27815 28083

EOG58GTJK

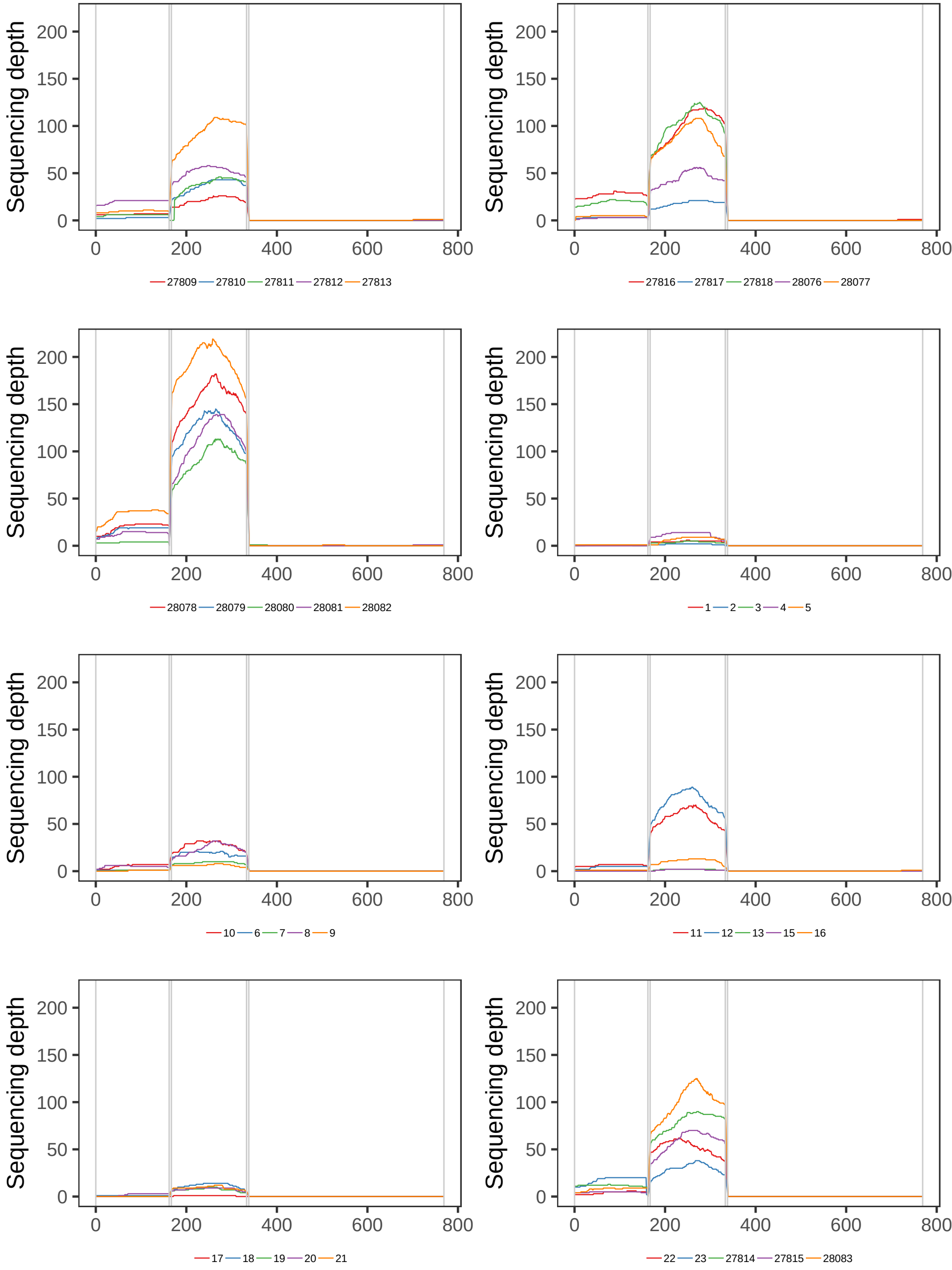
Sequencing depth
0
50
100
150
200
0
200
400
600
800
27809 27810 27811 27812 27813
27816 27817 27818 28076 28077
28078 28079 28080 28081 28082
1 2 3 4 5
10 6 7 8 9
11 12 13 15 16
17 18 19 20 21
22 23 27814 27815 28083

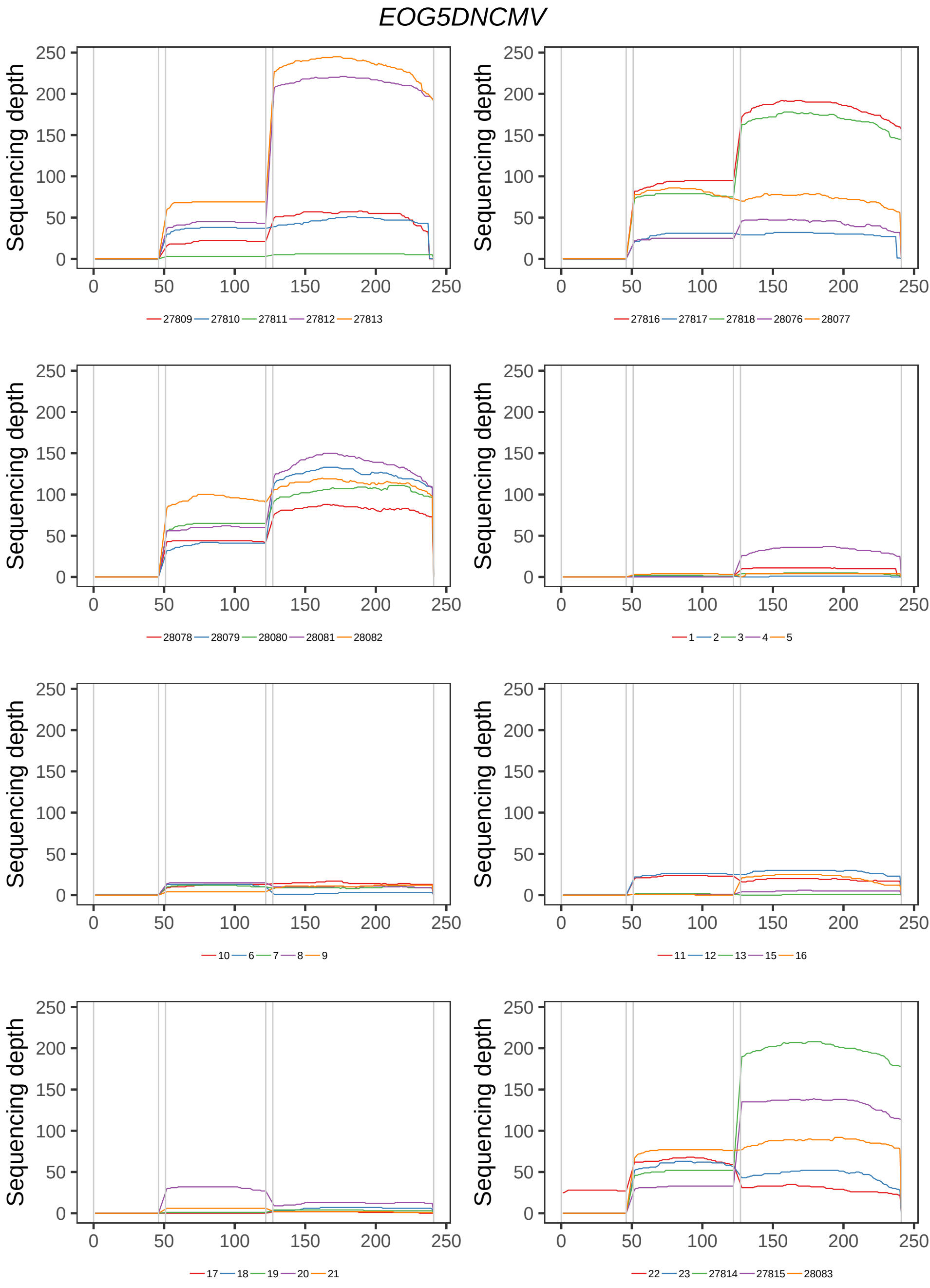
EOG5DNCMV
Sequencing depth
27809 27810 27811 27812 27813
27816 27817 27818 28076 28077
28078 28079 28080 28081 28082
1 2 3 4 5
10 6 7 8 9
11 12 13 15 16
17 18 19 20 21
22 23 27814 27815 28083

EOG5FBG99

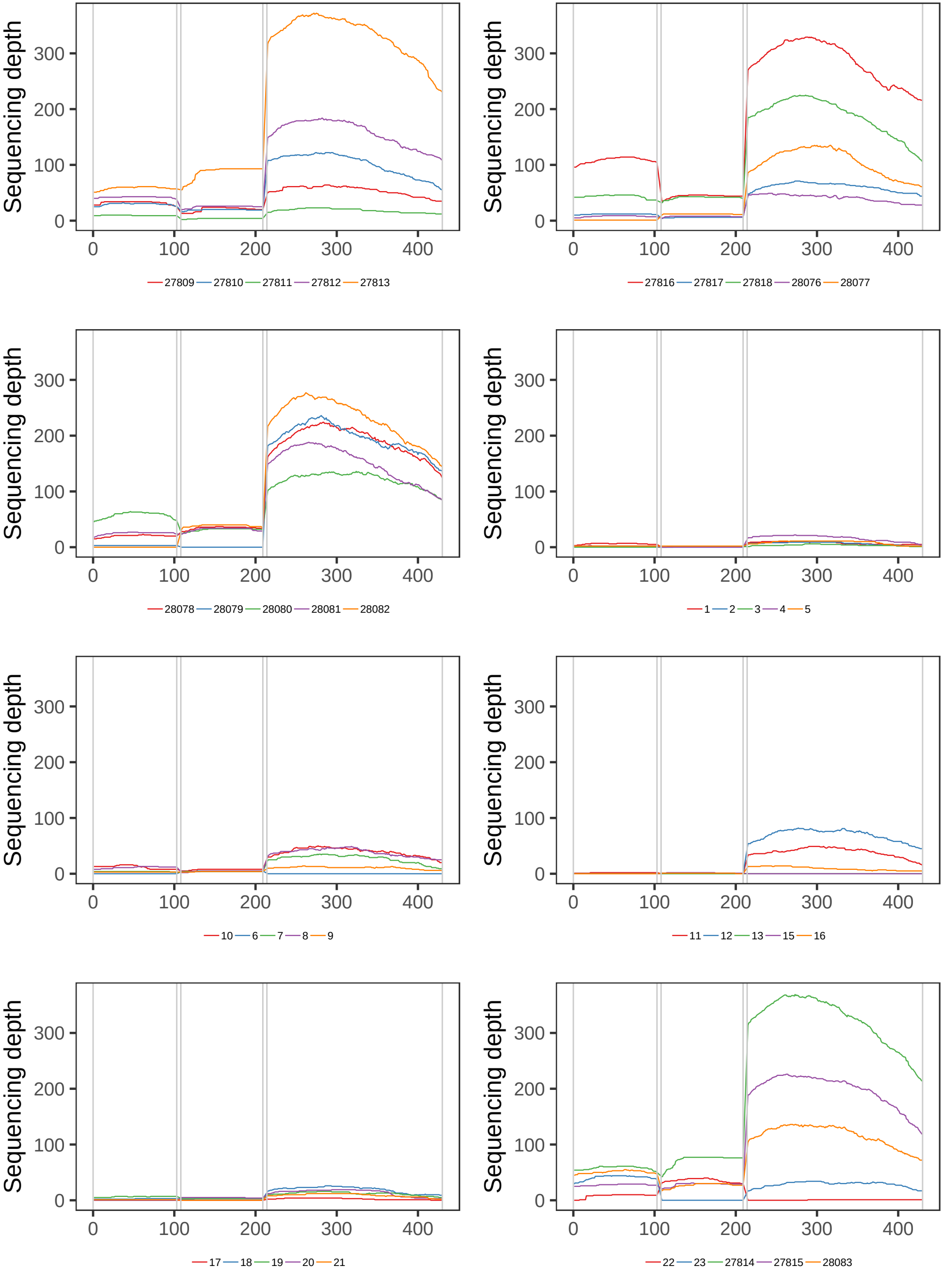
Sequencing depth
0
100
200
300
400
27809 27810 27811 27812 27813
27816 27817 27818 28076 28077
28078 28079 28080 28081 28082
1 2 3 4 5
10 6 7 8 9
11 12 13 15 16
17 18 19 20 21
22 23 27814 27815 28083

EOG5GXD3D

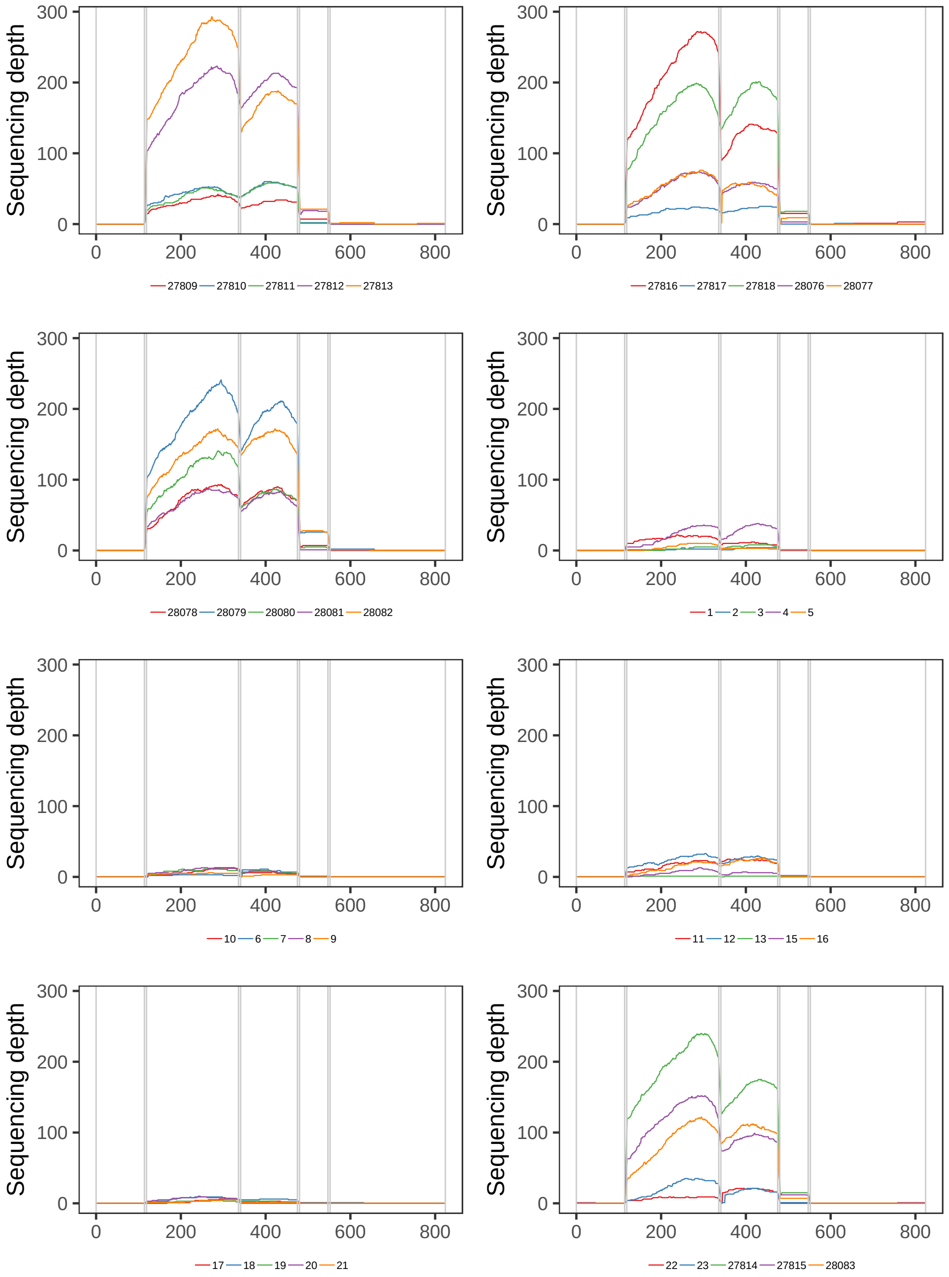
Sequencing depth
0
100
200
300
0
200
400
600
800
27809 27810 27811 27812 27813
27816 27817 27818 28076 28077
28078 28079 28080 28081 28082
1 2 3 4 5
10 6 7 8 9
11 12 13 15 16
17 18 19 20 21
22 23 27814 27815 28083

EOG5J6Q79

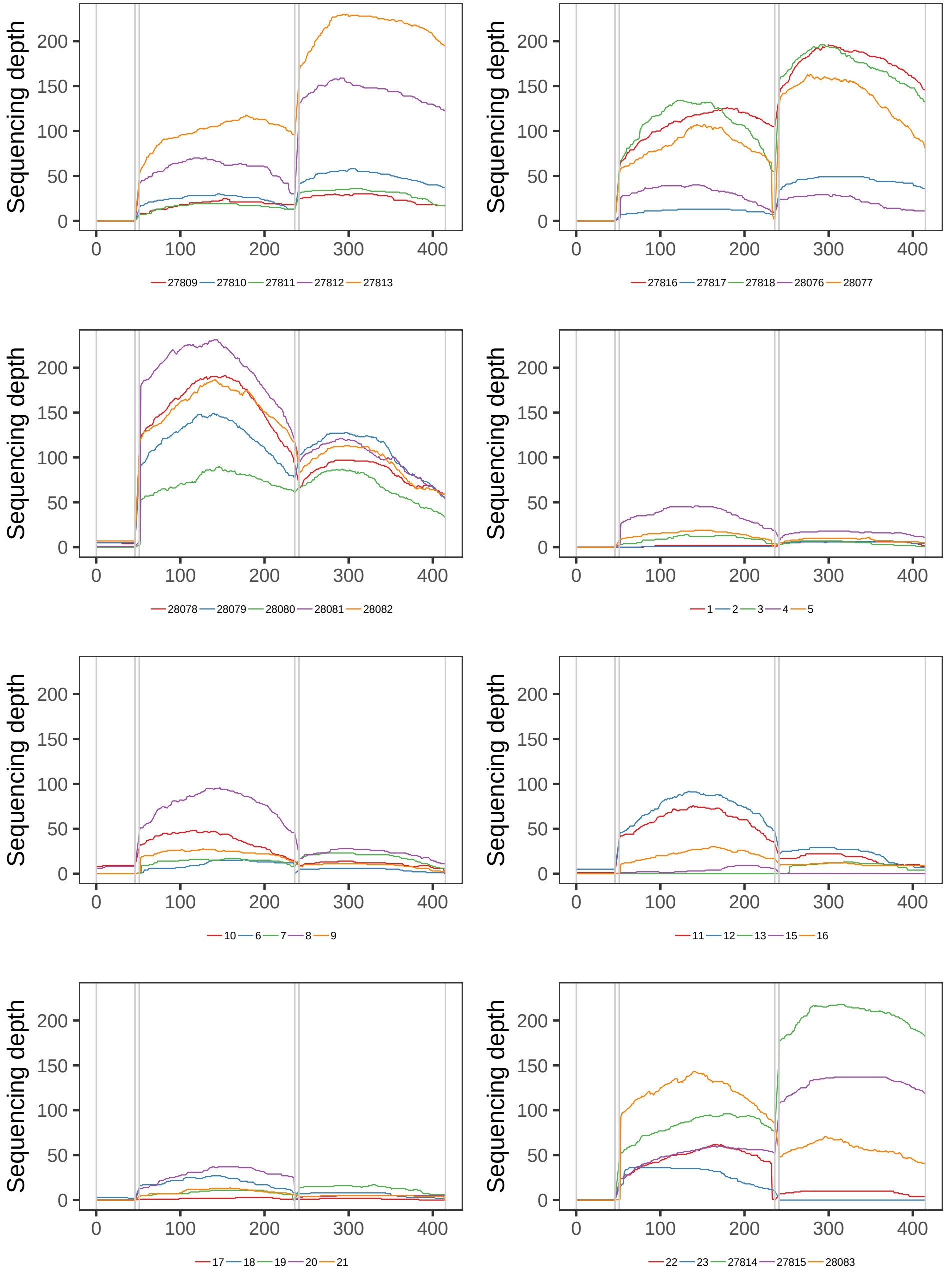
Sequencing depth
0
50
100
150
200
0
100
200
300
400
27809 27810 27811 27812 27813
27816 27817 27818 28076 28077
28078 28079 28080 28081 28082
1 2 3 4 5
10 6 7 8 9
11 12 13 15 16
17 18 19 20 21
22 23 27814 27815 28083

EOG5N2Z4Q

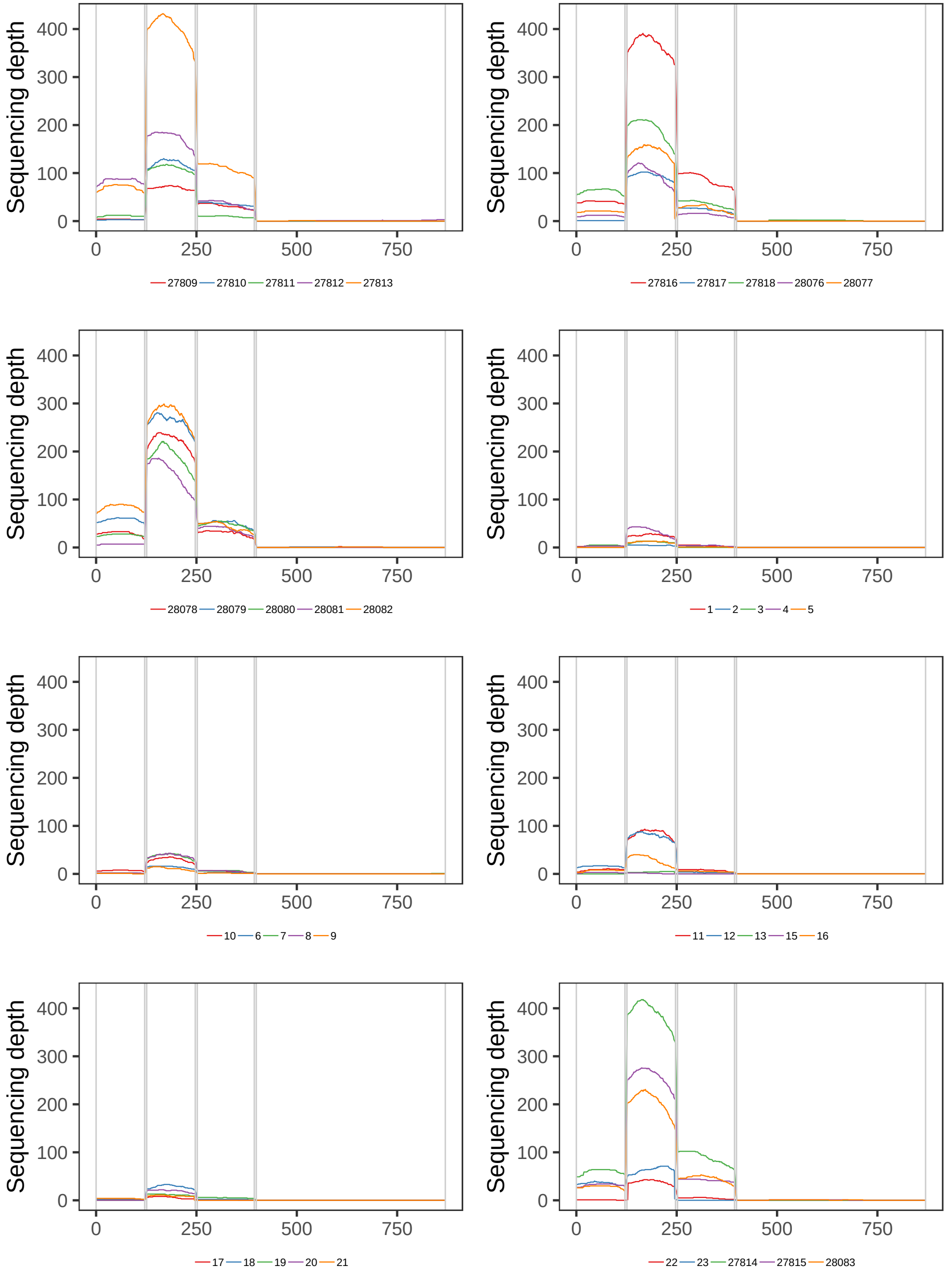

Sequencing depth
0
100
200
300
400
0
250
500
750
27809 27810 27811 27812 27813
27816 27817 27818 28076 28077
28078 28079 28080 28081 28082
1 2 3 4 5
10 6 7 8 9
11 12 13 15 16
17 18 19 20 21
22 23 27814 27815 28083

EOG5PC88F

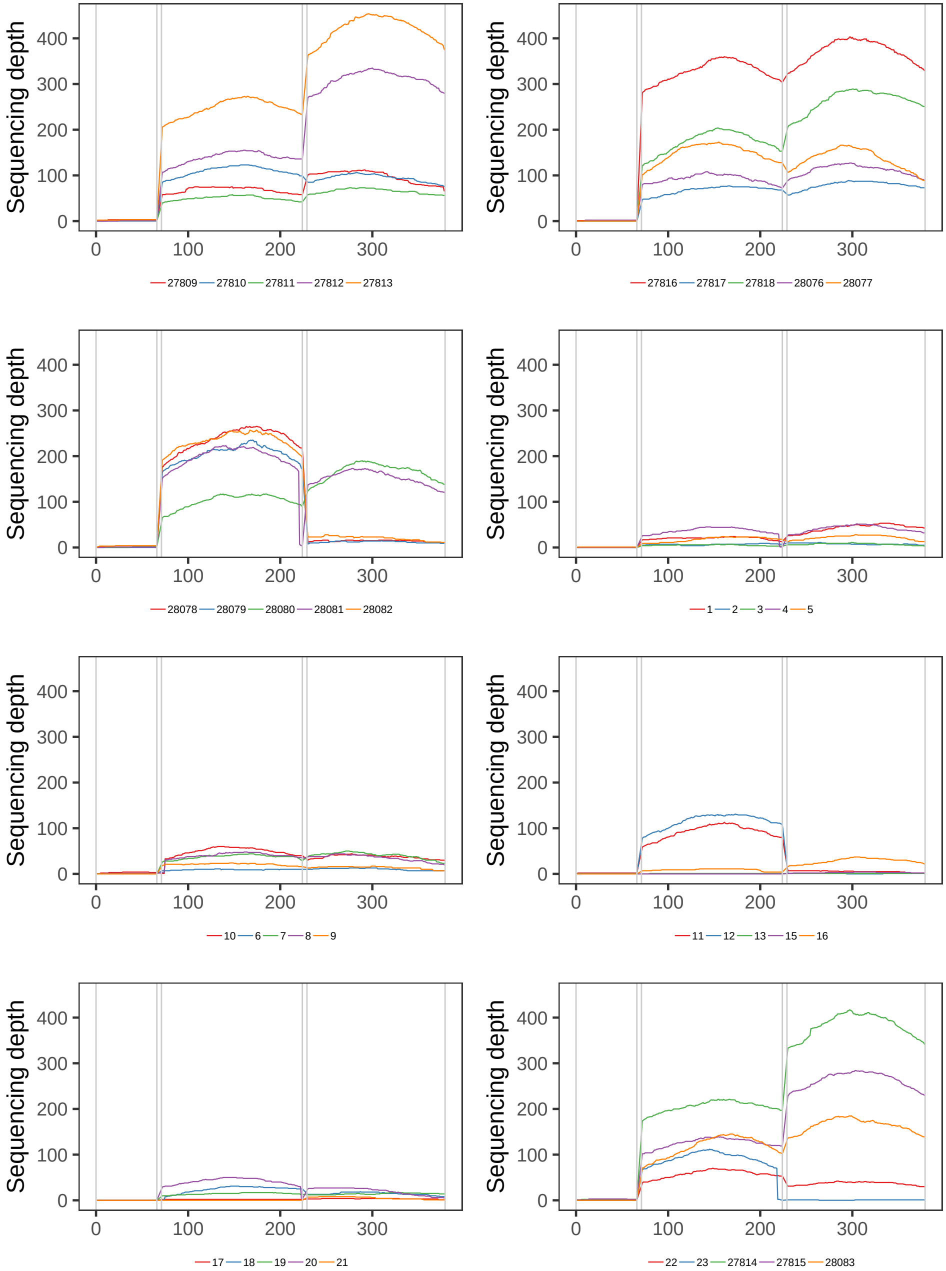
Sequencing depth
0
100
200
300
400
0
100
200
300
27809 27810 27811 27812 27813
27816 27817 27818 28076 28077
28078 28079 28080 28081 28082
1 2 3 4 5
10 6 7 8 9
11 12 13 15 16
17 18 19 20 21
22 23 27814 27815 28083

EOG5R4XJF

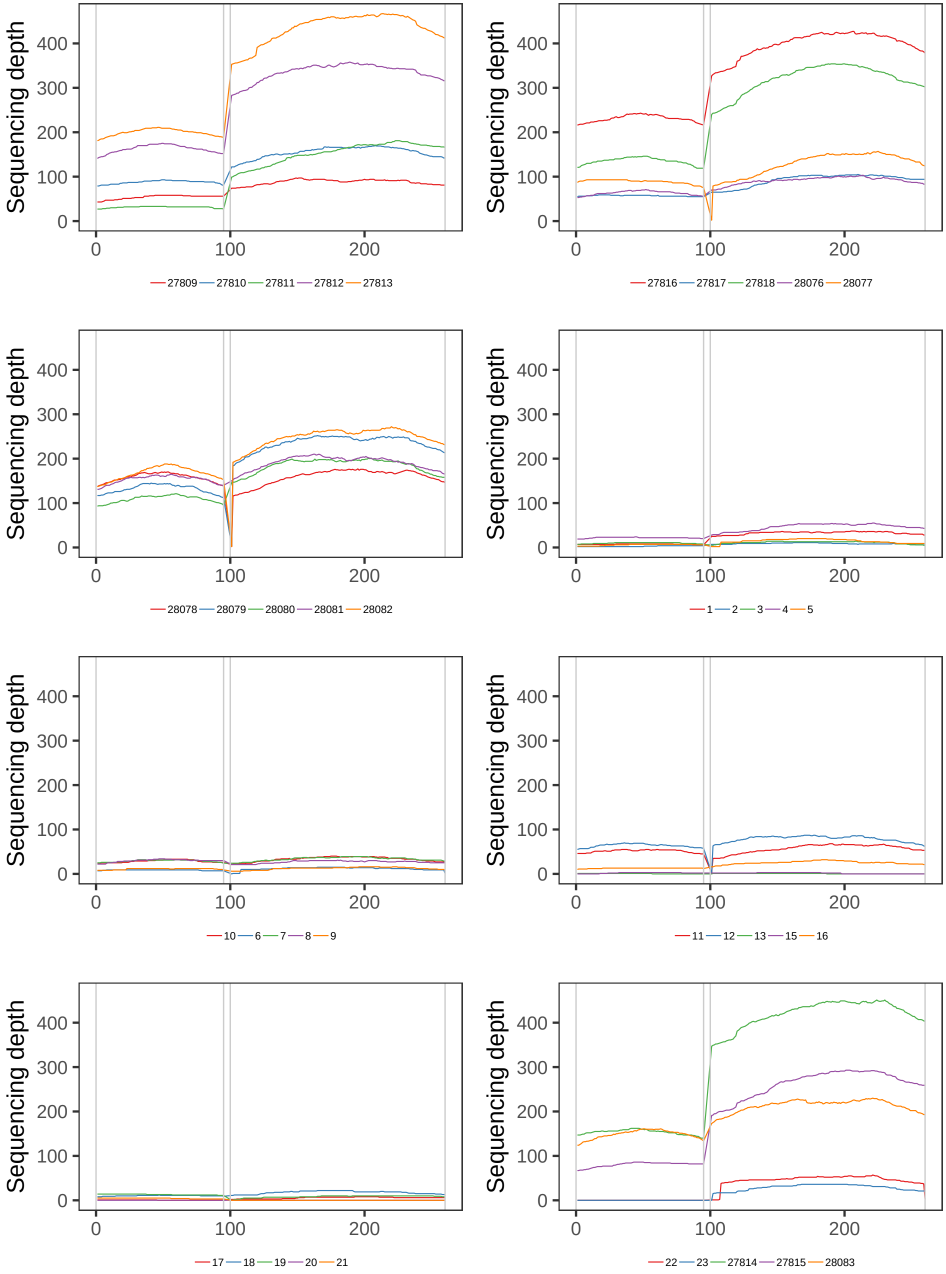

Sequencing depth
0
100
200
300
400
0
100
200
27809 27810 27811 27812 27813
27816 27817 27818 28076 28077
28078 28079 28080 28081 28082
1 2 3 4 5
10 6 7 8 9
11 12 13 15 16
17 18 19 20 21
22 23 27814 27815 28083

EOG5R7ST1

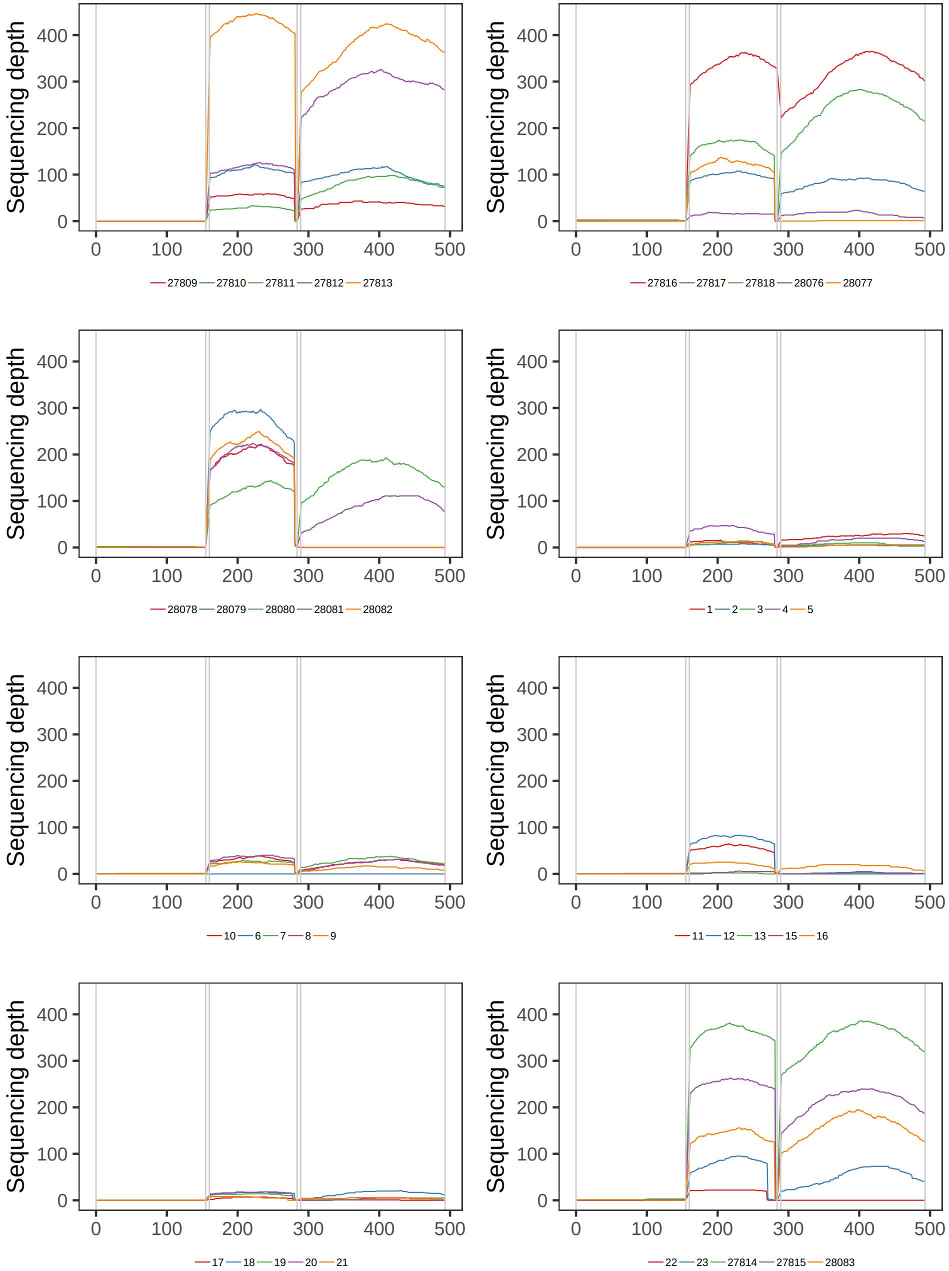
Sequencing depth
0
100
200
300
400
500
27809 27810 27811 27812 27813
27816 27817 27818 28076 28077
28078 28079 28080 28081 28082
1 2 3 4 5
10 6 7 8 9
11 12 13 15 16
17 18 19 20 21
22 23 27814 27815 28083

EOG5W0VV6

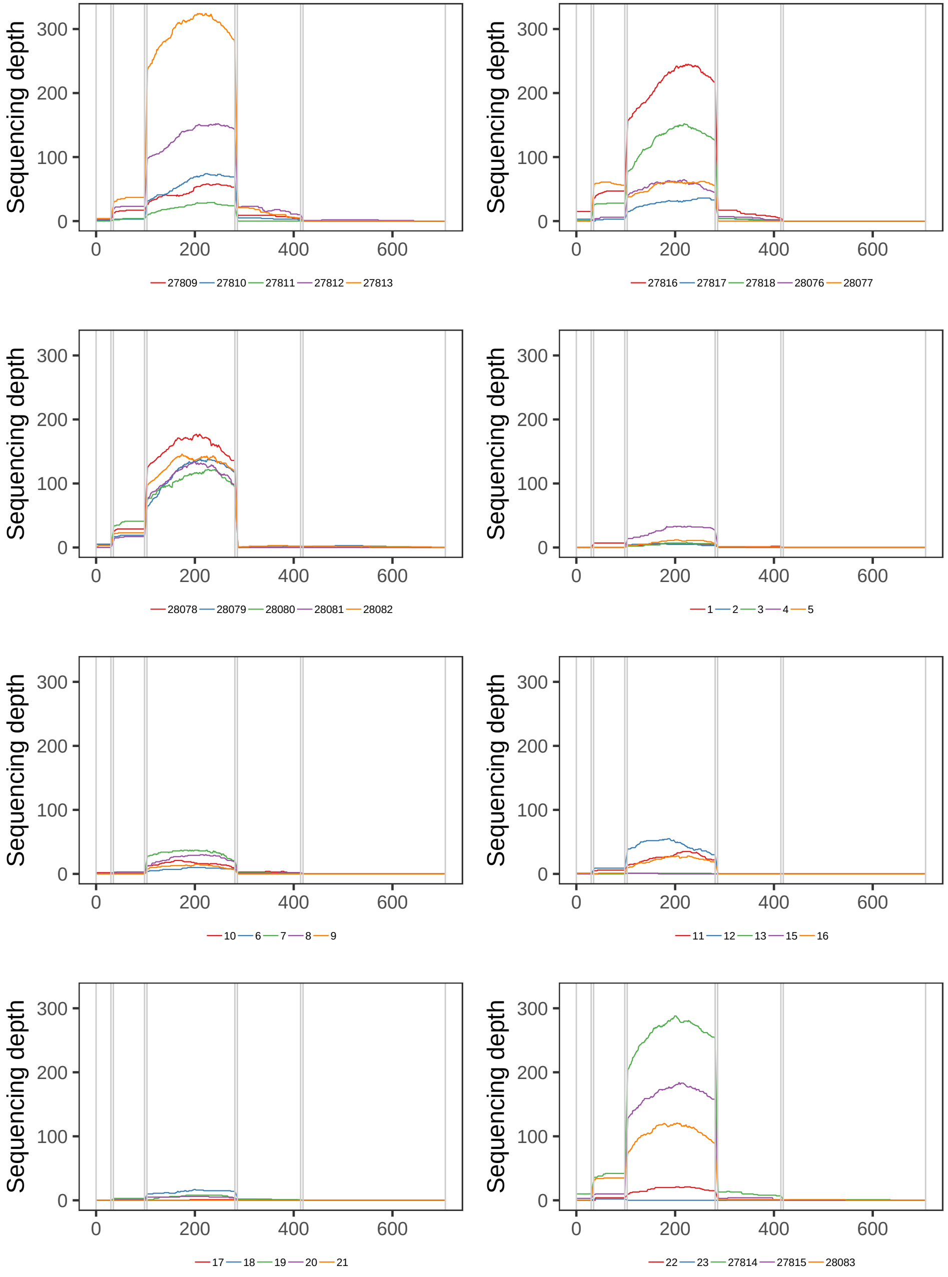
Sequencing depth
0
100
200
300
0
200
400
600
27809 27810 27811 27812 27813
27816 27817 27818 28076 28077
28078 28079 28080 28081 28082
1 2 3 4 5
10 6 7 8 9
11 12 13 15 16
17 18 19 20 21
22 23 27814 27815 28083

EOG5XSJ5X

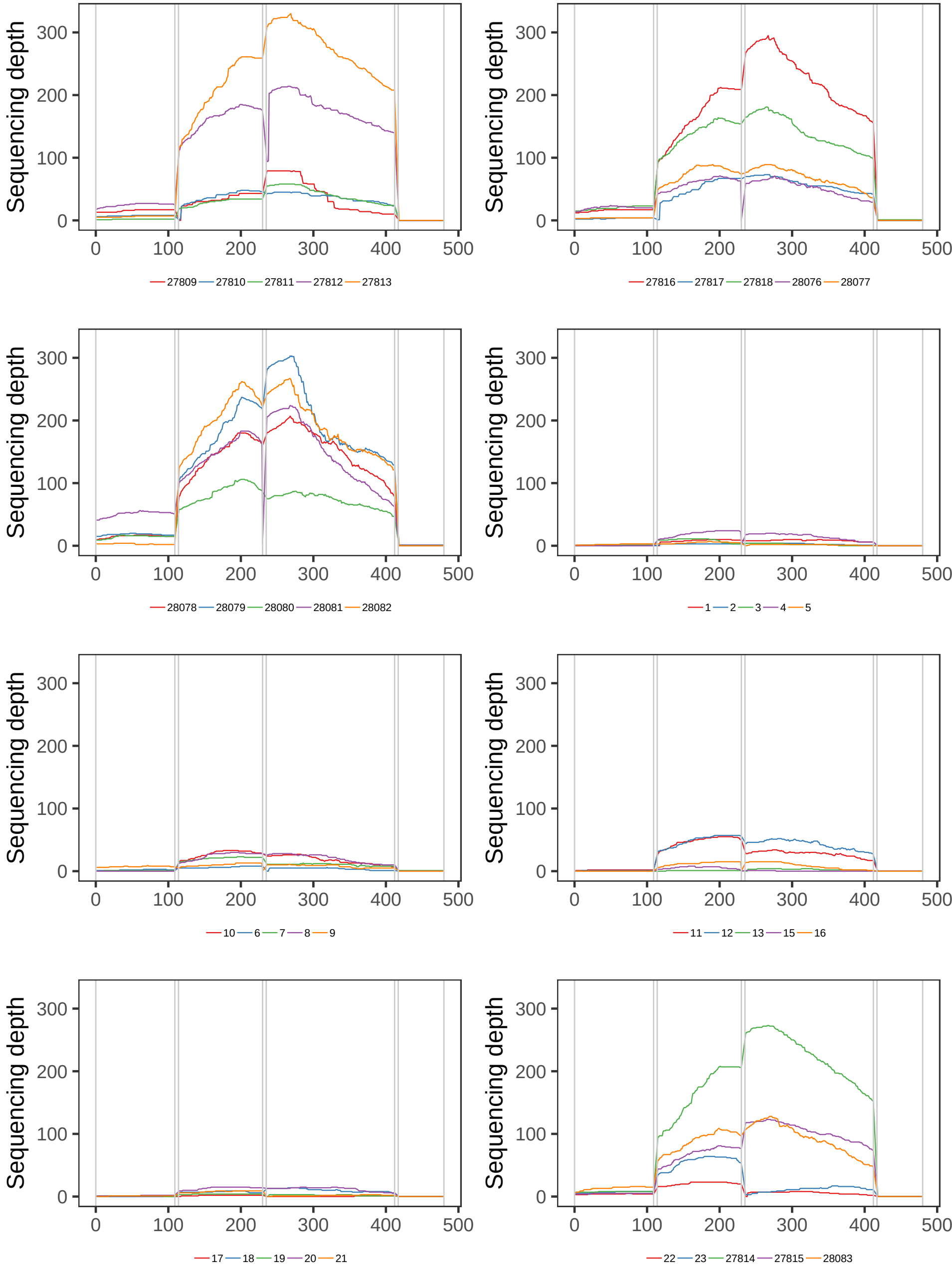

Sequencing depth
0
100
200
300
400
500
27809 27810 27811 27812 27813
27816 27817 27818 28076 28077
28078 28079 28080 28081 28082
1 2 3 4 5
10 6 7 8 9
11 12 13 15 16
17 18 19 20 21
22 23 27814 27815 28083

EOG50P2NZ

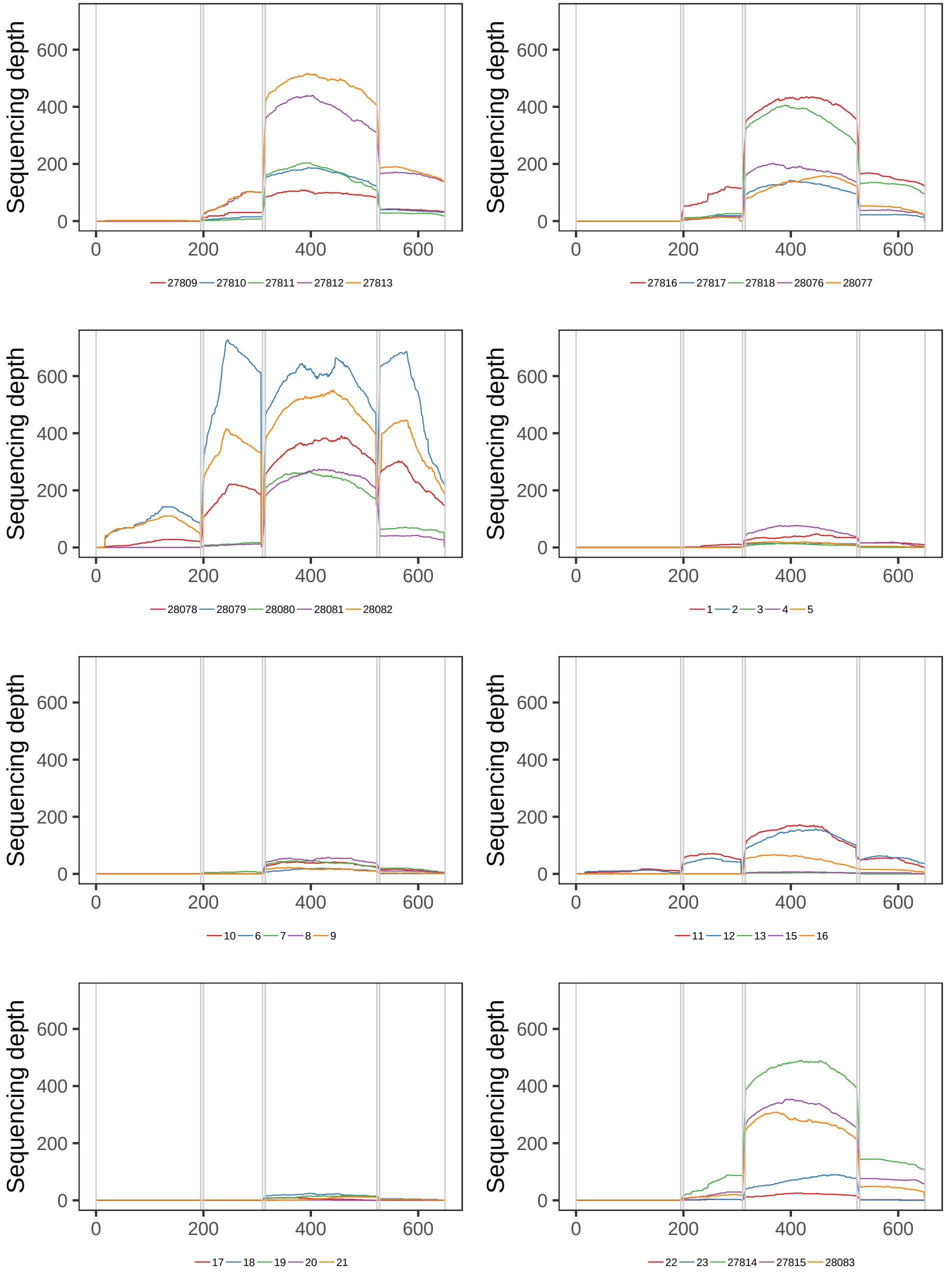

Sequencing depth
0
200
400
600
27809
27810
27811
27812
27813
27816
27817
27818
28076
28077
28078
28079
28080
28081
28082
1
2
3
4
5
10
6
7
8
9
11
12
13
15
16
17
18
19
20
21
22
23
27814
27815
28083

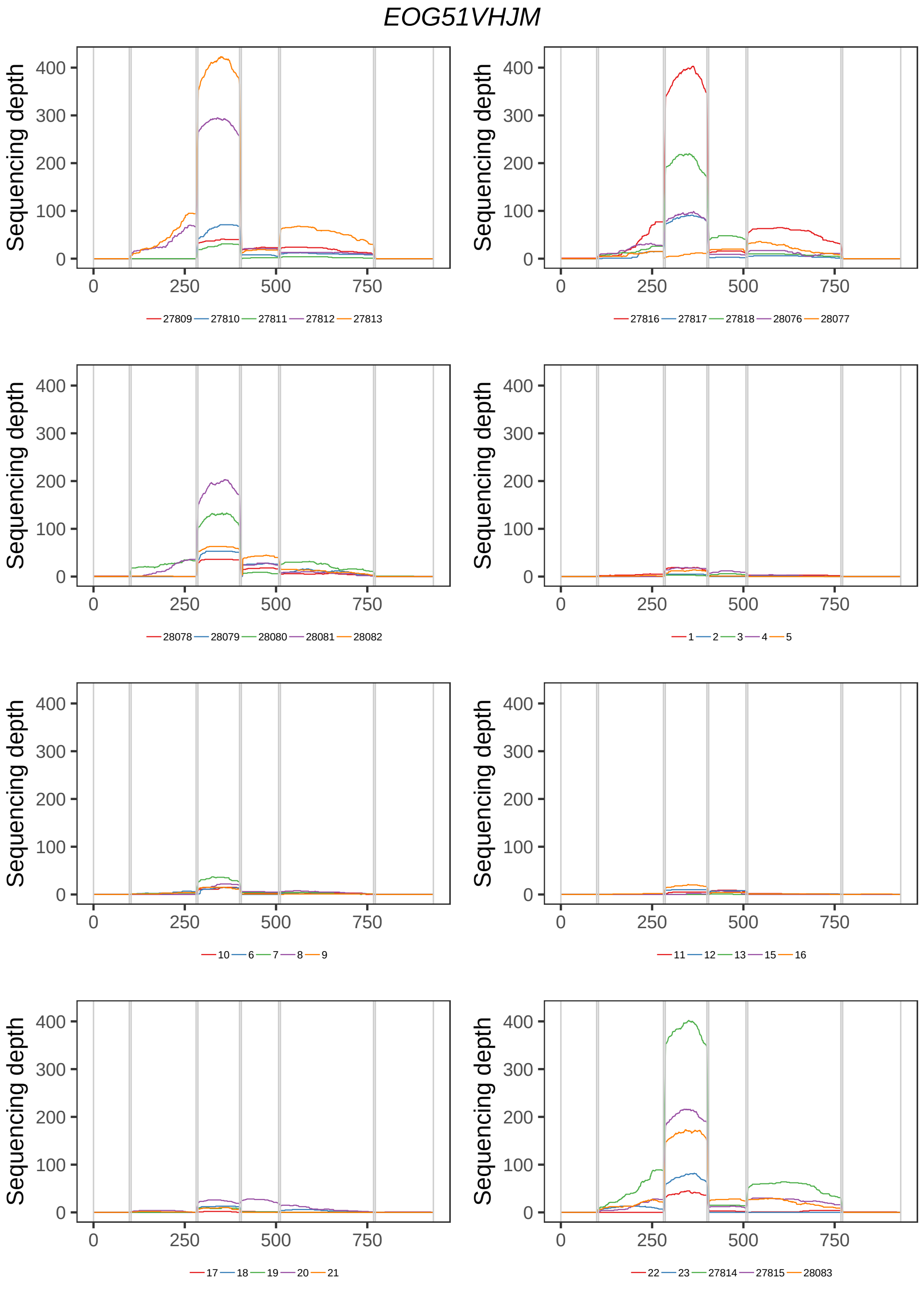
EOG51VHJM
Sequencing depth
27809 27810 27811 27812 27813
27816 27817 27818 28076 28077
28078 28079 28080 28081 28082
1 2 3 4 5
10 6 7 8 9
11 12 13 15 16
17 18 19 20 21
22 23 27814 27815 28083

EOG537PWS

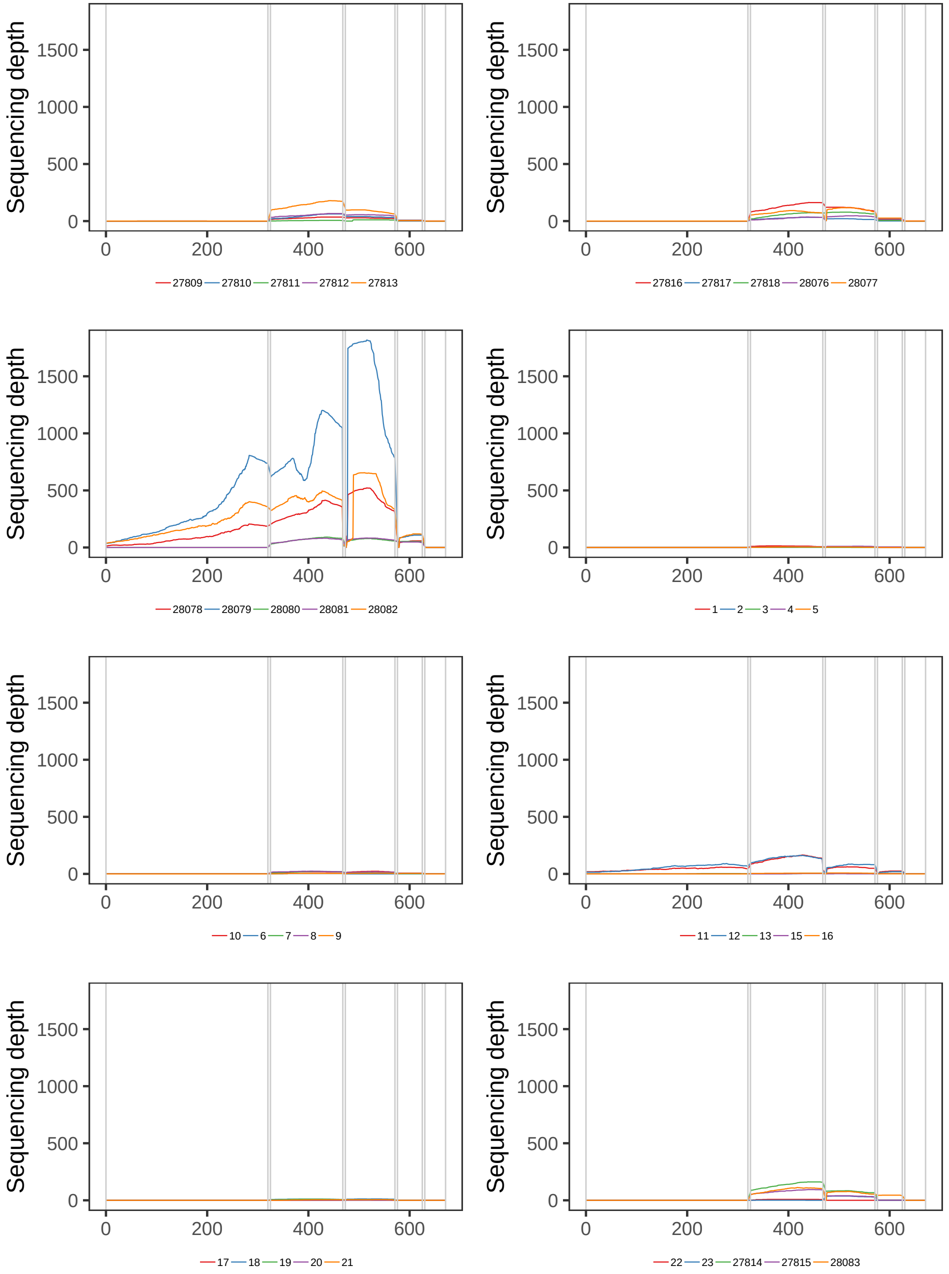

Sequencing depth
1500
1000
500
0
0
200
400
600
27809
27810
27811
27812
27813
27816
27817
27818
28076
28077
28078
28079
28080
28081
28082
1
2
3
4
5
10
6
7
8
9
11
12
13
15
16
17
18
19
20
21
22
23
27814
27815
28083

EOG53FFDB

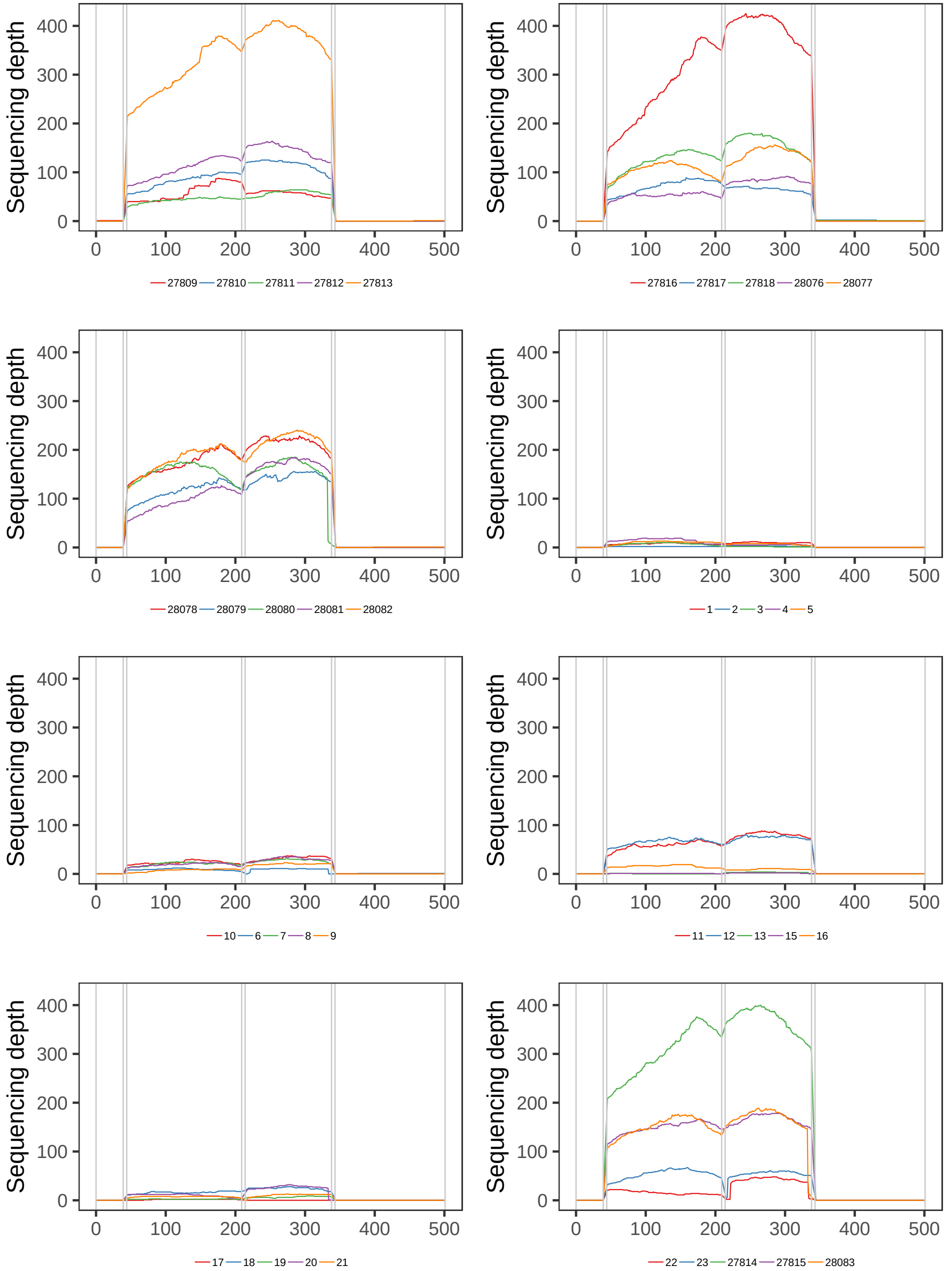
Sequencing depth
0
100
200
300
400
500
27809 27810 27811 27812 27813
27816 27817 27818 28076 28077
28078 28079 28080 28081 28082
1 2 3 4 5
10 6 7 8 9
11 12 13 15 16
17 18 19 20 21
22 23 27814 27815 28083

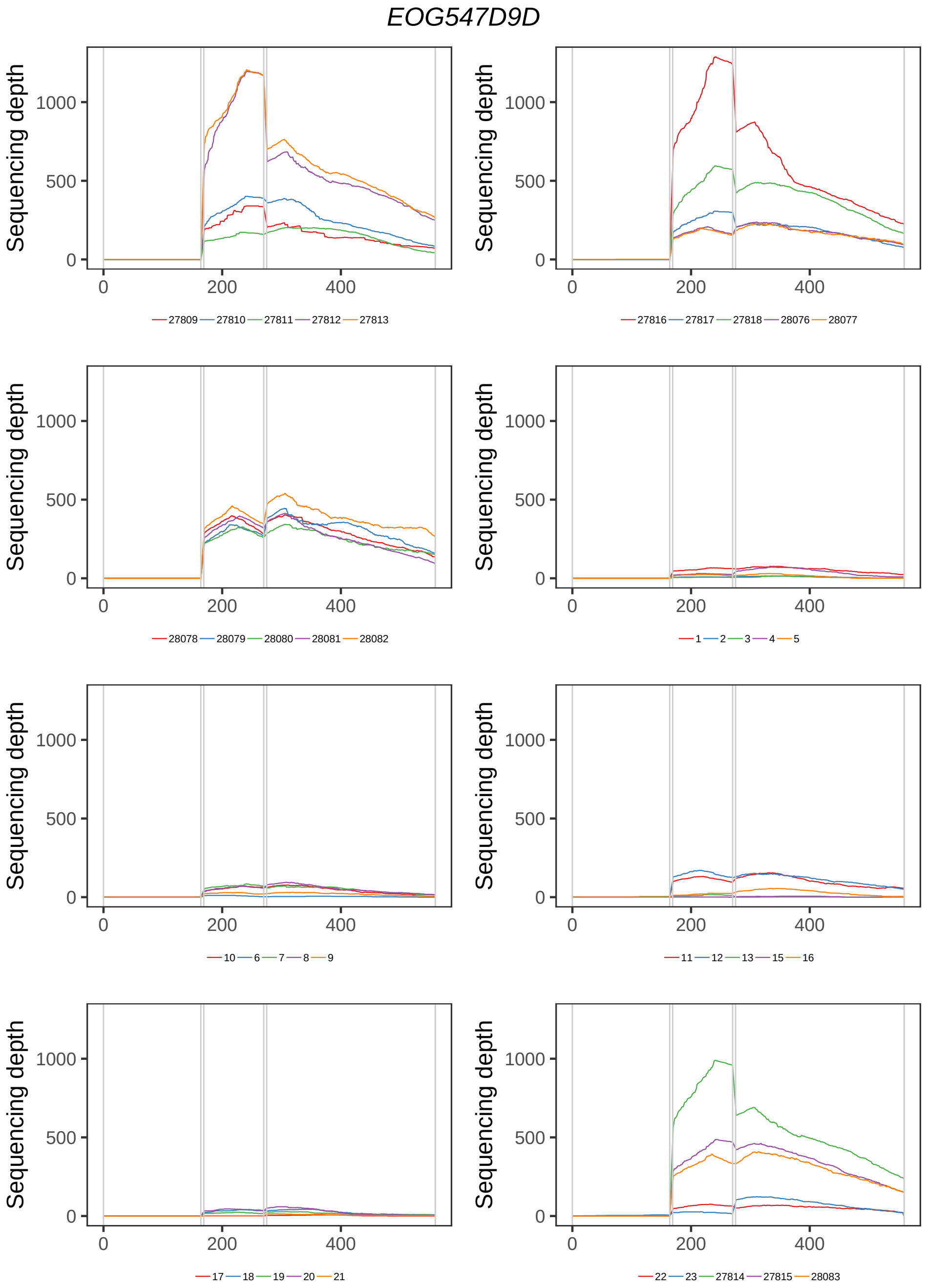
EOG547D9D
Sequencing depth
27809 27810 27811 27812 27813
27816 27817 27818 28076 28077
28078 28079 28080 28081 28082
1 2 3 4 5
10 6 7 8 9
11 12 13 15 16
17 18 19 20 21
22 23 27814 27815 28083

EOG54MW74

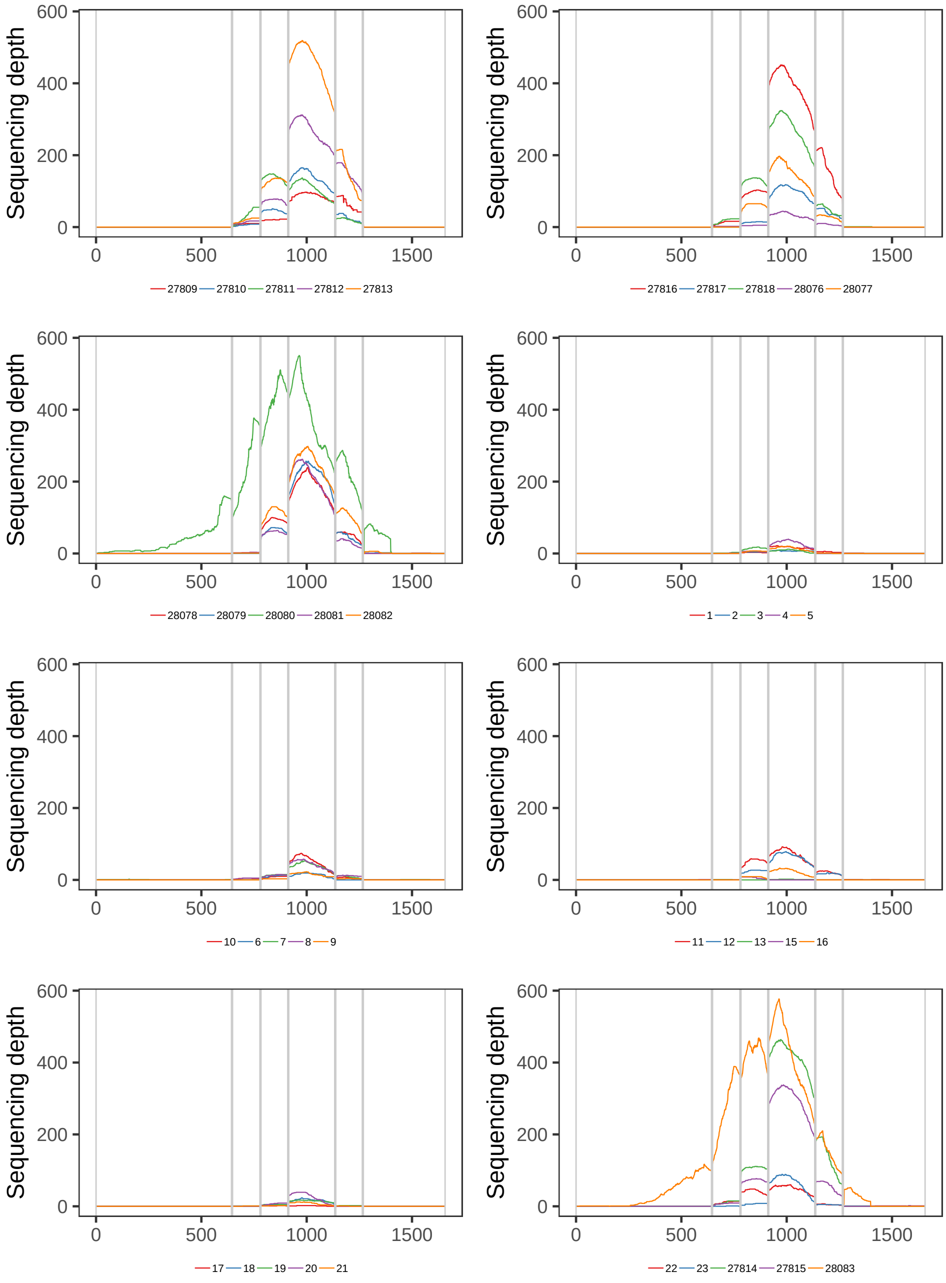
Sequencing depth
0
200
400
600
0
500
1000
1500
27809 27810 27811 27812 27813
27816 27817 27818 28076 28077
28078 28079 28080 28081 28082
1 2 3 4 5
10 6 7 8 9
11 12 13 15 16
17 18 19 20 21
22 23 27814 27815 28083

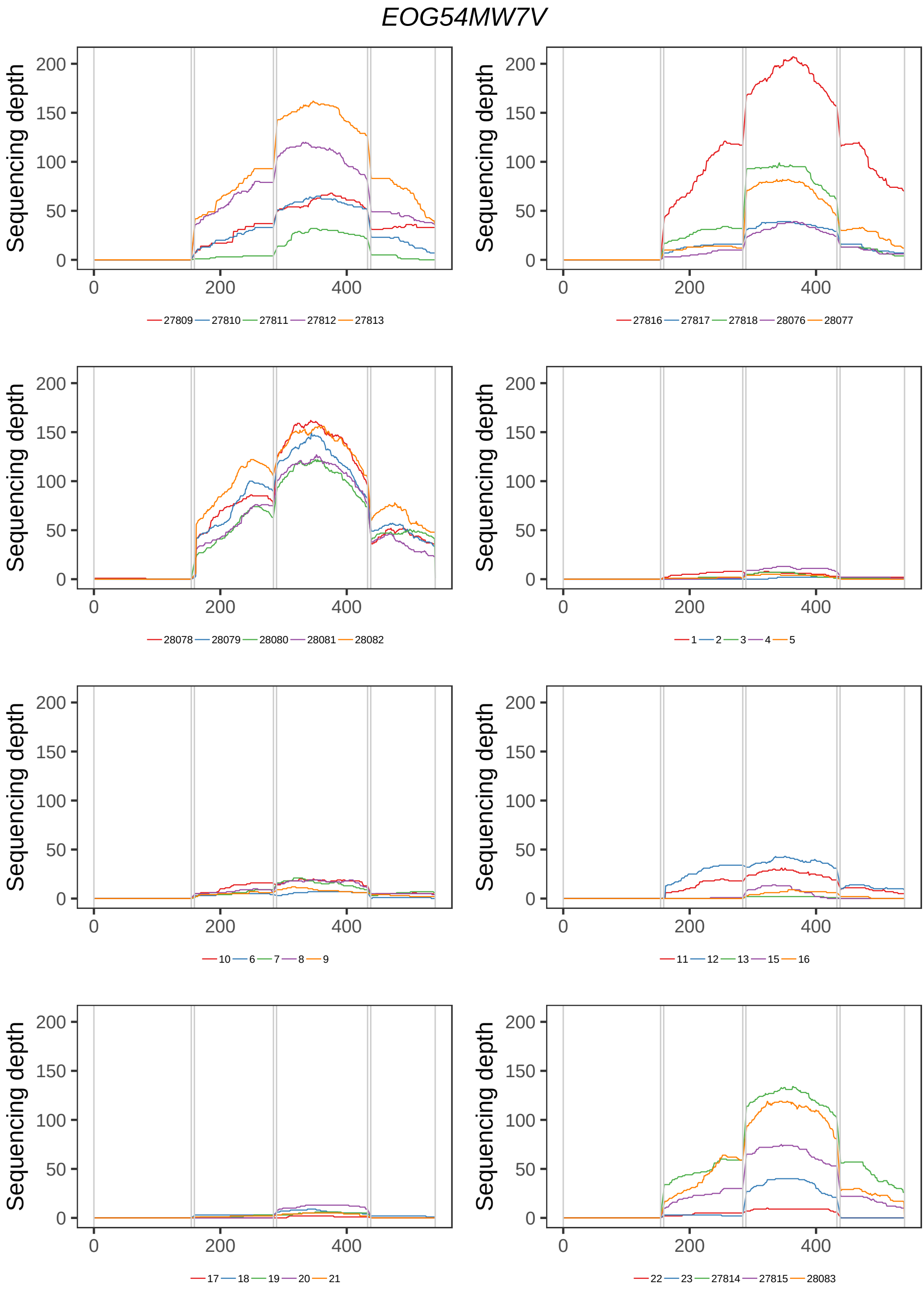

EOG54MW7V
Sequencing depth
0
50
100
150
200
0
200
400
27809
27810
27811
27812
27813
27816
27817
27818
28076
28077
28078
28079
28080
28081
28082
1
2
3
4
5
10
6
7
8
9
11
12
13
15
16
17
18
19
20
21
22
23
27814
27815
28083

EOG5G1JZS

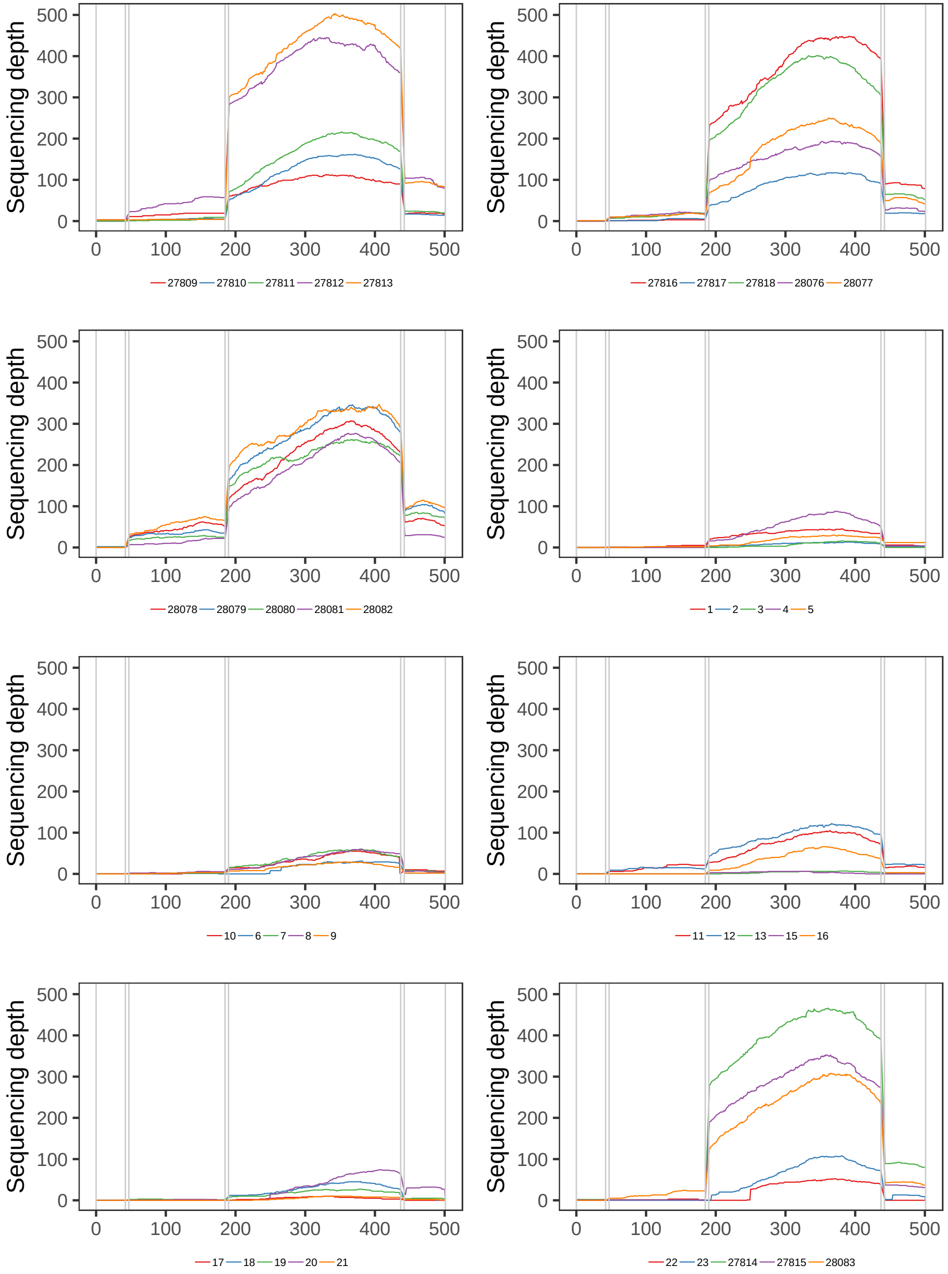

Sequencing depth
0
100
200
300
400
500
27809 27810 27811 27812 27813
27816 27817 27818 28076 28077
28078 28079 28080 28081 28082
1 2 3 4 5
10 6 7 8 9
11 12 13 15 16
17 18 19 20 21
22 23 27814 27815 28083

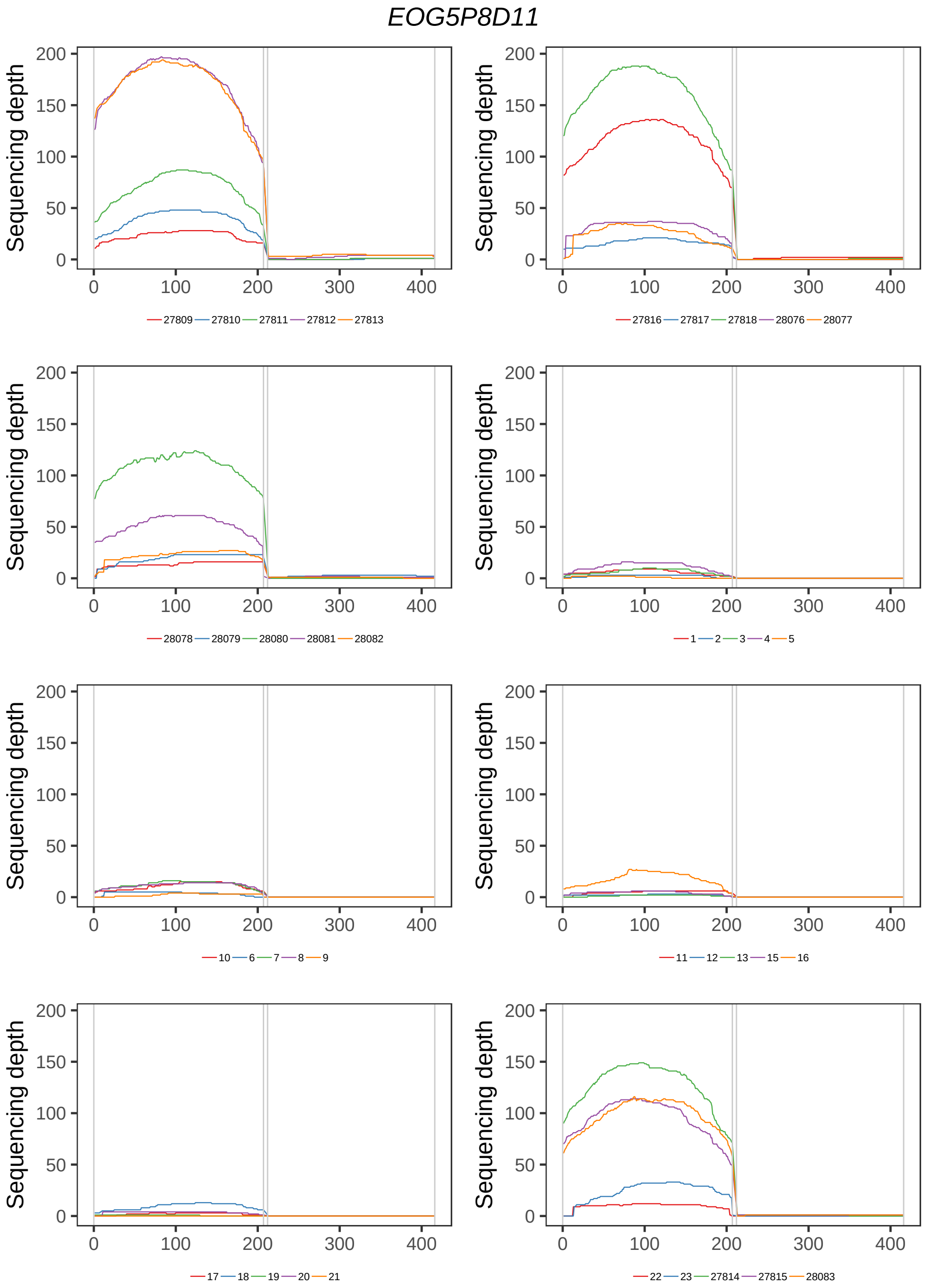

EOG5P8D11
Sequencing depth
0
50
100
150
200
0
100
200
300
400
27809 27810 27811 27812 27813
27816 27817 27818 28076 28077
28078 28079 28080 28081 28082
1 2 3 4 5
10 6 7 8 9
11 12 13 15 16
17 18 19 20 21
22 23 27814 27815 28083

EOG5RN8RS

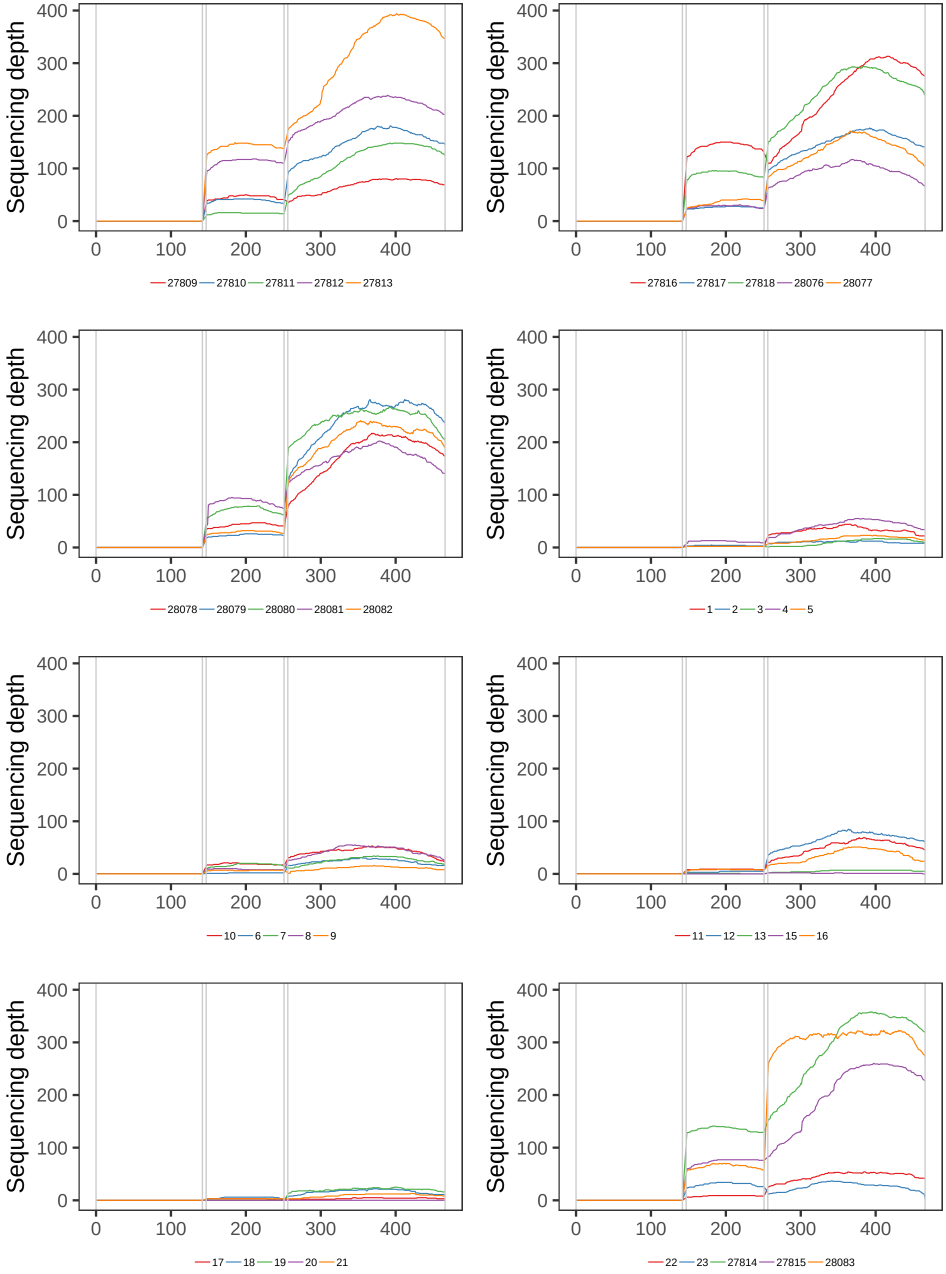
Sequencing depth
0
100
200
300
400
27809
27810
27811
27812
27813
27816
27817
27818
28076
28077
28078
28079
28080
28081
28082
1
2
3
4
5
10
6
7
8
9
11
12
13
15
16
17
18
19
20
21
22
23
27814
27815
28083

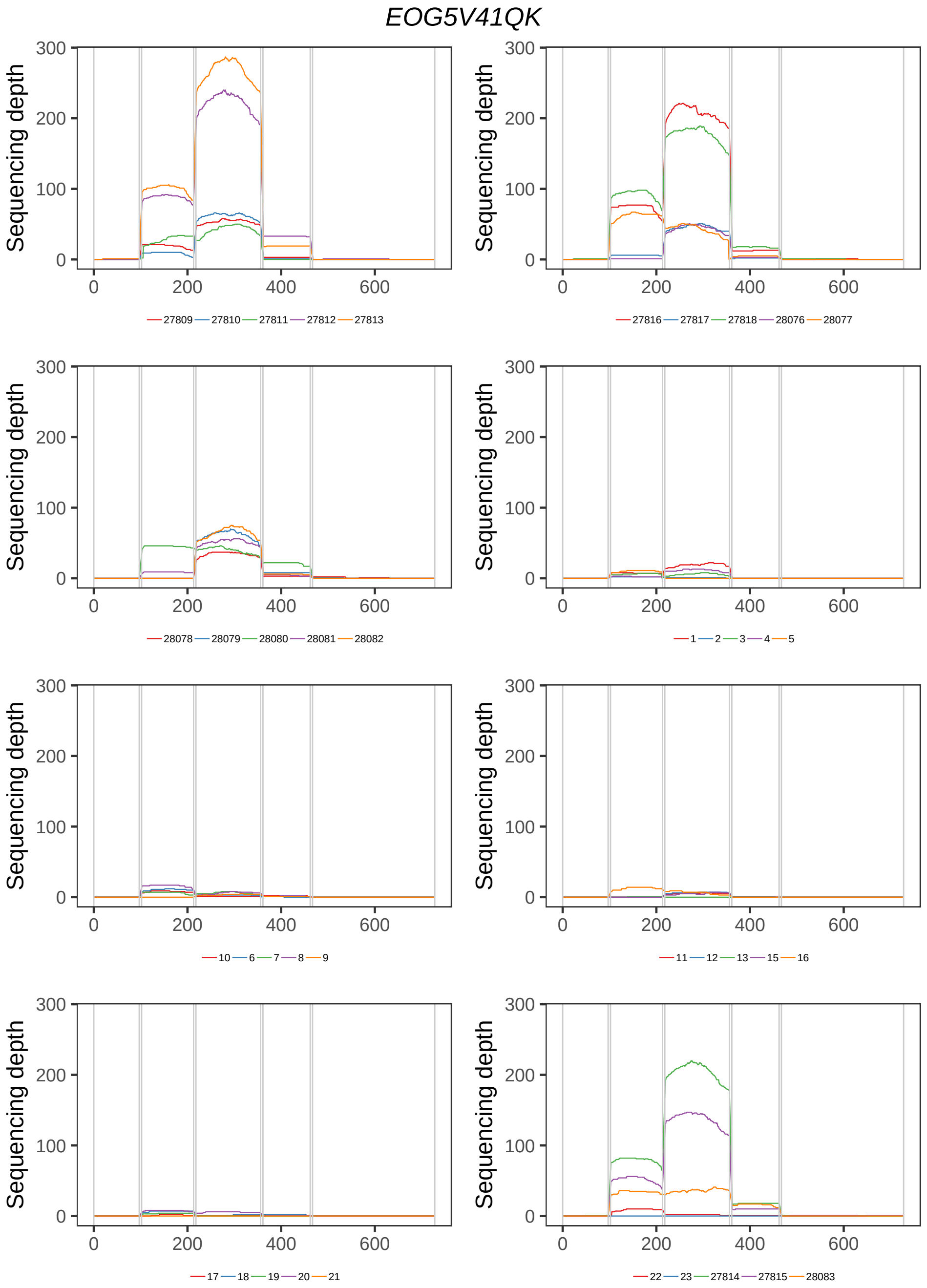
EOG5V41QK
Sequencing depth
0
100
200
300
0
200
400
600
27809 27810 27811 27812 27813
27816 27817 27818 28076 28077
28078 28079 28080 28081 28082
1 2 3 4 5
10 6 7 8 9
11 12 13 15 16
17 18 19 20 21
22 23 27814 27815 28083

EOG50CFXX

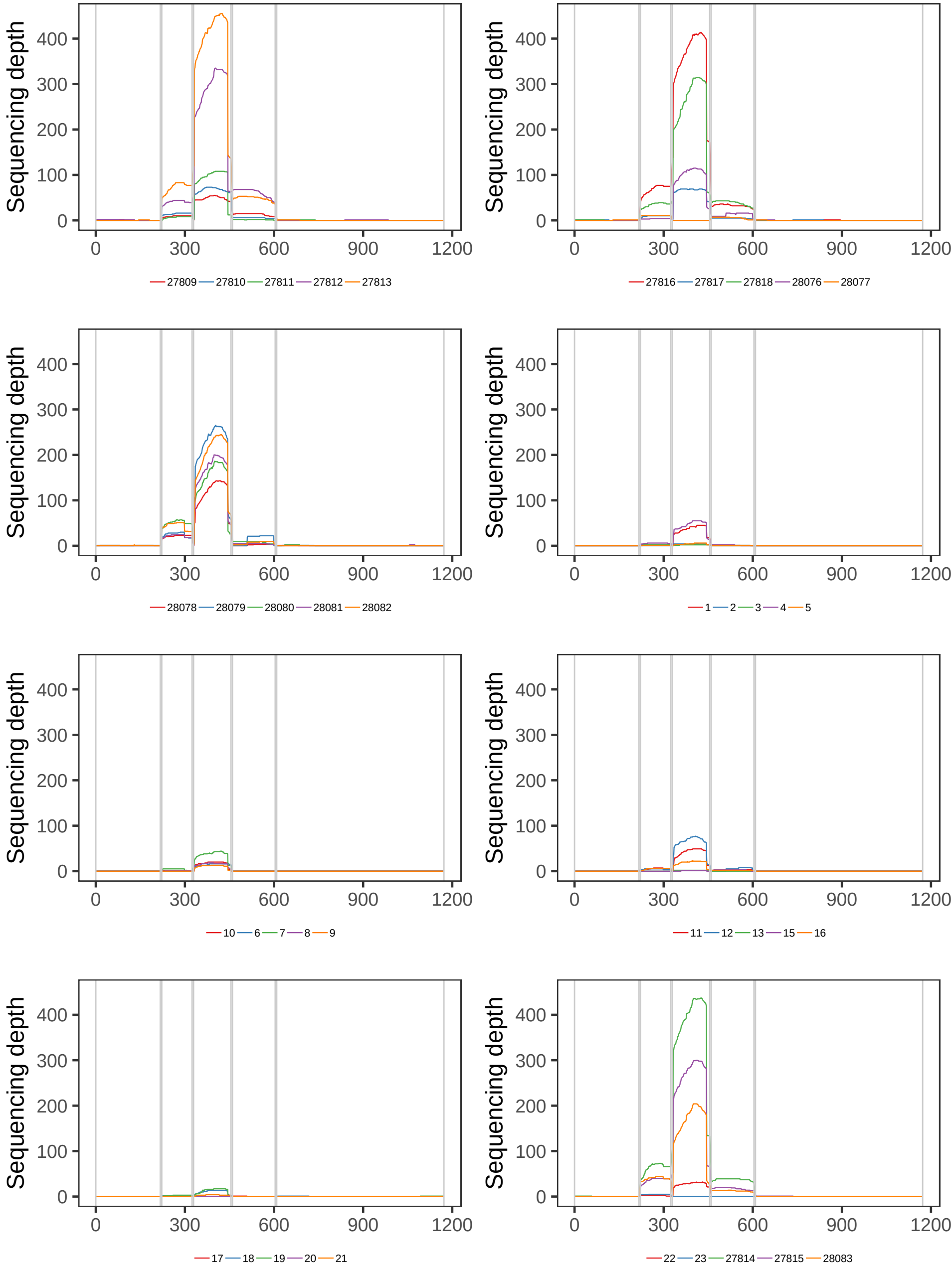
Sequencing depth
0
100
200
300
400
0
300
600
900
1200
27809 27810 27811 27812 27813
27816 27817 27818 28076 28077
28078 28079 28080 28081 28082
1 2 3 4 5
10 6 7 8 9
11 12 13 15 16
17 18 19 20 21
22 23 27814 27815 28083

EOG52NGGC

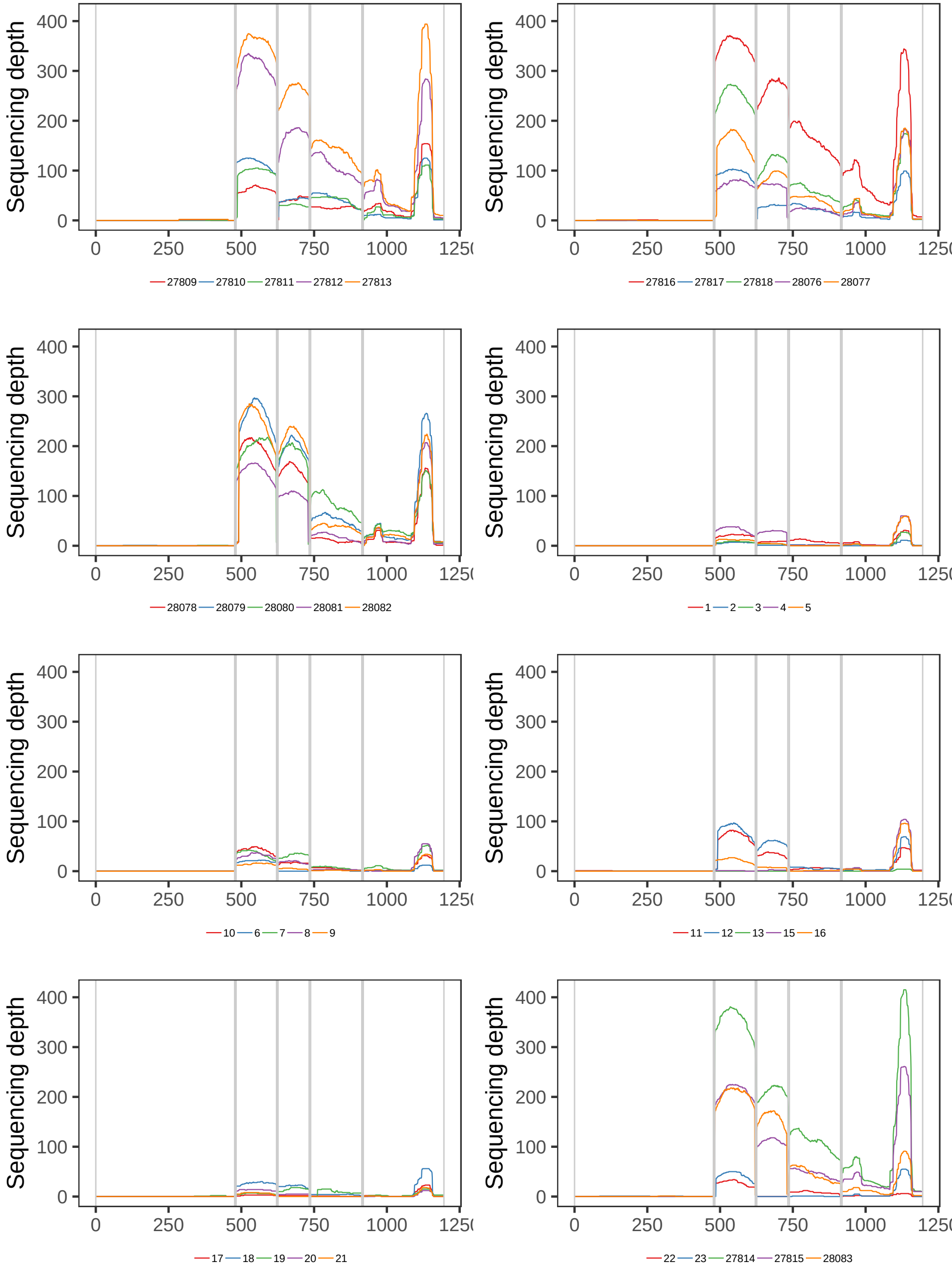
Sequencing depth
0
100
200
300
400
0
250
500
750
1000
1250
27809 27810 27811 27812 27813
27816 27817 27818 28076 28077
28078 28079 28080 28081 28082
1 2 3 4 5
10 6 7 8 9
11 12 13 15 16
17 18 19 20 21
22 23 27814 27815 28083

EOG53R238

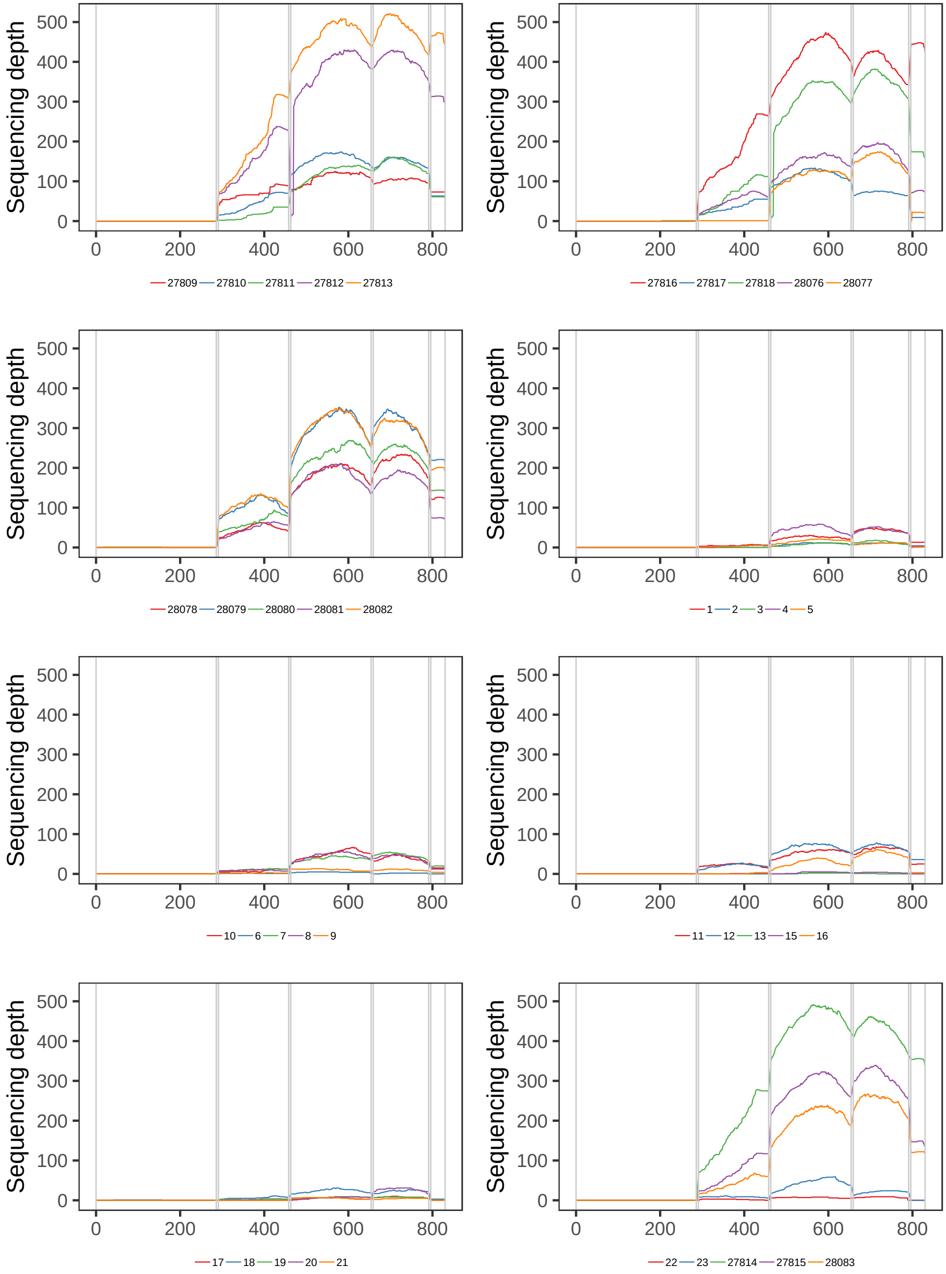
Sequencing depth
0
100
200
300
400
500
0
200
400
600
800
27809 27810 27811 27812 27813
27816 27817 27818 28076 28077
28078 28079 28080 28081 28082
1 2 3 4 5
10 6 7 8 9
11 12 13 15 16
17 18 19 20 21
22 23 27814 27815 28083

EOG56HDSV

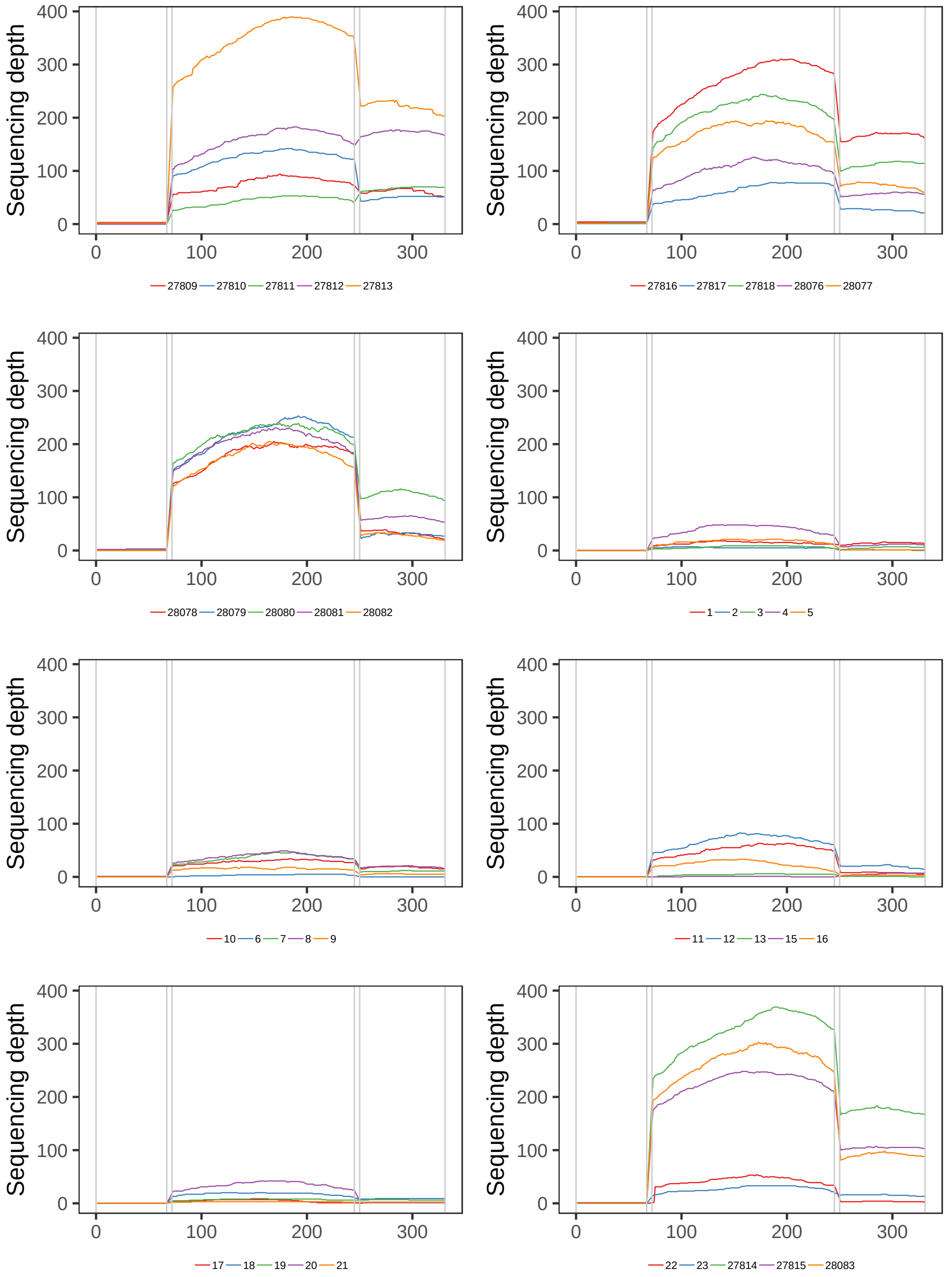
Sequencing depth
0
100
200
300
400
27809 27810 27811 27812 27813
27816 27817 27818 28076 28077
28078 28079 28080 28081 28082
1 2 3 4 5
10 6 7 8 9
11 12 13 15 16
17 18 19 20 21
22 23 27814 27815 28083

EOG56M91Q

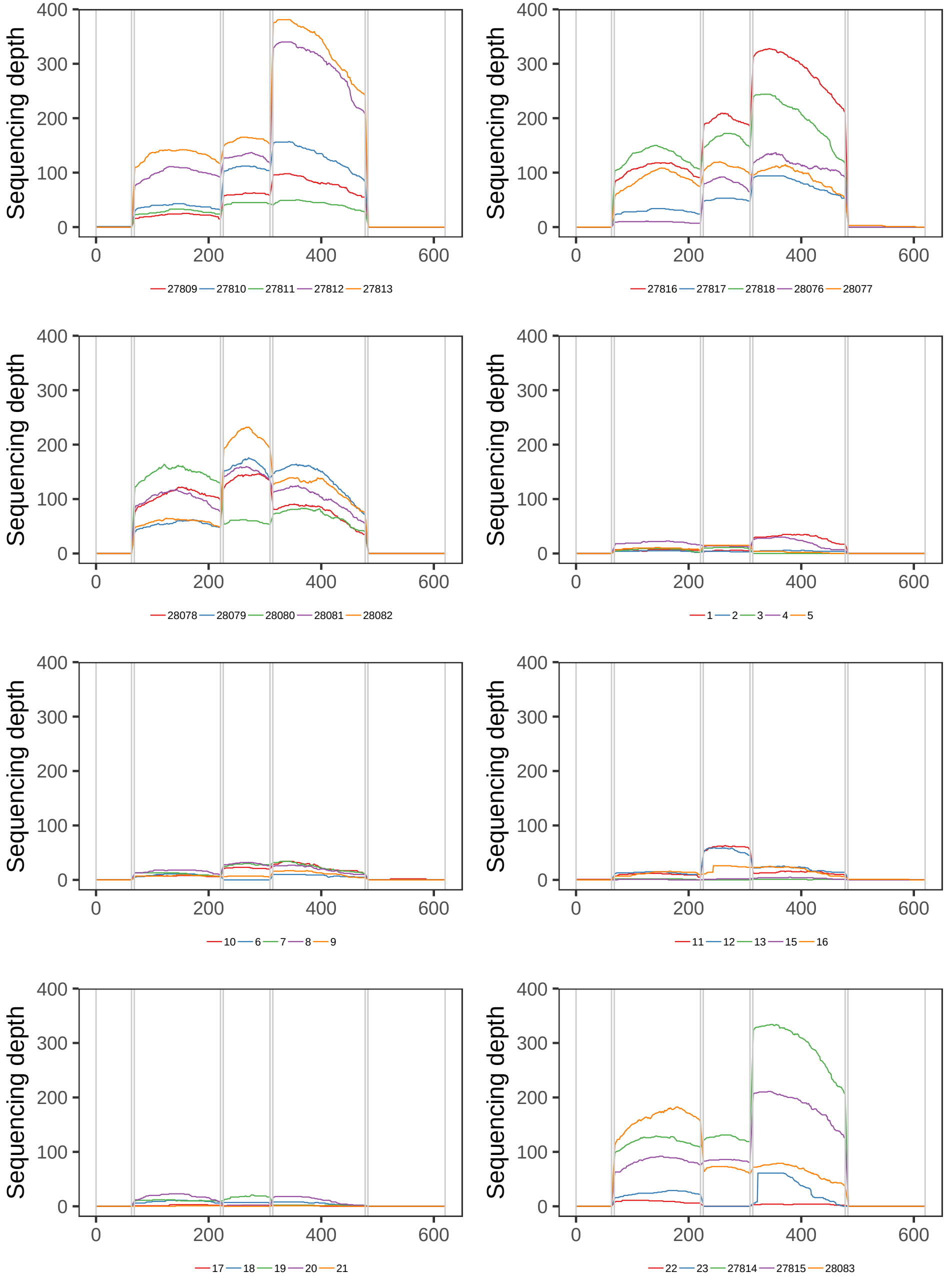

Sequencing depth
0
100
200
300
400
0
200
400
600
27809 27810 27811 27812 27813
27816 27817 27818 28076 28077
28078 28079 28080 28081 28082
1 2 3 4 5
10 6 7 8 9
11 12 13 15 16
17 18 19 20 21
22 23 27814 27815 28083

EOG5BK3KT

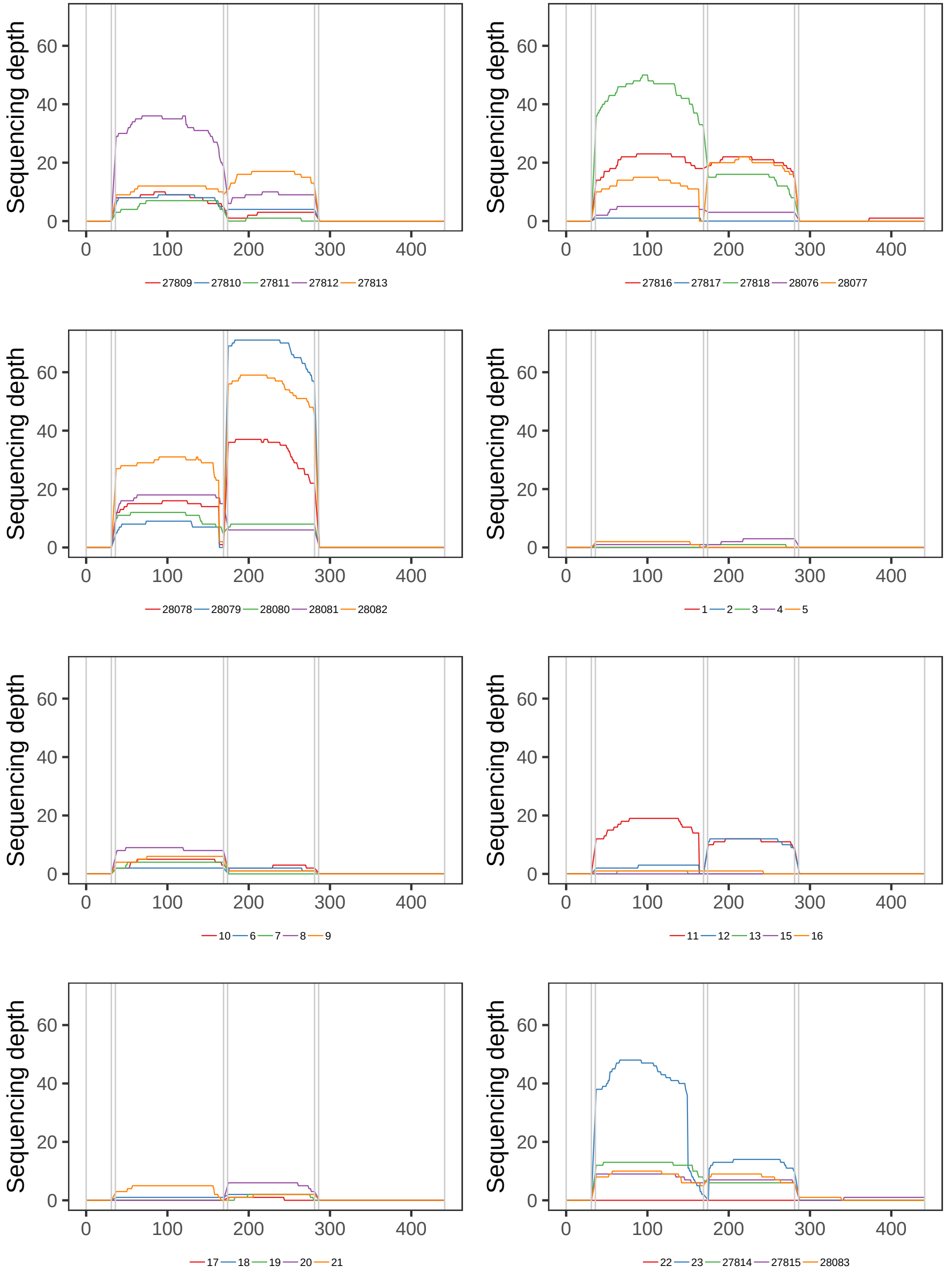
Sequencing depth
0
20
40
60
0
100
200
300
400
27809
27810
27811
27812
27813
27816
27817
27818
28076
28077
28078
28079
28080
28081
28082
1
2
3
4
5
10
6
7
8
9
11
12
13
15
16
17
18
19
20
21
22
23
27814
27815
28083

EOG5G1JXZ

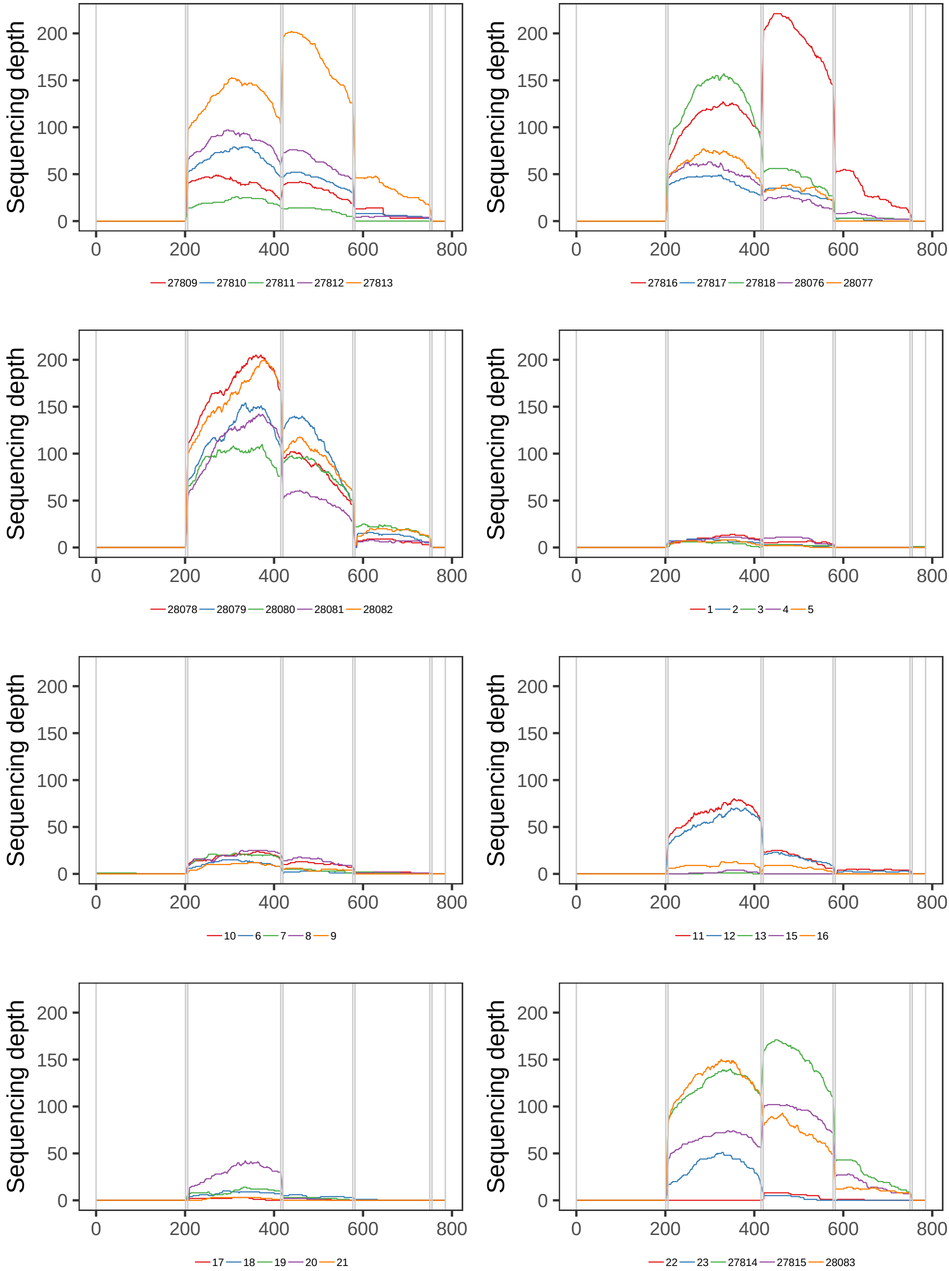
Sequencing depth
0
50
100
150
200
0
200
400
600
800
27809 27810 27811 27812 27813
27816 27817 27818 28076 28077
28078 28079 28080 28081 28082
1 2 3 4 5
10 6 7 8 9
11 12 13 15 16
17 18 19 20 21
22 23 27814 27815 28083

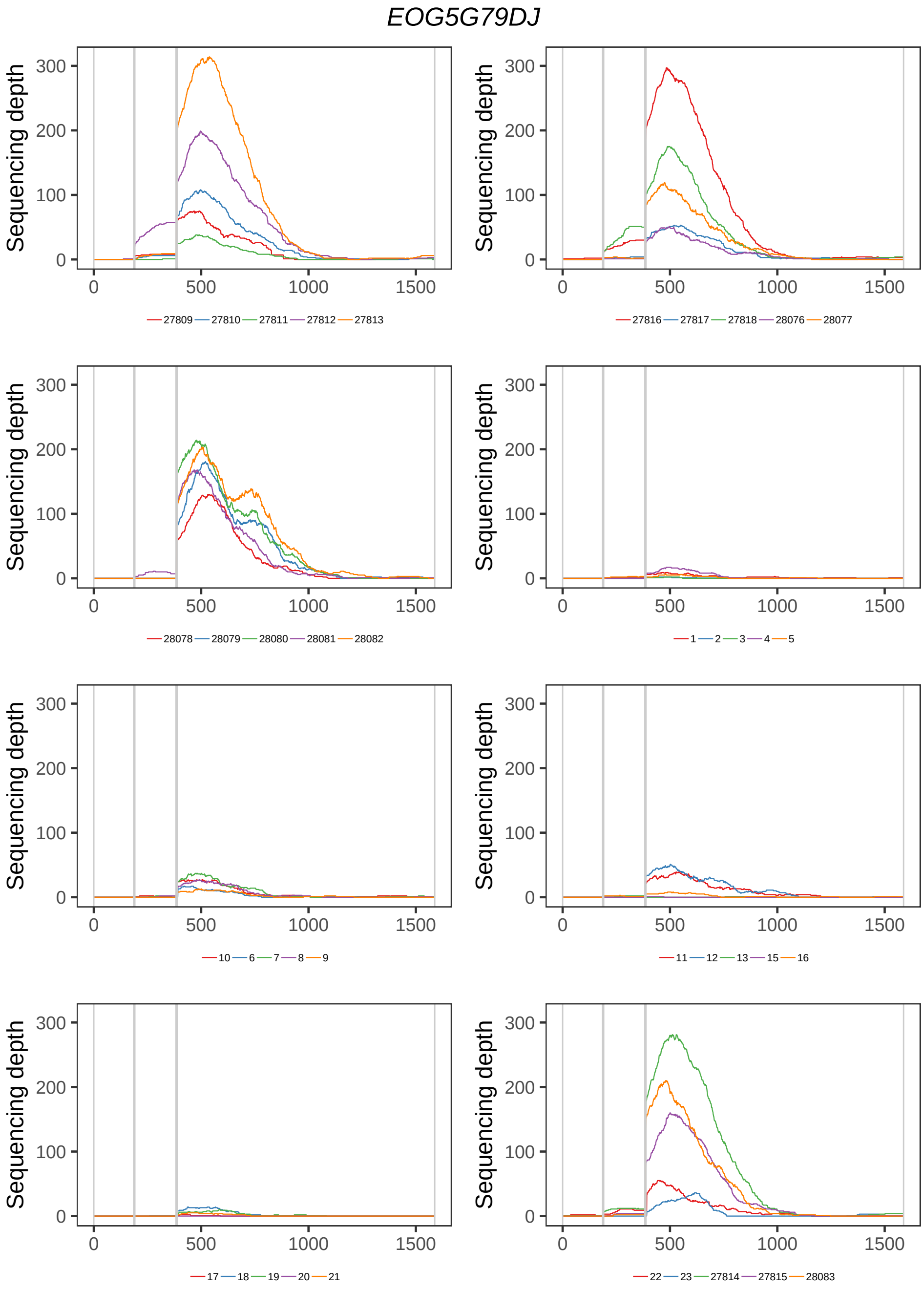
EOG5G79DJ
Sequencing depth
0
100
200
300
0
500
1000
1500
27809 27810 27811 27812 27813
27816 27817 27818 28076 28077
28078 28079 28080 28081 28082
1 2 3 4 5
10 6 7 8 9
11 12 13 15 16
17 18 19 20 21
22 23 27814 27815 28083

EOG5HDR91

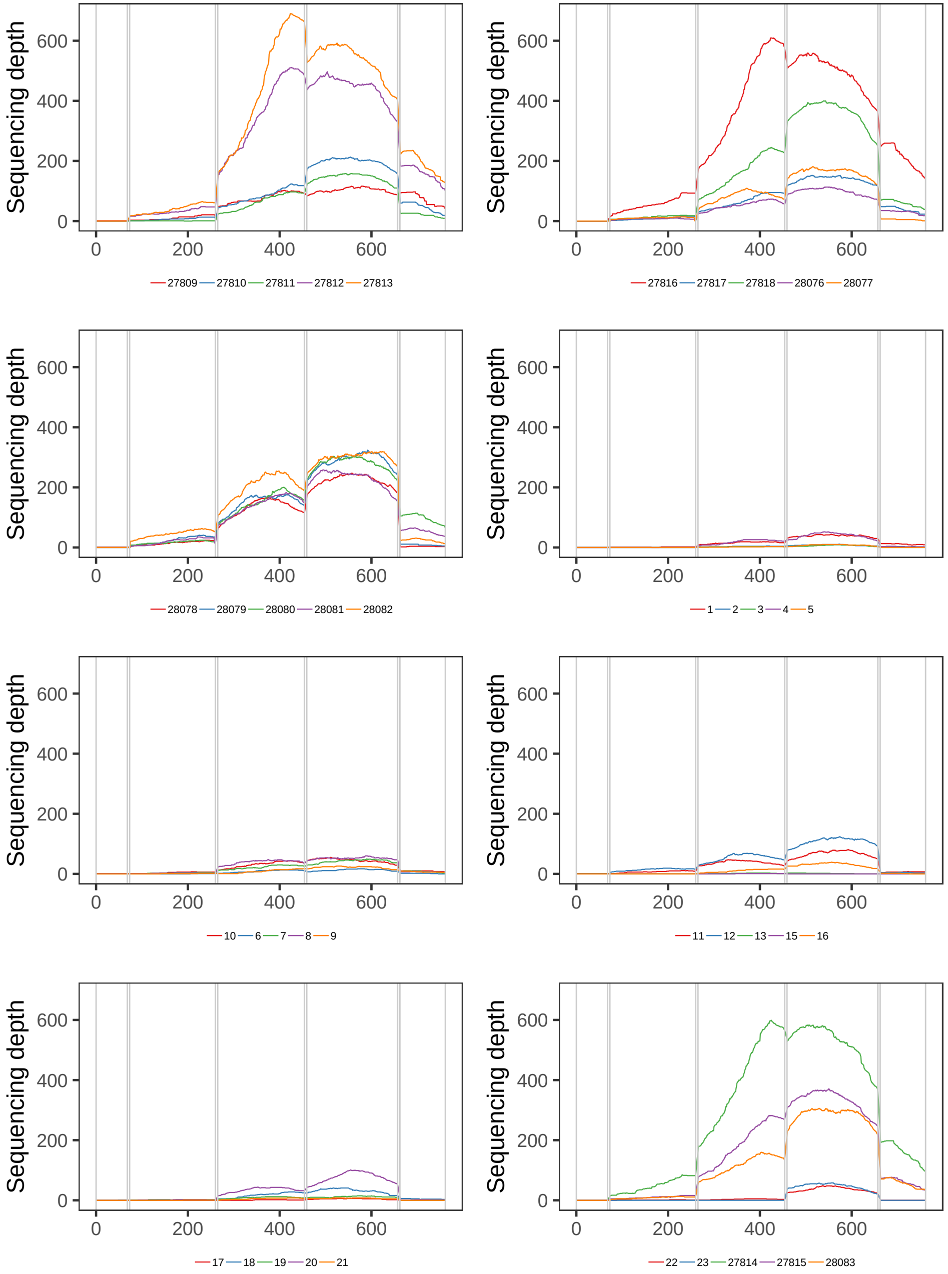
Sequencing depth
0
200
400
600
27809 27810 27811 27812 27813
27816 27817 27818 28076 28077
28078 28079 28080 28081 28082
1 2 3 4 5
10 6 7 8 9
11 12 13 15 16
17 18 19 20 21
22 23 27814 27815 28083

EOG5KKWJN

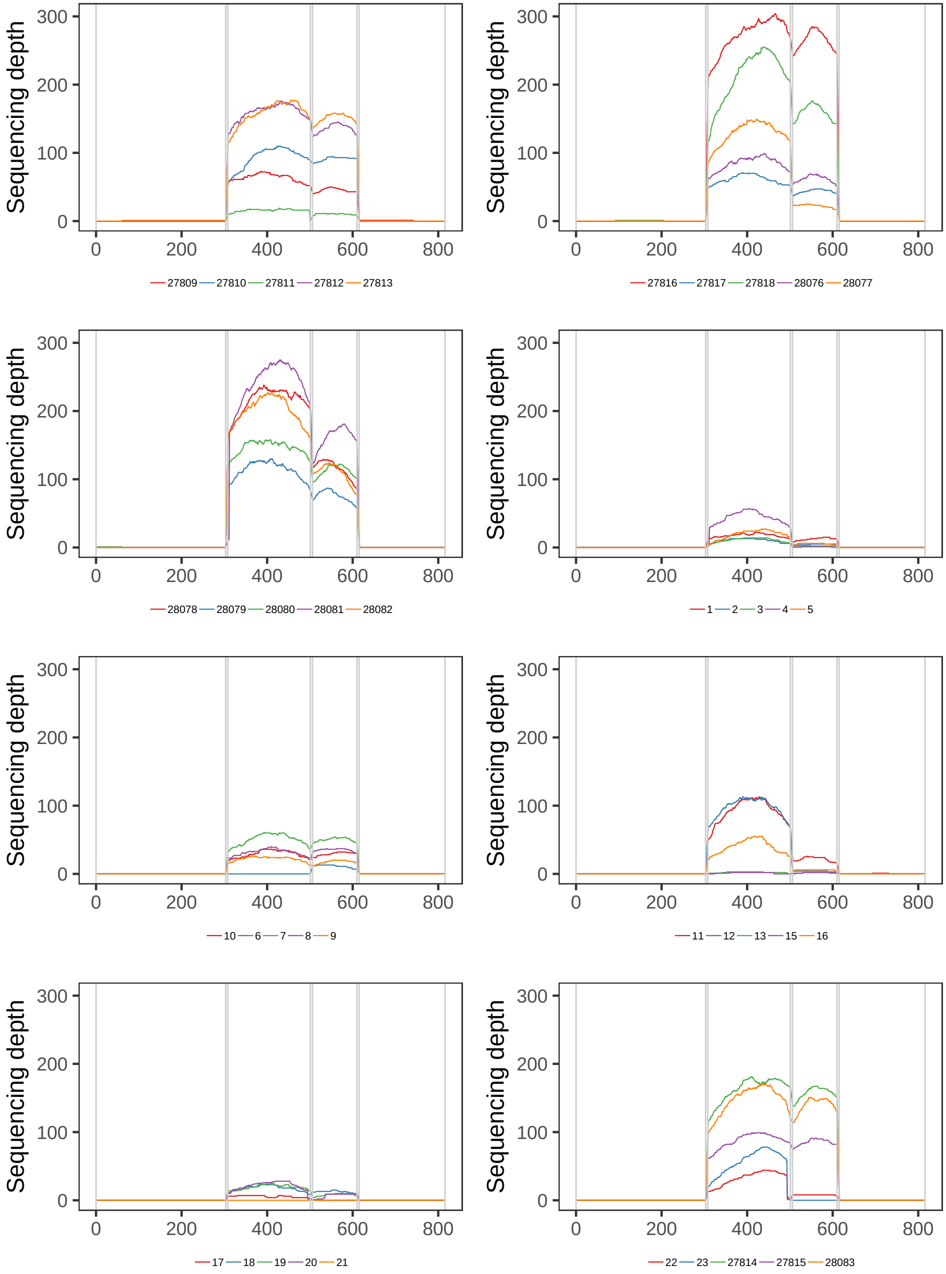
Sequencing depth
0
100
200
300
0
200
400
600
800
27809 27810 27811 27812 27813
27816 27817 27818 28076 28077
28078 28079 28080 28081 28082
1 2 3 4 5
10 6 7 8 9
11 12 13 15 16
17 18 19 20 21
22 23 27814 27815 28083

EOG5SN044

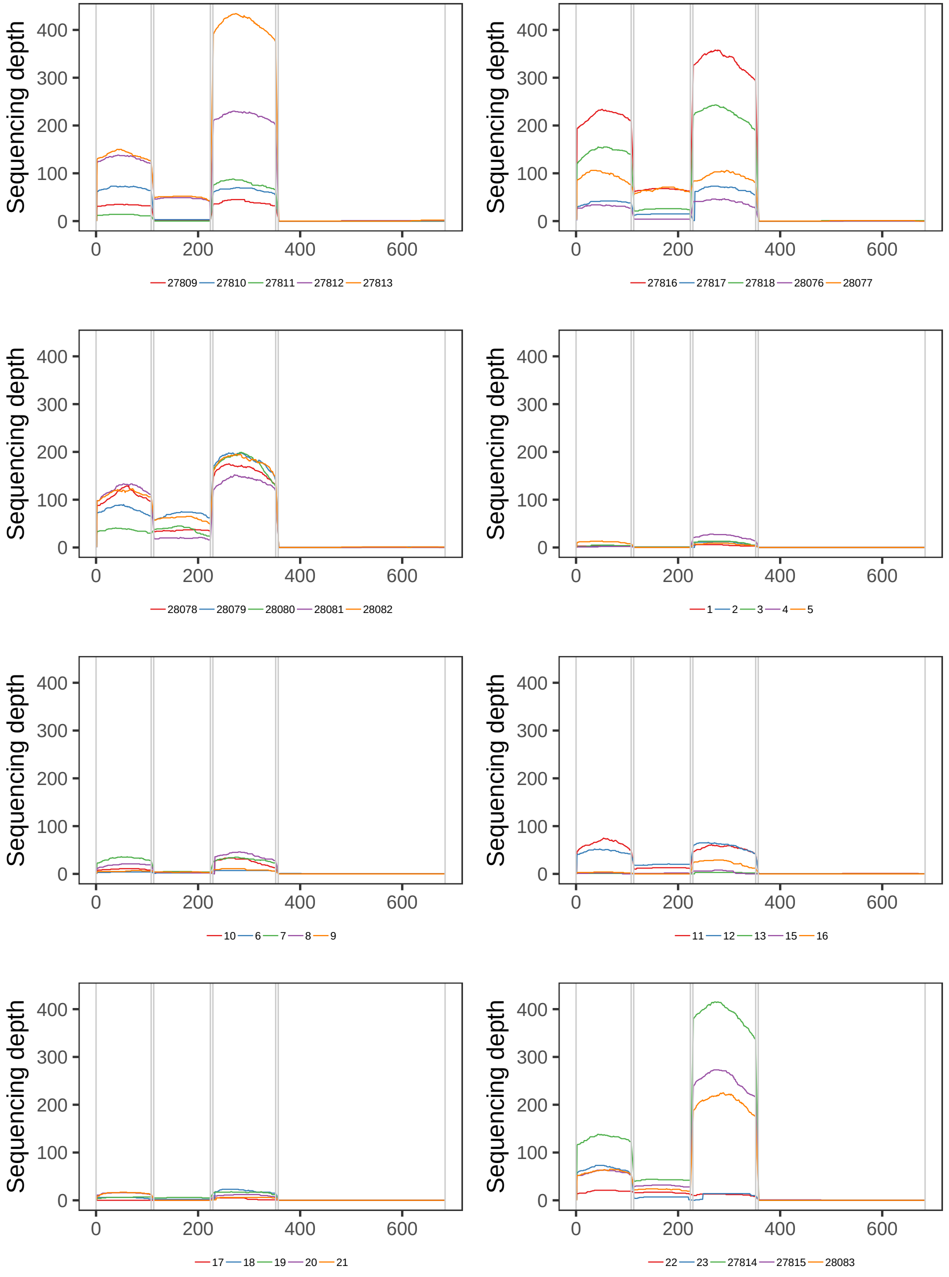

Sequencing depth
0
100
200
300
400
0
200
400
600
27809 27810 27811 27812 27813
27816 27817 27818 28076 28077
28078 28079 28080 28081 28082
1 2 3 4 5
10 6 7 8 9
11 12 13 15 16
17 18 19 20 21
22 23 27814 27815 28083

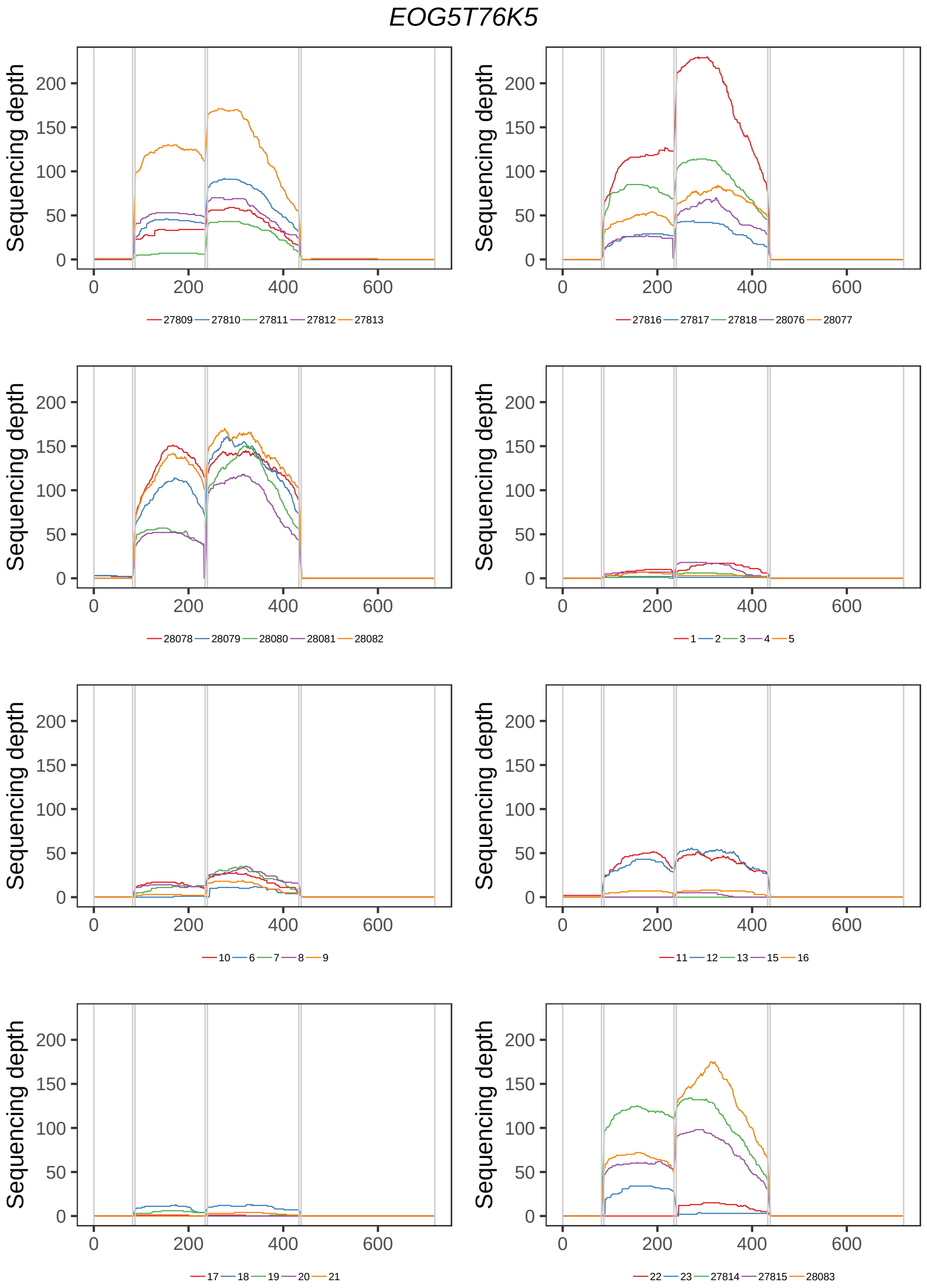
EOG5T76K5
Sequencing depth
0
50
100
150
200
0
200
400
600
27809 27810 27811 27812 27813
27816 27817 27818 28076 28077
28078 28079 28080 28081 28082
1 2 3 4 5
10 6 7 8 9
11 12 13 15 16
17 18 19 20 21
22 23 27814 27815 28083

EOG5W0VVN

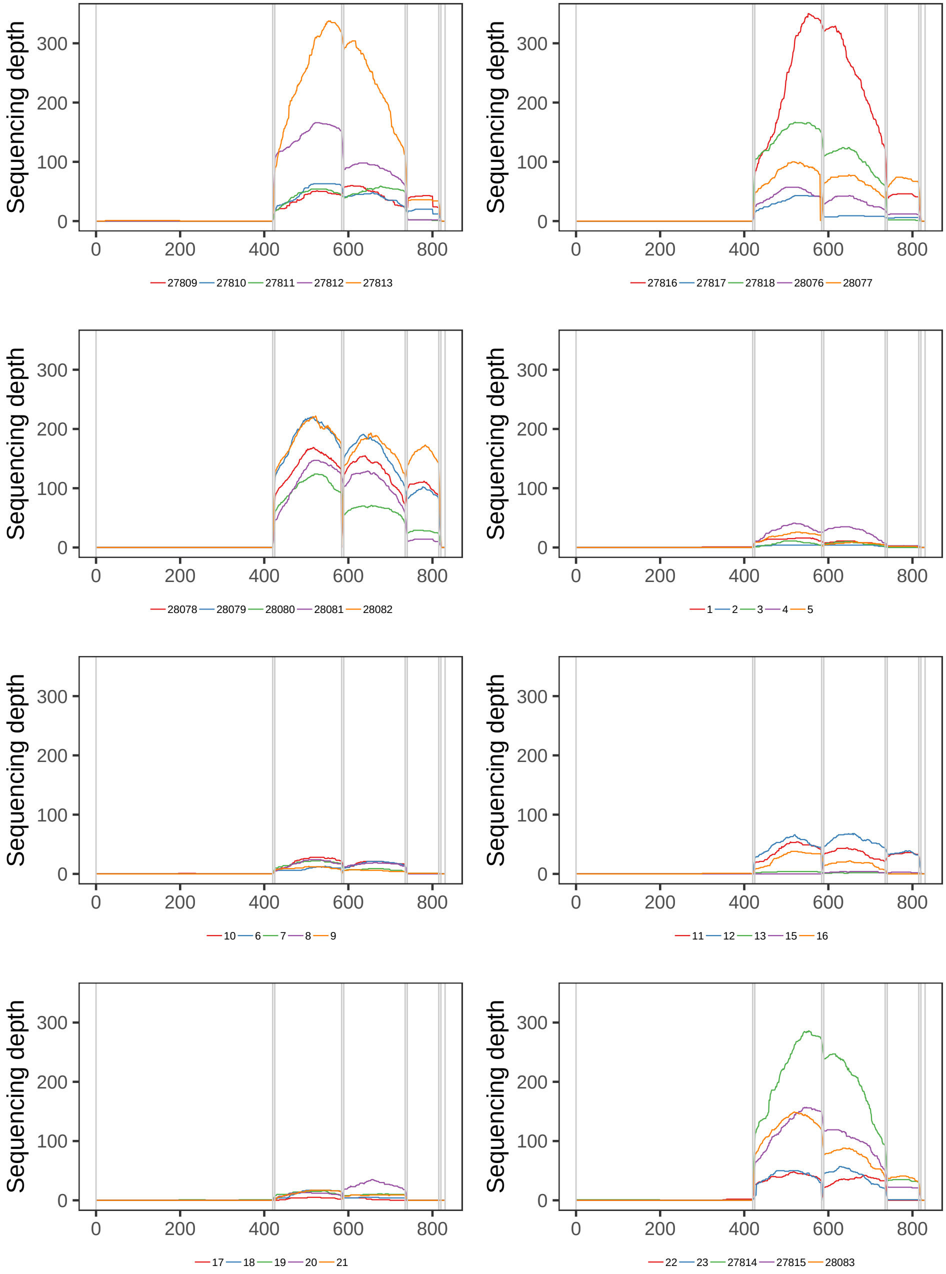
Sequencing depth
0
100
200
300
0
200
400
600
800
27809
27810
27811
27812
27813
27816
27817
27818
28076
28077
28078
28079
28080
28081
28082
1
2
3
4
5
10
6
7
8
9
11
12
13
15
16
17
18
19
20
21
22
23
27814
27815
28083

EOG5X3FHN

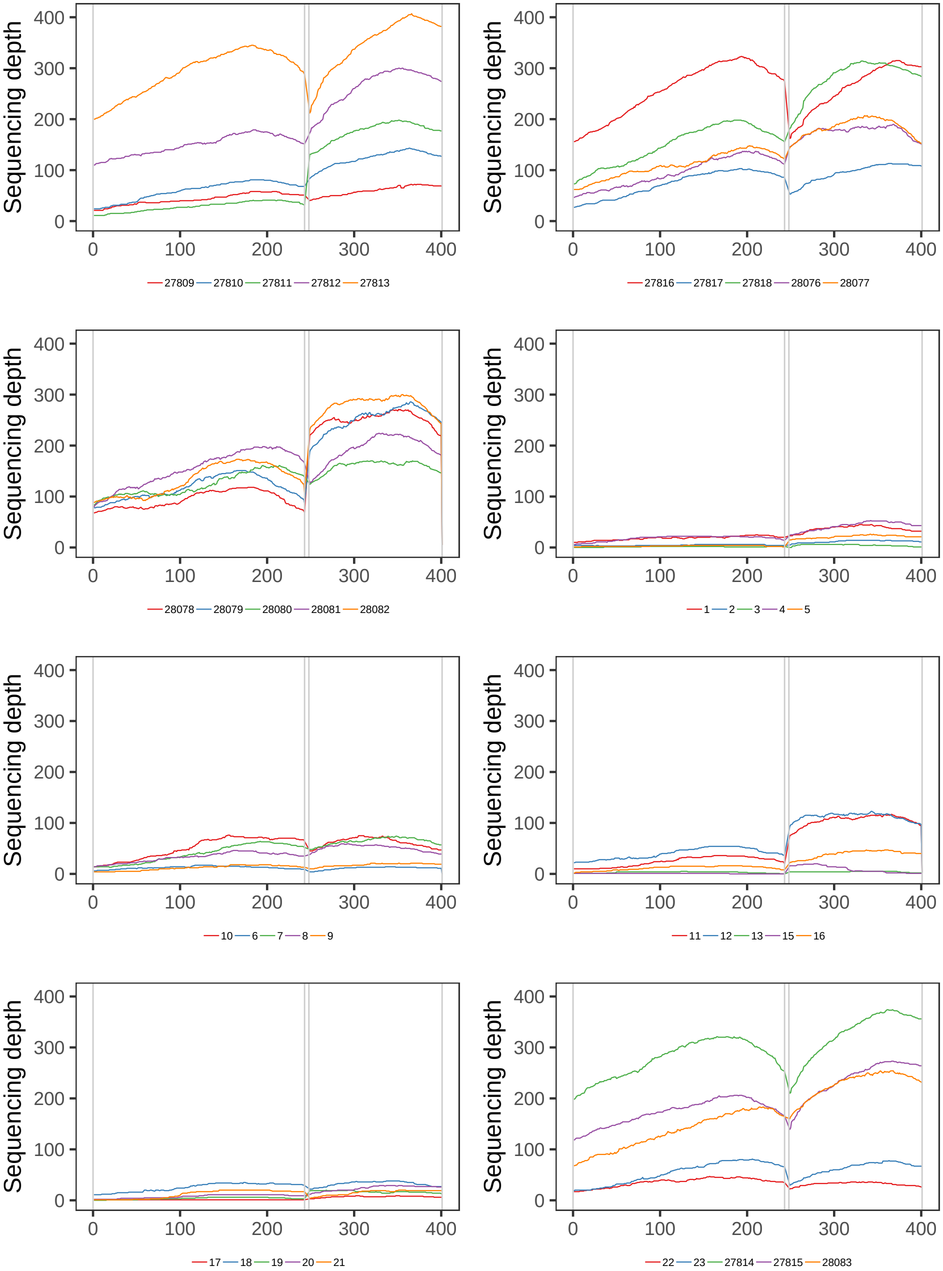
Sequencing depth
0
100
200
300
400
27809 27810 27811 27812 27813
27816 27817 27818 28076 28077
28078 28079 28080 28081 28082
1 2 3 4 5
10 6 7 8 9
11 12 13 15 16
17 18 19 20 21
22 23 27814 27815 28083

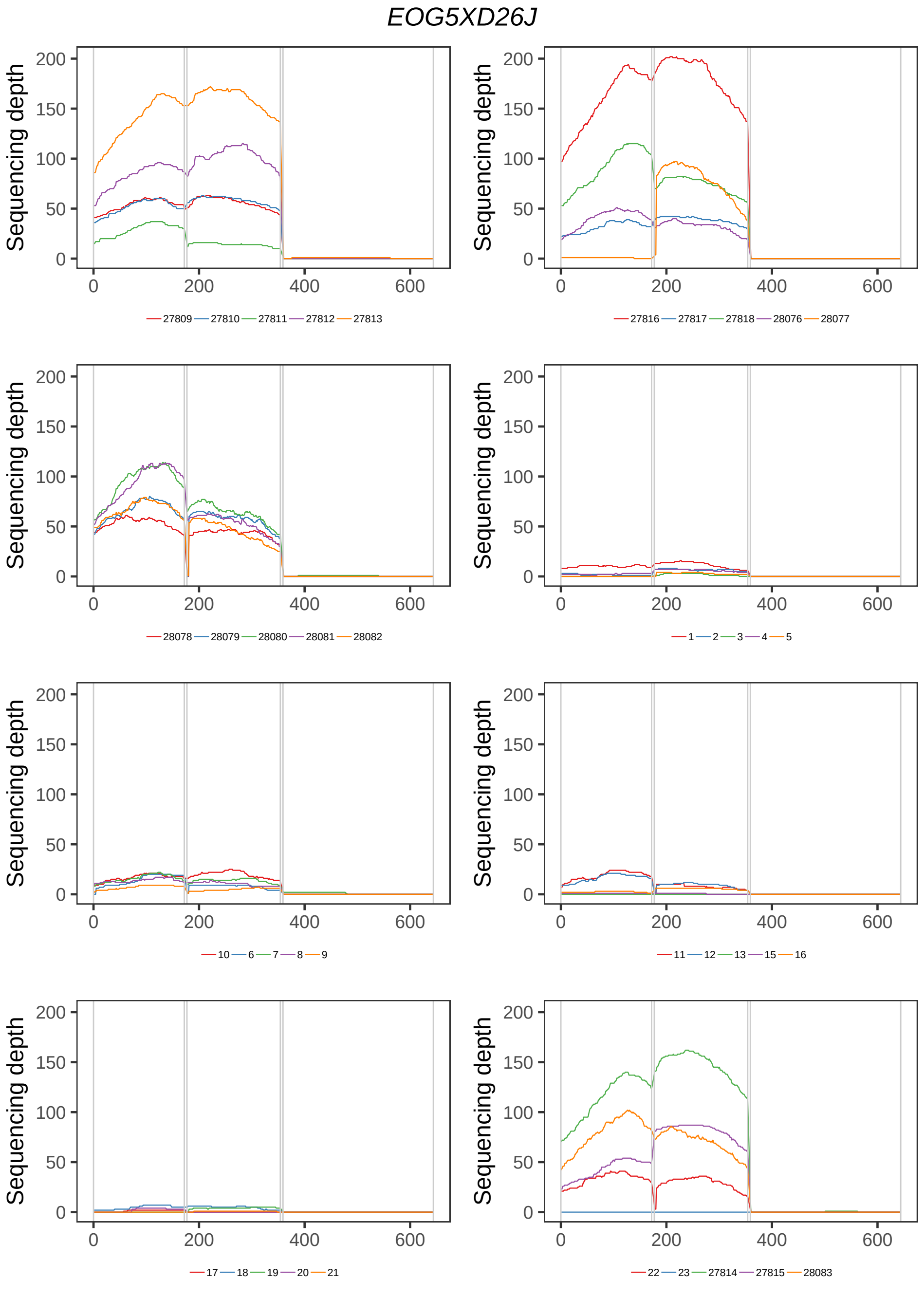
EOG5XD26J
Sequencing depth
0
50
100
150
200
0
200
400
600
27809
27810
27811
27812
27813
27816
27817
27818
28076
28077
28078
28079
28080
28081
28082
1
2
3
4
5
10
6
7
8
9
11
12
13
15
16
17
18
19
20
21
22
23
27814
27815
28083

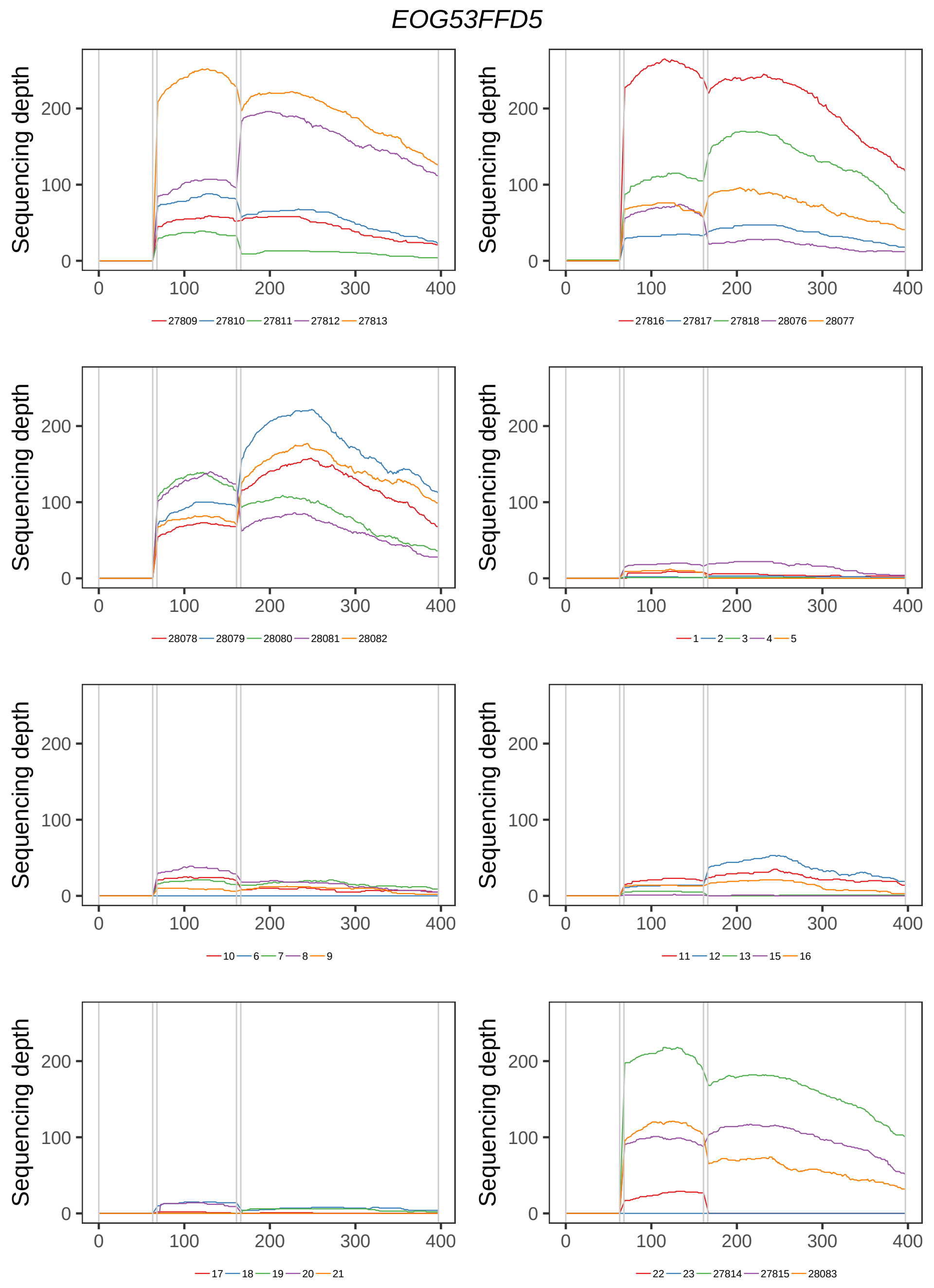
EOG53FFD5
Sequencing depth
0
100
200
300
400
27809
27810
27811
27812
27813
27816
27817
27818
28076
28077
28078
28079
28080
28081
28082
1
2
3
4
5
10
6
7
8
9
11
12
13
15
16
17
18
19
20
21
22
23
27814
27815
28083

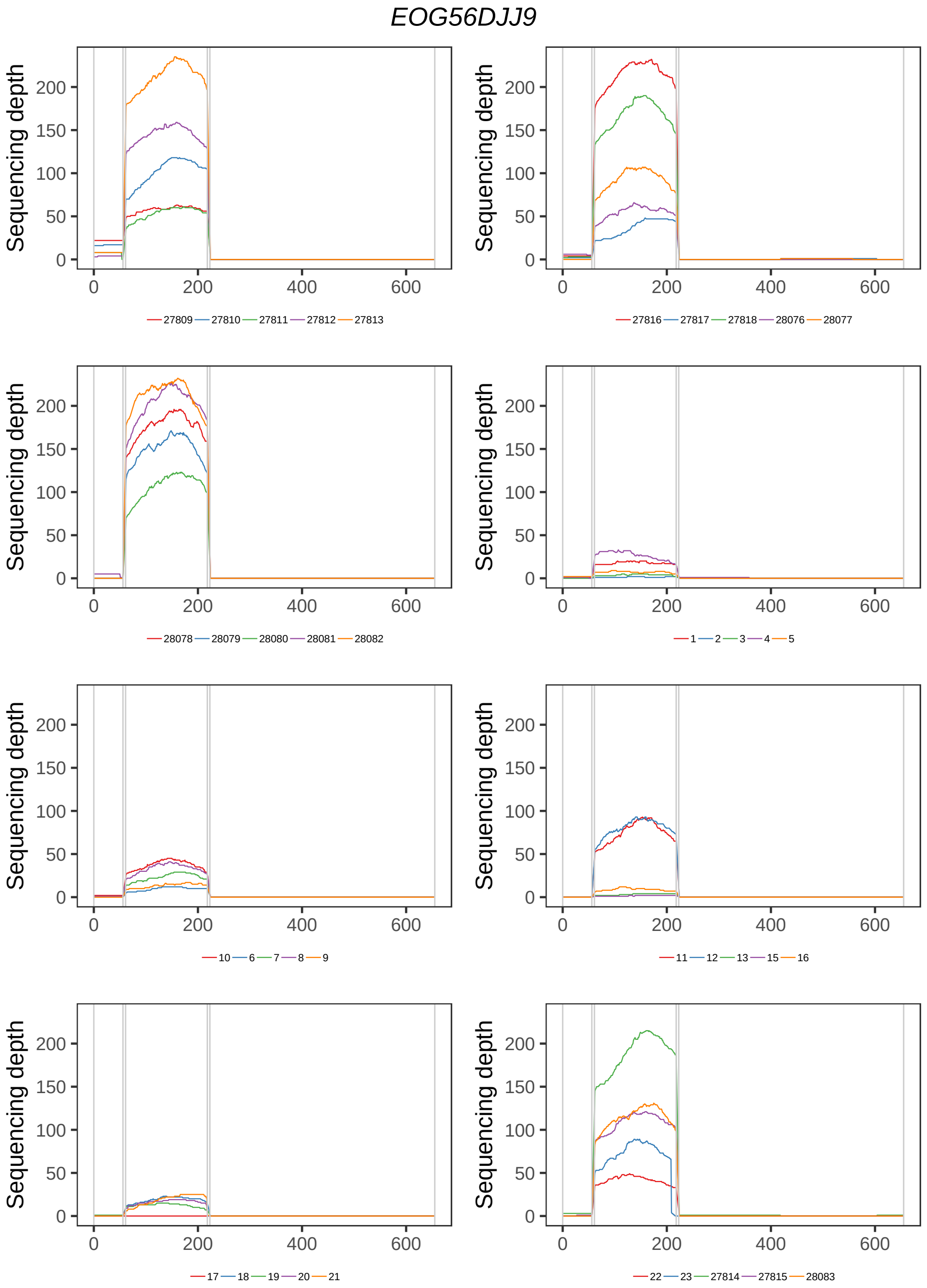
EOG56DJJ9
Sequencing depth
0
50
100
150
200
0
200
400
600
27809 27810 27811 27812 27813
27816 27817 27818 28076 28077
28078 28079 28080 28081 28082
1 2 3 4 5
10 6 7 8 9
11 12 13 15 16
17 18 19 20 21
22 23 27814 27815 28083

EOG576HG6

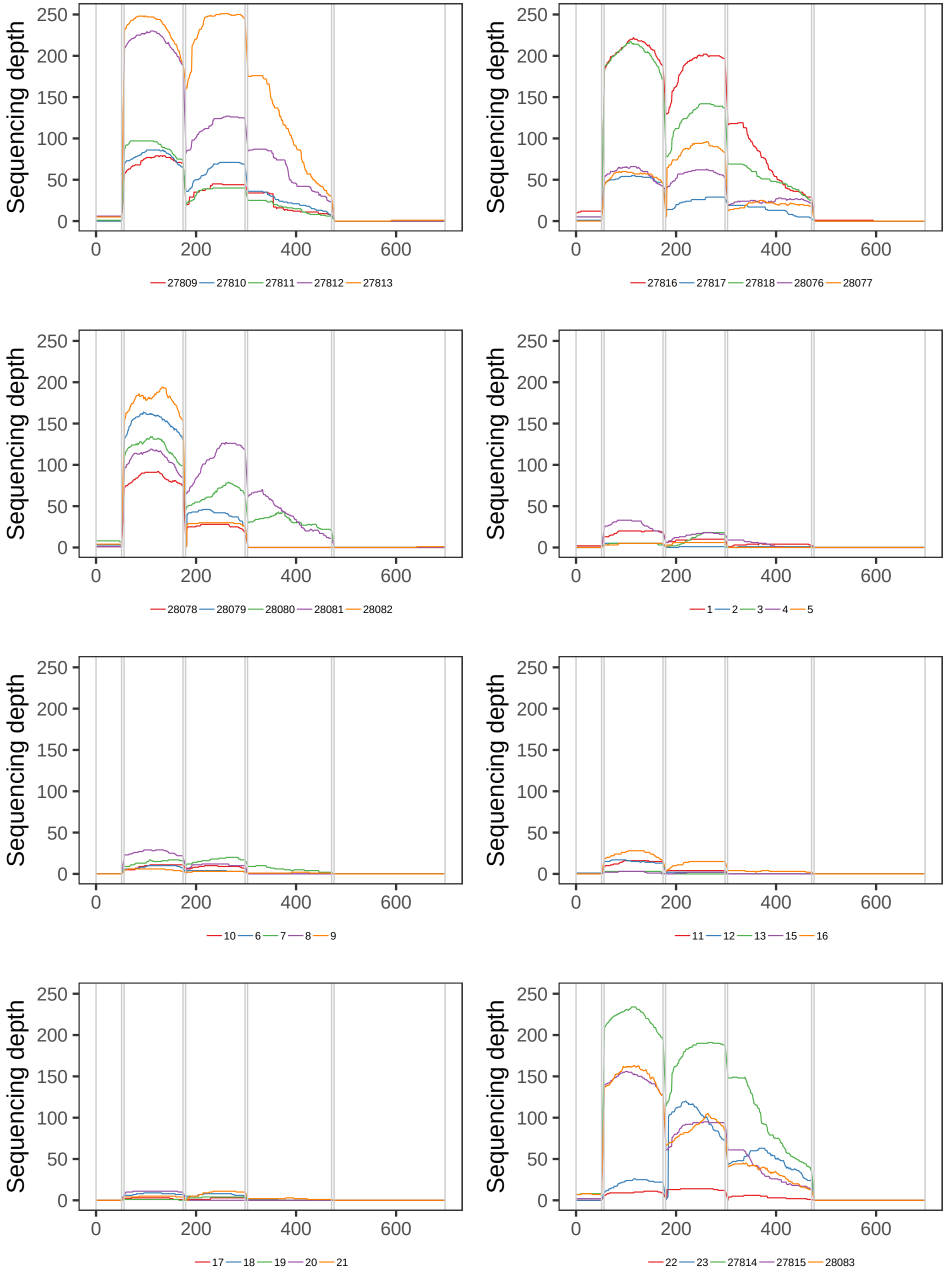
Sequencing depth
27809 27810 27811 27812 27813
27816 27817 27818 28076 28077
28078 28079 28080 28081 28082
1 2 3 4 5
10 6 7 8 9
11 12 13 15 16
17 18 19 20 21
22 23 27814 27815 28083

EOG57H457

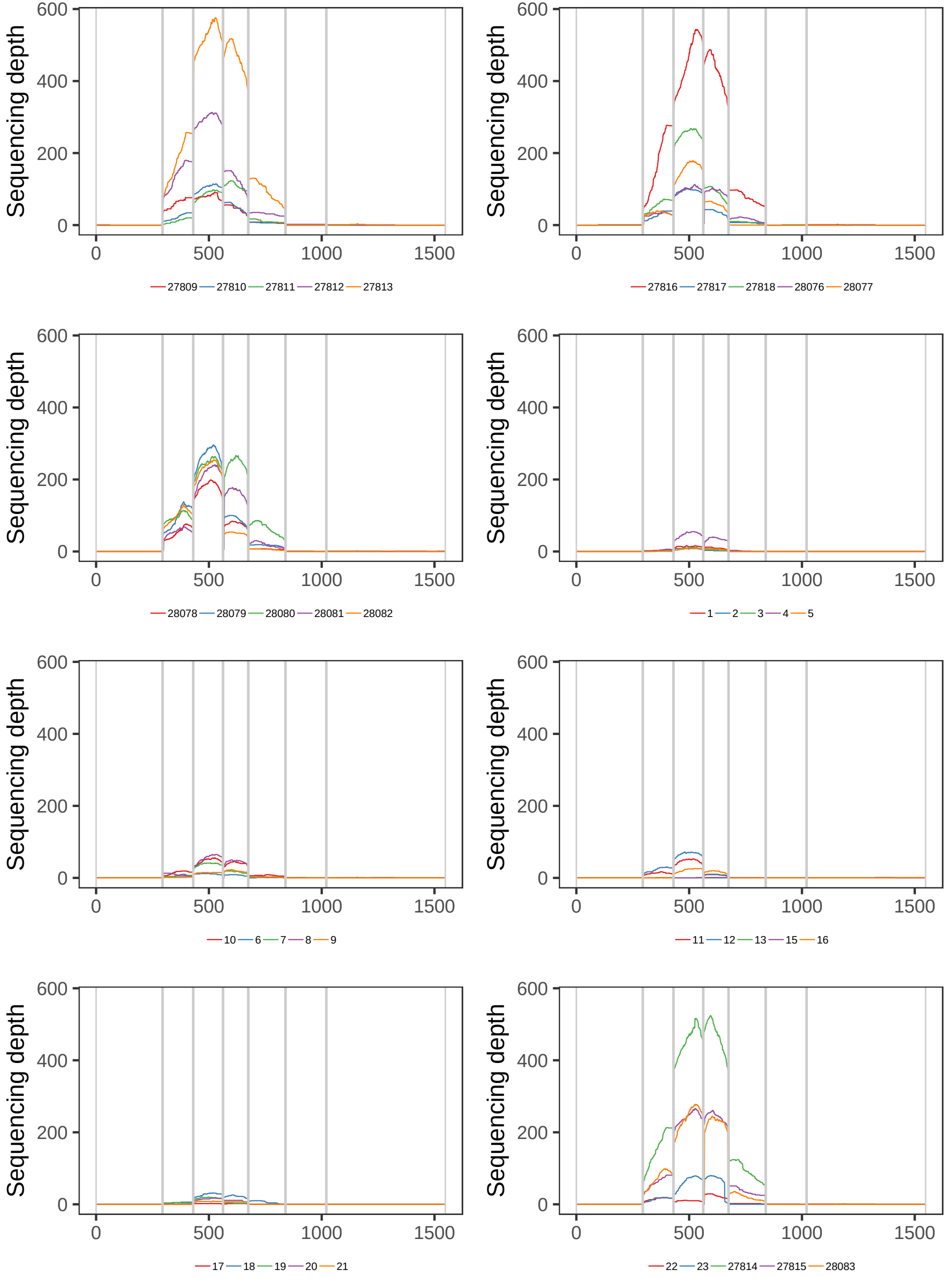
Sequencing depth
0
200
400
600
0
500
1000
1500
27809 27810 27811 27812 27813
27816 27817 27818 28076 28077
28078 28079 28080 28081 28082
1 2 3 4 5
10 6 7 8 9
11 12 13 15 16
17 18 19 20 21
22 23 27814 27815 28083

EOG59KD67

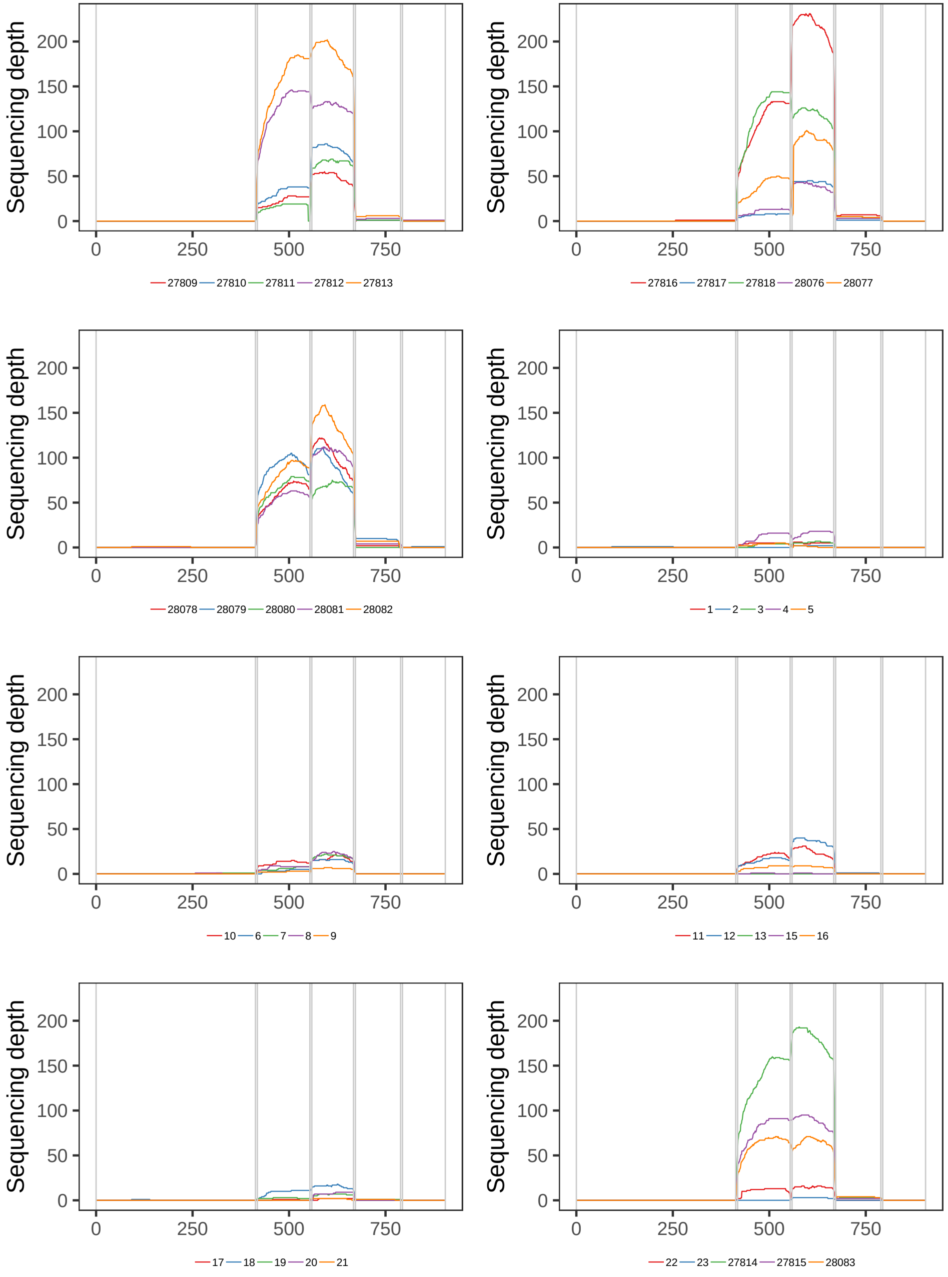
Sequencing depth
0
50
100
150
200
0
250
500
750
27809 27810 27811 27812 27813
27816 27817 27818 28076 28077
28078 28079 28080 28081 28082
1 2 3 4 5
10 6 7 8 9
11 12 13 15 16
17 18 19 20 21
22 23 27814 27815 28083

EOG5BCC40

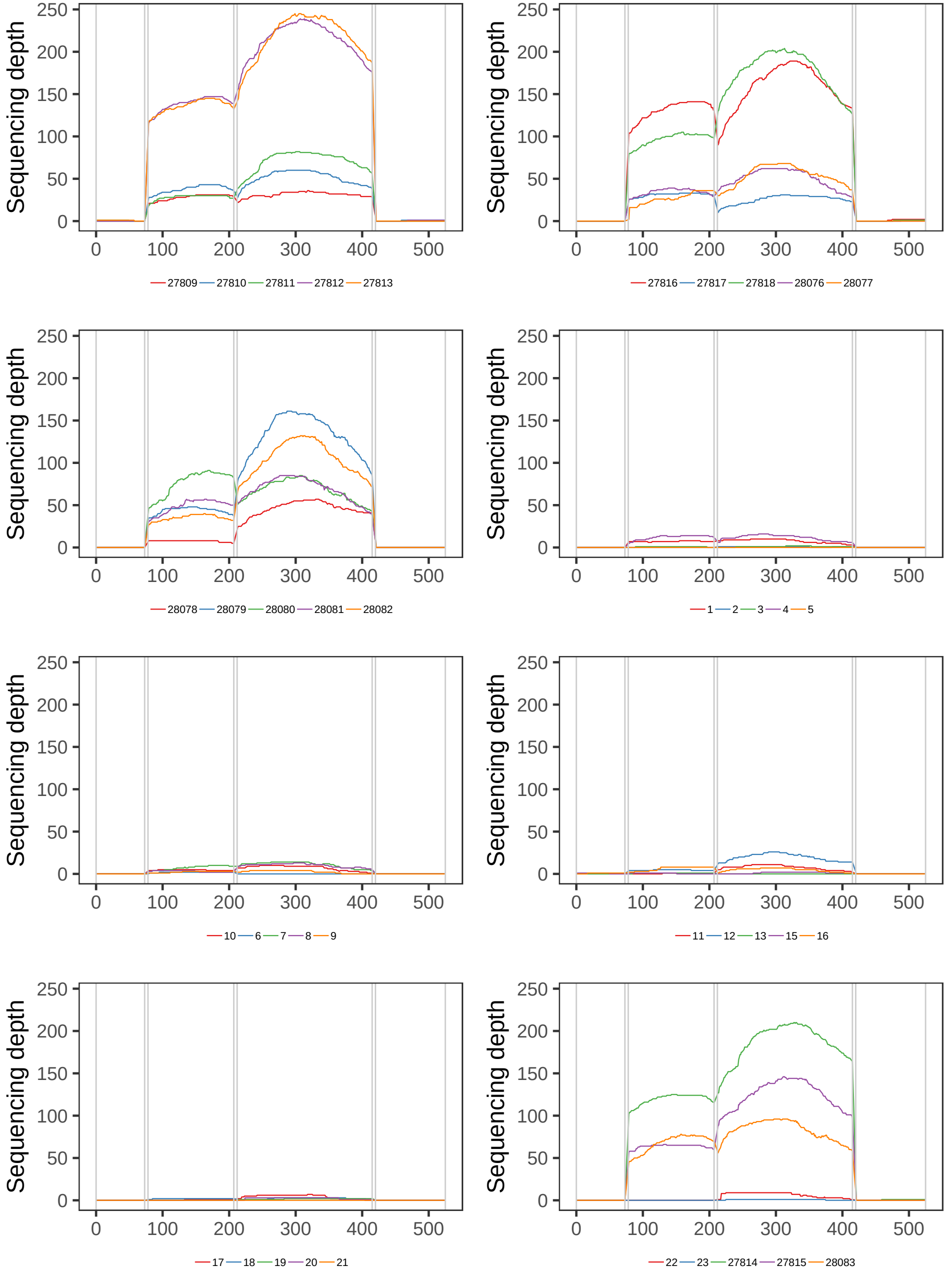
Sequencing depth
0
50
100
150
200
250
0
100
200
300
400
500
27809 27810 27811 27812 27813
27816 27817 27818 28076 28077
28078 28079 28080 28081 28082
1 2 3 4 5
10 6 7 8 9
11 12 13 15 16
17 18 19 20 21
22 23 27814 27815 28083

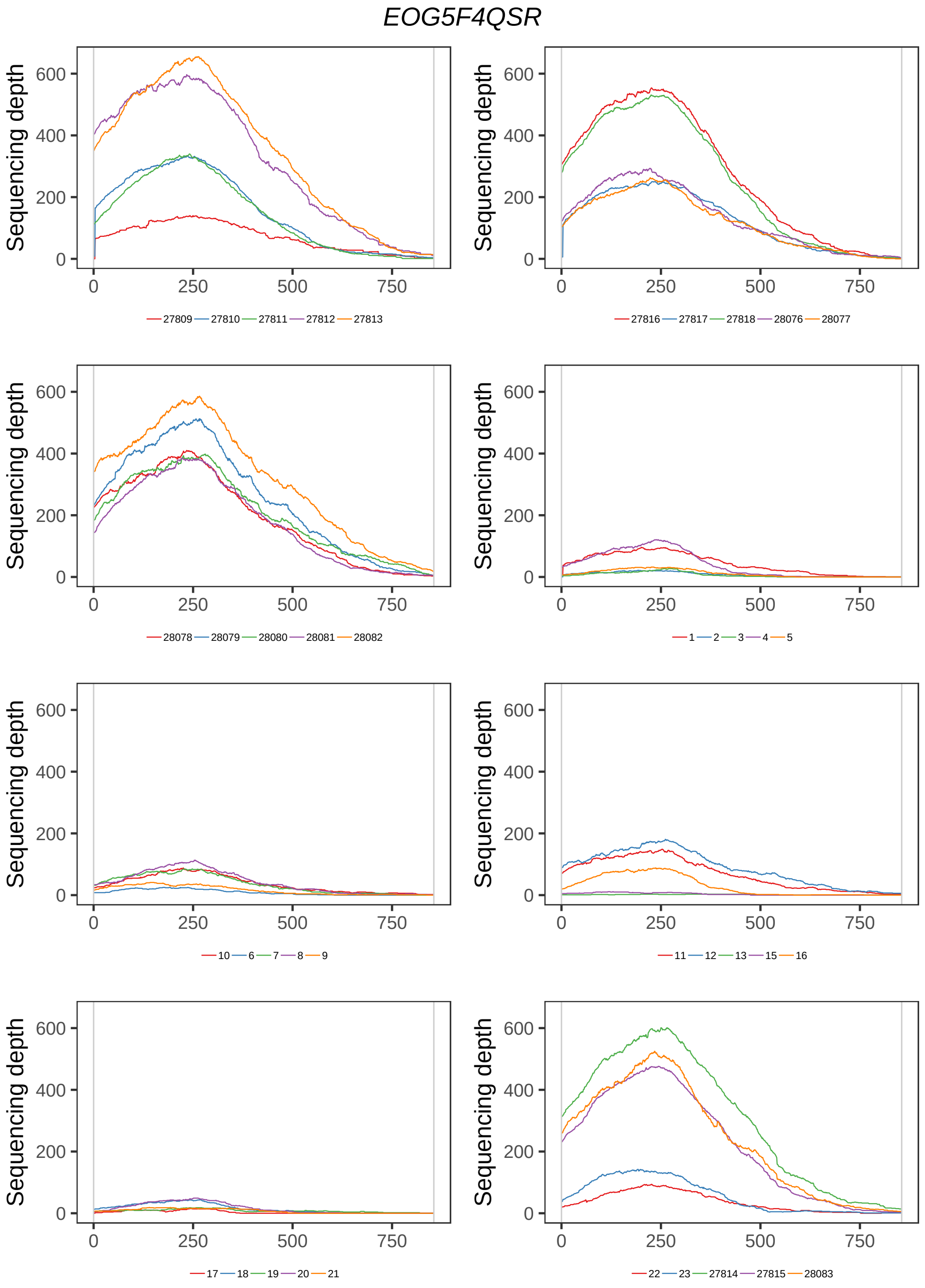
EOG5F4QSR
Sequencing depth
600
400
200
0
0
250
500
750
27809
27810
27811
27812
27813
27816
27817
27818
28076
28077
28078
28079
28080
28081
28082
1
2
3
4
5
10
6
7
8
9
11
12
13
15
16
17
18
19
20
21
22
23
27814
27815
28083

EOG5GTHVK

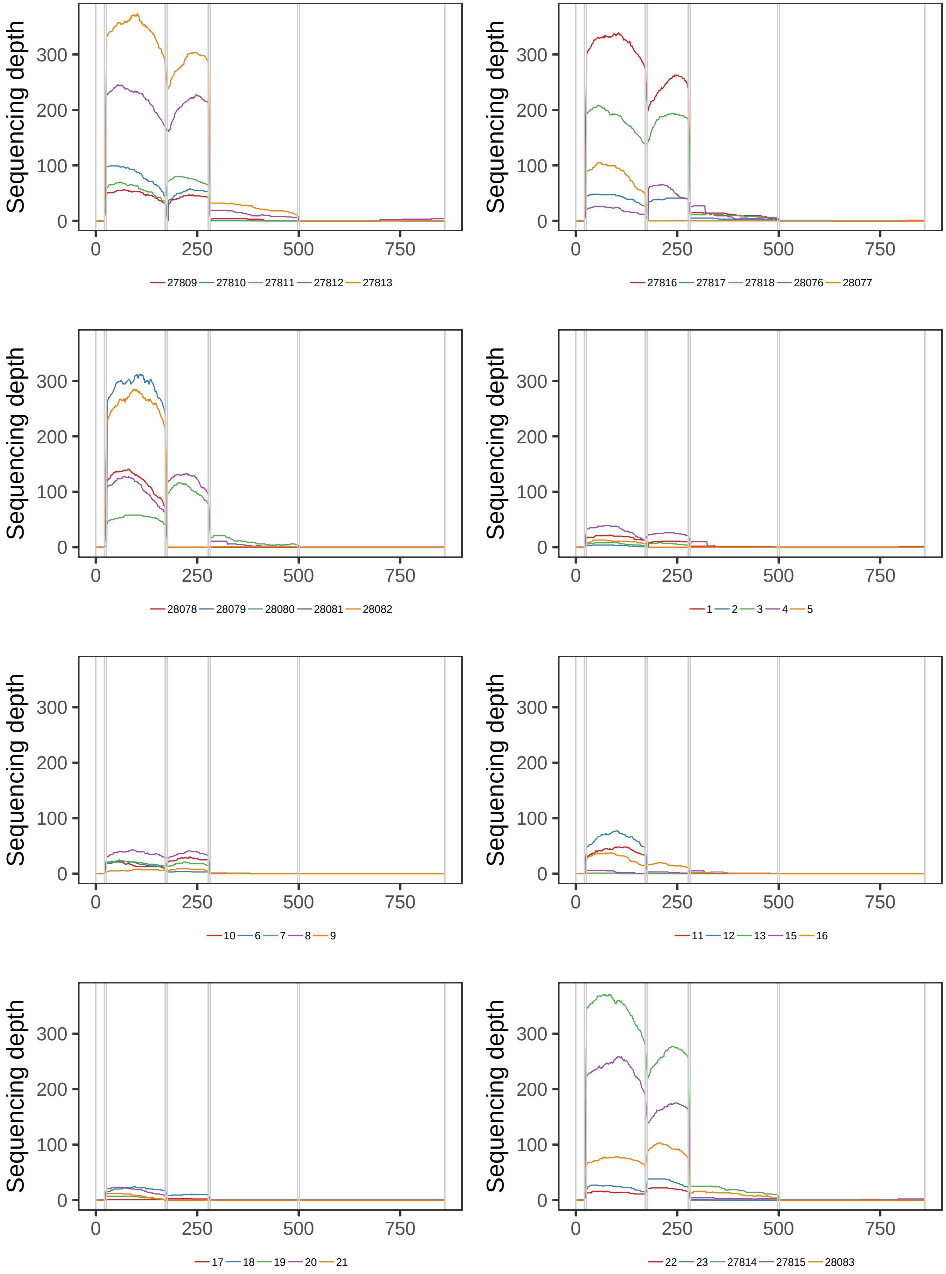
Sequencing depth
0
100
200
300
0
250
500
750
27809
27810
27811
27812
27813
27816
27817
27818
28076
28077
28078
28079
28080
28081
28082
1
2
3
4
5
10
6
7
8
9
11
12
13
15
16
17
18
19
20
21
22
23
27814
27815
28083

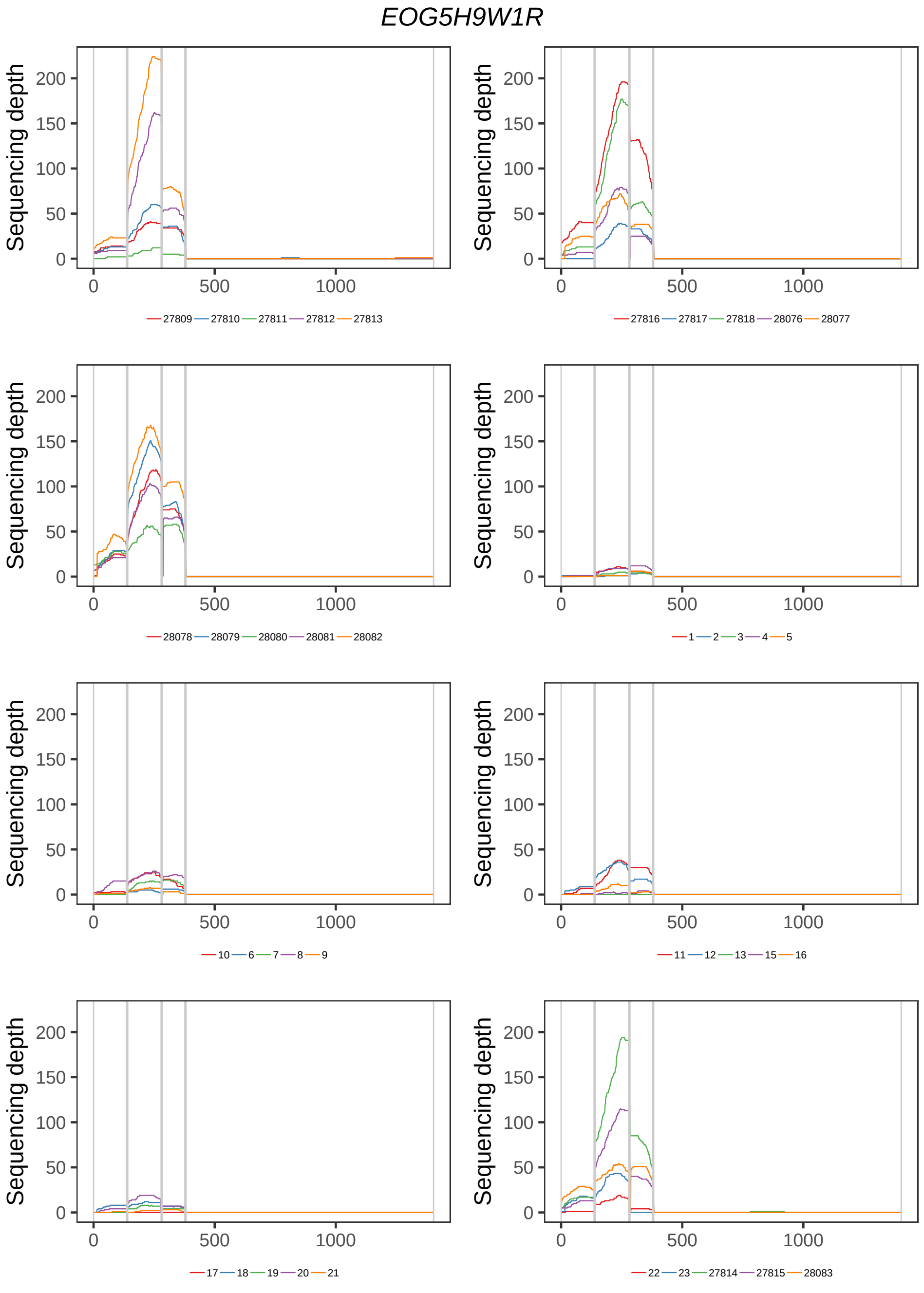
EOG5H9W1R
Sequencing depth
0
50
100
150
200
0
500
1000
27809 27810 27811 27812 27813
27816 27817 27818 28076 28077
28078 28079 28080 28081 28082
1 2 3 4 5
10 6 7 8 9
11 12 13 15 16
17 18 19 20 21
22 23 27814 27815 28083

EOG5HQC1B

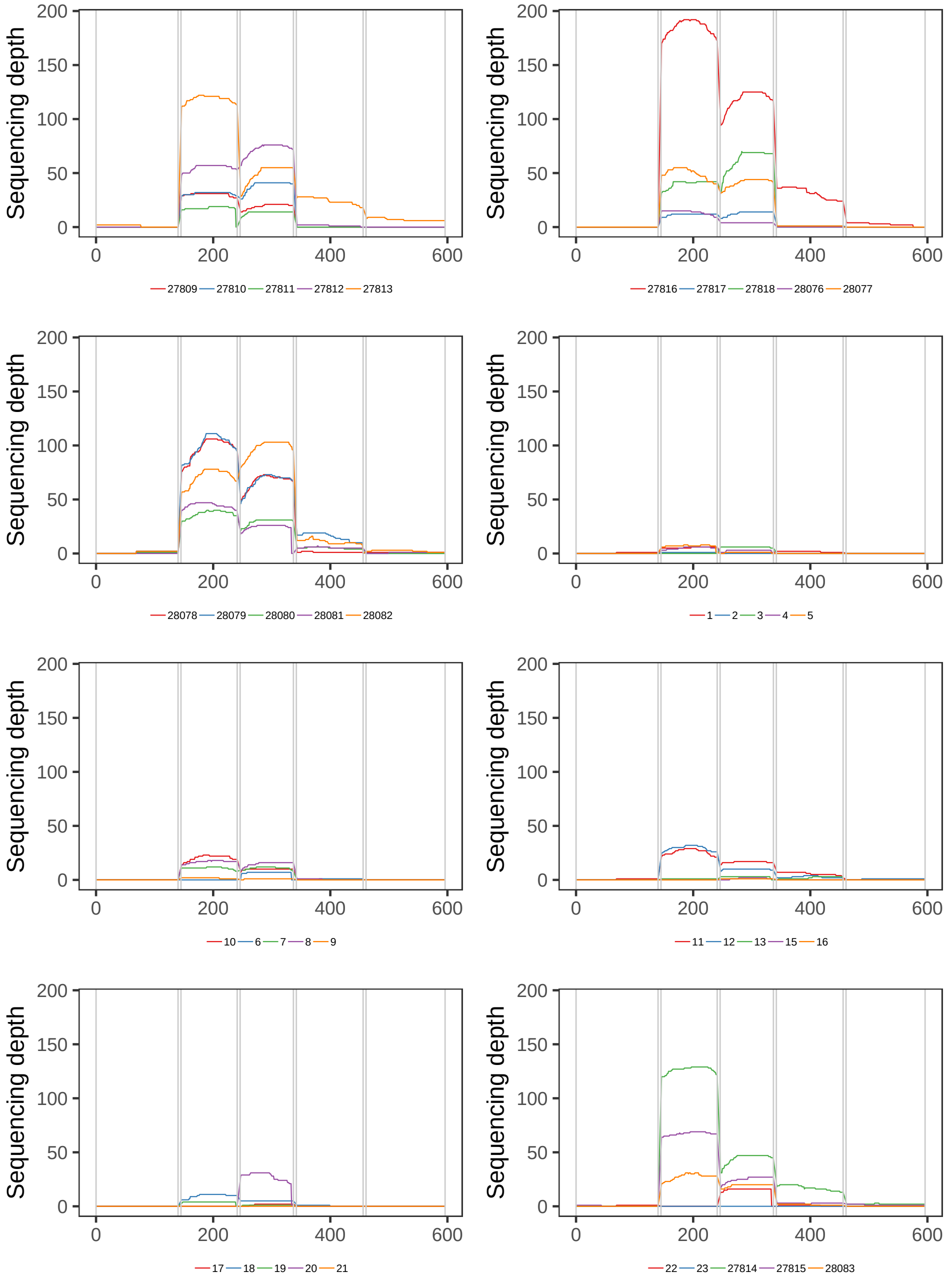
Sequencing depth
27809 27810 27811 27812 27813
27816 27817 27818 28076 28077
28078 28079 28080 28081 28082
1 2 3 4 5
10 6 7 8 9
11 12 13 15 16
17 18 19 20 21
22 23 27814 27815 28083

EOG5J3V0G

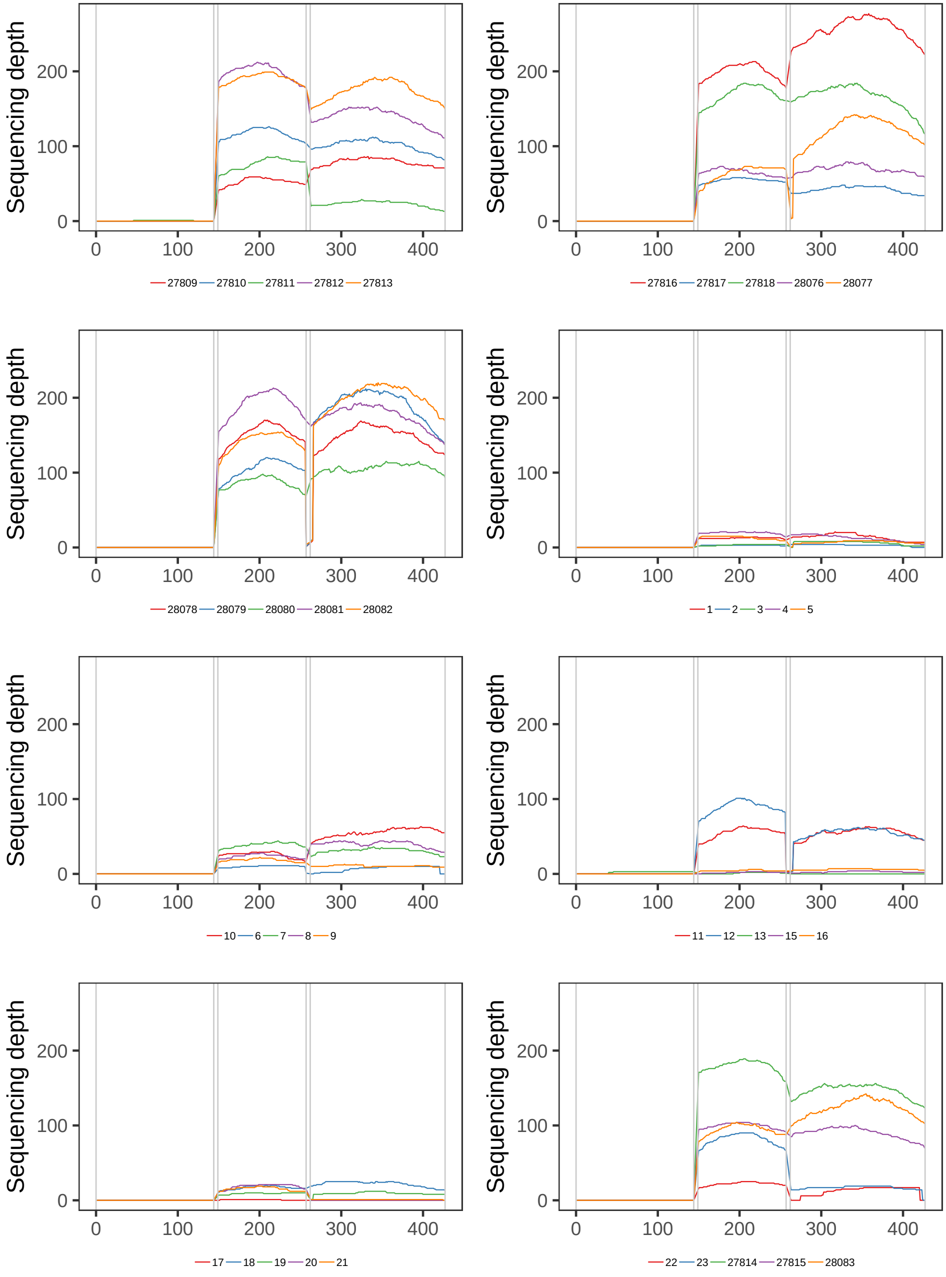
Sequencing depth
27809 27810 27811 27812 27813
27816 27817 27818 28076 28077
28078 28079 28080 28081 28082
1 2 3 4 5
10 6 7 8 9
11 12 13 15 16
17 18 19 20 21
22 23 27814 27815 28083

EOG5N2Z45

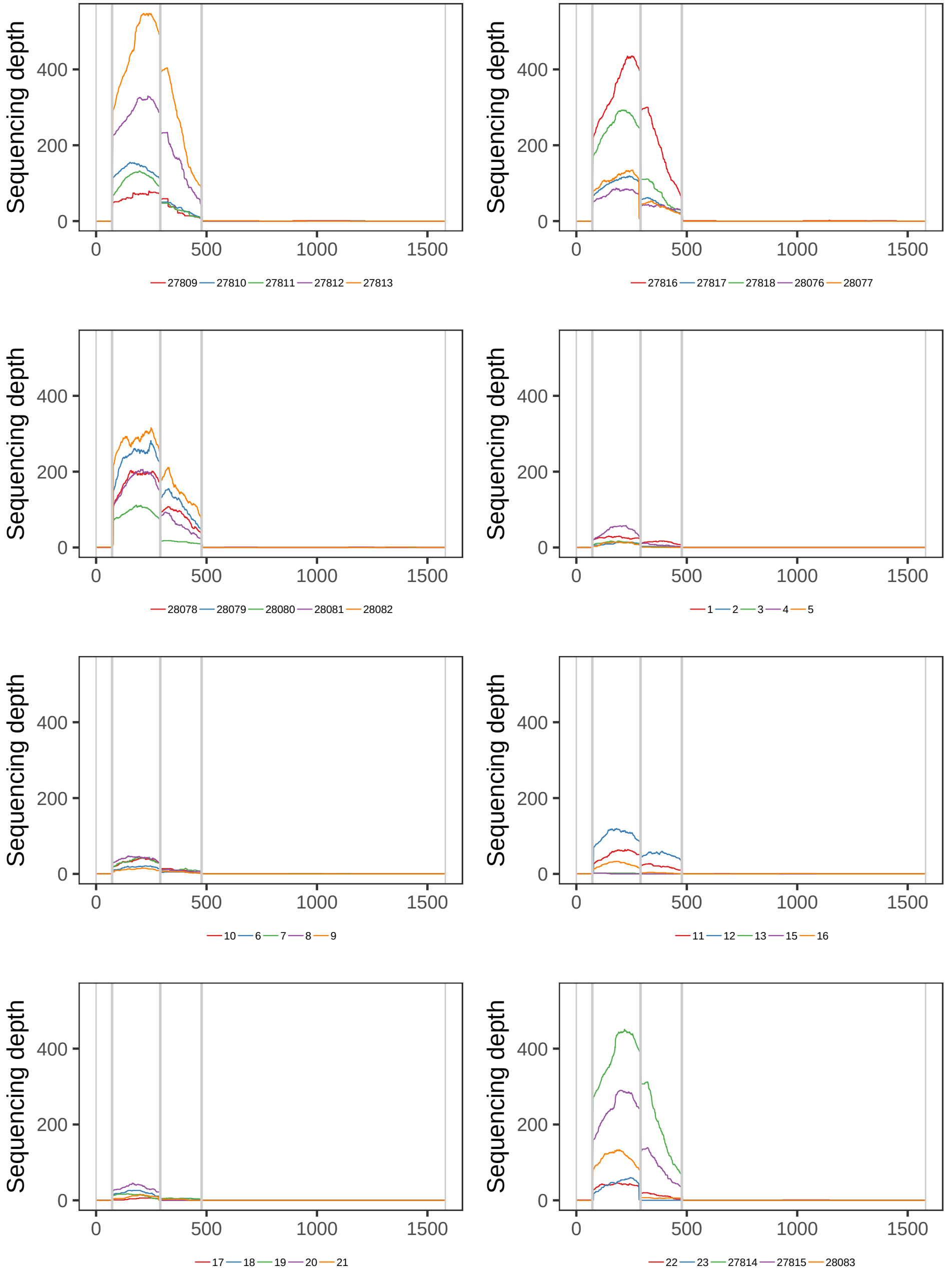

Sequencing depth
0
200
400
0
500
1000
1500
27809
27810
27811
27812
27813
27816
27817
27818
28076
28077
28078
28079
28080
28081
28082
1
2
3
4
5
10
6
7
8
9
11
12
13
15
16
17
18
19
20
21
22
23
27814
27815
28083

EOG5RN8RR

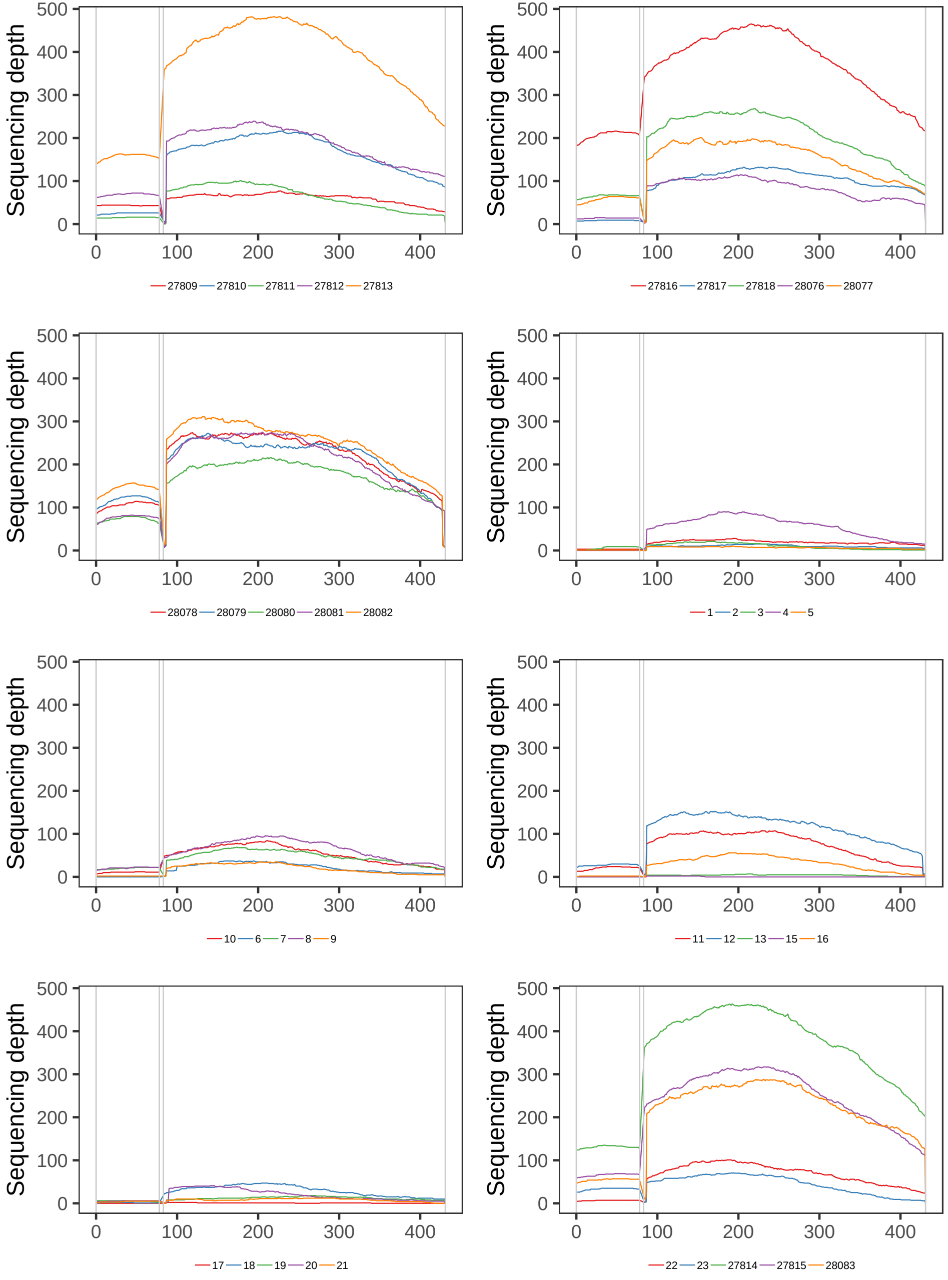
Sequencing depth
0
100
200
300
400
500
27809 27810 27811 27812 27813
27816 27817 27818 28076 28077
28078 28079 28080 28081 28082
1 2 3 4 5
10 6 7 8 9
11 12 13 15 16
17 18 19 20 21
22 23 27814 27815 28083

EOG5X69RV

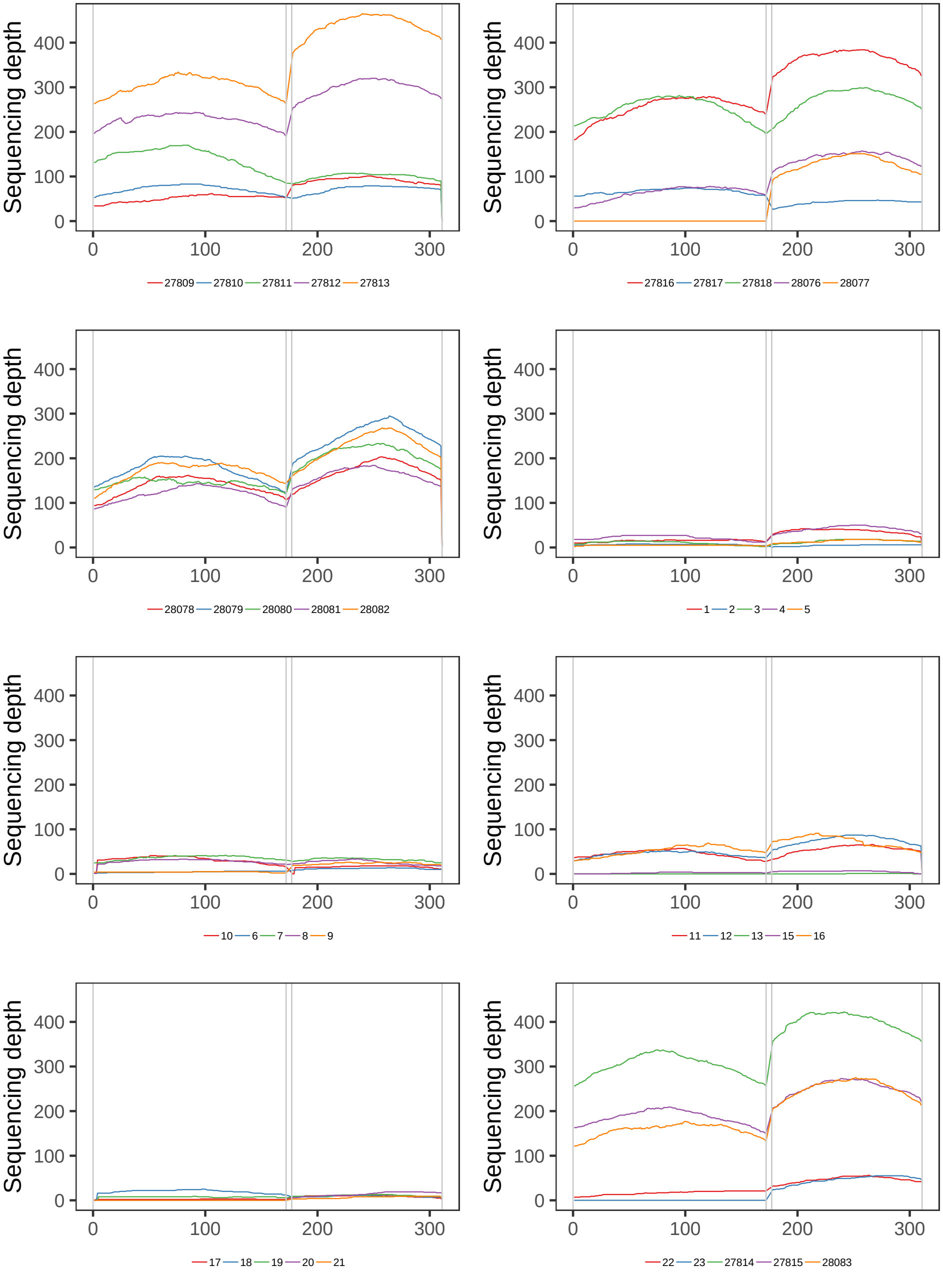
Sequencing depth
0
100
200
300
400
27809 27810 27811 27812 27813
27816 27817 27818 28076 28077
28078 28079 28080 28081 28082
1 2 3 4 5
10 6 7 8 9
11 12 13 15 16
17 18 19 20 21
22 23 27814 27815 28083

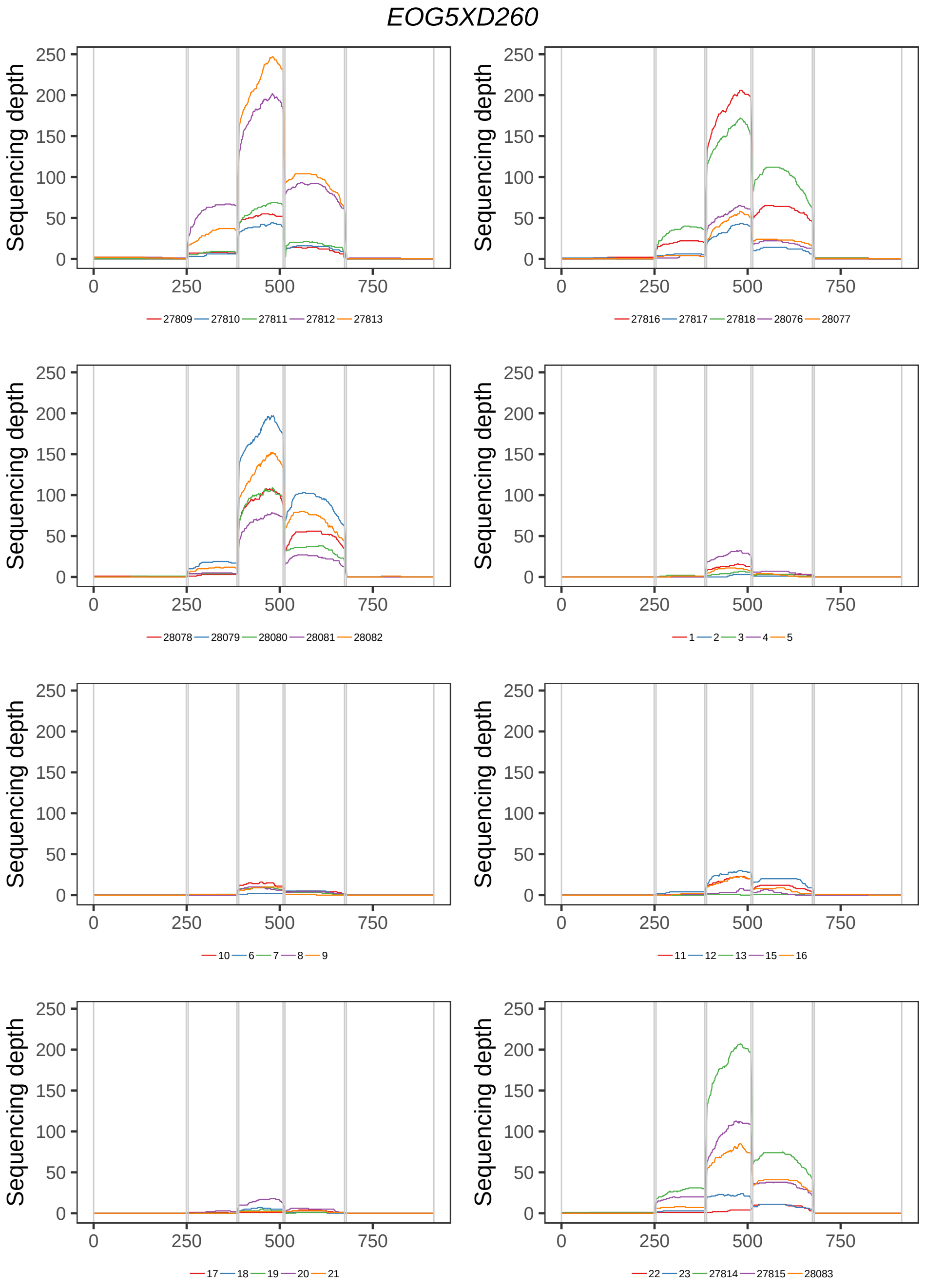
EOG5XD260
Sequencing depth
27809 27810 27811 27812 27813
27816 27817 27818 28076 28077
28078 28079 28080 28081 28082
1 2 3 4 5
10 6 7 8 9
11 12 13 15 16
17 18 19 20 21
22 23 27814 27815 28083

EOG518938

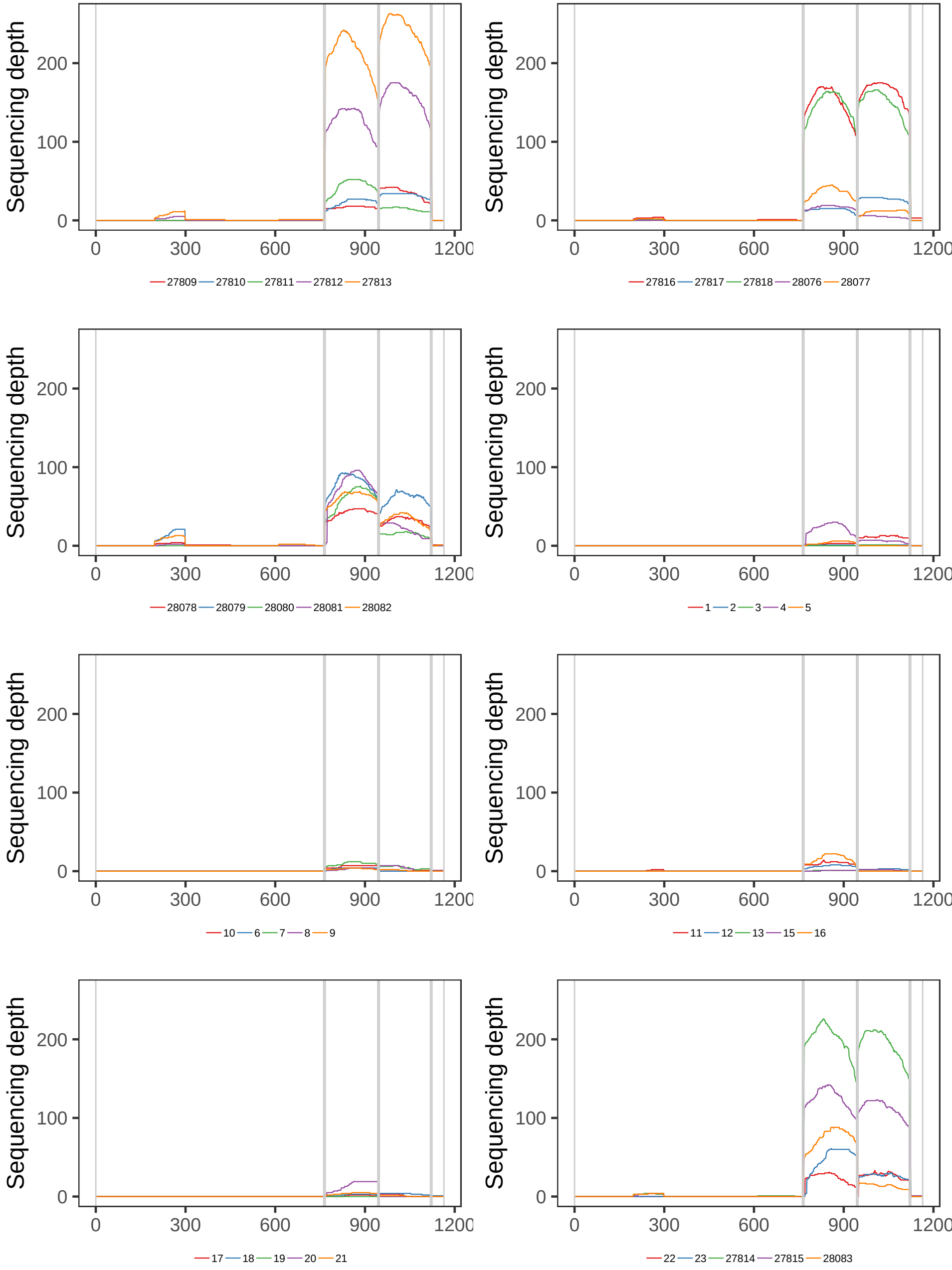
Sequencing depth
0
100
200
0
300
600
900
1200
27809 27810 27811 27812 27813
27816 27817 27818 28076 28077
28078 28079 28080 28081 28082
1 2 3 4 5
10 6 7 8 9
11 12 13 15 16
17 18 19 20 21
22 23 27814 27815 28083

EOG51G1M2

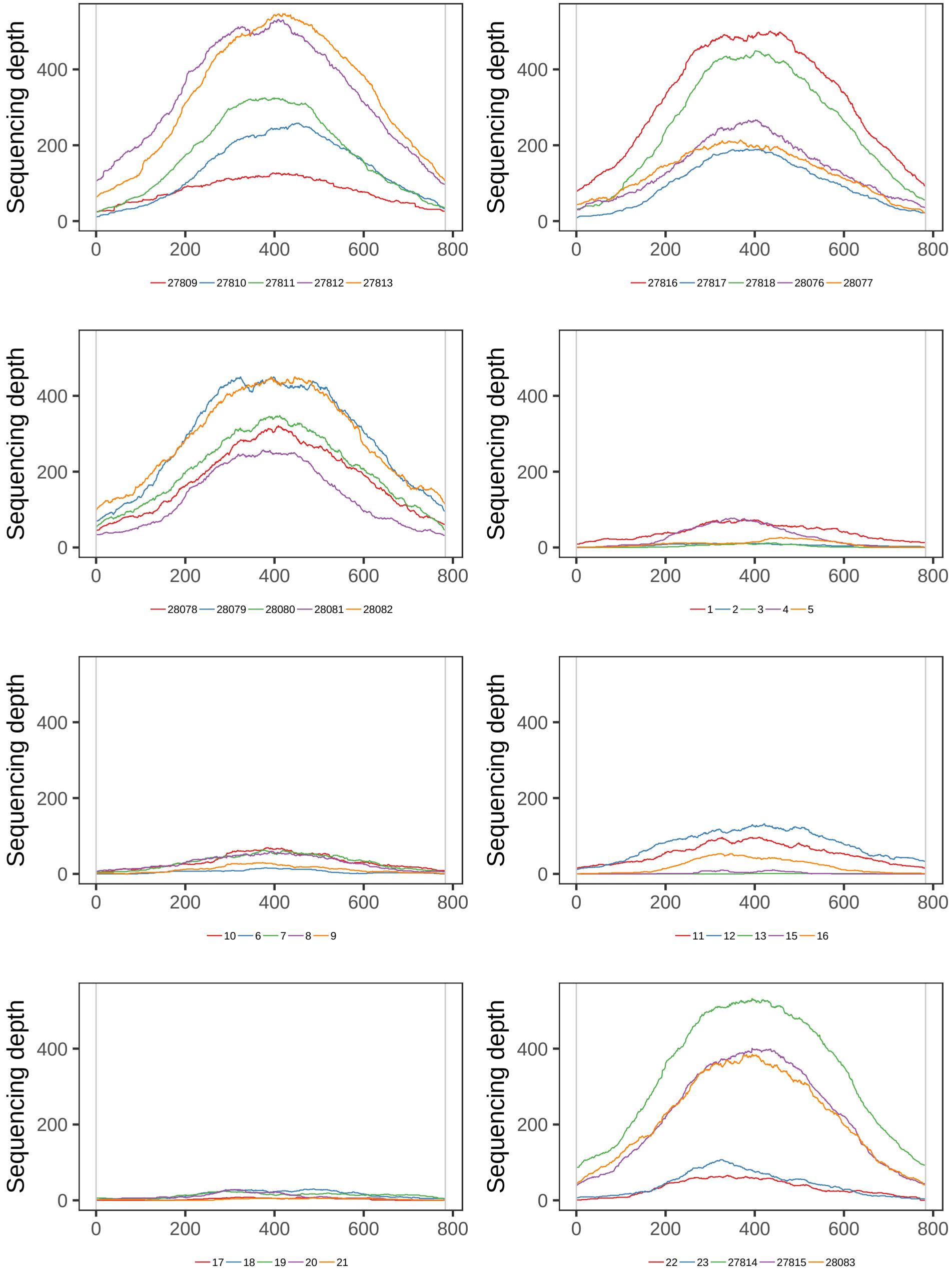
Sequencing depth
0
200
400
600
800
27809 27810 27811 27812 27813
27816 27817 27818 28076 28077
28078 28079 28080 28081 28082
1 2 3 4 5
10 6 7 8 9
11 12 13 15 16
17 18 19 20 21
22 23 27814 27815 28083

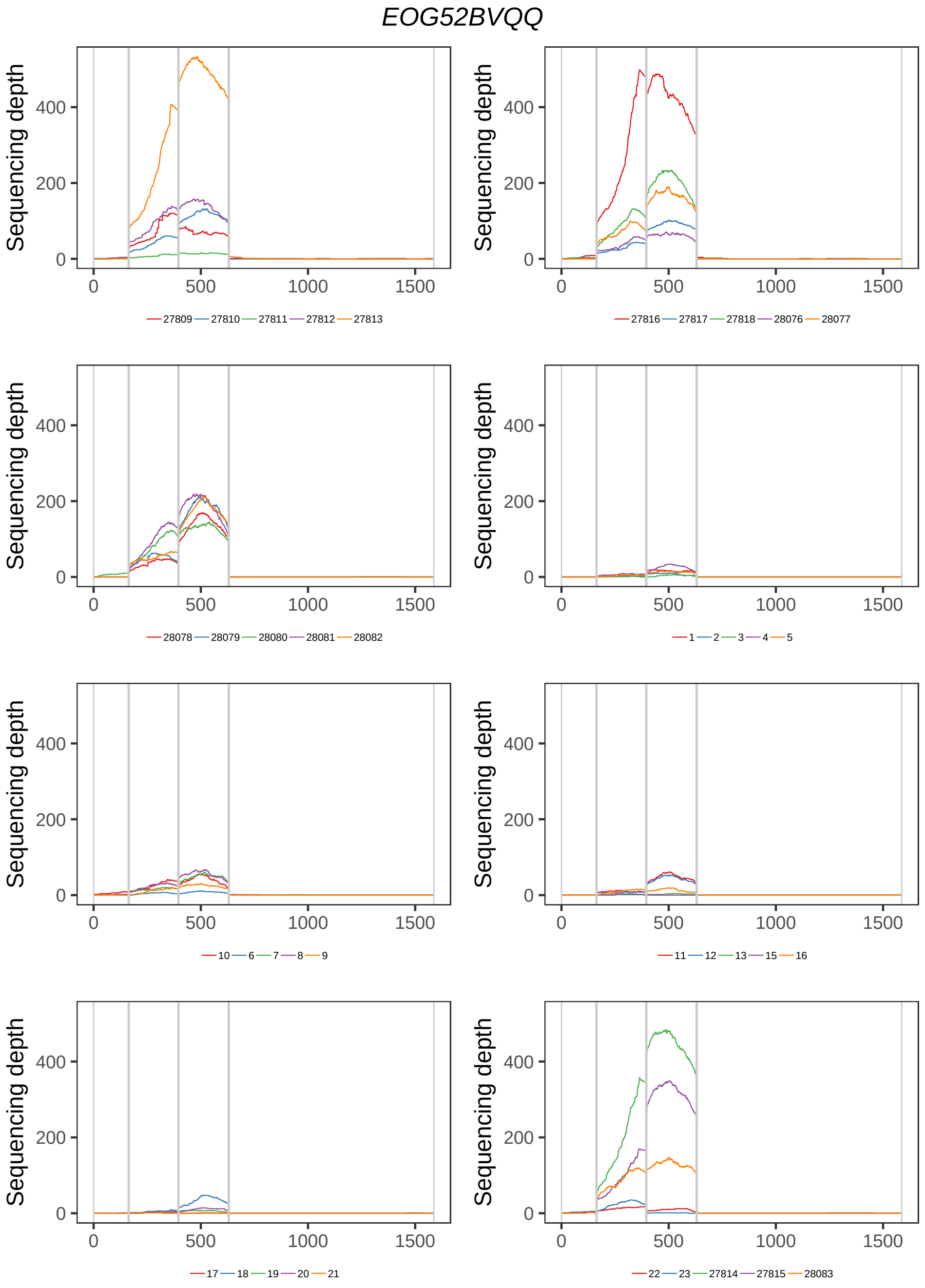

EOG52BVQQ
Sequencing depth
0
200
400
0
500
1000
1500
27809 27810 27811 27812 27813
27816 27817 27818 28076 28077
28078 28079 28080 28081 28082
1 2 3 4 5
10 6 7 8 9
11 12 13 15 16
17 18 19 20 21
22 23 27814 27815 28083

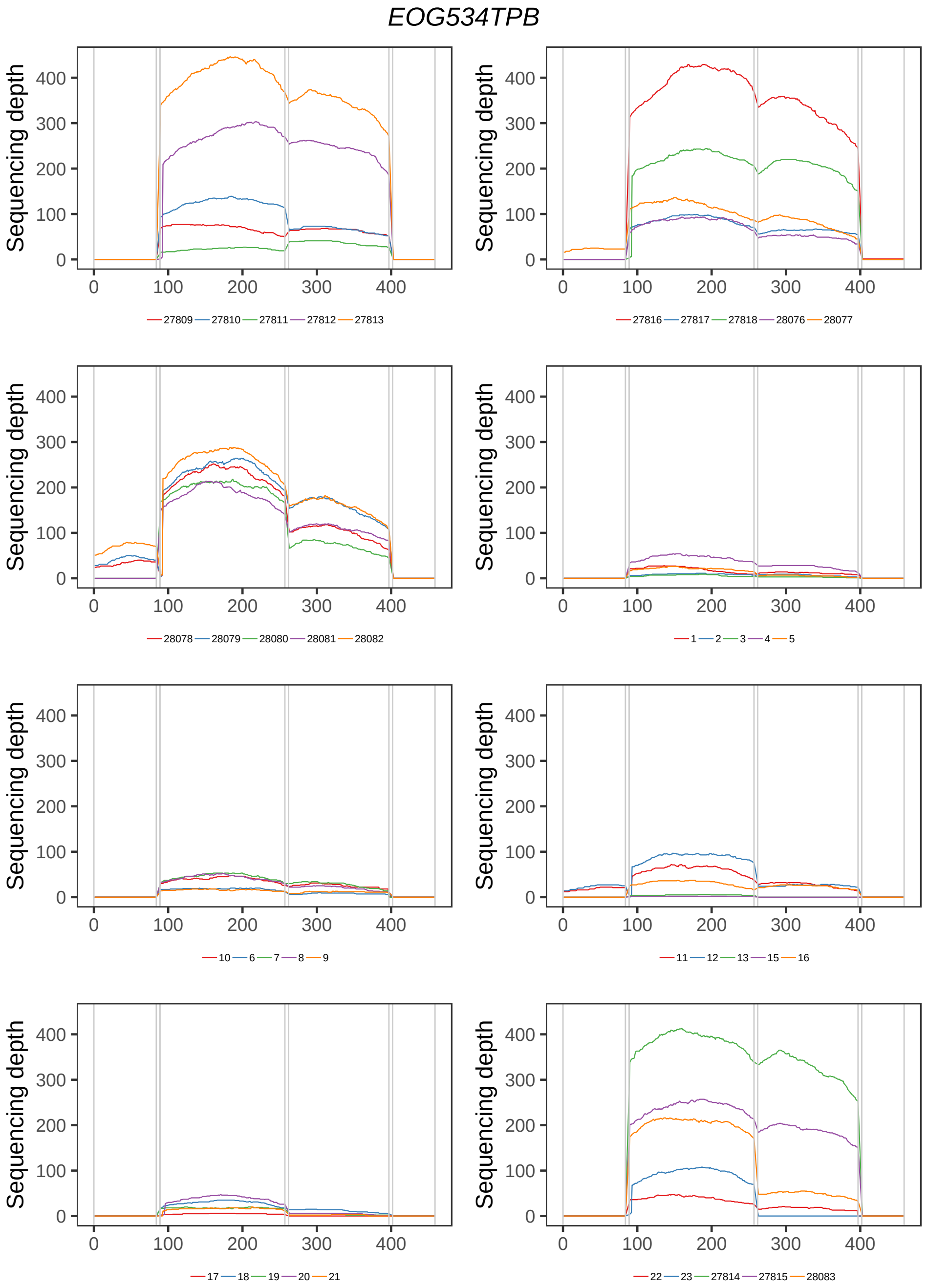
EOG534TPB
Sequencing depth
0
100
200
300
400
27809
27810
27811
27812
27813
27816
27817
27818
28076
28077
28078
28079
28080
28081
28082
1
2
3
4
5
10
6
7
8
9
11
12
13
15
16
17
18
19
20
21
22
23
27814
27815
28083

EOG54F4S9

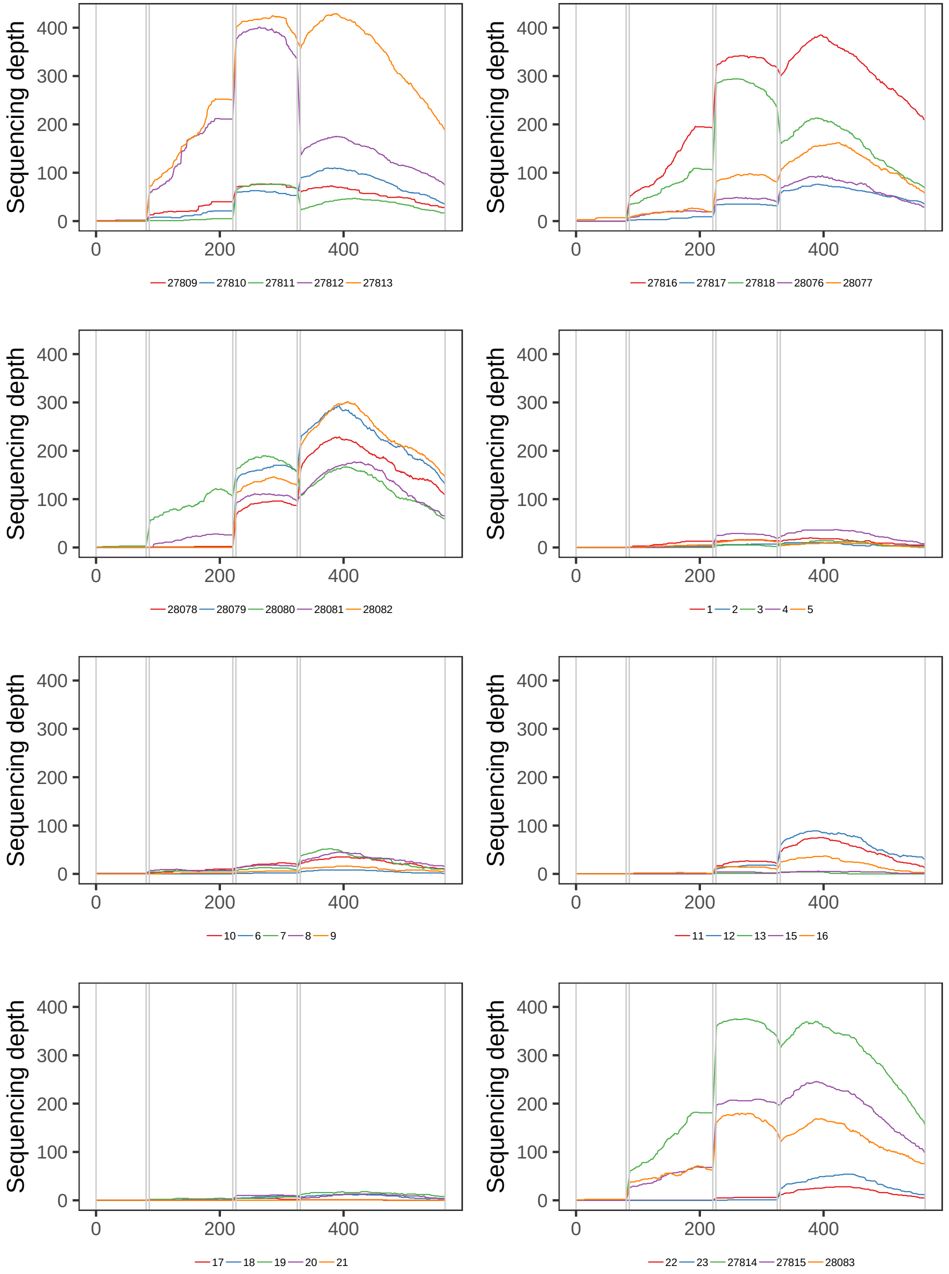
Sequencing depth
0
100
200
300
400
0
200
400
27809 27810 27811 27812 27813
27816 27817 27818 28076 28077
28078 28079 28080 28081 28082
1 2 3 4 5
10 6 7 8 9
11 12 13 15 16
17 18 19 20 21
22 23 27814 27815 28083

EOG54MW8B

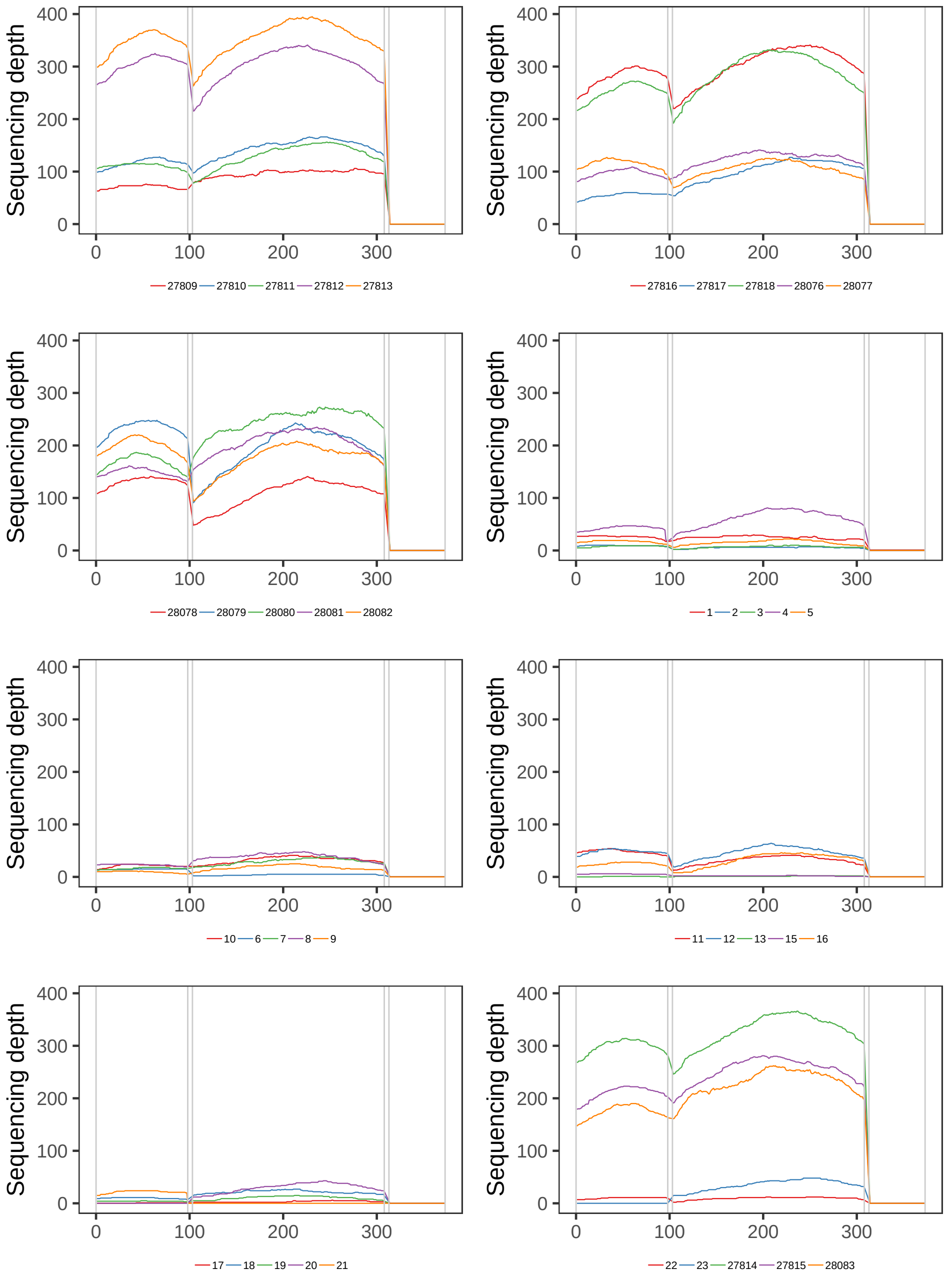
Sequencing depth
0
100
200
300
400
27809 27810 27811 27812 27813
27816 27817 27818 28076 28077
28078 28079 28080 28081 28082
1 2 3 4 5
10 6 7 8 9
11 12 13 15 16
17 18 19 20 21
22 23 27814 27815 28083

EOG54XGZ9

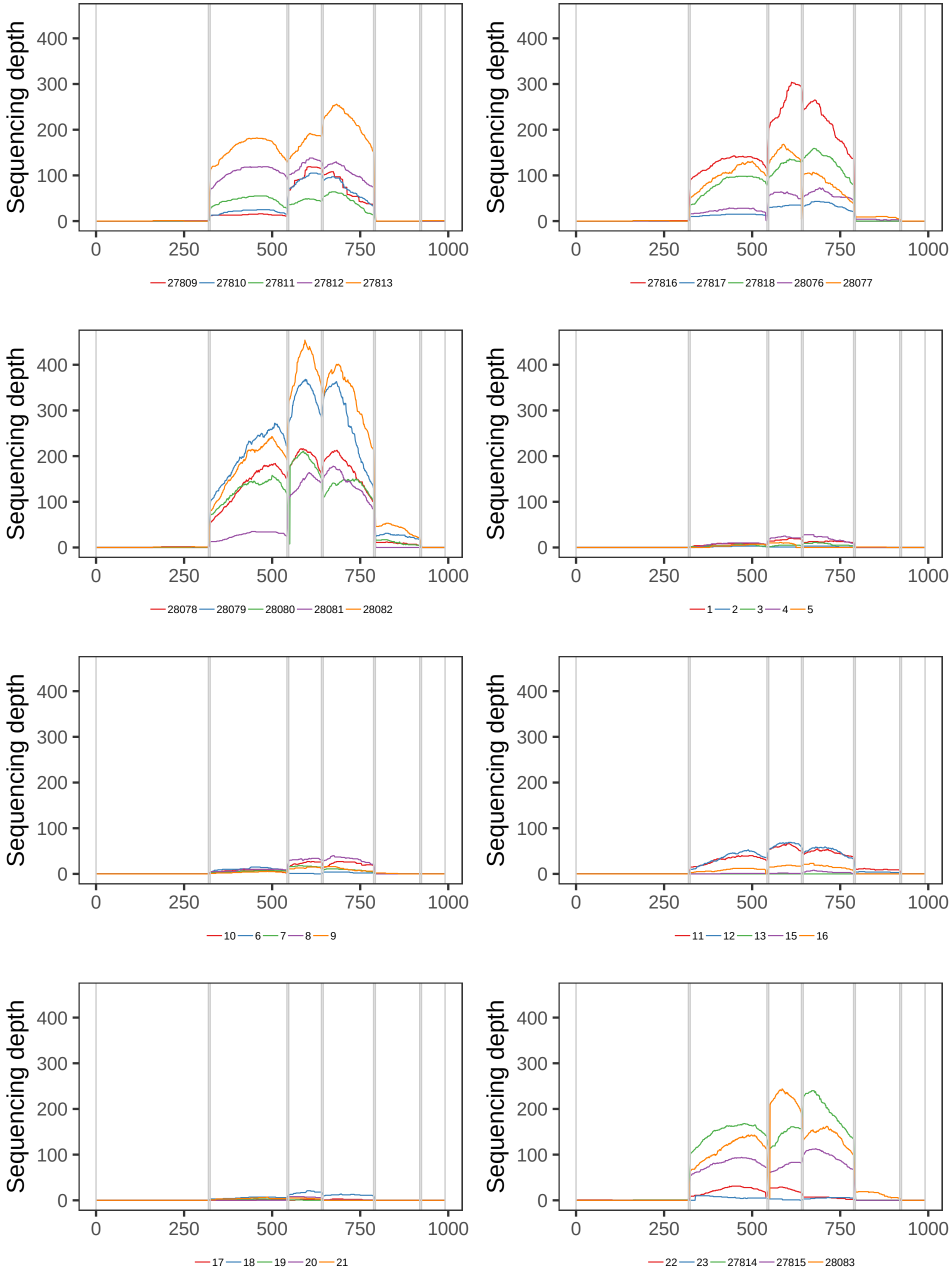

Sequencing depth
0
100
200
300
400
0
250
500
750
1000
27809 27810 27811 27812 27813
27816 27817 27818 28076 28077
28078 28079 28080 28081 28082
1 2 3 4 5
10 6 7 8 9
11 12 13 15 16
17 18 19 20 21
22 23 27814 27815 28083

EOG55HQCT

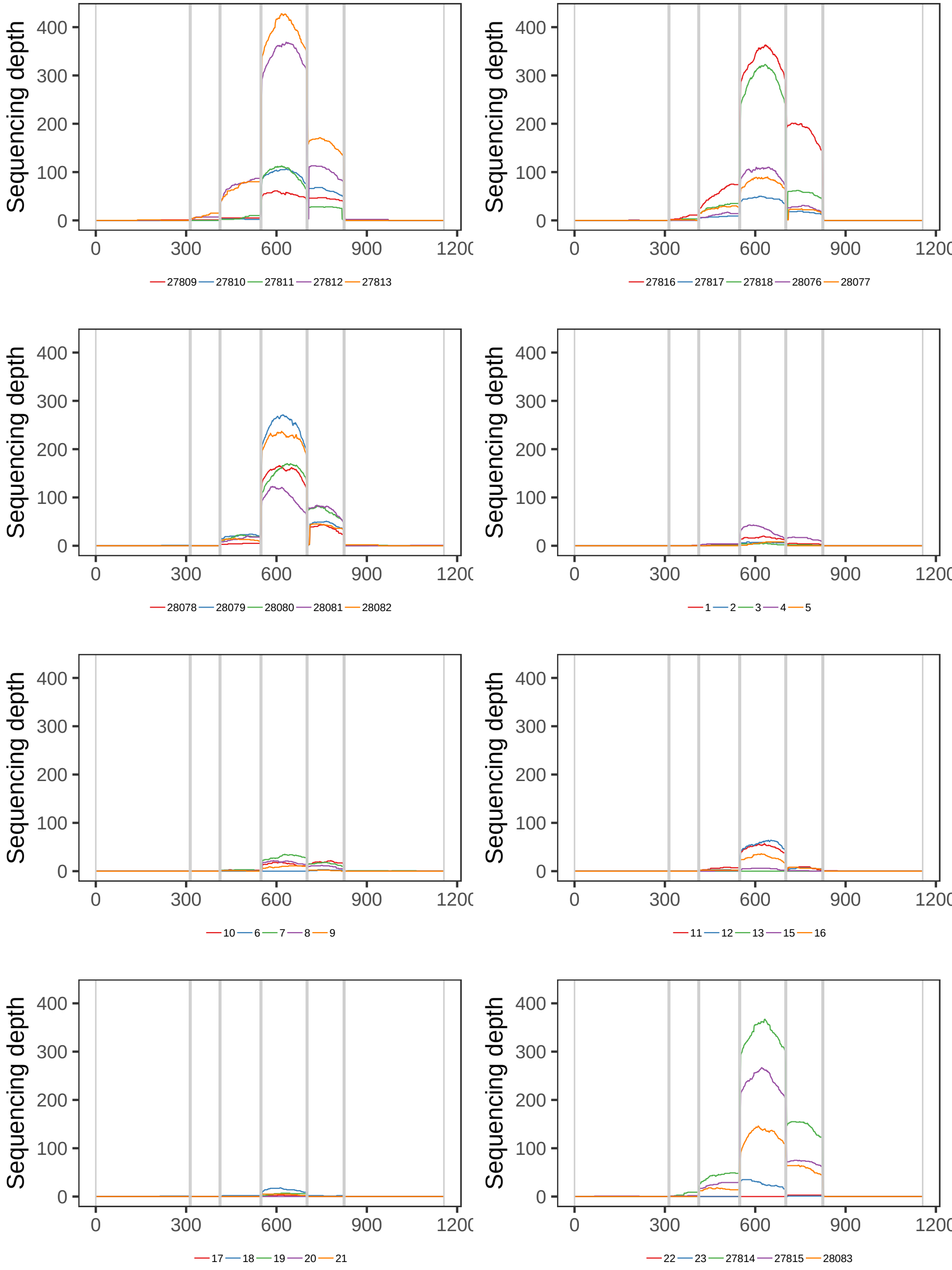
Sequencing depth
0
100
200
300
400
0
300
600
900
1200
27809 27810 27811 27812 27813
27816 27817 27818 28076 28077
28078 28079 28080 28081 28082
1 2 3 4 5
10 6 7 8 9
11 12 13 15 16
17 18 19 20 21
22 23 27814 27815 28083

EOG56M91V

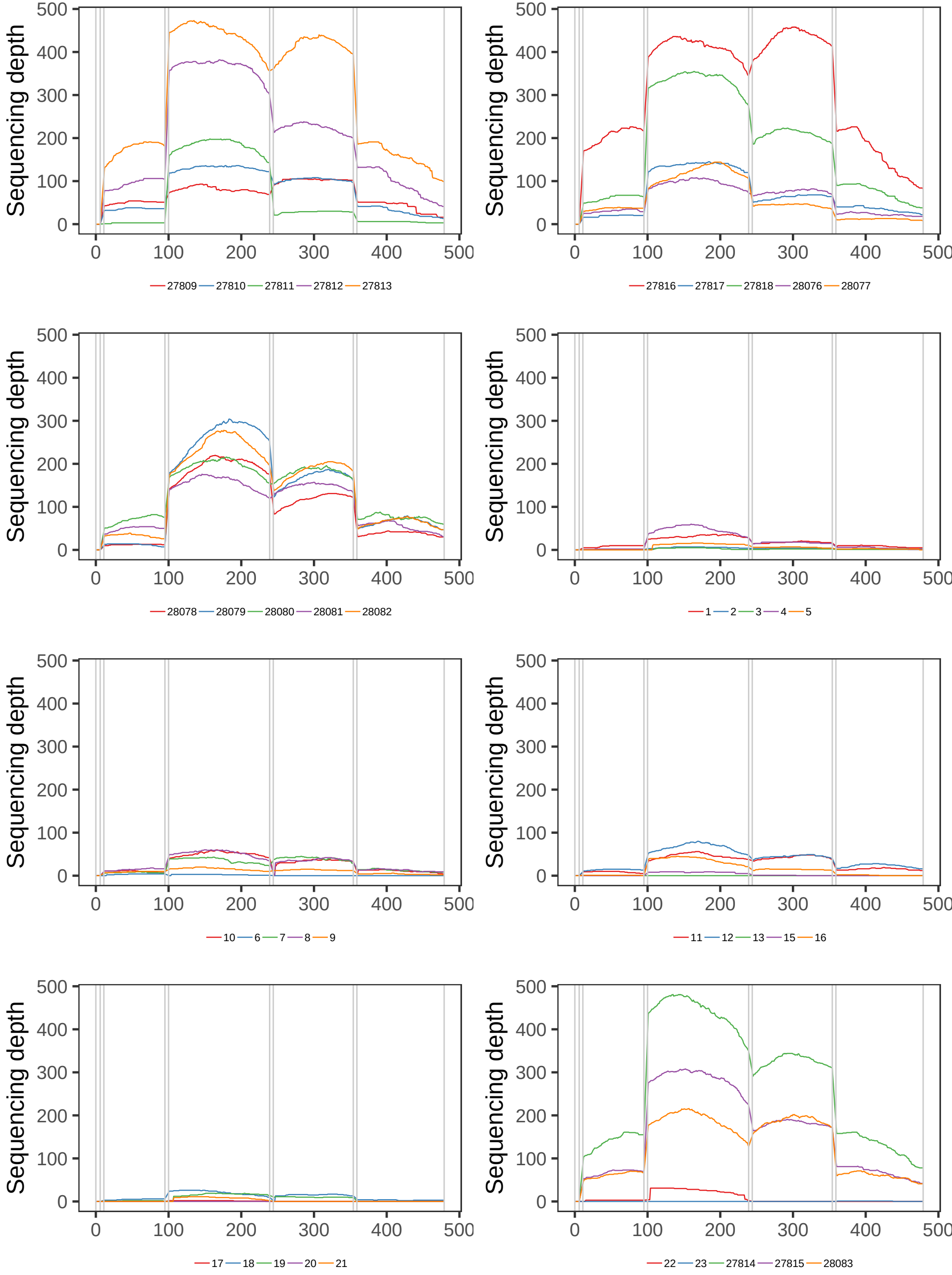
Sequencing depth
0
100
200
300
400
500
27809 27810 27811 27812 27813
27816 27817 27818 28076 28077
28078 28079 28080 28081 28082
1 2 3 4 5
10 6 7 8 9
11 12 13 15 16
17 18 19 20 21
22 23 27814 27815 28083

EOG589334

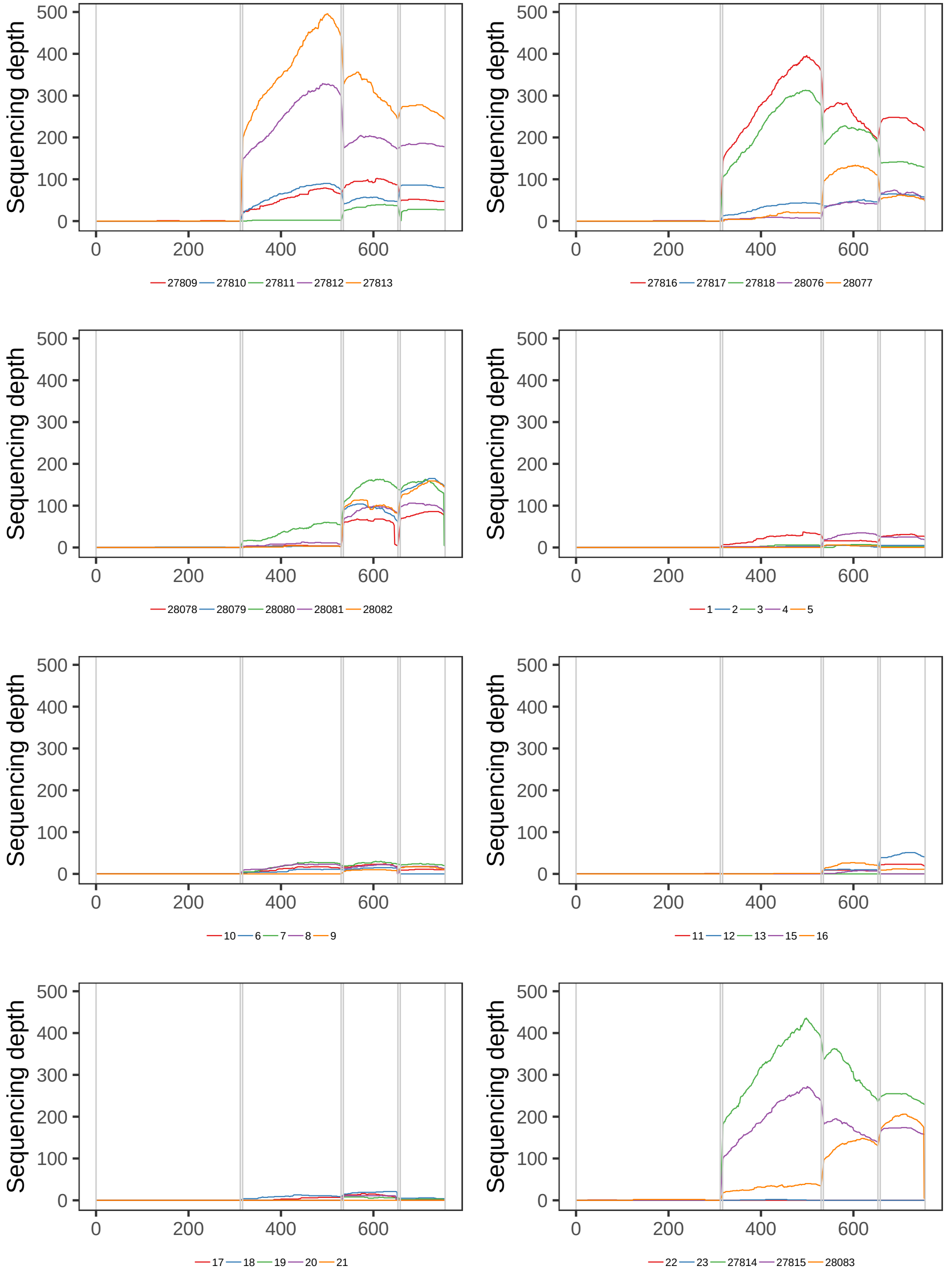

Sequencing depth
500
400
300
200
100
0
0
200
400
600
27809
27810
27811
27812
27813
27816
27817
27818
28076
28077
28078
28079
28080
28081
28082
1
2
3
4
5
10
6
7
8
9
11
12
13
15
16
17
18
19
20
21
22
23
27814
27815
28083

EOG58PK1K

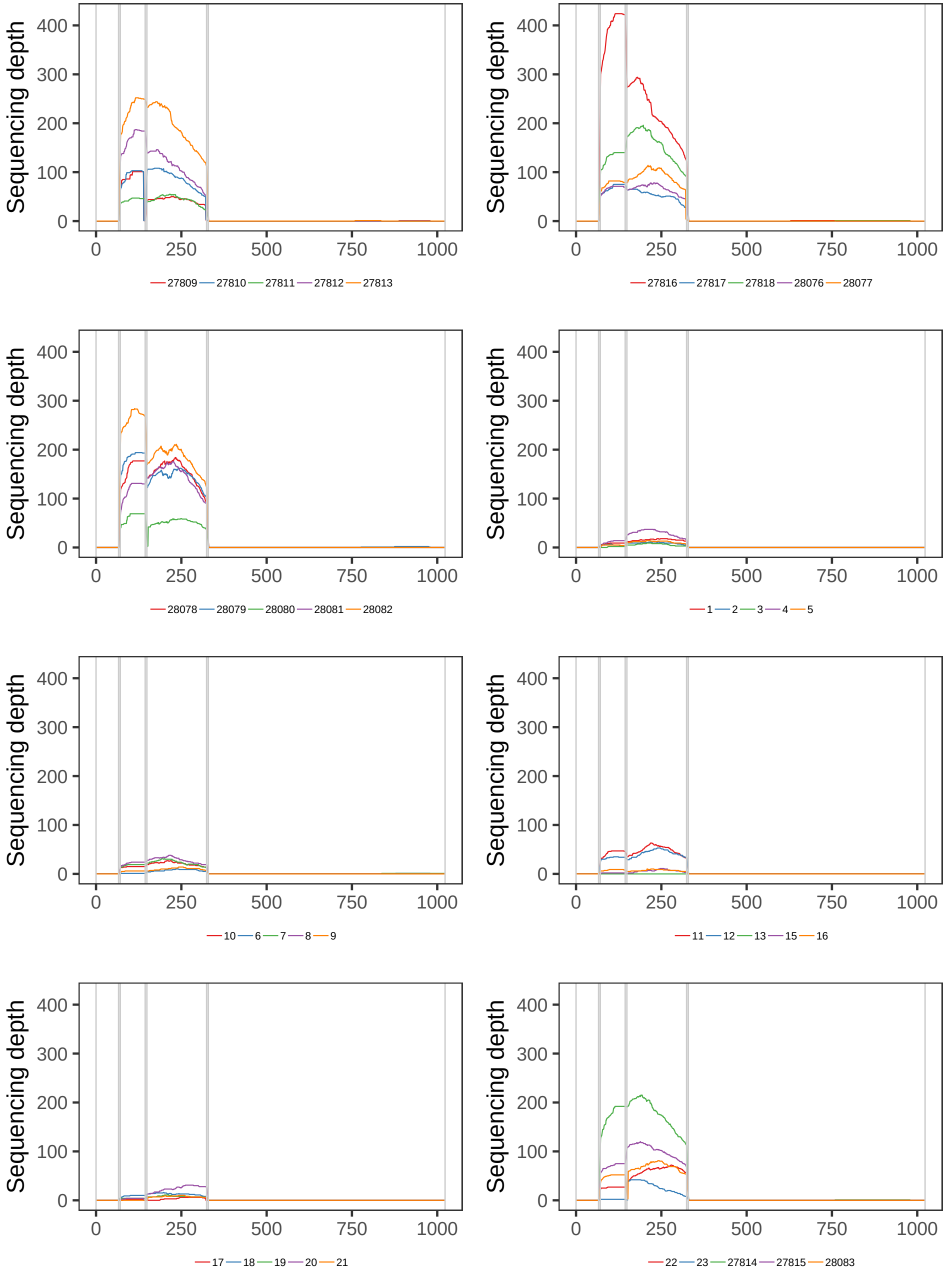

Sequencing depth
0
100
200
300
400
0
250
500
750
1000
27809 27810 27811 27812 27813
27816 27817 27818 28076 28077
28078 28079 28080 28081 28082
1 2 3 4 5
10 6 7 8 9
11 12 13 15 16
17 18 19 20 21
22 23 27814 27815 28083

EOG58PK21

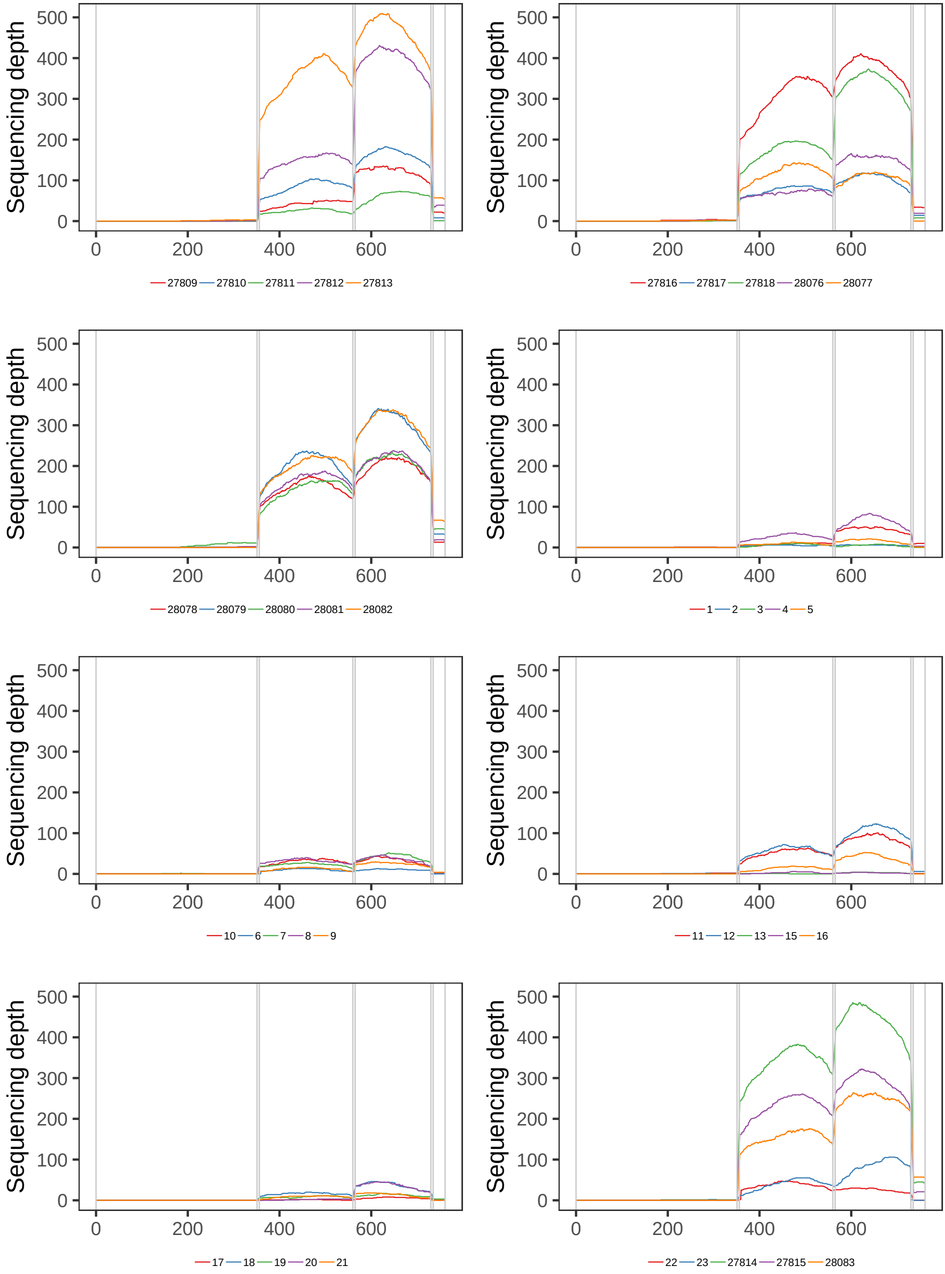

Sequencing depth
0
100
200
300
400
500
0
200
400
600
27809 27810 27811 27812 27813
27816 27817 27818 28076 28077
28078 28079 28080 28081 28082
1 2 3 4 5
10 6 7 8 9
11 12 13 15 16
17 18 19 20 21
22 23 27814 27815 28083

EOG5905RS

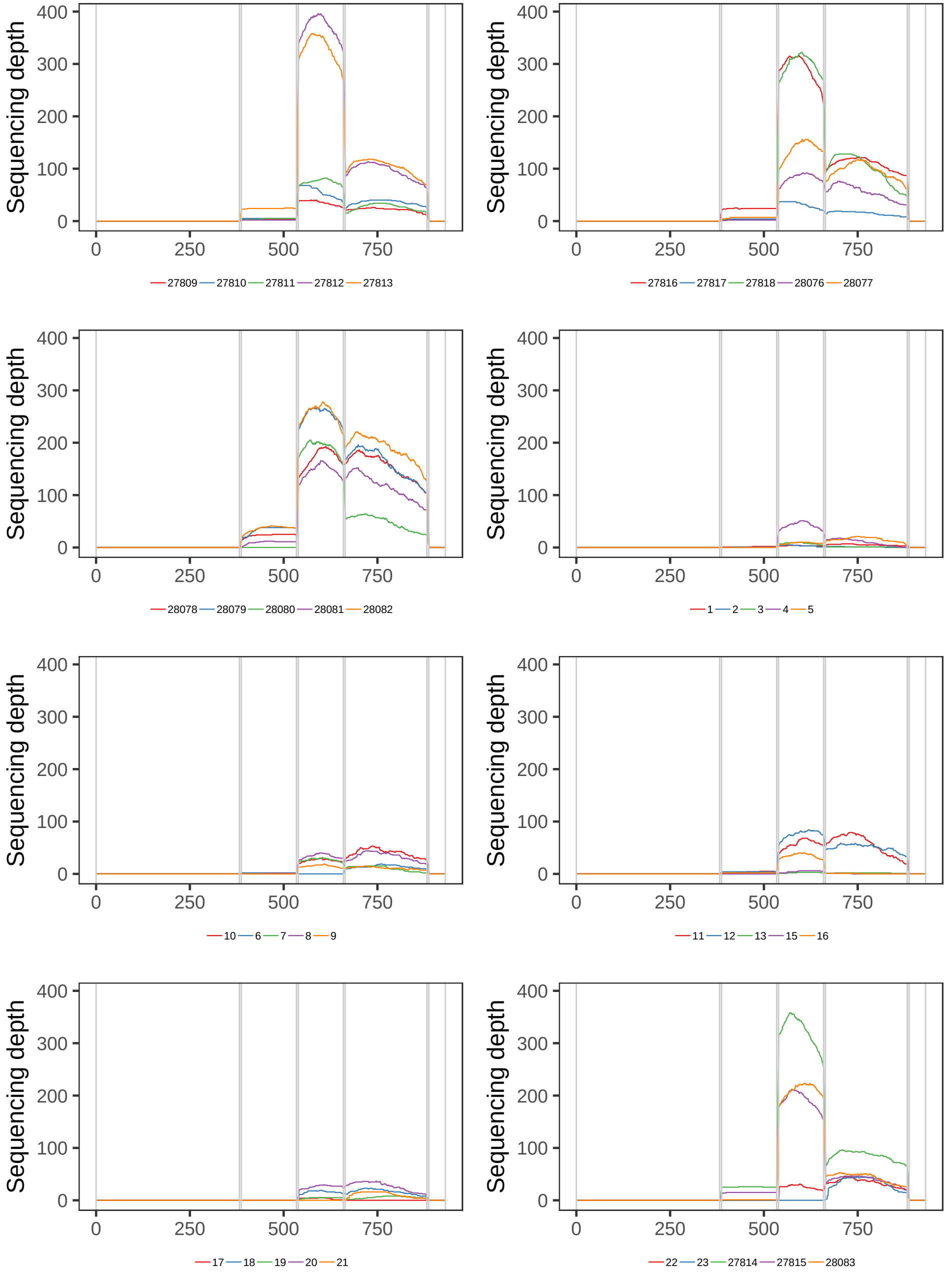
Sequencing depth
0
100
200
300
400
0
250
500
750
27809
27810
27811
27812
27813
27816
27817
27818
28076
28077
28078
28079
28080
28081
28082
1
2
3
4
5
10
6
7
8
9
11
12
13
15
16
17
18
19
20
21
22
23
27814
27815
28083

EOG5CC2GQ

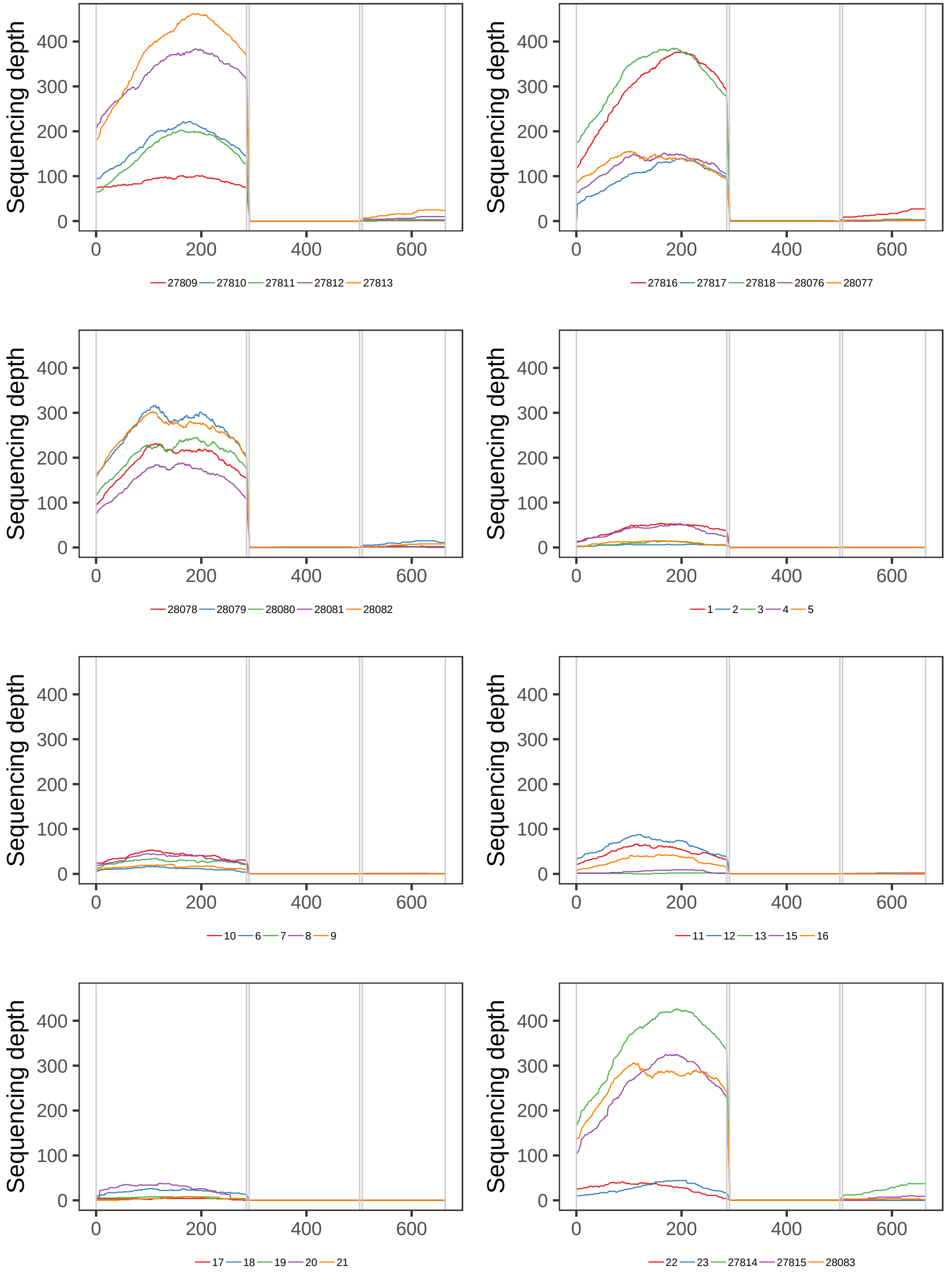
Sequencing depth
0
100
200
300
400
0
200
400
600
27809 27810 27811 27812 27813
27816 27817 27818 28076 28077
28078 28079 28080 28081 28082
1 2 3 4 5
10 6 7 8 9
11 12 13 15 16
17 18 19 20 21
22 23 27814 27815 28083

EOG5CJT0N

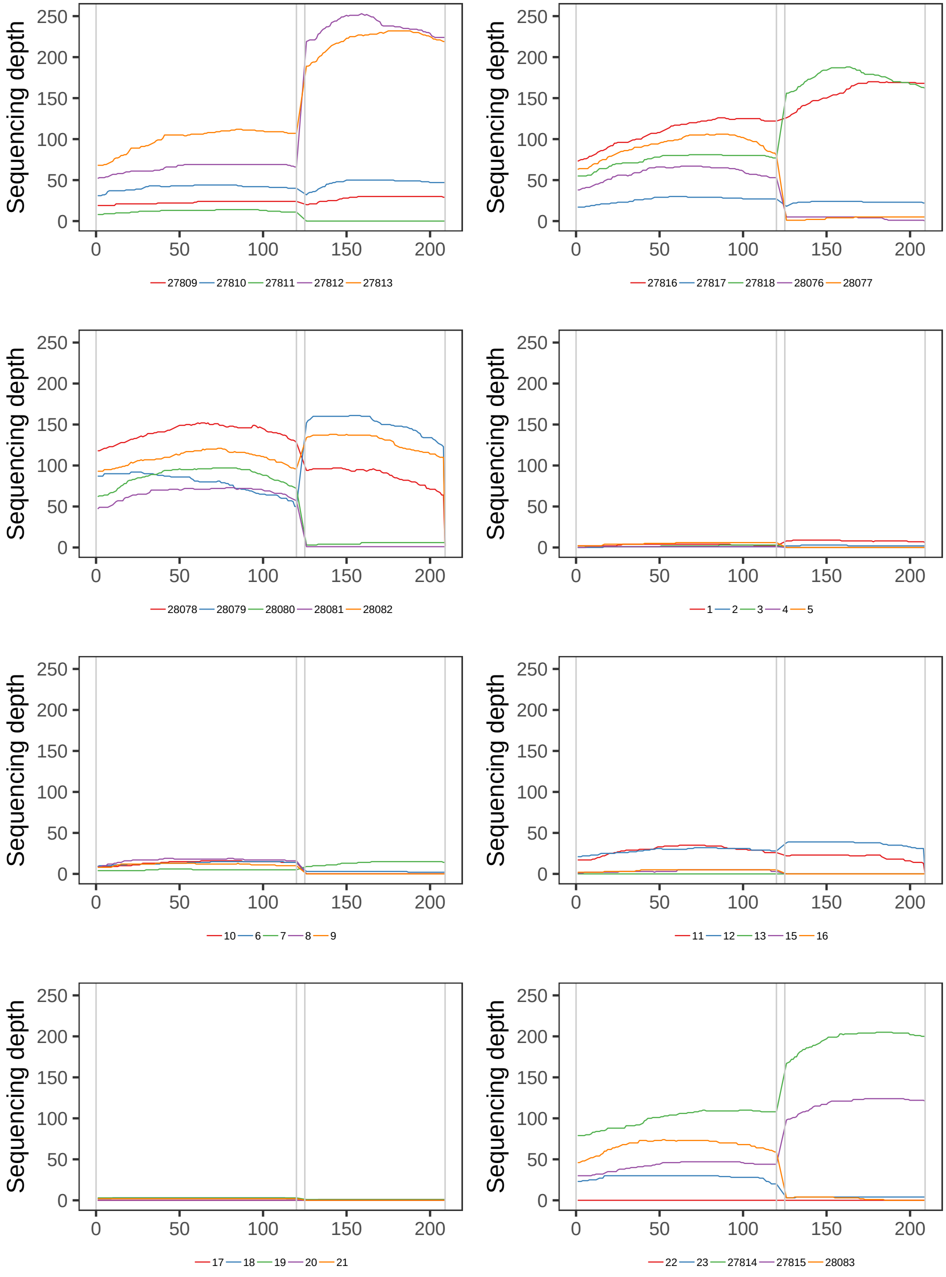
Sequencing depth
27809 27810 27811 27812 27813
27816 27817 27818 28076 28077
28078 28079 28080 28081 28082
1 2 3 4 5
10 6 7 8 9
11 12 13 15 16
17 18 19 20 21
22 23 27814 27815 28083

EOG5H70TQ

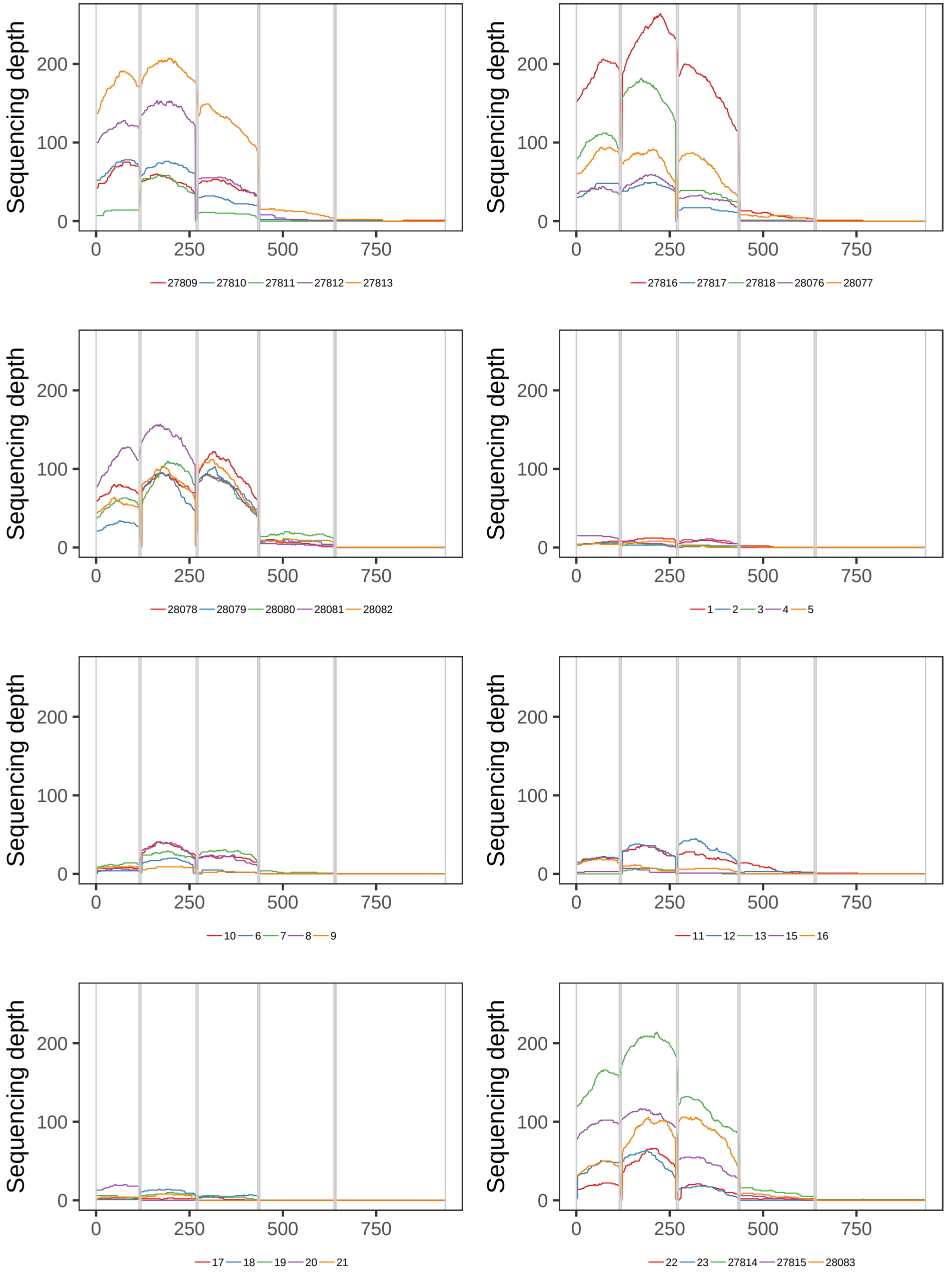

Sequencing depth
200
100
0
0
250
500
750
27809
27810
27811
27812
27813
27816
27817
27818
28076
28077
28078
28079
28080
28081
28082
1
2
3
4
5
10
6
7
8
9
11
12
13
15
16
17
18
19
20
21
22
23
27814
27815
28083

EOG5HT78P

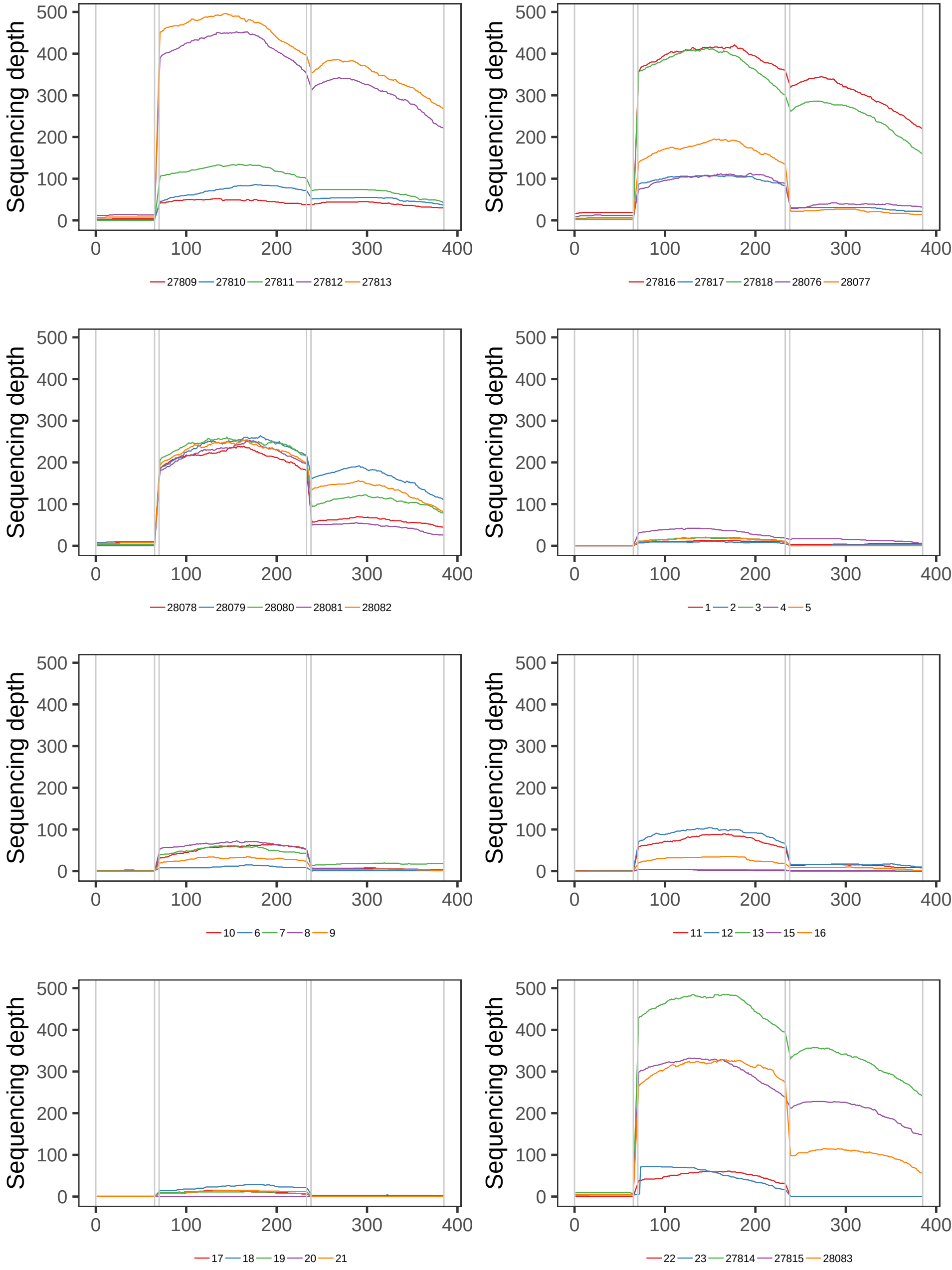
Sequencing depth
0
100
200
300
400
500
27809
27810
27811
27812
27813
27816
27817
27818
28076
28077
28078
28079
28080
28081
28082
1
2
3
4
5
10
6
7
8
9
11
12
13
15
16
17
18
19
20
21
22
23
27814
27815
28083

EOG5J6Q6D

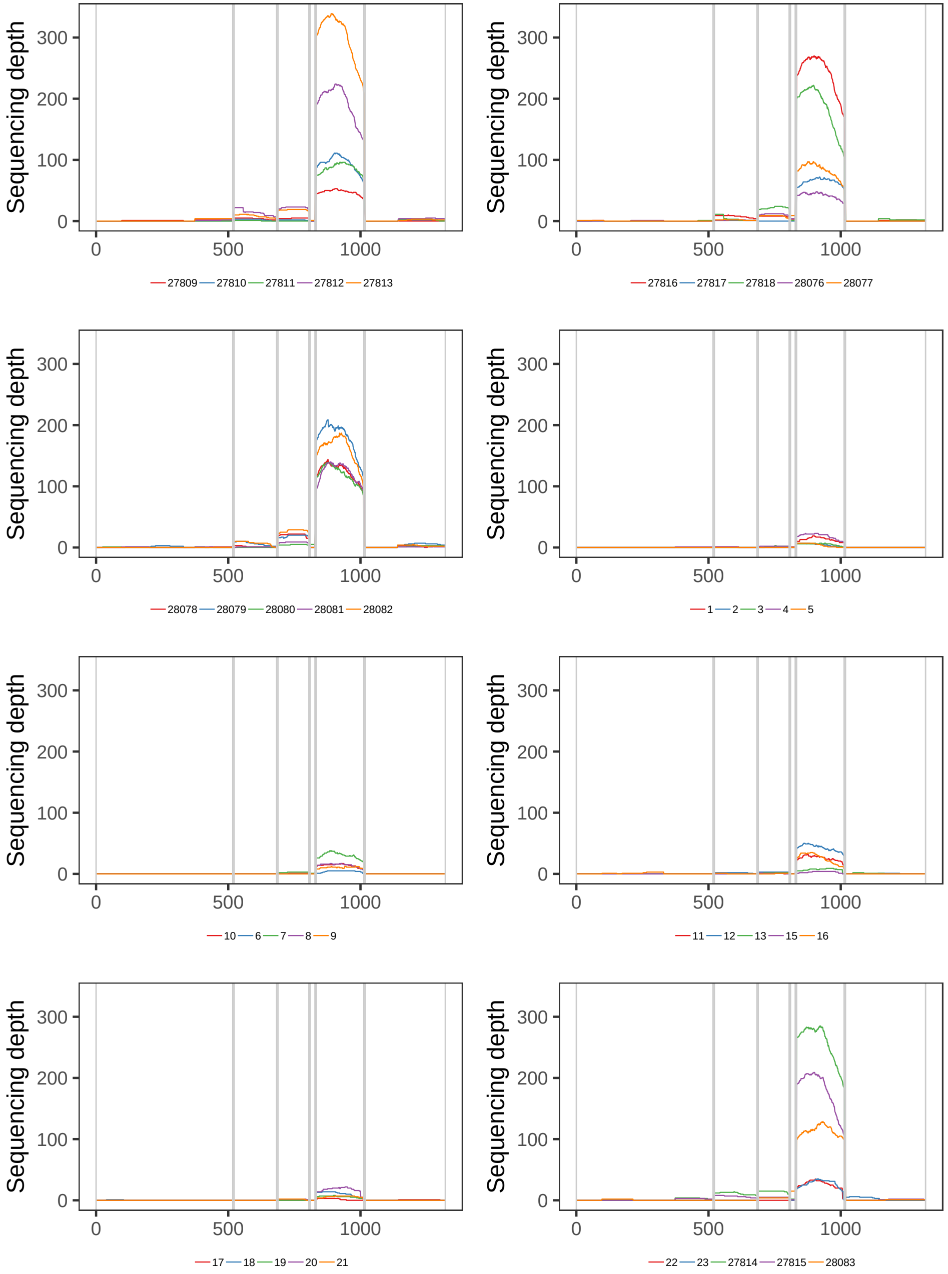
Sequencing depth
300
200
100
0
0
500
1000
27809 27810 27811 27812 27813
27816 27817 27818 28076 28077
28078 28079 28080 28081 28082
1 2 3 4 5
10 6 7 8 9
11 12 13 15 16
17 18 19 20 21
22 23 27814 27815 28083

EOG5KSN28

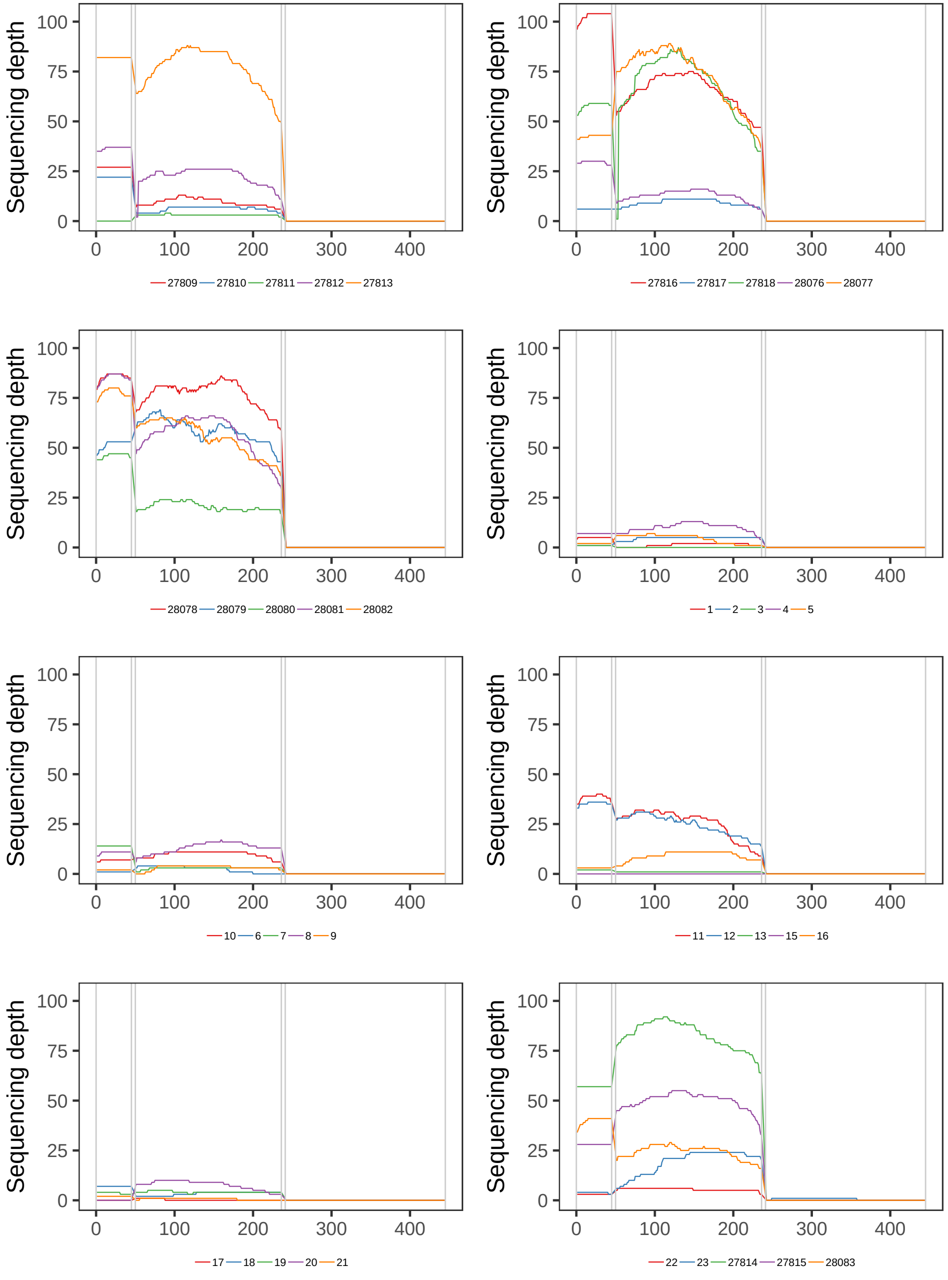
Sequencing depth
100
75
50
25
0
0
100
200
300
400
27809 27810 27811 27812 27813
27816 27817 27818 28076 28077
28078 28079 28080 28081 28082
1 2 3 4 5
10 6 7 8 9
11 12 13 15 16
17 18 19 20 21
22 23 27814 27815 28083

EOG5PC88N

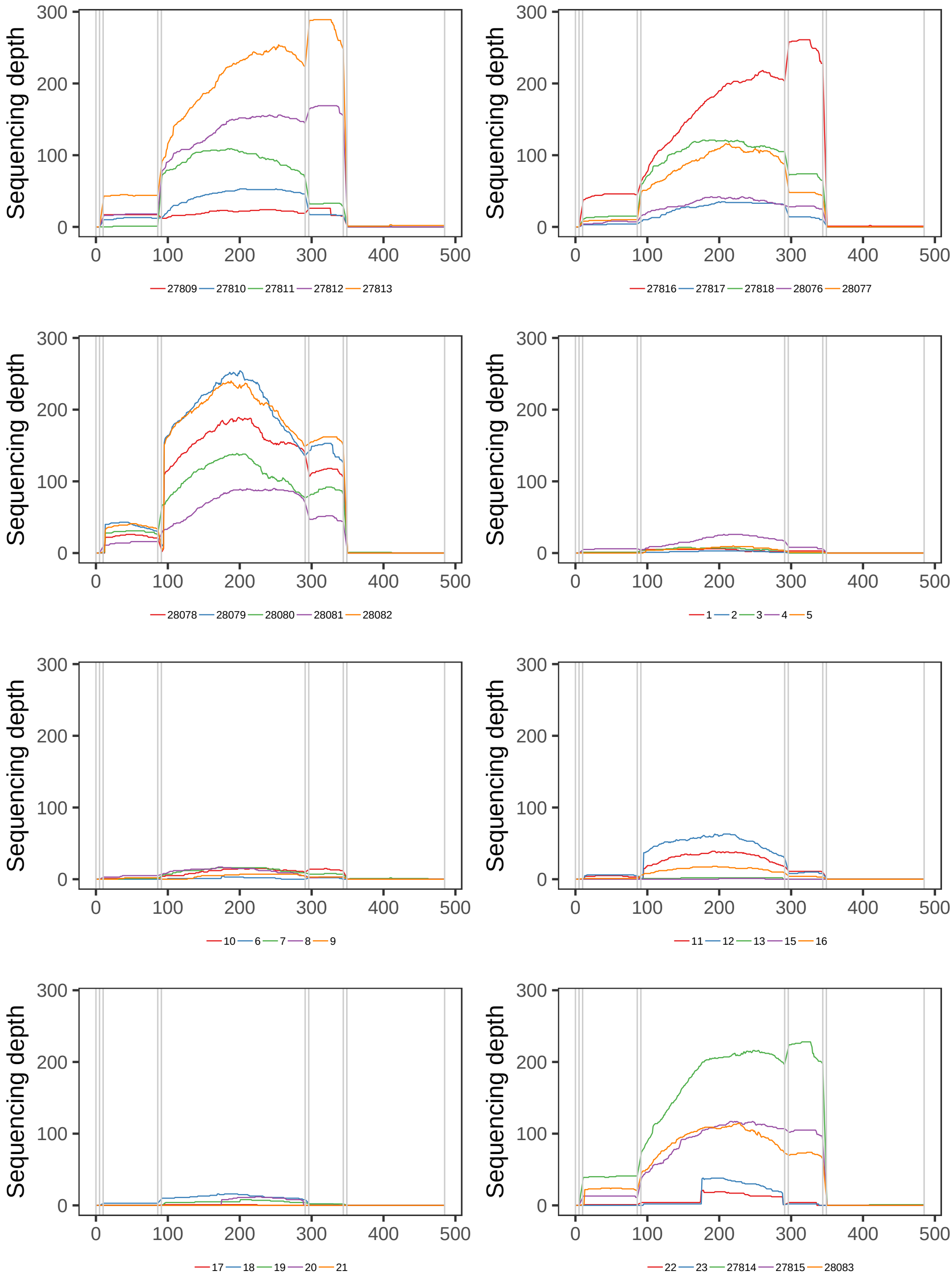

Sequencing depth
0
100
200
300
400
500
27809 27810 27811 27812 27813
27816 27817 27818 28076 28077
28078 28079 28080 28081 28082
1 2 3 4 5
10 6 7 8 9
11 12 13 15 16
17 18 19 20 21
22 23 27814 27815 28083

EOG5SXKVF

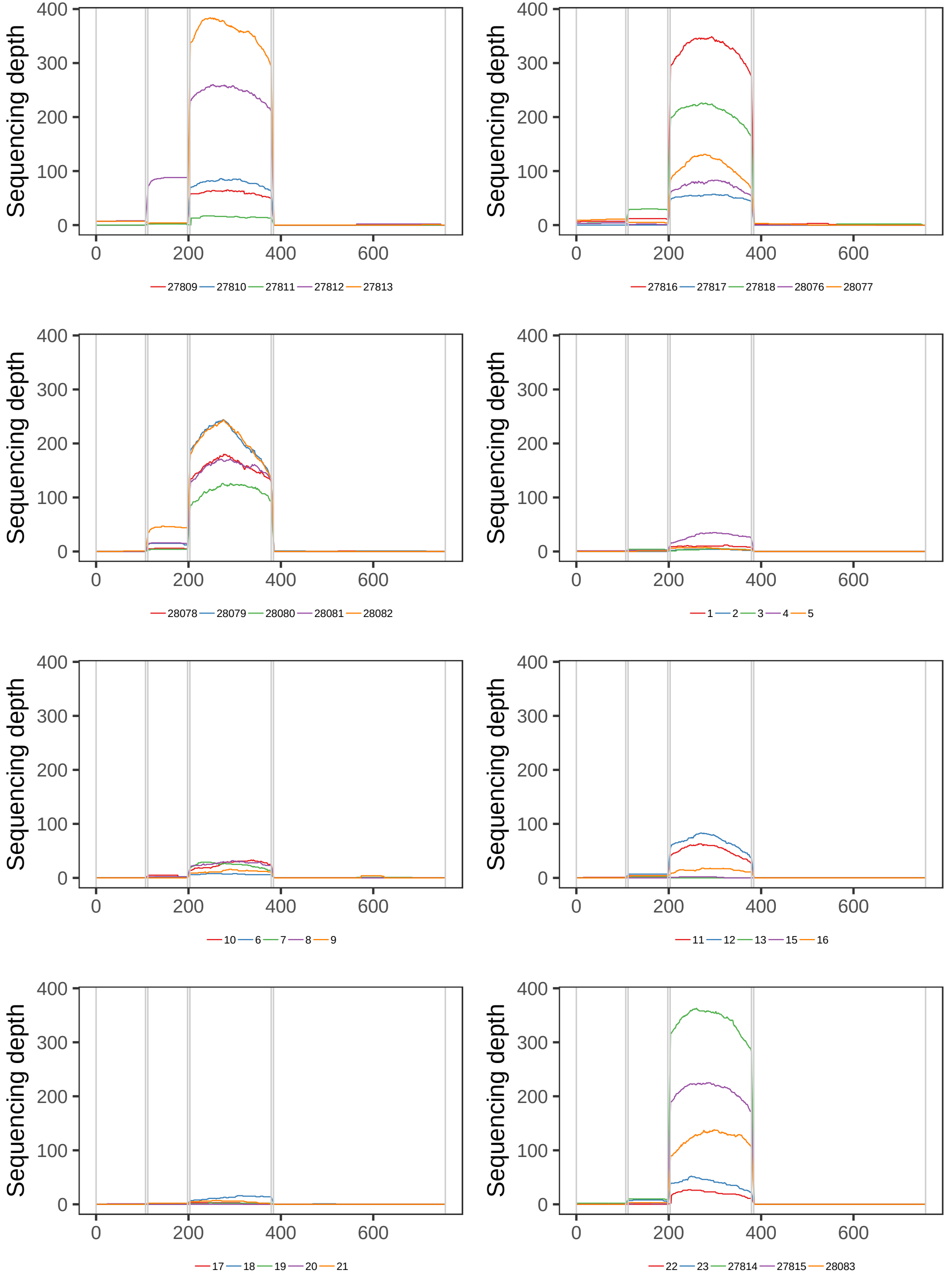
Sequencing depth
0
100
200
300
400
0
200
400
600
27809 27810 27811 27812 27813
27816 27817 27818 28076 28077
28078 28079 28080 28081 28082
1 2 3 4 5
10 6 7 8 9
11 12 13 15 16
17 18 19 20 21
22 23 27814 27815 28083

EOG5TDZ2X

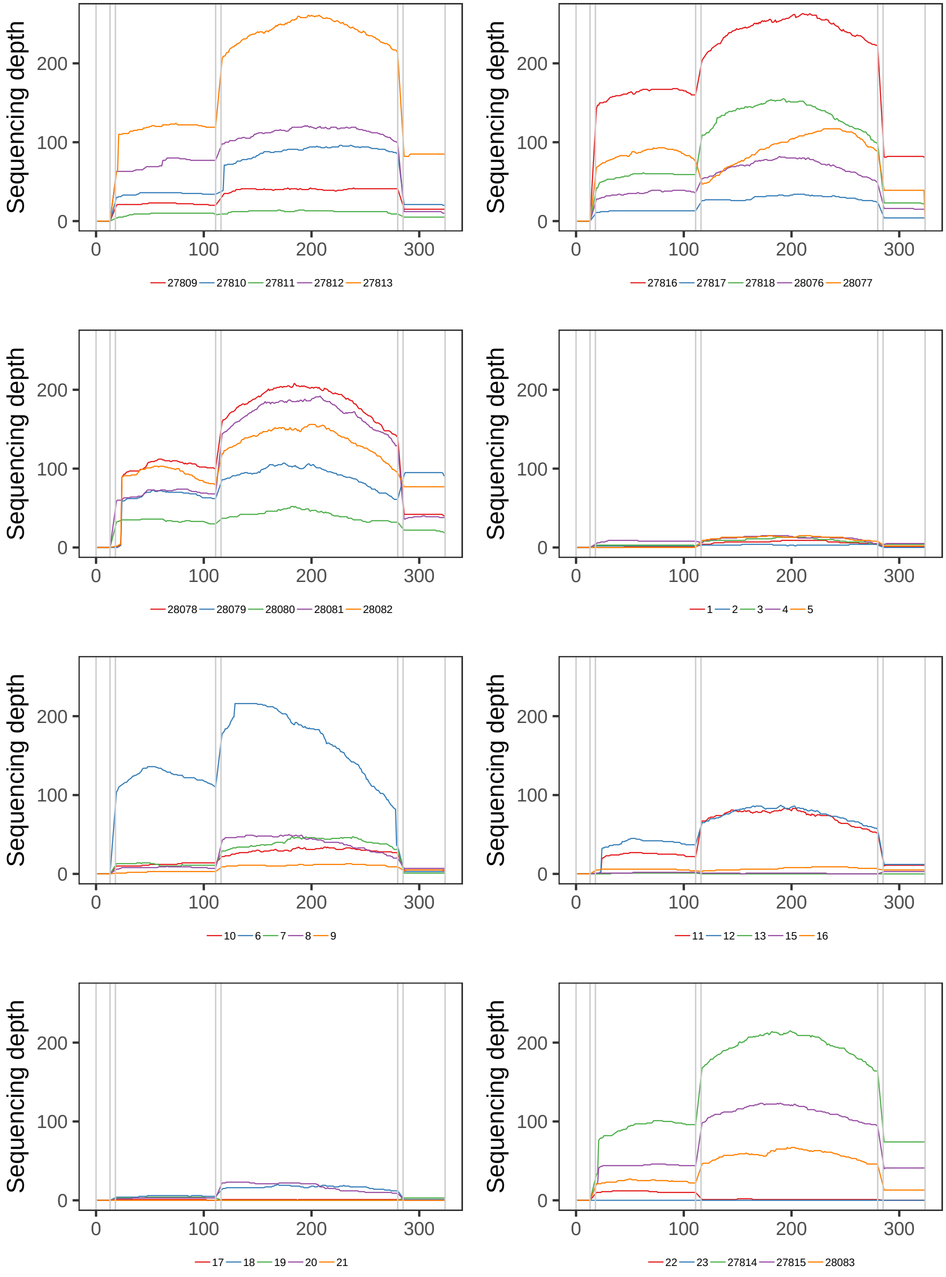
Sequencing depth
0
100
200
300
27809 27810 27811 27812 27813
27816 27817 27818 28076 28077
28078 28079 28080 28081 28082
1 2 3 4 5
10 6 7 8 9
11 12 13 15 16
17 18 19 20 21
22 23 27814 27815 28083

EOG5VQ851

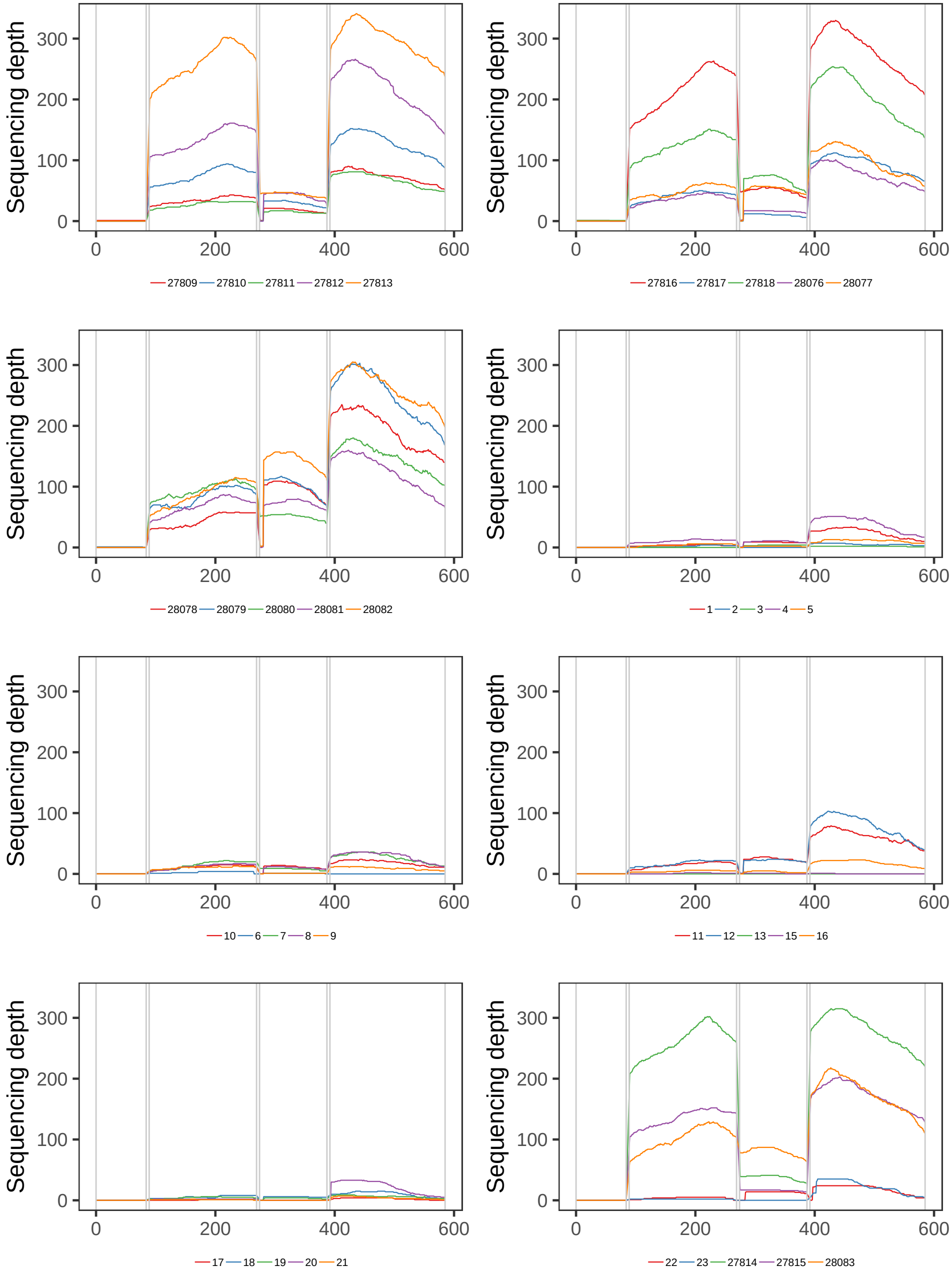
Sequencing depth
0
100
200
300
0
200
400
600
27809 27810 27811 27812 27813
27816 27817 27818 28076 28077
28078 28079 28080 28081 28082
1 2 3 4 5
10 6 7 8 9
11 12 13 15 16
17 18 19 20 21
22 23 27814 27815 28083

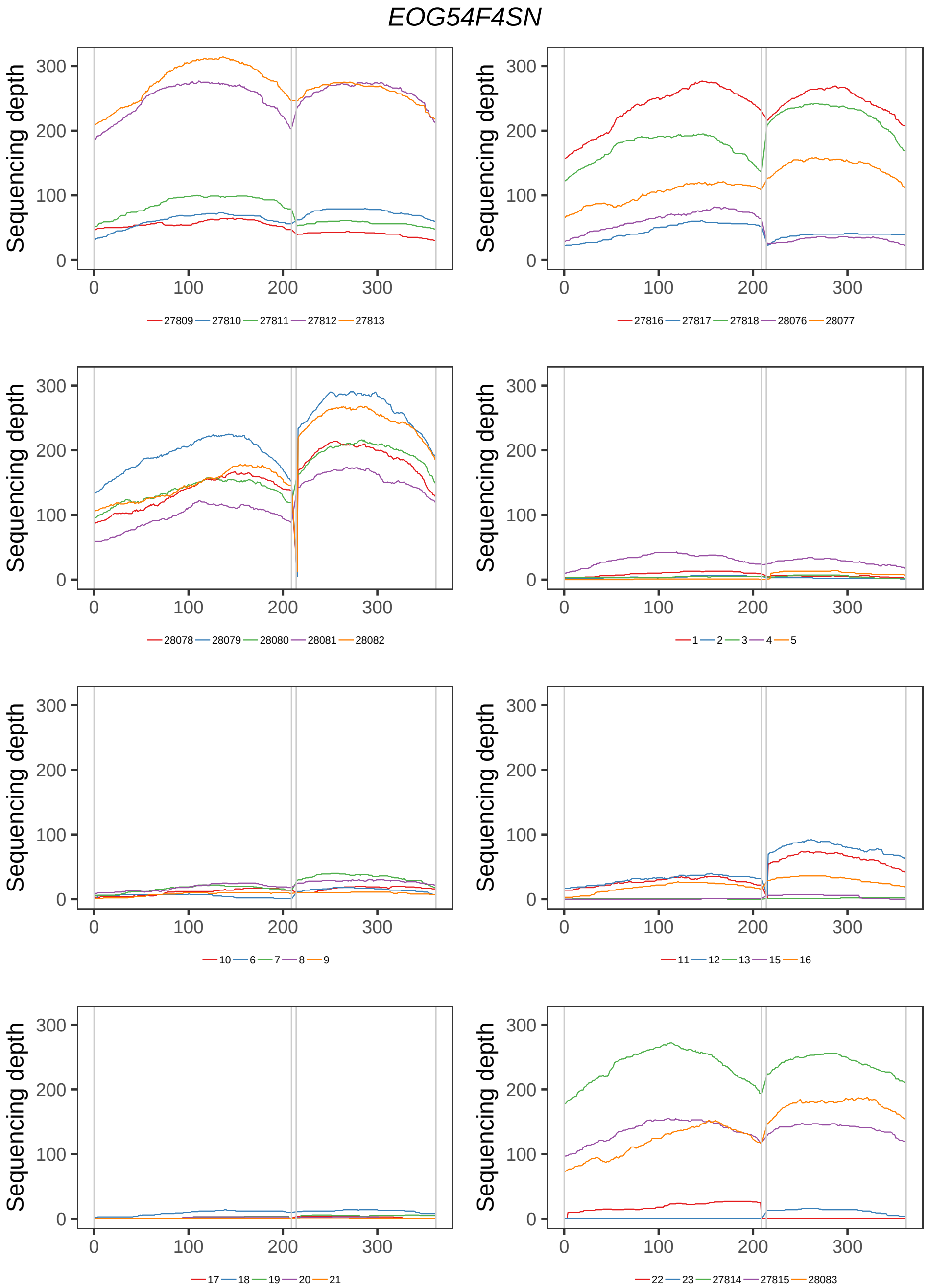

EOG54F4SN
Sequencing depth
0
100
200
300
27809 27810 27811 27812 27813
27816 27817 27818 28076 28077
28078 28079 28080 28081 28082
1 2 3 4 5
10 6 7 8 9
11 12 13 15 16
17 18 19 20 21
22 23 27814 27815 28083

EOG5547F7

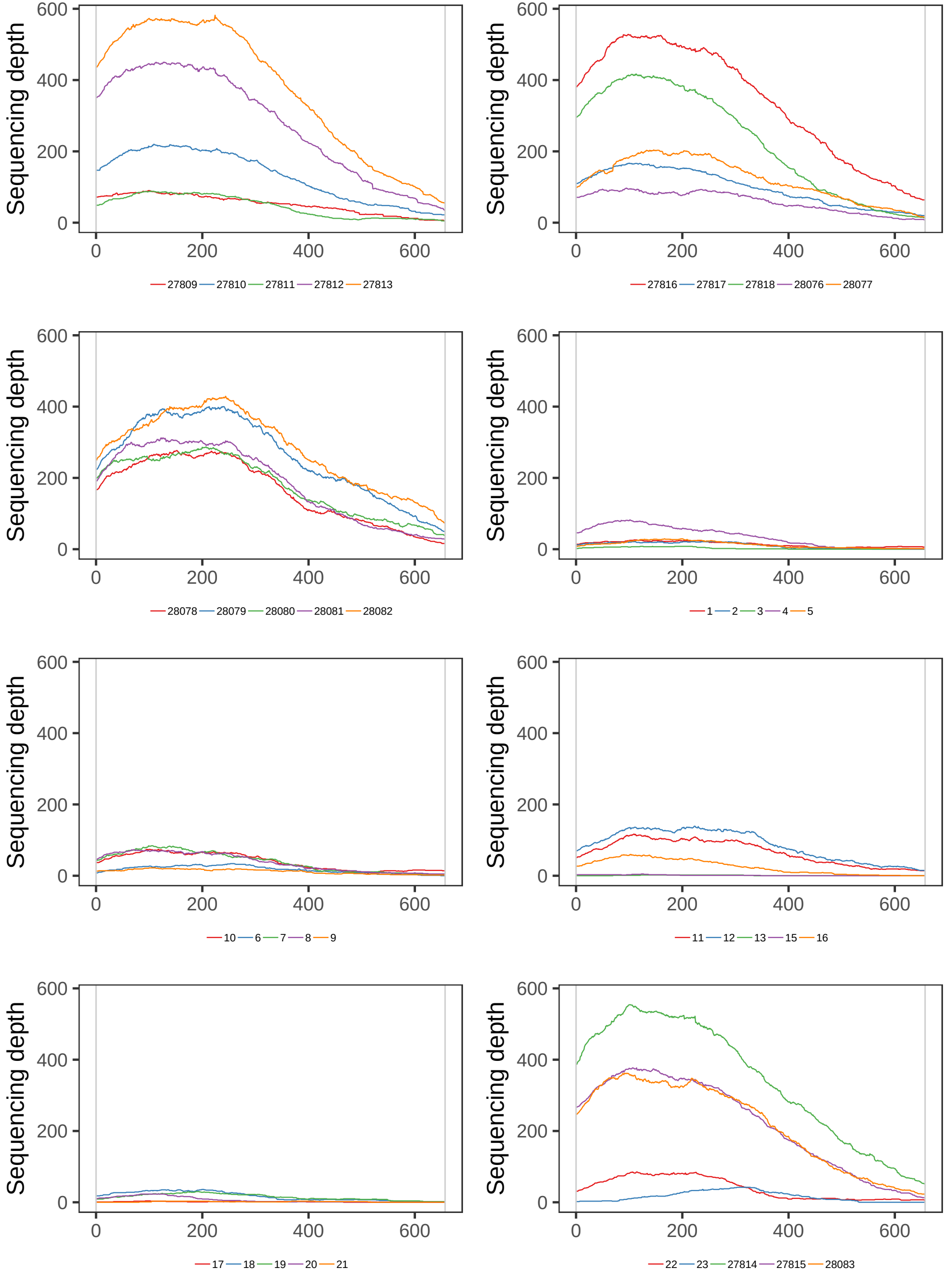
Sequencing depth
0
200
400
600
27809 27810 27811 27812 27813
27816 27817 27818 28076 28077
28078 28079 28080 28081 28082
1 2 3 4 5
10 6 7 8 9
11 12 13 15 16
17 18 19 20 21
22 23 27814 27815 28083

EOG569P8X

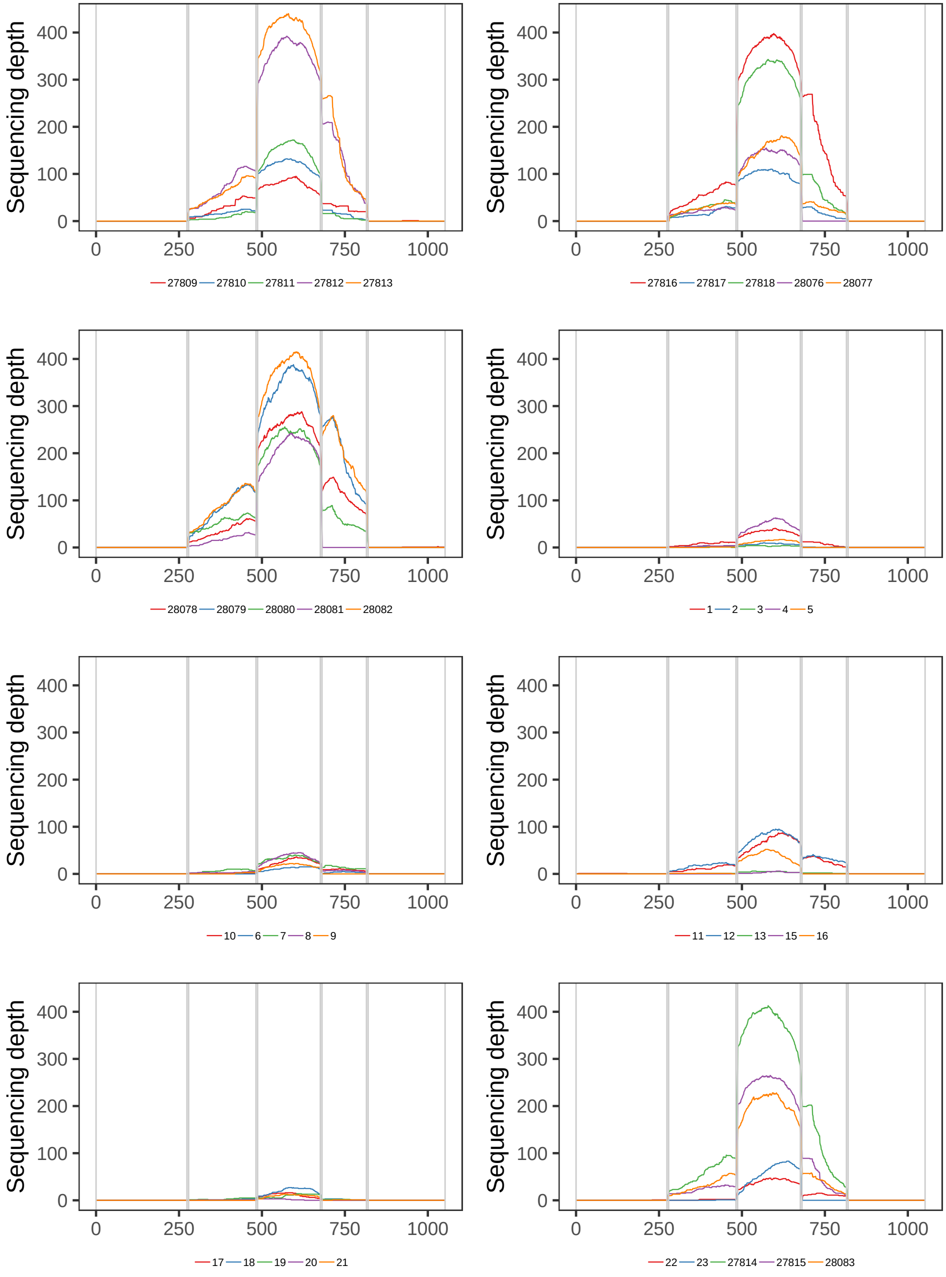
Sequencing depth
0
100
200
300
400
0
250
500
750
1000
27809
27810
27811
27812
27813
27816
27817
27818
28076
28077
28078
28079
28080
28081
28082
1
2
3
4
5
10
6
7
8
9
11
12
13
15
16
17
18
19
20
21
22
23
27814
27815
28083

EOG579CQD

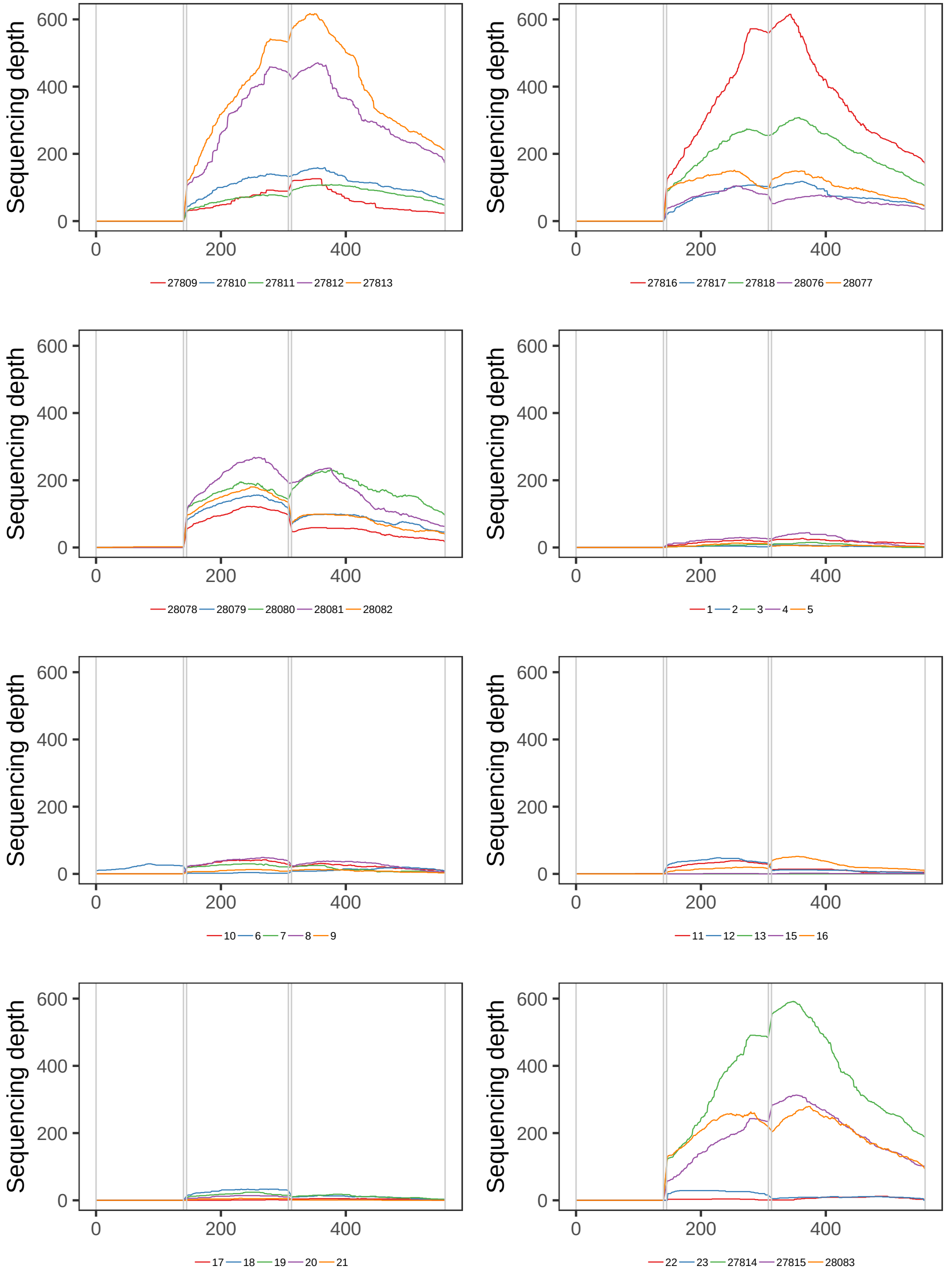
Sequencing depth
600
400
200
0
0
200
400
27809 27810 27811 27812 27813
27816 27817 27818 28076 28077
28078 28079 28080 28081 28082
1 2 3 4 5
10 6 7 8 9
11 12 13 15 16
17 18 19 20 21
22 23 27814 27815 28083

EOG58932Z

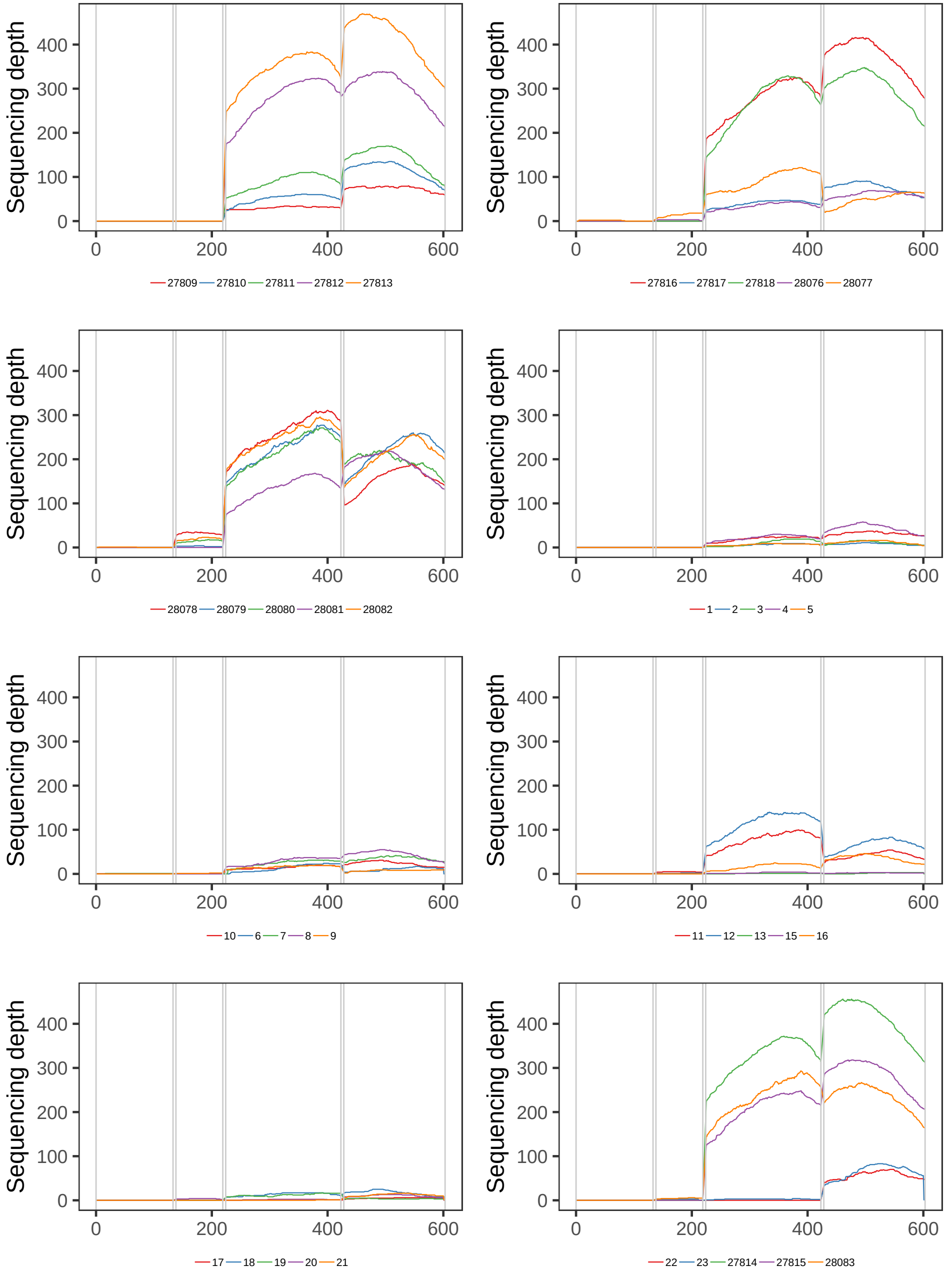
Sequencing depth
0
100
200
300
400
600
27809 27810 27811 27812 27813
27816 27817 27818 28076 28077
28078 28079 28080 28081 28082
1 2 3 4 5
10 6 7 8 9
11 12 13 15 16
17 18 19 20 21
22 23 27814 27815 28083

EOG595X6S

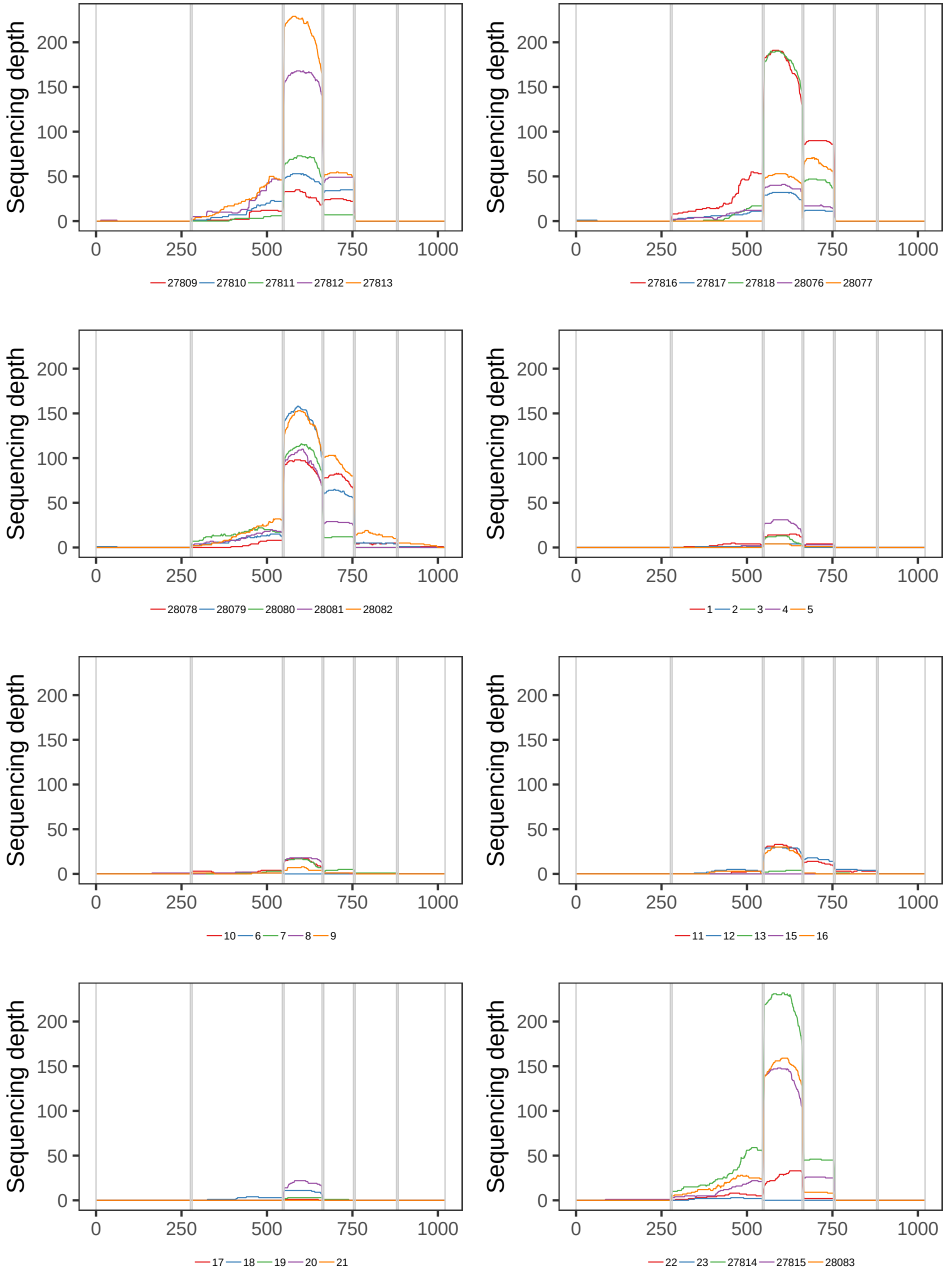

Sequencing depth
27809 27810 27811 27812 27813
27816 27817 27818 28076 28077
28078 28079 28080 28081 28082
1 2 3 4 5
10 6 7 8 9
11 12 13 15 16
17 18 19 20 21
22 23 27814 27815 28083

EOG598SGG

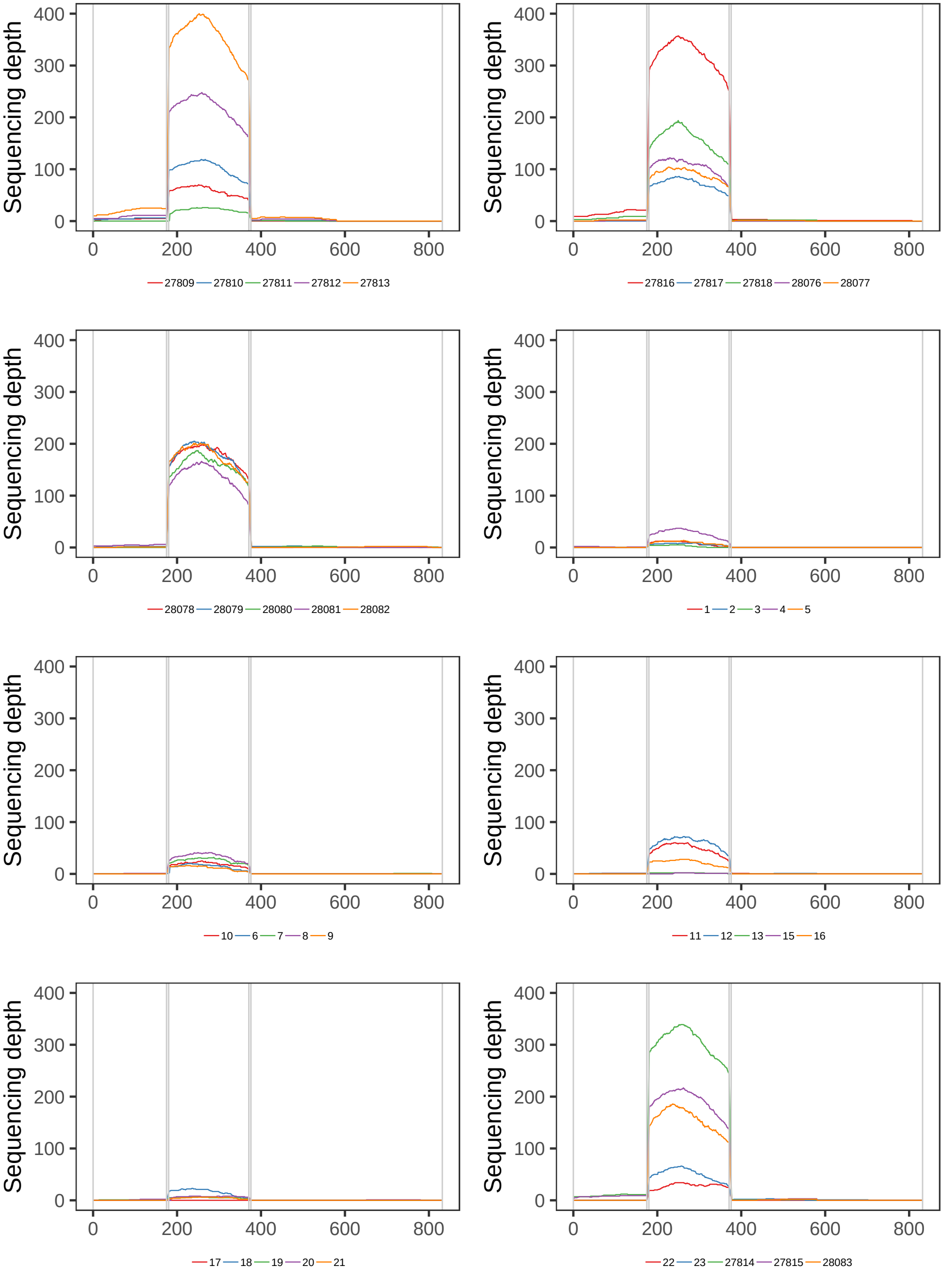
Sequencing depth
0
100
200
300
400
0
200
400
600
800
27809 27810 27811 27812 27813
27816 27817 27818 28076 28077
28078 28079 28080 28081 28082
1 2 3 4 5
10 6 7 8 9
11 12 13 15 16
17 18 19 20 21
22 23 27814 27815 28083

EOG5BZKK2

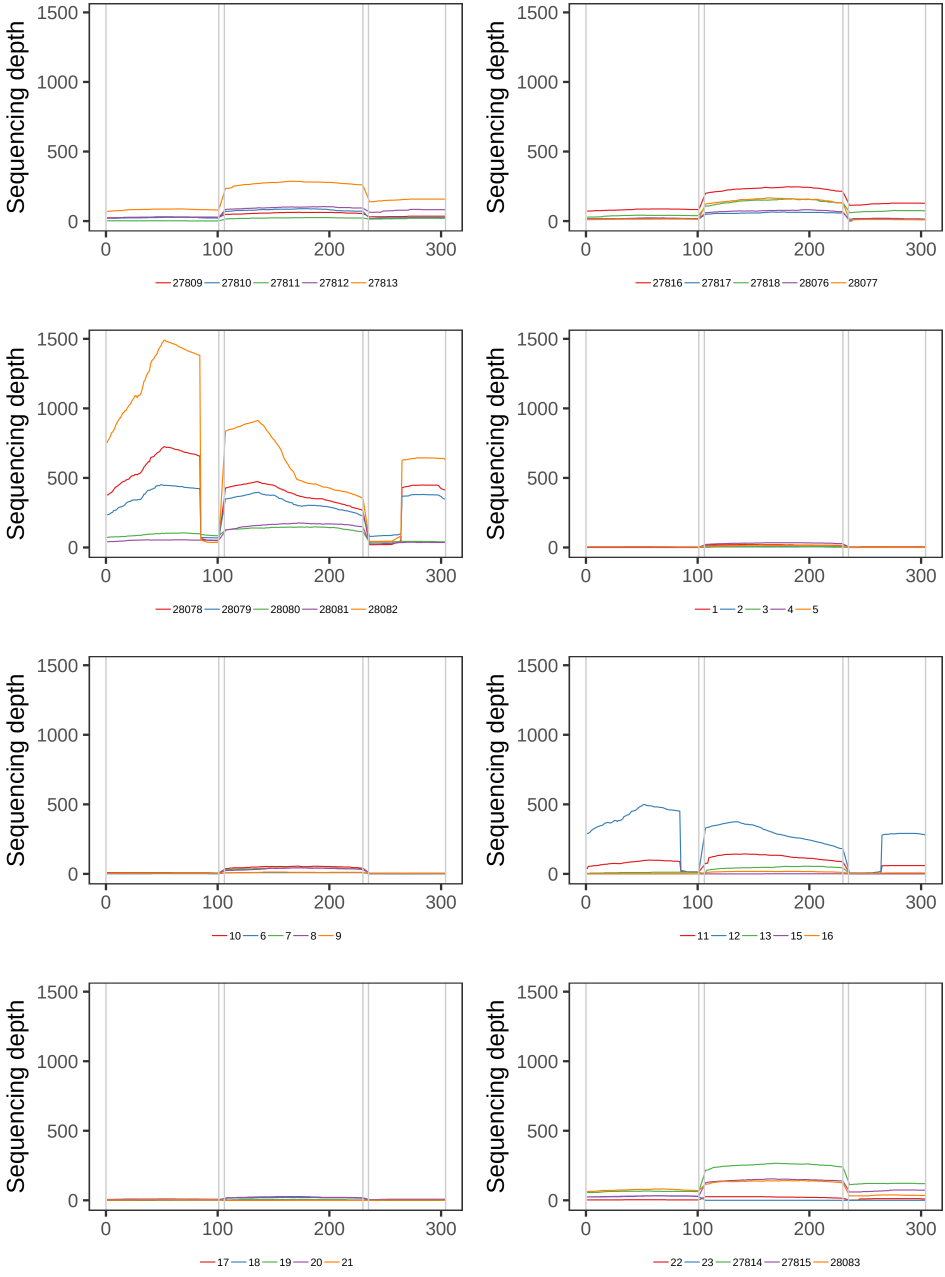
Sequencing depth
1500
1000
500
0
0
100
200
300
27809 27810 27811 27812 27813
27816 27817 27818 28076 28077
28078 28079 28080 28081 28082
1 2 3 4 5
10 6 7 8 9
11 12 13 15 16
17 18 19 20 21
22 23 27814 27815 28083

EOG5F4QTD

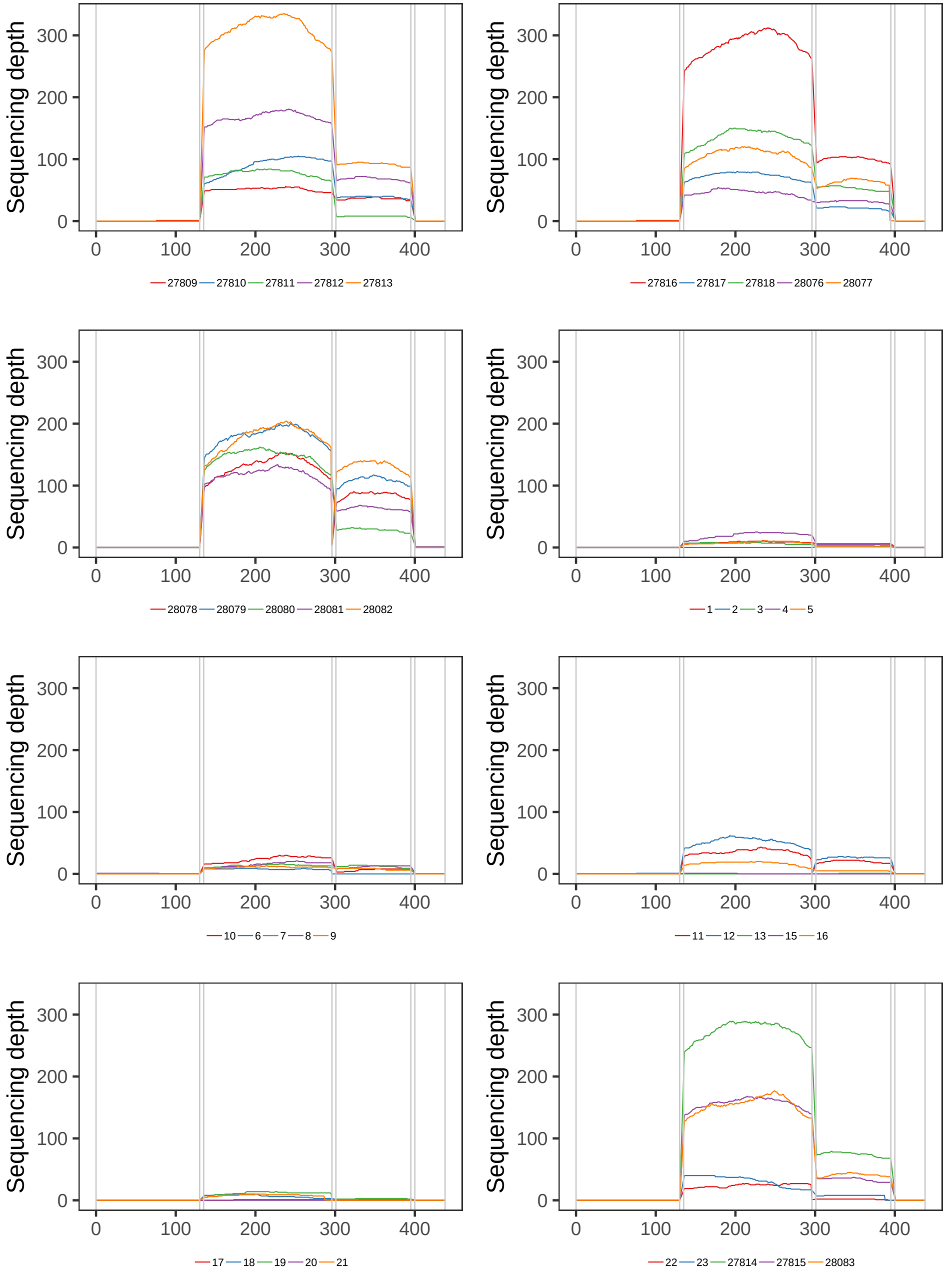
Sequencing depth
0
100
200
300
0
100
200
300
400
27809 27810 27811 27812 27813
27816 27817 27818 28076 28077
28078 28079 28080 28081 28082
1 2 3 4 5
10 6 7 8 9
11 12 13 15 16
17 18 19 20 21
22 23 27814 27815 28083

EOG5F7M20

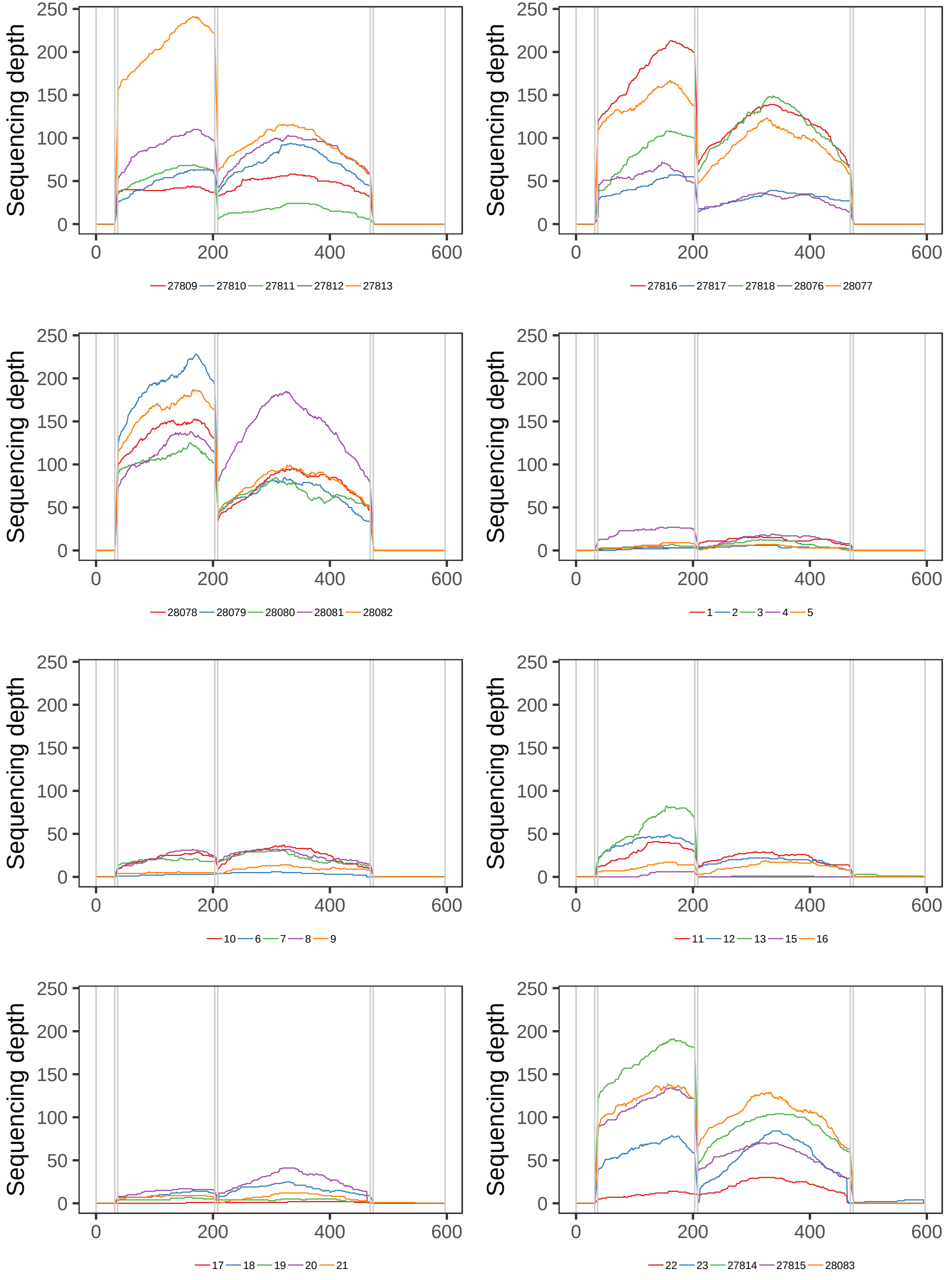
Sequencing depth
0 50 100 150 200 250
0 200 400 600
27809 27810 27811 27812 27813
27816 27817 27818 28076 28077
28078 28079 28080 28081 28082
1 2 3 4 5
10 6 7 8 9
11 12 13 15 16
17 18 19 20 21
22 23 27814 27815 28083

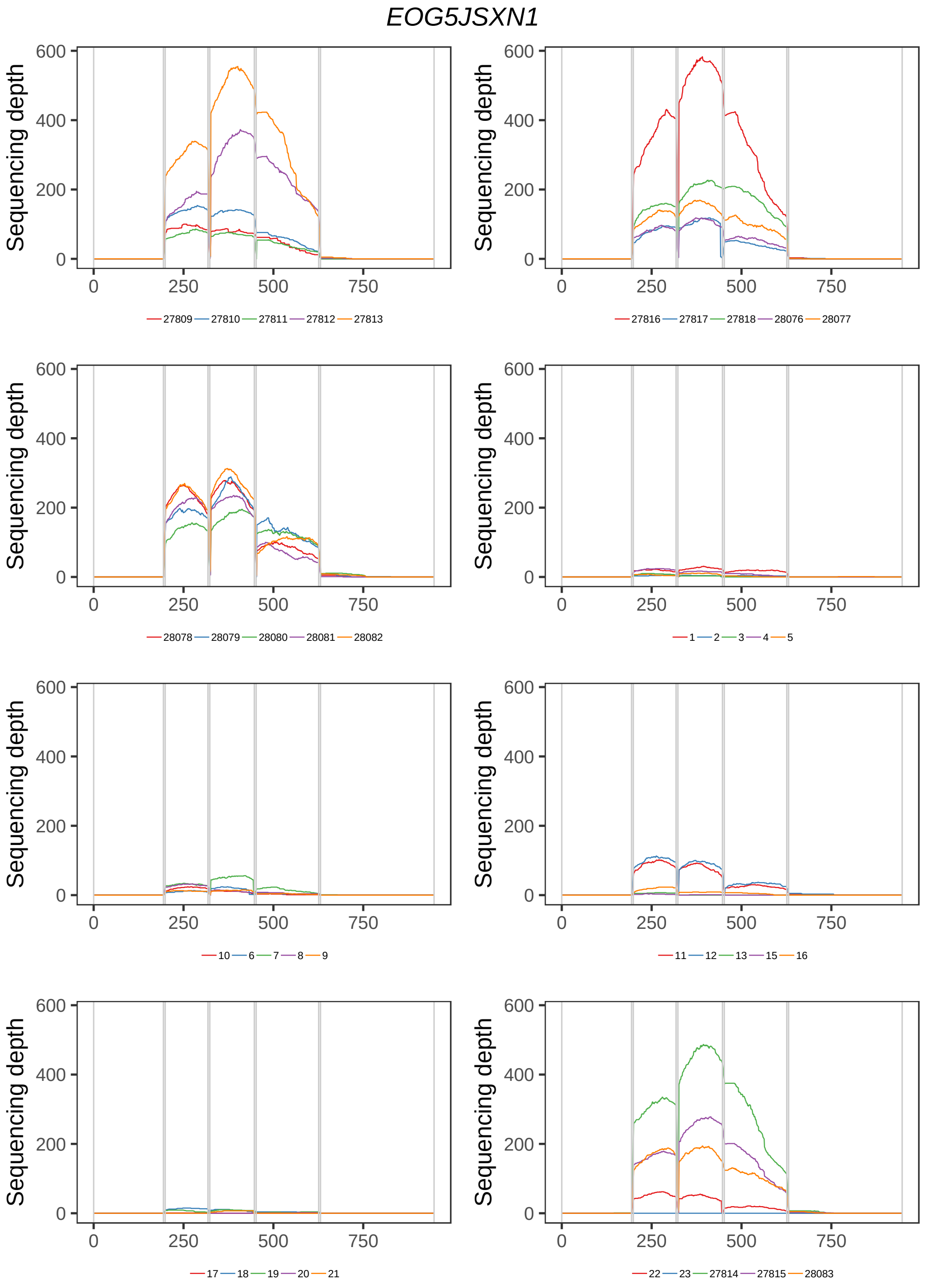
EOG5JSXN1
Sequencing depth
0
200
400
600
0
250
500
750
27809 27810 27811 27812 27813
27816 27817 27818 28076 28077
28078 28079 28080 28081 28082
1 2 3 4 5
10 6 7 8 9
11 12 13 15 16
17 18 19 20 21
22 23 27814 27815 28083

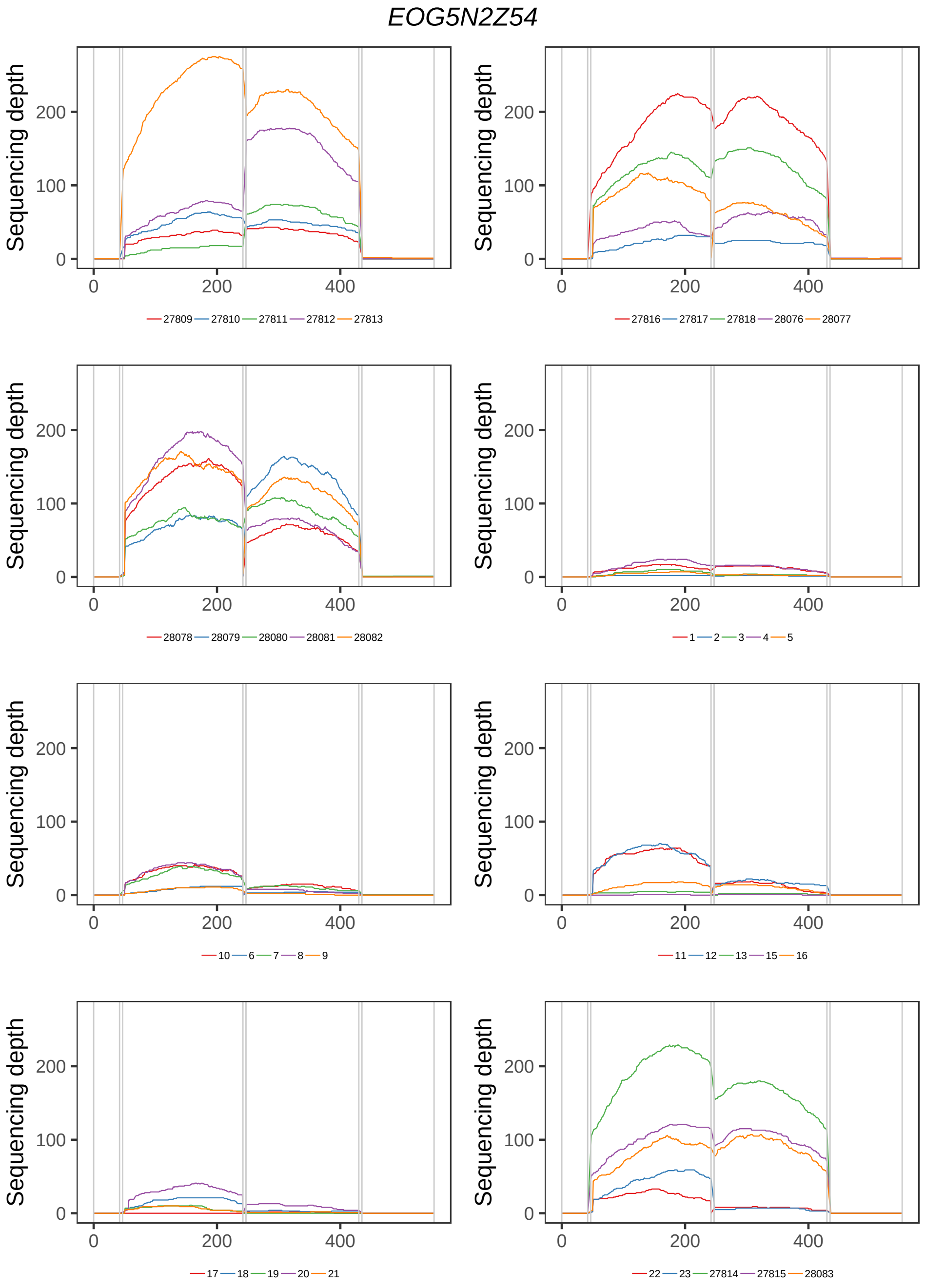

EOG5N2Z54
Sequencing depth
0
100
200
400
27809 27810 27811 27812 27813
27816 27817 27818 28076 28077
28078 28079 28080 28081 28082
1 2 3 4 5
10 6 7 8 9
11 12 13 15 16
17 18 19 20 21
22 23 27814 27815 28083

EOG5NZS8B

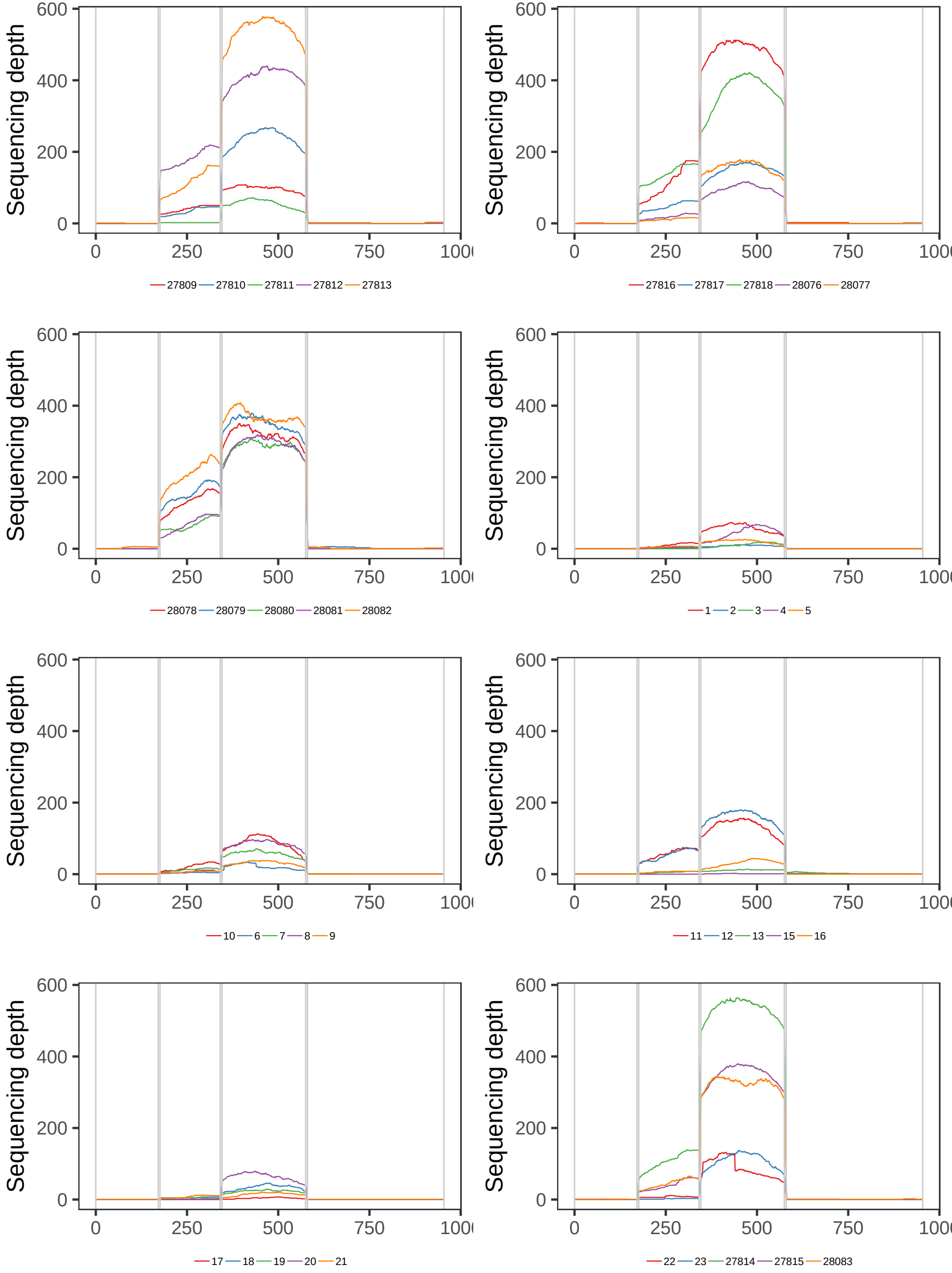
Sequencing depth
600
400
200
0
0
250
500
750
1000
27809 27810 27811 27812 27813
27816 27817 27818 28076 28077
28078 28079 28080 28081 28082
1 2 3 4 5
10 6 7 8 9
11 12 13 15 16
17 18 19 20 21
22 23 27814 27815 28083

EOG5PC878

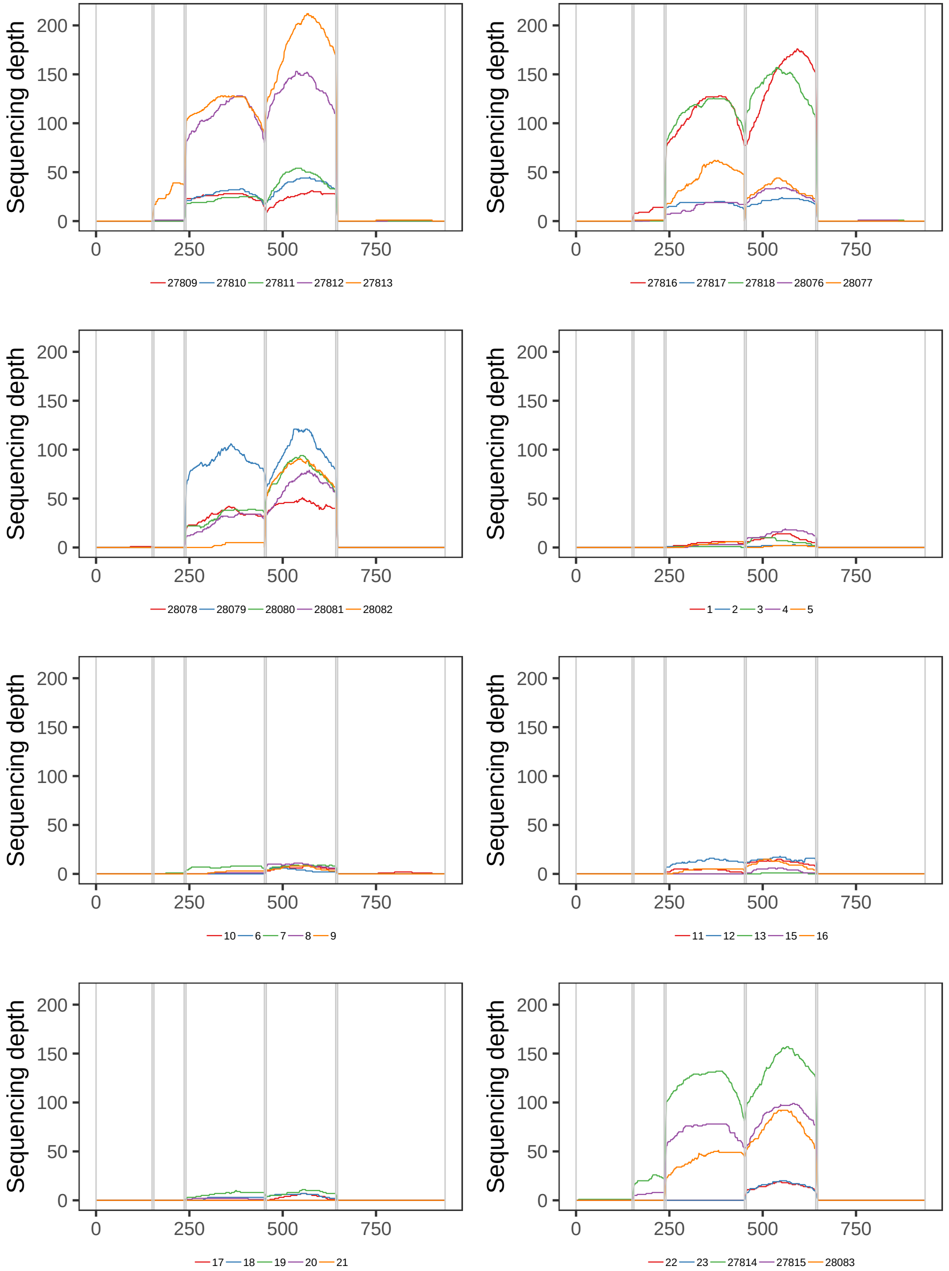
Sequencing depth
0
50
100
150
200
0
250
500
750
27809 27810 27811 27812 27813
27816 27817 27818 28076 28077
28078 28079 28080 28081 28082
1 2 3 4 5
10 6 7 8 9
11 12 13 15 16
17 18 19 20 21
22 23 27814 27815 28083

EOG5PG4HC

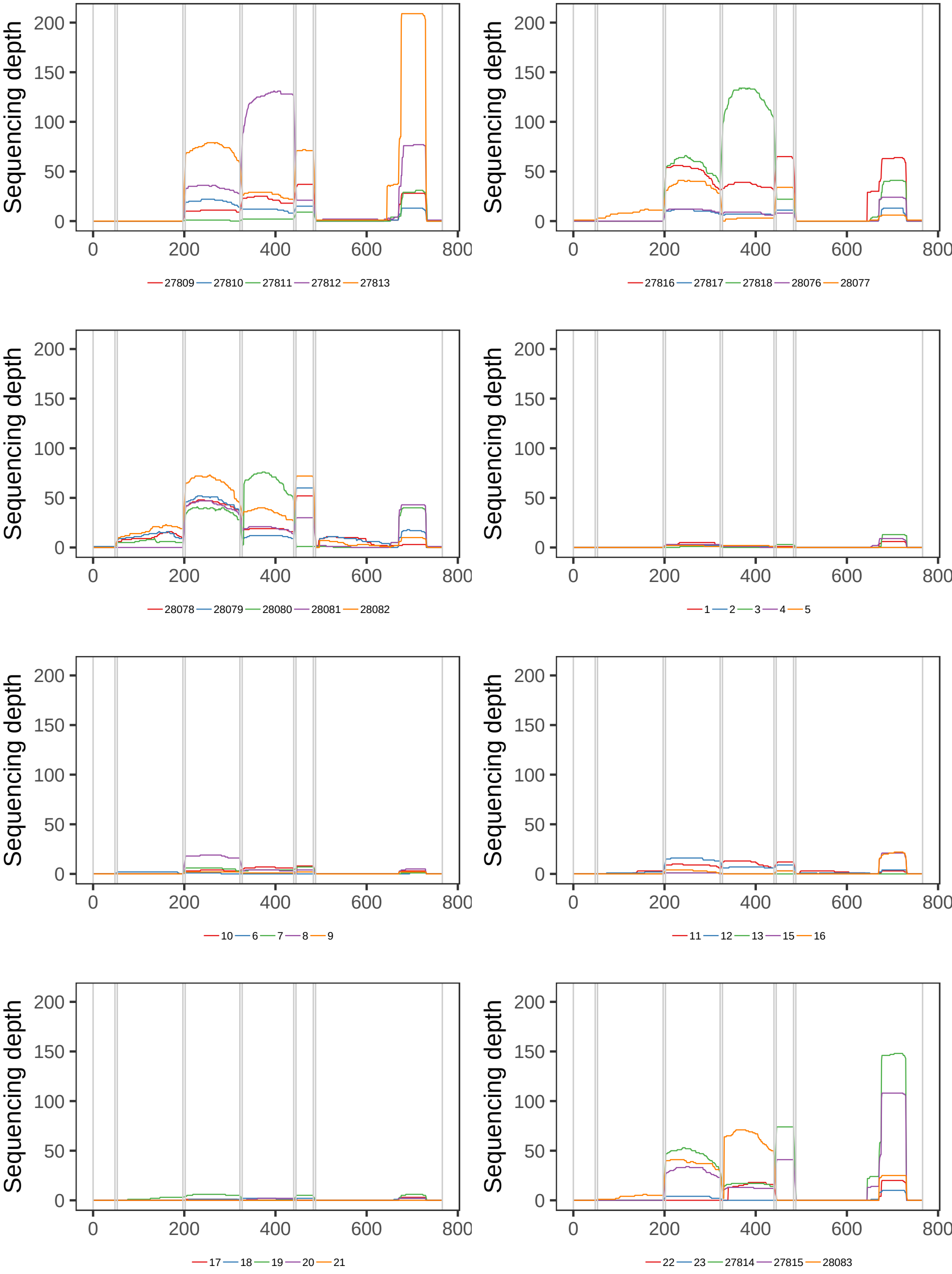

Sequencing depth
0
50
100
150
200
0
200
400
600
800
27809 27810 27811 27812 27813
27816 27817 27818 28076 28077
28078 28079 28080 28081 28082
1 2 3 4 5
10 6 7 8 9
11 12 13 15 16
17 18 19 20 21
22 23 27814 27815 28083

EOG5QNKBN

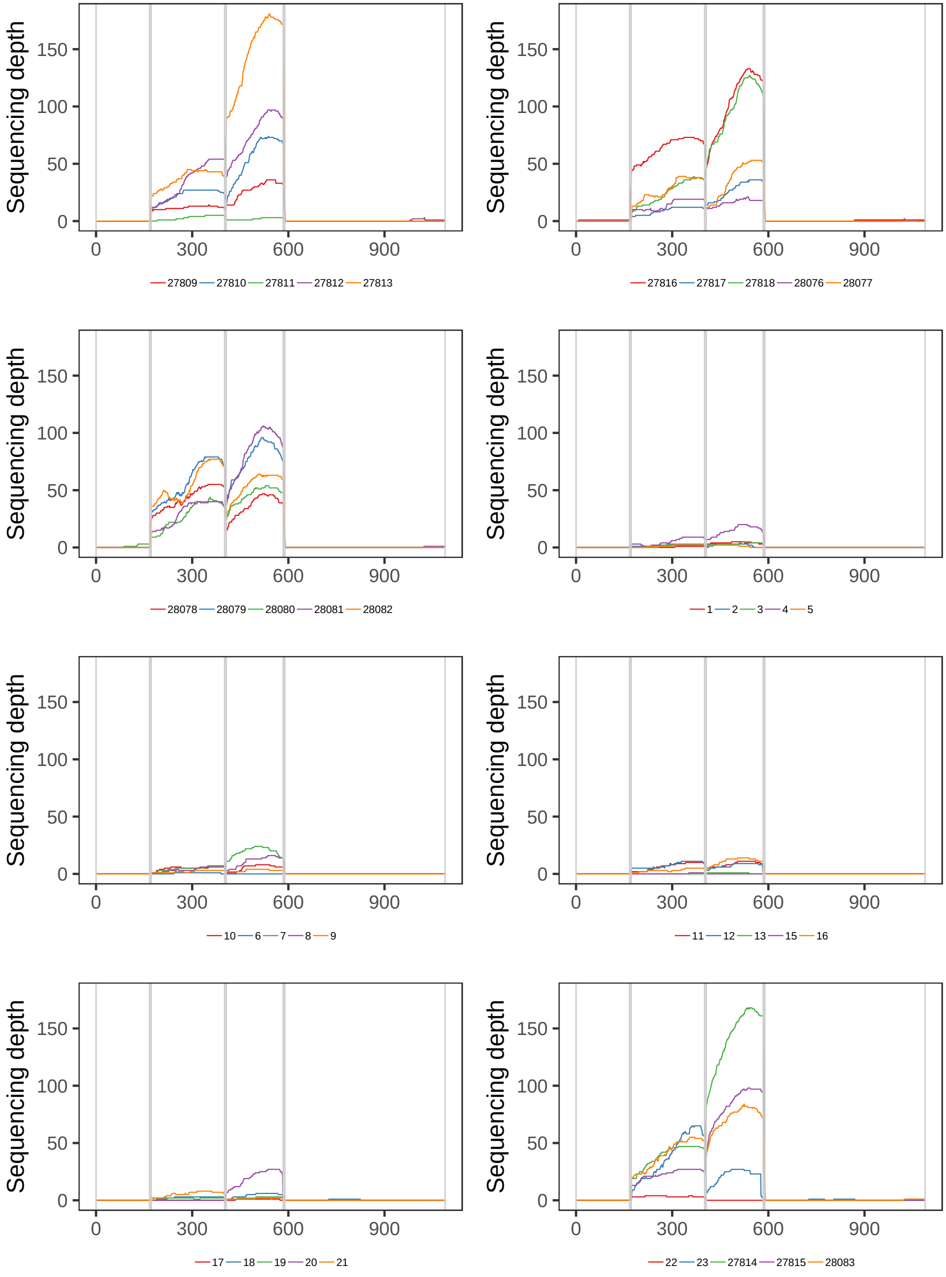
Sequencing depth
150
100
50
0
0
300
600
900
27809 27810 27811 27812 27813
27816 27817 27818 28076 28077
28078 28079 28080 28081 28082
1 2 3 4 5
10 6 7 8 9
11 12 13 15 16
17 18 19 20 21
22 23 27814 27815 28083

EOG5SBCDR

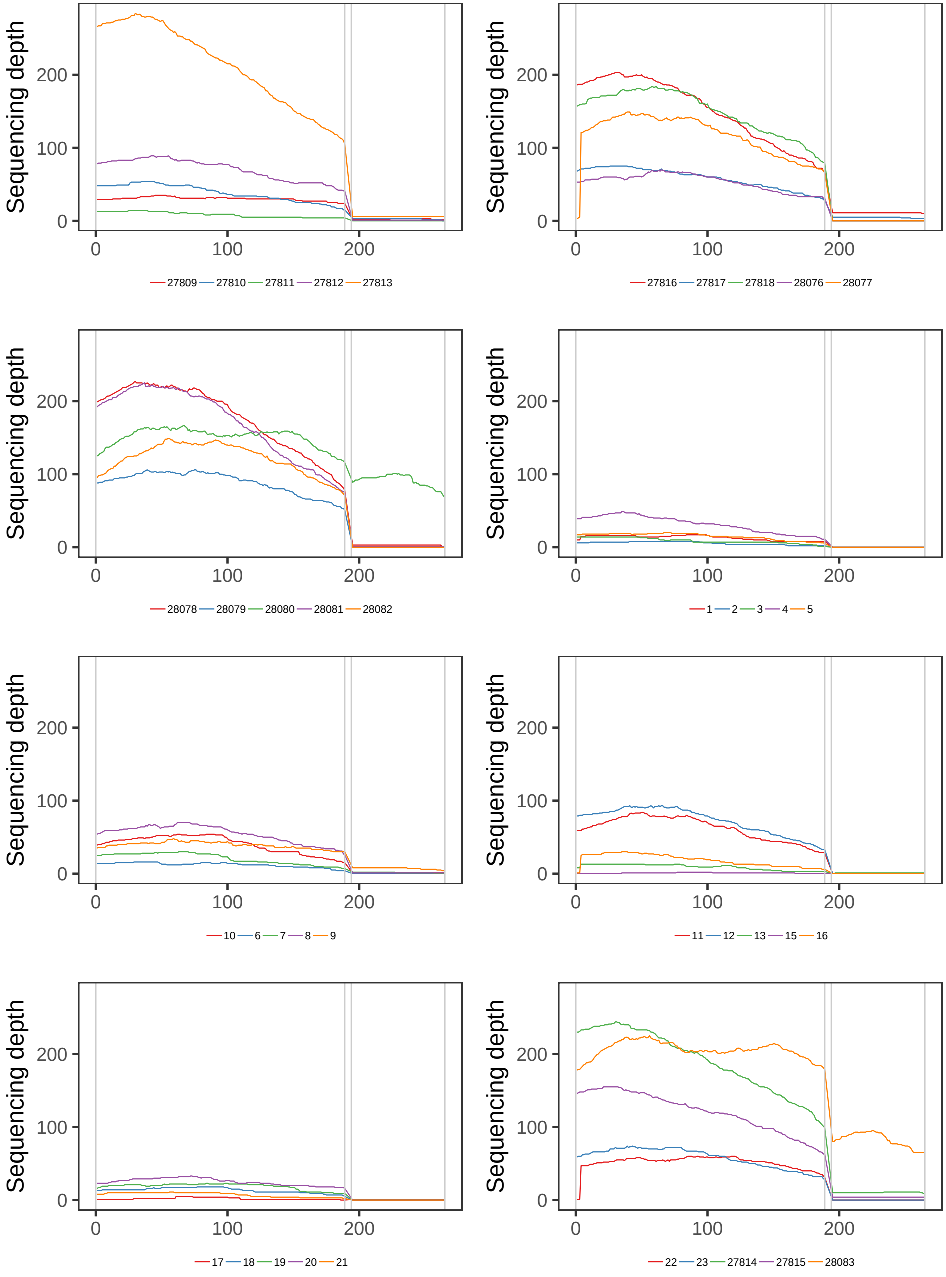
Sequencing depth
0
100
200
27809
27810
27811
27812
27813
27816
27817
27818
28076
28077
28078
28079
28080
28081
28082
1
2
3
4
5
10
6
7
8
9
11
12
13
15
16
17
18
19
20
21
22
23
27814
27815
28083

EOG5TTF10

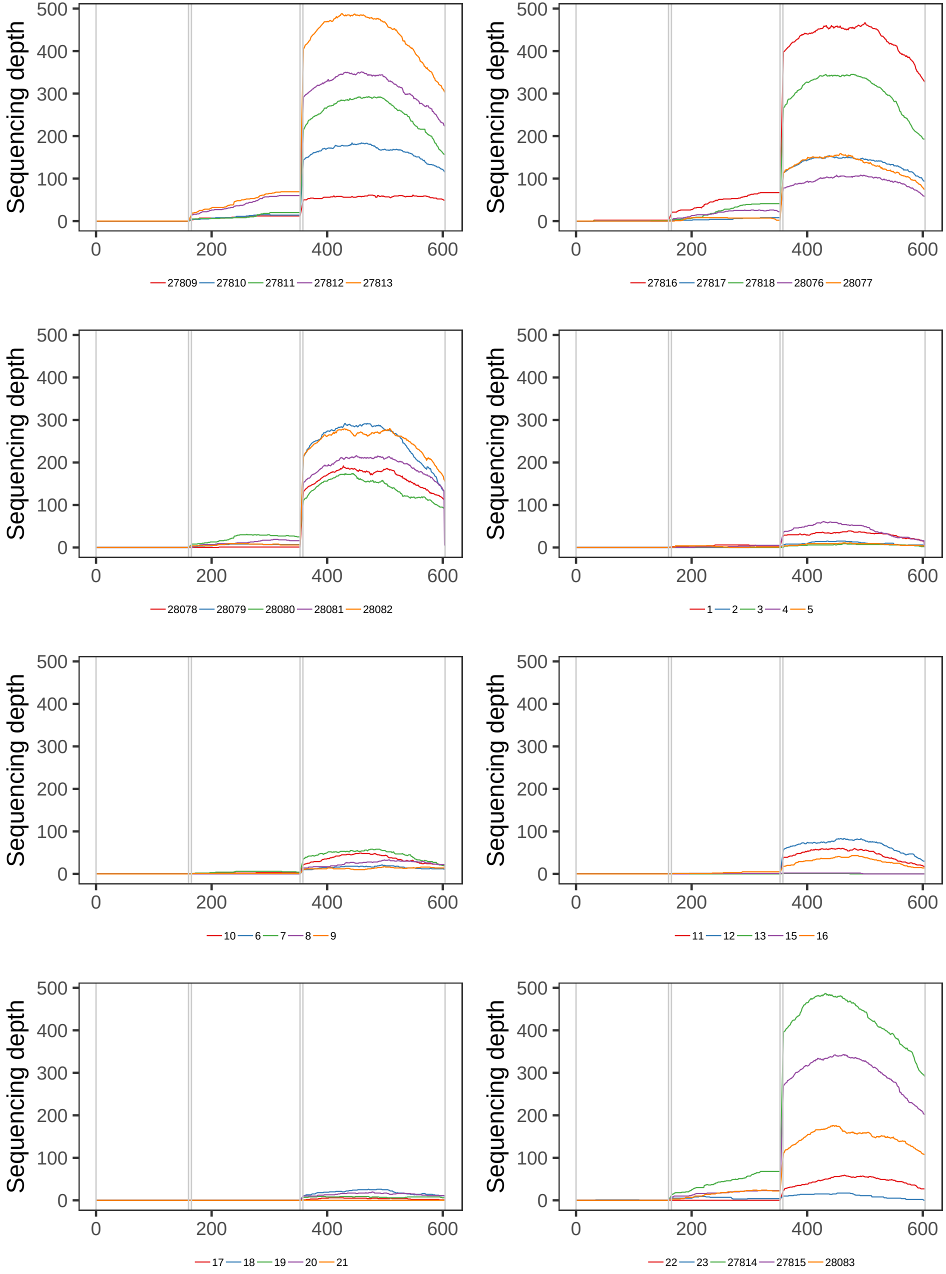
Sequencing depth
0
100
200
300
400
500
0
200
400
600
27809 27810 27811 27812 27813
27816 27817 27818 28076 28077
28078 28079 28080 28081 28082
1 2 3 4 5
10 6 7 8 9
11 12 13 15 16
17 18 19 20 21
22 23 27814 27815 28083

EOG51C5B7

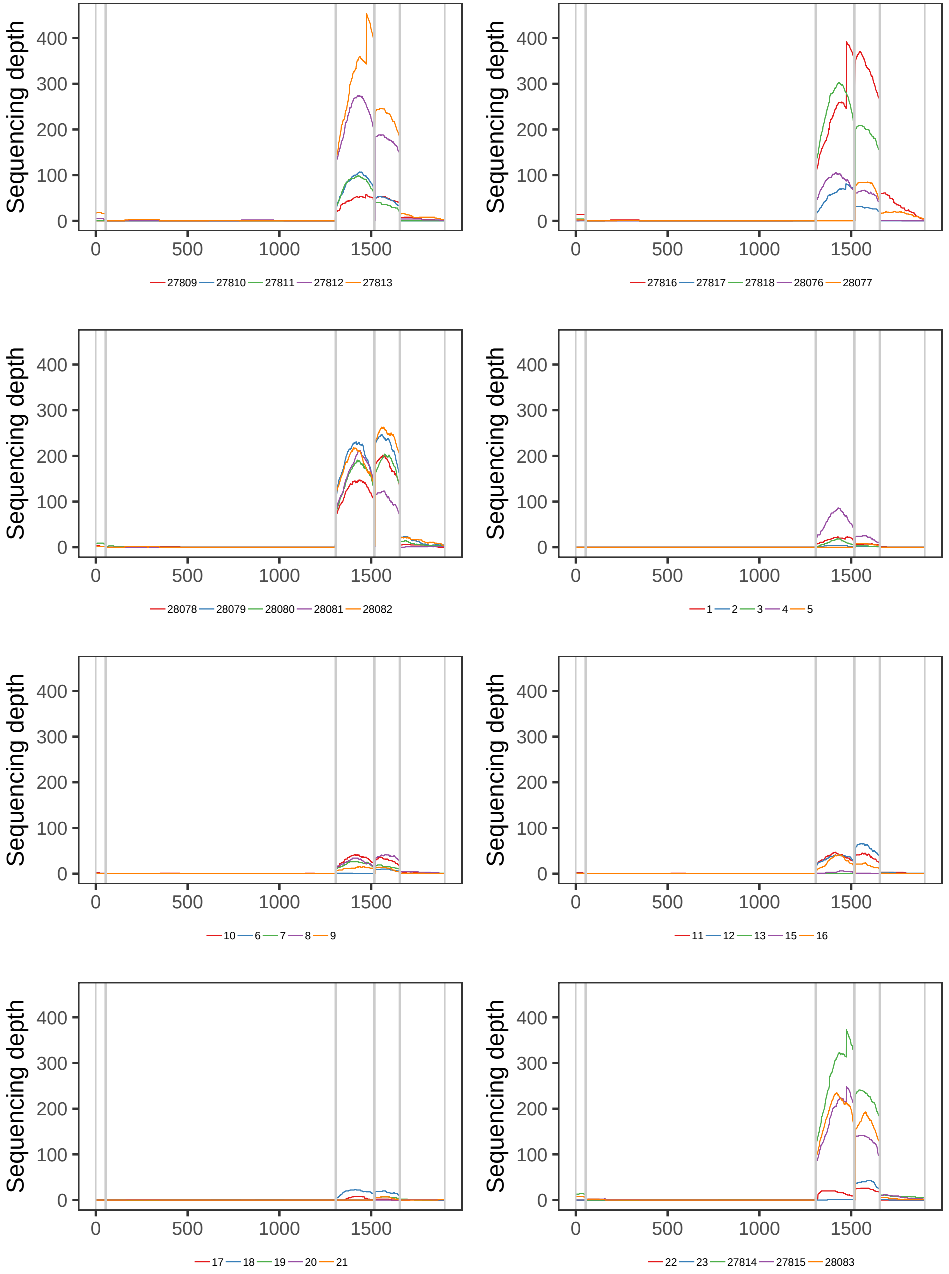

Sequencing depth
0
100
200
300
400
0
500
1000
1500
27809 27810 27811 27812 27813
27816 27817 27818 28076 28077
28078 28079 28080 28081 28082
1 2 3 4 5
10 6 7 8 9
11 12 13 15 16
17 18 19 20 21
22 23 27814 27815 28083

EOG52FR03

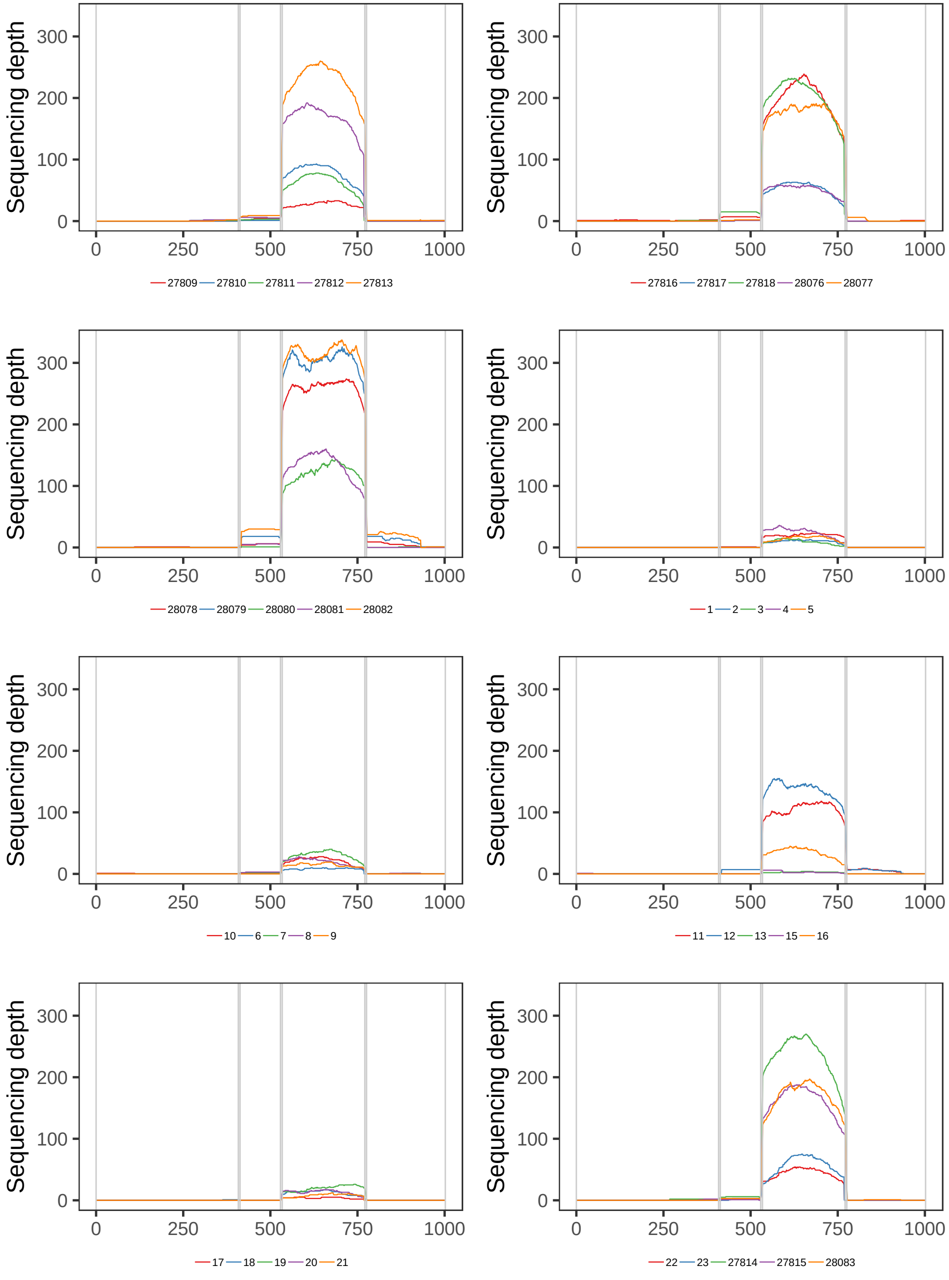

Sequencing depth
0
100
200
300
0
250
500
750
1000
27809 27810 27811 27812 27813
27816 27817 27818 28076 28077
28078 28079 28080 28081 28082
1 2 3 4 5
10 6 7 8 9
11 12 13 15 16
17 18 19 20 21
22 23 27814 27815 28083

EOG531ZDQ

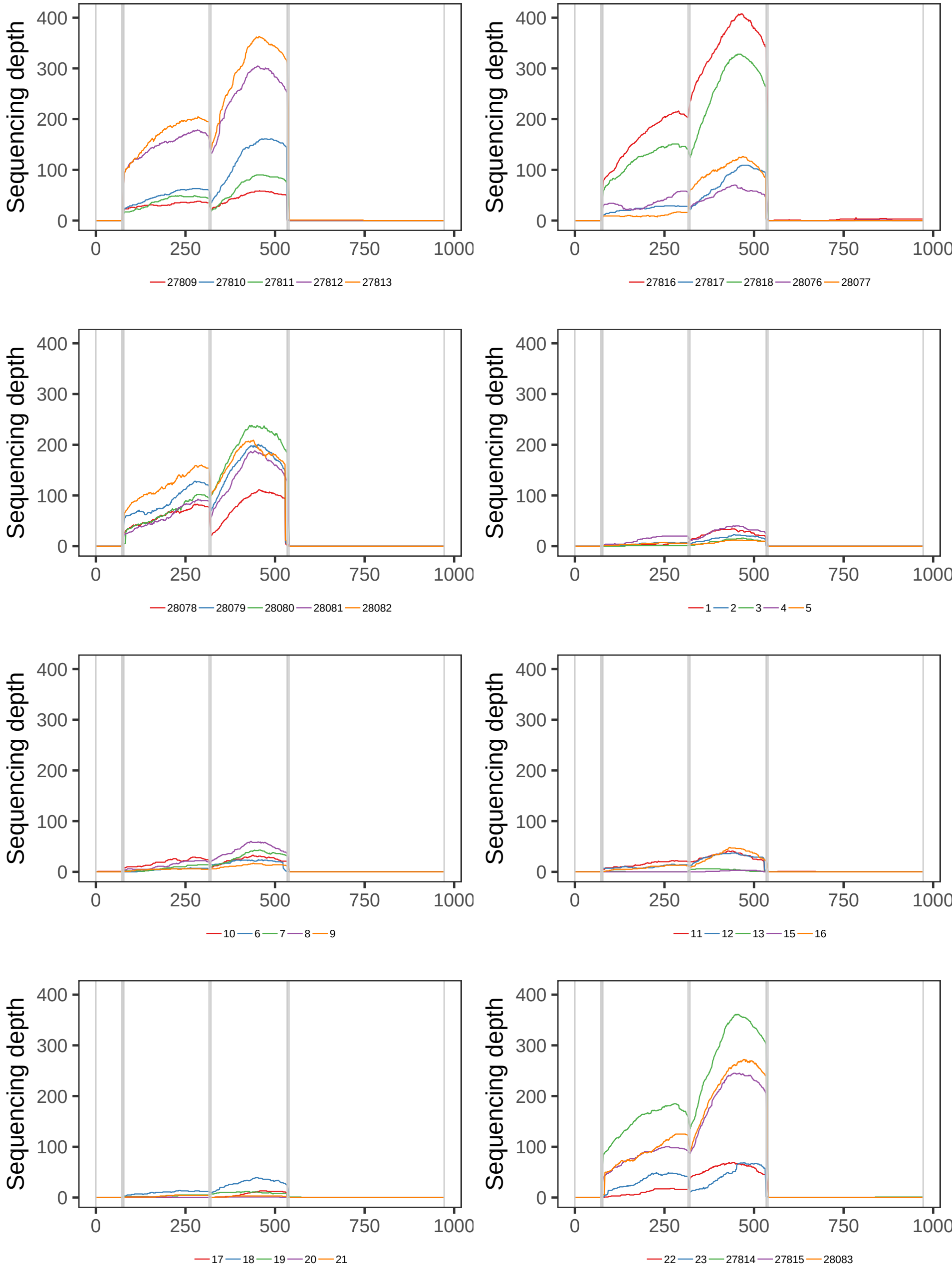
Sequencing depth
0
100
200
300
400
0
250
500
750
1000
27809 27810 27811 27812 27813
27816 27817 27818 28076 28077
28078 28079 28080 28081 28082
1 2 3 4 5
10 6 7 8 9
11 12 13 15 16
17 18 19 20 21
22 23 27814 27815 28083

EOG55DV52

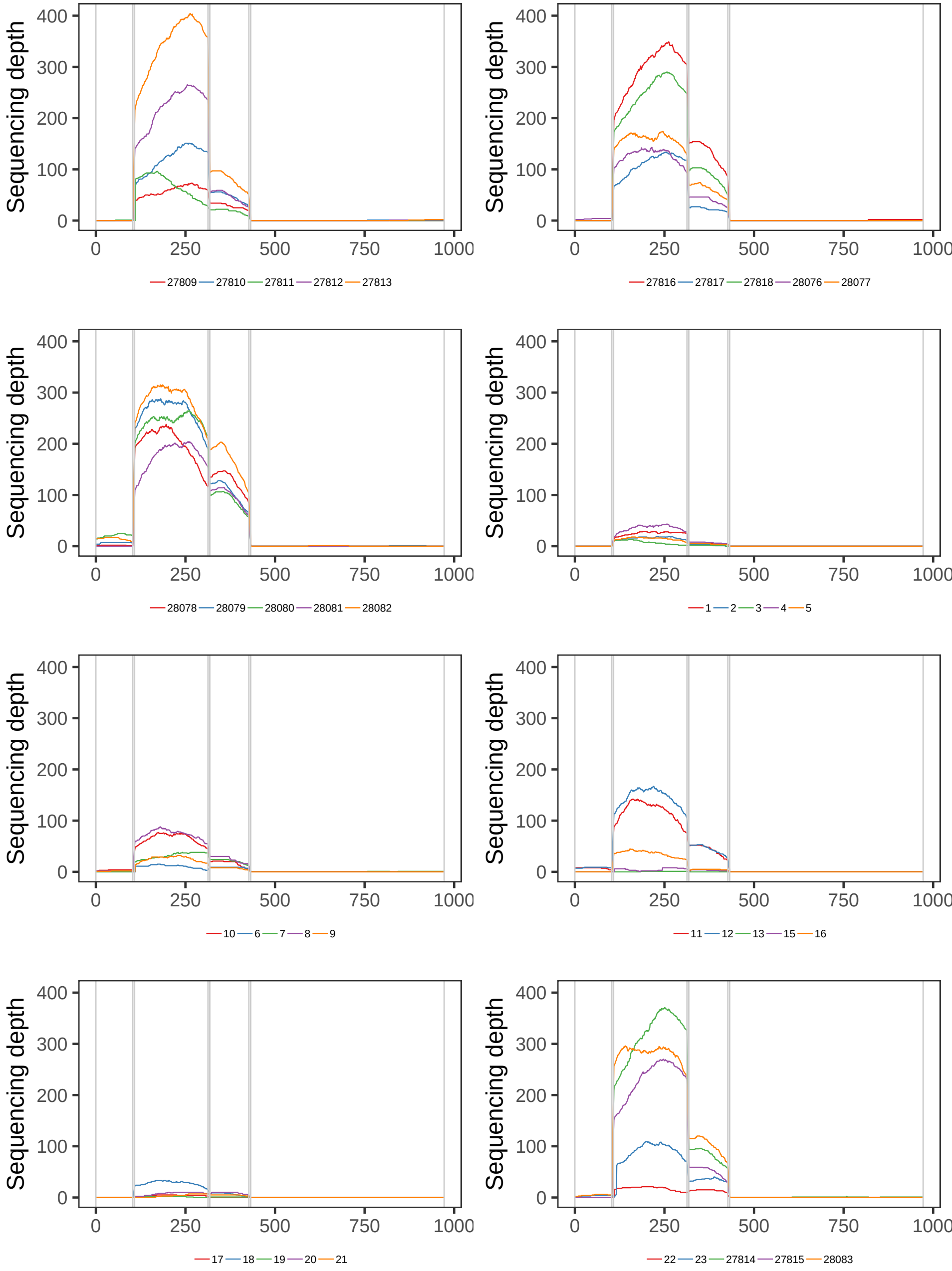

Sequencing depth
0
100
200
300
400
0
250
500
750
1000
27809 27810 27811 27812 27813
27816 27817 27818 28076 28077
28078 28079 28080 28081 28082
1 2 3 4 5
10 6 7 8 9
11 12 13 15 16
17 18 19 20 21
22 23 27814 27815 28083

EOG56DJJW

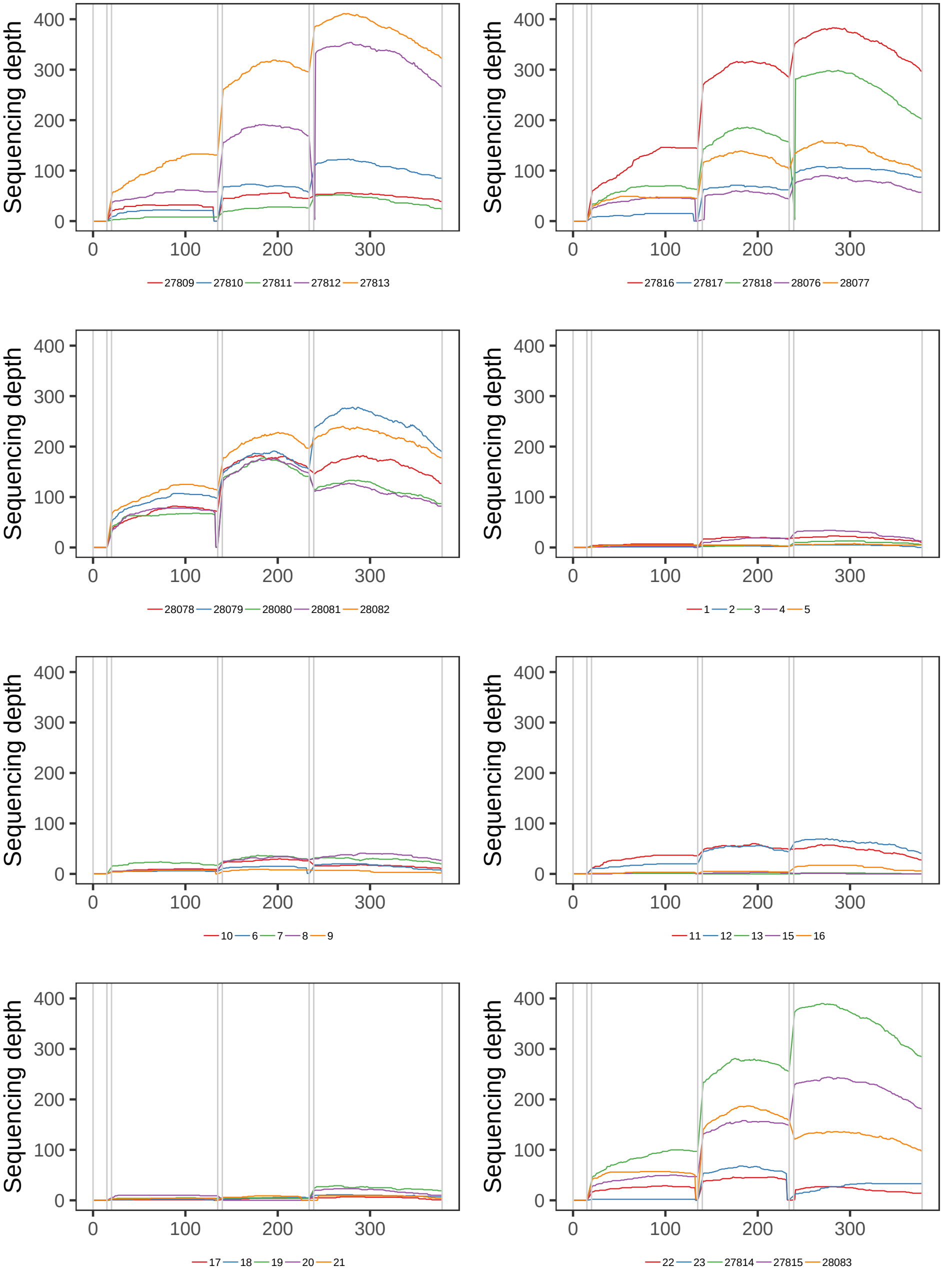
Sequencing depth
0
100
200
300
400
27809 27810 27811 27812 27813
27816 27817 27818 28076 28077
28078 28079 28080 28081 28082
1 2 3 4 5
10 6 7 8 9
11 12 13 15 16
17 18 19 20 21
22 23 27814 27815 28083

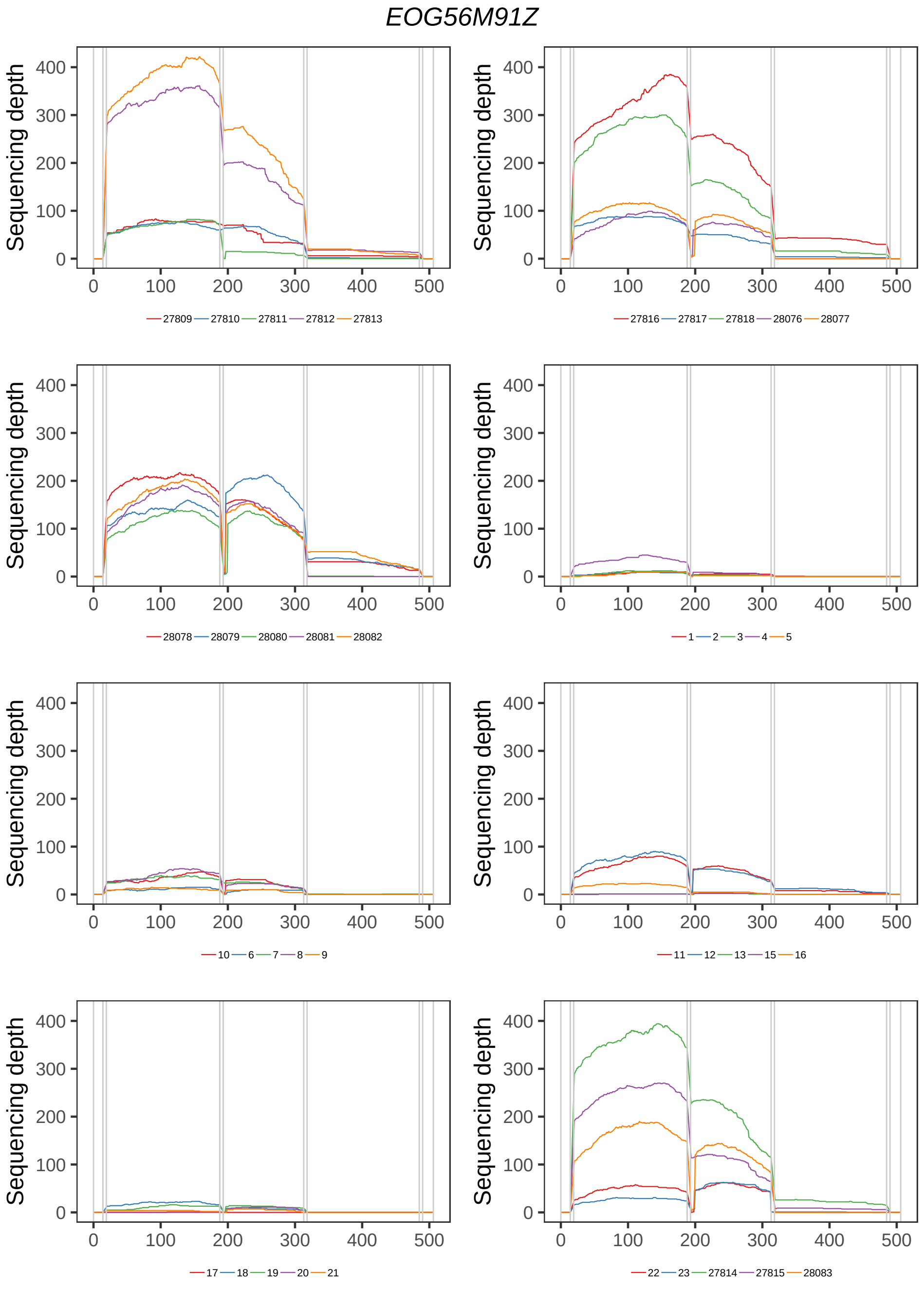
EOG56M91Z
Sequencing depth
27809 27810 27811 27812 27813
27816 27817 27818 28076 28077
28078 28079 28080 28081 28082
1 2 3 4 5
10 6 7 8 9
11 12 13 15 16
17 18 19 20 21
22 23 27814 27815 28083

EOG57M0D9

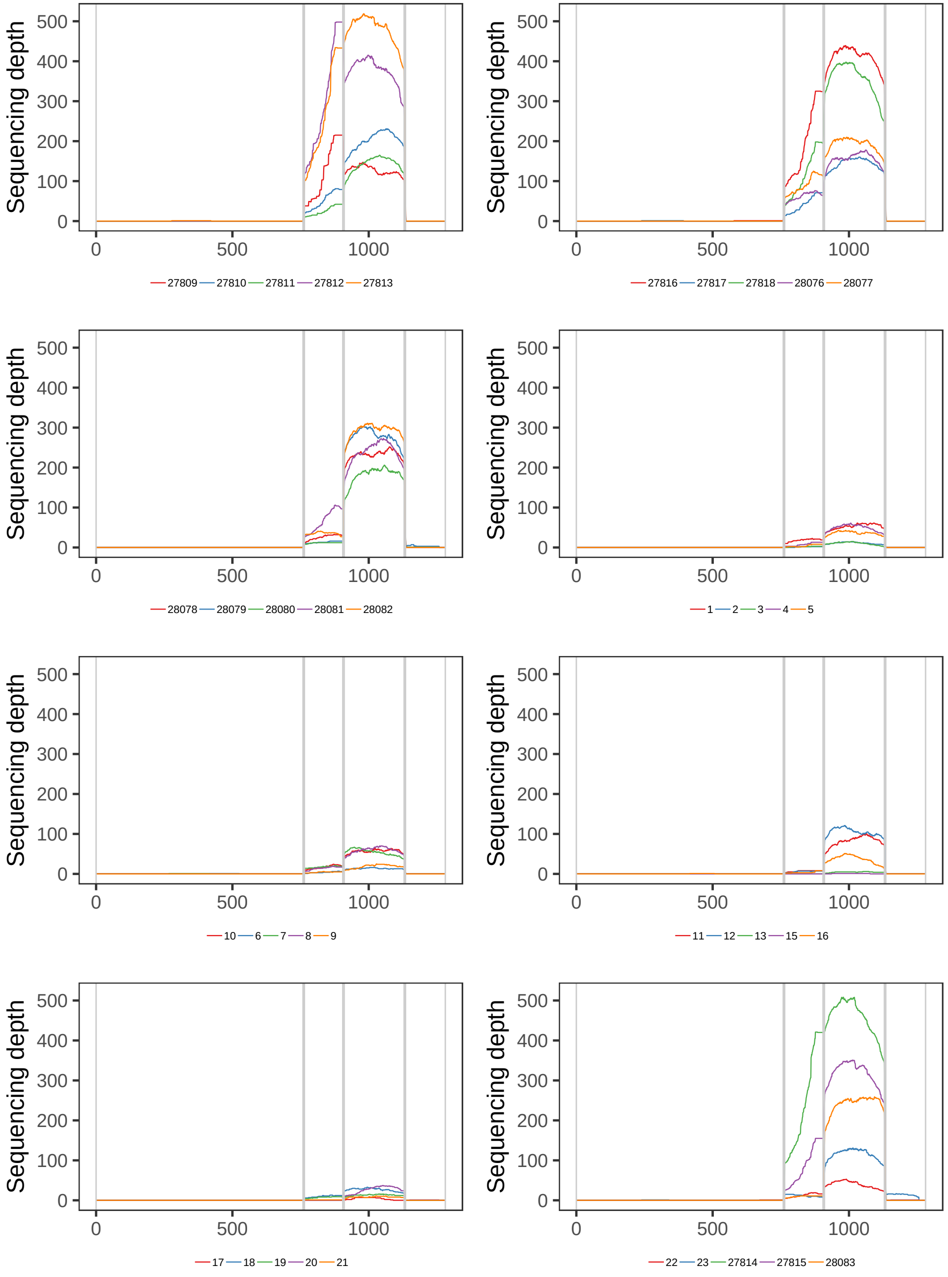
Sequencing depth
0
100
200
300
400
500
0
500
1000
27809 27810 27811 27812 27813
27816 27817 27818 28076 28077
28078 28079 28080 28081 28082
1 2 3 4 5
10 6 7 8 9
11 12 13 15 16
17 18 19 20 21
22 23 27814 27815 28083

EOG598SG9

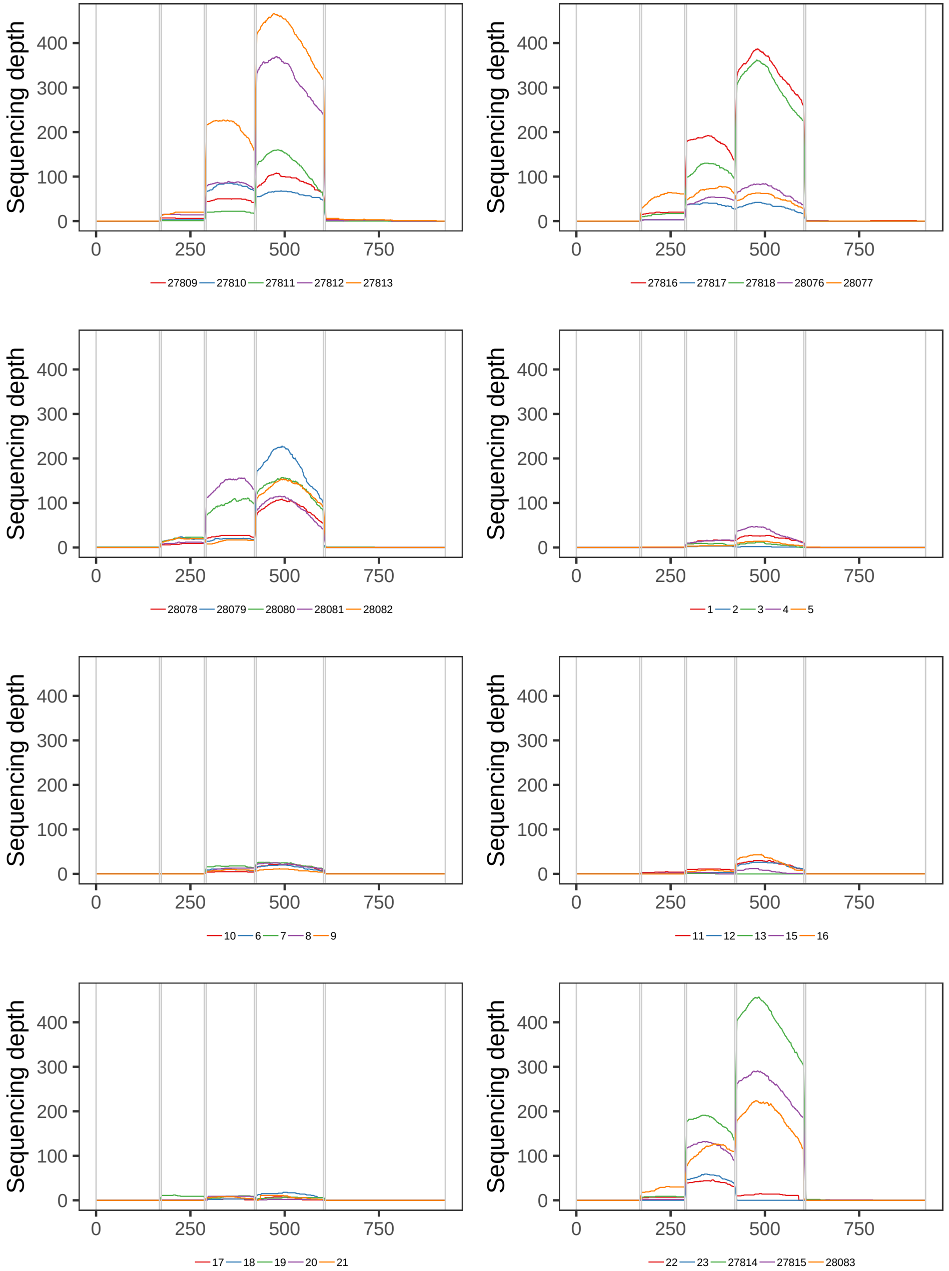

Sequencing depth
400
300
200
100
0
0
250
500
750
27809
27810
27811
27812
27813
27816
27817
27818
28076
28077
28078
28079
28080
28081
28082
1
2
3
4
5
10
6
7
8
9
11
12
13
15
16
17
18
19
20
21
22
23
27814
27815
28083

EOG5CJSZR

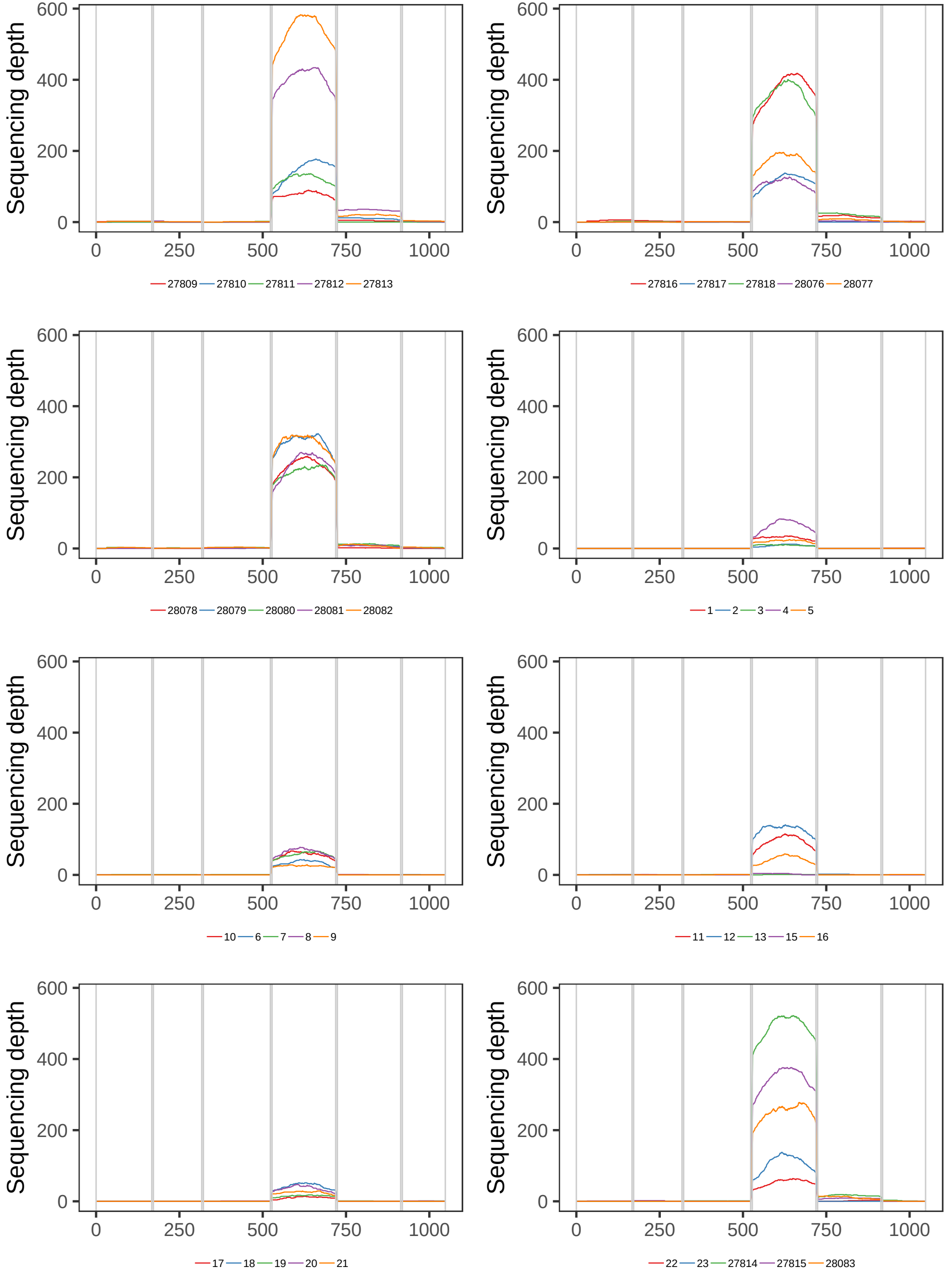
Sequencing depth
0
200
400
600
0
250
500
750
1000
27809 27810 27811 27812 27813
27816 27817 27818 28076 28077
28078 28079 28080 28081 28082
1 2 3 4 5
10 6 7 8 9
11 12 13 15 16
17 18 19 20 21
22 23 27814 27815 28083

EOG5FJ6RK

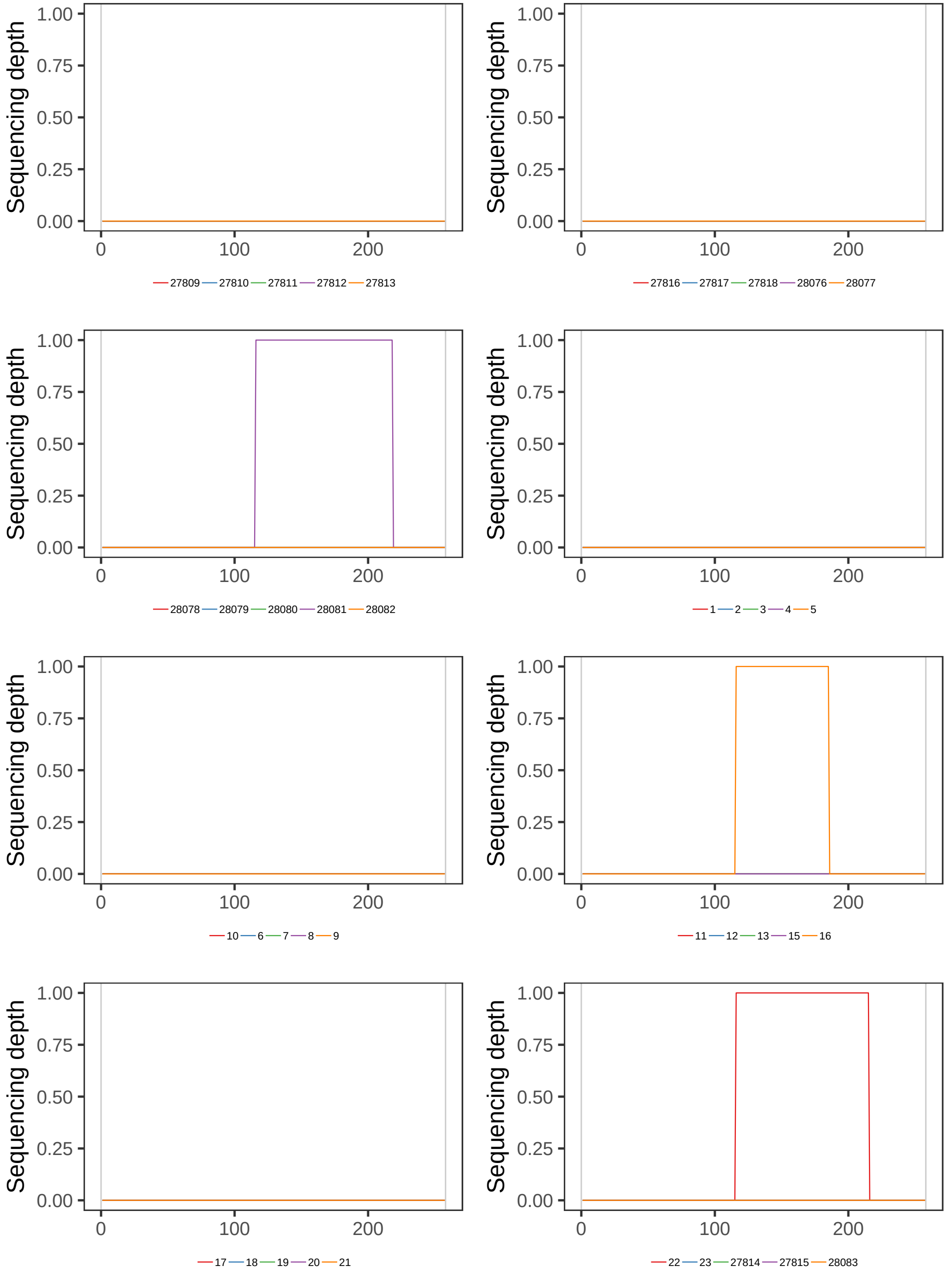
Sequencing depth
1.00
0.75
0.50
0.25
0.00
0
100
200
27809 27810 27811 27812 27813
27816 27817 27818 28076 28077
28078 28079 28080 28081 28082
1 2 3 4 5
10 6 7 8 9
11 12 13 15 16
17 18 19 20 21
22 23 27814 27815 28083

EOG5FTTGQ

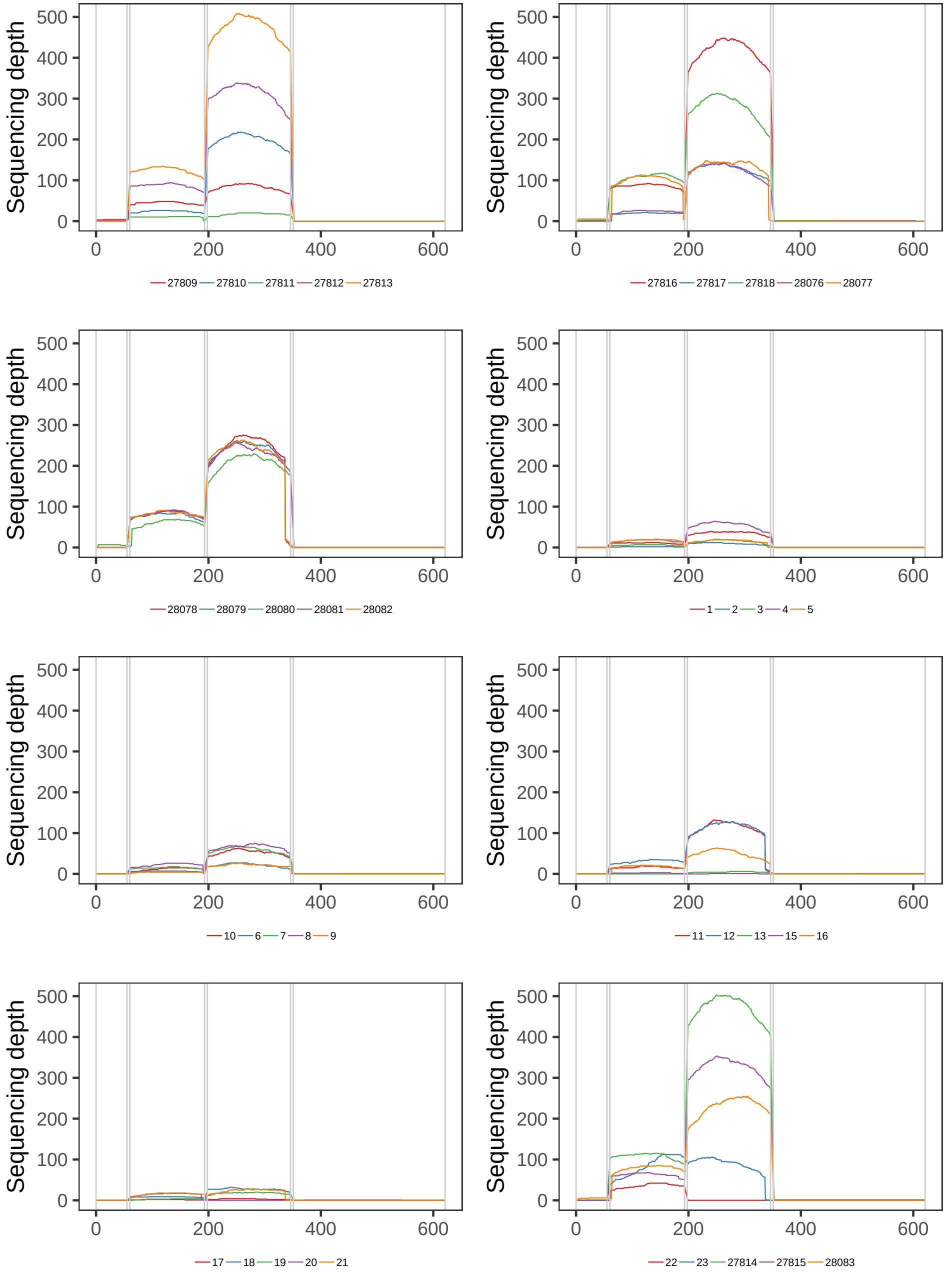
Sequencing depth
0
100
200
300
400
500
0
200
400
600
27809 27810 27811 27812 27813
27816 27817 27818 28076 28077
28078 28079 28080 28081 28082
1 2 3 4 5
10 6 7 8 9
11 12 13 15 16
17 18 19 20 21
22 23 27814 27815 28083

EOG5J6Q6J

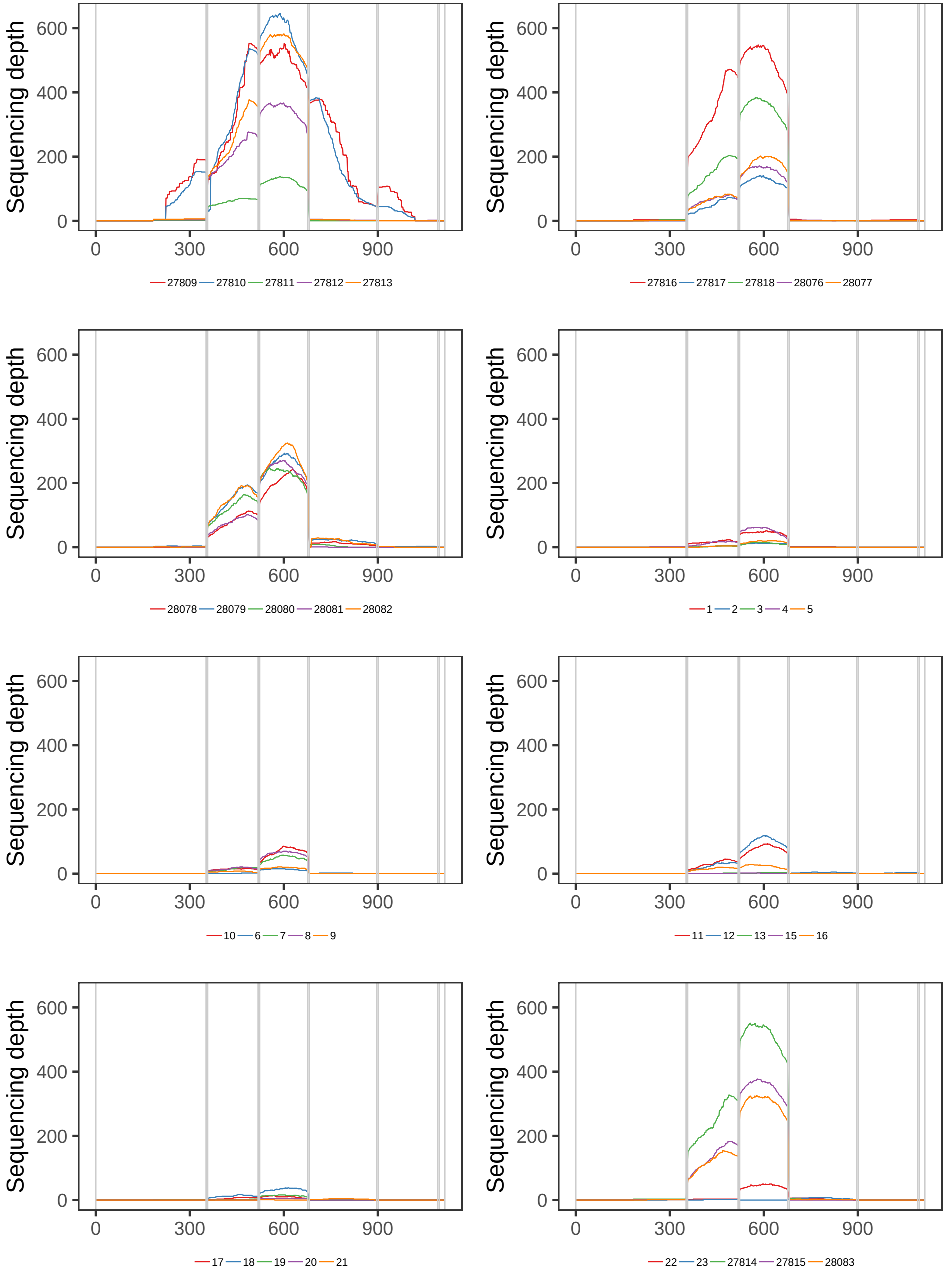

Sequencing depth
600
400
200
0
0
300
600
900
27809 27810 27811 27812 27813
27816 27817 27818 28076 28077
28078 28079 28080 28081 28082
1 2 3 4 5
10 6 7 8 9
11 12 13 15 16
17 18 19 20 21
22 23 27814 27815 28083

EOG5KSN1W

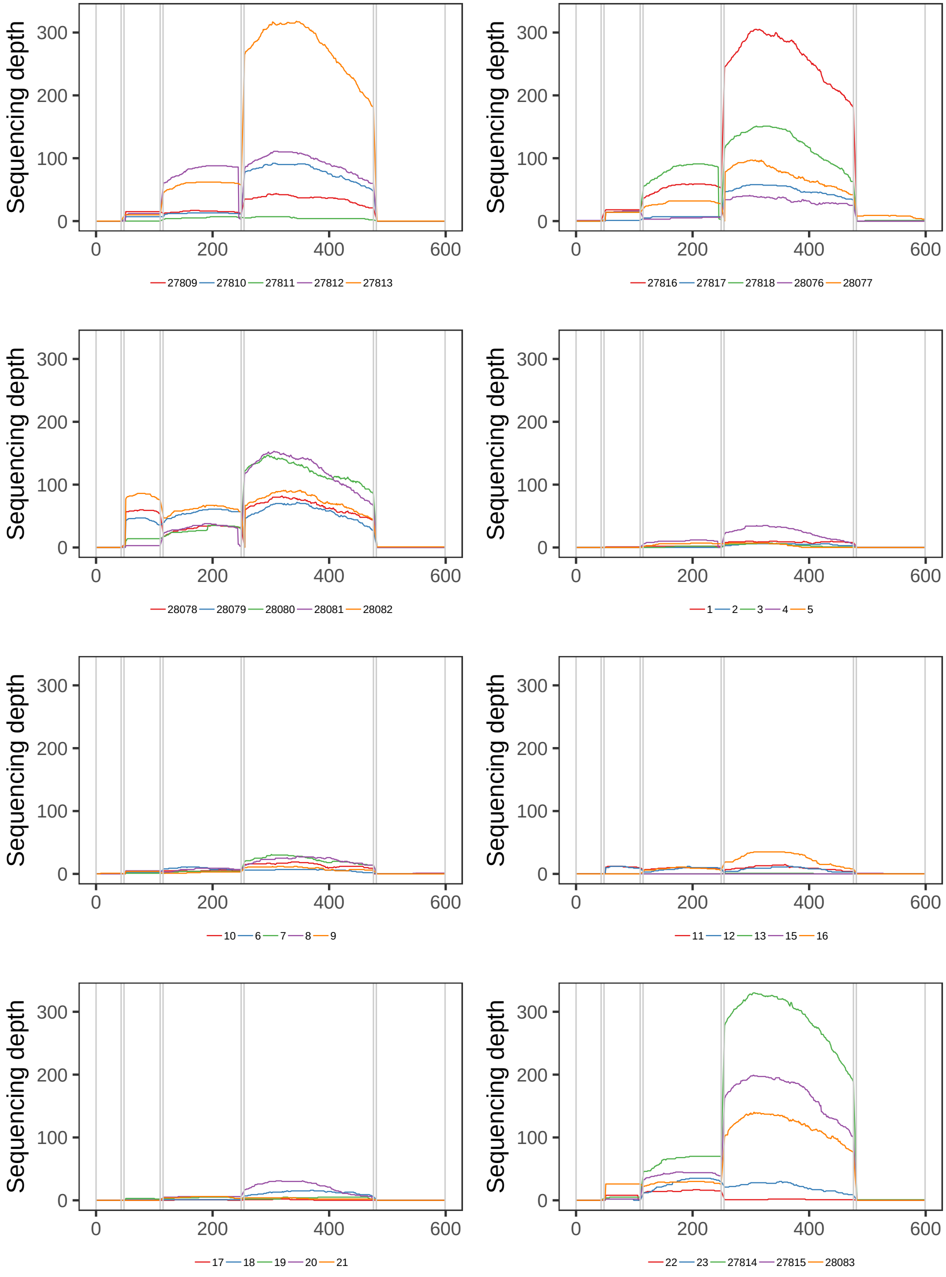
Sequencing depth
0
100
200
300
0
200
400
600
27809 27810 27811 27812 27813
27816 27817 27818 28076 28077
28078 28079 28080 28081 28082
1 2 3 4 5
10 6 7 8 9
11 12 13 15 16
17 18 19 20 21
22 23 27814 27815 28083

EOG5M907H

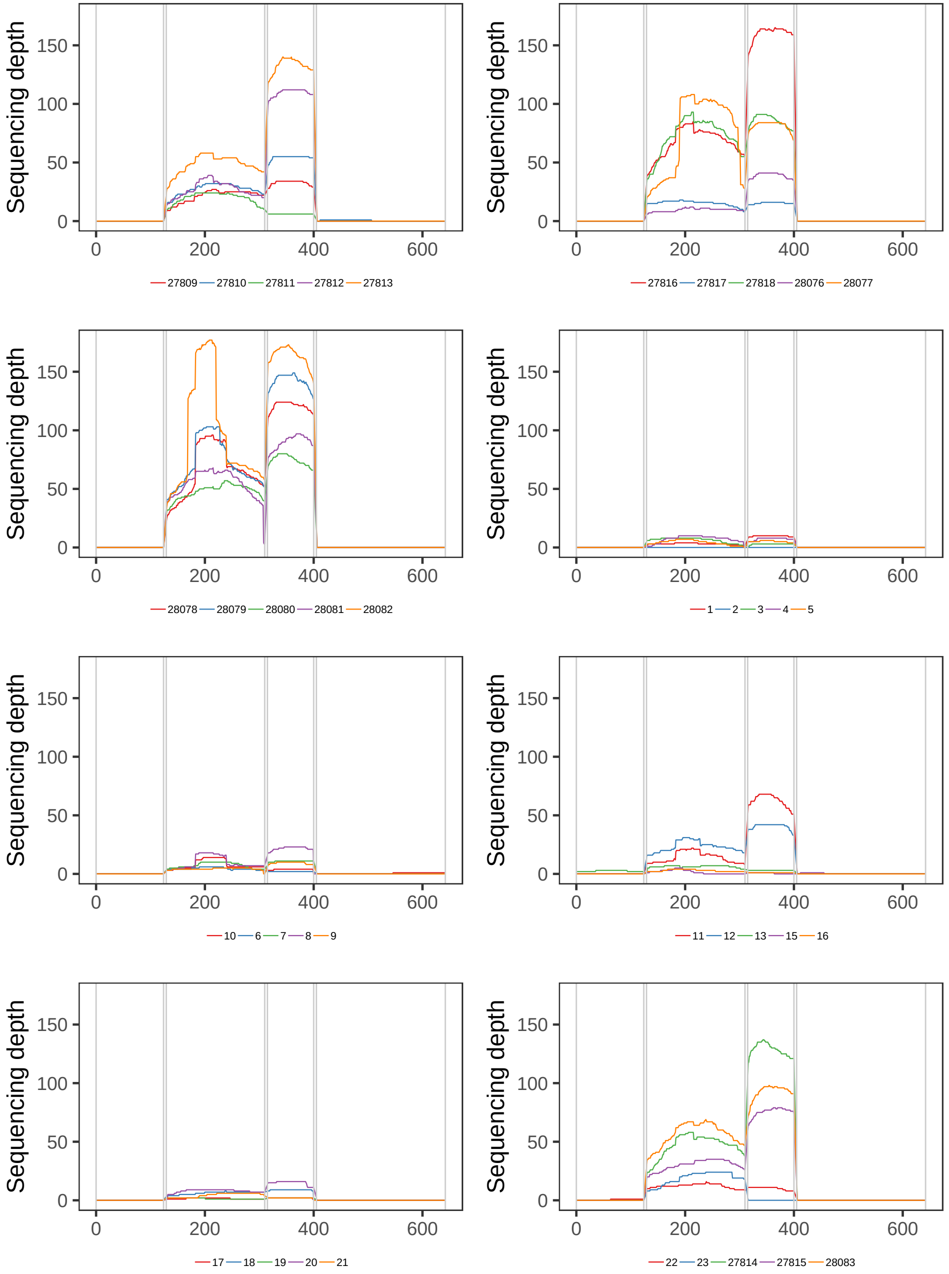
Sequencing depth
0
50
100
150
0
200
400
600
27809 27810 27811 27812 27813
27816 27817 27818 28076 28077
28078 28079 28080 28081 28082
1 2 3 4 5
10 6 7 8 9
11 12 13 15 16
17 18 19 20 21
22 23 27814 27815 28083

EOG5R229W

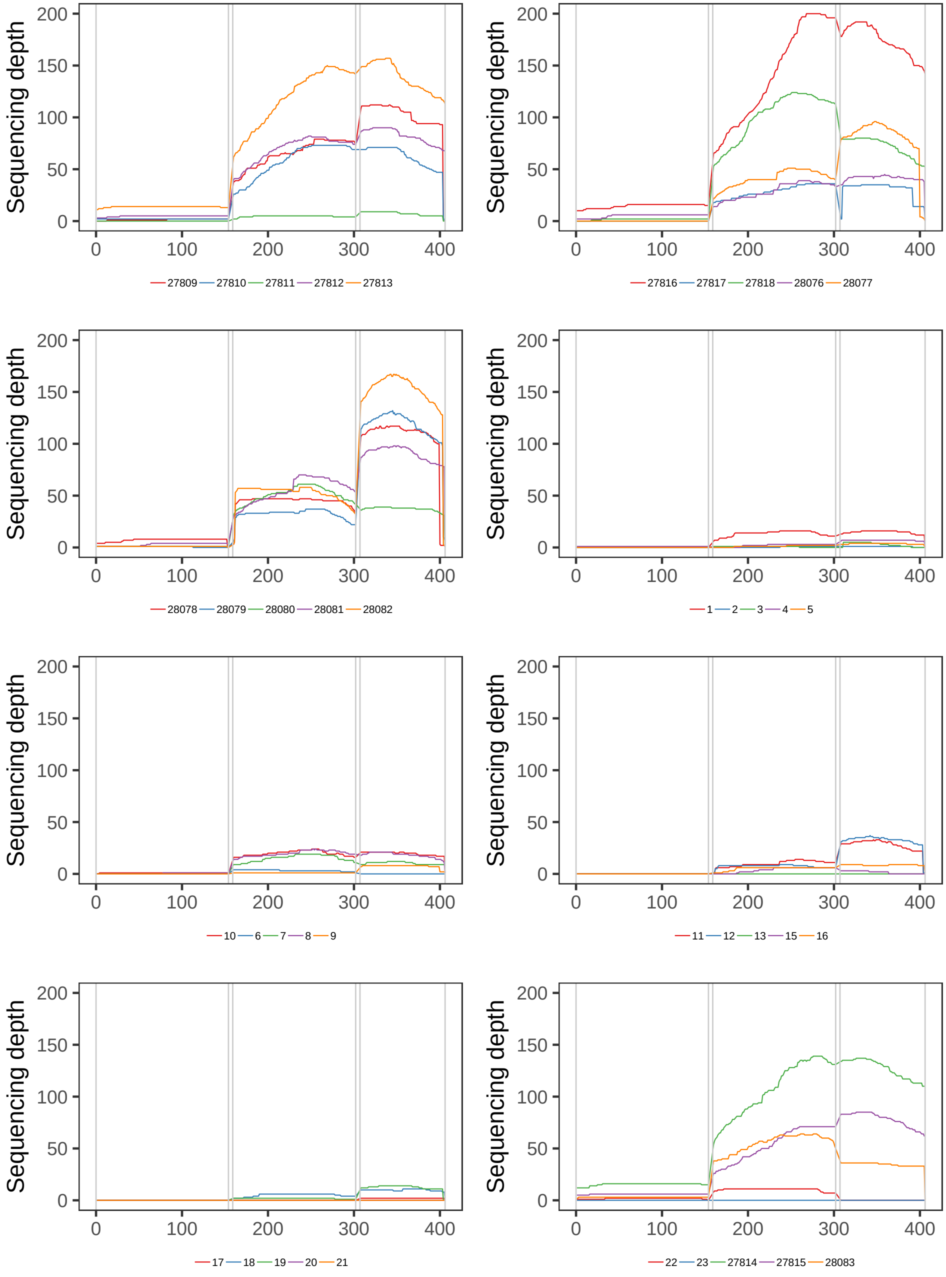
Sequencing depth
0
50
100
150
200
0
100
200
300
400
27809 27810 27811 27812 27813
27816 27817 27818 28076 28077
28078 28079 28080 28081 28082
1 2 3 4 5
10 6 7 8 9
11 12 13 15 16
17 18 19 20 21
22 23 27814 27815 28083

EOG5S7H5R

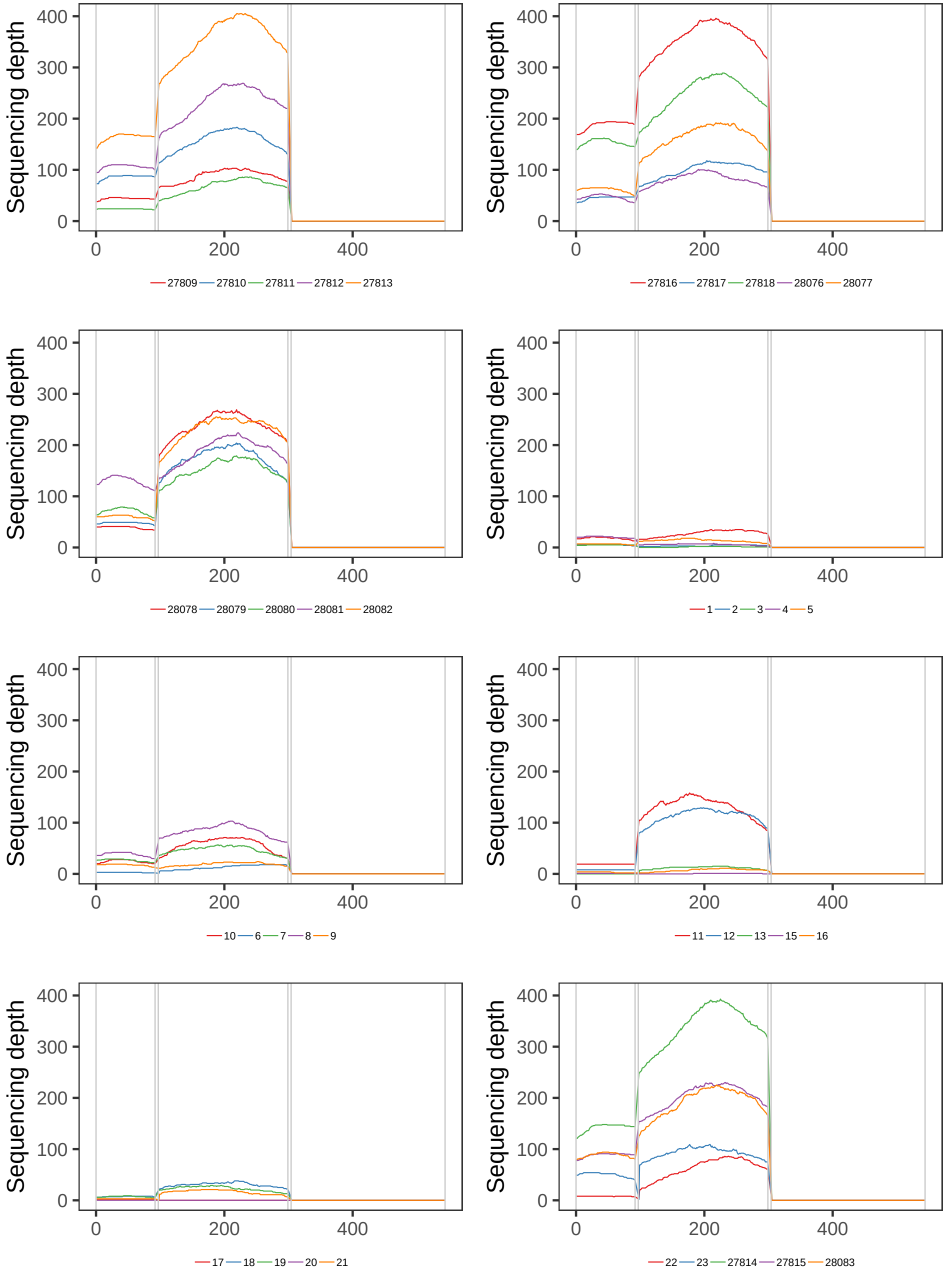
Sequencing depth
0
100
200
300
400
0
200
400
27809 27810 27811 27812 27813
27816 27817 27818 28076 28077
28078 28079 28080 28081 28082
1 2 3 4 5
10 6 7 8 9
11 12 13 15 16
17 18 19 20 21
22 23 27814 27815 28083

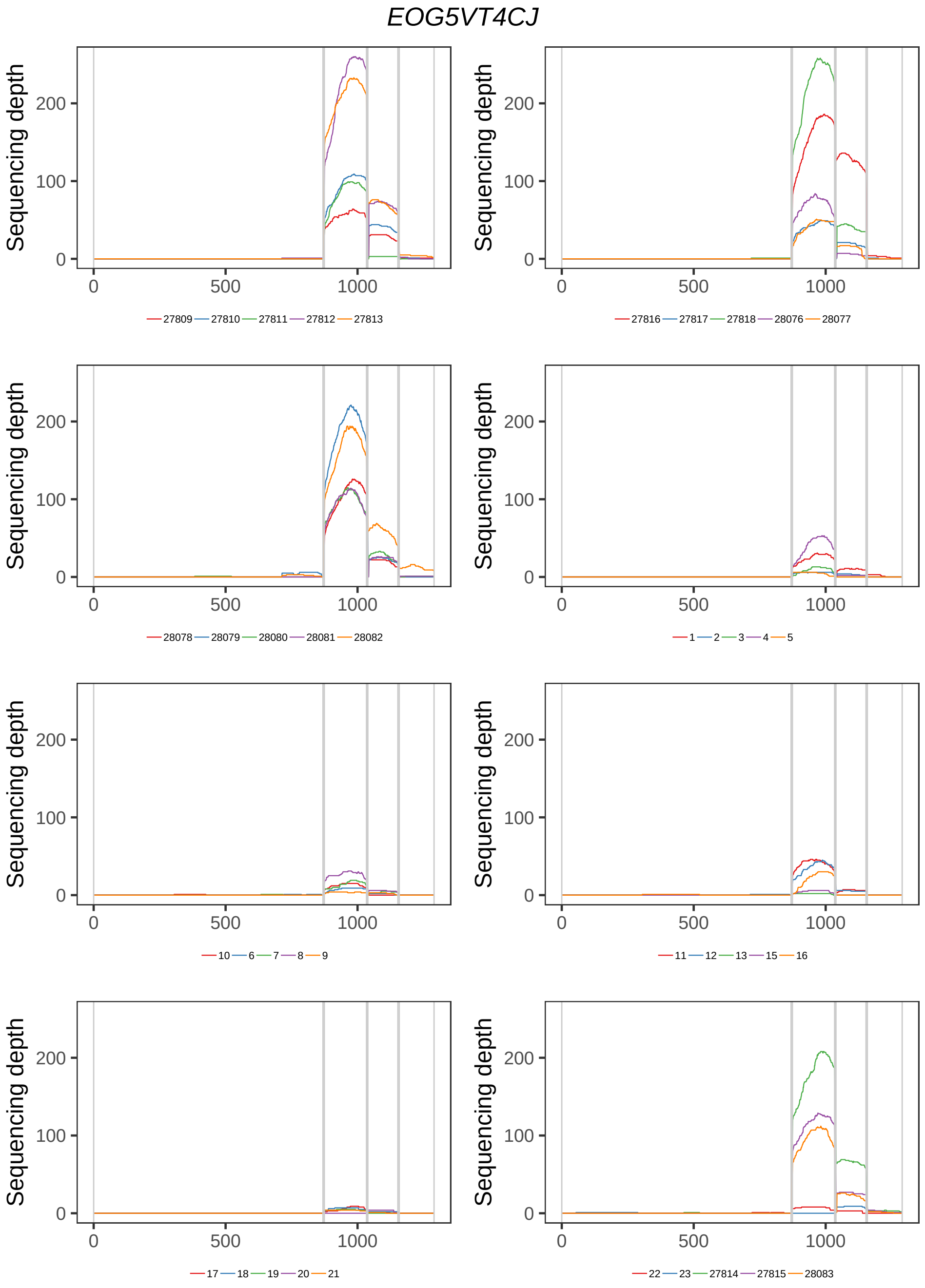
EOG5VT4CJ
Sequencing depth
0
100
200
0
500
1000
27809
27810
27811
27812
27813
27816
27817
27818
28076
28077
28078
28079
28080
28081
28082
1
2
3
4
5
10
6
7
8
9
11
12
13
15
16
17
18
19
20
21
22
23
27814
27815
28083

EOG5Z08P0

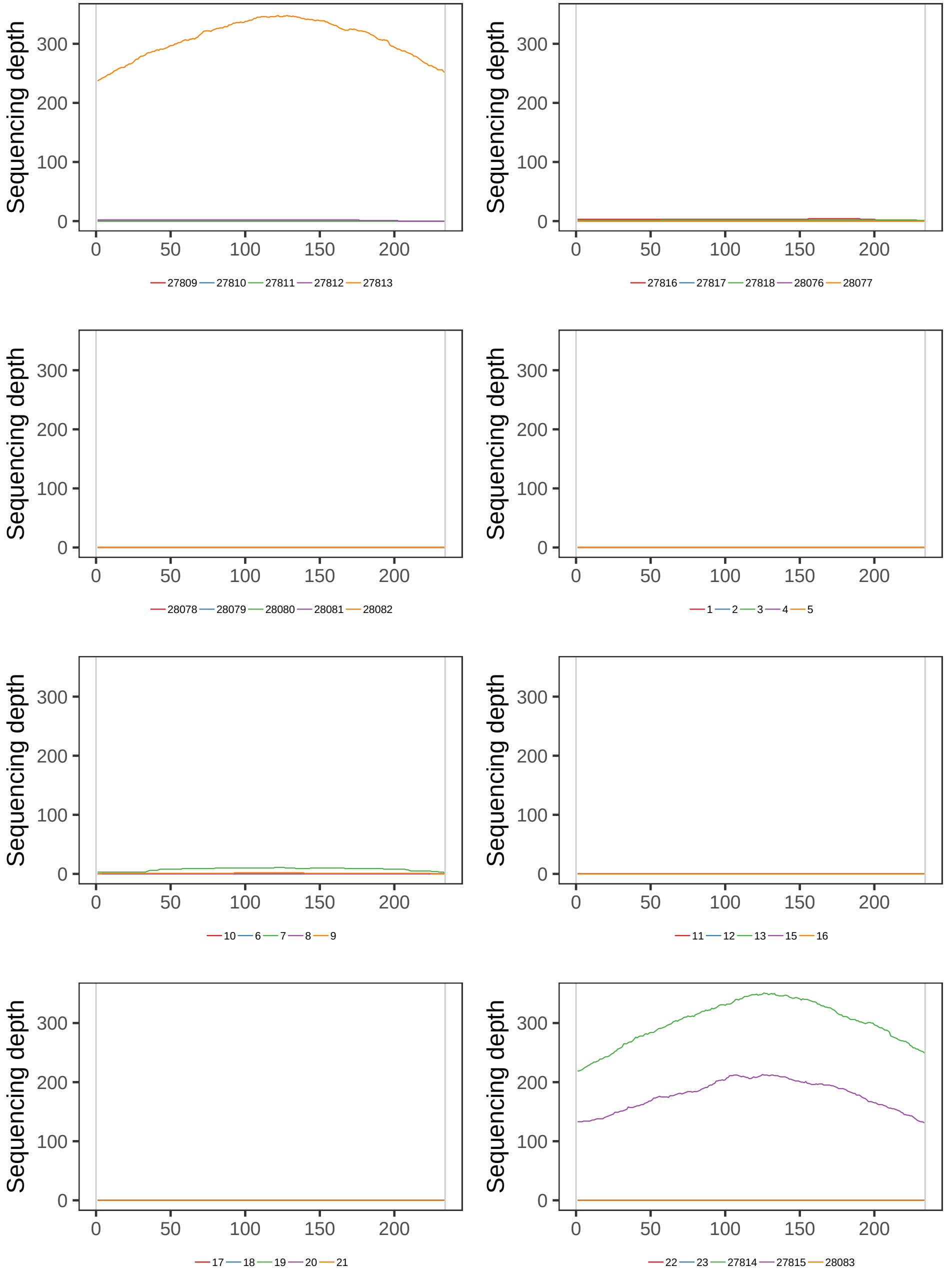
Sequencing depth
0
100
200
300
0
50
100
150
200
27809
27810
27811
27812
27813
27816
27817
27818
28076
28077
28078
28079
28080
28081
28082
1
2
3
4
5
10
6
7
8
9
11
12
13
15
16
17
18
19
20
21
22
23
27814
27815
28083

EOG5ZCRMD

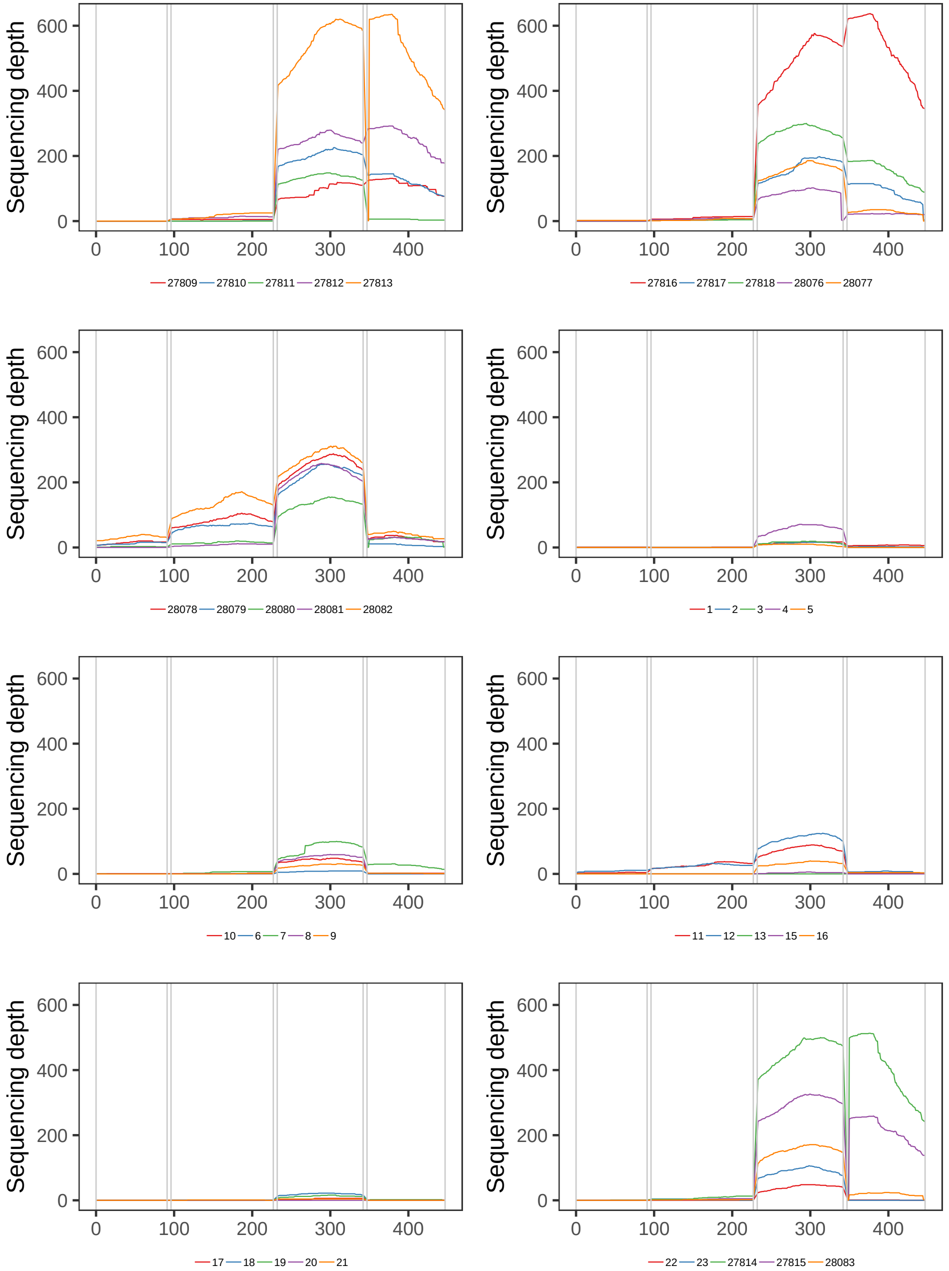

Sequencing depth
0
200
400
600
0
100
200
300
400
27809 27810 27811 27812 27813
27816 27817 27818 28076 28077
28078 28079 28080 28081 28082
1 2 3 4 5
10 6 7 8 9
11 12 13 15 16
17 18 19 20 21
22 23 27814 27815 28083

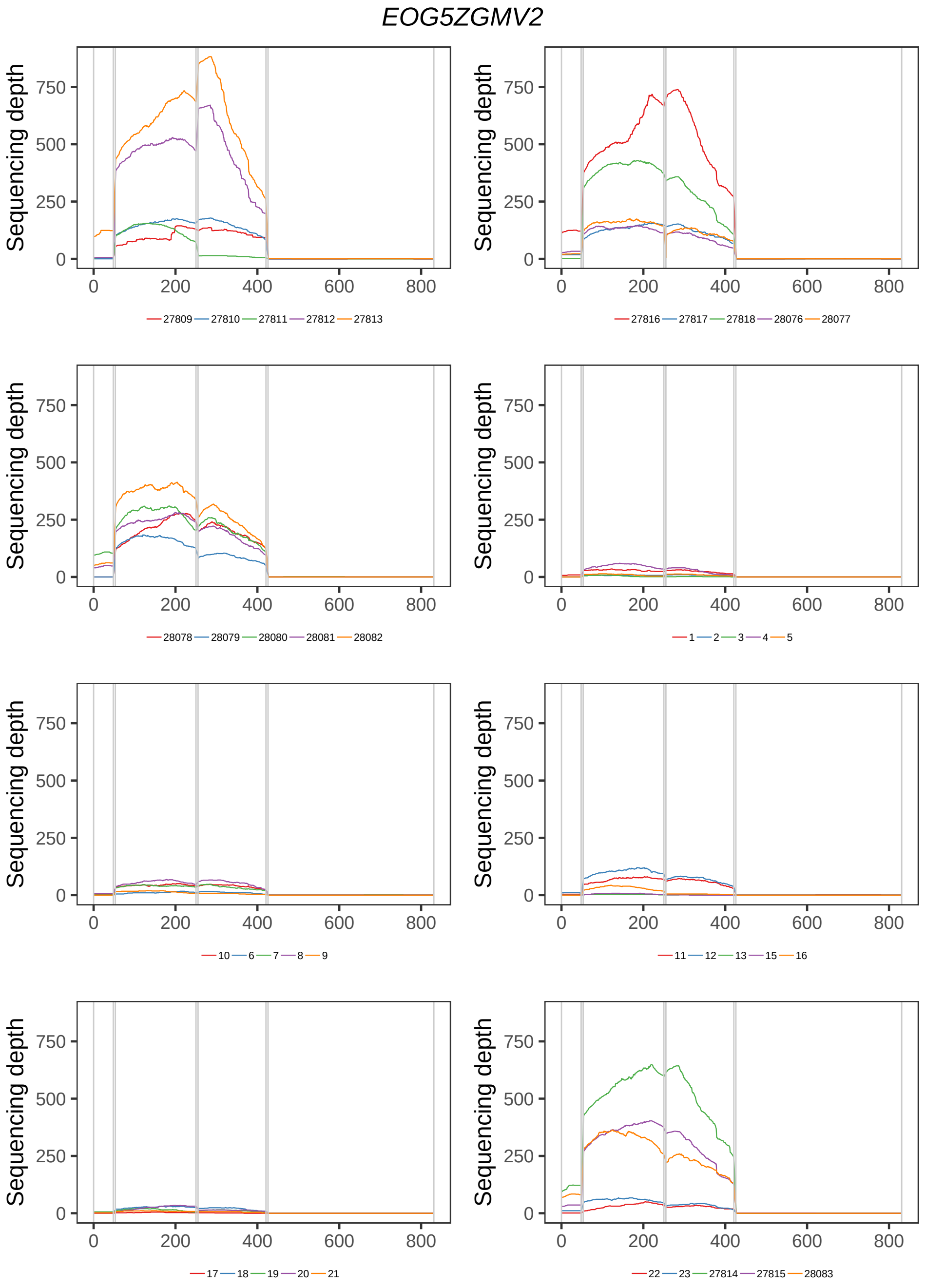
EOG5ZGMV2
Sequencing depth
0
250
500
750
0
200
400
600
800
27809 27810 27811 27812 27813
27816 27817 27818 28076 28077
28078 28079 28080 28081 28082
1 2 3 4 5
10 6 7 8 9
11 12 13 15 16
17 18 19 20 21
22 23 27814 27815 28083

EOG54MW8D

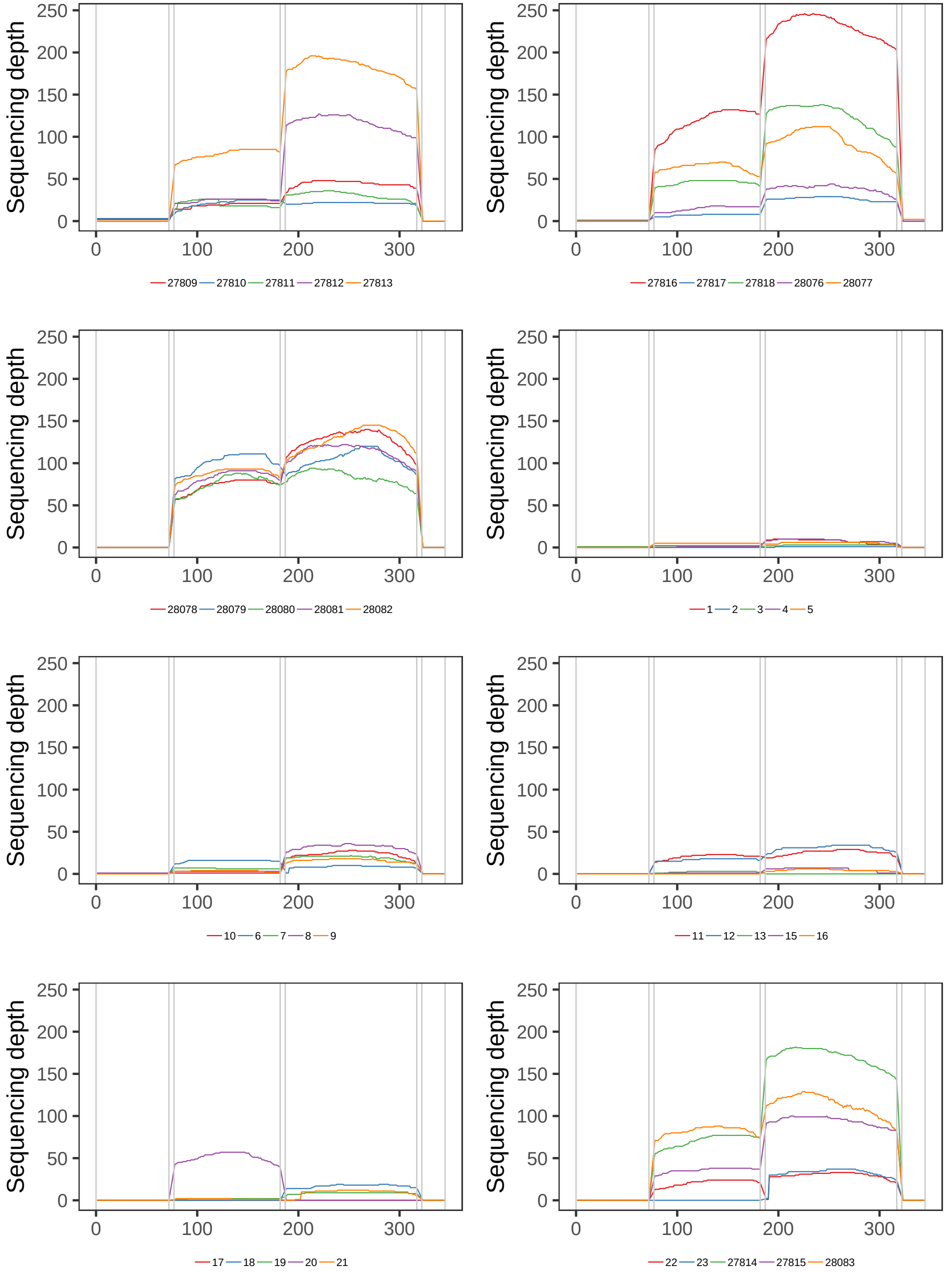
Sequencing depth
0
50
100
150
200
250
0
100
200
300
27809 27810 27811 27812 27813
27816 27817 27818 28076 28077
28078 28079 28080 28081 28082
1 2 3 4 5
10 6 7 8 9
11 12 13 15 16
17 18 19 20 21
22 23 27814 27815 28083

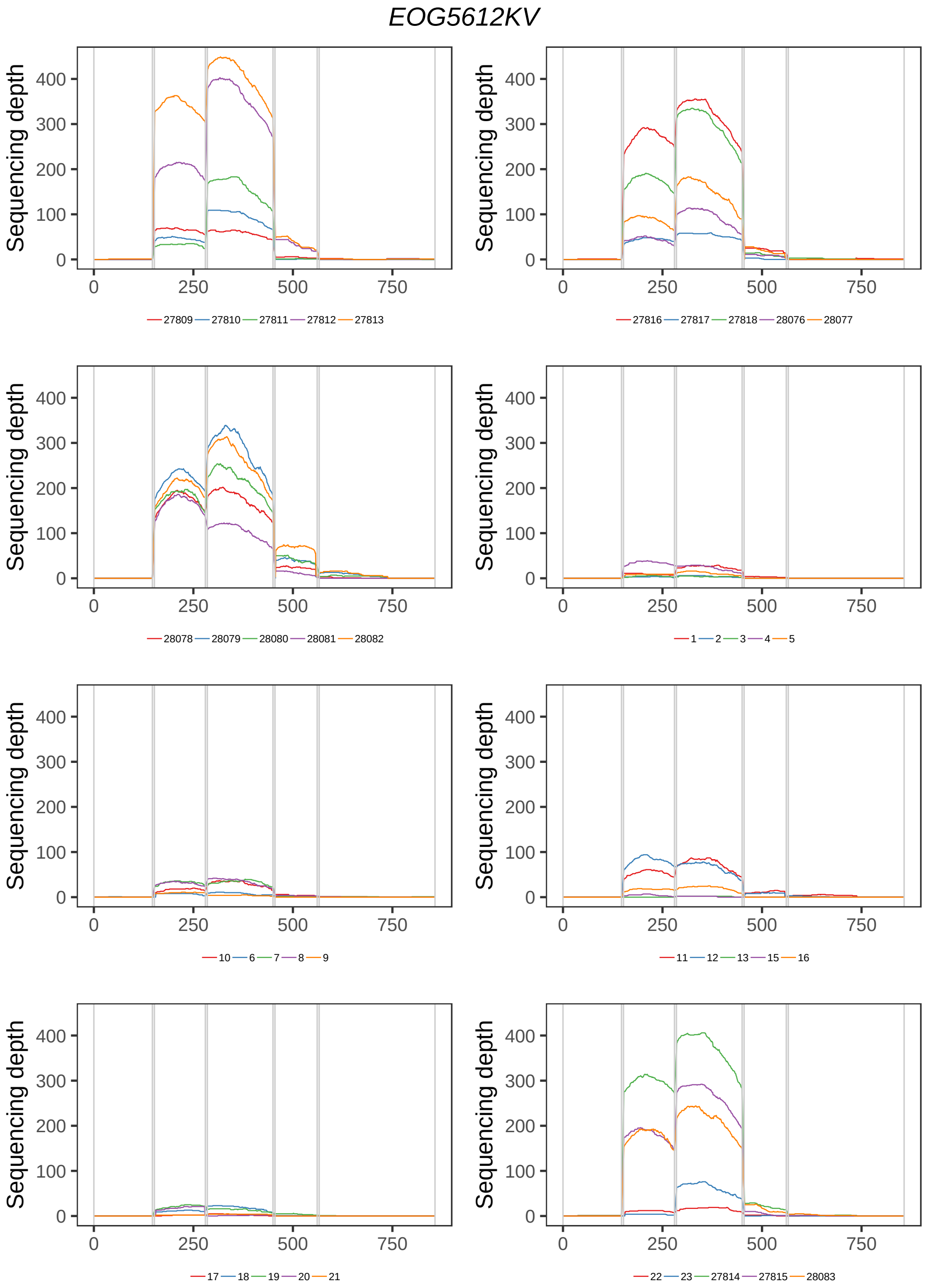
EOG5612KV
Sequencing depth
0
100
200
300
400
0
250
500
750
27809 27810 27811 27812 27813
27816 27817 27818 28076 28077
28078 28079 28080 28081 28082
1 2 3 4 5
10 6 7 8 9
11 12 13 15 16
17 18 19 20 21
22 23 27814 27815 28083

EOG58SF9H

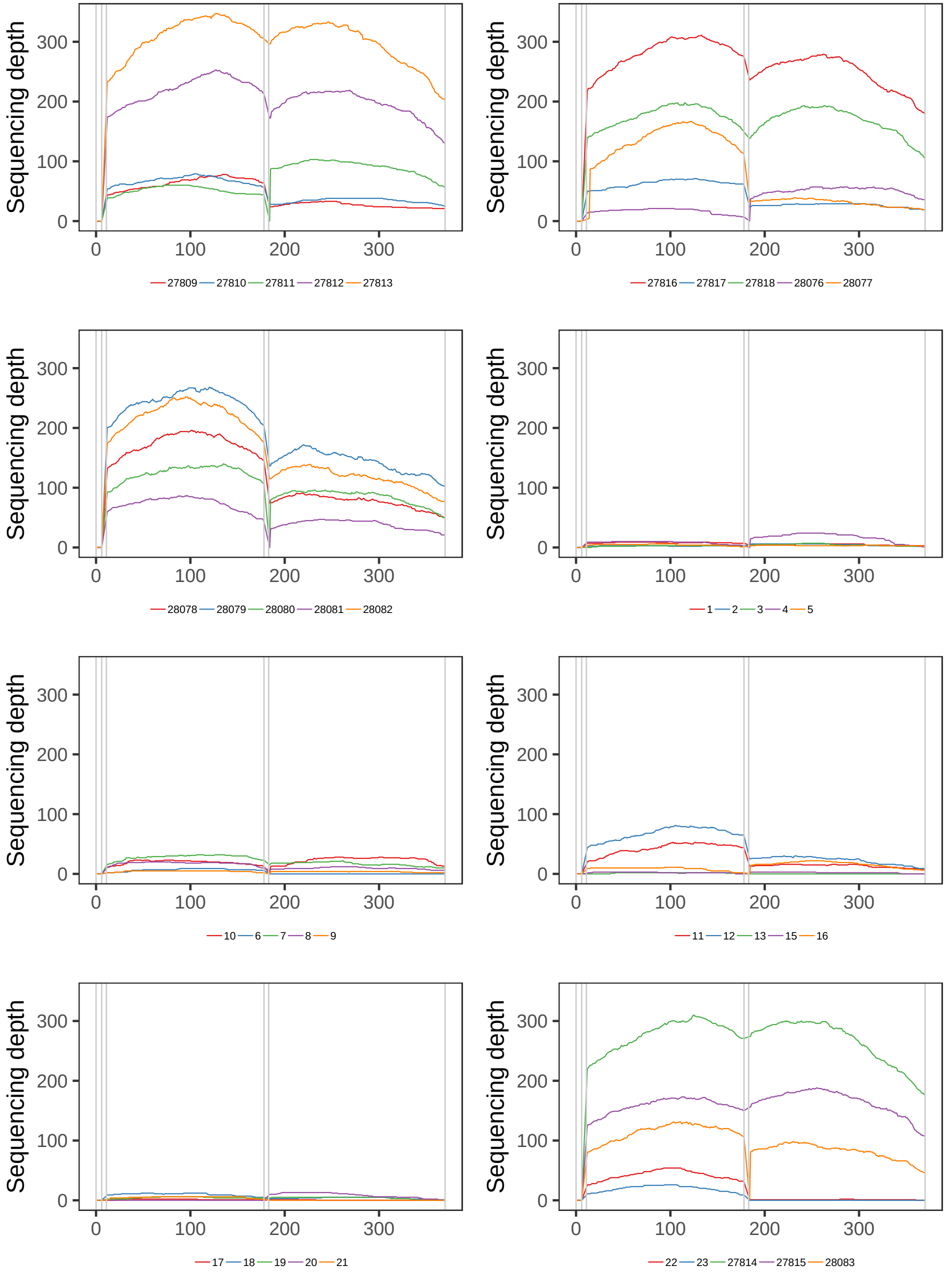

Sequencing depth
27809 27810 27811 27812 27813
27816 27817 27818 28076 28077
28078 28079 28080 28081 28082
1 2 3 4 5
10 6 7 8 9
11 12 13 15 16
17 18 19 20 21
22 23 27814 27815 28083

EOG5DR7TM

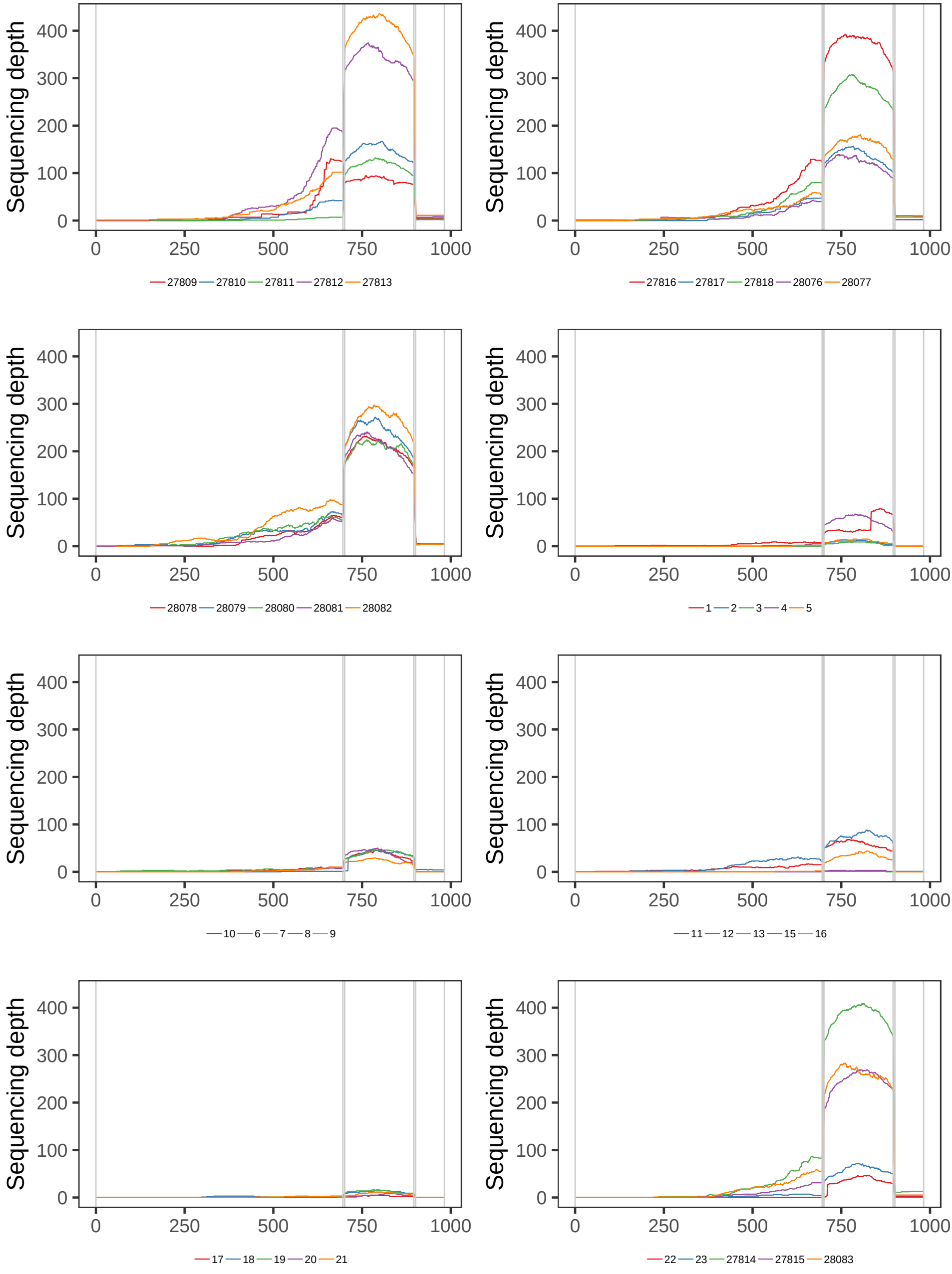

Sequencing depth
0
100
200
300
400
0
250
500
750
1000
27809 27810 27811 27812 27813
27816 27817 27818 28076 28077
28078 28079 28080 28081 28082
1 2 3 4 5
10 6 7 8 9
11 12 13 15 16
17 18 19 20 21
22 23 27814 27815 28083

EOG5G79DN

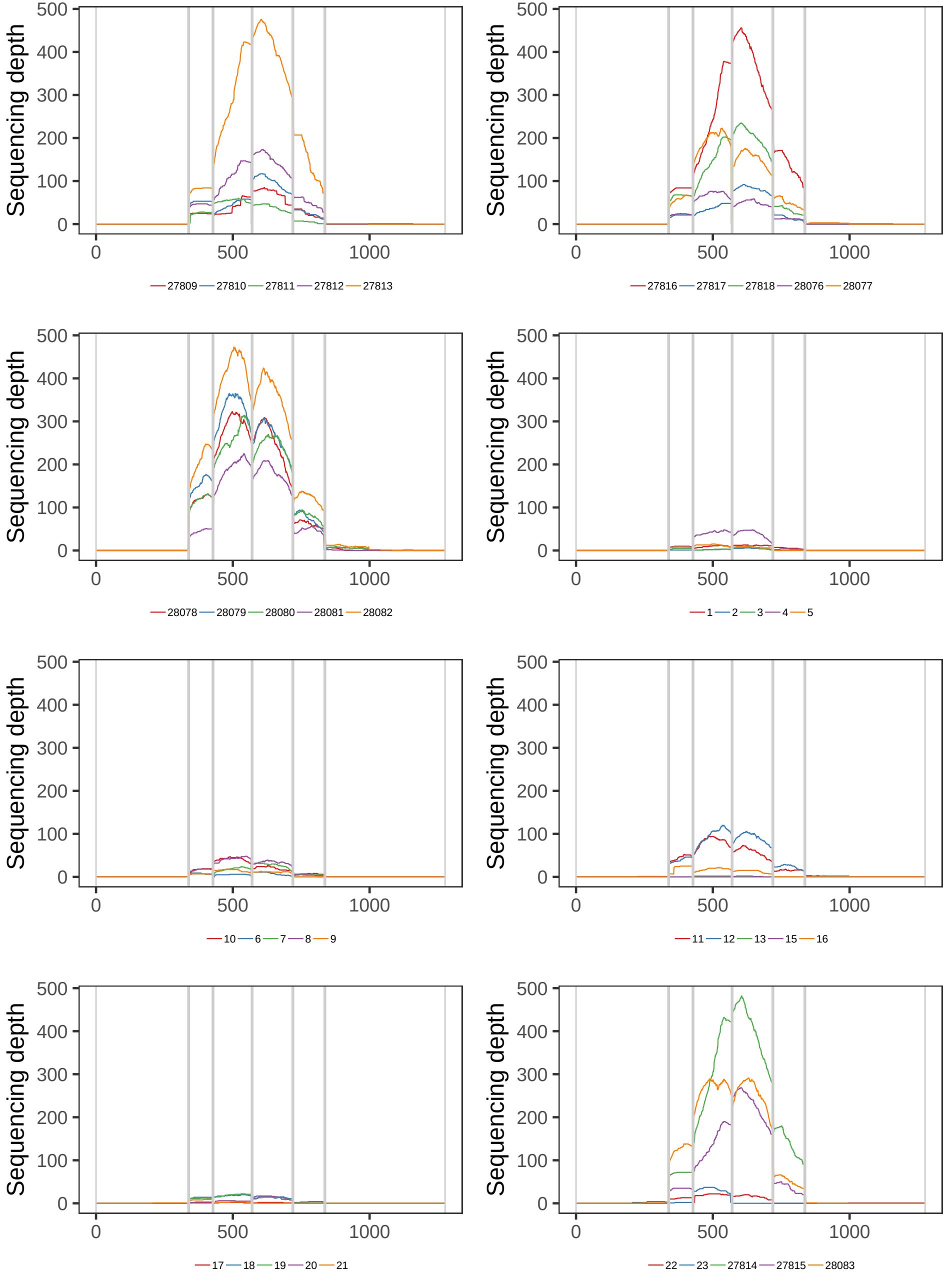
Sequencing depth
0
100
200
300
400
500
0
500
1000
27809 27810 27811 27812 27813
27816 27817 27818 28076 28077
28078 28079 28080 28081 28082
1 2 3 4 5
10 6 7 8 9
11 12 13 15 16
17 18 19 20 21
22 23 27814 27815 28083

EOG5J0ZR8

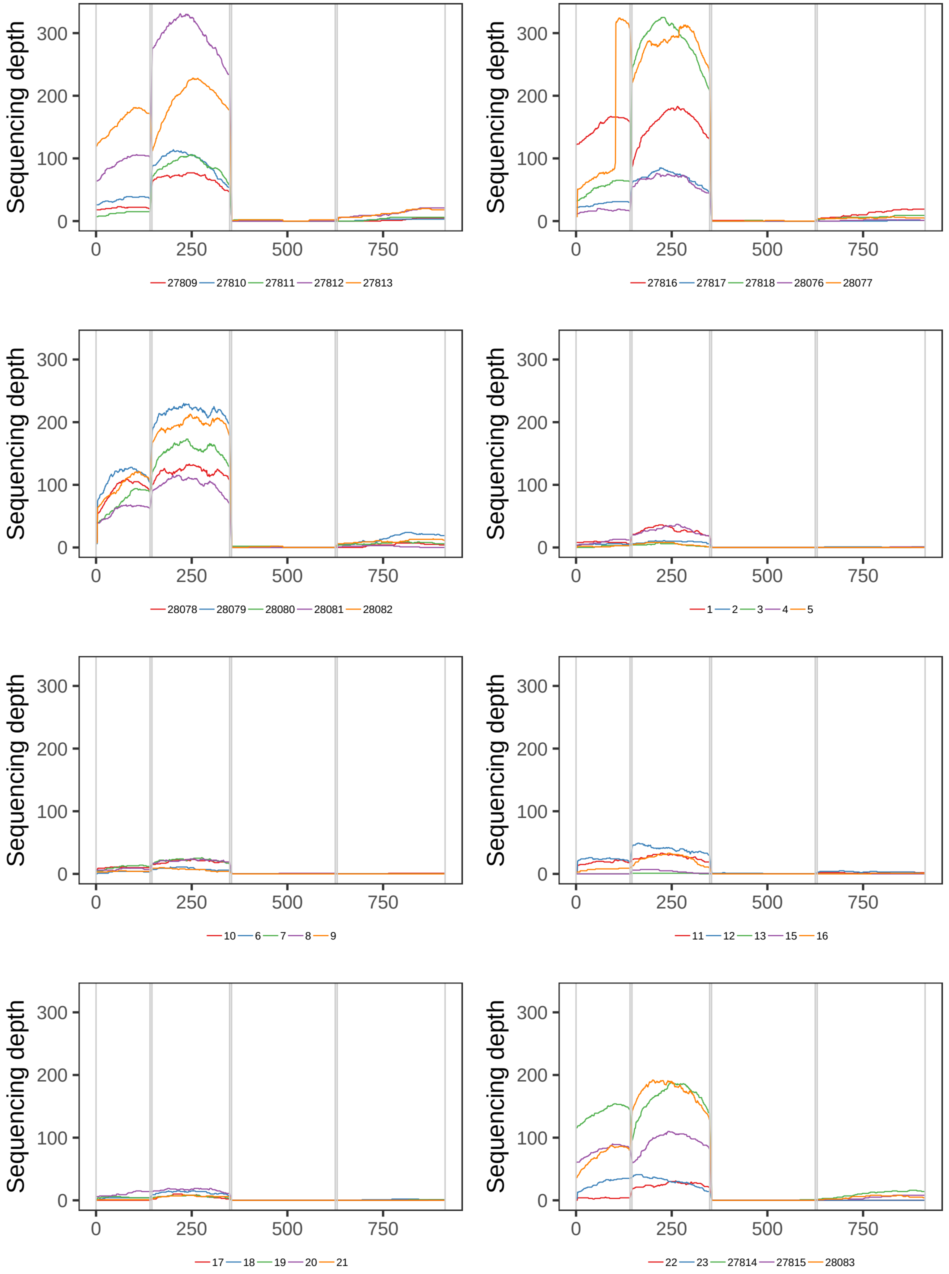

Sequencing depth
0
100
200
300
0
250
500
750
27809 27810 27811 27812 27813
27816 27817 27818 28076 28077
28078 28079 28080 28081 28082
1 2 3 4 5
10 6 7 8 9
11 12 13 15 16
17 18 19 20 21
22 23 27814 27815 28083

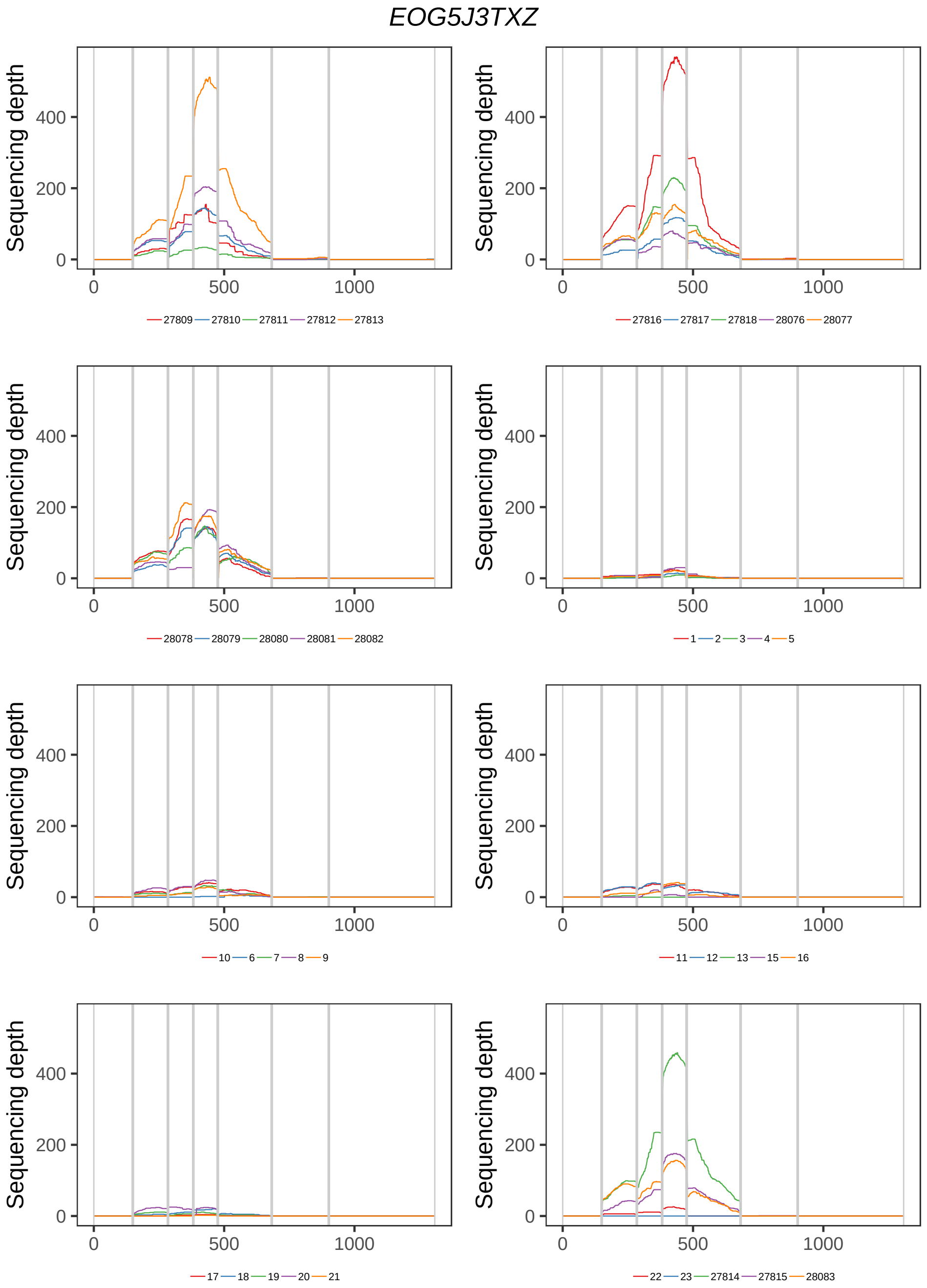
EOG5J3TXZ
Sequencing depth
0
200
400
0
500
1000
27809
27810
27811
27812
27813
27816
27817
27818
28076
28077
28078
28079
28080
28081
28082
1
2
3
4
5
10
6
7
8
9
11
12
13
15
16
17
18
19
20
21
22
23
27814
27815
28083

EOG5J9KGB

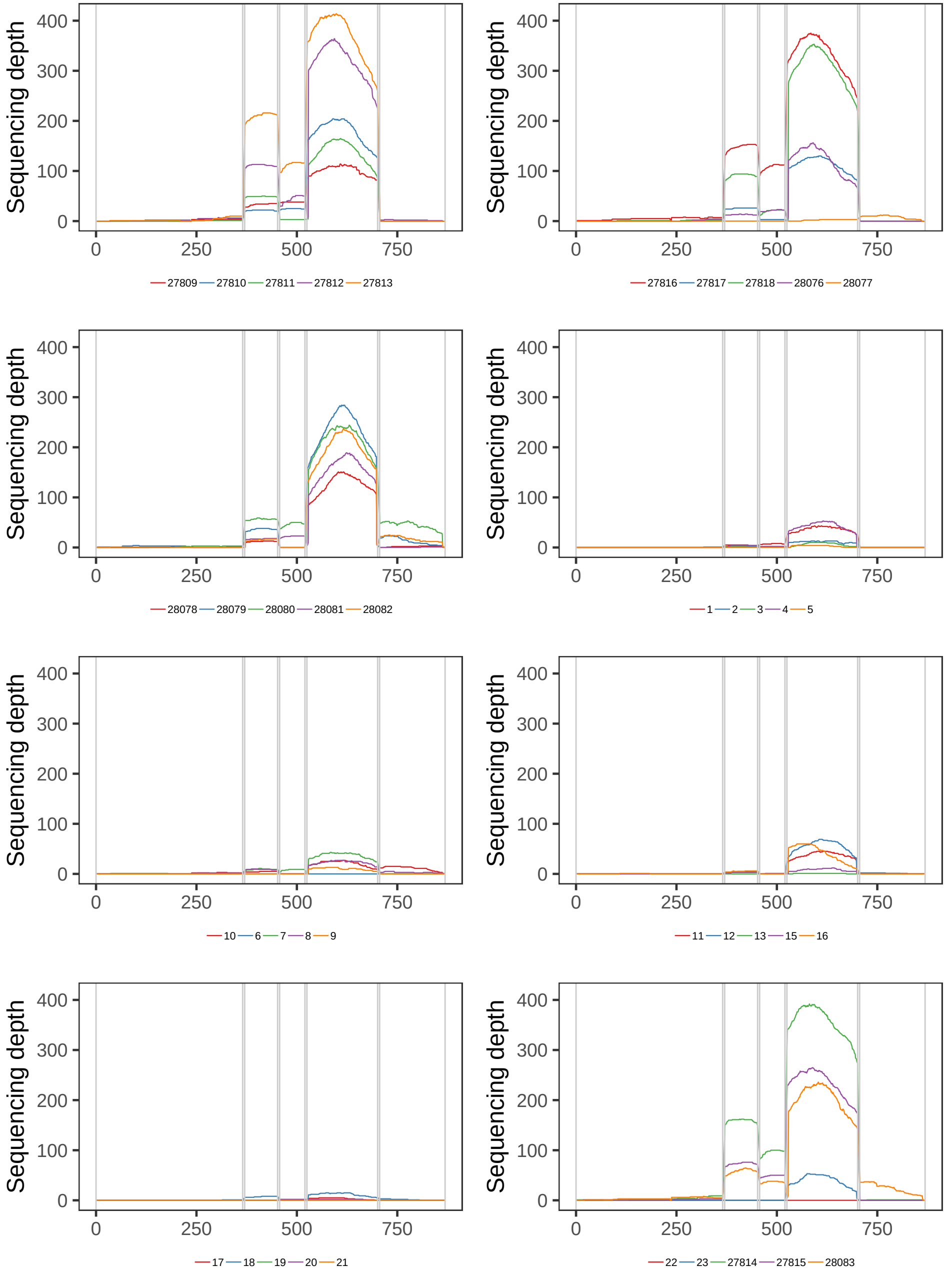
Sequencing depth
0
100
200
300
400
0
250
500
750
27809 27810 27811 27812 27813
27816 27817 27818 28076 28077
28078 28079 28080 28081 28082
1 2 3 4 5
10 6 7 8 9
11 12 13 15 16
17 18 19 20 21
22 23 27814 27815 28083

EOG5JDFPK

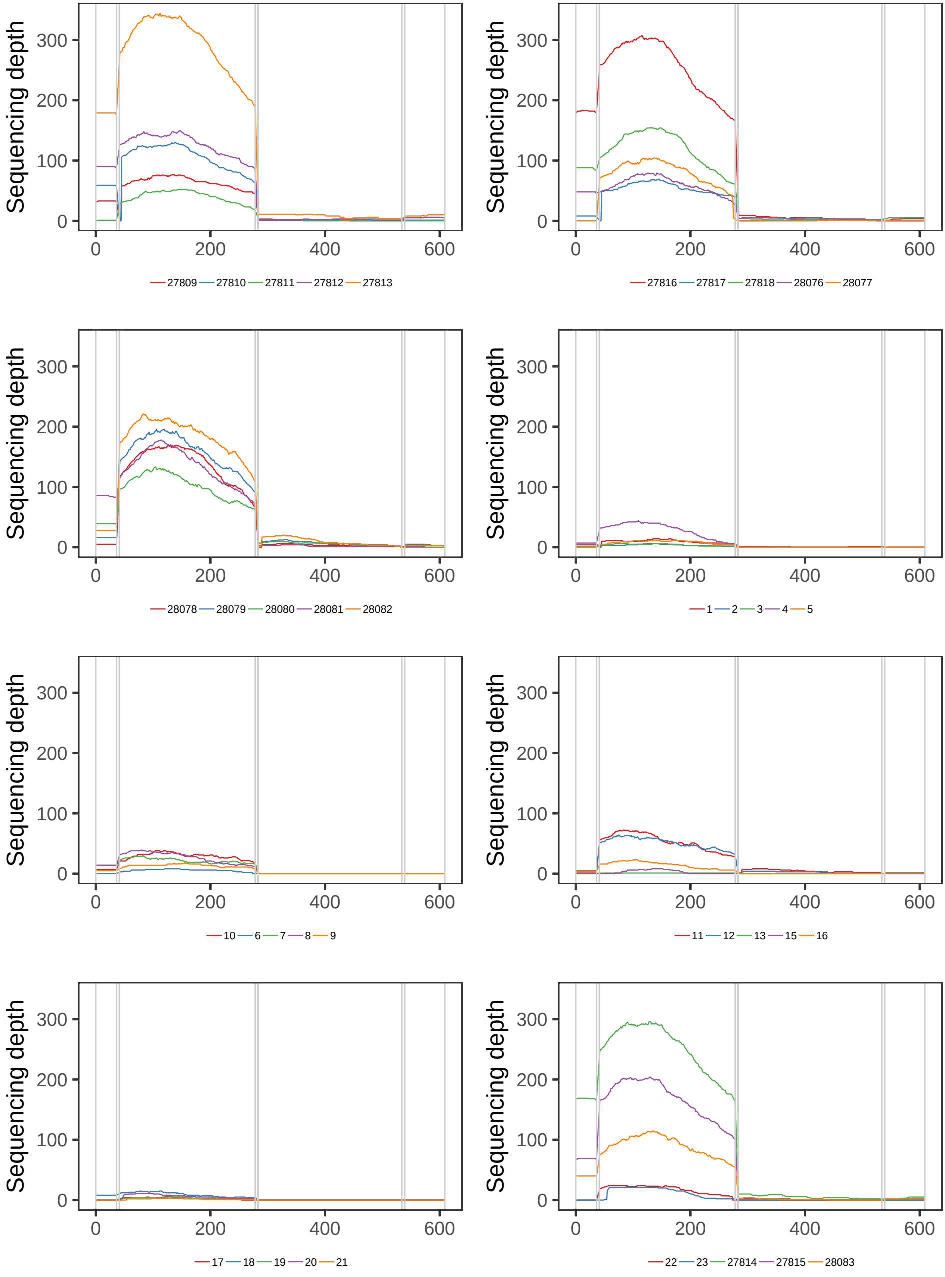

Sequencing depth
0
100
200
300
0
200
400
600
27809 27810 27811 27812 27813
27816 27817 27818 28076 28077
28078 28079 28080 28081 28082
1 2 3 4 5
10 6 7 8 9
11 12 13 15 16
17 18 19 20 21
22 23 27814 27815 28083

EOG5KWH7Z

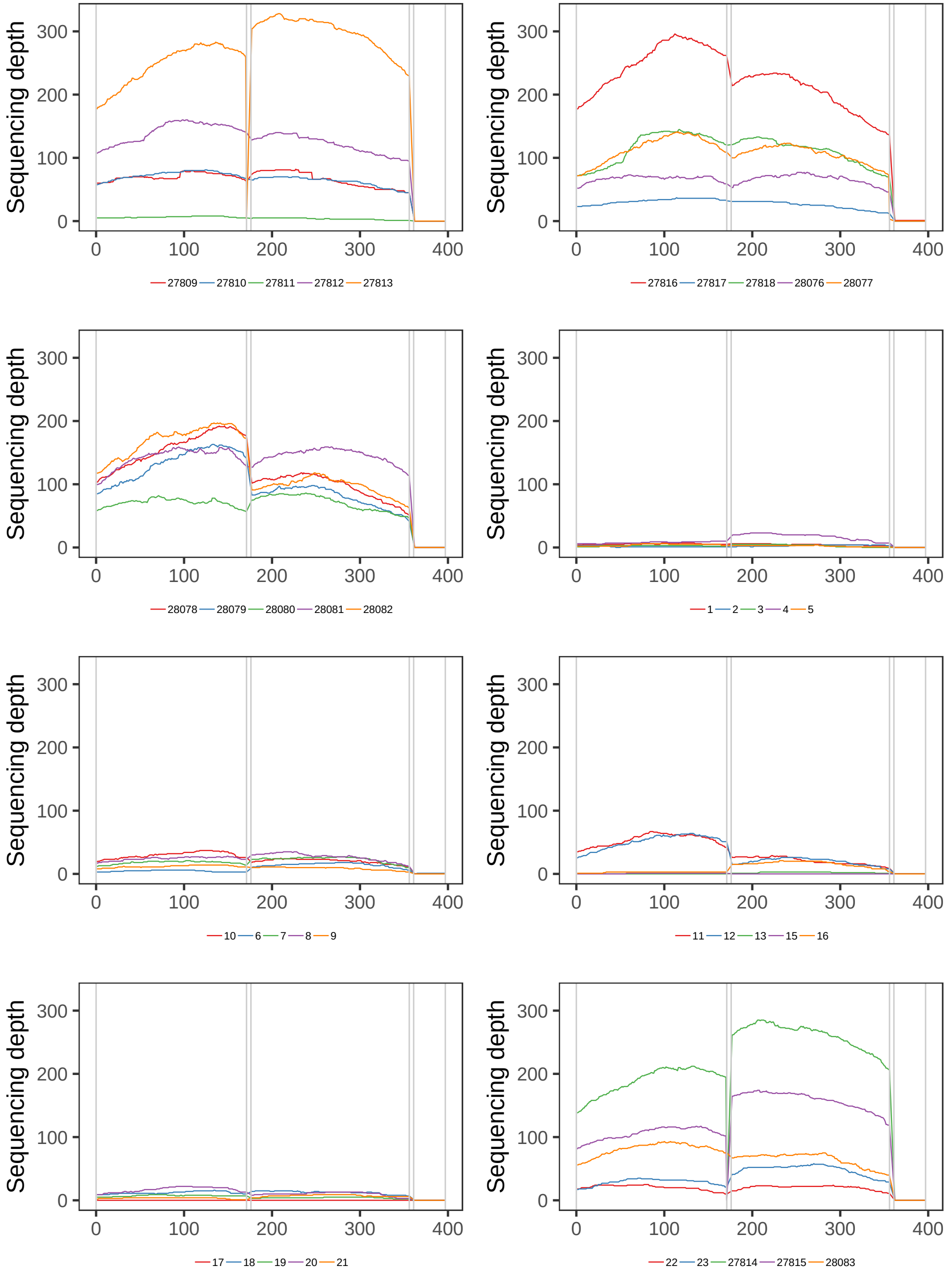
Sequencing depth
0
100
200
300
0
100
200
300
400
27809 27810 27811 27812 27813
27816 27817 27818 28076 28077
28078 28079 28080 28081 28082
1 2 3 4 5
10 6 7 8 9
11 12 13 15 16
17 18 19 20 21
22 23 27814 27815 28083

EOG5R228V

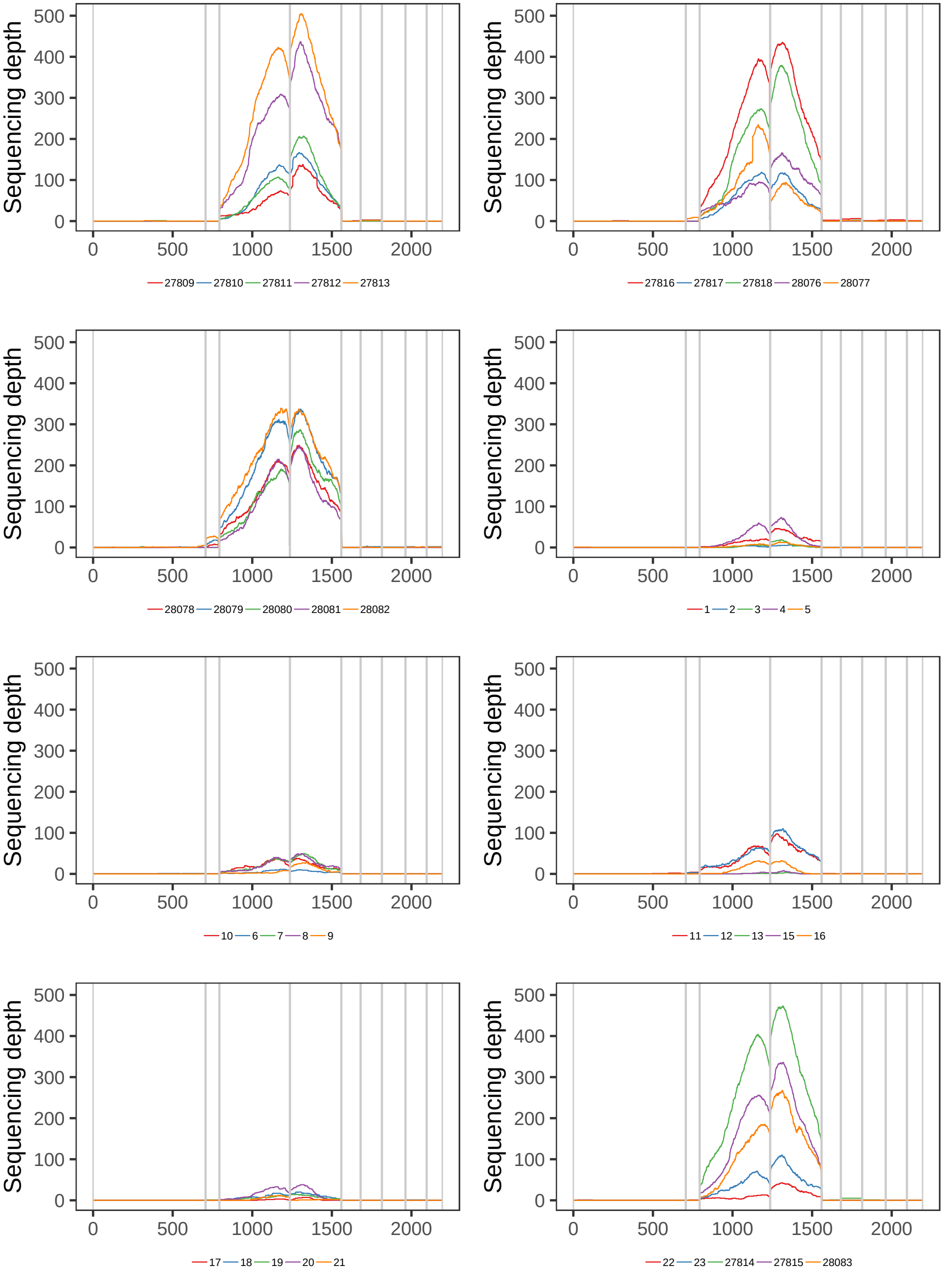
Sequencing depth
0
100
200
300
400
500
0
500
1000
1500
2000
27809 27810 27811 27812 27813
27816 27817 27818 28076 28077
28078 28079 28080 28081 28082
1 2 3 4 5
10 6 7 8 9
11 12 13 15 16
17 18 19 20 21
22 23 27814 27815 28083

EOG5R4XJ0

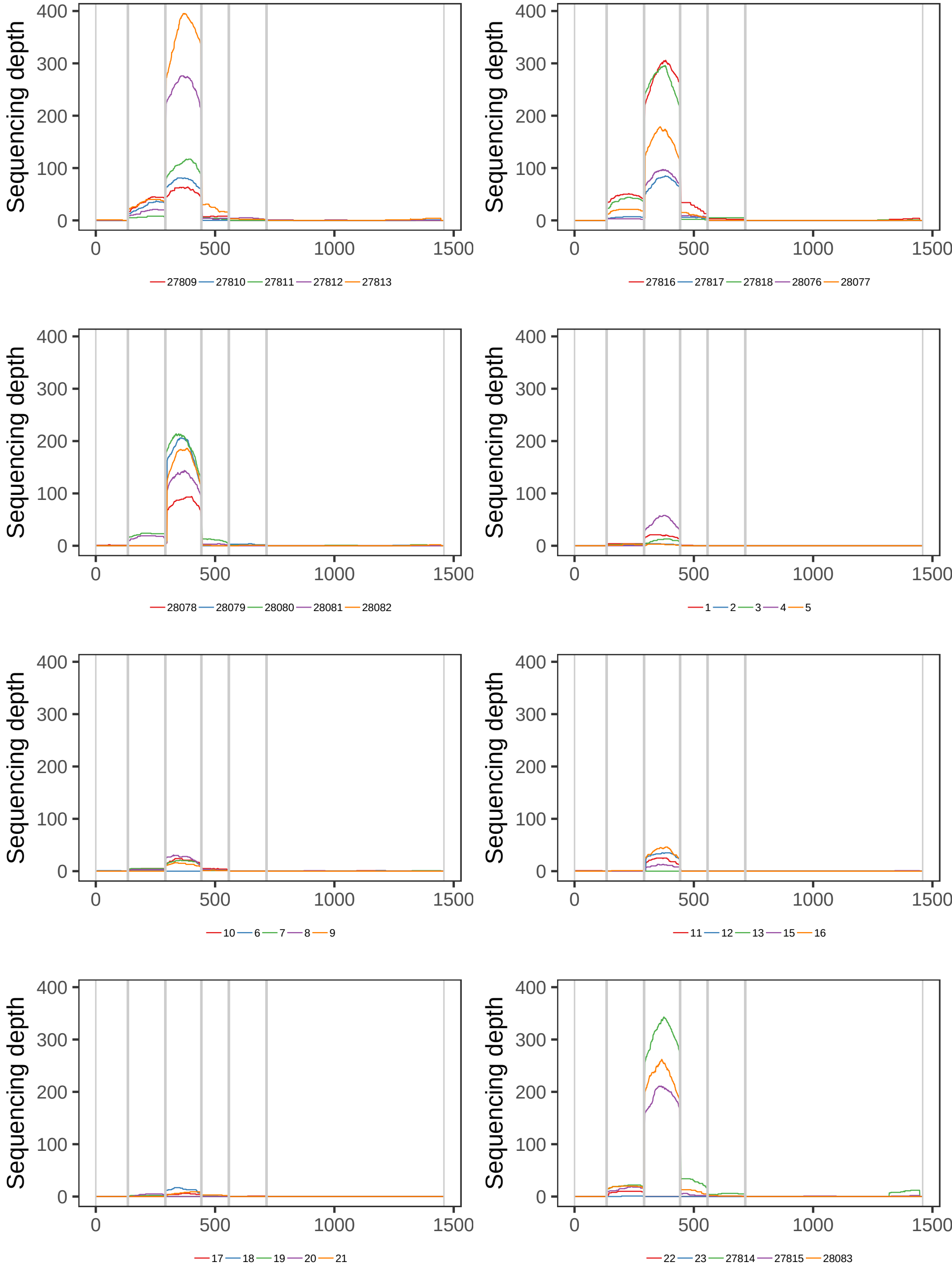
Sequencing depth
0
100
200
300
400
0
500
1000
1500
27809 27810 27811 27812 27813
27816 27817 27818 28076 28077
28078 28079 28080 28081 28082
1 2 3 4 5
10 6 7 8 9
11 12 13 15 16
17 18 19 20 21
22 23 27814 27815 28083

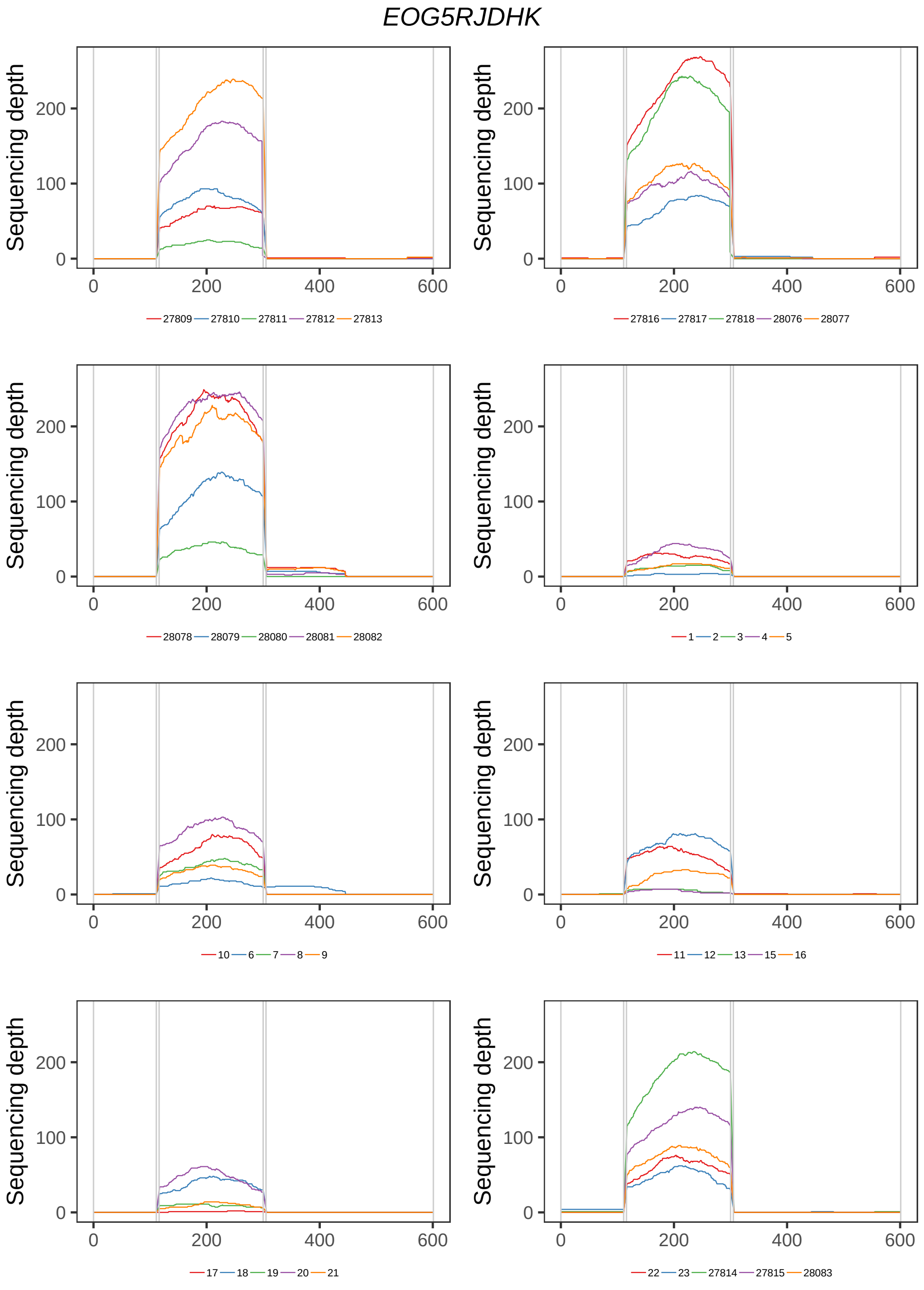
EOG5RJDHK
Sequencing depth
0
100
200
400
600
27809 27810 27811 27812 27813
27816 27817 27818 28076 28077
28078 28079 28080 28081 28082
1 2 3 4 5
10 6 7 8 9
11 12 13 15 16
17 18 19 20 21
22 23 27814 27815 28083

EOG5T76KM

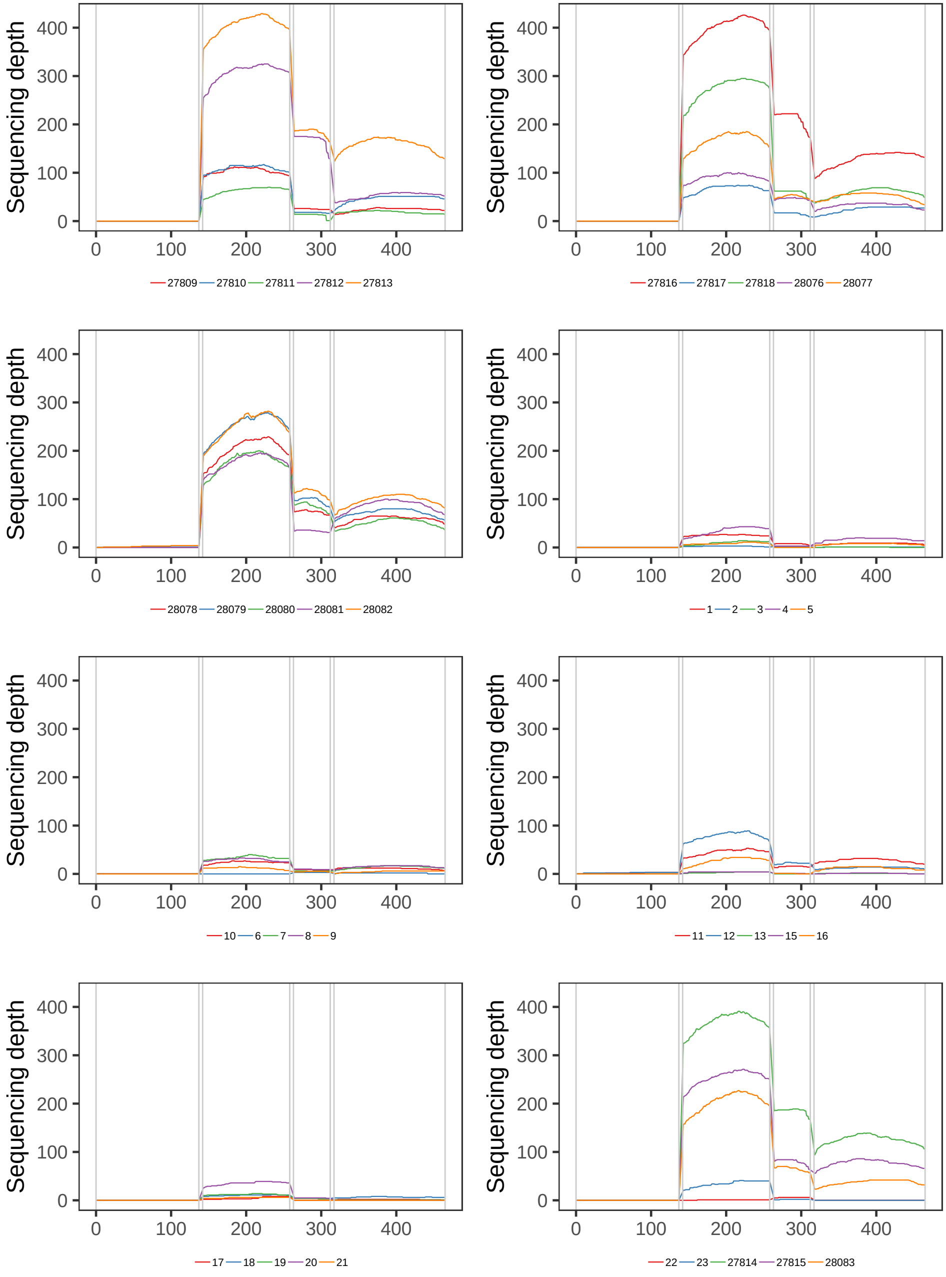

Sequencing depth
0
100
200
300
400
27809 27810 27811 27812 27813
27816 27817 27818 28076 28077
28078 28079 28080 28081 28082
1 2 3 4 5
10 6 7 8 9
11 12 13 15 16
17 18 19 20 21
22 23 27814 27815 28083

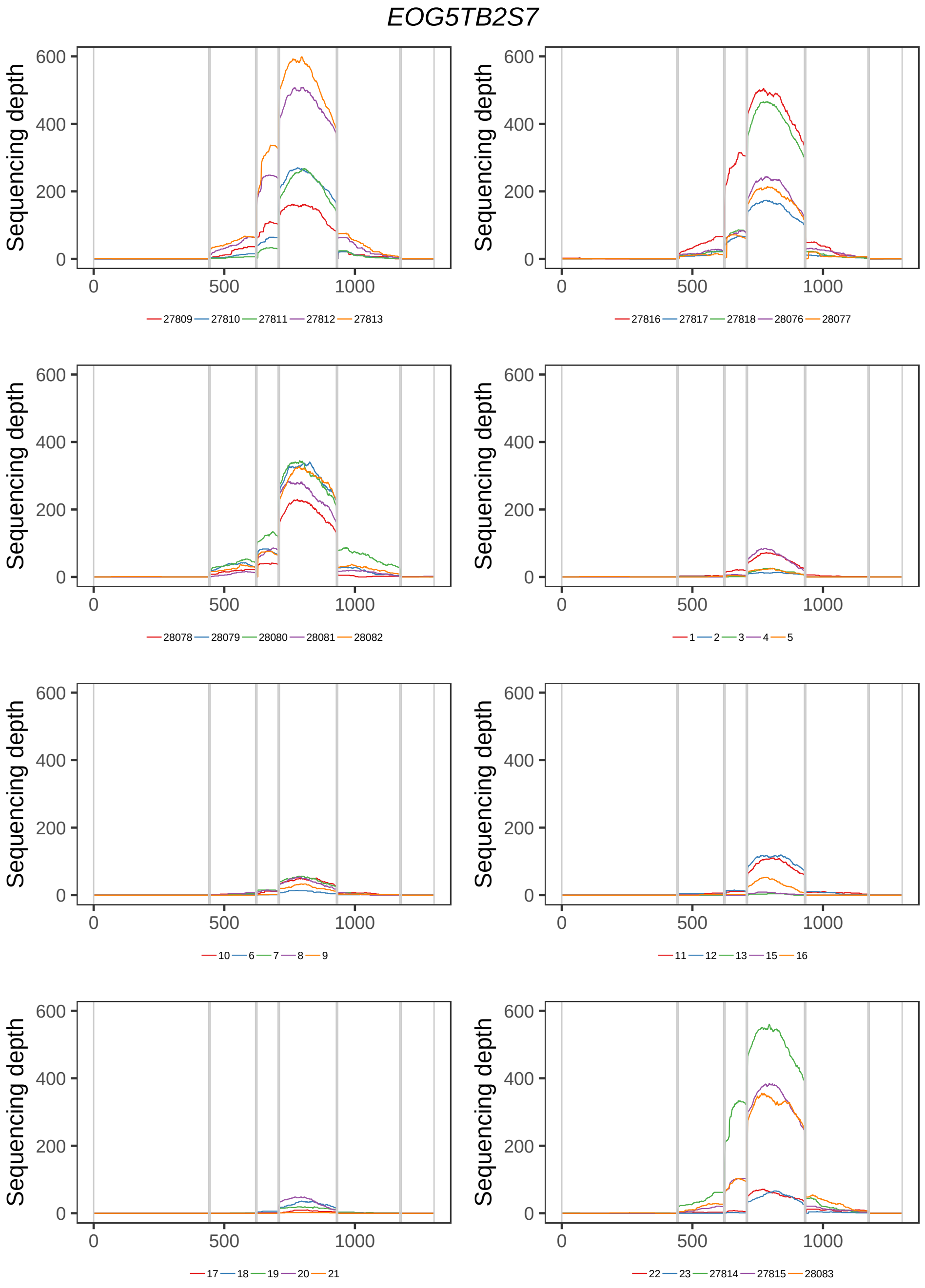

EOG5TB2S7
Sequencing depth
0
200
400
600
0
500
1000
27809
27810
27811
27812
27813
27816
27817
27818
28076
28077
28078
28079
28080
28081
28082
1
2
3
4
5
10
6
7
8
9
11
12
13
15
16
17
18
19
20
21
22
23
27814
27815
28083

EOG5VQ852

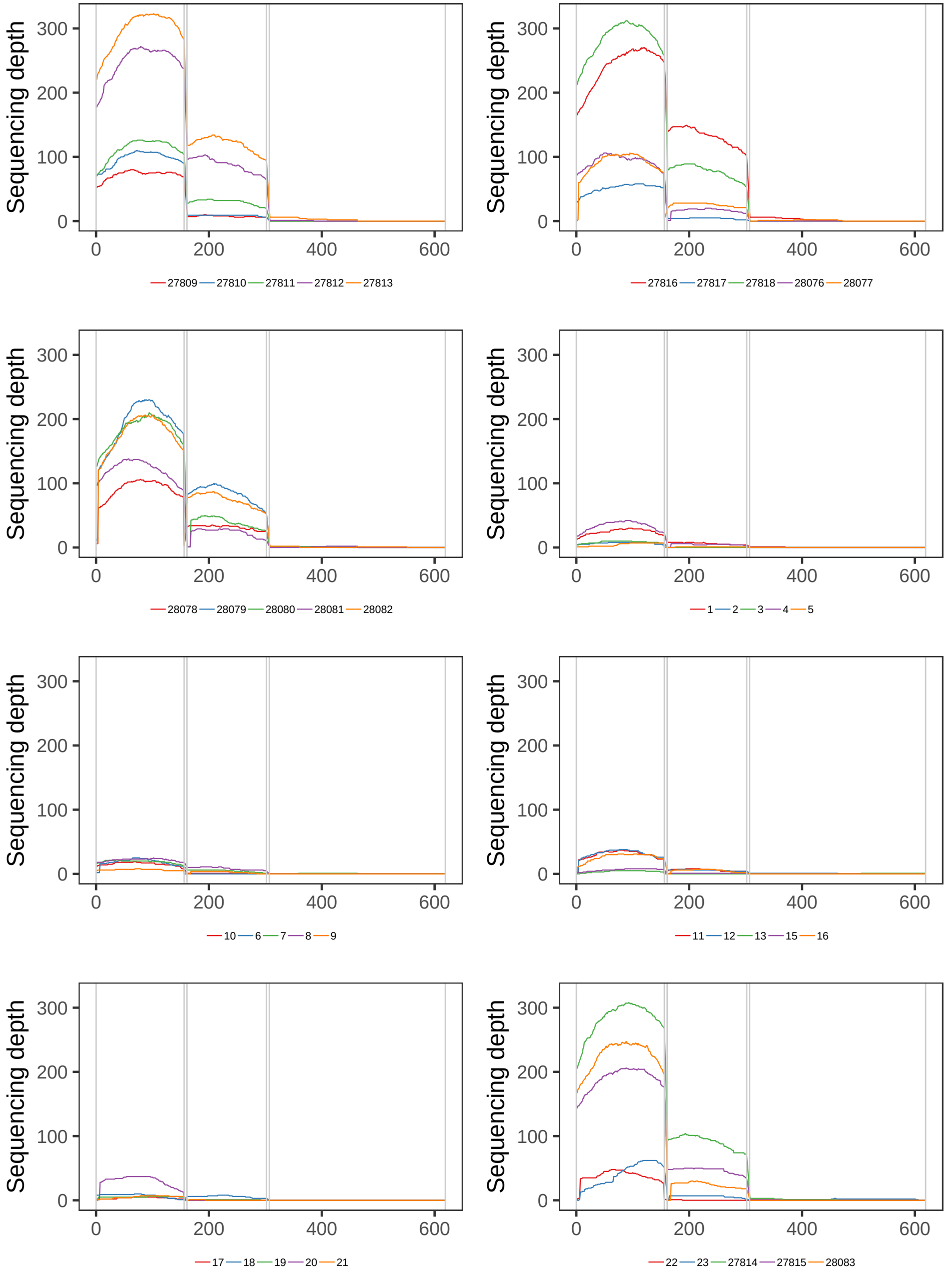
Sequencing depth
0
100
200
300
0
200
400
600
27809 27810 27811 27812 27813
27816 27817 27818 28076 28077
28078 28079 28080 28081 28082
1 2 3 4 5
10 6 7 8 9
11 12 13 15 16
17 18 19 20 21
22 23 27814 27815 28083

EOG5VQ85M

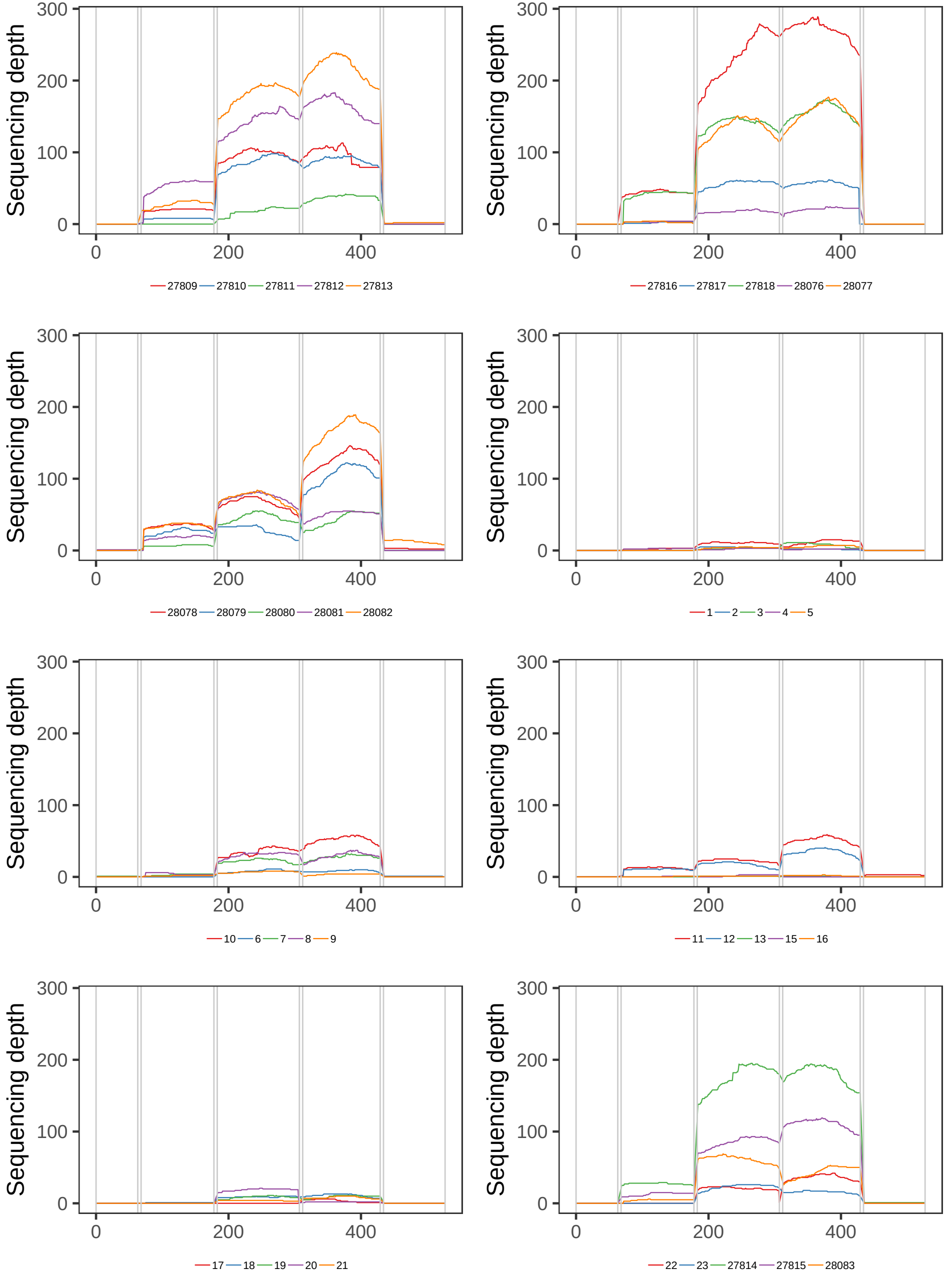
Sequencing depth
300
200
100
0
0
200
400
27809 27810 27811 27812 27813
27816 27817 27818 28076 28077
28078 28079 28080 28081 28082
1 2 3 4 5
10 6 7 8 9
11 12 13 15 16
17 18 19 20 21
22 23 27814 27815 28083

EOG50VT5N

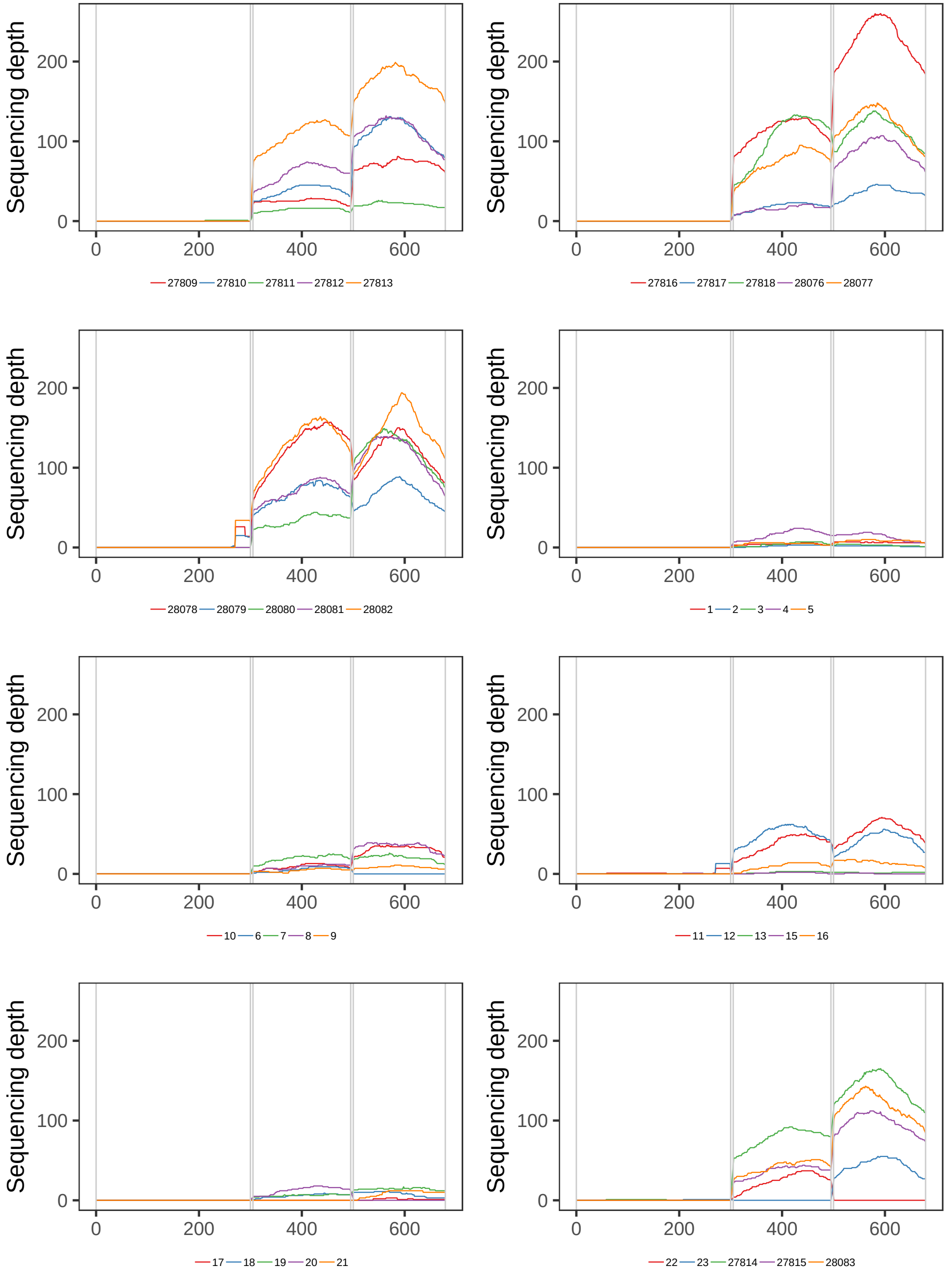
Sequencing depth
0
100
200
0
200
400
600
27809 27810 27811 27812 27813
27816 27817 27818 28076 28077
28078 28079 28080 28081 28082
1 2 3 4 5
10 6 7 8 9
11 12 13 15 16
17 18 19 20 21
22 23 27814 27815 28083

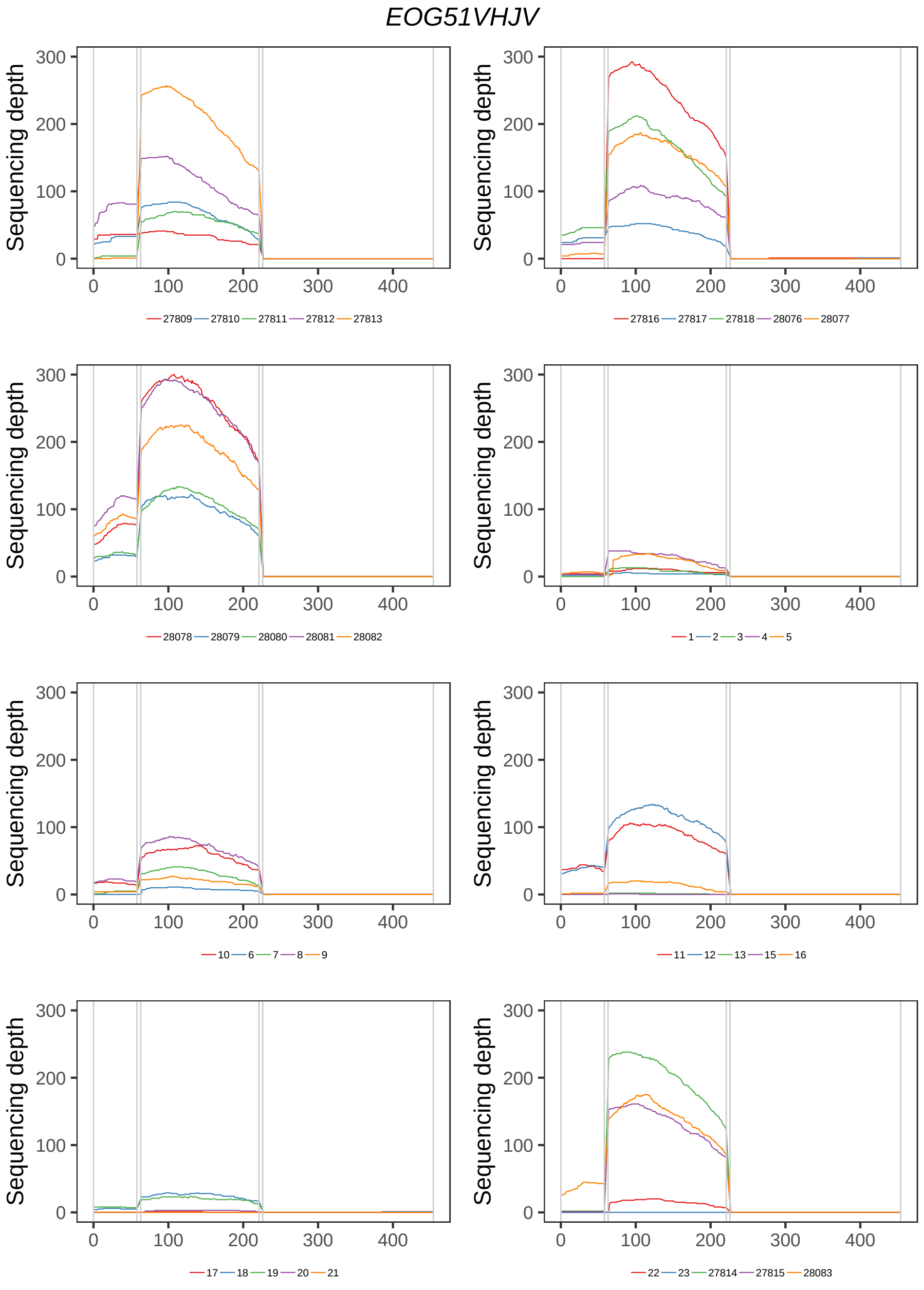

EOG51VHJV
Sequencing depth
300
200
100
0
0
100
200
300
400
27809 27810 27811 27812 27813
27816 27817 27818 28076 28077
28078 28079 28080 28081 28082
1 2 3 4 5
10 6 7 8 9
11 12 13 15 16
17 18 19 20 21
22 23 27814 27815 28083

EOG52FR0S

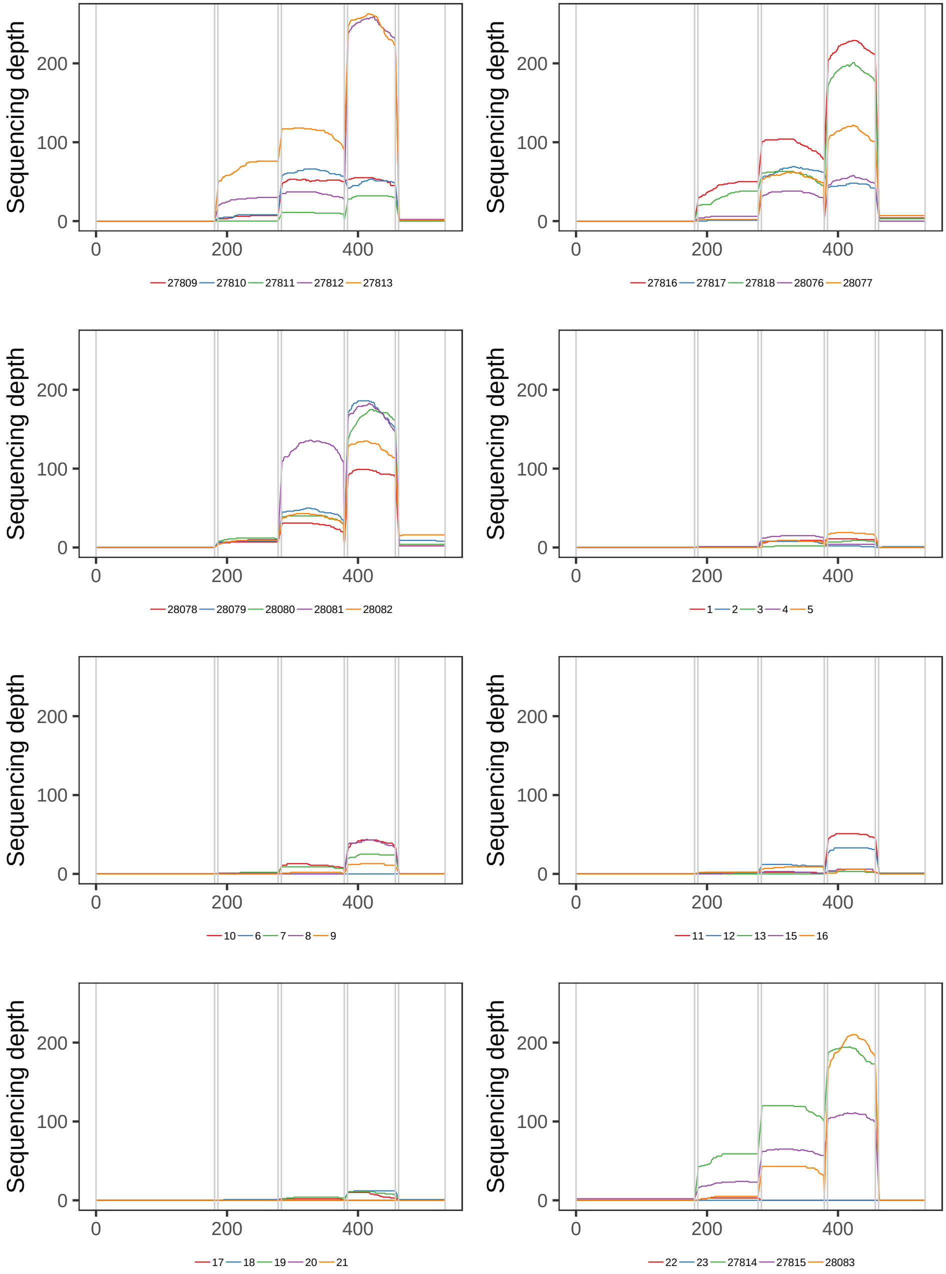
Sequencing depth
200
100
0
0
200
400
27809
27810
27811
27812
27813
27816
27817
27818
28076
28077
28078
28079
28080
28081
28082
1
2
3
4
5
10
6
7
8
9
11
12
13
15
16
17
18
19
20
21
22
23
27814
27815
28083

EOG56Q58J

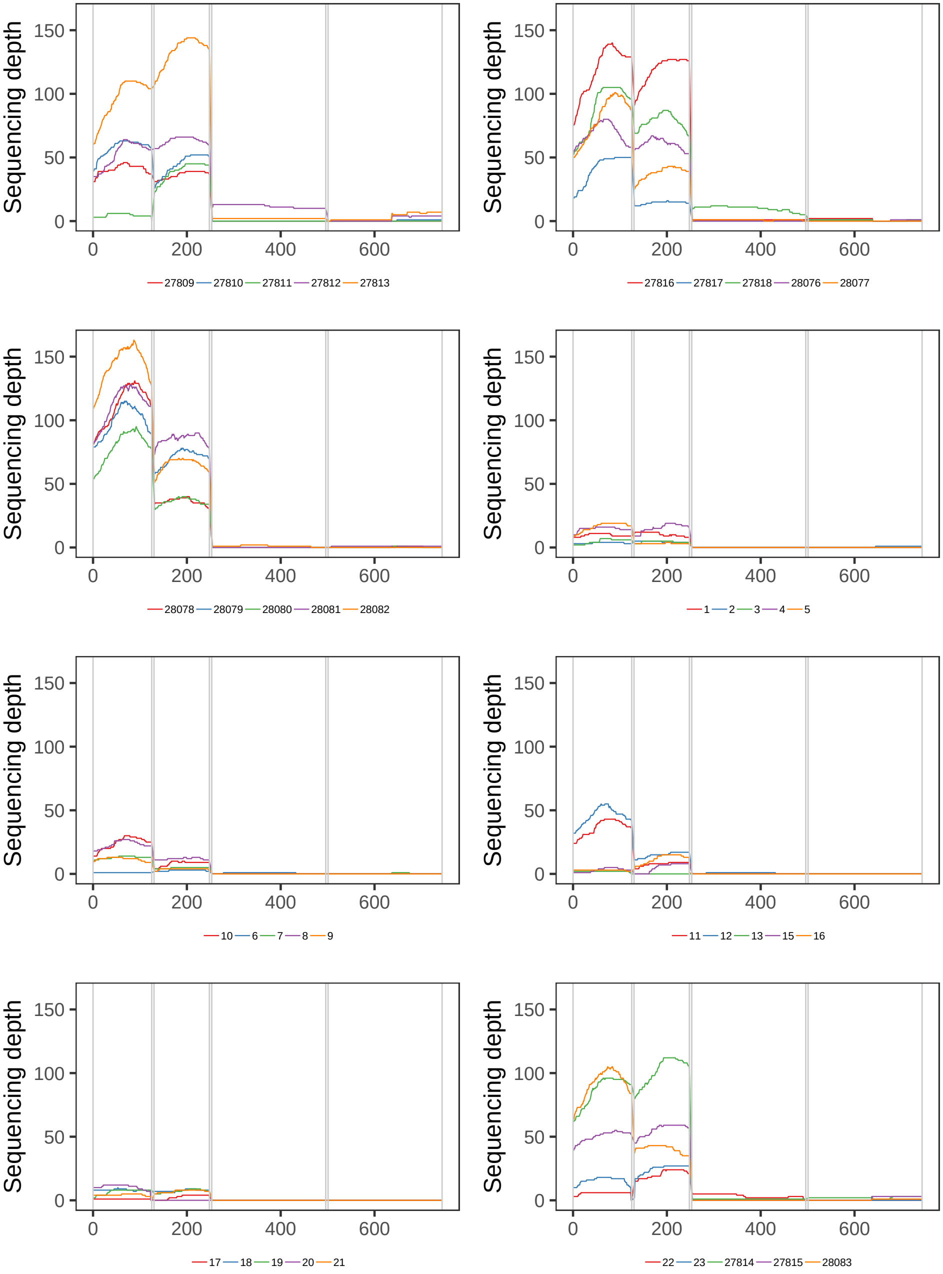

Sequencing depth
0
50
100
150
0
200
400
600
27809 27810 27811 27812 27813
27816 27817 27818 28076 28077
28078 28079 28080 28081 28082
1 2 3 4 5
10 6 7 8 9
11 12 13 15 16
17 18 19 20 21
22 23 27814 27815 28083

EOG580GDB

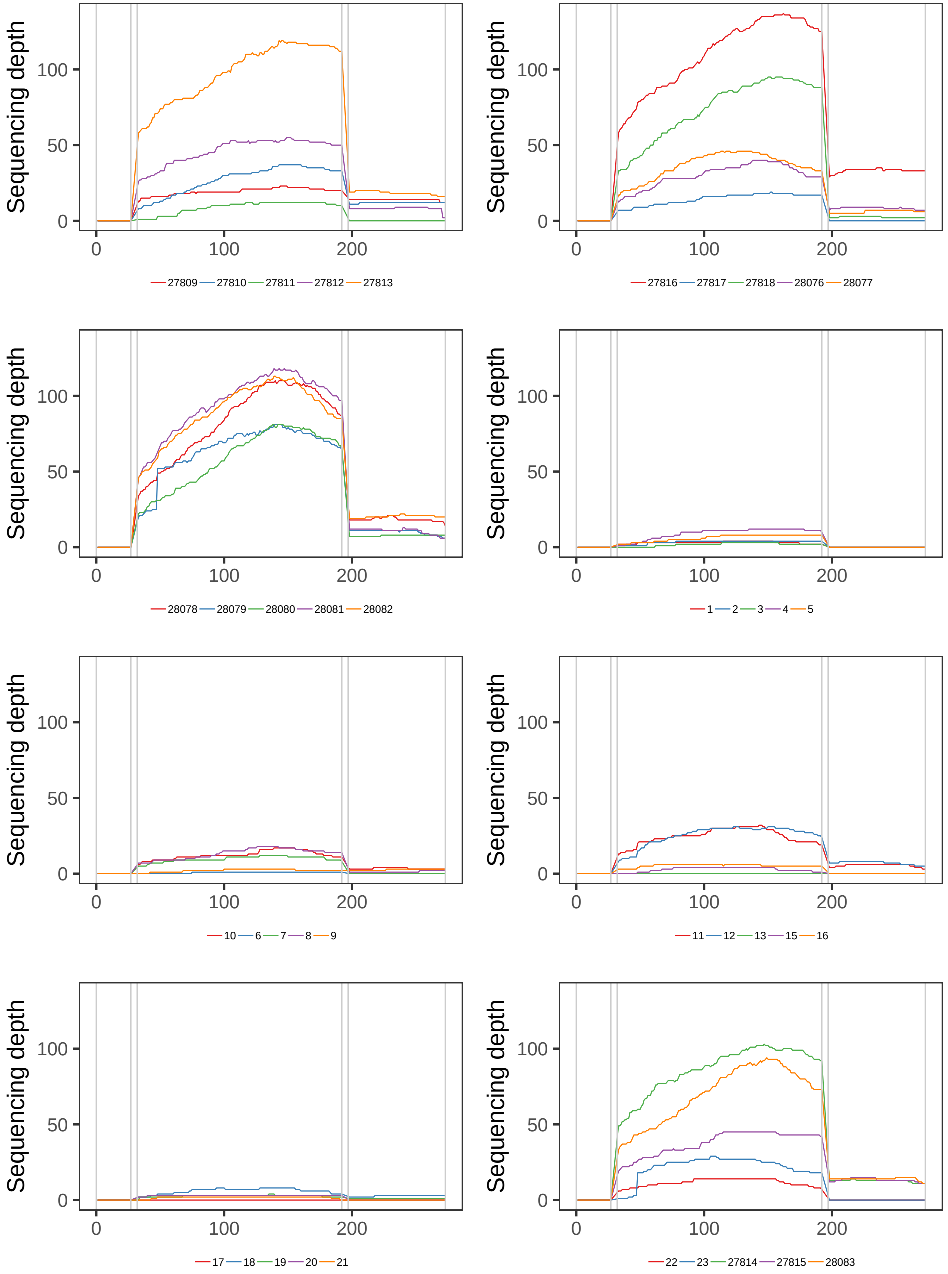
Sequencing depth
0
50
100
0
100
200
27809
27810
27811
27812
27813
27816
27817
27818
28076
28077
28078
28079
28080
28081
28082
1
2
3
4
5
10
6
7
8
9
11
12
13
15
16
17
18
19
20
21
22
23
27814
27815
28083

EOG5BNZTJ

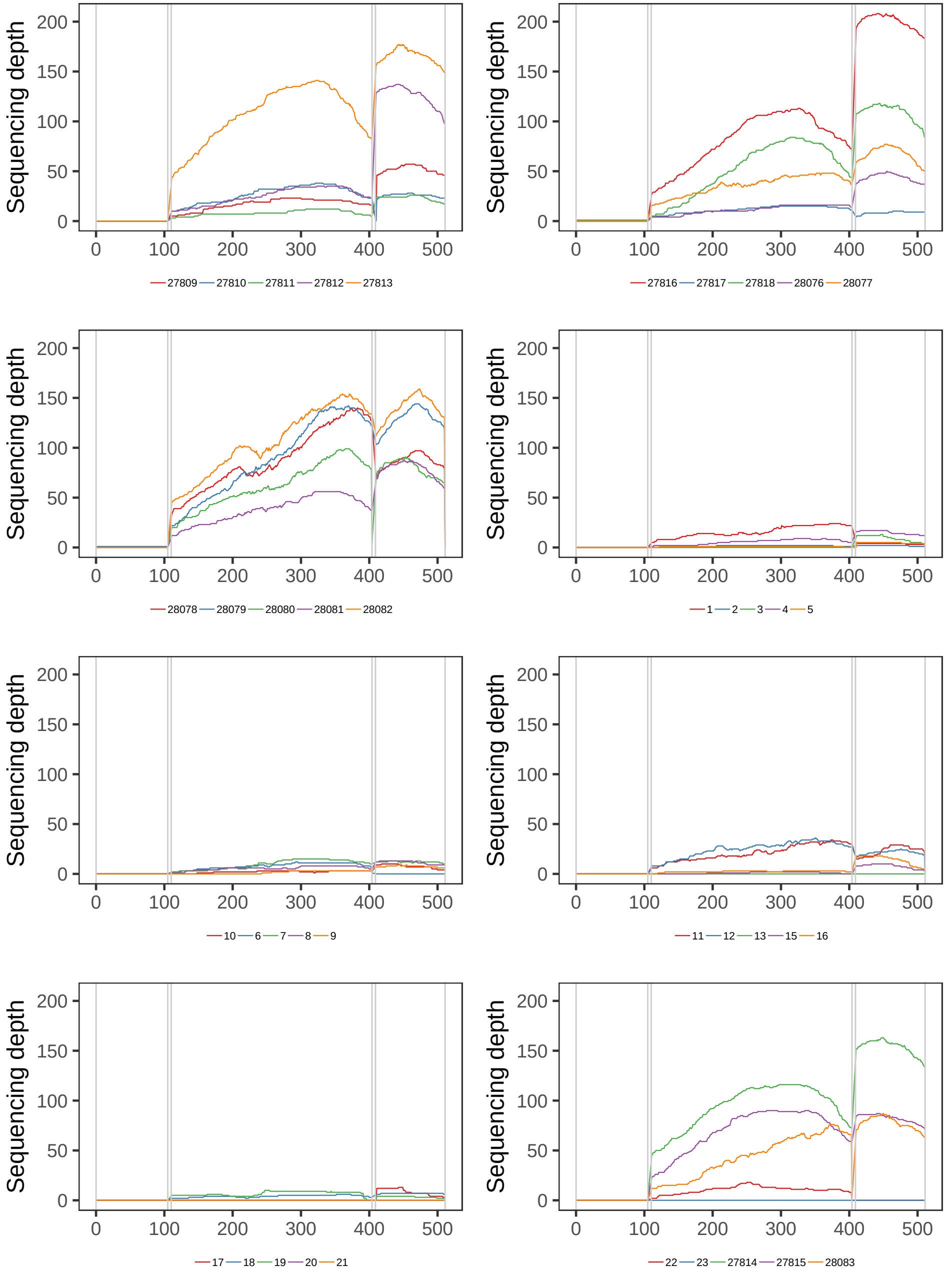
Sequencing depth
0
50
100
150
200
0
100
200
300
400
500
27809 27810 27811 27812 27813
27816 27817 27818 28076 28077
28078 28079 28080 28081 28082
1 2 3 4 5
10 6 7 8 9
11 12 13 15 16
17 18 19 20 21
22 23 27814 27815 28083

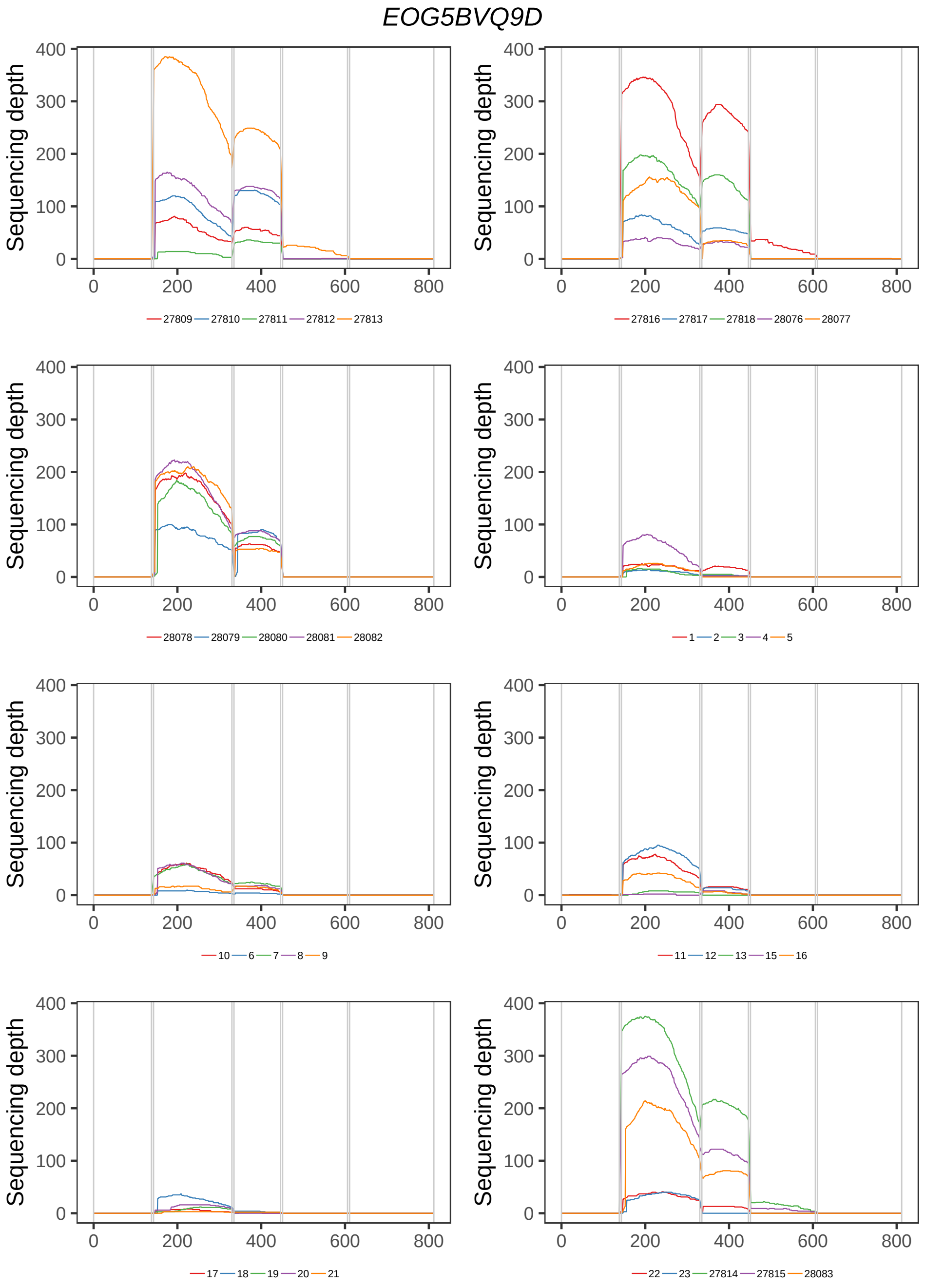
EOG5BVQ9D
Sequencing depth
27809 27810 27811 27812 27813
27816 27817 27818 28076 28077
28078 28079 28080 28081 28082
1 2 3 4 5
10 6 7 8 9
11 12 13 15 16
17 18 19 20 21
22 23 27814 27815 28083

EOG5CJT0B

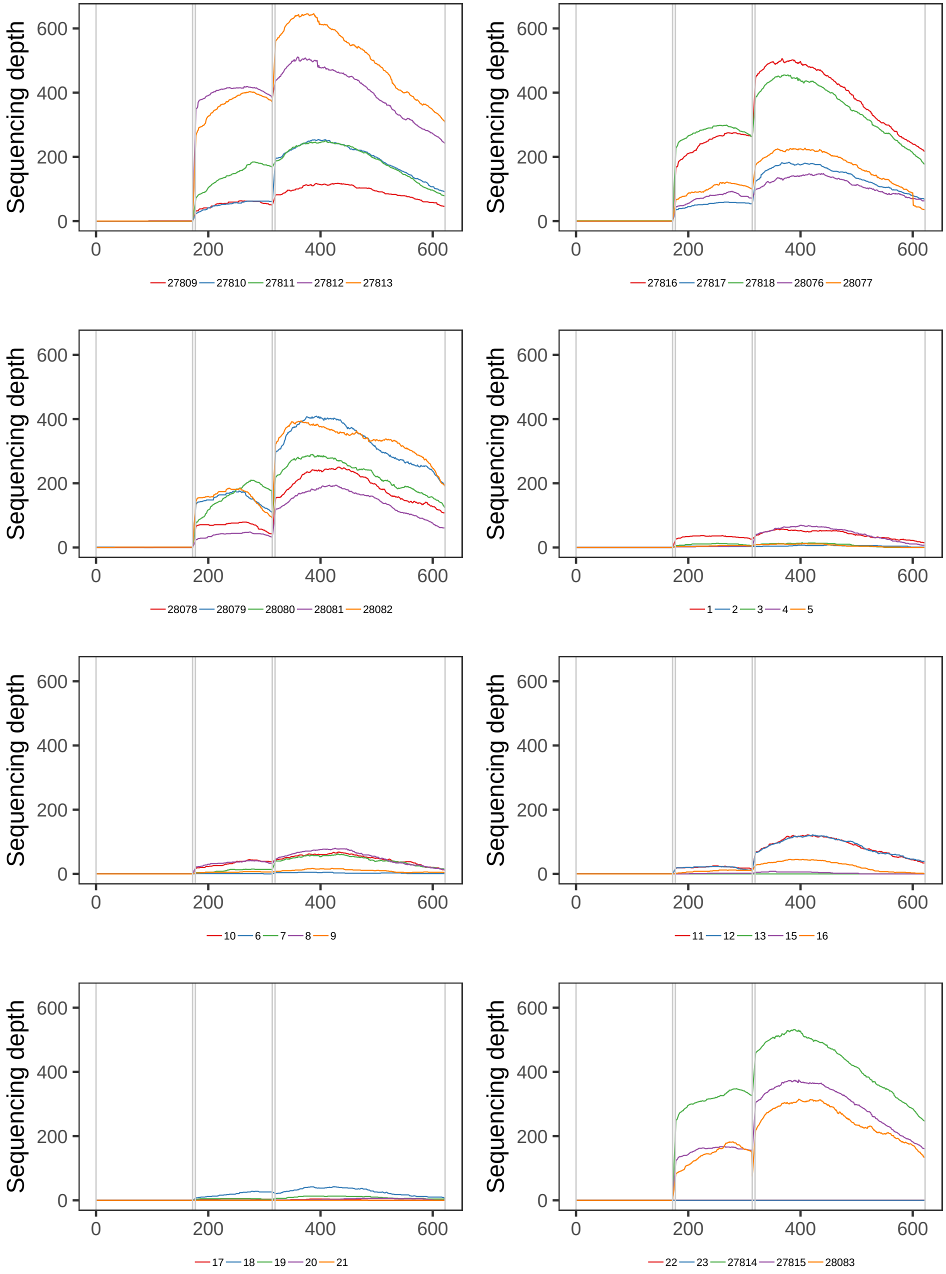
Sequencing depth
0
200
400
600
27809 27810 27811 27812 27813
27816 27817 27818 28076 28077
28078 28079 28080 28081 28082
1 2 3 4 5
10 6 7 8 9
11 12 13 15 16
17 18 19 20 21
22 23 27814 27815 28083

EOG5DFN58

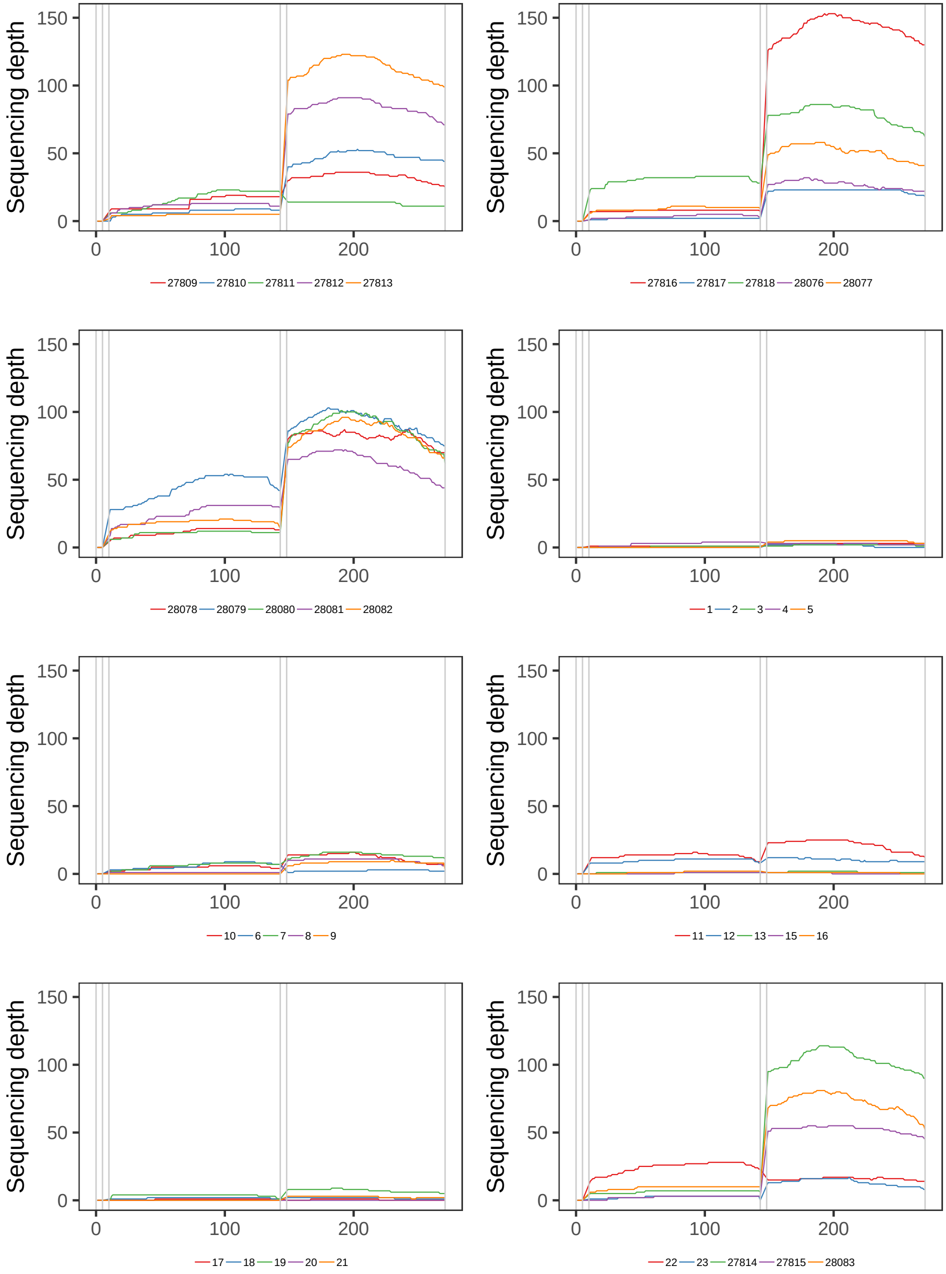

Sequencing depth
150
100
50
0
0
100
200
27809 27810 27811 27812 27813
27816 27817 27818 28076 28077
28078 28079 28080 28081 28082
1 2 3 4 5
10 6 7 8 9
11 12 13 15 16
17 18 19 20 21
22 23 27814 27815 28083

EOG5GB5NB

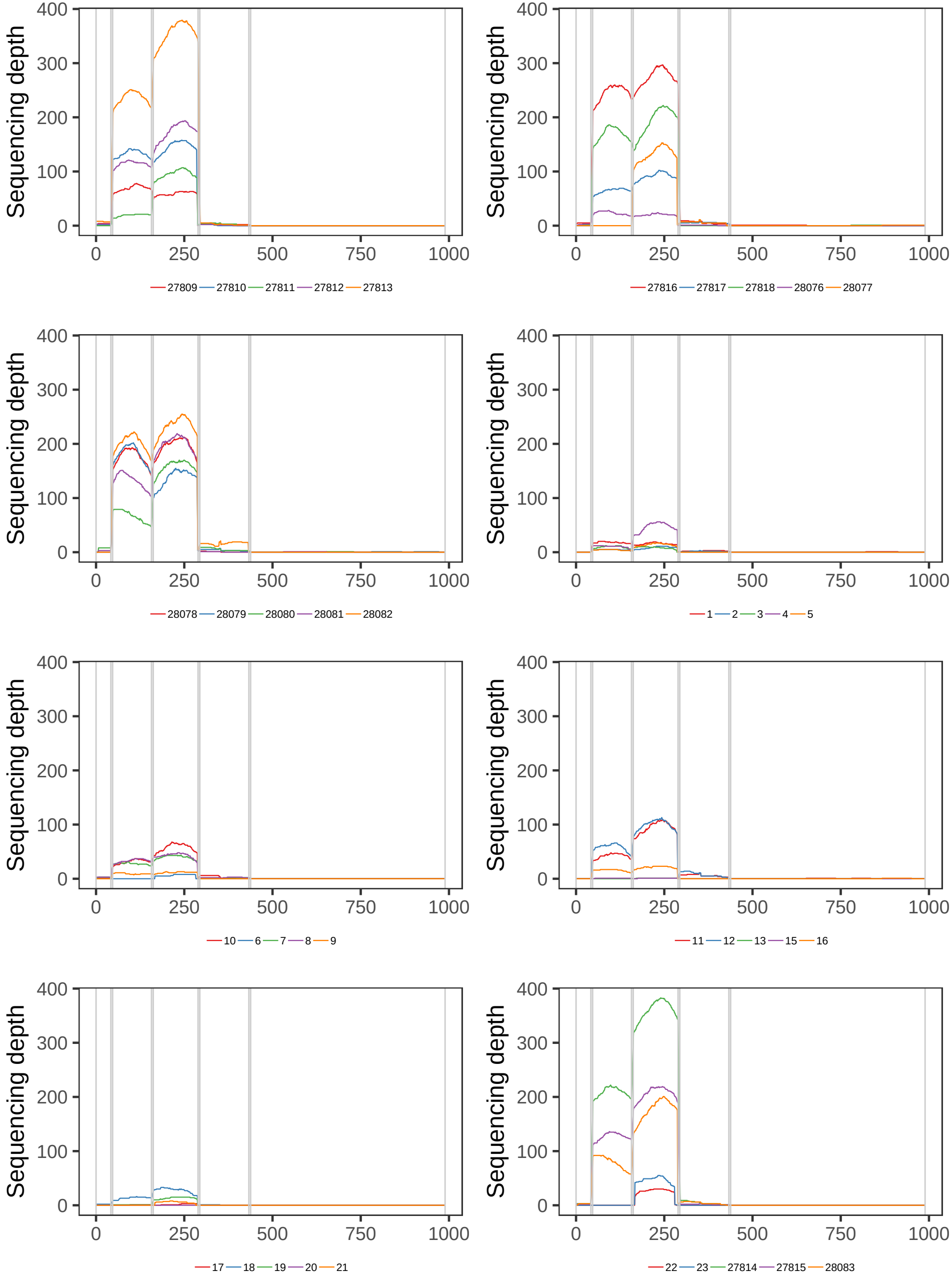
Sequencing depth
0
100
200
300
400
0
250
500
750
1000
27809 27810 27811 27812 27813
27816 27817 27818 28076 28077
28078 28079 28080 28081 28082
1 2 3 4 5
10 6 7 8 9
11 12 13 15 16
17 18 19 20 21
22 23 27814 27815 28083

EOG5HDR9D

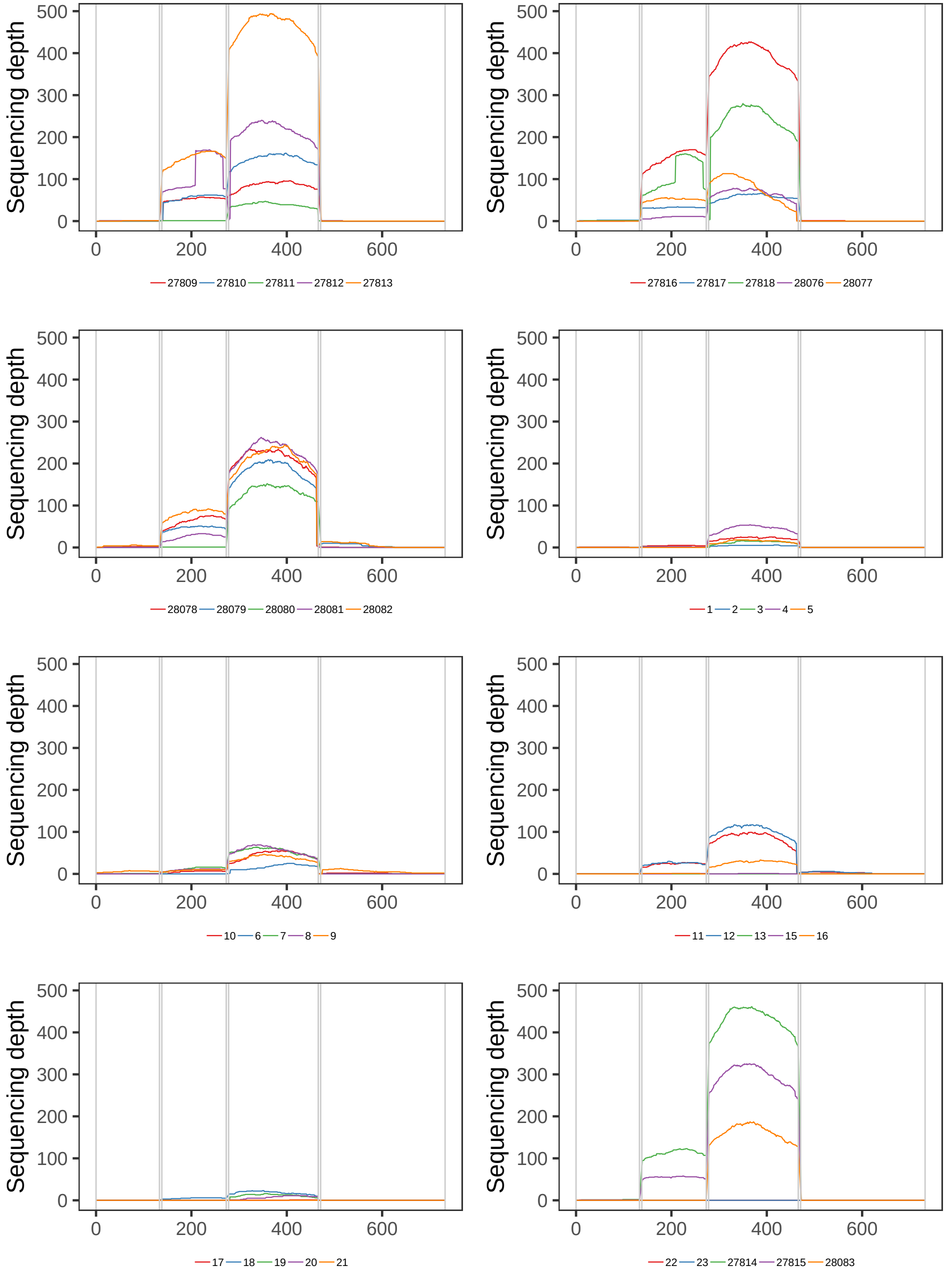

Sequencing depth
500
400
300
200
100
0
0
200
400
600
27809
27810
27811
27812
27813
27816
27817
27818
28076
28077
28078
28079
28080
28081
28082
1
2
3
4
5
10
6
7
8
9
11
12
13
15
16
17
18
19
20
21
22
23
27814
27815
28083

EOG5HX3GR

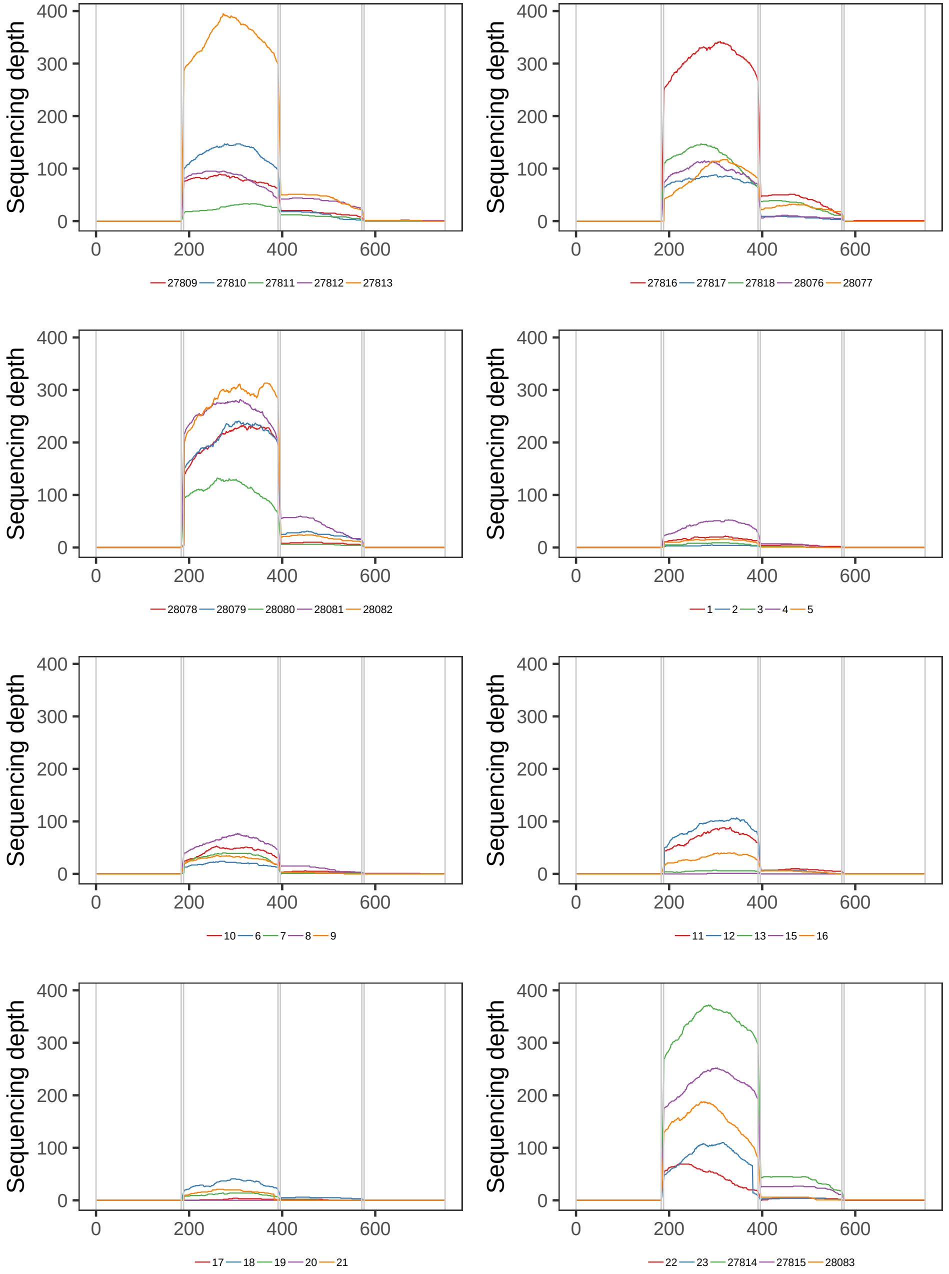
Sequencing depth
400
300
200
100
0
0
200
400
600
27809 27810 27811 27812 27813
27816 27817 27818 28076 28077
28078 28079 28080 28081 28082
1 2 3 4 5
10 6 7 8 9
11 12 13 15 16
17 18 19 20 21
22 23 27814 27815 28083

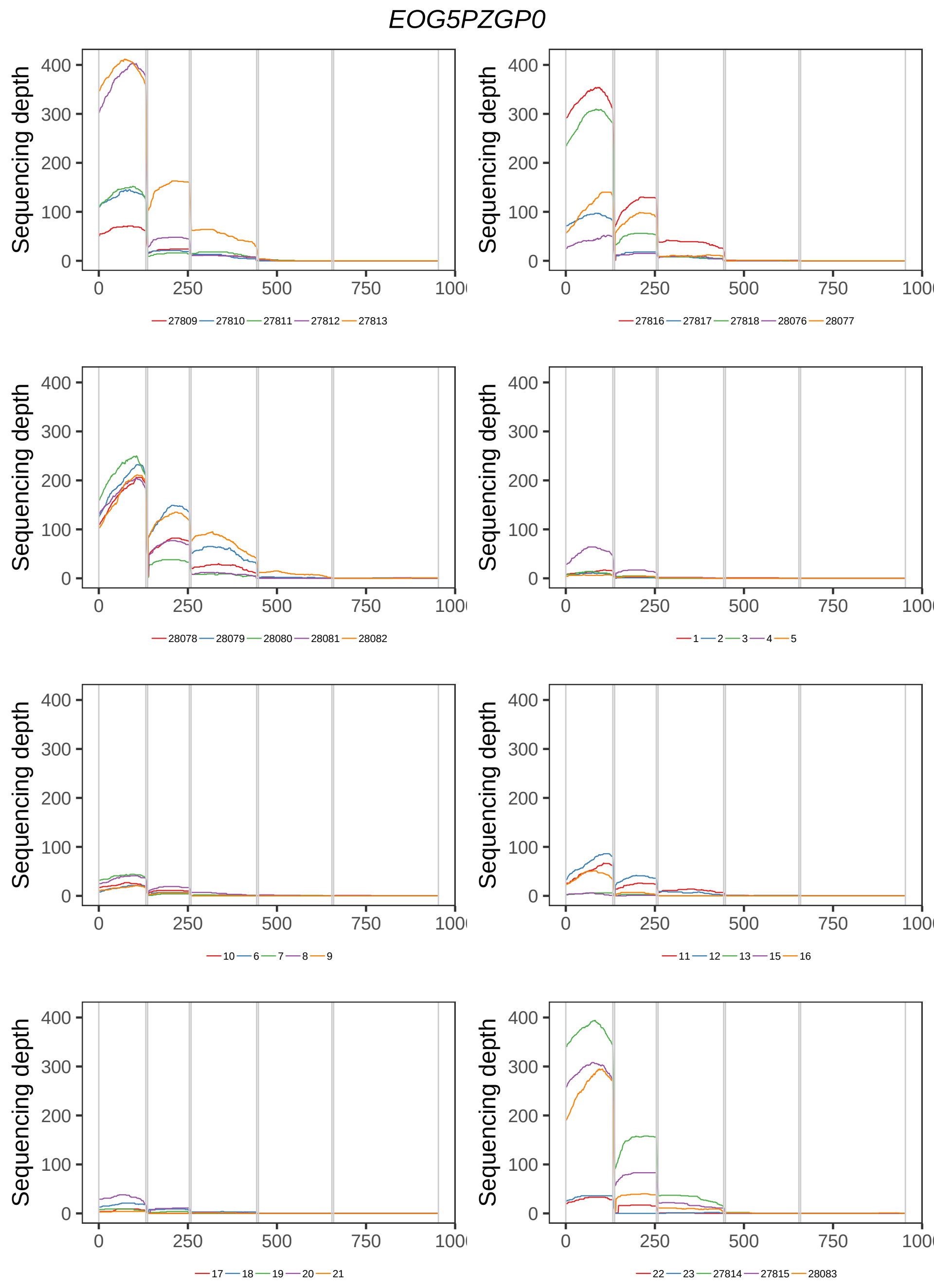
EOG5PZGP0
Sequencing depth
0
100
200
300
400
0
250
500
750
100
27809 27810 27811 27812 27813
27816 27817 27818 28076 28077
28078 28079 28080 28081 28082
1 2 3 4 5
10 6 7 8 9
11 12 13 15 16
17 18 19 20 21
22 23 27814 27815 28083

EOG5QNKC5

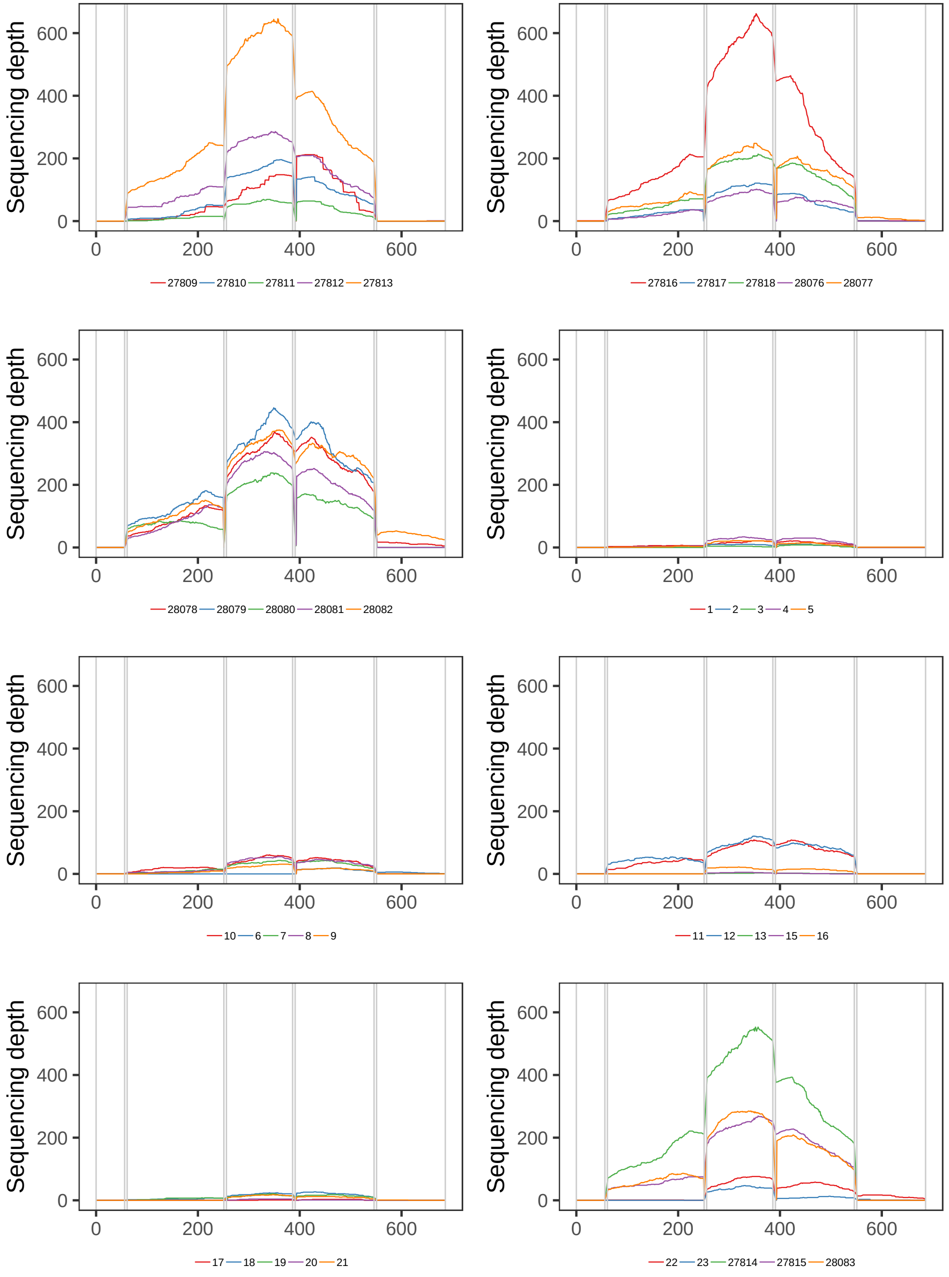
Sequencing depth
0
200
400
600
27809 27810 27811 27812 27813
27816 27817 27818 28076 28077
28078 28079 28080 28081 28082
1 2 3 4 5
10 6 7 8 9
11 12 13 15 16
17 18 19 20 21
22 23 27814 27815 28083

EOG5RN8RD

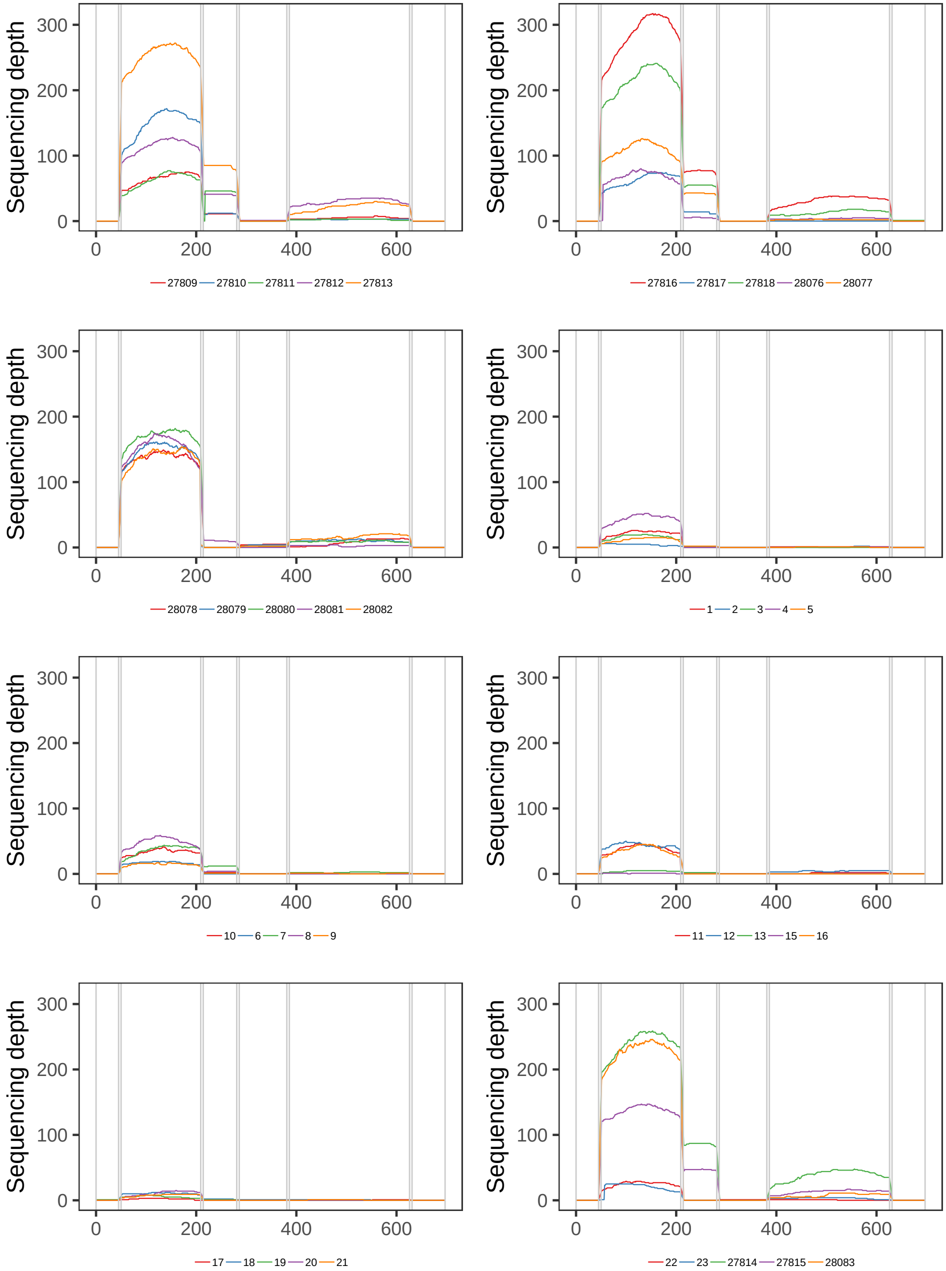
Sequencing depth
0
100
200
300
0
200
400
600
27809
27810
27811
27812
27813
27816
27817
27818
28076
28077
28078
28079
28080
28081
28082
1
2
3
4
5
10
6
7
8
9
11
12
13
15
16
17
18
19
20
21
22
23
27814
27815
28083

EOG5STQMD

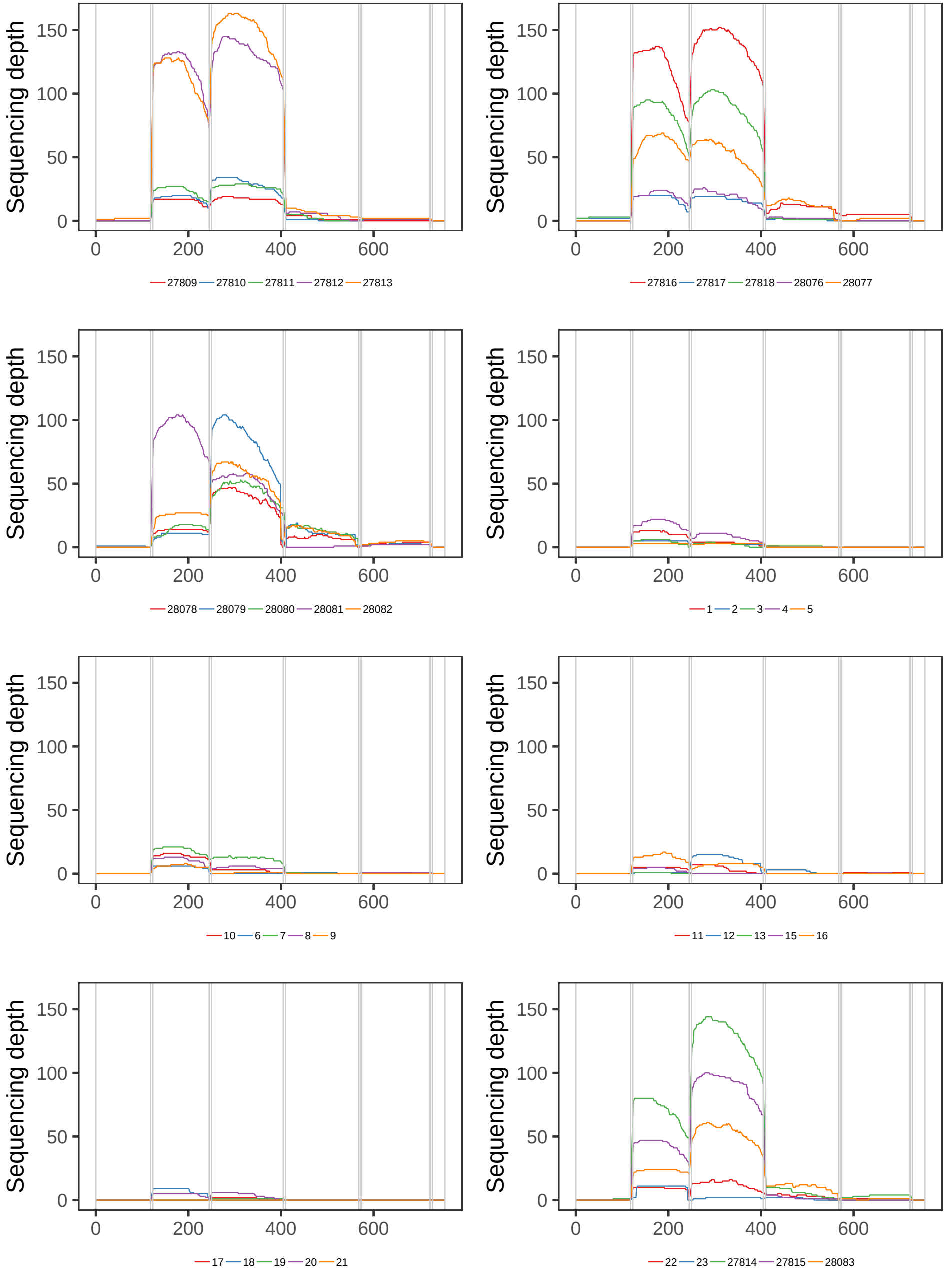

Sequencing depth
0
50
100
150
0
200
400
600
27809 27810 27811 27812 27813
27816 27817 27818 28076 28077
28078 28079 28080 28081 28082
1 2 3 4 5
10 6 7 8 9
11 12 13 15 16
17 18 19 20 21
22 23 27814 27815 28083

EOG5TMPHT

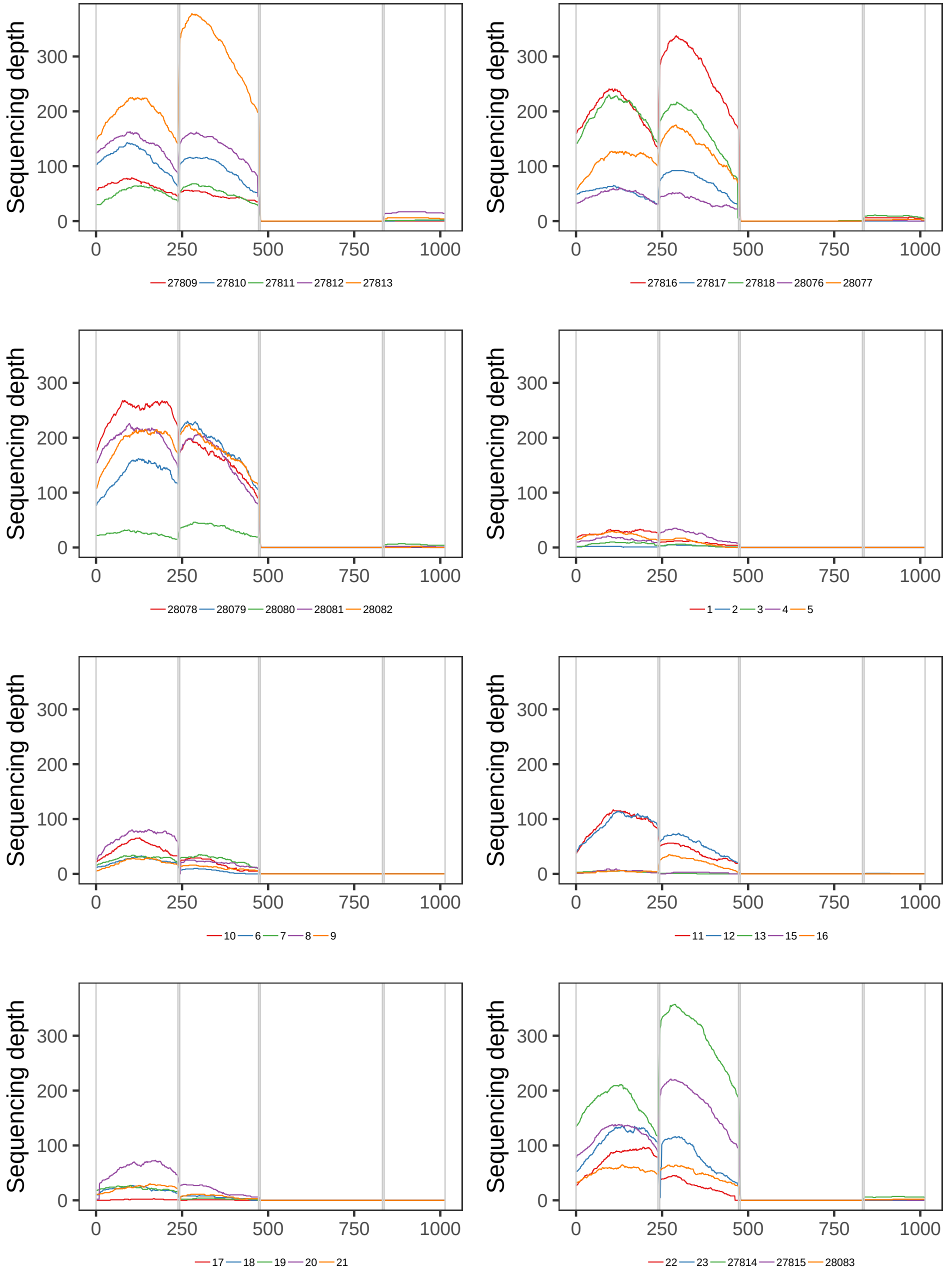

Sequencing depth
0
100
200
300
0
250
500
750
1000
27809
27810
27811
27812
27813
27816
27817
27818
28076
28077
28078
28079
28080
28081
28082
1
2
3
4
5
10
6
7
8
9
11
12
13
15
16
17
18
19
20
21
22
23
27814
27815
28083

EOG5TTF07

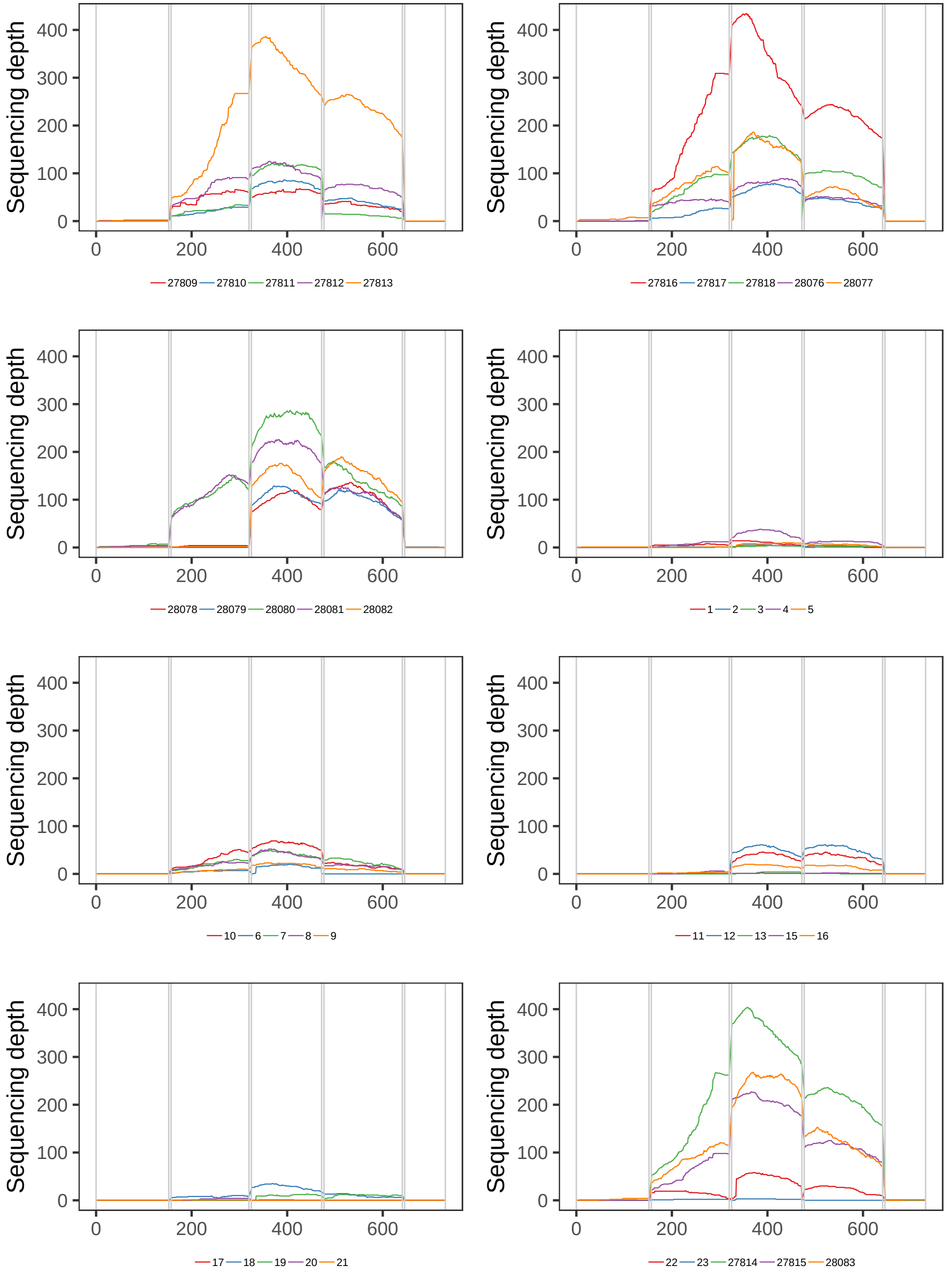
Sequencing depth
0
100
200
300
400
0
200
400
600
27809 27810 27811 27812 27813
27816 27817 27818 28076 28077
28078 28079 28080 28081 28082
1 2 3 4 5
10 6 7 8 9
11 12 13 15 16
17 18 19 20 21
22 23 27814 27815 28083

EOG5WSTS5

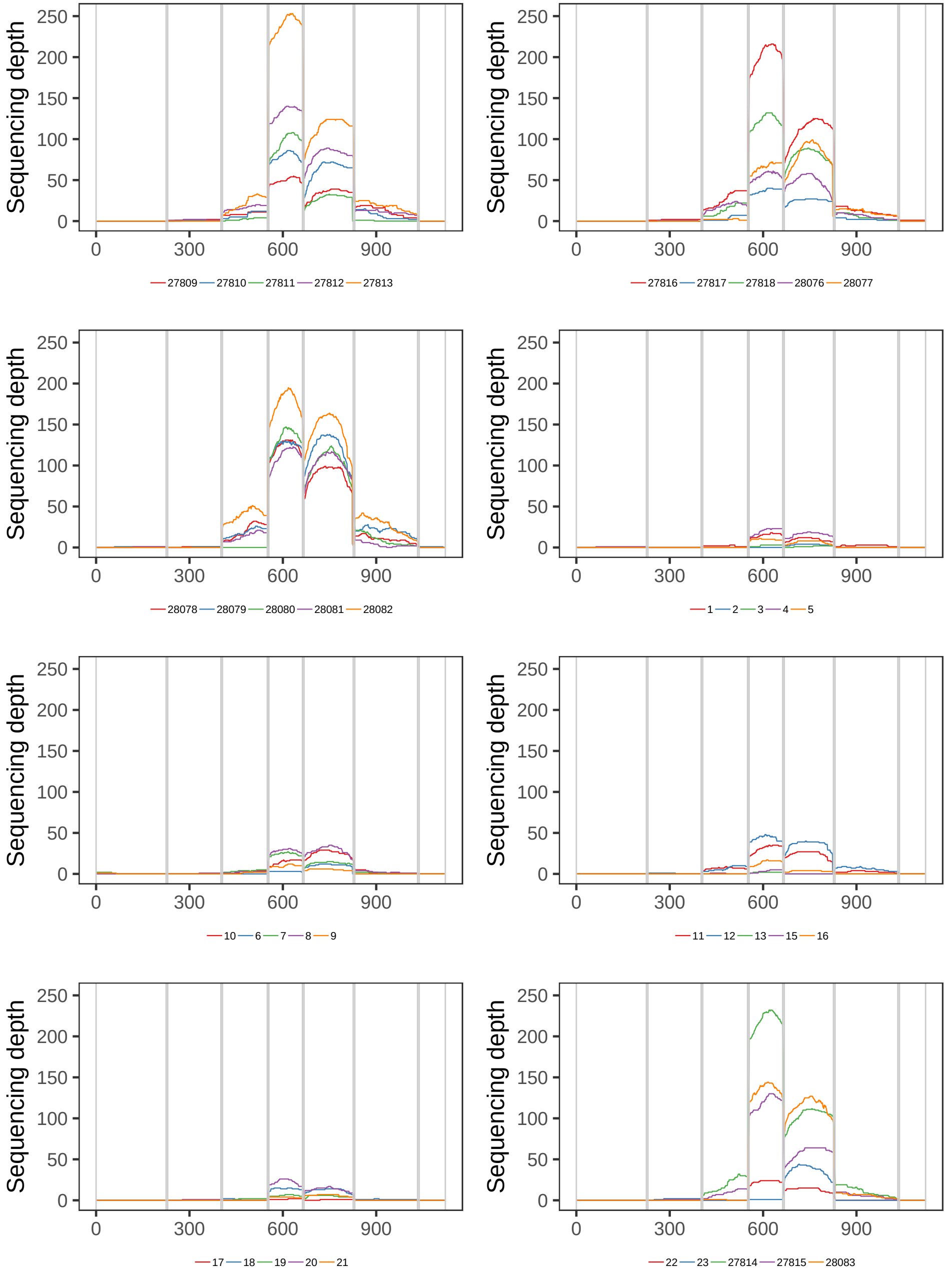

Sequencing depth
250
200
150
100
50
0
0
300
600
900
27809 27810 27811 27812 27813
27816 27817 27818 28076 28077
28078 28079 28080 28081 28082
1 2 3 4 5
10 6 7 8 9
11 12 13 15 16
17 18 19 20 21
22 23 27814 27815 28083

EOG5Z08MJ

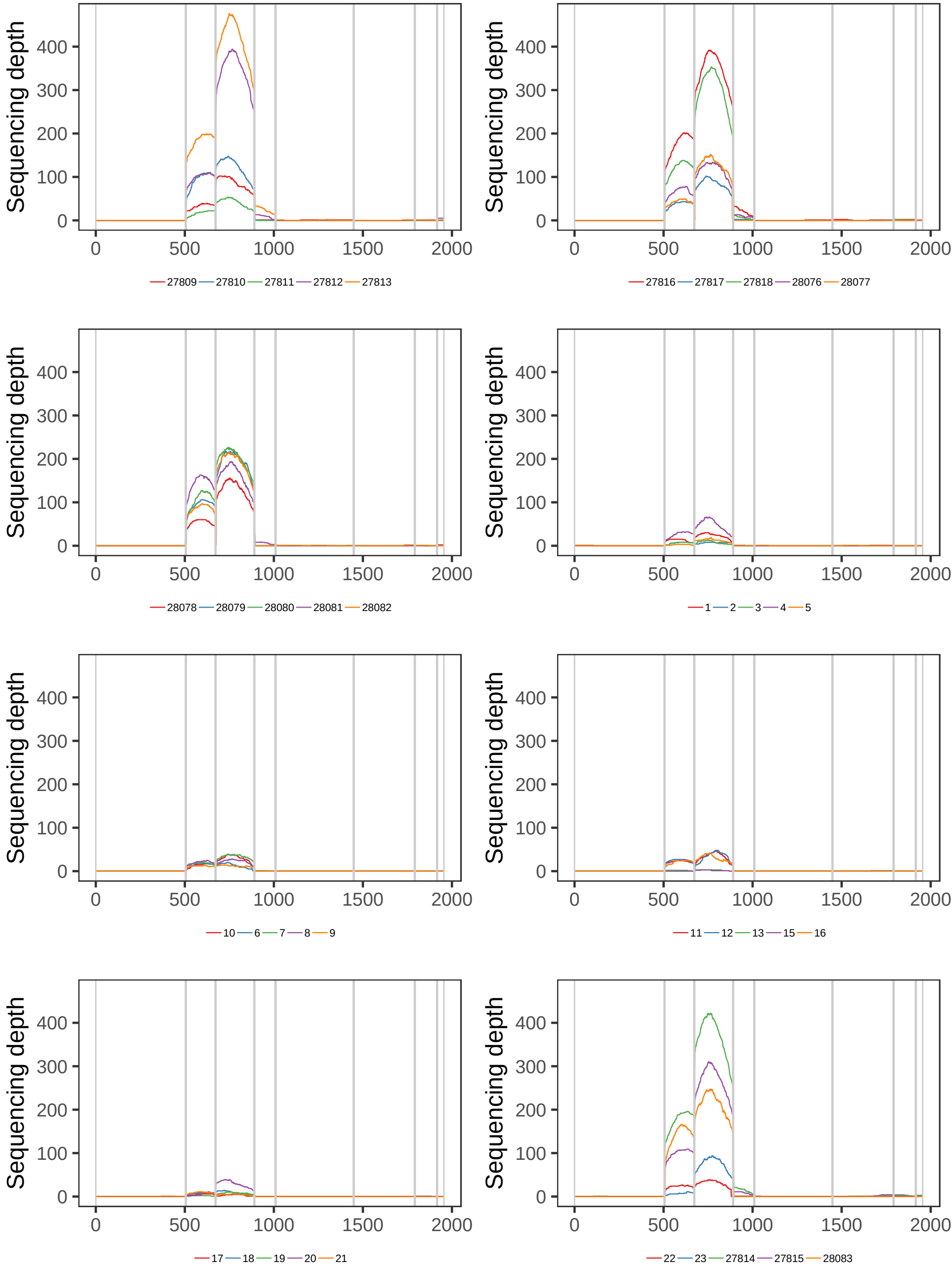
Sequencing depth
0
100
200
300
400
0
500
1000
1500
2000
27809 27810 27811 27812 27813
27816 27817 27818 28076 28077
28078 28079 28080 28081 28082
1 2 3 4 5
10 6 7 8 9
11 12 13 15 16
17 18 19 20 21
22 23 27814 27815 28083

EOG50RXWM

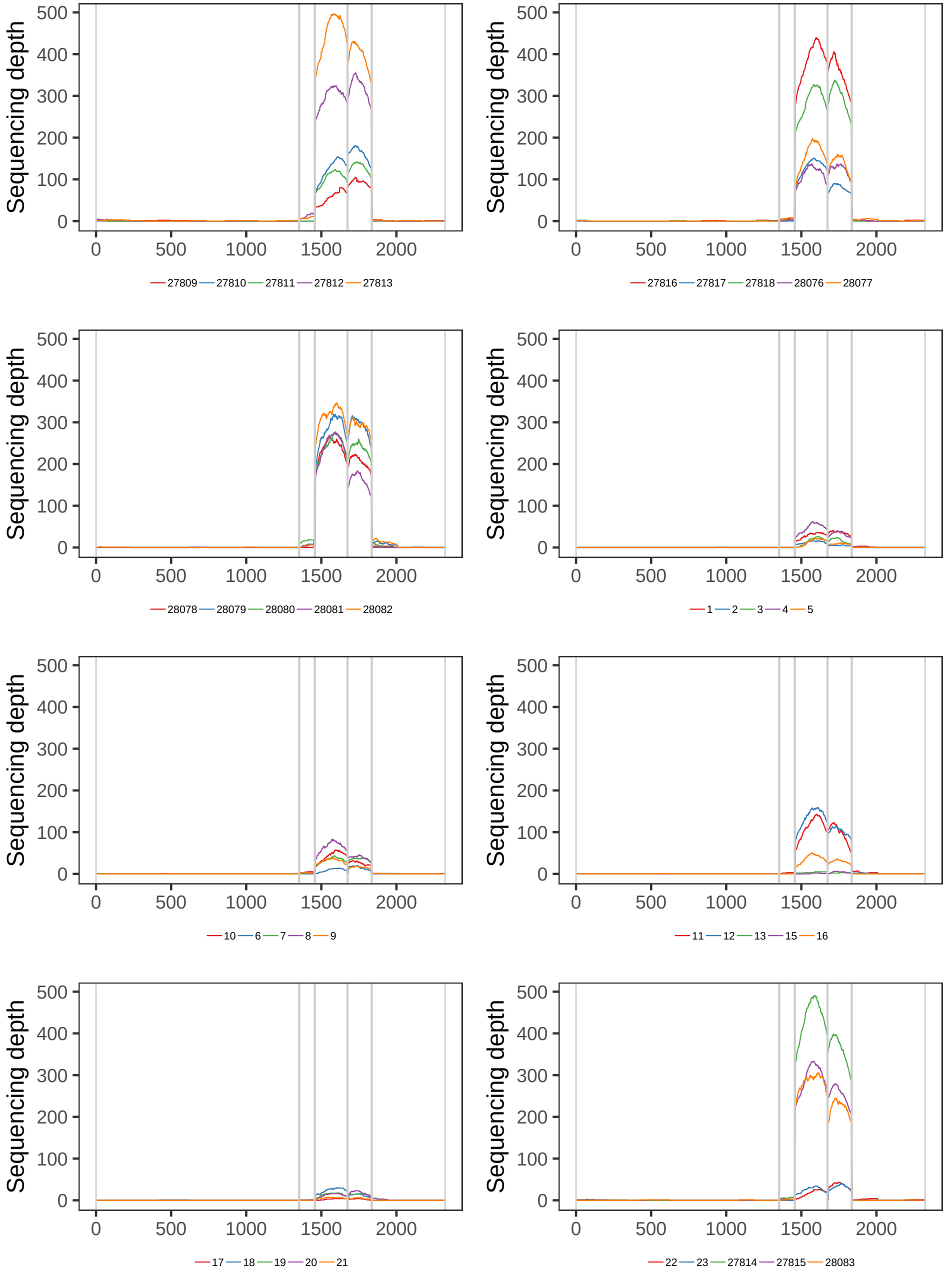

Sequencing depth
0
100
200
300
400
500
0
500
1000
1500
2000
27809 27810 27811 27812 27813
27816 27817 27818 28076 28077
28078 28079 28080 28081 28082
1 2 3 4 5
10 6 7 8 9
11 12 13 15 16
17 18 19 20 21
22 23 27814 27815 28083

EOG50RXX6

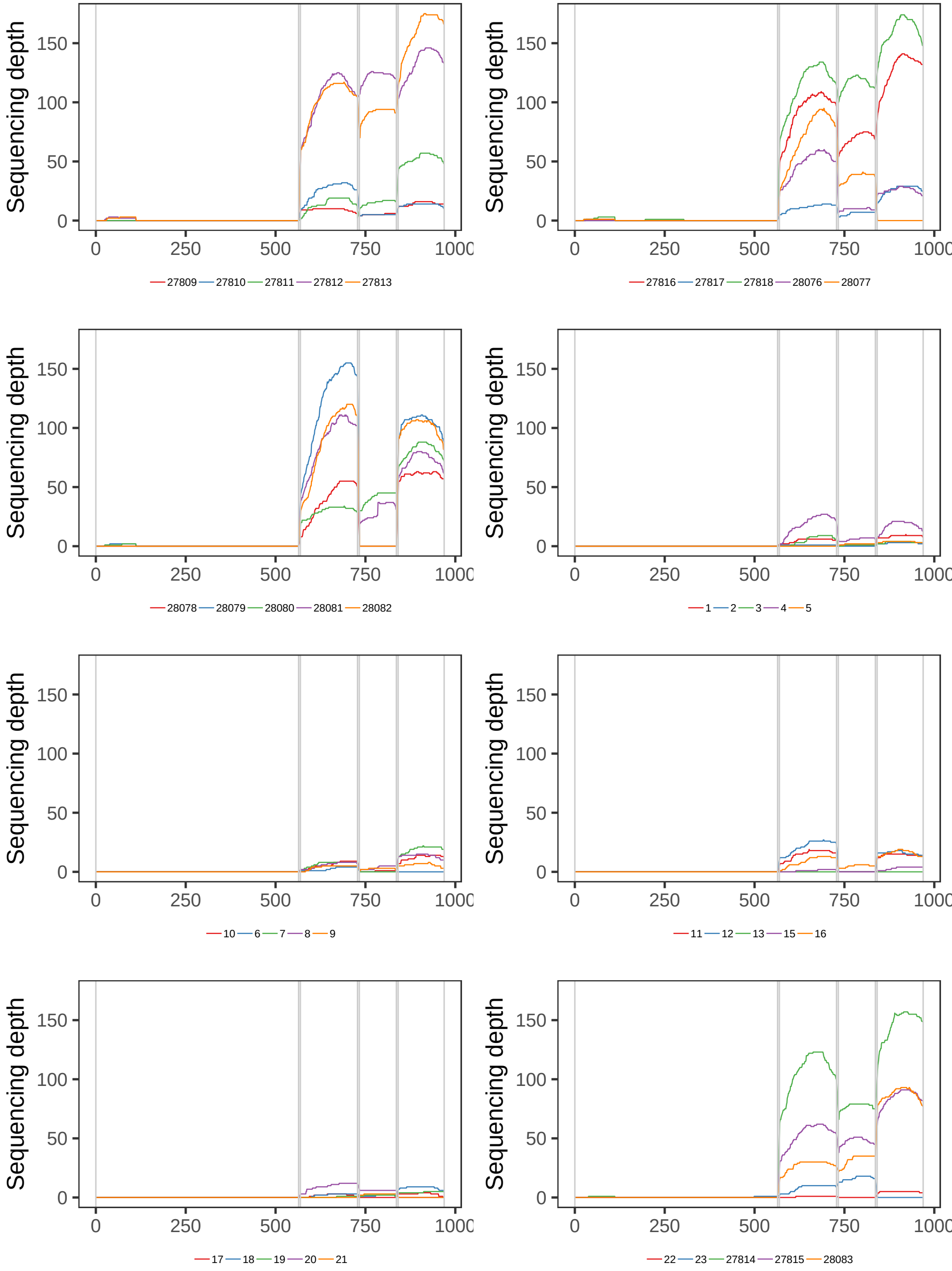

Sequencing depth
0
50
100
150
0
250
500
750
1000
27809 27810 27811 27812 27813
27816 27817 27818 28076 28077
28078 28079 28080 28081 28082
1 2 3 4 5
10 6 7 8 9
11 12 13 15 16
17 18 19 20 21
22 23 27814 27815 28083

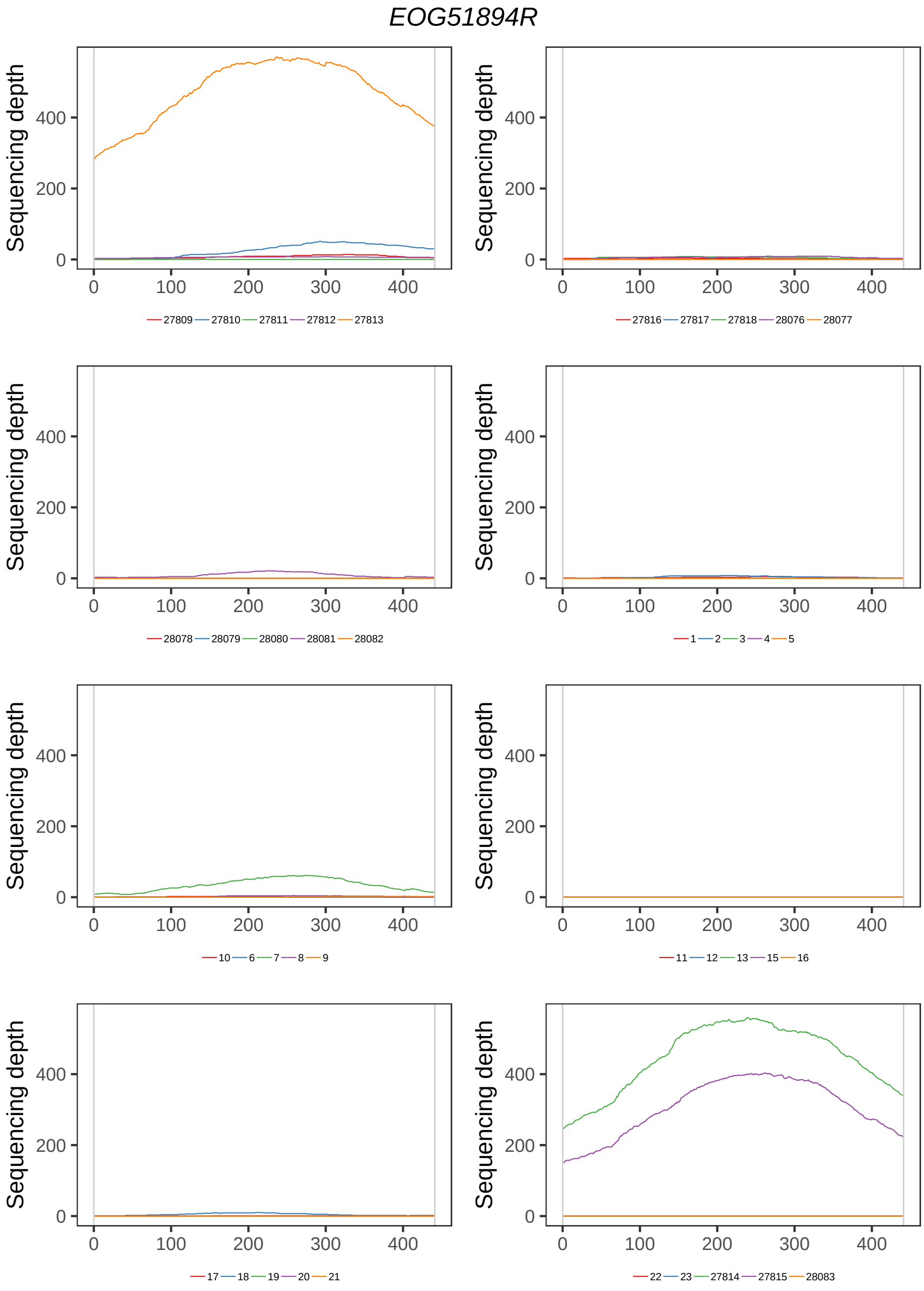
EOG51894R
Sequencing depth
0
200
400
0
100
200
300
400
27809
27810
27811
27812
27813
27816
27817
27818
28076
28077
28078
28079
28080
28081
28082
1
2
3
4
5
10
6
7
8
9
11
12
13
15
16
17
18
19
20
21
22
23
27814
27815
28083

EOG5280GK

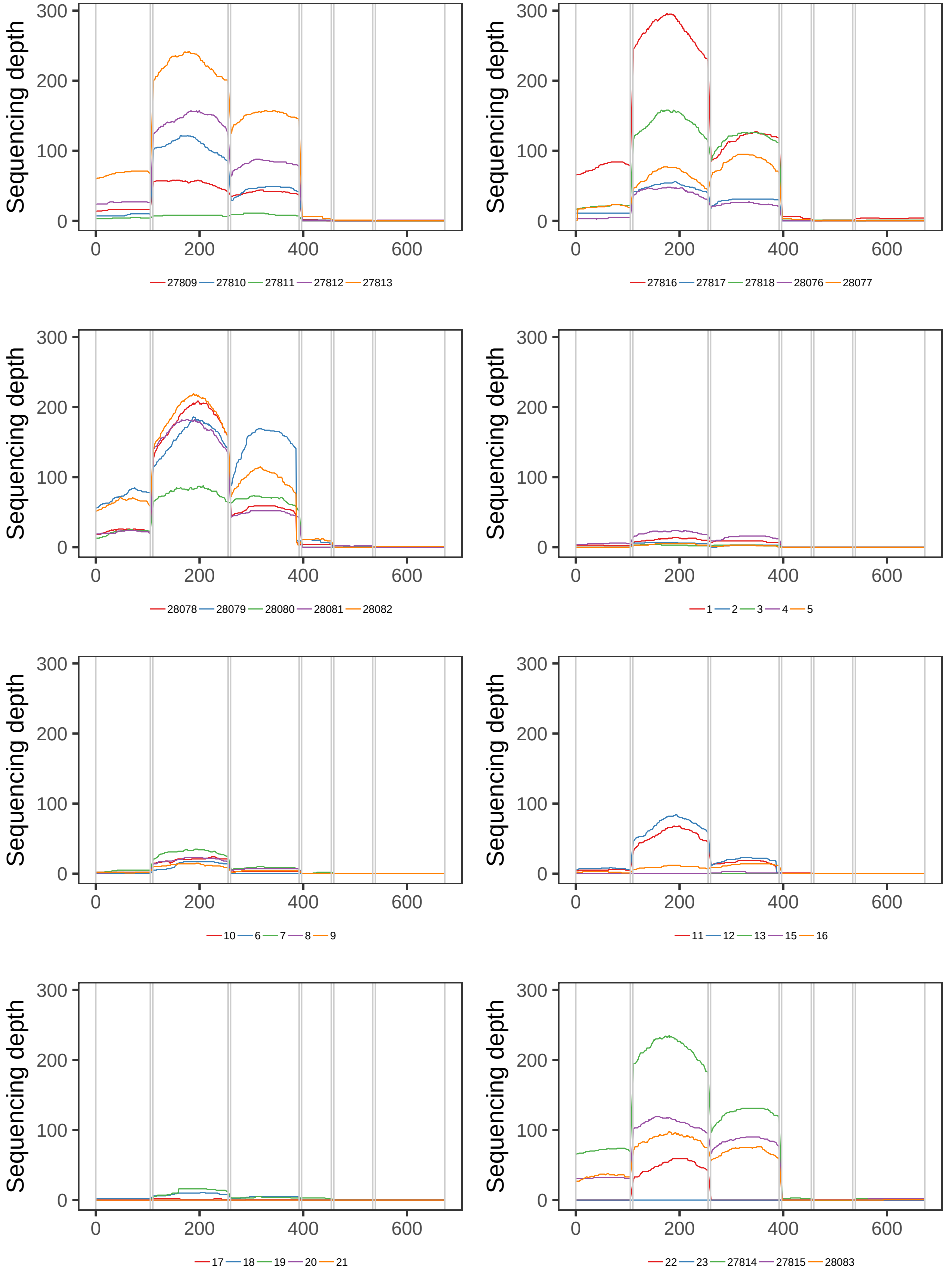

Sequencing depth
0
100
200
300
0
200
400
600
27809 27810 27811 27812 27813
27816 27817 27818 28076 28077
28078 28079 28080 28081 28082
1 2 3 4 5
10 6 7 8 9
11 12 13 15 16
17 18 19 20 21
22 23 27814 27815 28083

EOG52Z36M

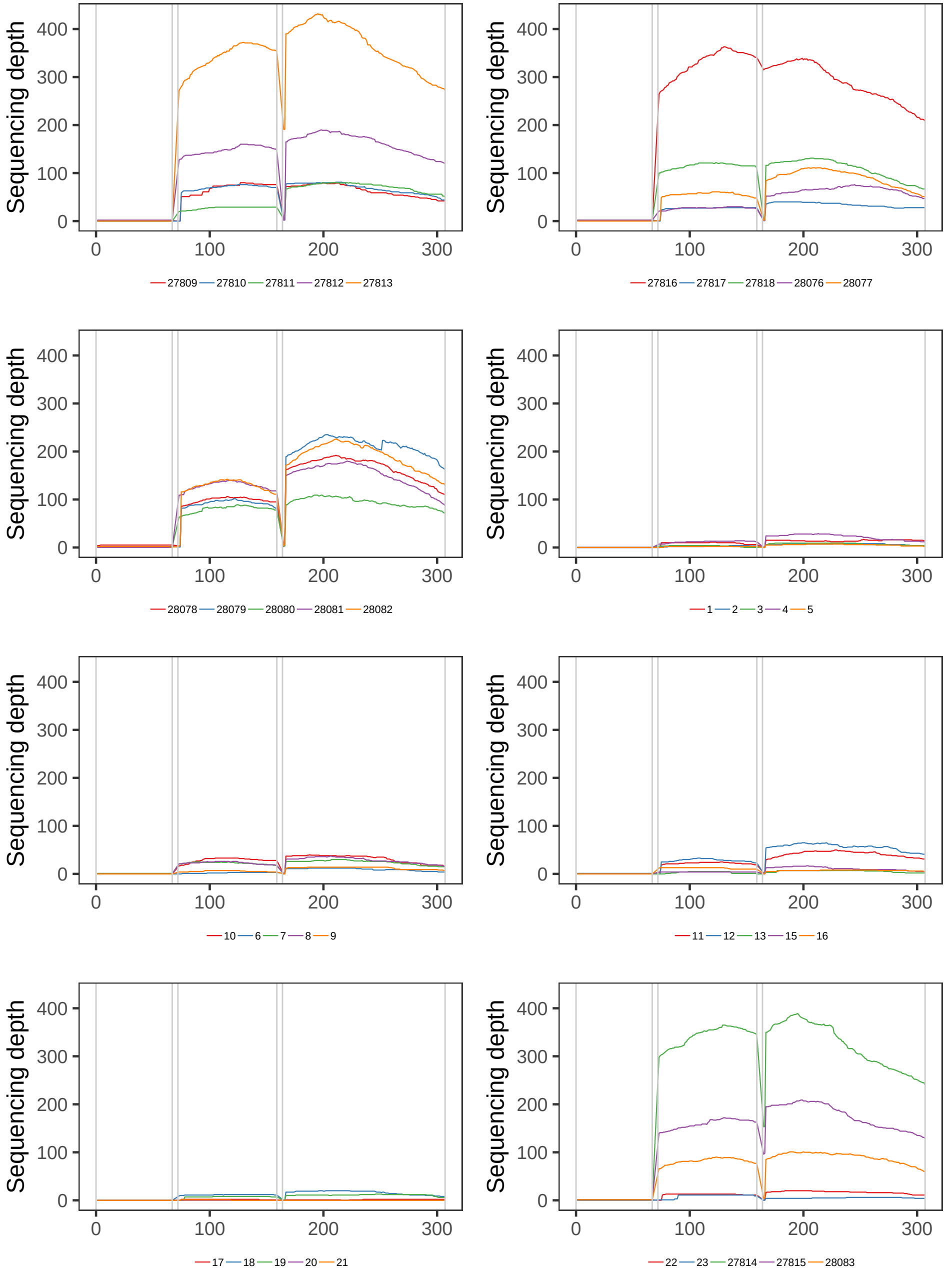
Sequencing depth
0
100
200
300
400
27809
27810
27811
27812
27813
27816
27817
27818
28076
28077
28078
28079
28080
28081
28082
1
2
3
4
5
10
6
7
8
9
11
12
13
15
16
17
18
19
20
21
22
23
27814
27815
28083

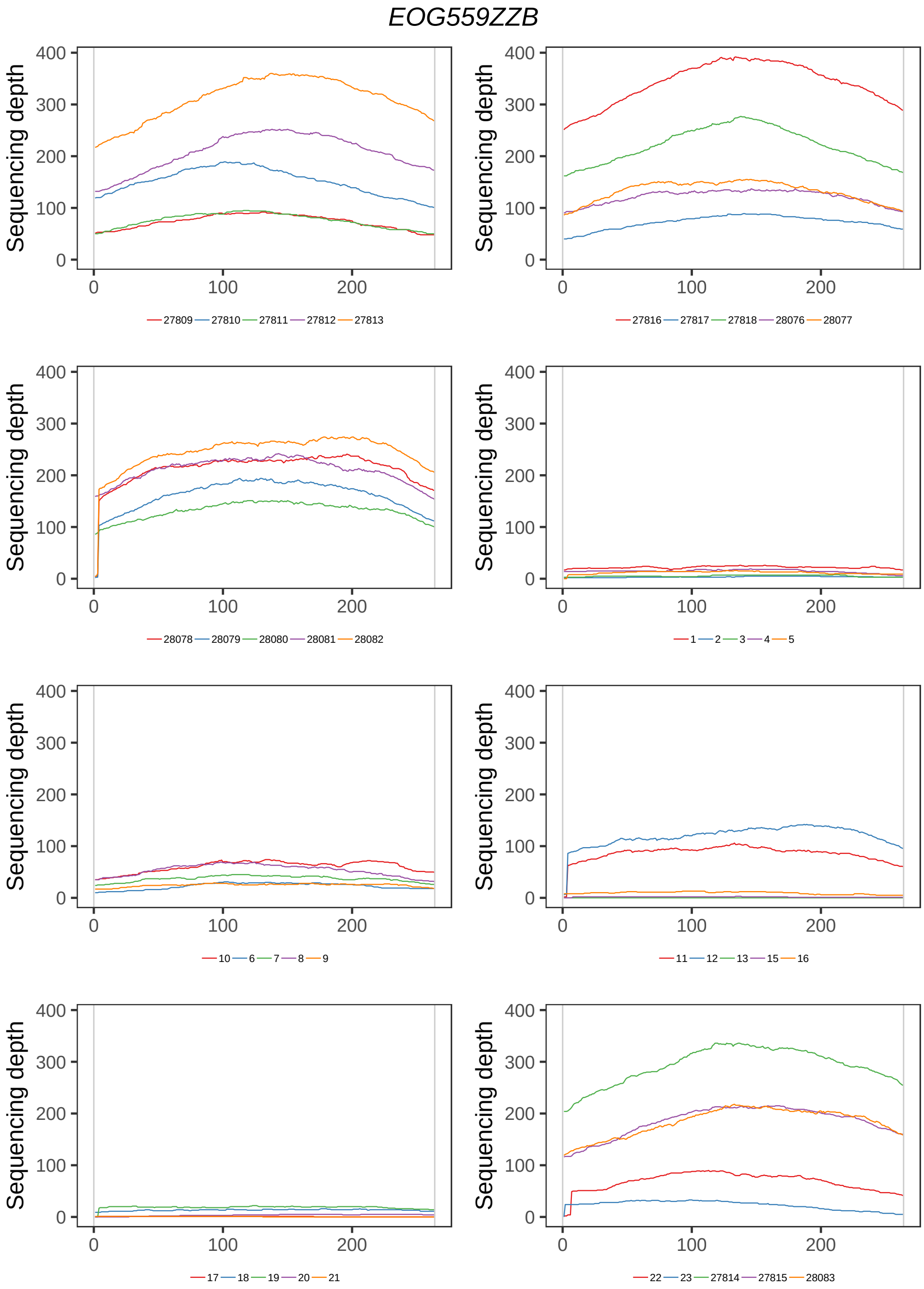
EOG559ZZB
Sequencing depth
27809 27810 27811 27812 27813
27816 27817 27818 28076 28077
28078 28079 28080 28081 28082
1 2 3 4 5
10 6 7 8 9
11 12 13 15 16
17 18 19 20 21
22 23 27814 27815 28083

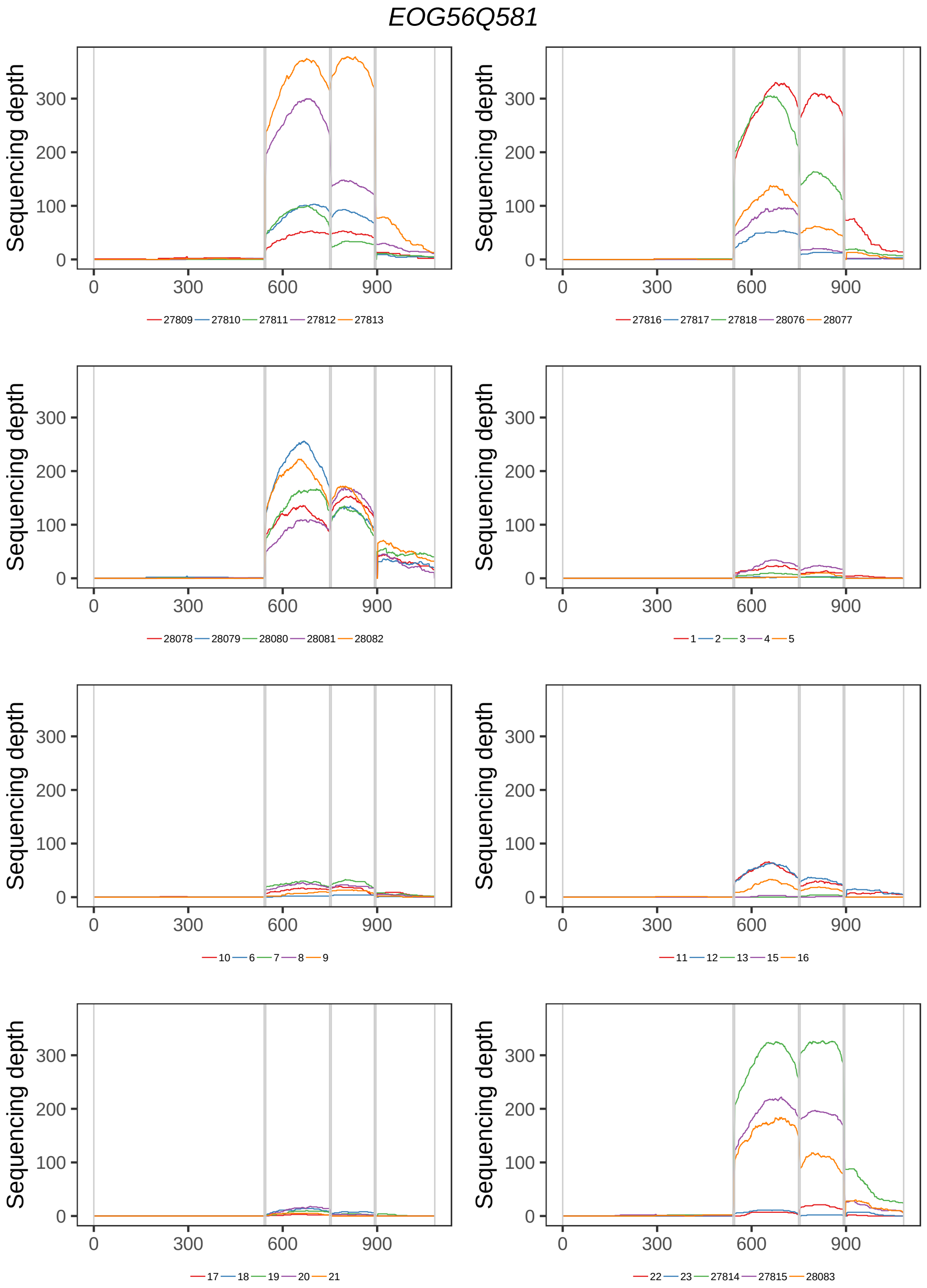
EOG56Q581
Sequencing depth
0
100
200
300
0
300
600
900
27809
27810
27811
27812
27813
27816
27817
27818
28076
28077
28078
28079
28080
28081
28082
1
2
3
4
5
10
6
7
8
9
11
12
13
15
16
17
18
19
20
21
22
23
27814
27815
28083

EOG59ZW4H

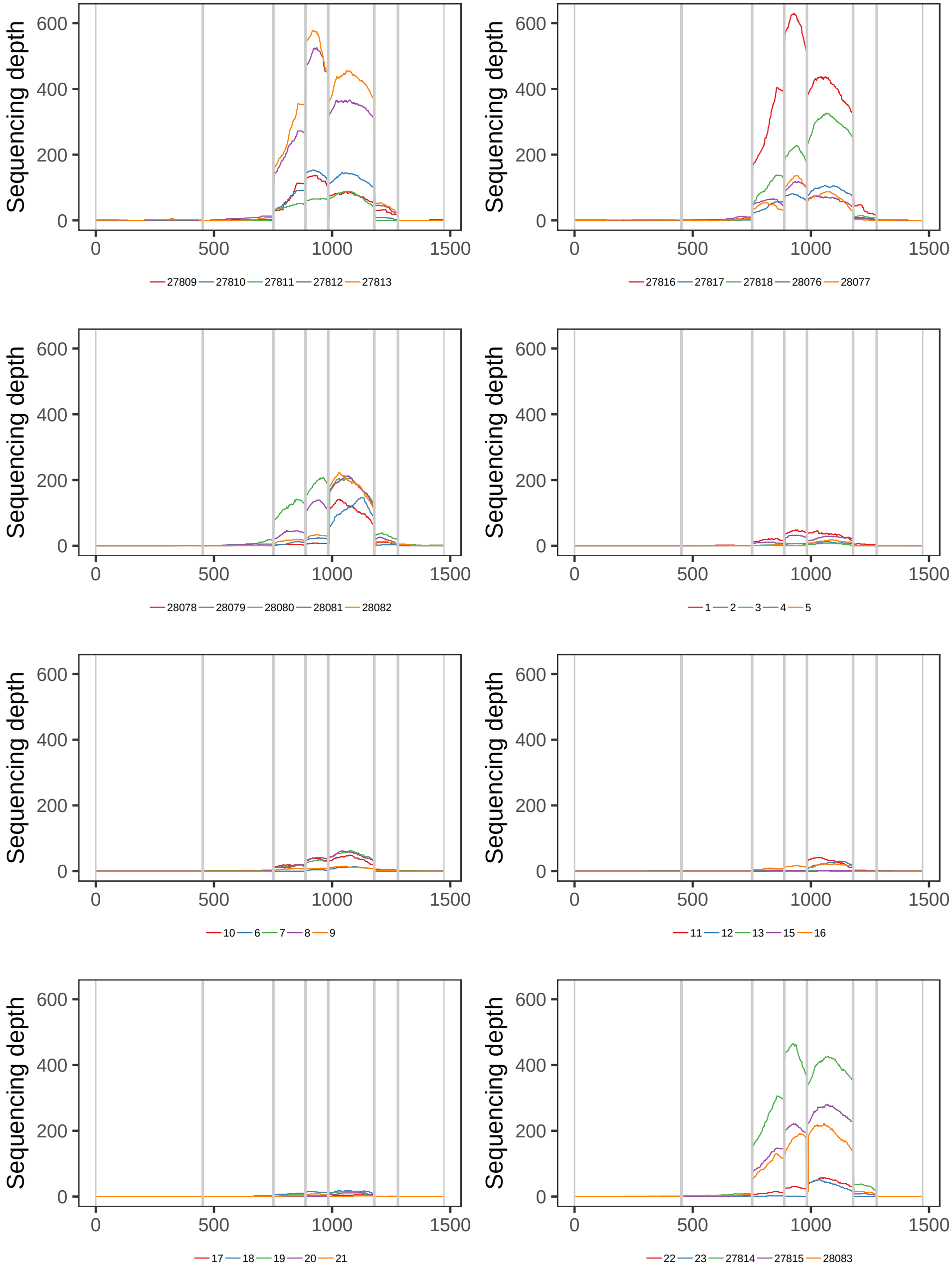

Sequencing depth
600
400
200
0
0
500
1000
1500
27809
27810
27811
27812
27813
27816
27817
27818
28076
28077
28078
28079
28080
28081
28082
1
2
3
4
5
10
6
7
8
9
11
12
13
15
16
17
18
19
20
21
22
23
27814
27815
28083

EOG5CNP70

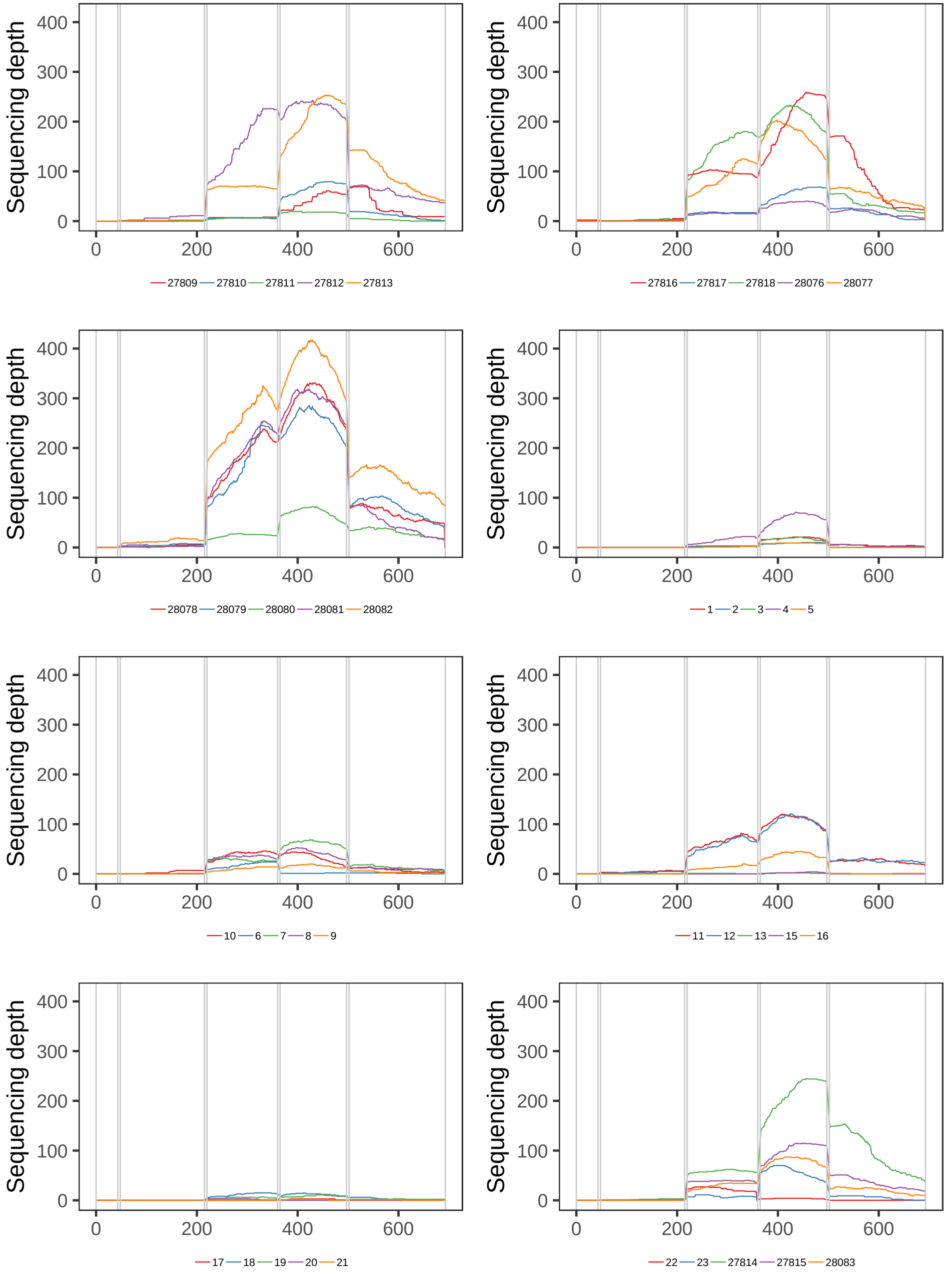
Sequencing depth
0
100
200
300
400
0
200
400
600
27809 27810 27811 27812 27813
27816 27817 27818 28076 28077
28078 28079 28080 28081 28082
1 2 3 4 5
10 6 7 8 9
11 12 13 15 16
17 18 19 20 21
22 23 27814 27815 28083

EOG5MSBDQ

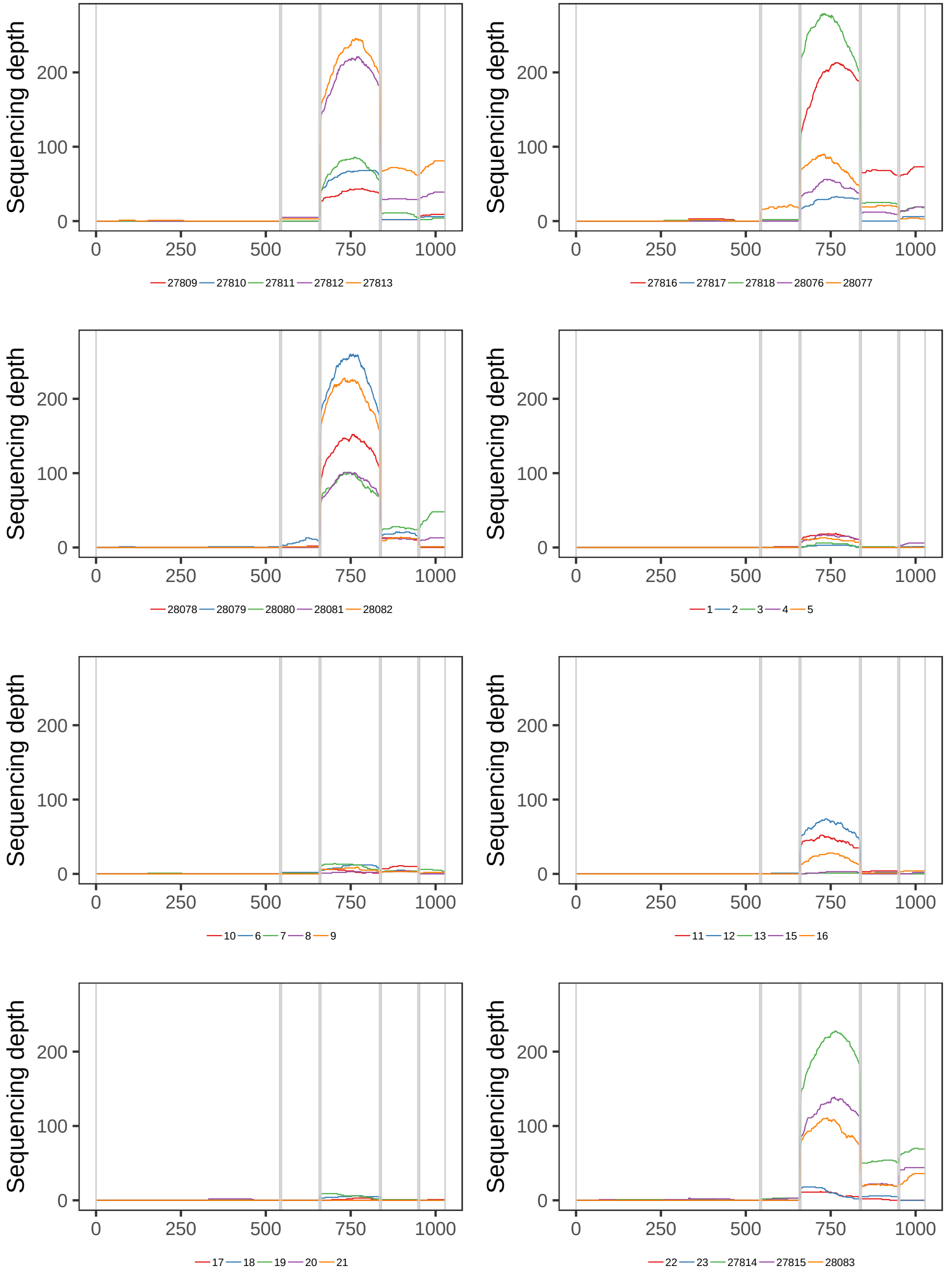
Sequencing depth
0
100
200
0
250
500
750
1000
27809 27810 27811 27812 27813
27816 27817 27818 28076 28077
28078 28079 28080 28081 28082
1 2 3 4 5
10 6 7 8 9
11 12 13 15 16
17 18 19 20 21
22 23 27814 27815 28083

EOG5NK9BM

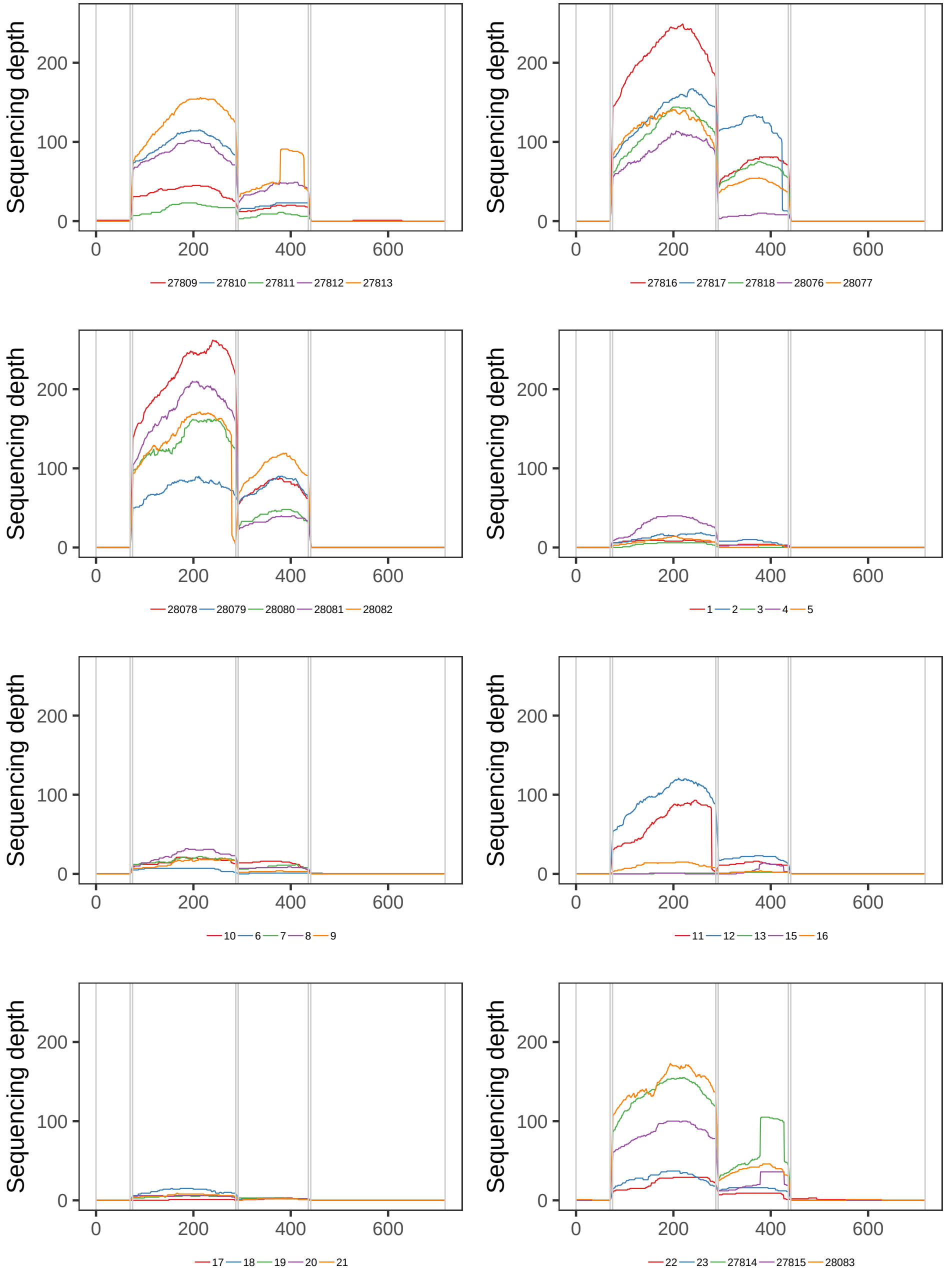

Sequencing depth
0
100
200
0
200
400
600
27809
27810
27811
27812
27813
27816
27817
27818
28076
28077
28078
28079
28080
28081
28082
1
2
3
4
5
10
6
7
8
9
11
12
13
15
16
17
18
19
20
21
22
23
27814
27815
28083

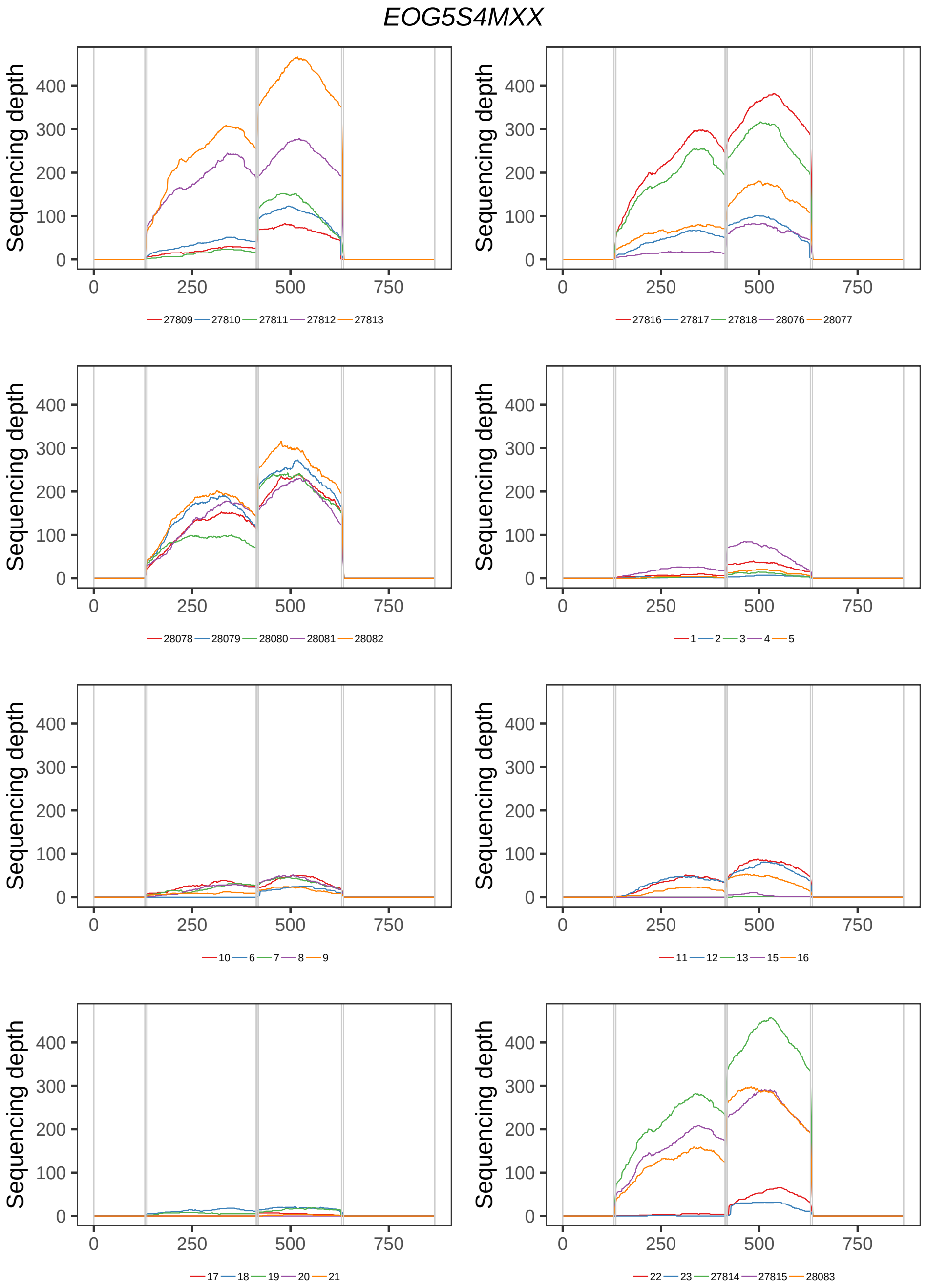
EOG5S4MXX
Sequencing depth
0
100
200
300
400
0
250
500
750
27809
27810
27811
27812
27813
27816
27817
27818
28076
28077
28078
28079
28080
28081
28082
1
2
3
4
5
10
6
7
8
9
11
12
13
15
16
17
18
19
20
21
22
23
27814
27815
28083

EOG5V15HC

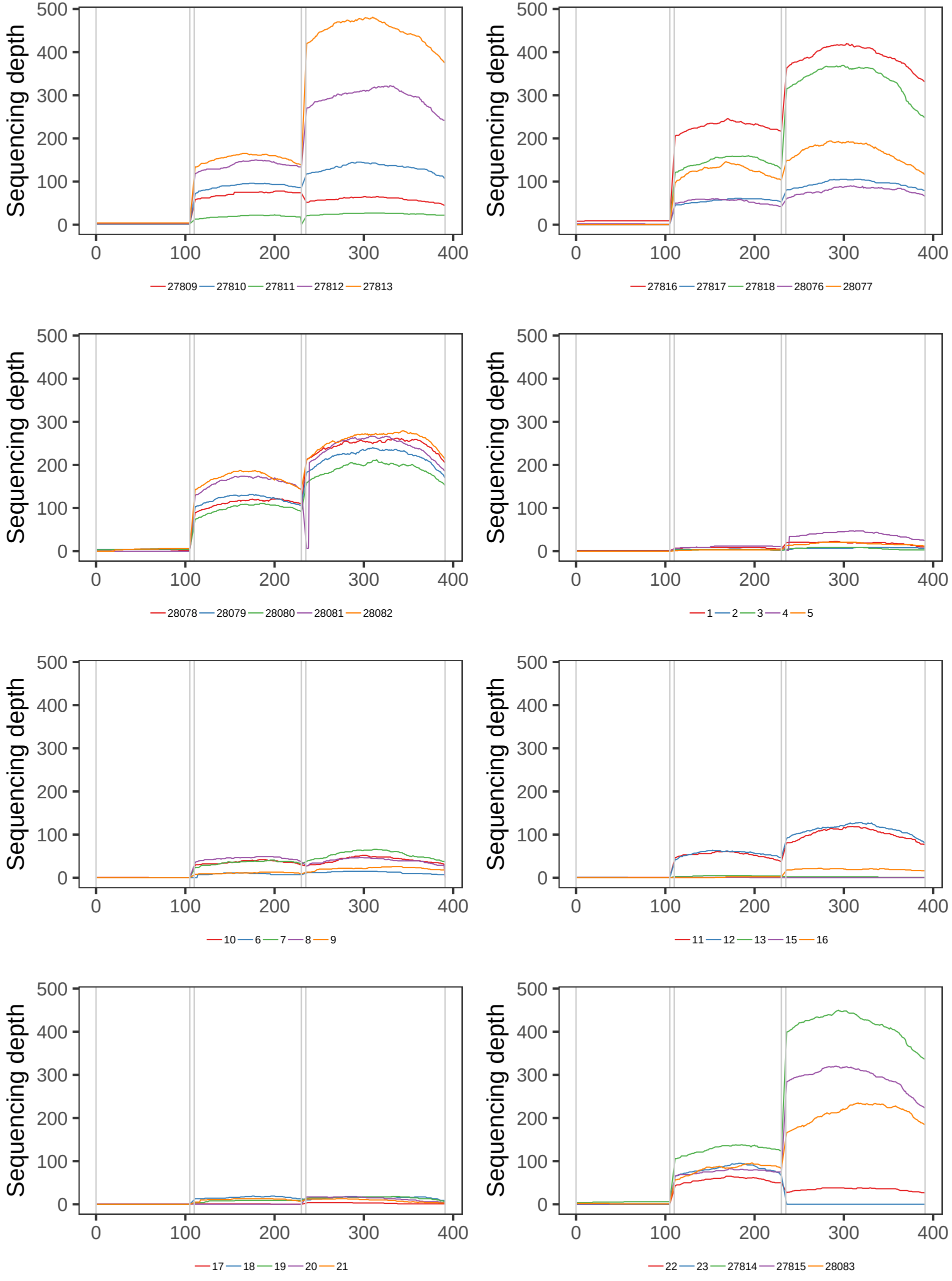
Sequencing depth
0
100
200
300
400
500
27809
27810
27811
27812
27813
27816
27817
27818
28076
28077
28078
28079
28080
28081
28082
1
2
3
4
5
10
6
7
8
9
11
12
13
15
16
17
18
19
20
21
22
23
27814
27815
28083

EOG5VT4C5

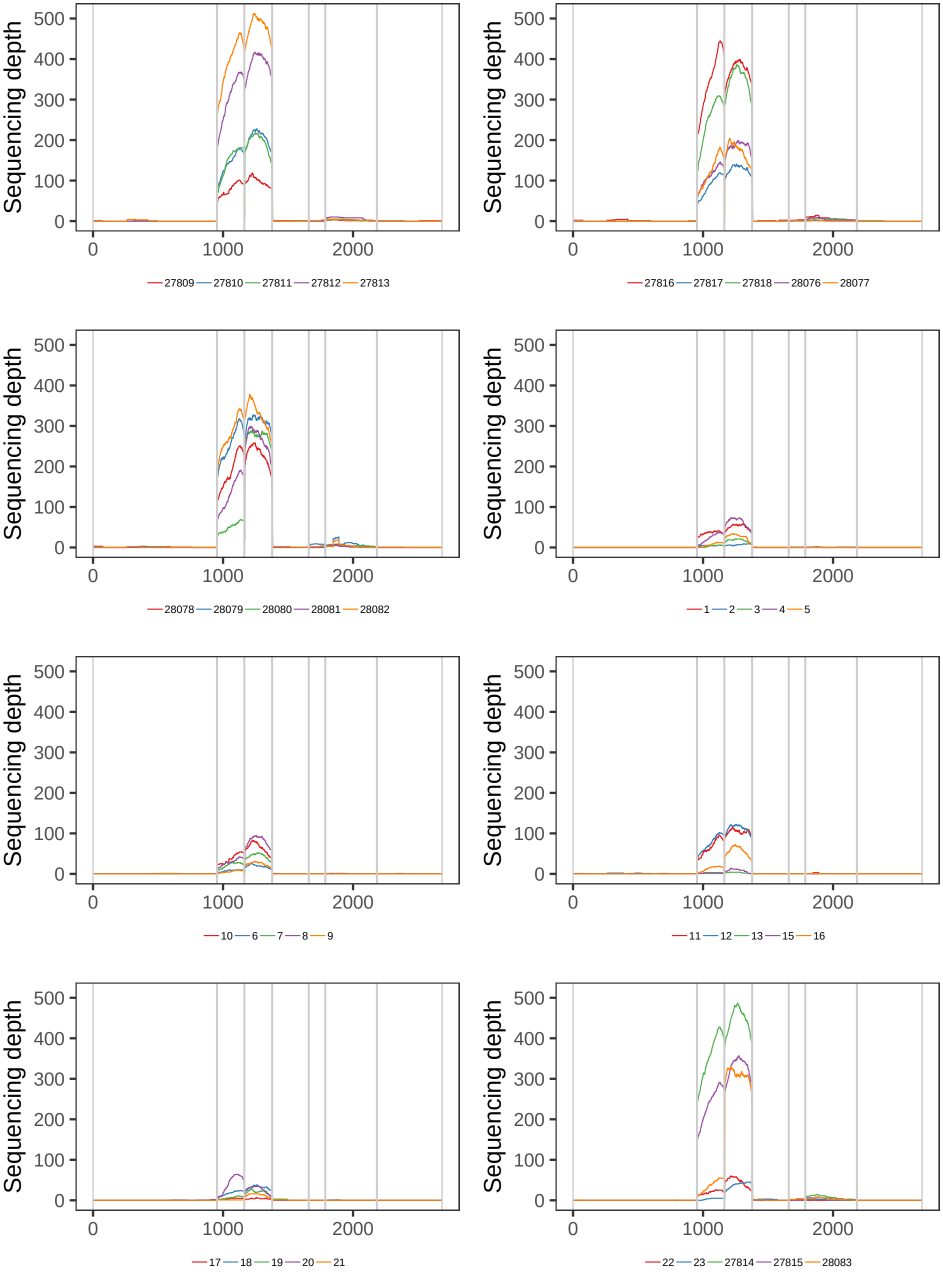
Sequencing depth
0
100
200
300
400
500
0
1000
2000
27809 27810 27811 27812 27813
27816 27817 27818 28076 28077
28078 28079 28080 28081 28082
1 2 3 4 5
10 6 7 8 9
11 12 13 15 16
17 18 19 20 21
22 23 27814 27815 28083

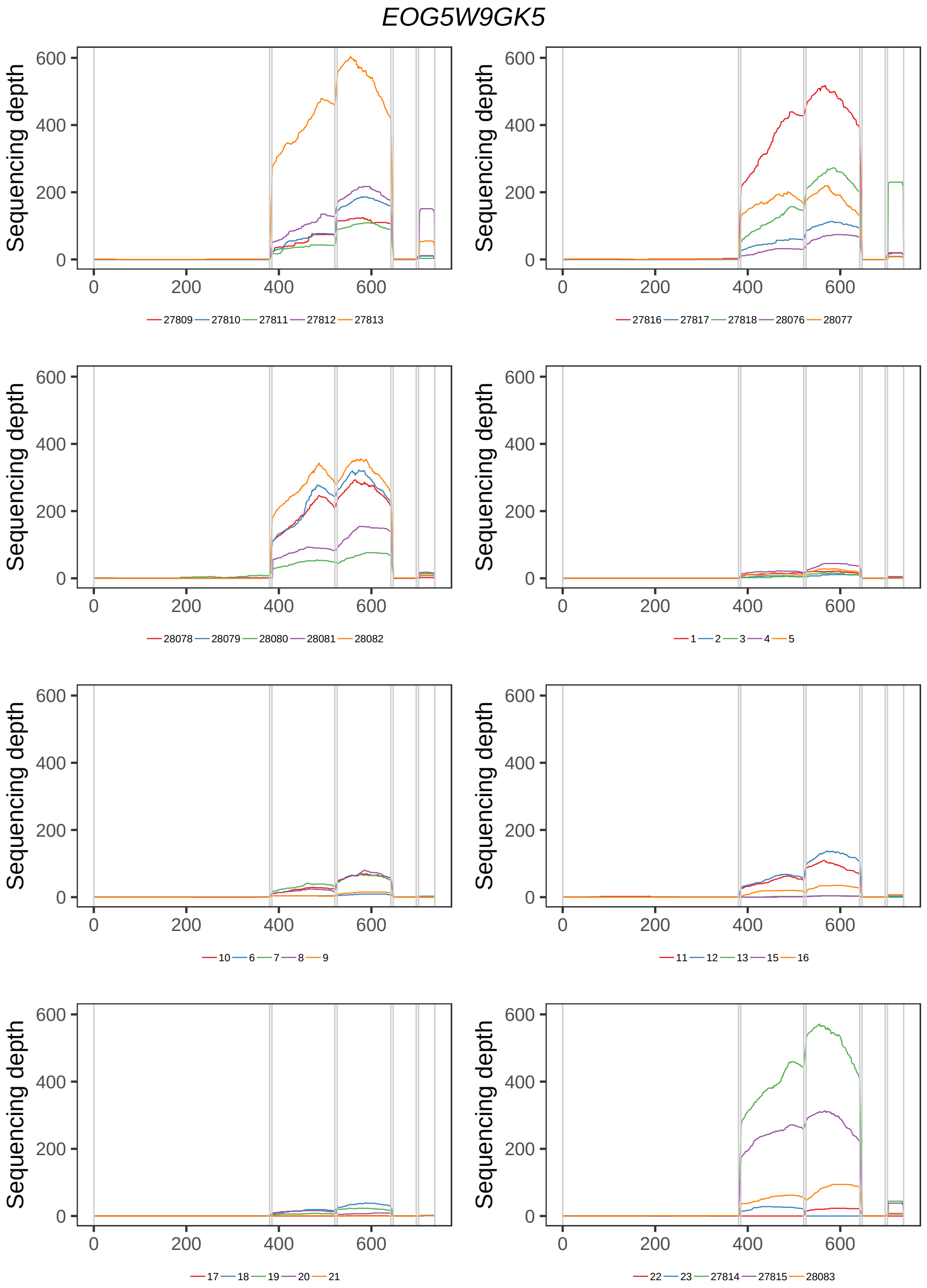
EOG5W9GK5
Sequencing depth
27809 27810 27811 27812 27813
27816 27817 27818 28076 28077
28078 28079 28080 28081 28082
1 2 3 4 5
10 6 7 8 9
11 12 13 15 16
17 18 19 20 21
22 23 27814 27815 28083

EOG5XPNZF

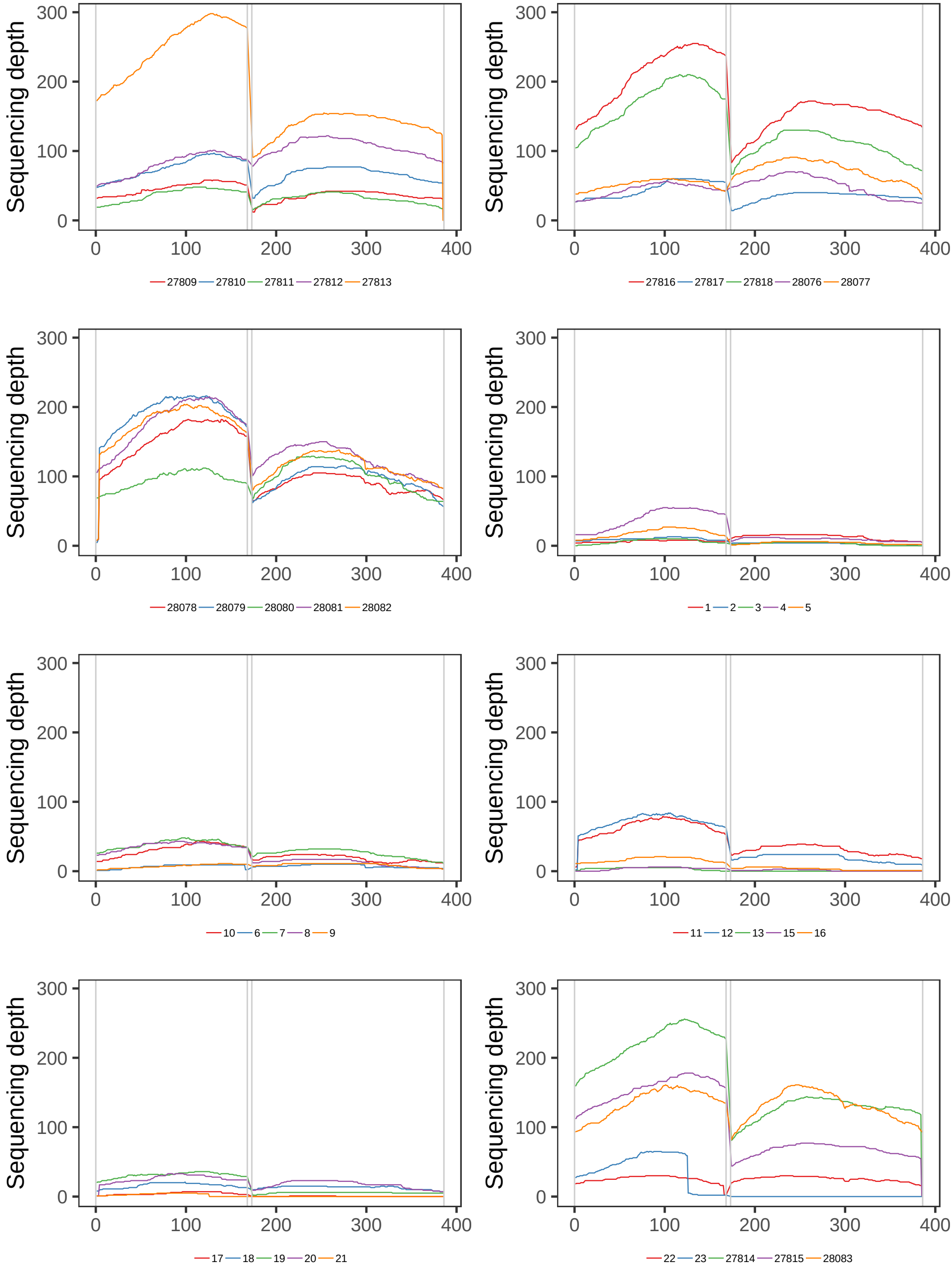

Sequencing depth
300
200
100
0
0
100
200
300
400
27809
27810
27811
27812
27813
27816
27817
27818
28076
28077
28078
28079
28080
28081
28082
1
2
3
4
5
10
6
7
8
9
11
12
13
15
16
17
18
19
20
21
22
23
27814
27815
28083

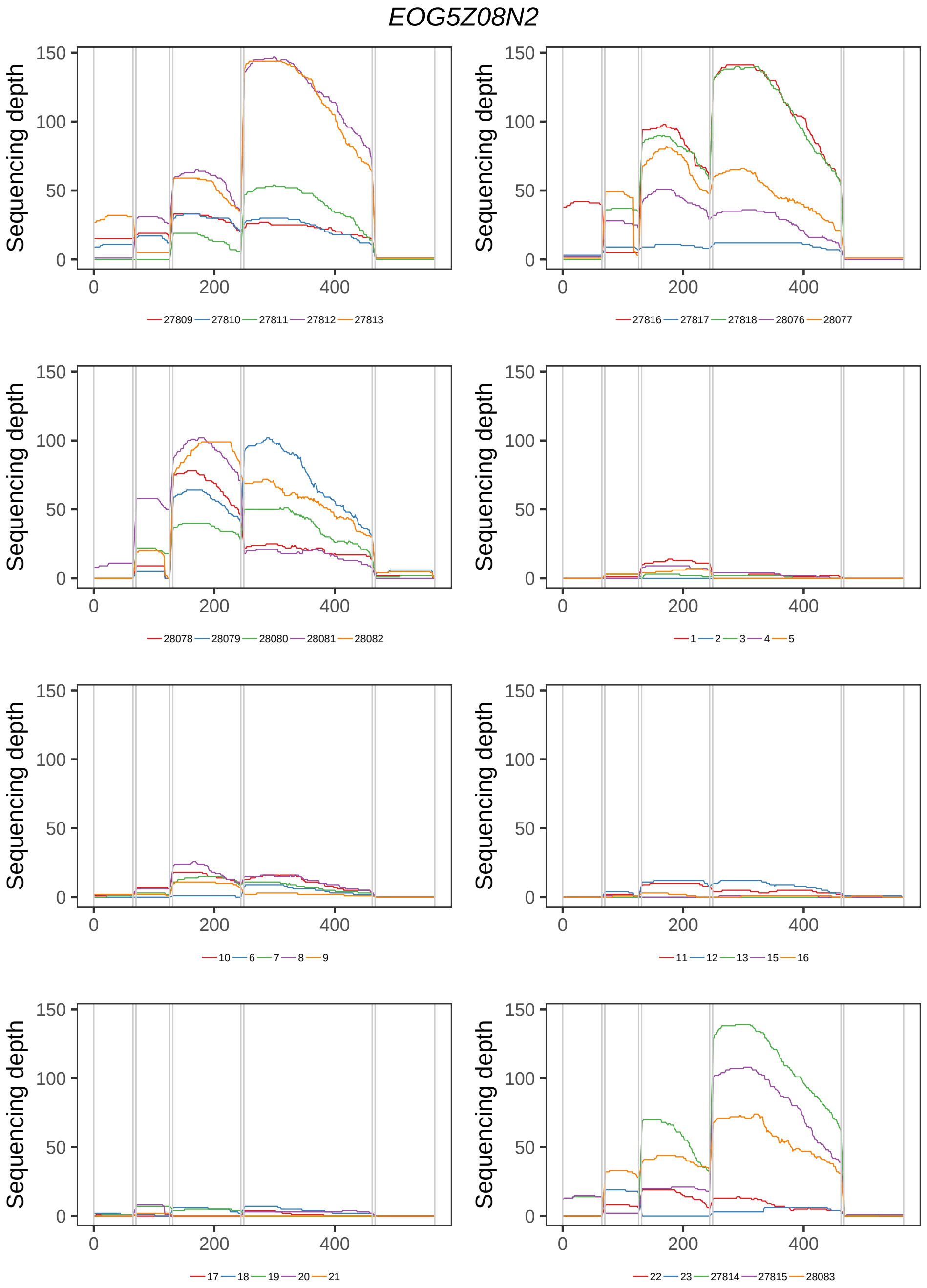
EOG5Z08N2
Sequencing depth
150
100
50
0
0
200
400
27809
27810
27811
27812
27813
27816
27817
27818
28076
28077
28078
28079
28080
28081
28082
1
2
3
4
5
10
6
7
8
9
11
12
13
15
16
17
18
19
20
21
22
23
27814
27815
28083

EOG525481

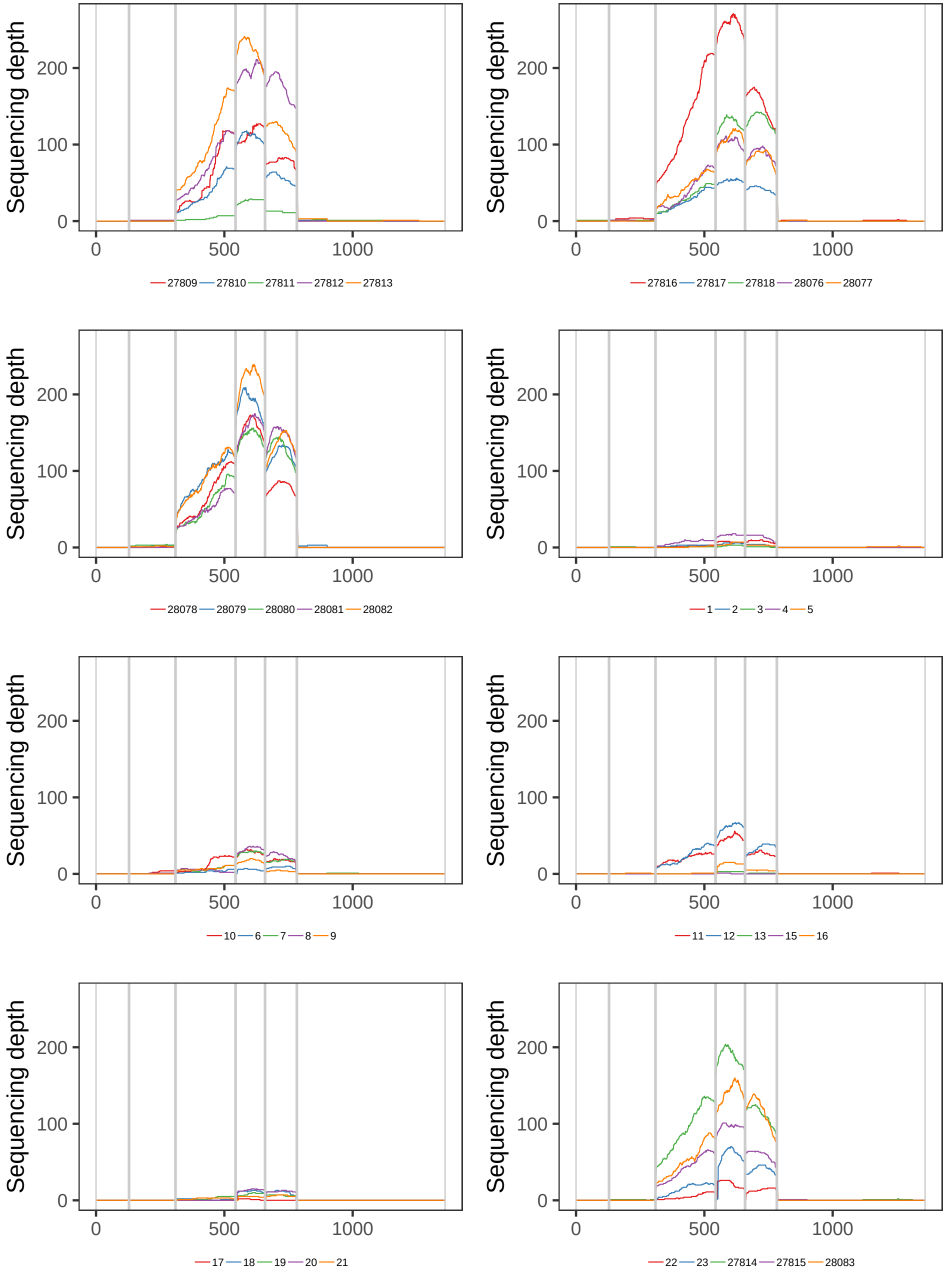
Sequencing depth
0
100
200
0
500
1000
27809 27810 27811 27812 27813
27816 27817 27818 28076 28077
28078 28079 28080 28081 28082
1 2 3 4 5
10 6 7 8 9
11 12 13 15 16
17 18 19 20 21
22 23 27814 27815 28083

EOG537PX3

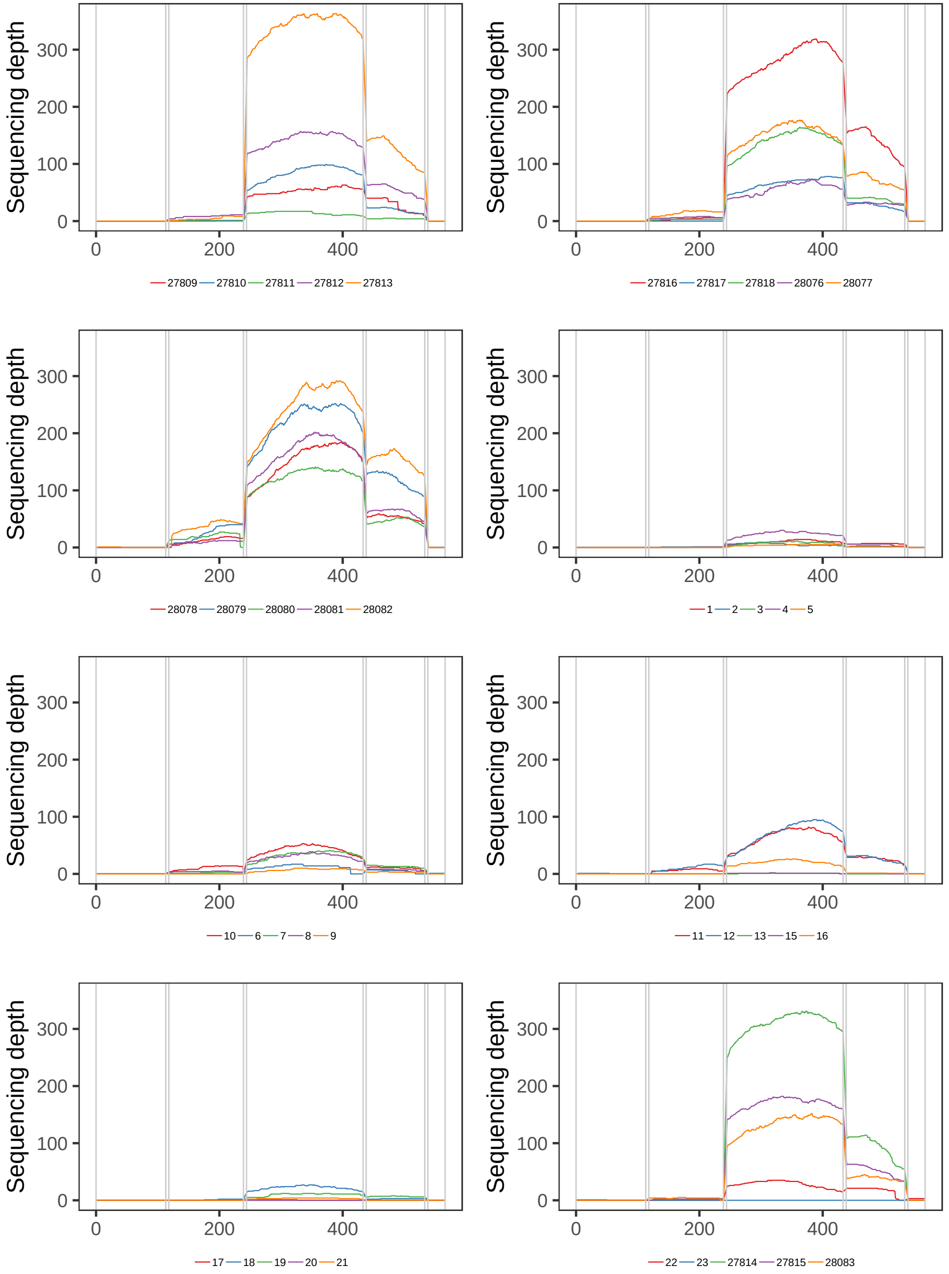
Sequencing depth
0
100
200
300
0
200
400
27809
27810
27811
27812
27813
27816
27817
27818
28076
28077
28078
28079
28080
28081
28082
1
2
3
4
5
10
6
7
8
9
11
12
13
15
16
17
18
19
20
21
22
23
27814
27815
28083

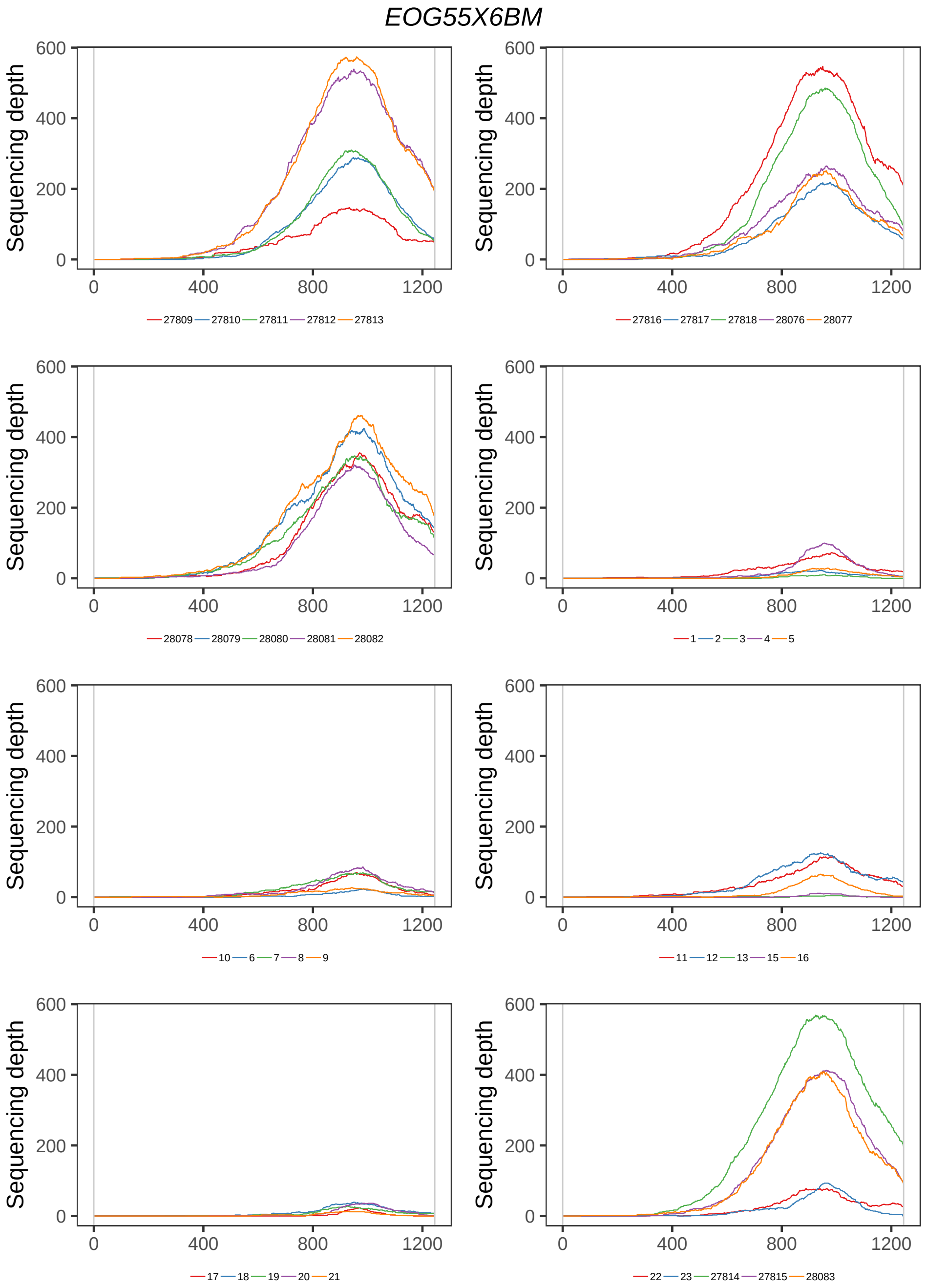
EOG55X6BM
Sequencing depth
0
200
400
600
0
400
800
1200
27809
27810
27811
27812
27813
27816
27817
27818
28076
28077
28078
28079
28080
28081
28082
1
2
3
4
5
10
6
7
8
9
11
12
13
15
16
17
18
19
20
21
22
23
27814
27815
28083

EOG570S0J

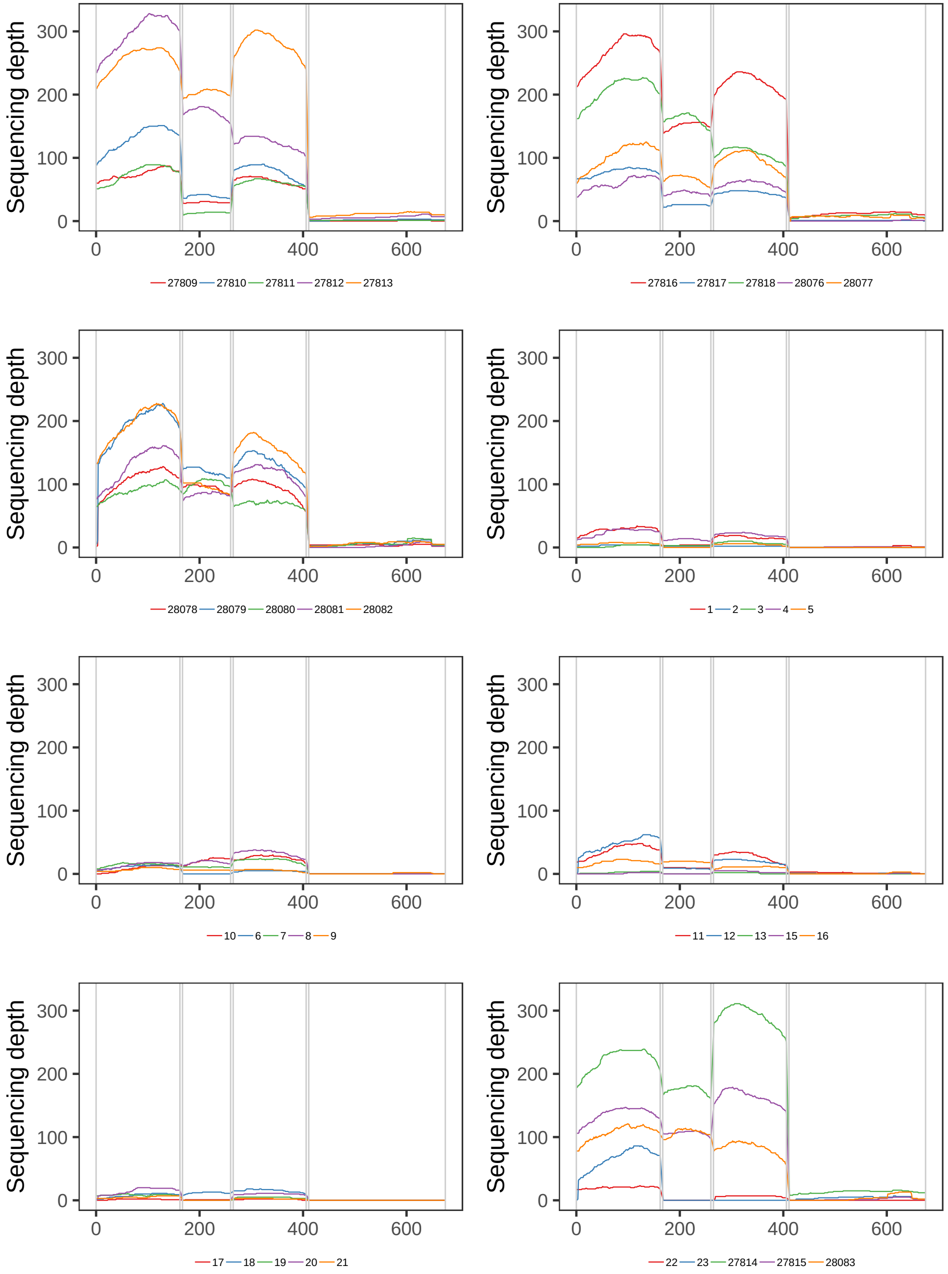
Sequencing depth
0
100
200
300
0
200
400
600
27809 27810 27811 27812 27813
27816 27817 27818 28076 28077
28078 28079 28080 28081 28082
1 2 3 4 5
10 6 7 8 9
11 12 13 15 16
17 18 19 20 21
22 23 27814 27815 28083

EOG59320R

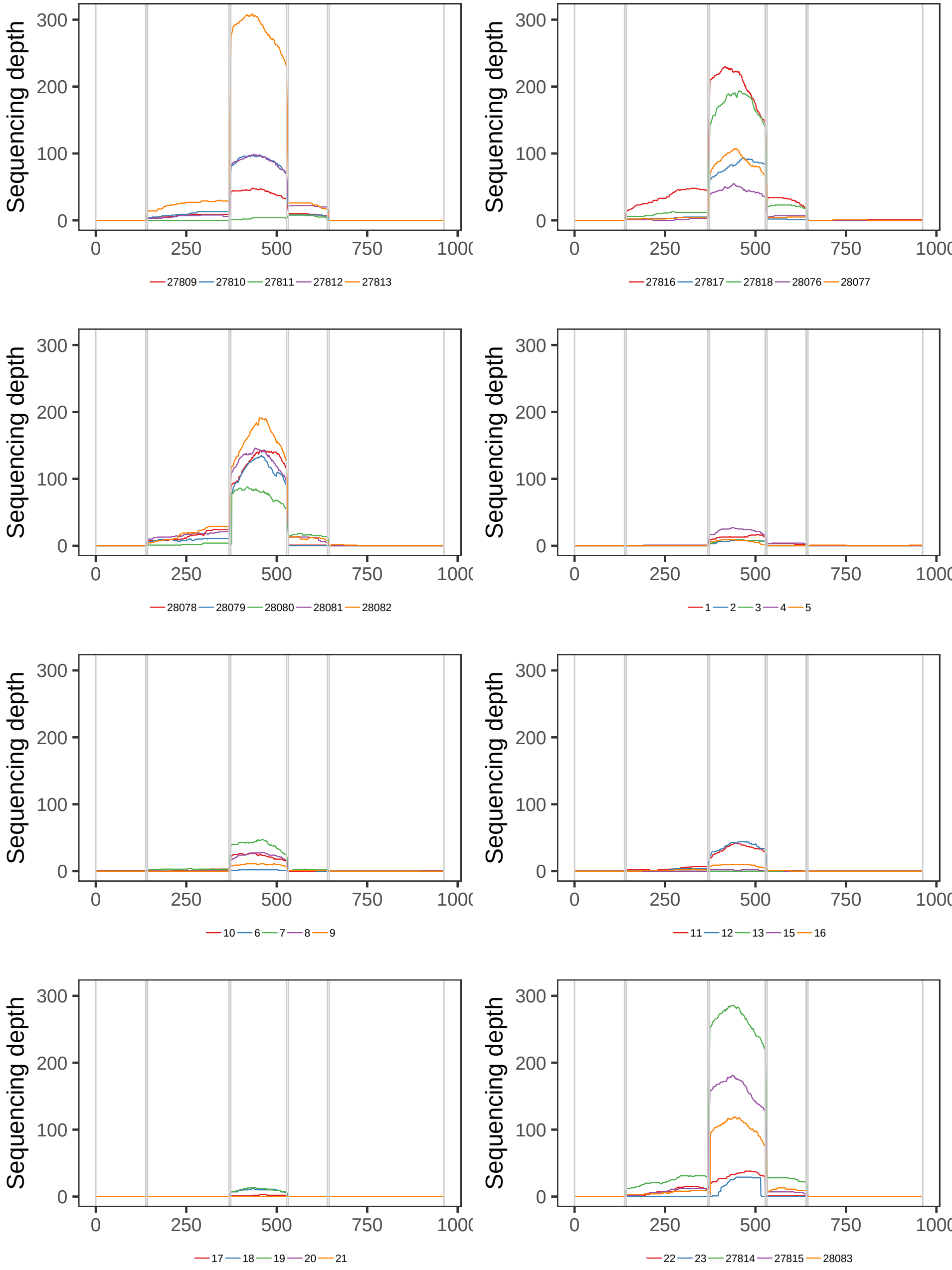
Sequencing depth
300
200
100
0
0
250
500
750
1000
27809 27810 27811 27812 27813
27816 27817 27818 28076 28077
28078 28079 28080 28081 28082
1 2 3 4 5
10 6 7 8 9
11 12 13 15 16
17 18 19 20 21
22 23 27814 27815 28083

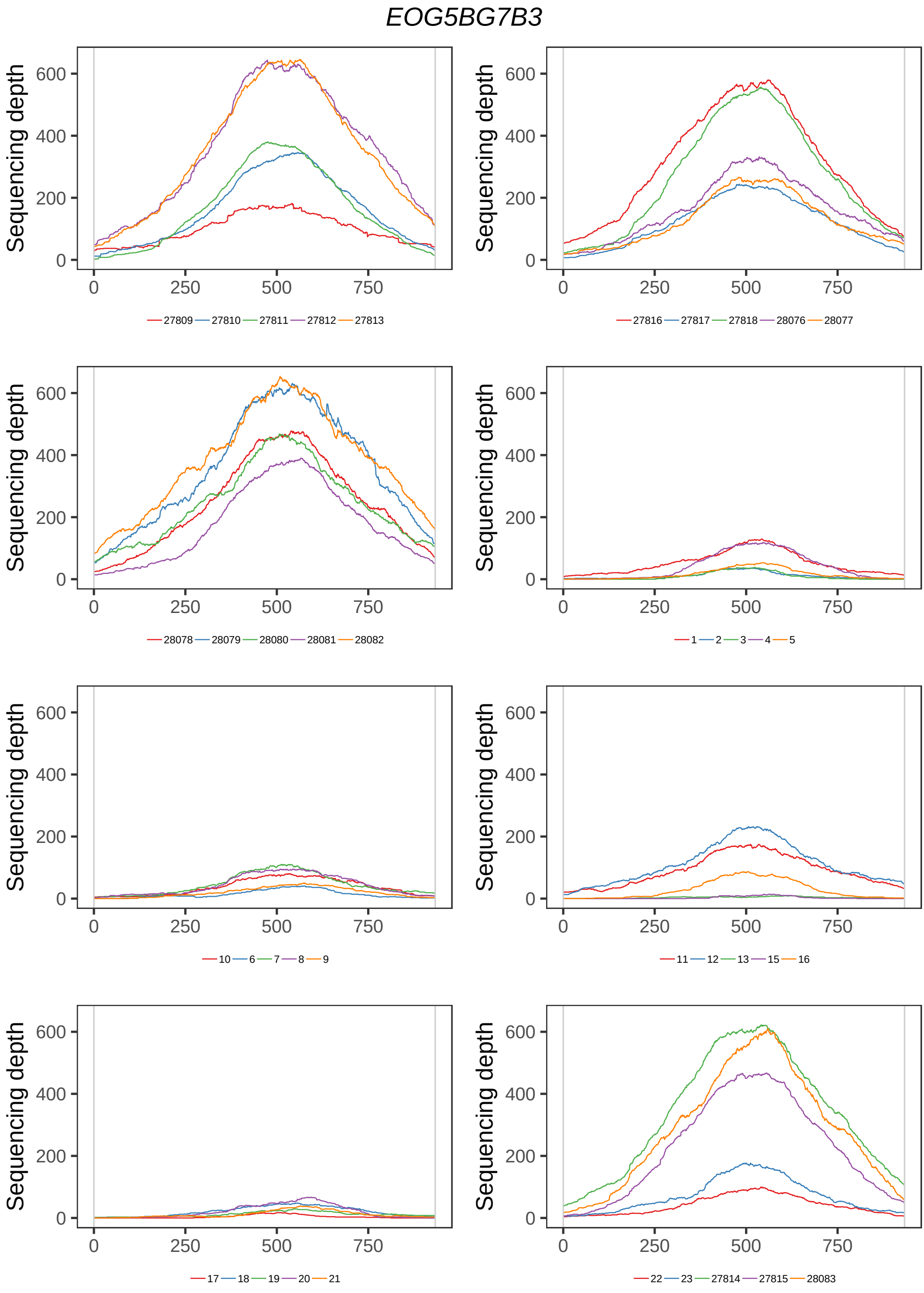
EOG5BG7B3
Sequencing depth
27809 27810 27811 27812 27813
27816 27817 27818 28076 28077
28078 28079 28080 28081 28082
1 2 3 4 5
10 6 7 8 9
11 12 13 15 16
17 18 19 20 21
22 23 27814 27815 28083

EOG5HHMHT

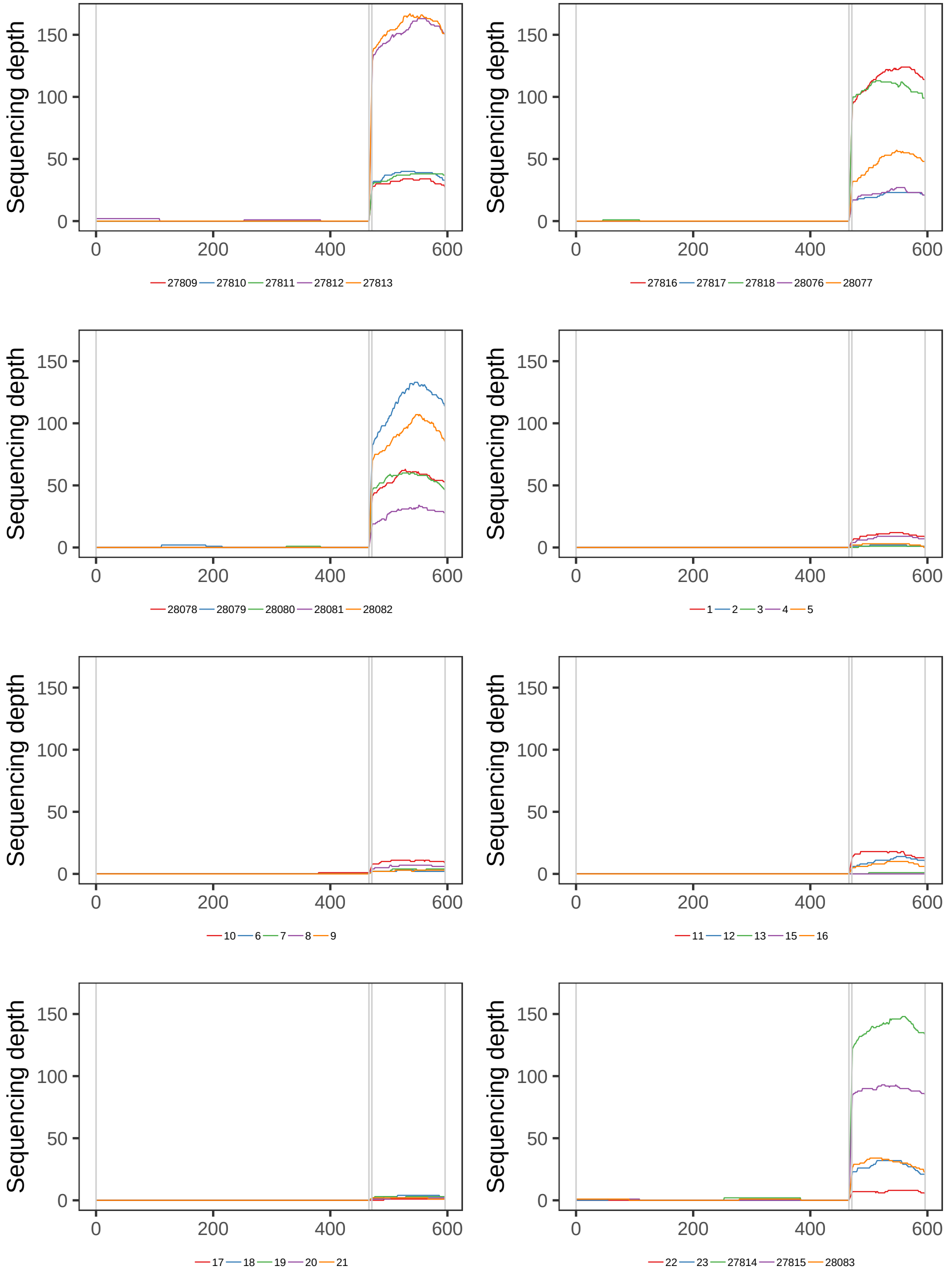

Sequencing depth
150
100
50
0
0
200
400
600
27809
27810
27811
27812
27813
27816
27817
27818
28076
28077
28078
28079
28080
28081
28082
1
2
3
4
5
10
6
7
8
9
11
12
13
15
16
17
18
19
20
21
22
23
27814
27815
28083

EOG5HT789

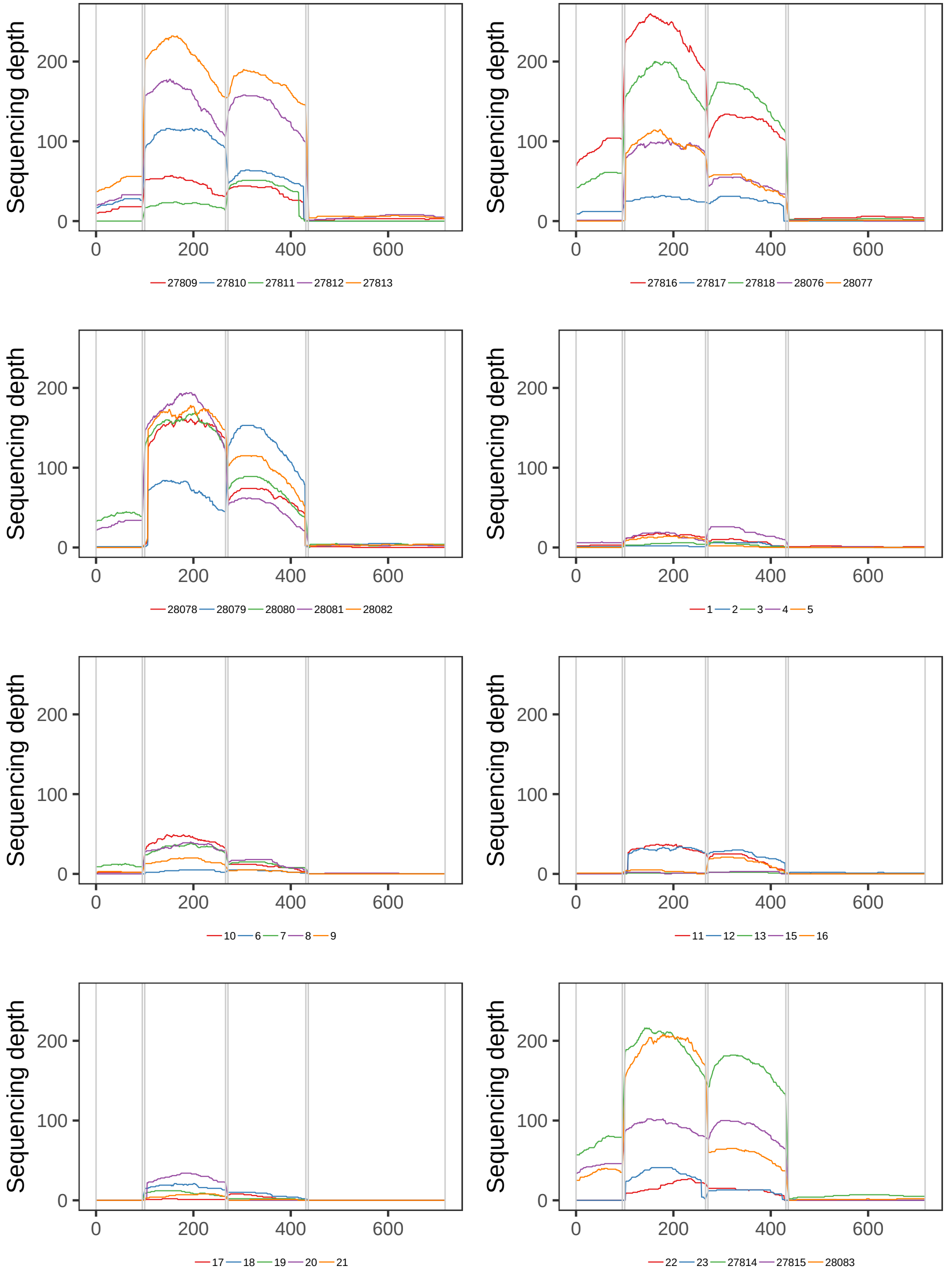

Sequencing depth
0
100
200
0
200
400
600
27809
27810
27811
27812
27813
27816
27817
27818
28076
28077
28078
28079
28080
28081
28082
1
2
3
4
5
10
6
7
8
9
11
12
13
15
16
17
18
19
20
21
22
23
27814
27815
28083

EOG5J9KG6

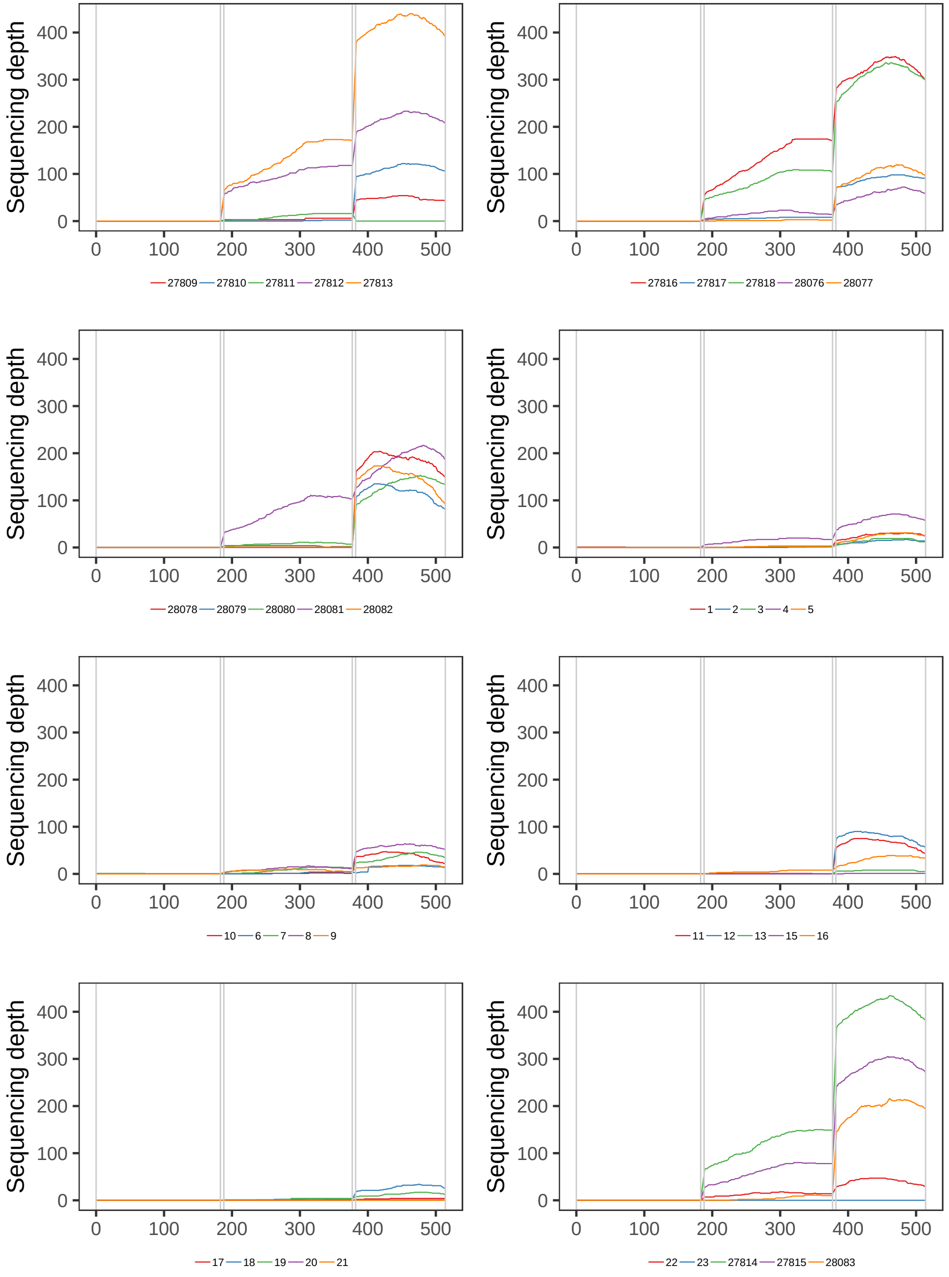
Sequencing depth
0
100
200
300
400
500
27809 27810 27811 27812 27813
27816 27817 27818 28076 28077
28078 28079 28080 28081 28082
1 2 3 4 5
10 6 7 8 9
11 12 13 15 16
17 18 19 20 21
22 23 27814 27815 28083

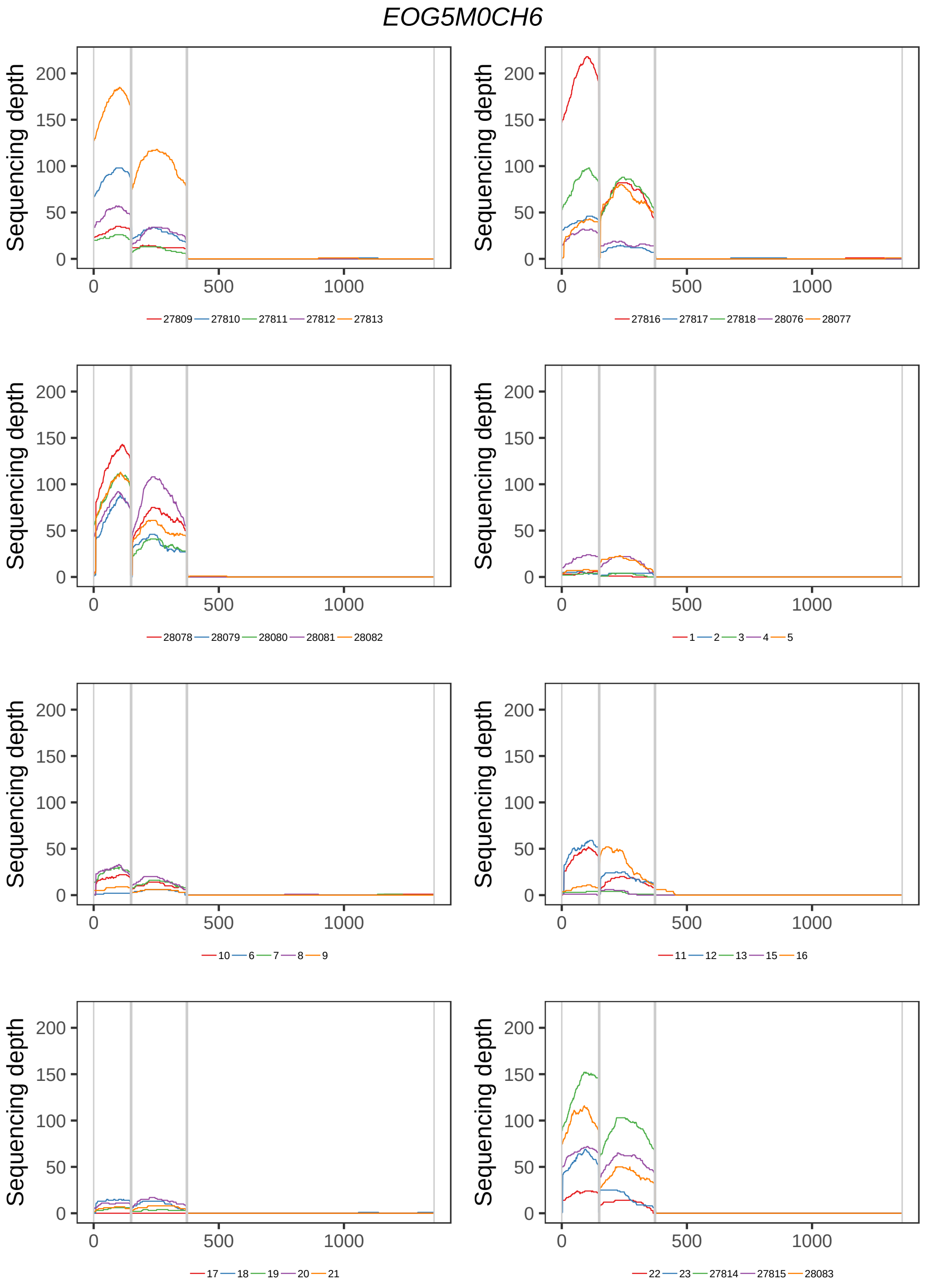
EOG5M0CH6
Sequencing depth
0
50
100
150
200
500
1000
27809 27810 27811 27812 27813
27816 27817 27818 28076 28077
28078 28079 28080 28081 28082
1 2 3 4 5
10 6 7 8 9
11 12 13 15 16
17 18 19 20 21
22 23 27814 27815 28083

EOG5MKKZ4

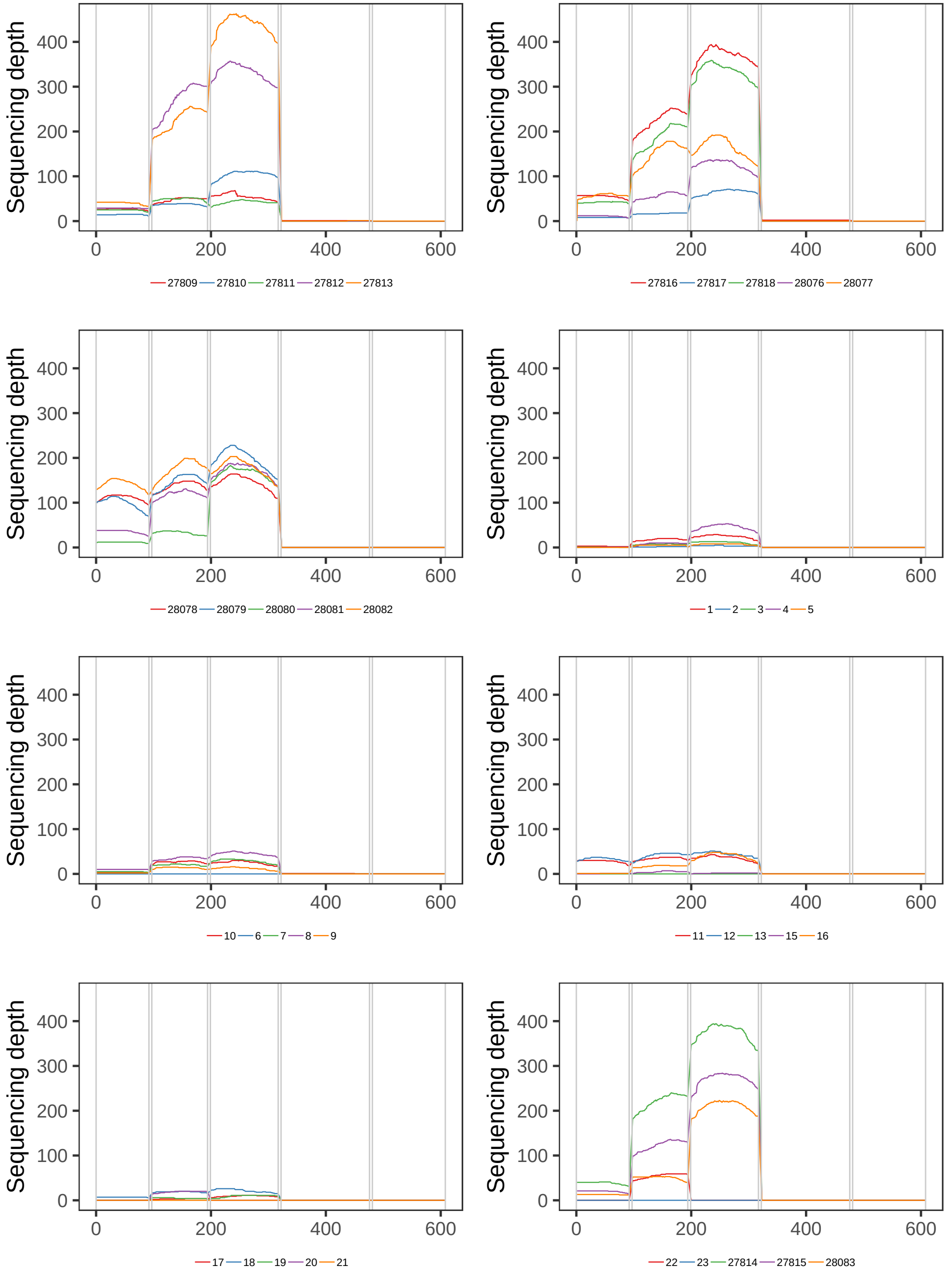
Sequencing depth
0
100
200
300
400
0
200
400
600
27809 27810 27811 27812 27813
27816 27817 27818 28076 28077
28078 28079 28080 28081 28082
1 2 3 4 5
10 6 7 8 9
11 12 13 15 16
17 18 19 20 21
22 23 27814 27815 28083

EOG5N5TCN

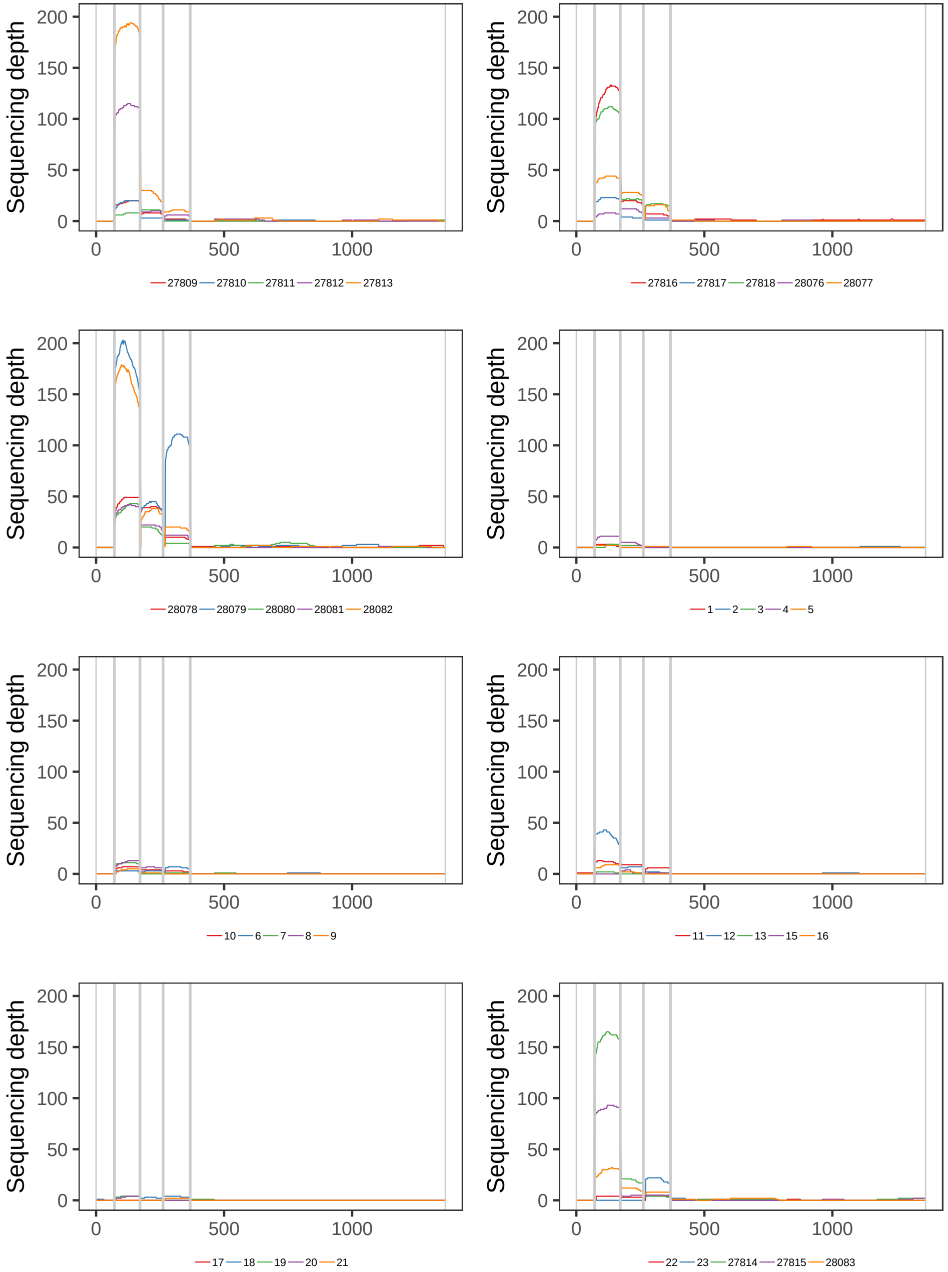
Sequencing depth
0
50
100
150
200
0
500
1000
27809 27810 27811 27812 27813
27816 27817 27818 28076 28077
28078 28079 28080 28081 28082
1 2 3 4 5
10 6 7 8 9
11 12 13 15 16
17 18 19 20 21
22 23 27814 27815 28083

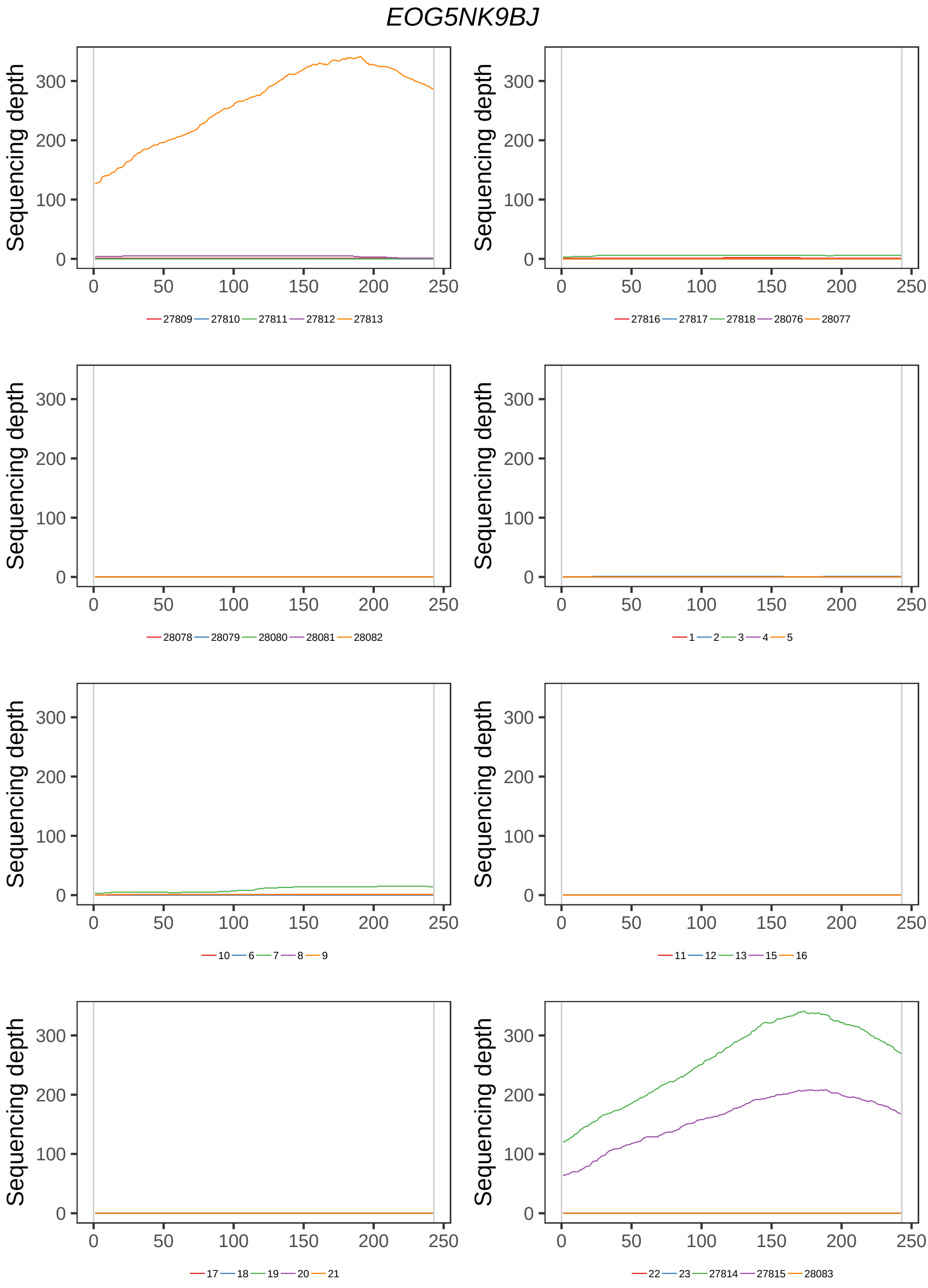

EOG5NK9BJ
Sequencing depth
0
100
200
300
0
50
100
150
200
250
27809
27810
27811
27812
27813
27816
27817
27818
28076
28077
28078
28079
28080
28081
28082
1
2
3
4
5
10
6
7
8
9
11
12
13
15
16
17
18
19
20
21
22
23
27814
27815
28083

EOG5PK0QJ

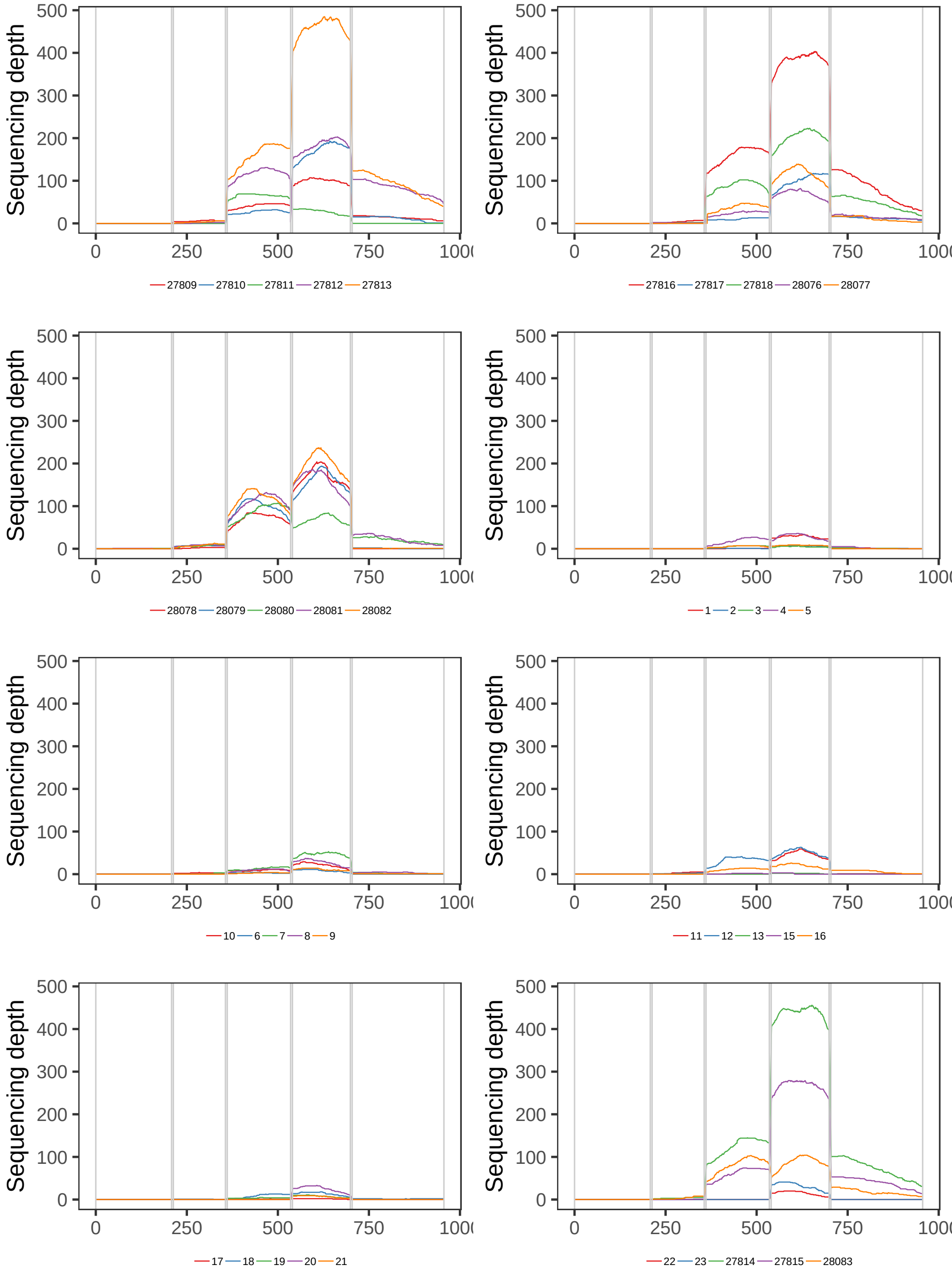

Sequencing depth
500
400
300
200
100
0
0
250
500
750
1000
27809 27810 27811 27812 27813
27816 27817 27818 28076 28077
28078 28079 28080 28081 28082
1 2 3 4 5
10 6 7 8 9
11 12 13 15 16
17 18 19 20 21
22 23 27814 27815 28083

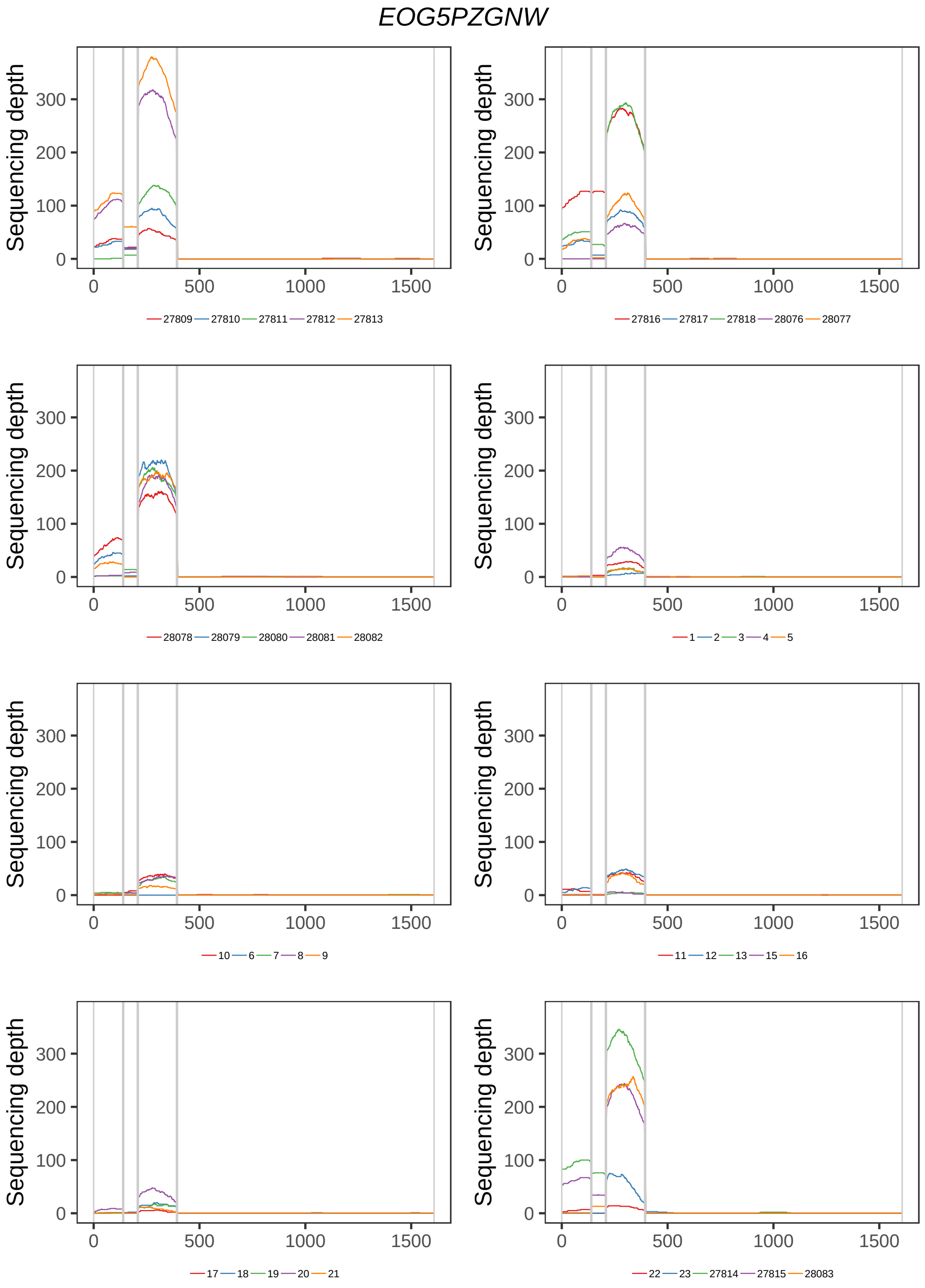
EOG5PZGNW
Sequencing depth
0
100
200
300
500
1000
1500
27809
27810
27811
27812
27813
27816
27817
27818
28076
28077
28078
28079
28080
28081
28082
1
2
3
4
5
10
6
7
8
9
11
12
13
15
16
17
18
19
20
21
22
23
27814
27815
28083

EOG5Q2BX6

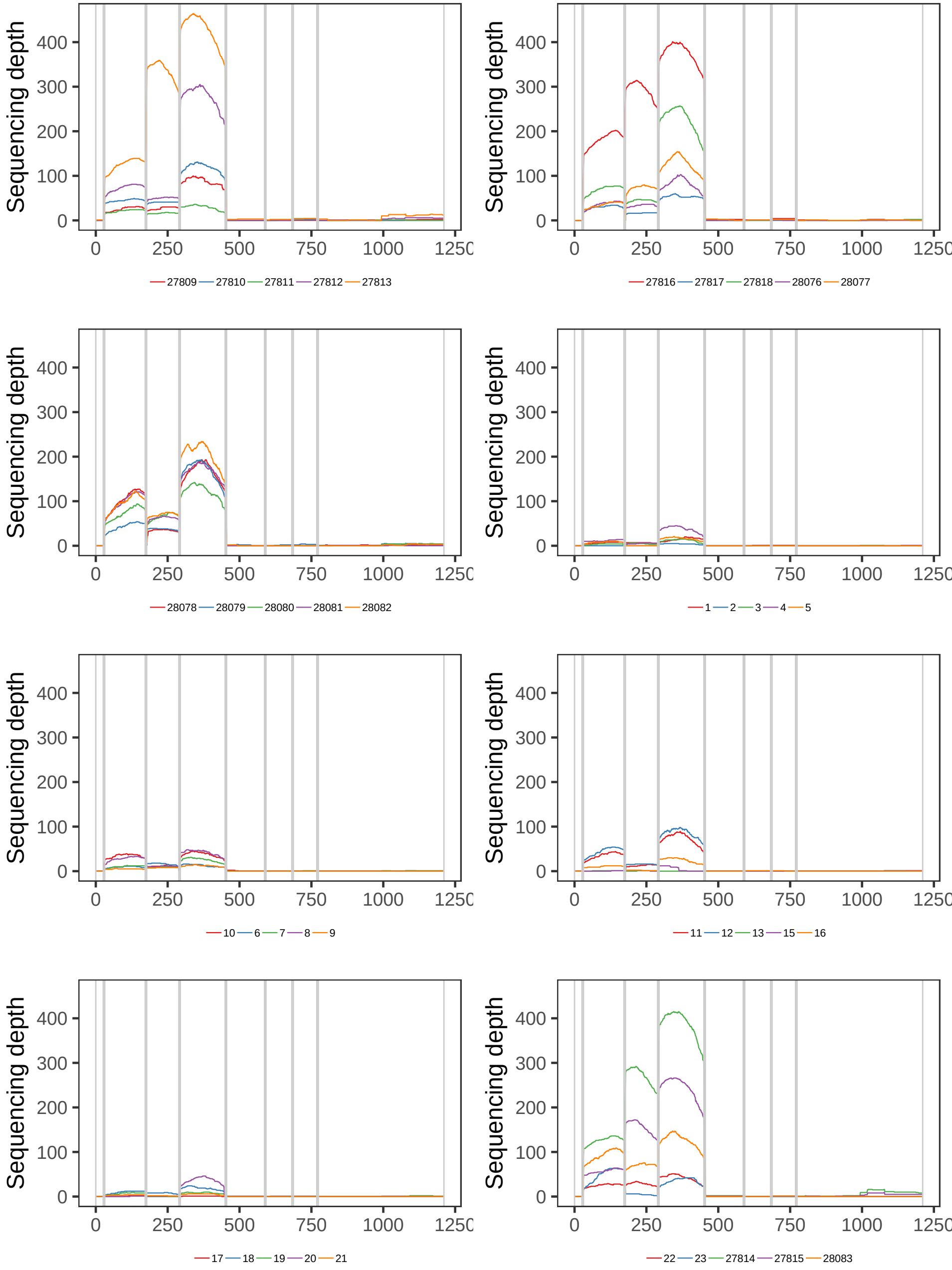

Sequencing depth
0
100
200
300
400
0
250
500
750
1000
1250
27809 27810 27811 27812 27813
27816 27817 27818 28076 28077
28078 28079 28080 28081 28082
1 2 3 4 5
10 6 7 8 9
11 12 13 15 16
17 18 19 20 21
22 23 27814 27815 28083

EOG5QFTW0

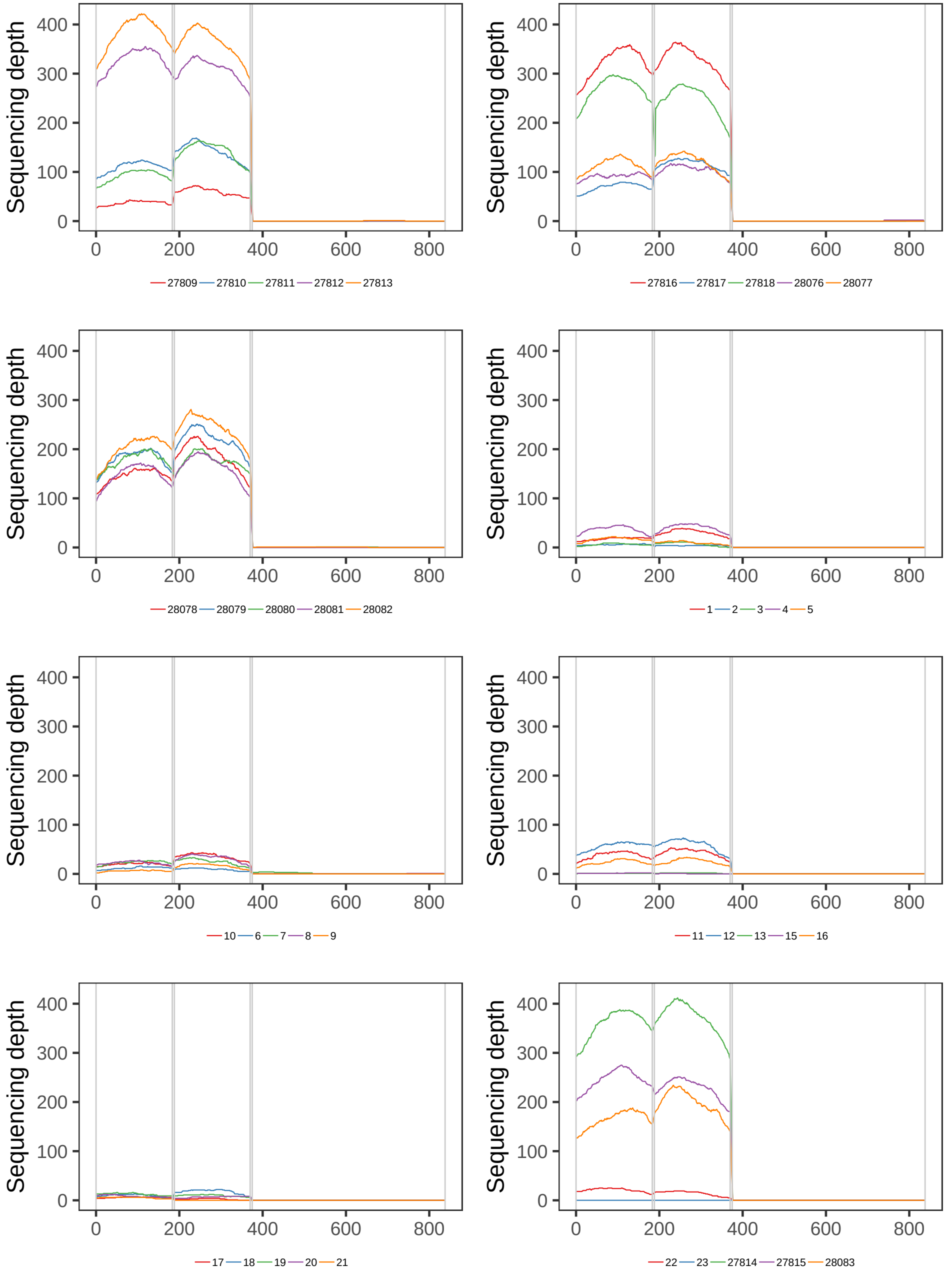

Sequencing depth
0
100
200
300
400
0
200
400
600
800
27809 27810 27811 27812 27813
27816 27817 27818 28076 28077
28078 28079 28080 28081 28082
1 2 3 4 5
10 6 7 8 9
11 12 13 15 16
17 18 19 20 21
22 23 27814 27815 28083

EOG5TX96W

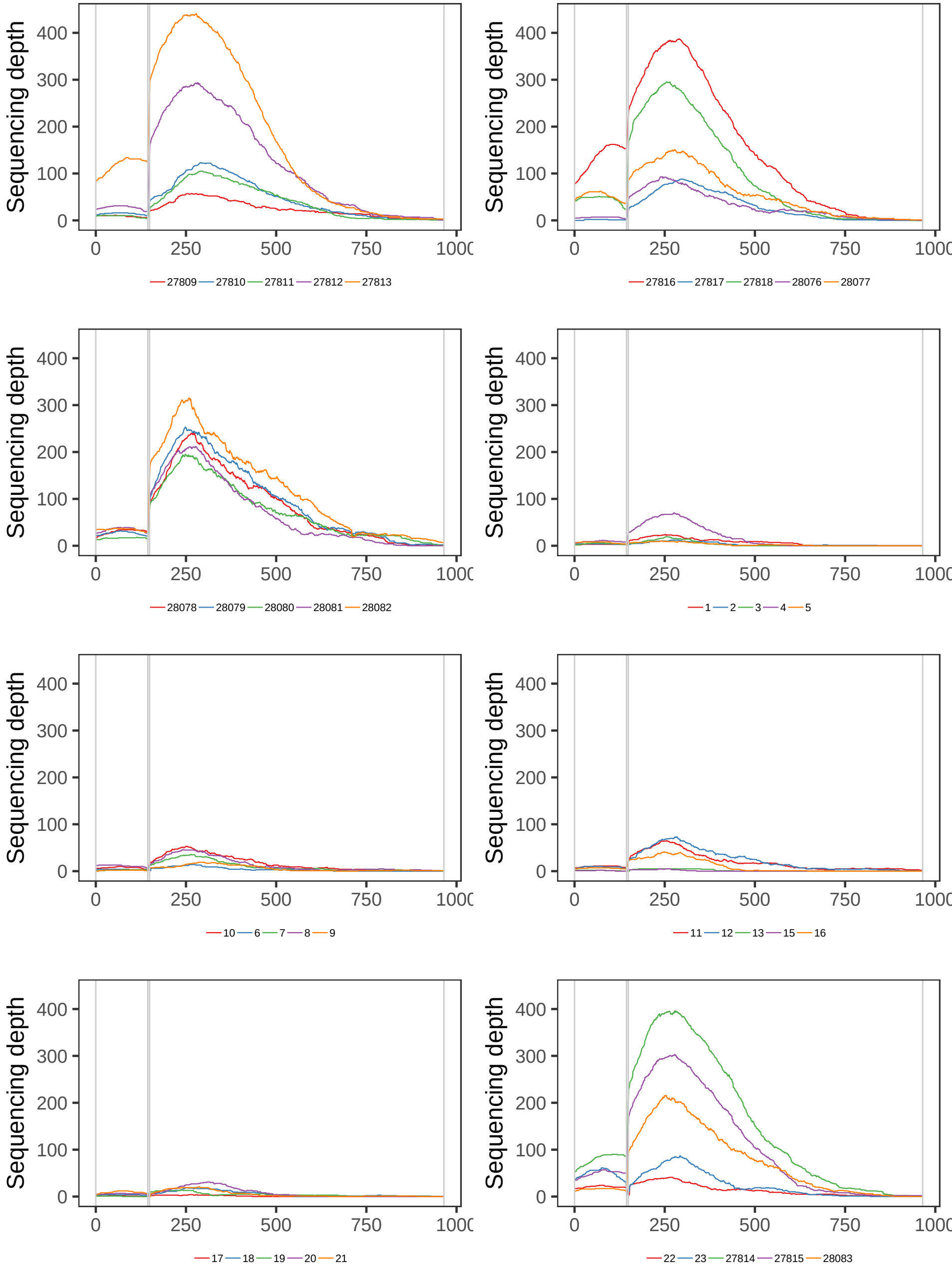
Sequencing depth
0
100
200
300
400
0
250
500
750
1000
27809 27810 27811 27812 27813
27816 27817 27818 28076 28077
28078 28079 28080 28081 28082
1 2 3 4 5
10 6 7 8 9
11 12 13 15 16
17 18 19 20 21
22 23 27814 27815 28083

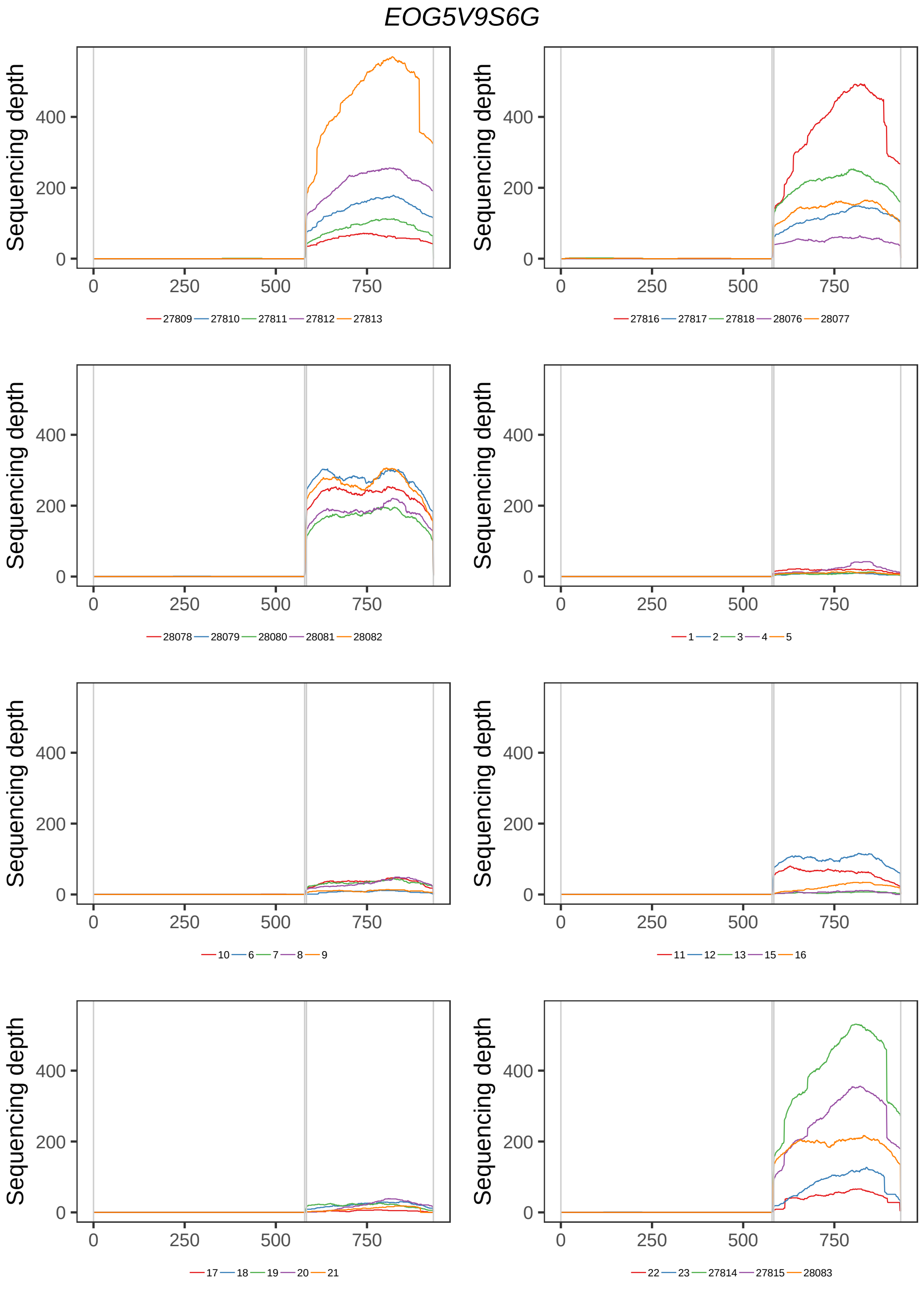
EOG5V9S6G
Sequencing depth
27809 27810 27811 27812 27813
27816 27817 27818 28076 28077
28078 28079 28080 28081 28082
1 2 3 4 5
10 6 7 8 9
11 12 13 15 16
17 18 19 20 21
22 23 27814 27815 28083

EOG5Z8WC1

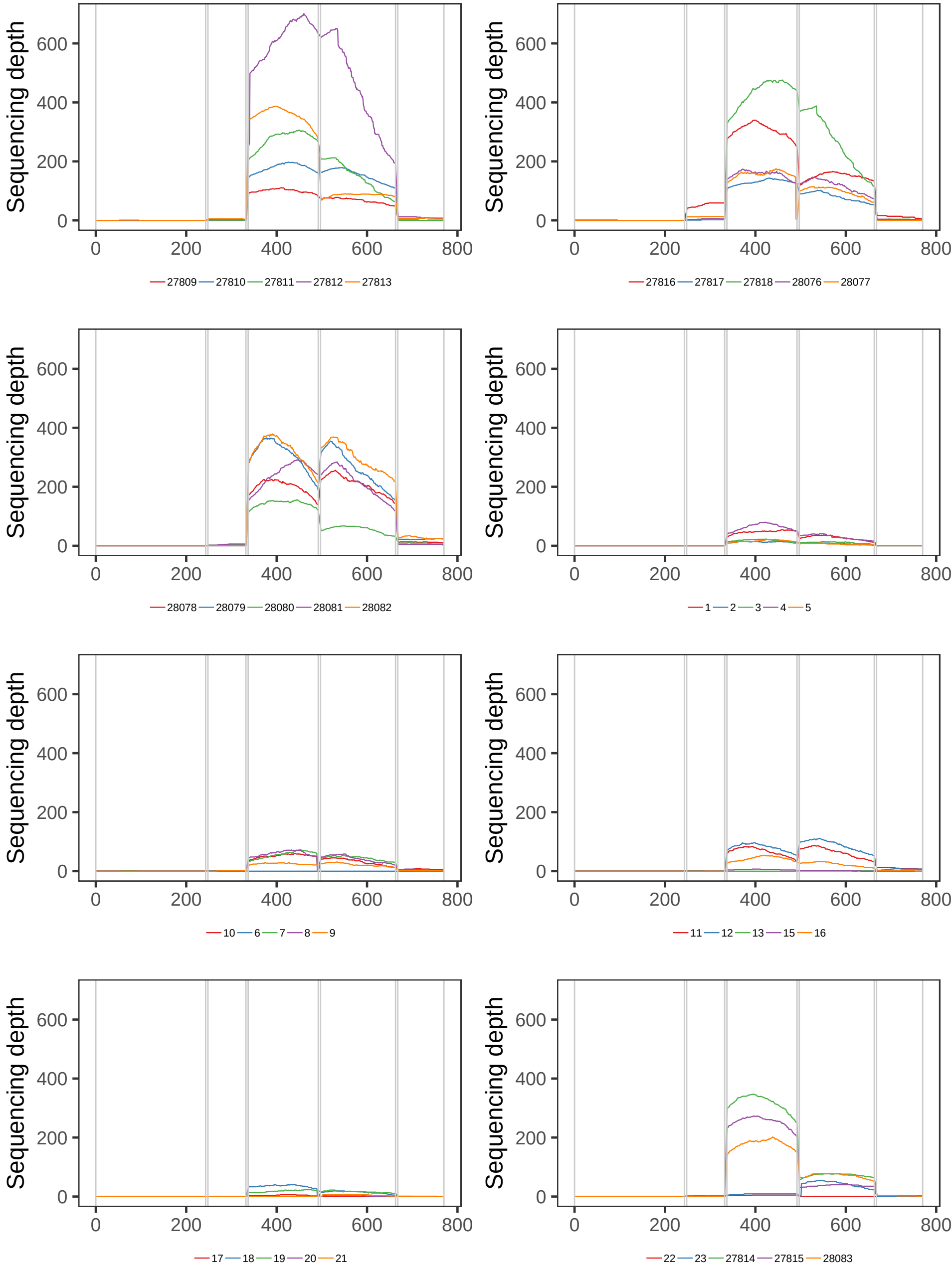
Sequencing depth
27809 27810 27811 27812 27813
27816 27817 27818 28076 28077
28078 28079 28080 28081 28082
1 2 3 4 5
10 6 7 8 9
11 12 13 15 16
17 18 19 20 21
22 23 27814 27815 28083

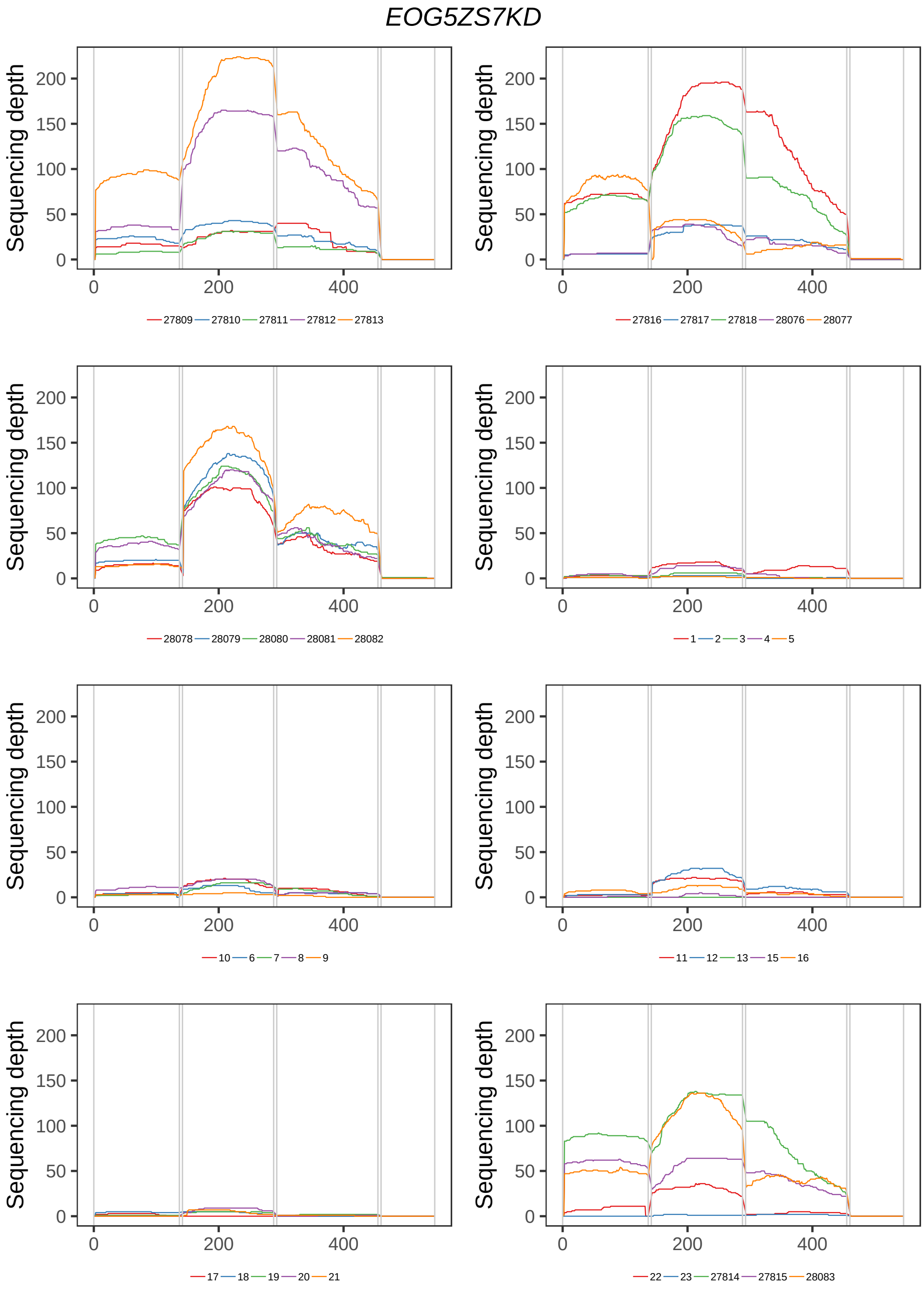
EOG5ZS7KD
Sequencing depth
0
50
100
150
200
0
200
400
27809 27810 27811 27812 27813
27816 27817 27818 28076 28077
28078 28079 28080 28081 28082
1 2 3 4 5
10 6 7 8 9
11 12 13 15 16
17 18 19 20 21
22 23 27814 27815 28083

EOG5ZW3TJ

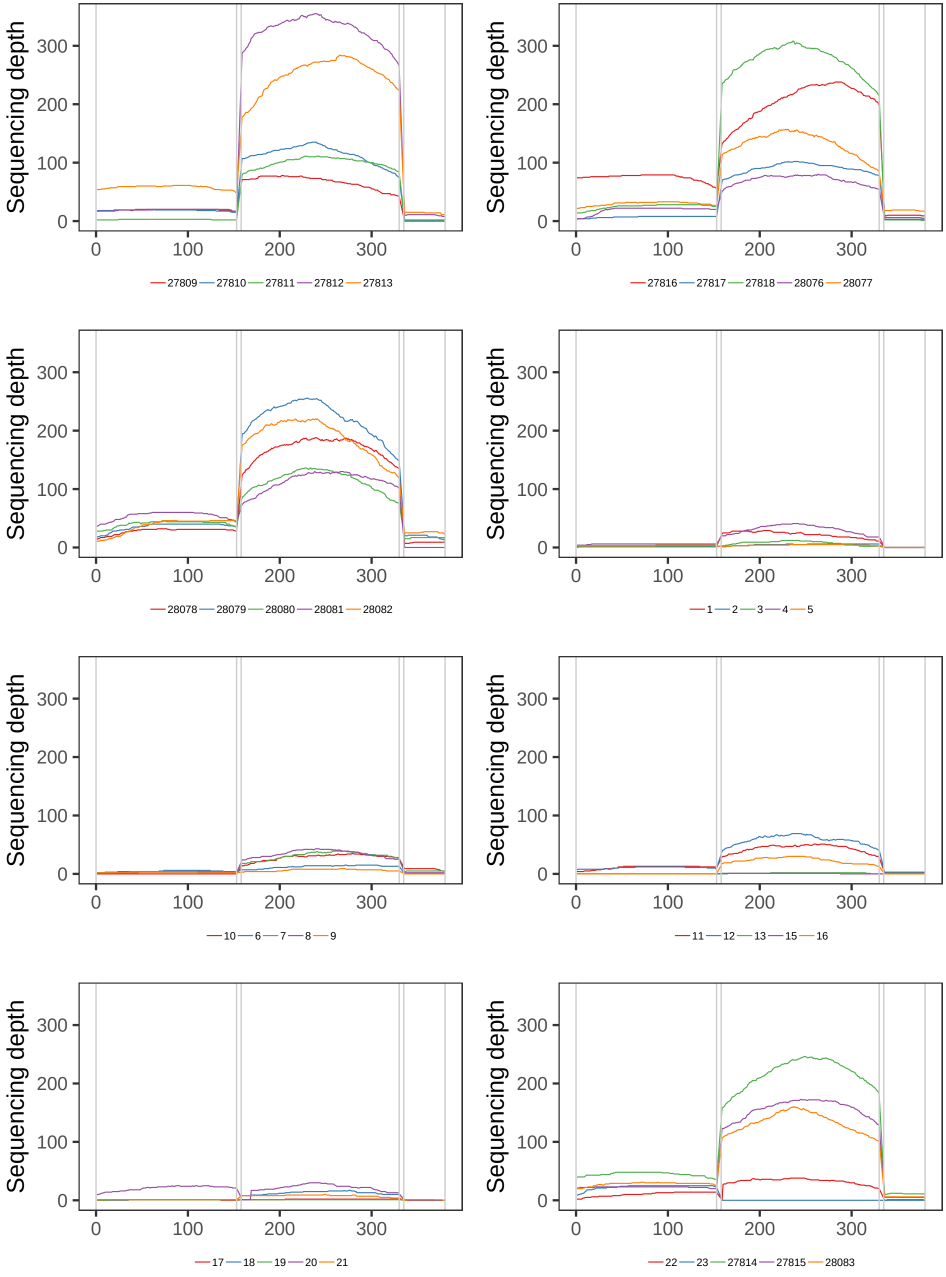

Sequencing depth
0
100
200
300
27809 27810 27811 27812 27813
27816 27817 27818 28076 28077
28078 28079 28080 28081 28082
1 2 3 4 5
10 6 7 8 9
11 12 13 15 16
17 18 19 20 21
22 23 27814 27815 28083

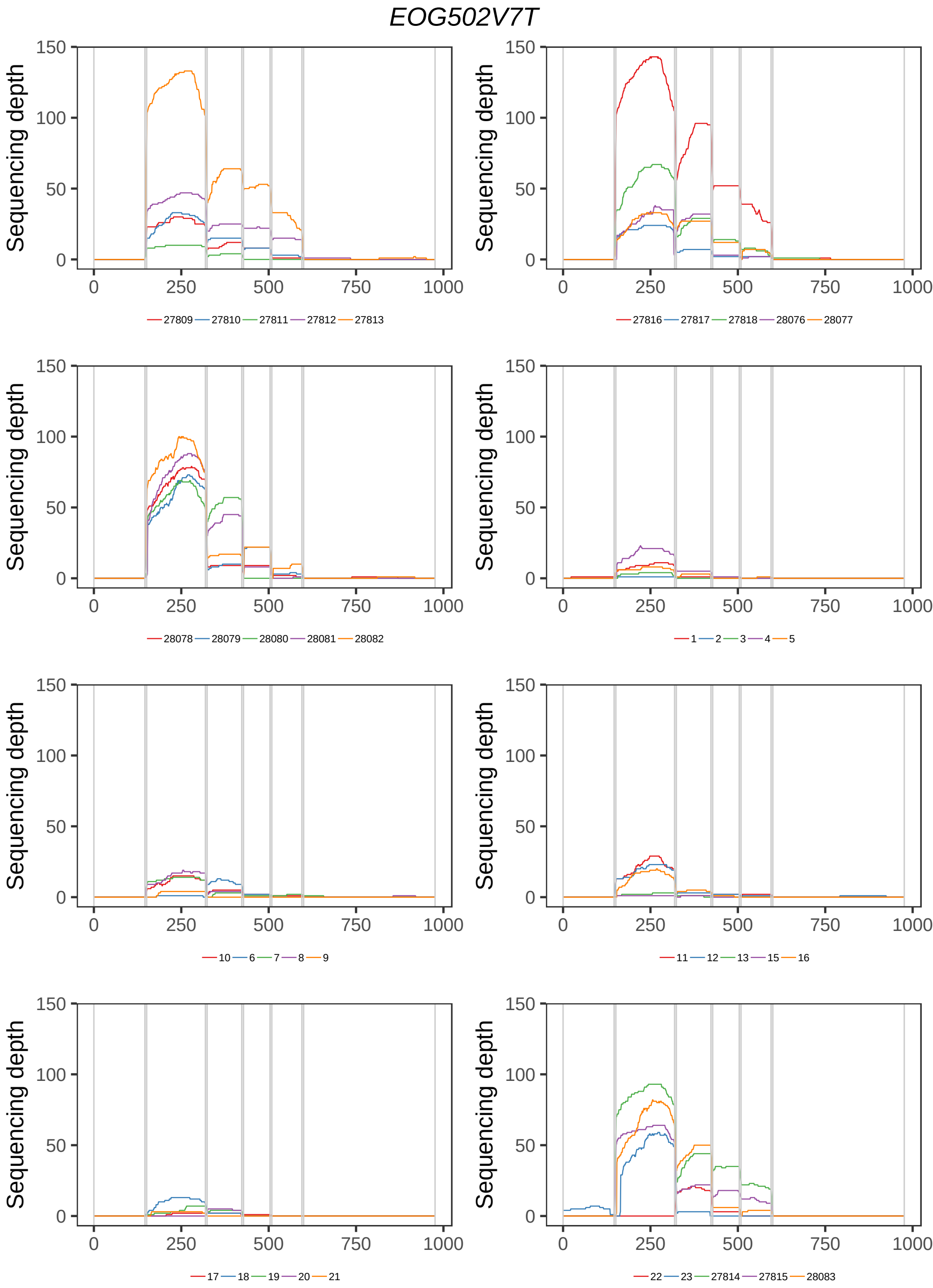
EOG502V7T
Sequencing depth
27809 27810 27811 27812 27813
27816 27817 27818 28076 28077
28078 28079 28080 28081 28082
1 2 3 4 5
10 6 7 8 9
11 12 13 15 16
17 18 19 20 21
22 23 27814 27815 28083

EOG51G1KR

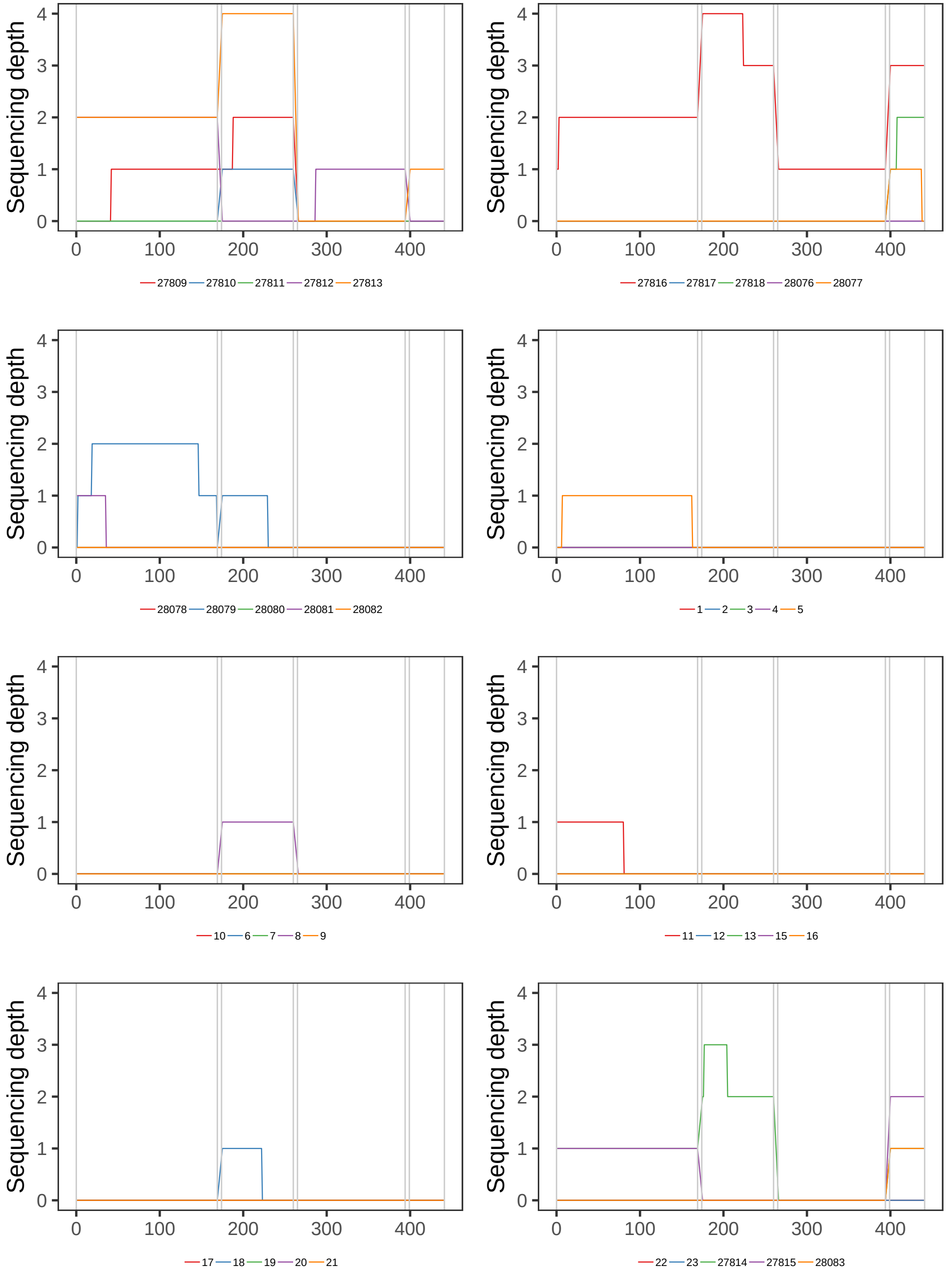

Sequencing depth
27809 27810 27811 27812 27813
27816 27817 27818 28076 28077
28078 28079 28080 28081 28082
1 2 3 4 5
10 6 7 8 9
11 12 13 15 16
17 18 19 20 21
22 23 27814 27815 28083

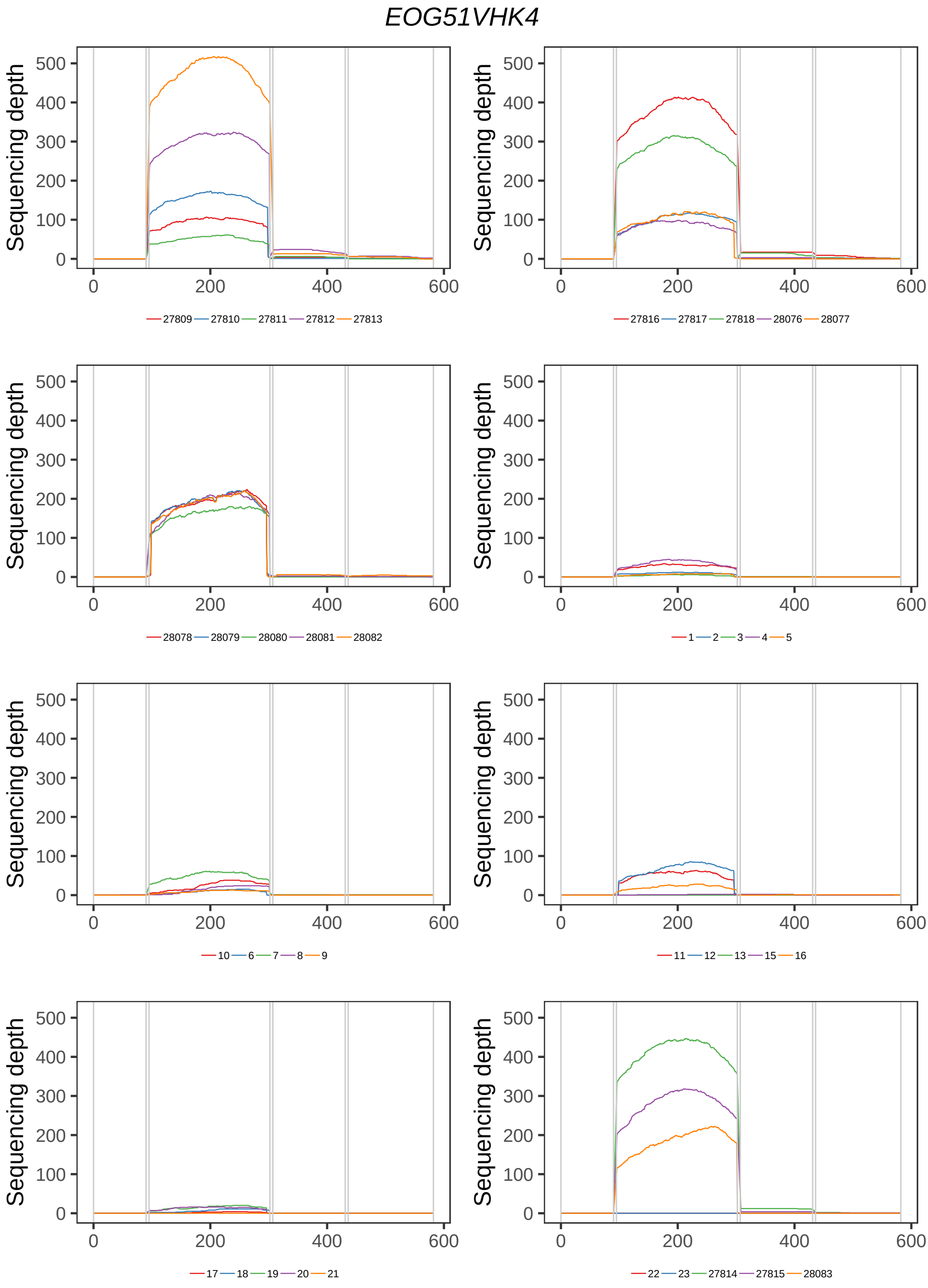
EOG51VHK4
Sequencing depth
27809 27810 27811 27812 27813
27816 27817 27818 28076 28077
28078 28079 28080 28081 28082
1 2 3 4 5
10 6 7 8 9
11 12 13 15 16
17 18 19 20 21
22 23 27814 27815 28083

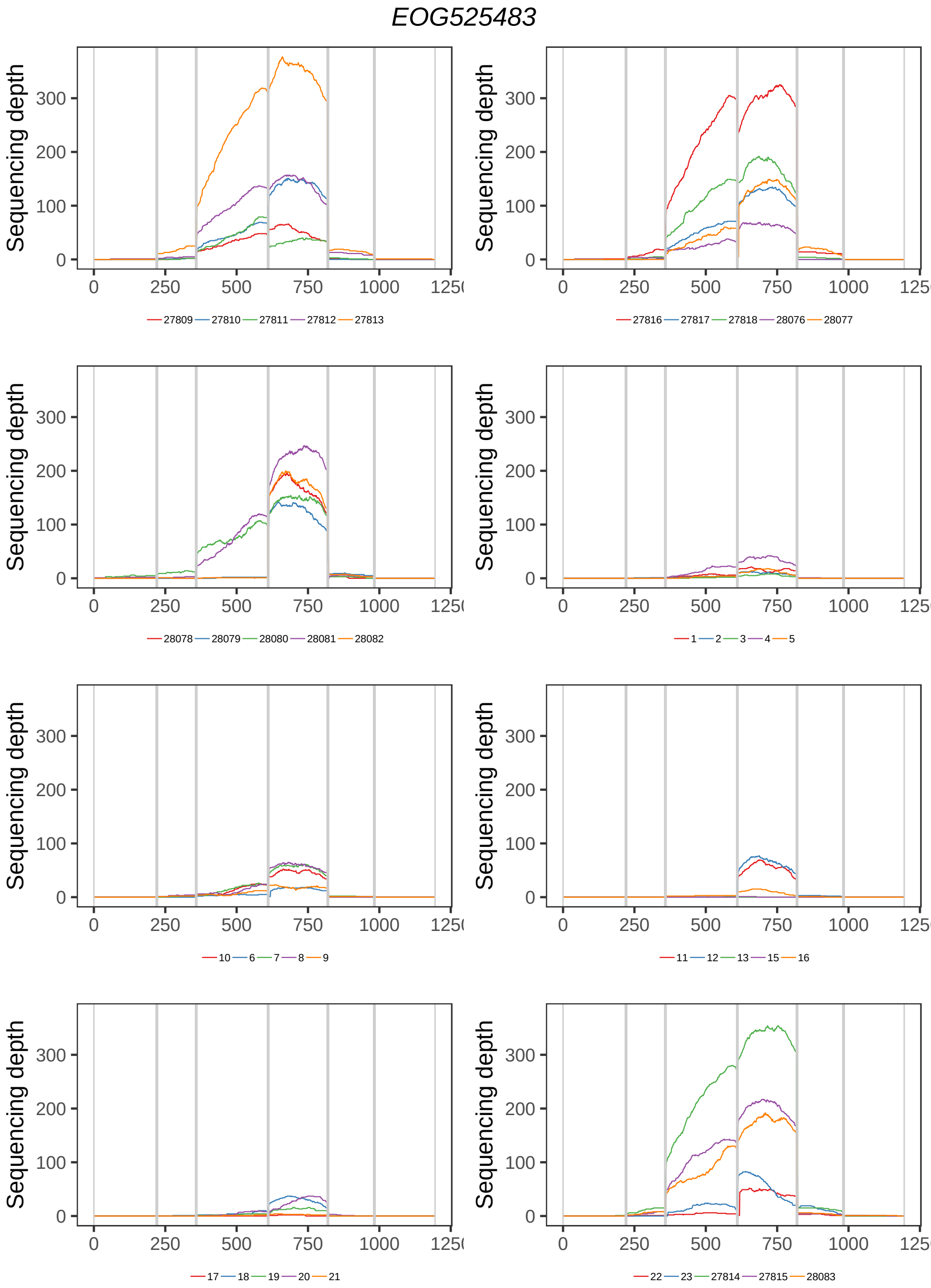
EOG525483
Sequencing depth
0
100
200
300
0
250
500
750
1000
1250
27809
27810
27811
27812
27813
27816
27817
27818
28076
28077
28078
28079
28080
28081
28082
1
2
3
4
5
10
6
7
8
9
11
12
13
15
16
17
18
19
20
21
22
23
27814
27815
28083

EOG559ZWQ

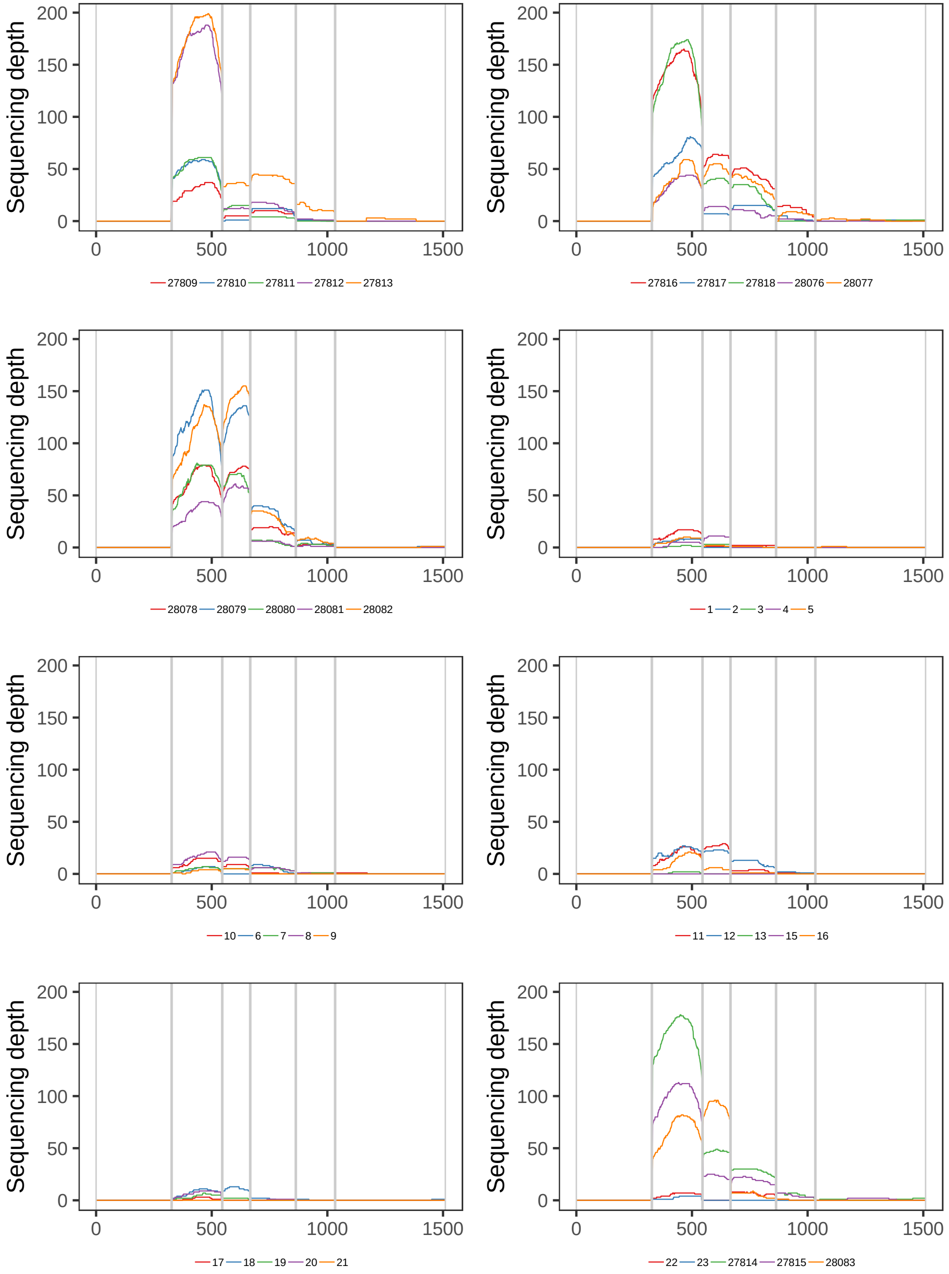

Sequencing depth
0
50
100
150
200
0
500
1000
1500
27809 27810 27811 27812 27813
27816 27817 27818 28076 28077
28078 28079 28080 28081 28082
1 2 3 4 5
10 6 7 8 9
11 12 13 15 16
17 18 19 20 21
22 23 27814 27815 28083

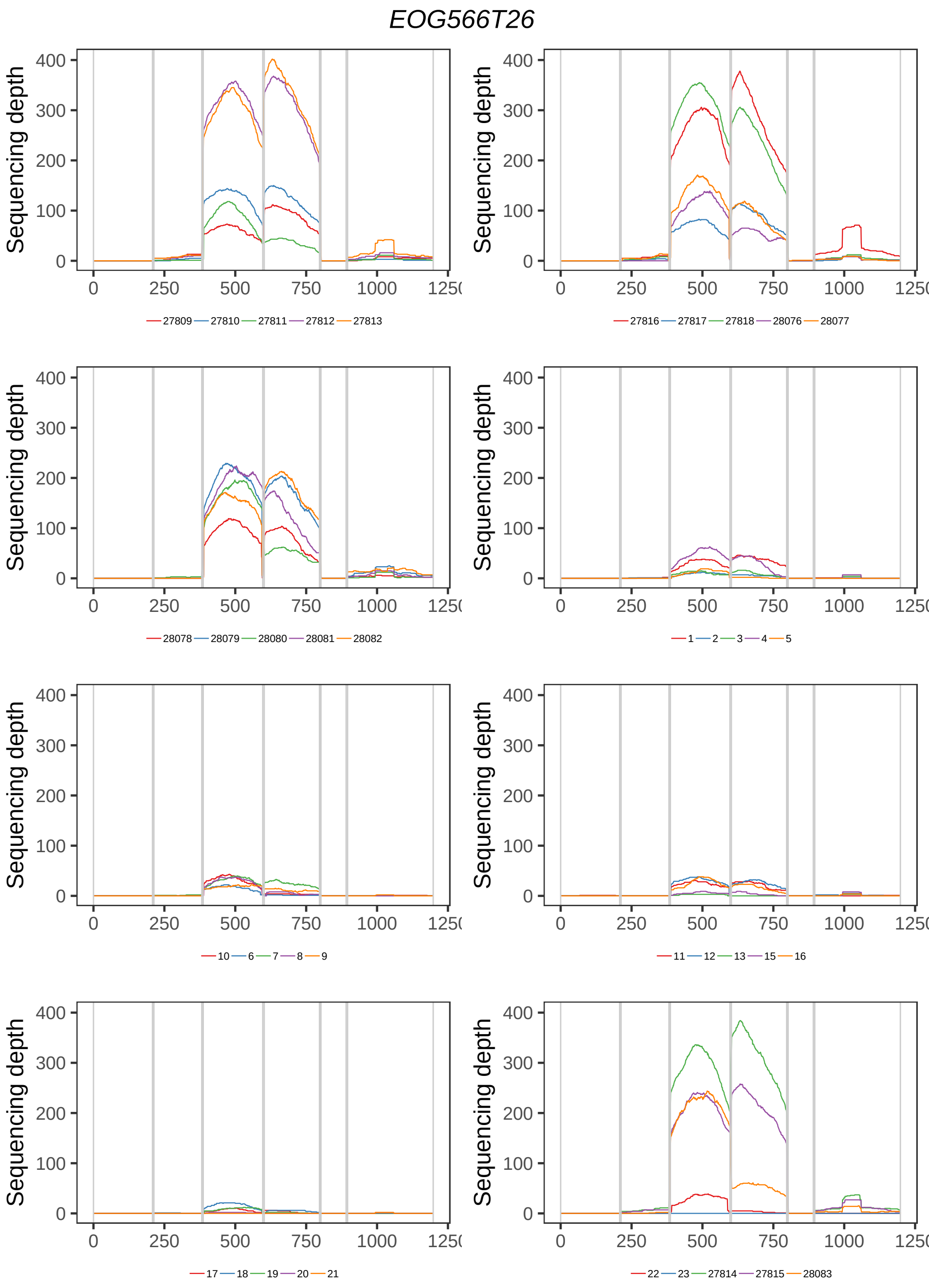

EOG566T26
Sequencing depth
0
100
200
300
400
0
250
500
750
1000
1250
27809 27810 27811 27812 27813
27816 27817 27818 28076 28077
28078 28079 28080 28081 28082
1 2 3 4 5
10 6 7 8 9
11 12 13 15 16
17 18 19 20 21
22 23 27814 27815 28083

EOG5BCC3F

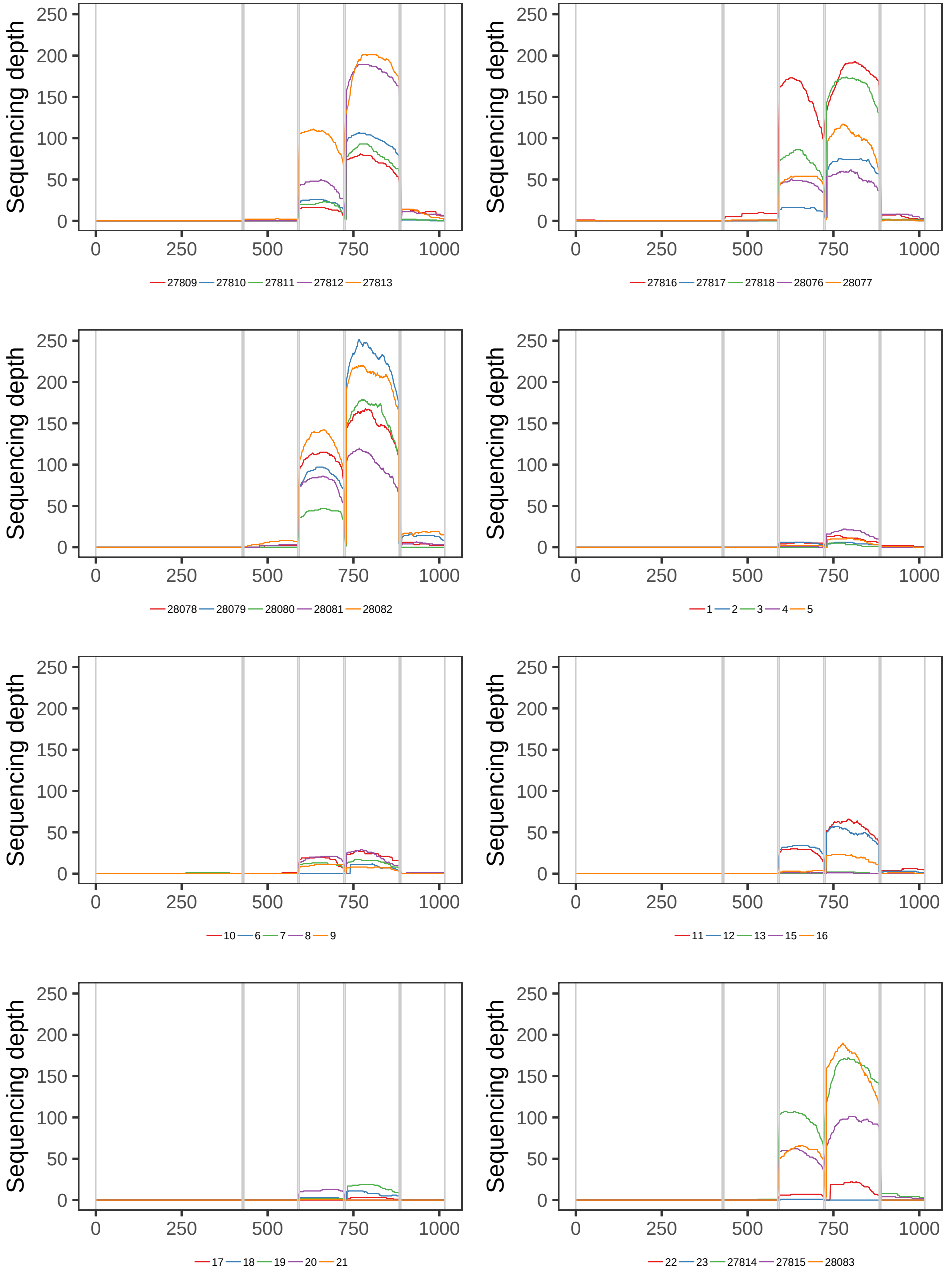

Sequencing depth
27809
27810
27811
27812
27813
27816
27817
27818
28076
28077
28078
28079
28080
28081
28082
1
2
3
4
5
10
6
7
8
9
11
12
13
15
16
17
18
19
20
21
22
23
27814
27815
28083

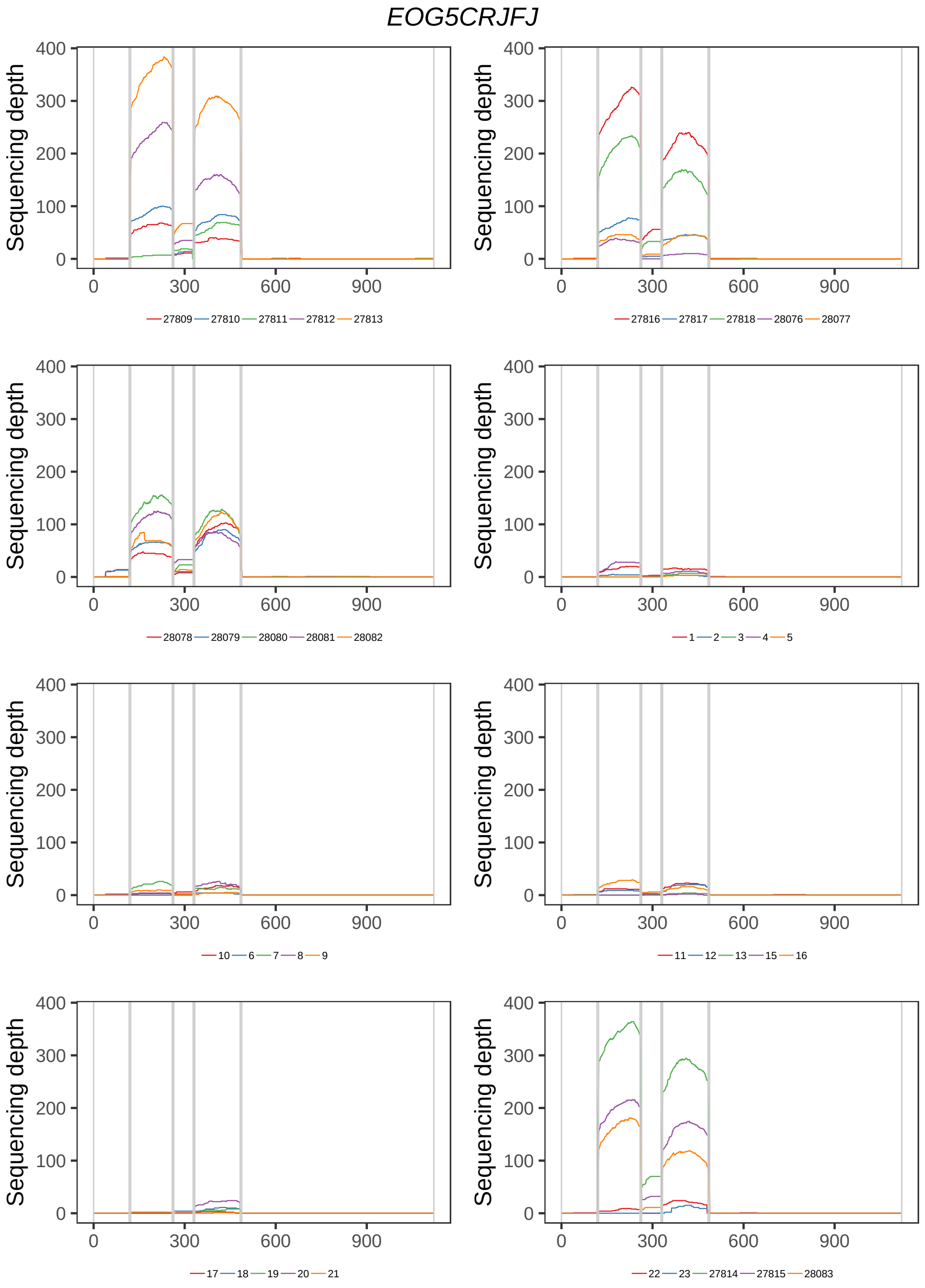
EOG5CRJFJ
Sequencing depth
0
100
200
300
400
0
300
600
900
27809 27810 27811 27812 27813
27816 27817 27818 28076 28077
28078 28079 28080 28081 28082
1 2 3 4 5
10 6 7 8 9
11 12 13 15 16
17 18 19 20 21
22 23 27814 27815 28083

EOG5DR7TD

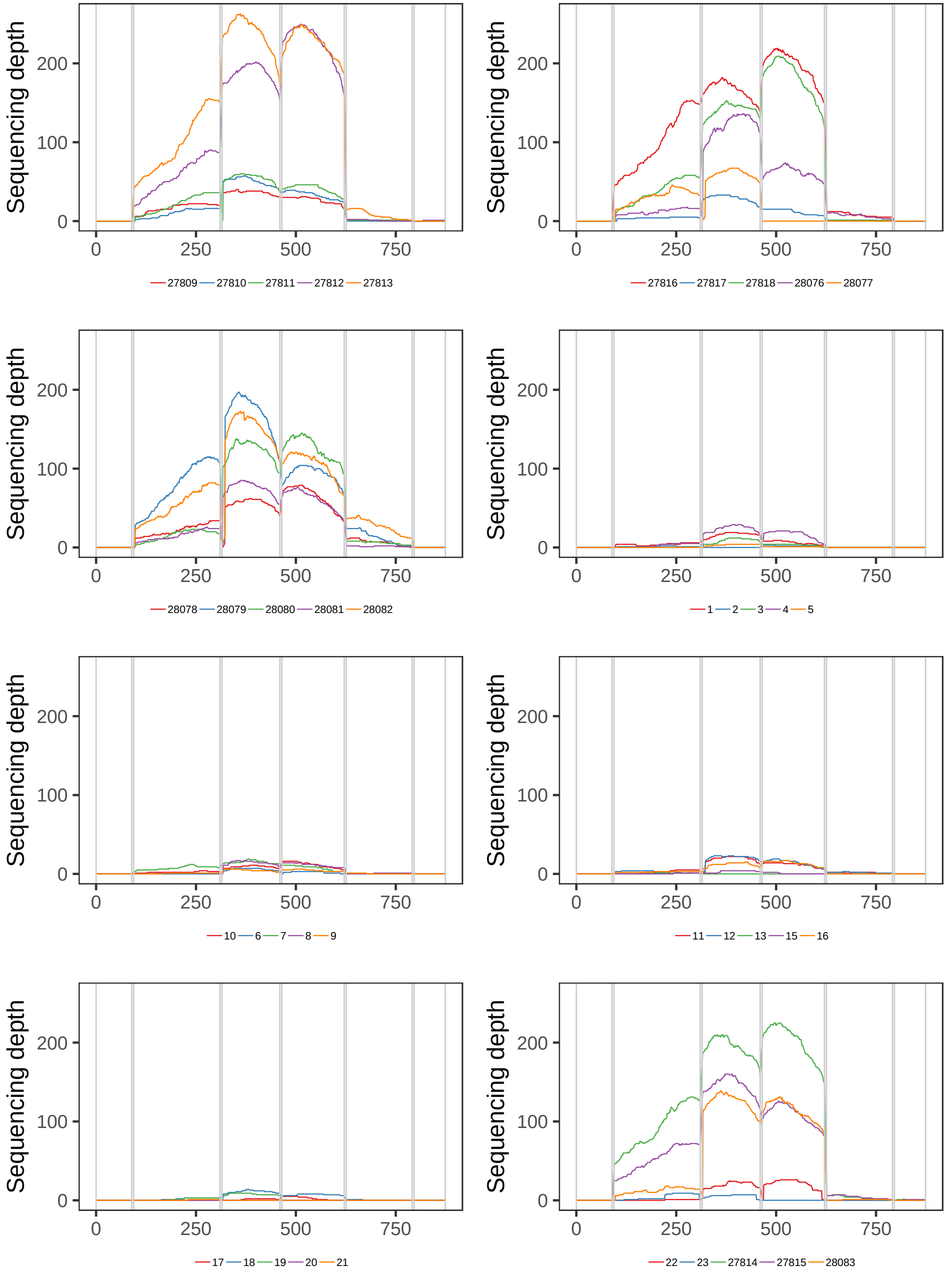
Sequencing depth
0
100
200
0
250
500
750
27809
27810
27811
27812
27813
27816
27817
27818
28076
28077
28078
28079
28080
28081
28082
1
2
3
4
5
10
6
7
8
9
11
12
13
15
16
17
18
19
20
21
22
23
27814
27815
28083

EOG5FFBHM

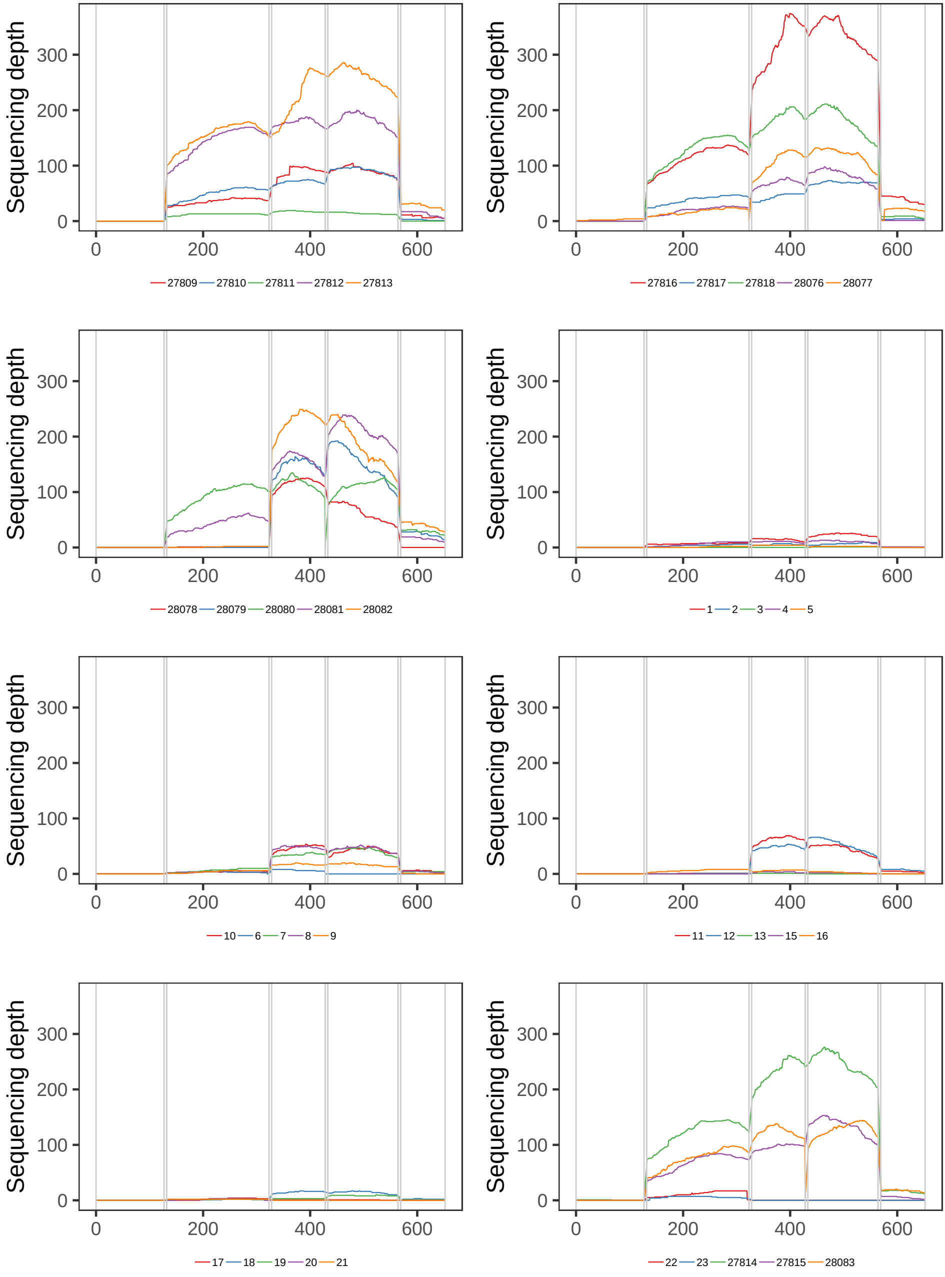
Sequencing depth
27809 27810 27811 27812 27813
27816 27817 27818 28076 28077
28078 28079 28080 28081 28082
1 2 3 4 5
10 6 7 8 9
11 12 13 15 16
17 18 19 20 21
22 23 27814 27815 28083

EOG5GF1X9

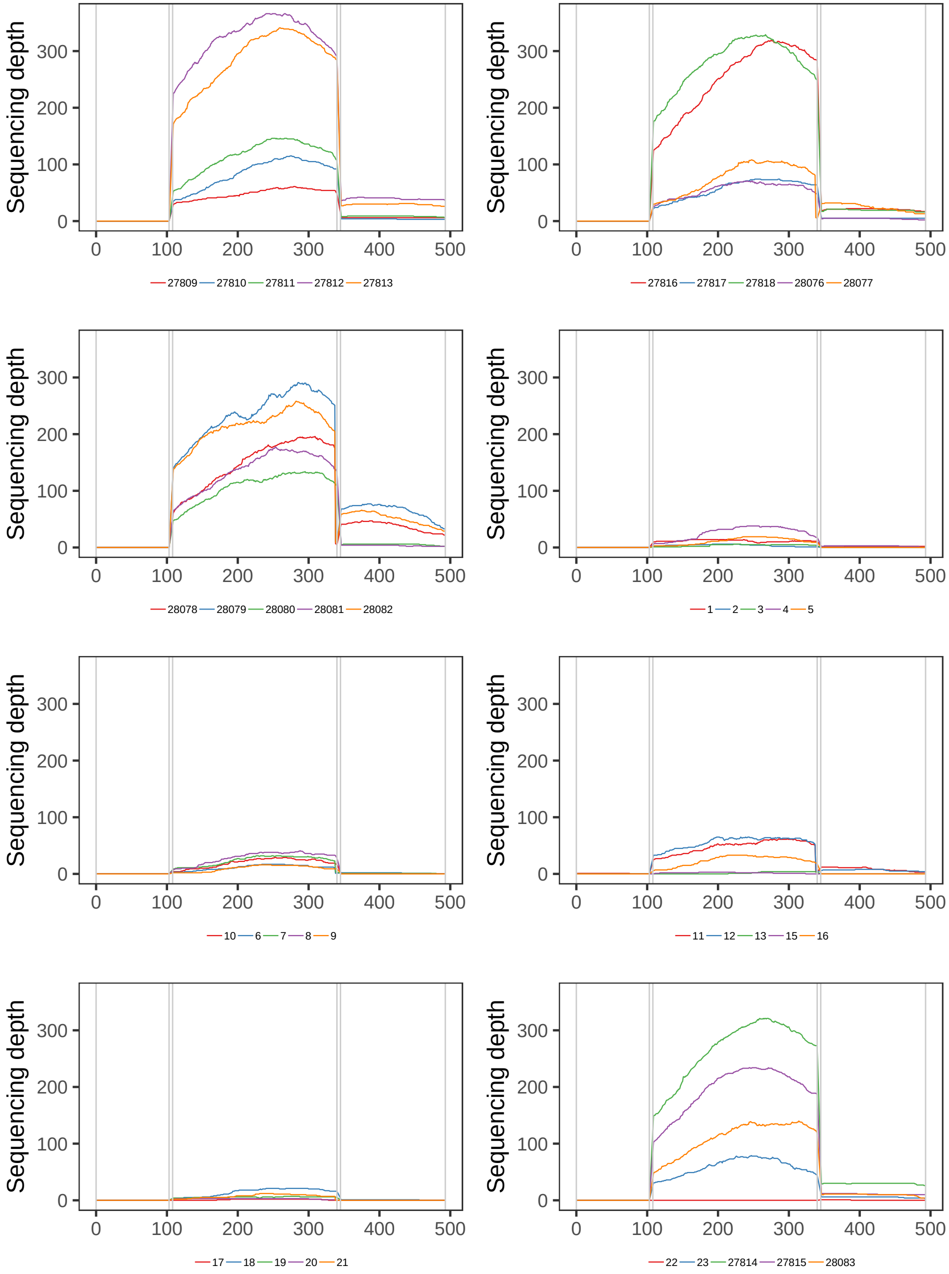

Sequencing depth
0
100
200
300
400
500
27809 27810 27811 27812 27813
27816 27817 27818 28076 28077
28078 28079 28080 28081 28082
1 2 3 4 5
10 6 7 8 9
11 12 13 15 16
17 18 19 20 21
22 23 27814 27815 28083

EOG5GMSDC

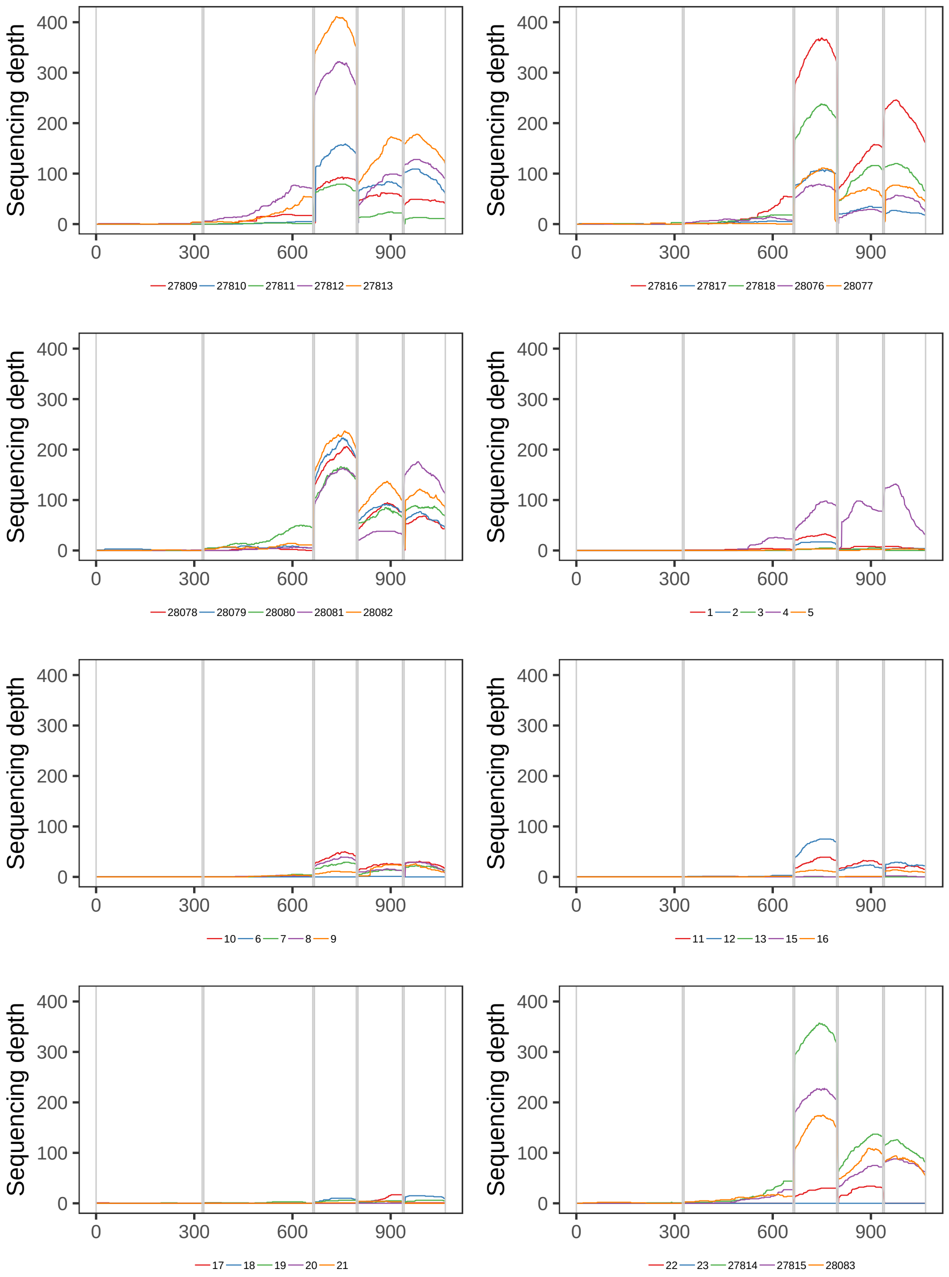

Sequencing depth
0
100
200
300
400
0
300
600
900
27809 27810 27811 27812 27813
27816 27817 27818 28076 28077
28078 28079 28080 28081 28082
1 2 3 4 5
10 6 7 8 9
11 12 13 15 16
17 18 19 20 21
22 23 27814 27815 28083

EOG5H189T

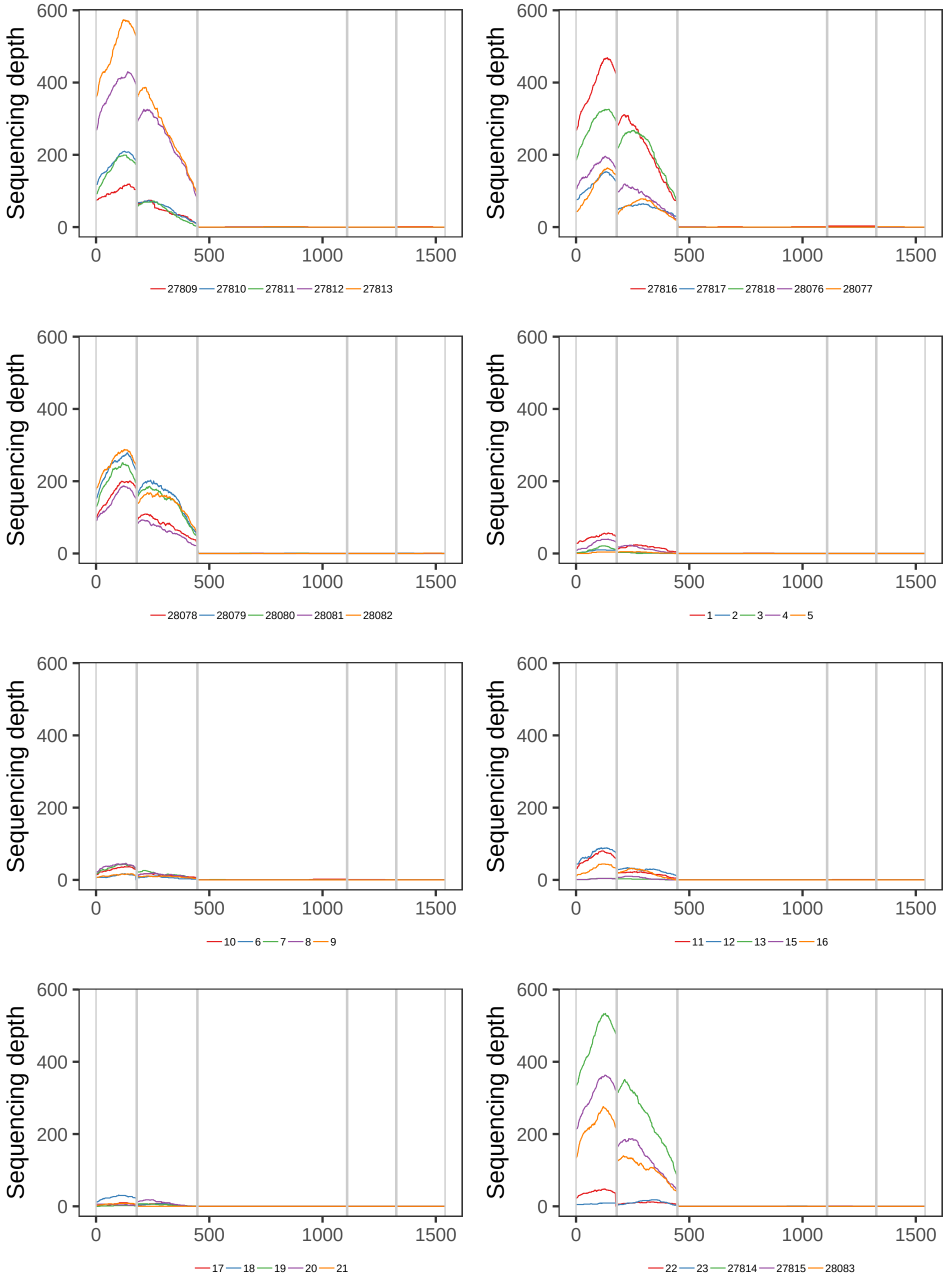
Sequencing depth
600
400
200
0
0
500
1000
1500
27809 27810 27811 27812 27813
27816 27817 27818 28076 28077
28078 28079 28080 28081 28082
1 2 3 4 5
10 6 7 8 9
11 12 13 15 16
17 18 19 20 21
22 23 27814 27815 28083

EOG5KH1B9

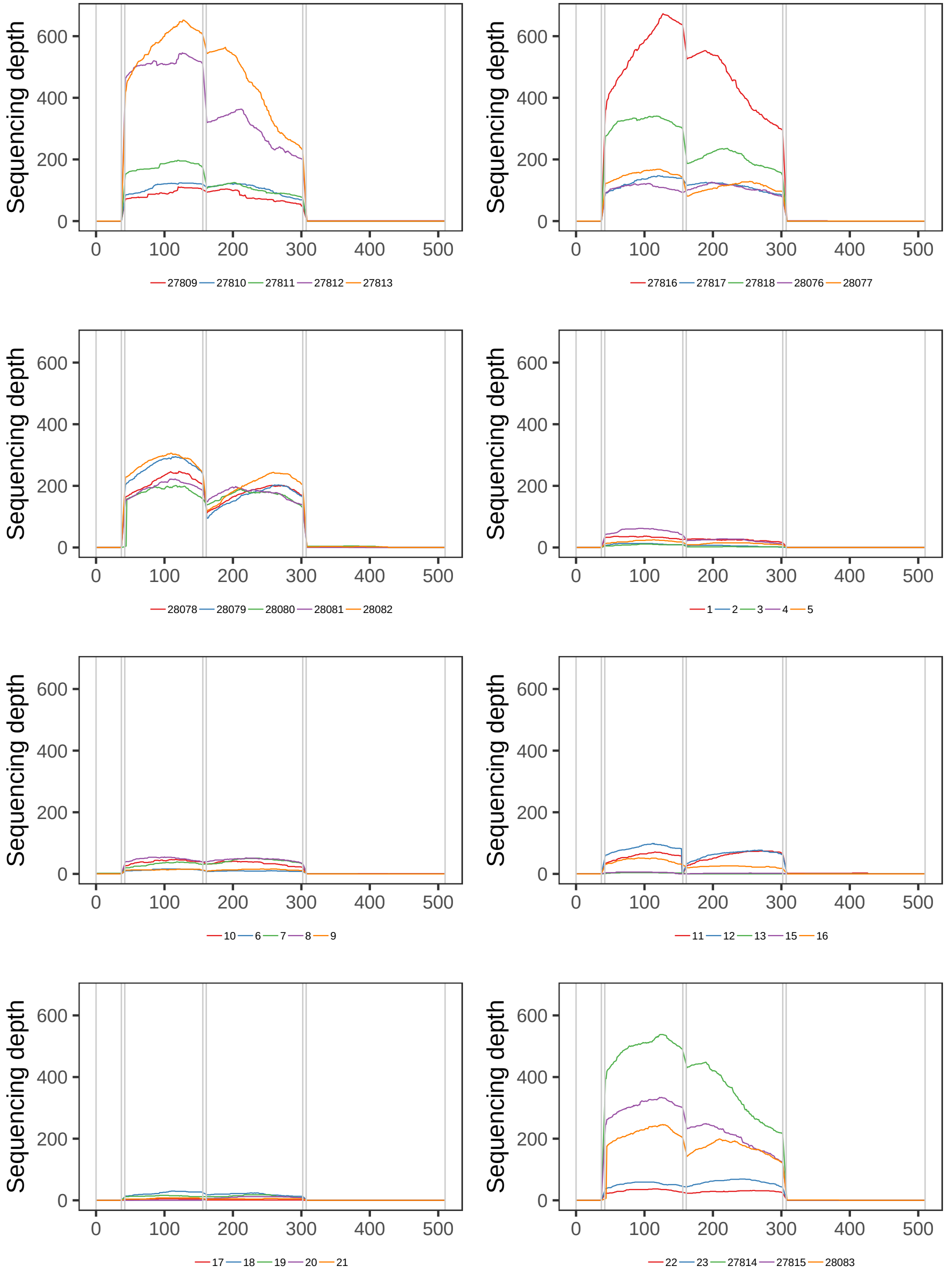
Sequencing depth
0
200
400
600
0
100
200
300
400
500
27809
27810
27811
27812
27813
27816
27817
27818
28076
28077
28078
28079
28080
28081
28082
1
2
3
4
5
10
6
7
8
9
11
12
13
15
16
17
18
19
20
21
22
23
27814
27815
28083

EOG5MPG5V

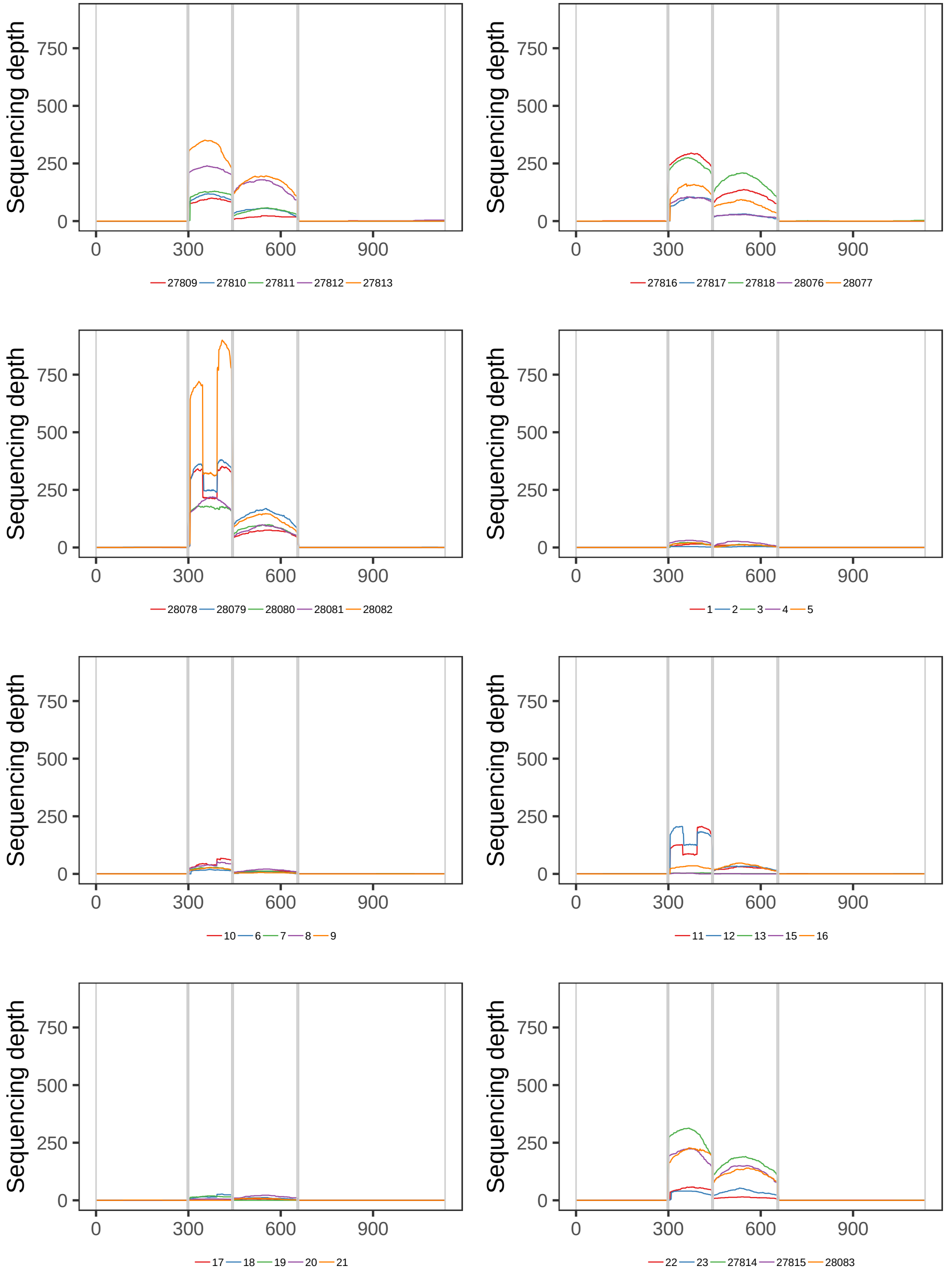
Sequencing depth
750
500
250
0
0
300
600
900
27809 27810 27811 27812 27813
27816 27817 27818 28076 28077
28078 28079 28080 28081 28082
1 2 3 4 5
10 6 7 8 9
11 12 13 15 16
17 18 19 20 21
22 23 27814 27815 28083

EOG5N5TCD

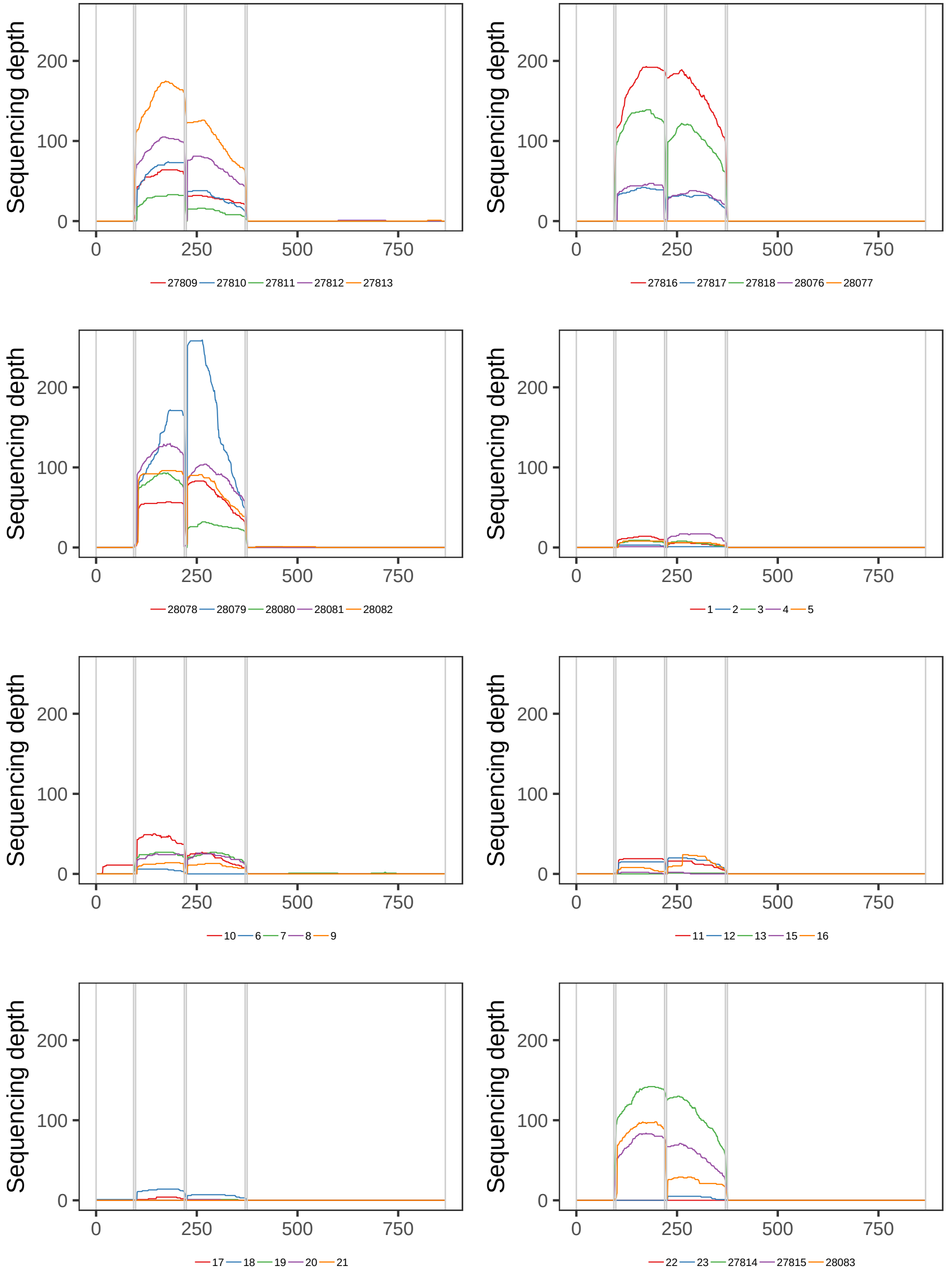
Sequencing depth
0
100
200
0
250
500
750
27809 27810 27811 27812 27813
27816 27817 27818 28076 28077
28078 28079 28080 28081 28082
1 2 3 4 5
10 6 7 8 9
11 12 13 15 16
17 18 19 20 21
22 23 27814 27815 28083

EOG5P2NK1

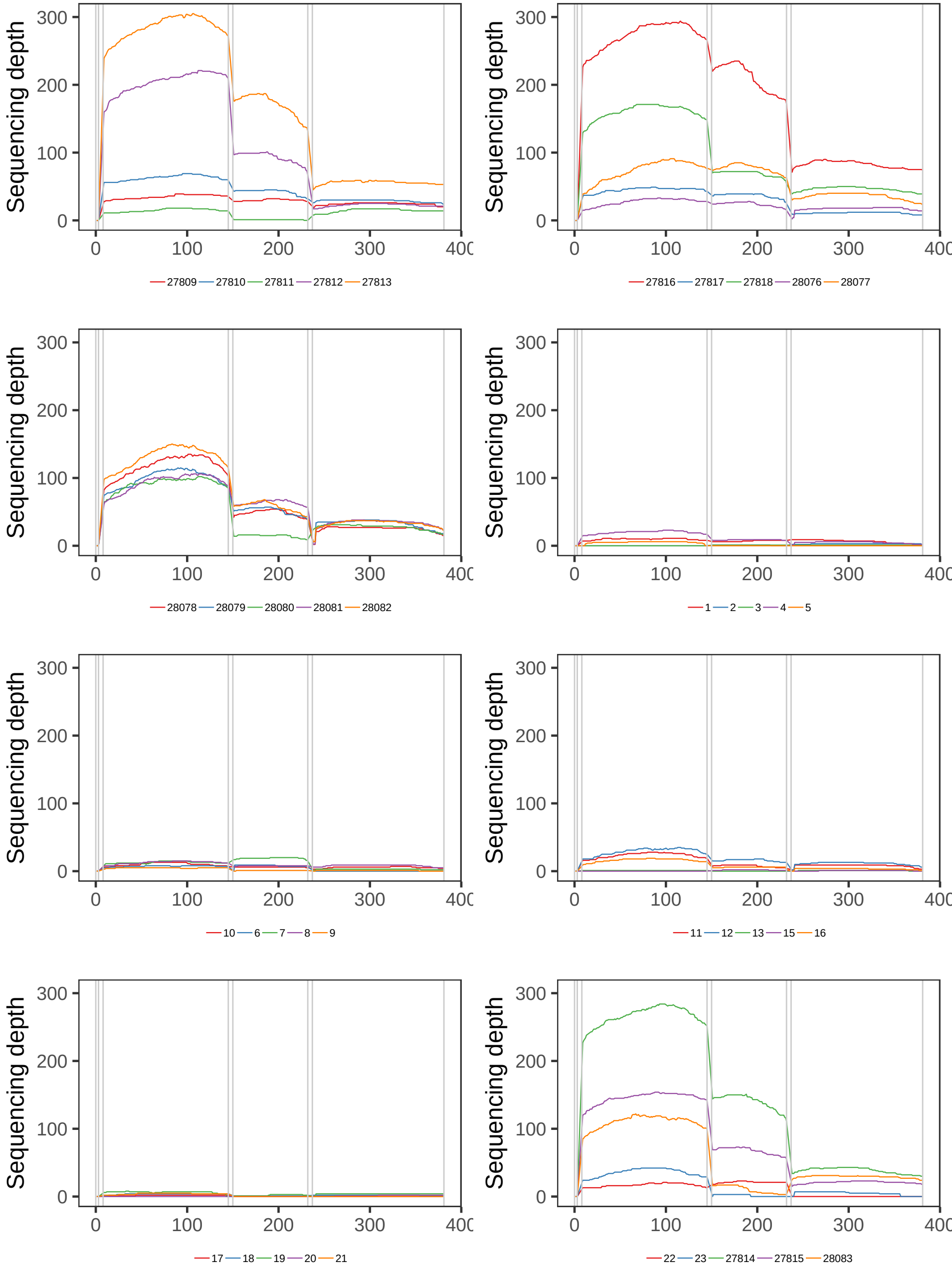
Sequencing depth
0
100
200
300
400
27809 27810 27811 27812 27813
27816 27817 27818 28076 28077
28078 28079 28080 28081 28082
1 2 3 4 5
10 6 7 8 9
11 12 13 15 16
17 18 19 20 21
22 23 27814 27815 28083

EOG5PVMDR

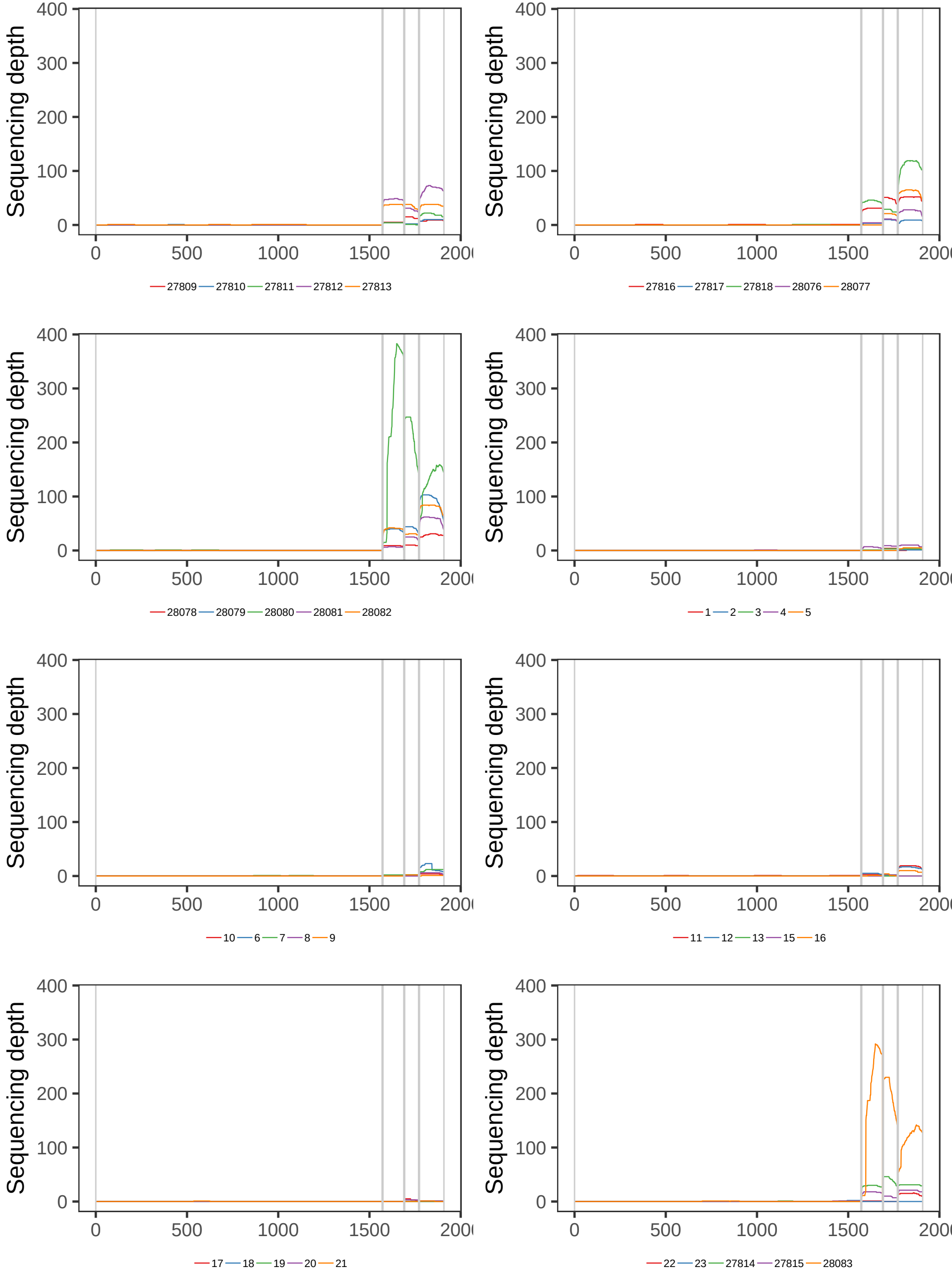

Sequencing depth
0
100
200
300
400
0
500
1000
1500
2000
27809 27810 27811 27812 27813
27816 27817 27818 28076 28077
28078 28079 28080 28081 28082
1 2 3 4 5
10 6 7 8 9
11 12 13 15 16
17 18 19 20 21
22 23 27814 27815 28083

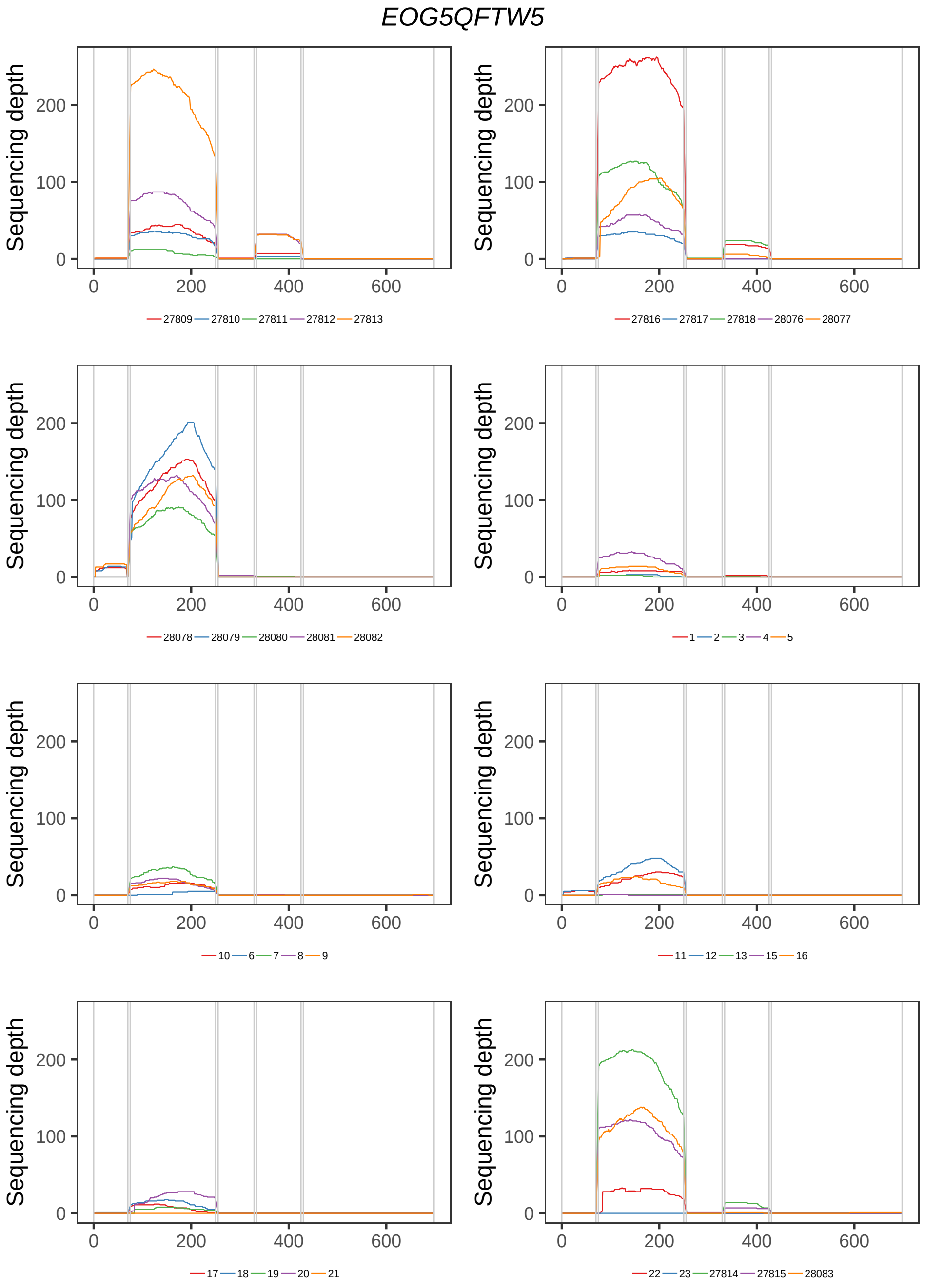

EOG5QFTW5
Sequencing depth
0
100
200
0
200
400
600
27809
27810
27811
27812
27813
27816
27817
27818
28076
28077
28078
28079
28080
28081
28082
1
2
3
4
5
10
6
7
8
9
11
12
13
15
16
17
18
19
20
21
22
23
27814
27815
28083

EOG5W3R42

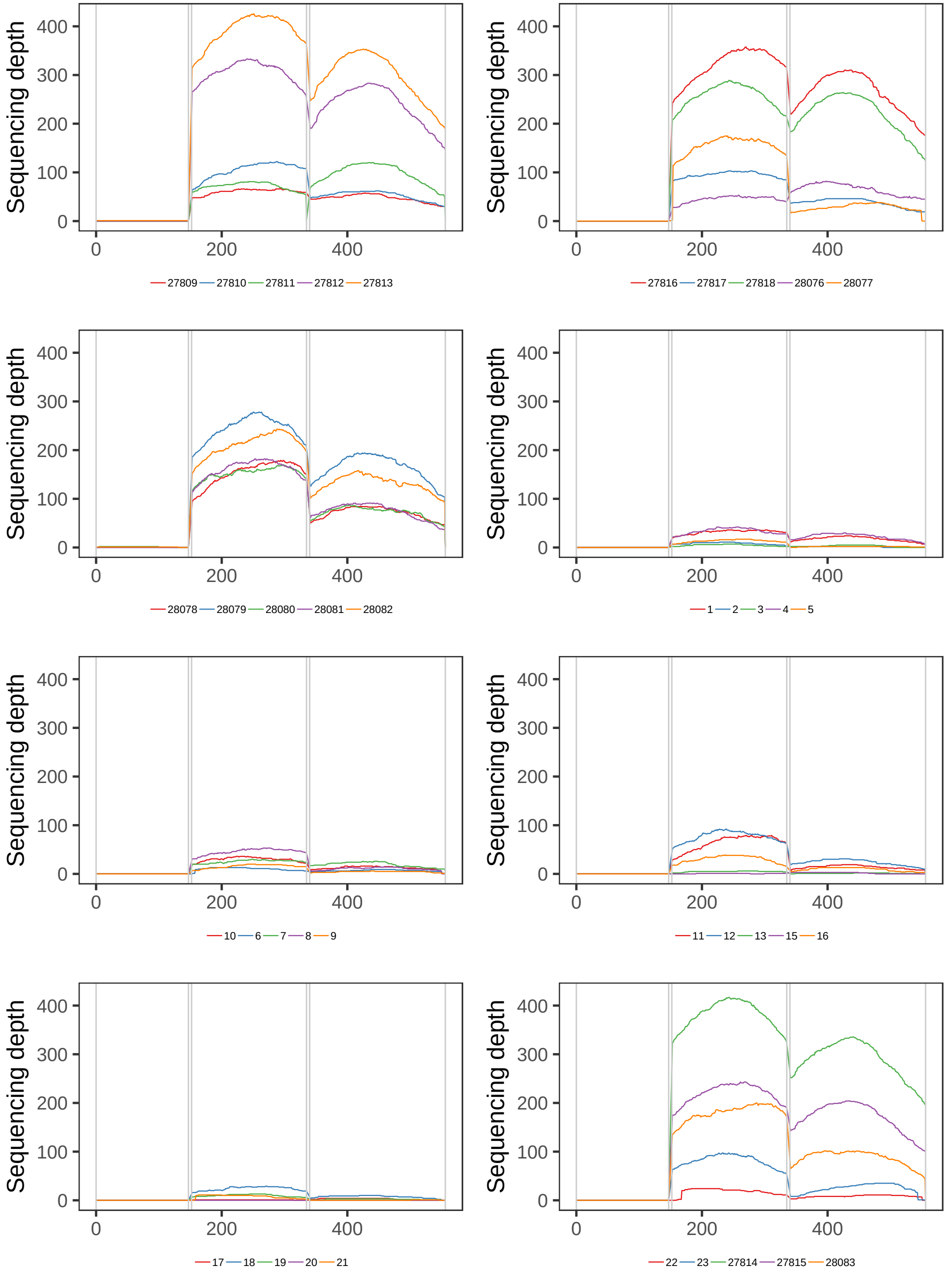
Sequencing depth
0
100
200
300
400
0
200
400
27809 27810 27811 27812 27813
27816 27817 27818 28076 28077
28078 28079 28080 28081 28082
1 2 3 4 5
10 6 7 8 9
11 12 13 15 16
17 18 19 20 21
22 23 27814 27815 28083

EOG5WDBV4

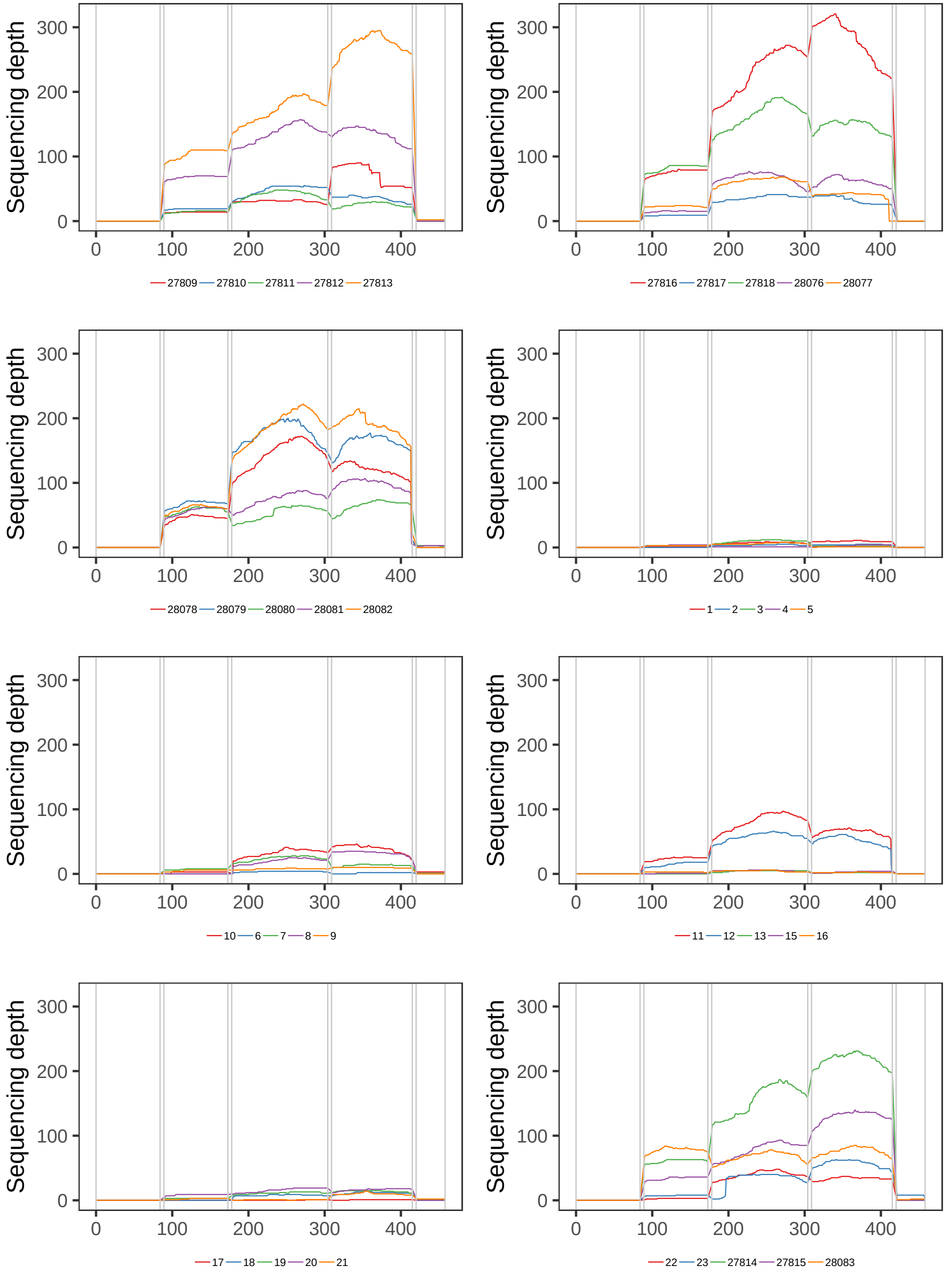
Sequencing depth
0
100
200
300
400
27809 27810 27811 27812 27813
27816 27817 27818 28076 28077
28078 28079 28080 28081 28082
1 2 3 4 5
10 6 7 8 9
11 12 13 15 16
17 18 19 20 21
22 23 27814 27815 28083

EOG5XWDDP

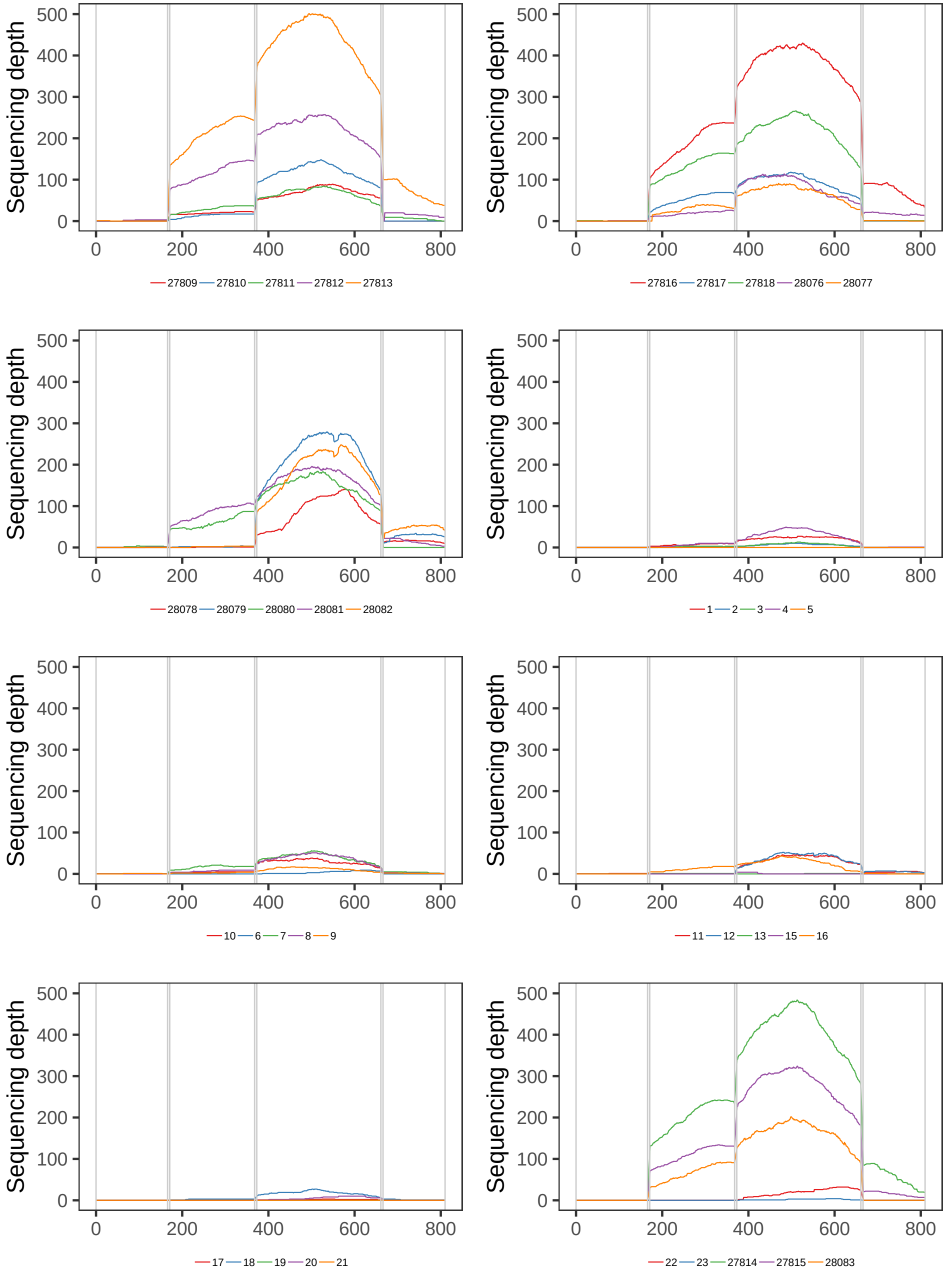
Sequencing depth
0
100
200
300
400
500
0
200
400
600
800
27809 27810 27811 27812 27813
27816 27817 27818 28076 28077
28078 28079 28080 28081 28082
1 2 3 4 5
10 6 7 8 9
11 12 13 15 16
17 18 19 20 21
22 23 27814 27815 28083

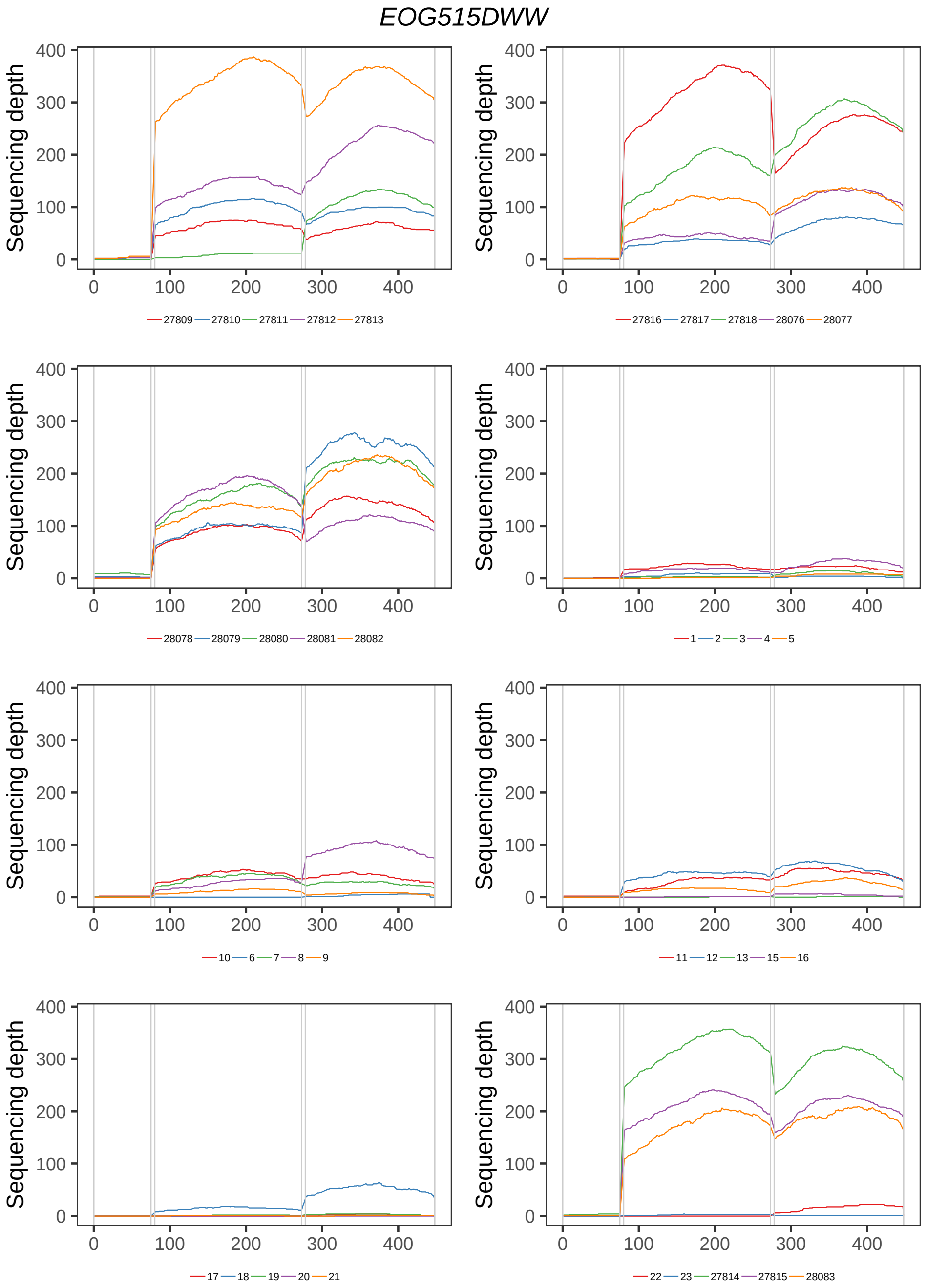
EOG515DWW
Sequencing depth
27809 27810 27811 27812 27813
27816 27817 27818 28076 28077
28078 28079 28080 28081 28082
1 2 3 4 5
10 6 7 8 9
11 12 13 15 16
17 18 19 20 21
22 23 27814 27815 28083

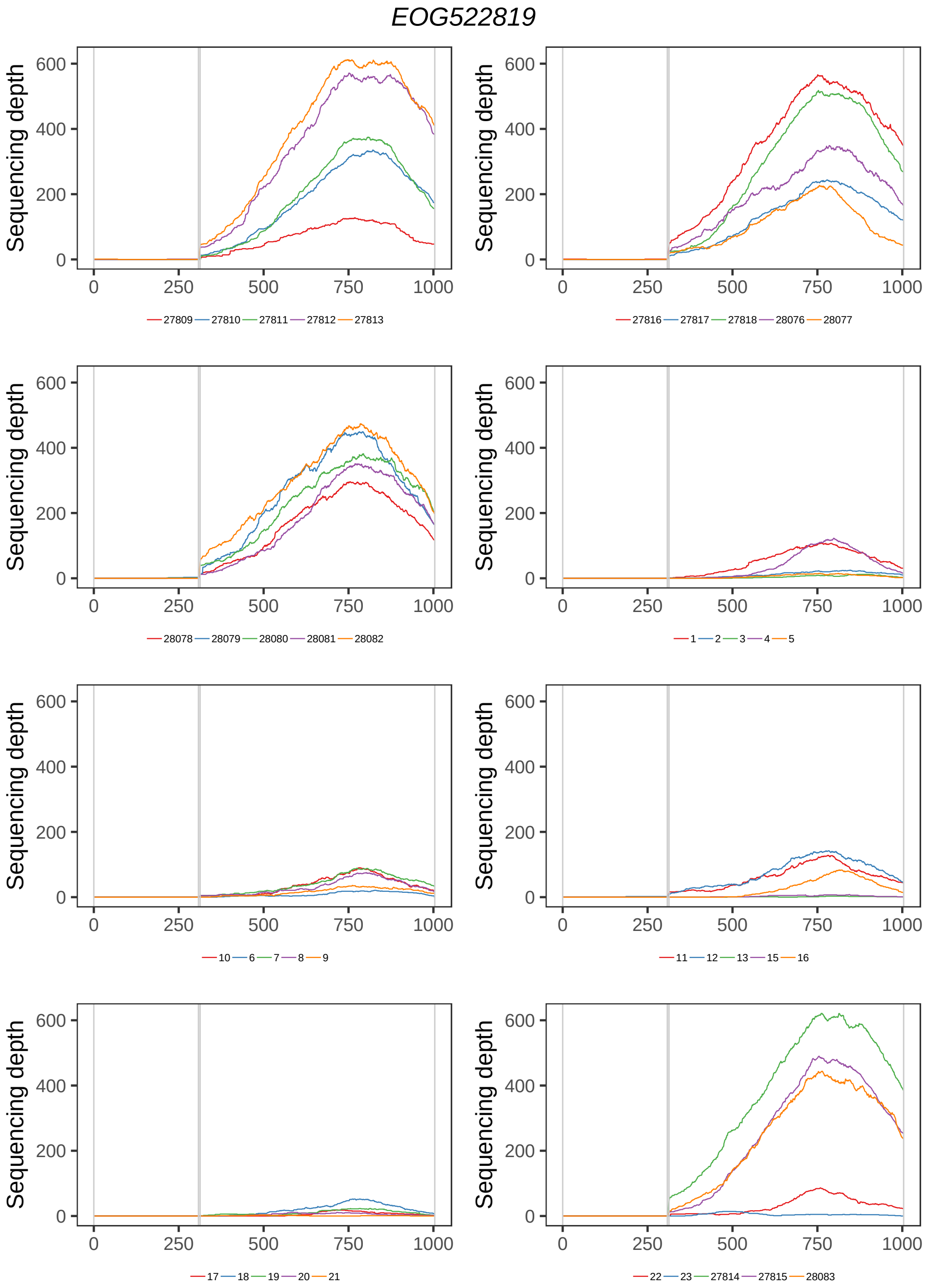
EOG522819
Sequencing depth
27809 27810 27811 27812 27813
27816 27817 27818 28076 28077
28078 28079 28080 28081 28082
1 2 3 4 5
10 6 7 8 9
11 12 13 15 16
17 18 19 20 21
22 23 27814 27815 28083

EOG541NSV

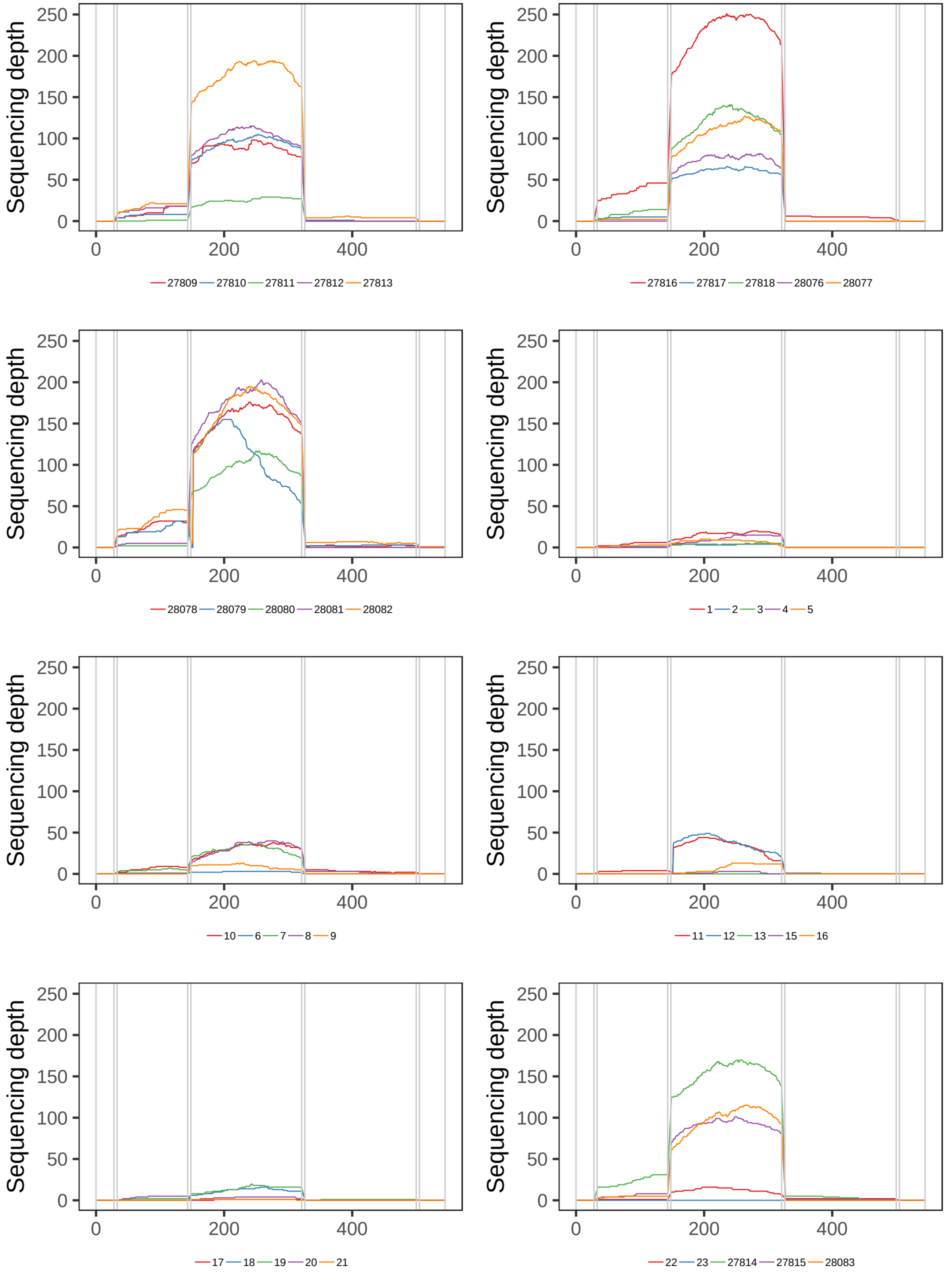
Sequencing depth
0
50
100
150
200
250
0
200
400
27809
27810
27811
27812
27813
27816
27817
27818
28076
28077
28078
28079
28080
28081
28082
1
2
3
4
5
10
6
7
8
9
11
12
13
15
16
17
18
19
20
21
22
23
27814
27815
28083

EOG56DJJG

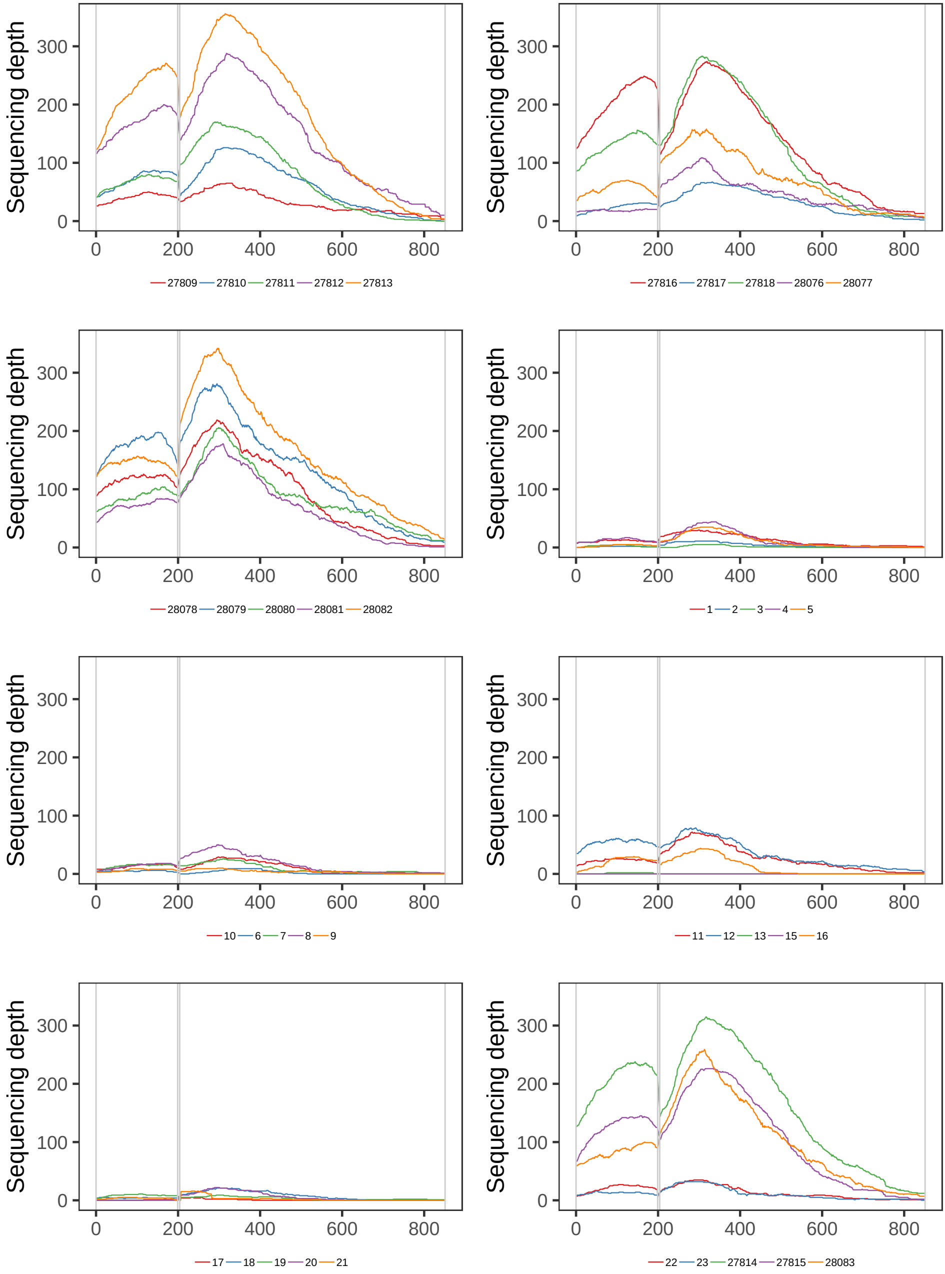
Sequencing depth
0
100
200
300
0
200
400
600
800
27809 27810 27811 27812 27813
27816 27817 27818 28076 28077
28078 28079 28080 28081 28082
1 2 3 4 5
10 6 7 8 9
11 12 13 15 16
17 18 19 20 21
22 23 27814 27815 28083

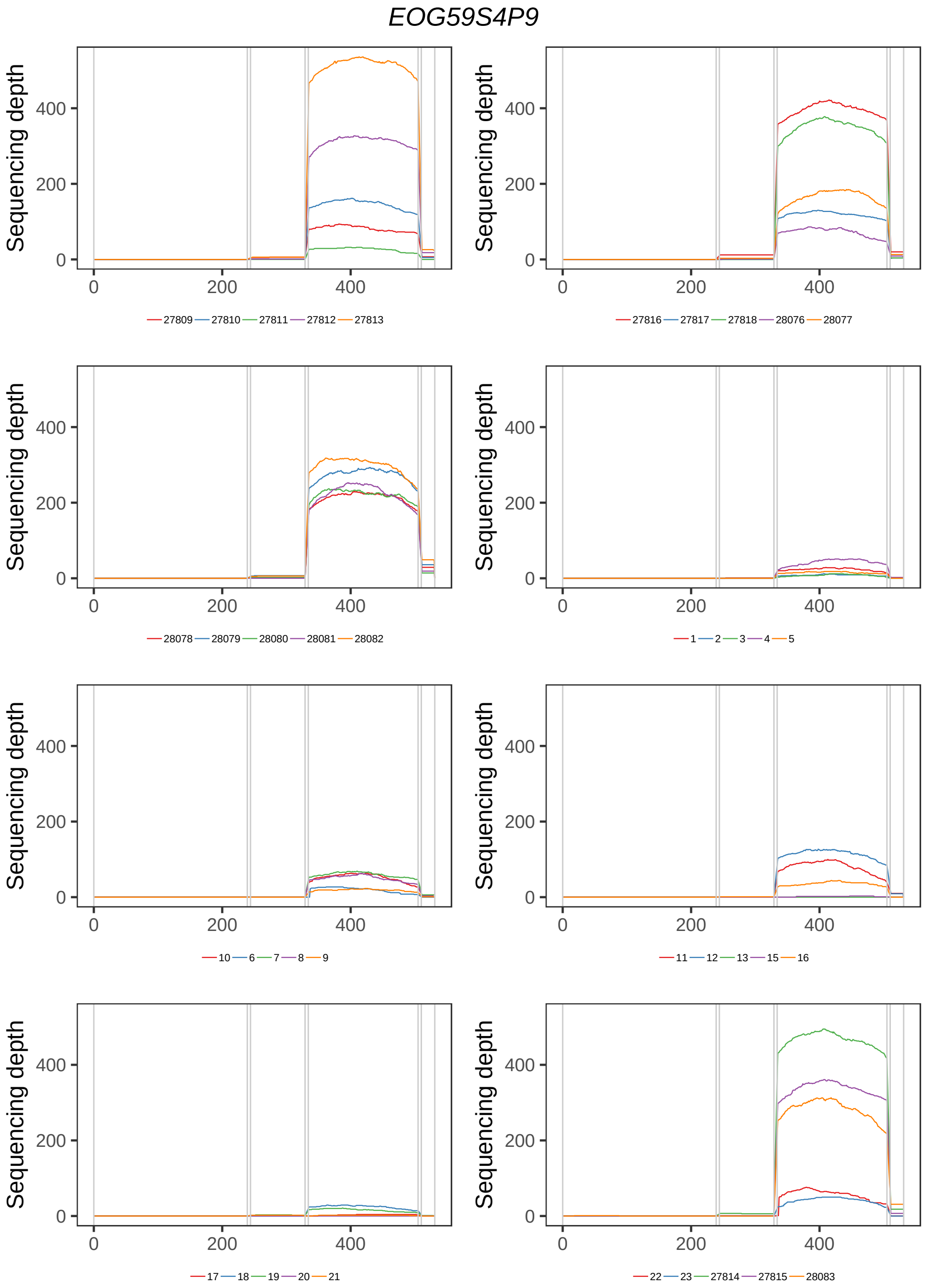
EOG59S4P9
Sequencing depth
0
200
400
27809 27810 27811 27812 27813
27816 27817 27818 28076 28077
28078 28079 28080 28081 28082
1 2 3 4 5
10 6 7 8 9
11 12 13 15 16
17 18 19 20 21
22 23 27814 27815 28083

EOG5B5MM6

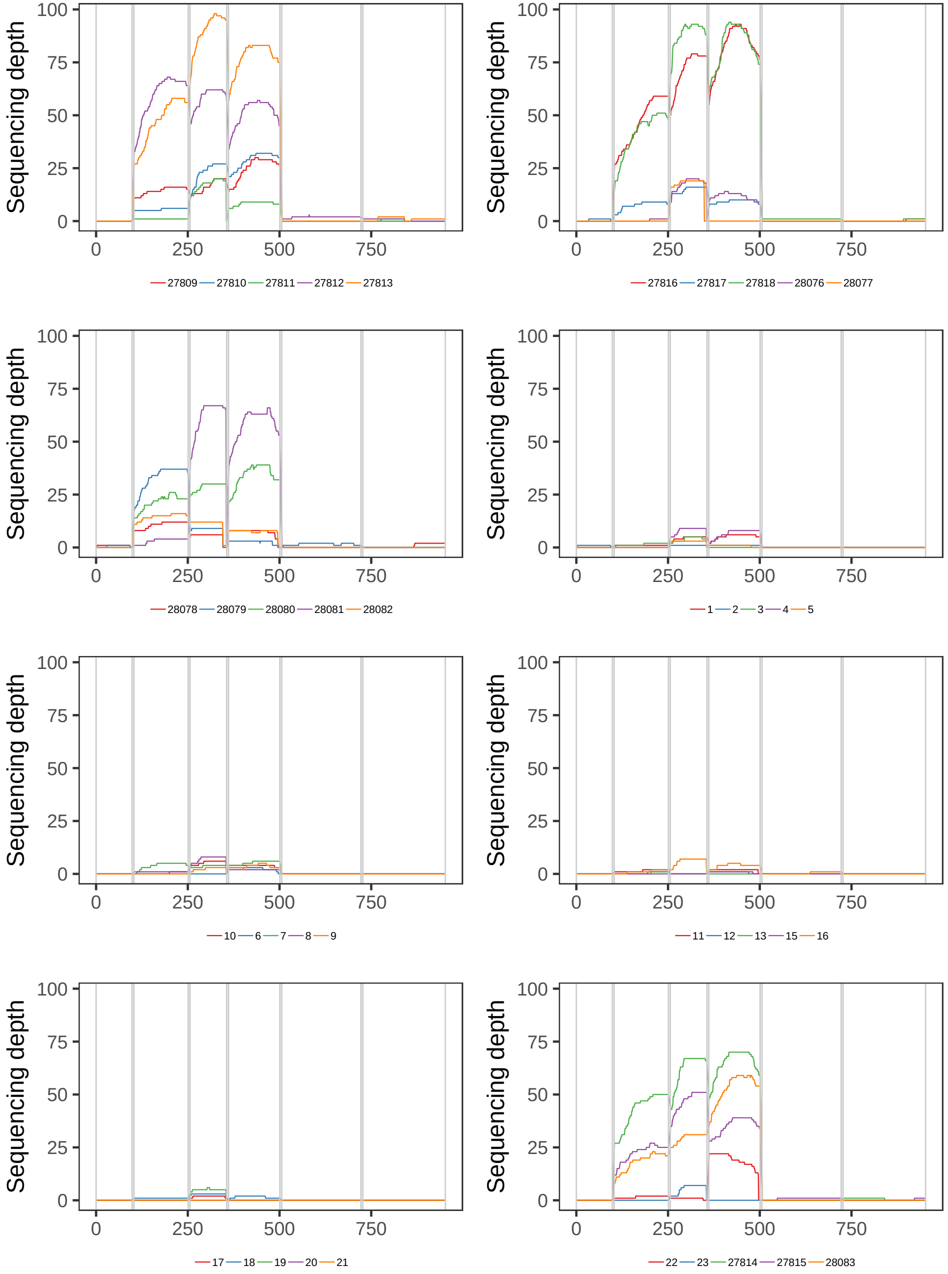
Sequencing depth
0
25
50
75
100
0
250
500
750
27809 27810 27811 27812 27813
27816 27817 27818 28076 28077
28078 28079 28080 28081 28082
1 2 3 4 5
10 6 7 8 9
11 12 13 15 16
17 18 19 20 21
22 23 27814 27815 28083

EOG5BK3JZ

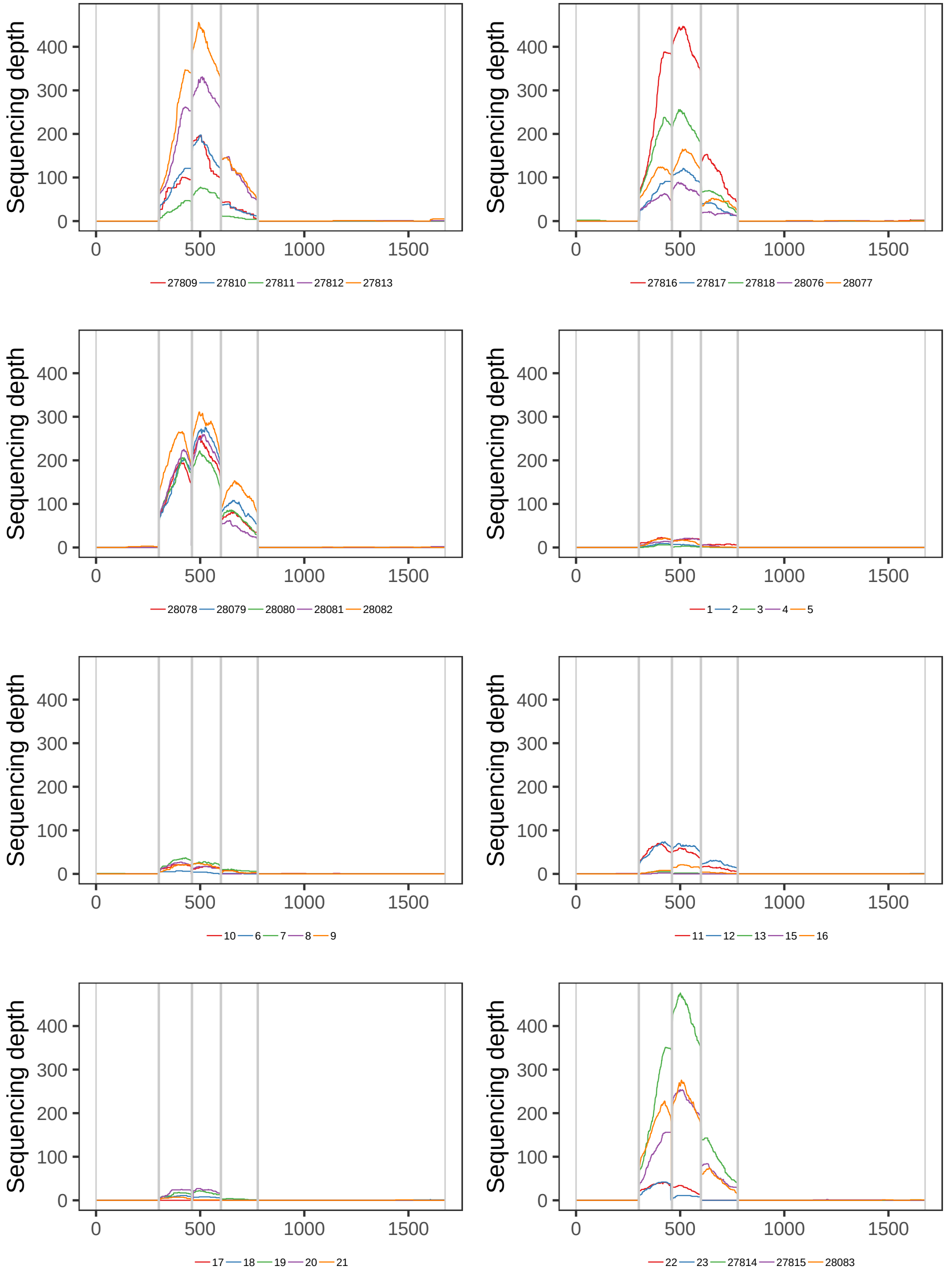
Sequencing depth
0
100
200
300
400
0
500
1000
1500
27809 27810 27811 27812 27813
27816 27817 27818 28076 28077
28078 28079 28080 28081 28082
1 2 3 4 5
10 6 7 8 9
11 12 13 15 16
17 18 19 20 21
22 23 27814 27815 28083

EOG5D51D7

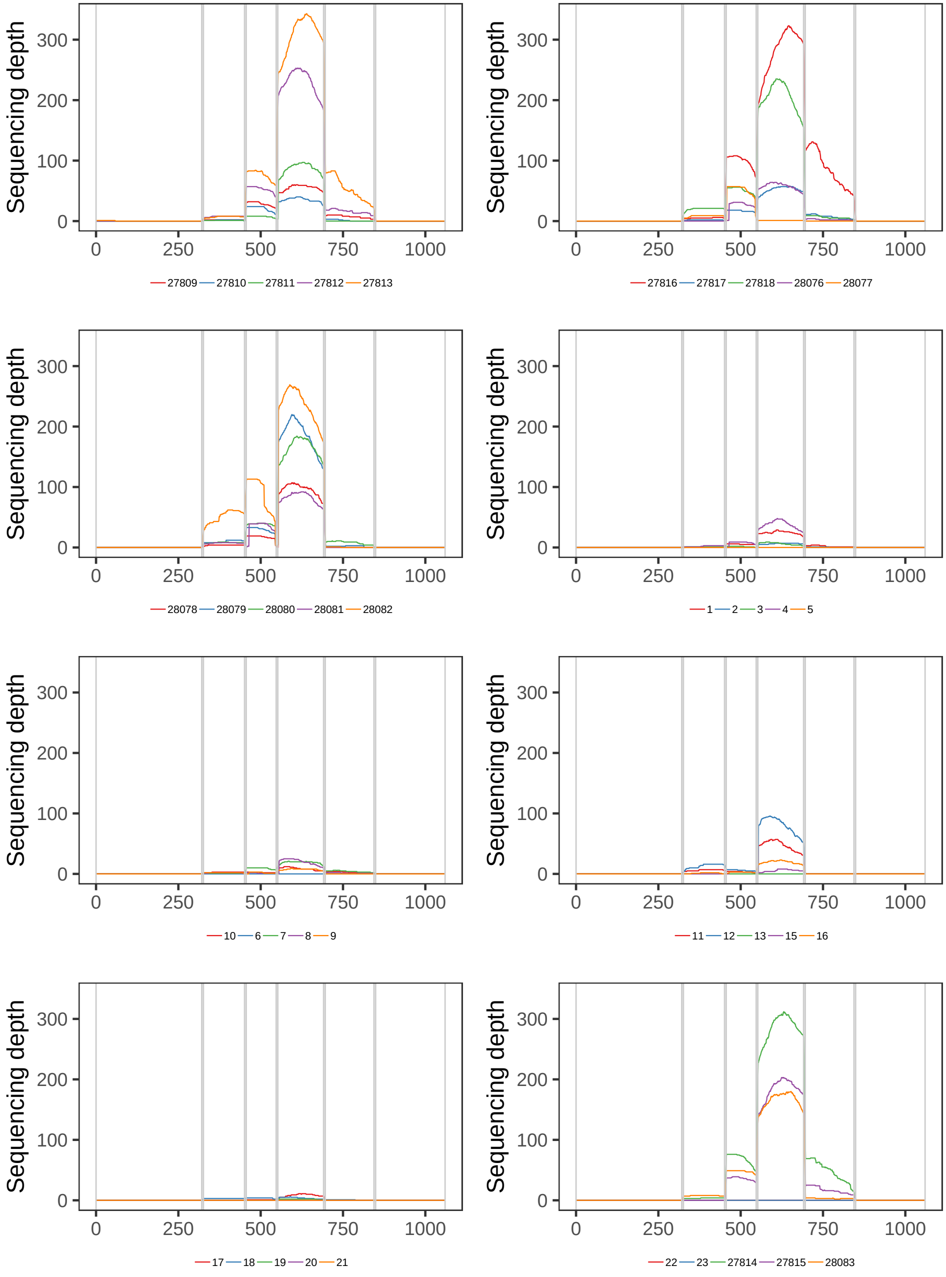
Sequencing depth
0
100
200
300
0
250
500
750
1000
27809 27810 27811 27812 27813
27816 27817 27818 28076 28077
28078 28079 28080 28081 28082
1 2 3 4 5
10 6 7 8 9
11 12 13 15 16
17 18 19 20 21
22 23 27814 27815 28083

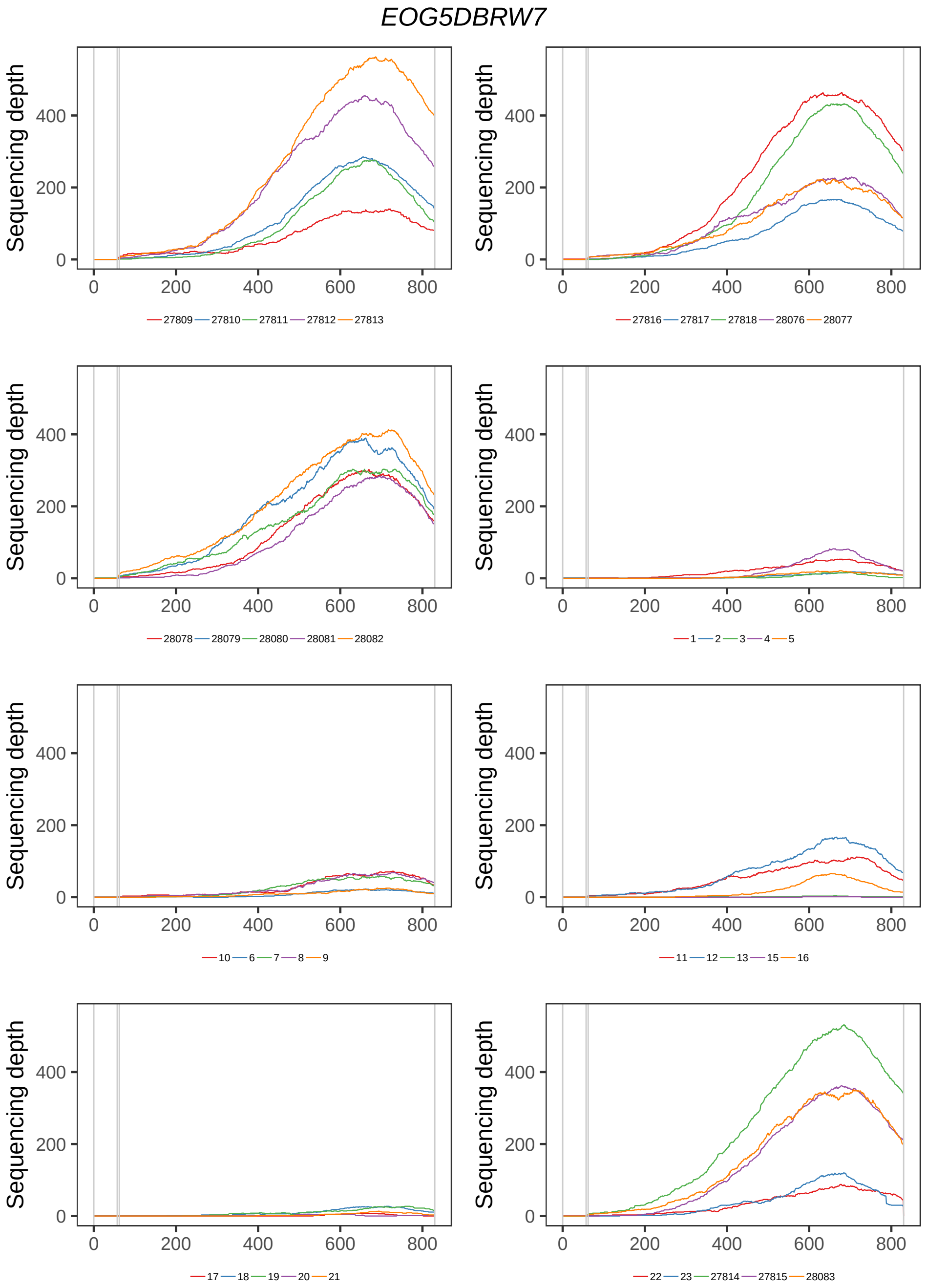
EOG5DBRW7
Sequencing depth
27809 27810 27811 27812 27813
27816 27817 27818 28076 28077
28078 28079 28080 28081 28082
1 2 3 4 5
10 6 7 8 9
11 12 13 15 16
17 18 19 20 21
22 23 27814 27815 28083

EOG5G4F6H

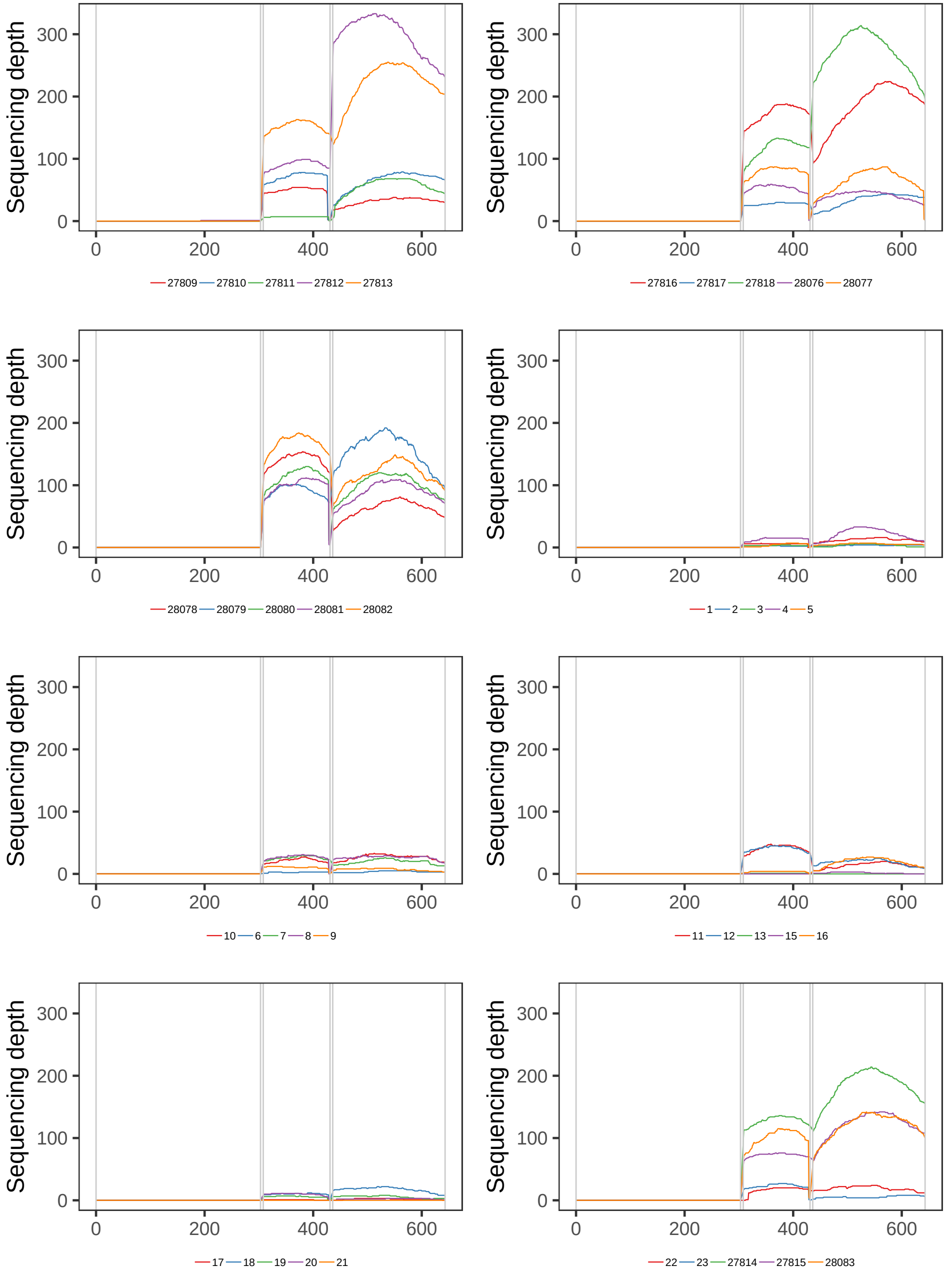

Sequencing depth
300
200
100
0
0
200
400
600
27809
27810
27811
27812
27813
27816
27817
27818
28076
28077
28078
28079
28080
28081
28082
1
2
3
4
5
10
6
7
8
9
11
12
13
15
16
17
18
19
20
21
22
23
27814
27815
28083

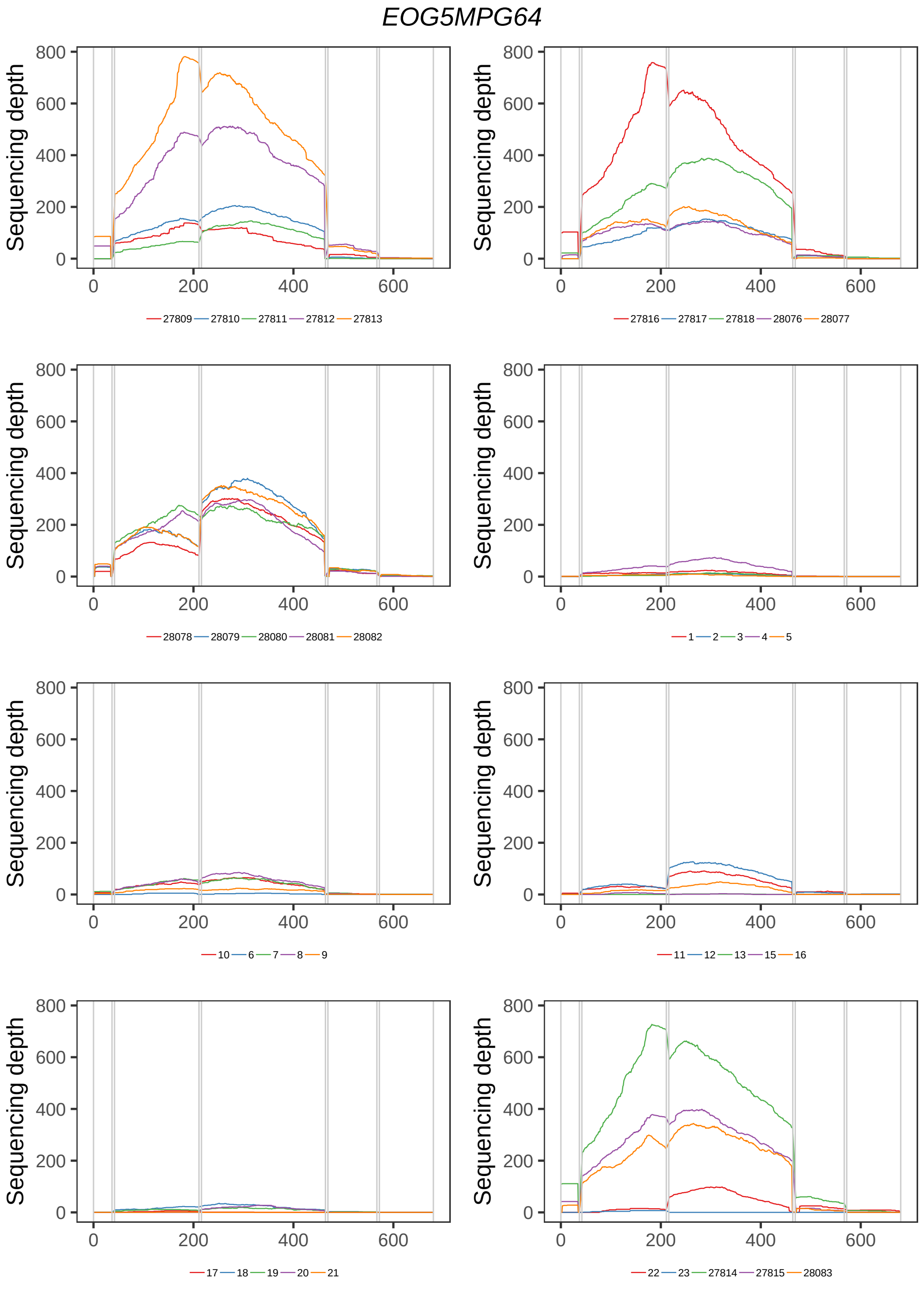
EOG5MPG64
Sequencing depth
800
600
400
200
0
0
200
400
600
27809 27810 27811 27812 27813
27816 27817 27818 28076 28077
28078 28079 28080 28081 28082
1 2 3 4 5
10 6 7 8 9
11 12 13 15 16
17 18 19 20 21
22 23 27814 27815 28083

EOG5MSBF8

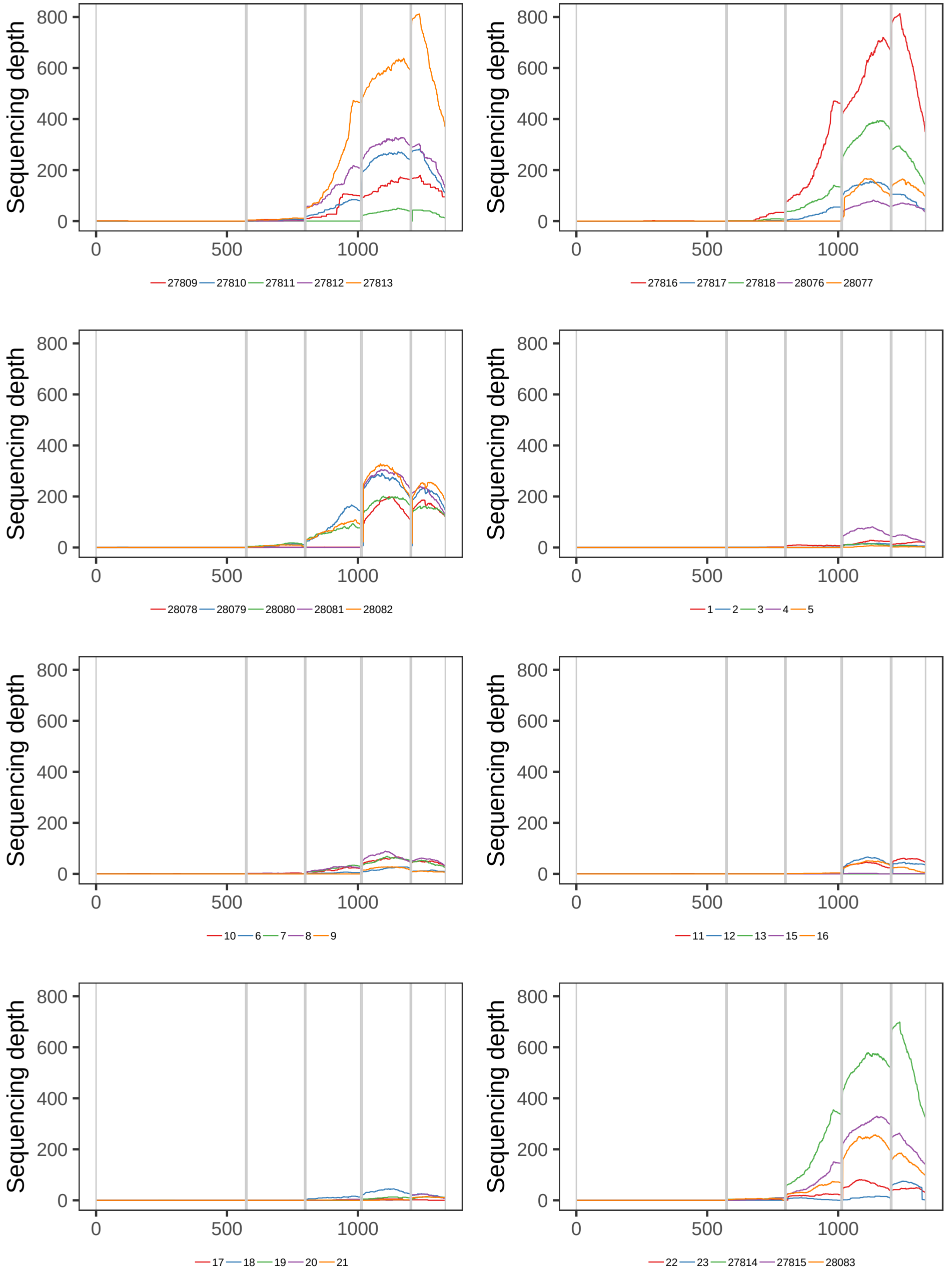
Sequencing depth
800
600
400
200
0
0
500
1000
27809 27810 27811 27812 27813
27816 27817 27818 28076 28077
28078 28079 28080 28081 28082
1 2 3 4 5
10 6 7 8 9
11 12 13 15 16
17 18 19 20 21
22 23 27814 27815 28083

EOG5MW6NZ

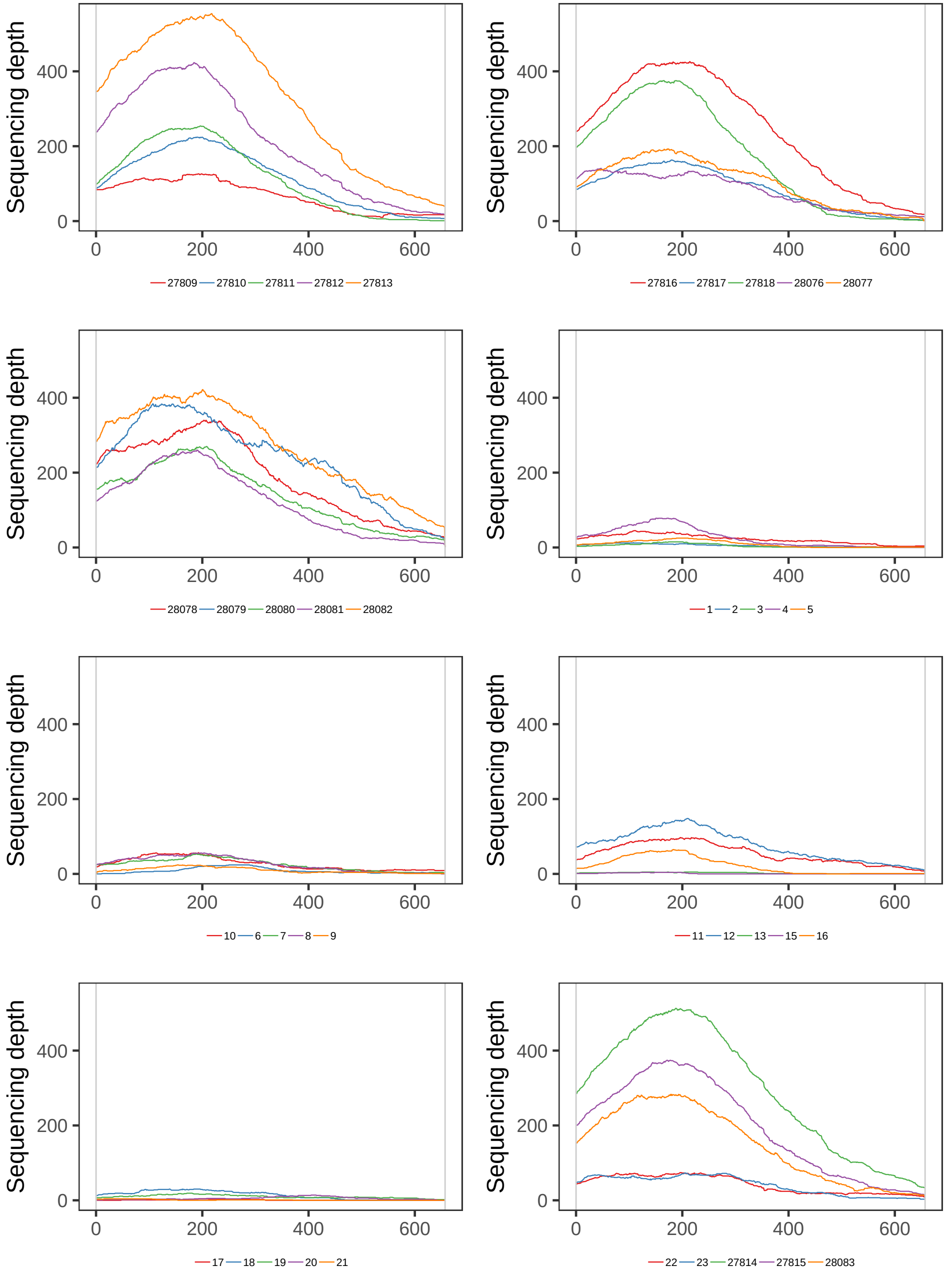

Sequencing depth
400
200
0
0
200
400
600
27809 27810 27811 27812 27813
27816 27817 27818 28076 28077
28078 28079 28080 28081 28082
1 2 3 4 5
10 6 7 8 9
11 12 13 15 16
17 18 19 20 21
22 23 27814 27815 28083

EOG5QNKB5

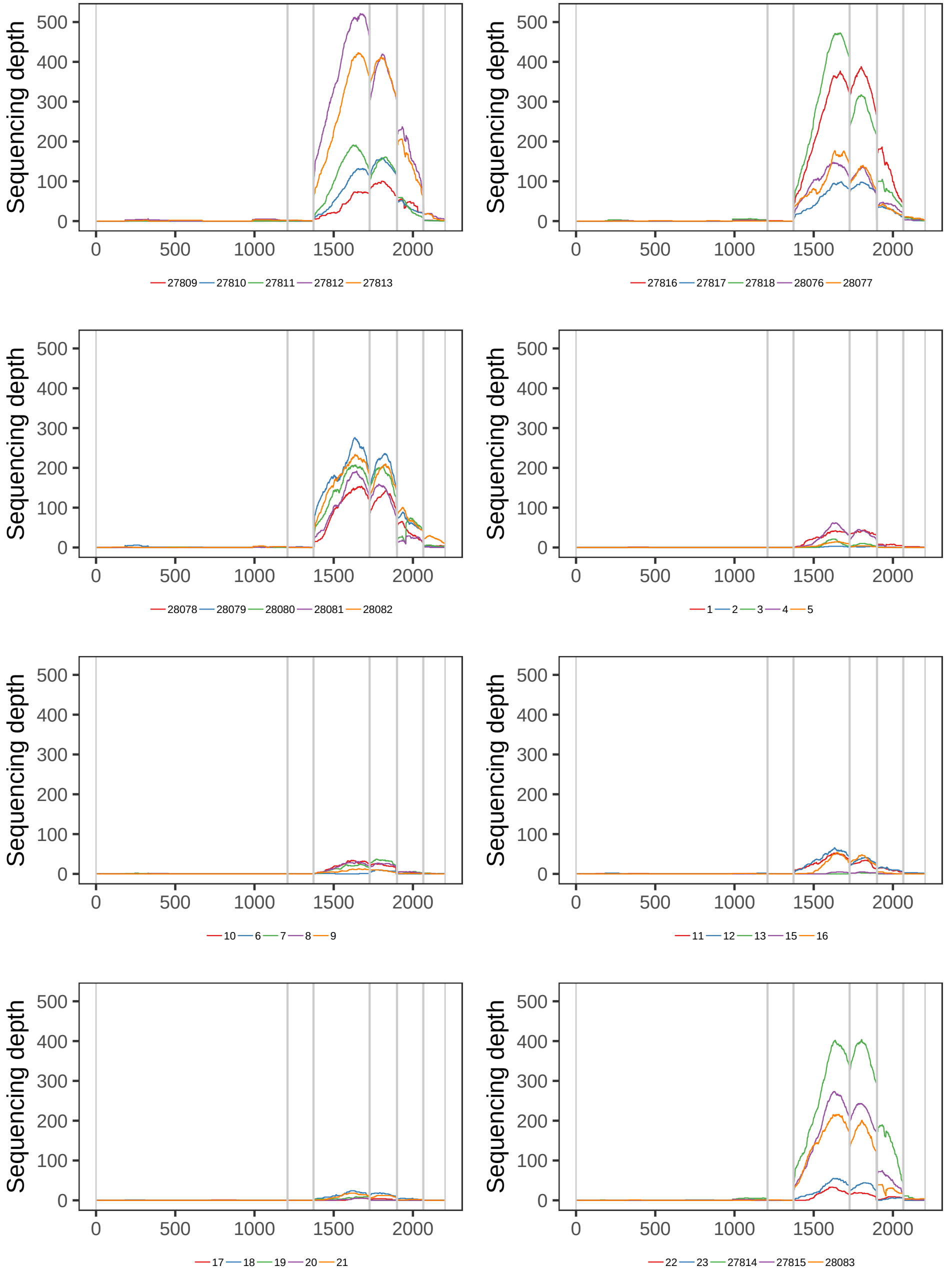
Sequencing depth
0
100
200
300
400
500
0
500
1000
1500
2000
27809 27810 27811 27812 27813
27816 27817 27818 28076 28077
28078 28079 28080 28081 28082
1 2 3 4 5
10 6 7 8 9
11 12 13 15 16
17 18 19 20 21
22 23 27814 27815 28083

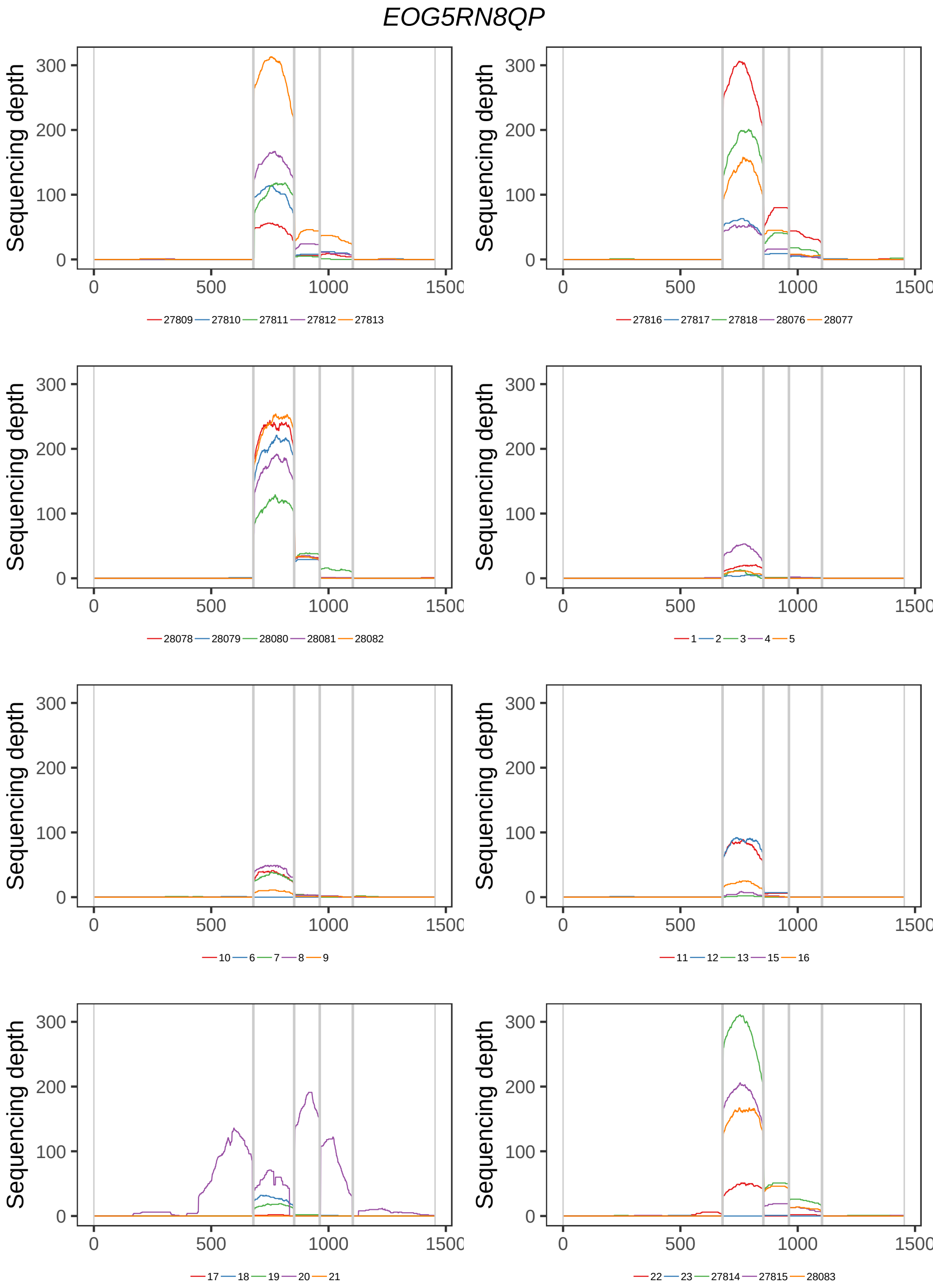
EOG5RN8QP
Sequencing depth
0
100
200
300
0
500
1000
1500
27809
27810
27811
27812
27813
27816
27817
27818
28076
28077
28078
28079
28080
28081
28082
1
2
3
4
5
10
6
7
8
9
11
12
13
15
16
17
18
19
20
21
22
23
27814
27815
28083

EOG5SXKTM

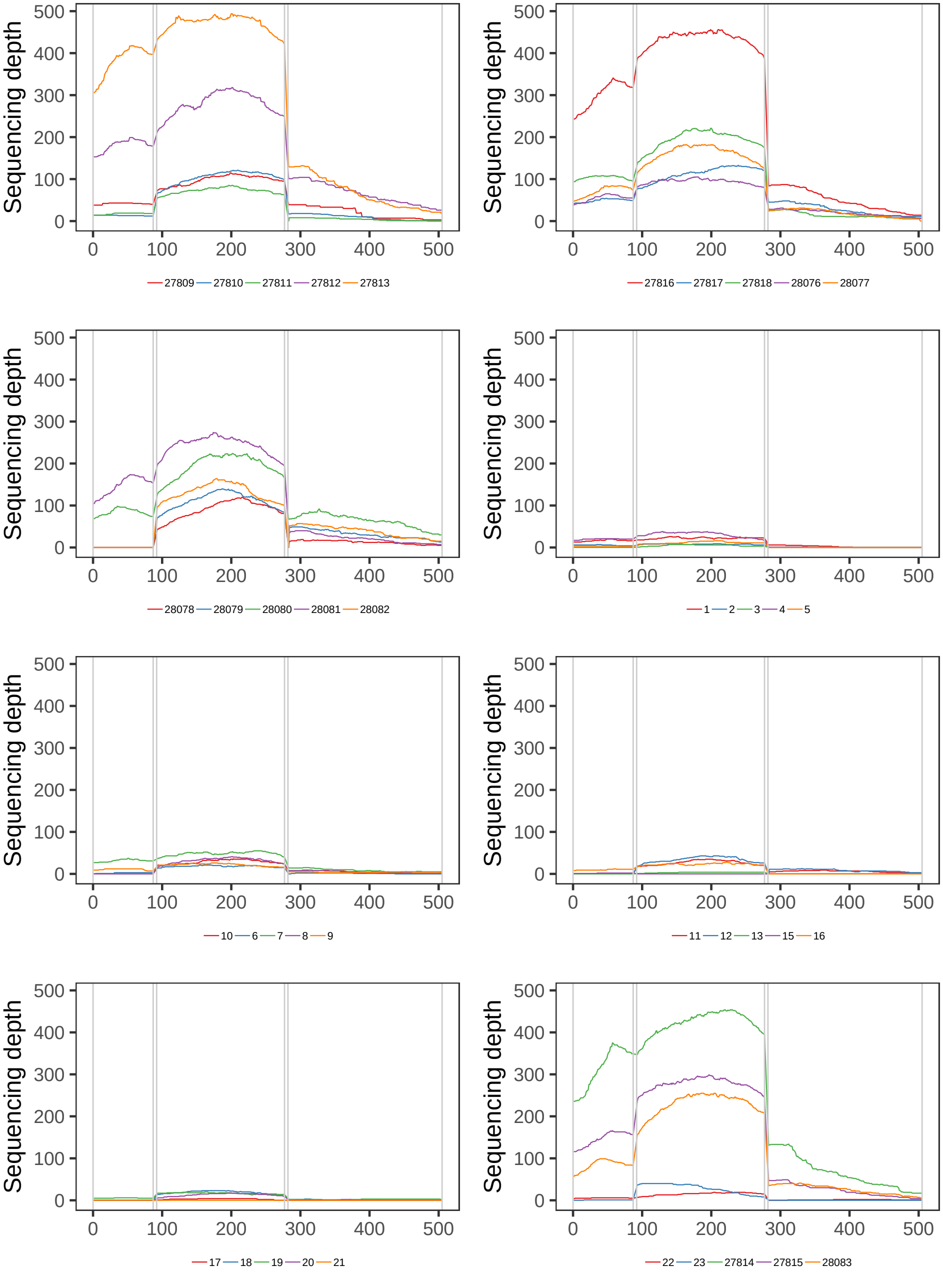
Sequencing depth
0
100
200
300
400
500
27809 27810 27811 27812 27813
27816 27817 27818 28076 28077
28078 28079 28080 28081 28082
1 2 3 4 5
10 6 7 8 9
11 12 13 15 16
17 18 19 20 21
22 23 27814 27815 28083

EOG5T1G2P

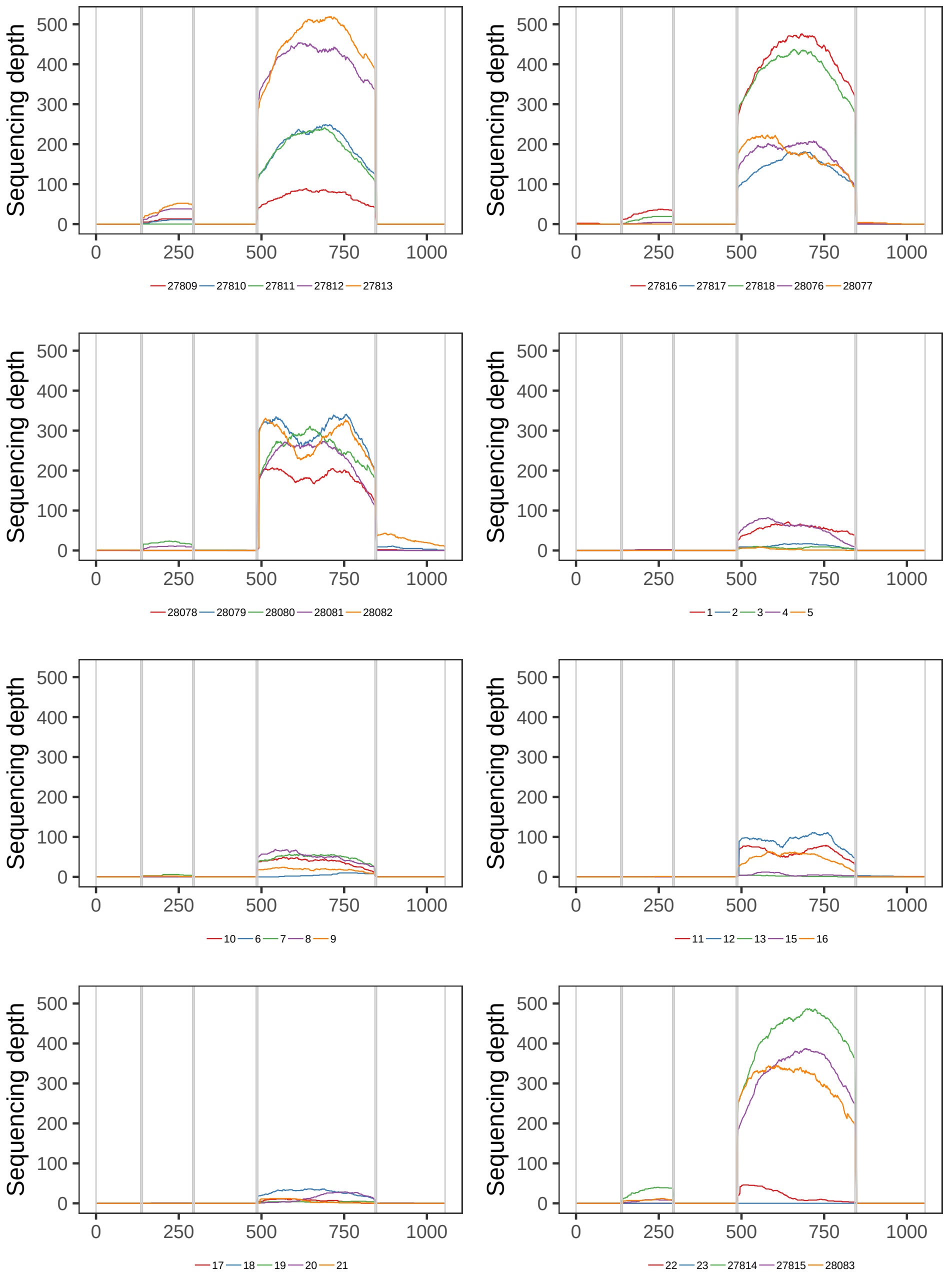
Sequencing depth
0
100
200
300
400
500
0
250
500
750
1000
27809 27810 27811 27812 27813
27816 27817 27818 28076 28077
28078 28079 28080 28081 28082
1 2 3 4 5
10 6 7 8 9
11 12 13 15 16
17 18 19 20 21
22 23 27814 27815 28083

EOG5VMCX4

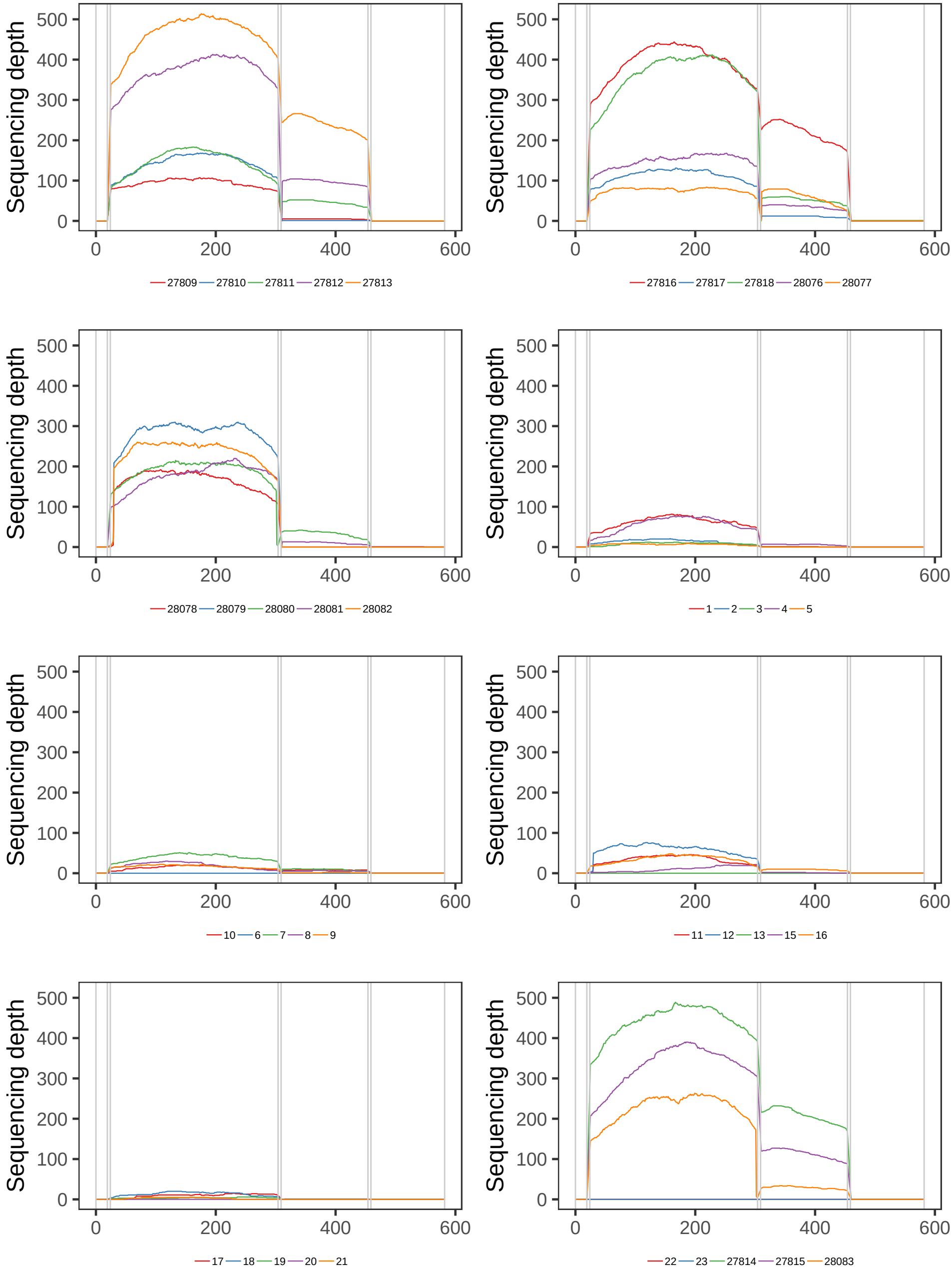
Sequencing depth
0
100
200
300
400
500
0
200
400
600
27809 27810 27811 27812 27813
27816 27817 27818 28076 28077
28078 28079 28080 28081 28082
1 2 3 4 5
10 6 7 8 9
11 12 13 15 16
17 18 19 20 21
22 23 27814 27815 28083

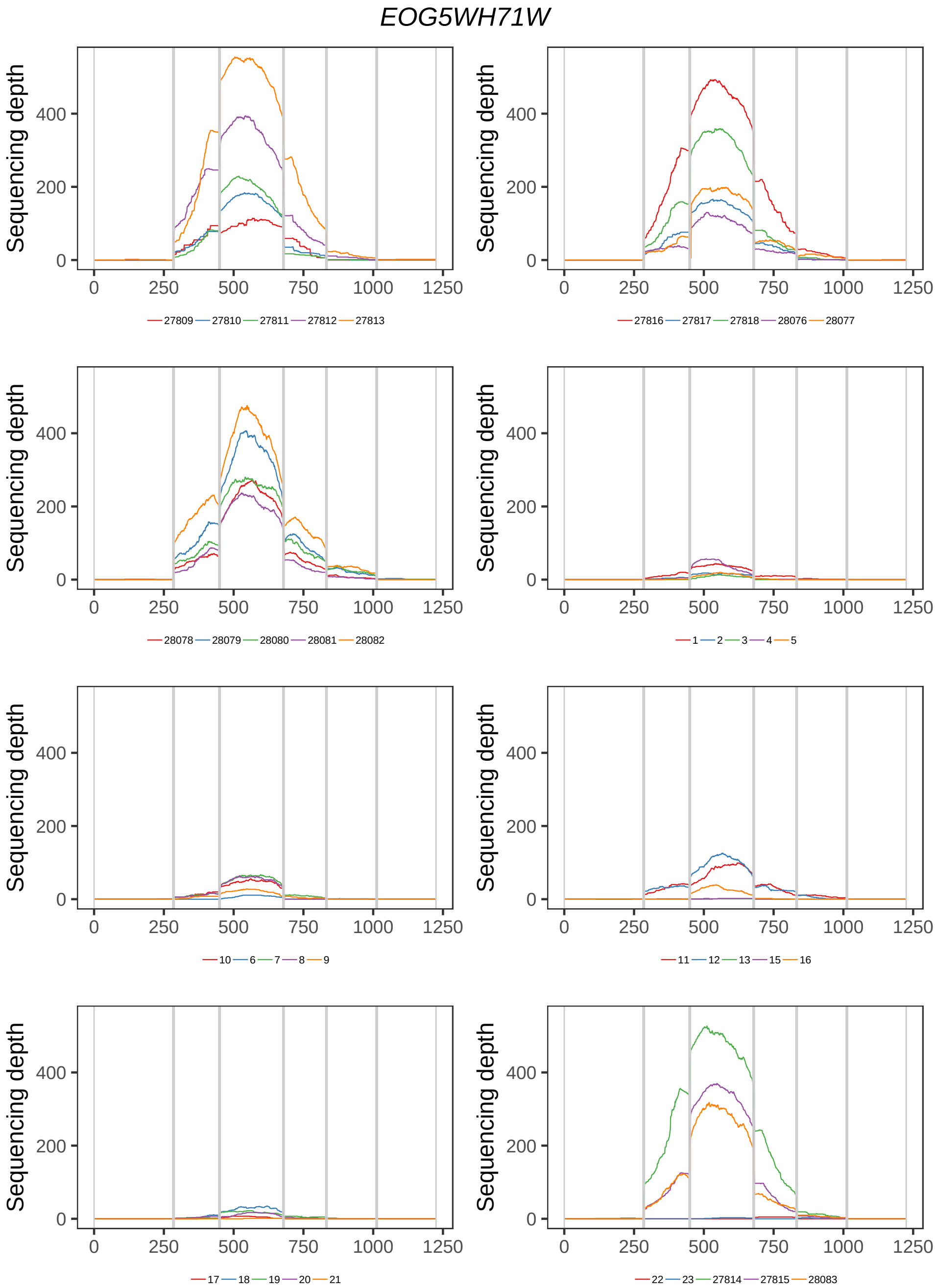
EOG5WH71W
Sequencing depth
0
200
400
0
250
500
750
1000
1250
27809
27810
27811
27812
27813
27816
27817
27818
28076
28077
28078
28079
28080
28081
28082
1
2
3
4
5
10
6
7
8
9
11
12
13
15
16
17
18
19
20
21
22
23
27814
27815
28083

EOG5WSTS9

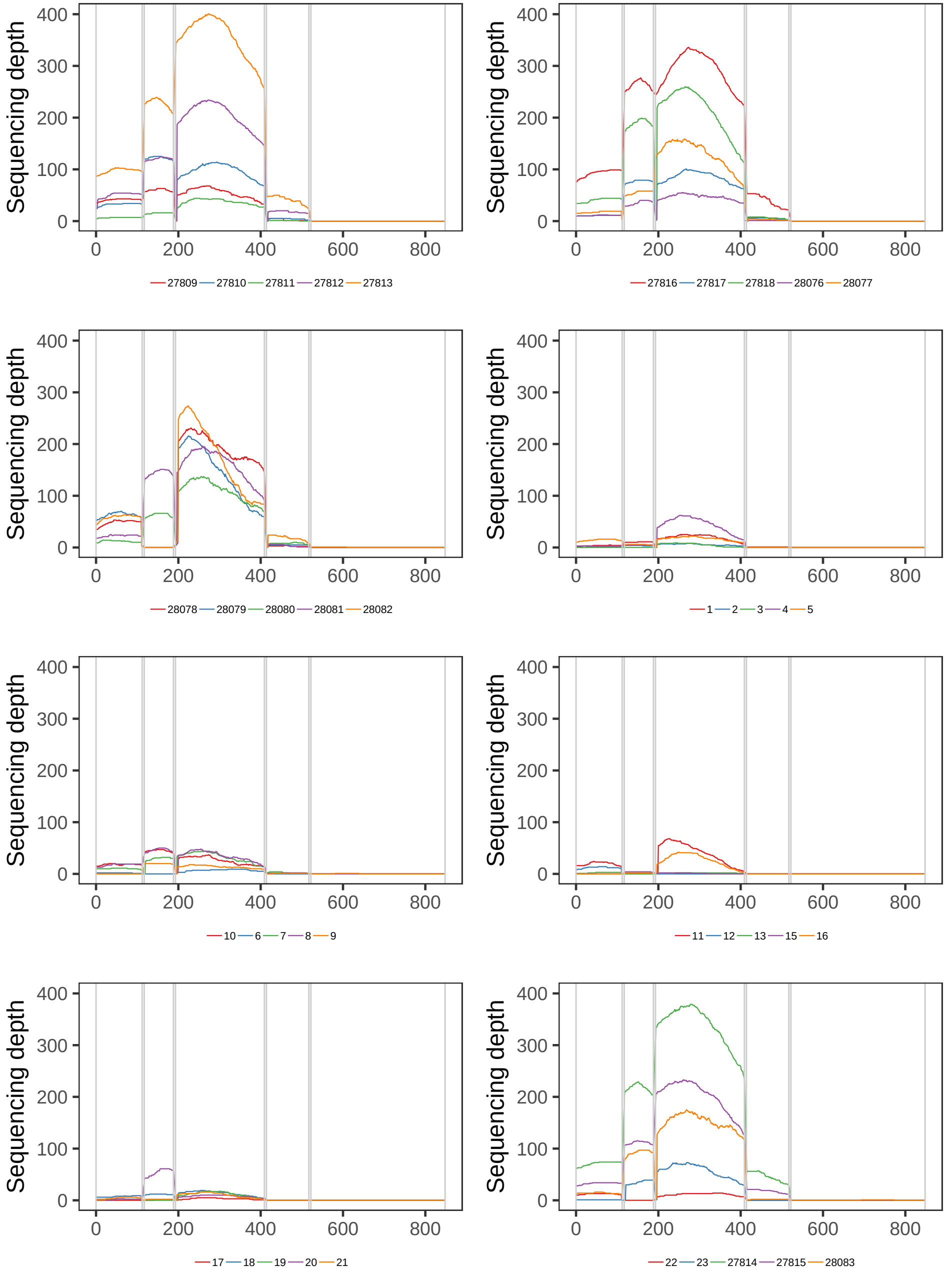

Sequencing depth
27809 27810 27811 27812 27813
27816 27817 27818 28076 28077
28078 28079 28080 28081 28082
1 2 3 4 5
10 6 7 8 9
11 12 13 15 16
17 18 19 20 21
22 23 27814 27815 28083

EOG54F4SC

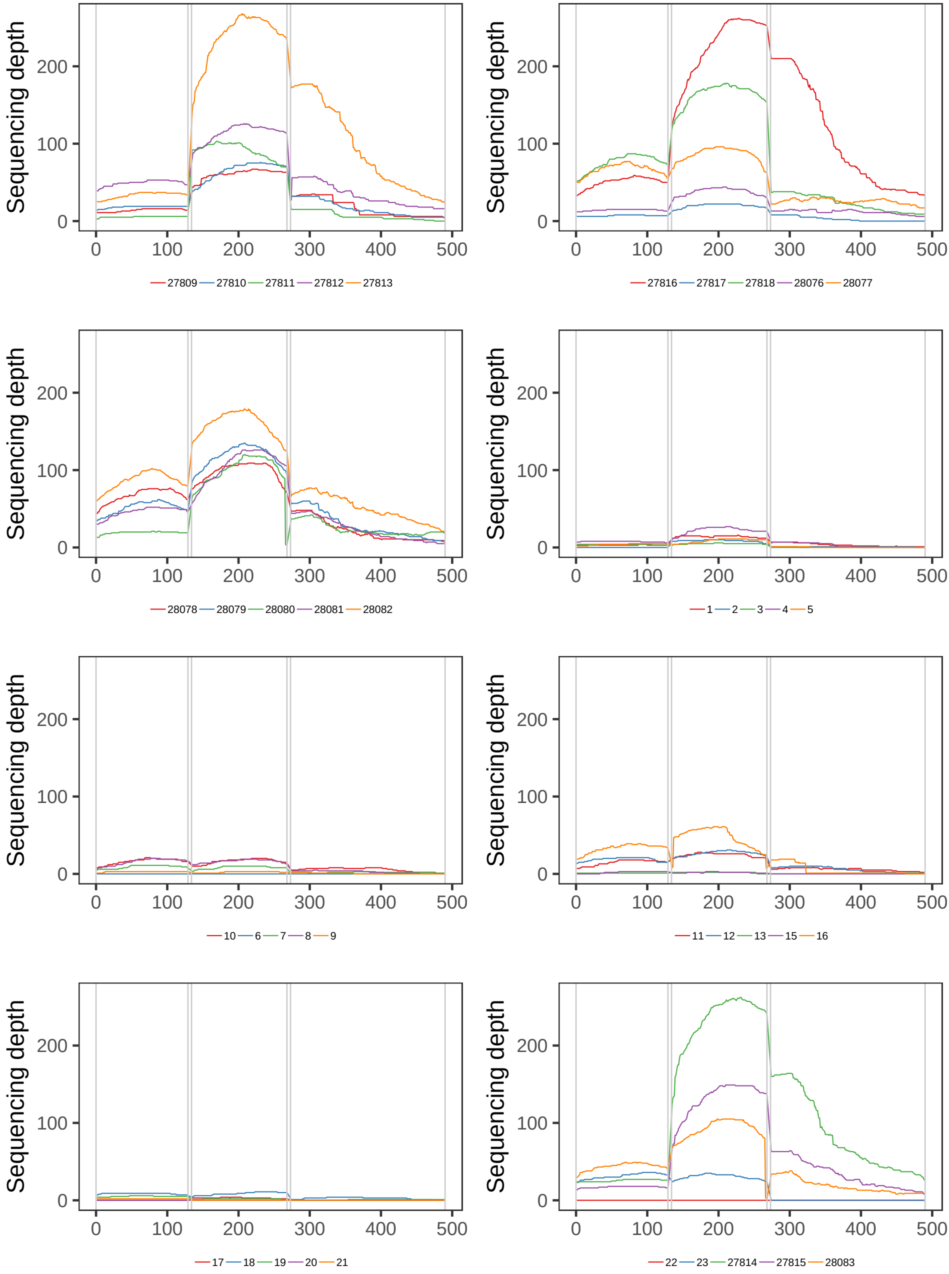
Sequencing depth
0
100
200
300
400
500
27809
27810
27811
27812
27813
27816
27817
27818
28076
28077
28078
28079
28080
28081
28082
1
2
3
4
5
10
6
7
8
9
11
12
13
15
16
17
18
19
20
21
22
23
27814
27815
28083

EOG54J111

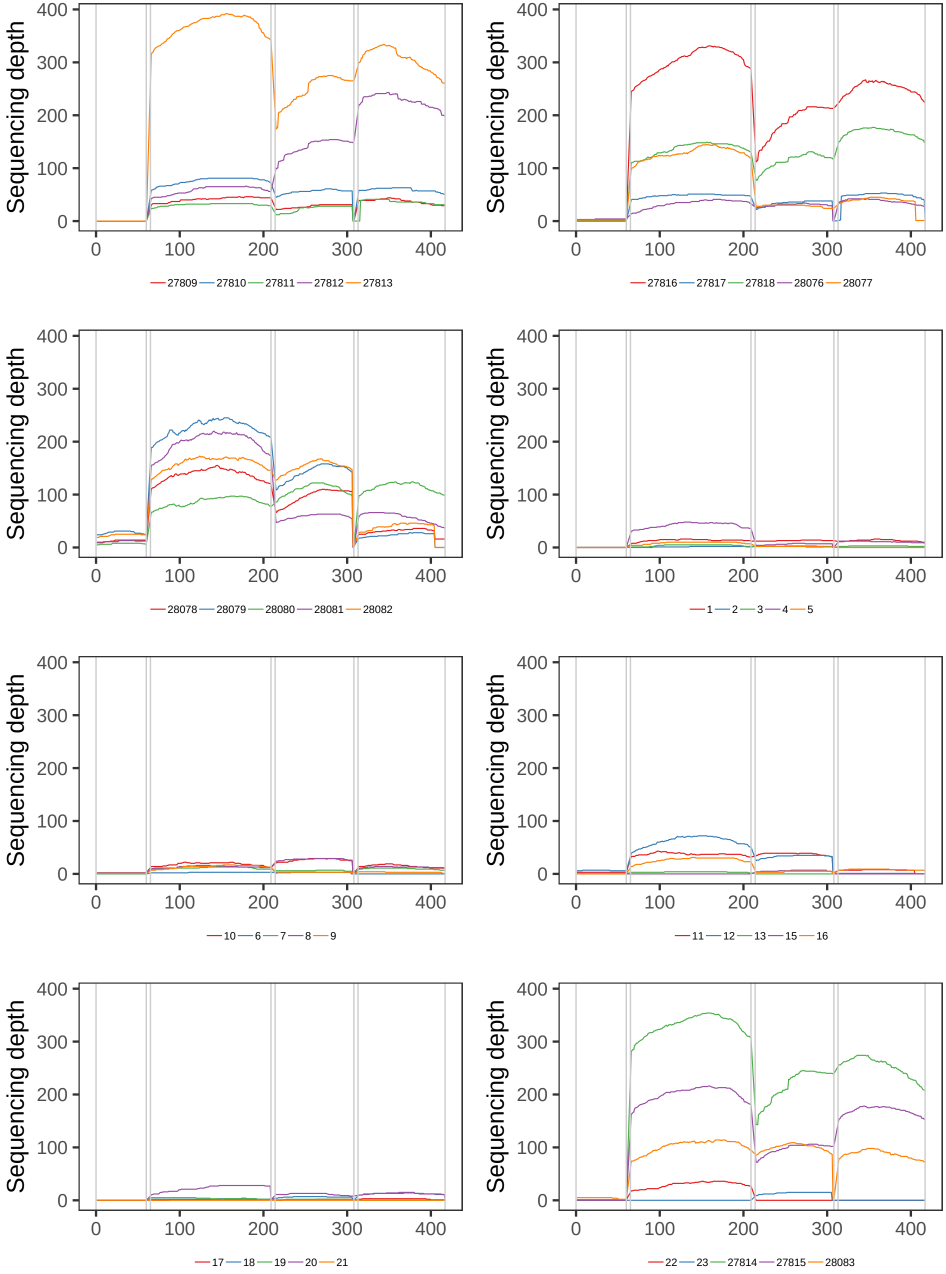

Sequencing depth
0
100
200
300
400
27809 27810 27811 27812 27813
27816 27817 27818 28076 28077
28078 28079 28080 28081 28082
1 2 3 4 5
10 6 7 8 9
11 12 13 15 16
17 18 19 20 21
22 23 27814 27815 28083

EOG579CQ2

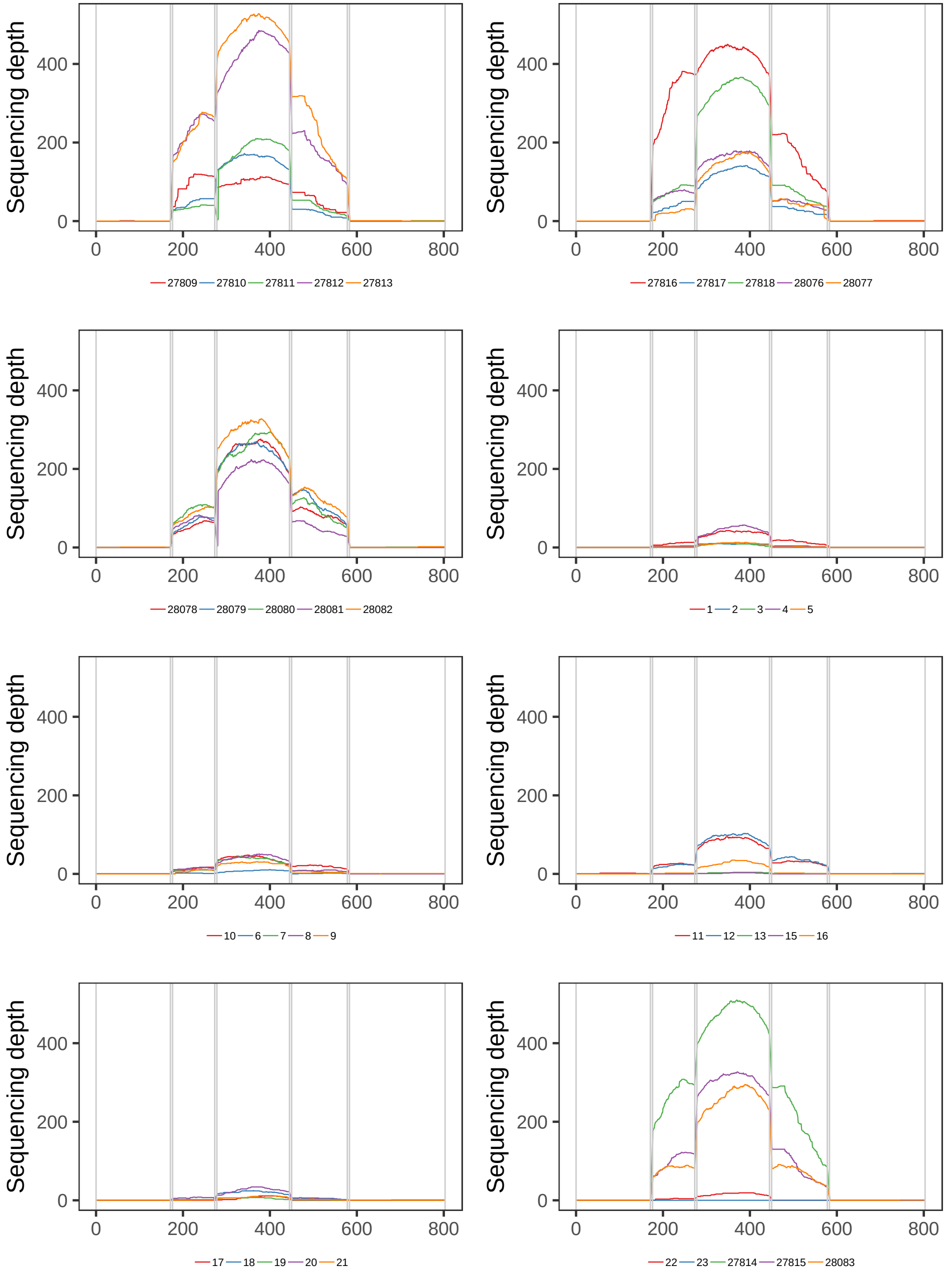

Sequencing depth
0
200
400
600
800
27809 27810 27811 27812 27813
27816 27817 27818 28076 28077
28078 28079 28080 28081 28082
1 2 3 4 5
10 6 7 8 9
11 12 13 15 16
17 18 19 20 21
22 23 27814 27815 28083

EOG59CNPW

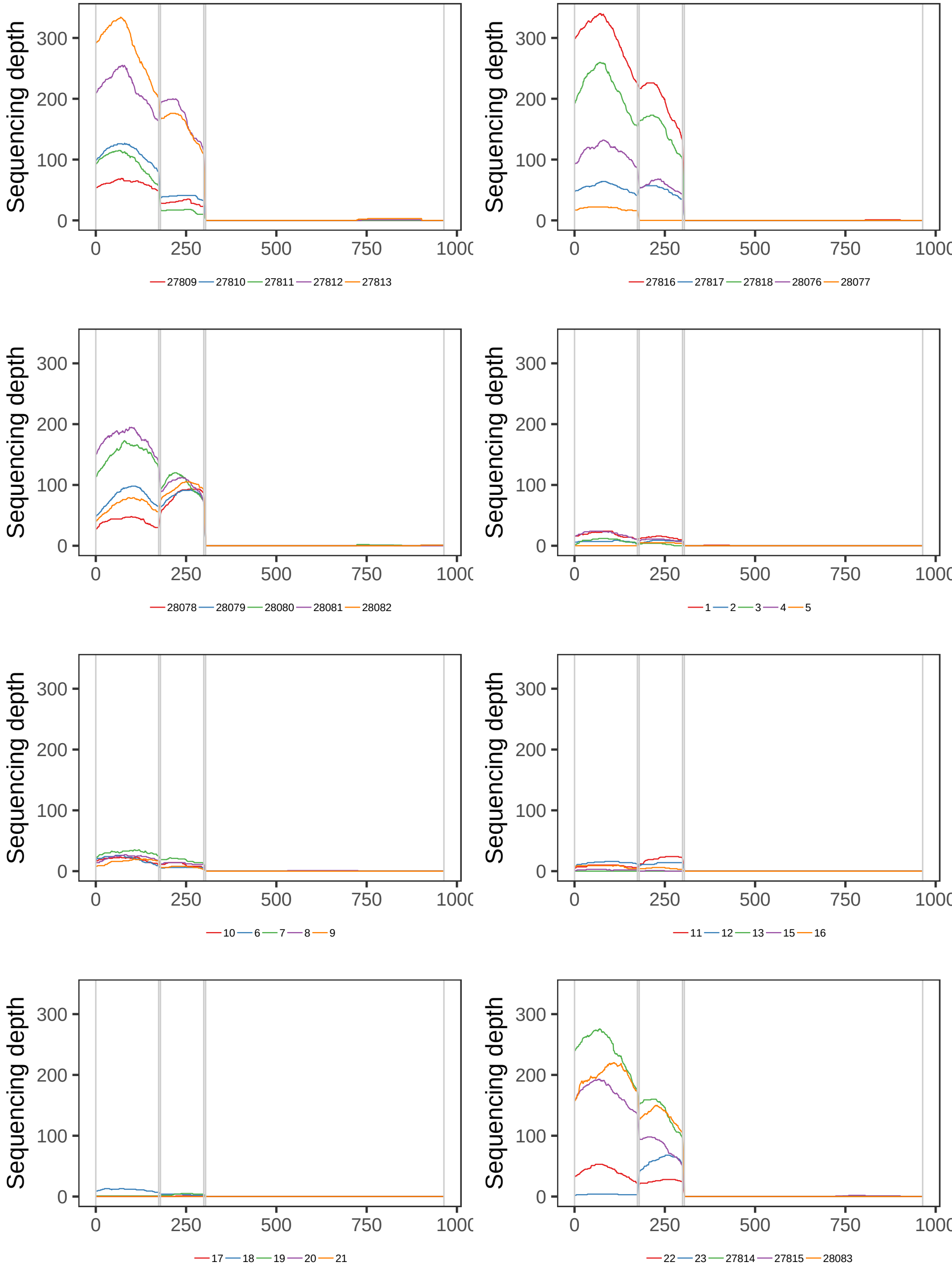
Sequencing depth
0
100
200
300
0
250
500
750
1000
27809 27810 27811 27812 27813
27816 27817 27818 28076 28077
28078 28079 28080 28081 28082
1 2 3 4 5
10 6 7 8 9
11 12 13 15 16
17 18 19 20 21
22 23 27814 27815 28083

EOG5DV43H

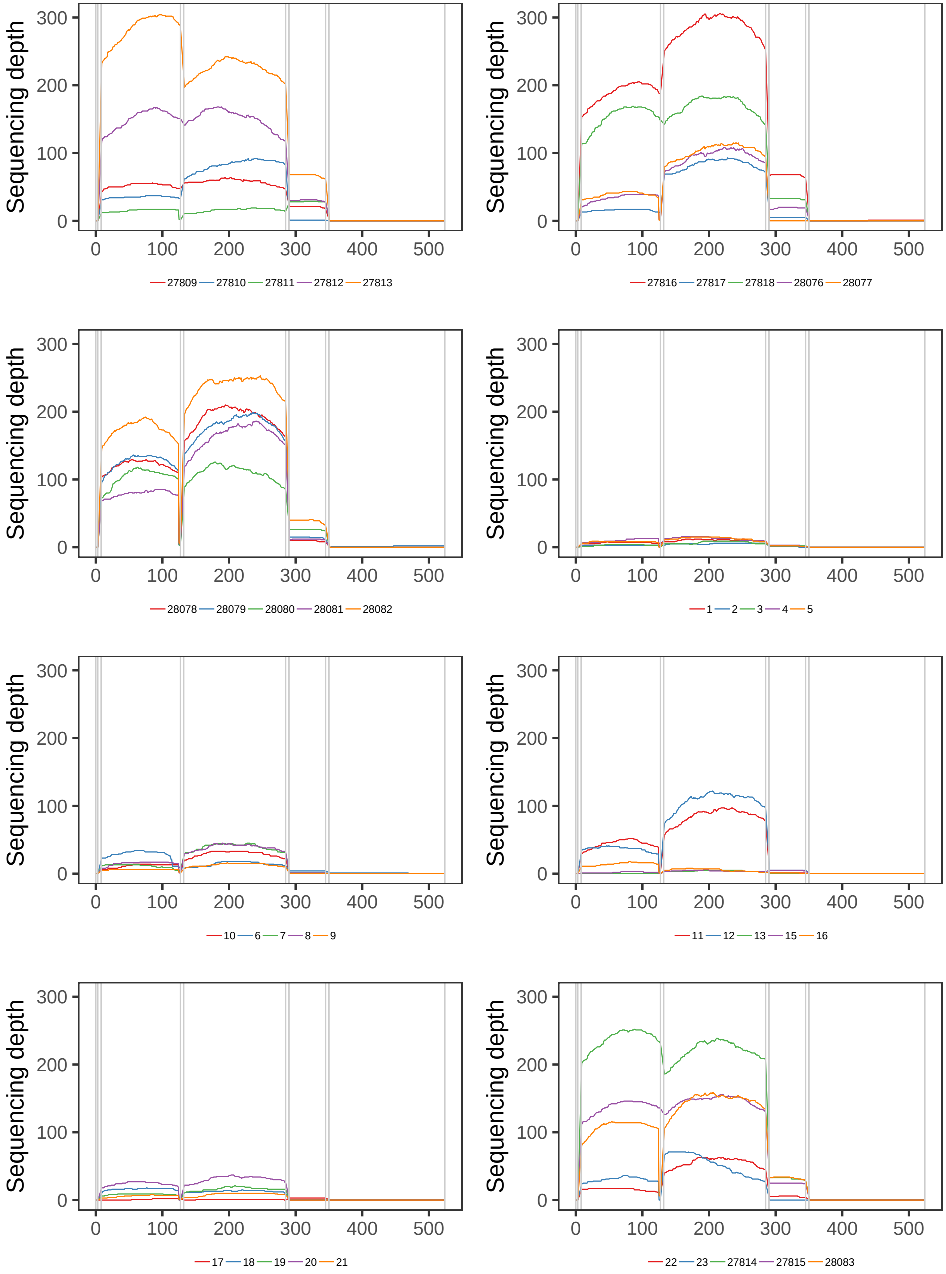
Sequencing depth
0
100
200
300
400
500
27809 27810 27811 27812 27813
27816 27817 27818 28076 28077
28078 28079 28080 28081 28082
1 2 3 4 5
10 6 7 8 9
11 12 13 15 16
17 18 19 20 21
22 23 27814 27815 28083

EOG5FXPQX

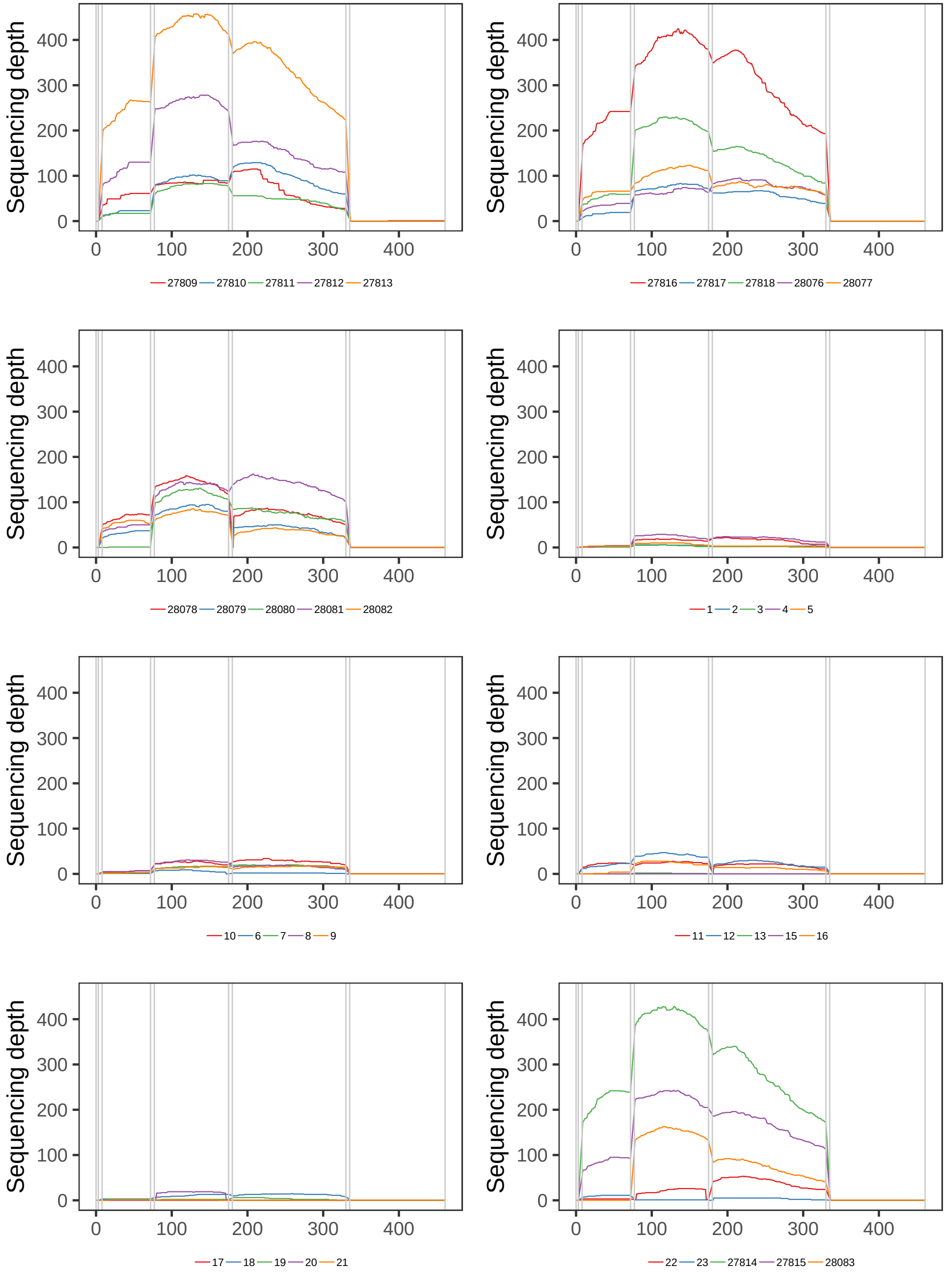

Sequencing depth
0
100
200
300
400
27809
27810
27811
27812
27813
27816
27817
27818
28076
28077
28078
28079
28080
28081
28082
1
2
3
4
5
10
6
7
8
9
11
12
13
15
16
17
18
19
20
21
22
23
27814
27815
28083

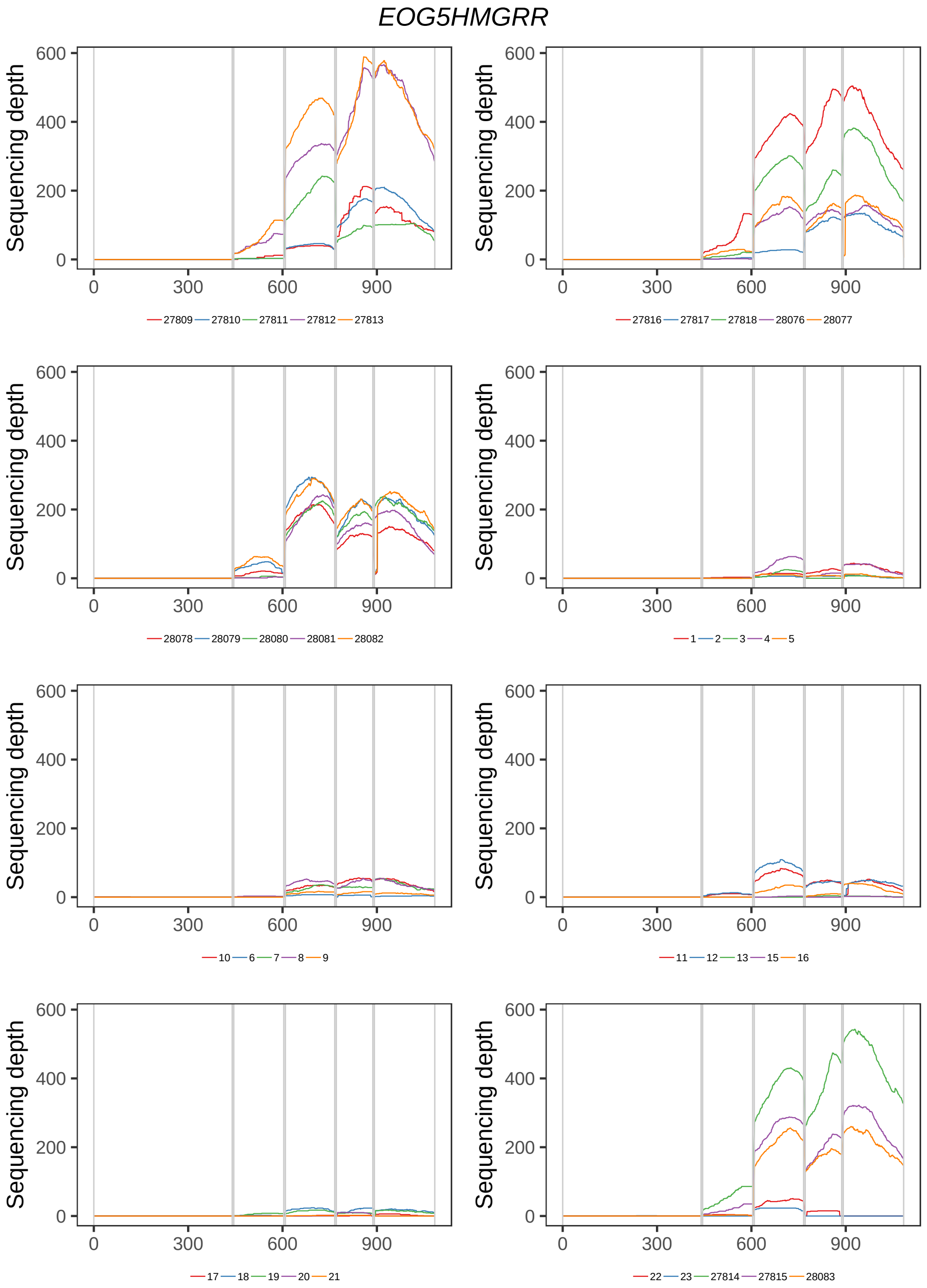
EOG5HMGRR
Sequencing depth
600
400
200
0
0
300
600
900
27809
27810
27811
27812
27813
27816
27817
27818
28076
28077
28078
28079
28080
28081
28082
1
2
3
4
5
10
6
7
8
9
11
12
13
15
16
17
18
19
20
21
22
23
27814
27815
28083

EOG5J6Q6W

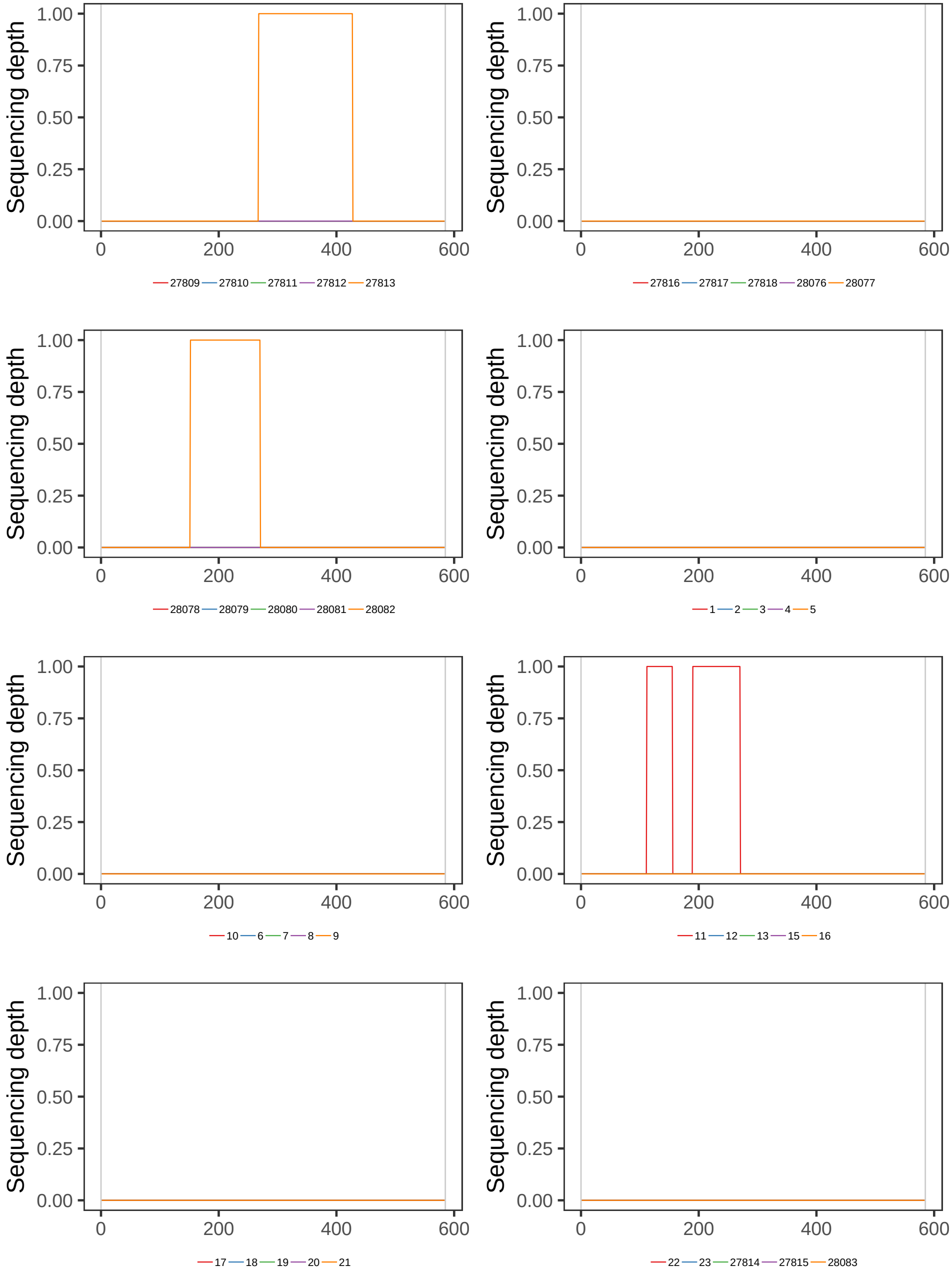

Sequencing depth
1.00
0.75
0.50
0.25
0.00
0
200
400
600
27809 27810 27811 27812 27813
27816 27817 27818 28076 28077
28078 28079 28080 28081 28082
1 2 3 4 5
10 6 7 8 9
11 12 13 15 16
17 18 19 20 21
22 23 27814 27815 28083

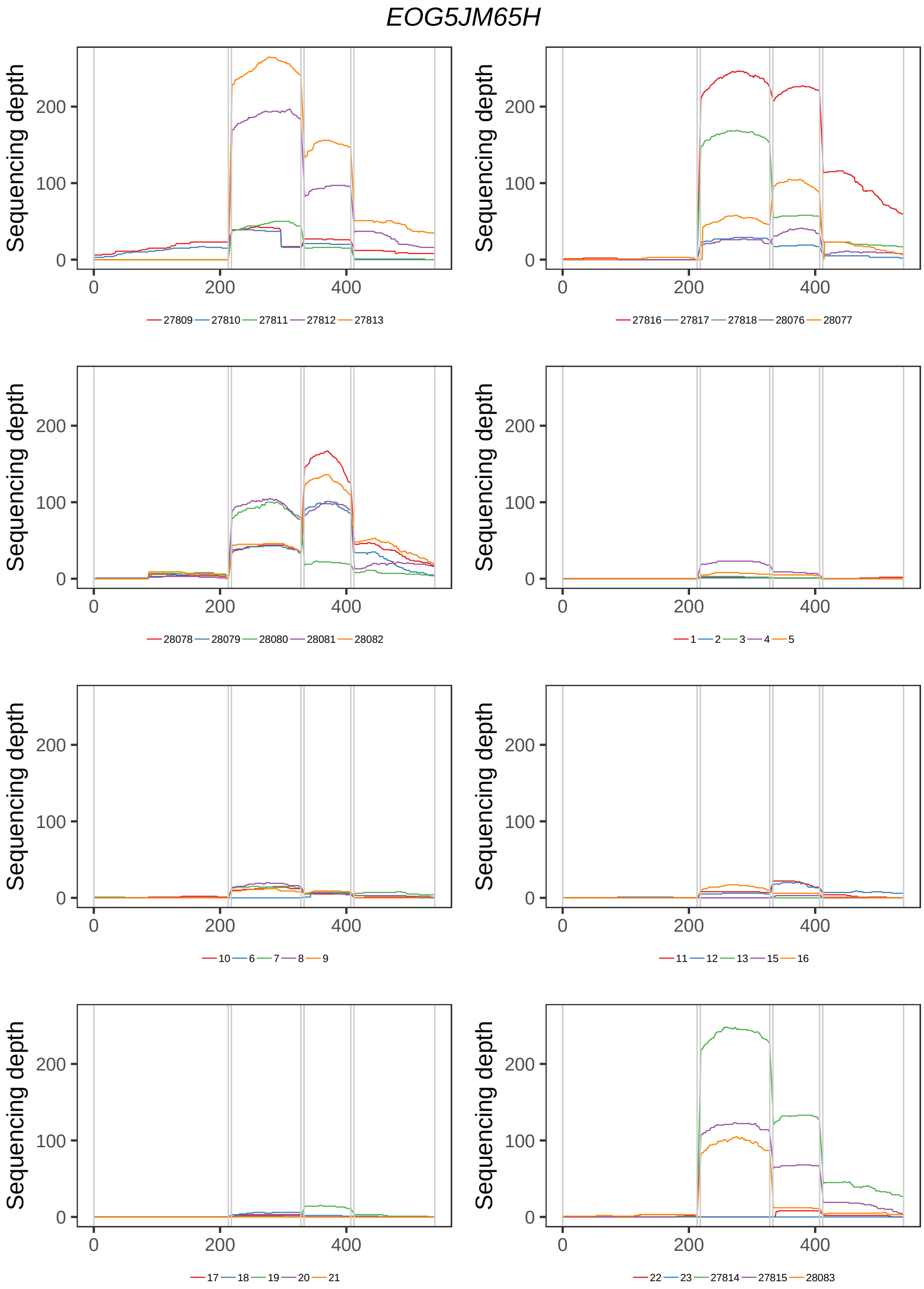
EOG5JM65H
Sequencing depth
0
100
200
0
200
400
27809 27810 27811 27812 27813
27816 27817 27818 28076 28077
28078 28079 28080 28081 28082
1 2 3 4 5
10 6 7 8 9
11 12 13 15 16
17 18 19 20 21
22 23 27814 27815 28083

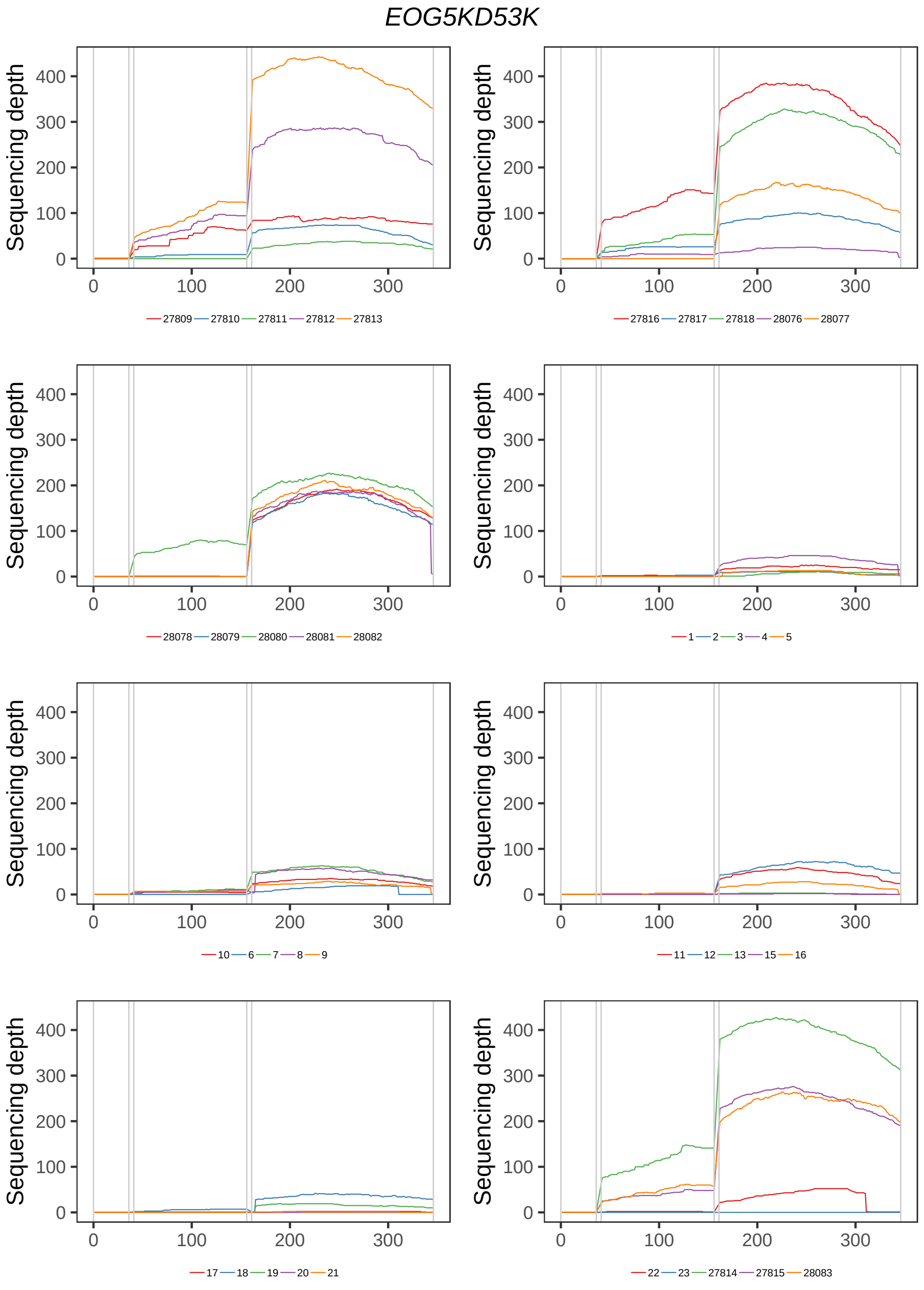
EOG5KD53K
Sequencing depth
0
100
200
300
400
27809
27810
27811
27812
27813
27816
27817
27818
28076
28077
28078
28079
28080
28081
28082
1
2
3
4
5
10
6
7
8
9
11
12
13
15
16
17
18
19
20
21
22
23
27814
27815
28083

EOG5M907S

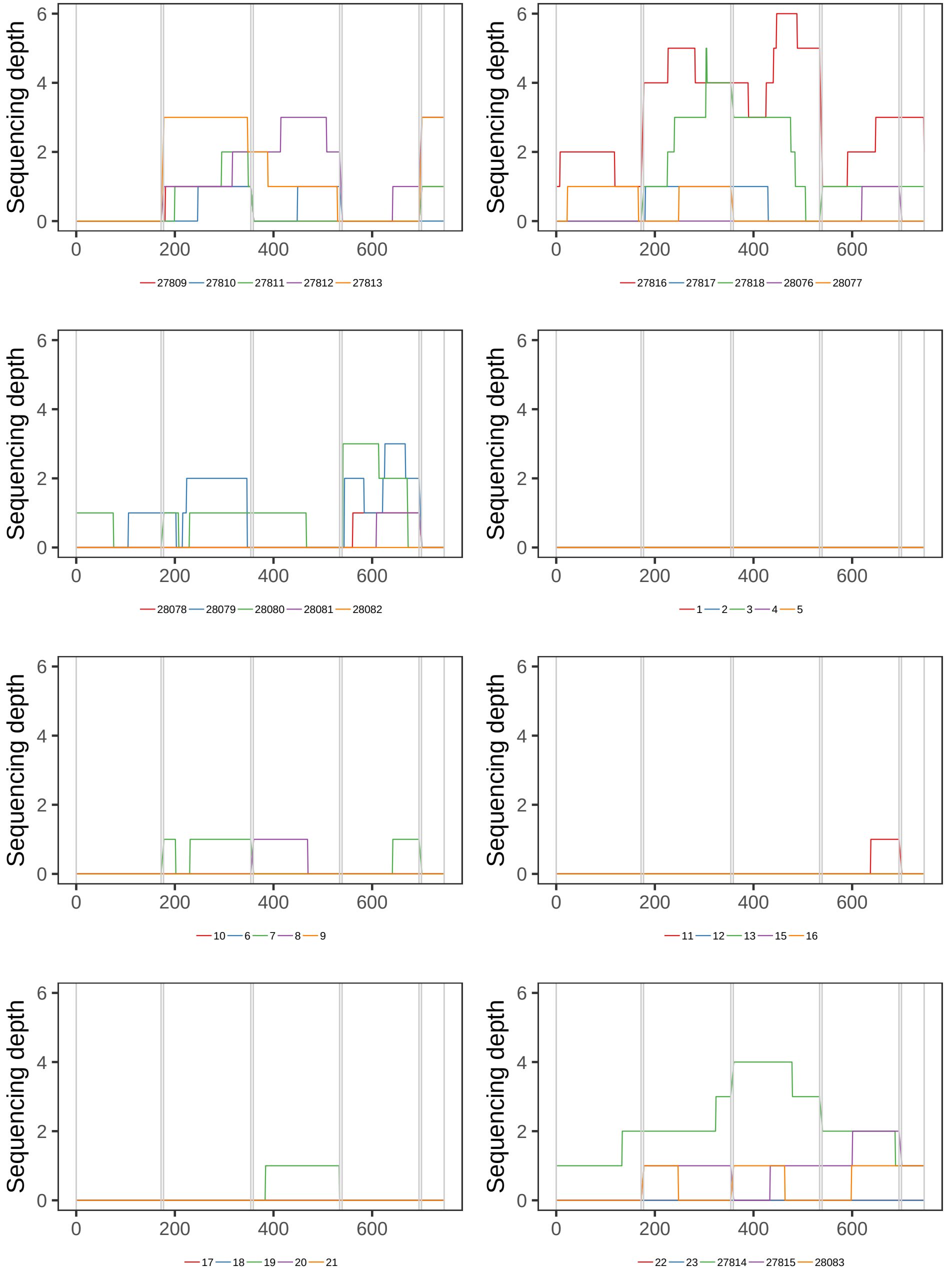
Sequencing depth
27809 27810 27811 27812 27813
27816 27817 27818 28076 28077
28078 28079 28080 28081 28082
1 2 3 4 5
10 6 7 8 9
11 12 13 15 16
17 18 19 20 21
22 23 27814 27815 28083

EOG5N02WJ

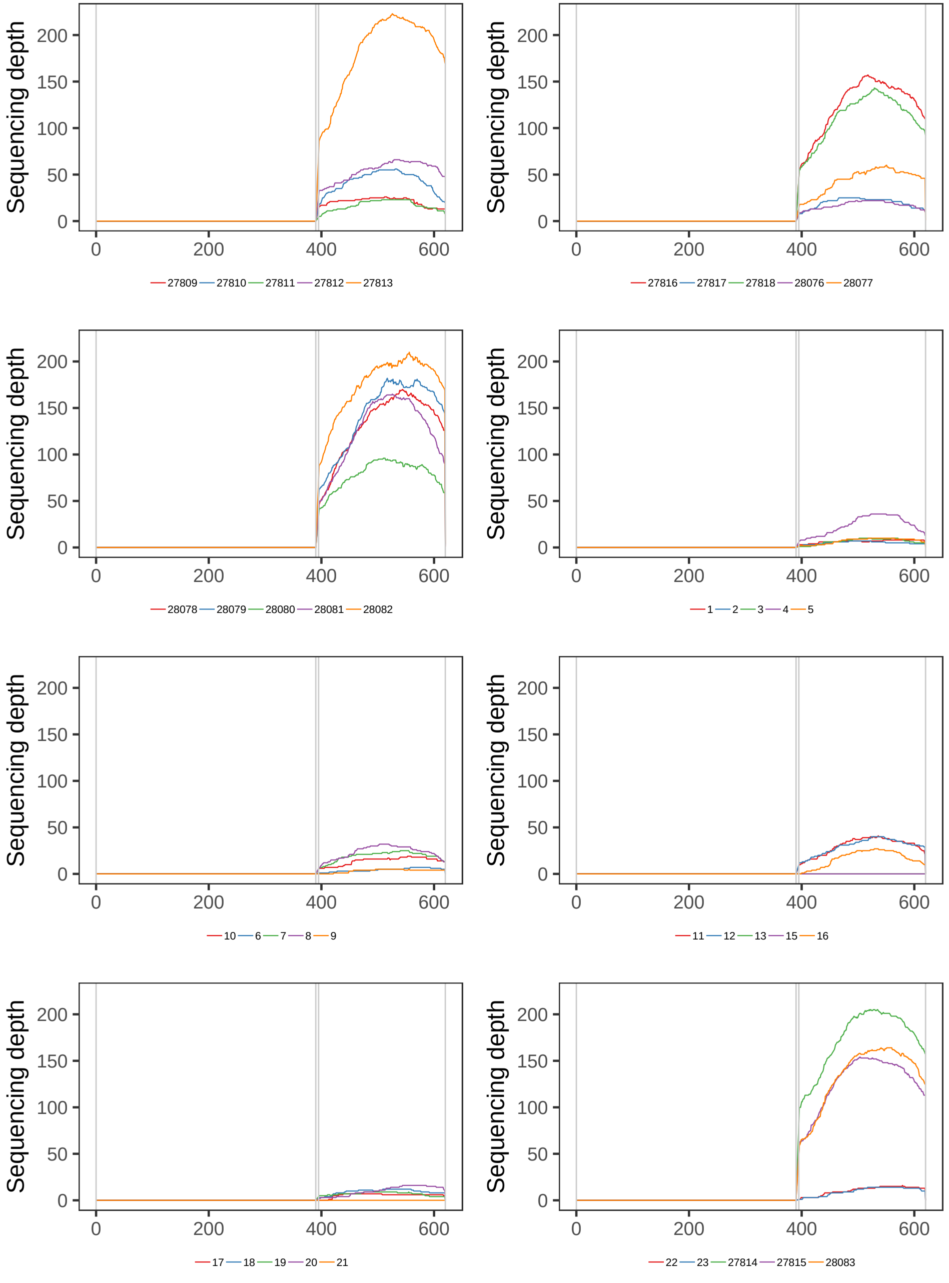
Sequencing depth
0
50
100
150
200
0
200
400
600
27809 27810 27811 27812 27813
27816 27817 27818 28076 28077
28078 28079 28080 28081 28082
1 2 3 4 5
10 6 7 8 9
11 12 13 15 16
17 18 19 20 21
22 23 27814 27815 28083

EOG5QV9V2

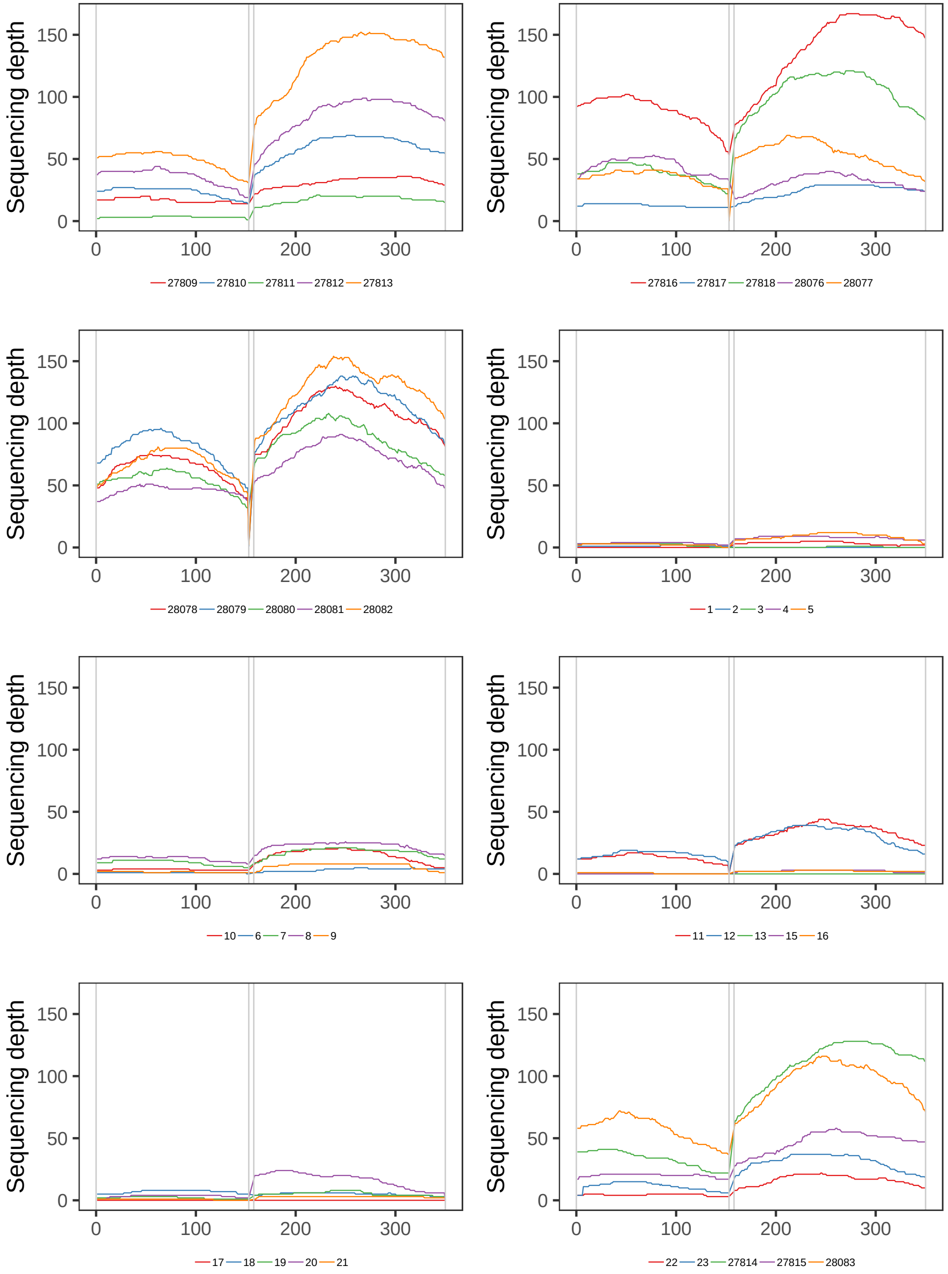
Sequencing depth
0
50
100
150
0
100
200
300
27809 27810 27811 27812 27813
27816 27817 27818 28076 28077
28078 28079 28080 28081 28082
1 2 3 4 5
10 6 7 8 9
11 12 13 15 16
17 18 19 20 21
22 23 27814 27815 28083

EOG5QZ633

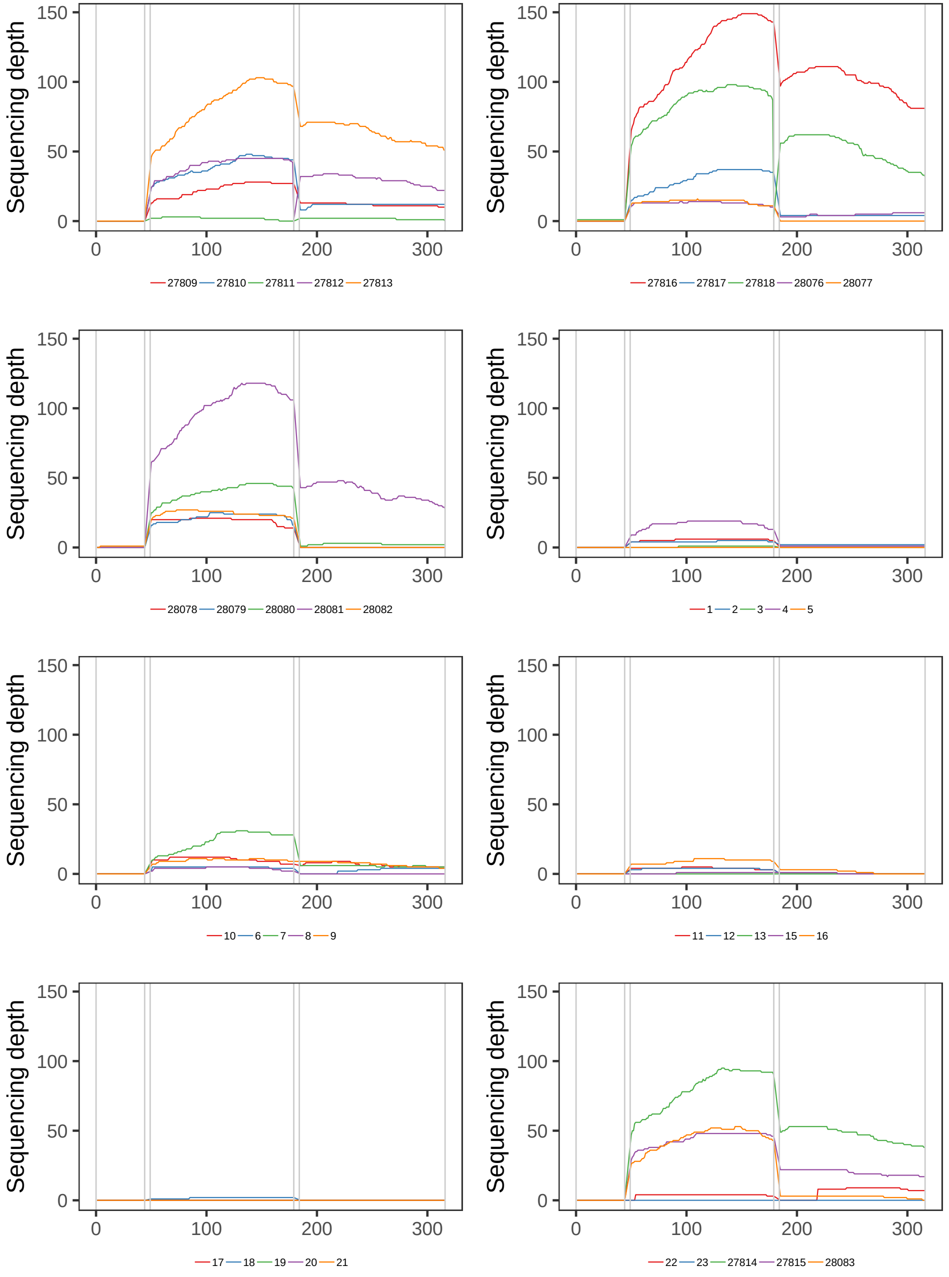

Sequencing depth
150
100
50
0
0
100
200
300
27809 27810 27811 27812 27813
27816 27817 27818 28076 28077
28078 28079 28080 28081 28082
1 2 3 4 5
10 6 7 8 9
11 12 13 15 16
17 18 19 20 21
22 23 27814 27815 28083

EOG5T4BBS

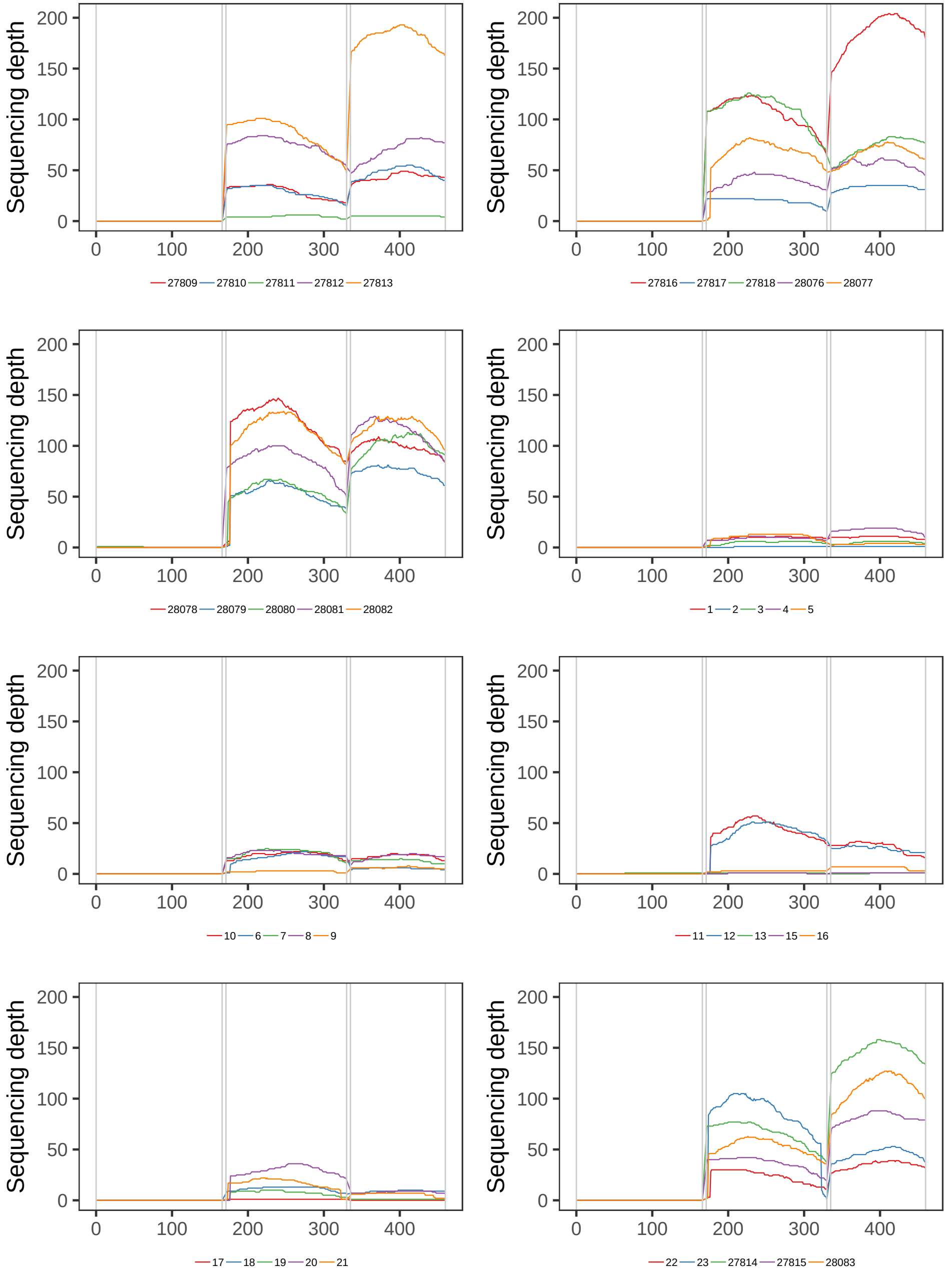
Sequencing depth
0
50
100
150
200
0
100
200
300
400
27809 27810 27811 27812 27813
27816 27817 27818 28076 28077
28078 28079 28080 28081 28082
1 2 3 4 5
10 6 7 8 9
11 12 13 15 16
17 18 19 20 21
22 23 27814 27815 28083

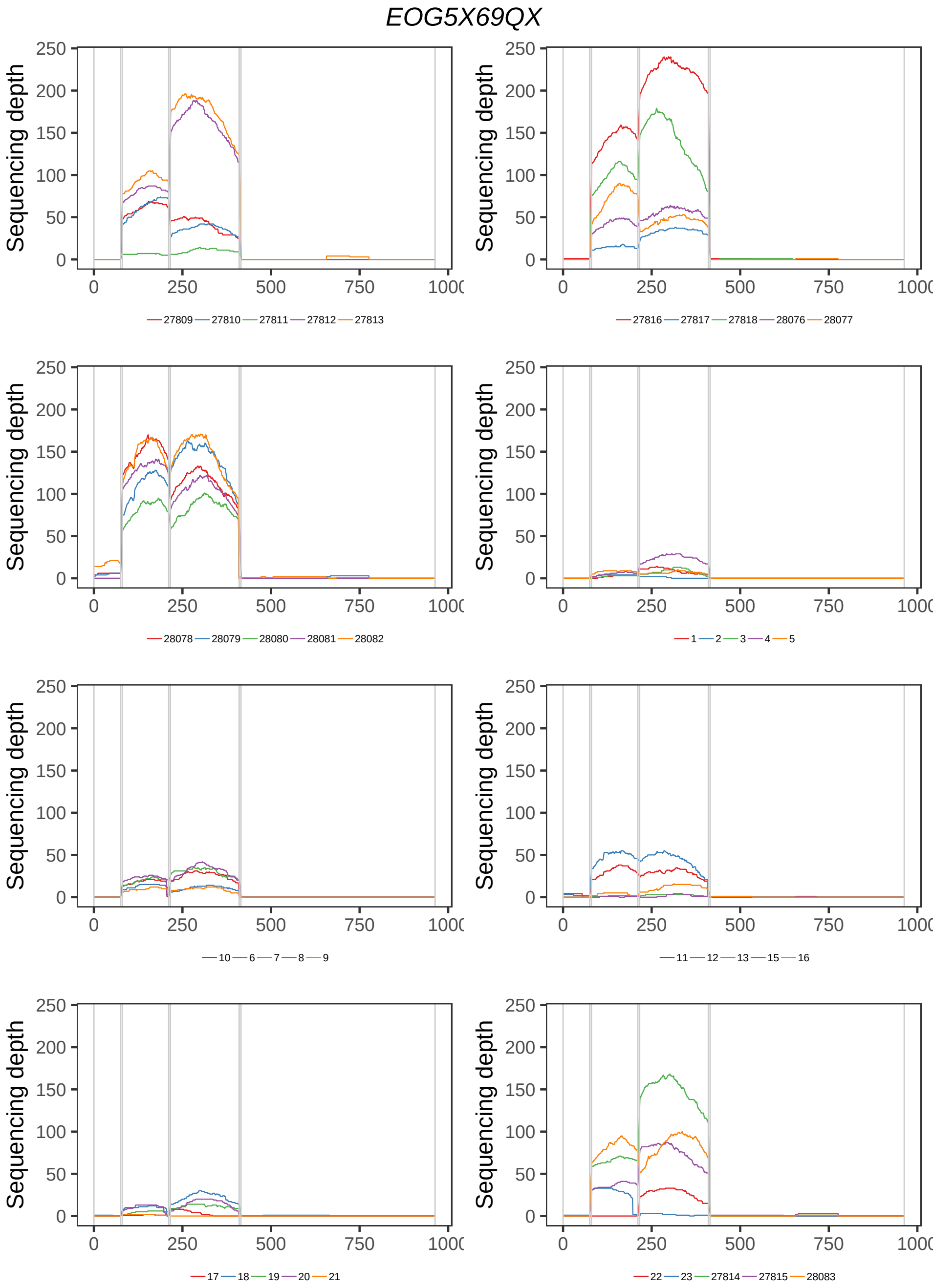

EOG5X69QX
Sequencing depth
27809 27810 27811 27812 27813
27816 27817 27818 28076 28077
28078 28079 28080 28081 28082
1 2 3 4 5
10 6 7 8 9
11 12 13 15 16
17 18 19 20 21
22 23 27814 27815 28083

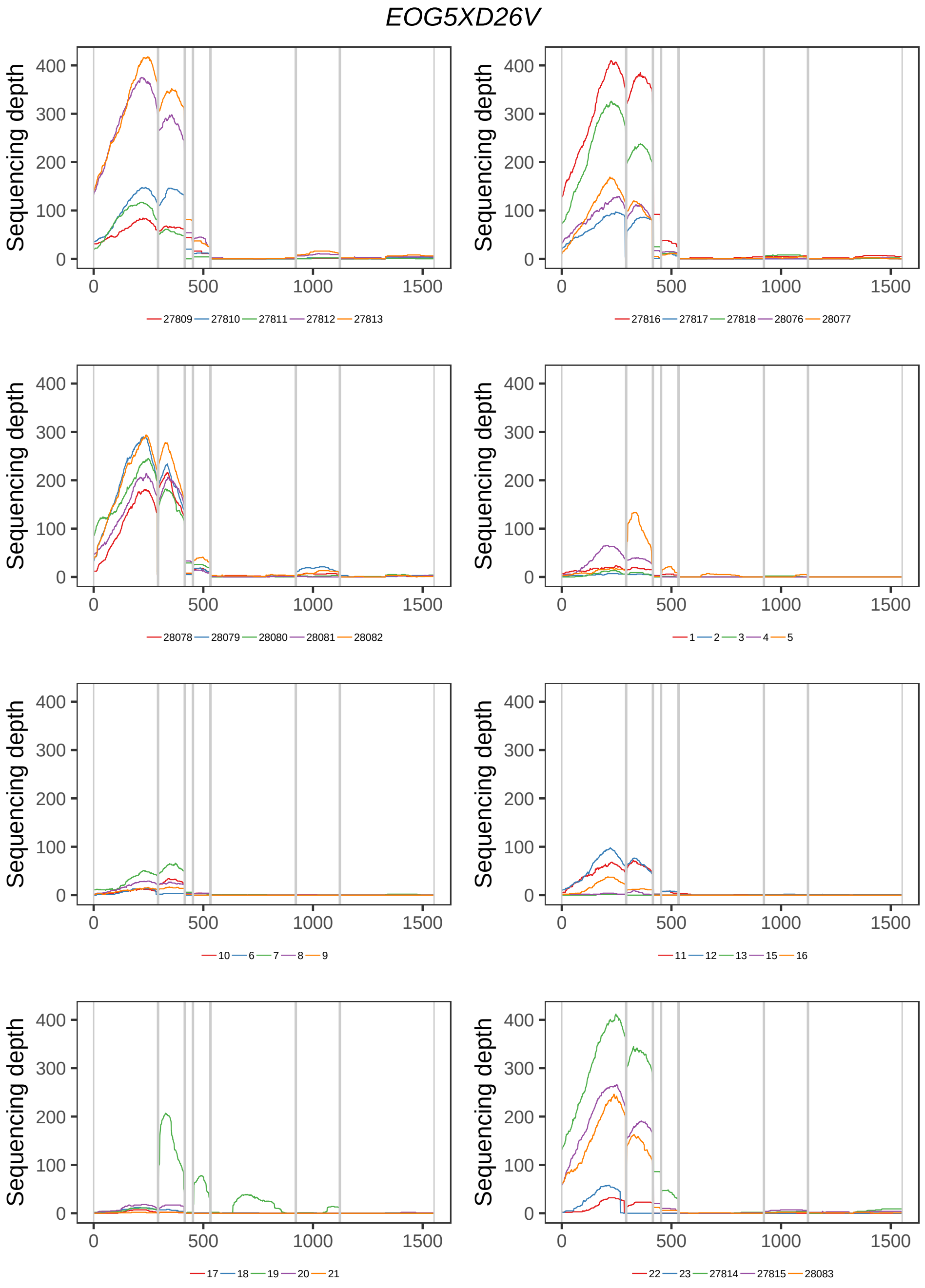

EOG5XD26V
Sequencing depth
0
100
200
300
400
500
1000
1500
27809 27810 27811 27812 27813
27816 27817 27818 28076 28077
28078 28079 28080 28081 28082
1 2 3 4 5
10 6 7 8 9
11 12 13 15 16
17 18 19 20 21
22 23 27814 27815 28083

EOG51G1M8

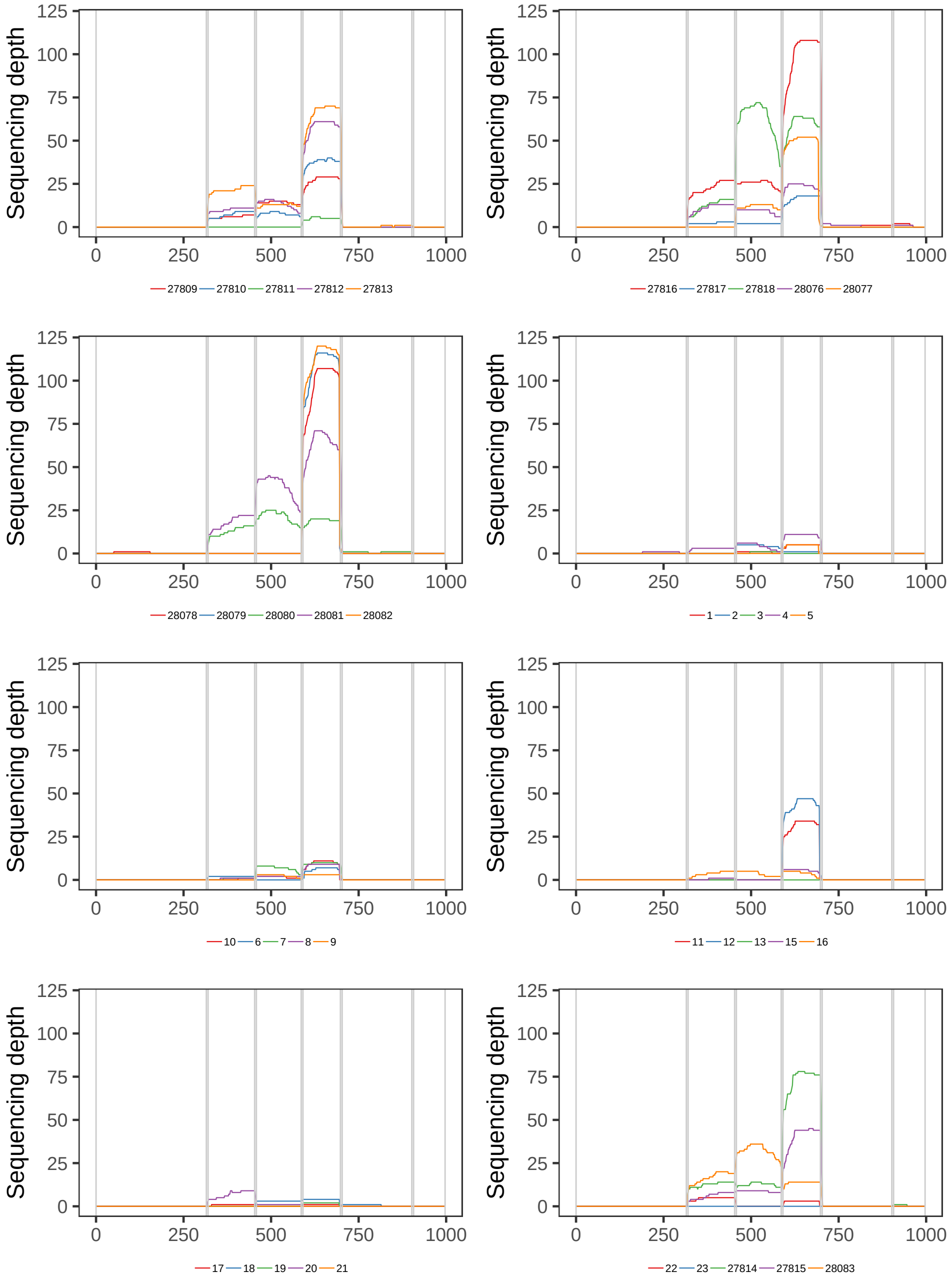

Sequencing depth
27809 27810 27811 27812 27813
27816 27817 27818 28076 28077
28078 28079 28080 28081 28082
1 2 3 4 5
10 6 7 8 9
11 12 13 15 16
17 18 19 20 21
22 23 27814 27815 28083

EOG51JWTT

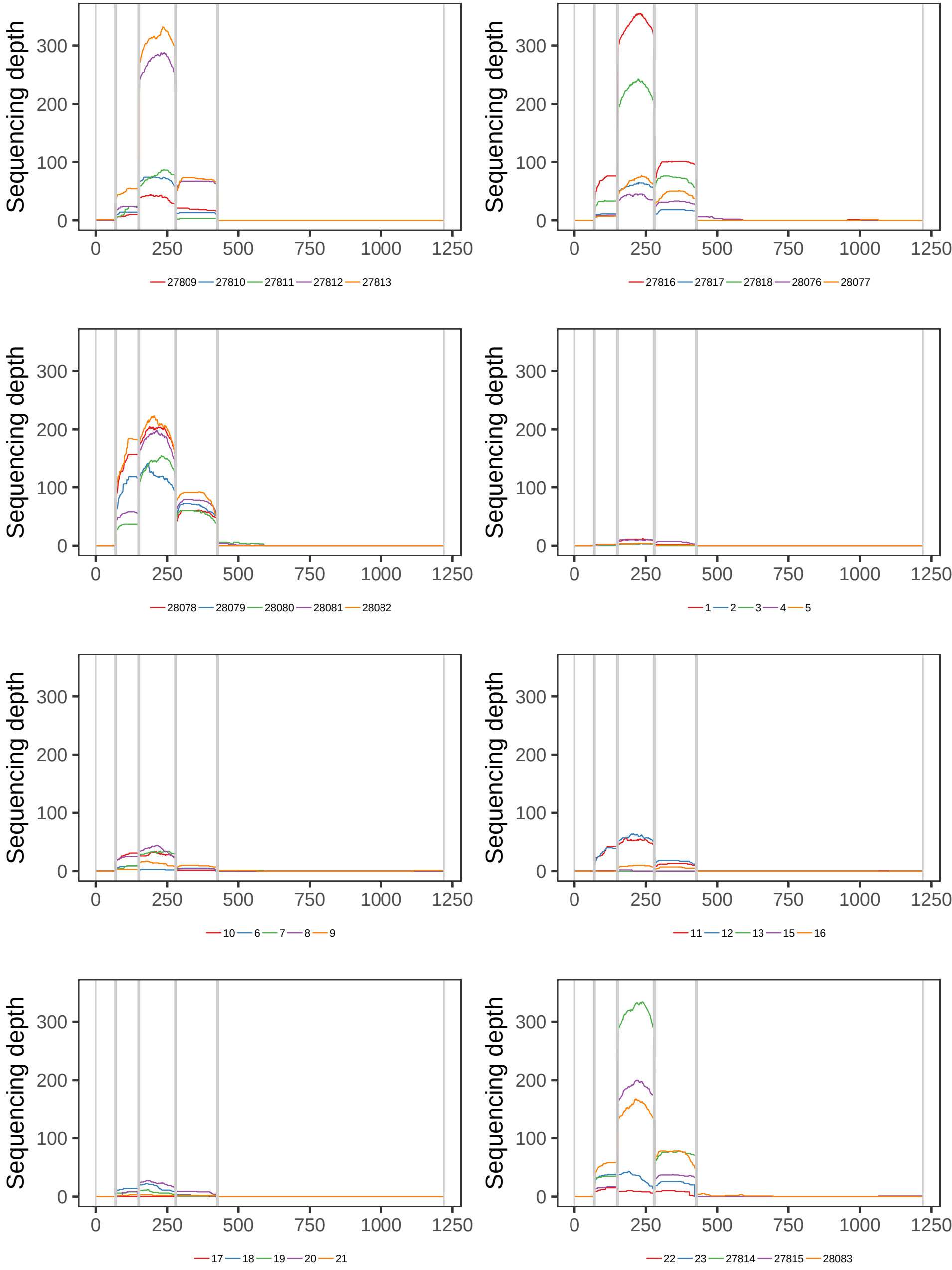

Sequencing depth
0
100
200
300
0
250
500
750
1000
1250
27809 27810 27811 27812 27813
27816 27817 27818 28076 28077
28078 28079 28080 28081 28082
1 2 3 4 5
10 6 7 8 9
11 12 13 15 16
17 18 19 20 21
22 23 27814 27815 28083

EOG55DV50

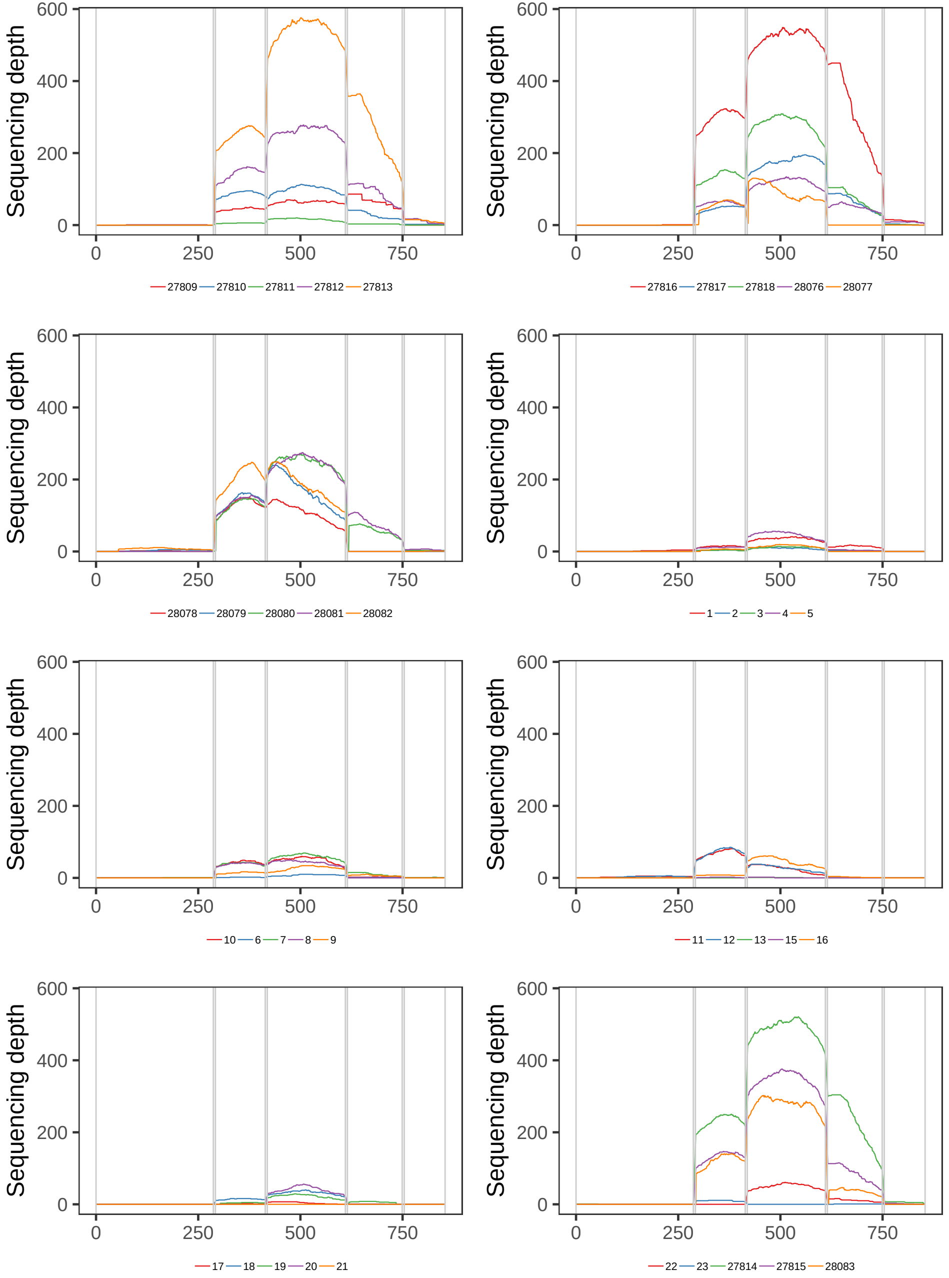
Sequencing depth
600
400
200
0
0
250
500
750
27809
27810
27811
27812
27813
27816
27817
27818
28076
28077
28078
28079
28080
28081
28082
1
2
3
4
5
10
6
7
8
9
11
12
13
15
16
17
18
19
20
21
22
23
27814
27815
28083

EOG55MKMK

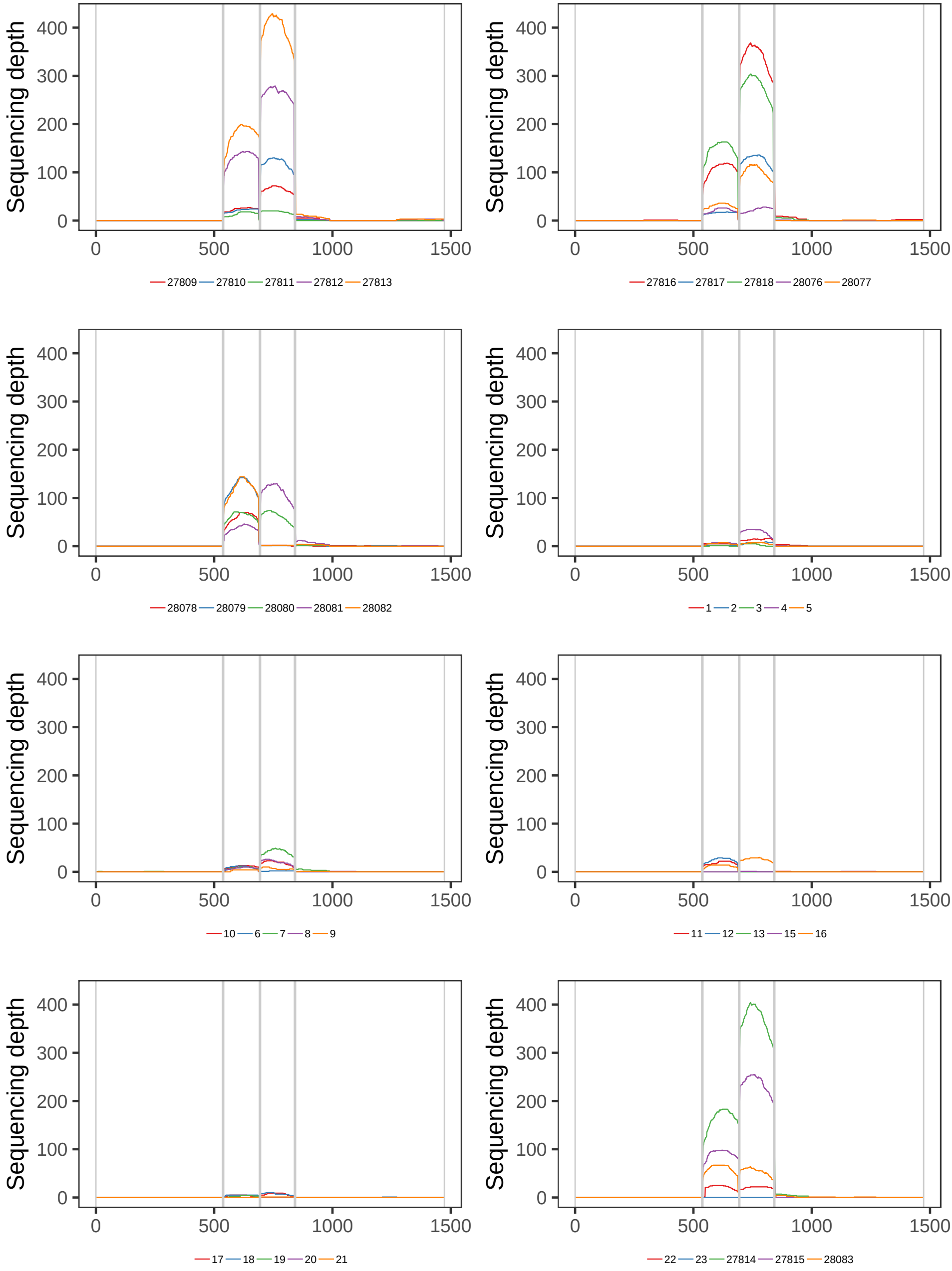

Sequencing depth
27809
27810
27811
27812
27813
27816
27817
27818
28076
28077
28078
28079
28080
28081
28082
1
2
3
4
5
10
6
7
8
9
11
12
13
15
16
17
18
19
20
21
22
23
27814
27815
28083

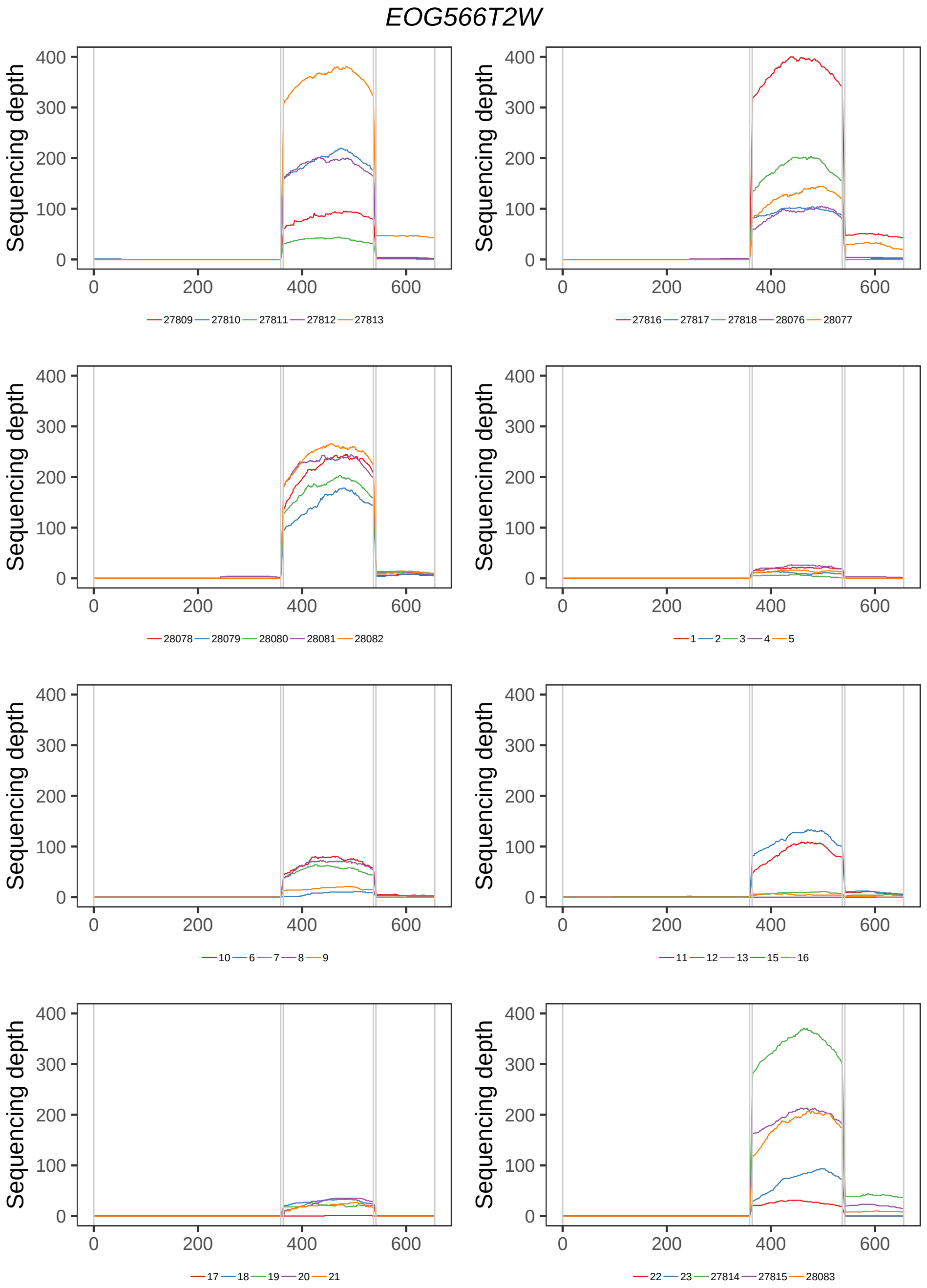
EOG566T2W
Sequencing depth
0
100
200
300
400
0
200
400
600
27809 27810 27811 27812 27813
27816 27817 27818 28076 28077
28078 28079 28080 28081 28082
1 2 3 4 5
10 6 7 8 9
11 12 13 15 16
17 18 19 20 21
22 23 27814 27815 28083

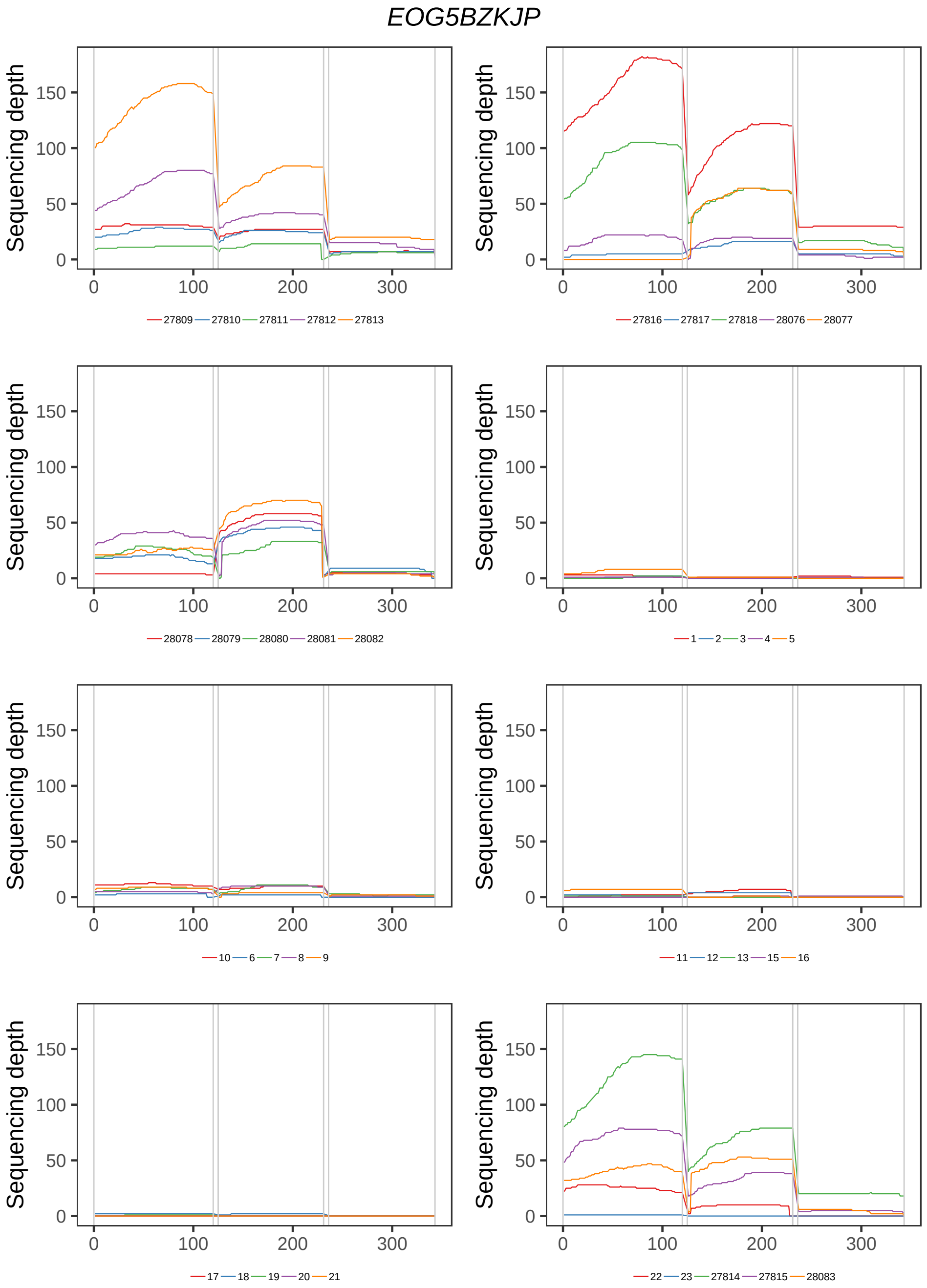
EOG5BZKJP
Sequencing depth
27809 27810 27811 27812 27813
27816 27817 27818 28076 28077
28078 28079 28080 28081 28082
1 2 3 4 5
10 6 7 8 9
11 12 13 15 16
17 18 19 20 21
22 23 27814 27815 28083

EOG5CJSZX

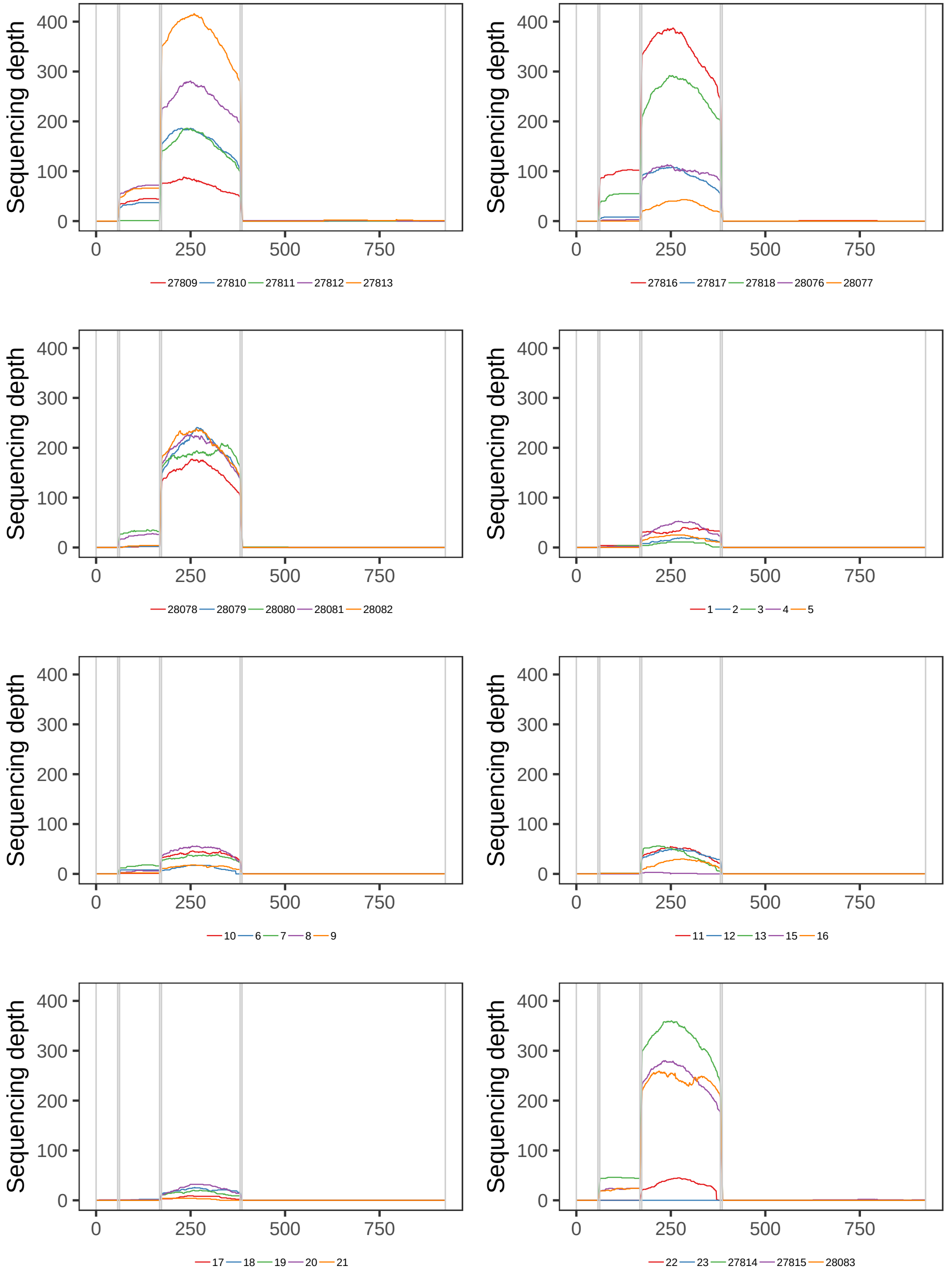
Sequencing depth
0
100
200
300
400
0
250
500
750
27809 27810 27811 27812 27813
27816 27817 27818 28076 28077
28078 28079 28080 28081 28082
1 2 3 4 5
10 6 7 8 9
11 12 13 15 16
17 18 19 20 21
22 23 27814 27815 28083

EOG5DNCM7

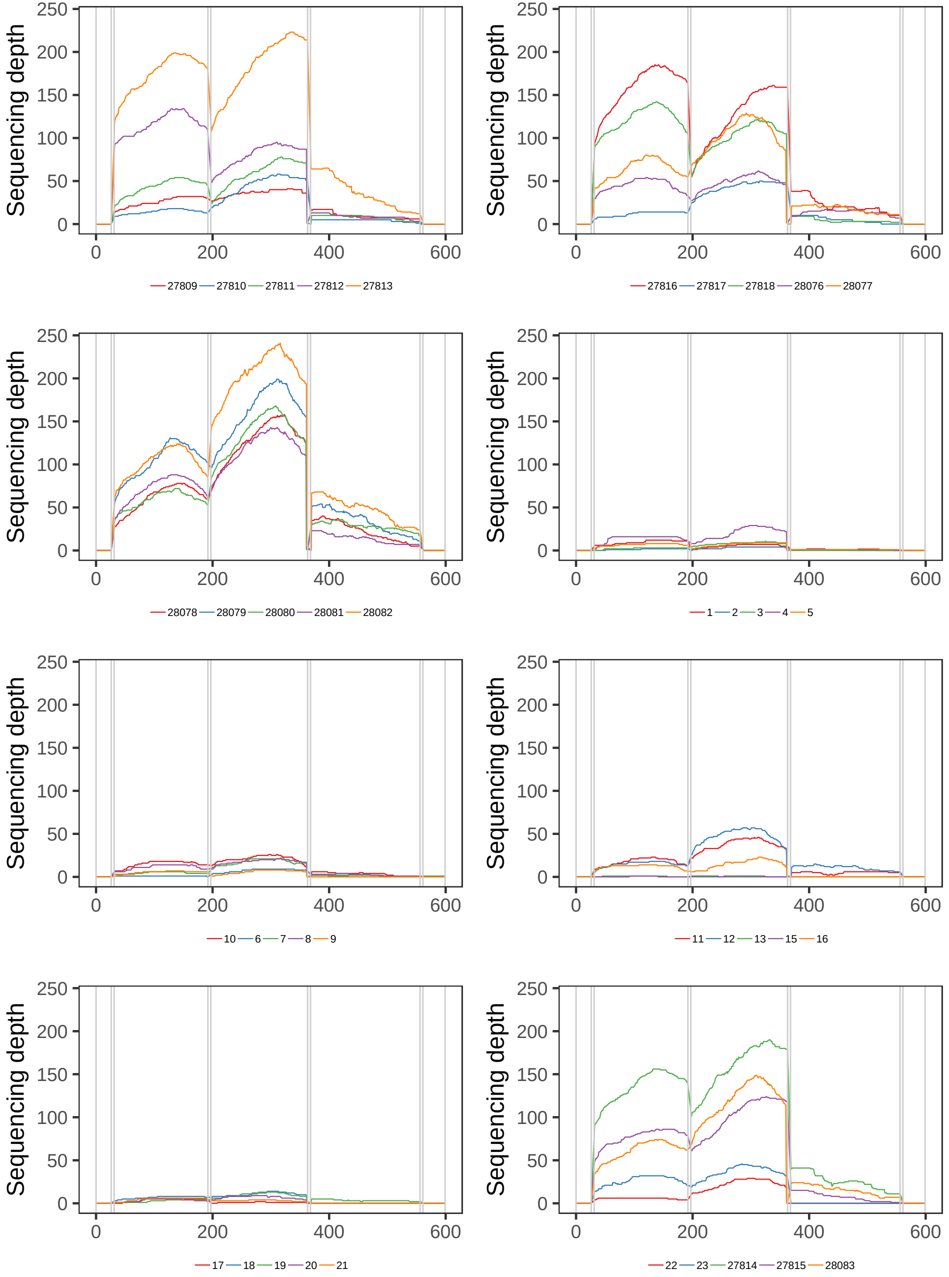
Sequencing depth
0
50
100
150
200
250
0
200
400
600
27809 27810 27811 27812 27813
27816 27817 27818 28076 28077
28078 28079 28080 28081 28082
1 2 3 4 5
10 6 7 8 9
11 12 13 15 16
17 18 19 20 21
22 23 27814 27815 28083

EOG5DZ09F

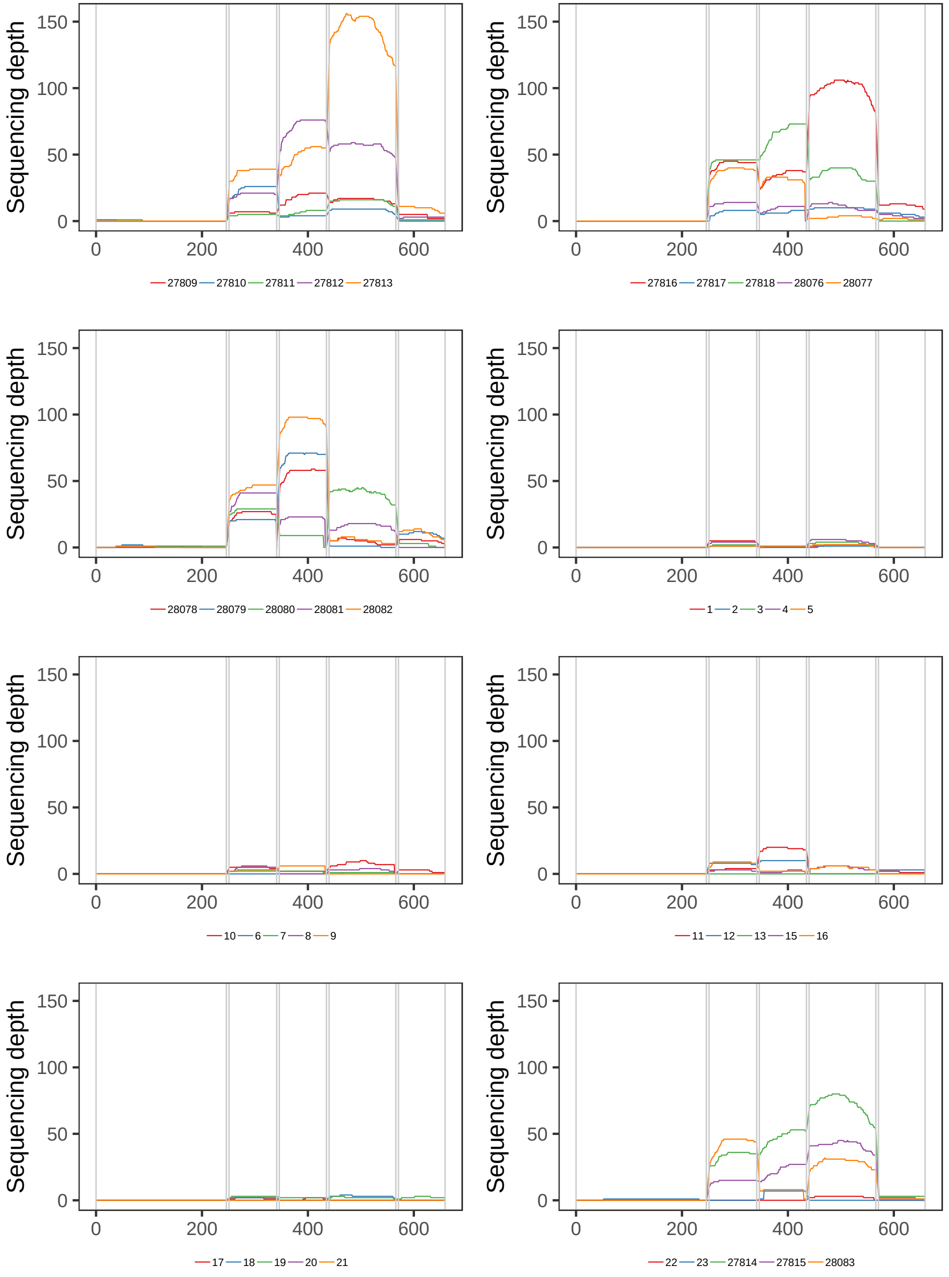
Sequencing depth
0
50
100
150
0
200
400
600
27809 27810 27811 27812 27813
27816 27817 27818 28076 28077
28078 28079 28080 28081 28082
1 2 3 4 5
10 6 7 8 9
11 12 13 15 16
17 18 19 20 21
22 23 27814 27815 28083

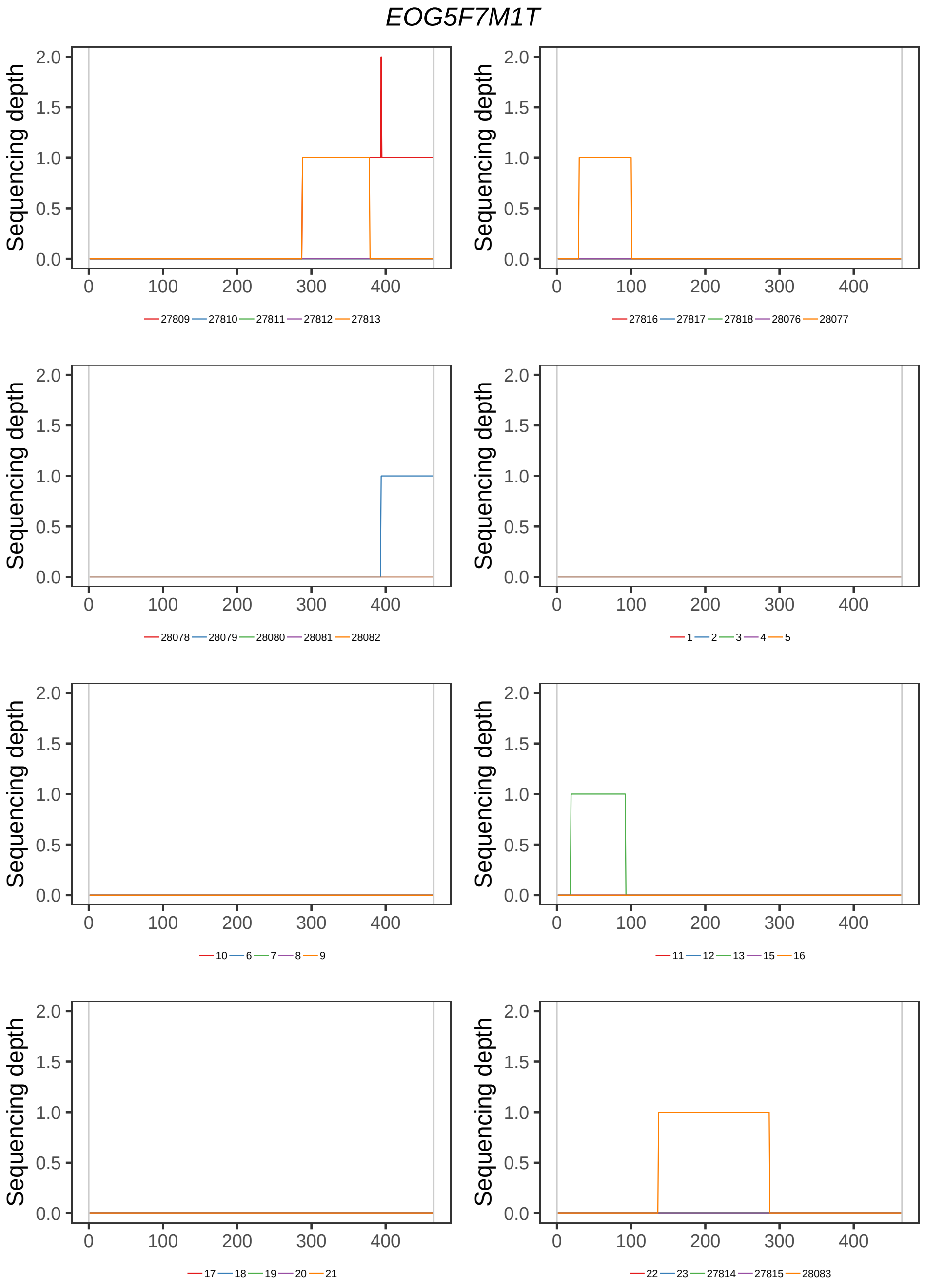

EOG5F7M1T
Sequencing depth
27809 27810 27811 27812 27813
27816 27817 27818 28076 28077
28078 28079 28080 28081 28082
1 2 3 4 5
10 6 7 8 9
11 12 13 15 16
17 18 19 20 21
22 23 27814 27815 28083

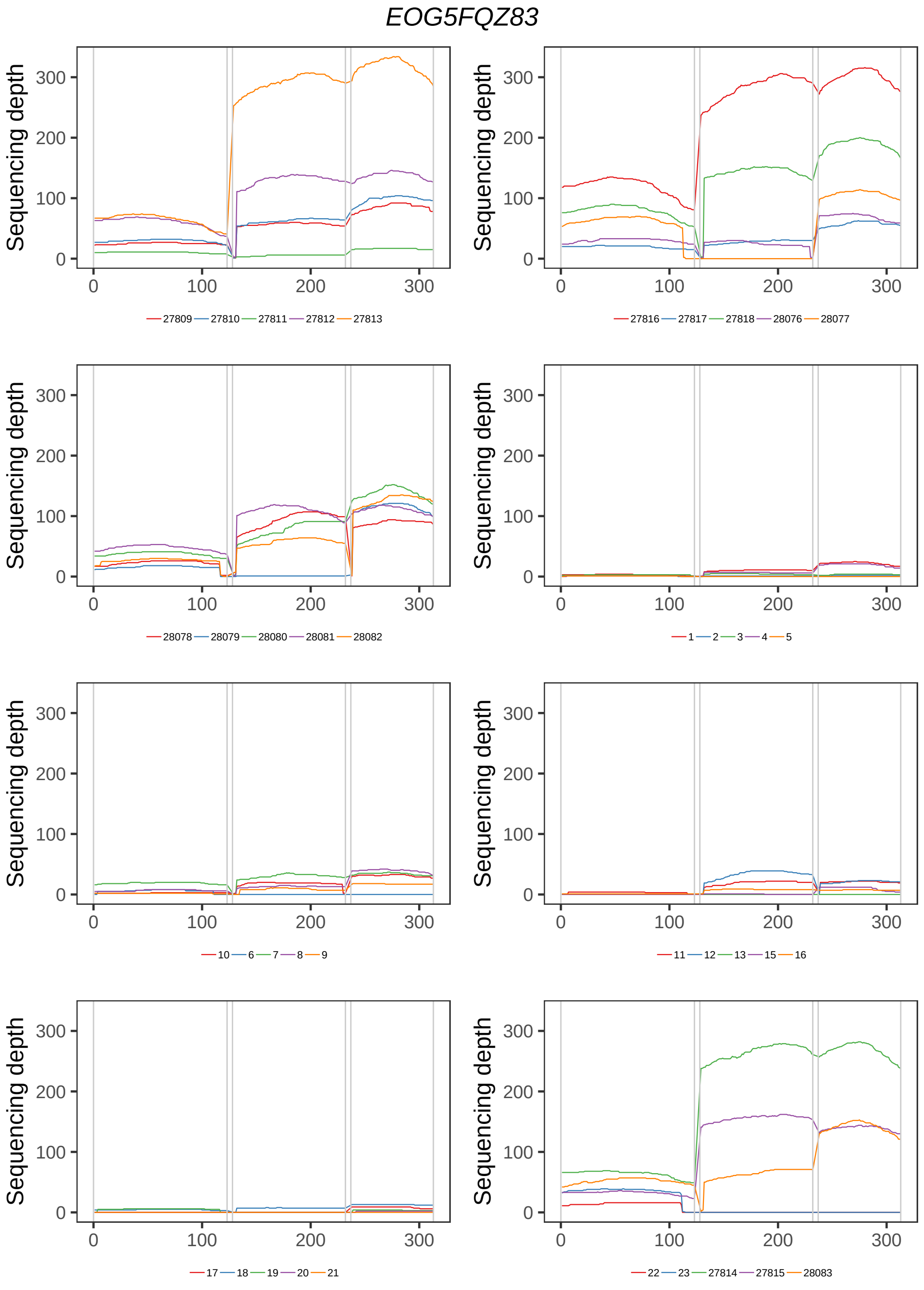
EOG5FQZ83
Sequencing depth
0
100
200
300
27809
27810
27811
27812
27813
27816
27817
27818
28076
28077
28078
28079
28080
28081
28082
1
2
3
4
5
10
6
7
8
9
11
12
13
15
16
17
18
19
20
21
22
23
27814
27815
28083

EOG5GB5NQ

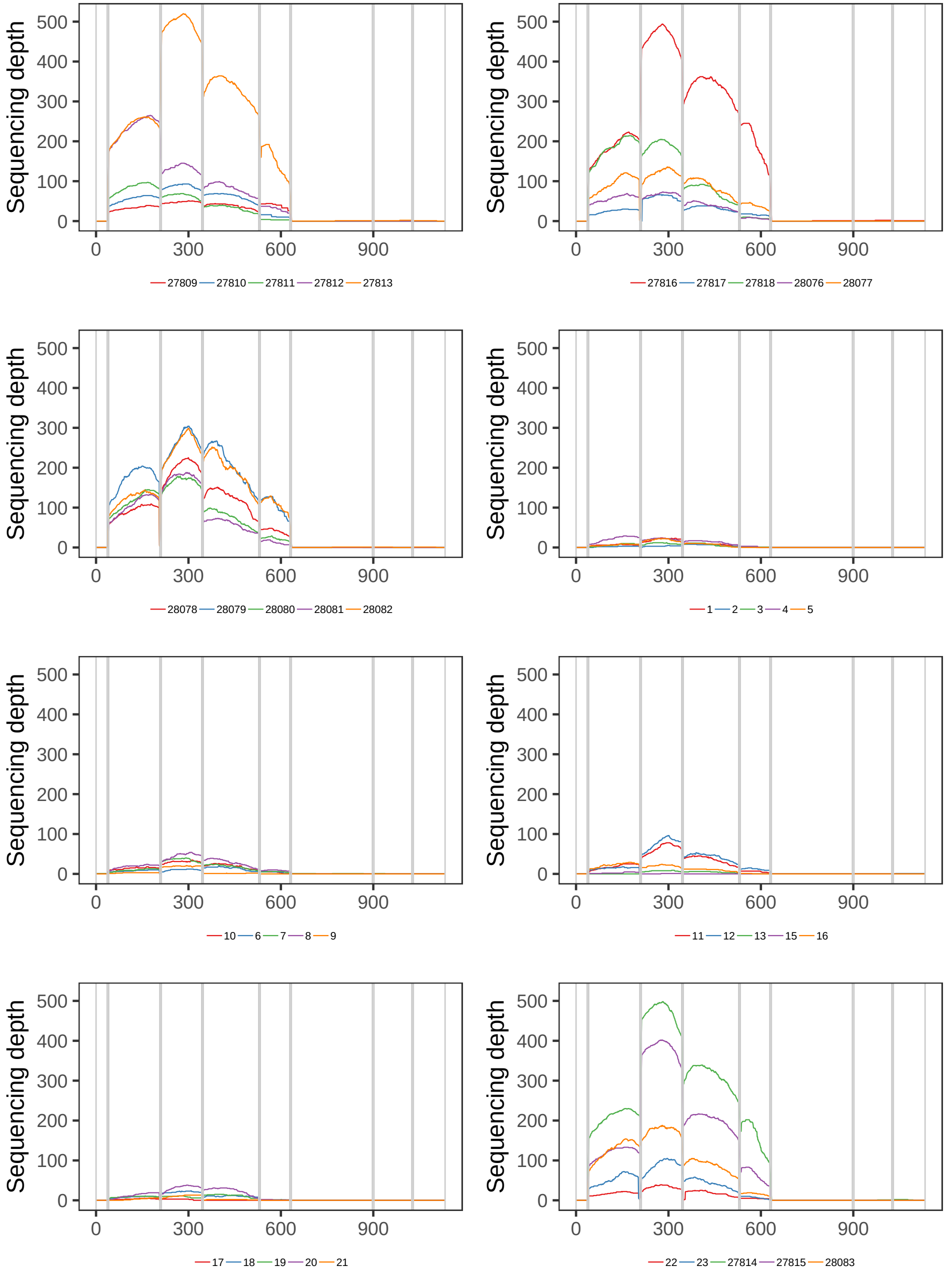
Sequencing depth
0
100
200
300
400
500
0
300
600
900
27809 27810 27811 27812 27813
27816 27817 27818 28076 28077
28078 28079 28080 28081 28082
1 2 3 4 5
10 6 7 8 9
11 12 13 15 16
17 18 19 20 21
22 23 27814 27815 28083

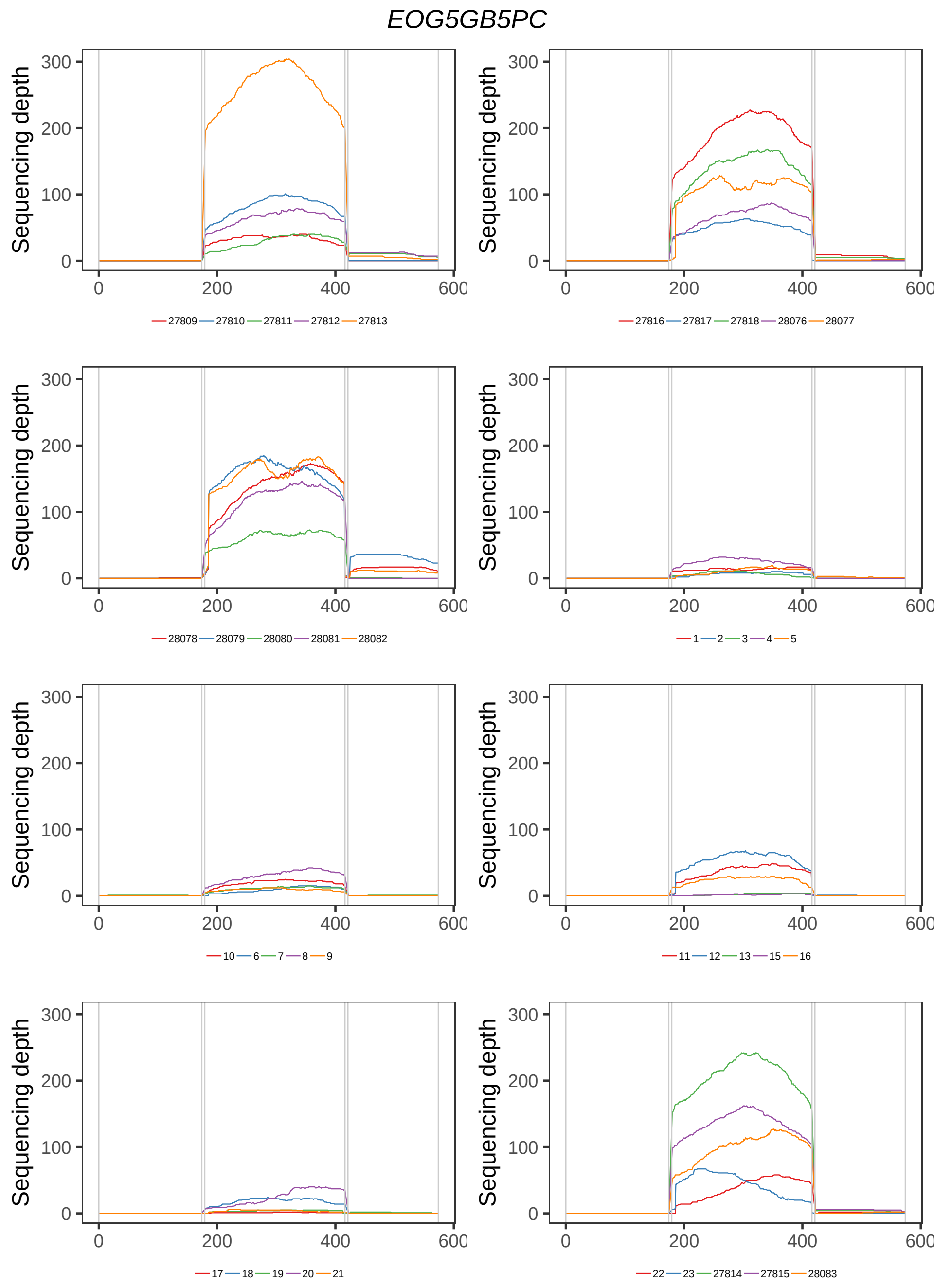
EOG5GB5PC
Sequencing depth
300
200
100
0
0
200
400
600
27809
27810
27811
27812
27813
27816
27817
27818
28076
28077
28078
28079
28080
28081
28082
1
2
3
4
5
10
6
7
8
9
11
12
13
15
16
17
18
19
20
21
22
23
27814
27815
28083

EOG5GXD33

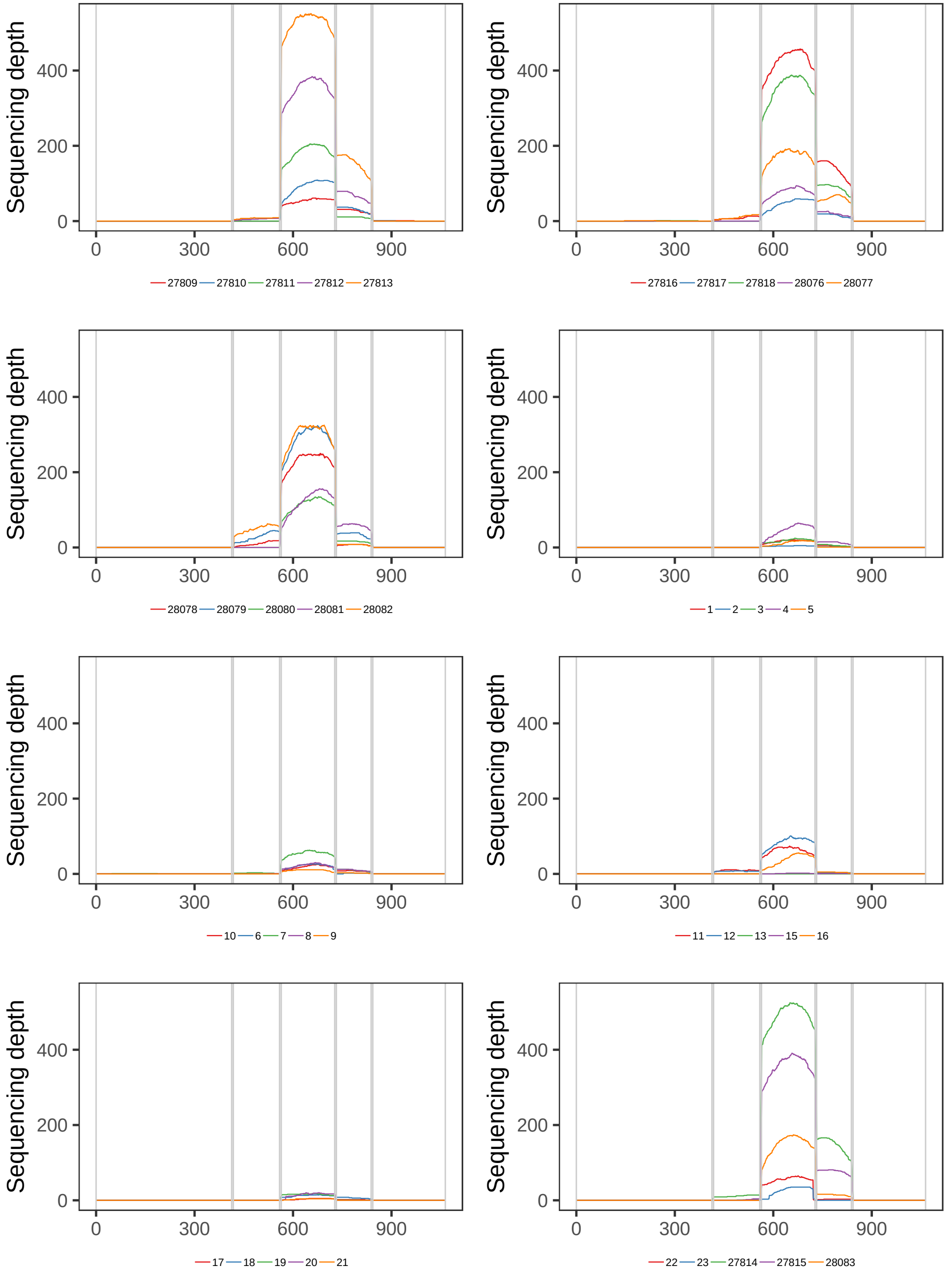
Sequencing depth
0
200
400
0
300
600
900
27809 27810 27811 27812 27813
27816 27817 27818 28076 28077
28078 28079 28080 28081 28082
1 2 3 4 5
10 6 7 8 9
11 12 13 15 16
17 18 19 20 21
22 23 27814 27815 28083

EOG5K0P4R

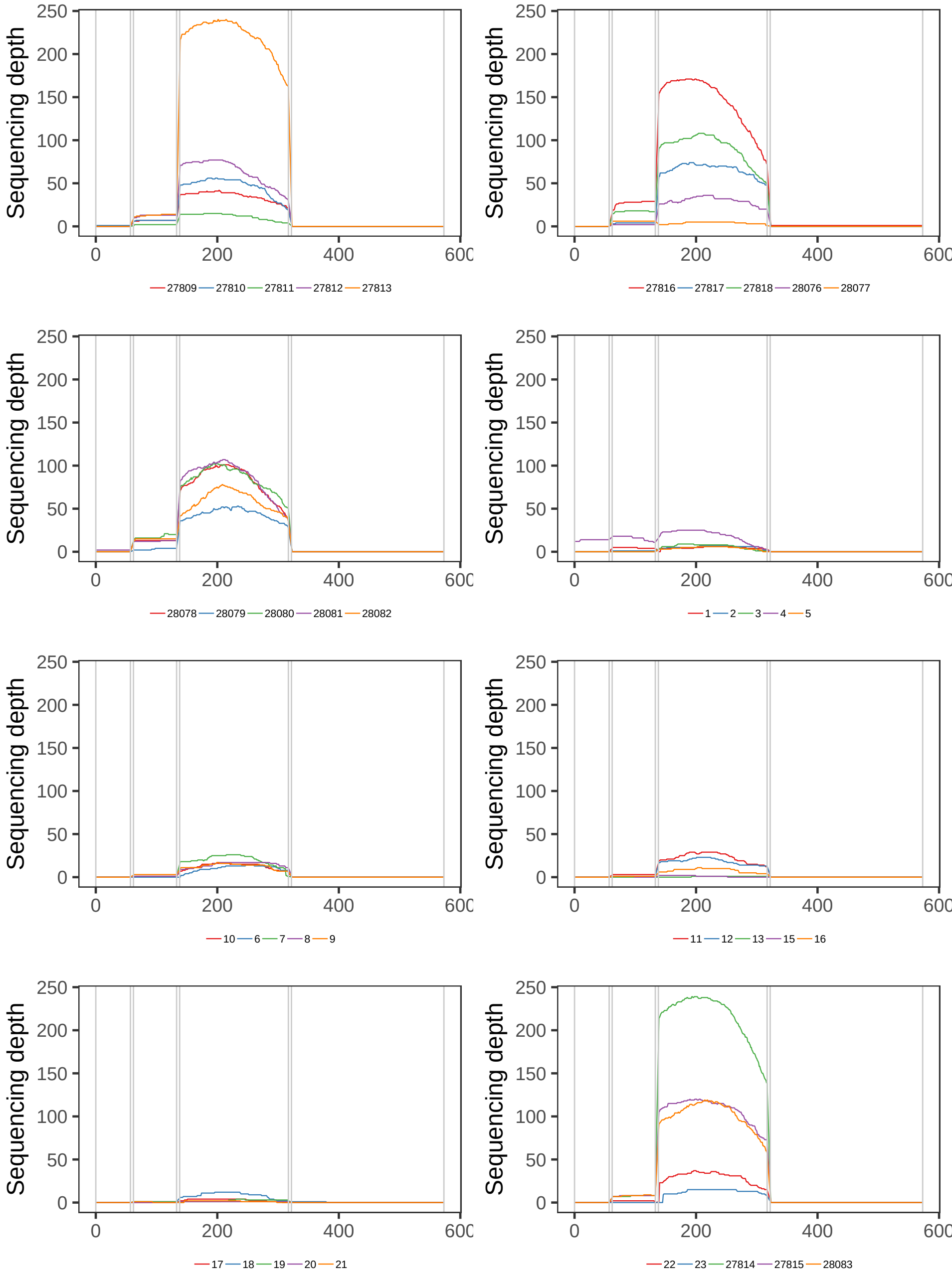
Sequencing depth
0
50
100
150
200
250
0
200
400
600
27809 27810 27811 27812 27813
27816 27817 27818 28076 28077
28078 28079 28080 28081 28082
1 2 3 4 5
10 6 7 8 9
11 12 13 15 16
17 18 19 20 21
22 23 27814 27815 28083

EOG5M906Z

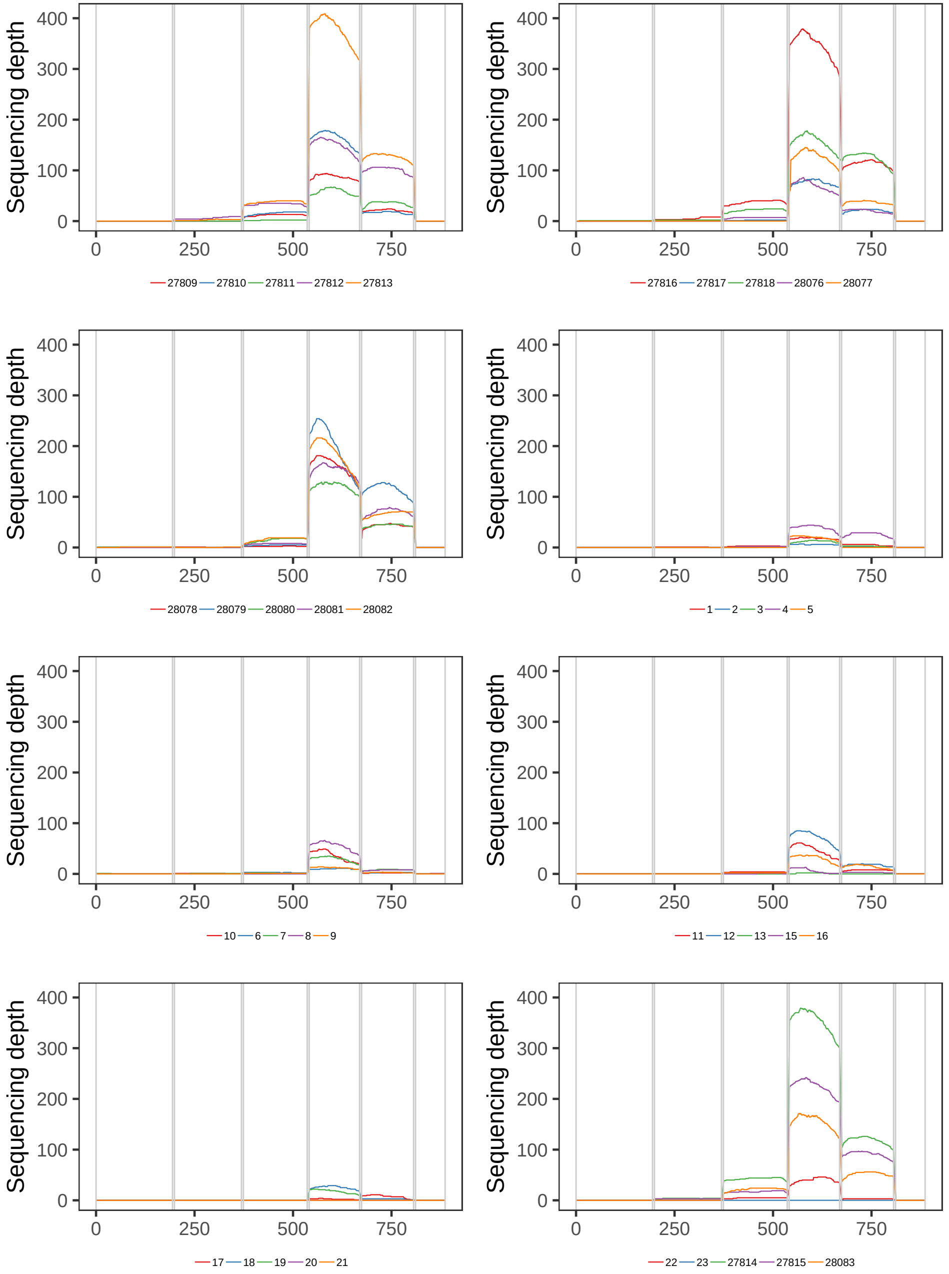
Sequencing depth
400
300
200
100
0
250
500
750
27809
27810
27811
27812
27813
27816
27817
27818
28076
28077
28078
28079
28080
28081
28082
1
2
3
4
5
10
6
7
8
9
11
12
13
15
16
17
18
19
20
21
22
23
27814
27815
28083

EOG5NVX1S

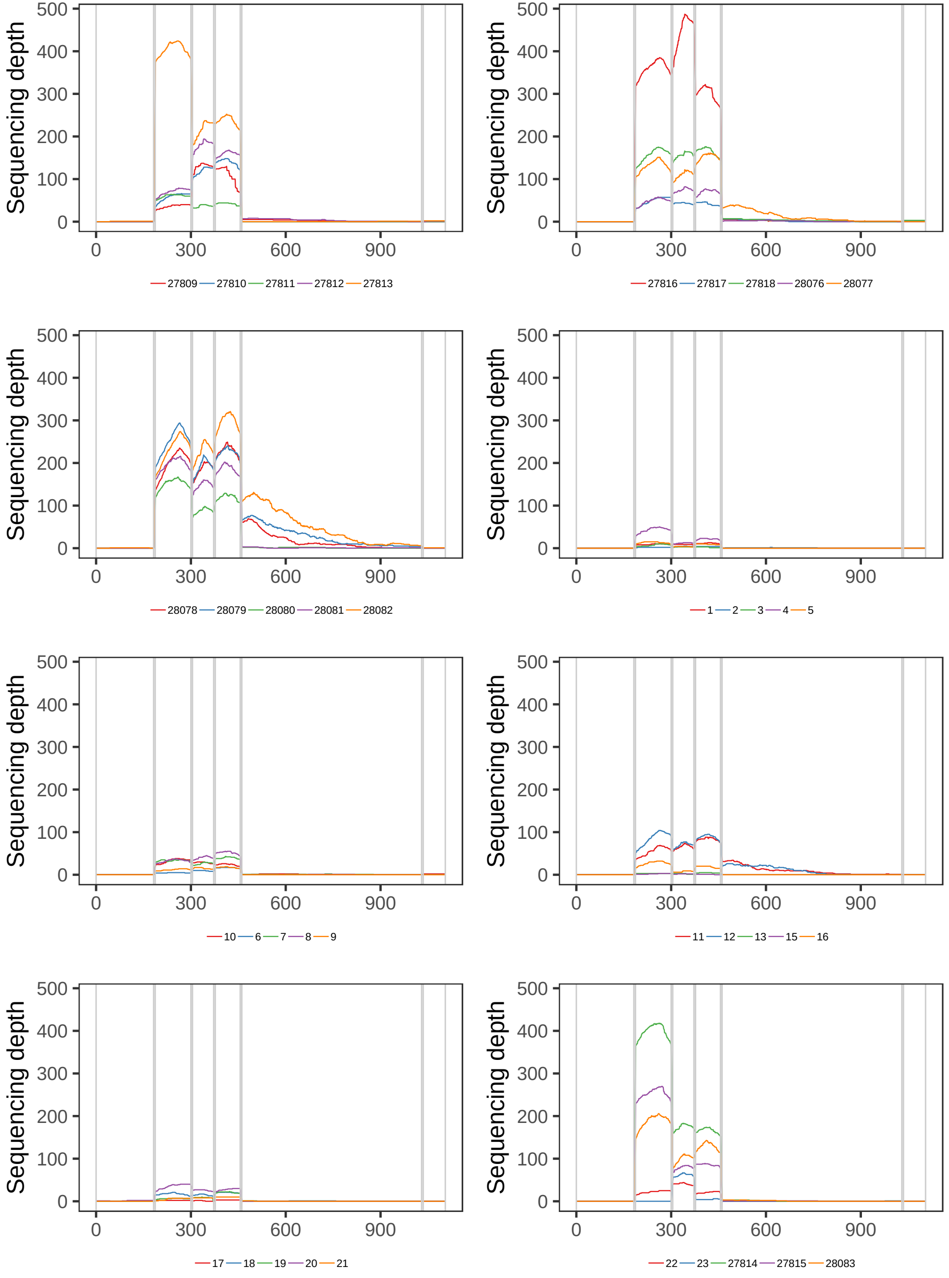
Sequencing depth
500
400
300
200
100
0
0
300
600
900
27809 27810 27811 27812 27813
27816 27817 27818 28076 28077
28078 28079 28080 28081 28082
1 2 3 4 5
10 6 7 8 9
11 12 13 15 16
17 18 19 20 21
22 23 27814 27815 28083

EOG5QZ631

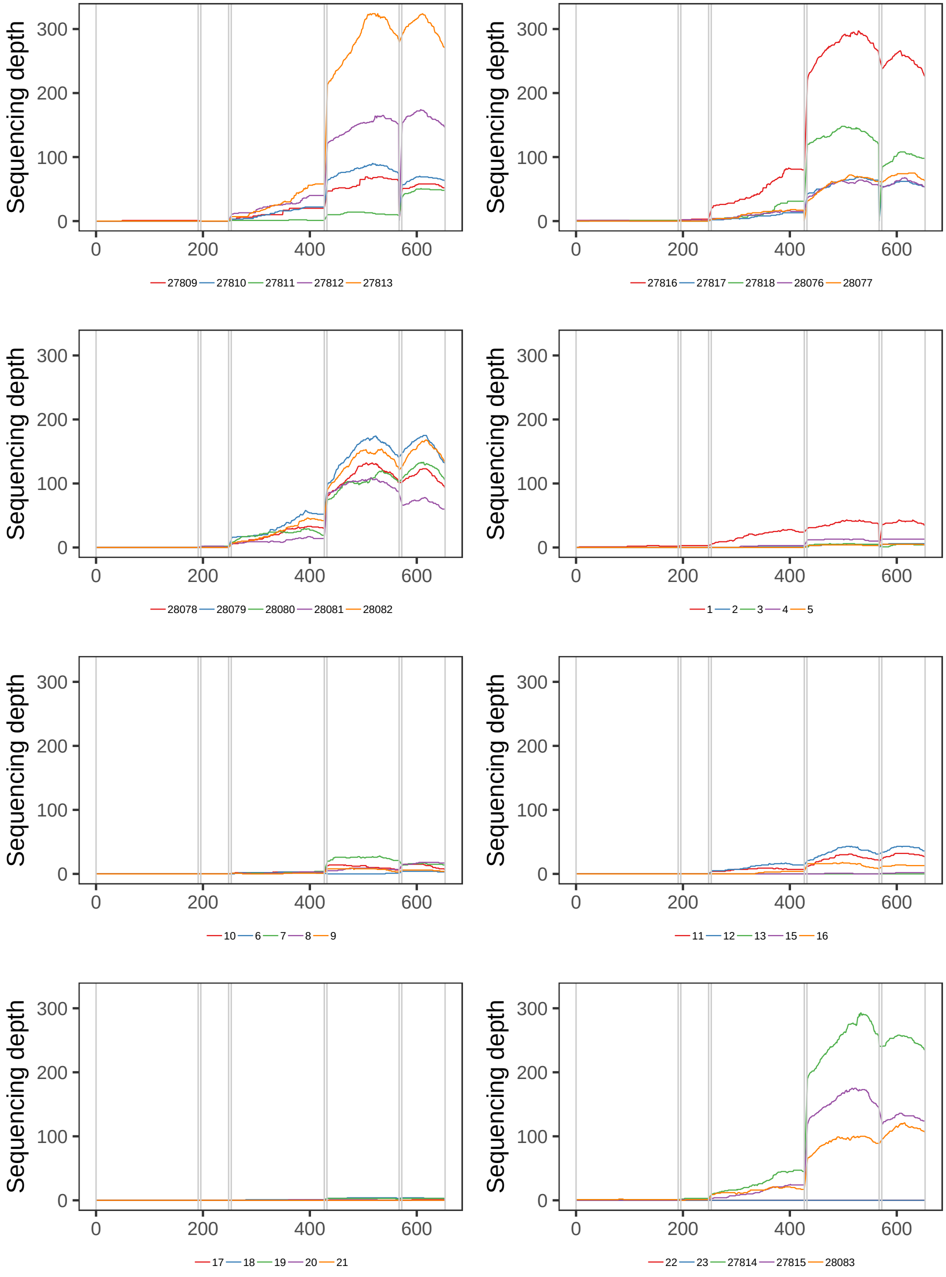
Sequencing depth
0
100
200
300
0
200
400
600
27809 27810 27811 27812 27813
27816 27817 27818 28076 28077
28078 28079 28080 28081 28082
1 2 3 4 5
10 6 7 8 9
11 12 13 15 16
17 18 19 20 21
22 23 27814 27815 28083

EOG5RBP20

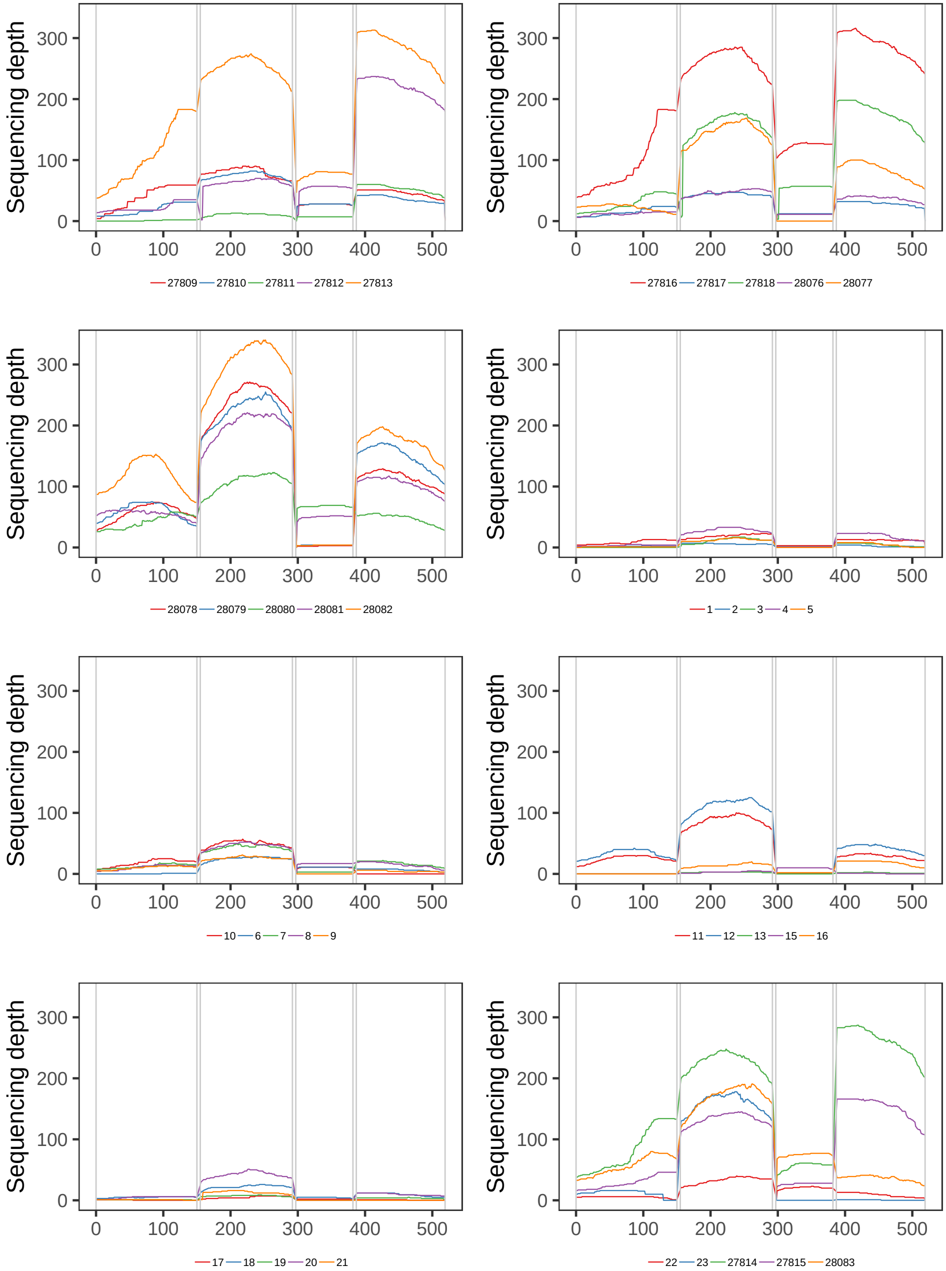
Sequencing depth
0
100
200
300
400
500
27809 27810 27811 27812 27813
27816 27817 27818 28076 28077
28078 28079 28080 28081 28082
1 2 3 4 5
10 6 7 8 9
11 12 13 15 16
17 18 19 20 21
22 23 27814 27815 28083

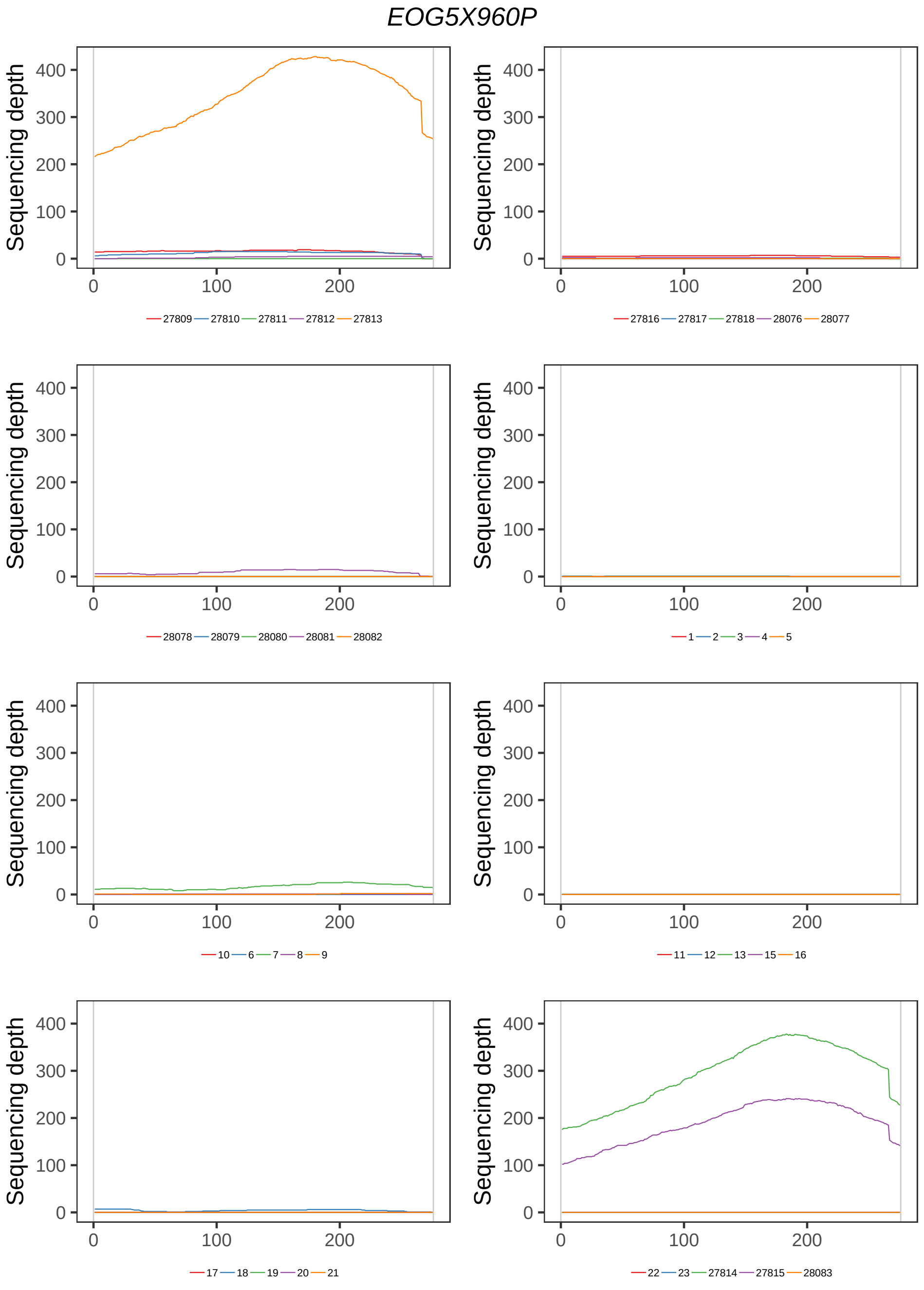
EOG5X960P
Sequencing depth
400
300
200
100
0
0
100
200
27809 27810 27811 27812 27813
27816 27817 27818 28076 28077
28078 28079 28080 28081 28082
1 2 3 4 5
10 6 7 8 9
11 12 13 15 16
17 18 19 20 21
22 23 27814 27815 28083

EOG52RBP8

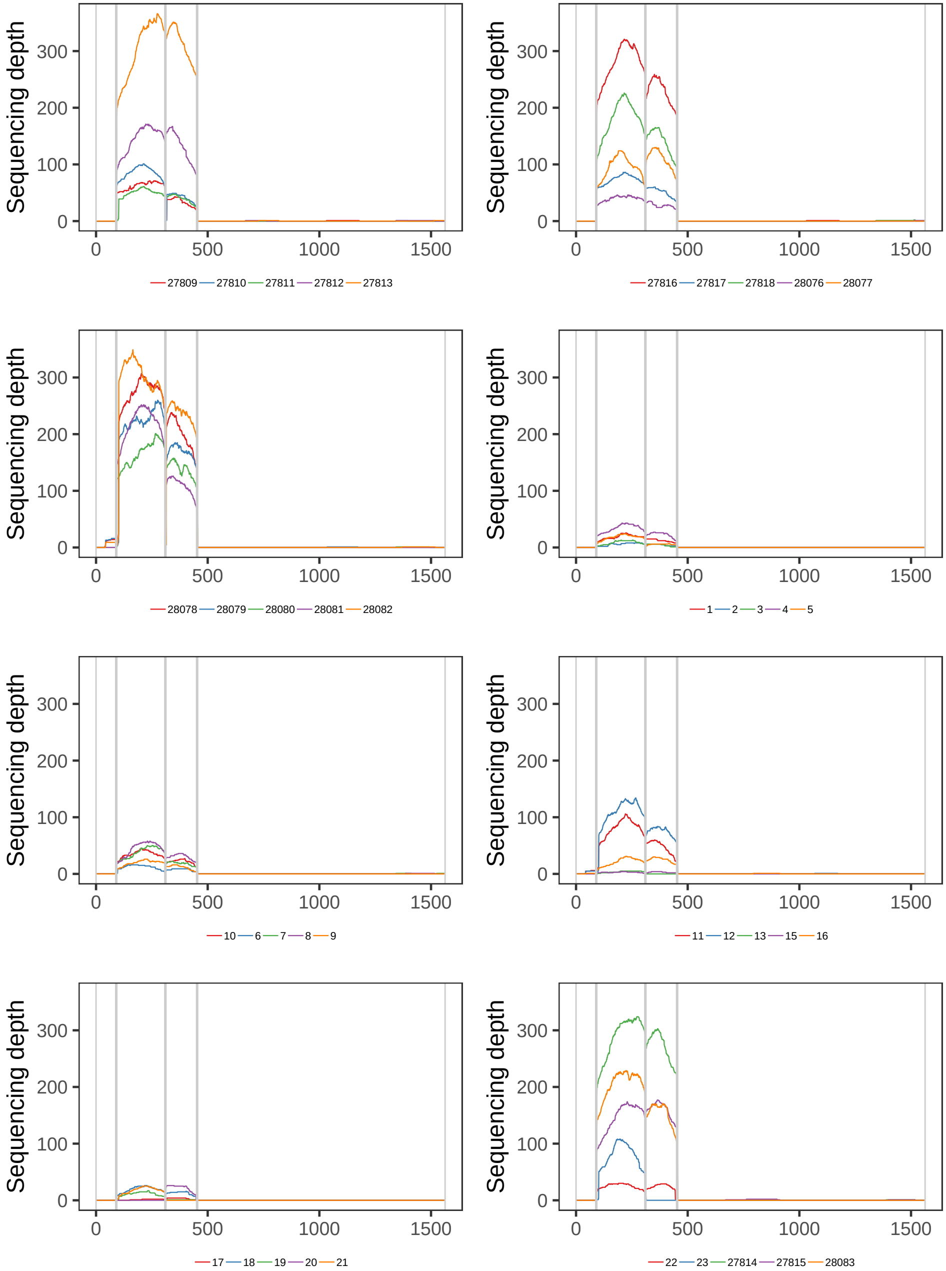
Sequencing depth
0
100
200
300
0
500
1000
1500
27809 27810 27811 27812 27813
27816 27817 27818 28076 28077
28078 28079 28080 28081 28082
1 2 3 4 5
10 6 7 8 9
11 12 13 15 16
17 18 19 20 21
22 23 27814 27815 28083

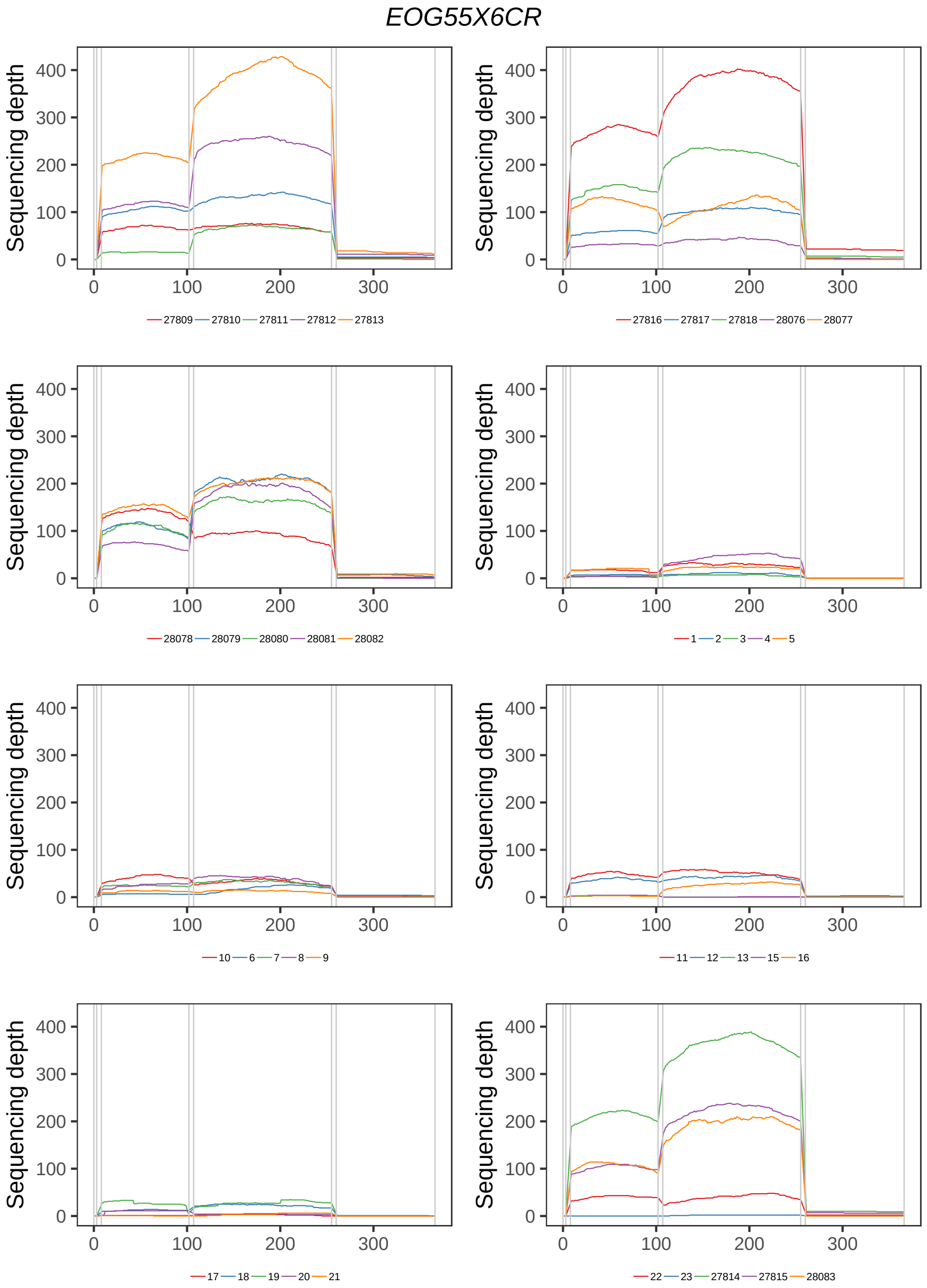
EOG55X6CR
Sequencing depth
27809 27810 27811 27812 27813
27816 27817 27818 28076 28077
28078 28079 28080 28081 28082
1 2 3 4 5
10 6 7 8 9
11 12 13 15 16
17 18 19 20 21
22 23 27814 27815 28083

EOG563XTD

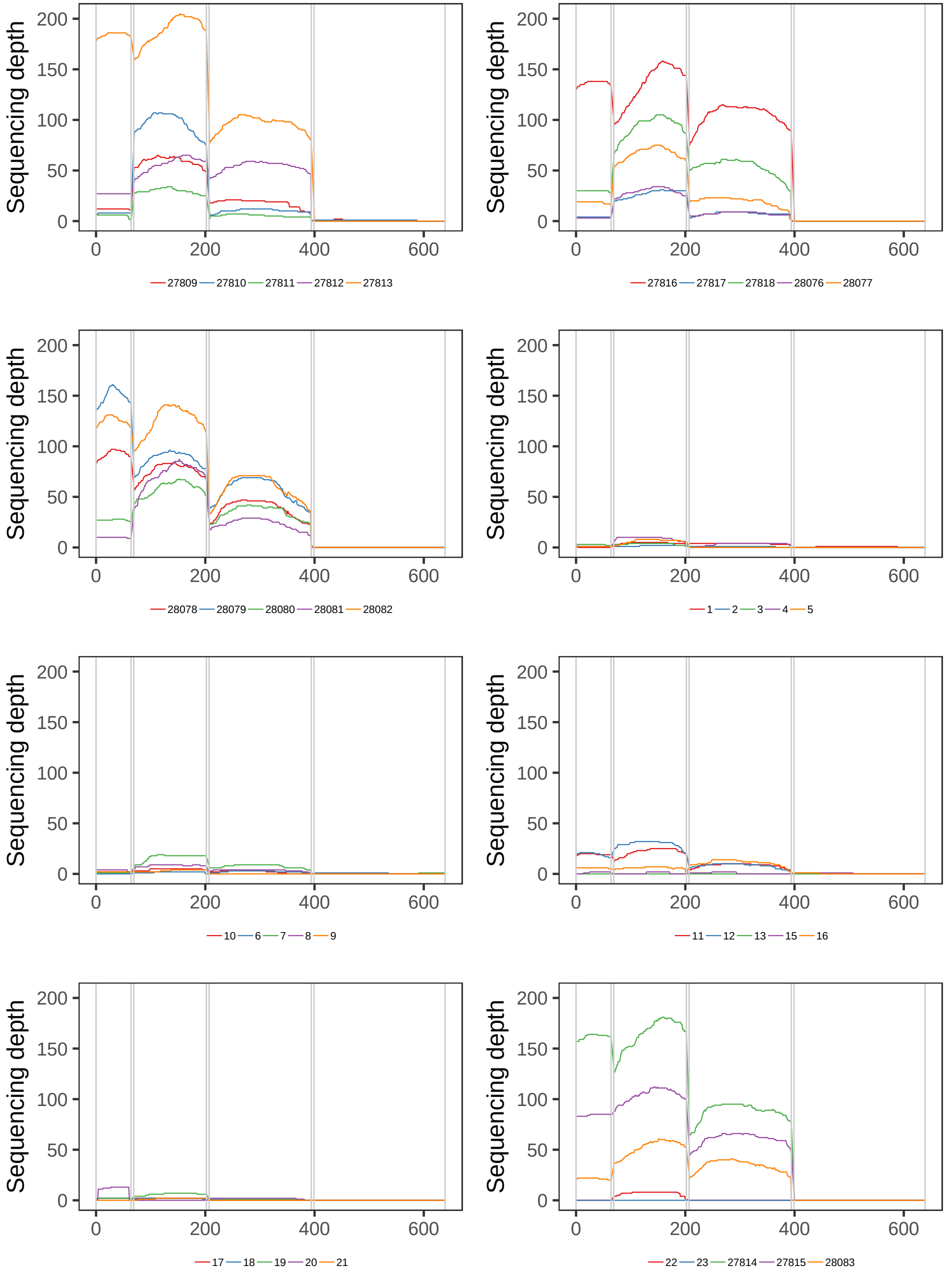
Sequencing depth
0
50
100
150
200
0
200
400
600
27809 27810 27811 27812 27813
27816 27817 27818 28076 28077
28078 28079 28080 28081 28082
1 2 3 4 5
10 6 7 8 9
11 12 13 15 16
17 18 19 20 21
22 23 27814 27815 28083

EOG57SQWM

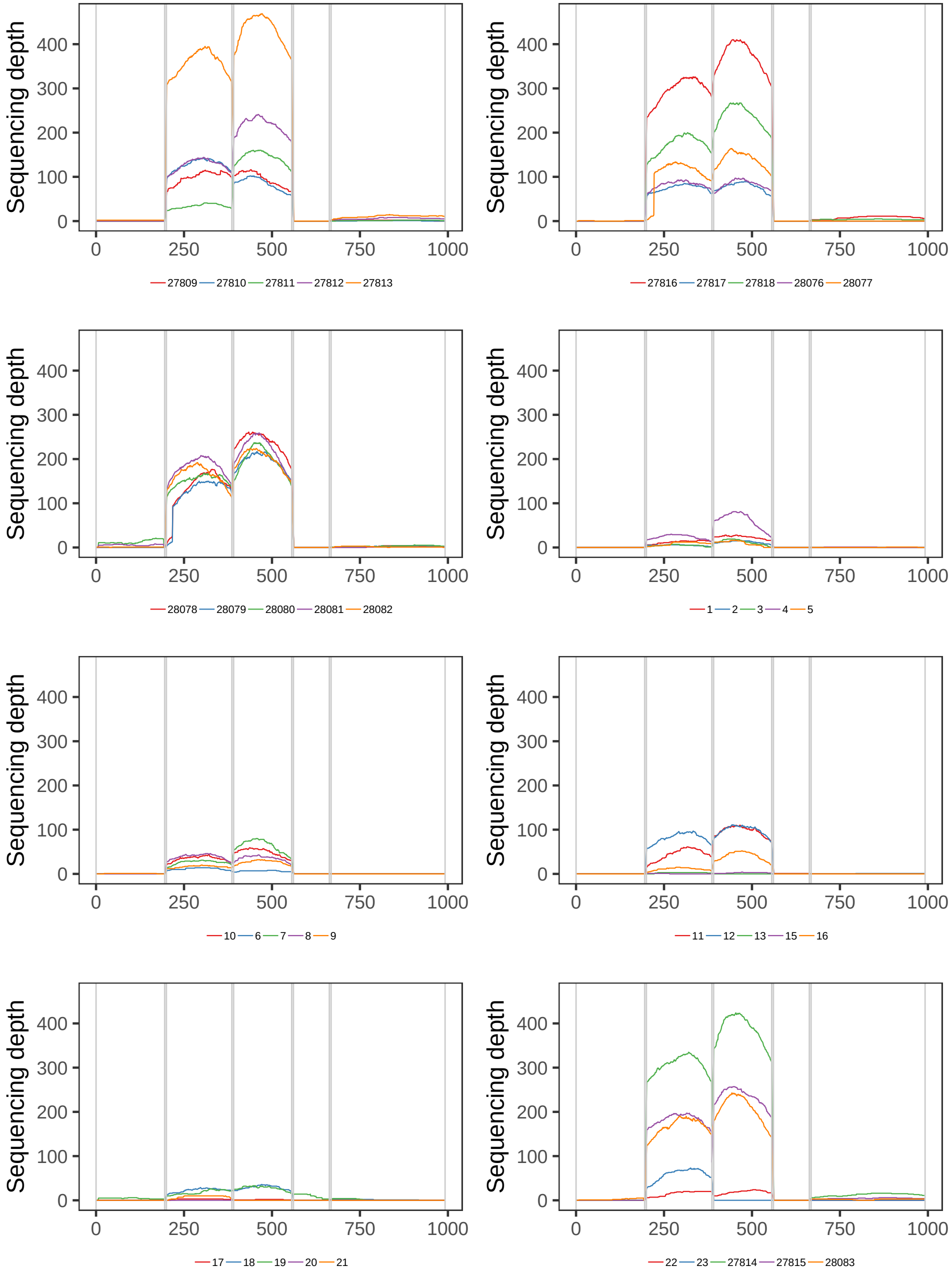

Sequencing depth
400
300
200
100
0
0
250
500
750
1000
27809 27810 27811 27812 27813
27816 27817 27818 28076 28077
28078 28079 28080 28081 28082
1 2 3 4 5
10 6 7 8 9
11 12 13 15 16
17 18 19 20 21
22 23 27814 27815 28083

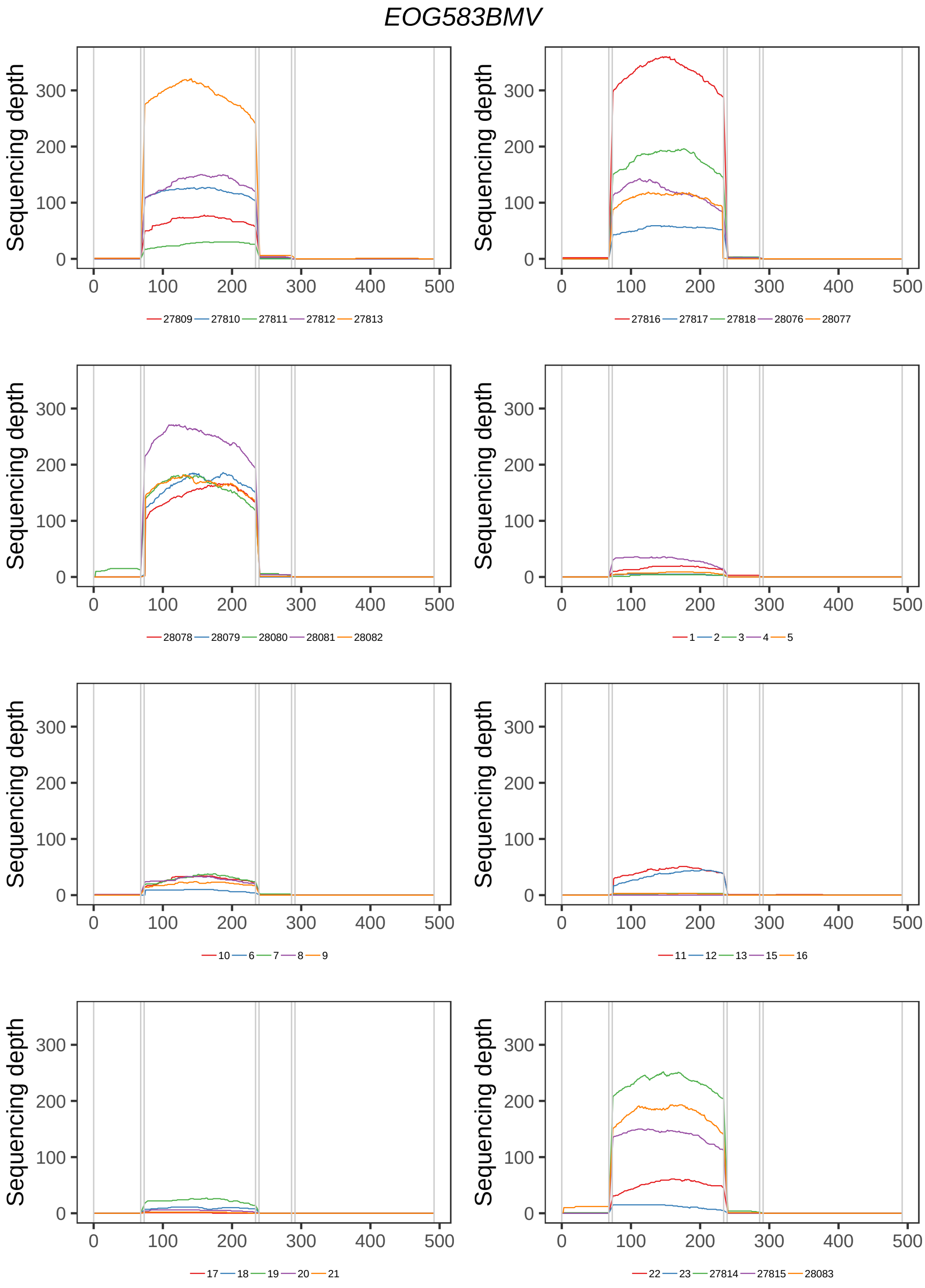

EOG583BMV
Sequencing depth
0
100
200
300
400
500
27809 27810 27811 27812 27813
27816 27817 27818 28076 28077
28078 28079 28080 28081 28082
1 2 3 4 5
10 6 7 8 9
11 12 13 15 16
17 18 19 20 21
22 23 27814 27815 28083

EOG58CZ9M

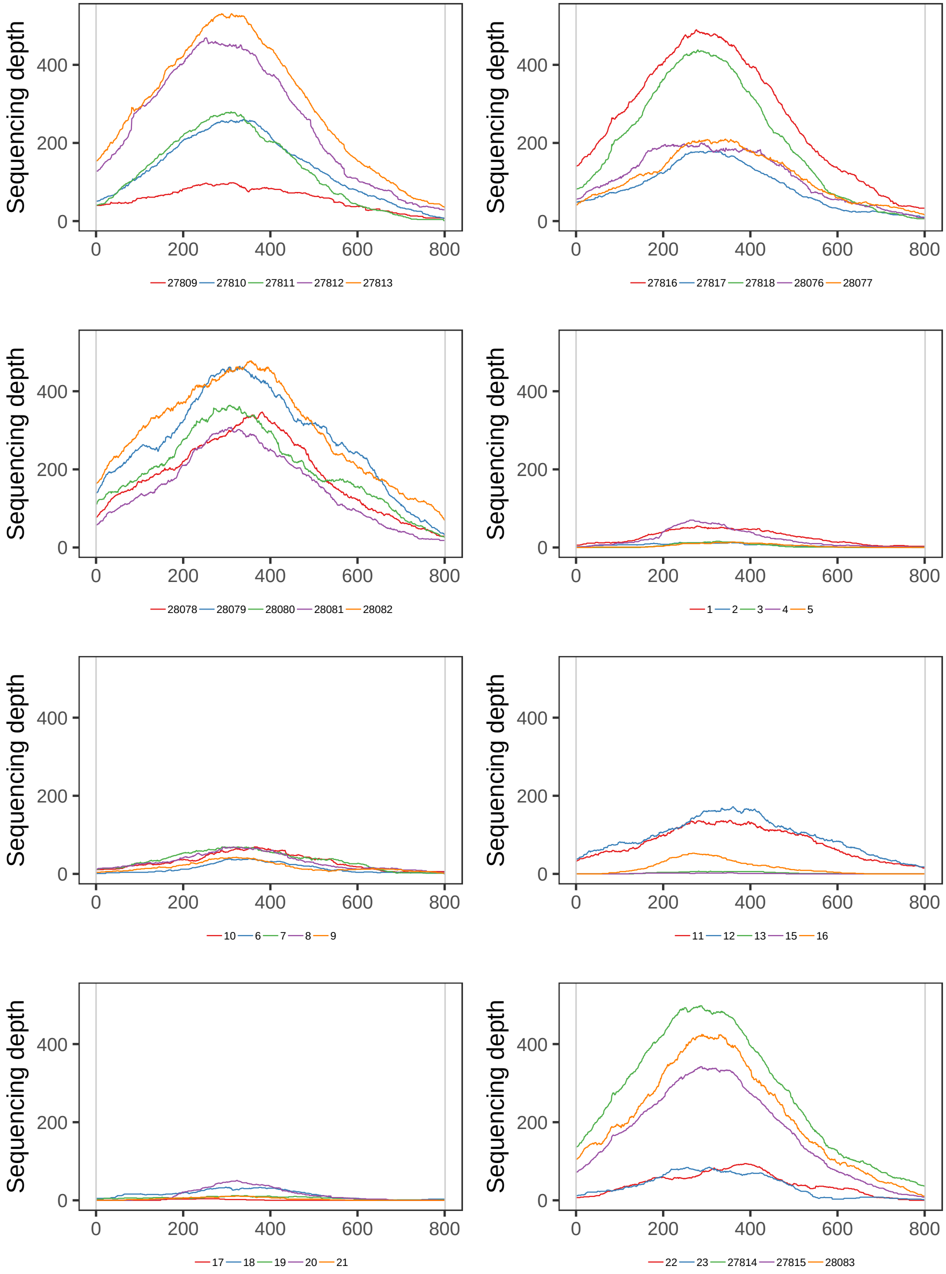
Sequencing depth
0
200
400
600
800
27809 27810 27811 27812 27813
27816 27817 27818 28076 28077
28078 28079 28080 28081 28082
1 2 3 4 5
10 6 7 8 9
11 12 13 15 16
17 18 19 20 21
22 23 27814 27815 28083

EOG598SG5

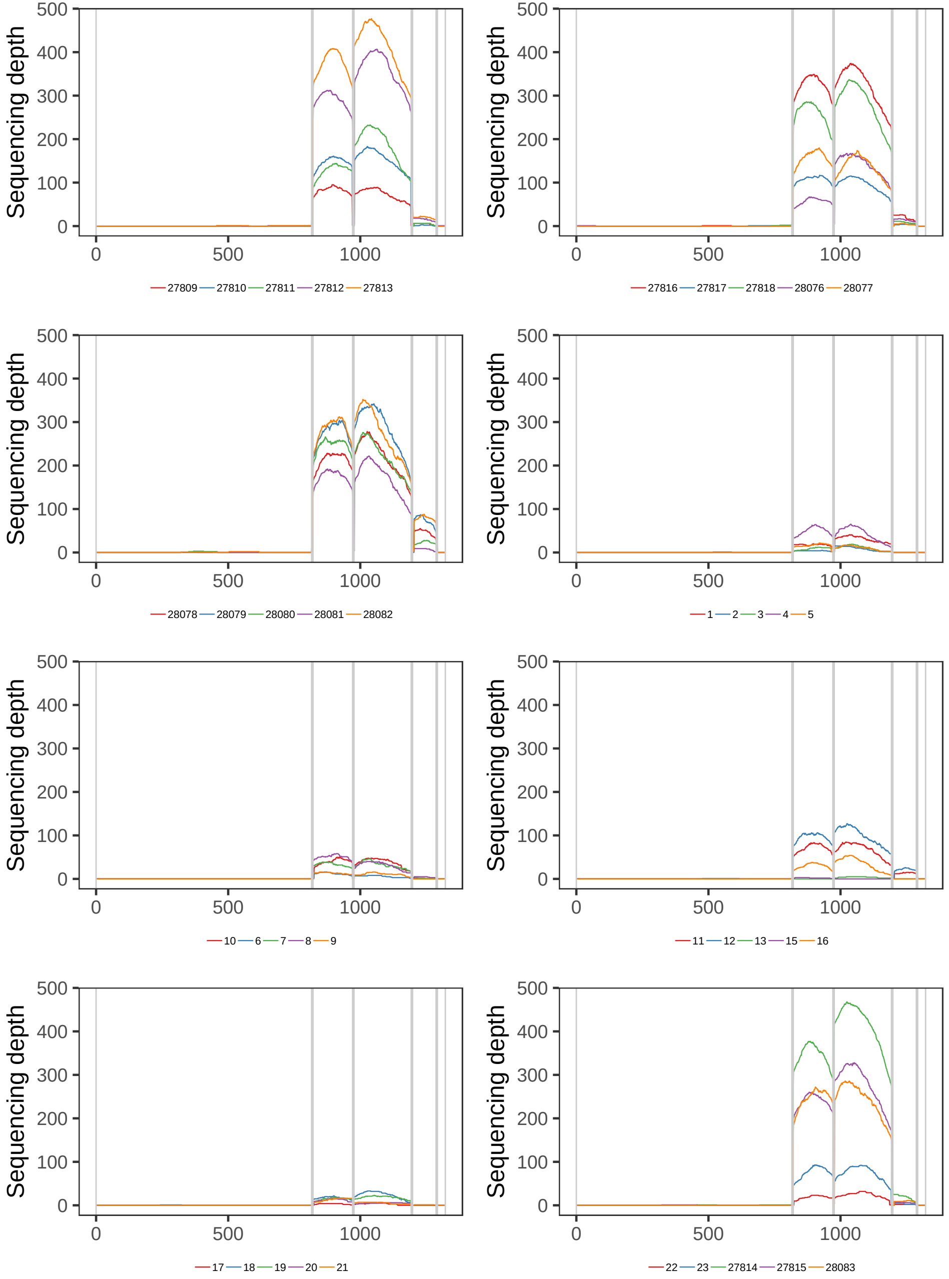
Sequencing depth
0
100
200
300
400
500
0
500
1000
27809 27810 27811 27812 27813
27816 27817 27818 28076 28077
28078 28079 28080 28081 28082
1 2 3 4 5
10 6 7 8 9
11 12 13 15 16
17 18 19 20 21
22 23 27814 27815 28083

EOG5BK3JV

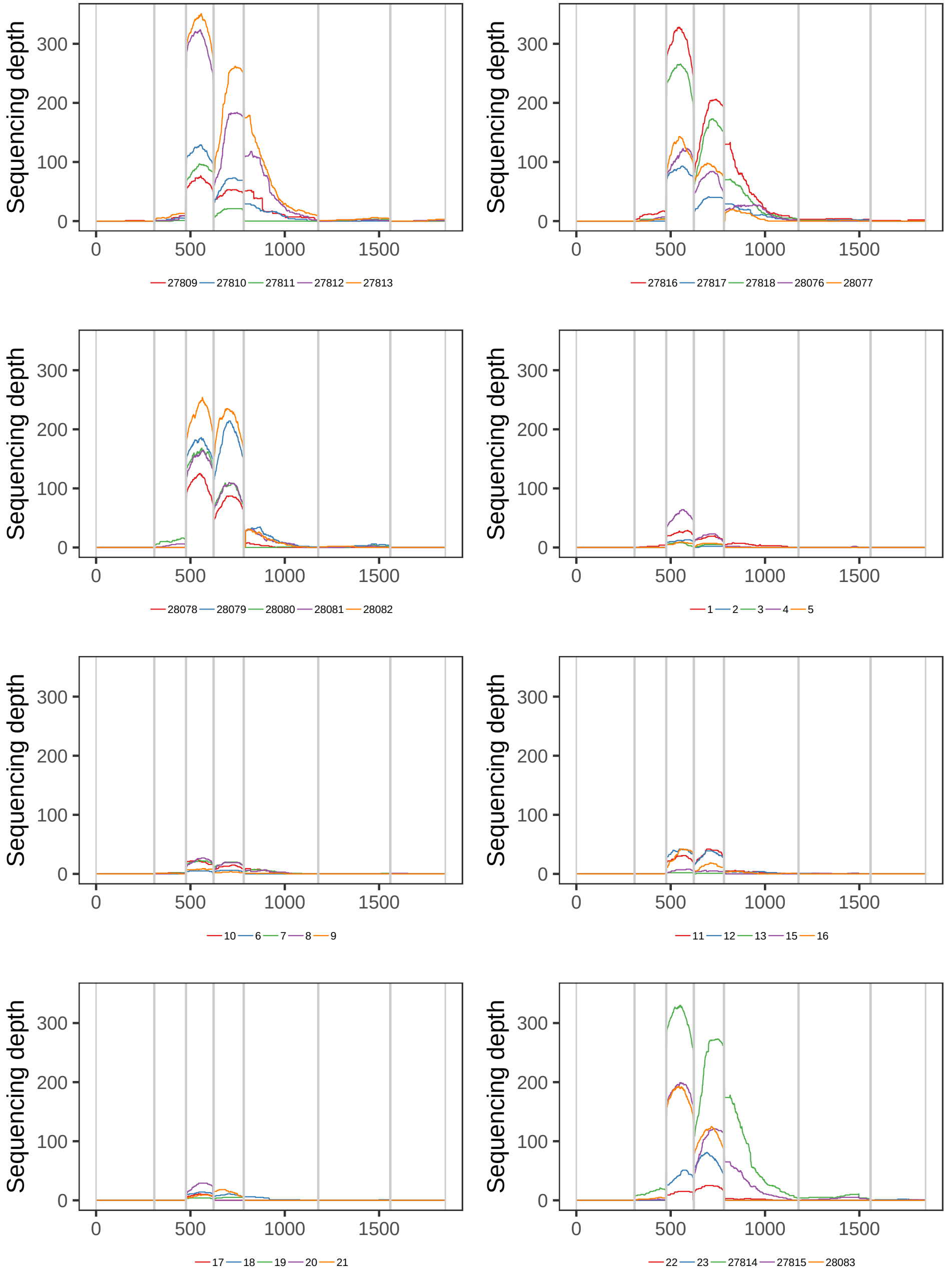
Sequencing depth
0
100
200
300
0
500
1000
1500
27809 27810 27811 27812 27813
27816 27817 27818 28076 28077
28078 28079 28080 28081 28082
1 2 3 4 5
10 6 7 8 9
11 12 13 15 16
17 18 19 20 21
22 23 27814 27815 28083

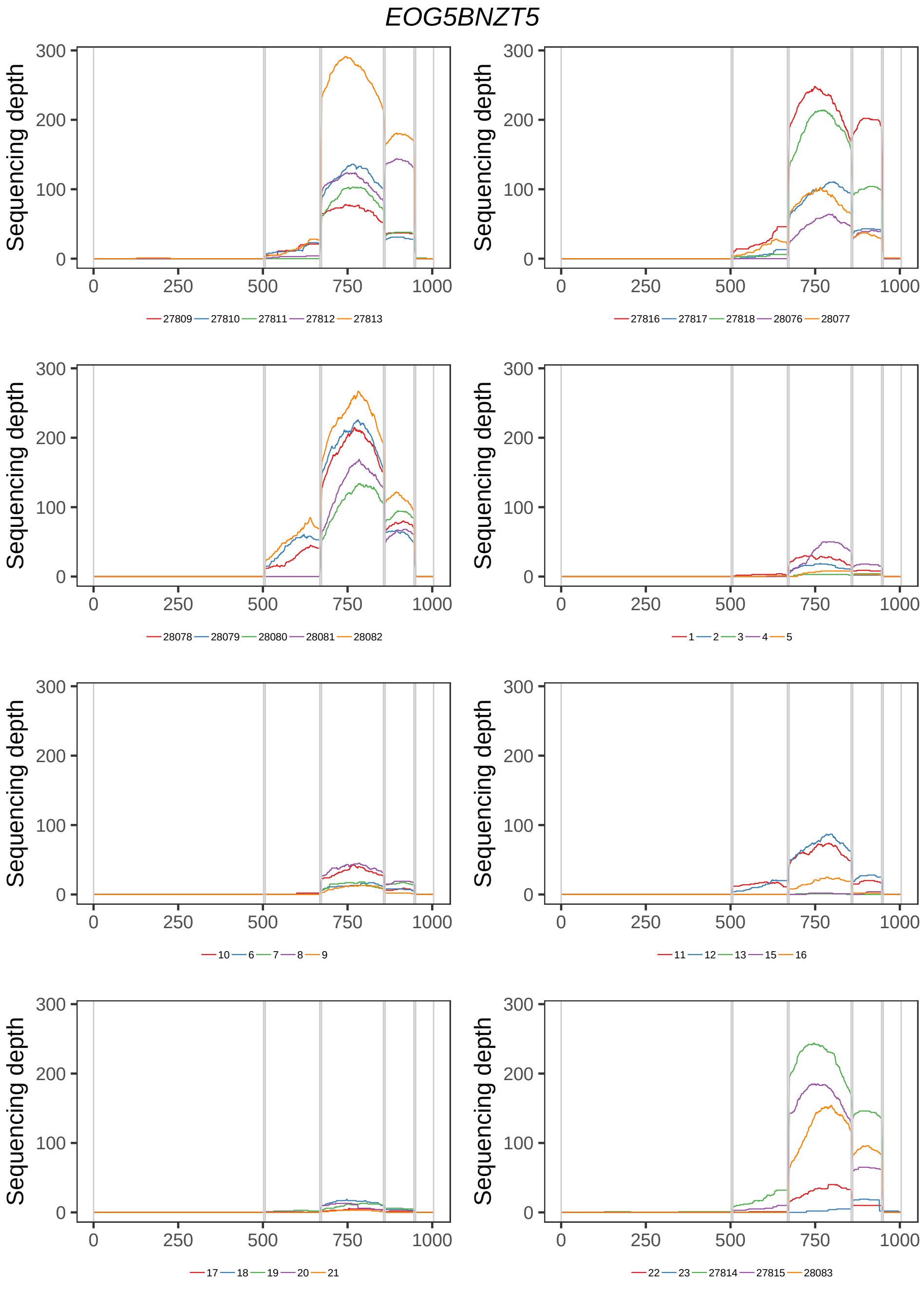
EOG5BNZT5
Sequencing depth
27809 27810 27811 27812 27813
27816 27817 27818 28076 28077
28078 28079 28080 28081 28082
1 2 3 4 5
10 6 7 8 9
11 12 13 15 16
17 18 19 20 21
22 23 27814 27815 28083

EOG5D255M

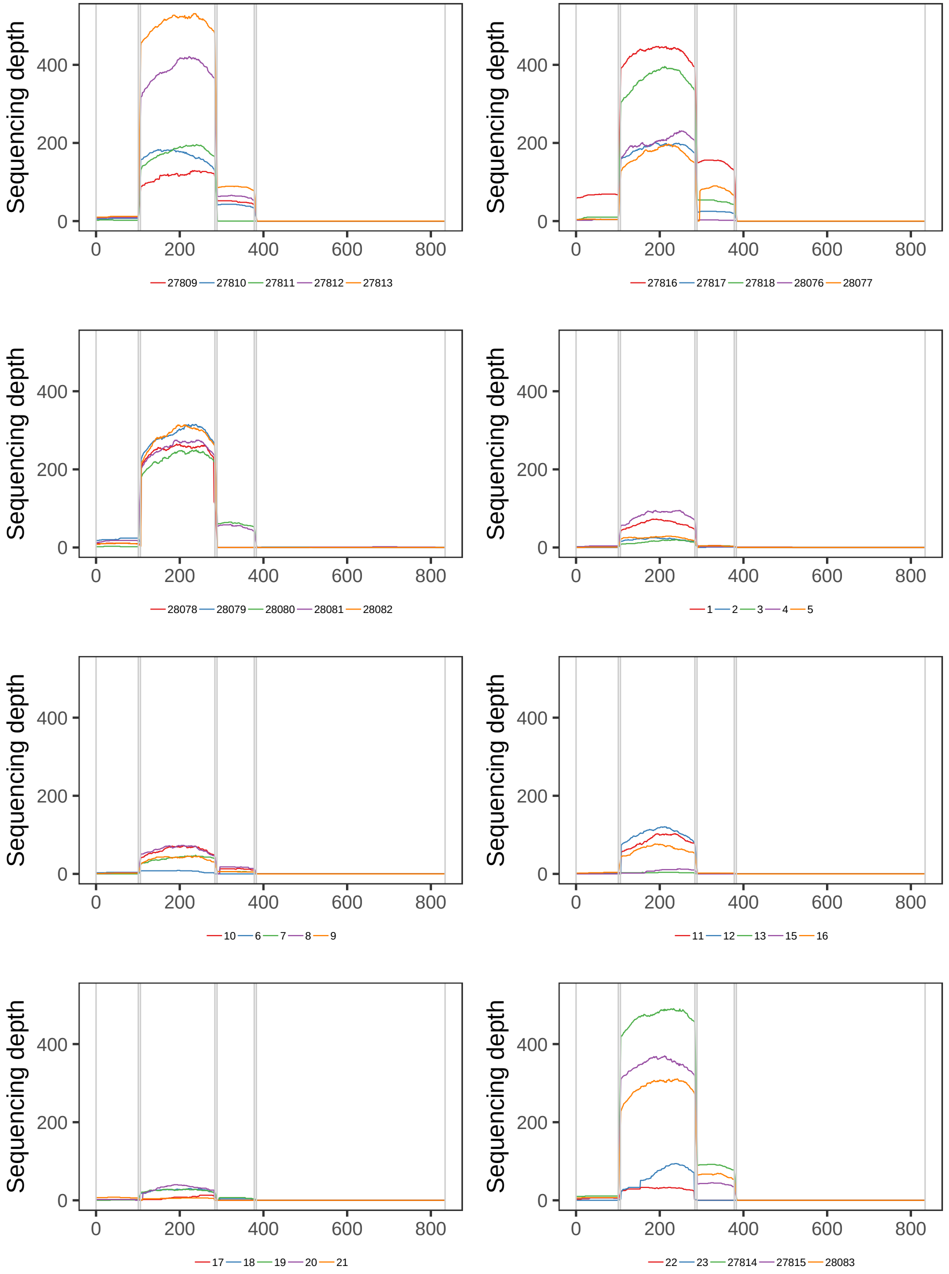
Sequencing depth
0
200
400
0
200
400
600
800
27809 27810 27811 27812 27813
27816 27817 27818 28076 28077
28078 28079 28080 28081 28082
1 2 3 4 5
10 6 7 8 9
11 12 13 15 16
17 18 19 20 21
22 23 27814 27815 28083

EOG5DFN3X

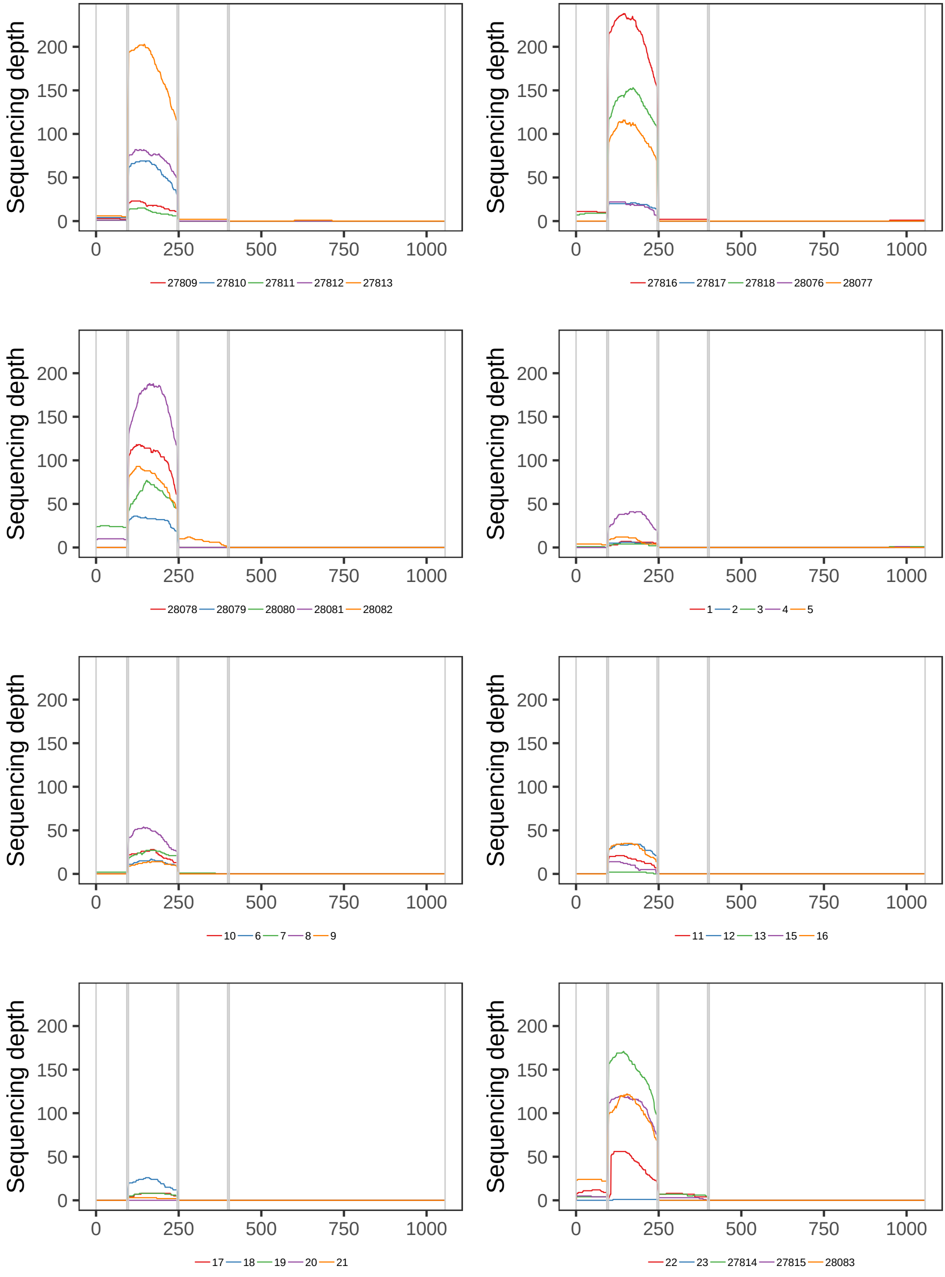
Sequencing depth
0
50
100
150
200
250
500
750
1000
27809 27810 27811 27812 27813
27816 27817 27818 28076 28077
28078 28079 28080 28081 28082
1 2 3 4 5
10 6 7 8 9
11 12 13 15 16
17 18 19 20 21
22 23 27814 27815 28083

EOG5G79D7

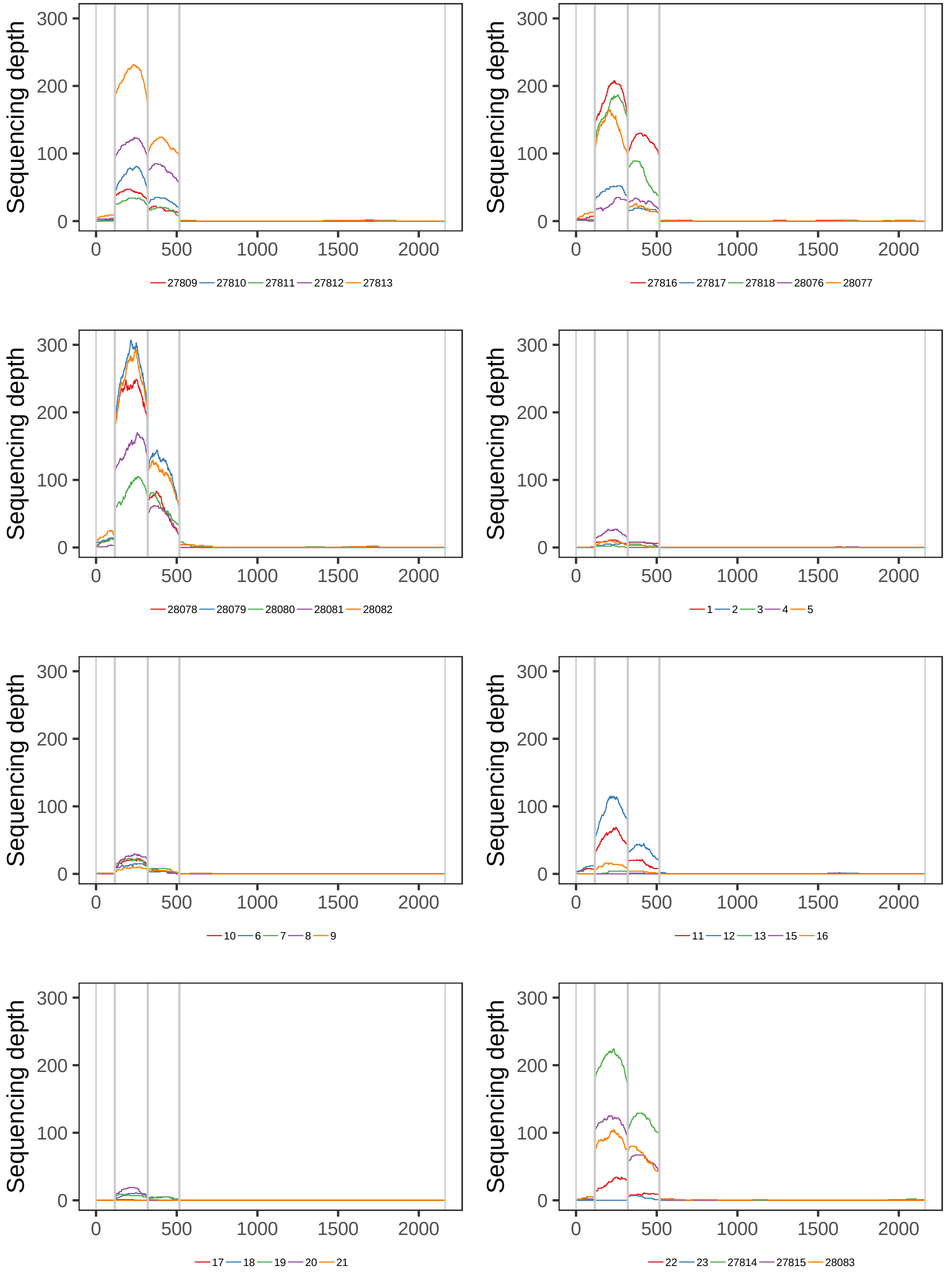
Sequencing depth
0
100
200
300
0
500
1000
1500
2000
27809 27810 27811 27812 27813
27816 27817 27818 28076 28077
28078 28079 28080 28081 28082
1 2 3 4 5
10 6 7 8 9
11 12 13 15 16
17 18 19 20 21
22 23 27814 27815 28083

EOG5GB5N6

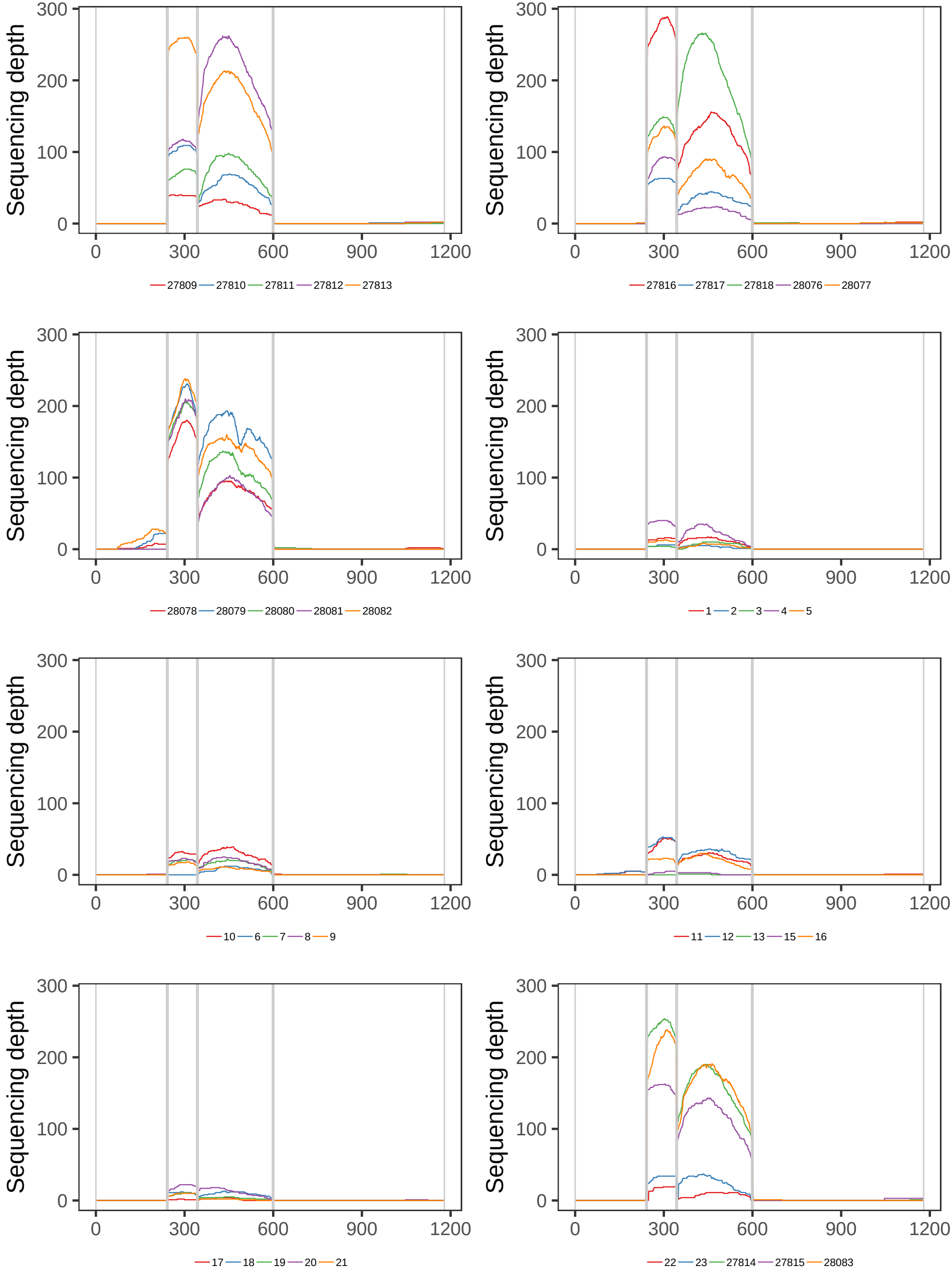
Sequencing depth
0
100
200
300
0
300
600
900
1200
27809 27810 27811 27812 27813
27816 27817 27818 28076 28077
28078 28079 28080 28081 28082
1 2 3 4 5
10 6 7 8 9
11 12 13 15 16
17 18 19 20 21
22 23 27814 27815 28083

EOG5HQC0Q

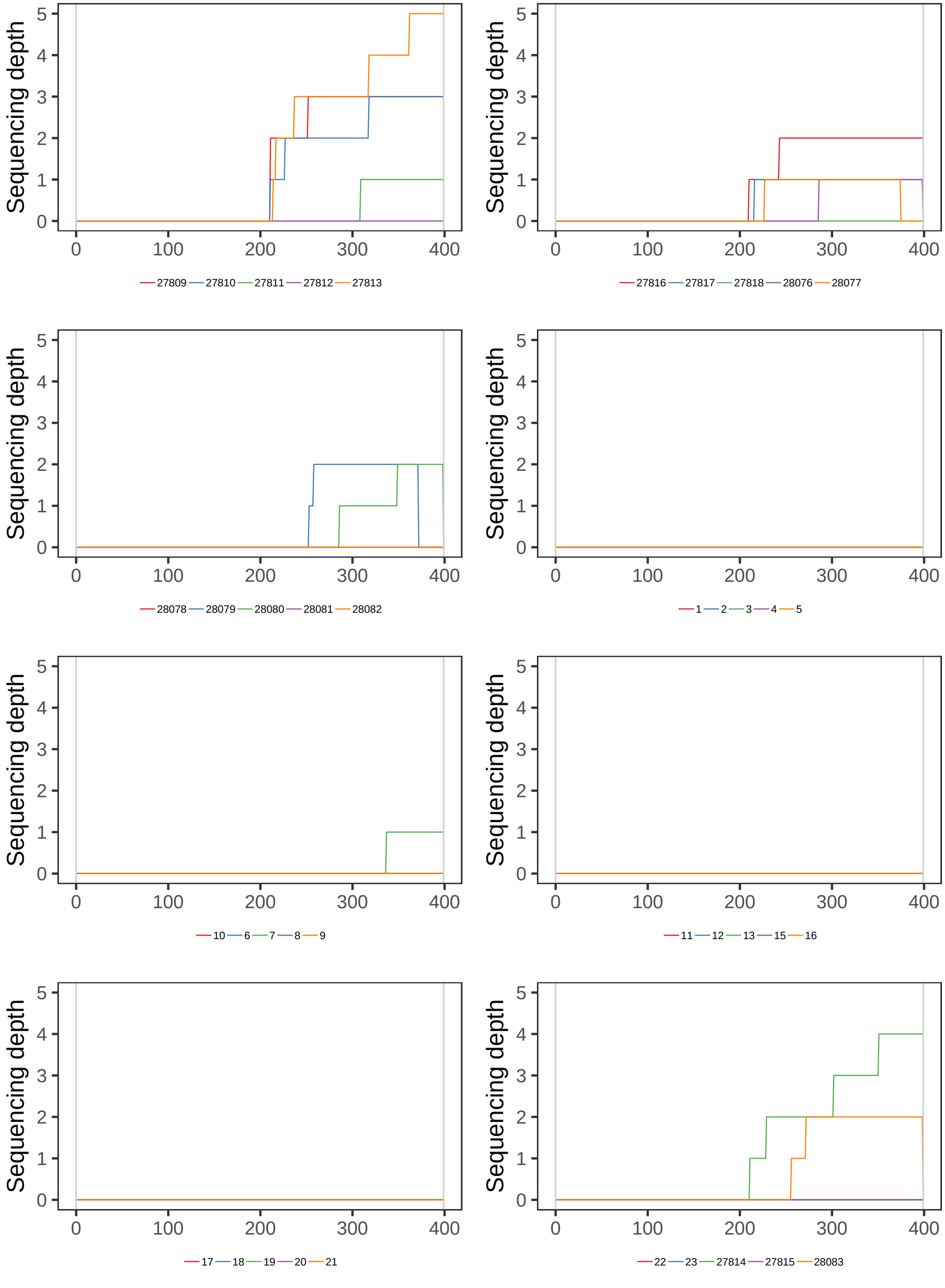

Sequencing depth
27809
27810
27811
27812
27813
27816
27817
27818
28076
28077
28078
28079
28080
28081
28082
1
2
3
4
5
10
6
7
8
9
11
12
13
15
16
17
18
19
20
21
22
23
27814
27815
28083

EOG5M640X

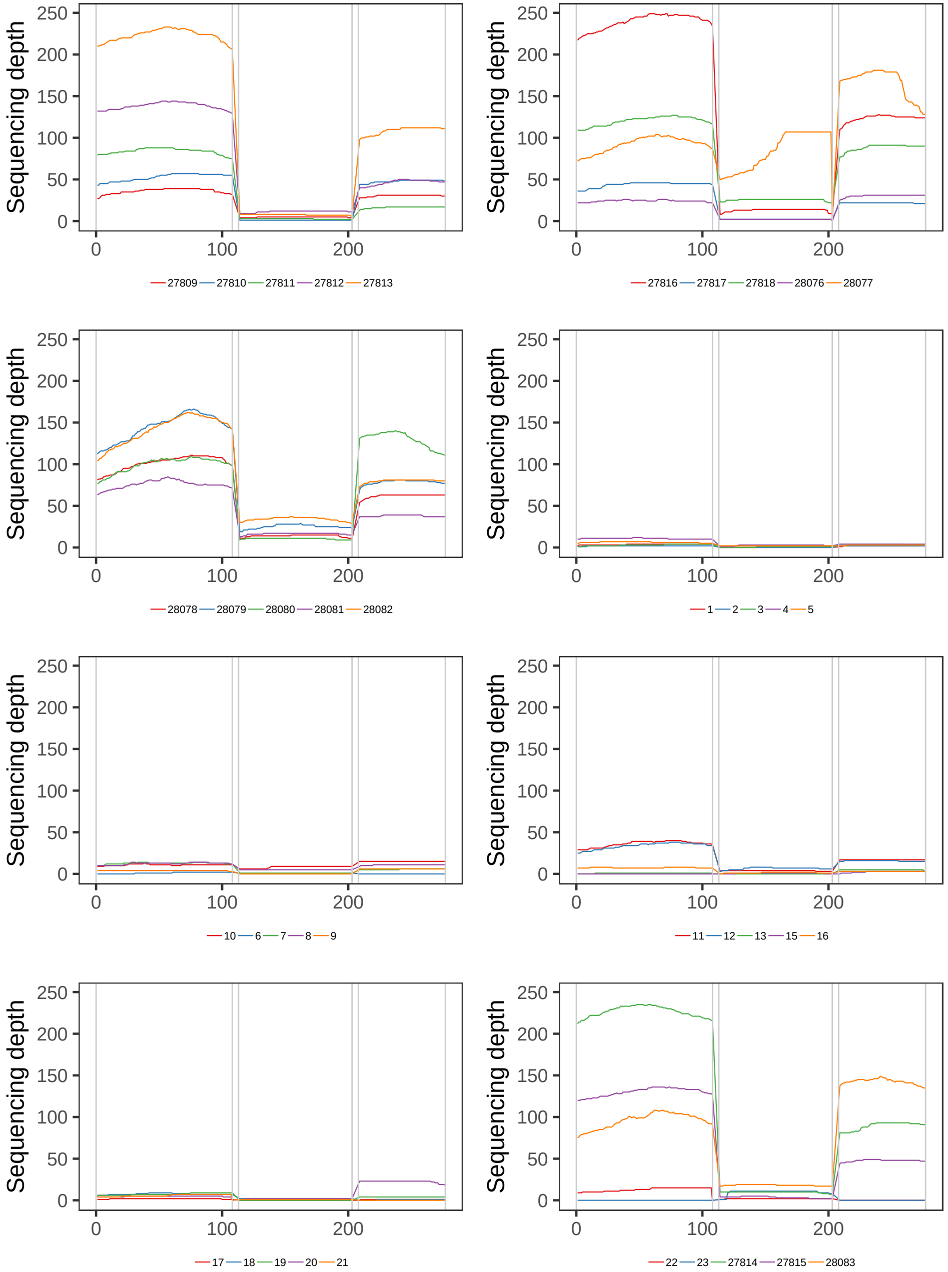
Sequencing depth
0
50
100
150
200
250
0
100
200
27809 27810 27811 27812 27813
27816 27817 27818 28076 28077
28078 28079 28080 28081 28082
1 2 3 4 5
10 6 7 8 9
11 12 13 15 16
17 18 19 20 21
22 23 27814 27815 28083

EOG5MCVFG

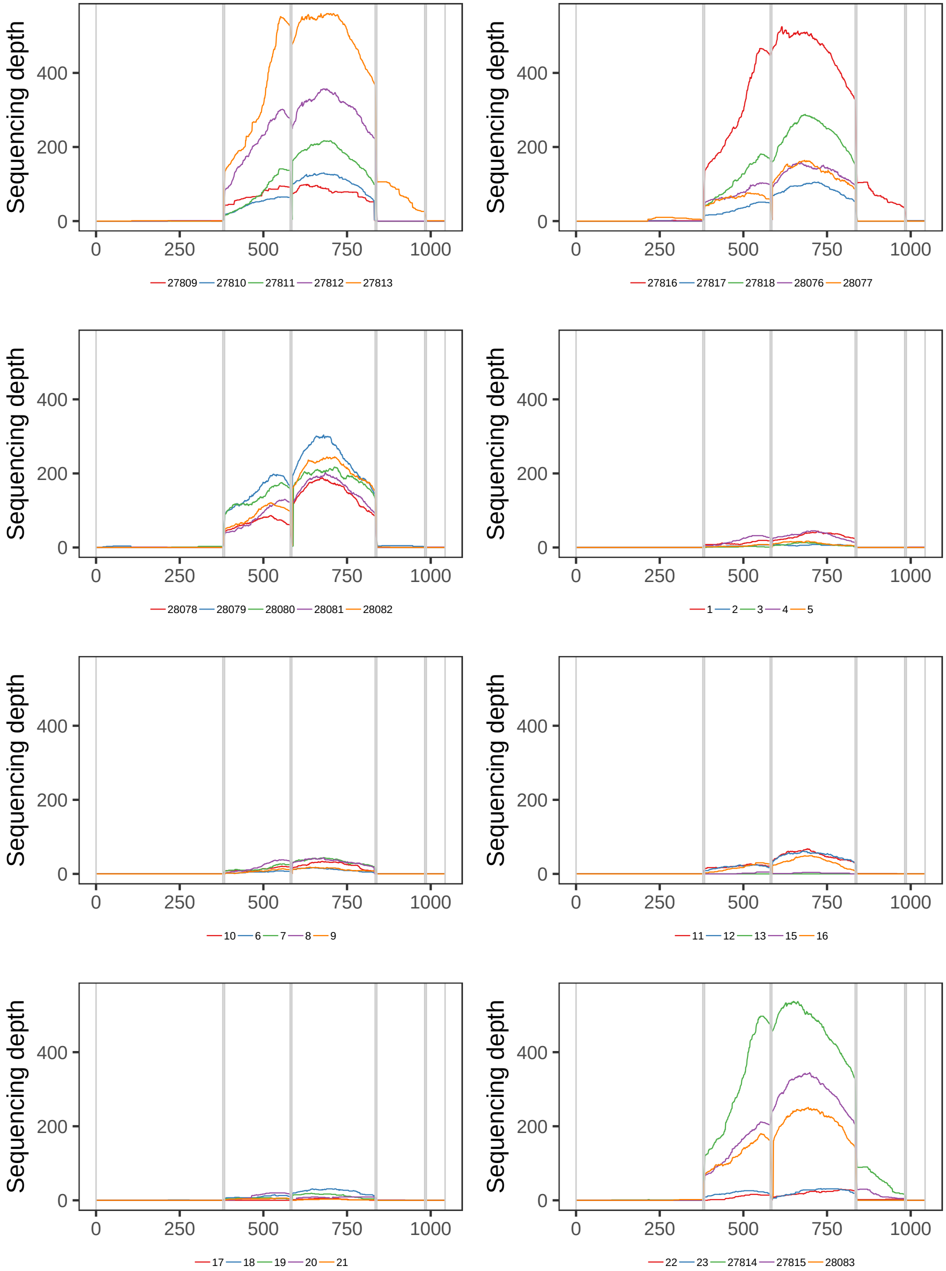

Sequencing depth
0
200
400
0
250
500
750
1000
27809
27810
27811
27812
27813
27816
27817
27818
28076
28077
28078
28079
28080
28081
28082
1
2
3
4
5
10
6
7
8
9
11
12
13
15
16
17
18
19
20
21
22
23
27814
27815
28083

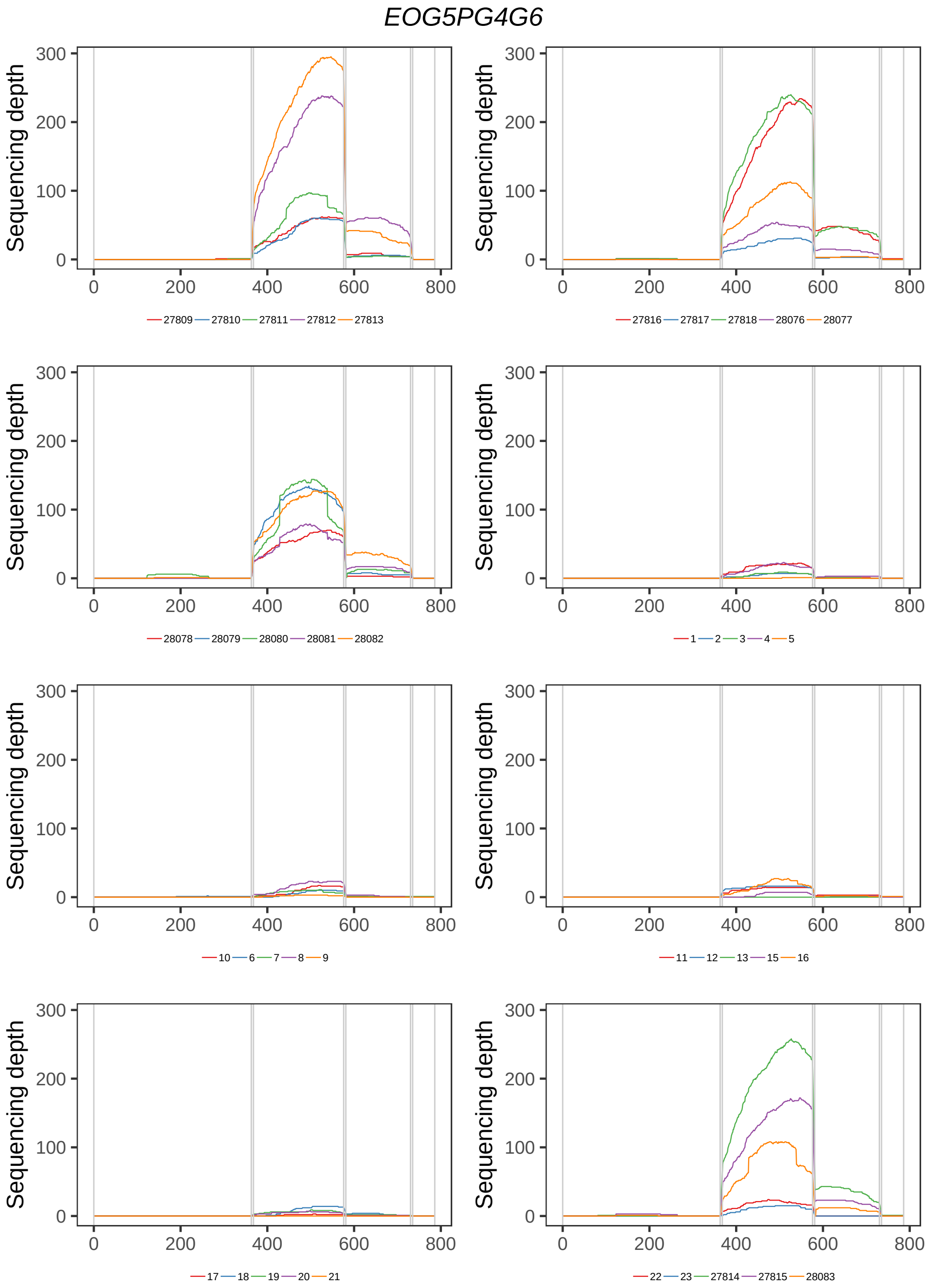
EOG5PG4G6
Sequencing depth
0
100
200
300
0
200
400
600
800
27809 27810 27811 27812 27813
27816 27817 27818 28076 28077
28078 28079 28080 28081 28082
1 2 3 4 5
10 6 7 8 9
11 12 13 15 16
17 18 19 20 21
22 23 27814 27815 28083

EOG5W6MBV

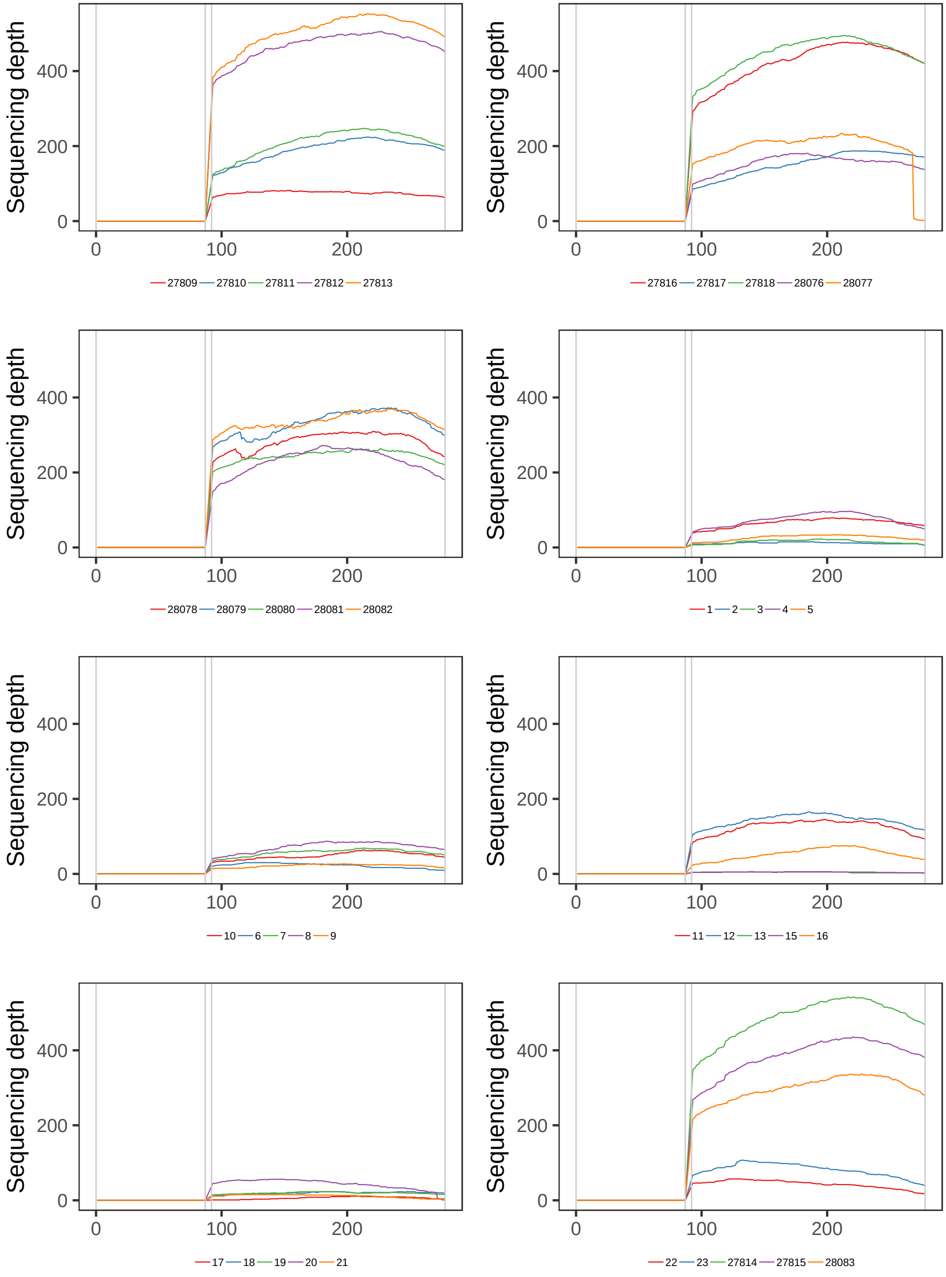

Sequencing depth
0
200
400
0
100
200
27809
27810
27811
27812
27813
27816
27817
27818
28076
28077
28078
28079
28080
28081
28082
1
2
3
4
5
10
6
7
8
9
11
12
13
15
16
17
18
19
20
21
22
23
27814
27815
28083

EOG5WPZK0

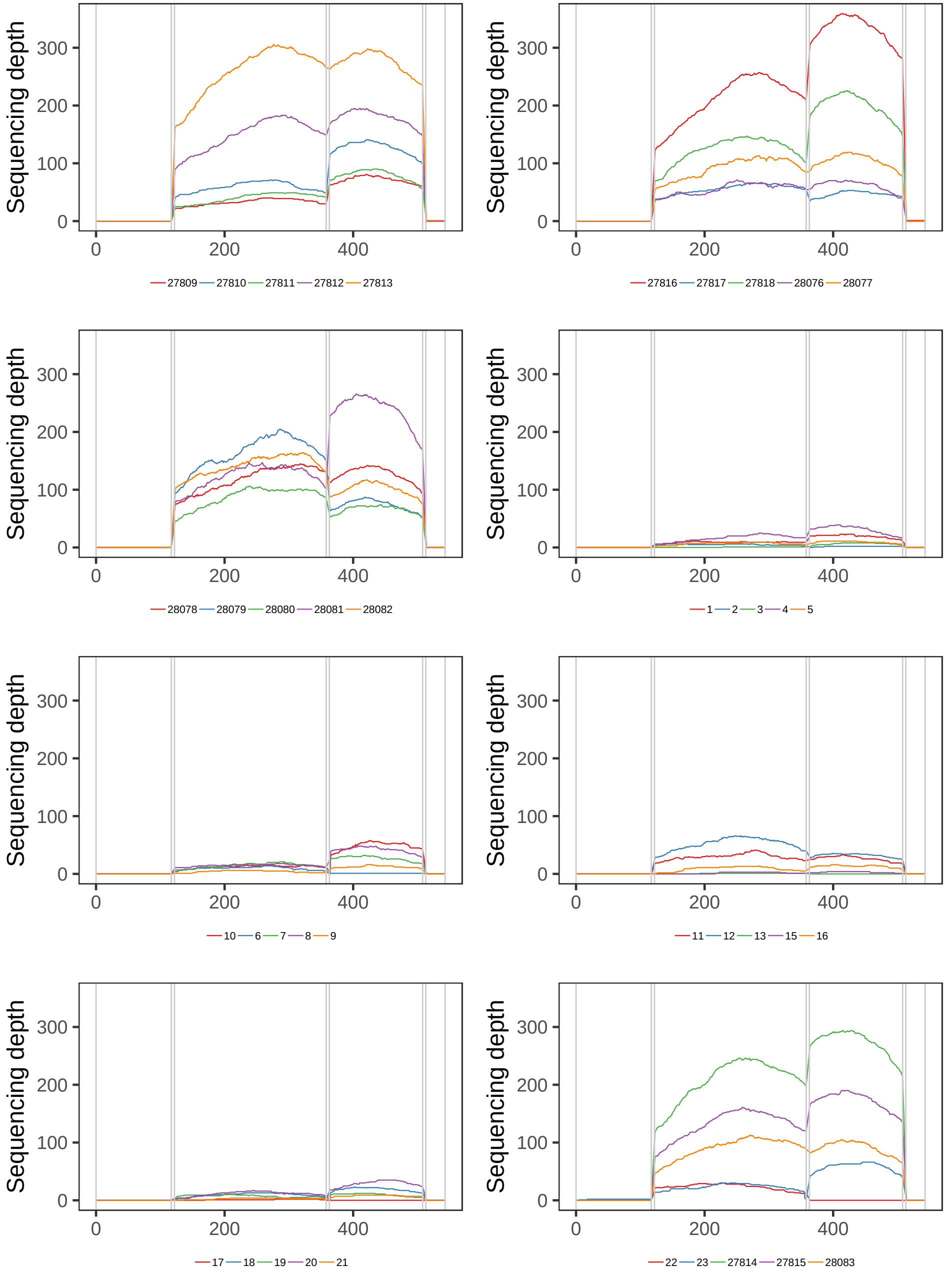

Sequencing depth
0
100
200
300
0
200
400
27809 27810 27811 27812 27813
27816 27817 27818 28076 28077
28078 28079 28080 28081 28082
1 2 3 4 5
10 6 7 8 9
11 12 13 15 16
17 18 19 20 21
22 23 27814 27815 28083

EOG5ZKH31

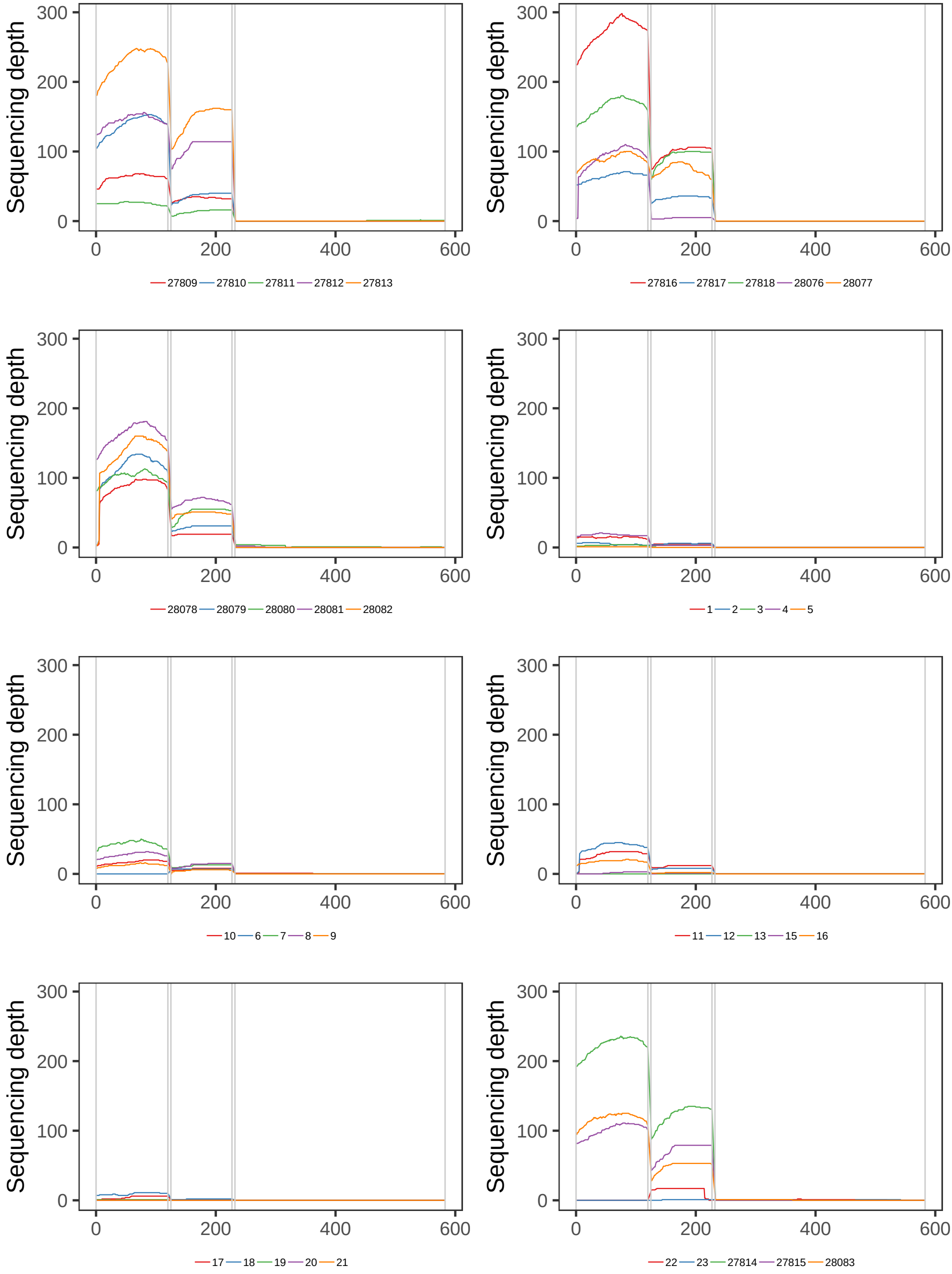
Sequencing depth
0
100
200
300
0
200
400
600
27809 27810 27811 27812 27813
27816 27817 27818 28076 28077
28078 28079 28080 28081 28082
1 2 3 4 5
10 6 7 8 9
11 12 13 15 16
17 18 19 20 21
22 23 27814 27815 28083

EOG5ZW3T3

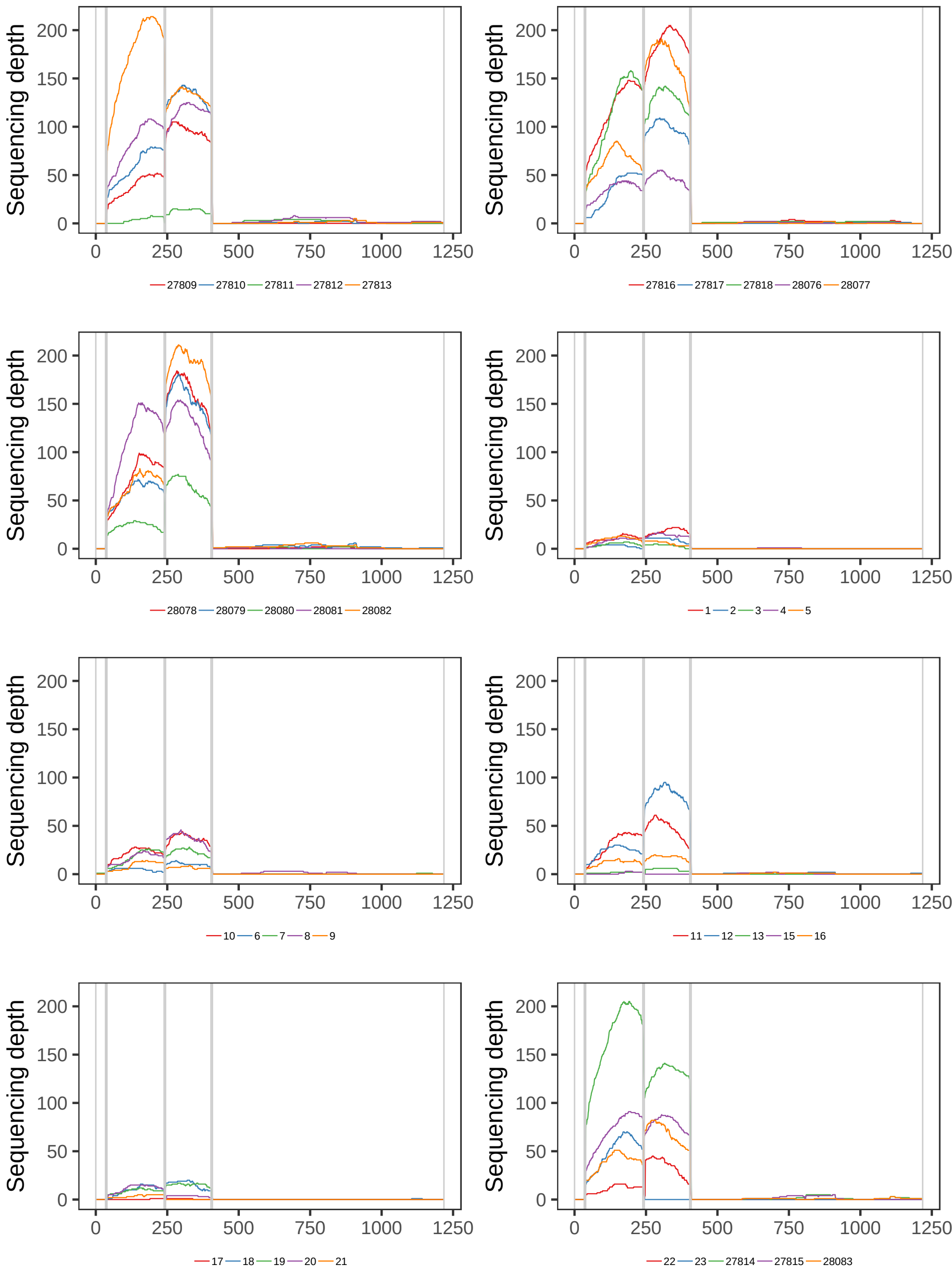
Sequencing depth
0
50
100
150
200
0
250
500
750
1000
1250
27809 27810 27811 27812 27813
27816 27817 27818 28076 28077
28078 28079 28080 28081 28082
1 2 3 4 5
10 6 7 8 9
11 12 13 15 16
17 18 19 20 21
22 23 27814 27815 28083

EOG54J10Q

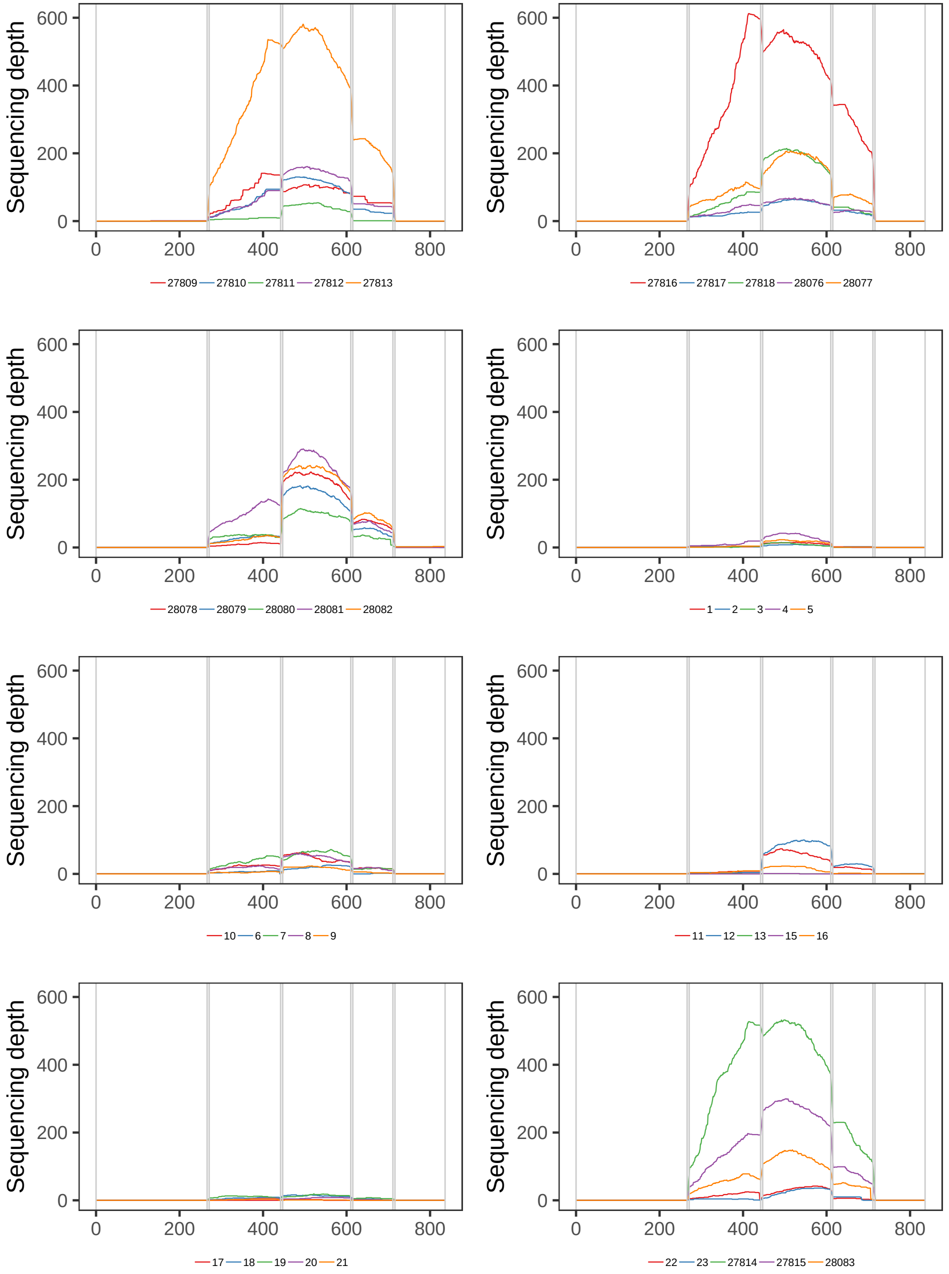

Sequencing depth
0
200
400
600
0
200
400
600
800
27809 27810 27811 27812 27813
27816 27817 27818 28076 28077
28078 28079 28080 28081 28082
1 2 3 4 5
10 6 7 8 9
11 12 13 15 16
17 18 19 20 21
22 23 27814 27815 28083

EOG54J110

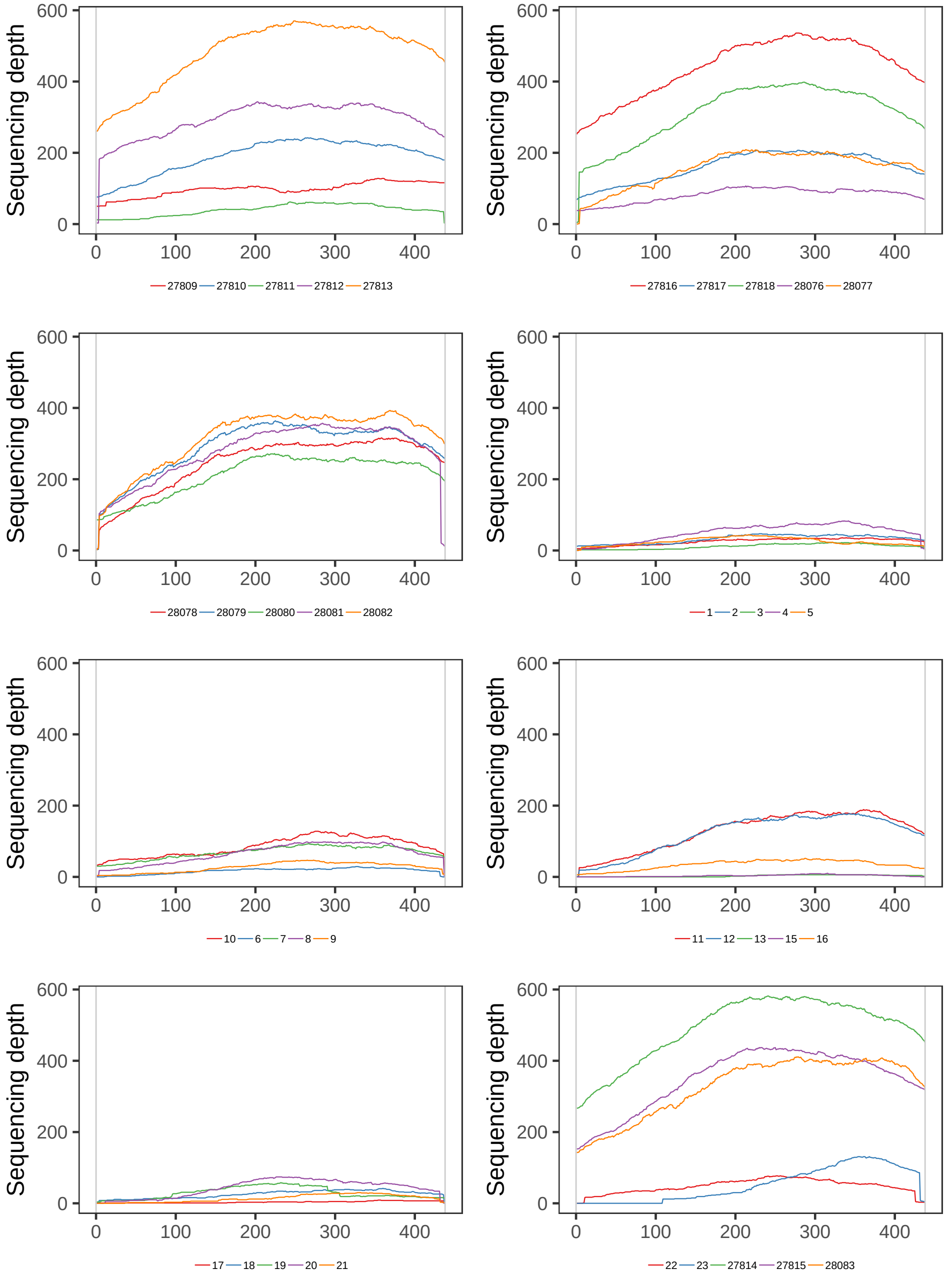
Sequencing depth
600
400
200
0
0
100
200
300
400
27809 27810 27811 27812 27813
27816 27817 27818 28076 28077
28078 28079 28080 28081 28082
1 2 3 4 5
10 6 7 8 9
11 12 13 15 16
17 18 19 20 21
22 23 27814 27815 28083

EOG54J11S

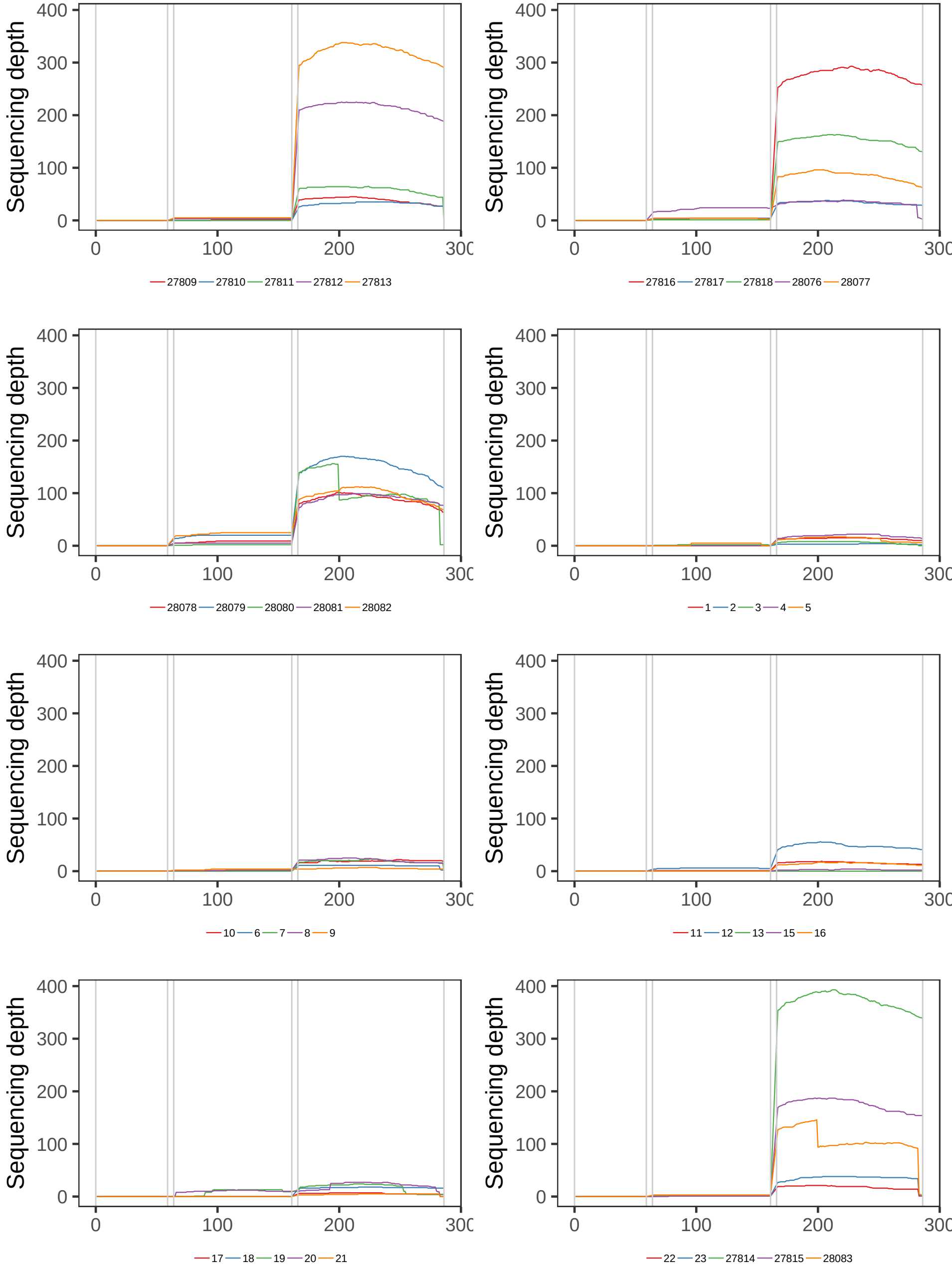
Sequencing depth
400
300
200
100
0
0
100
200
300
27809 27810 27811 27812 27813
27816 27817 27818 28076 28077
28078 28079 28080 28081 28082
1 2 3 4 5
10 6 7 8 9
11 12 13 15 16
17 18 19 20 21
22 23 27814 27815 28083

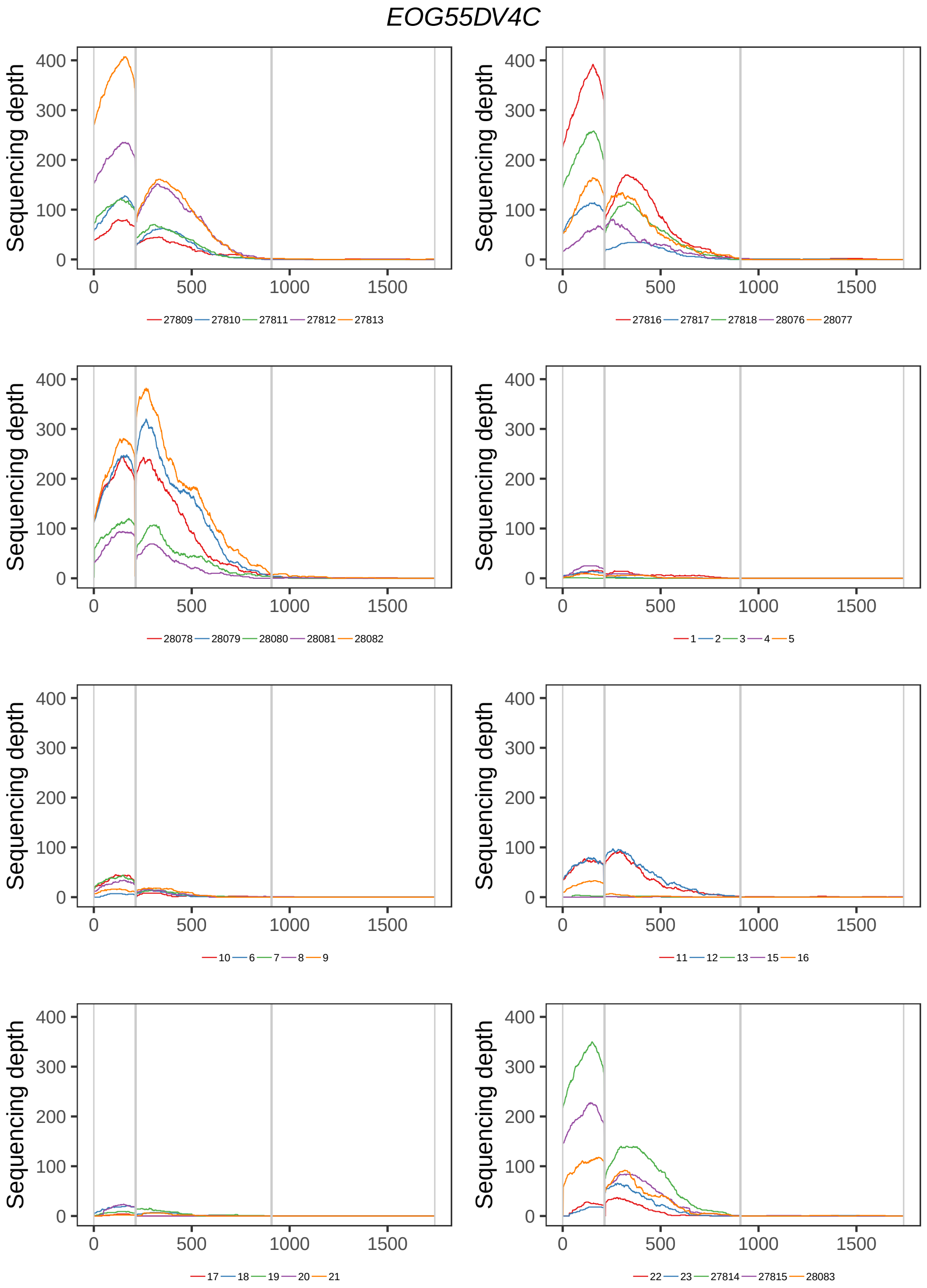

EOG55DV4C
Sequencing depth
27809 27810 27811 27812 27813
27816 27817 27818 28076 28077
28078 28079 28080 28081 28082
1 2 3 4 5
10 6 7 8 9
11 12 13 15 16
17 18 19 20 21
22 23 27814 27815 28083

EOG55DV4M

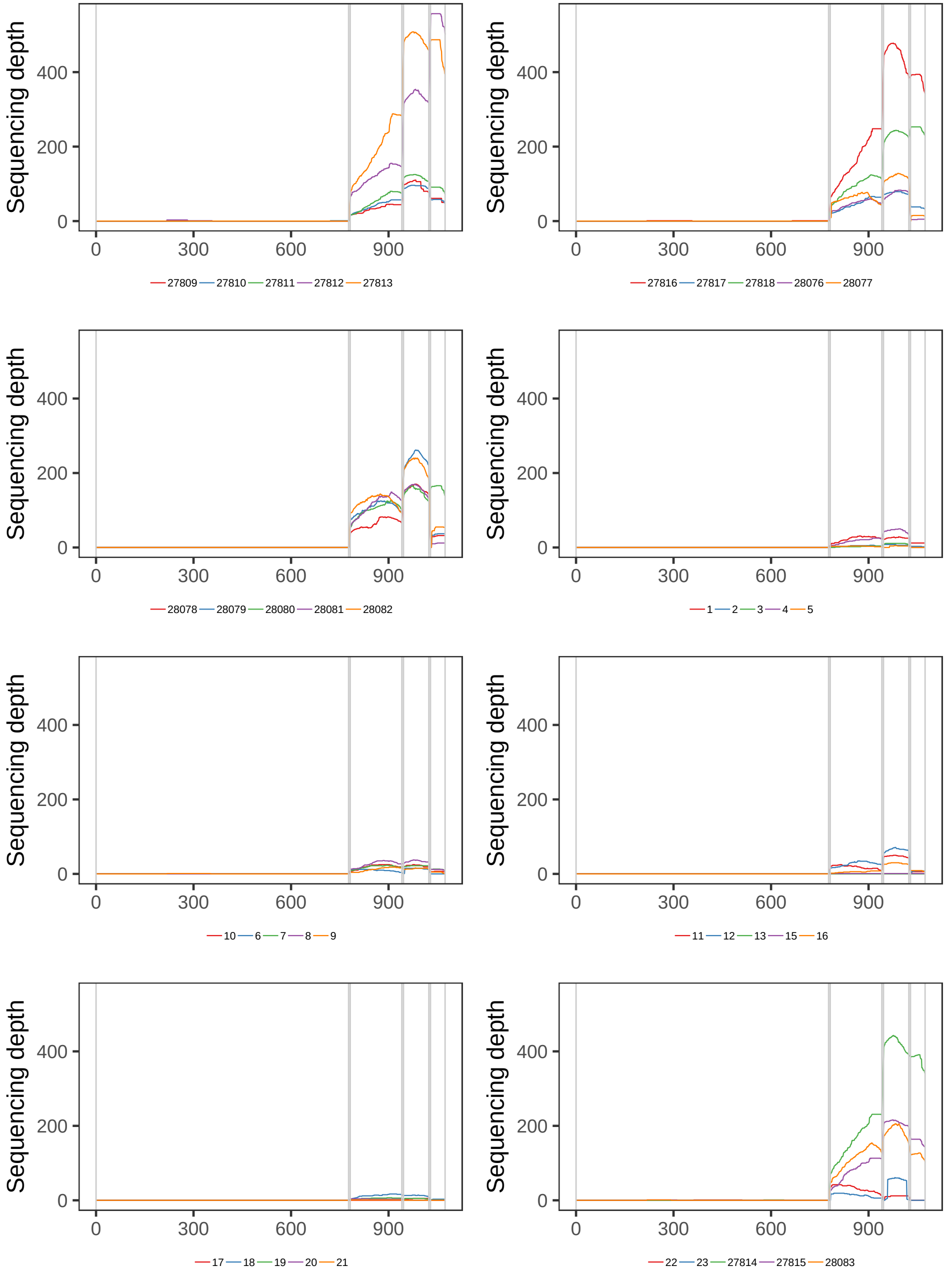
Sequencing depth
0
200
400
0
300
600
900
27809
27810
27811
27812
27813
27816
27817
27818
28076
28077
28078
28079
28080
28081
28082
1
2
3
4
5
10
6
7
8
9
11
12
13
15
16
17
18
19
20
21
22
23
27814
27815
28083

EOG57H45W

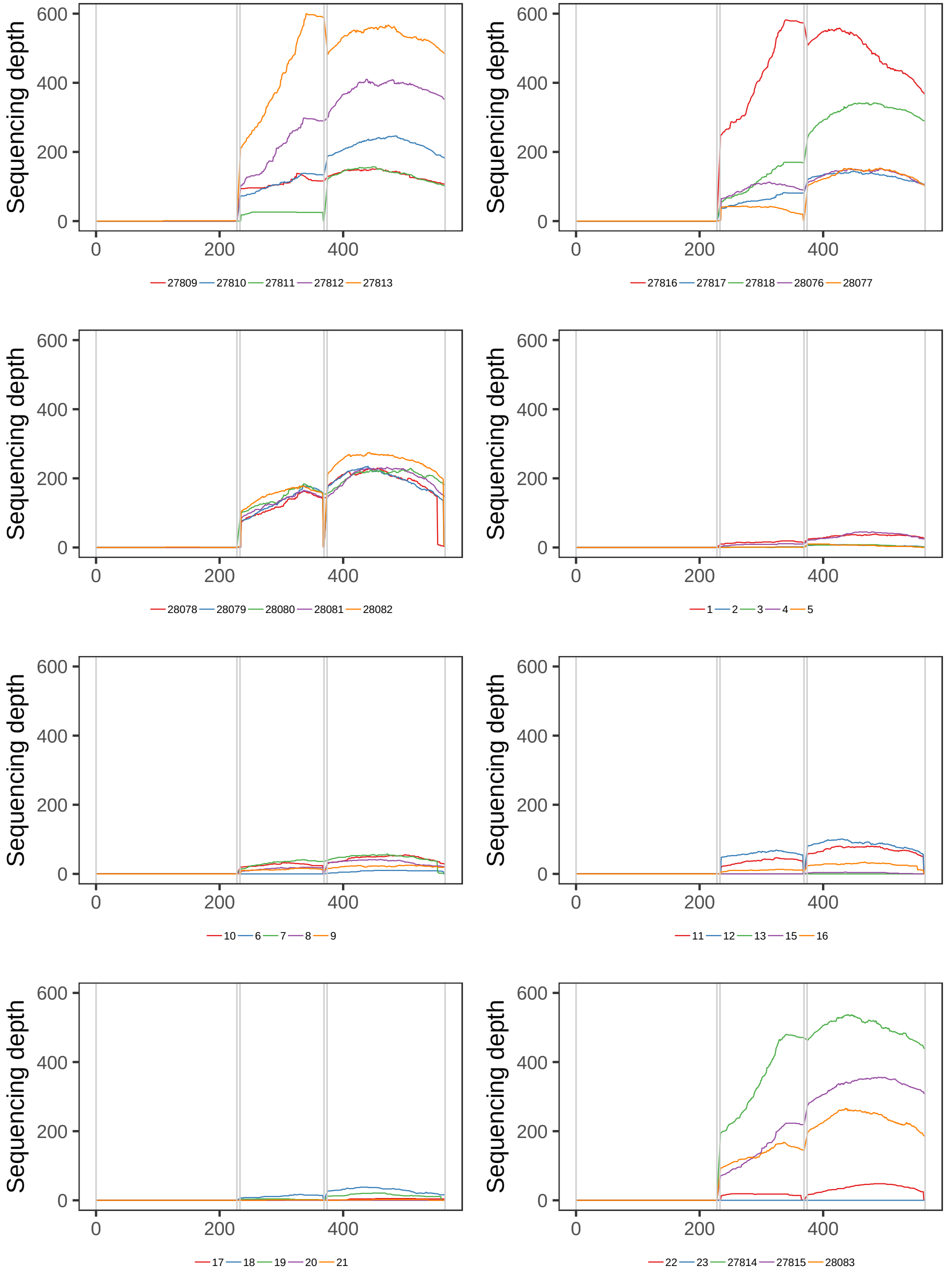

Sequencing depth
600
400
200
0
0
200
400
27809
27810
27811
27812
27813
27816
27817
27818
28076
28077
28078
28079
28080
28081
28082
1
2
3
4
5
10
6
7
8
9
11
12
13
15
16
17
18
19
20
21
22
23
27814
27815
28083

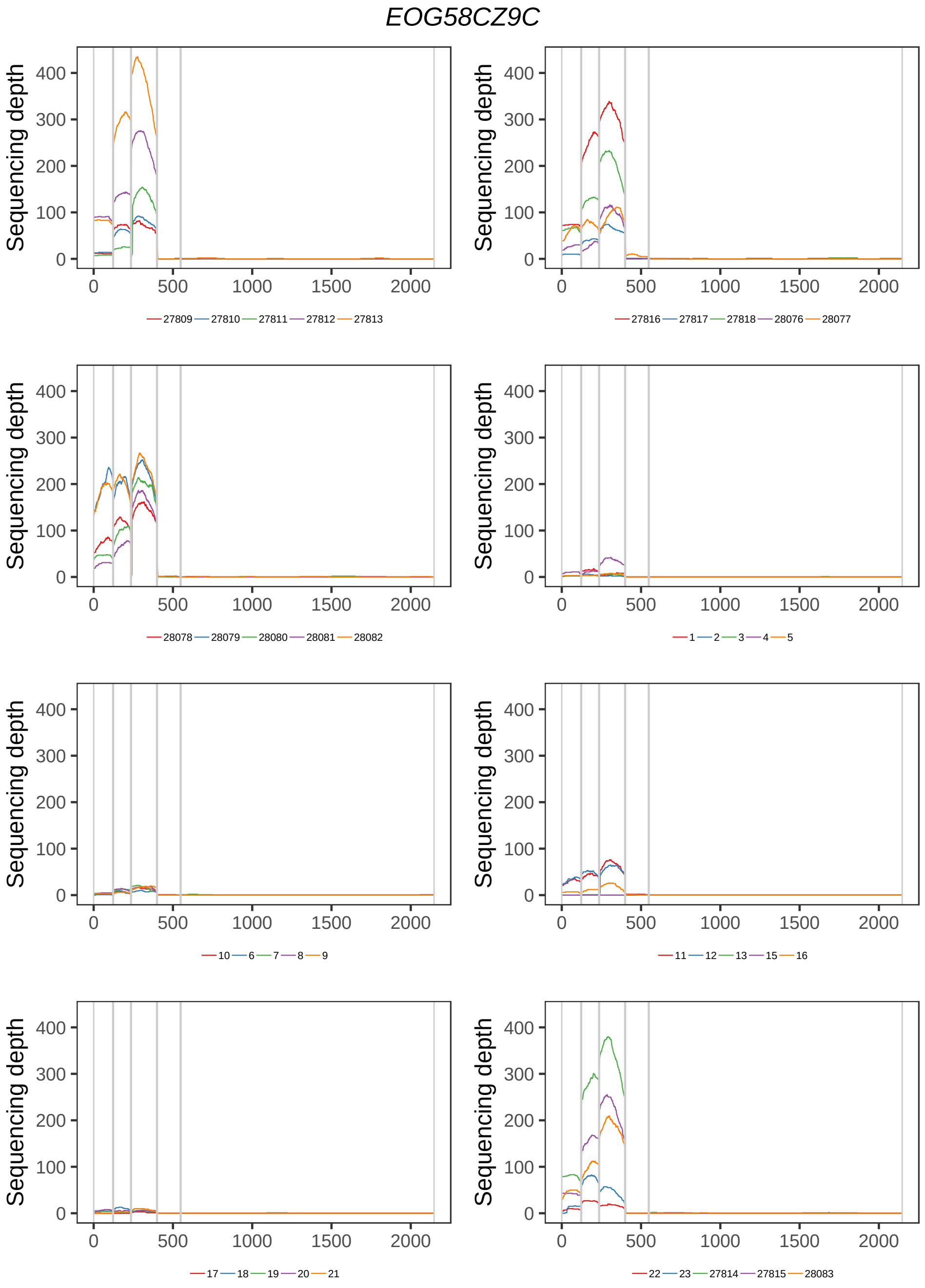
EOG58CZ9C
Sequencing depth
0
100
200
300
400
500
1000
1500
2000
27809 27810 27811 27812 27813
27816 27817 27818 28076 28077
28078 28079 28080 28081 28082
1 2 3 4 5
10 6 7 8 9
11 12 13 15 16
17 18 19 20 21
22 23 27814 27815 28083

EOG58W9HW

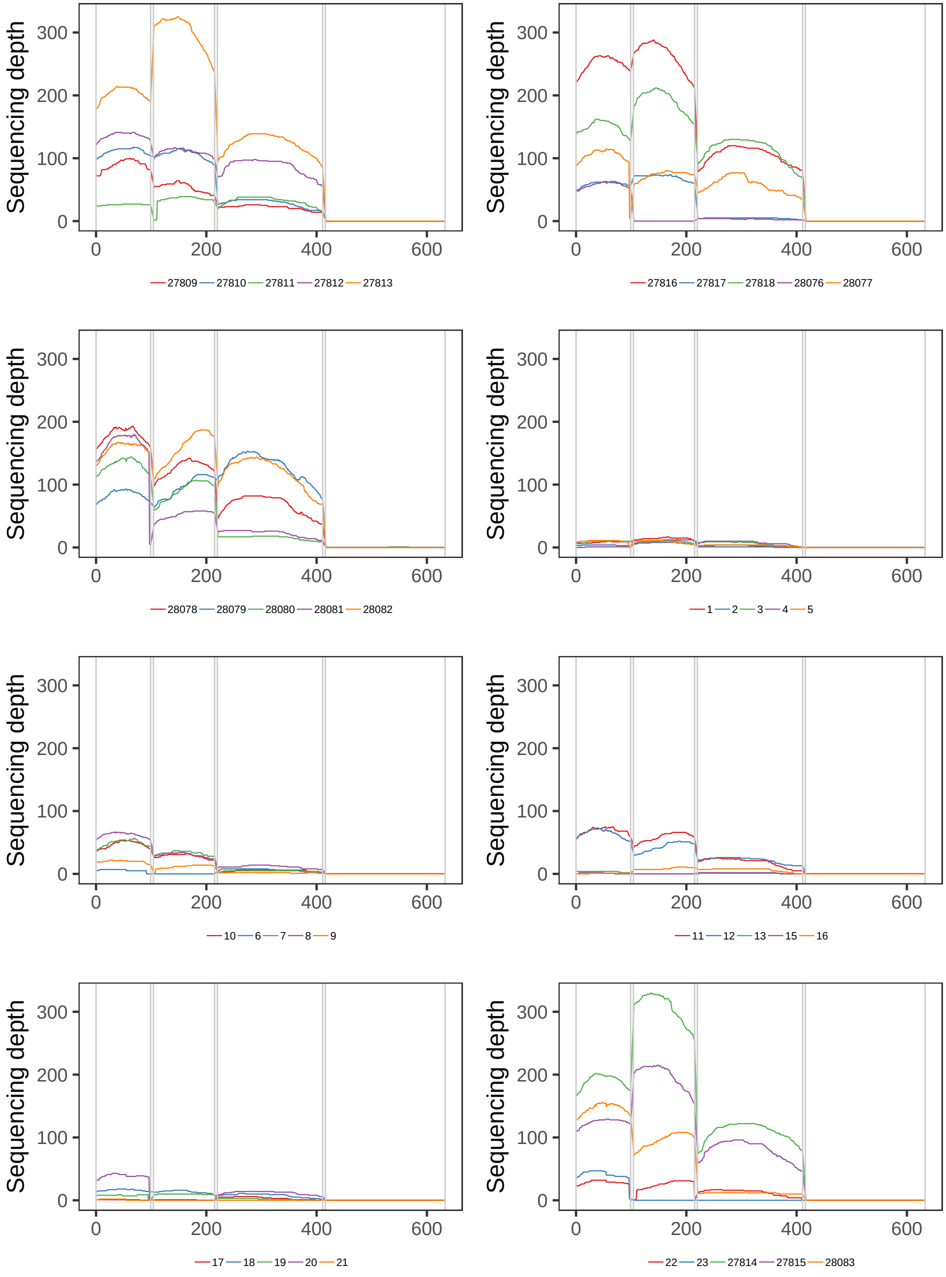

Sequencing depth
0
100
200
300
0
200
400
600
27809
27810
27811
27812
27813
27816
27817
27818
28076
28077
28078
28079
28080
28081
28082
1
2
3
4
5
10
6
7
8
9
11
12
13
15
16
17
18
19
20
21
22
23
27814
27815
28083

EOG5905SN

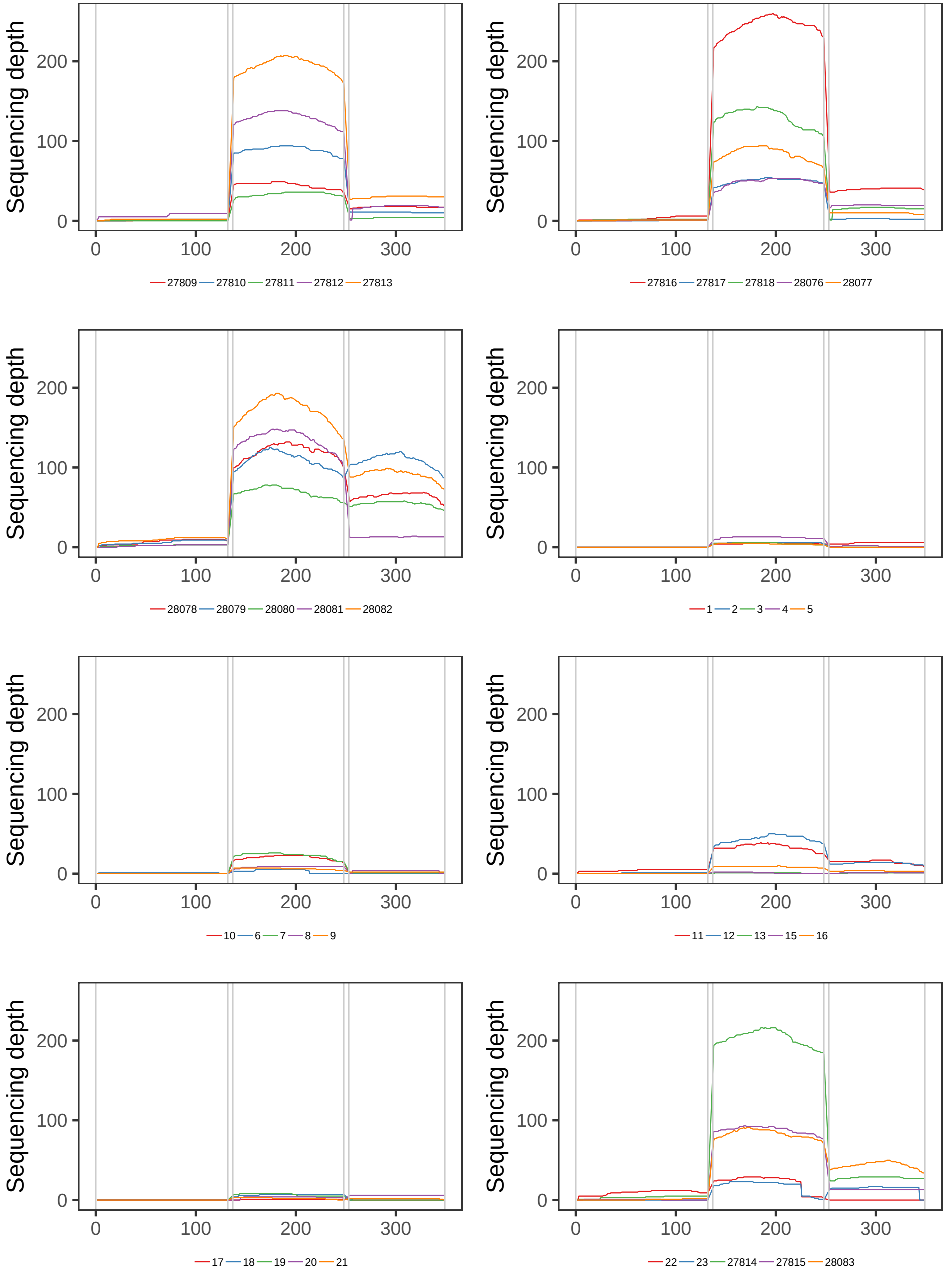
Sequencing depth
0
100
200
300
27809
27810
27811
27812
27813
27816
27817
27818
28076
28077
28078
28079
28080
28081
28082
1
2
3
4
5
10
6
7
8
9
11
12
13
15
16
17
18
19
20
21
22
23
27814
27815
28083

EOG5BG7B9

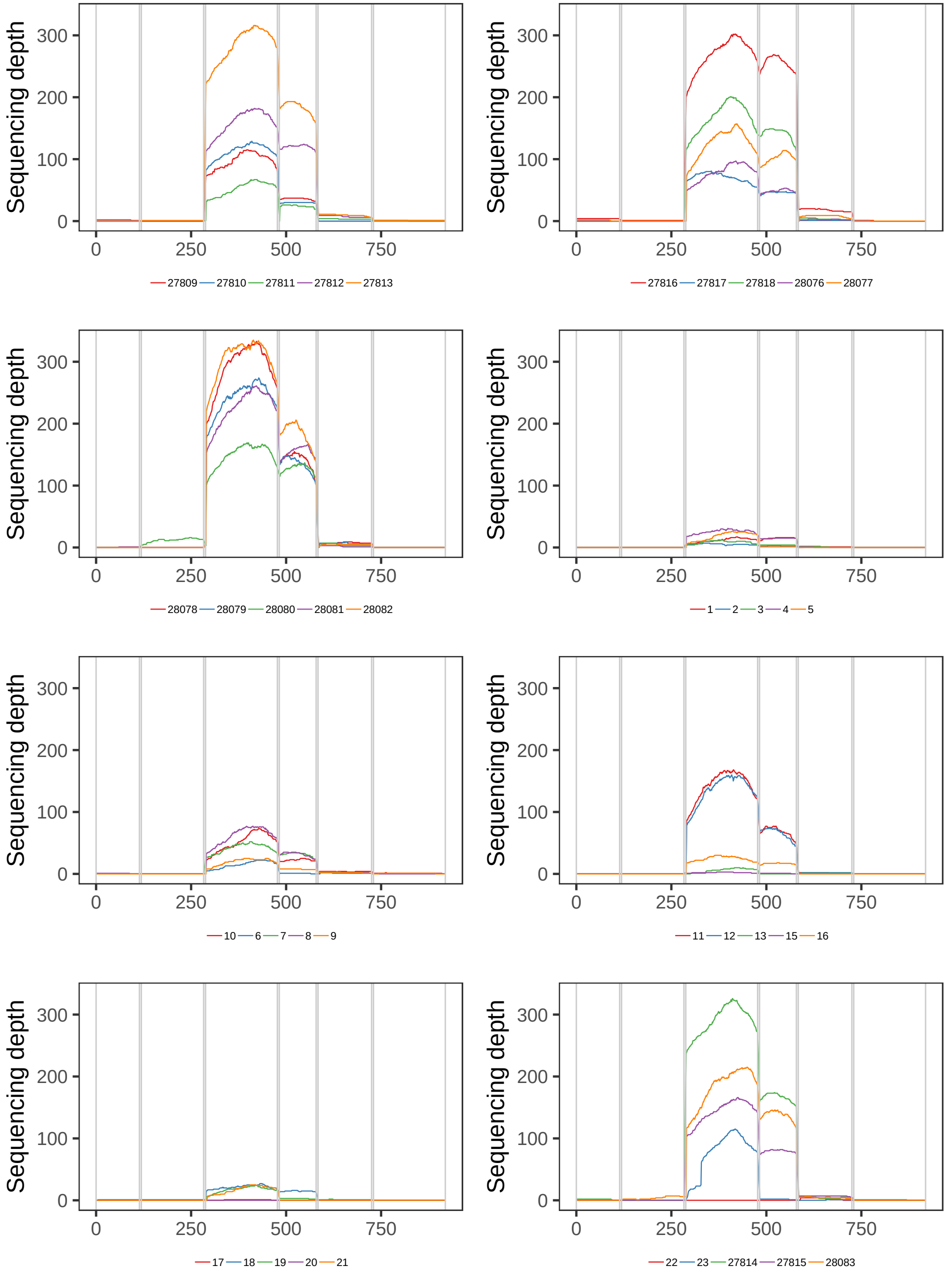
Sequencing depth
0
100
200
300
0
250
500
750
27809 27810 27811 27812 27813
27816 27817 27818 28076 28077
28078 28079 28080 28081 28082
1 2 3 4 5
10 6 7 8 9
11 12 13 15 16
17 18 19 20 21
22 23 27814 27815 28083

EOG5BZKHV

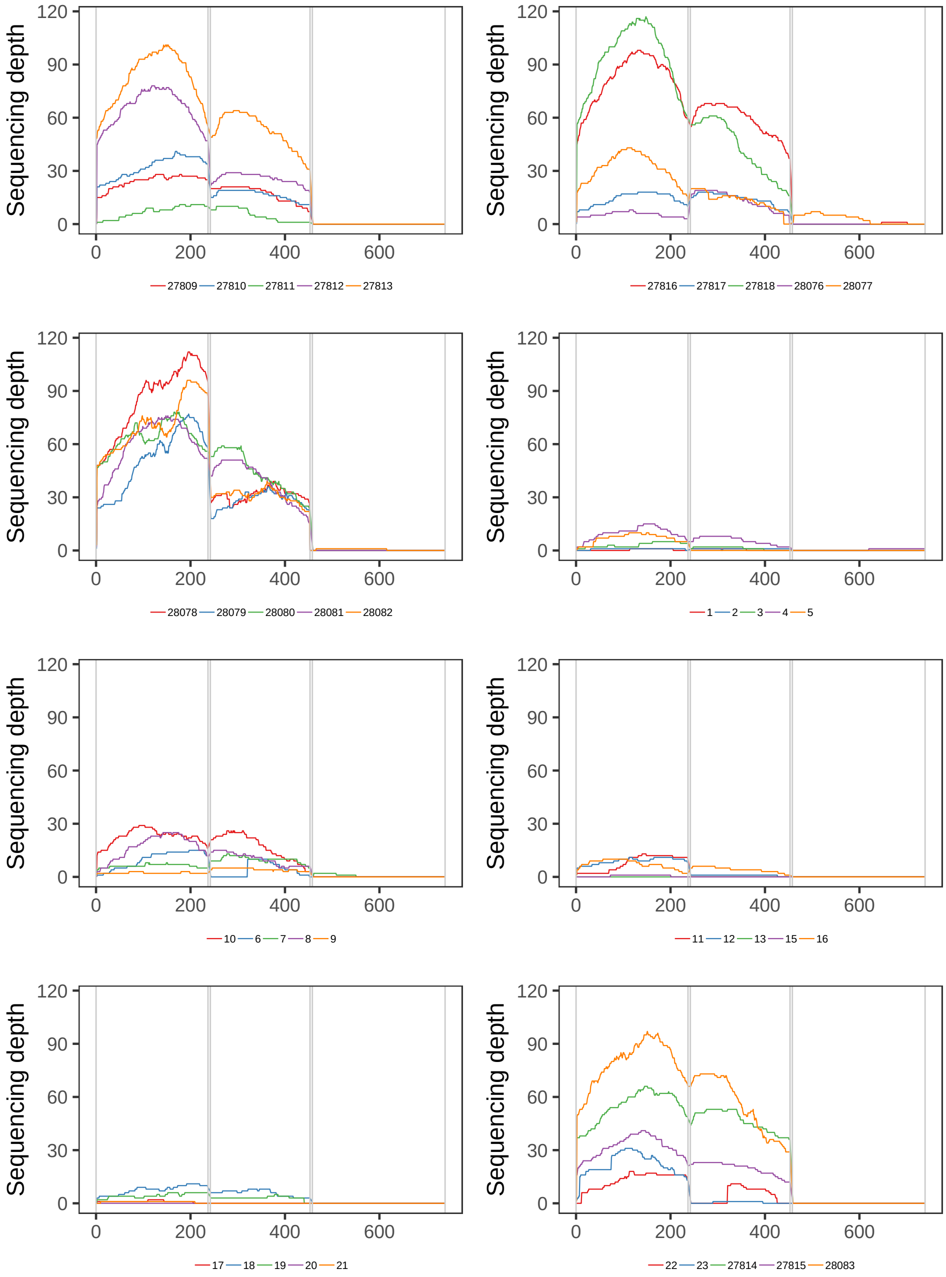
Sequencing depth
120
90
60
30
0
0
200
400
600
27809 27810 27811 27812 27813
27816 27817 27818 28076 28077
28078 28079 28080 28081 28082
1 2 3 4 5
10 6 7 8 9
11 12 13 15 16
17 18 19 20 21
22 23 27814 27815 28083

EOG5C5B12

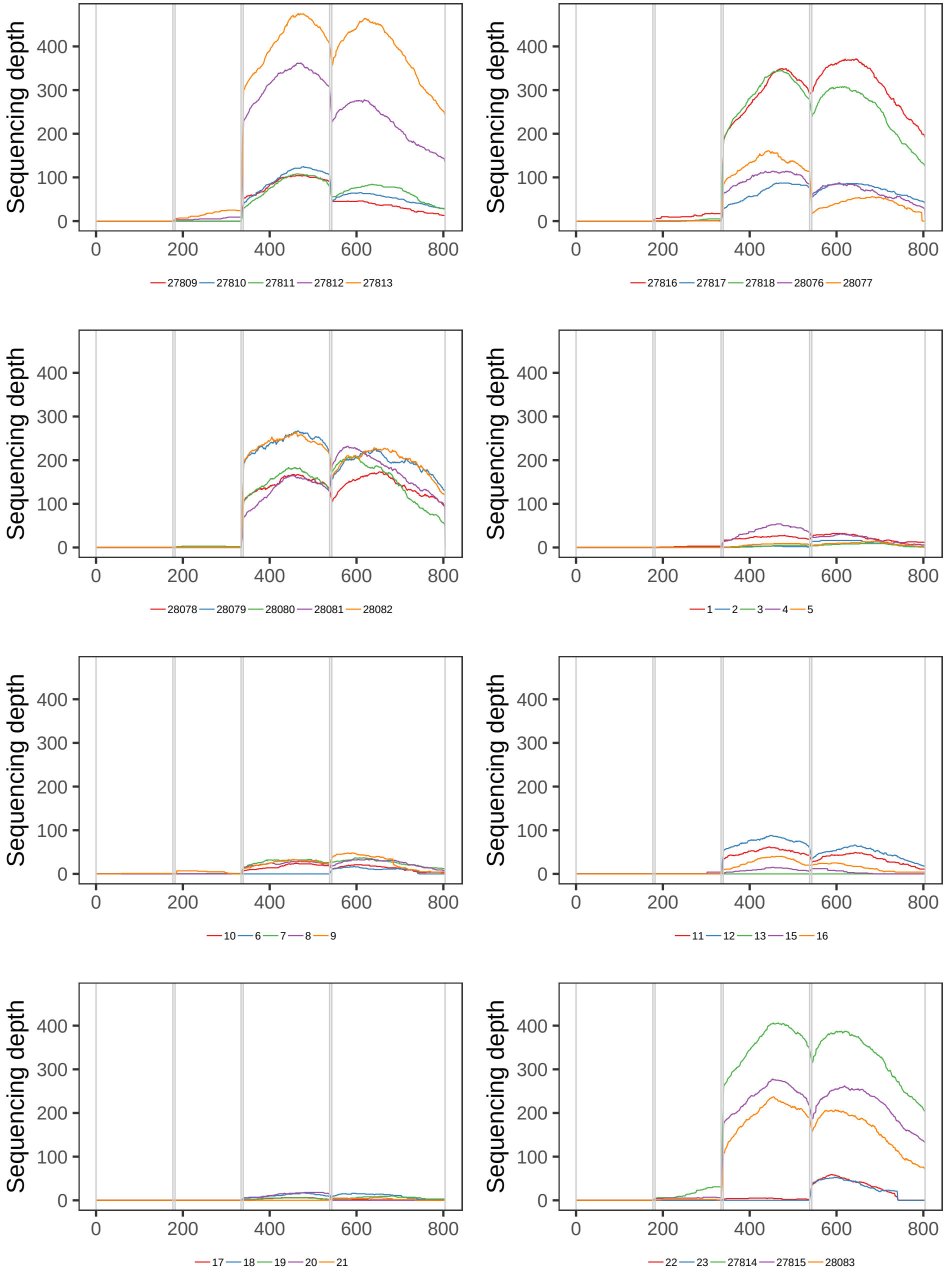
Sequencing depth
0
100
200
300
400
0
200
400
600
800
27809
27810
27811
27812
27813
27816
27817
27818
28076
28077
28078
28079
28080
28081
28082
1
2
3
4
5
10
6
7
8
9
11
12
13
15
16
17
18
19
20
21
22
23
27814
27815
28083

EOG5CFXQ8

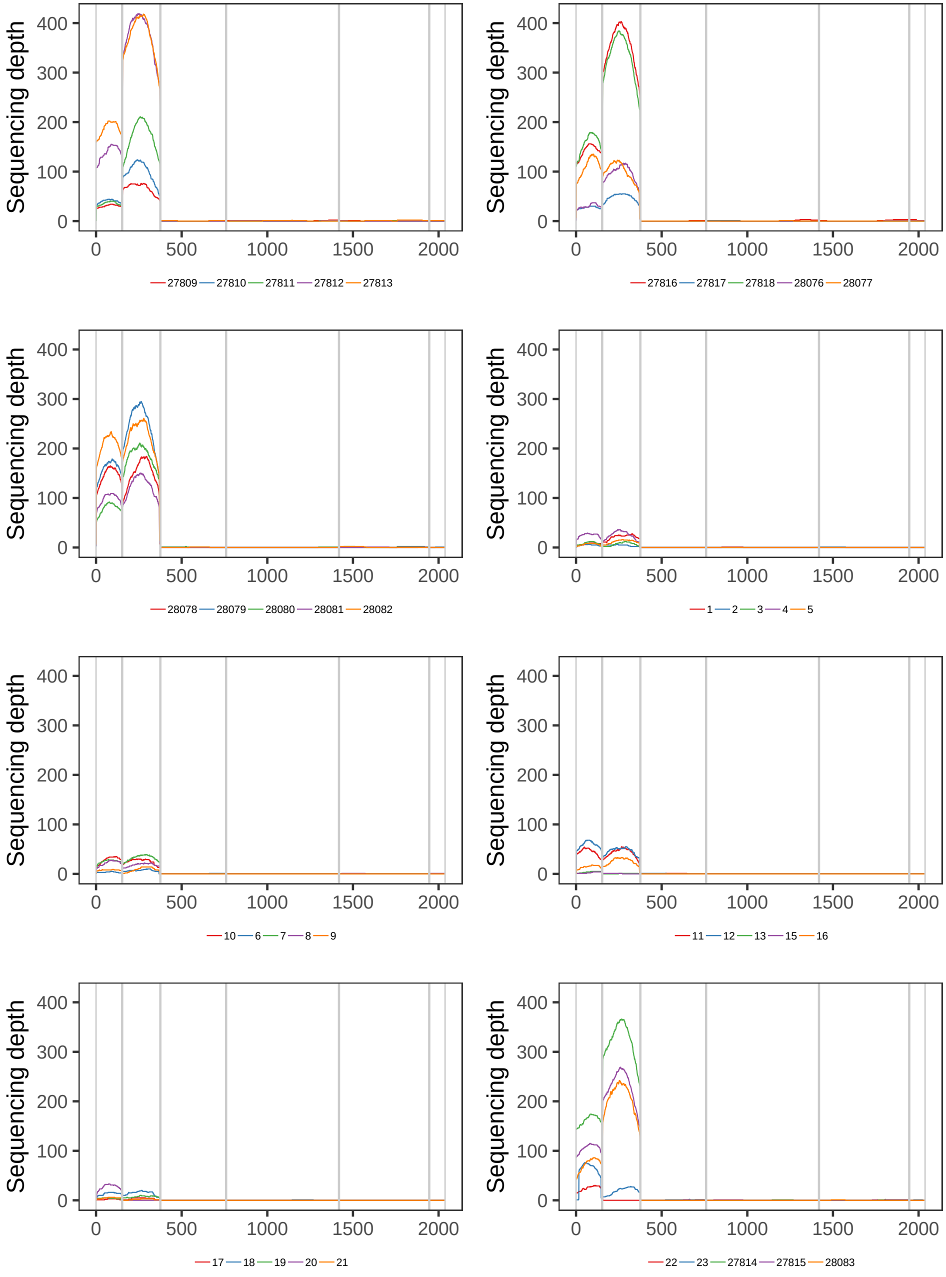
Sequencing depth
0
100
200
300
400
0
500
1000
1500
2000
27809 27810 27811 27812 27813
27816 27817 27818 28076 28077
28078 28079 28080 28081 28082
1 2 3 4 5
10 6 7 8 9
11 12 13 15 16
17 18 19 20 21
22 23 27814 27815 28083

EOG5DR7TC

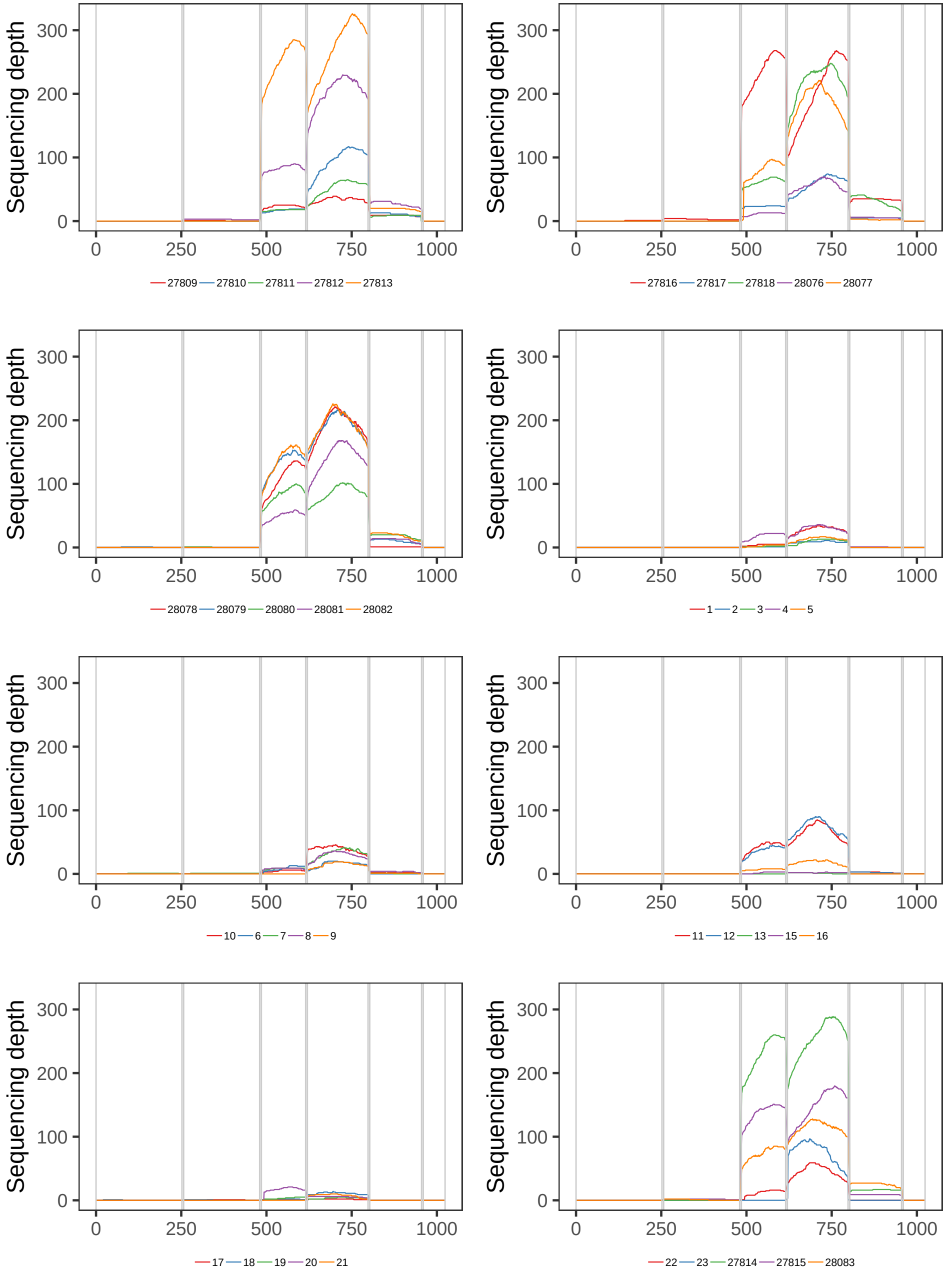

Sequencing depth
300
200
100
0
0
250
500
750
1000
27809 27810 27811 27812 27813
27816 27817 27818 28076 28077
28078 28079 28080 28081 28082
1 2 3 4 5
10 6 7 8 9
11 12 13 15 16
17 18 19 20 21
22 23 27814 27815 28083

EOG5HQC0C

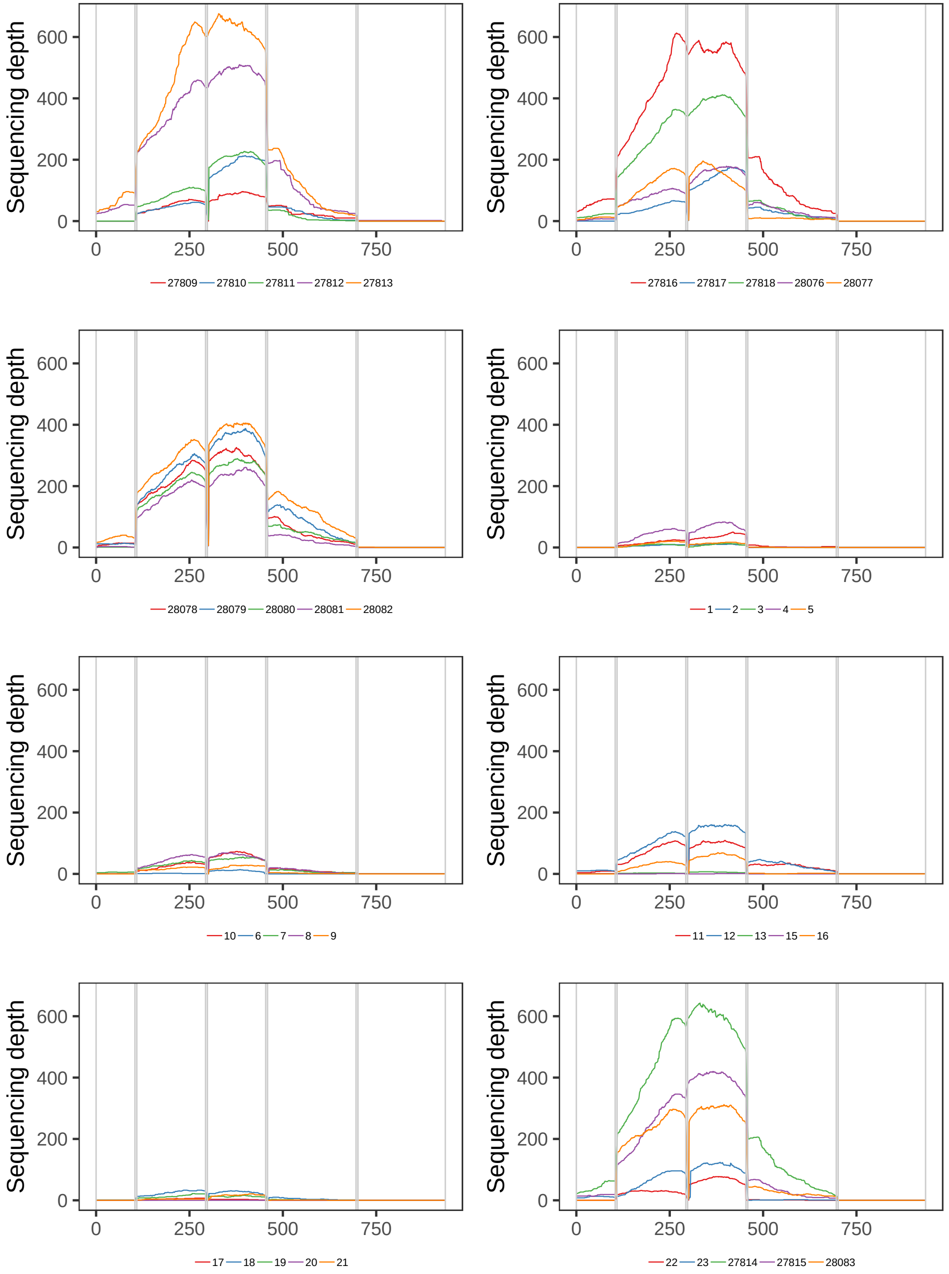
Sequencing depth
0
200
400
600
0
250
500
750
27809
27810
27811
27812
27813
27816
27817
27818
28076
28077
28078
28079
28080
28081
28082
1
2
3
4
5
10
6
7
8
9
11
12
13
15
16
17
18
19
20
21
22
23
27814
27815
28083

EOG5HT785

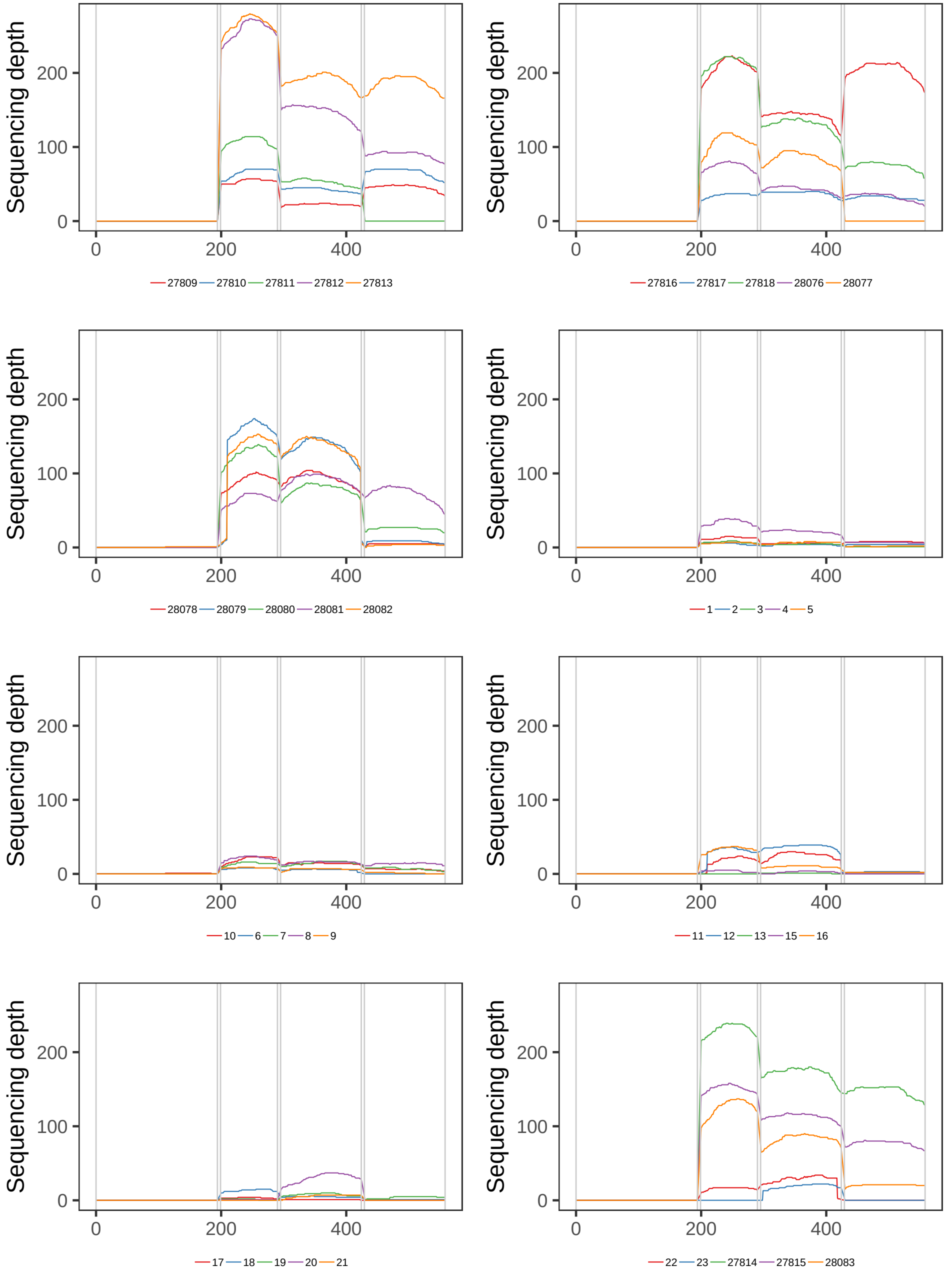

Sequencing depth
27809 27810 27811 27812 27813
27816 27817 27818 28076 28077
28078 28079 28080 28081 28082
1 2 3 4 5
10 6 7 8 9
11 12 13 15 16
17 18 19 20 21
22 23 27814 27815 28083

EOG5M63ZZ

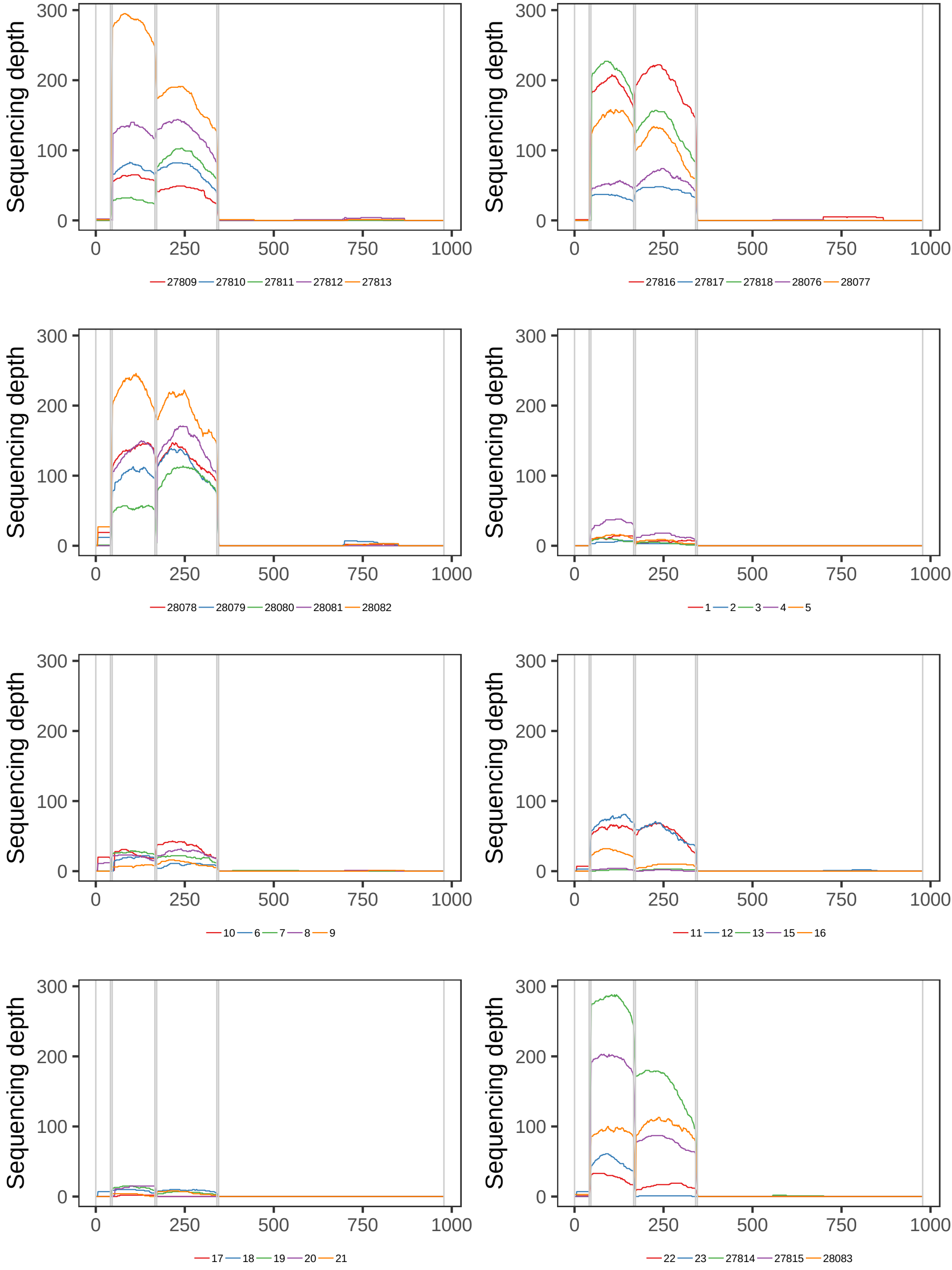

Sequencing depth
300
200
100
0
250
500
750
1000
27809 27810 27811 27812 27813
27816 27817 27818 28076 28077
28078 28079 28080 28081 28082
1 2 3 4 5
10 6 7 8 9
11 12 13 15 16
17 18 19 20 21
22 23 27814 27815 28083

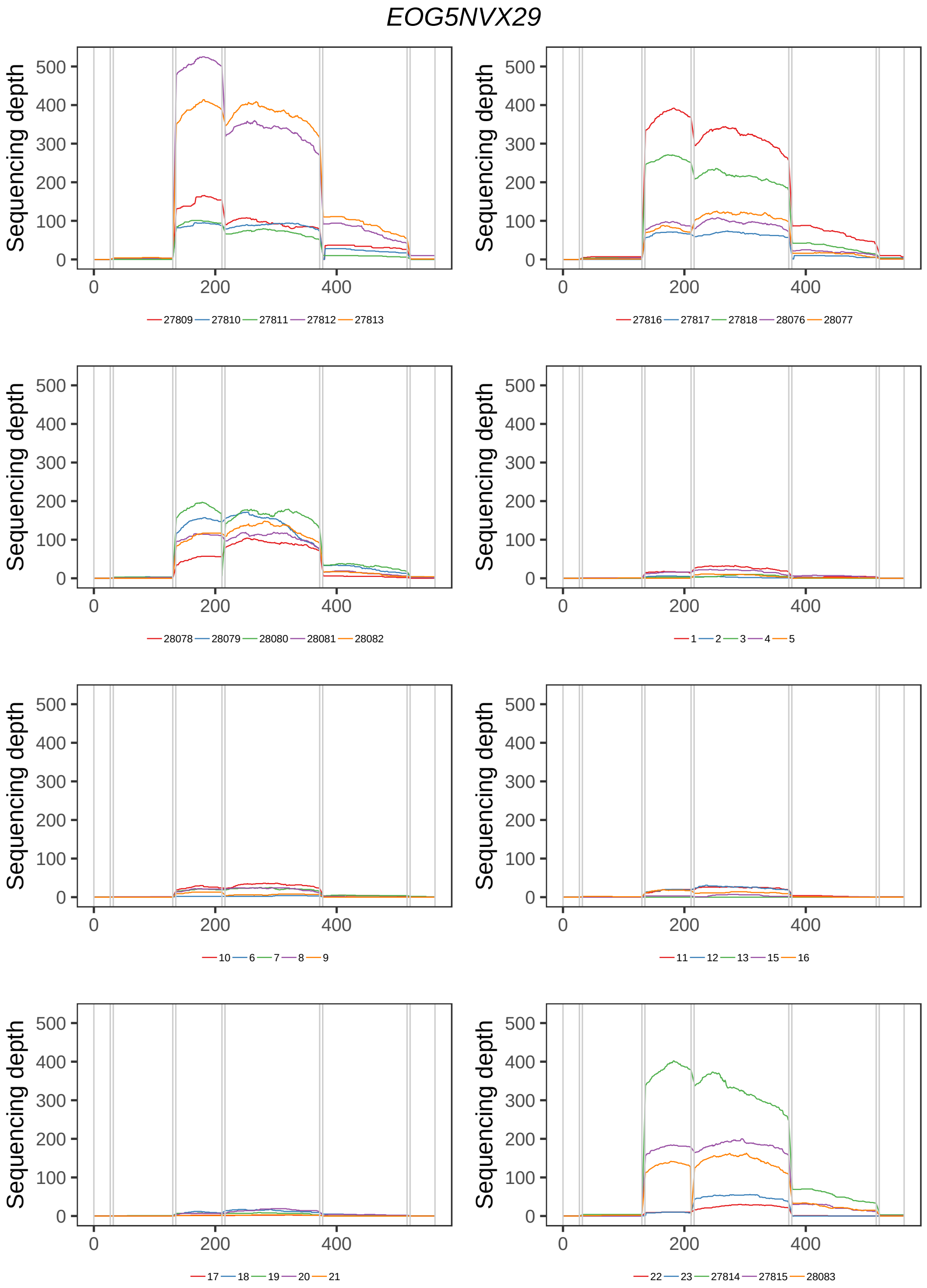
EOG5NVX29
Sequencing depth
0
100
200
300
400
500
0
200
400
27809
27810
27811
27812
27813
27816
27817
27818
28076
28077
28078
28079
28080
28081
28082
1
2
3
4
5
10
6
7
8
9
11
12
13
15
16
17
18
19
20
21
22
23
27814
27815
28083

EOG5Q574T

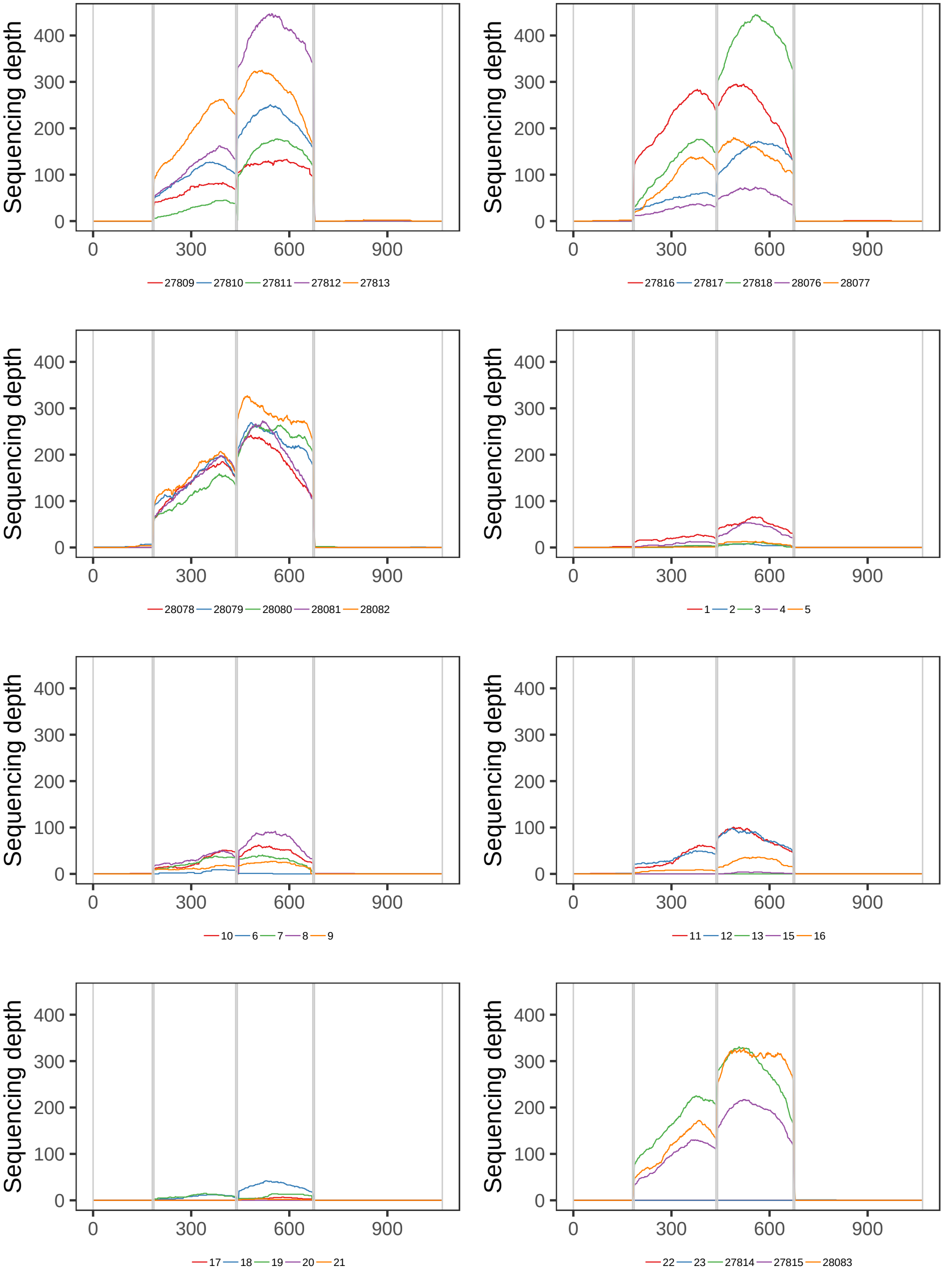
Sequencing depth
0
100
200
300
400
0
300
600
900
27809 27810 27811 27812 27813
27816 27817 27818 28076 28077
28078 28079 28080 28081 28082
1 2 3 4 5
10 6 7 8 9
11 12 13 15 16
17 18 19 20 21
22 23 27814 27815 28083

EOG5Q574V

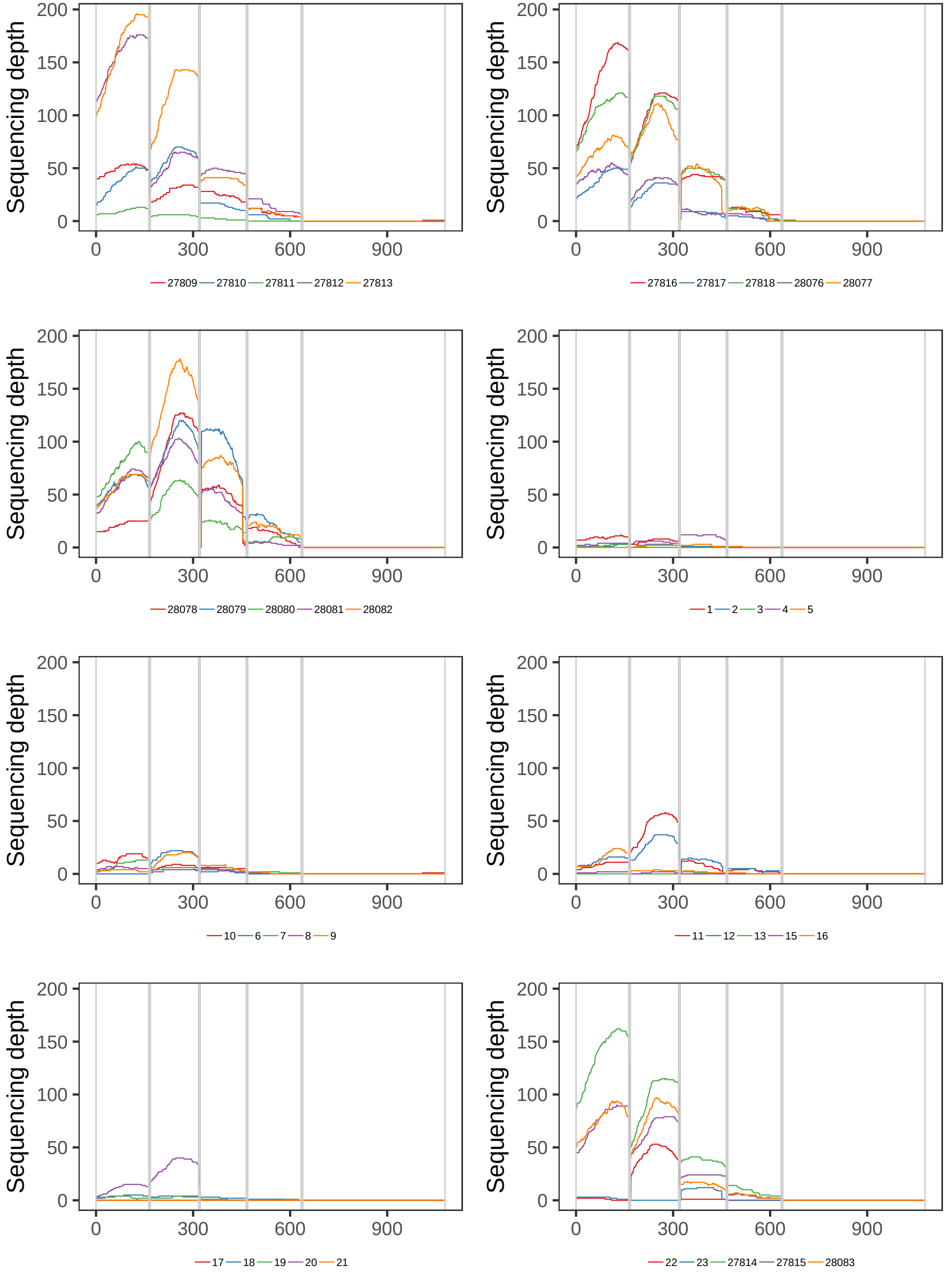
Sequencing depth
200
150
100
50
0
0
300
600
900
27809 27810 27811 27812 27813
27816 27817 27818 28076 28077
28078 28079 28080 28081 28082
1 2 3 4 5
10 6 7 8 9
11 12 13 15 16
17 18 19 20 21
22 23 27814 27815 28083

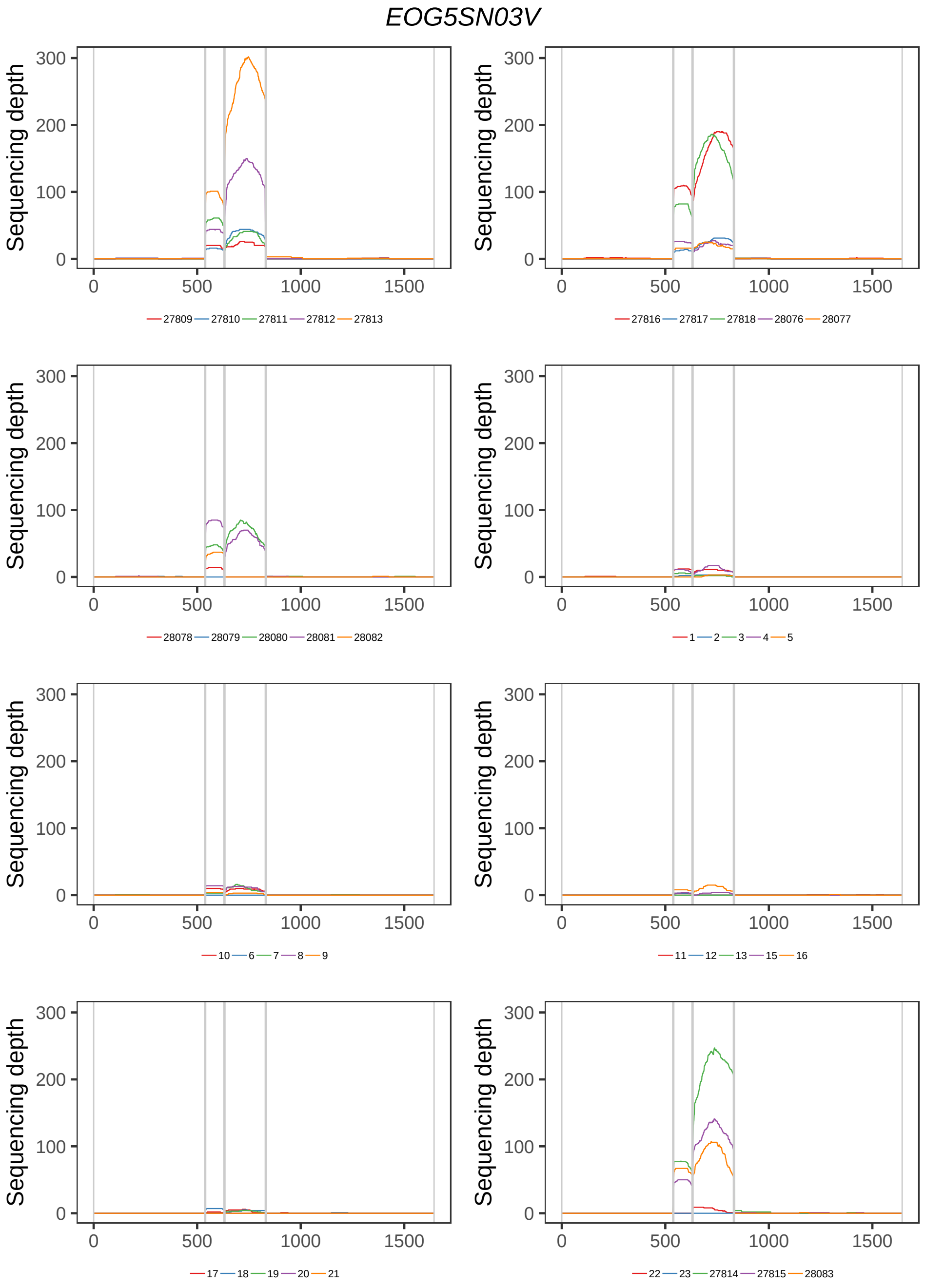

EOG5SN03V
Sequencing depth
27809 27810 27811 27812 27813
27816 27817 27818 28076 28077
28078 28079 28080 28081 28082
1 2 3 4 5
10 6 7 8 9
11 12 13 15 16
17 18 19 20 21
22 23 27814 27815 28083

EOG5VQ84J

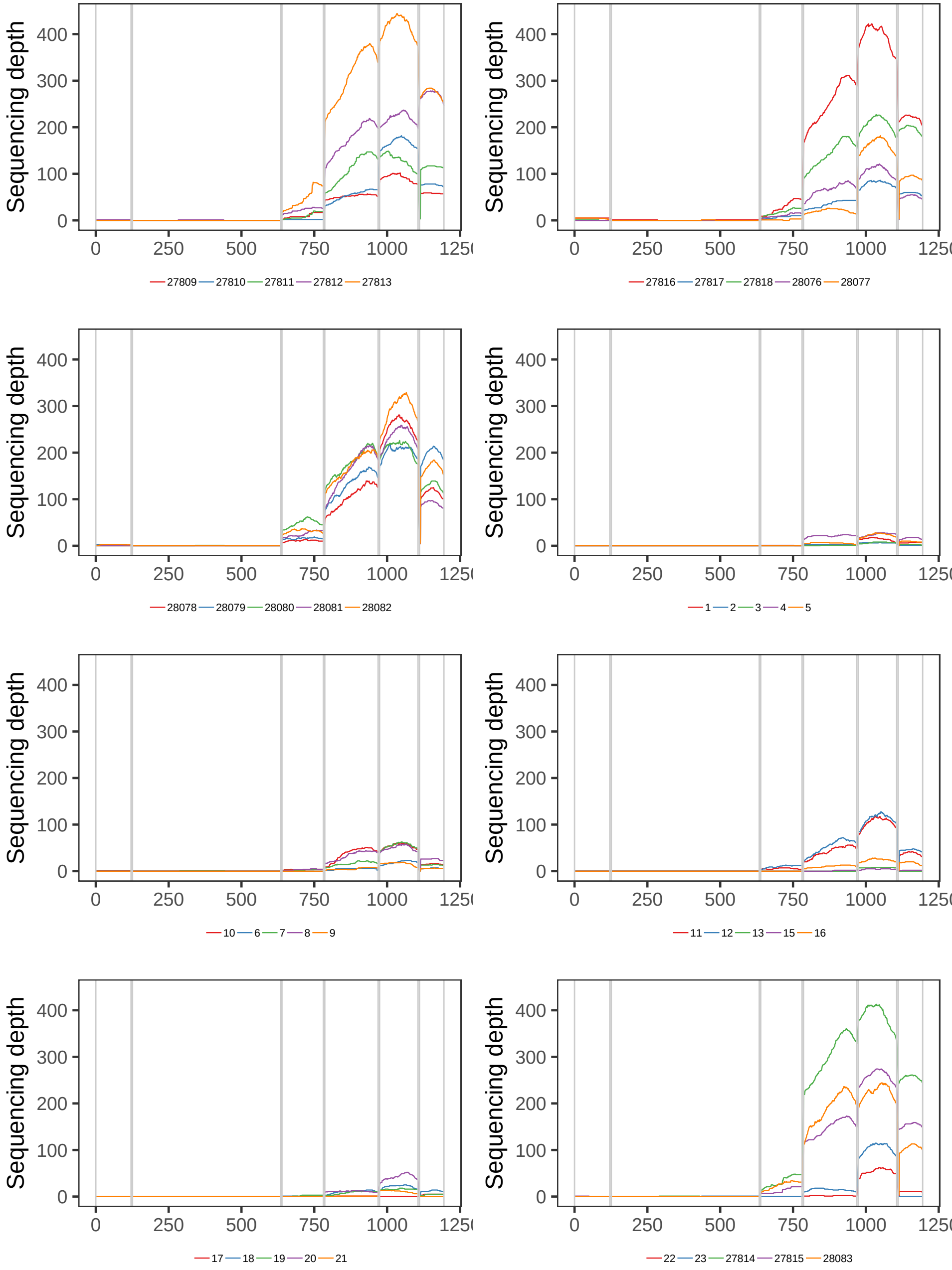
Sequencing depth
0
100
200
300
400
0
250
500
750
1000
1250
27809 27810 27811 27812 27813
27816 27817 27818 28076 28077
28078 28079 28080 28081 28082
1 2 3 4 5
10 6 7 8 9
11 12 13 15 16
17 18 19 20 21
22 23 27814 27815 28083

EOG5XGXFB

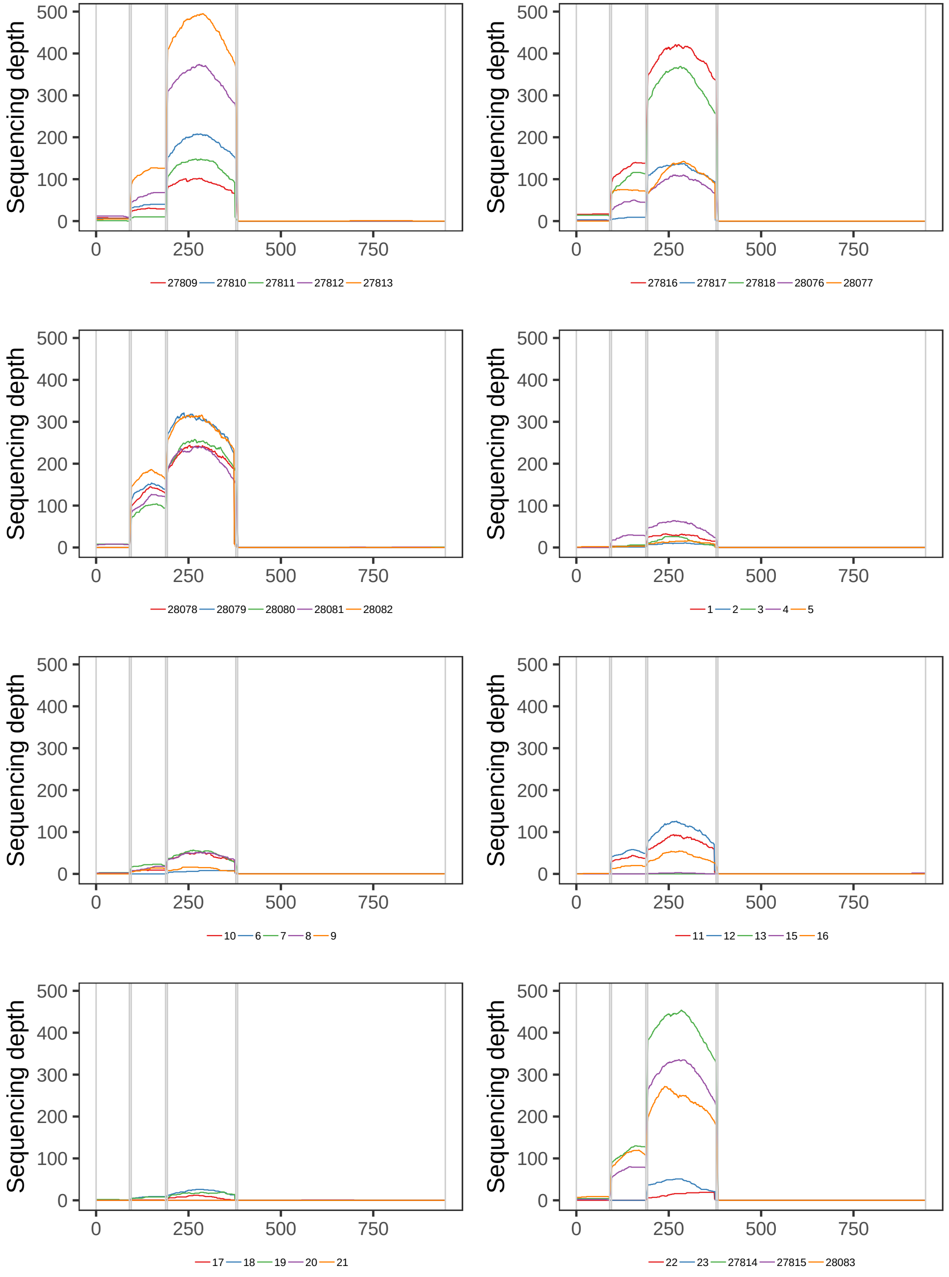

Sequencing depth
500
400
300
200
100
0
0
250
500
750
27809 27810 27811 27812 27813
27816 27817 27818 28076 28077
28078 28079 28080 28081 28082
1 2 3 4 5
10 6 7 8 9
11 12 13 15 16
17 18 19 20 21
22 23 27814 27815 28083

EOG5Z34VX

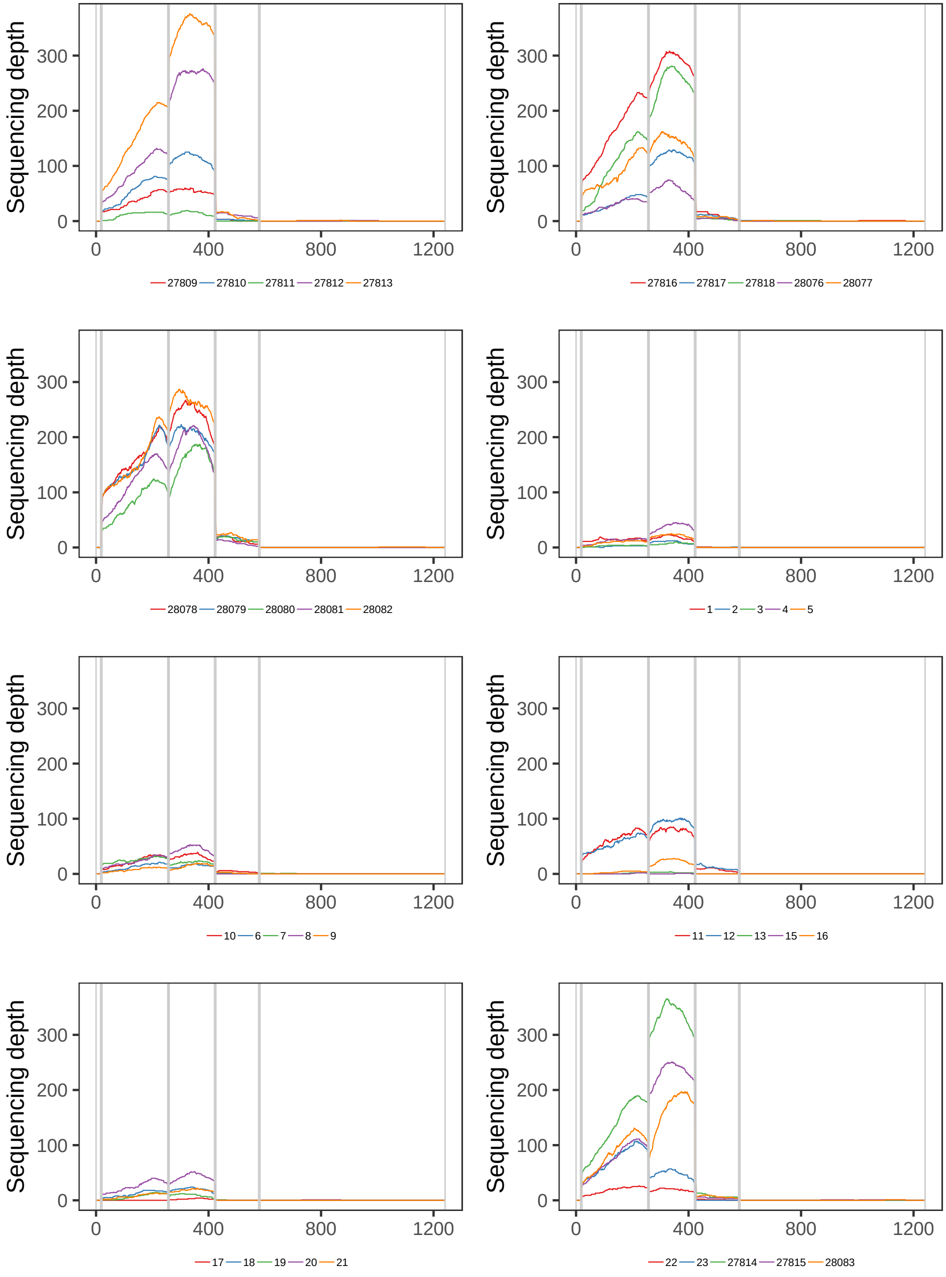
Sequencing depth
0
100
200
300
0
400
800
1200
27809 27810 27811 27812 27813
27816 27817 27818 28076 28077
28078 28079 28080 28081 28082
1 2 3 4 5
10 6 7 8 9
11 12 13 15 16
17 18 19 20 21
22 23 27814 27815 28083

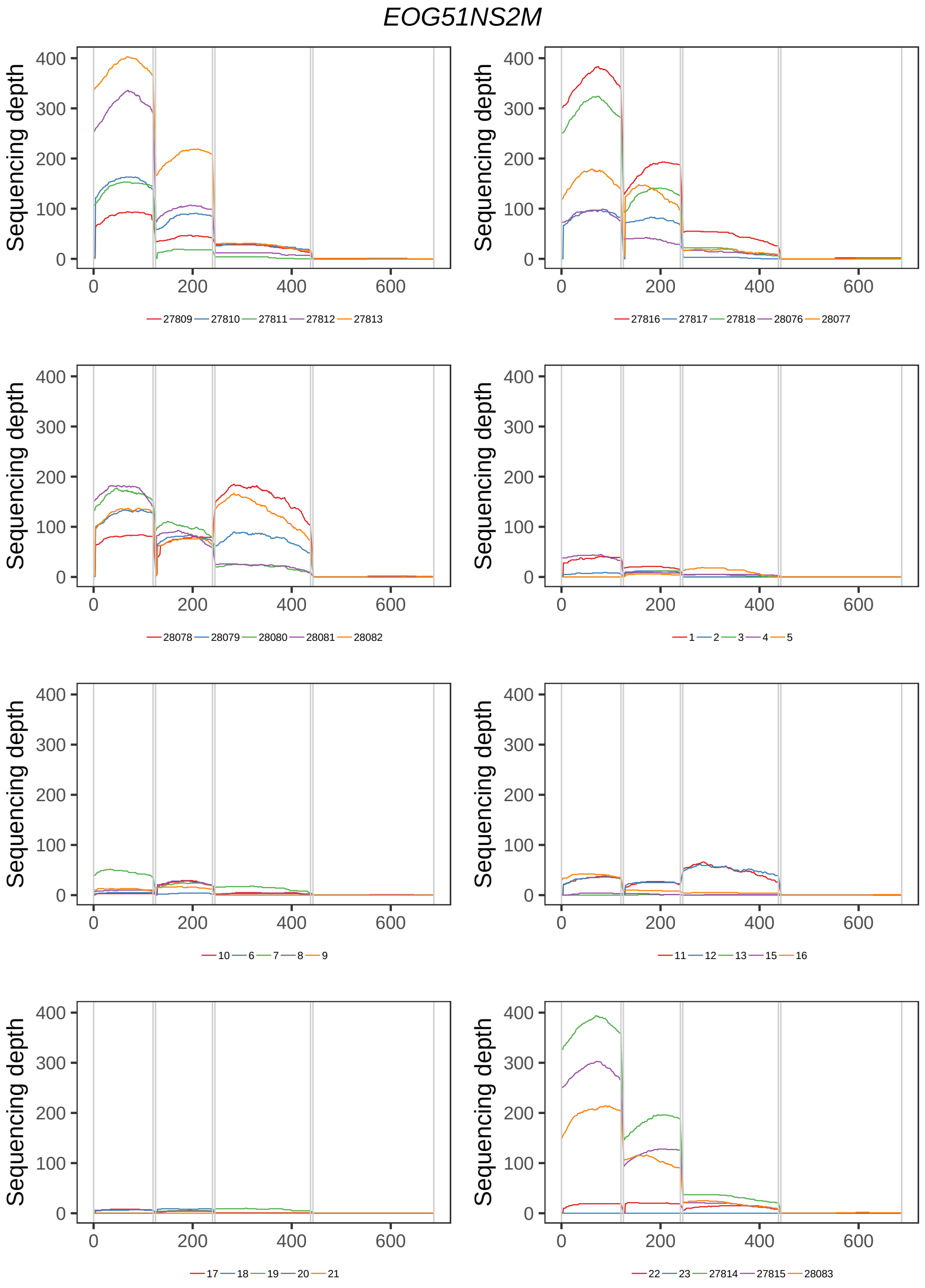
EOG51NS2M
Sequencing depth
0
100
200
300
400
600
27809 27810 27811 27812 27813
27816 27817 27818 28076 28077
28078 28079 28080 28081 28082
1 2 3 4 5
10 6 7 8 9
11 12 13 15 16
17 18 19 20 21
22 23 27814 27815 28083

EOG59S4NJ

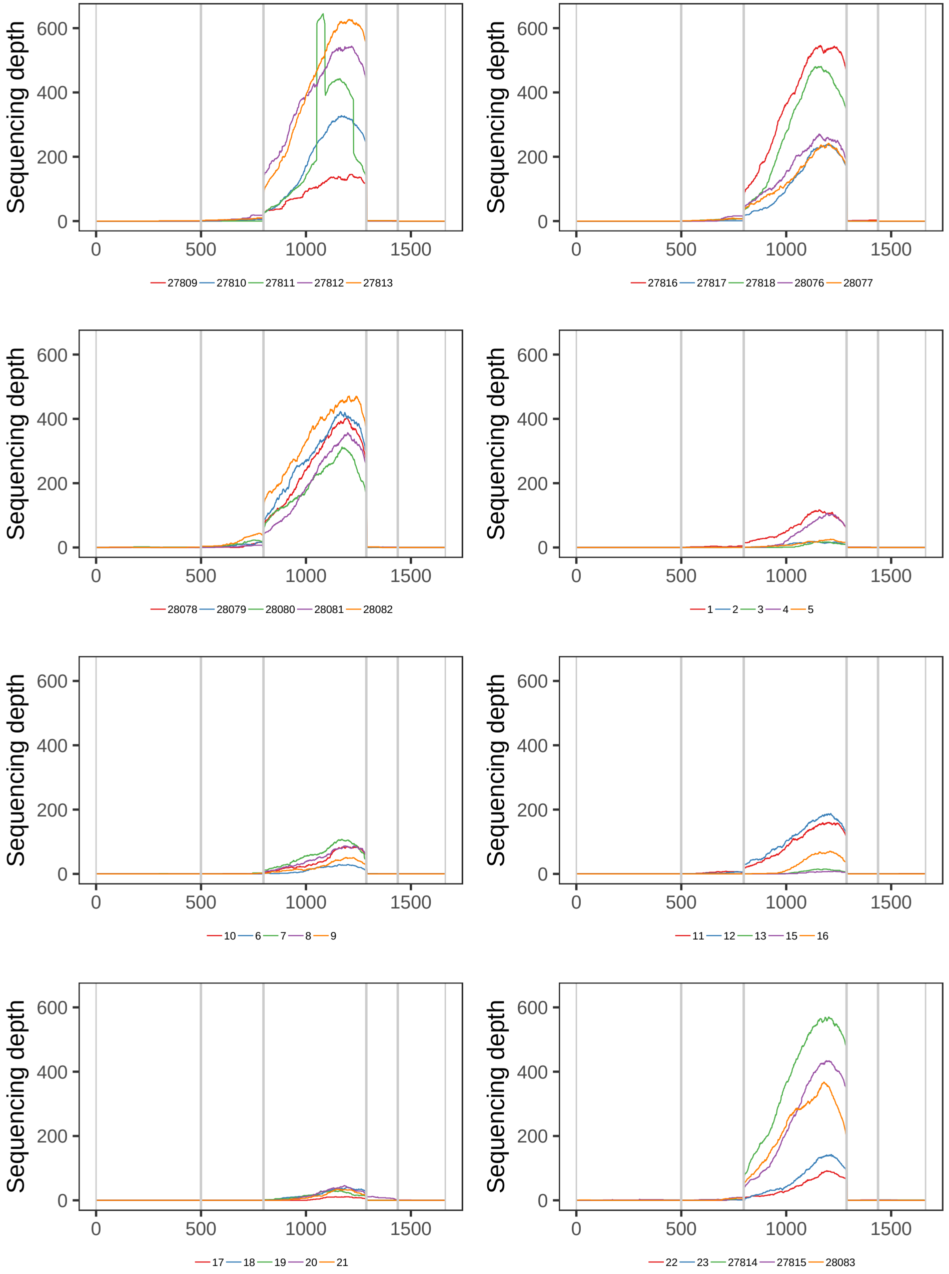
Sequencing depth
0
200
400
600
0
500
1000
1500
27809 27810 27811 27812 27813
27816 27817 27818 28076 28077
28078 28079 28080 28081 28082
1 2 3 4 5
10 6 7 8 9
11 12 13 15 16
17 18 19 20 21
22 23 27814 27815 28083

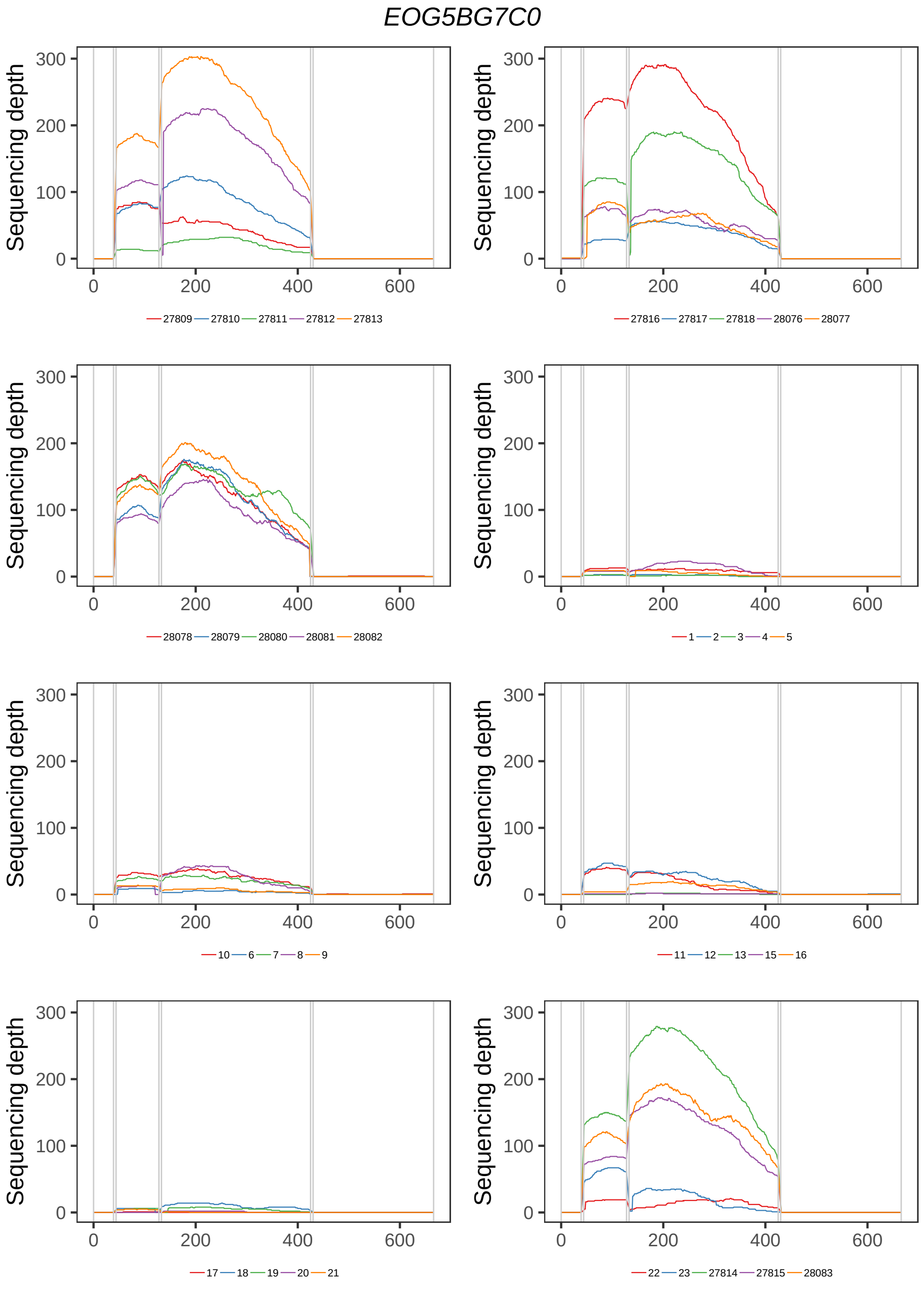
EOG5BG7C0
Sequencing depth
27809 27810 27811 27812 27813
27816 27817 27818 28076 28077
28078 28079 28080 28081 28082
1 2 3 4 5
10 6 7 8 9
11 12 13 15 16
17 18 19 20 21
22 23 27814 27815 28083

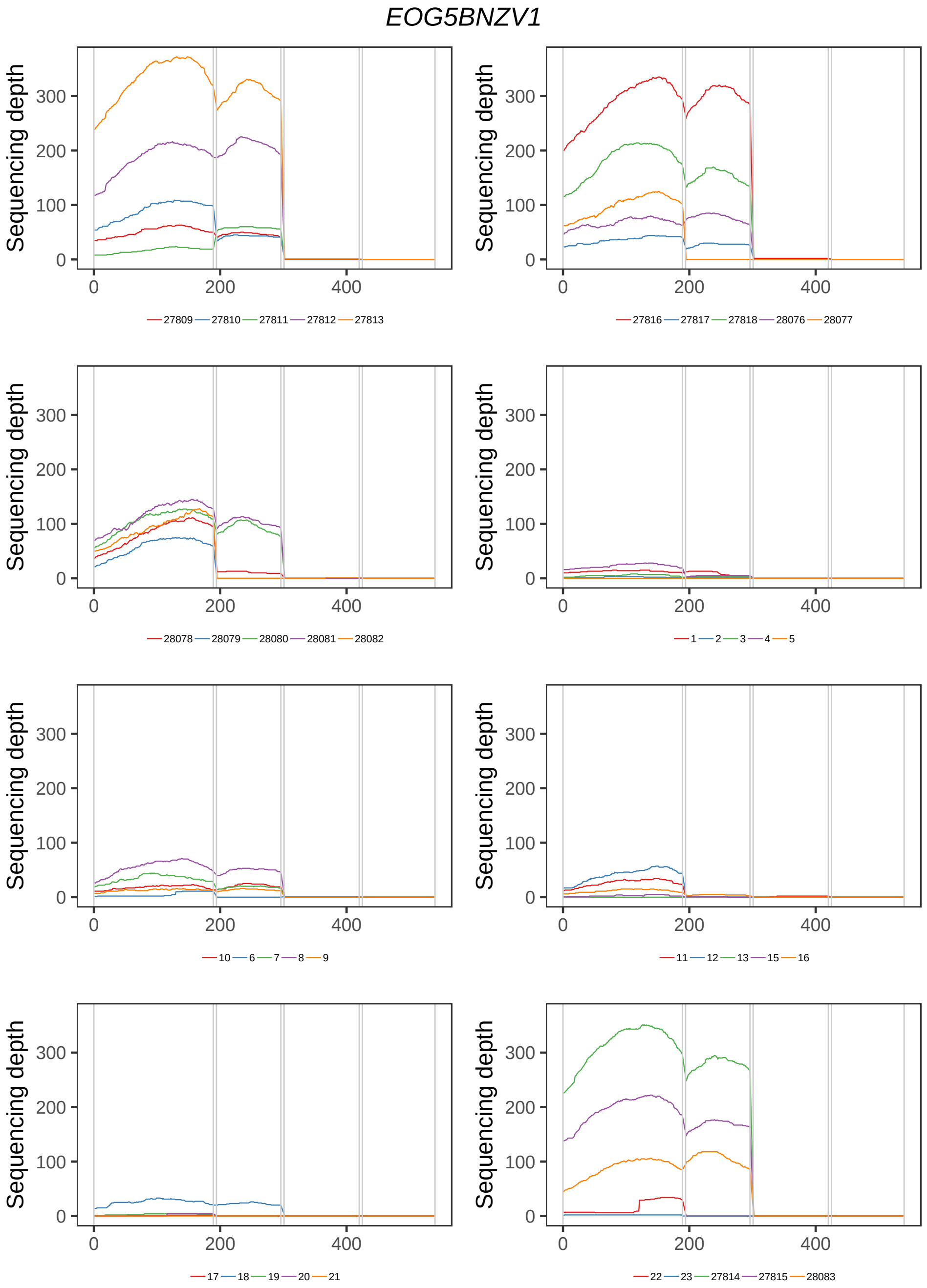
EOG5BNZV1
Sequencing depth
27809 27810 27811 27812 27813
27816 27817 27818 28076 28077
28078 28079 28080 28081 28082
1 2 3 4 5
10 6 7 8 9
11 12 13 15 16
17 18 19 20 21
22 23 27814 27815 28083

EOG5C868D

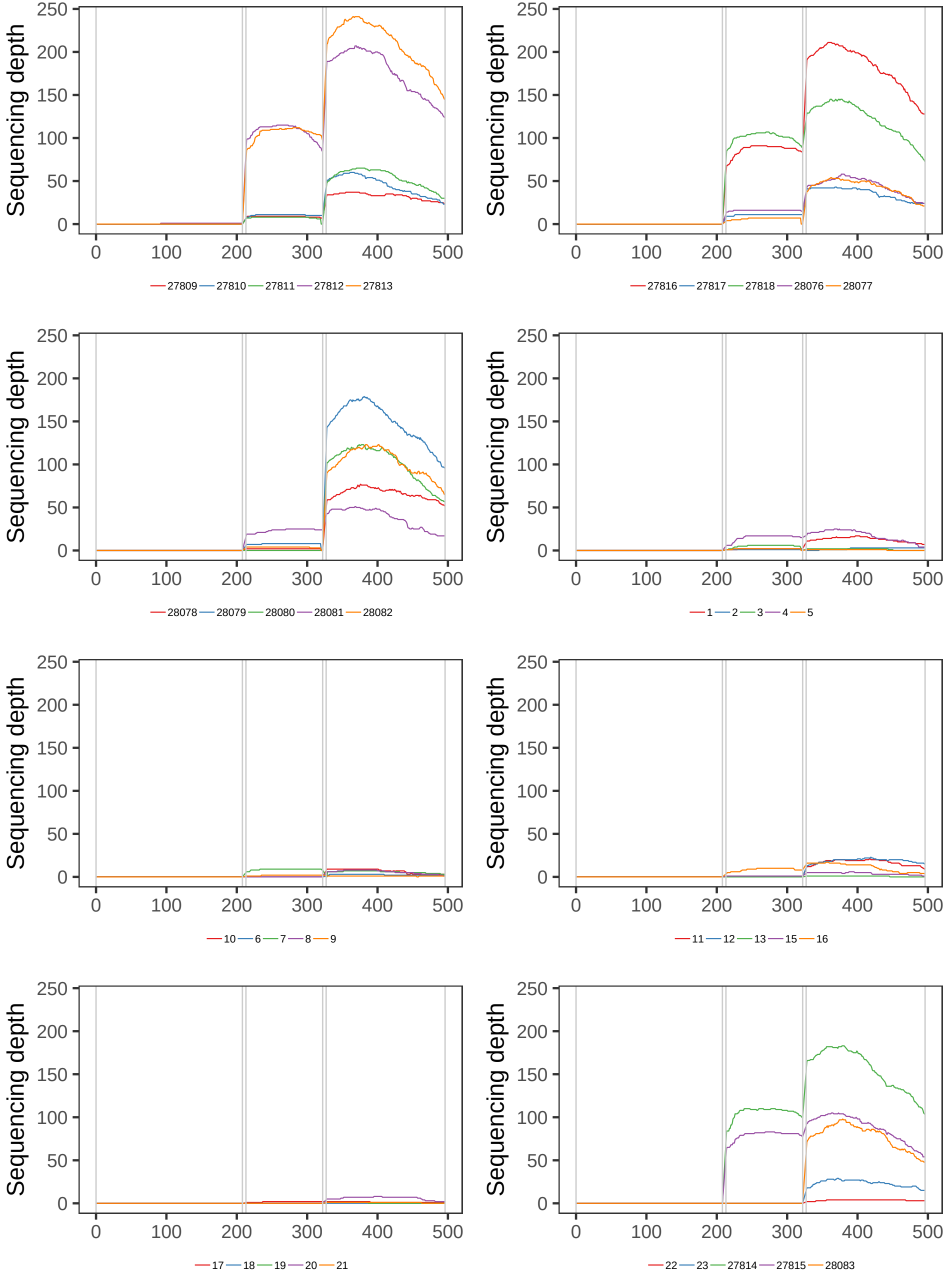
Sequencing depth
250
200
150
100
50
0
0
100
200
300
400
500
27809 27810 27811 27812 27813
27816 27817 27818 28076 28077
28078 28079 28080 28081 28082
1 2 3 4 5
10 6 7 8 9
11 12 13 15 16
17 18 19 20 21
22 23 27814 27815 28083

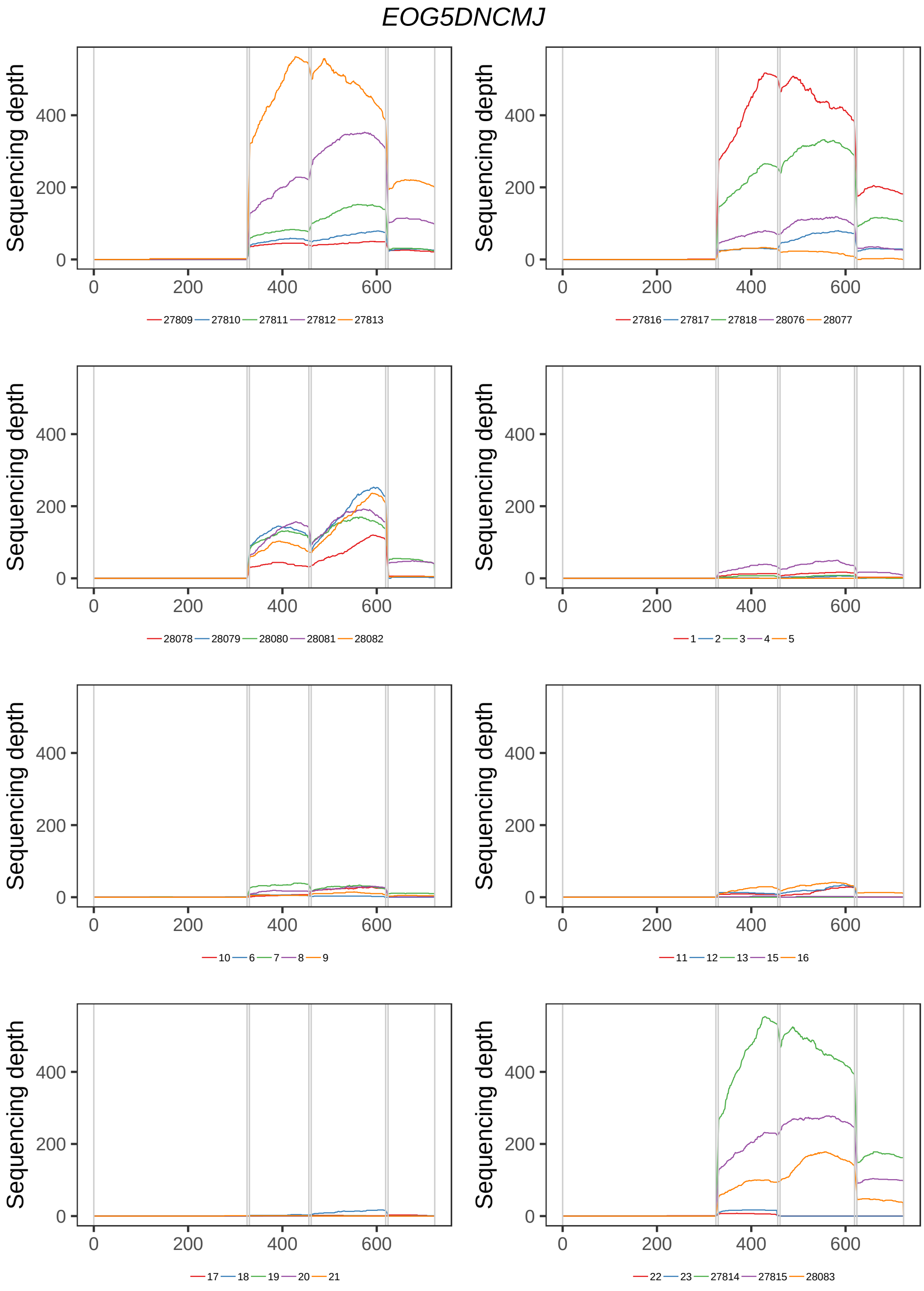
EOG5DNCMJ
Sequencing depth
0
200
400
600
27809 27810 27811 27812 27813
27816 27817 27818 28076 28077
28078 28079 28080 28081 28082
1 2 3 4 5
10 6 7 8 9
11 12 13 15 16
17 18 19 20 21
22 23 27814 27815 28083

EOG5FXPQ3

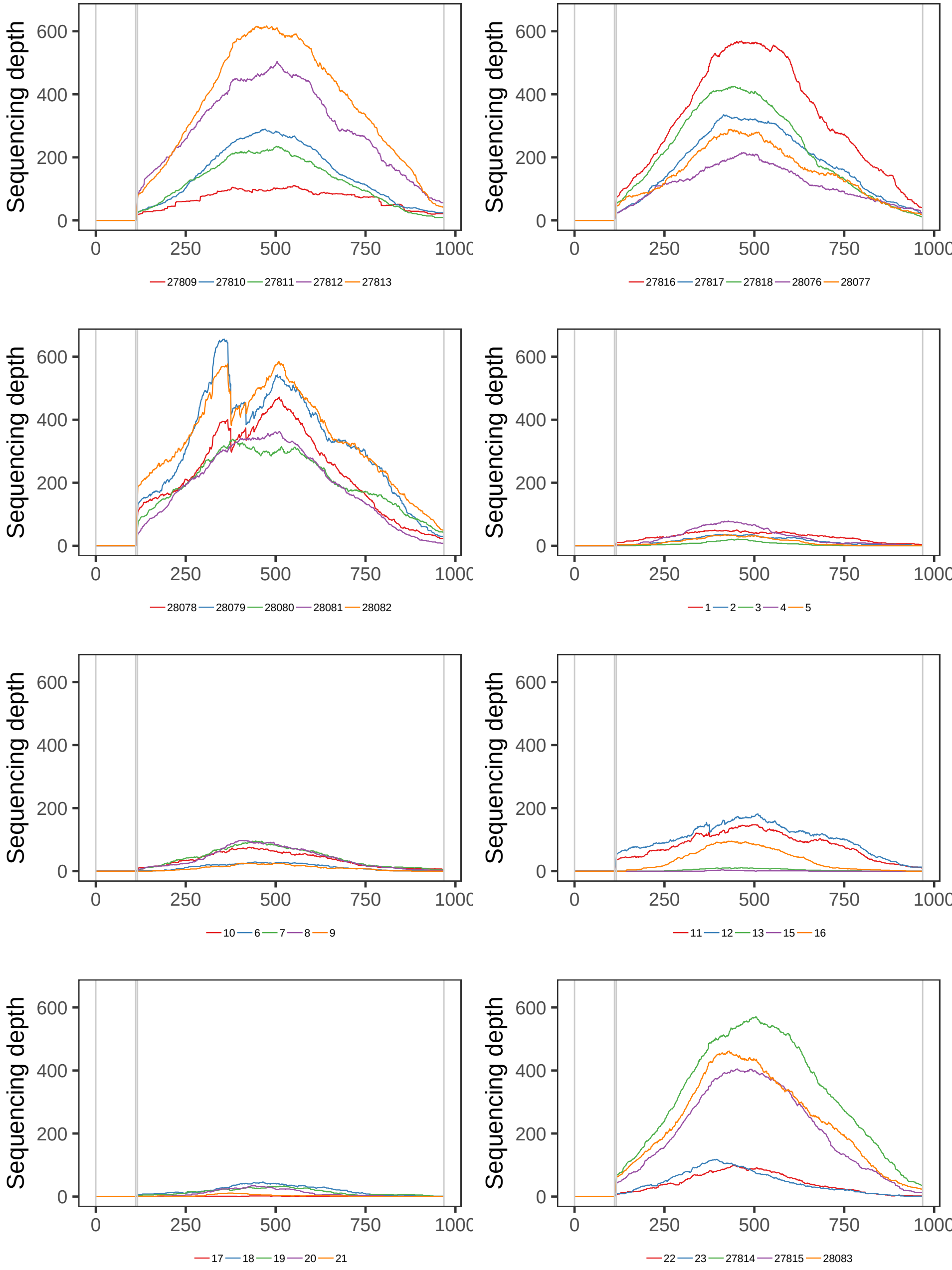
Sequencing depth
0
200
400
600
0
250
500
750
1000
27809 27810 27811 27812 27813
27816 27817 27818 28076 28077
28078 28079 28080 28081 28082
1 2 3 4 5
10 6 7 8 9
11 12 13 15 16
17 18 19 20 21
22 23 27814 27815 28083

EOG5H18B7

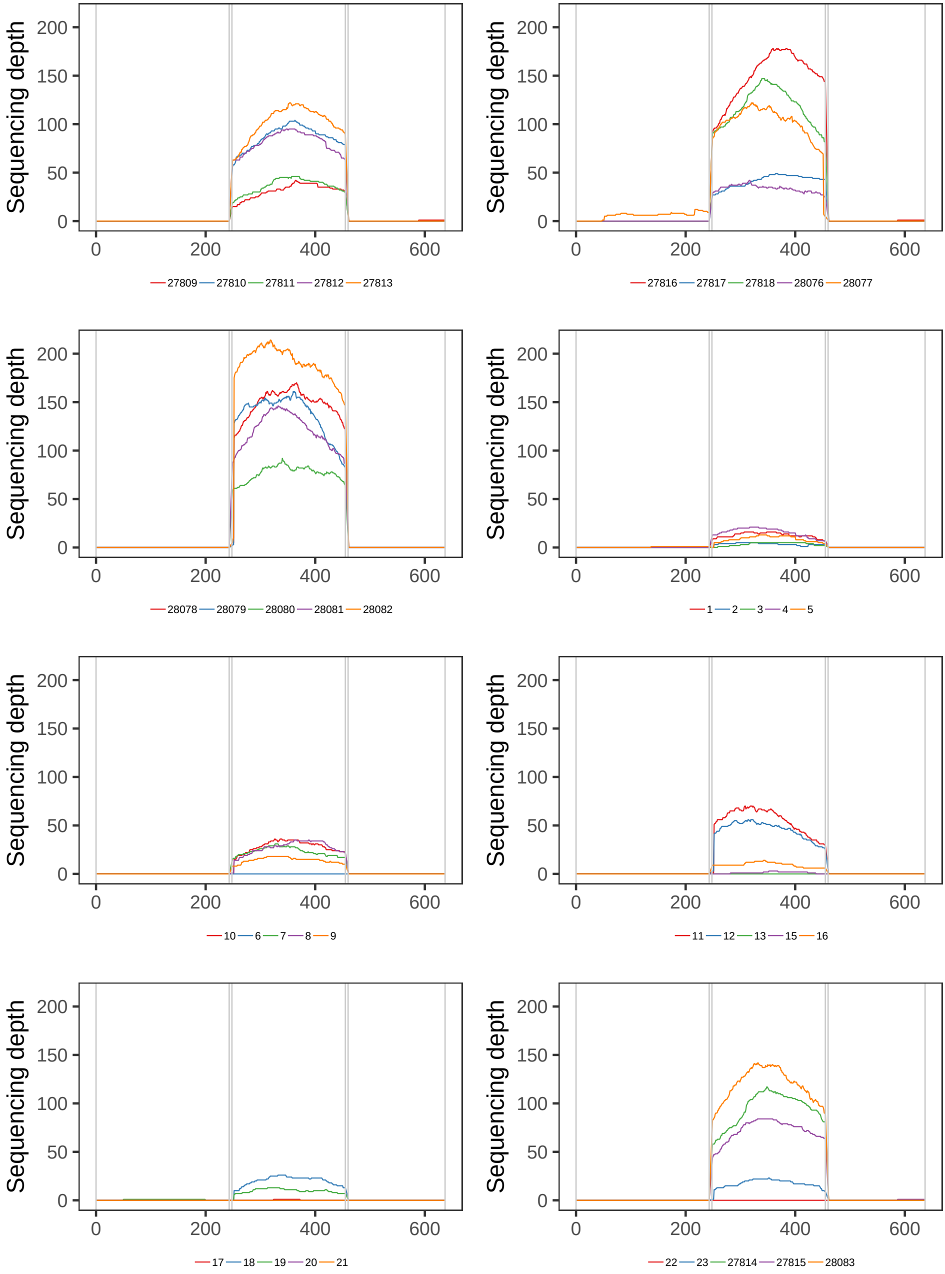
Sequencing depth
0
50
100
150
200
0
200
400
600
27809 27810 27811 27812 27813
27816 27817 27818 28076 28077
28078 28079 28080 28081 28082
1 2 3 4 5
10 6 7 8 9
11 12 13 15 16
17 18 19 20 21
22 23 27814 27815 28083

EOG5HHMHN

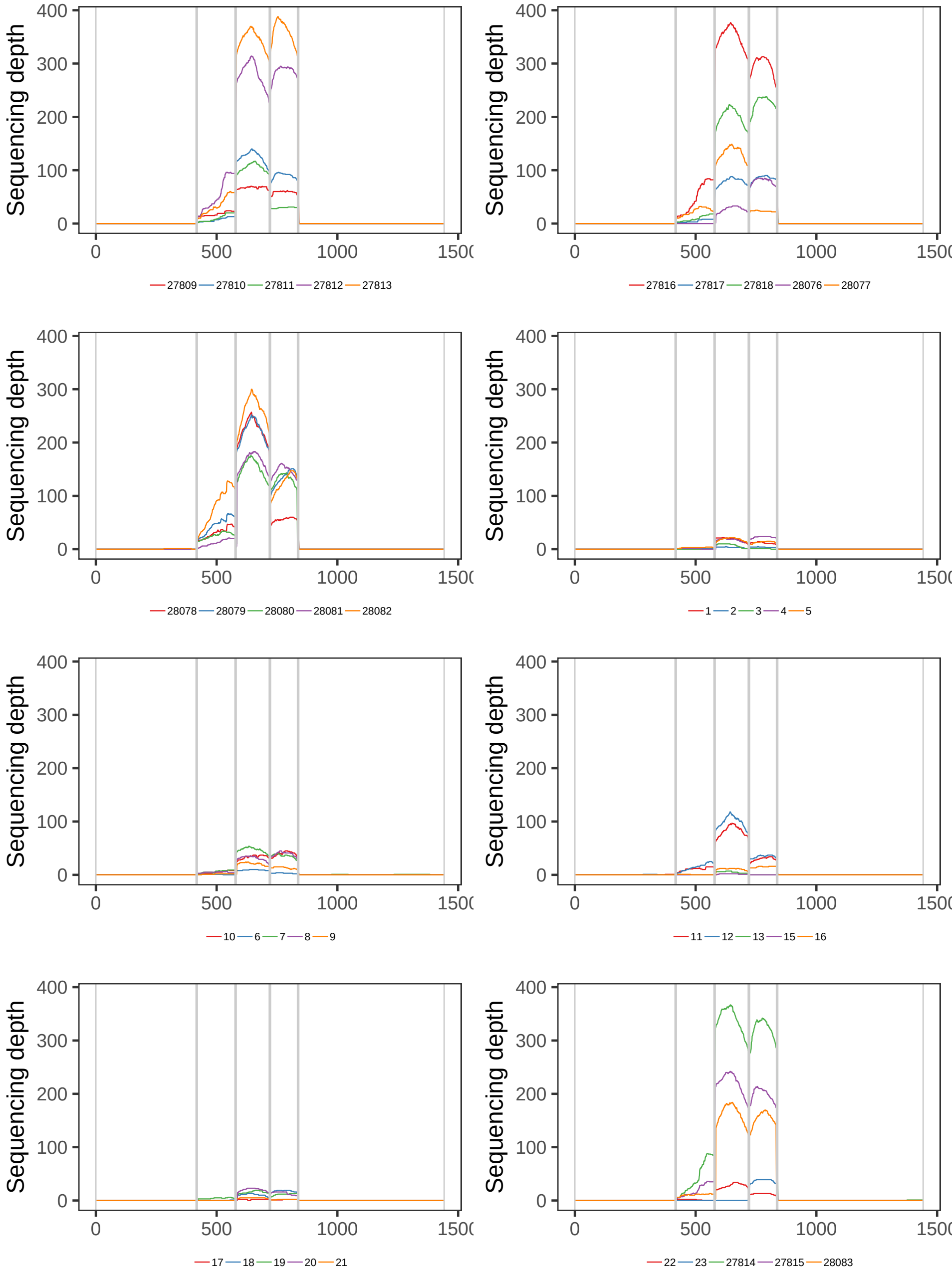

Sequencing depth
0
100
200
300
400
0
500
1000
1500
27809 27810 27811 27812 27813
27816 27817 27818 28076 28077
28078 28079 28080 28081 28082
1 2 3 4 5
10 6 7 8 9
11 12 13 15 16
17 18 19 20 21
22 23 27814 27815 28083

EOG5N5TBZ

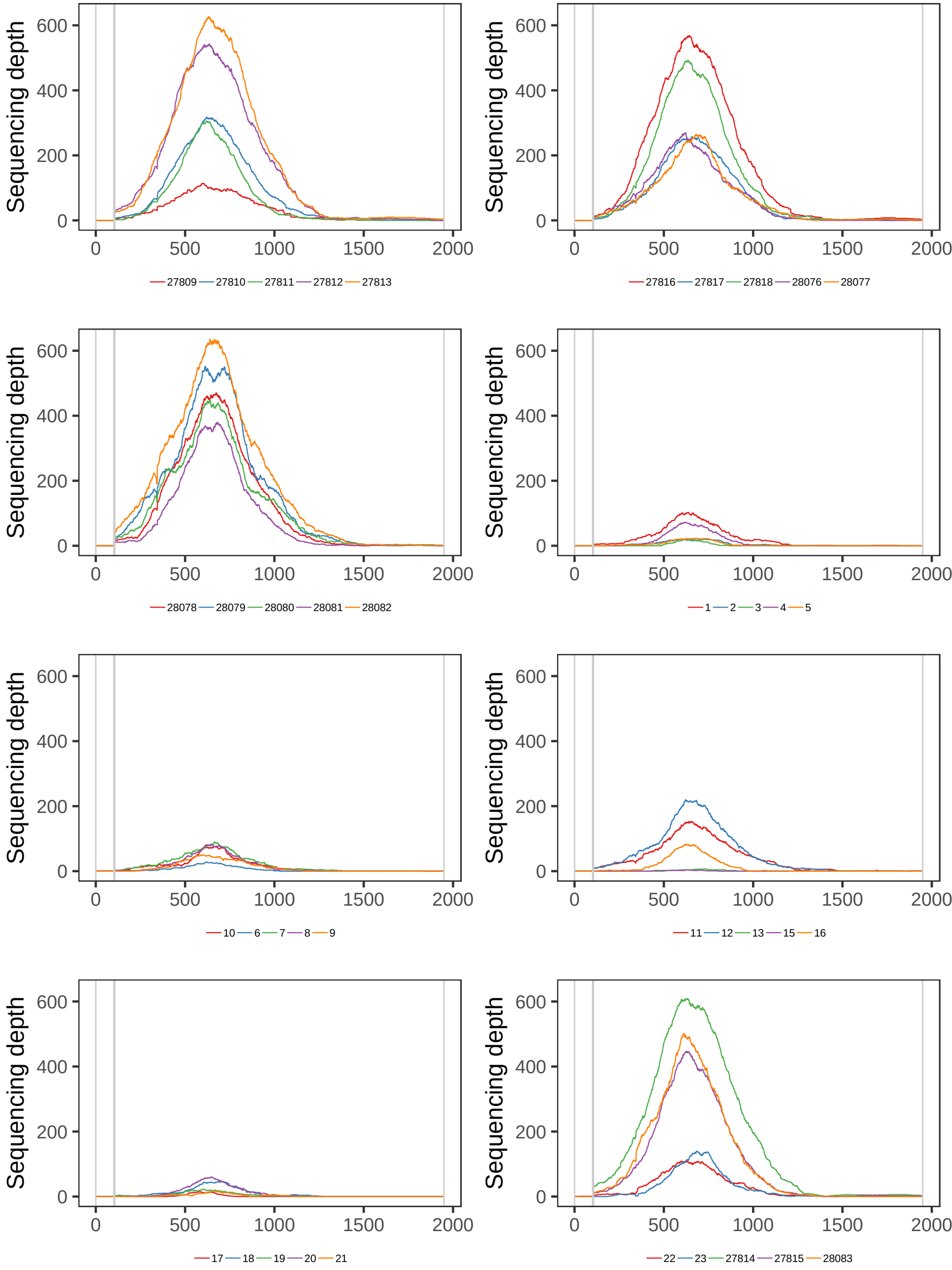
Sequencing depth
600
400
200
0
0
500
1000
1500
2000
27809
27810
27811
27812
27813
27816
27817
27818
28076
28077
28078
28079
28080
28081
28082
1
2
3
4
5
10
6
7
8
9
11
12
13
15
16
17
18
19
20
21
22
23
27814
27815
28083

EOG5NCJV6

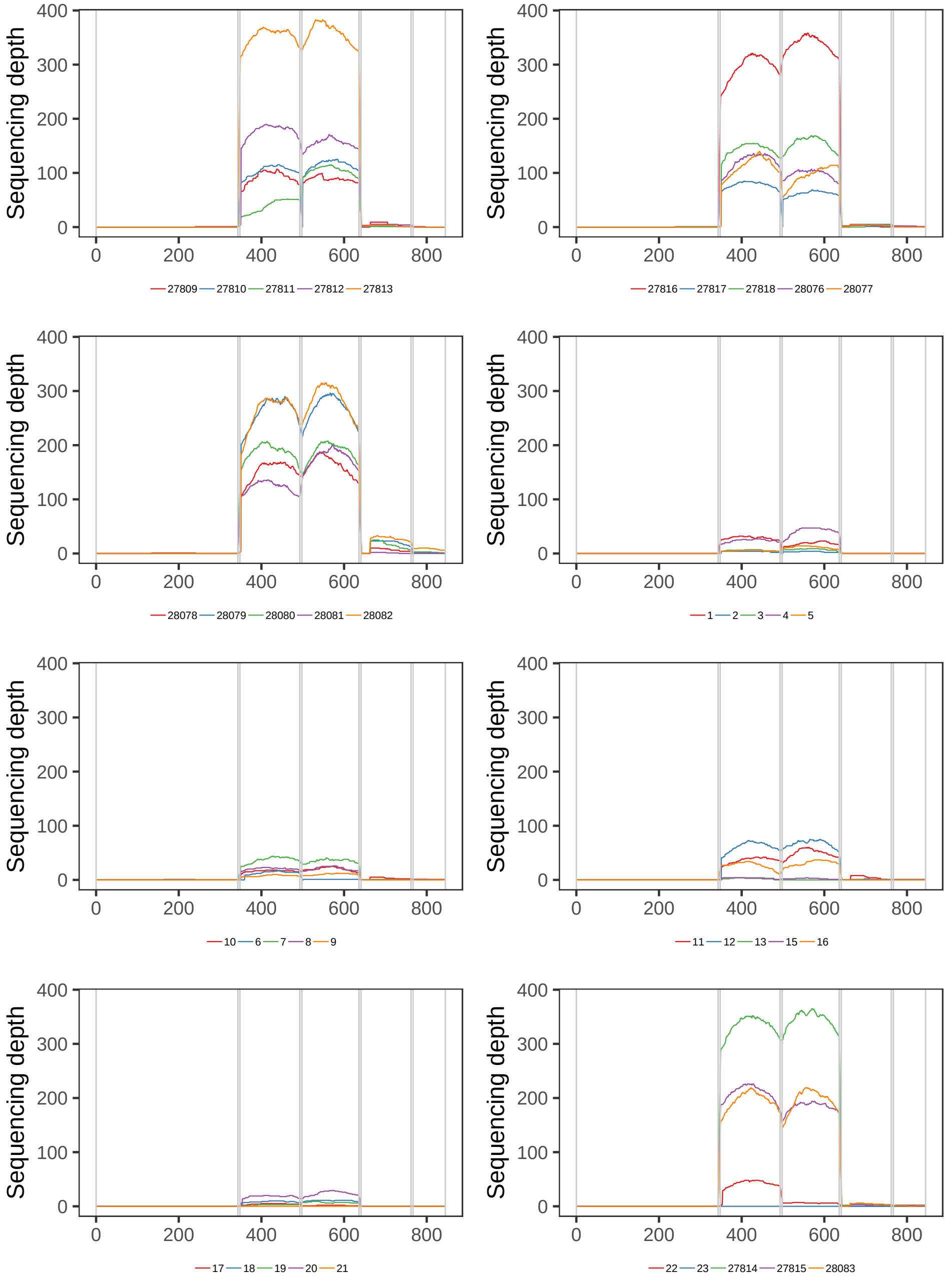
Sequencing depth
400
300
200
100
0
0
200
400
600
800
27809 27810 27811 27812 27813
27816 27817 27818 28076 28077
28078 28079 28080 28081 28082
1 2 3 4 5
10 6 7 8 9
11 12 13 15 16
17 18 19 20 21
22 23 27814 27815 28083

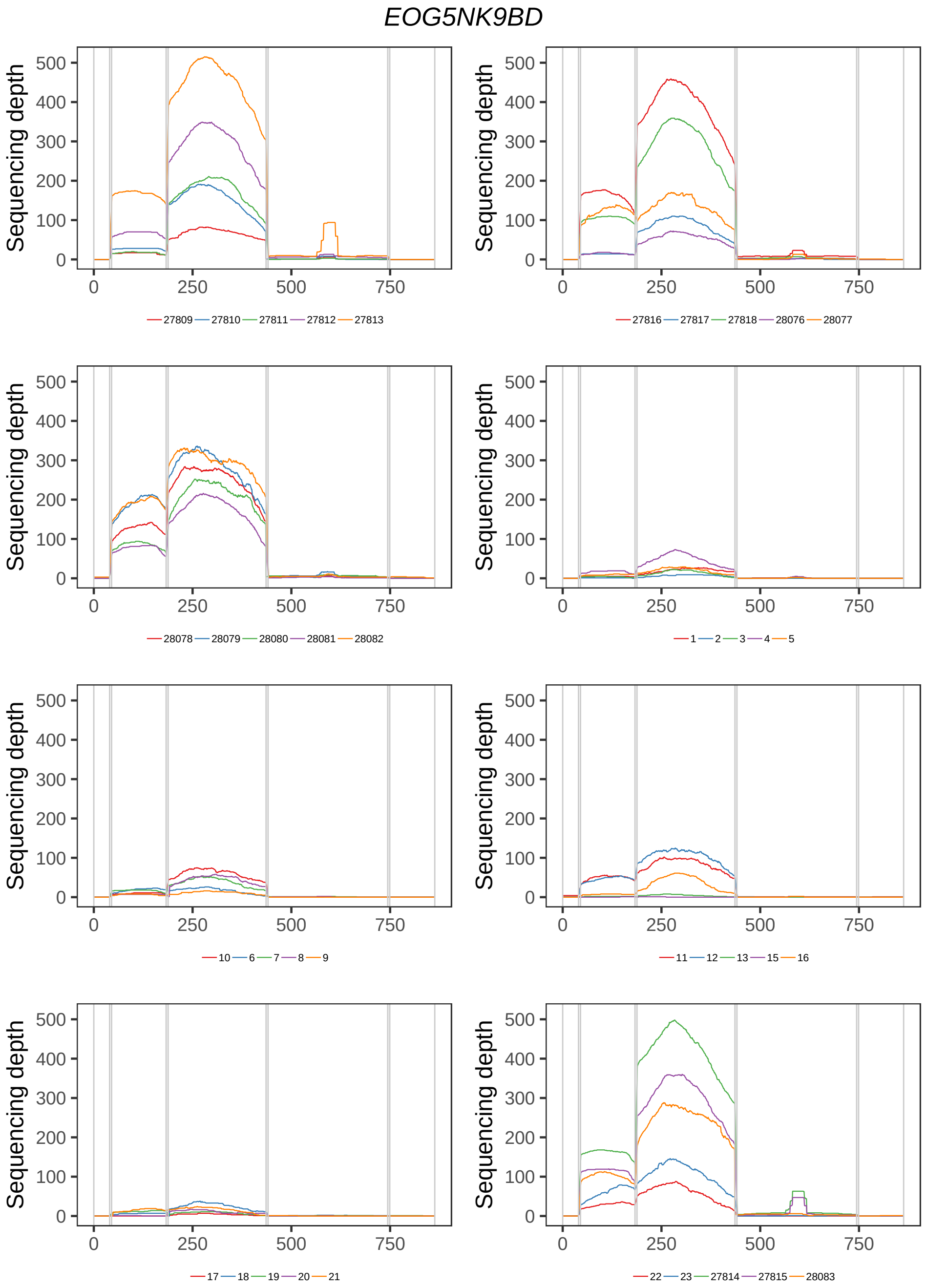
EOG5NK9BD
Sequencing depth
0
100
200
300
400
500
0
250
500
750
27809
27810
27811
27812
27813
27816
27817
27818
28076
28077
28078
28079
28080
28081
28082
1
2
3
4
5
10
6
7
8
9
11
12
13
15
16
17
18
19
20
21
22
23
27814
27815
28083

EOG5R2296

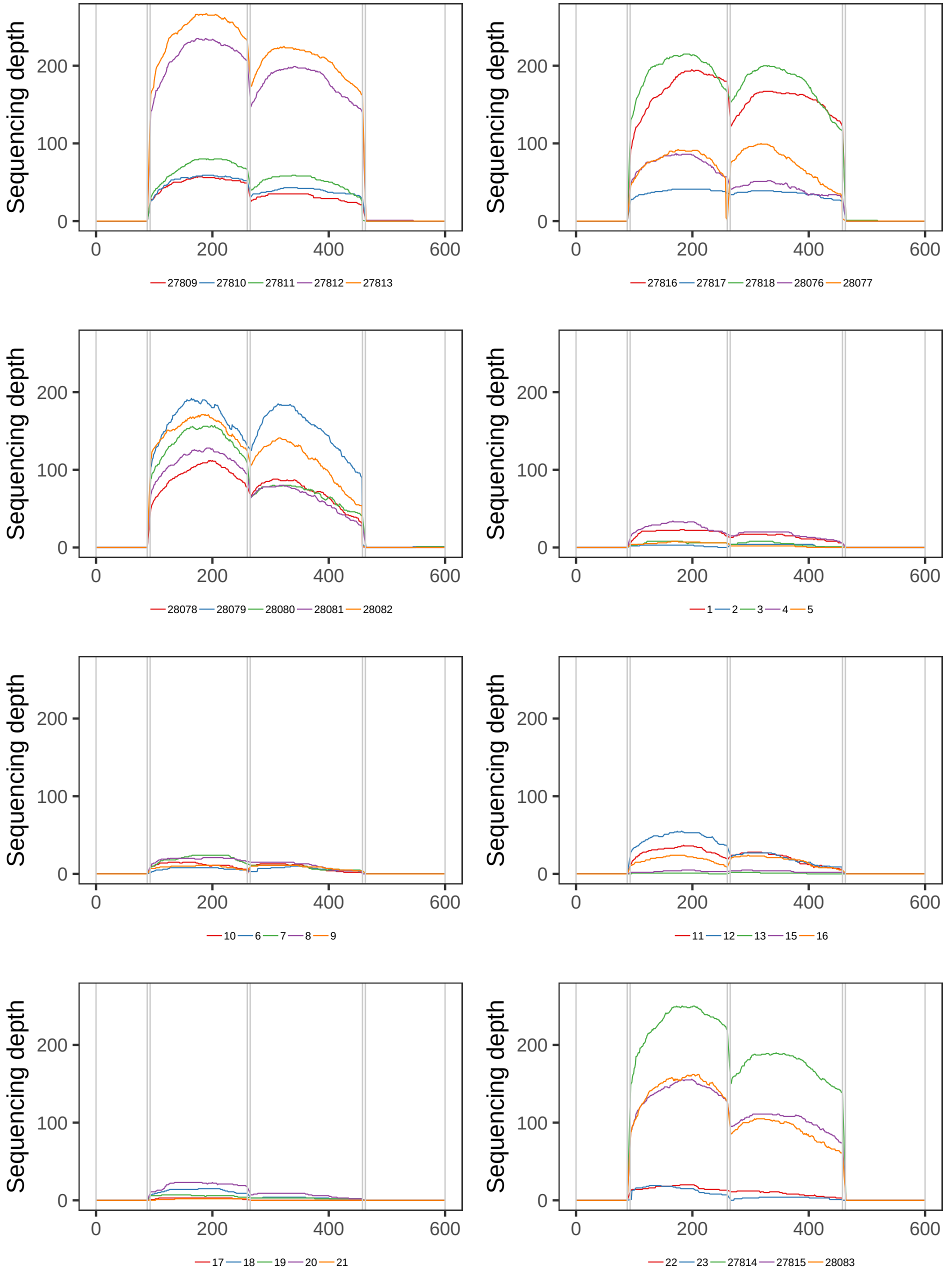
Sequencing depth
0
100
200
0
200
400
600
27809
27810
27811
27812
27813
27816
27817
27818
28076
28077
28078
28079
28080
28081
28082
1
2
3
4
5
10
6
7
8
9
11
12
13
15
16
17
18
19
20
21
22
23
27814
27815
28083

EOG5SBCD9

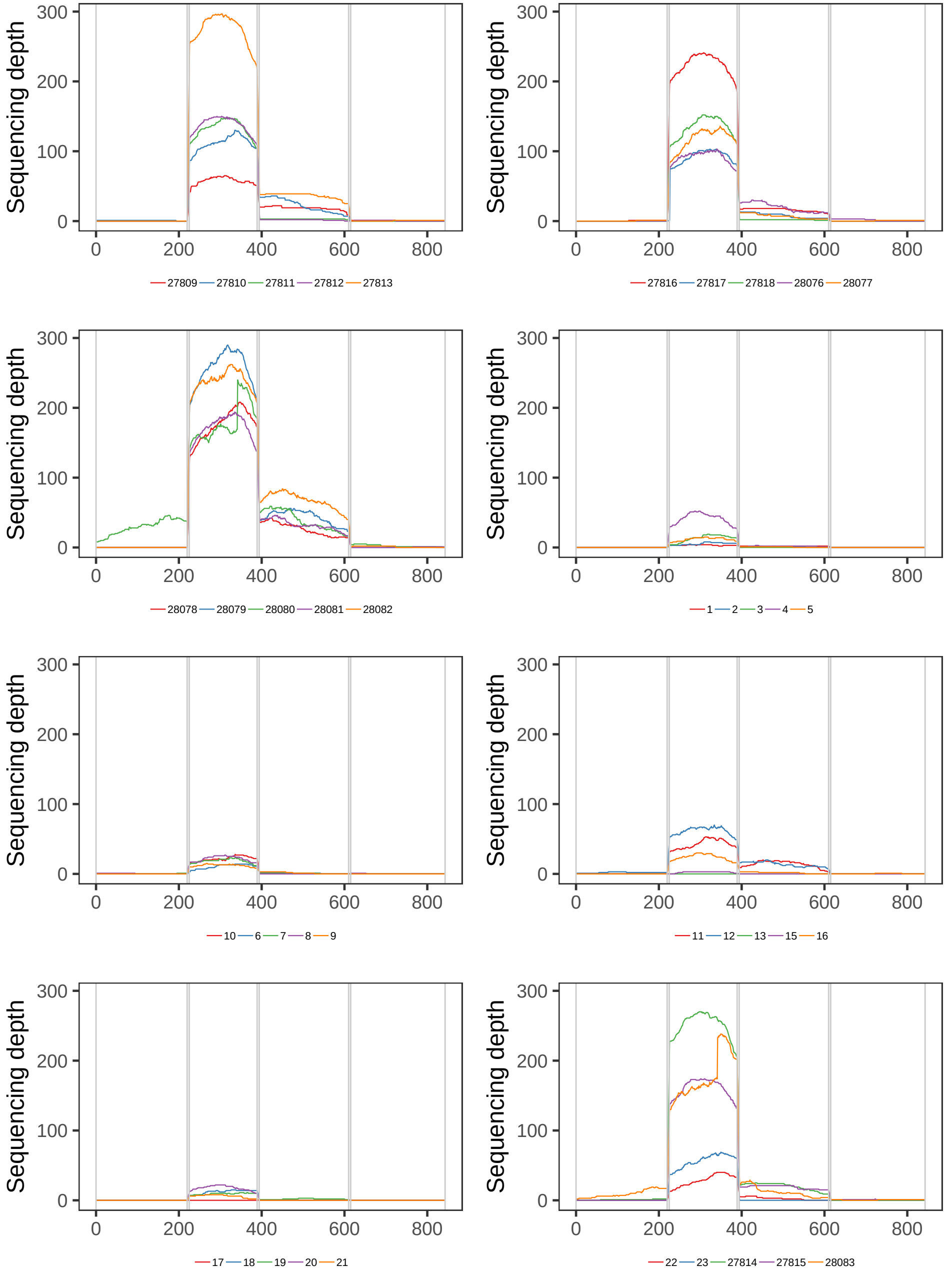

Sequencing depth
300
200
100
0
0
200
400
600
800
27809 27810 27811 27812 27813
27816 27817 27818 28076 28077
28078 28079 28080 28081 28082
1 2 3 4 5
10 6 7 8 9
11 12 13 15 16
17 18 19 20 21
22 23 27814 27815 28083

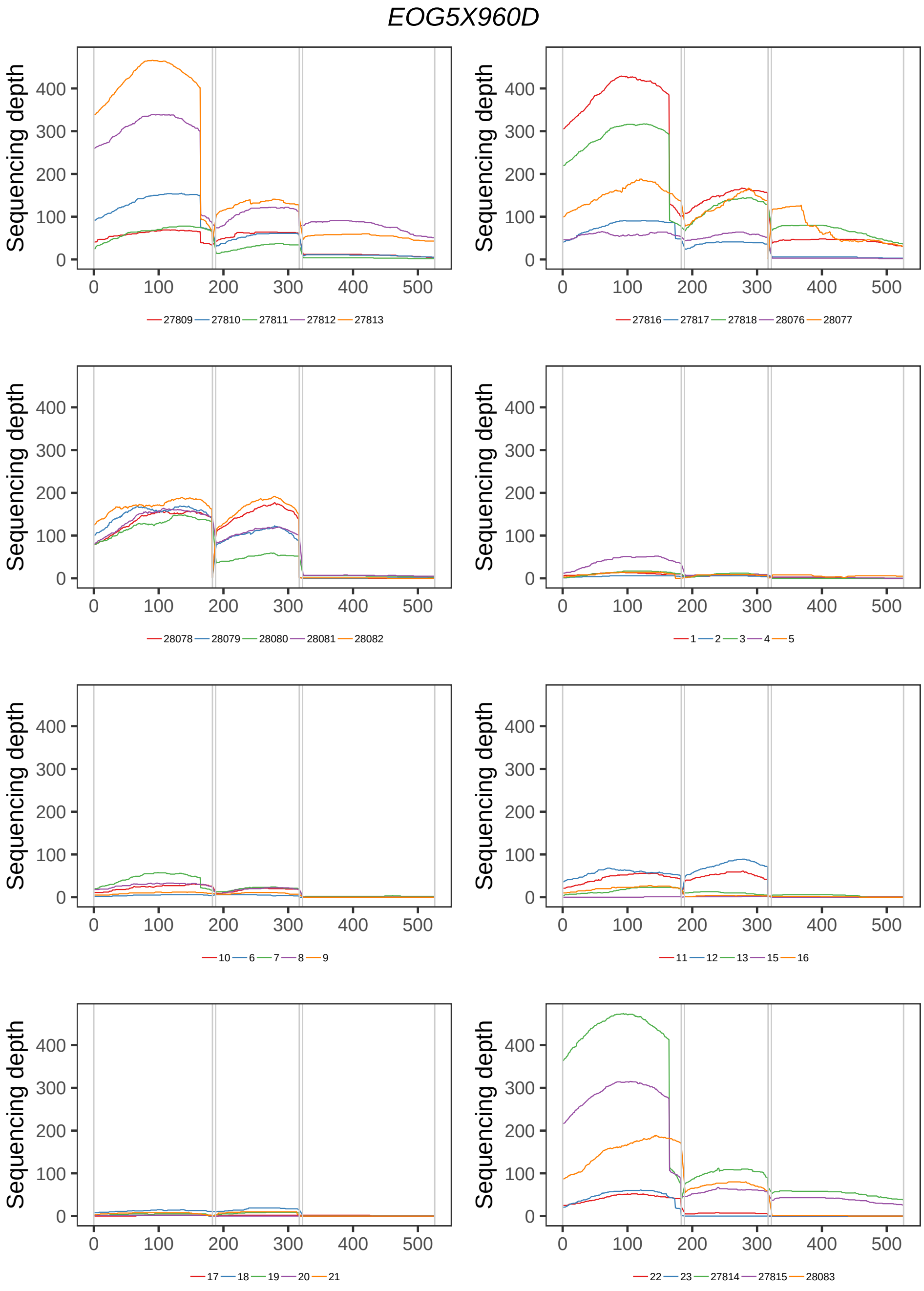

EOG5X960D
Sequencing depth
27809 27810 27811 27812 27813
27816 27817 27818 28076 28077
28078 28079 28080 28081 28082
1 2 3 4 5
10 6 7 8 9
11 12 13 15 16
17 18 19 20 21
22 23 27814 27815 28083

EOG5XWDDJ

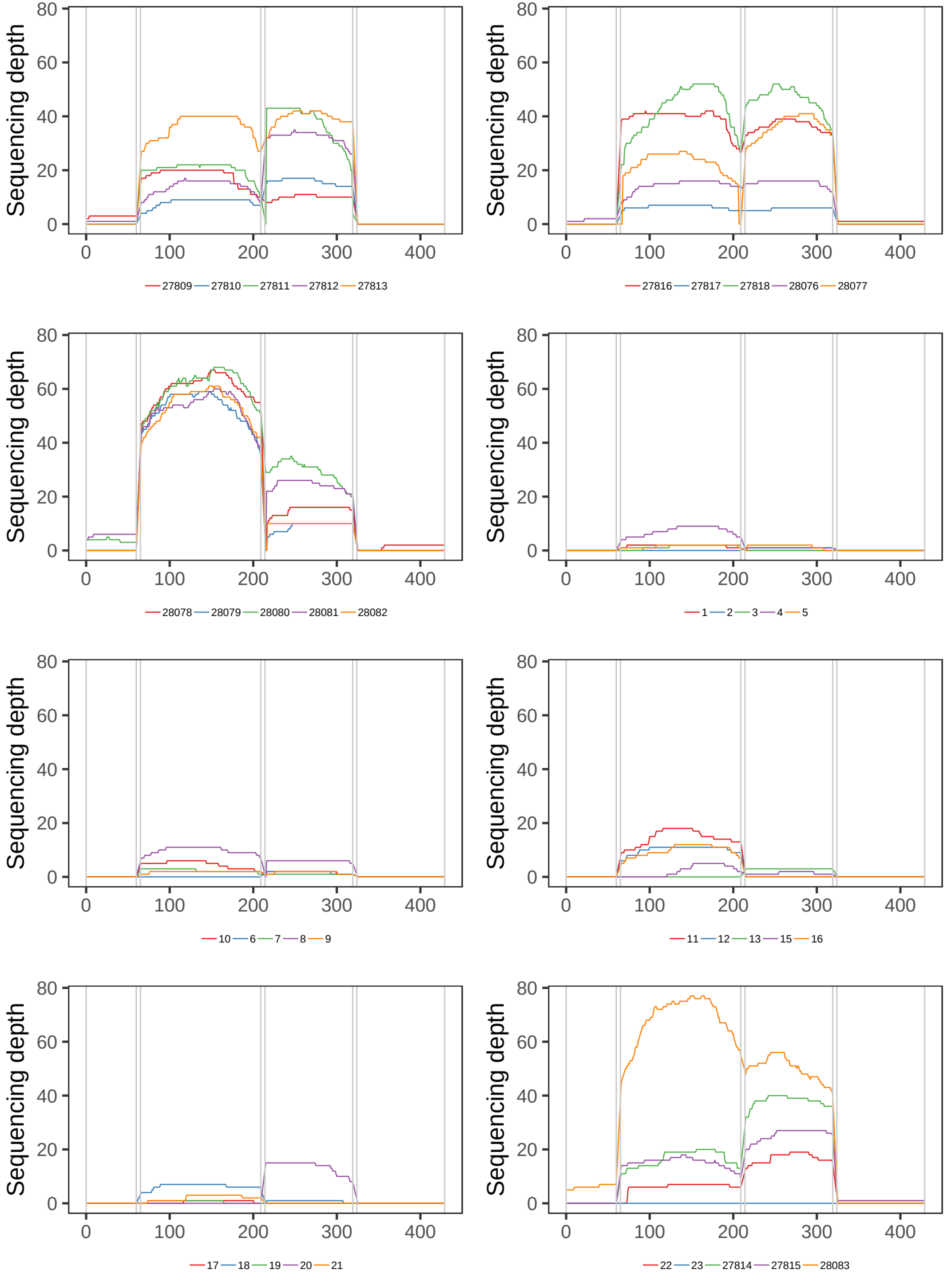
Sequencing depth
0
20
40
60
80
0
100
200
300
400
27809 27810 27811 27812 27813
27816 27817 27818 28076 28077
28078 28079 28080 28081 28082
1 2 3 4 5
10 6 7 8 9
11 12 13 15 16
17 18 19 20 21
22 23 27814 27815 28083

EOG5ZGMV6

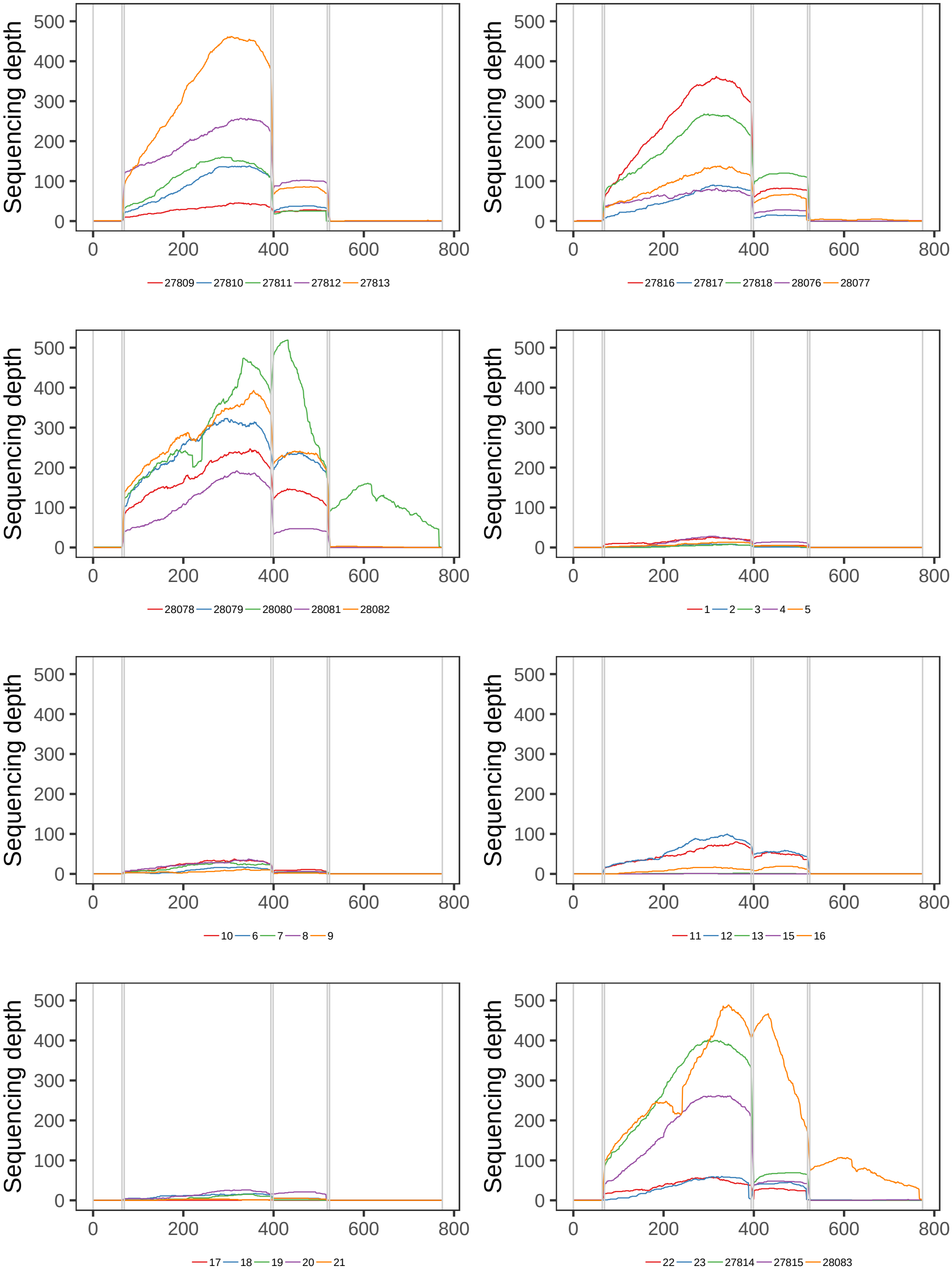
Sequencing depth
0
100
200
300
400
500
0
200
400
600
800
27809 27810 27811 27812 27813
27816 27817 27818 28076 28077
28078 28079 28080 28081 28082
1 2 3 4 5
10 6 7 8 9
11 12 13 15 16
17 18 19 20 21
22 23 27814 27815 28083

EOG5ZPCC0

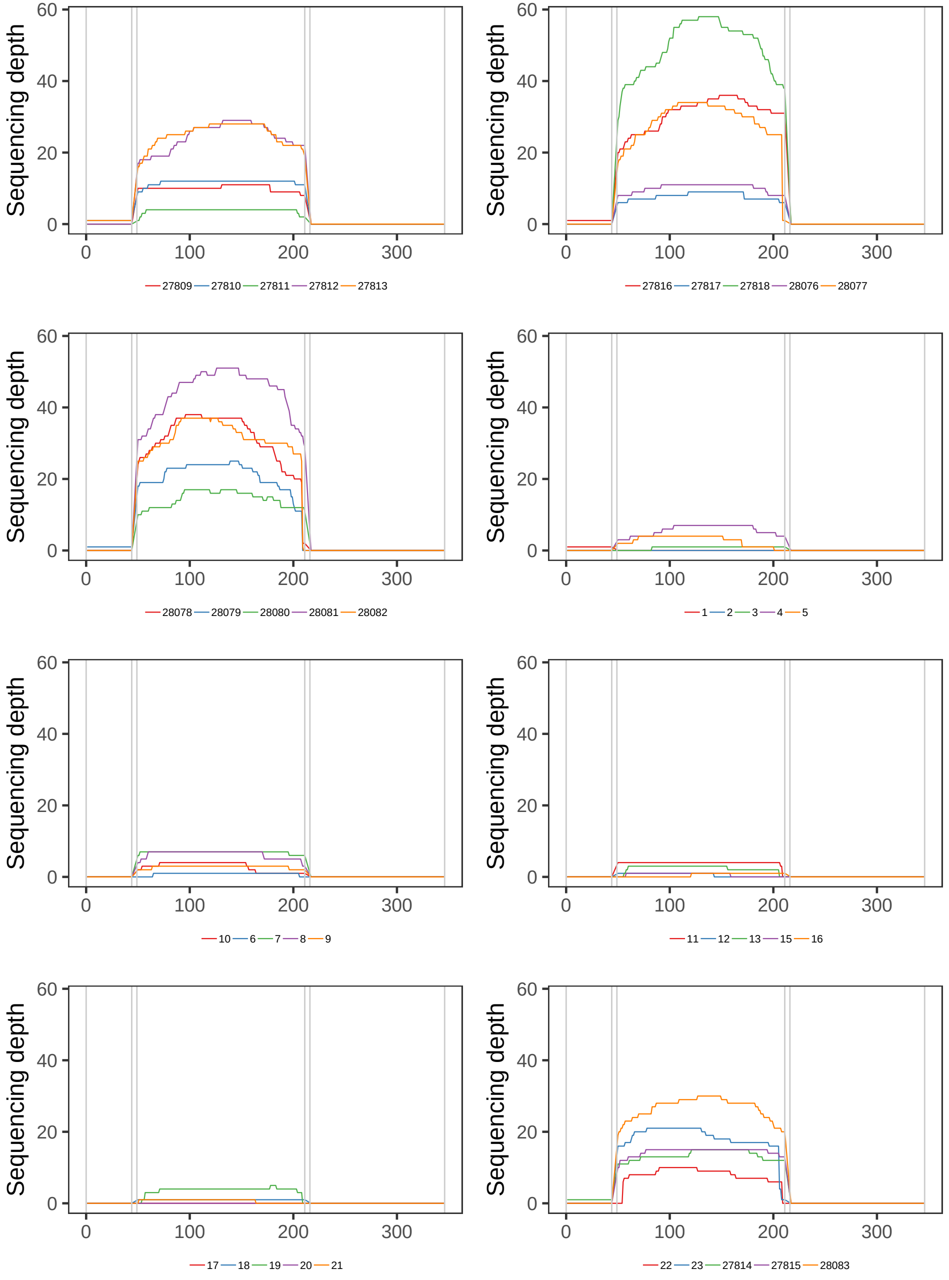

Sequencing depth
0
20
40
60
0
100
200
300
27809
27810
27811
27812
27813
27816
27817
27818
28076
28077
28078
28079
28080
28081
28082
1
2
3
4
5
10
6
7
8
9
11
12
13
15
16
17
18
19
20
21
22
23
27814
27815
28083

EOG51JWTZ

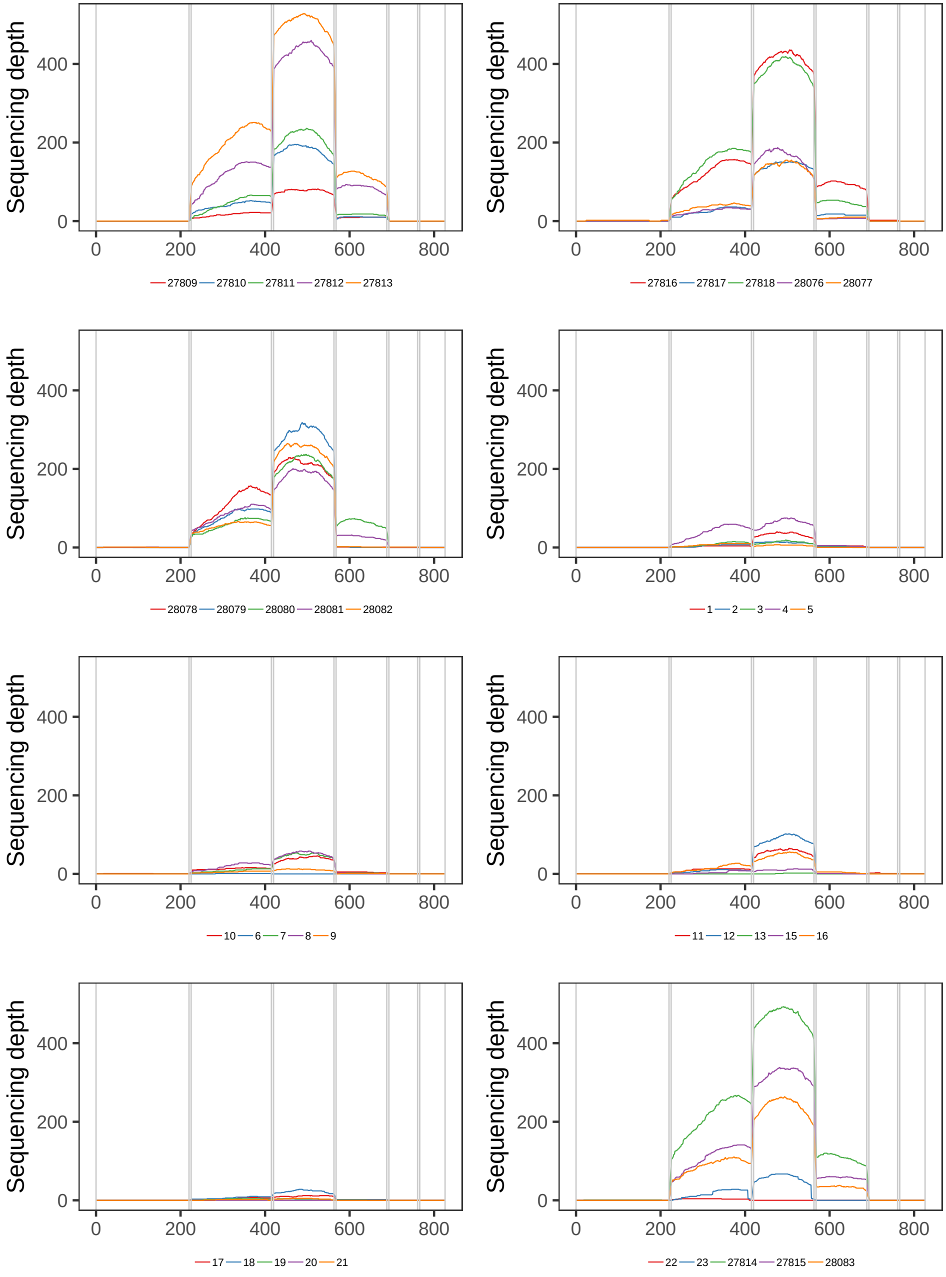

Sequencing depth
400
200
0
0
200
400
600
800
27809
27810
27811
27812
27813
27816
27817
27818
28076
28077
28078
28079
28080
28081
28082
1
2
3
4
5
10
6
7
8
9
11
12
13
15
16
17
18
19
20
21
22
23
27814
27815
28083

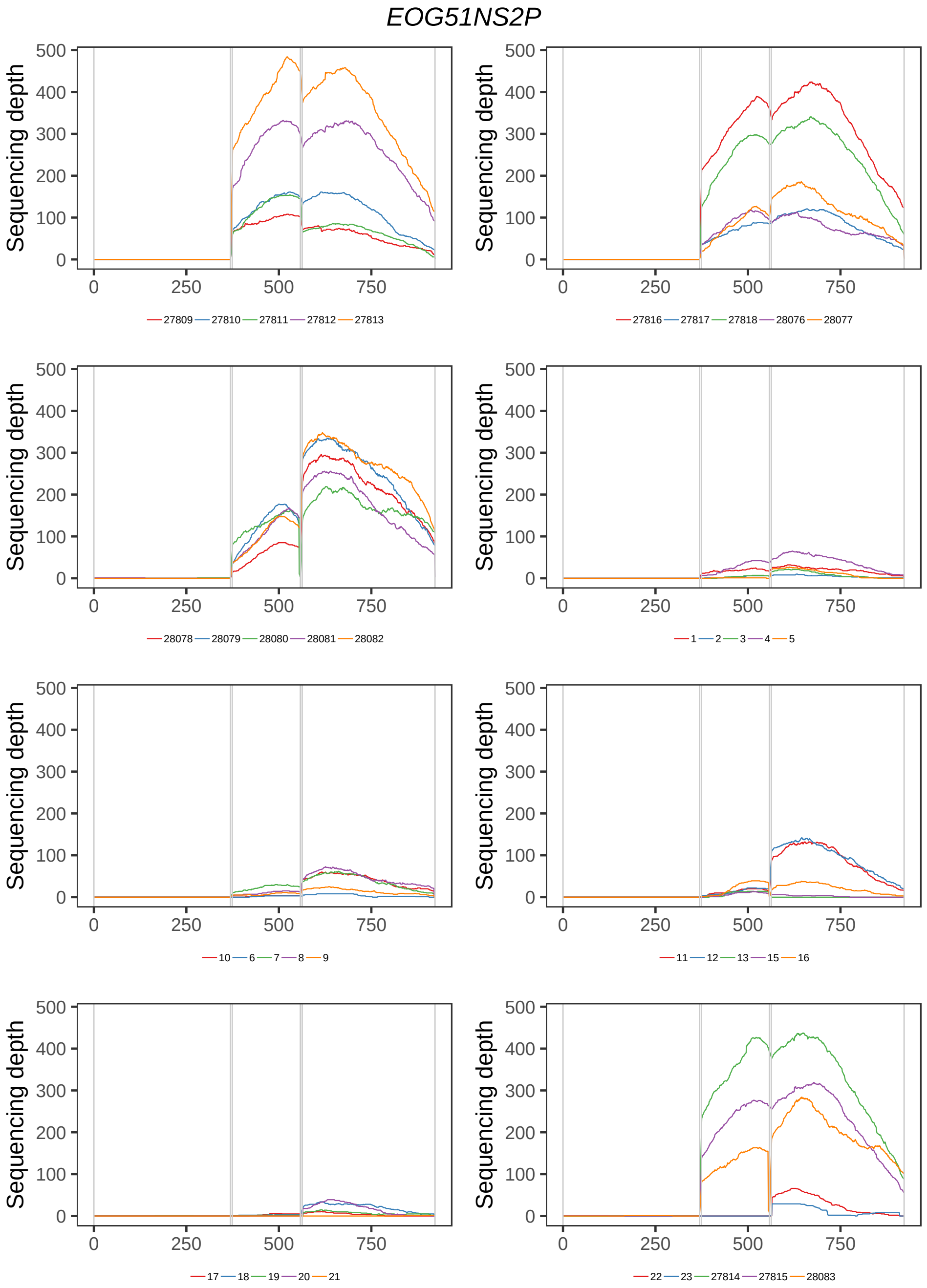
EOG51NS2P
Sequencing depth
27809 27810 27811 27812 27813
27816 27817 27818 28076 28077
28078 28079 28080 28081 28082
1 2 3 4 5
10 6 7 8 9
11 12 13 15 16
17 18 19 20 21
22 23 27814 27815 28083

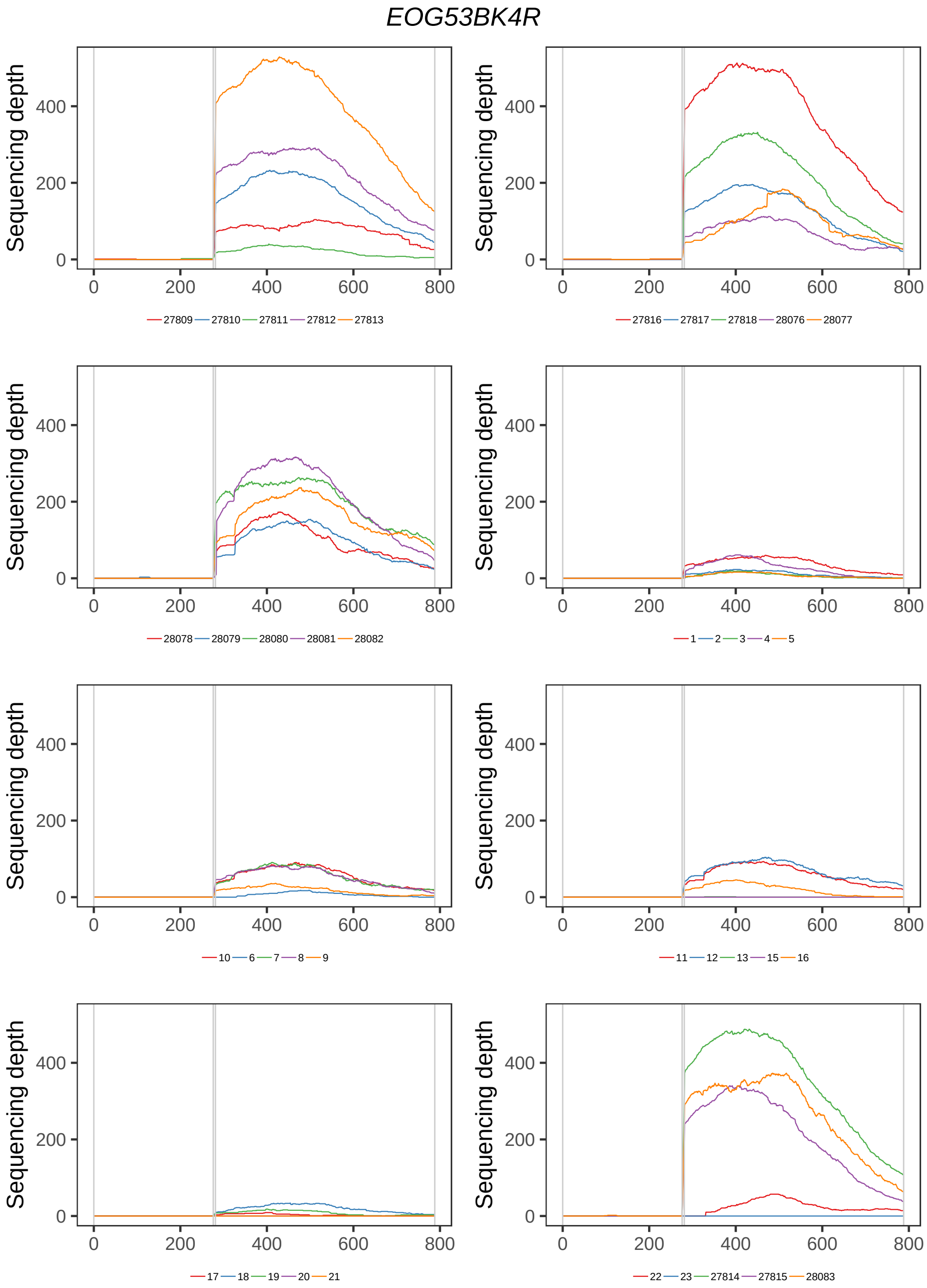
EOG53BK4R
Sequencing depth
0
200
400
600
800
27809 27810 27811 27812 27813
27816 27817 27818 28076 28077
28078 28079 28080 28081 28082
1 2 3 4 5
10 6 7 8 9
11 12 13 15 16
17 18 19 20 21
22 23 27814 27815 28083

EOG54QRGS

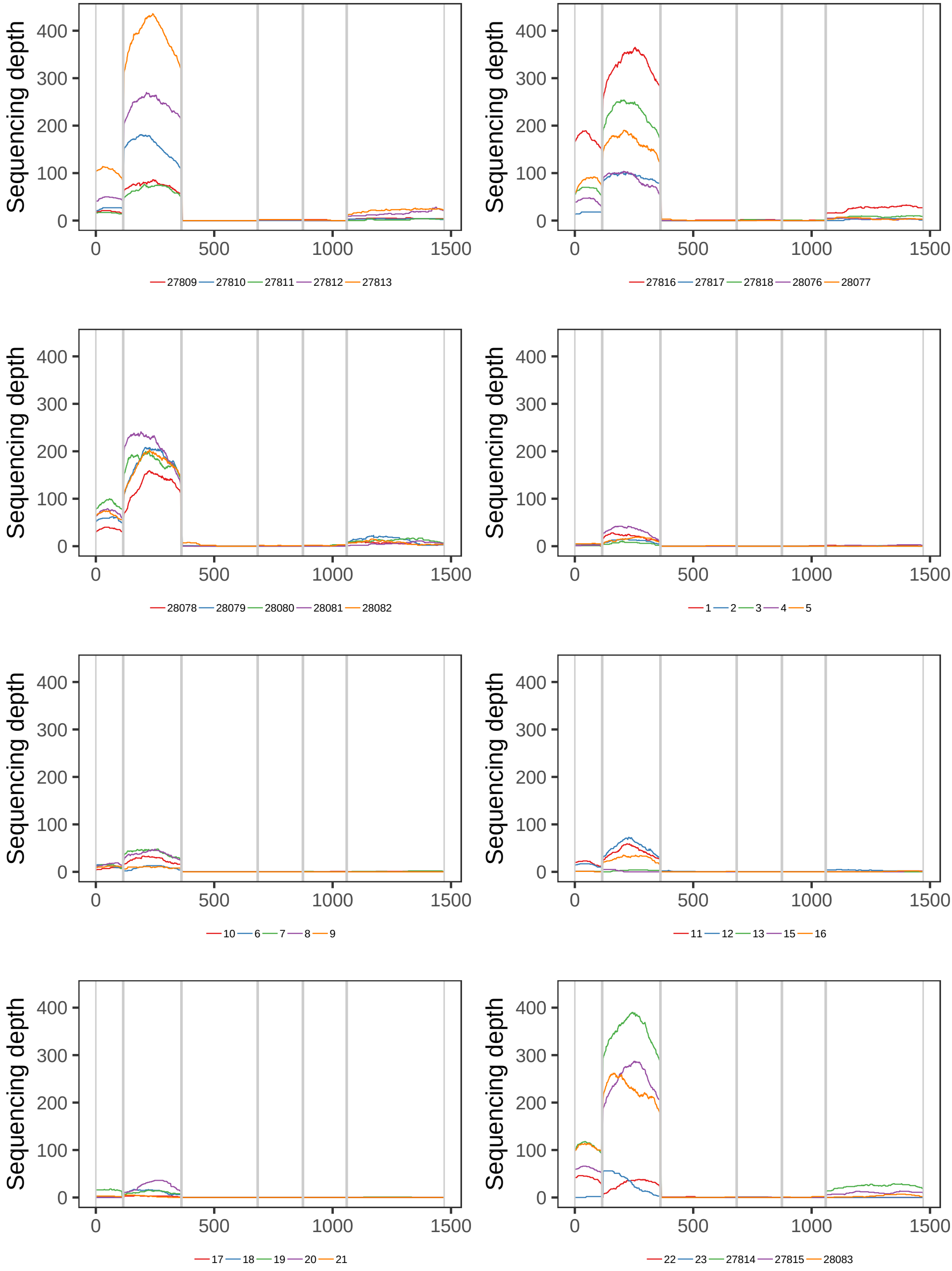
Sequencing depth
0
100
200
300
400
0
500
1000
1500
27809 27810 27811 27812 27813
27816 27817 27818 28076 28077
28078 28079 28080 28081 28082
1 2 3 4 5
10 6 7 8 9
11 12 13 15 16
17 18 19 20 21
22 23 27814 27815 28083

EOG56WWR4

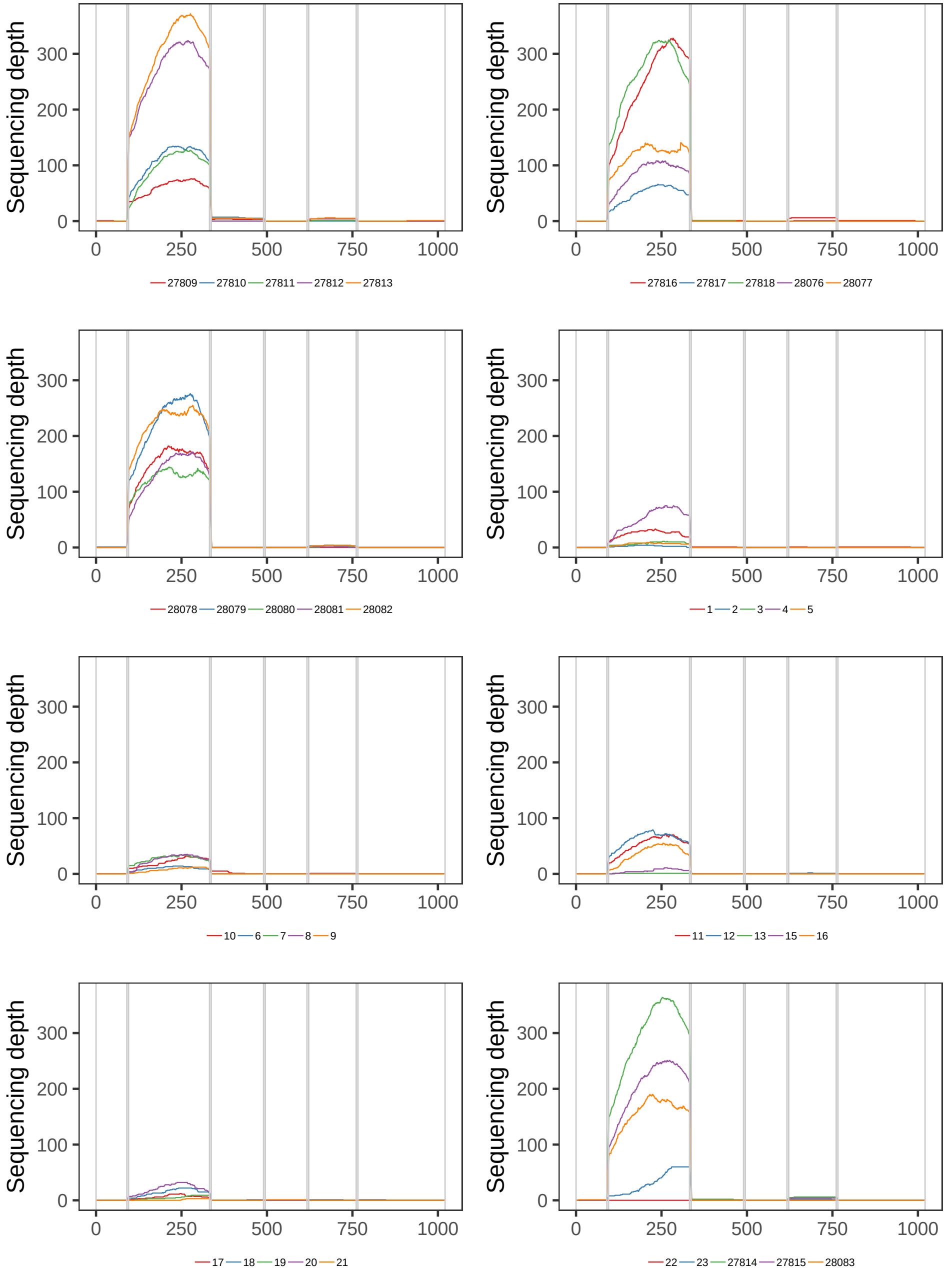
Sequencing depth
0
100
200
300
0
250
500
750
1000
27809 27810 27811 27812 27813
27816 27817 27818 28076 28077
28078 28079 28080 28081 28082
1 2 3 4 5
10 6 7 8 9
11 12 13 15 16
17 18 19 20 21
22 23 27814 27815 28083

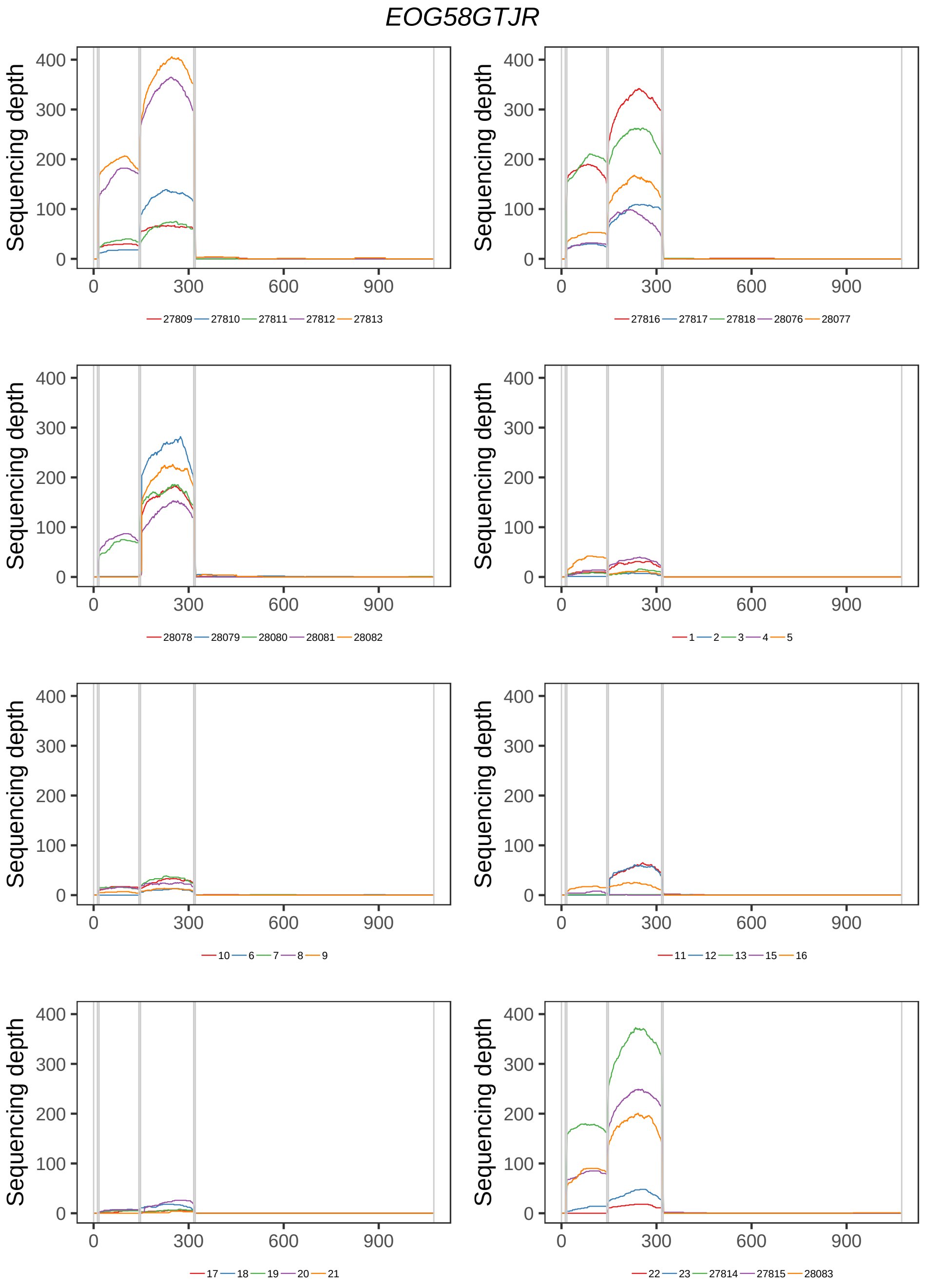
EOG58GTJR
Sequencing depth
0
100
200
300
400
0
300
600
900
27809 27810 27811 27812 27813
27816 27817 27818 28076 28077
28078 28079 28080 28081 28082
1 2 3 4 5
10 6 7 8 9
11 12 13 15 16
17 18 19 20 21
22 23 27814 27815 28083

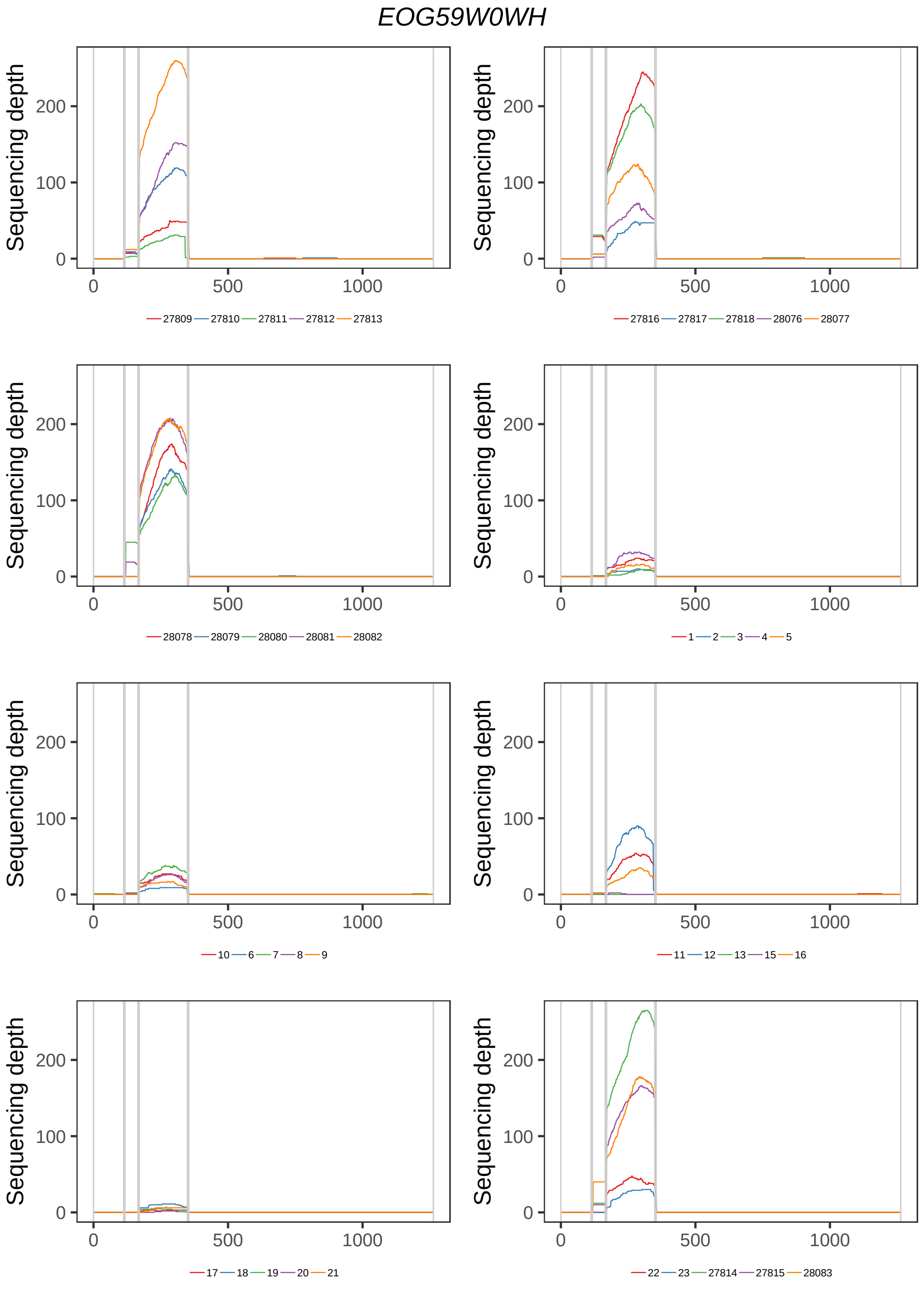

EOG59W0WH
Sequencing depth
0
100
200
0
500
1000
27809 27810 27811 27812 27813
27816 27817 27818 28076 28077
28078 28079 28080 28081 28082
1 2 3 4 5
10 6 7 8 9
11 12 13 15 16
17 18 19 20 21
22 23 27814 27815 28083

EOG5CC2H0

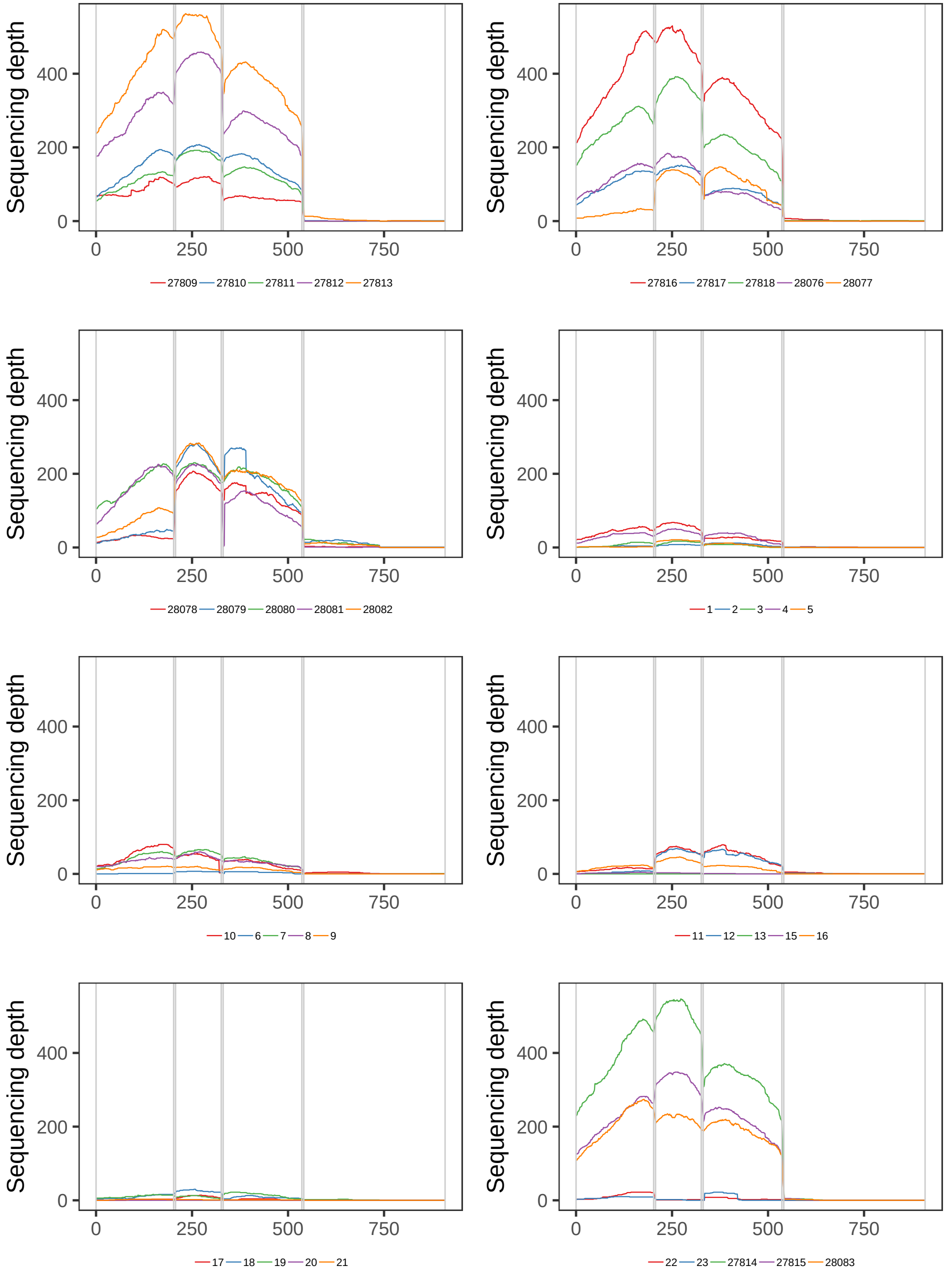

Sequencing depth
0
200
400
0
250
500
750
27809 27810 27811 27812 27813
27816 27817 27818 28076 28077
28078 28079 28080 28081 28082
1 2 3 4 5
10 6 7 8 9
11 12 13 15 16
17 18 19 20 21
22 23 27814 27815 28083

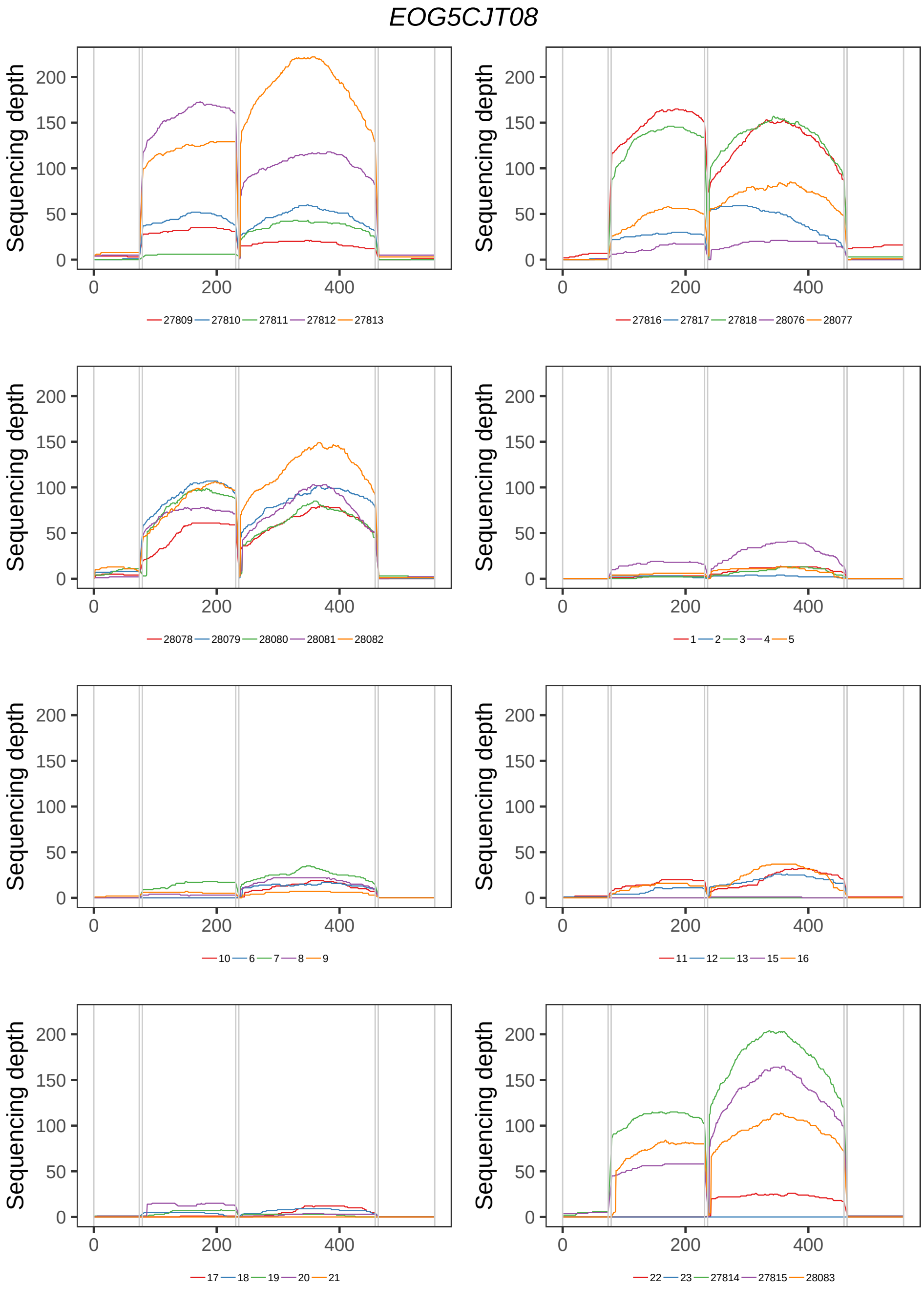

EOG5CJT08
Sequencing depth
0
50
100
150
200
0
200
400
27809
27810
27811
27812
27813
27816
27817
27818
28076
28077
28078
28079
28080
28081
28082
1
2
3
4
5
10
6
7
8
9
11
12
13
15
16
17
18
19
20
21
22
23
27814
27815
28083

EOG5HT77W

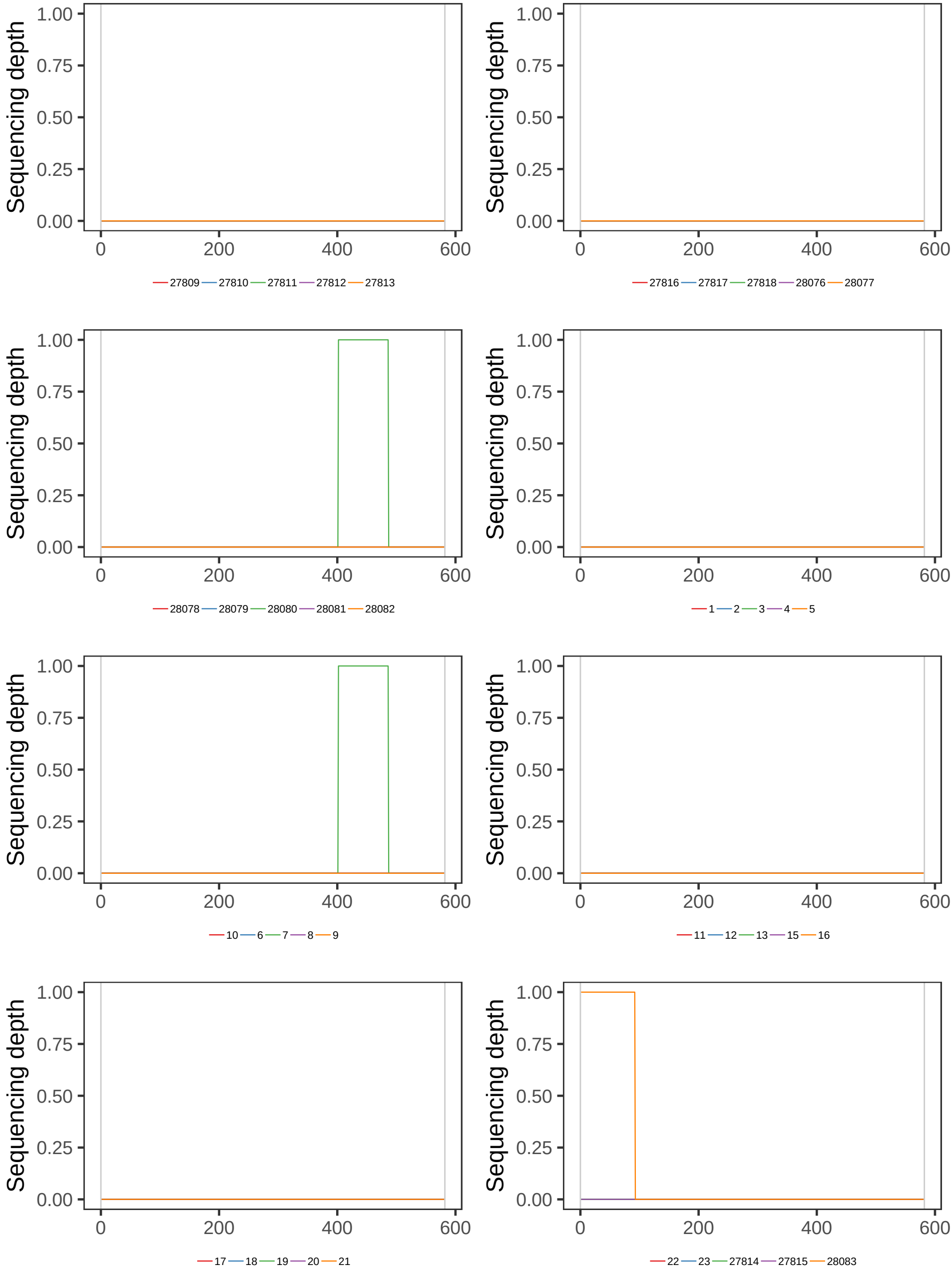
Sequencing depth
1.00
0.75
0.50
0.25
0.00
0
200
400
600
27809 27810 27811 27812 27813
27816 27817 27818 28076 28077
28078 28079 28080 28081 28082
1 2 3 4 5
10 6 7 8 9
11 12 13 15 16
17 18 19 20 21
22 23 27814 27815 28083

EOG5PVMDN

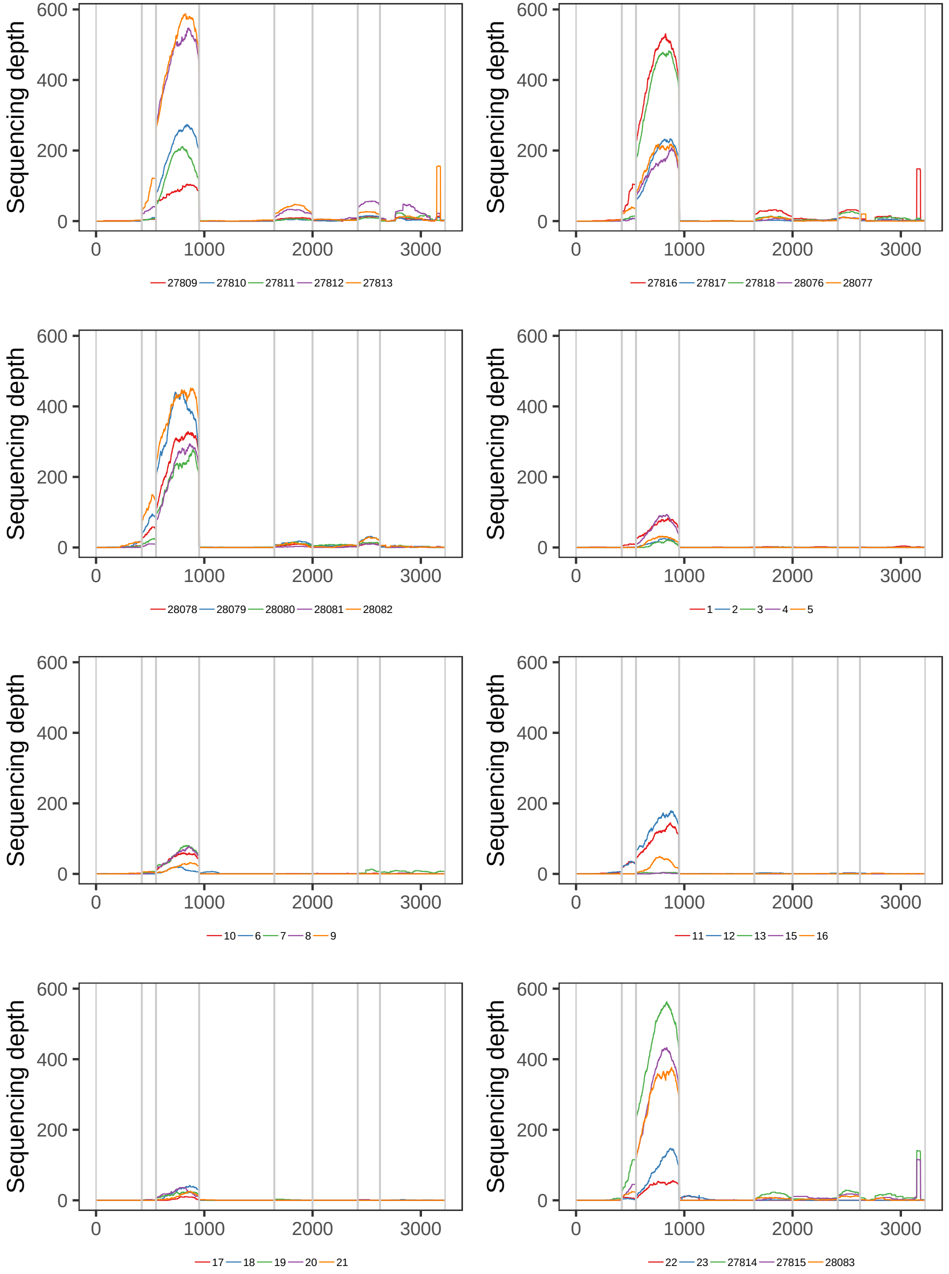
Sequencing depth
600
400
200
0
0
1000
2000
3000
27809 27810 27811 27812 27813
27816 27817 27818 28076 28077
28078 28079 28080 28081 28082
1 2 3 4 5
10 6 7 8 9
11 12 13 15 16
17 18 19 20 21
22 23 27814 27815 28083

EOG5R229S

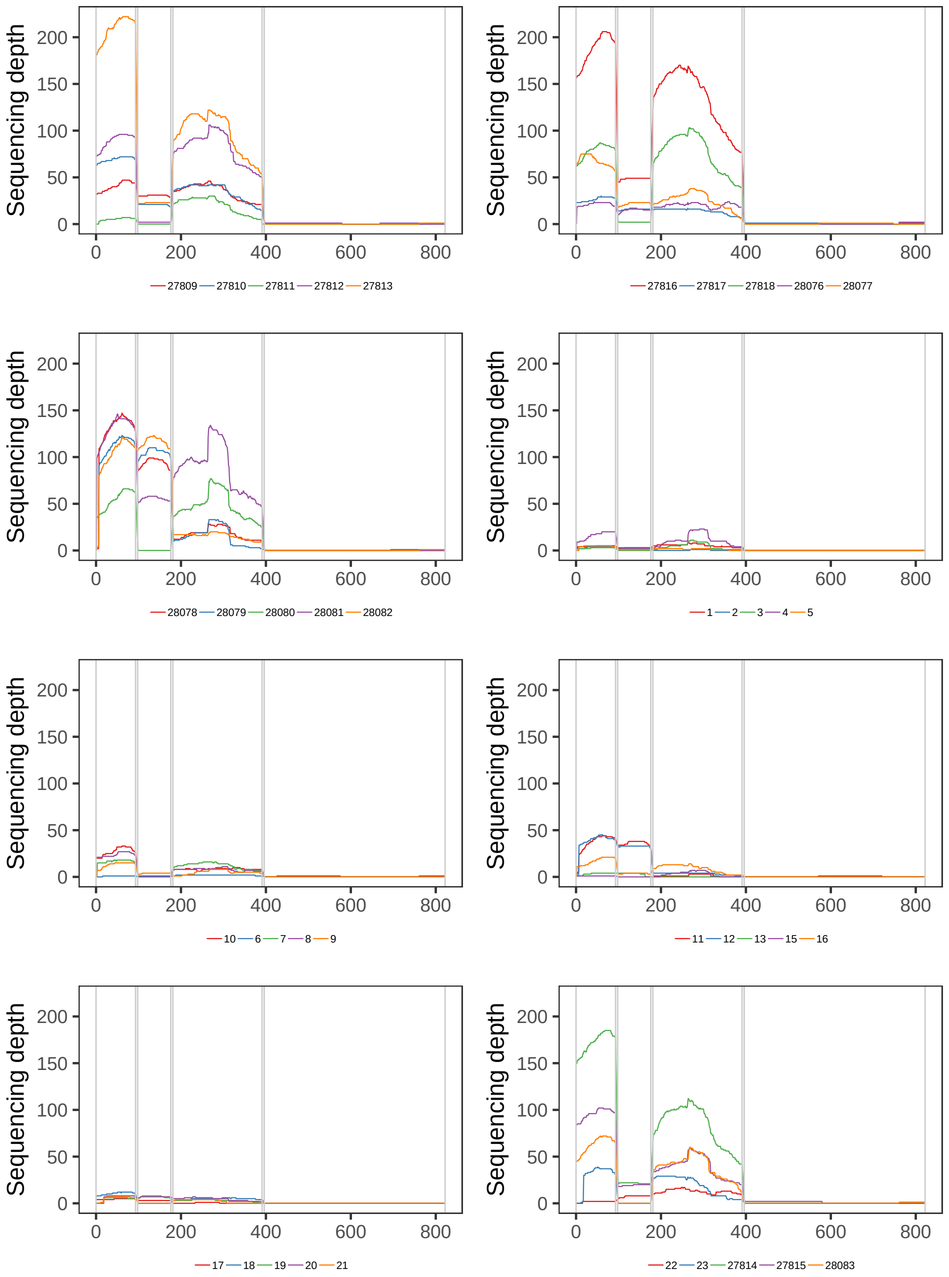

Sequencing depth
0
50
100
150
200
0
200
400
600
800
27809
27810
27811
27812
27813
27816
27817
27818
28076
28077
28078
28079
28080
28081
28082
1
2
3
4
5
10
6
7
8
9
11
12
13
15
16
17
18
19
20
21
22
23
27814
27815
28083

EOG5V9S5X

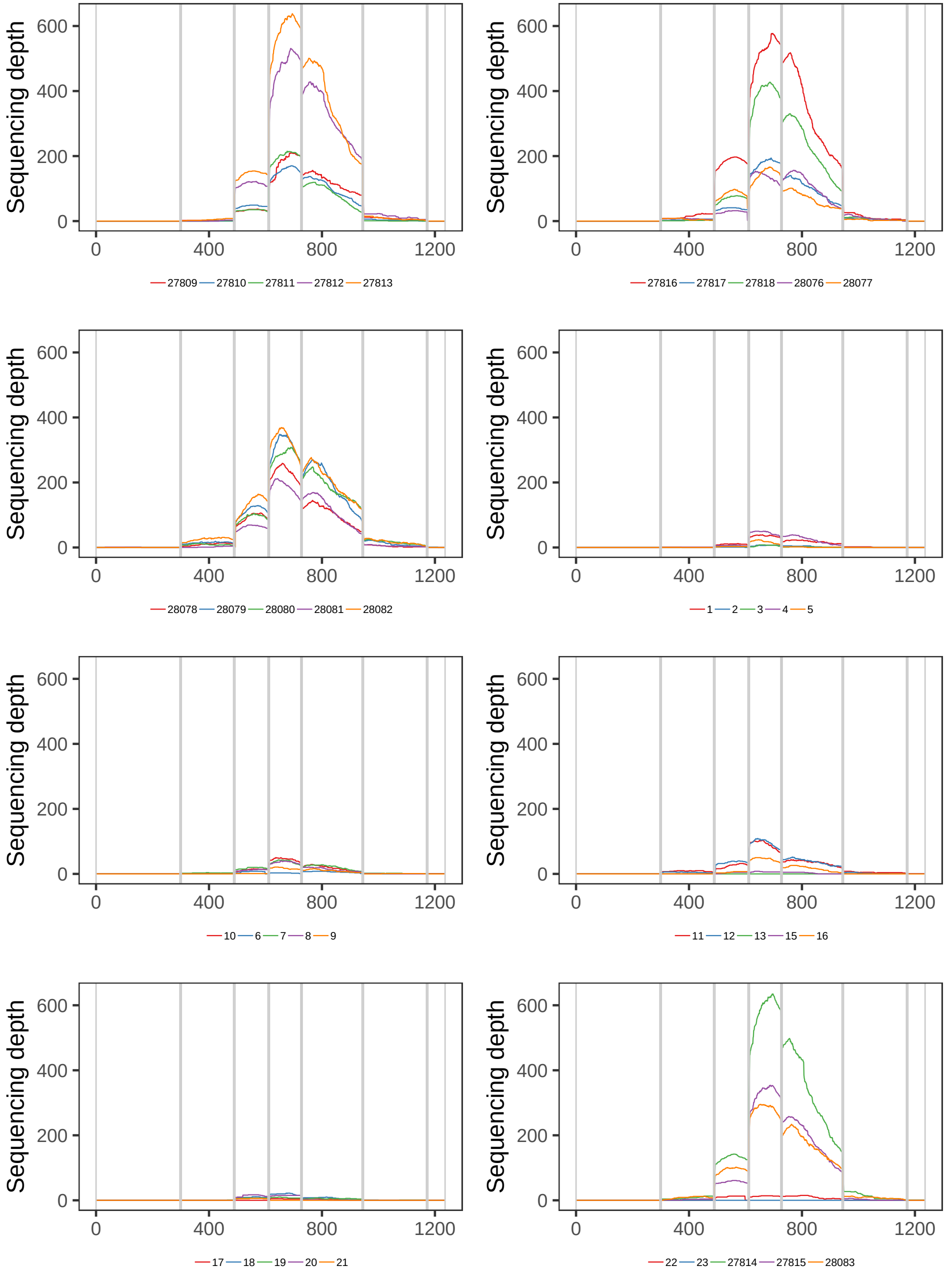
Sequencing depth
600
400
200
0
0
400
800
1200
27809 27810 27811 27812 27813
27816 27817 27818 28076 28077
28078 28079 28080 28081 28082
1 2 3 4 5
10 6 7 8 9
11 12 13 15 16
17 18 19 20 21
22 23 27814 27815 28083

EOG5V9S6M

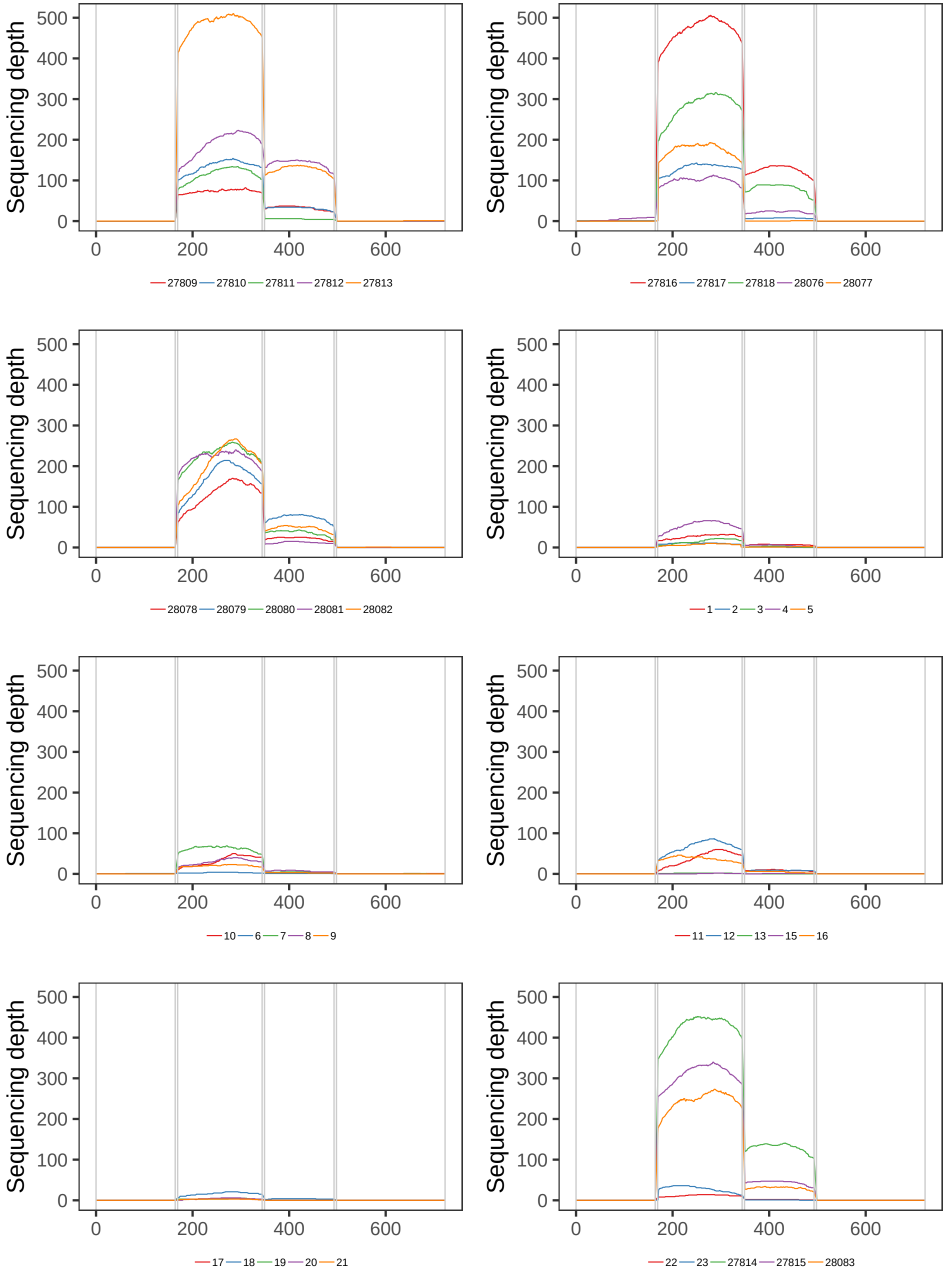

Sequencing depth
500
400
300
200
100
0
0
200
400
600
27809 27810 27811 27812 27813
27816 27817 27818 28076 28077
28078 28079 28080 28081 28082
1 2 3 4 5
10 6 7 8 9
11 12 13 15 16
17 18 19 20 21
22 23 27814 27815 28083

EOG5VT4C7

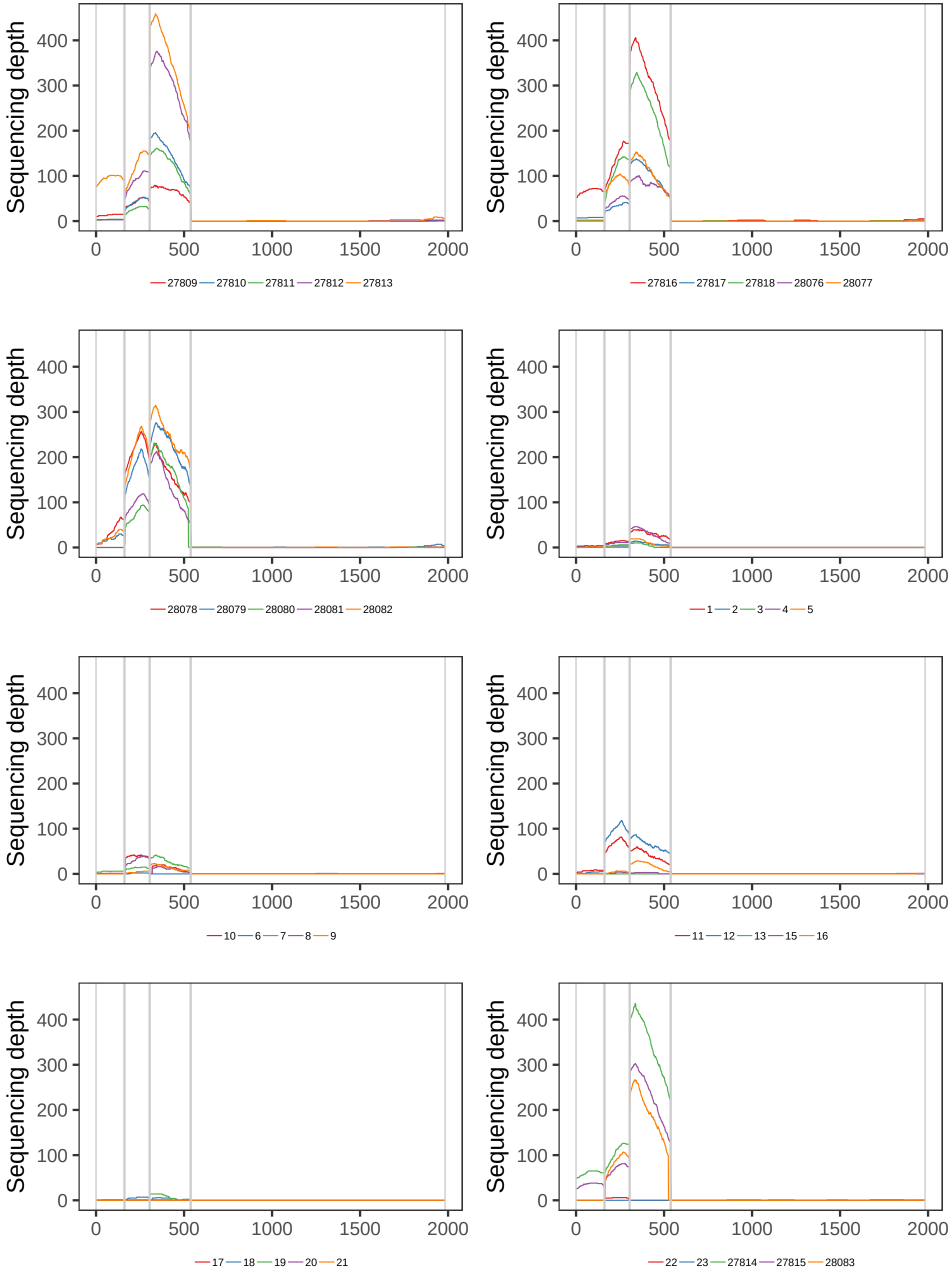
Sequencing depth
0
100
200
300
400
0
500
1000
1500
2000
27809 27810 27811 27812 27813
27816 27817 27818 28076 28077
28078 28079 28080 28081 28082
1 2 3 4 5
10 6 7 8 9
11 12 13 15 16
17 18 19 20 21
22 23 27814 27815 28083

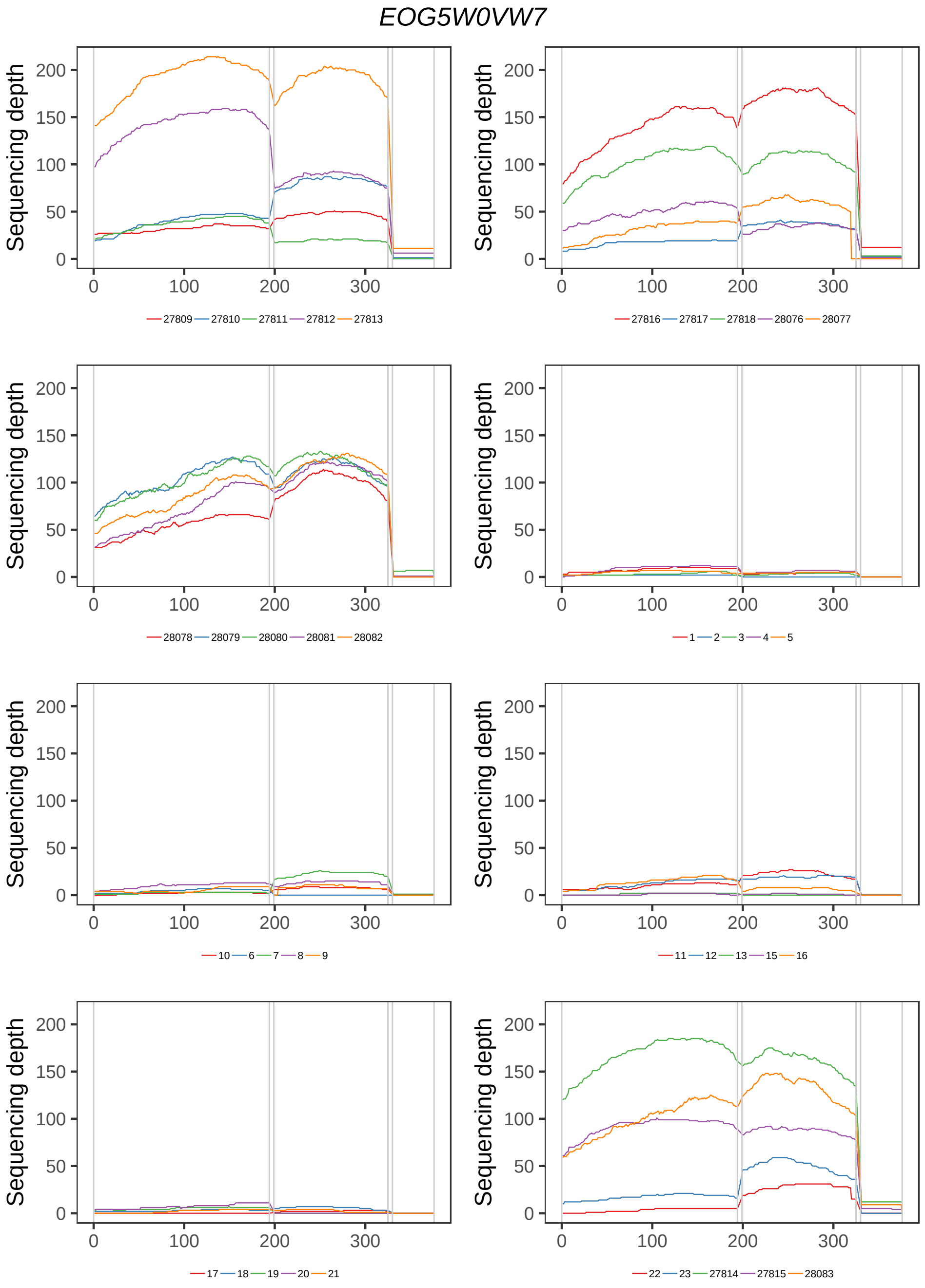
EOG5W0VW7
Sequencing depth
27809 27810 27811 27812 27813
27816 27817 27818 28076 28077
28078 28079 28080 28081 28082
1 2 3 4 5
10 6 7 8 9
11 12 13 15 16
17 18 19 20 21
22 23 27814 27815 28083

EOG5WM39H

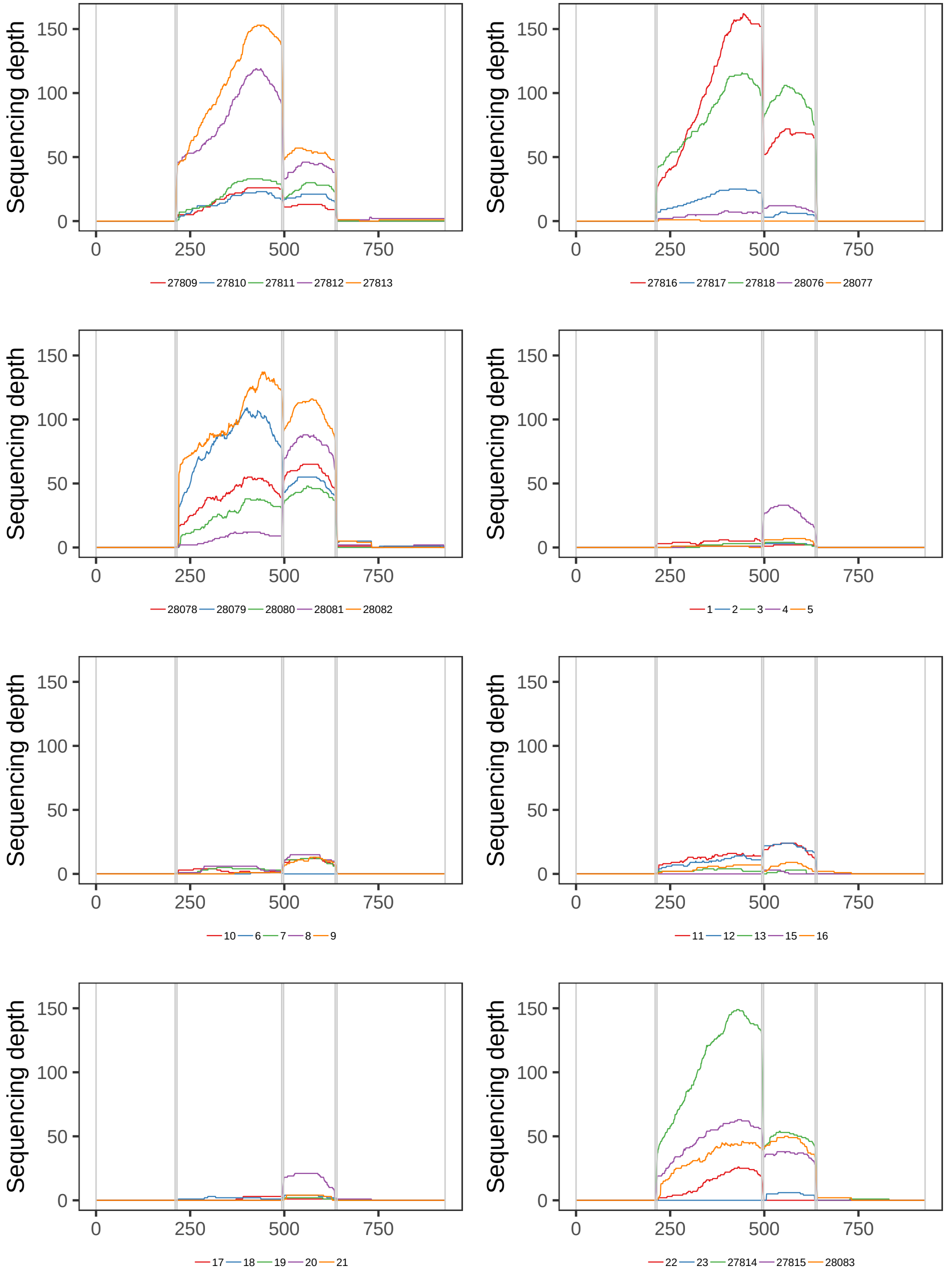

Sequencing depth
150
100
50
0
0
250
500
750
27809 27810 27811 27812 27813
27816 27817 27818 28076 28077
28078 28079 28080 28081 28082
1 2 3 4 5
10 6 7 8 9
11 12 13 15 16
17 18 19 20 21
22 23 27814 27815 28083

EOG5WPZHS

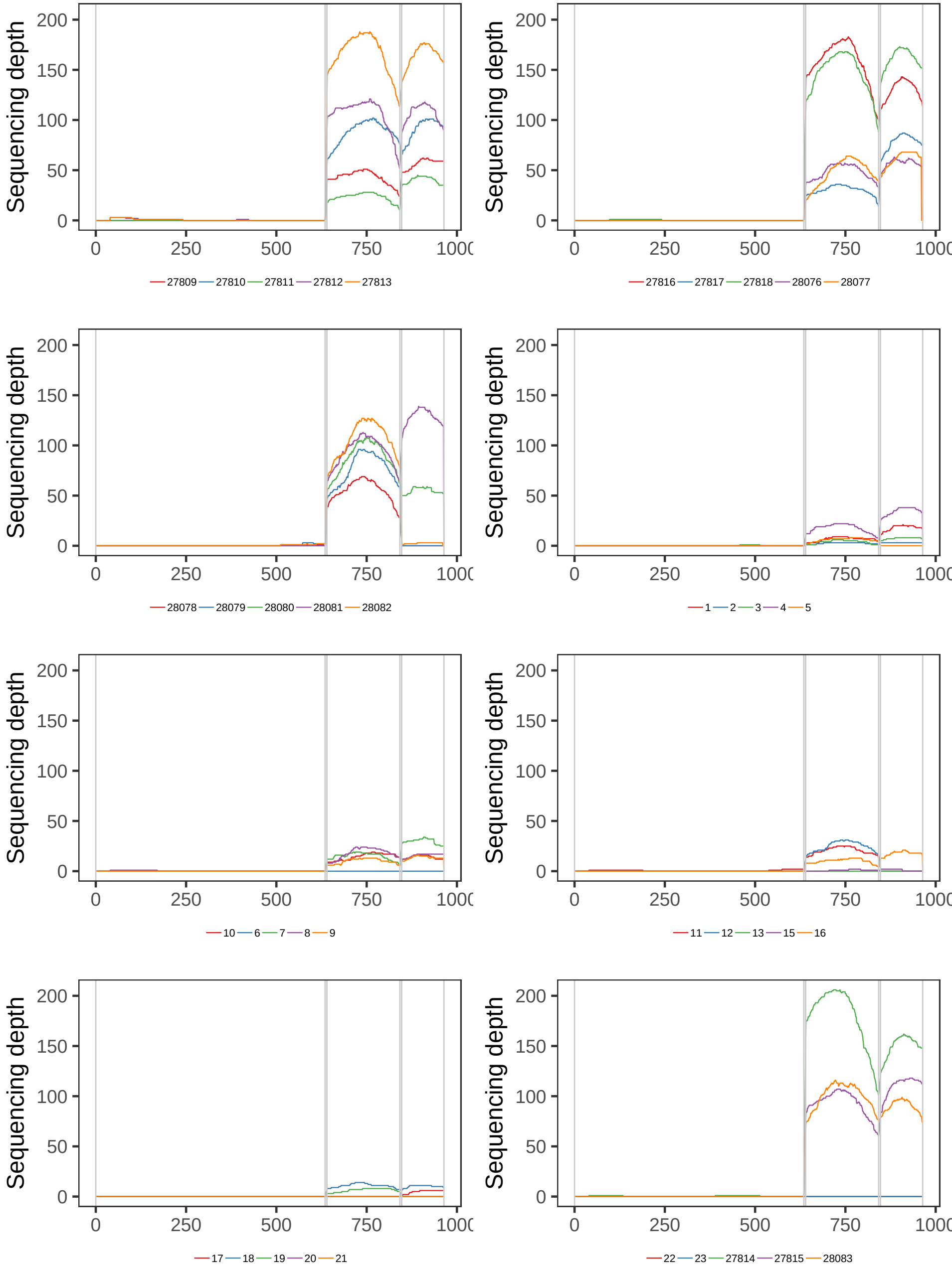

Sequencing depth
0
50
100
150
200
0
250
500
750
1000
27809 27810 27811 27812 27813
27816 27817 27818 28076 28077
28078 28079 28080 28081 28082
1 2 3 4 5
10 6 7 8 9
11 12 13 15 16
17 18 19 20 21
22 23 27814 27815 28083

EOG5ZGMTV

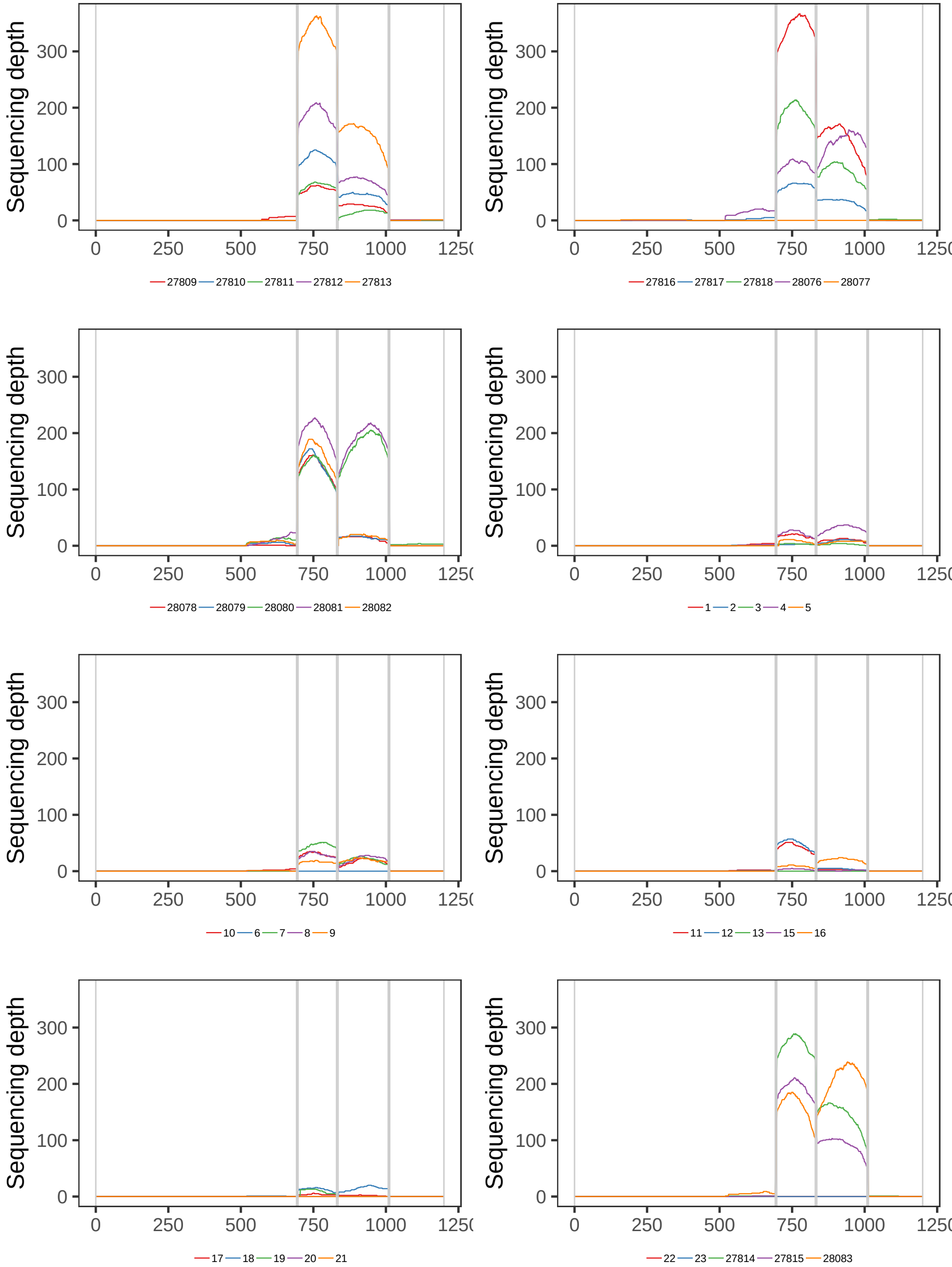
Sequencing depth
0
100
200
300
0
250
500
750
1000
1250
27809
27810
27811
27812
27813
27816
27817
27818
28076
28077
28078
28079
28080
28081
28082
1
2
3
4
5
10
6
7
8
9
11
12
13
15
16
17
18
19
20
21
22
23
27814
27815
28083

EOG512JN5

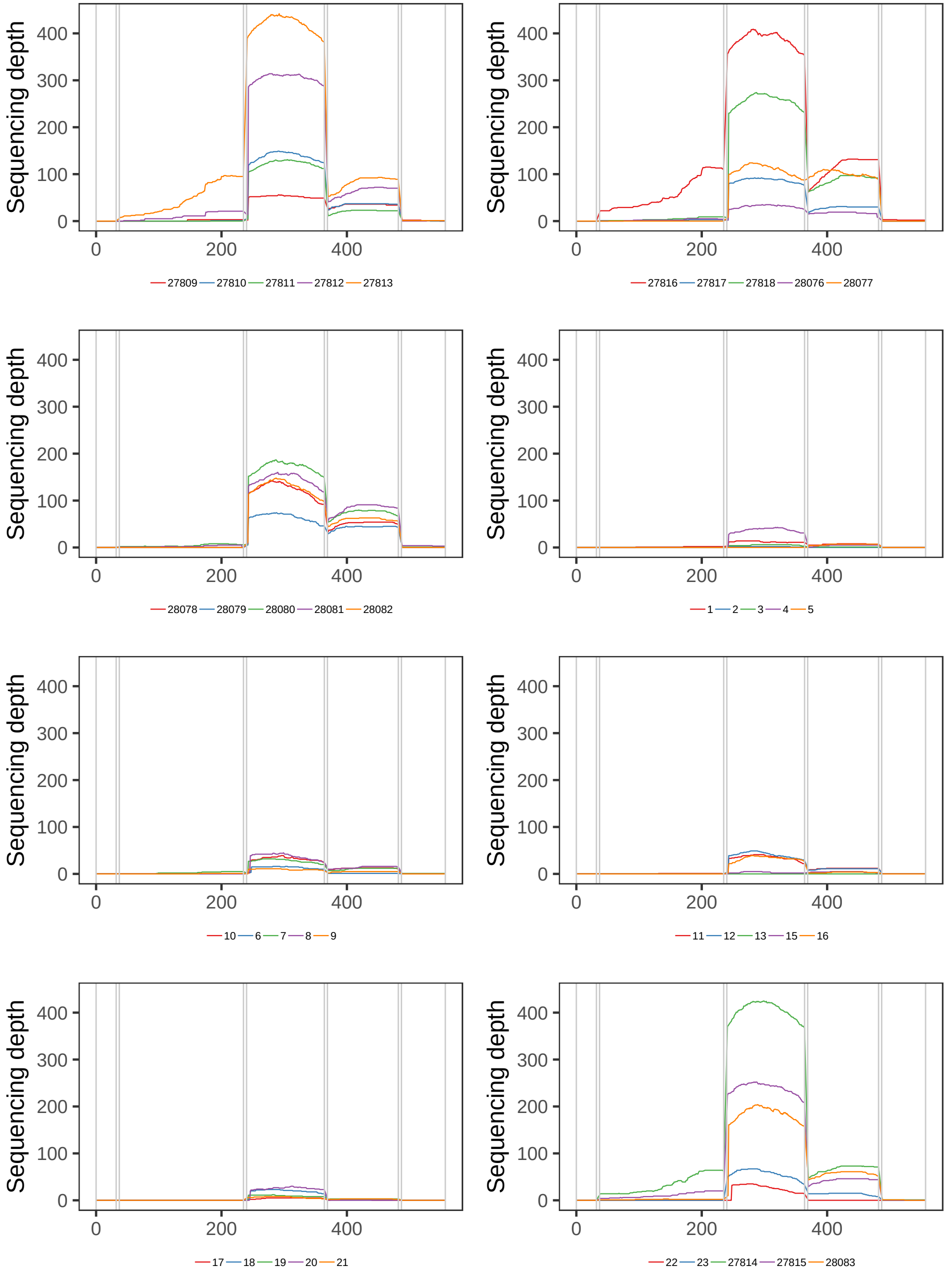
Sequencing depth
0
100
200
300
400
0
200
400
27809 27810 27811 27812 27813
27816 27817 27818 28076 28077
28078 28079 28080 28081 28082
1 2 3 4 5
10 6 7 8 9
11 12 13 15 16
17 18 19 20 21
22 23 27814 27815 28083

EOG54B8HX

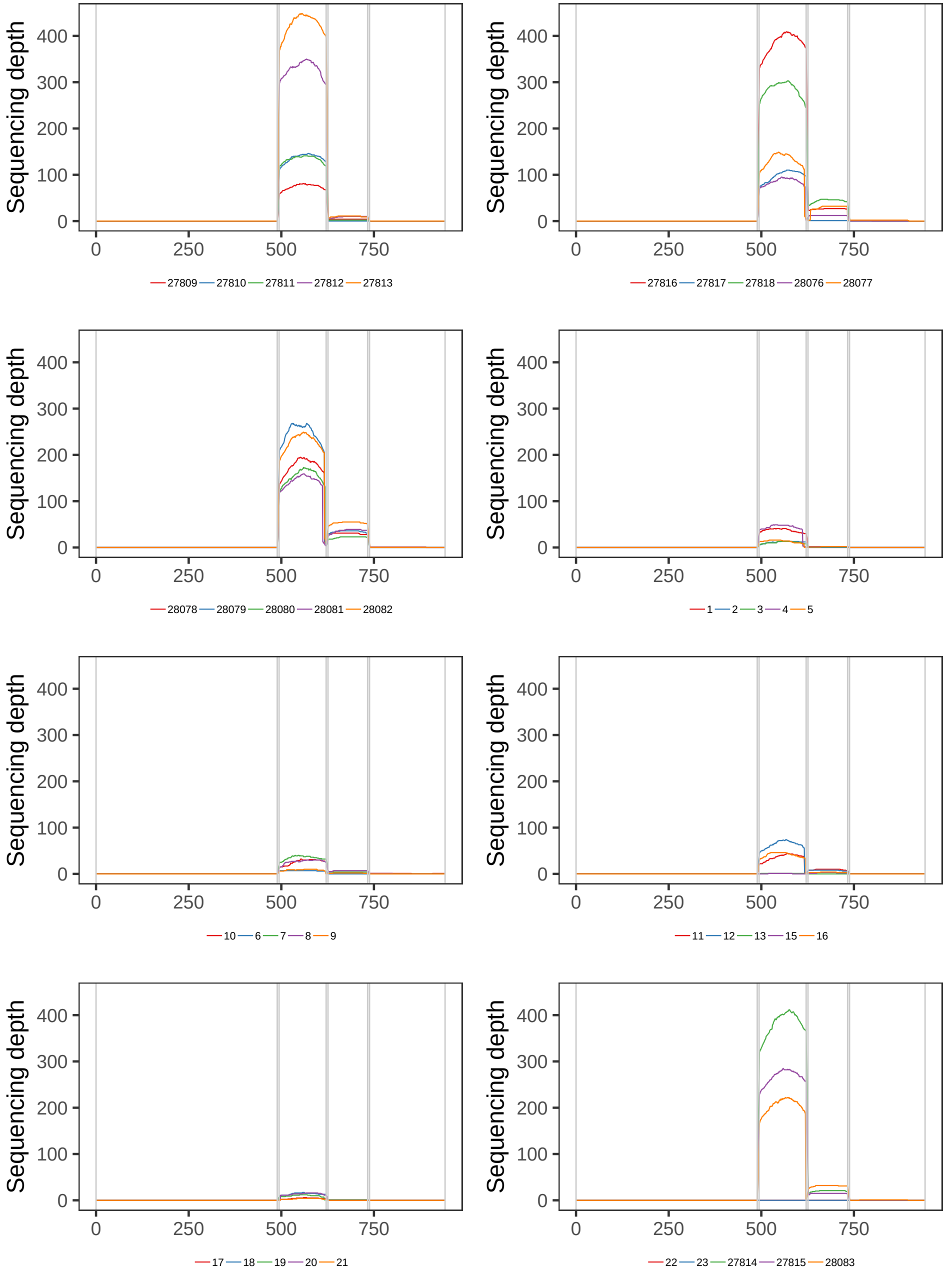

Sequencing depth
0
100
200
300
400
0
250
500
750
27809
27810
27811
27812
27813
27816
27817
27818
28076
28077
28078
28079
28080
28081
28082
1
2
3
4
5
10
6
7
8
9
11
12
13
15
16
17
18
19
20
21
22
23
27814
27815
28083

EOG55DV67

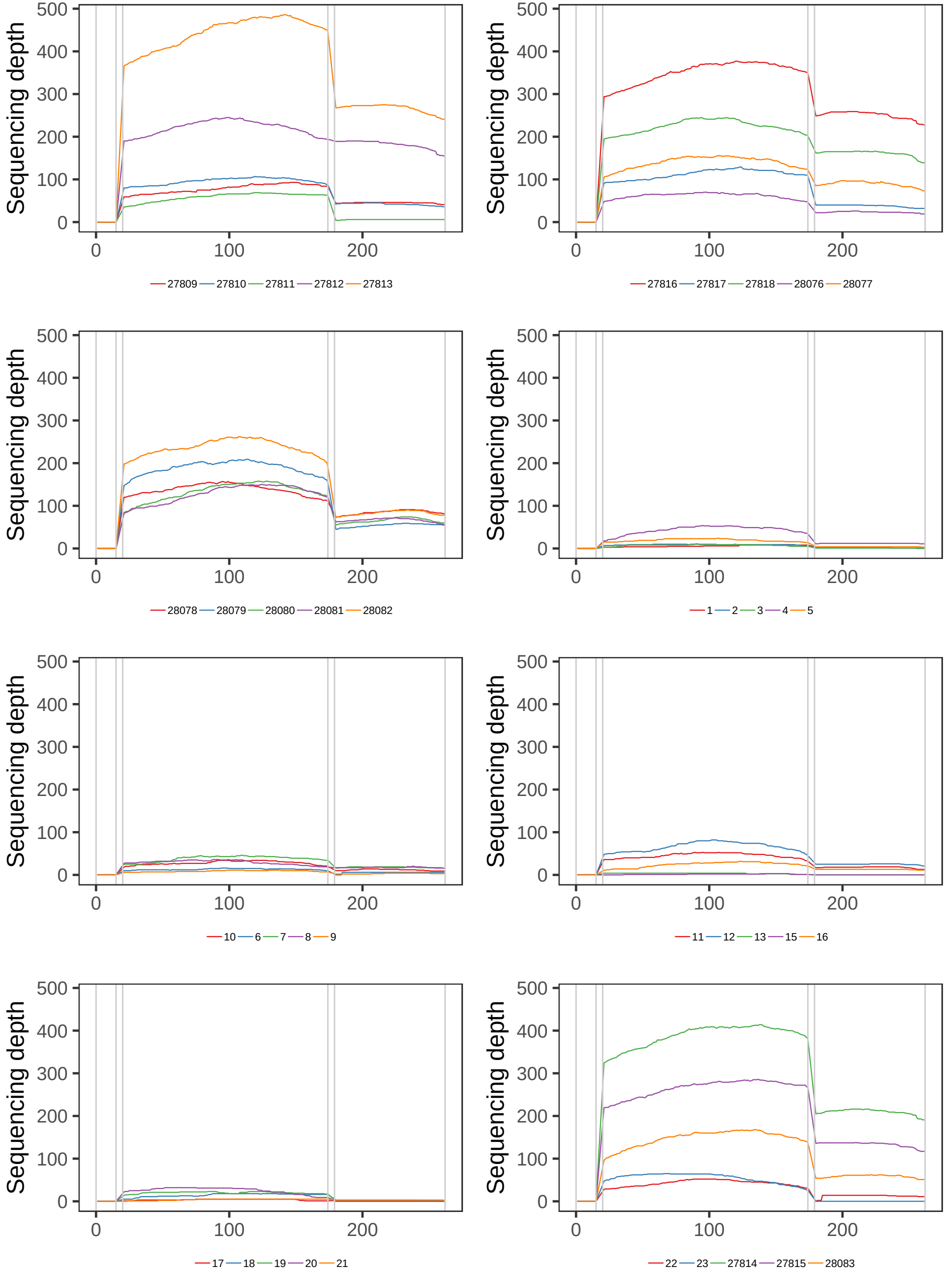
Sequencing depth
0
100
200
300
400
500
0
100
200
27809 27810 27811 27812 27813
27816 27817 27818 28076 28077
28078 28079 28080 28081 28082
1 2 3 4 5
10 6 7 8 9
11 12 13 15 16
17 18 19 20 21
22 23 27814 27815 28083

EOG59320H

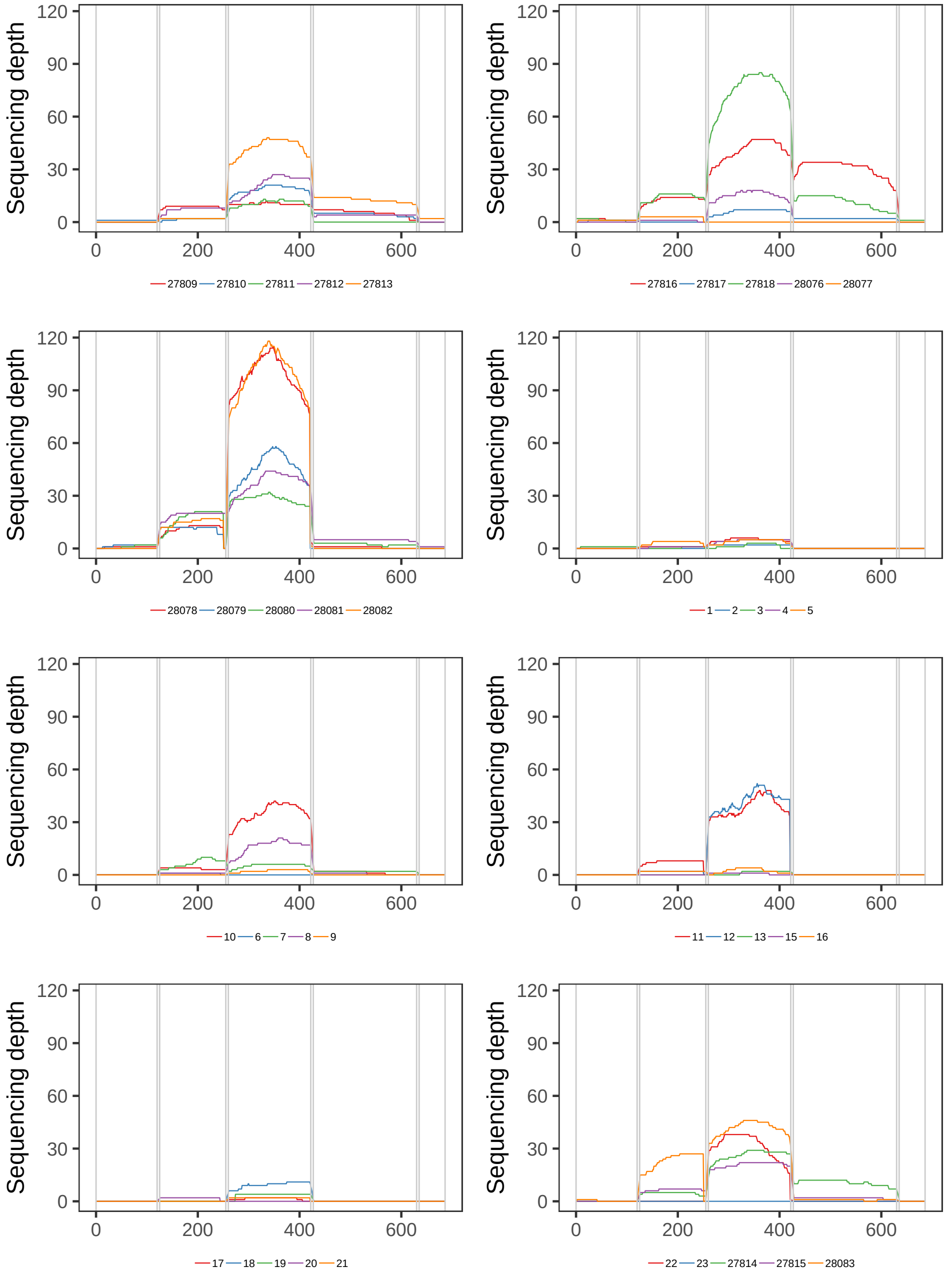
Sequencing depth
0
30
60
90
120
0
200
400
600
27809 27810 27811 27812 27813
27816 27817 27818 28076 28077
28078 28079 28080 28081 28082
1 2 3 4 5
10 6 7 8 9
11 12 13 15 16
17 18 19 20 21
22 23 27814 27815 28083

EOG5CNP6H

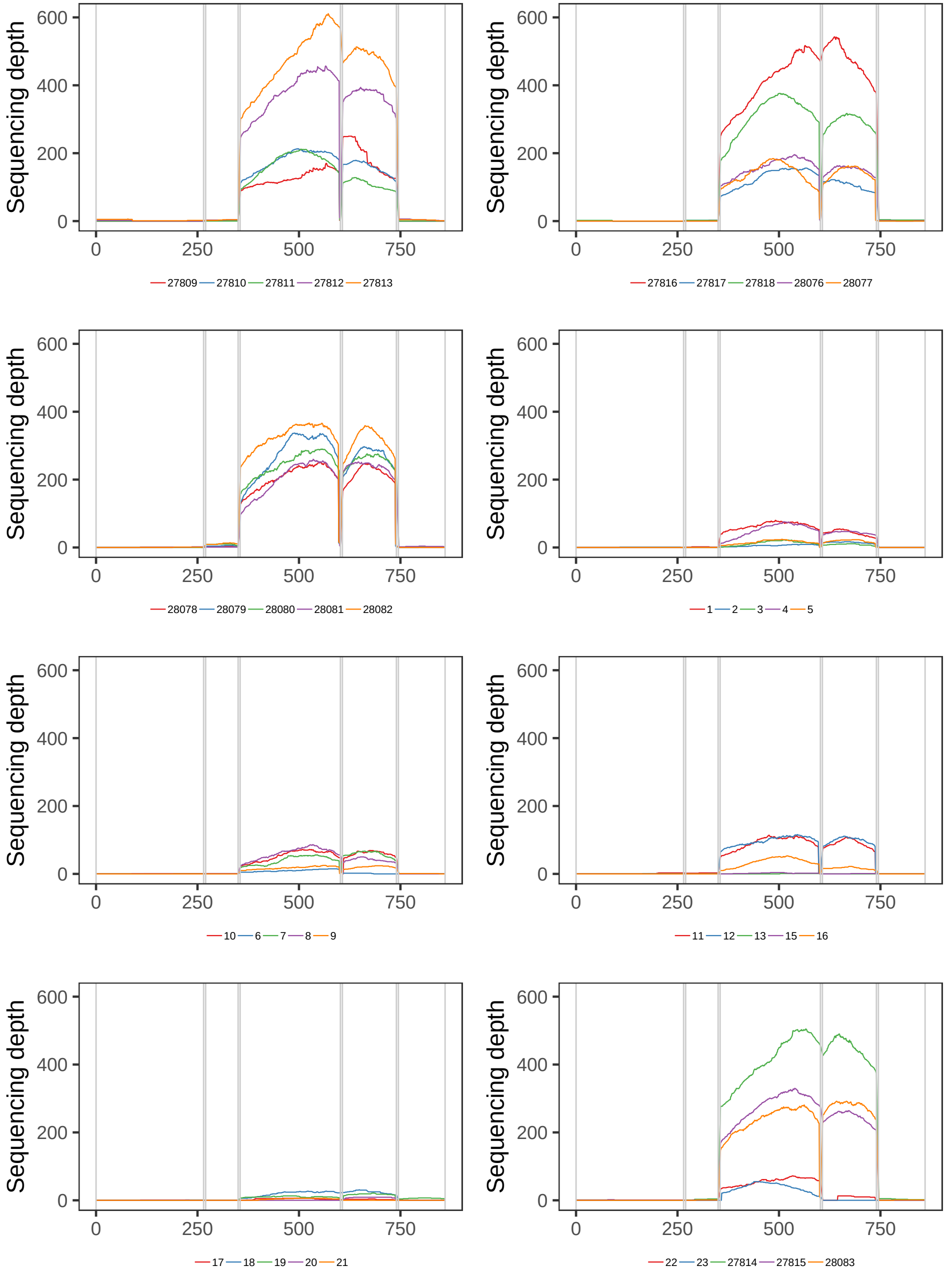
Sequencing depth
600
400
200
0
0
250
500
750
27809 27810 27811 27812 27813
27816 27817 27818 28076 28077
28078 28079 28080 28081 28082
1 2 3 4 5
10 6 7 8 9
11 12 13 15 16
17 18 19 20 21
22 23 27814 27815 28083

EOG5FJ6RG

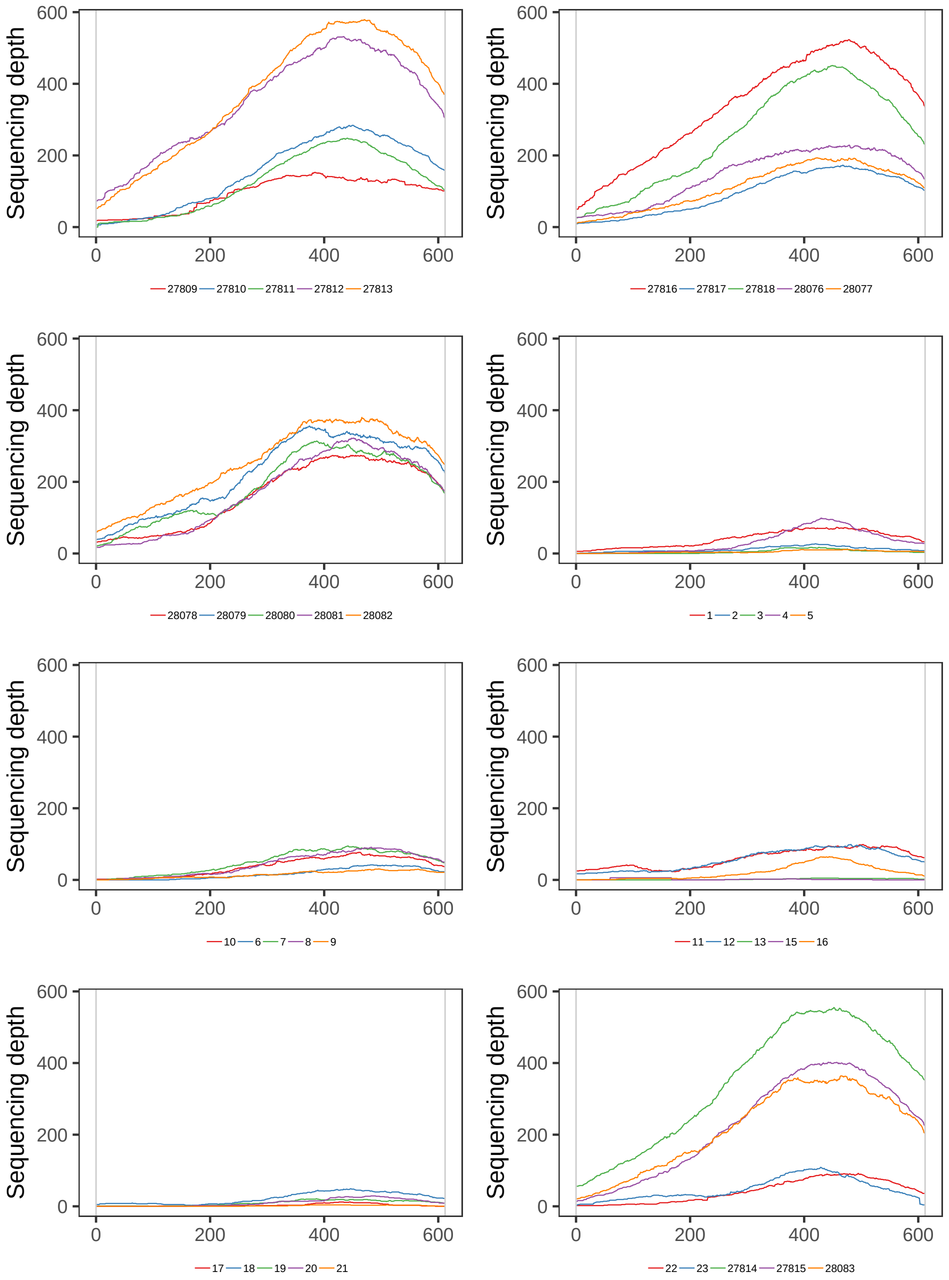
Sequencing depth
0
200
400
600
27809 27810 27811 27812 27813
27816 27817 27818 28076 28077
28078 28079 28080 28081 28082
1 2 3 4 5
10 6 7 8 9
11 12 13 15 16
17 18 19 20 21
22 23 27814 27815 28083

EOG5H9W34

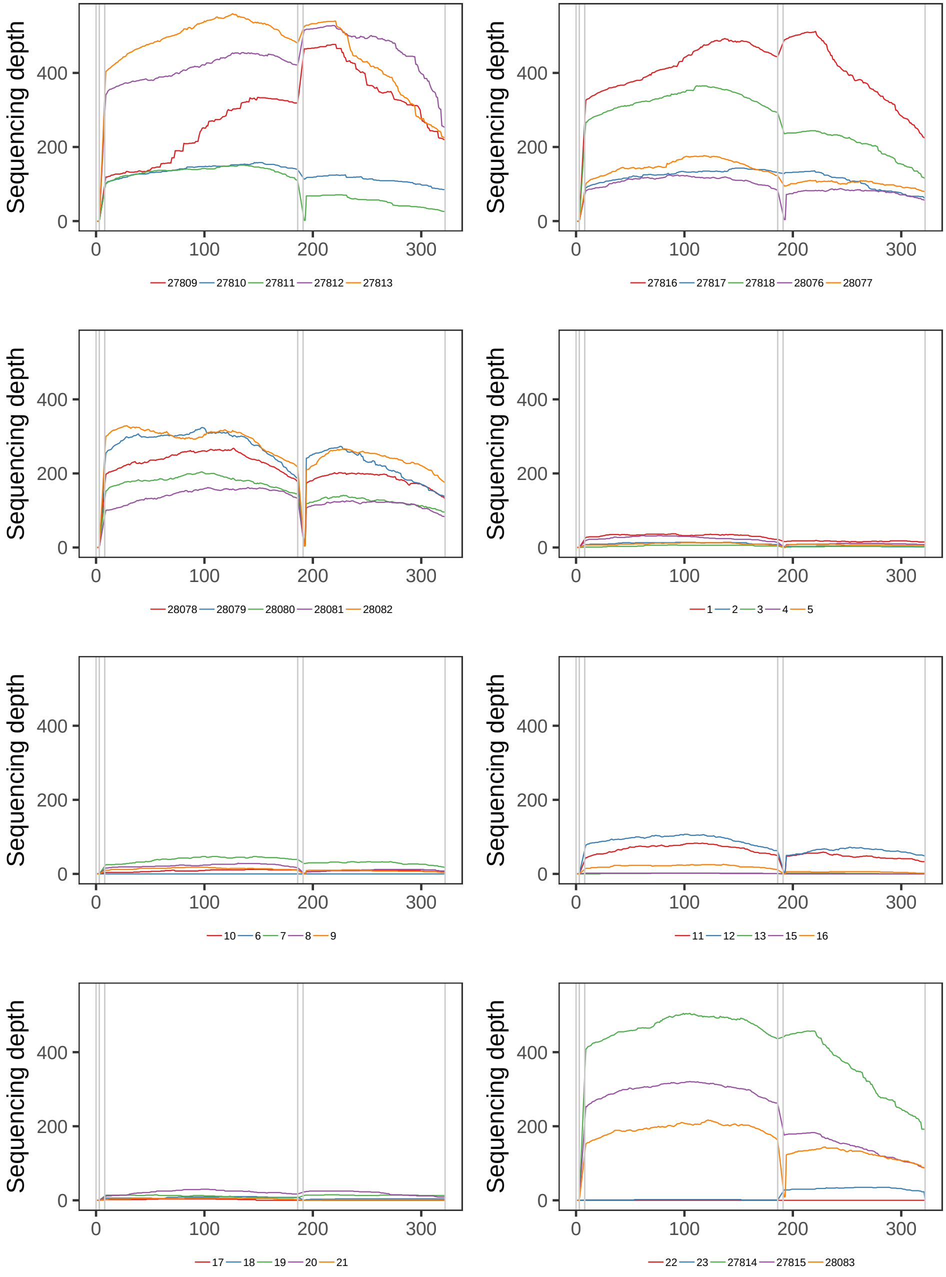

Sequencing depth
0
200
400
0
100
200
300
27809
27810
27811
27812
27813
27816
27817
27818
28076
28077
28078
28079
28080
28081
28082
1
2
3
4
5
10
6
7
8
9
11
12
13
15
16
17
18
19
20
21
22
23
27814
27815
28083

EOG5JQ2D1

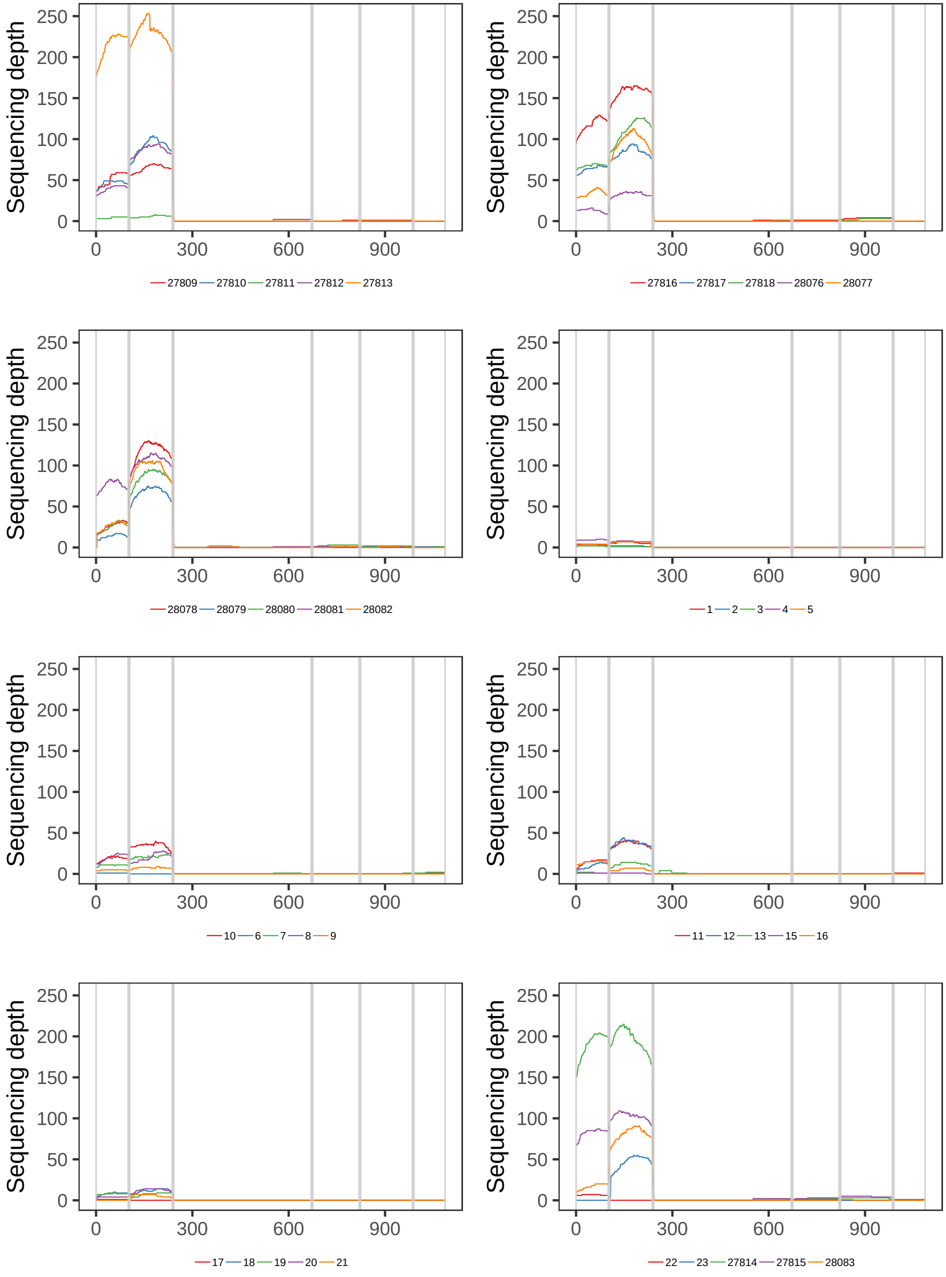
Sequencing depth
250
200
150
100
50
0
300
600
900
27809 27810 27811 27812 27813
27816 27817 27818 28076 28077
28078 28079 28080 28081 28082
1 2 3 4 5
10 6 7 8 9
11 12 13 15 16
17 18 19 20 21
22 23 27814 27815 28083

EOG5Q2BWR

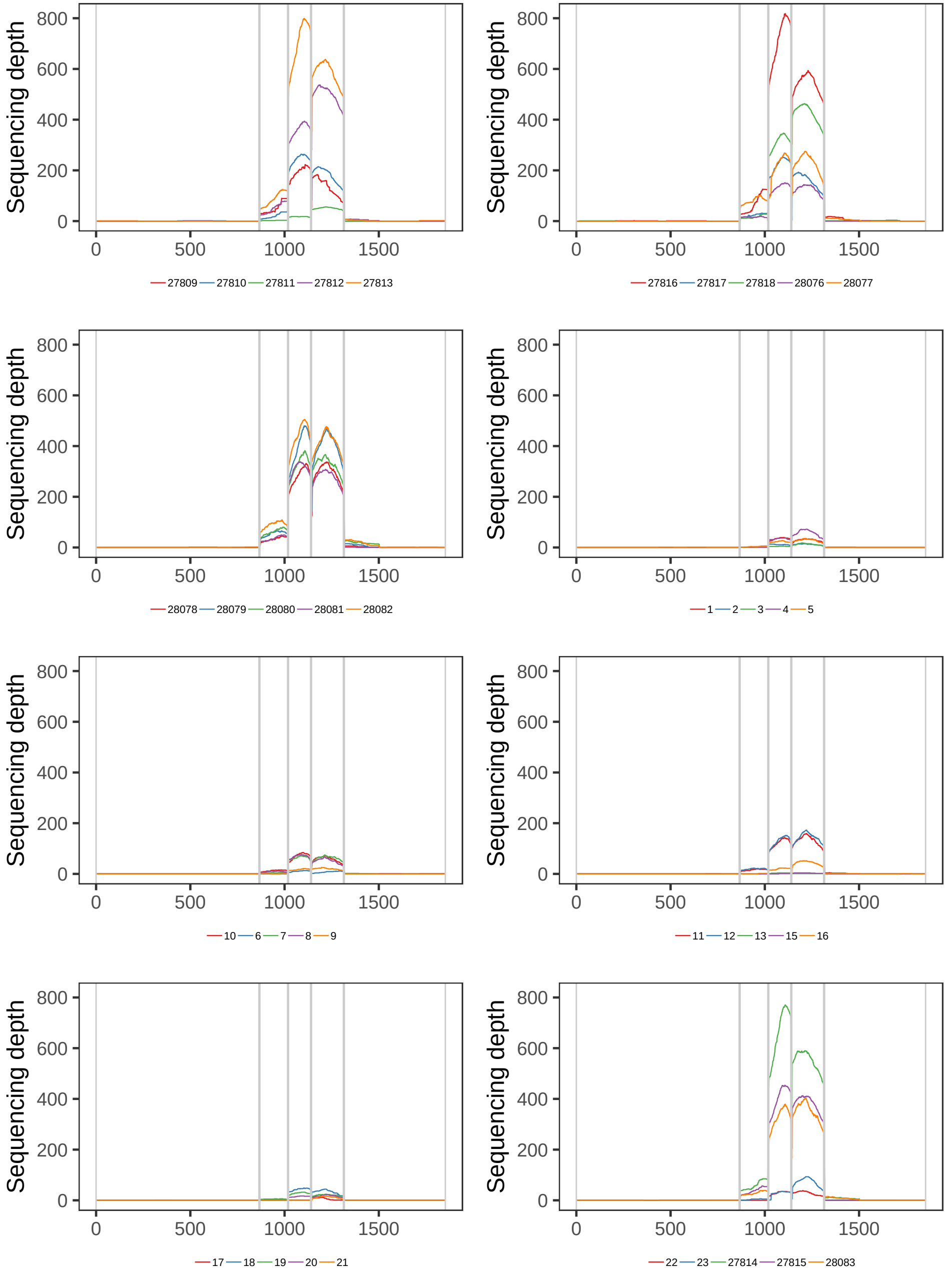
Sequencing depth
0
200
400
600
800
0
500
1000
1500
27809
27810
27811
27812
27813
27816
27817
27818
28076
28077
28078
28079
28080
28081
28082
1
2
3
4
5
10
6
7
8
9
11
12
13
15
16
17
18
19
20
21
22
23
27814
27815
28083

EOG5R7SRV

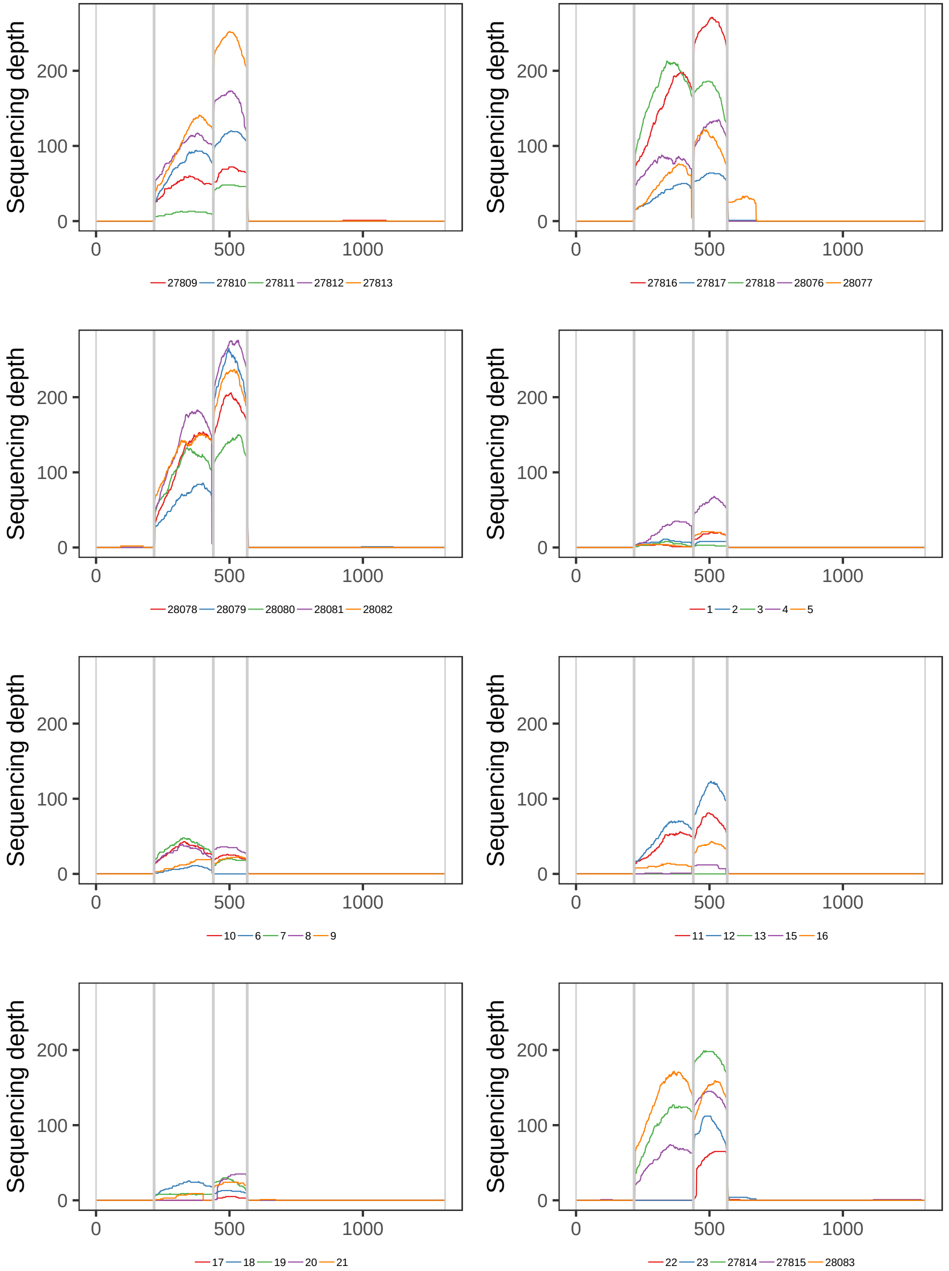
Sequencing depth
0
100
200
0
500
1000
27809 27810 27811 27812 27813
27816 27817 27818 28076 28077
28078 28079 28080 28081 28082
1 2 3 4 5
10 6 7 8 9
11 12 13 15 16
17 18 19 20 21
22 23 27814 27815 28083

EOG5VHHP6

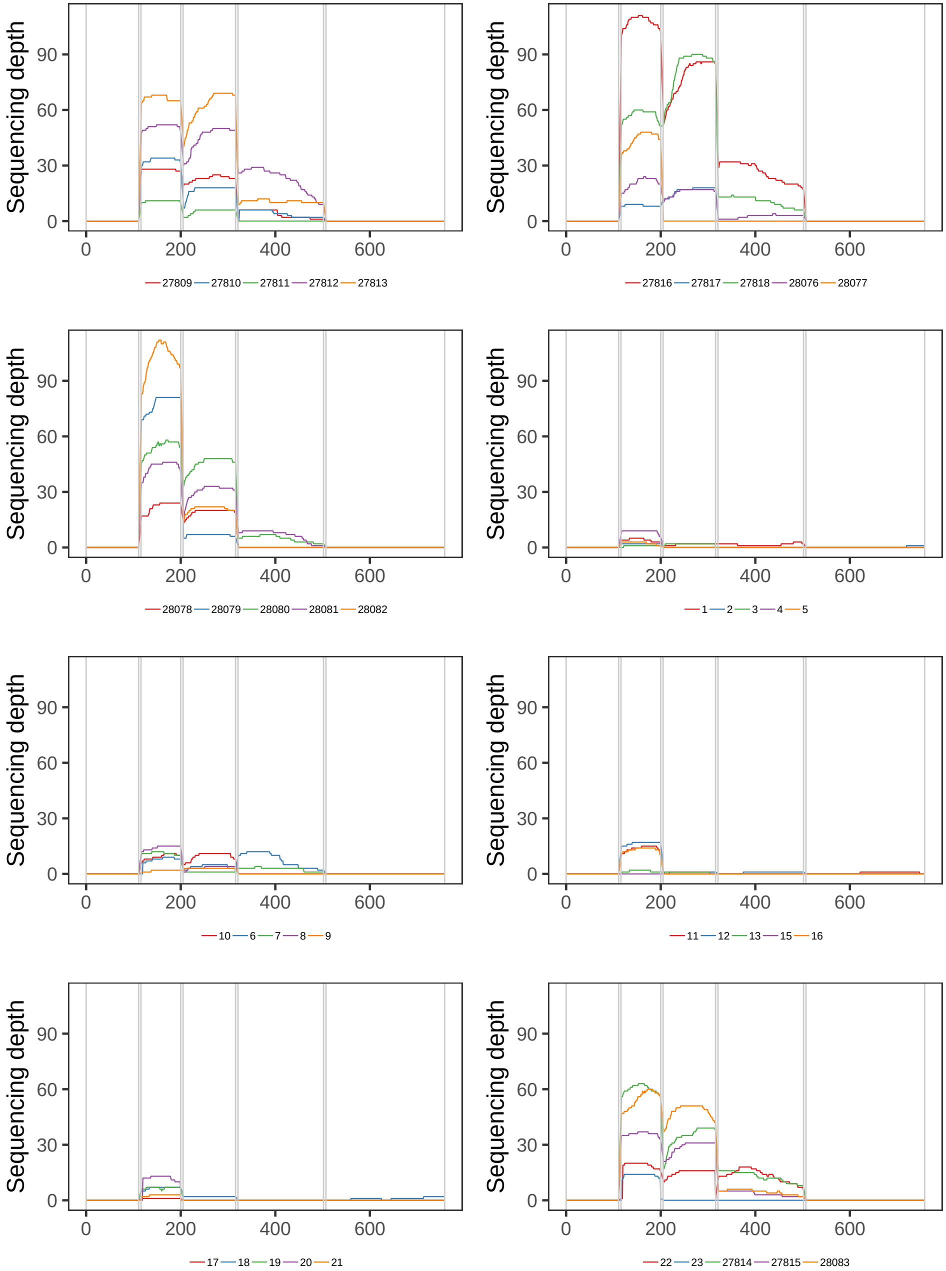

Sequencing depth
0
30
60
90
0
200
400
600
27809
27810
27811
27812
27813
27816
27817
27818
28076
28077
28078
28079
28080
28081
28082
1
2
3
4
5
10
6
7
8
9
11
12
13
15
16
17
18
19
20
21
22
23
27814
27815
28083

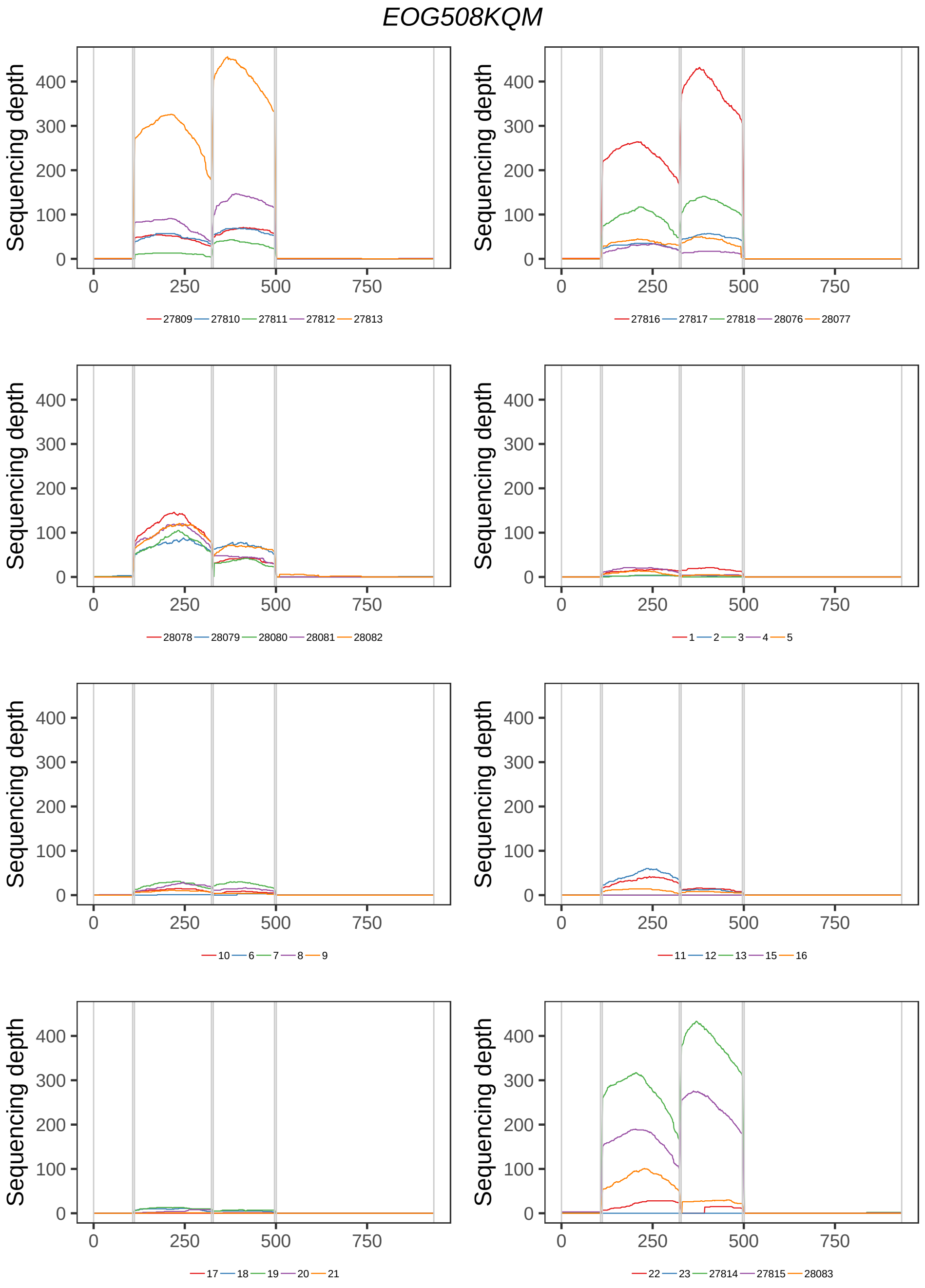

EOG508KQM
Sequencing depth
0
100
200
300
400
0
250
500
750
27809 27810 27811 27812 27813
27816 27817 27818 28076 28077
28078 28079 28080 28081 28082
1 2 3 4 5
10 6 7 8 9
11 12 13 15 16
17 18 19 20 21
22 23 27814 27815 28083

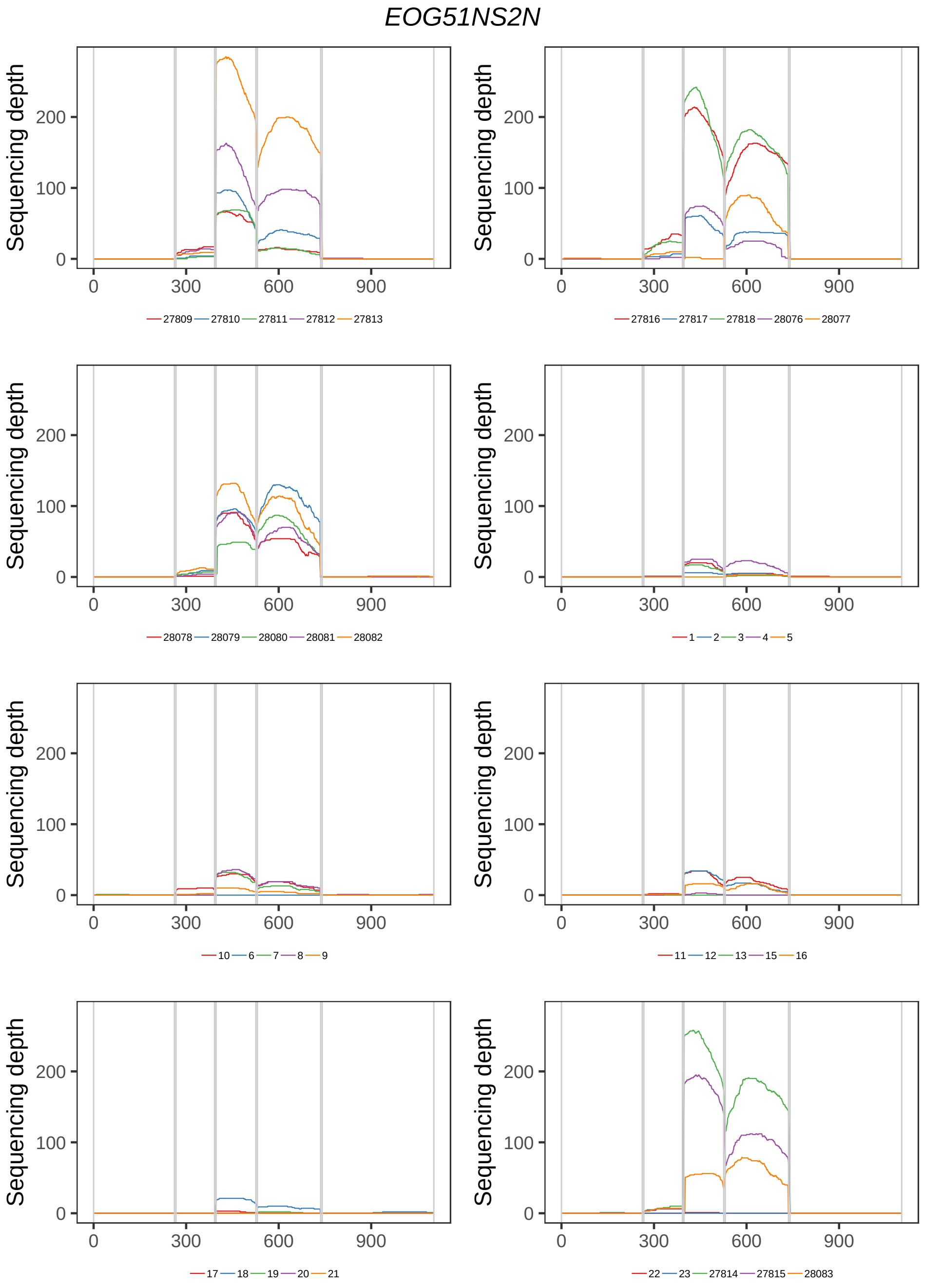

EOG51NS2N
Sequencing depth
0
100
200
300
600
900
27809
27810
27811
27812
27813
27816
27817
27818
28076
28077
28078
28079
28080
28081
28082
1
2
3
4
5
10
6
7
8
9
11
12
13
15
16
17
18
19
20
21
22
23
27814
27815
28083

EOG5D51CX

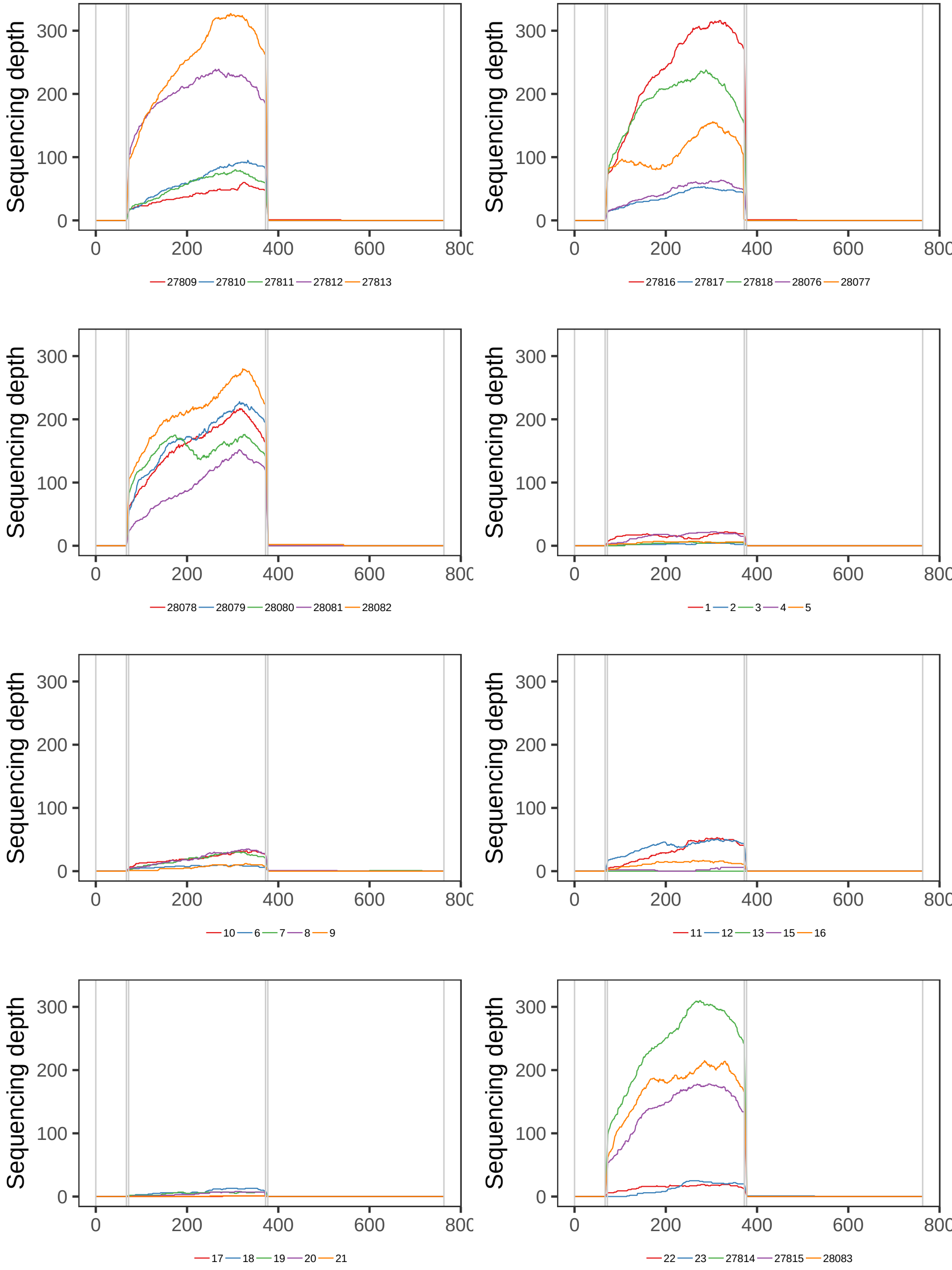

Sequencing depth
300
200
100
0
0
200
400
600
800
27809
27810
27811
27812
27813
27816
27817
27818
28076
28077
28078
28079
28080
28081
28082
1
2
3
4
5
10
6
7
8
9
11
12
13
15
16
17
18
19
20
21
22
23
27814
27815
28083

EOG5F4QSX

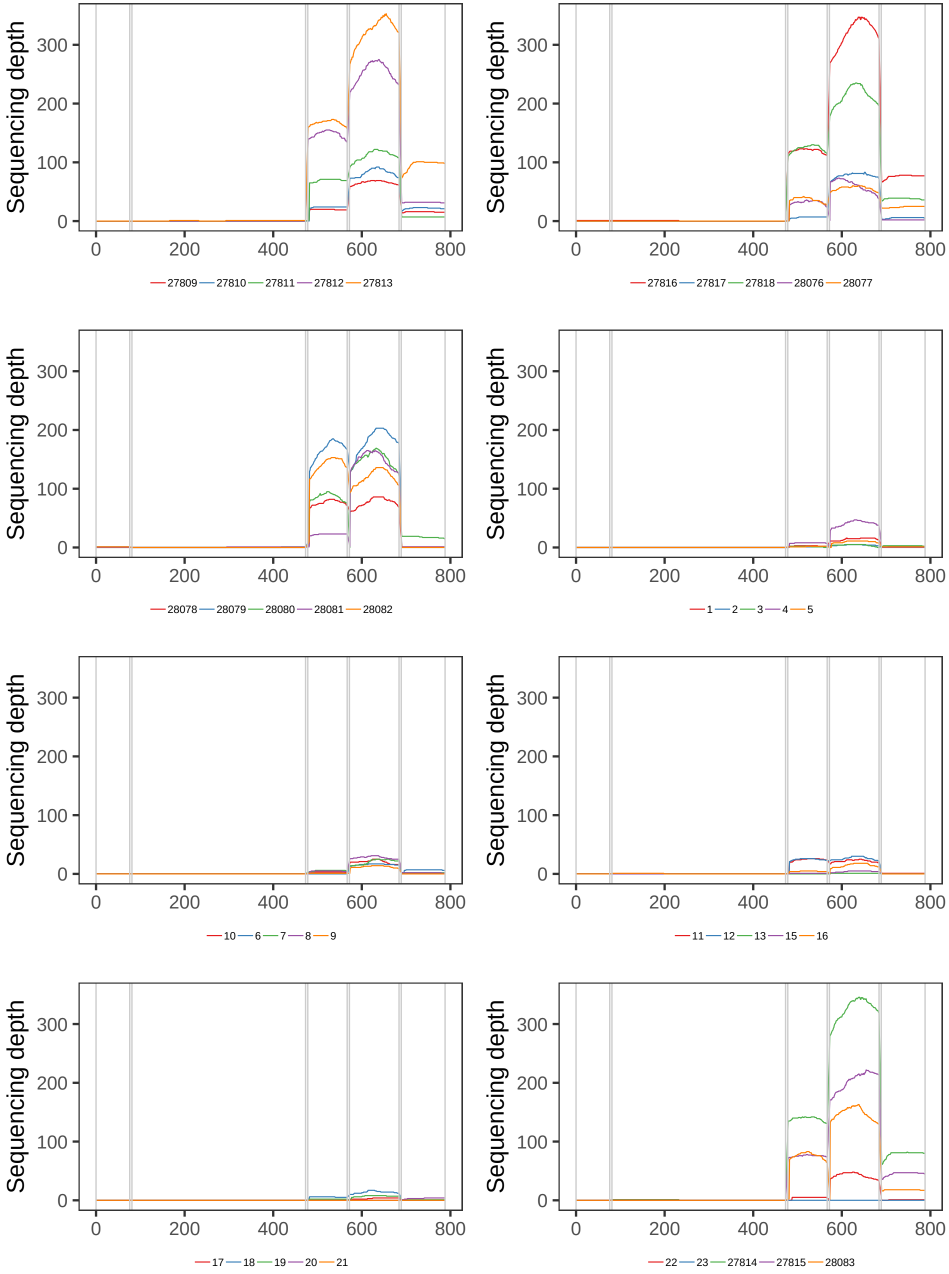
Sequencing depth
0
100
200
300
0
200
400
600
800
27809 27810 27811 27812 27813
27816 27817 27818 28076 28077
28078 28079 28080 28081 28082
1 2 3 4 5
10 6 7 8 9
11 12 13 15 16
17 18 19 20 21
22 23 27814 27815 28083

EOG5FXPPT

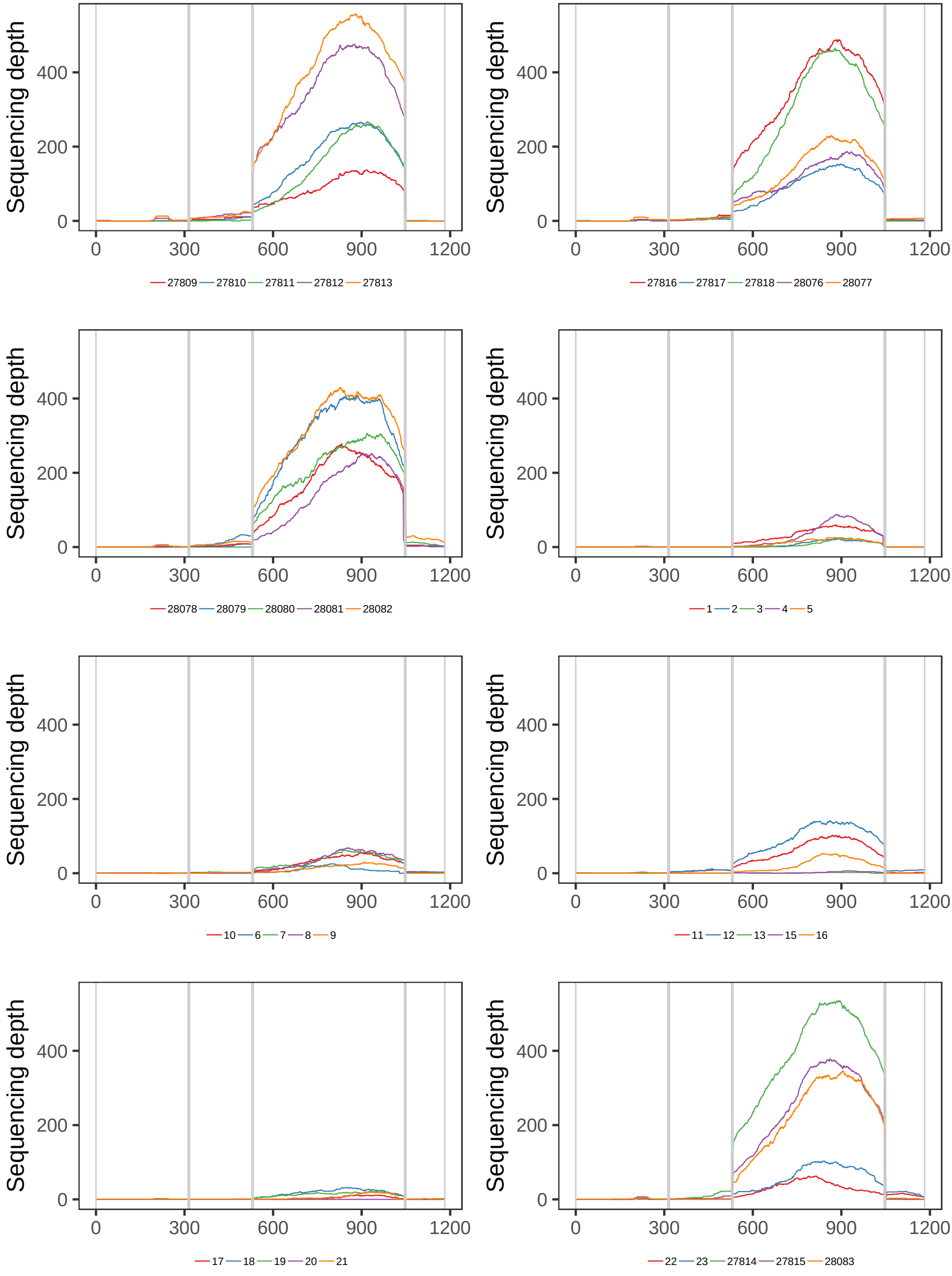
Sequencing depth
0
200
400
0
300
600
900
1200
27809 27810 27811 27812 27813
27816 27817 27818 28076 28077
28078 28079 28080 28081 28082
1 2 3 4 5
10 6 7 8 9
11 12 13 15 16
17 18 19 20 21
22 23 27814 27815 28083

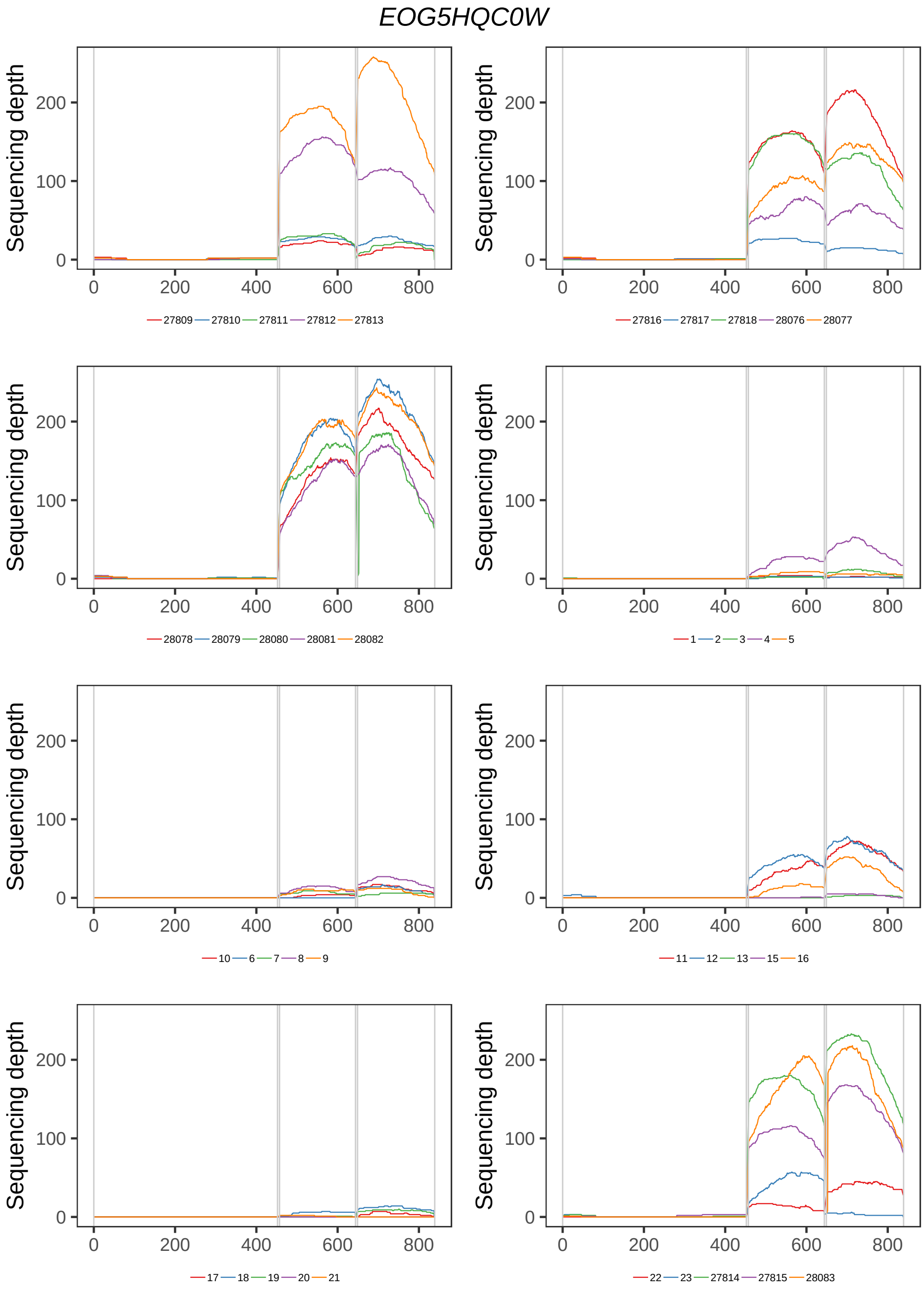

EOG5HQC0W
Sequencing depth
0
100
200
0
200
400
600
800
27809 27810 27811 27812 27813
27816 27817 27818 28076 28077
28078 28079 28080 28081 28082
1 2 3 4 5
10 6 7 8 9
11 12 13 15 16
17 18 19 20 21
22 23 27814 27815 28083

EOG5S7H64

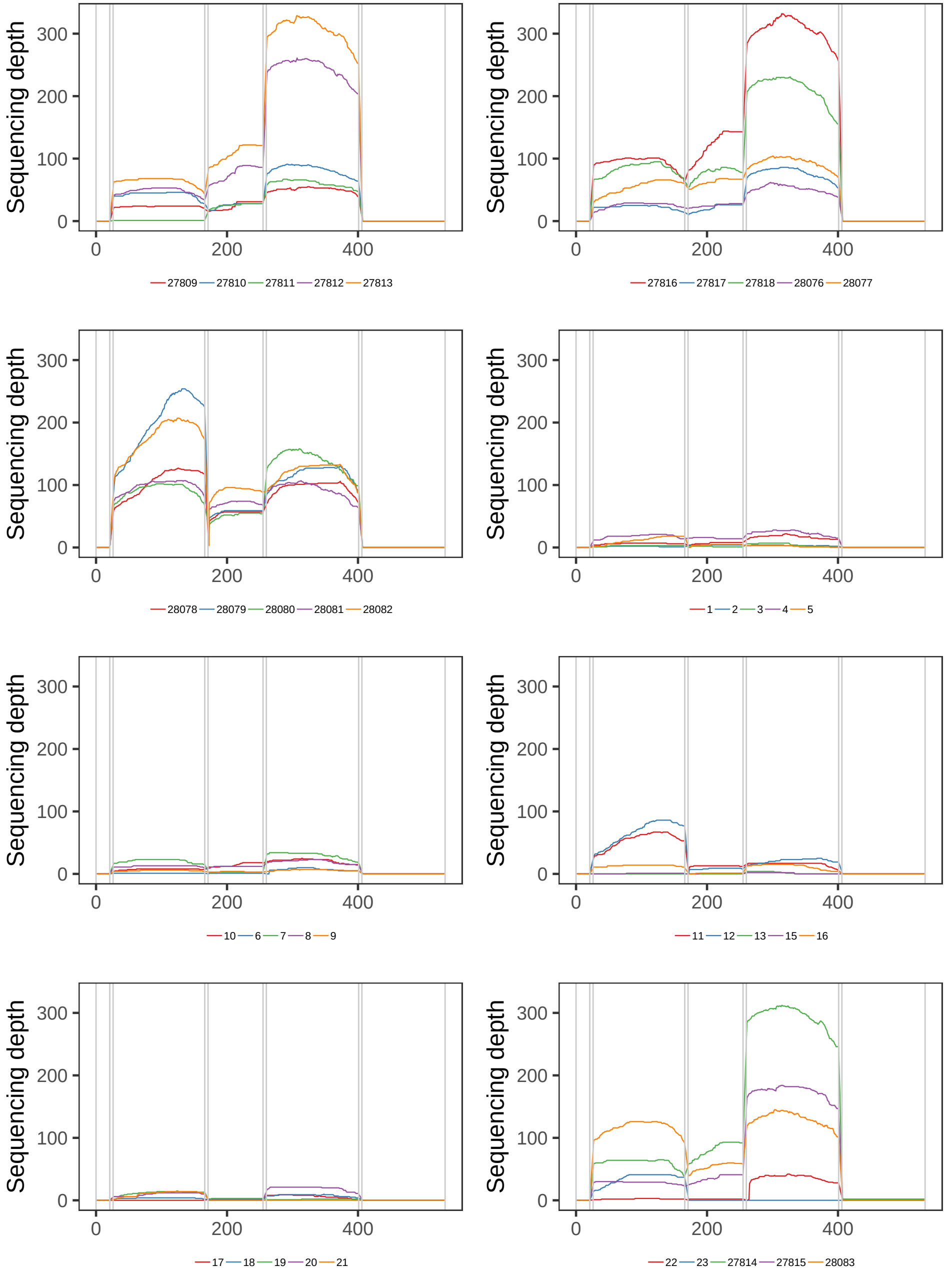
Sequencing depth
0
100
200
300
0
200
400
27809 27810 27811 27812 27813
27816 27817 27818 28076 28077
28078 28079 28080 28081 28082
1 2 3 4 5
10 6 7 8 9
11 12 13 15 16
17 18 19 20 21
22 23 27814 27815 28083

EOG5W9GK9

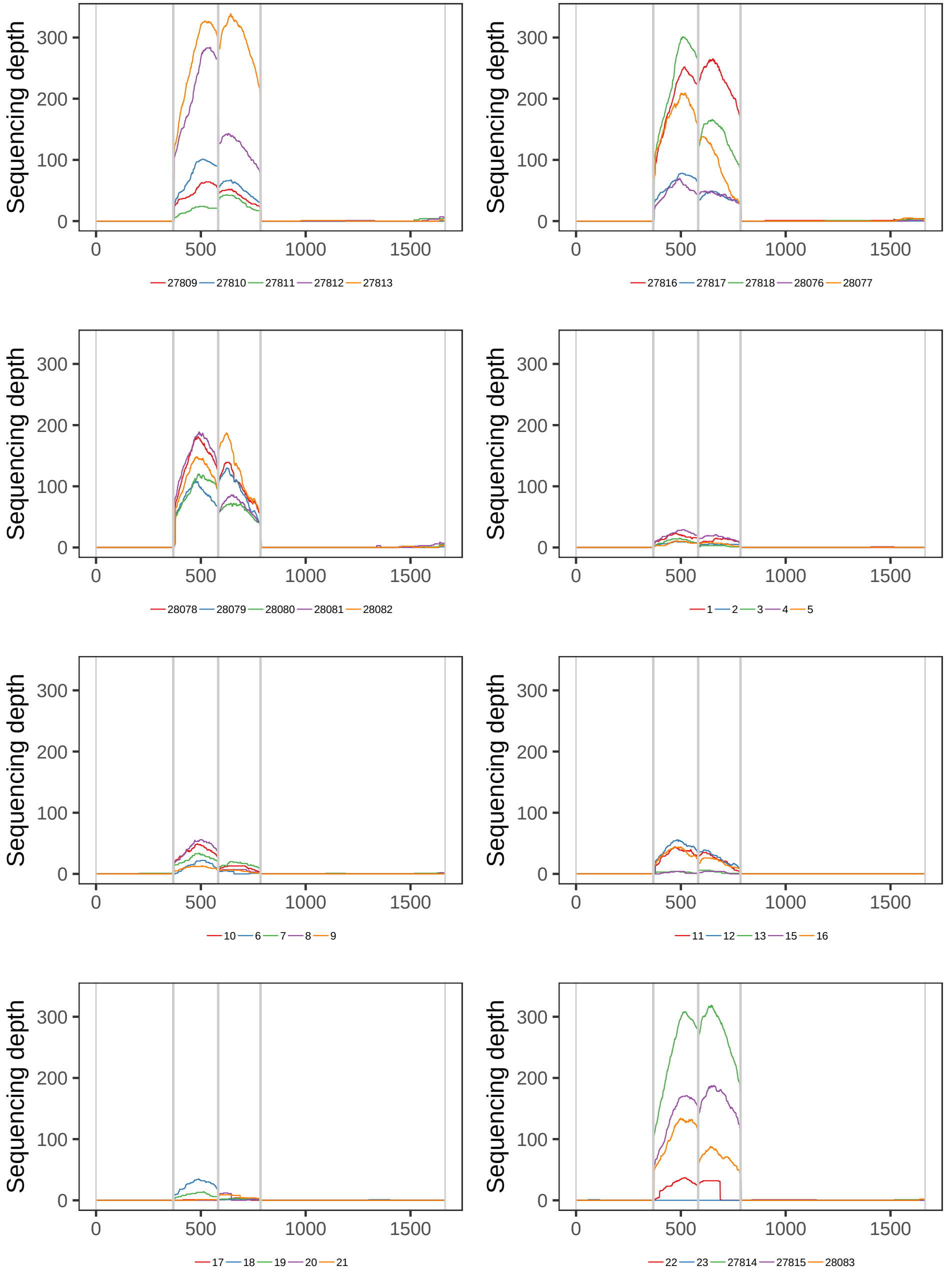
Sequencing depth
0
100
200
300
500
1000
1500
27809 27810 27811 27812 27813
27816 27817 27818 28076 28077
28078 28079 28080 28081 28082
1 2 3 4 5
10 6 7 8 9
11 12 13 15 16
17 18 19 20 21
22 23 27814 27815 28083

EOG5X95ZH

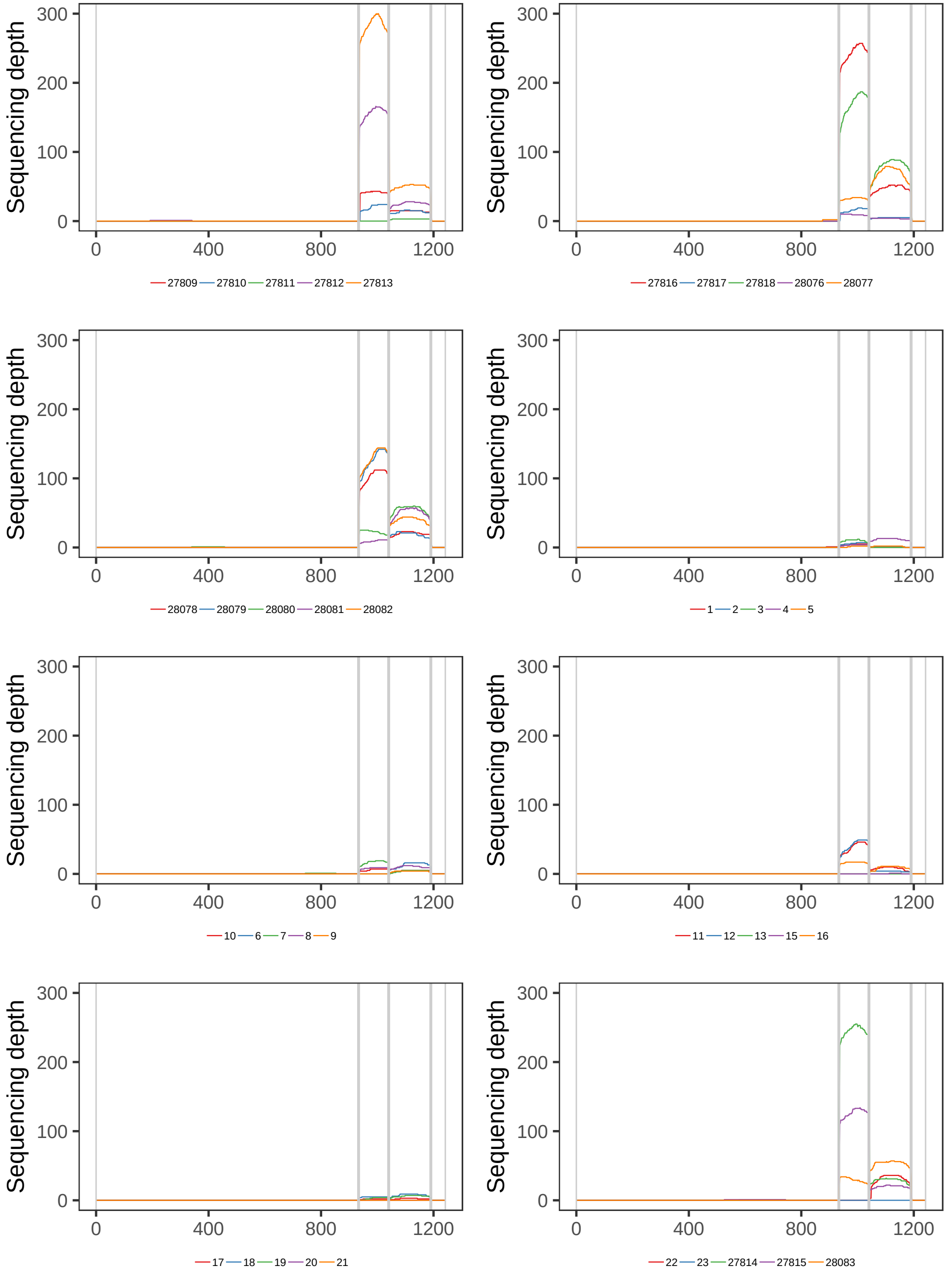
Sequencing depth
300
200
100
0
0
400
800
1200
27809 27810 27811 27812 27813
27816 27817 27818 28076 28077
28078 28079 28080 28081 28082
1 2 3 4 5
10 6 7 8 9
11 12 13 15 16
17 18 19 20 21
22 23 27814 27815 28083

EOG58GTKD

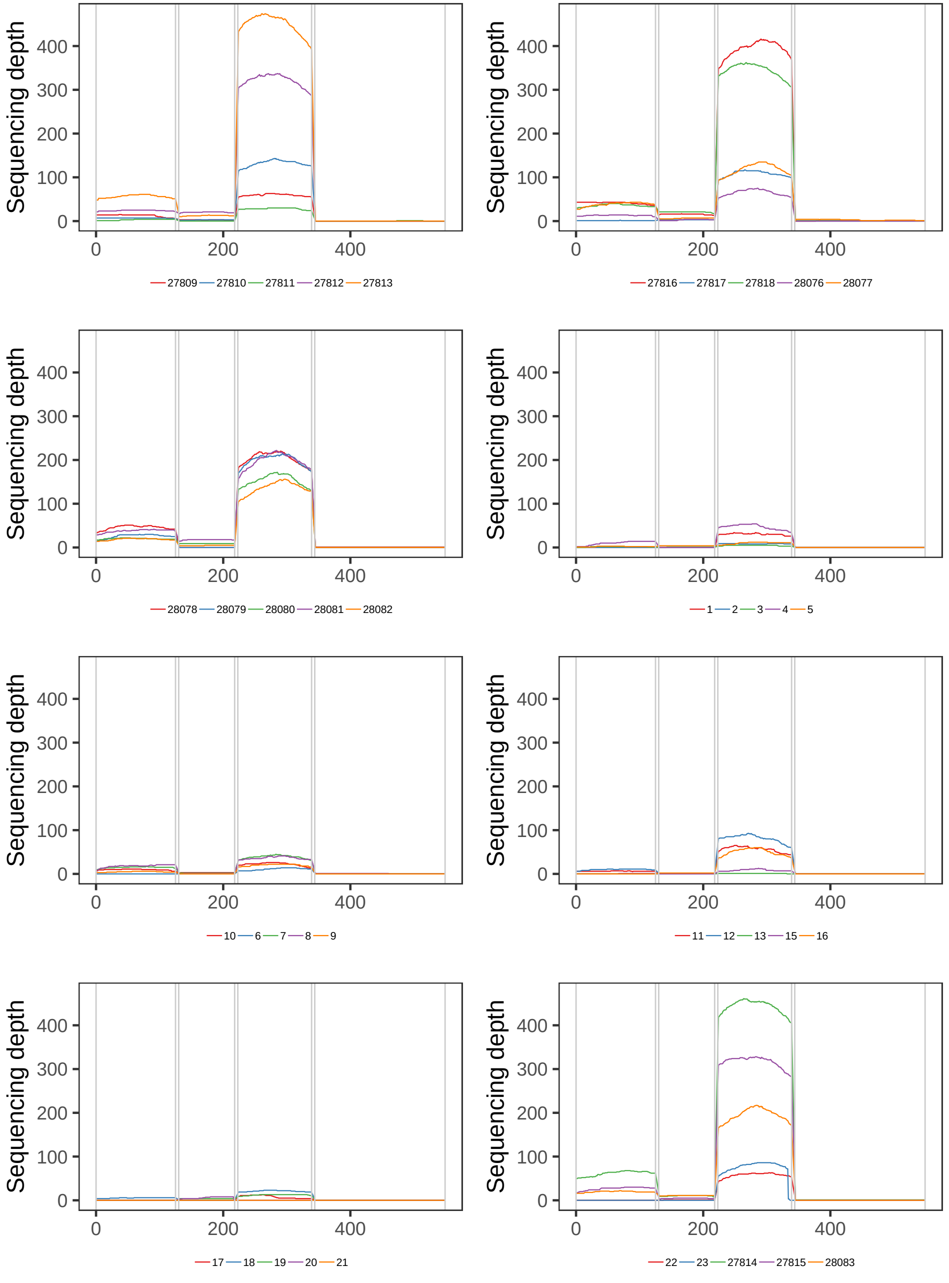

Sequencing depth
0
100
200
300
400
0
200
400
27809 27810 27811 27812 27813
27816 27817 27818 28076 28077
28078 28079 28080 28081 28082
1 2 3 4 5
10 6 7 8 9
11 12 13 15 16
17 18 19 20 21
22 23 27814 27815 28083

EOG5DNCM5

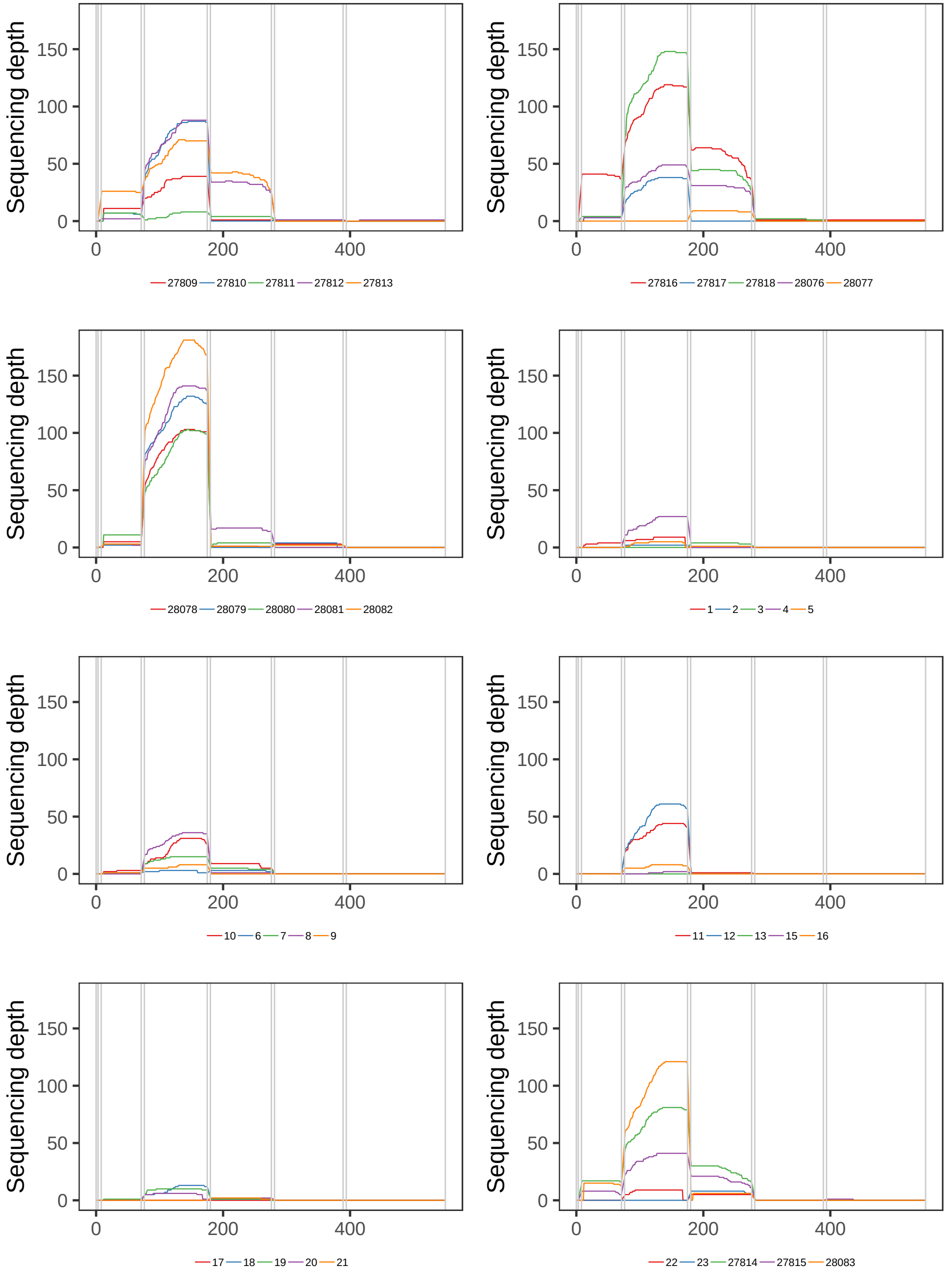
Sequencing depth
150
100
50
0
0
200
400
27809
27810
27811
27812
27813
27816
27817
27818
28076
28077
28078
28079
28080
28081
28082
1
2
3
4
5
10
6
7
8
9
11
12
13
15
16
17
18
19
20
21
22
23
27814
27815
28083

EOG5DV42P

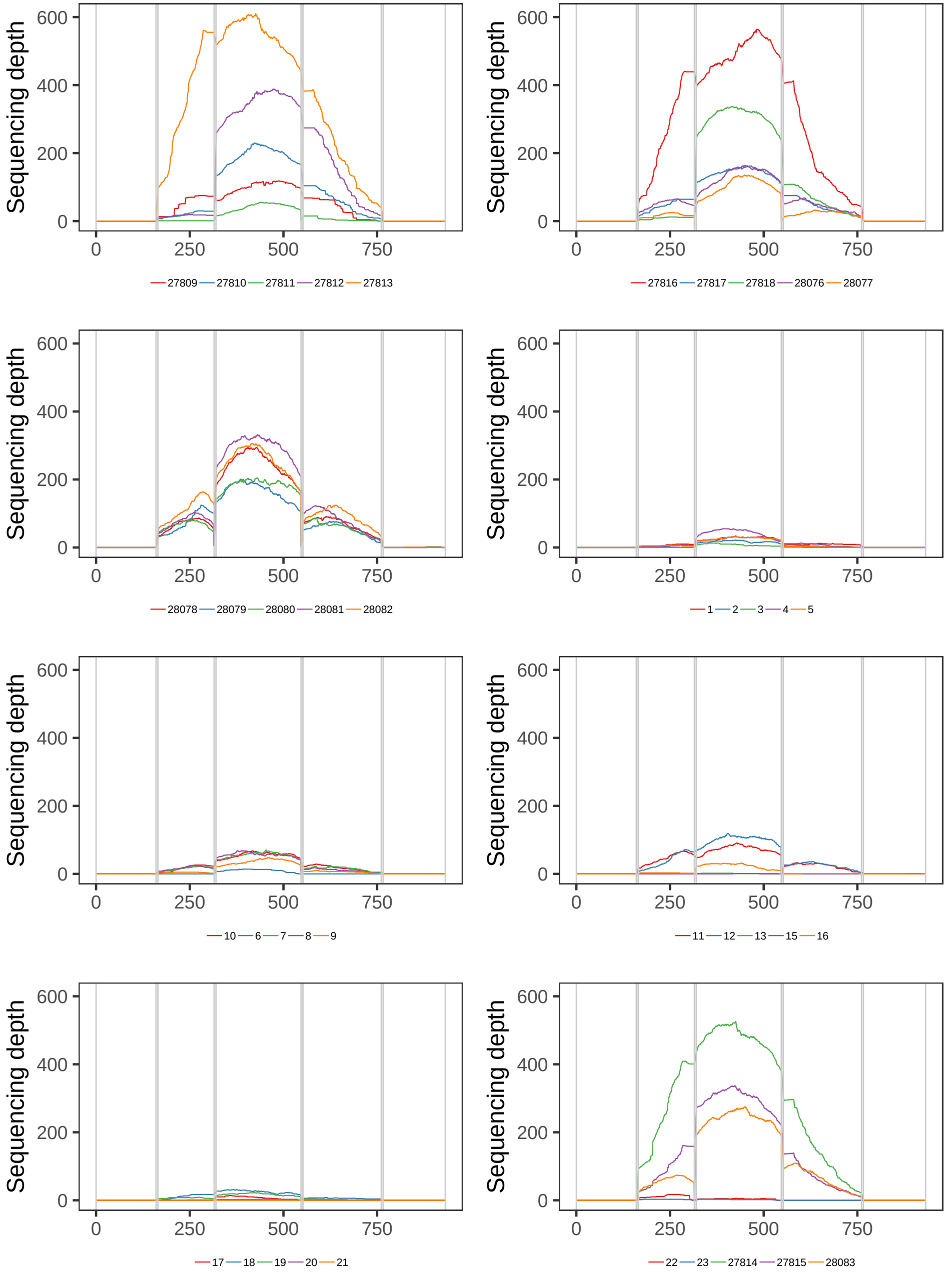
Sequencing depth
0
200
400
600
0
250
500
750
27809 27810 27811 27812 27813
27816 27817 27818 28076 28077
28078 28079 28080 28081 28082
1 2 3 4 5
10 6 7 8 9
11 12 13 15 16
17 18 19 20 21
22 23 27814 27815 28083

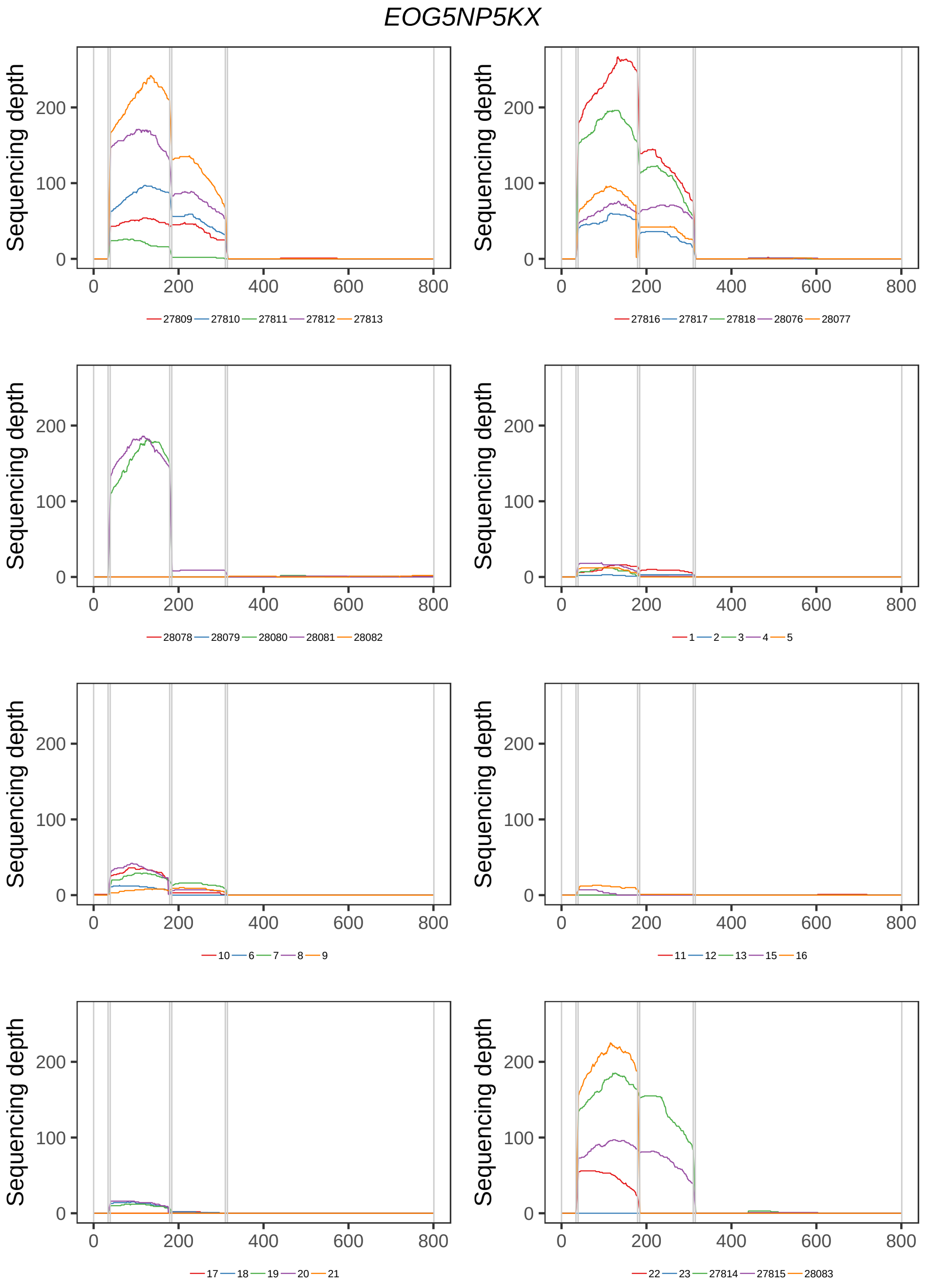
EOG5NP5KX
Sequencing depth
0
100
200
0
200
400
600
800
27809
27810
27811
27812
27813
27816
27817
27818
28076
28077
28078
28079
28080
28081
28082
1
2
3
4
5
10
6
7
8
9
11
12
13
15
16
17
18
19
20
21
22
23
27814
27815
28083

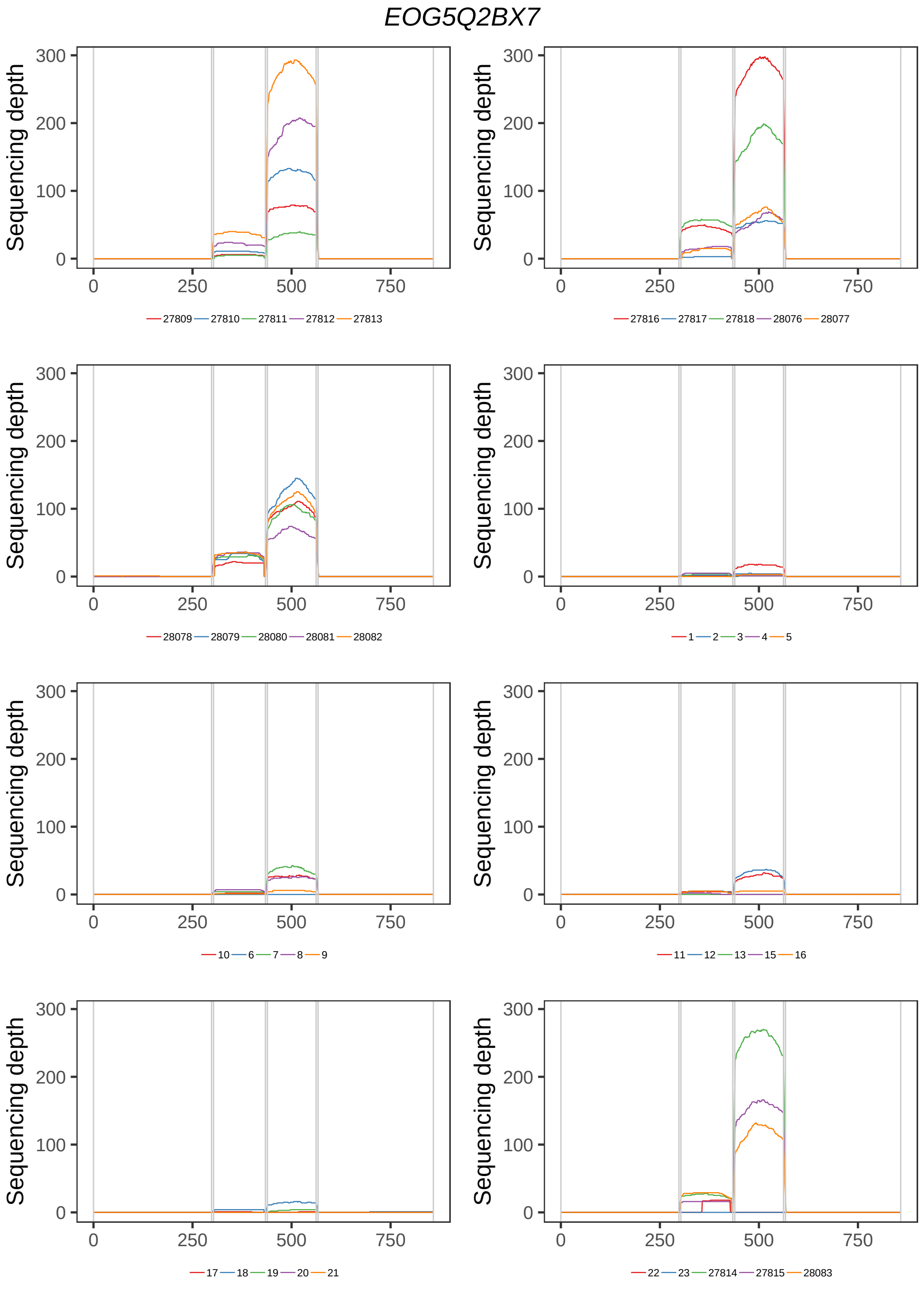
EOG5Q2BX7
Sequencing depth
27809 27810 27811 27812 27813
27816 27817 27818 28076 28077
28078 28079 28080 28081 28082
1 2 3 4 5
10 6 7 8 9
11 12 13 15 16
17 18 19 20 21
22 23 27814 27815 28083

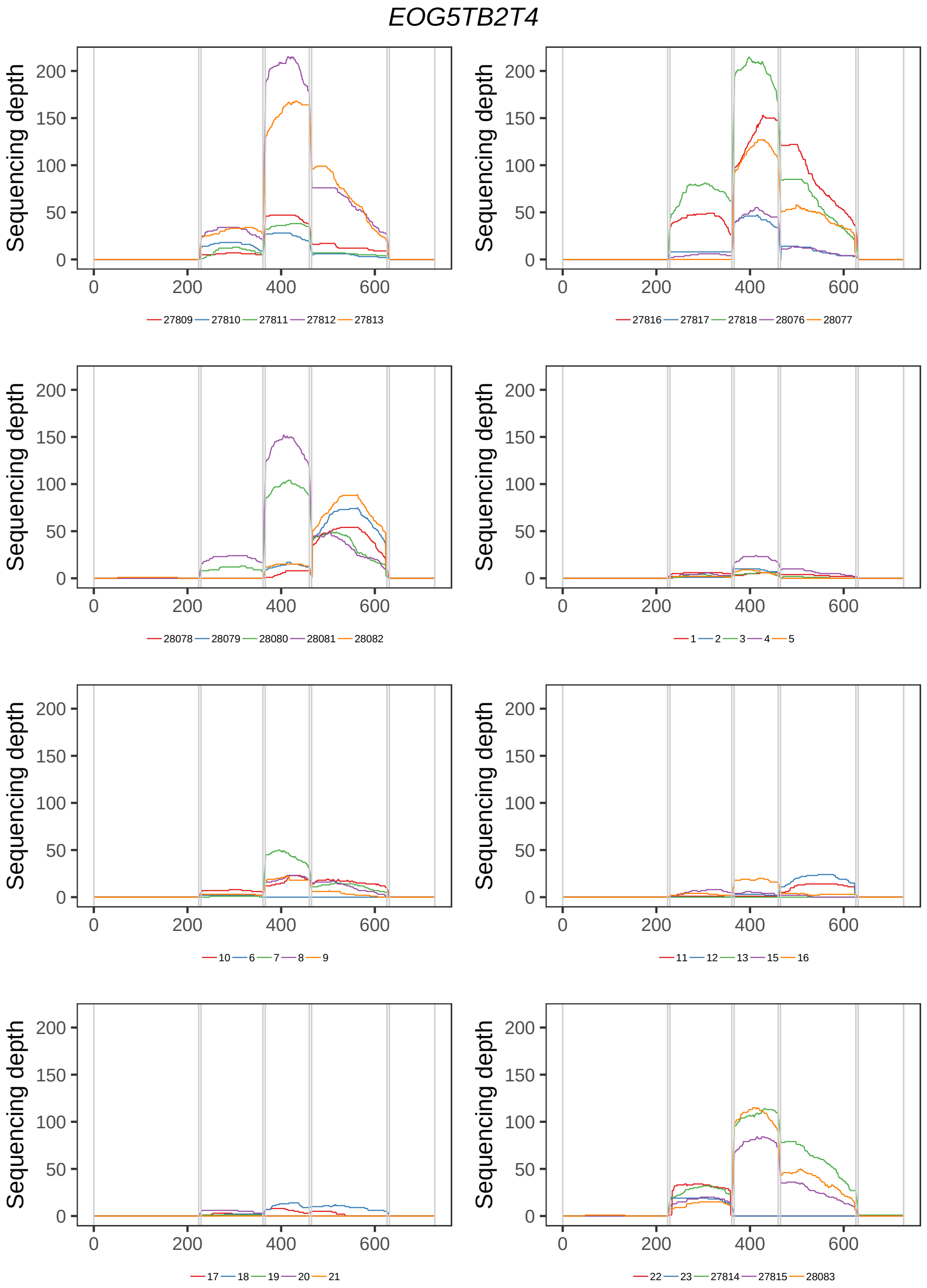
EOG5TB2T4
Sequencing depth
27809 27810 27811 27812 27813
27816 27817 27818 28076 28077
28078 28079 28080 28081 28082
1 2 3 4 5
10 6 7 8 9
11 12 13 15 16
17 18 19 20 21
22 23 27814 27815 28083

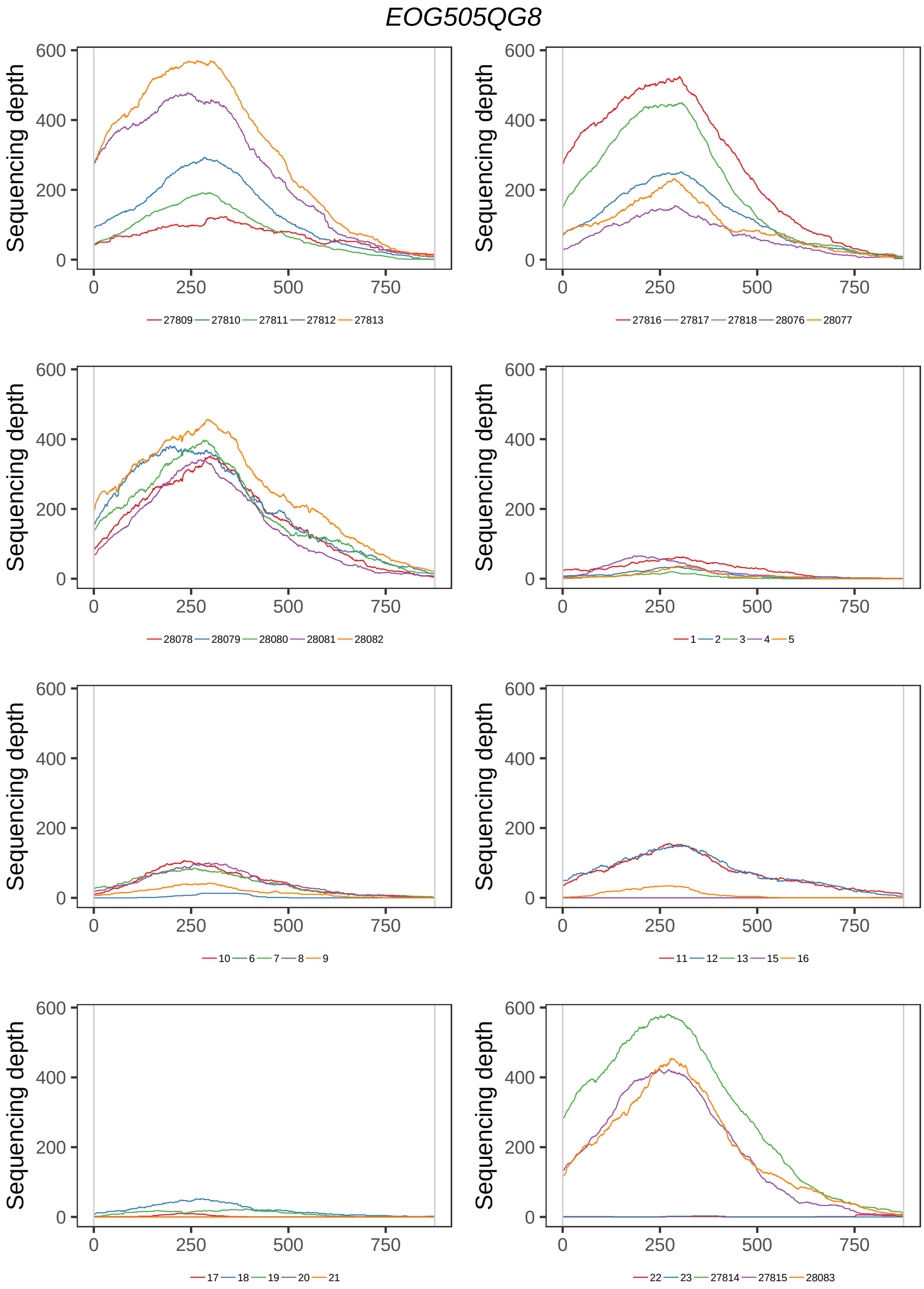
EOG505QG8
Sequencing depth
600
400
200
0
250
500
750
27809
27810
27811
27812
27813
27816
27817
27818
28076
28077
28078
28079
28080
28081
28082
1
2
3
4
5
10
6
7
8
9
11
12
13
15
16
17
18
19
20
21
22
23
27814
27815
28083

EOG515DWK

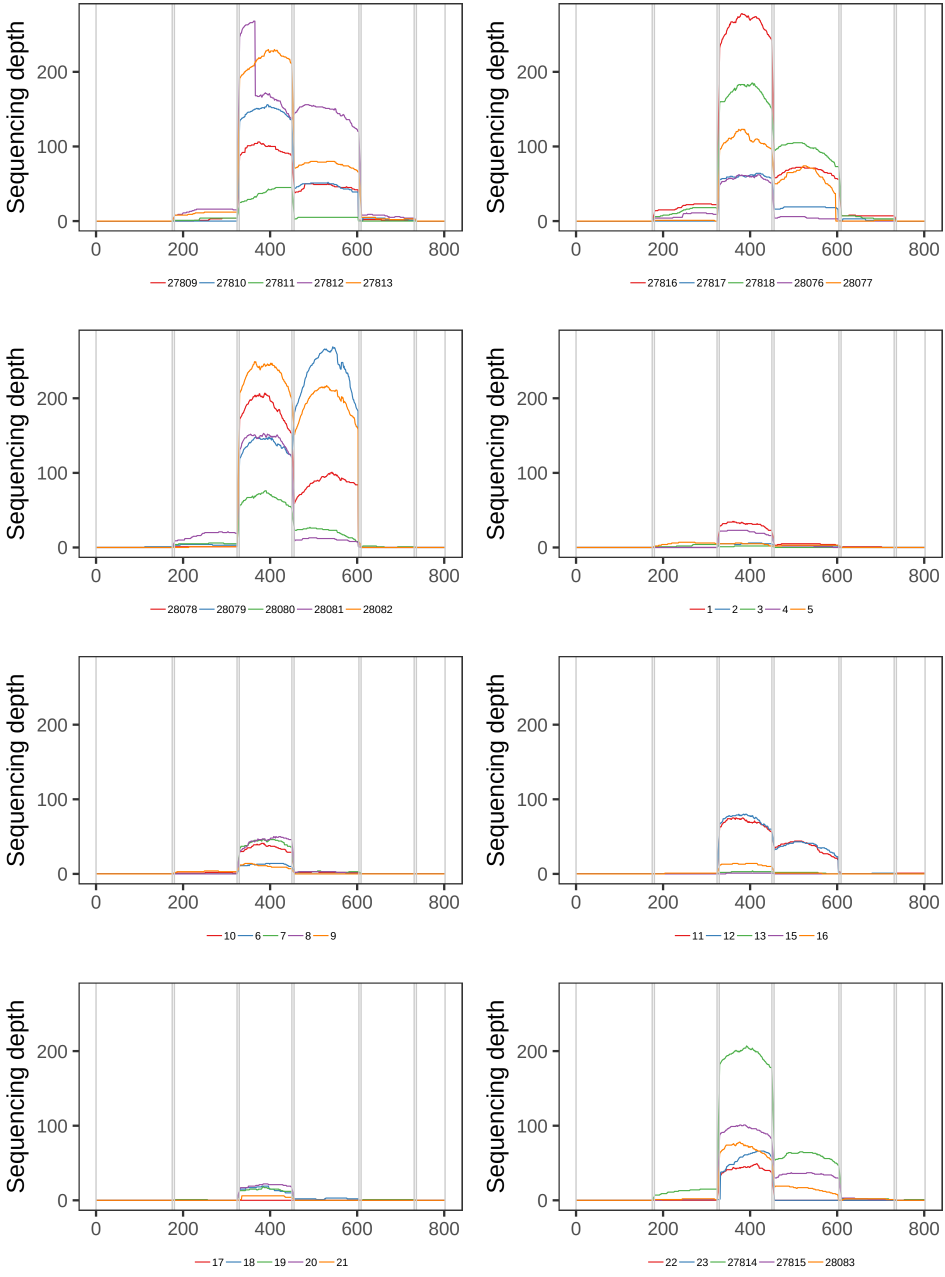
Sequencing depth
0
100
200
0
200
400
600
800
27809 27810 27811 27812 27813
27816 27817 27818 28076 28077
28078 28079 28080 28081 28082
1 2 3 4 5
10 6 7 8 9
11 12 13 15 16
17 18 19 20 21
22 23 27814 27815 28083

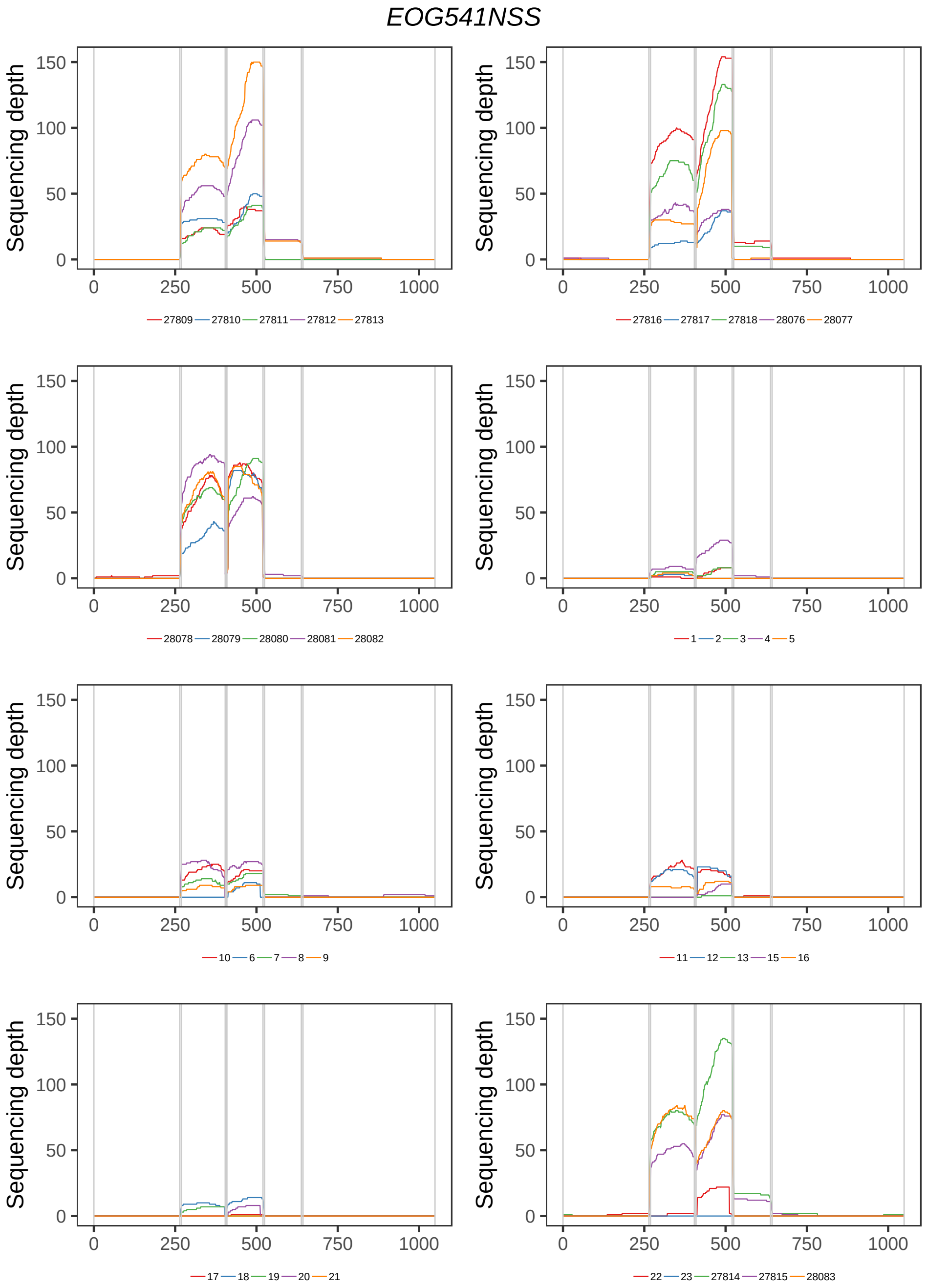
EOG541NSS
Sequencing depth
0
50
100
150
0
250
500
750
1000
27809
27810
27811
27812
27813
27816
27817
27818
28076
28077
28078
28079
28080
28081
28082
1
2
3
4
5
10
6
7
8
9
11
12
13
15
16
17
18
19
20
21
22
23
27814
27815
28083

EOG58PK1S

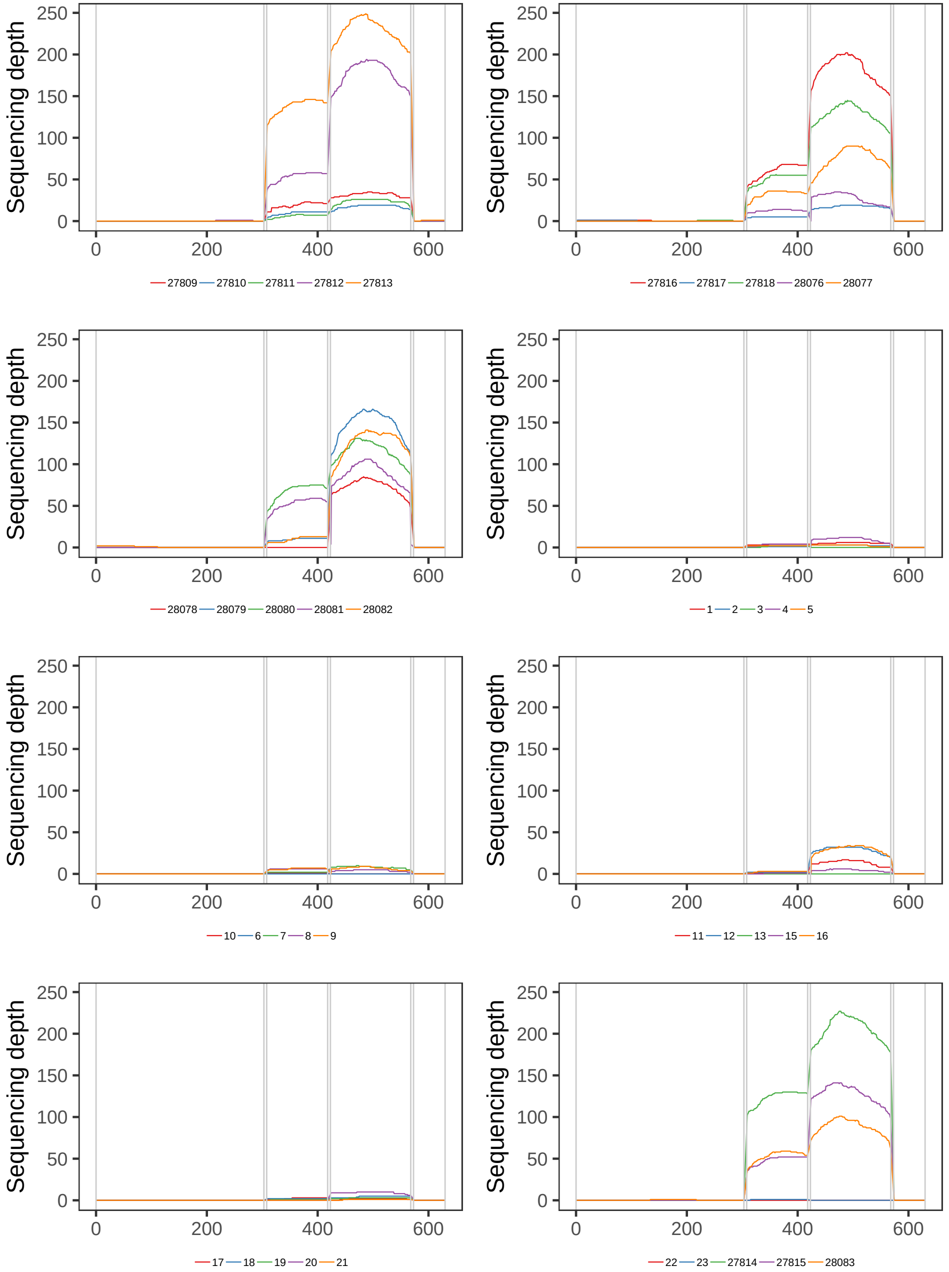
Sequencing depth
250
200
150
100
50
0
0
200
400
600
27809
27810
27811
27812
27813
27816
27817
27818
28076
28077
28078
28079
28080
28081
28082
1
2
3
4
5
10
6
7
8
9
11
12
13
15
16
17
18
19
20
21
22
23
27814
27815
28083

EOG59KD62

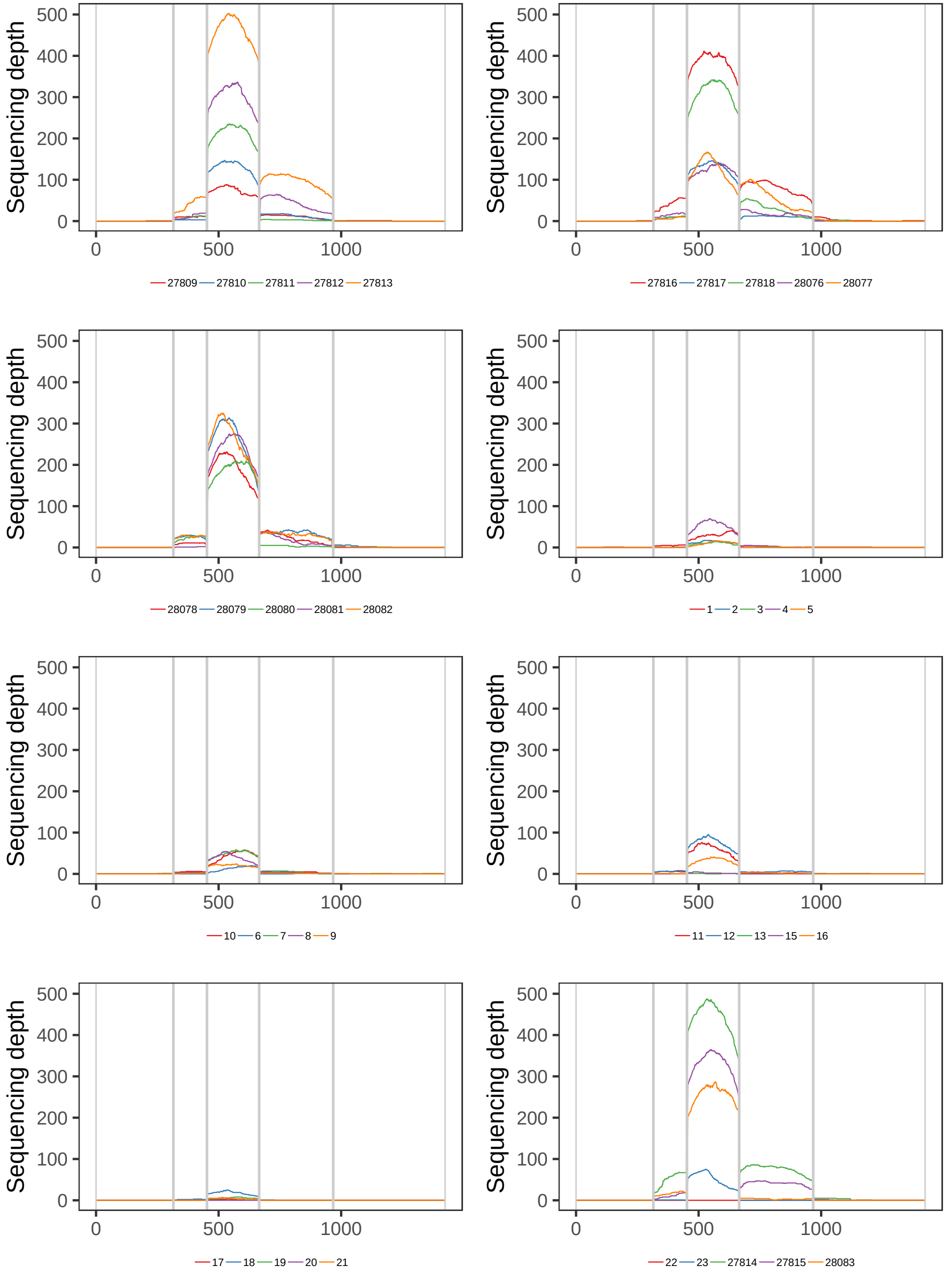
Sequencing depth
500
400
300
200
100
0
0
500
1000
27809 27810 27811 27812 27813
27816 27817 27818 28076 28077
28078 28079 28080 28081 28082
1 2 3 4 5
10 6 7 8 9
11 12 13 15 16
17 18 19 20 21
22 23 27814 27815 28083

EOG59W0WR

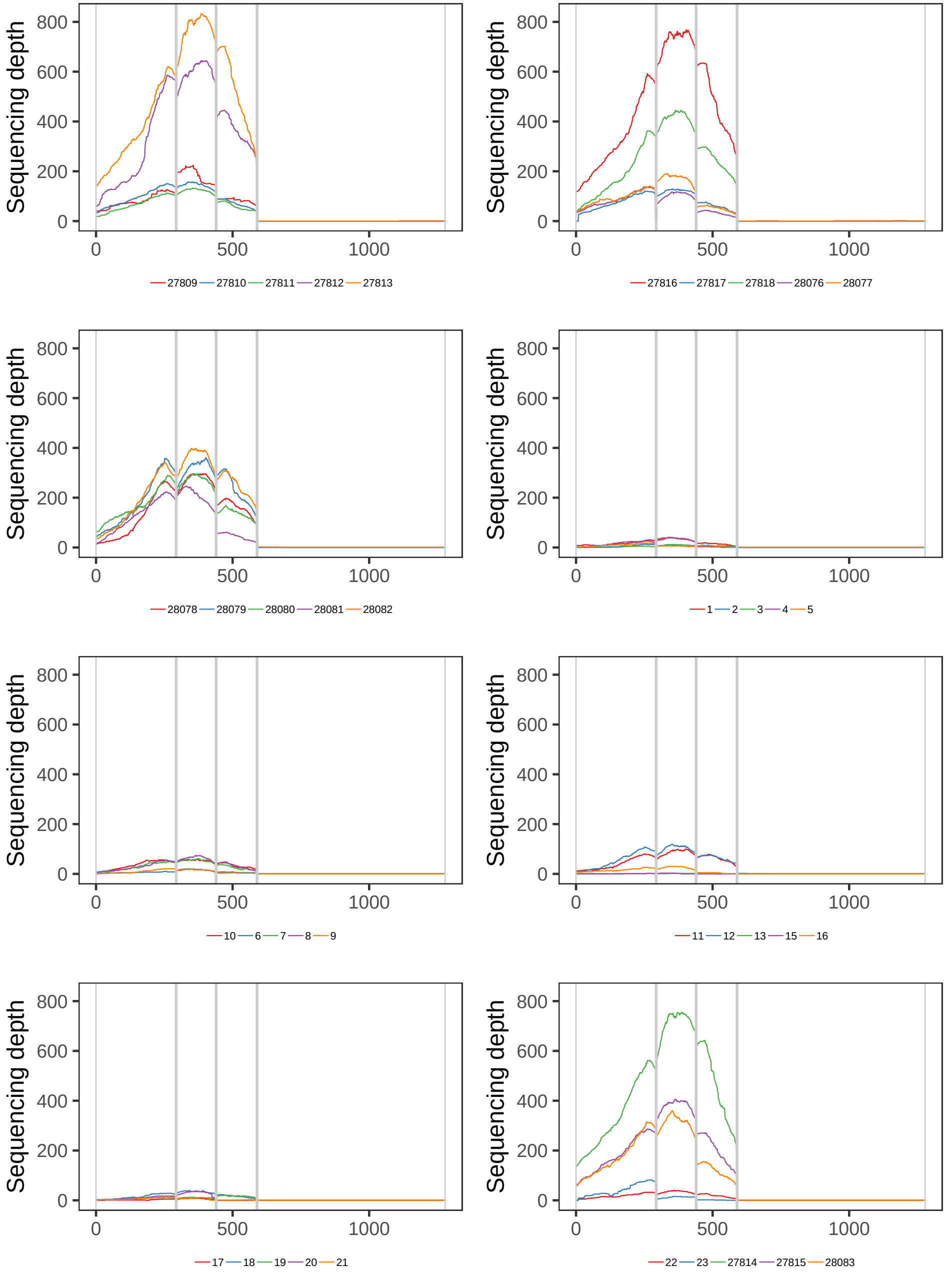
Sequencing depth
0
200
400
600
800
0
500
1000
27809 27810 27811 27812 27813
27816 27817 27818 28076 28077
28078 28079 28080 28081 28082
1 2 3 4 5
10 6 7 8 9
11 12 13 15 16
17 18 19 20 21
22 23 27814 27815 28083

EOG5G4F5W

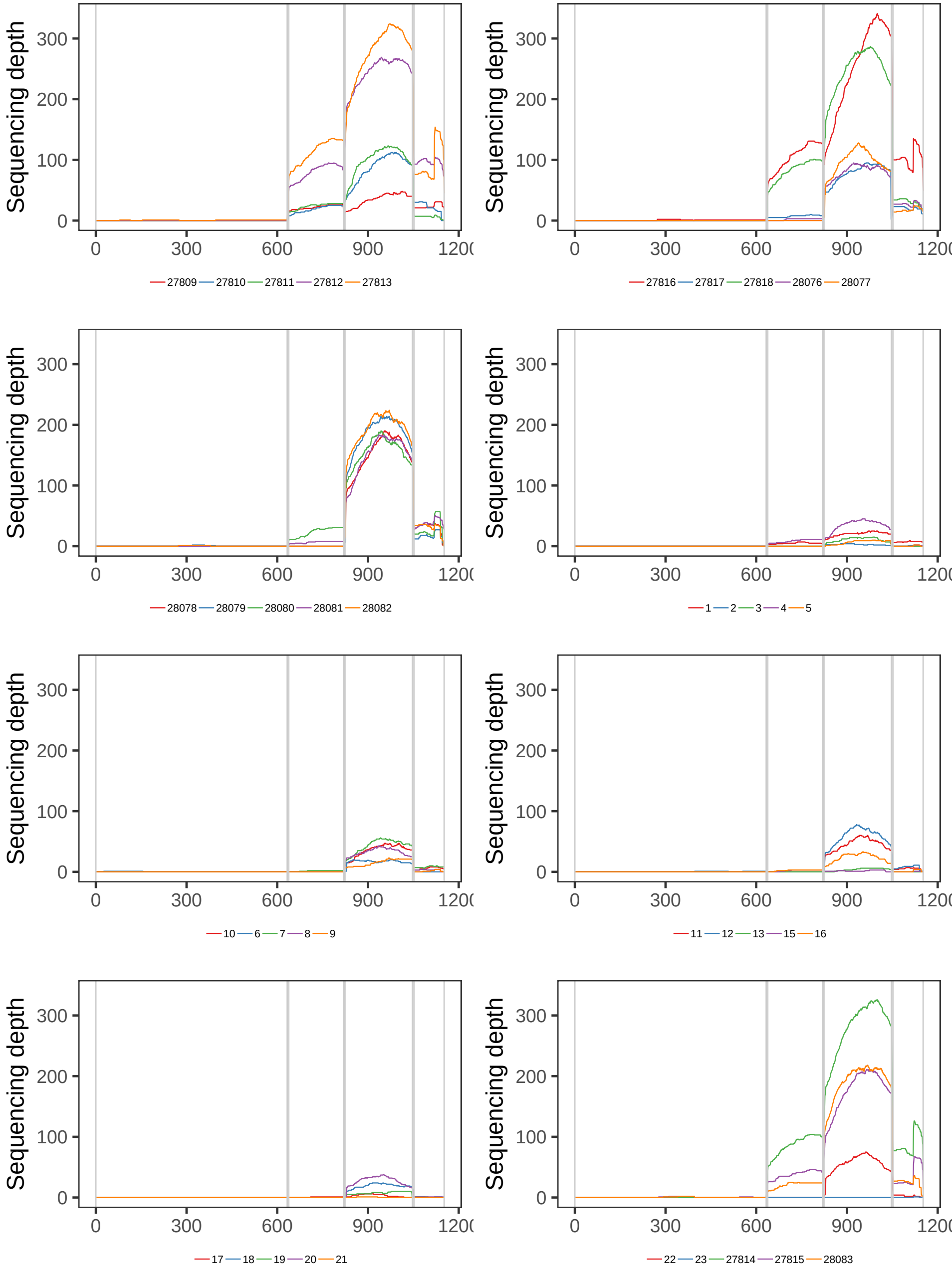

Sequencing depth
300
200
100
0
0
300
600
900
1200
27809
27810
27811
27812
27813
27816
27817
27818
28076
28077
28078
28079
28080
28081
28082
1
2
3
4
5
10
6
7
8
9
11
12
13
15
16
17
18
19
20
21
22
23
27814
27815
28083

EOG5Q83D3

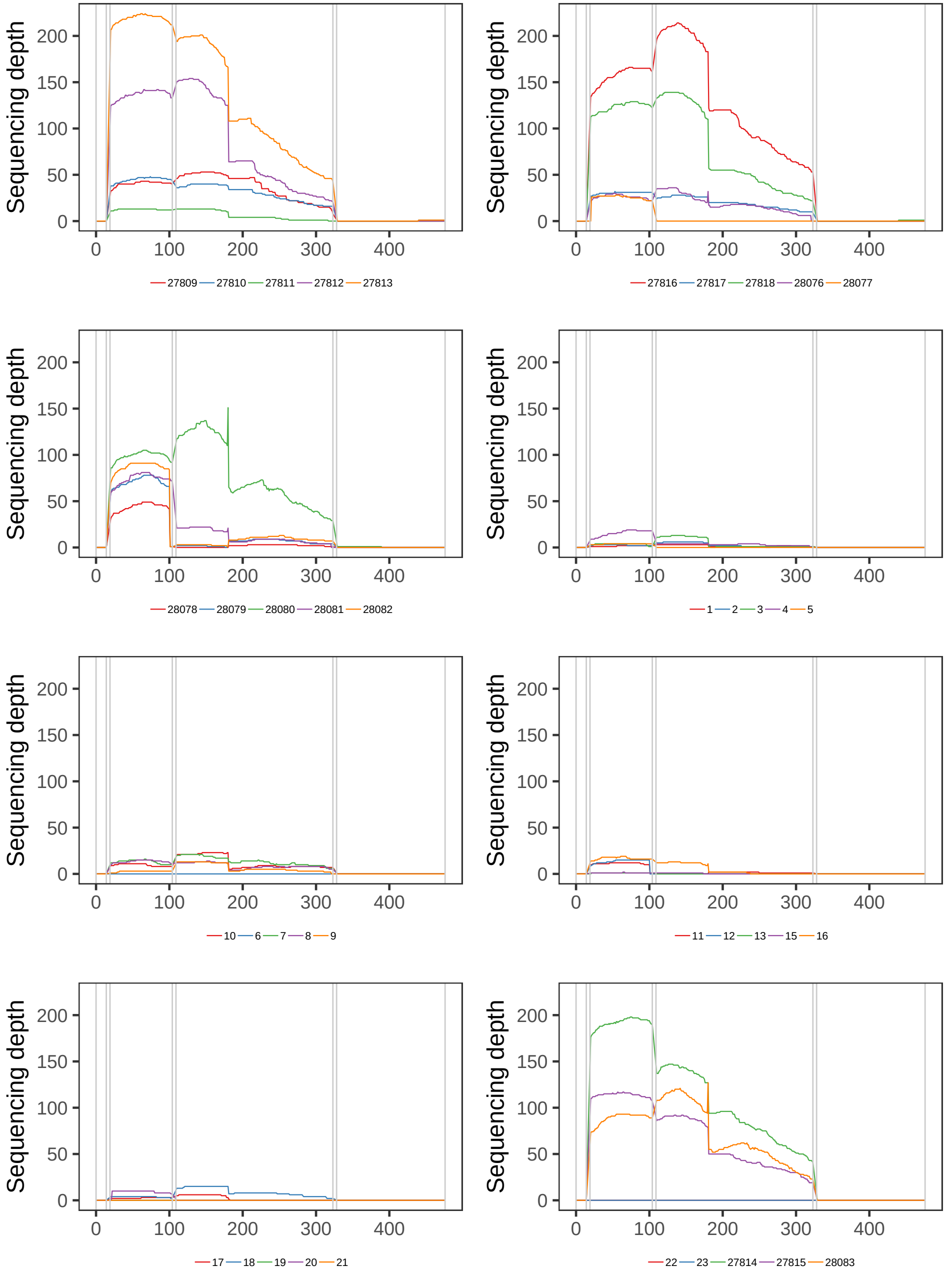
Sequencing depth
0
50
100
150
200
0
100
200
300
400
27809 27810 27811 27812 27813
27816 27817 27818 28076 28077
28078 28079 28080 28081 28082
1 2 3 4 5
10 6 7 8 9
11 12 13 15 16
17 18 19 20 21
22 23 27814 27815 28083

EOG5SXKTQ

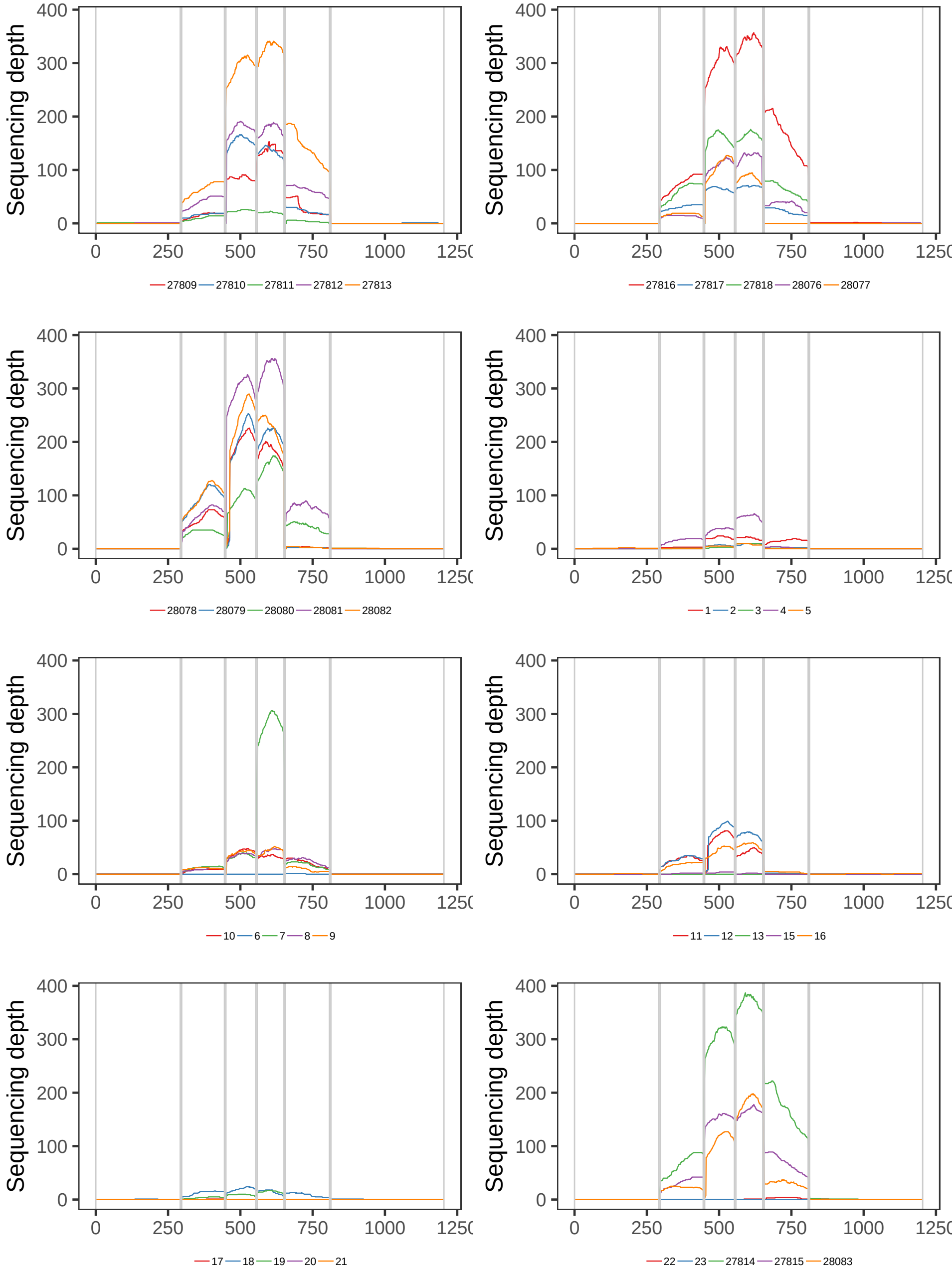
Sequencing depth
0
100
200
300
400
0
250
500
750
1000
1250
27809 27810 27811 27812 27813
27816 27817 27818 28076 28077
28078 28079 28080 28081 28082
1 2 3 4 5
10 6 7 8 9
11 12 13 15 16
17 18 19 20 21
22 23 27814 27815 28083

EOG5V9S6Q

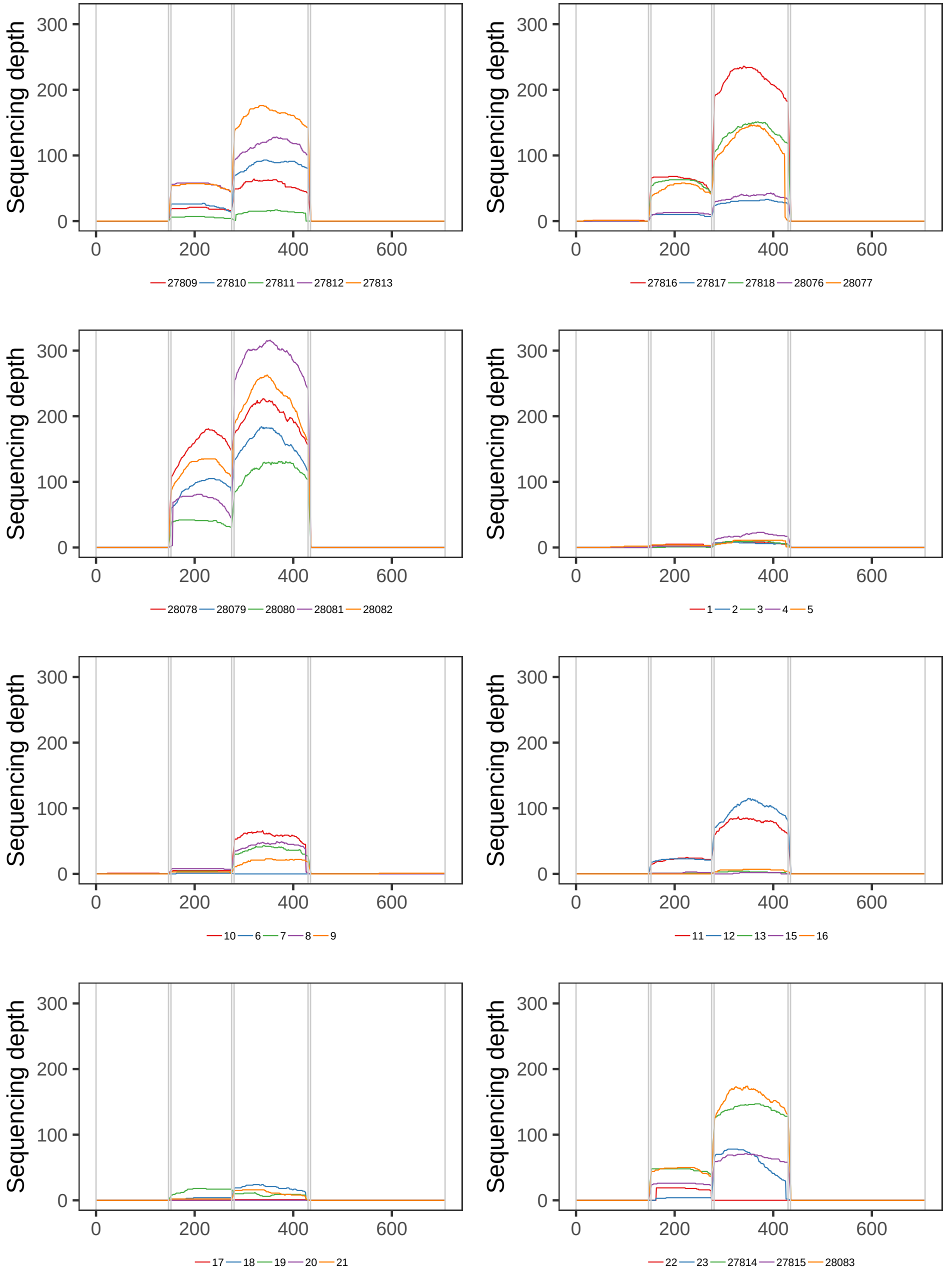

Sequencing depth
0
100
200
300
0
200
400
600
27809
27810
27811
27812
27813
27816
27817
27818
28076
28077
28078
28079
28080
28081
28082
1
2
3
4
5
10
6
7
8
9
11
12
13
15
16
17
18
19
20
21
22
23
27814
27815
28083

EOG52V6XB

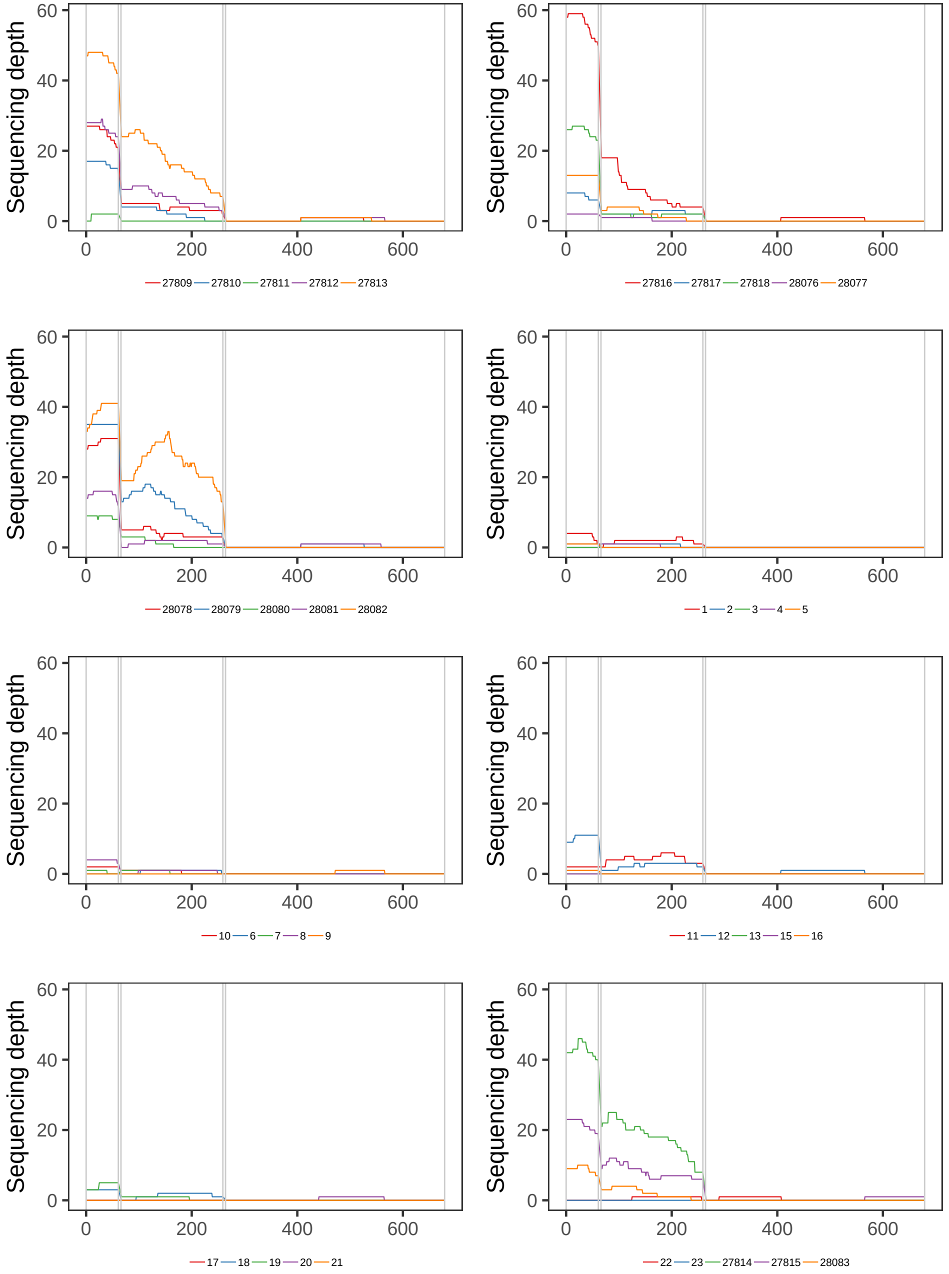
Sequencing depth
0
20
40
60
0
200
400
600
27809
27810
27811
27812
27813
27816
27817
27818
28076
28077
28078
28079
28080
28081
28082
1
2
3
4
5
10
6
7
8
9
11
12
13
15
16
17
18
19
20
21
22
23
27814
27815
28083

EOG559ZXR

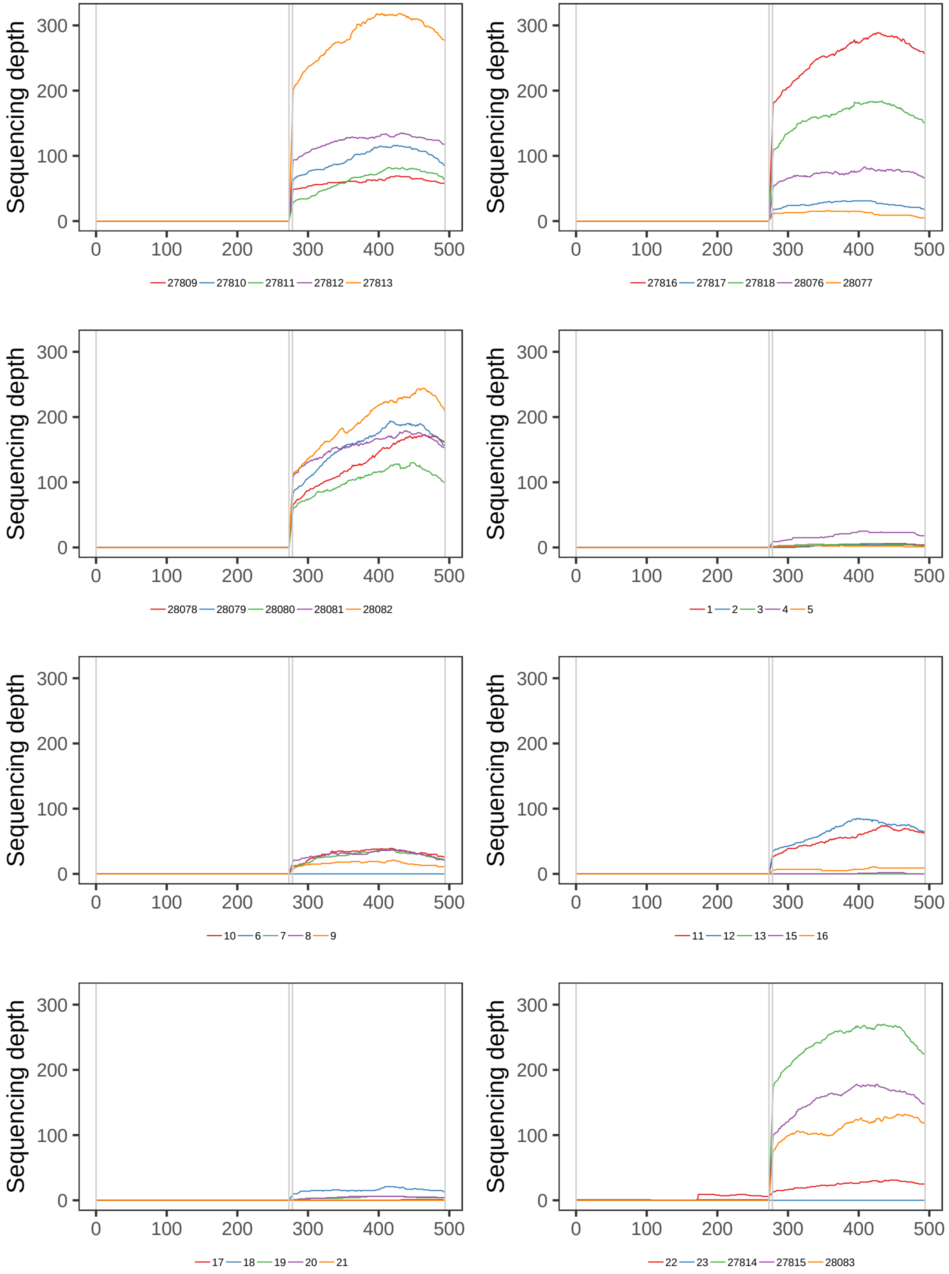
Sequencing depth
0
100
200
300
400
500
27809 27810 27811 27812 27813
27816 27817 27818 28076 28077
28078 28079 28080 28081 28082
1 2 3 4 5
10 6 7 8 9
11 12 13 15 16
17 18 19 20 21
22 23 27814 27815 28083

EOG576HGM

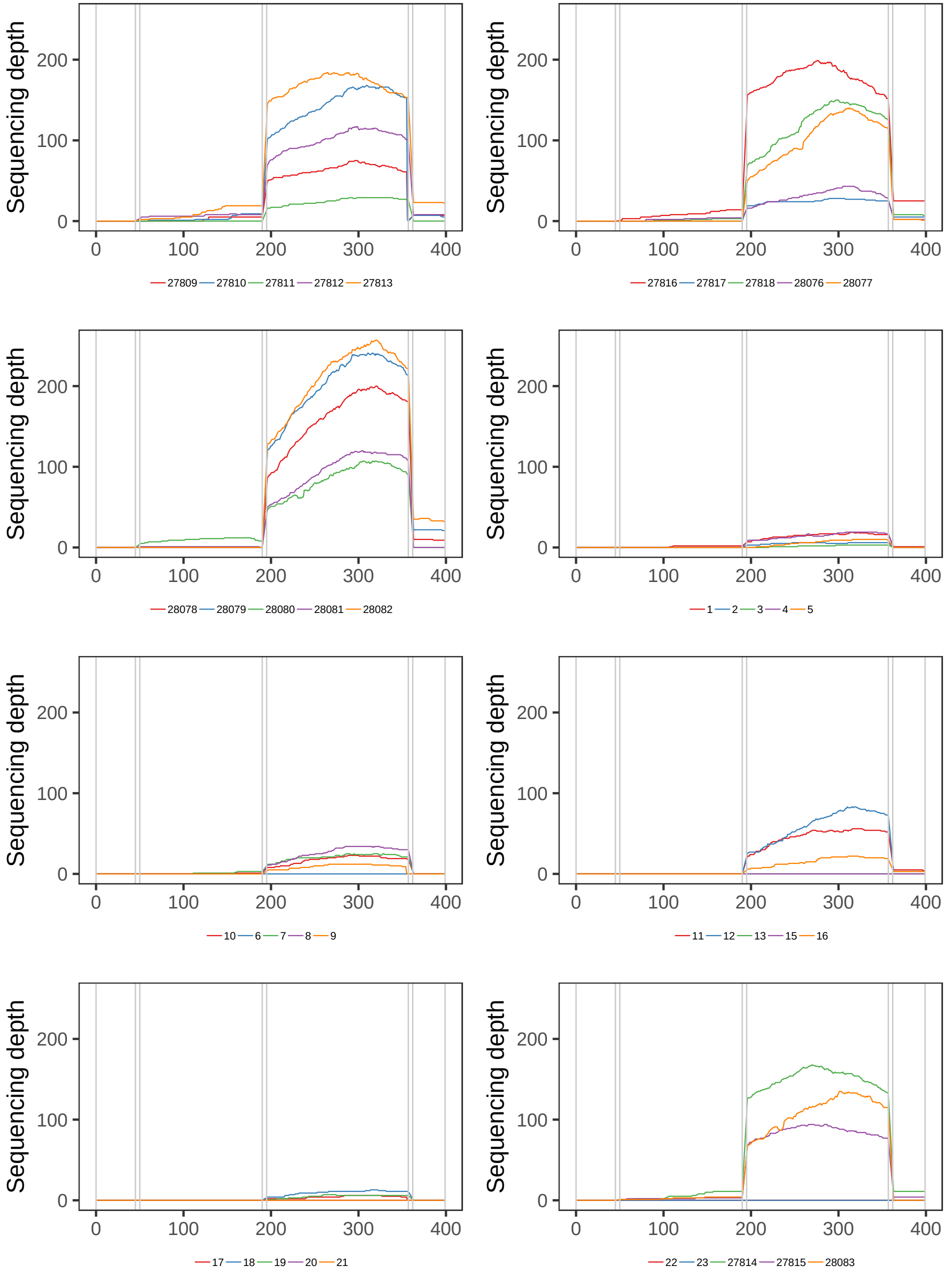

Sequencing depth
0
100
200
0
100
200
300
400
27809
27810
27811
27812
27813
27816
27817
27818
28076
28077
28078
28079
28080
28081
28082
1
2
3
4
5
10
6
7
8
9
11
12
13
15
16
17
18
19
20
21
22
23
27814
27815
28083

EOG5K3JC4

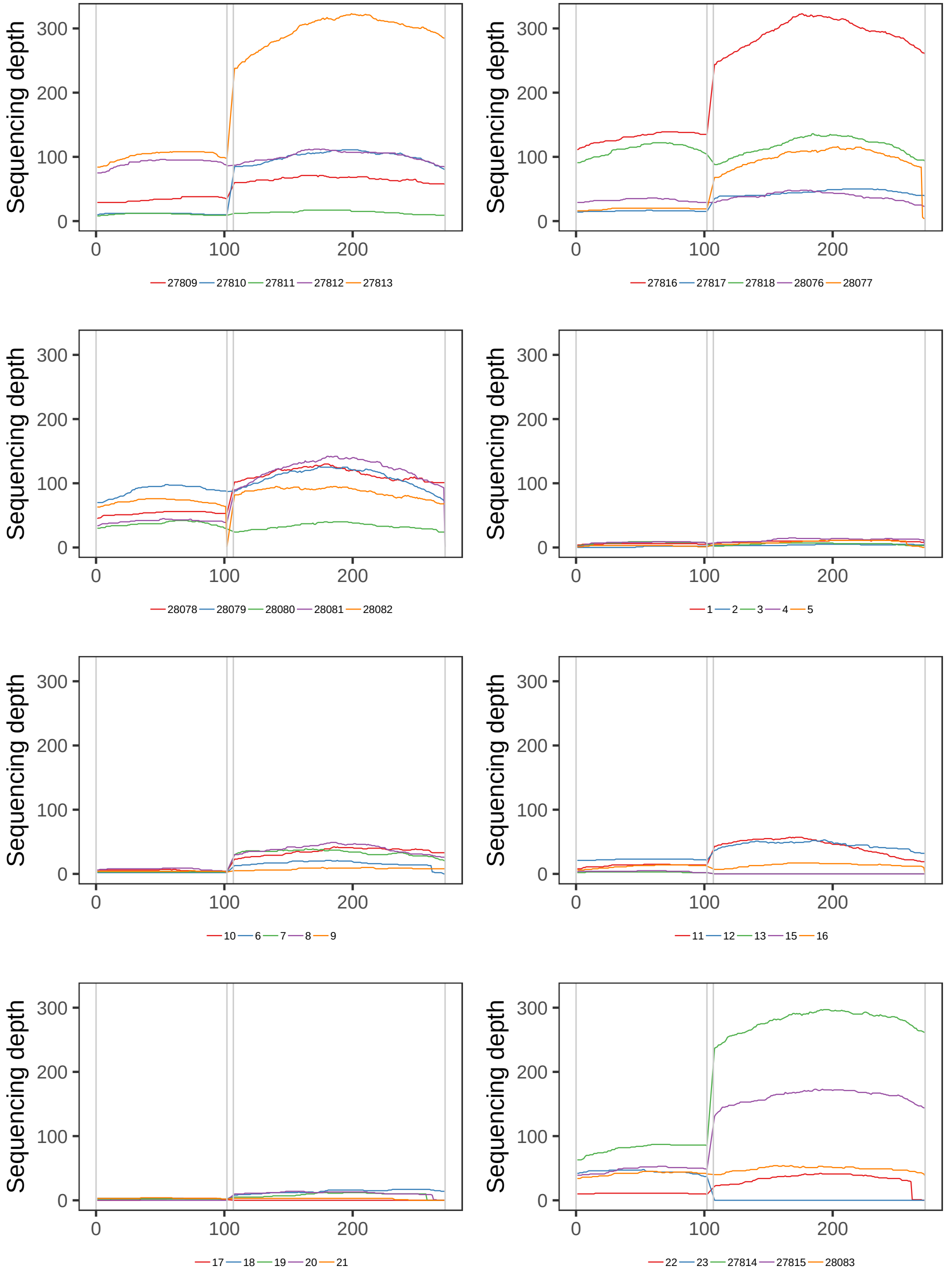

Sequencing depth
0
100
200
300
27809
27810
27811
27812
27813
27816
27817
27818
28076
28077
28078
28079
28080
28081
28082
1
2
3
4
5
10
6
7
8
9
11
12
13
15
16
17
18
19
20
21
22
23
27814
27815
28083

EOG5MGQPM

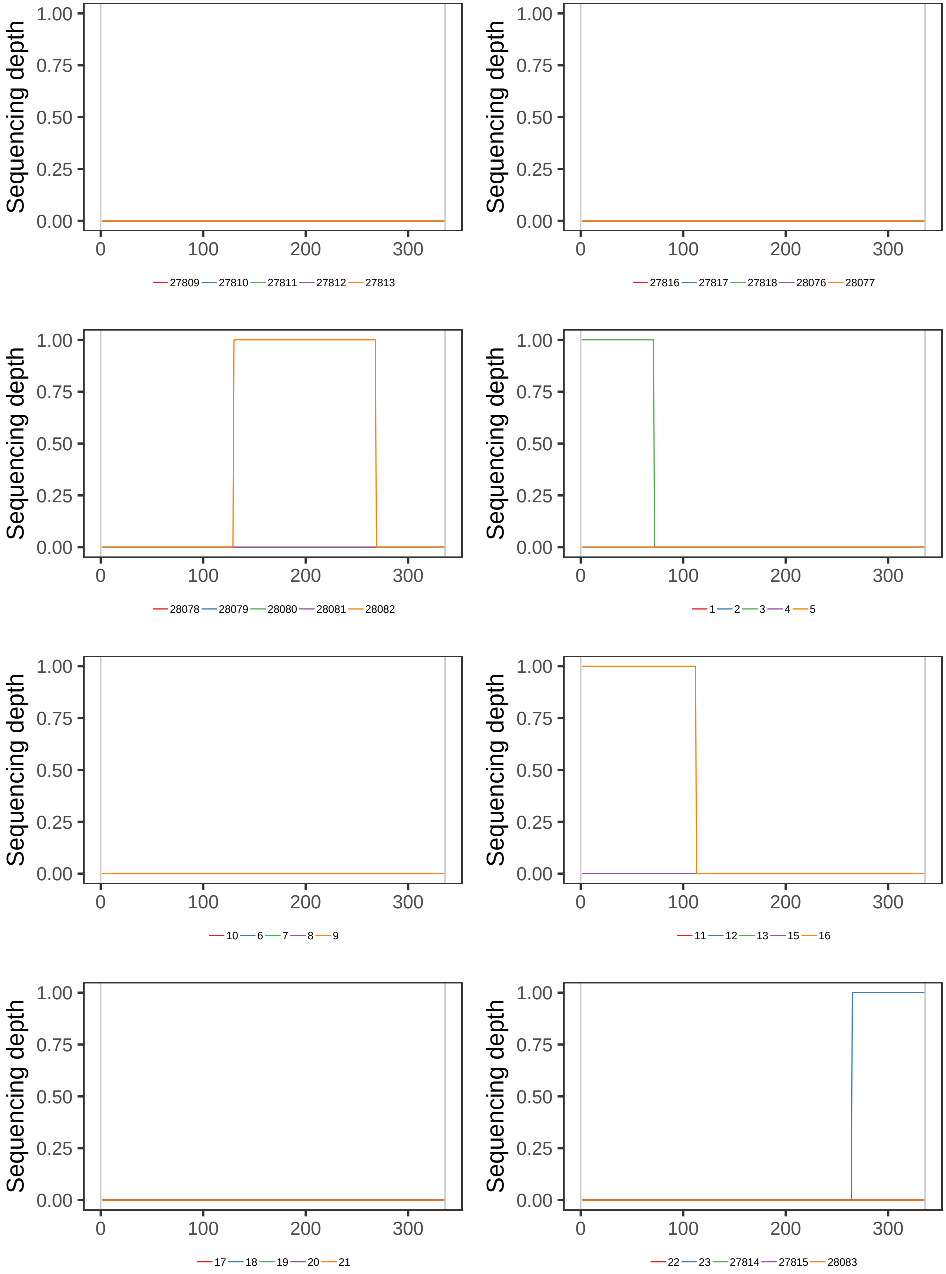
Sequencing depth
1.00
0.75
0.50
0.25
0.00
0
100
200
300
27809 27810 27811 27812 27813
27816 27817 27818 28076 28077
28078 28079 28080 28081 28082
1 2 3 4 5
10 6 7 8 9
11 12 13 15 16
17 18 19 20 21
22 23 27814 27815 28083

EOG5DJHC3

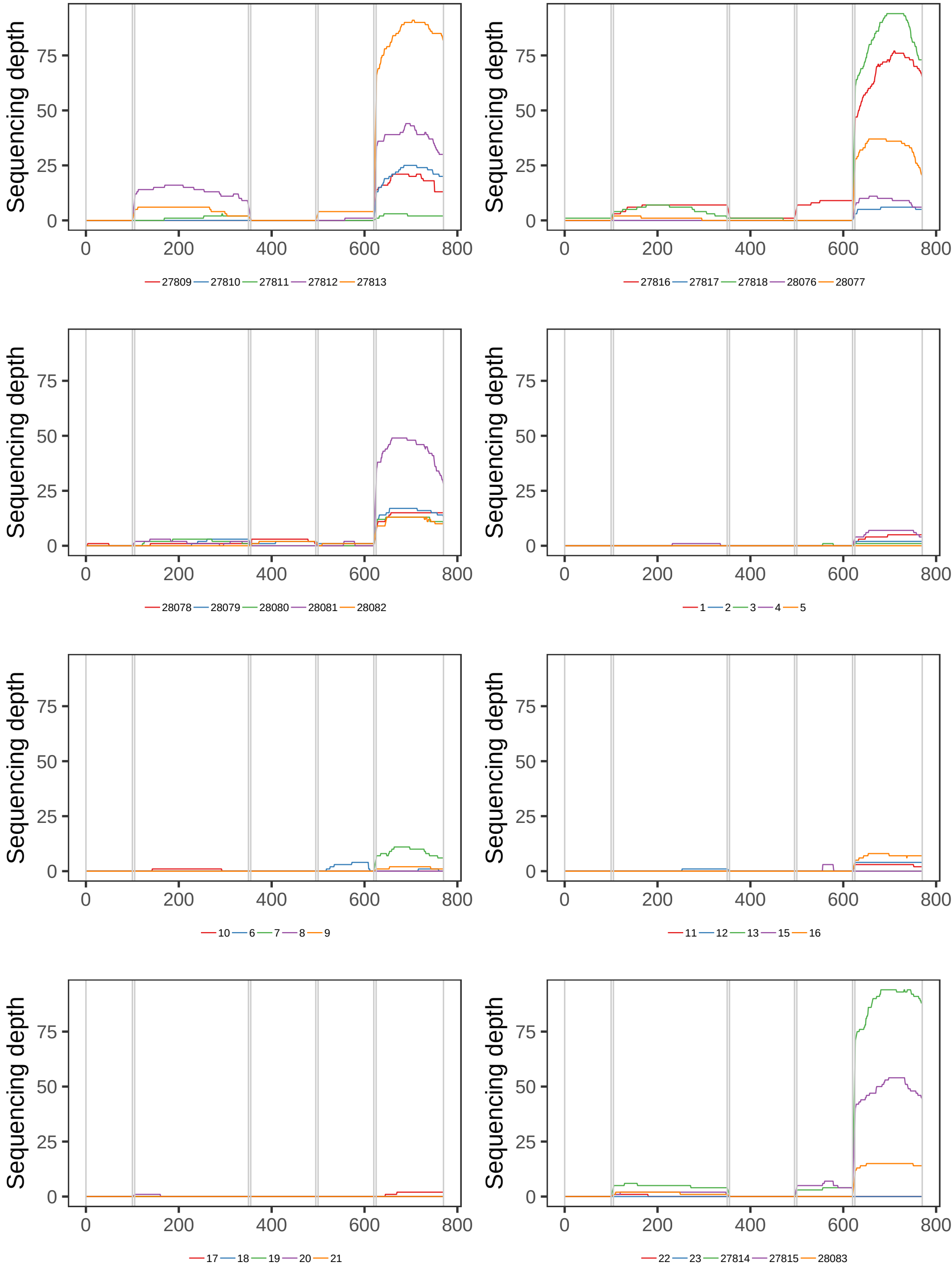
Sequencing depth
0
25
50
75
0
200
400
600
800
27809
27810
27811
27812
27813
27816
27817
27818
28076
28077
28078
28079
28080
28081
28082
1
2
3
4
5
10
6
7
8
9
11
12
13
15
16
17
18
19
20
21
22
23
27814
27815
28083

EOG5NCJVK

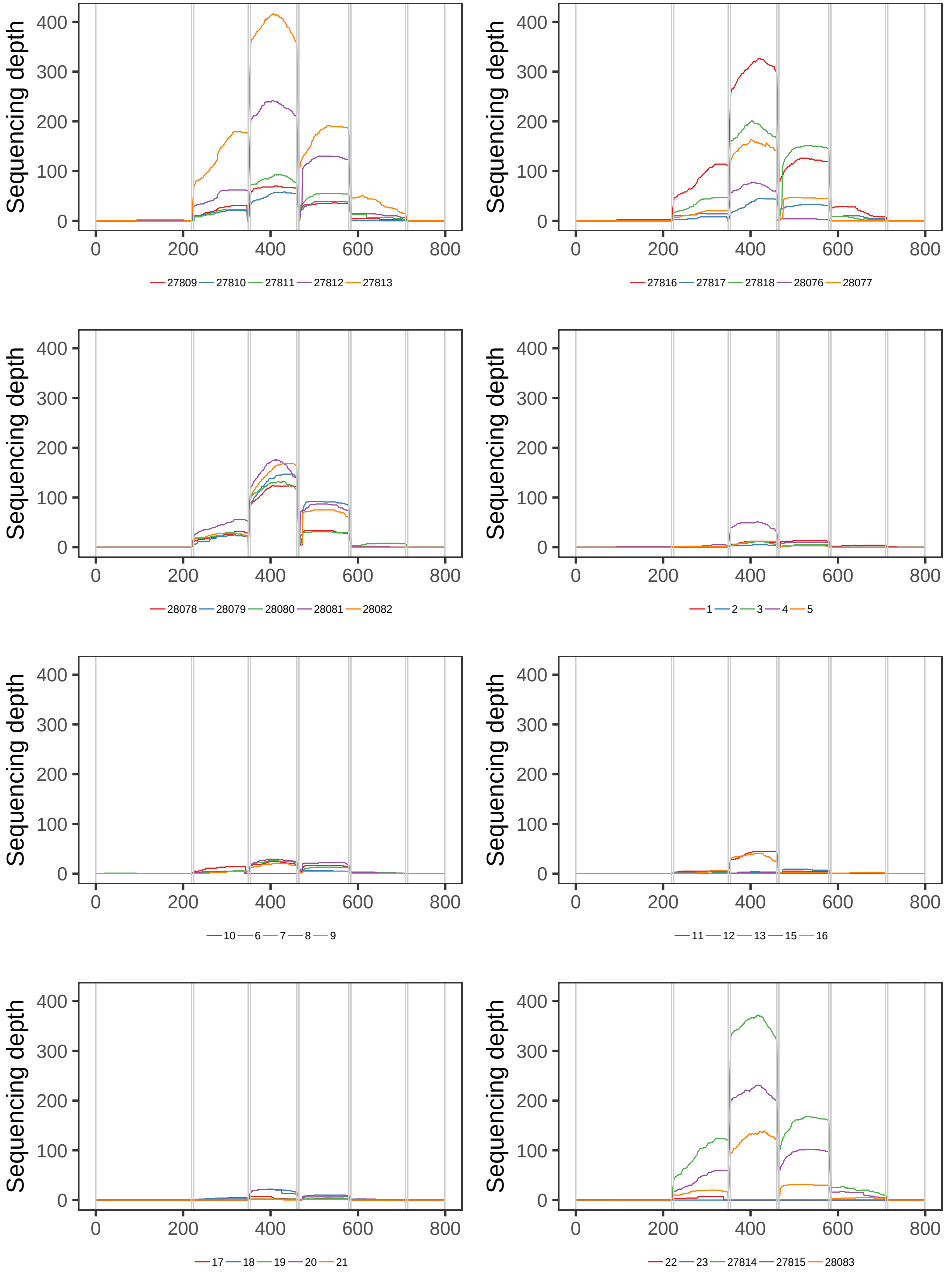
Sequencing depth
0
100
200
300
400
0
200
400
600
800
27809 27810 27811 27812 27813
27816 27817 27818 28076 28077
28078 28079 28080 28081 28082
1 2 3 4 5
10 6 7 8 9
11 12 13 15 16
17 18 19 20 21
22 23 27814 27815 28083

EOG50000C

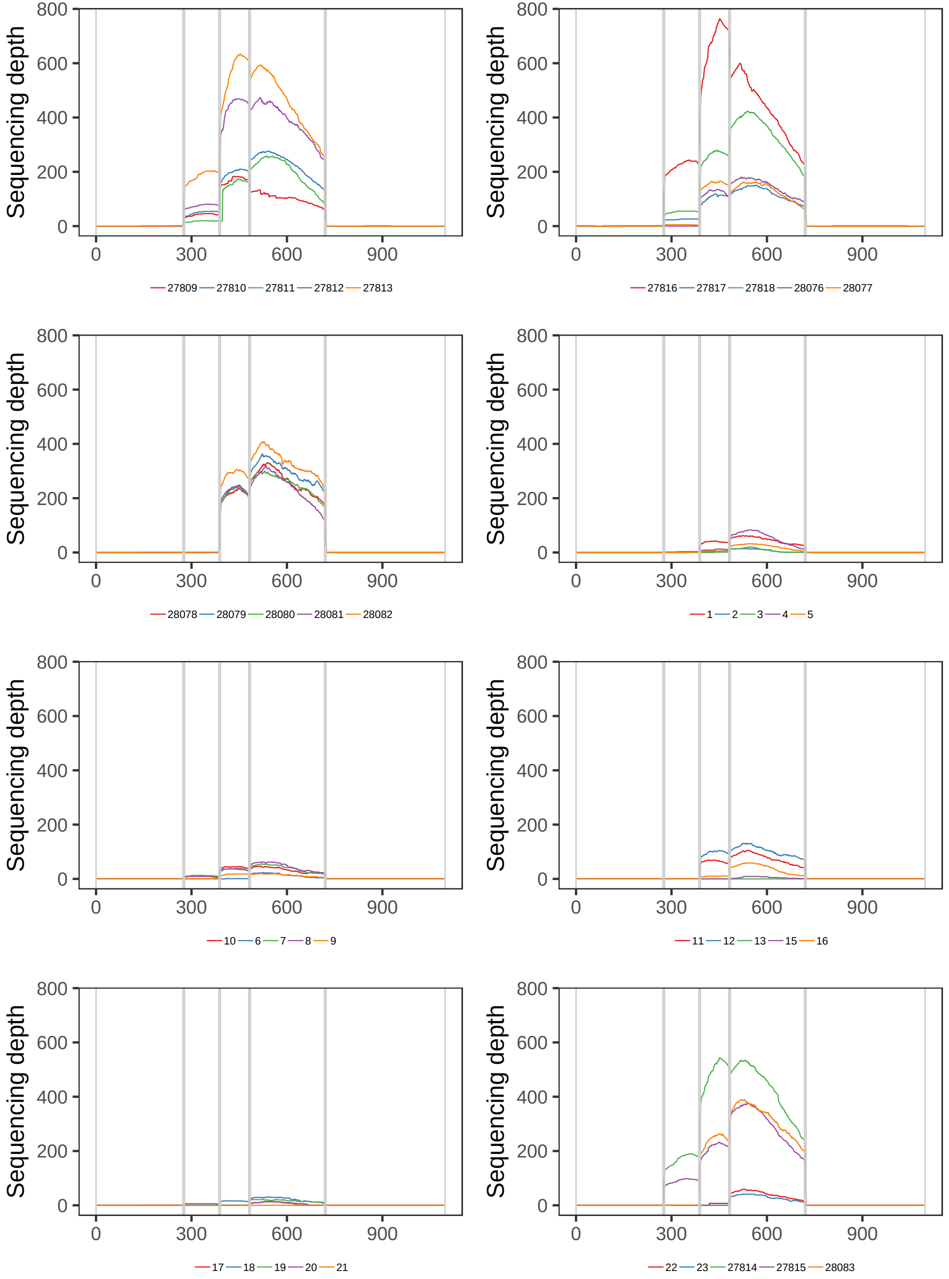
Sequencing depth
27809 27810 27811 27812 27813
27816 27817 27818 28076 28077
28078 28079 28080 28081 28082
1 2 3 4 5
10 6 7 8 9
11 12 13 15 16
17 18 19 20 21
22 23 27814 27815 28083

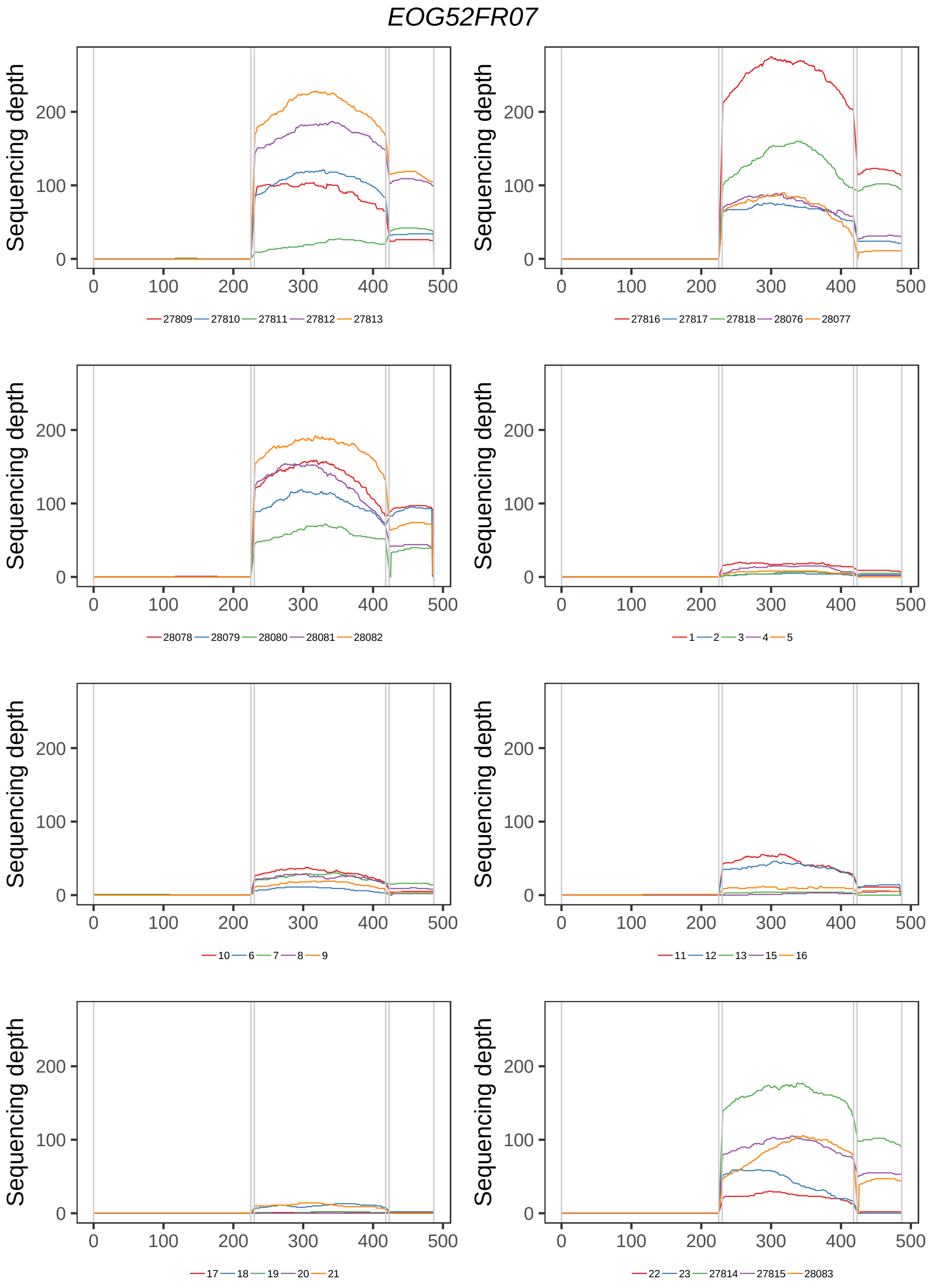
EOG52FR07
Sequencing depth
0
100
200
300
400
500
27809 27810 27811 27812 27813
27816 27817 27818 28076 28077
28078 28079 28080 28081 28082
1 2 3 4 5
10 6 7 8 9
11 12 13 15 16
17 18 19 20 21
22 23 27814 27815 28083

EOG5BRV22

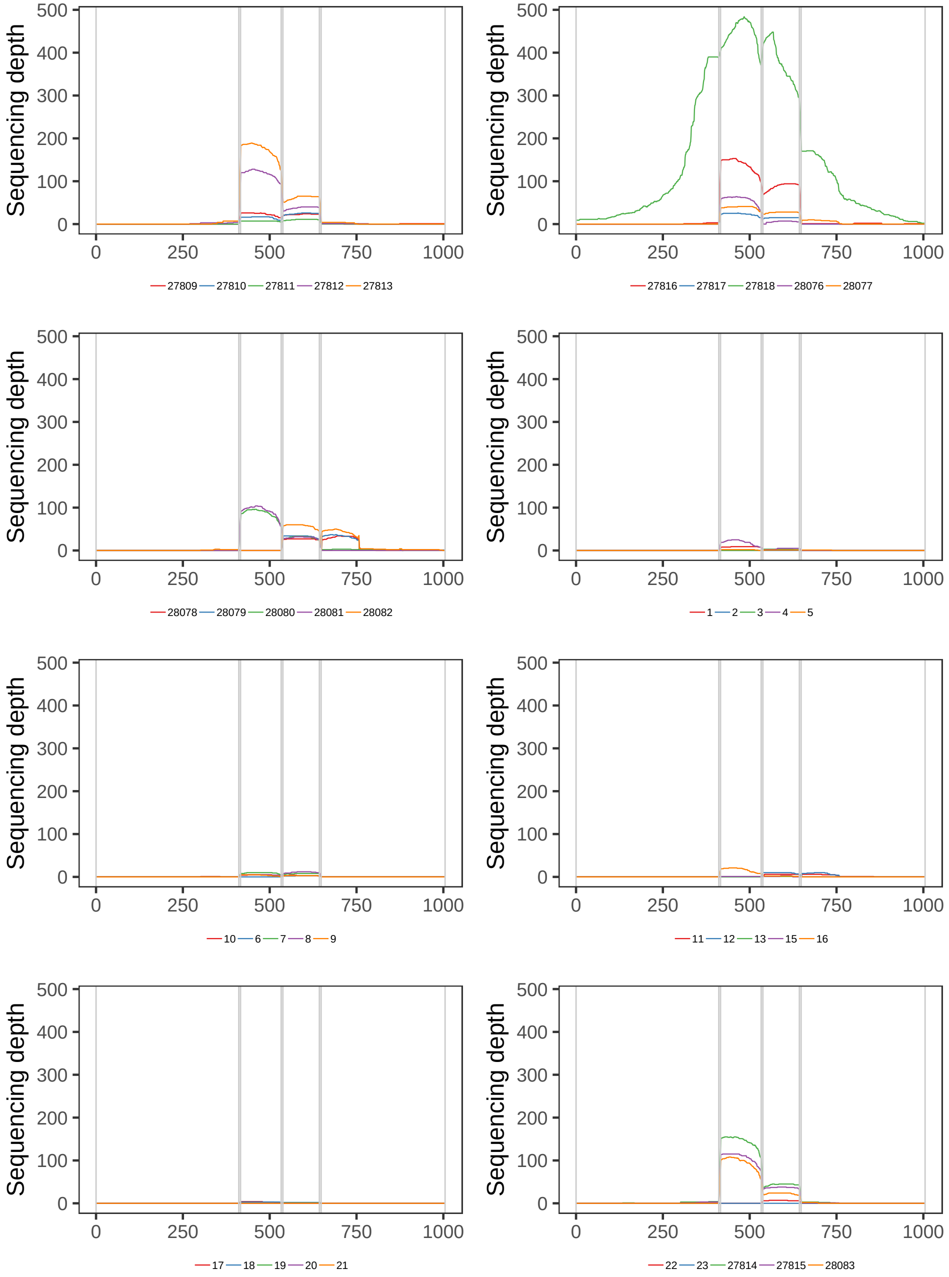
Sequencing depth
0
100
200
300
400
500
0
250
500
750
1000
27809 27810 27811 27812 27813
27816 27817 27818 28076 28077
28078 28079 28080 28081 28082
1 2 3 4 5
10 6 7 8 9
11 12 13 15 16
17 18 19 20 21
22 23 27814 27815 28083

EOG5H44KK

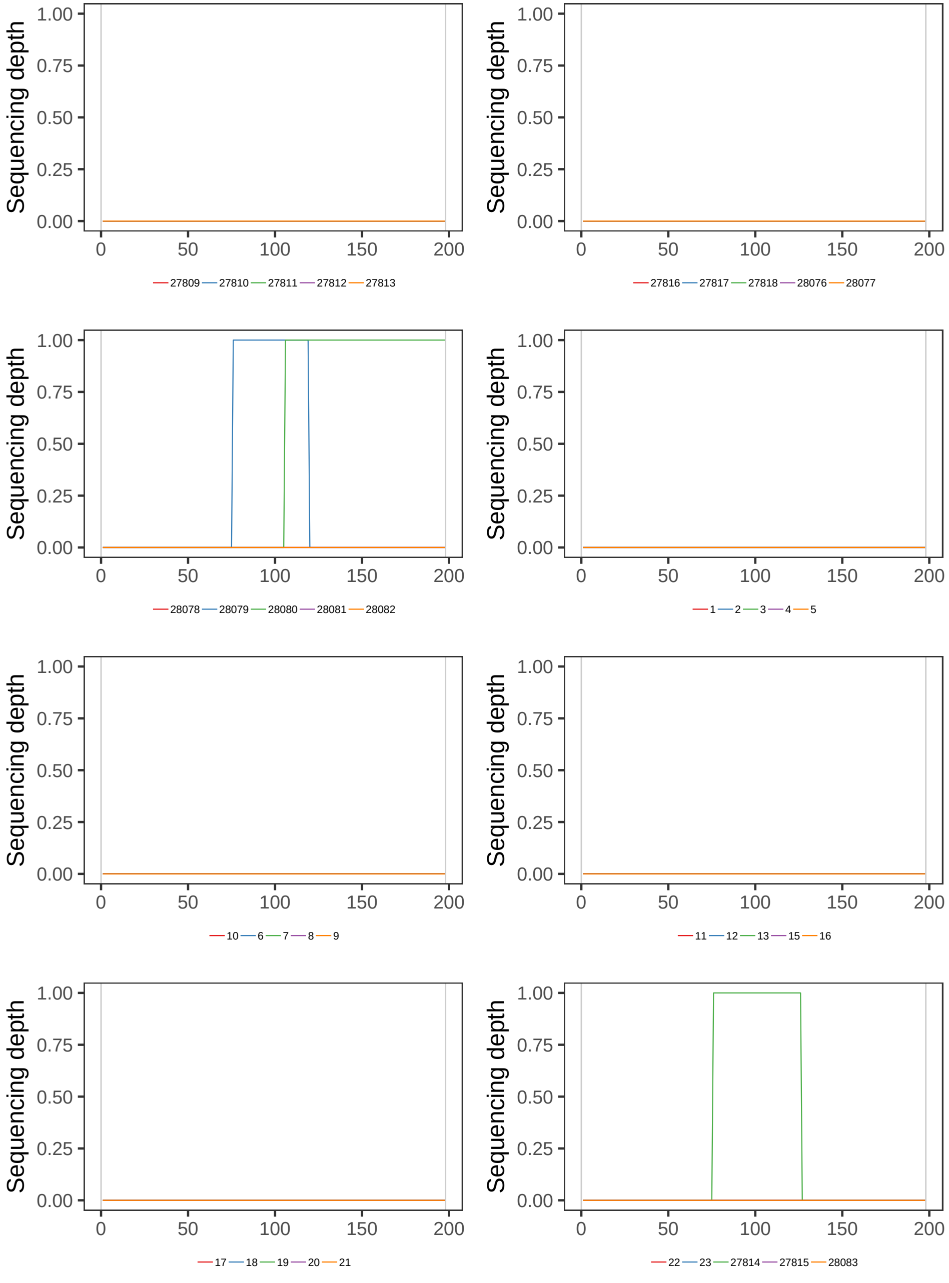

Sequencing depth
1.00
0.75
0.50
0.25
0.00
0
50
100
150
200
27809
27810
27811
27812
27813
27816
27817
27818
28076
28077
28078
28079
28080
28081
28082
1
2
3
4
5
10
6
7
8
9
11
12
13
15
16
17
18
19
20
21
22
23
27814
27815
28083

EOG5HX3GW

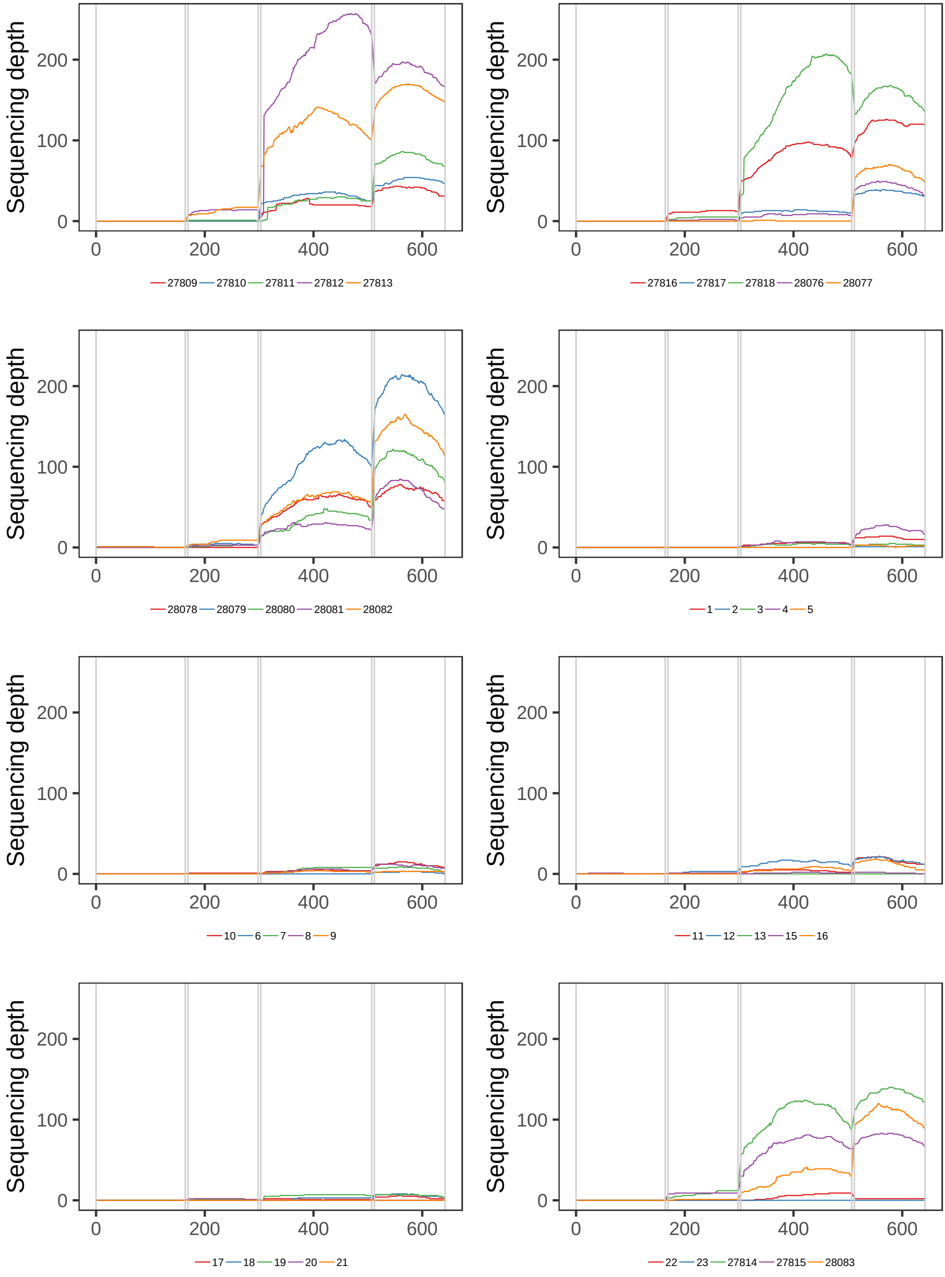
Sequencing depth
0
100
200
0
200
400
600
27809 27810 27811 27812 27813
27816 27817 27818 28076 28077
28078 28079 28080 28081 28082
1 2 3 4 5
10 6 7 8 9
11 12 13 15 16
17 18 19 20 21
22 23 27814 27815 28083

EOG5PG4GF

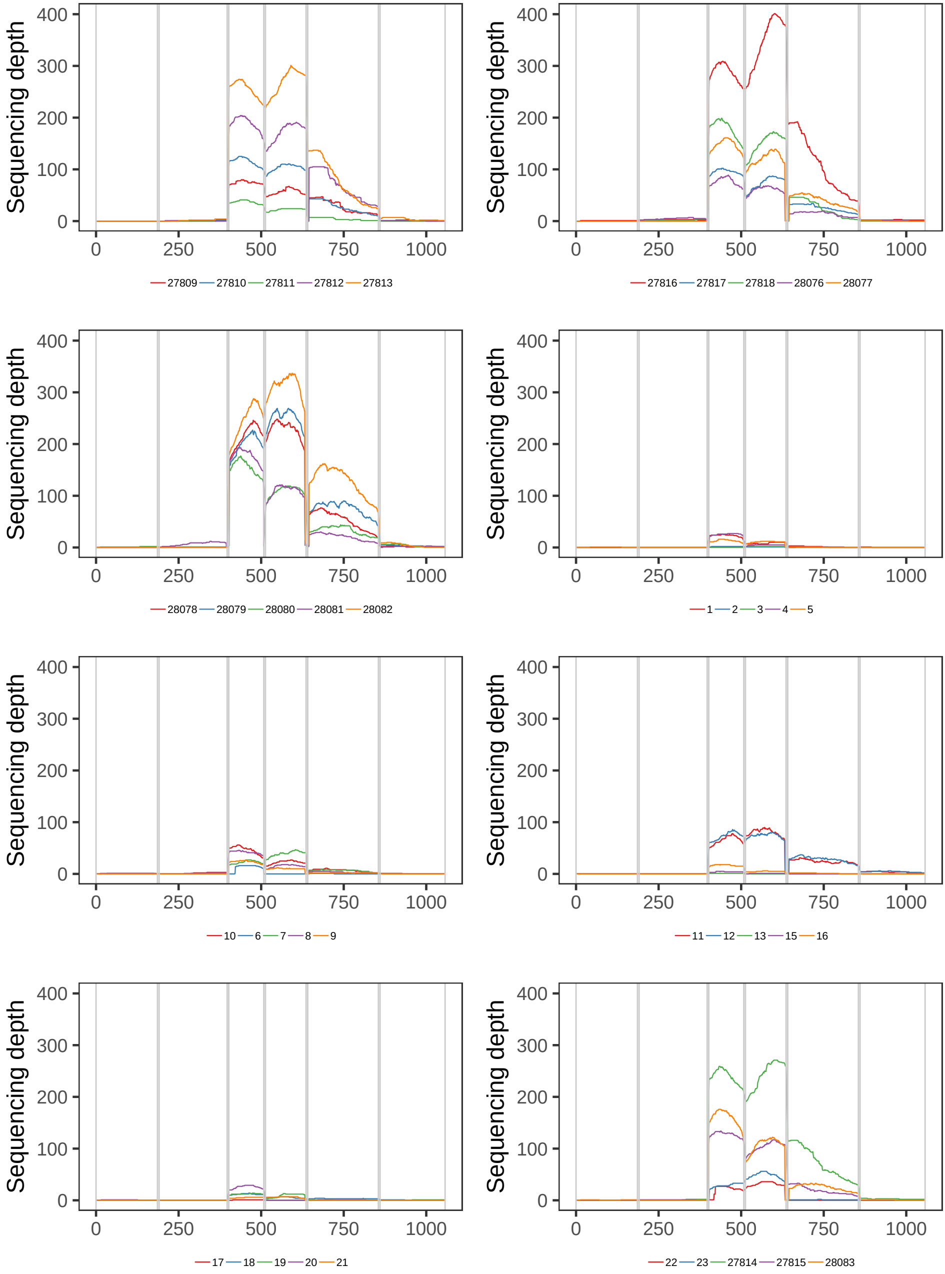
Sequencing depth
0
100
200
300
400
0
250
500
750
1000
27809 27810 27811 27812 27813
27816 27817 27818 28076 28077
28078 28079 28080 28081 28082
1 2 3 4 5
10 6 7 8 9
11 12 13 15 16
17 18 19 20 21
22 23 27814 27815 28083

EOG5S1RQG

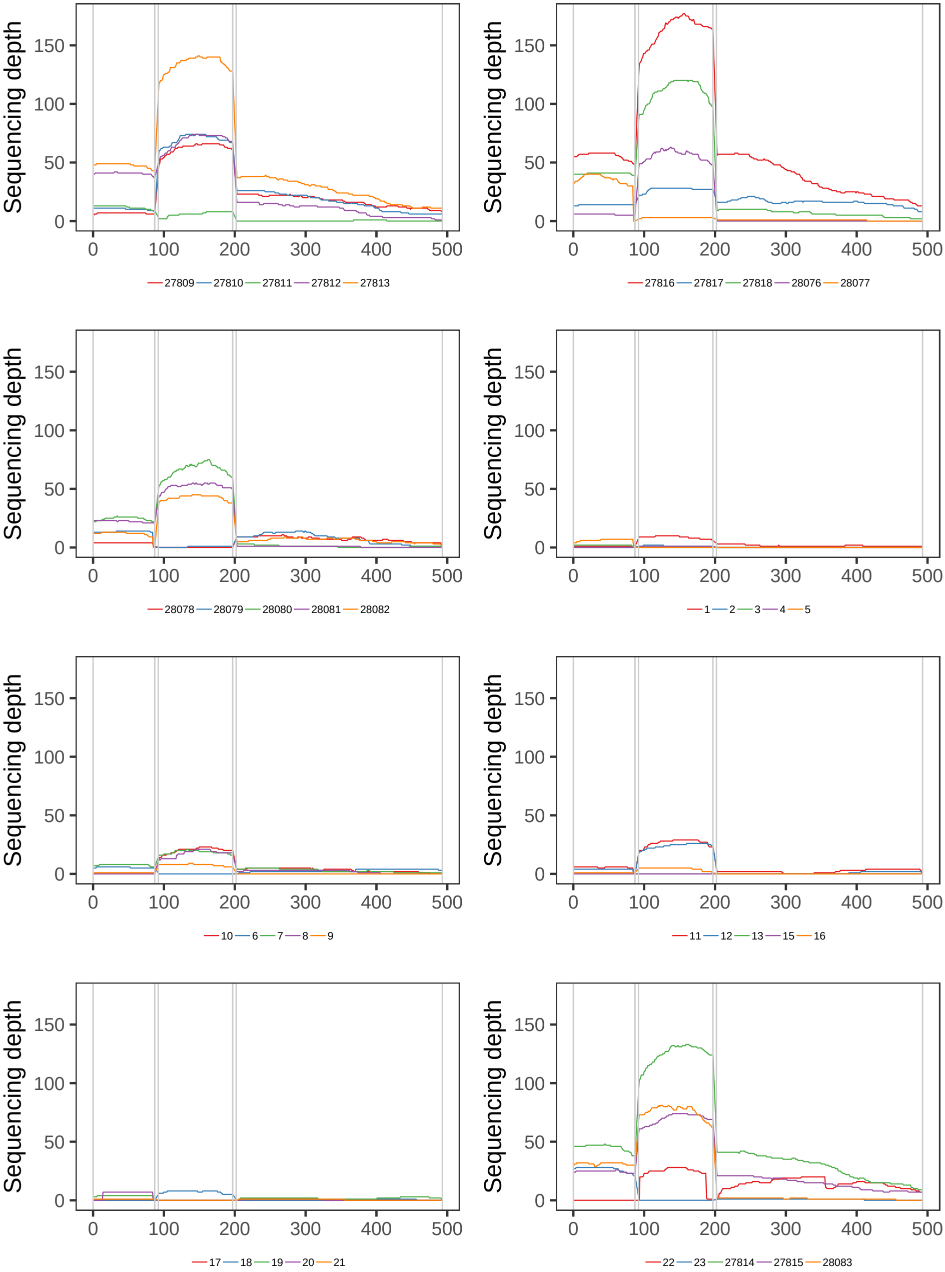
Sequencing depth
0
50
100
150
0
100
200
300
400
500
27809 27810 27811 27812 27813
27816 27817 27818 28076 28077
28078 28079 28080 28081 28082
1 2 3 4 5
10 6 7 8 9
11 12 13 15 16
17 18 19 20 21
22 23 27814 27815 28083

EOG5VMCWC

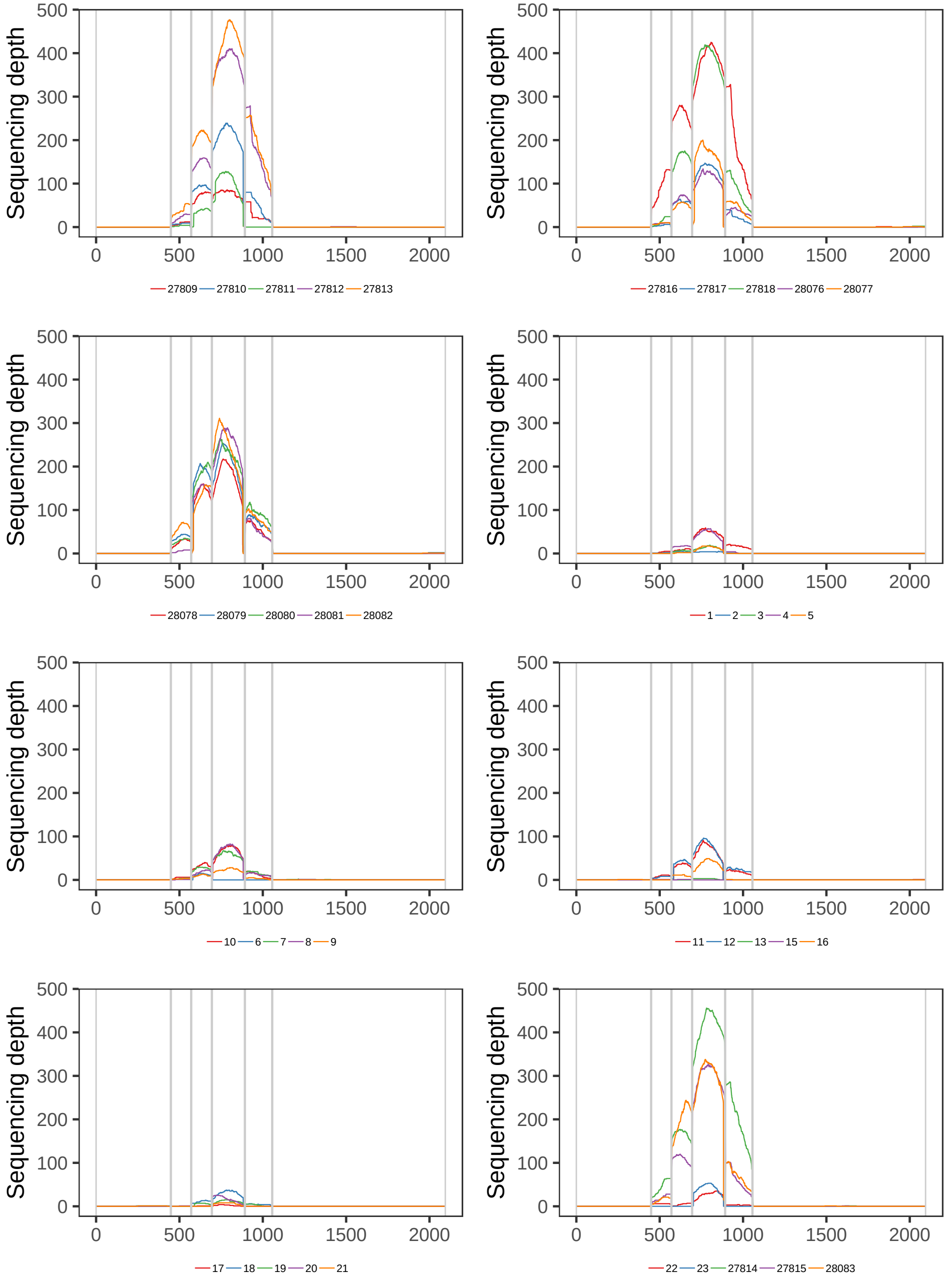

Sequencing depth
0
100
200
300
400
500
0
500
1000
1500
2000
27809 27810 27811 27812 27813
27816 27817 27818 28076 28077
28078 28079 28080 28081 28082
1 2 3 4 5
10 6 7 8 9
11 12 13 15 16
17 18 19 20 21
22 23 27814 27815 28083

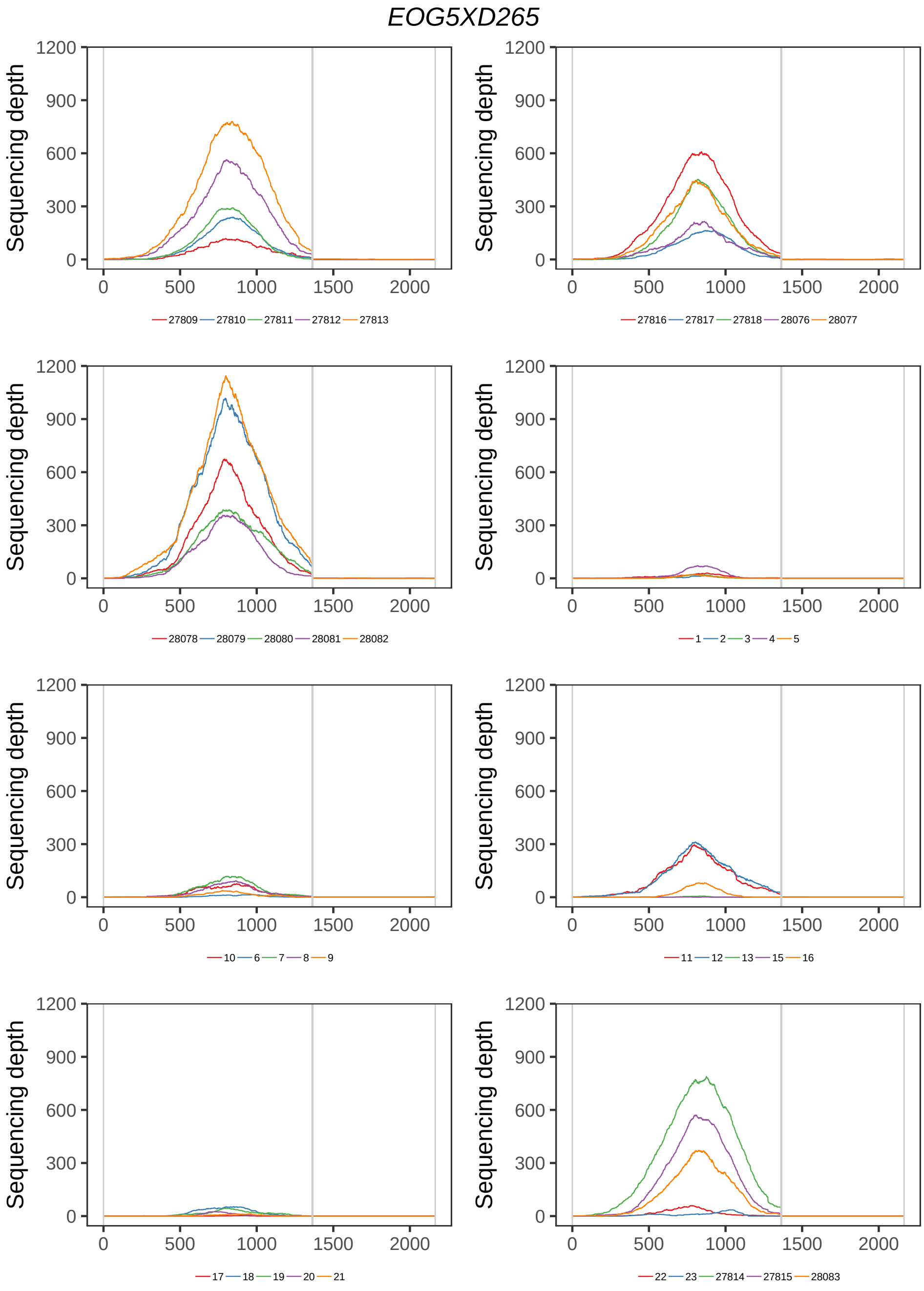
EOG5XD265
Sequencing depth
0
300
600
900
1200
0
500
1000
1500
2000
27809 27810 27811 27812 27813
27816 27817 27818 28076 28077
28078 28079 28080 28081 28082
1 2 3 4 5
10 6 7 8 9
11 12 13 15 16
17 18 19 20 21
22 23 27814 27815 28083

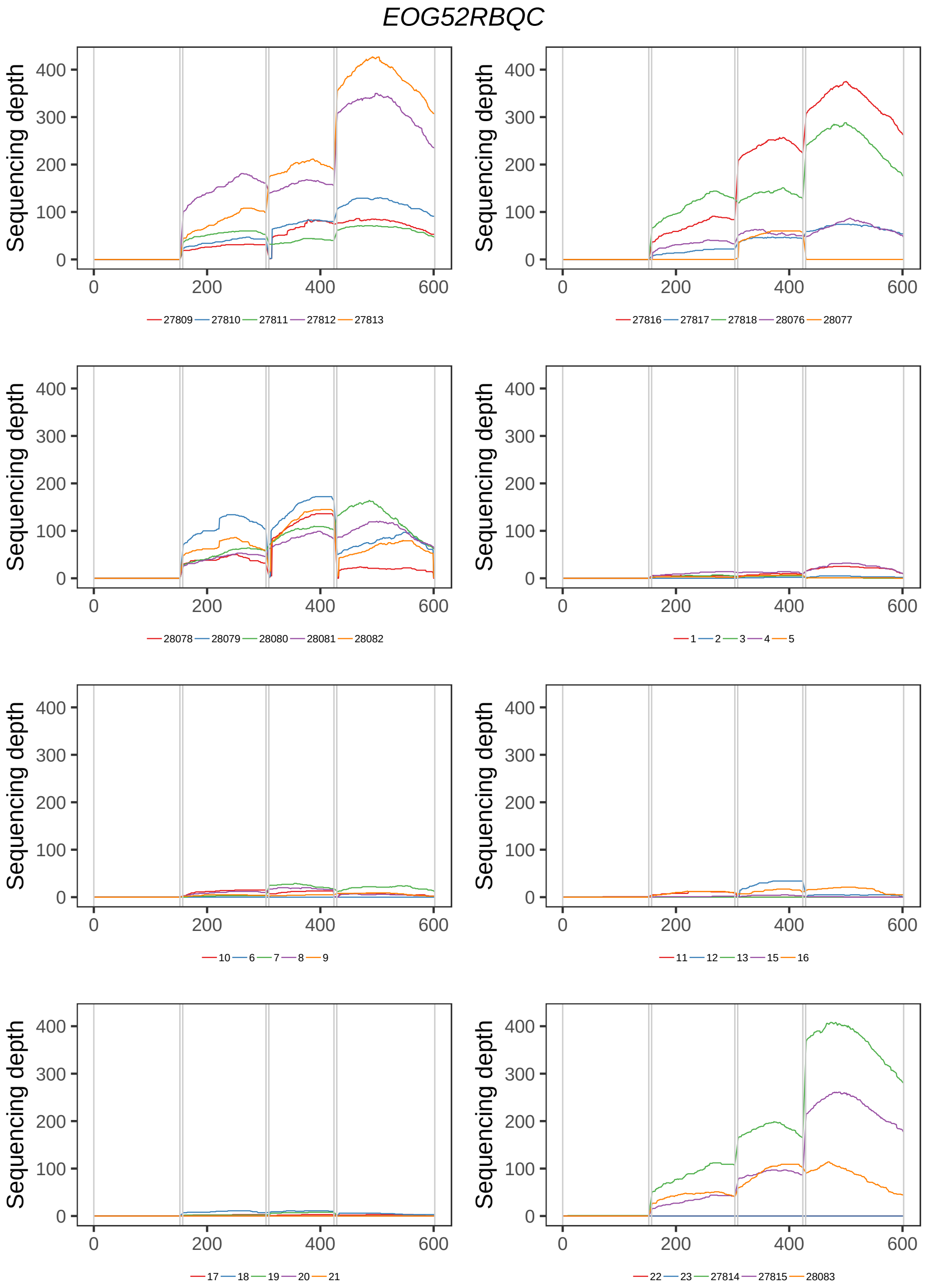
EOG52RBQC
Sequencing depth
0
100
200
300
400
0
200
400
600
27809 27810 27811 27812 27813
27816 27817 27818 28076 28077
28078 28079 28080 28081 28082
1 2 3 4 5
10 6 7 8 9
11 12 13 15 16
17 18 19 20 21
22 23 27814 27815 28083

EOG541NSZ

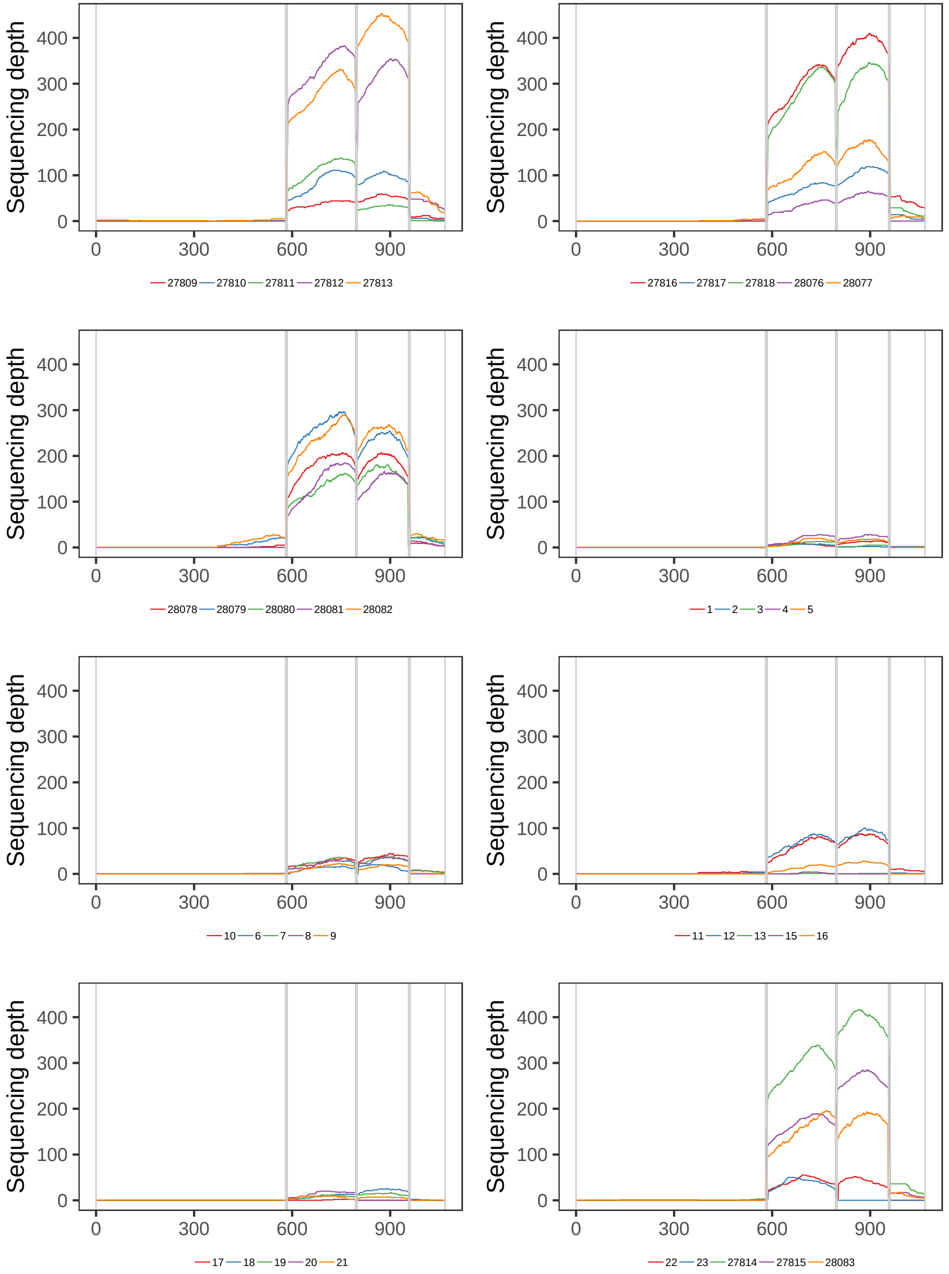
Sequencing depth
0
100
200
300
400
0
300
600
900
27809 27810 27811 27812 27813
27816 27817 27818 28076 28077
28078 28079 28080 28081 28082
1 2 3 4 5
10 6 7 8 9
11 12 13 15 16
17 18 19 20 21
22 23 27814 27815 28083

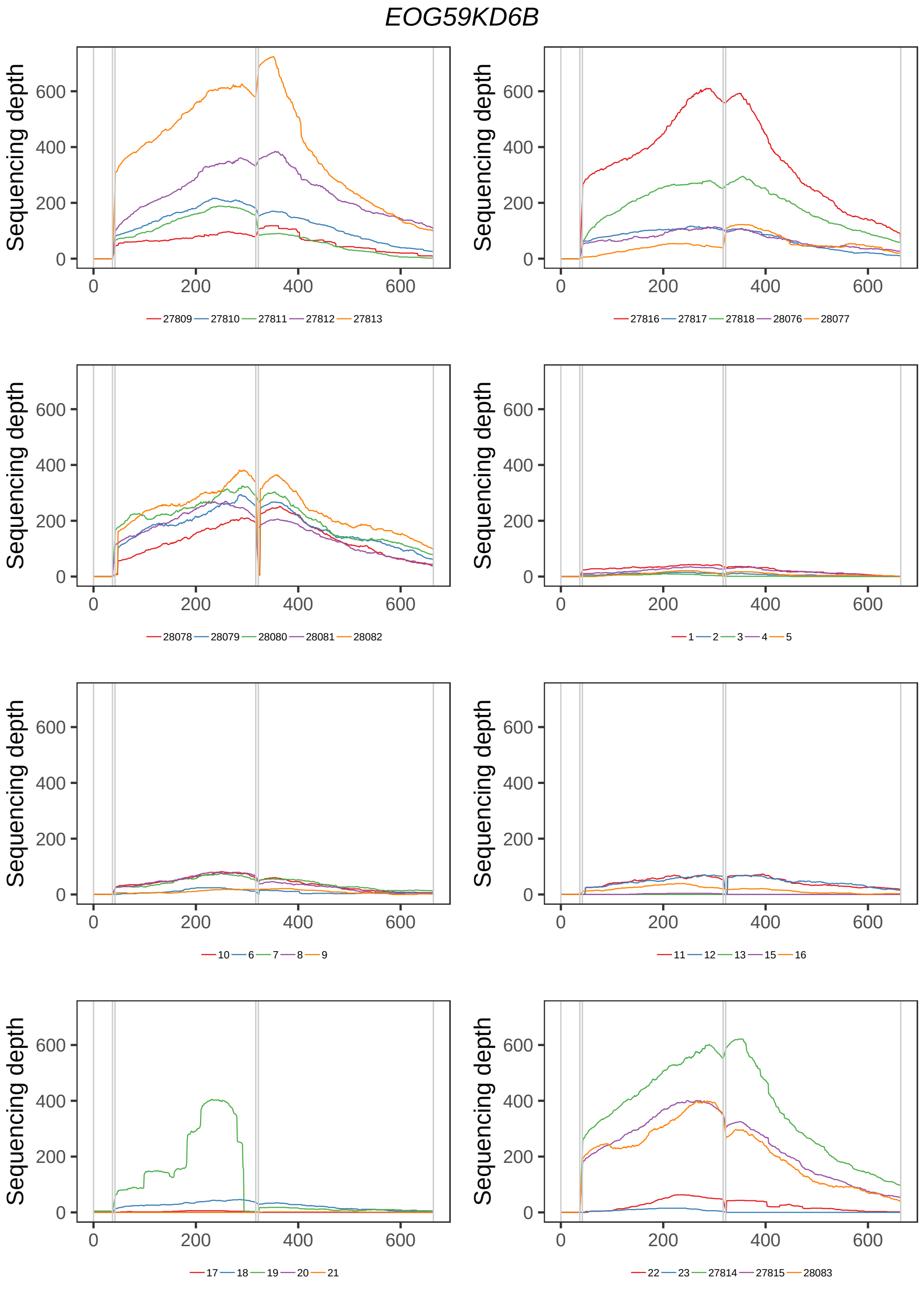
EOG59KD6B
Sequencing depth
27809 27810 27811 27812 27813
27816 27817 27818 28076 28077
28078 28079 28080 28081 28082
1 2 3 4 5
10 6 7 8 9
11 12 13 15 16
17 18 19 20 21
22 23 27814 27815 28083

EOG5DNCMM

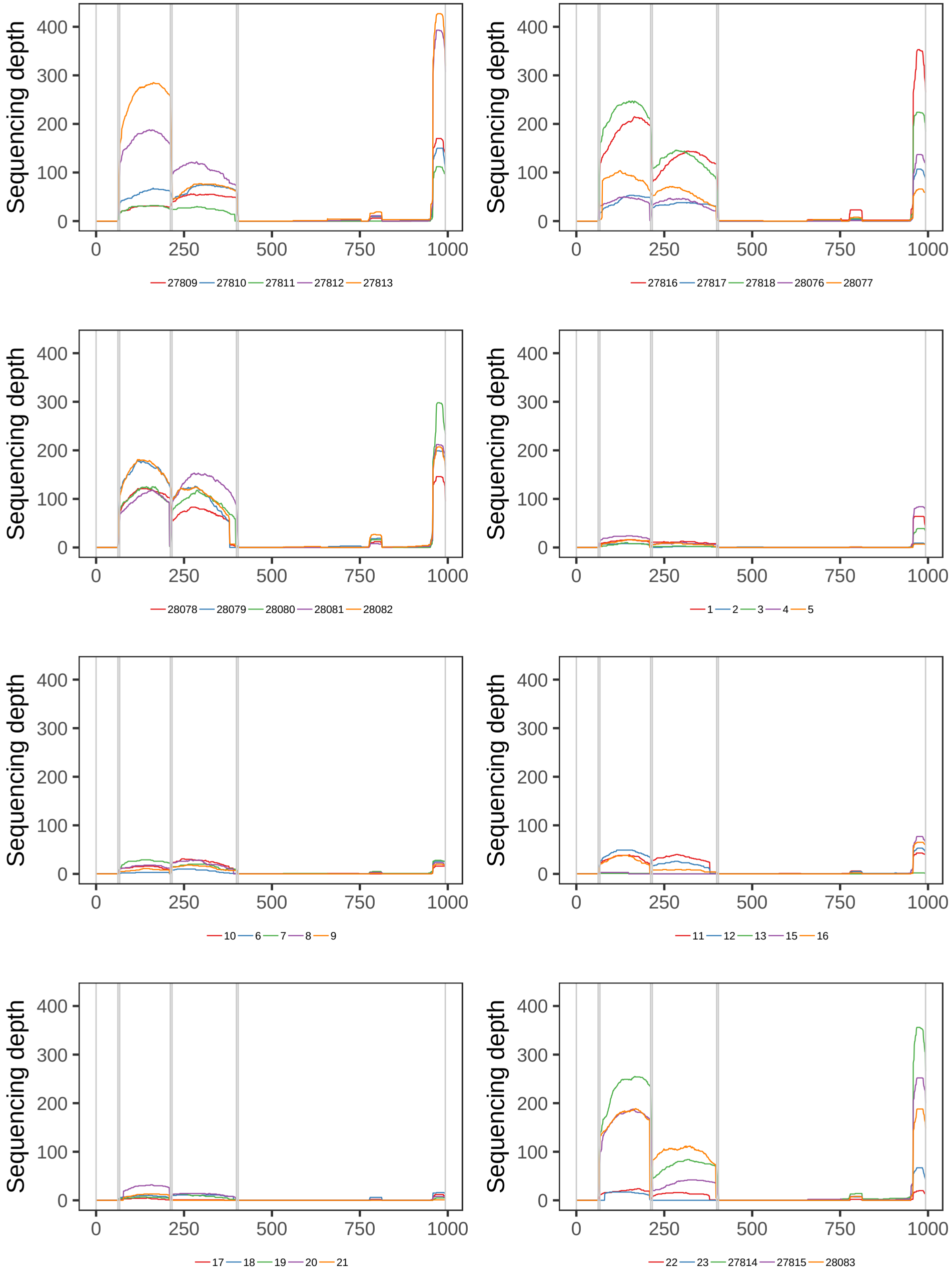
Sequencing depth
0
100
200
300
400
0
250
500
750
1000
27809 27810 27811 27812 27813
27816 27817 27818 28076 28077
28078 28079 28080 28081 28082
1 2 3 4 5
10 6 7 8 9
11 12 13 15 16
17 18 19 20 21
22 23 27814 27815 28083

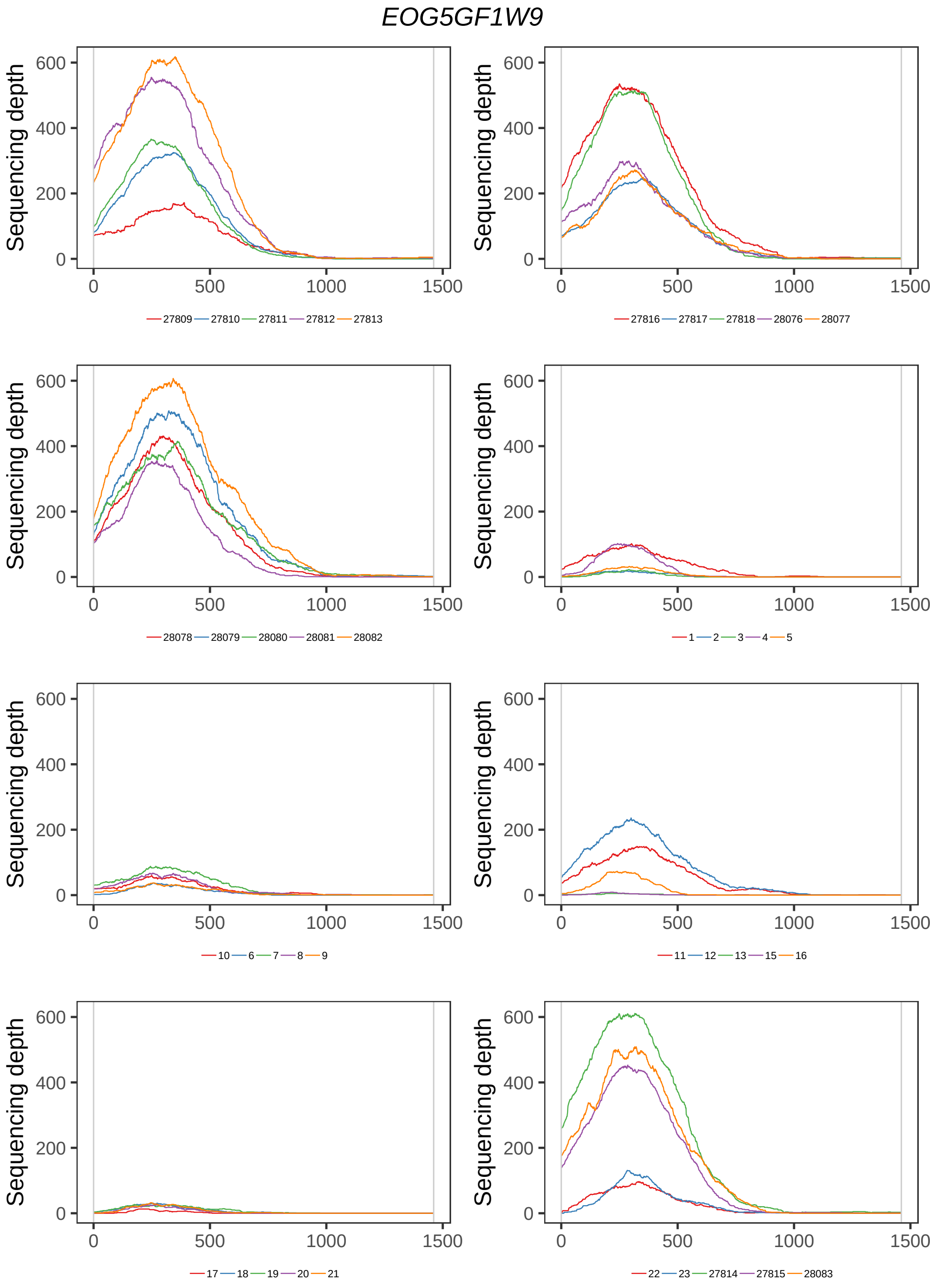
EOG5GF1W9
Sequencing depth
0
200
400
600
0
500
1000
1500
27809 27810 27811 27812 27813
27816 27817 27818 28076 28077
28078 28079 28080 28081 28082
1 2 3 4 5
10 6 7 8 9
11 12 13 15 16
17 18 19 20 21
22 23 27814 27815 28083

EOG5QBZNB

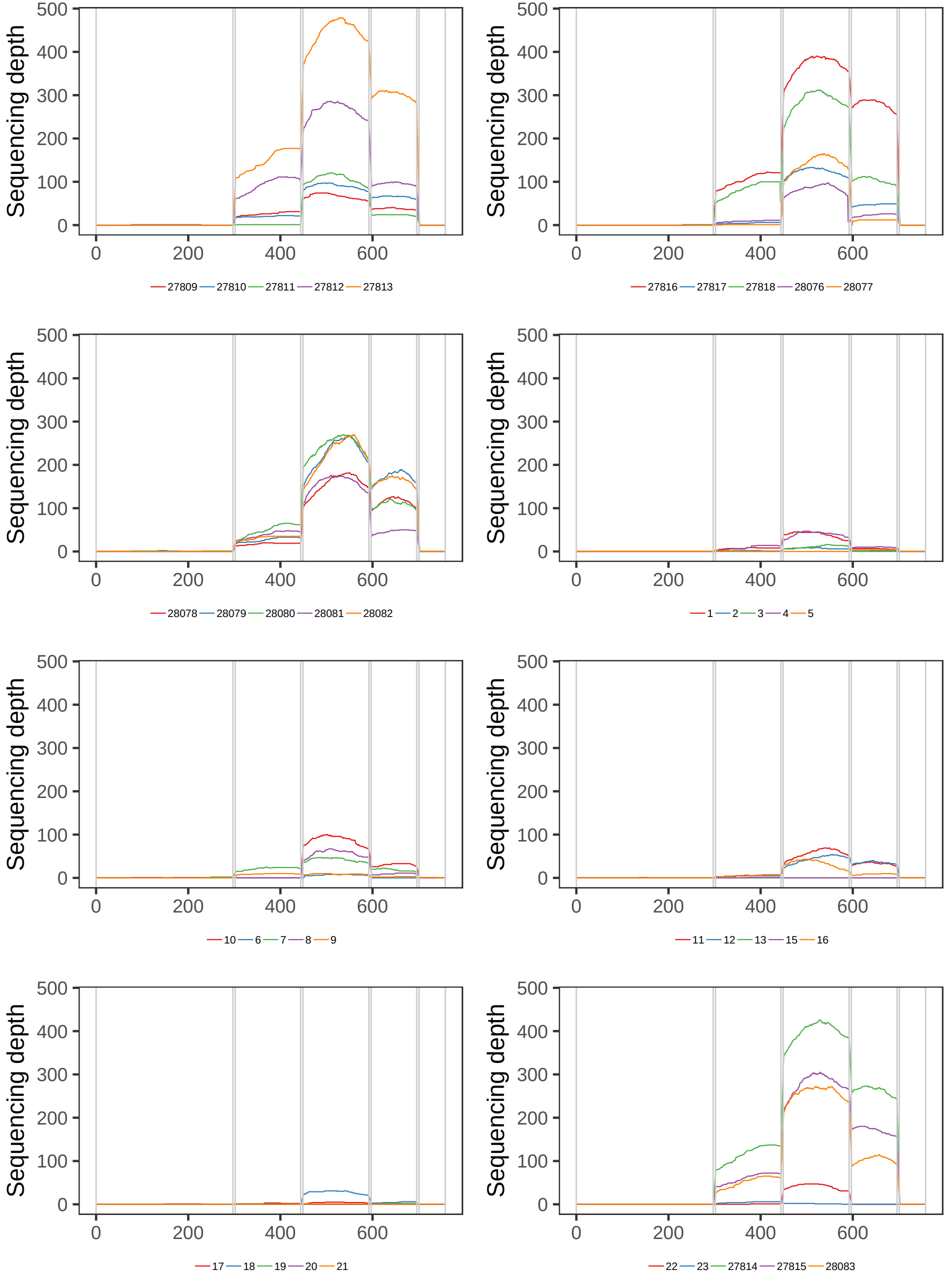
Sequencing depth
0
100
200
300
400
500
0
200
400
600
27809 27810 27811 27812 27813
27816 27817 27818 28076 28077
28078 28079 28080 28081 28082
1 2 3 4 5
10 6 7 8 9
11 12 13 15 16
17 18 19 20 21
22 23 27814 27815 28083

EOG5VHHP8

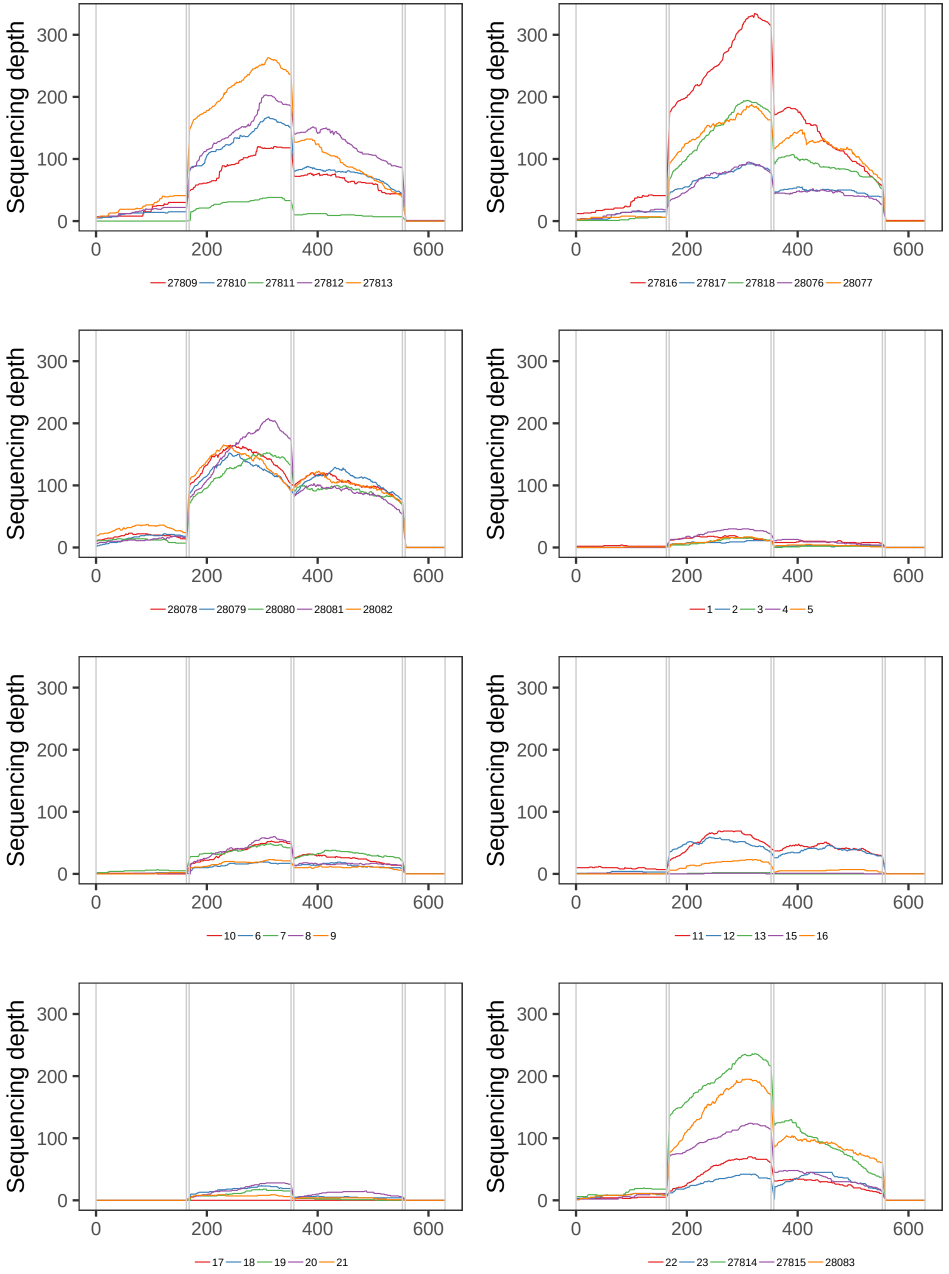
Sequencing depth
0
100
200
300
0
200
400
600
27809 27810 27811 27812 27813
27816 27817 27818 28076 28077
28078 28079 28080 28081 28082
1 2 3 4 5
10 6 7 8 9
11 12 13 15 16
17 18 19 20 21
22 23 27814 27815 28083

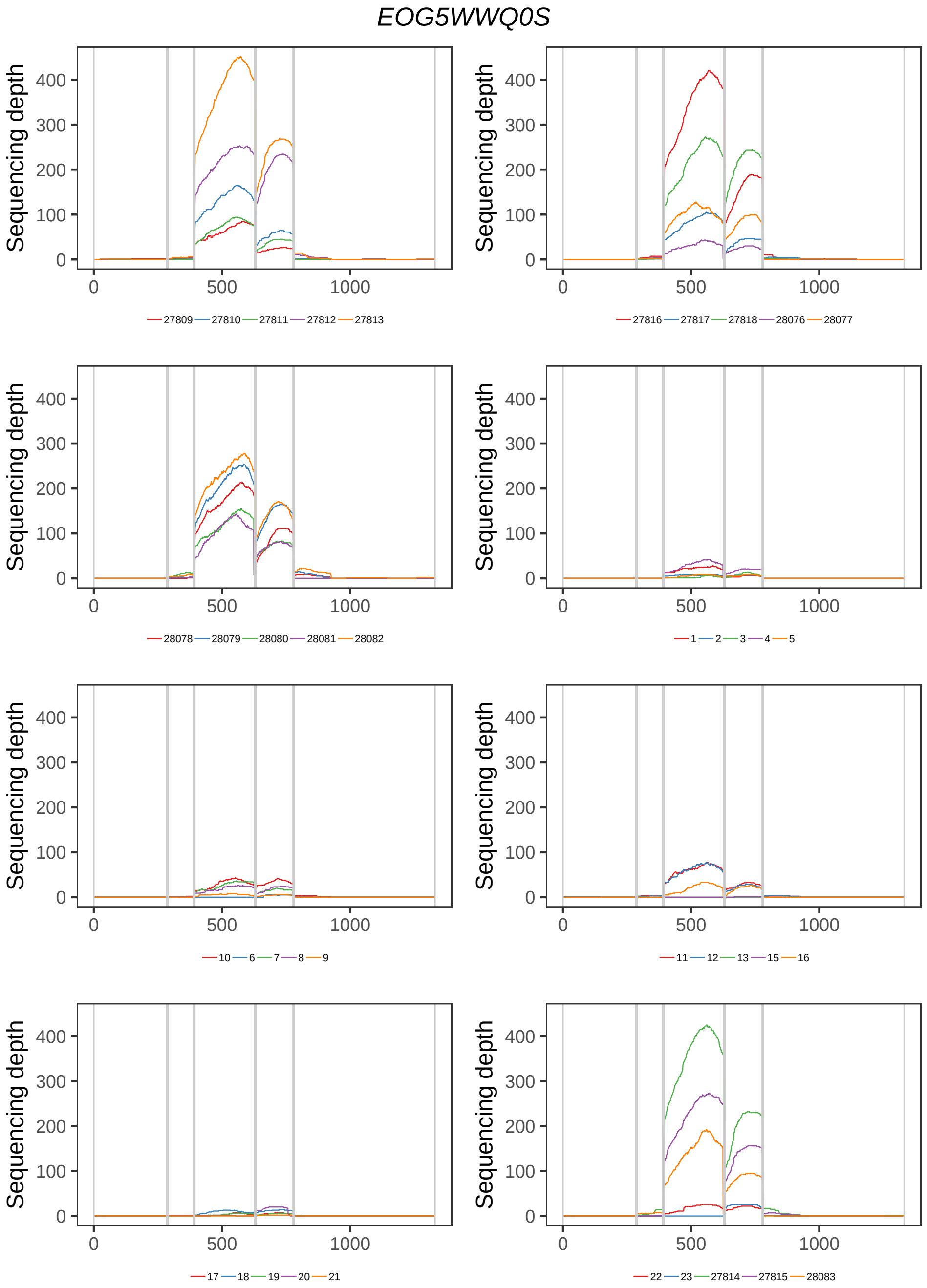
EOG5WWQ0S
Sequencing depth
0
100
200
300
400
0
500
1000
27809
27810
27811
27812
27813
27816
27817
27818
28076
28077
28078
28079
28080
28081
28082
1
2
3
4
5
10
6
7
8
9
11
12
13
15
16
17
18
19
20
21
22
23
27814
27815
28083

EOG559ZWD

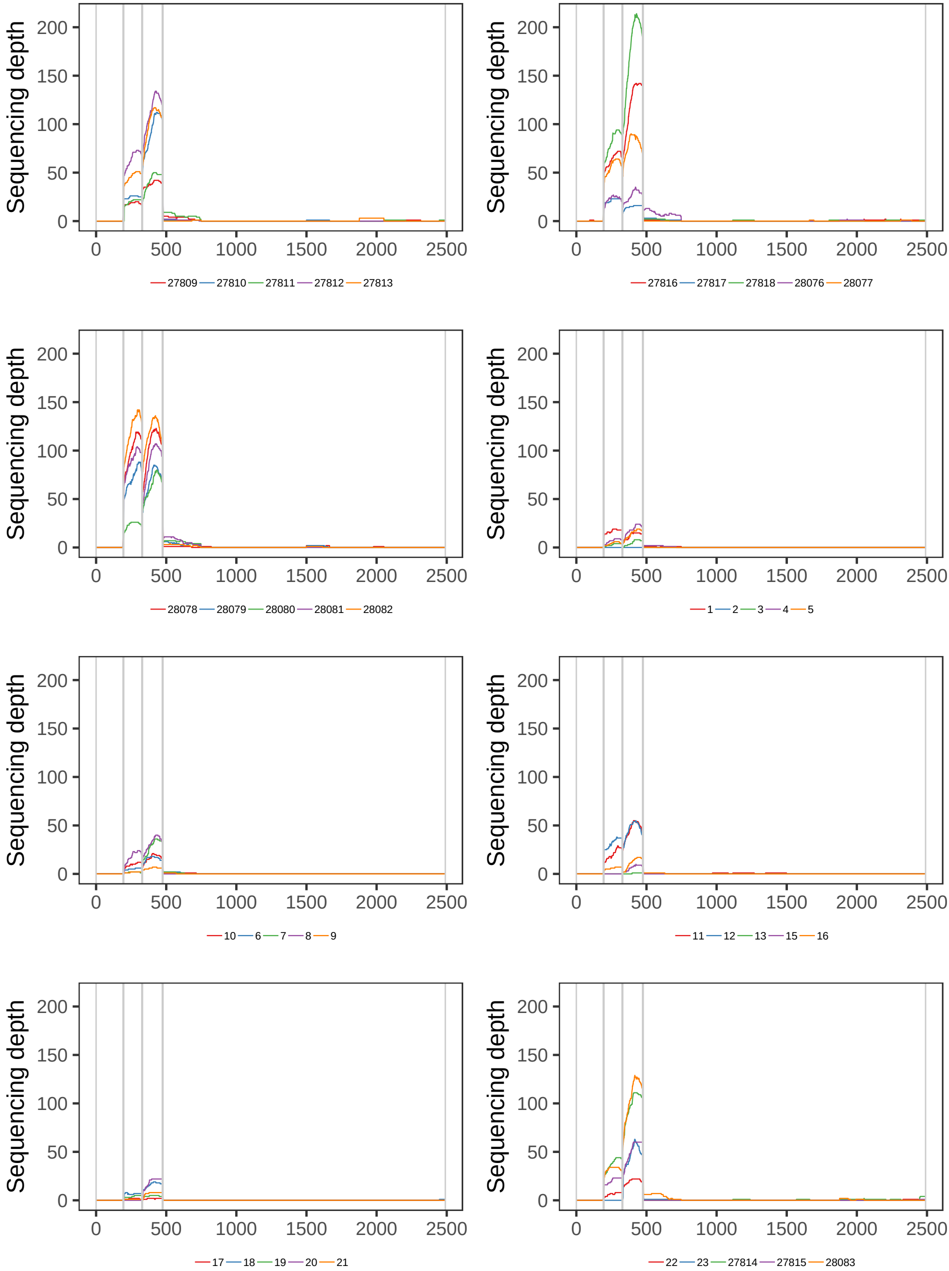
Sequencing depth
0
50
100
150
200
0
500
1000
1500
2000
2500
27809 27810 27811 27812 27813
27816 27817 27818 28076 28077
28078 28079 28080 28081 28082
1 2 3 4 5
10 6 7 8 9
11 12 13 15 16
17 18 19 20 21
22 23 27814 27815 28083

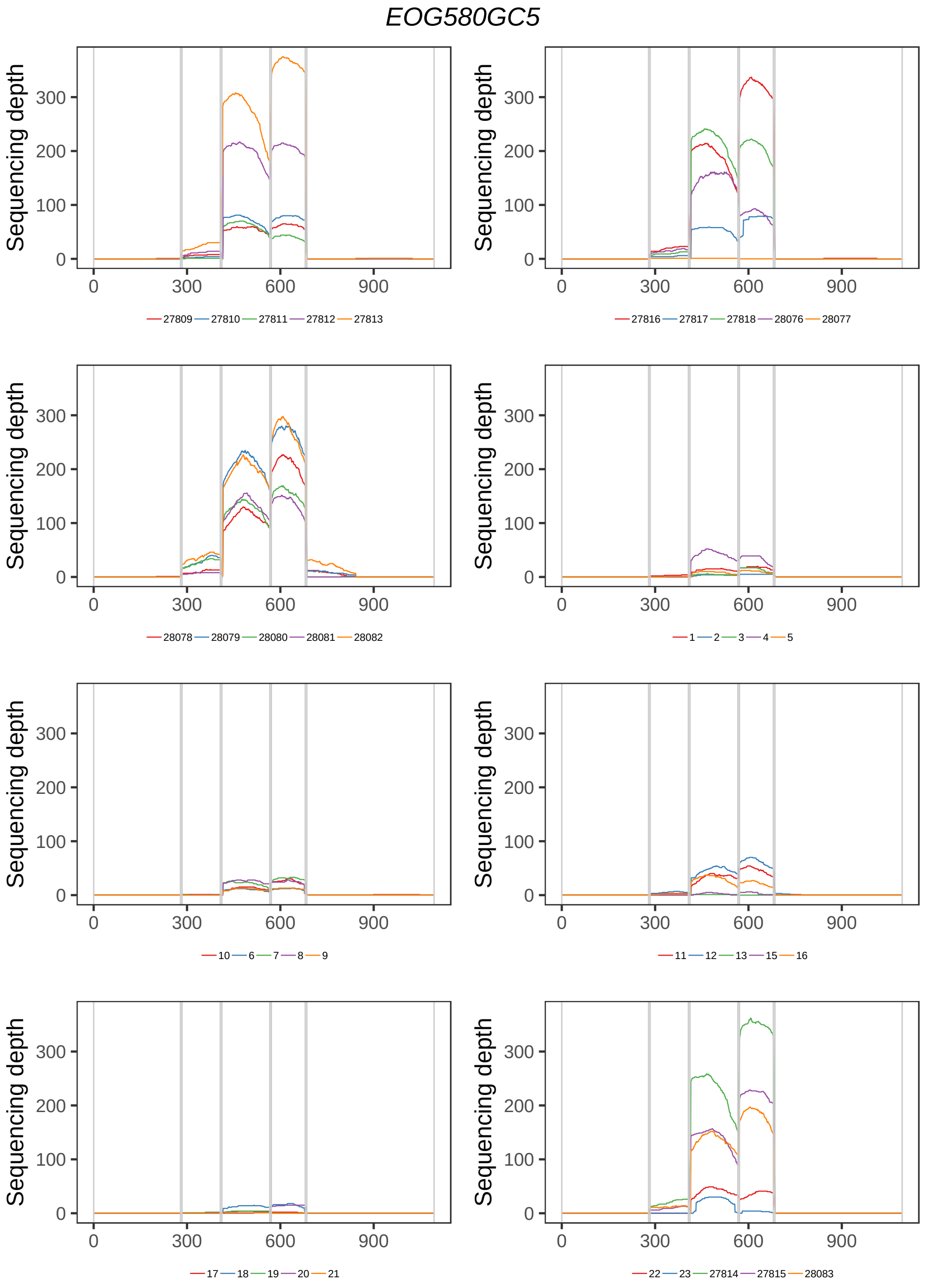
EOG580GC5
Sequencing depth
0
100
200
300
0
300
600
900
27809 27810 27811 27812 27813
27816 27817 27818 28076 28077
28078 28079 28080 28081 28082
1 2 3 4 5
10 6 7 8 9
11 12 13 15 16
17 18 19 20 21
22 23 27814 27815 28083

EOG5C5B0W

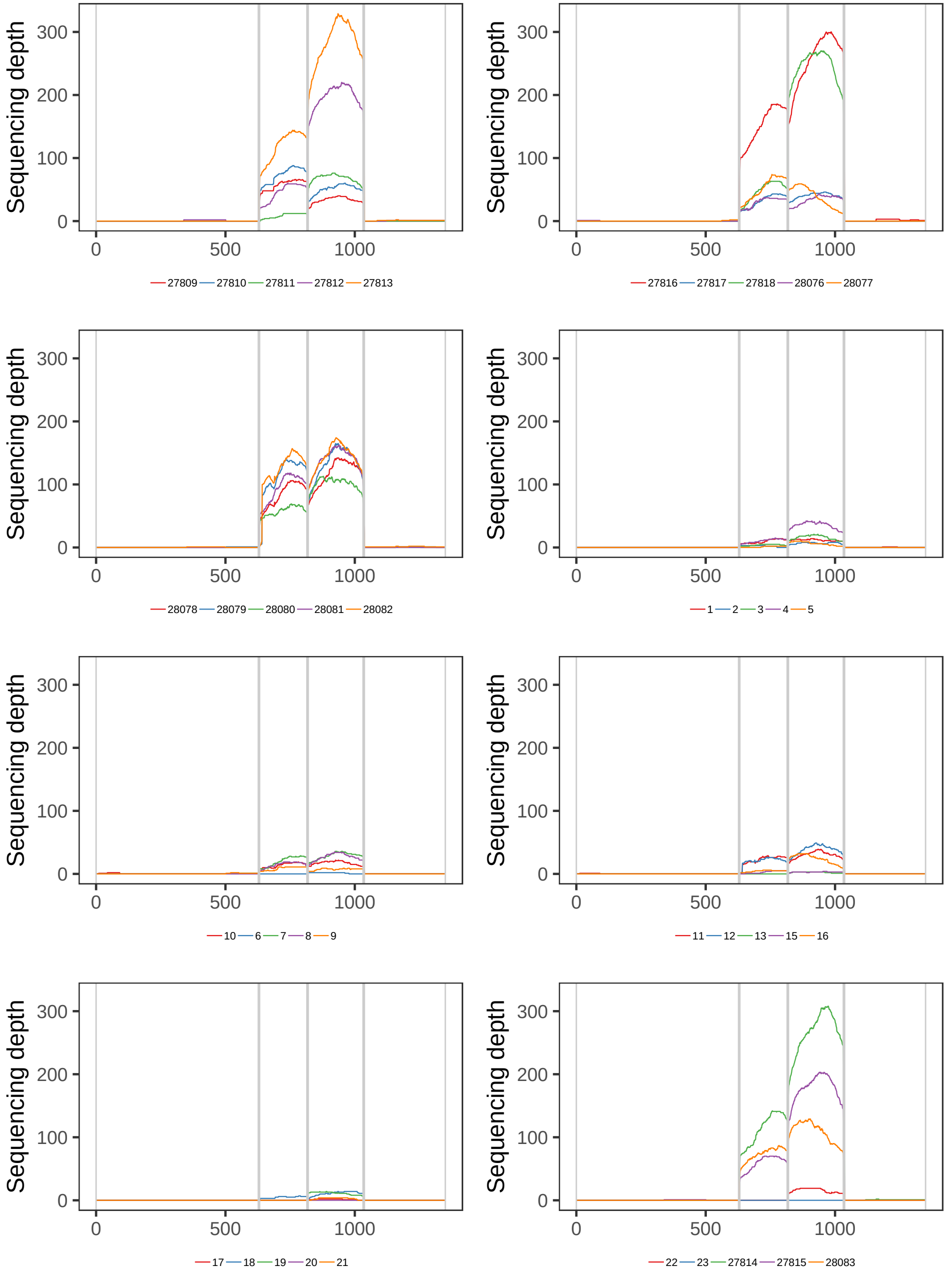
Sequencing depth
300
200
100
0
0
500
1000
27809 27810 27811 27812 27813
27816 27817 27818 28076 28077
28078 28079 28080 28081 28082
1 2 3 4 5
10 6 7 8 9
11 12 13 15 16
17 18 19 20 21
22 23 27814 27815 28083

EOG5FXPQR

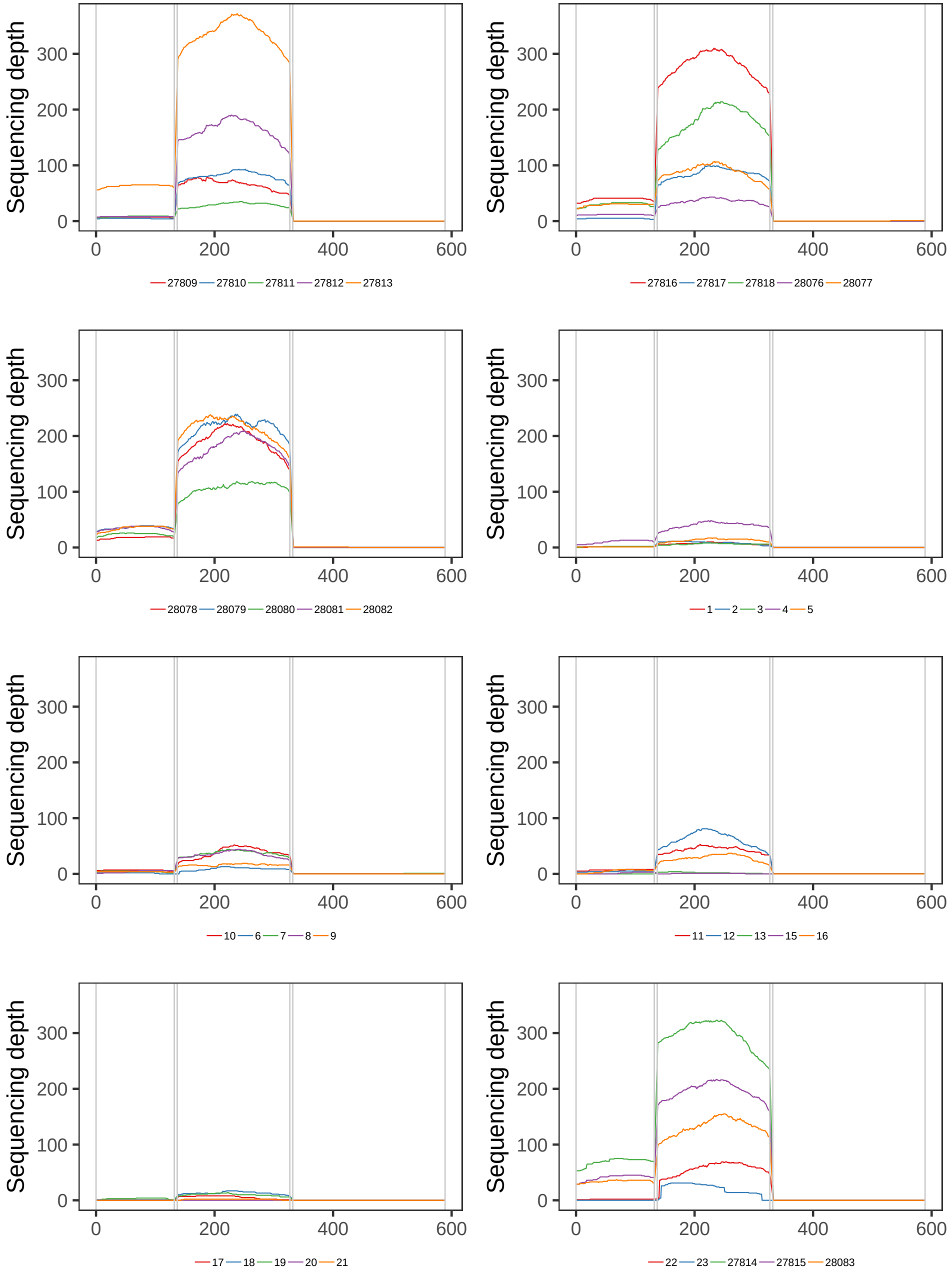

Sequencing depth
0
100
200
300
0
200
400
600
27809 27810 27811 27812 27813
27816 27817 27818 28076 28077
28078 28079 28080 28081 28082
1 2 3 4 5
10 6 7 8 9
11 12 13 15 16
17 18 19 20 21
22 23 27814 27815 28083

EOG5H44M5

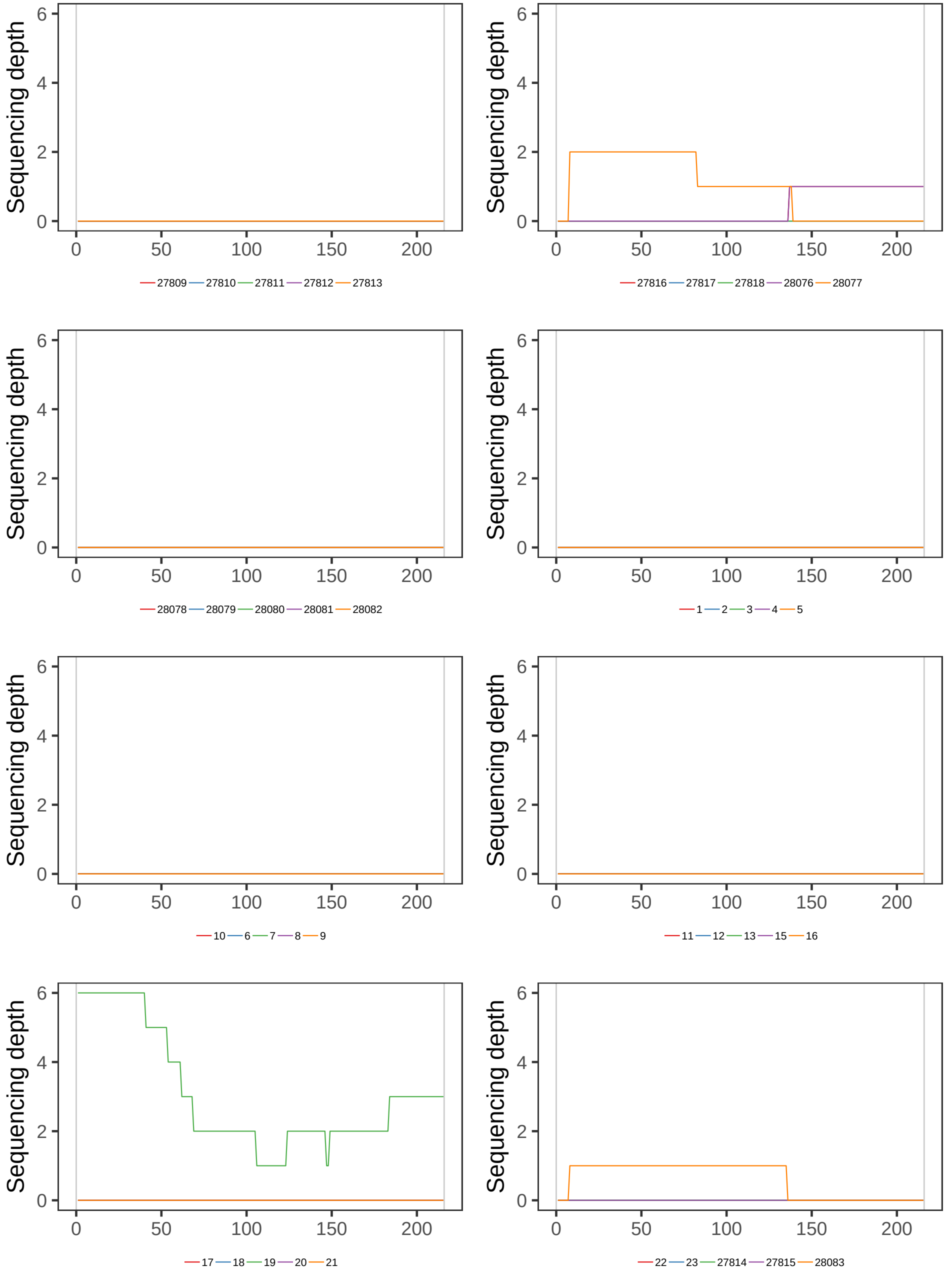
Sequencing depth
27809 27810 27811 27812 27813
27816 27817 27818 28076 28077
28078 28079 28080 28081 28082
1 2 3 4 5
10 6 7 8 9
11 12 13 15 16
17 18 19 20 21
22 23 27814 27815 28083

EOG5N8PMF

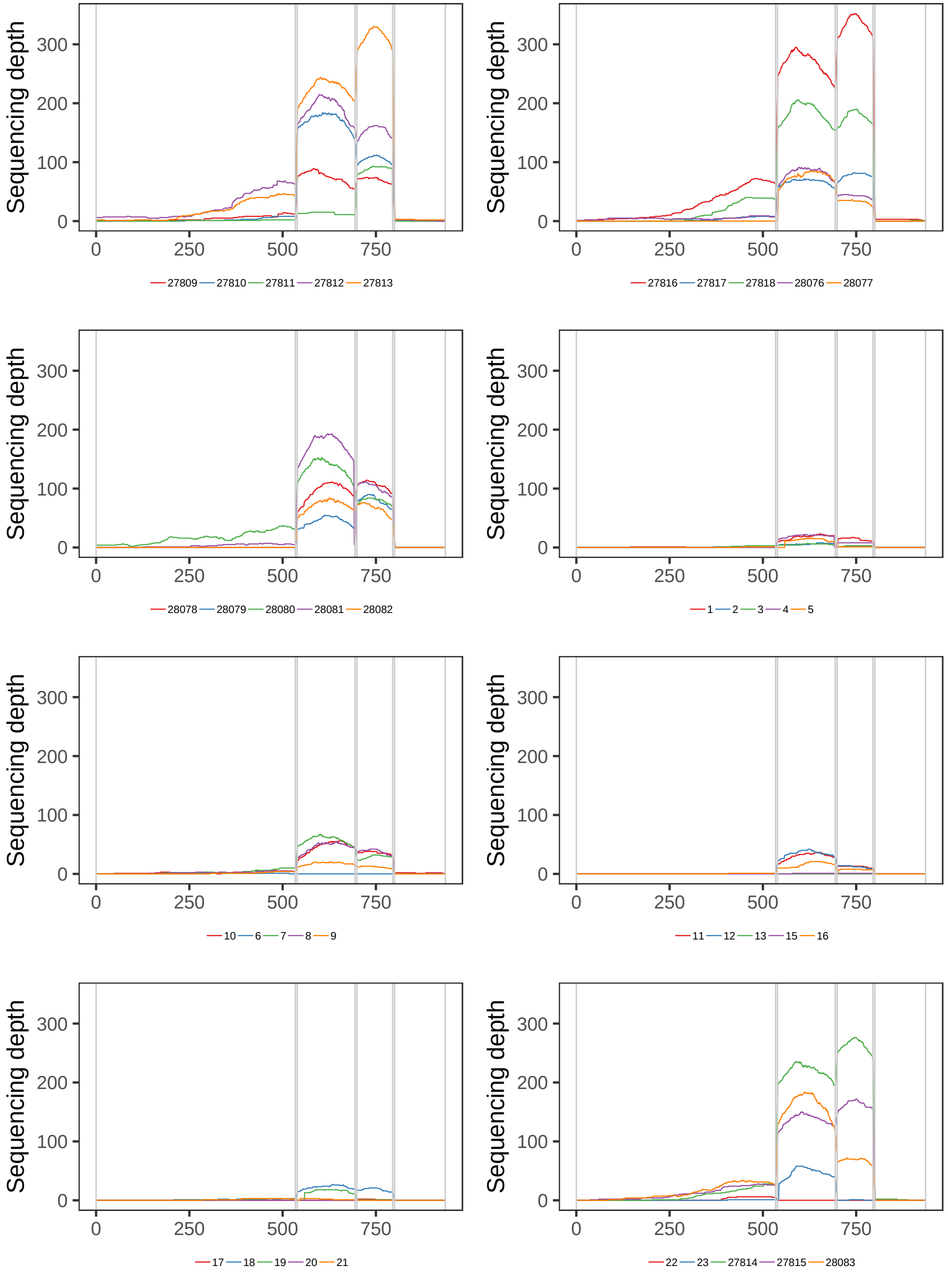
Sequencing depth
0
100
200
300
0
250
500
750
27809 27810 27811 27812 27813
27816 27817 27818 28076 28077
28078 28079 28080 28081 28082
1 2 3 4 5
10 6 7 8 9
11 12 13 15 16
17 18 19 20 21
22 23 27814 27815 28083

EOG515DWD

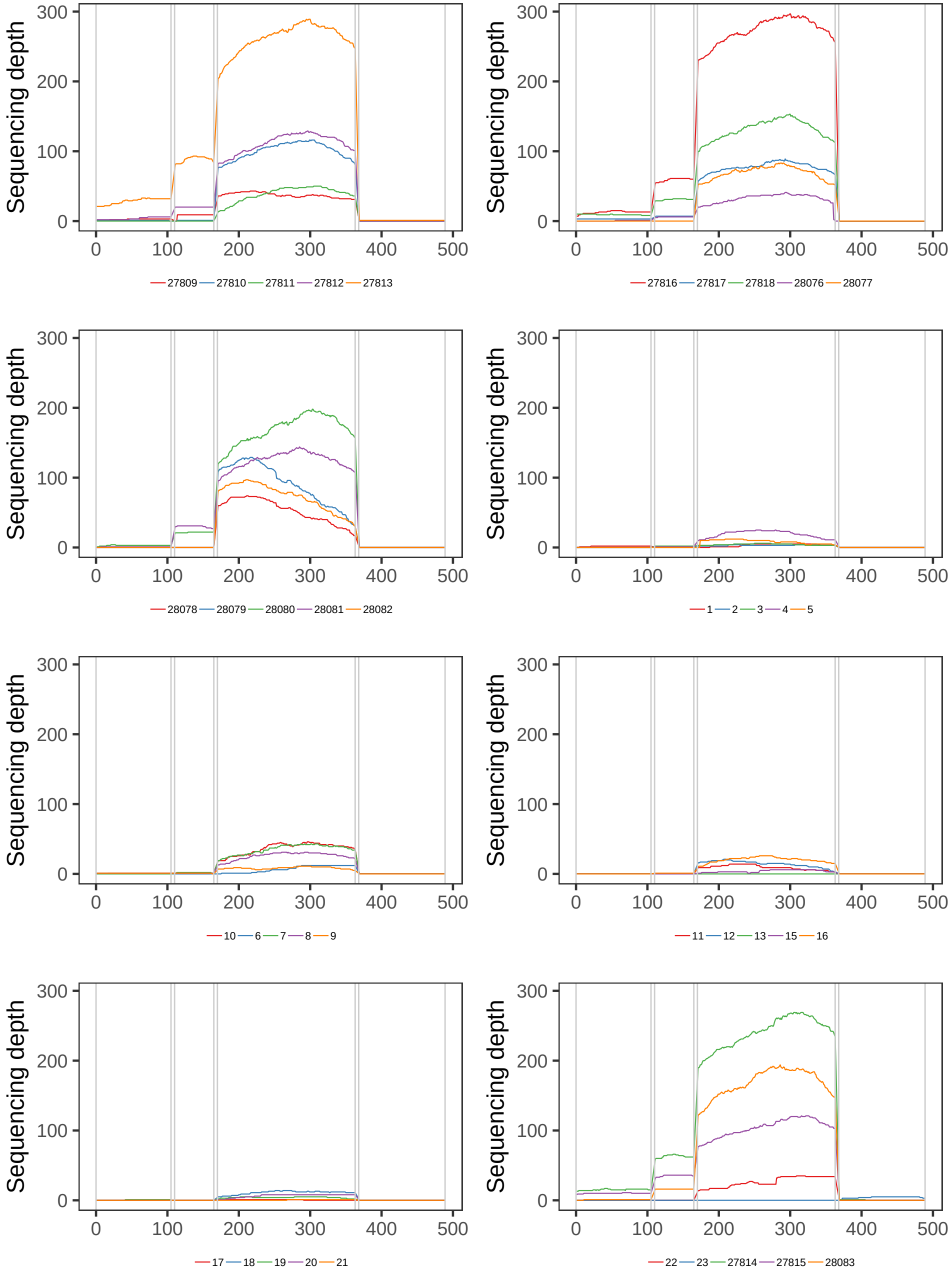
Sequencing depth
0
100
200
300
400
500
27809 27810 27811 27812 27813
27816 27817 27818 28076 28077
28078 28079 28080 28081 28082
1 2 3 4 5
10 6 7 8 9
11 12 13 15 16
17 18 19 20 21
22 23 27814 27815 28083

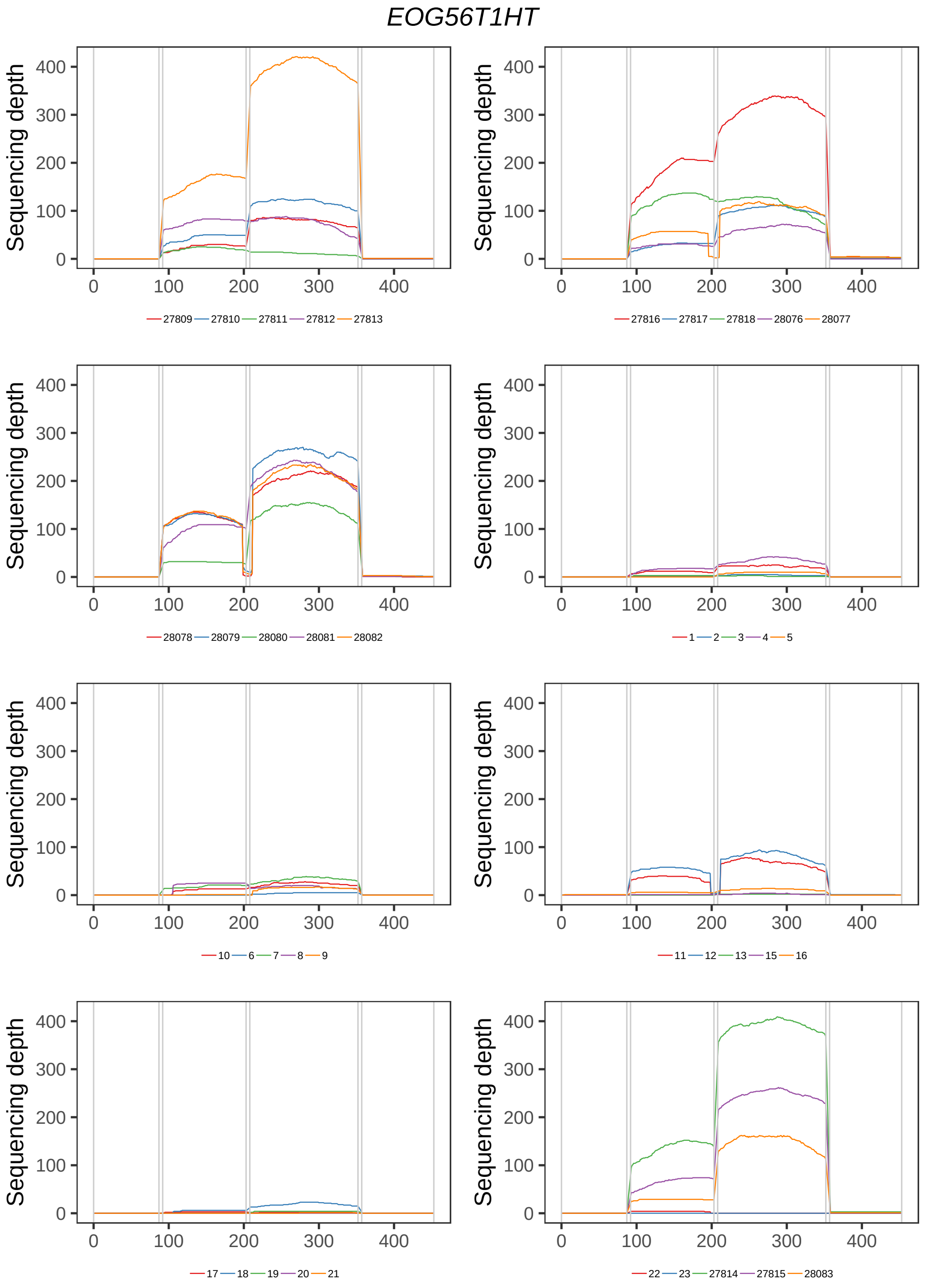
EOG56T1HT
Sequencing depth
0
100
200
300
400
27809 27810 27811 27812 27813
27816 27817 27818 28076 28077
28078 28079 28080 28081 28082
1 2 3 4 5
10 6 7 8 9
11 12 13 15 16
17 18 19 20 21
22 23 27814 27815 28083

EOG5HHMHJ

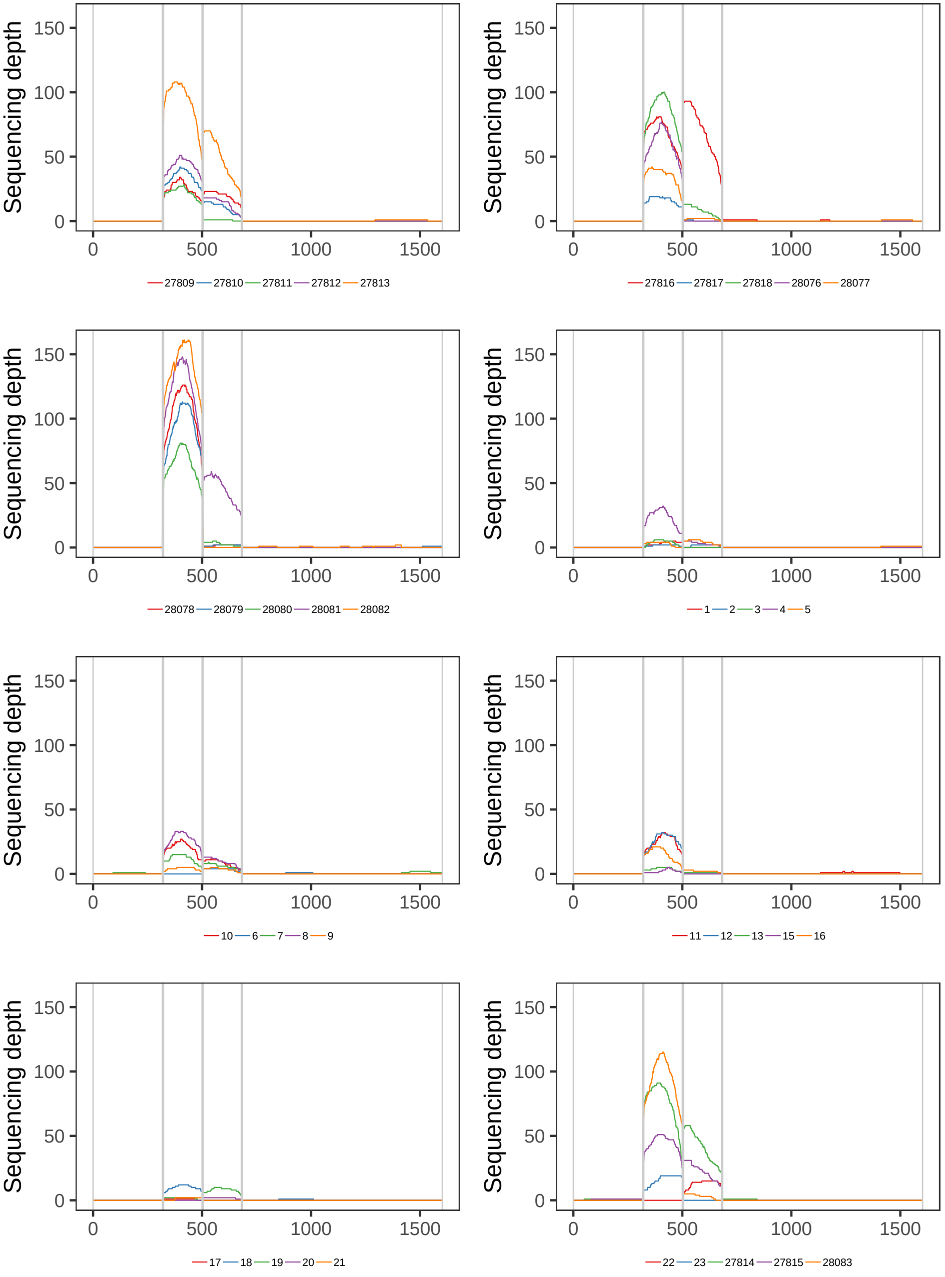
Sequencing depth
0
50
100
150
0
500
1000
1500
27809 27810 27811 27812 27813
27816 27817 27818 28076 28077
28078 28079 28080 28081 28082
1 2 3 4 5
10 6 7 8 9
11 12 13 15 16
17 18 19 20 21
22 23 27814 27815 28083

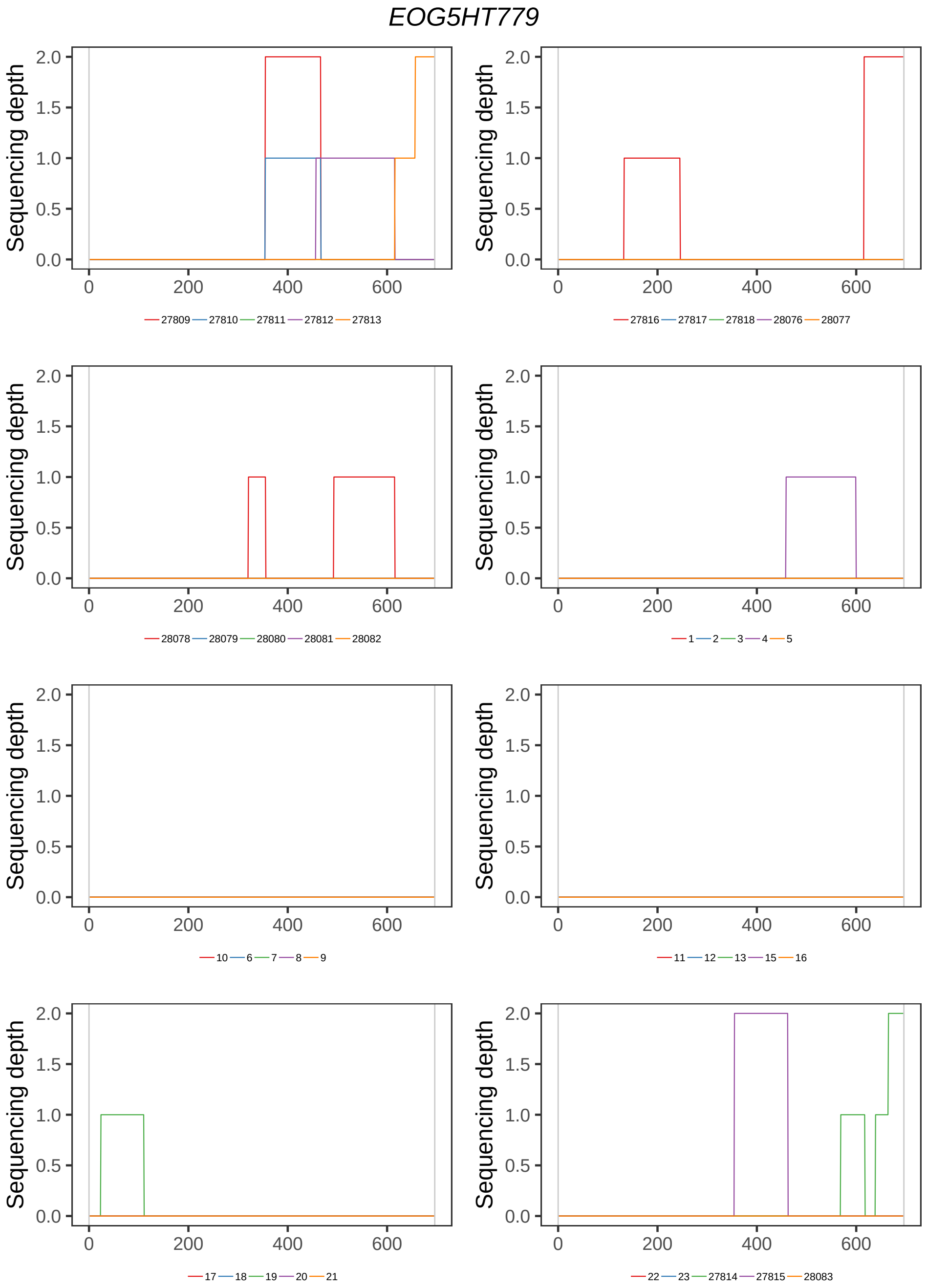

EOG5HT779
Sequencing depth
2.0
1.5
1.0
0.5
0.0
0
200
400
600
27809
27810
27811
27812
27813
27816
27817
27818
28076
28077
28078
28079
28080
28081
28082
1
2
3
4
5
10
6
7
8
9
11
12
13
15
16
17
18
19
20
21
22
23
27814
27815
28083

EOG5JDFQ8

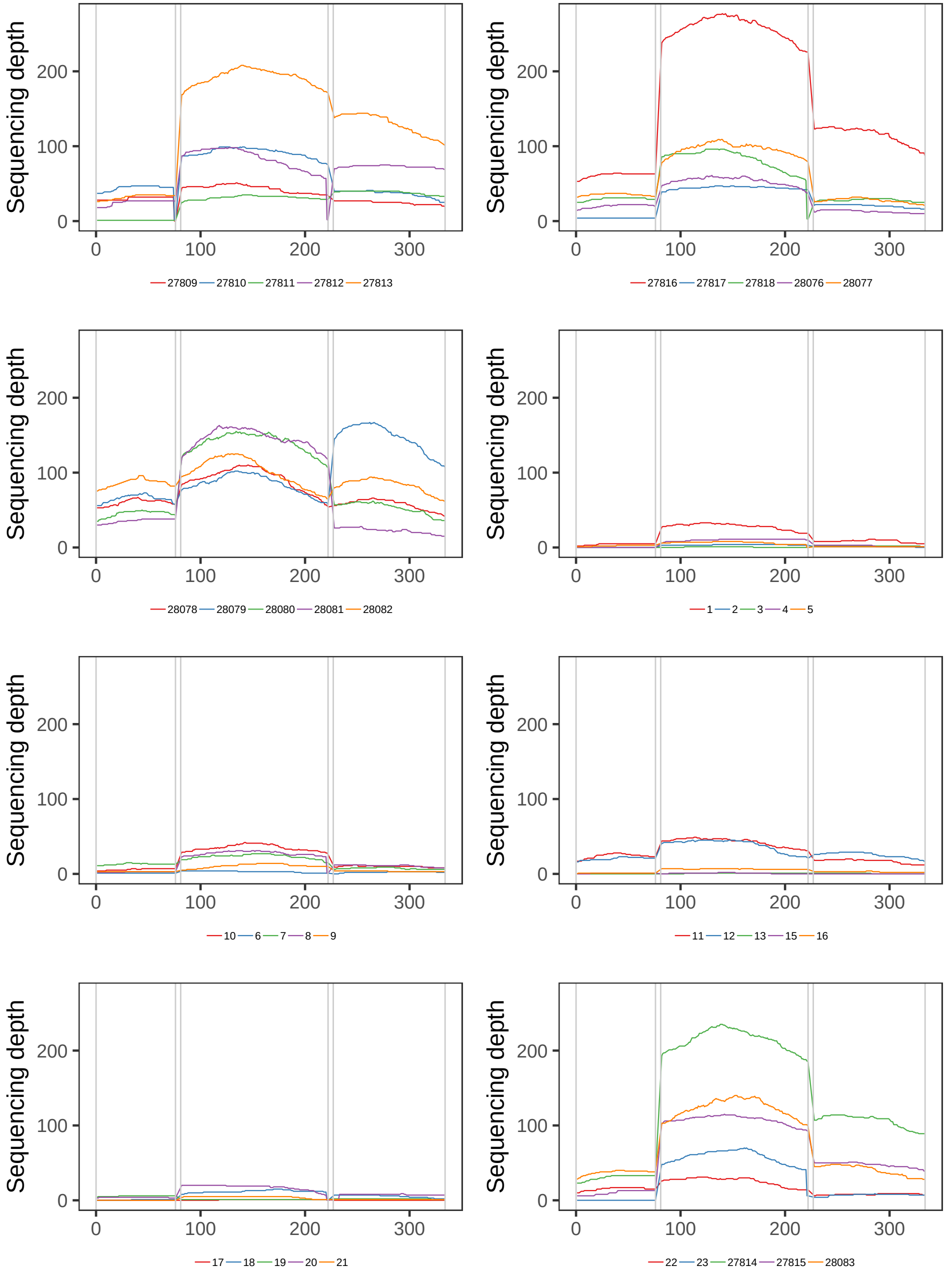
Sequencing depth
0
100
200
300
27809 27810 27811 27812 27813
27816 27817 27818 28076 28077
28078 28079 28080 28081 28082
1 2 3 4 5
10 6 7 8 9
11 12 13 15 16
17 18 19 20 21
22 23 27814 27815 28083

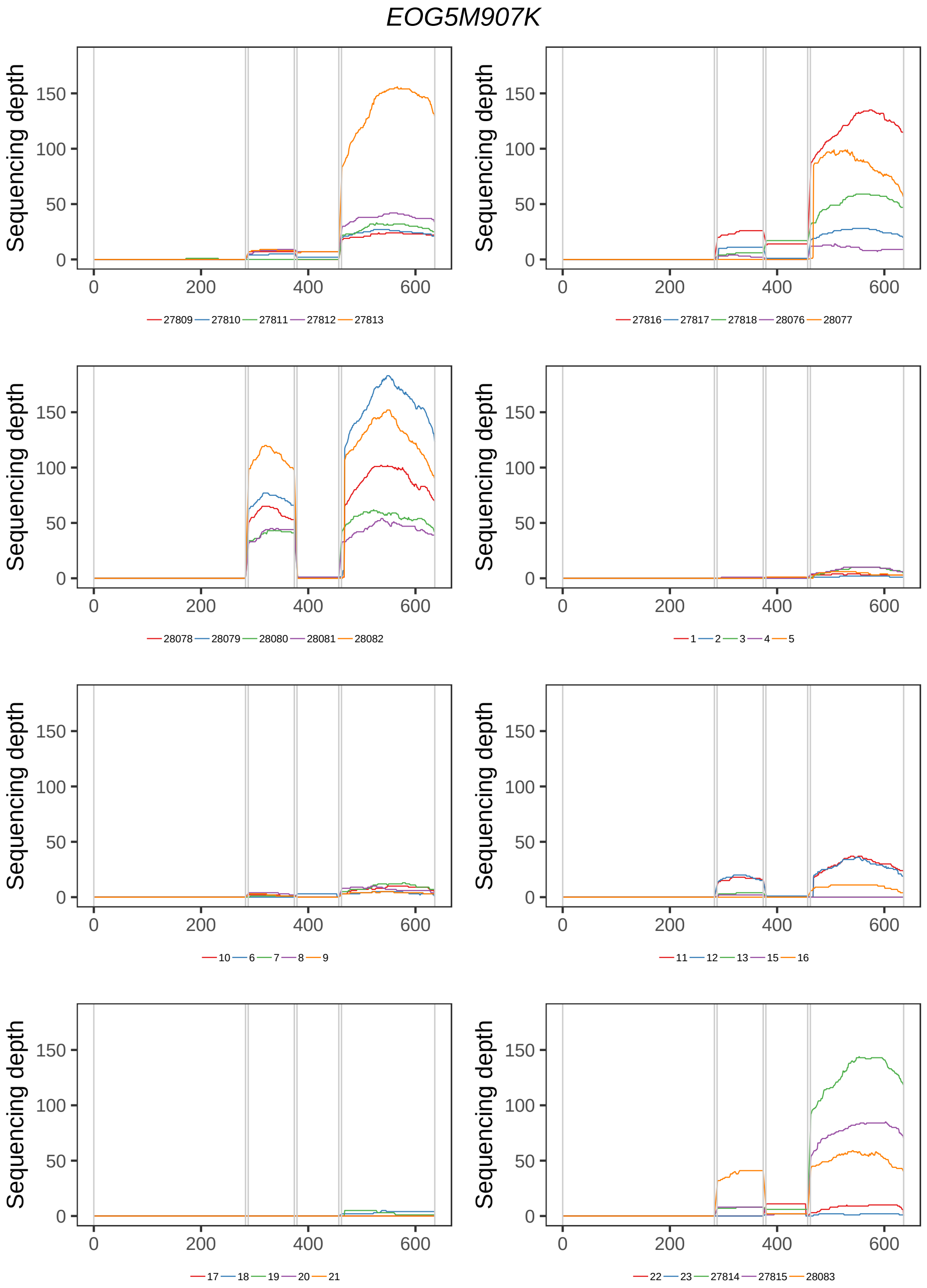
EOG5M907K
Sequencing depth
0
50
100
150
0
200
400
600
27809
27810
27811
27812
27813
27816
27817
27818
28076
28077
28078
28079
28080
28081
28082
1
2
3
4
5
10
6
7
8
9
11
12
13
15
16
17
18
19
20
21
22
23
27814
27815
28083

EOG5PZGNT

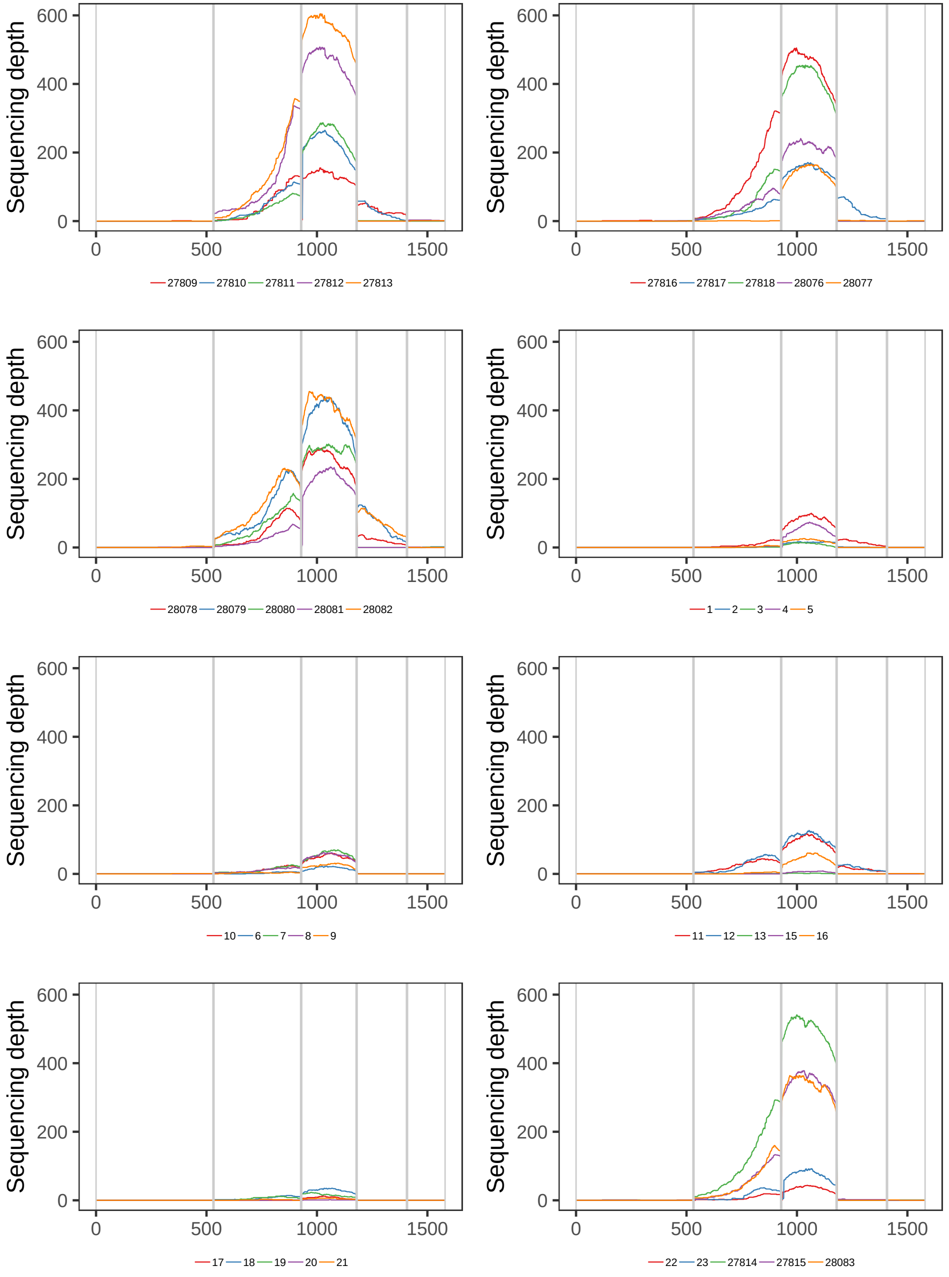
Sequencing depth
0
200
400
600
0
500
1000
1500
27809
27810
27811
27812
27813
27816
27817
27818
28076
28077
28078
28079
28080
28081
28082
1
2
3
4
5
10
6
7
8
9
11
12
13
15
16
17
18
19
20
21
22
23
27814
27815
28083

EOG5R229F

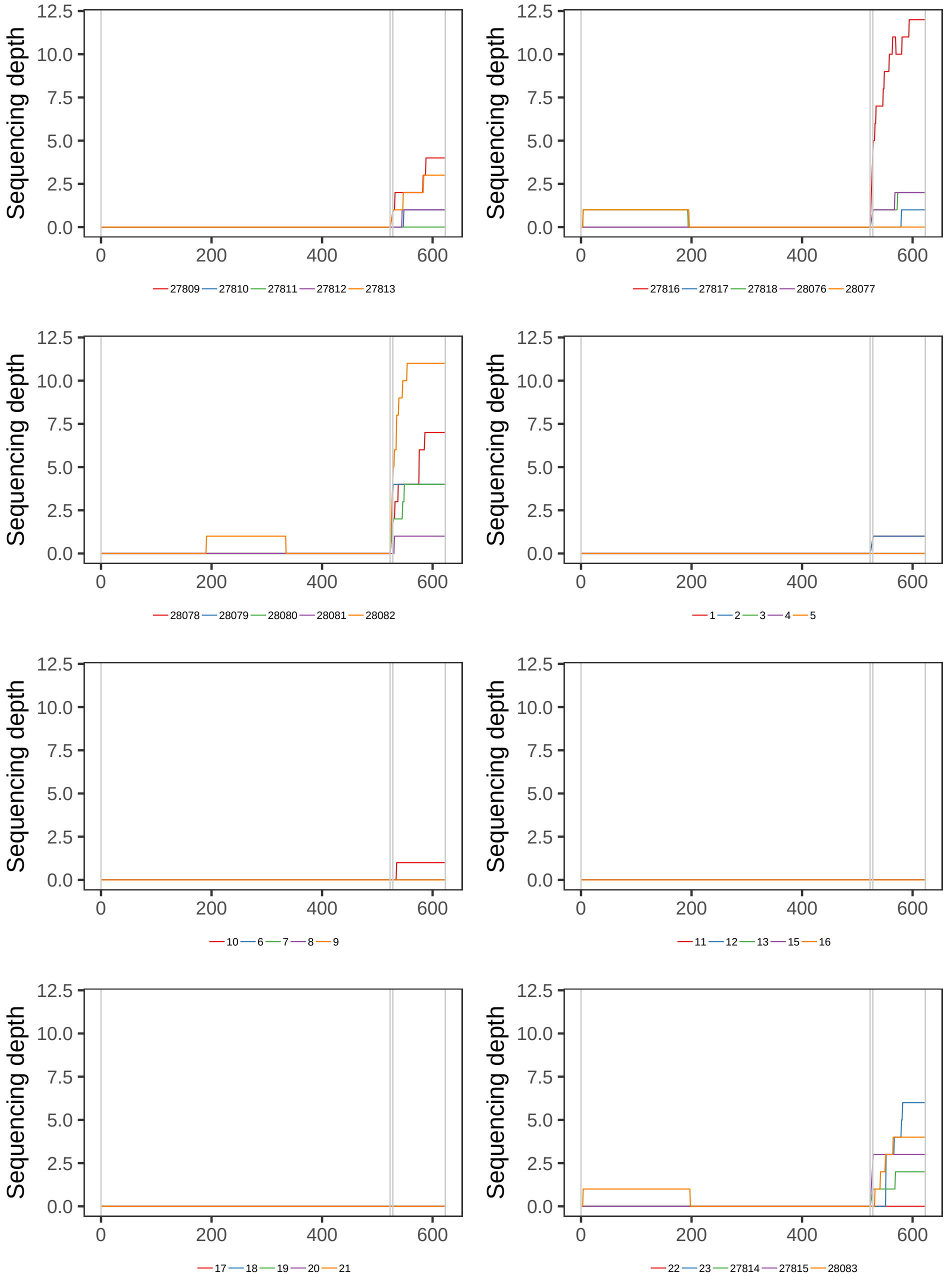

Sequencing depth
12.5
10.0
7.5
5.0
2.5
0.0
0
200
400
600
27809 27810 27811 27812 27813
27816 27817 27818 28076 28077
28078 28079 28080 28081 28082
1 2 3 4 5
10 6 7 8 9
11 12 13 15 16
17 18 19 20 21
22 23 27814 27815 28083

EOG5VDNF9

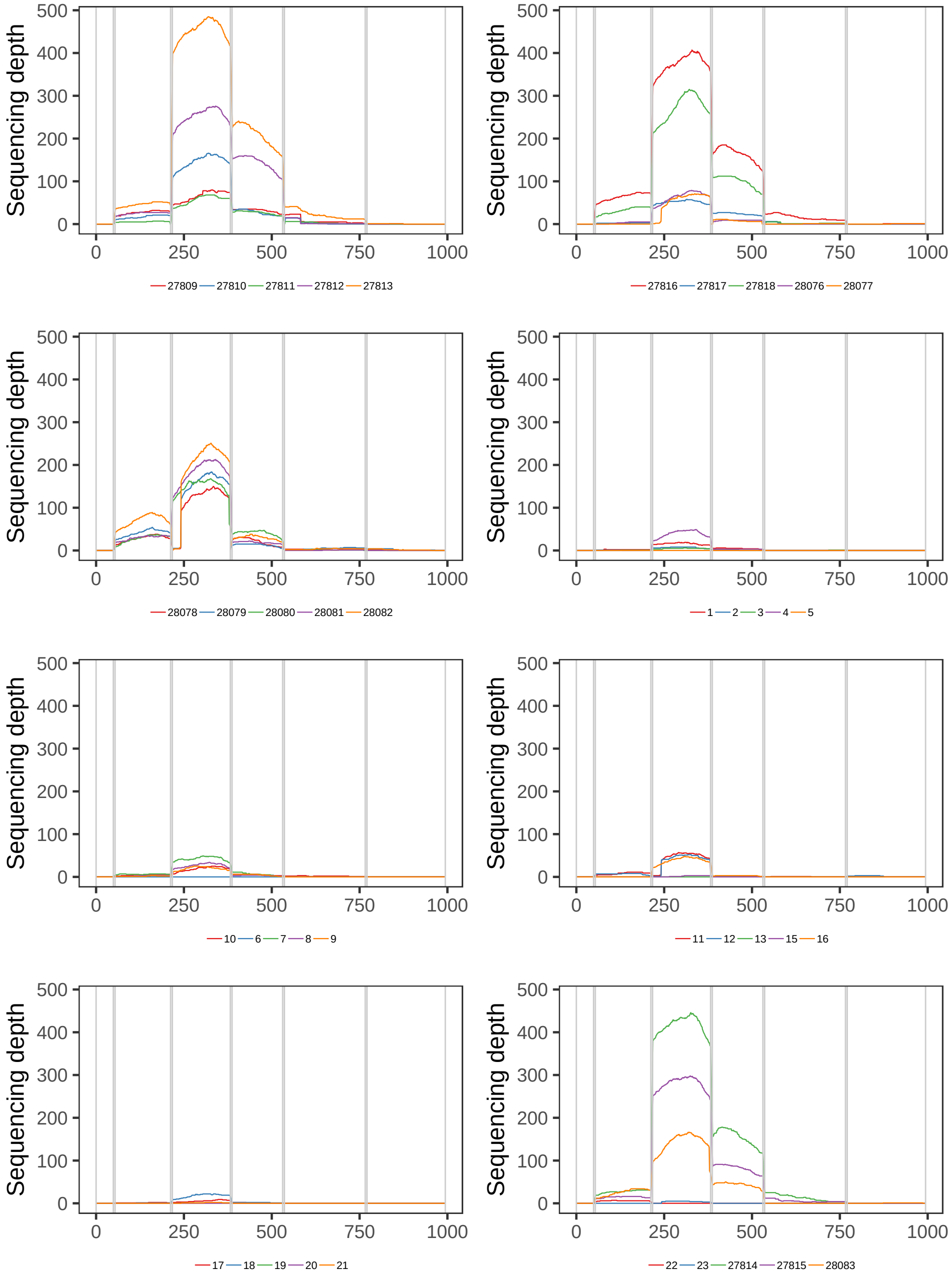
Sequencing depth
0
100
200
300
400
500
0
250
500
750
1000
27809 27810 27811 27812 27813
27816 27817 27818 28076 28077
28078 28079 28080 28081 28082
1 2 3 4 5
10 6 7 8 9
11 12 13 15 16
17 18 19 20 21
22 23 27814 27815 28083

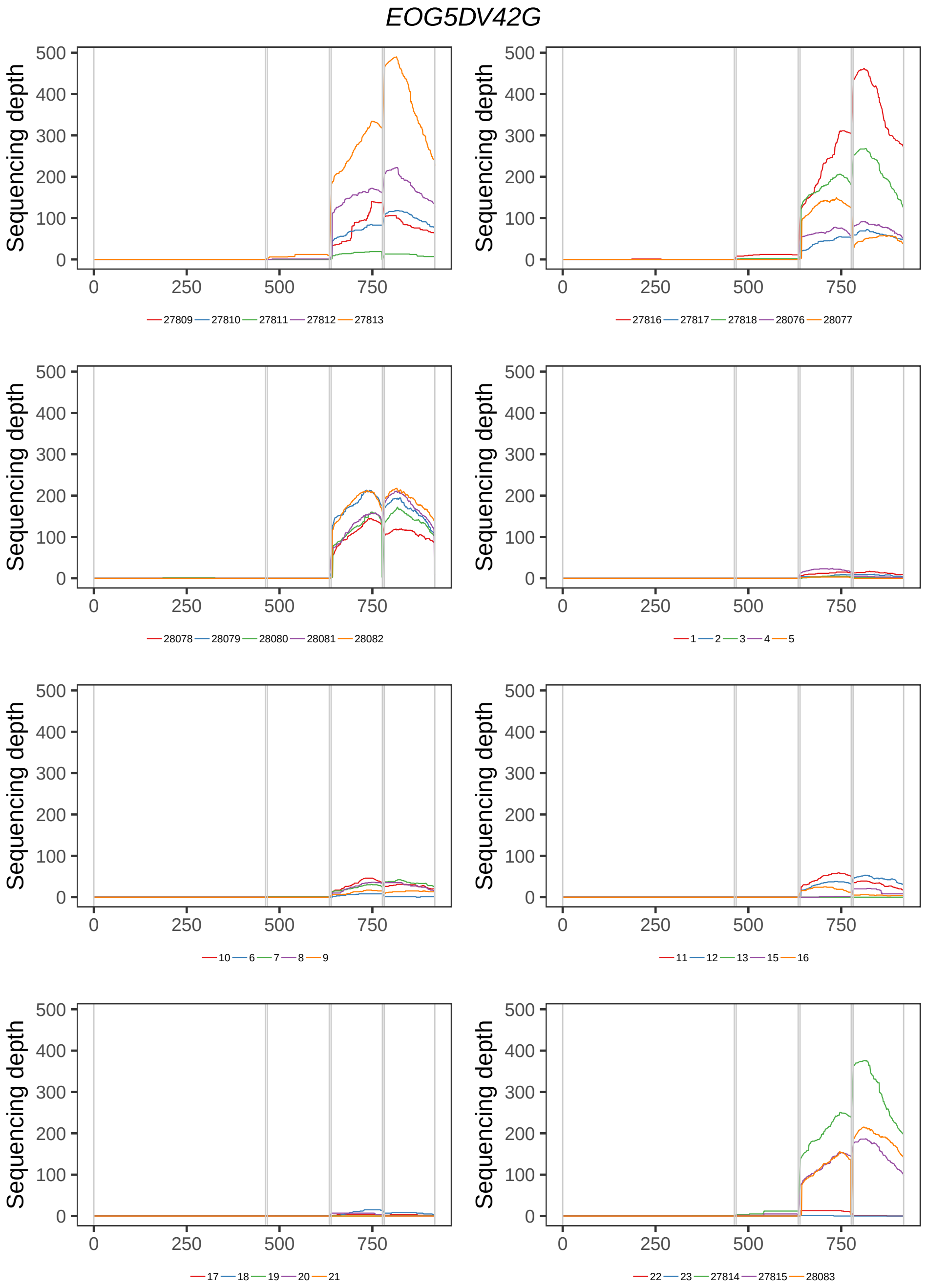
EOG5DV42G
Sequencing depth
27809 27810 27811 27812 27813
27816 27817 27818 28076 28077
28078 28079 28080 28081 28082
1 2 3 4 5
10 6 7 8 9
11 12 13 15 16
17 18 19 20 21
22 23 27814 27815 28083

EOG5S1RP7

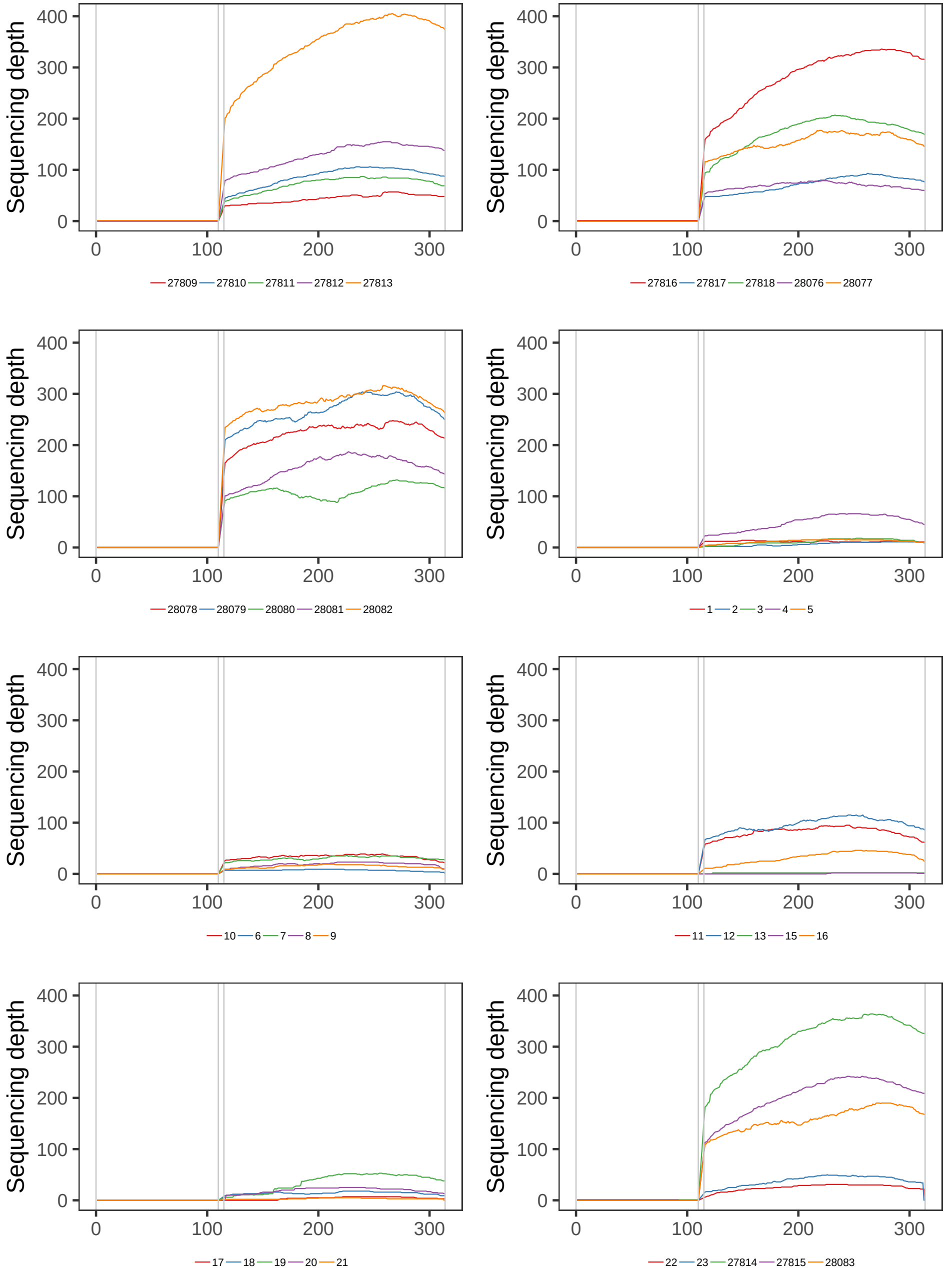

Sequencing depth
0
100
200
300
400
27809
27810
27811
27812
27813
27816
27817
27818
28076
28077
28078
28079
28080
28081
28082
1
2
3
4
5
10
6
7
8
9
11
12
13
15
16
17
18
19
20
21
22
23
27814
27815
28083

EOG5SBCF1

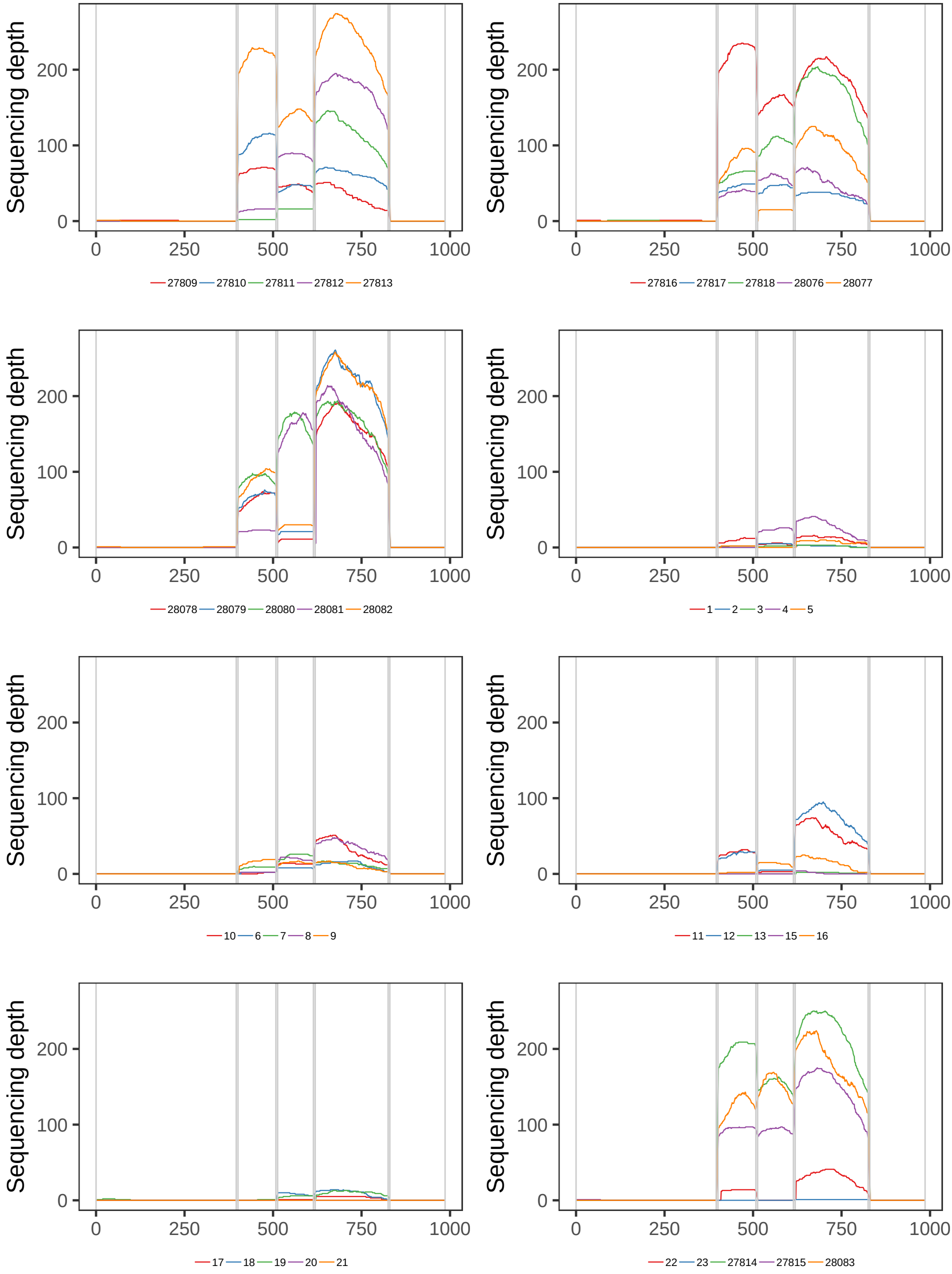
Sequencing depth
0
100
200
0
250
500
750
1000
27809
27810
27811
27812
27813
27816
27817
27818
28076
28077
28078
28079
28080
28081
28082
1
2
3
4
5
10
6
7
8
9
11
12
13
15
16
17
18
19
20
21
22
23
27814
27815
28083

EOG5VT4CH

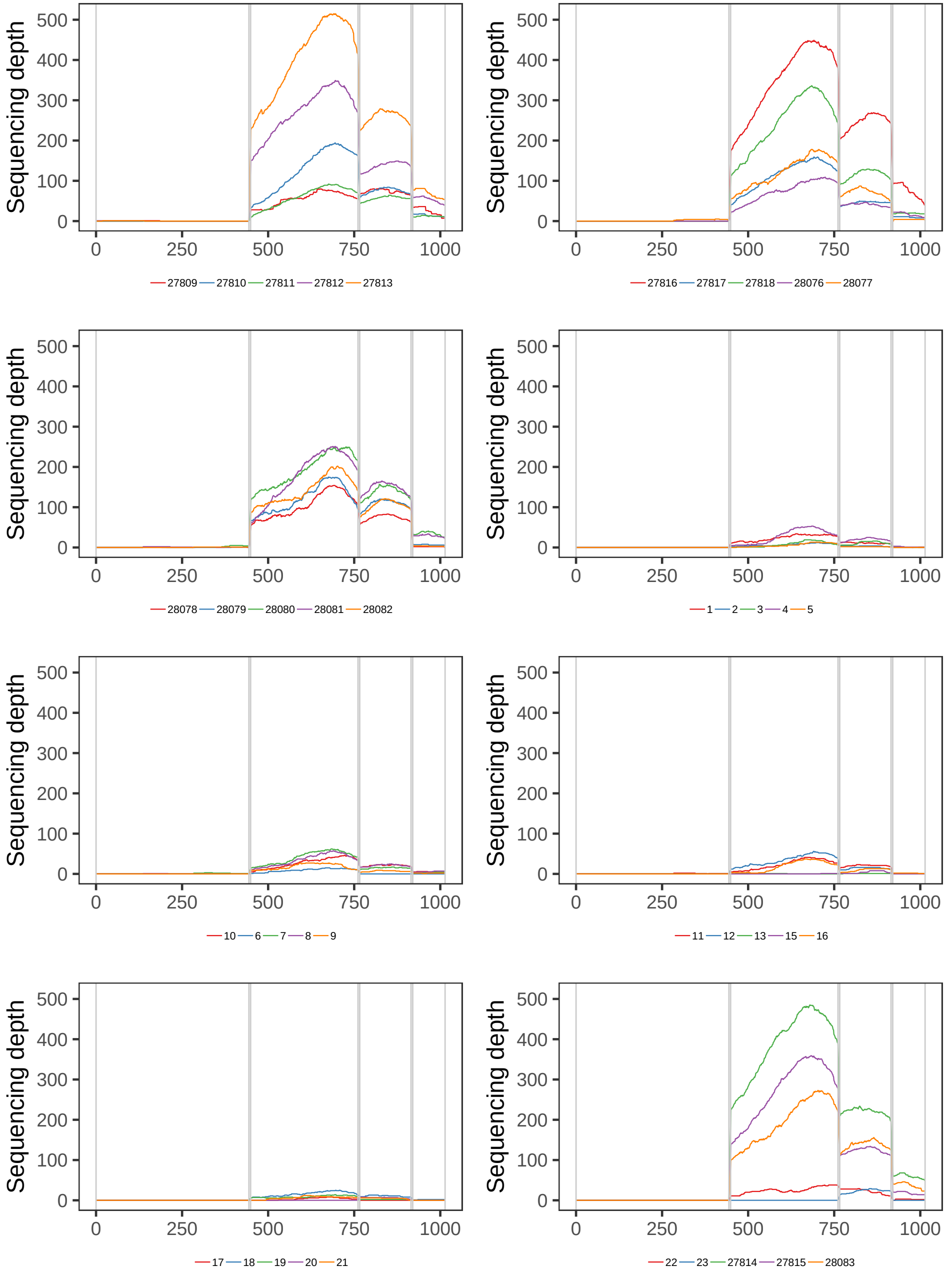
Sequencing depth
0
100
200
300
400
500
0
250
500
750
1000
27809 27810 27811 27812 27813
27816 27817 27818 28076 28077
28078 28079 28080 28081 28082
1 2 3 4 5
10 6 7 8 9
11 12 13 15 16
17 18 19 20 21
22 23 27814 27815 28083

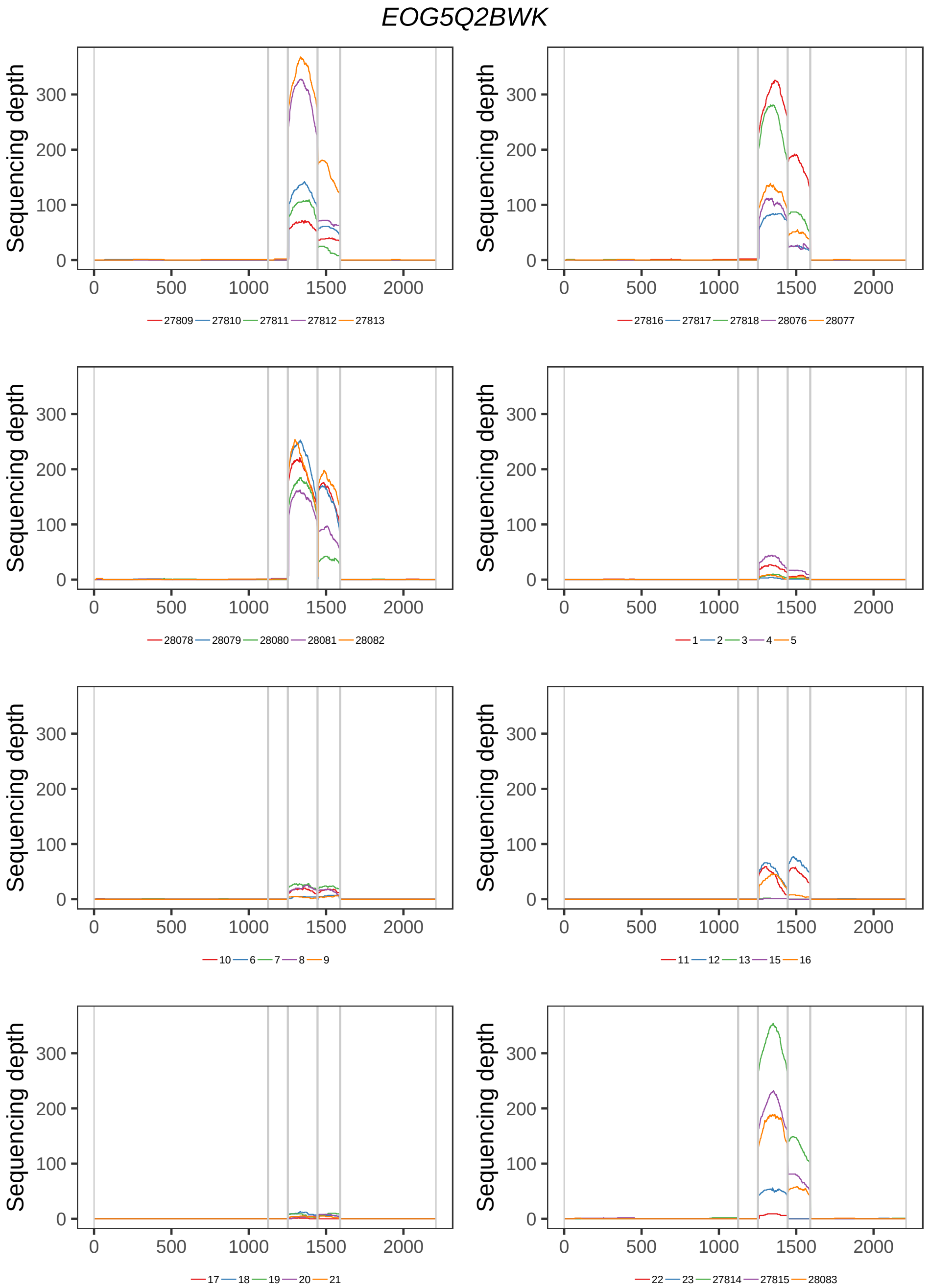
EOG5Q2BWK
Sequencing depth
0
100
200
300
0
500
1000
1500
2000
27809
27810
27811
27812
27813
27816
27817
27818
28076
28077
28078
28079
28080
28081
28082
1
2
3
4
5
10
6
7
8
9
11
12
13
15
16
17
18
19
20
21
22
23
27814
27815
28083

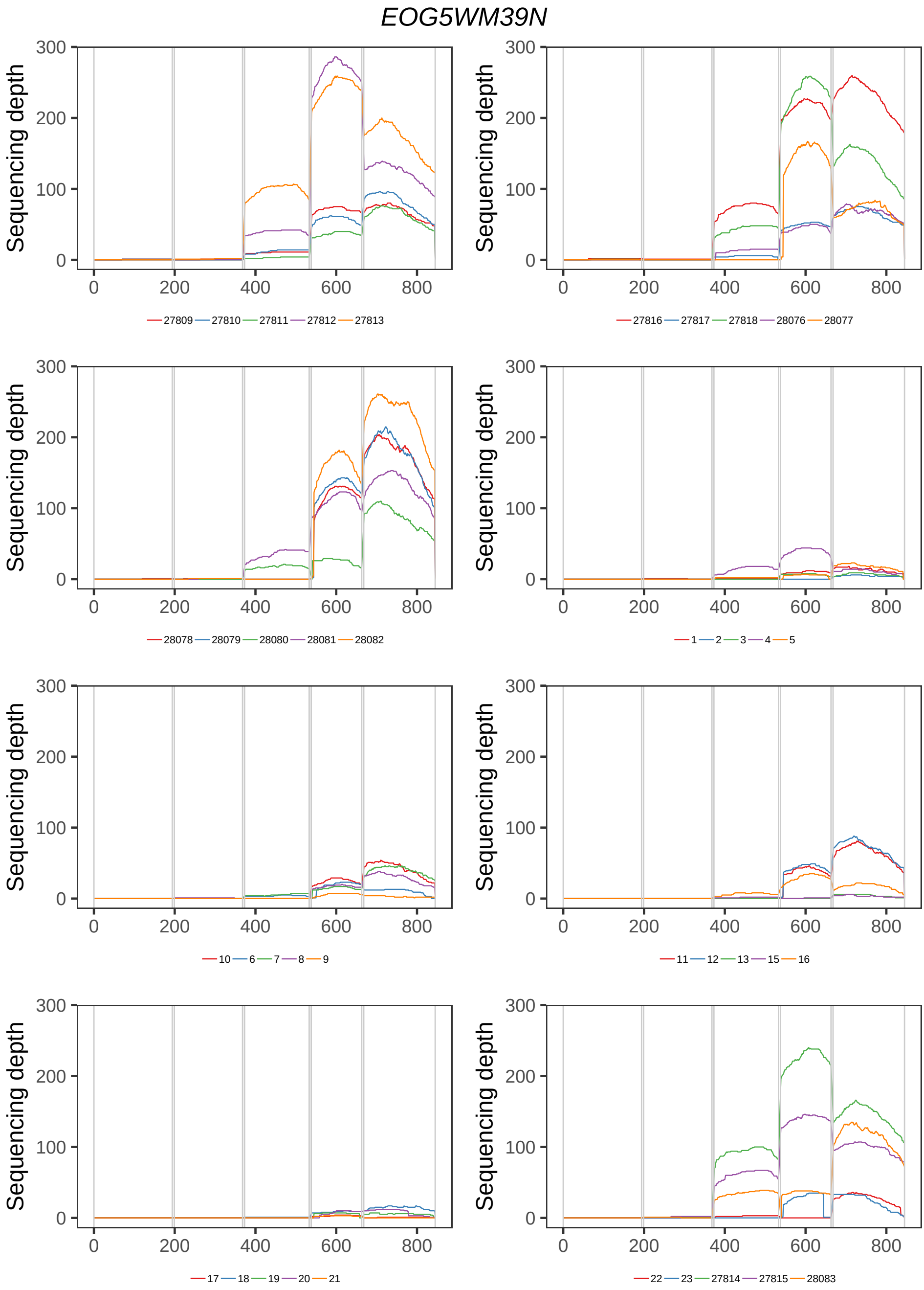
EOG5WM39N
Sequencing depth
27809
27810
27811
27812
27813
27816
27817
27818
28076
28077
28078
28079
28080
28081
28082
1
2
3
4
5
10
6
7
8
9
11
12
13
15
16
17
18
19
20
21
22
23
27814
27815
28083
