# Supplementary figures and images for: Development and evaluation of a custom bait design based on 469 single-copy protein-coding genes for exon capture of isopods (Philosciidae: Haloniscus)
Source: PLoS One. 2021 Sep 17;16(9):e0256861. doi: 10.1371/journal.pone.0256861 (PMC8448321; doi:10.1371/journal.pone.0256861)

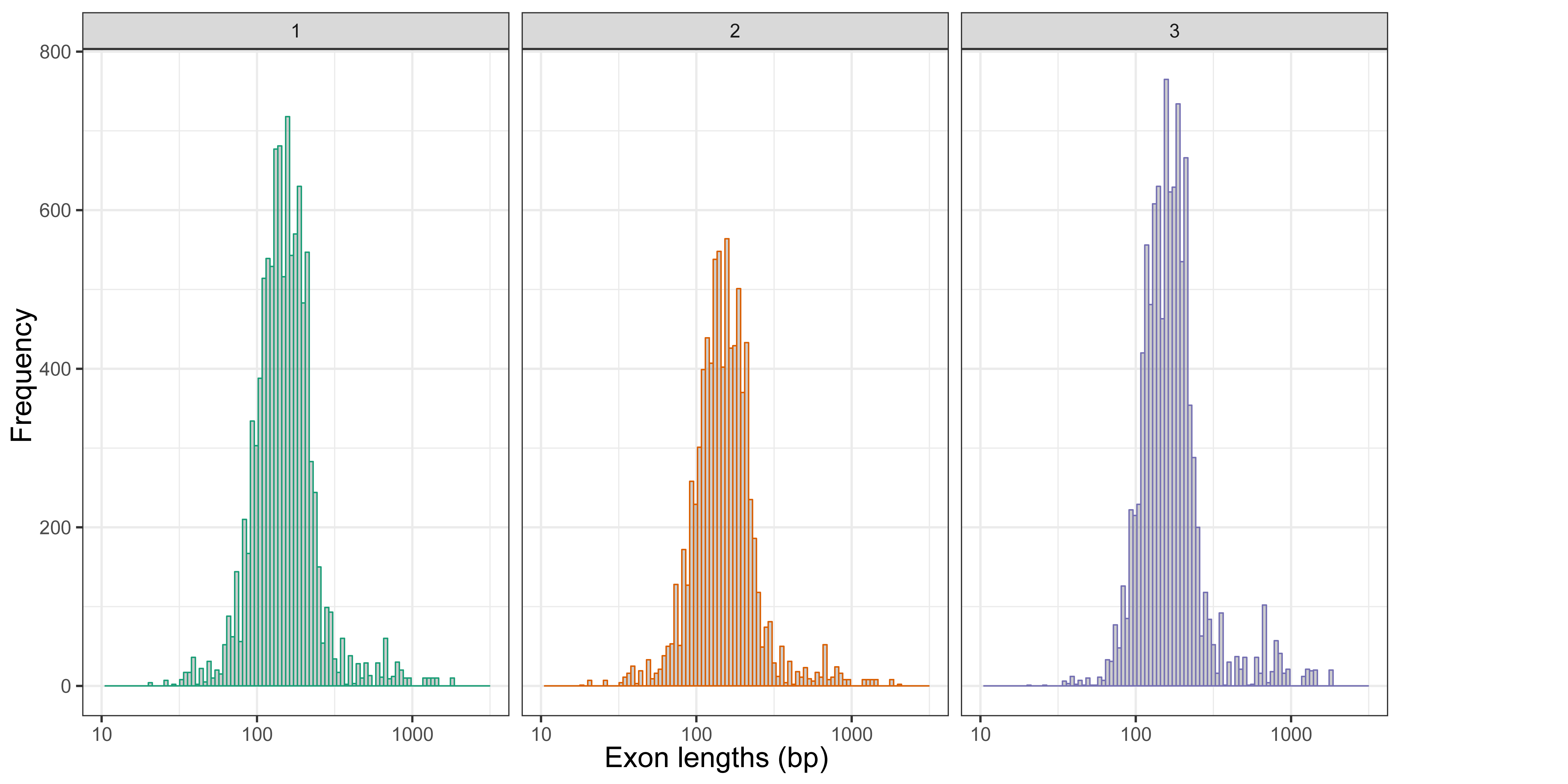

Supplement: S4 Fig — Run 1 is indicated in green, run 2 in orange, and run 3 in purple. (TIF) [file pone.0256861.s004.tif]

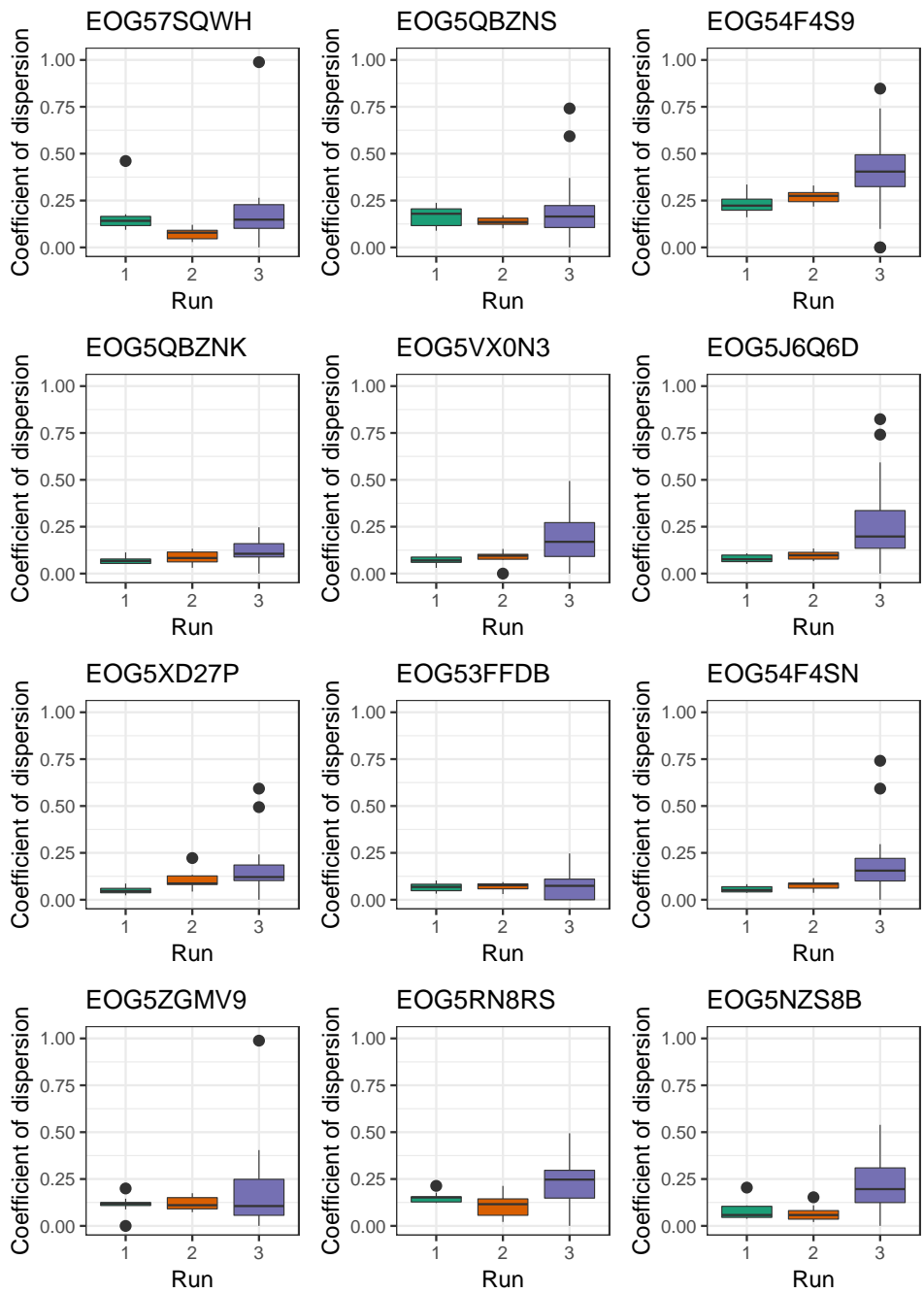

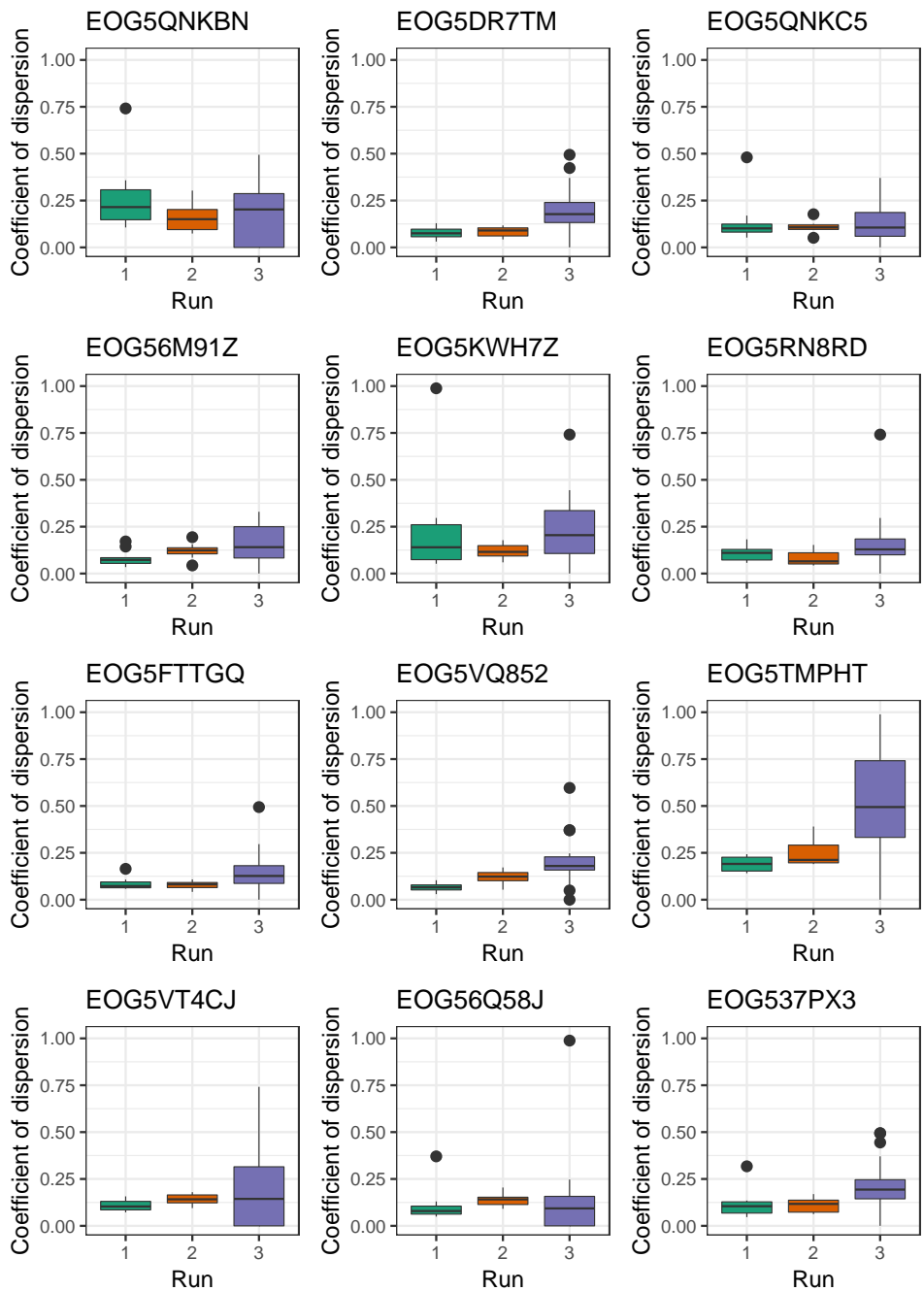

EOG59320R

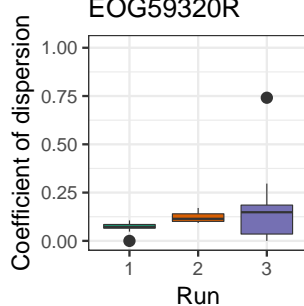

EOG5BK3JZ

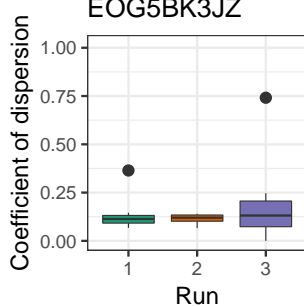

EOG5G79D7

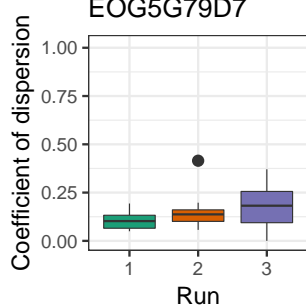

EOG5HHMHT

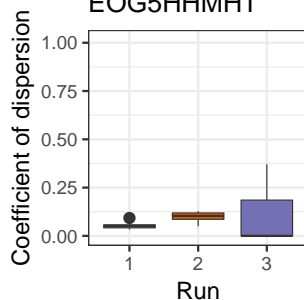

EOG579CQ2

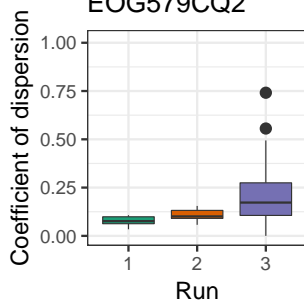

EOG54J11S

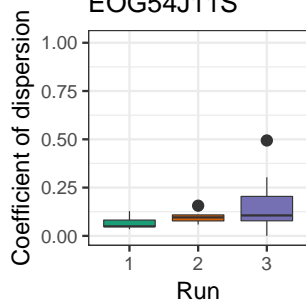

EOG5M0CH6

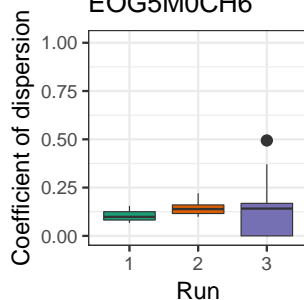

EOG5M906Z

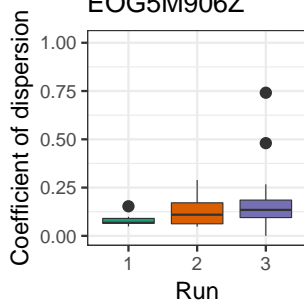

EOG5C868D

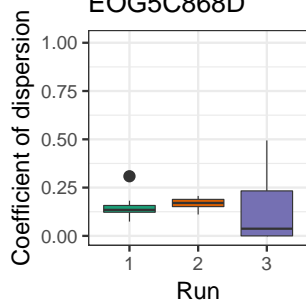

EOG541NSV

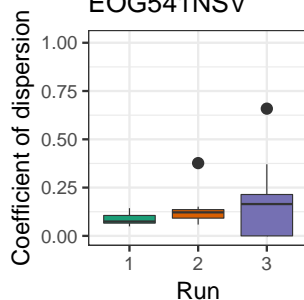

EOG563XTD

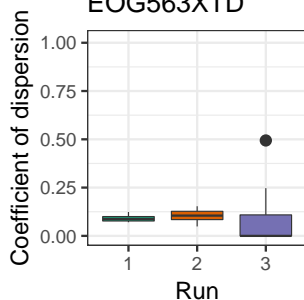

EOG5DNMJ

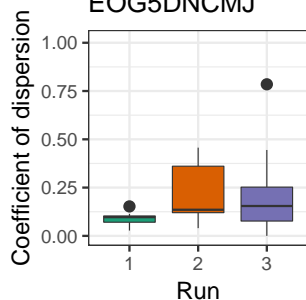

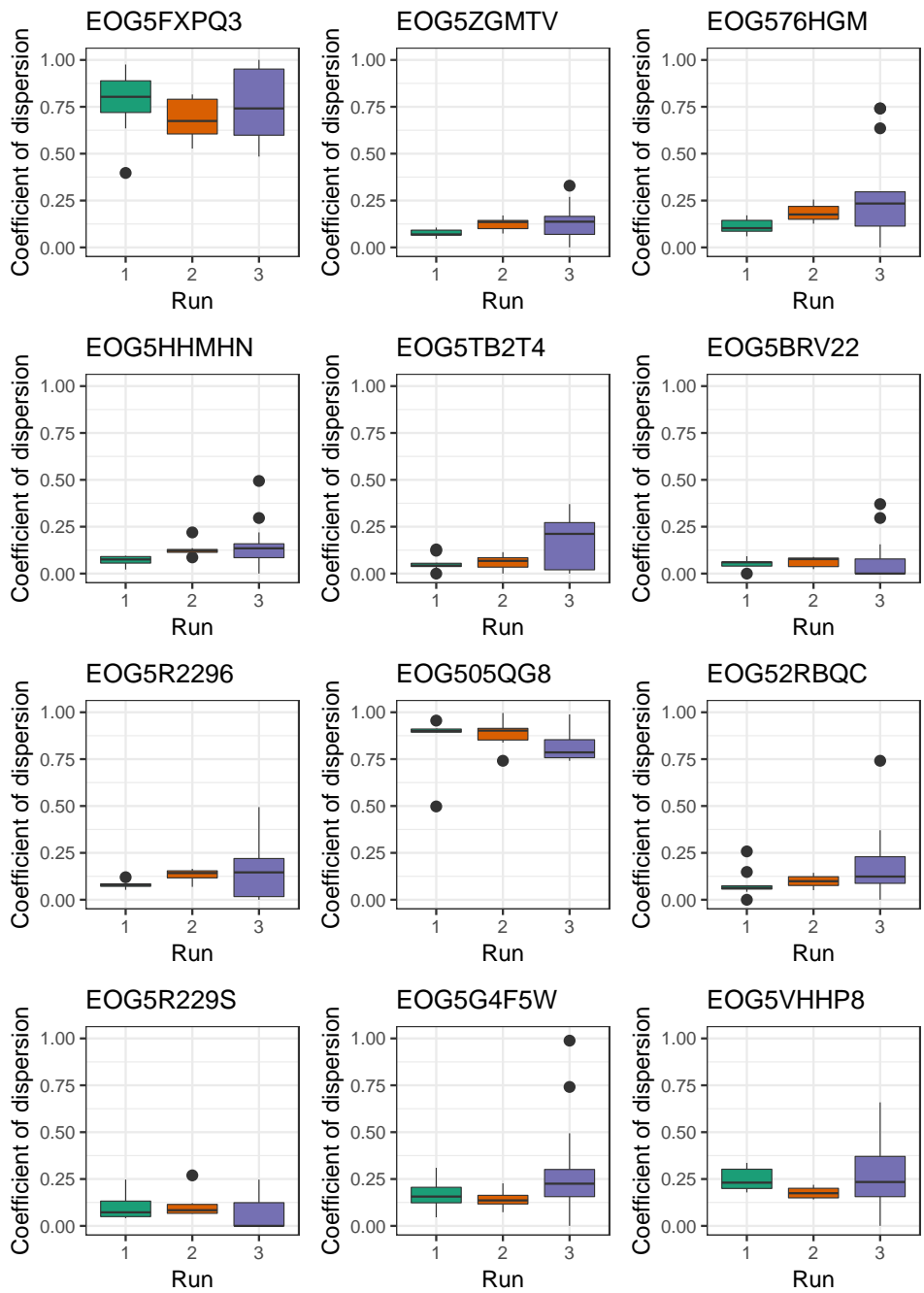

EOG5S1RP7

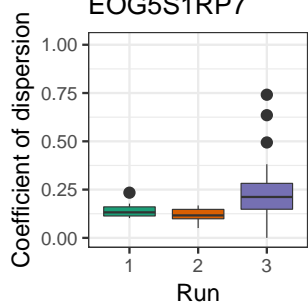

Supplement: S9 Fig — Run 1 is depicted in green, run 2 in orange, and run 3 in purple. The ID of each OG is specified above plots. Horizontal lines show median CD, vertical lines depict boxplot whiskers, and solid points represent outliers. (PDF) [file pone.0256861.s009.pdf]

A

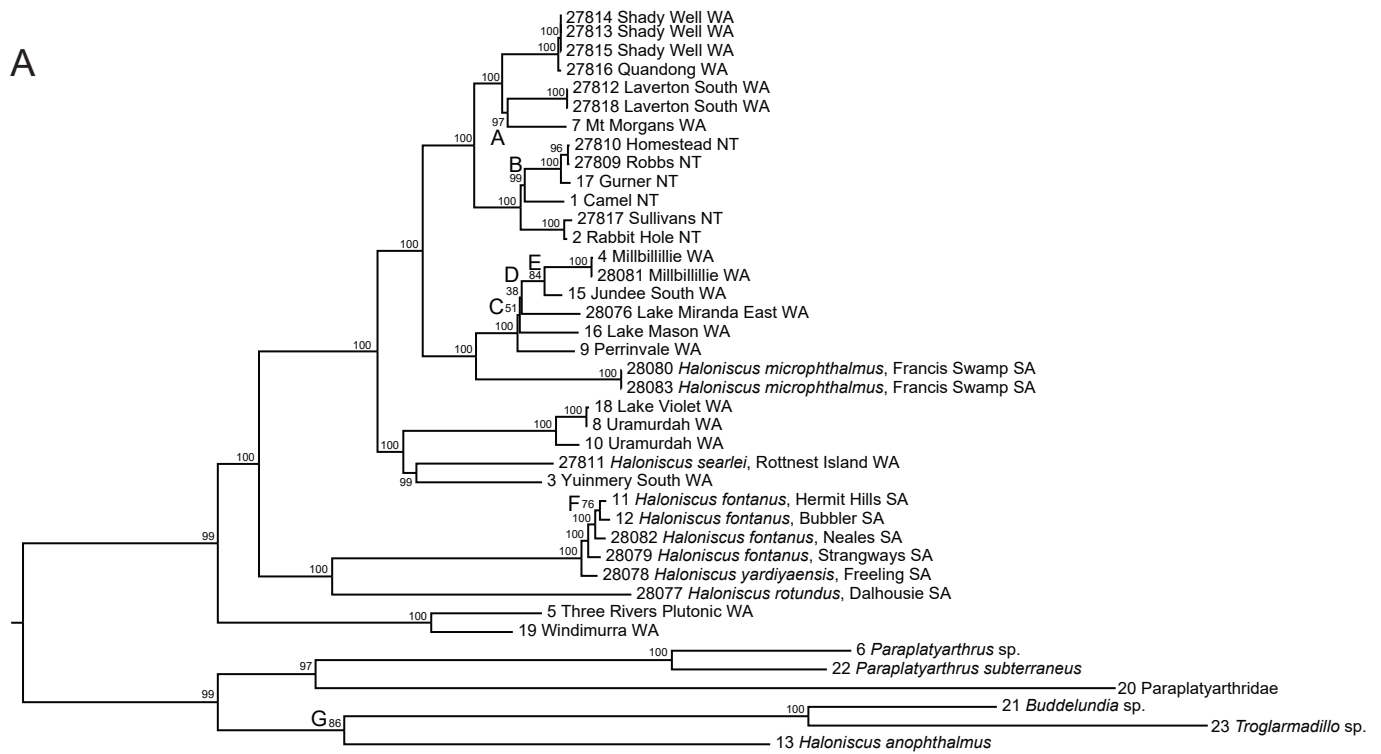

B

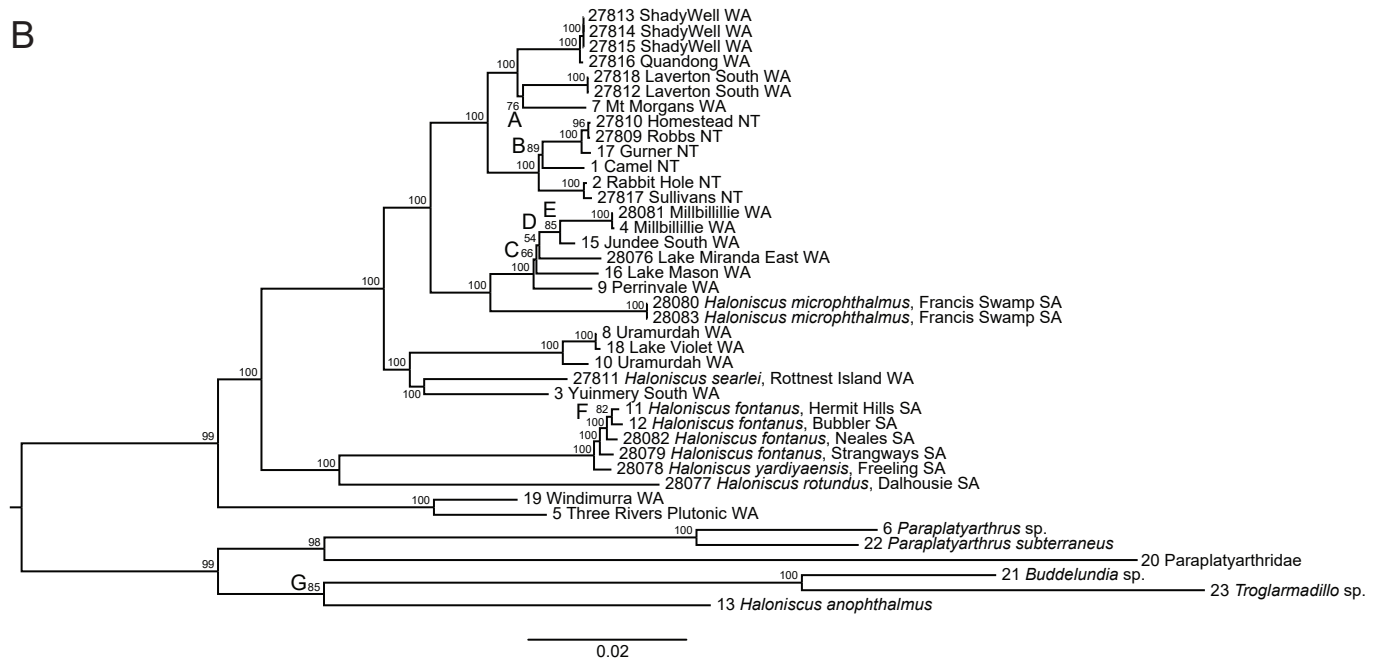

C

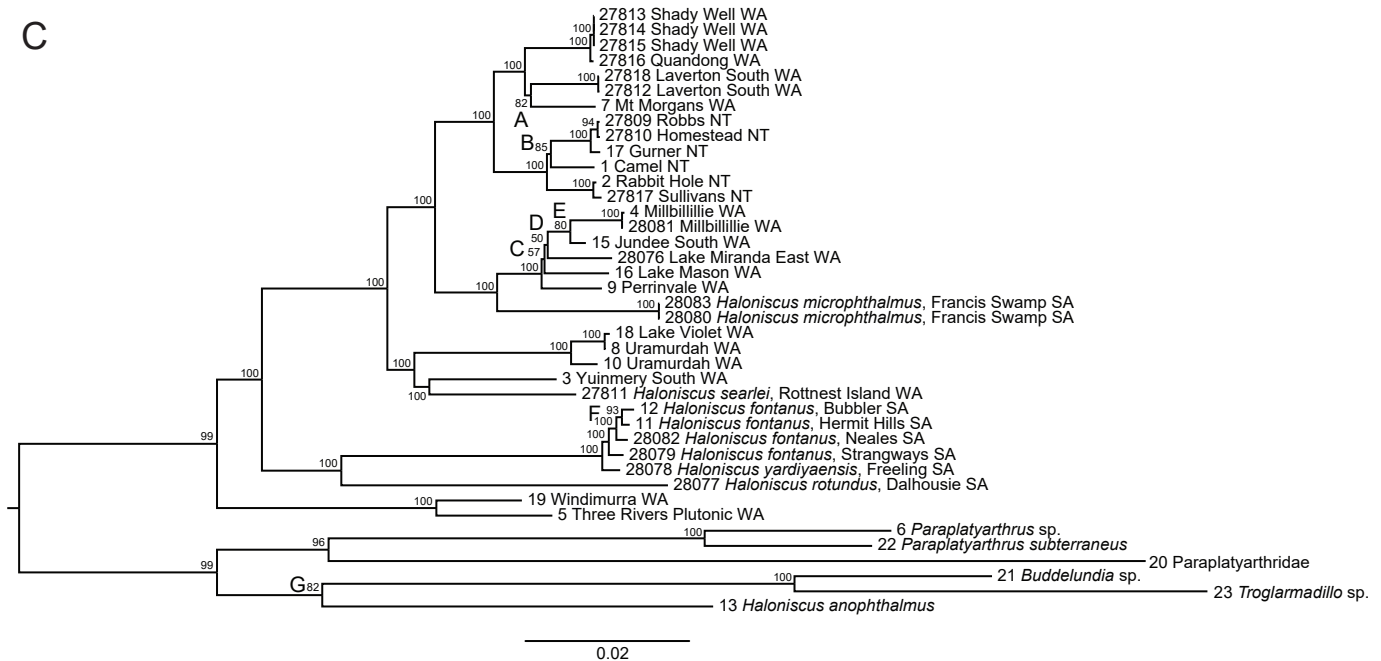

Supplement: S10 Fig — Dataset A has a 25% missing sequence threshold, dataset B a 50% threshold, and dataset C a 75% threshold. ML bootstrap values are indicated on branches, with letters A–G representing support values that differ significantly across the phylogenies and/or are referred to in the text. Isopod taxa are listed by species name and/or collection locality. Included abbreviations are as follows: WA = Western Australia, SA = South Australia, and NT = Northern Territory. (PDF) [file pone.0256861.s010.pdf]

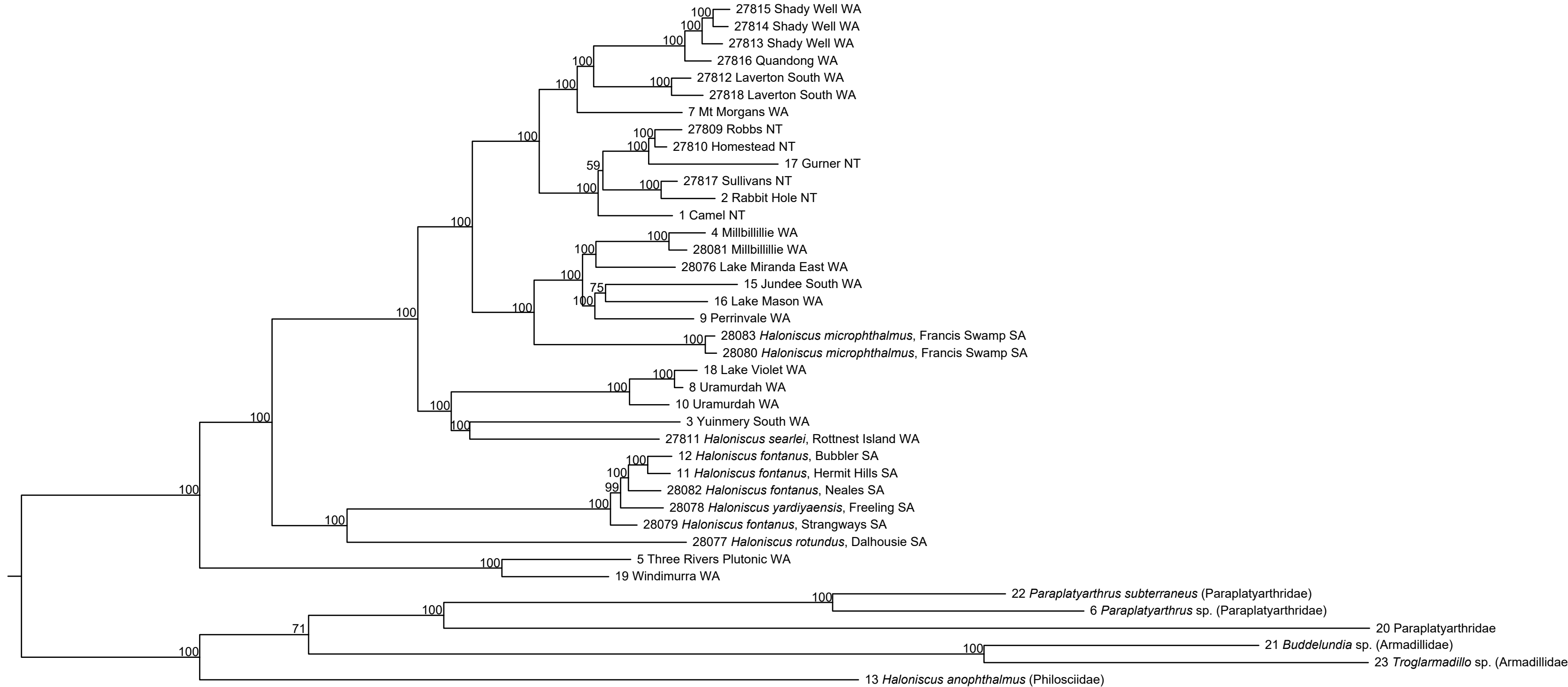

Supplement: S11 Fig — ML bootstrap values are indicated on branches and isopod taxa are listed by species name and/or collection locality. Included abbreviations are as follows: WA = Western Australia, SA = South Australia, and NT = Northern Territory. (PDF) [file pone.0256861.s011.pdf]
