# Supplementary material for: Development and evaluation of a custom bait design based on 469 single-copy protein-coding genes for exon capture of isopods (Philosciidae: Haloniscus)
Source: PLoS One. 2021 Sep 17;16(9):e0256861. doi: 10.1371/journal.pone.0256861 (PMC8448321; doi:10.1371/journal.pone.0256861)
Supplement: S5 Fig — Run 1 is depicted in green, run 2 in orange, and run 3 in purple. The ID of each OG is specified above plots. Horizontal lines are median sequencing depths, vertical lines depict boxplot whiskers, and solid points represent outliers. (PDF) [file pone.0256861.s005.pdf]

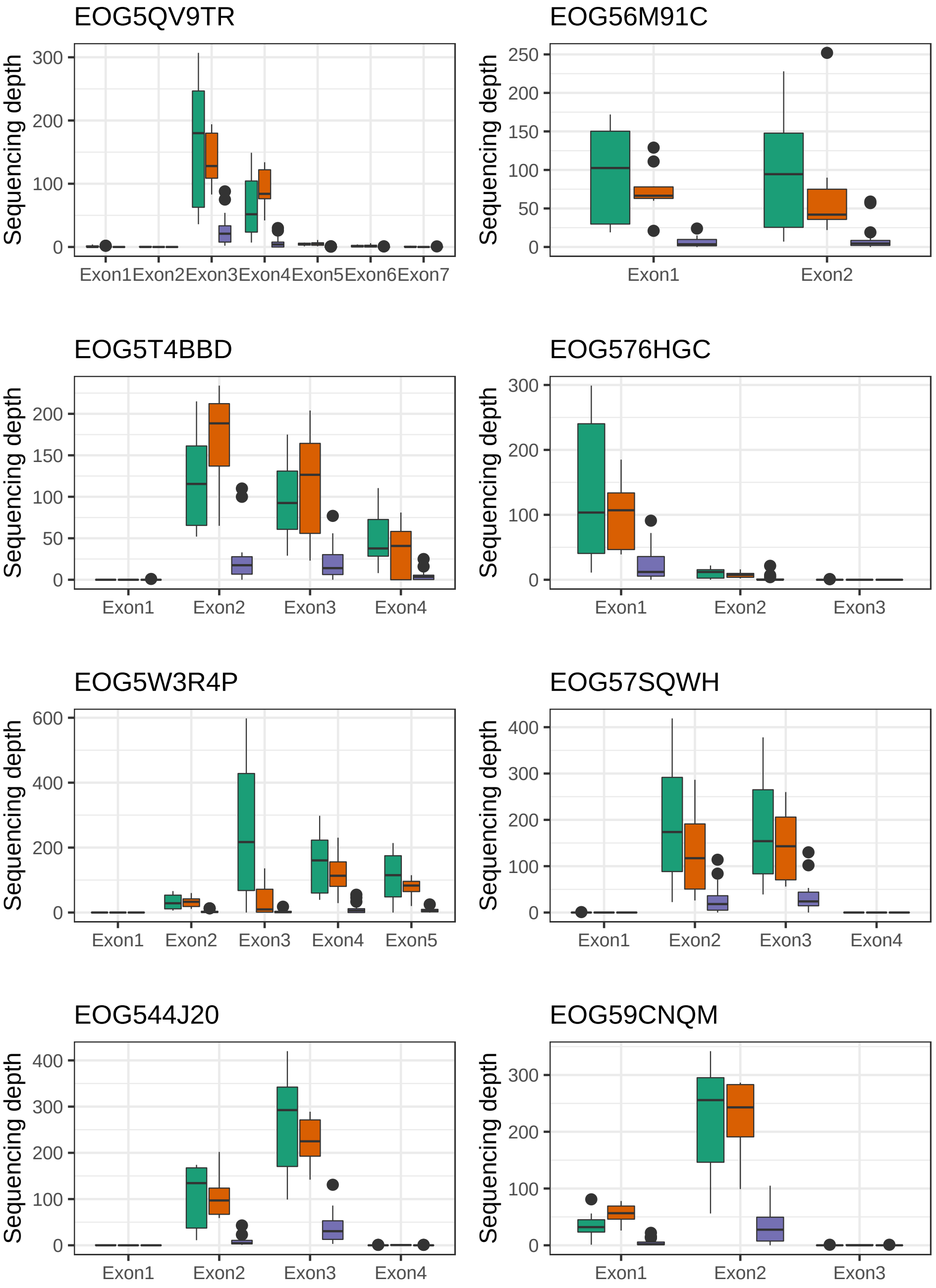
EOG5QV9TR
Sequencing depth
0
100
200
300
Exon1
Exon2
Exon3
Exon4
Exon5
Exon6
Exon7
EOG56M91C
Sequencing depth
0
50
100
150
200
250
Exon1
Exon2
EOG5T4BBD
Sequencing depth
0
50
100
150
200
Exon1
Exon2
Exon3
Exon4
EOG576HGC
Sequencing depth
0
100
200
300
Exon1
Exon2
Exon3
EOG5W3R4P
Sequencing depth
0
200
400
600
Exon1
Exon2
Exon3
Exon4
Exon5
EOG57SQWH
Sequencing depth
0
100
200
300
400
Exon1
Exon2
Exon3
Exon4
EOG544J20
Sequencing depth
0
100
200
300
400
Exon1
Exon2
Exon3
Exon4
EOG59CNQM
Sequencing depth
0
100
200
300
Exon1
Exon2
Exon3

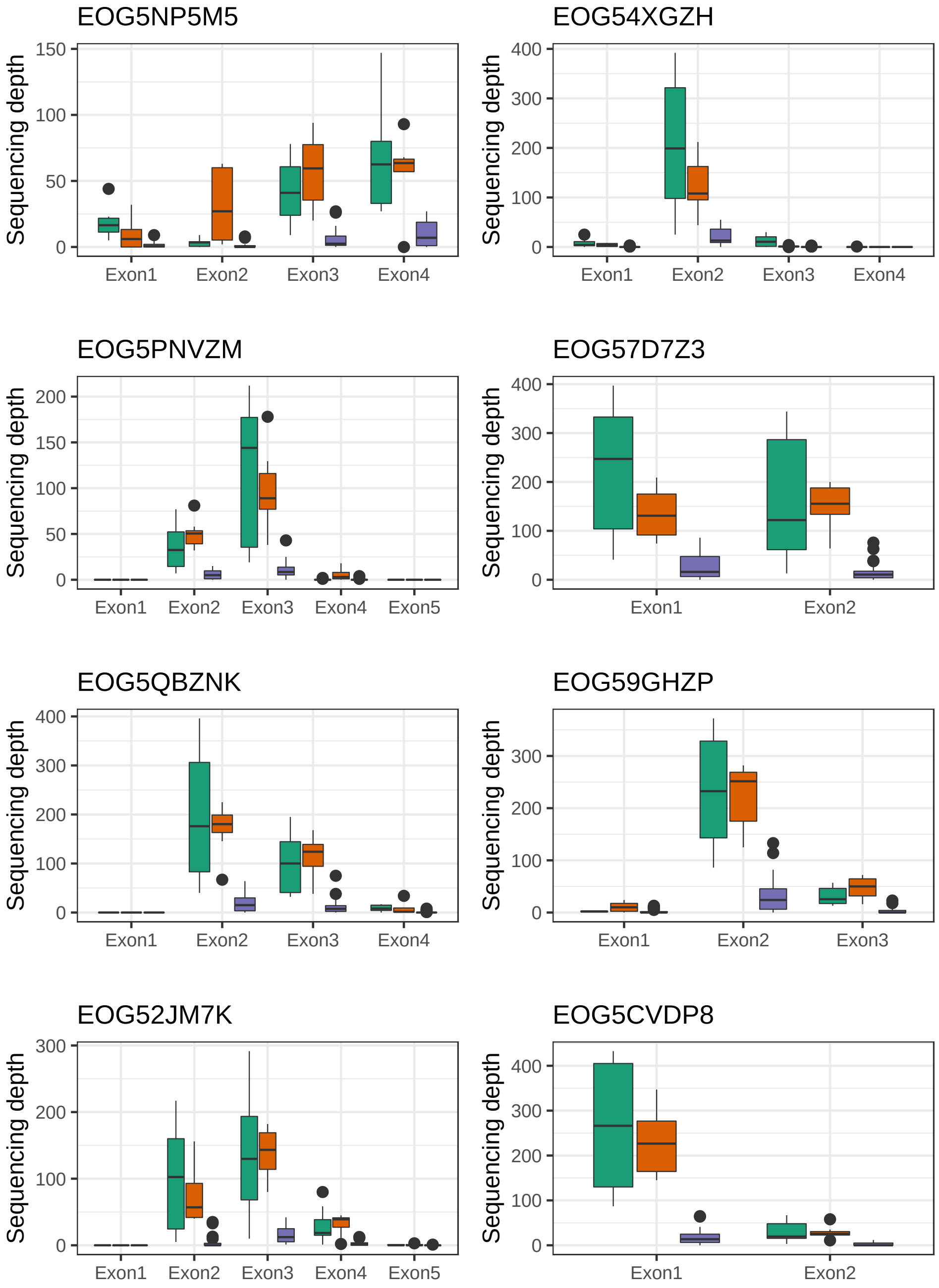
EOG5NP5M5
Sequencing depth
0
50
100
150
Exon1
Exon2
Exon3
Exon4
EOG54XGZH
Sequencing depth
0
100
200
300
400
Exon1
Exon2
Exon3
Exon4
EOG5PNVZM
Sequencing depth
0
50
100
150
200
Exon1
Exon2
Exon3
Exon4
Exon5
EOG57D7Z3
Sequencing depth
0
100
200
300
400
Exon1
Exon2
EOG5QBZNK
Sequencing depth
0
100
200
300
400
Exon1
Exon2
Exon3
Exon4
EOG59GHZP
Sequencing depth
0
100
200
300
Exon1
Exon2
Exon3
EOG52JM7K
Sequencing depth
0
100
200
300
Exon1
Exon2
Exon3
Exon4
Exon5
EOG5CVDP8
Sequencing depth
0
100
200
300
400
Exon1
Exon2

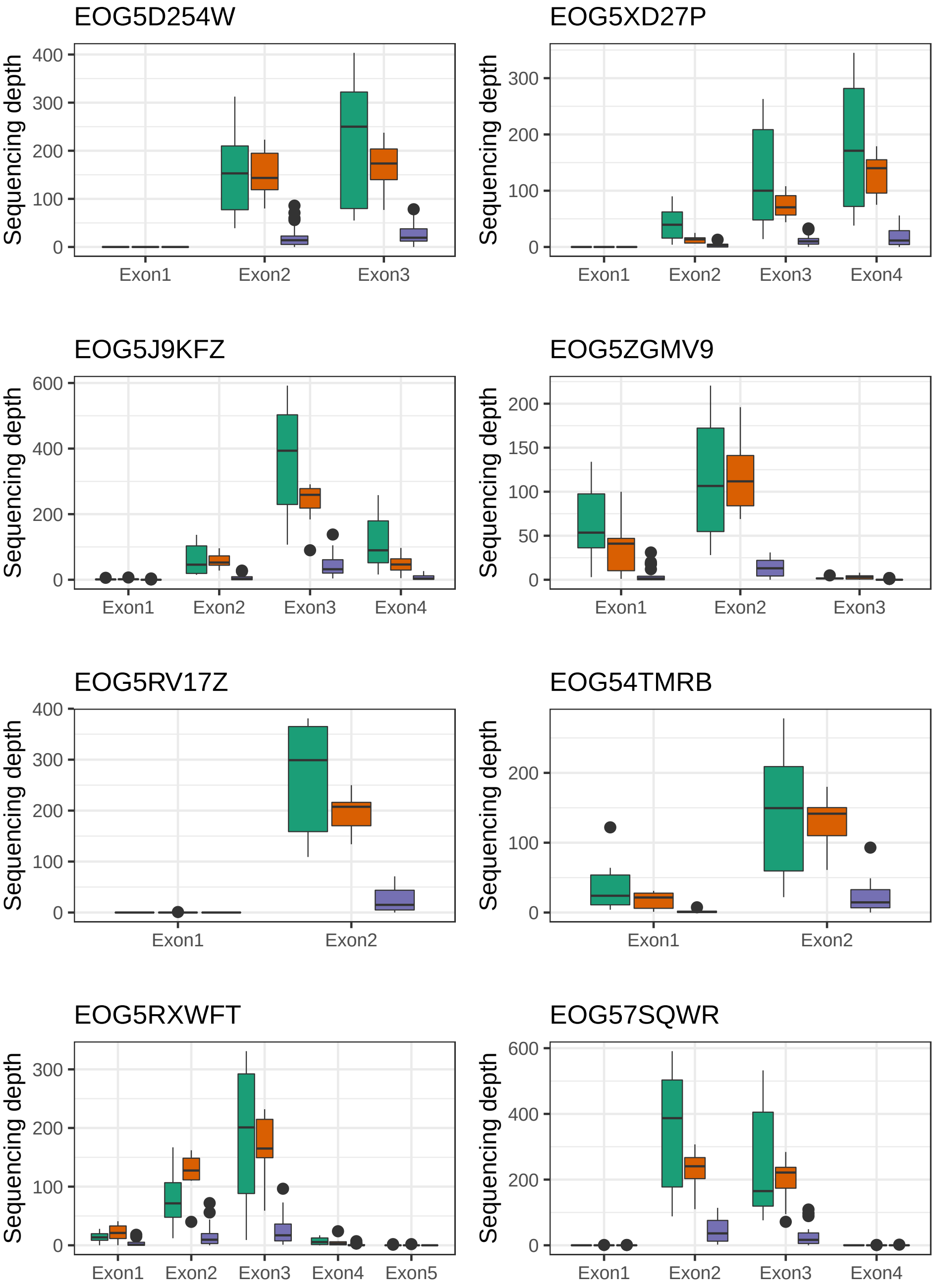
EOG5D254W
EOG5XD27P
EOG5J9KFZ
EOG5ZGMV9
EOG5RV17Z
EOG54TMRB
EOG5RXWFT
EOG57SQWR
Sequencing depth
Exon1
Exon2
Exon3
Exon4
Exon5

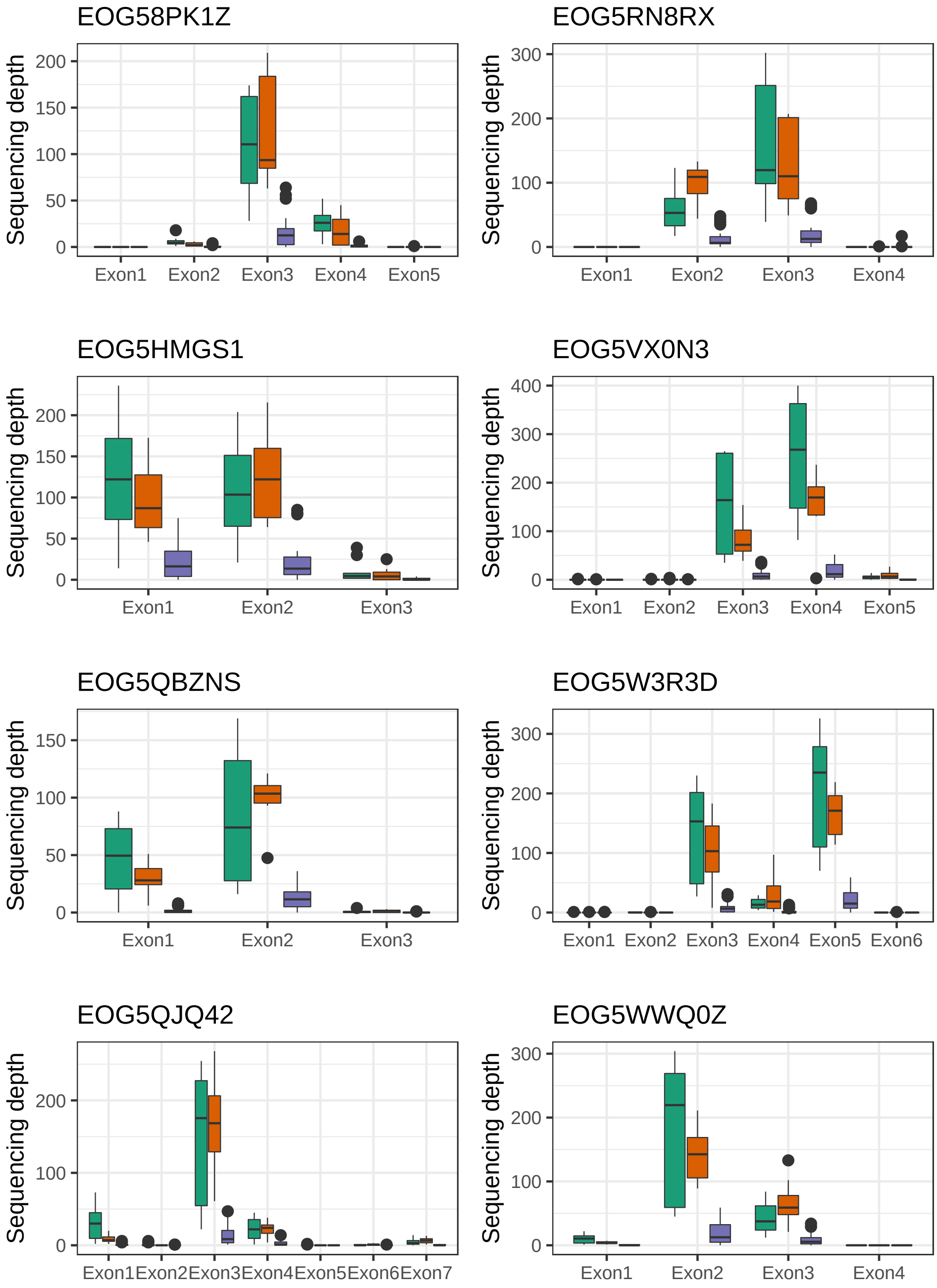
EOG58PK1Z
EOG5RN8RX
EOG5HMGS1
EOG5VX0N3
EOG5QBZNS
EOG5W3R3D
EOG5QJQ42
EOG5WWQ0Z
Sequencing depth
Exon1
Exon2
Exon3
Exon4
Exon5
Exon6
Exon7

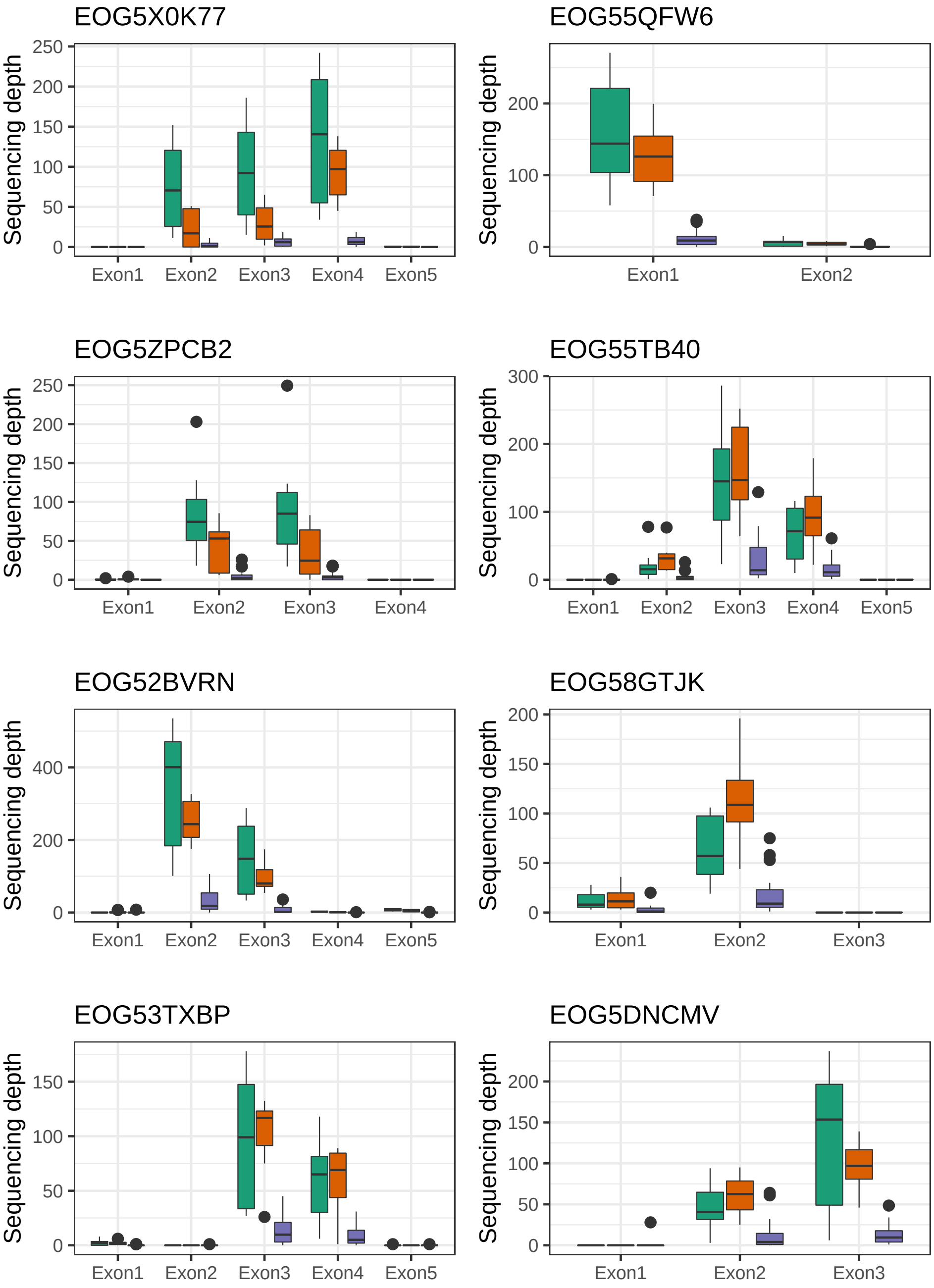
EOG5X0K77
EOG55QFW6
EOG5ZPCB2
EOG55TB40
EOG52BVRN
EOG58GTJK
EOG53TXBP
EOG5DNCMV
Sequencing depth
Exon1
Exon2
Exon3
Exon4
Exon5
0
50
100
150
200
250
300
400

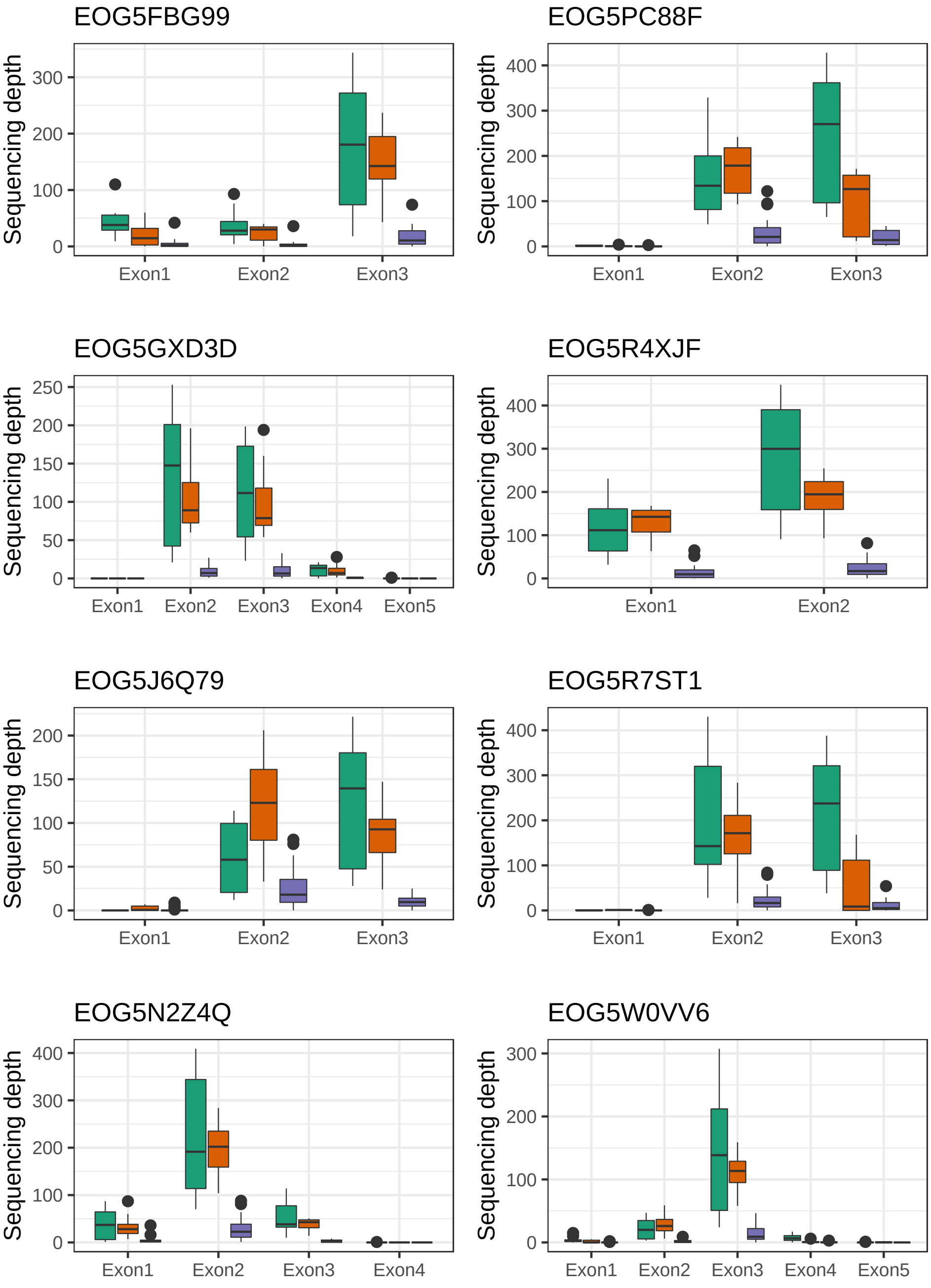
EOG5FBG99
Sequencing depth
0
100
200
300
Exon1
Exon2
Exon3
EOG5PC88F
Sequencing depth
0
100
200
300
400
Exon1
Exon2
Exon3
EOG5GXD3D
Sequencing depth
0
50
100
150
200
250
Exon1
Exon2
Exon3
Exon4
Exon5
EOG5R4XJF
Sequencing depth
0
100
200
300
400
Exon1
Exon2
EOG5J6Q79
Sequencing depth
0
50
100
150
200
Exon1
Exon2
Exon3
EOG5R7ST1
Sequencing depth
0
100
200
300
400
Exon1
Exon2
Exon3
EOG5N2Z4Q
Sequencing depth
0
100
200
300
400
Exon1
Exon2
Exon3
Exon4
EOG5W0VV6
Sequencing depth
0
100
200
300
Exon1
Exon2
Exon3
Exon4
Exon5

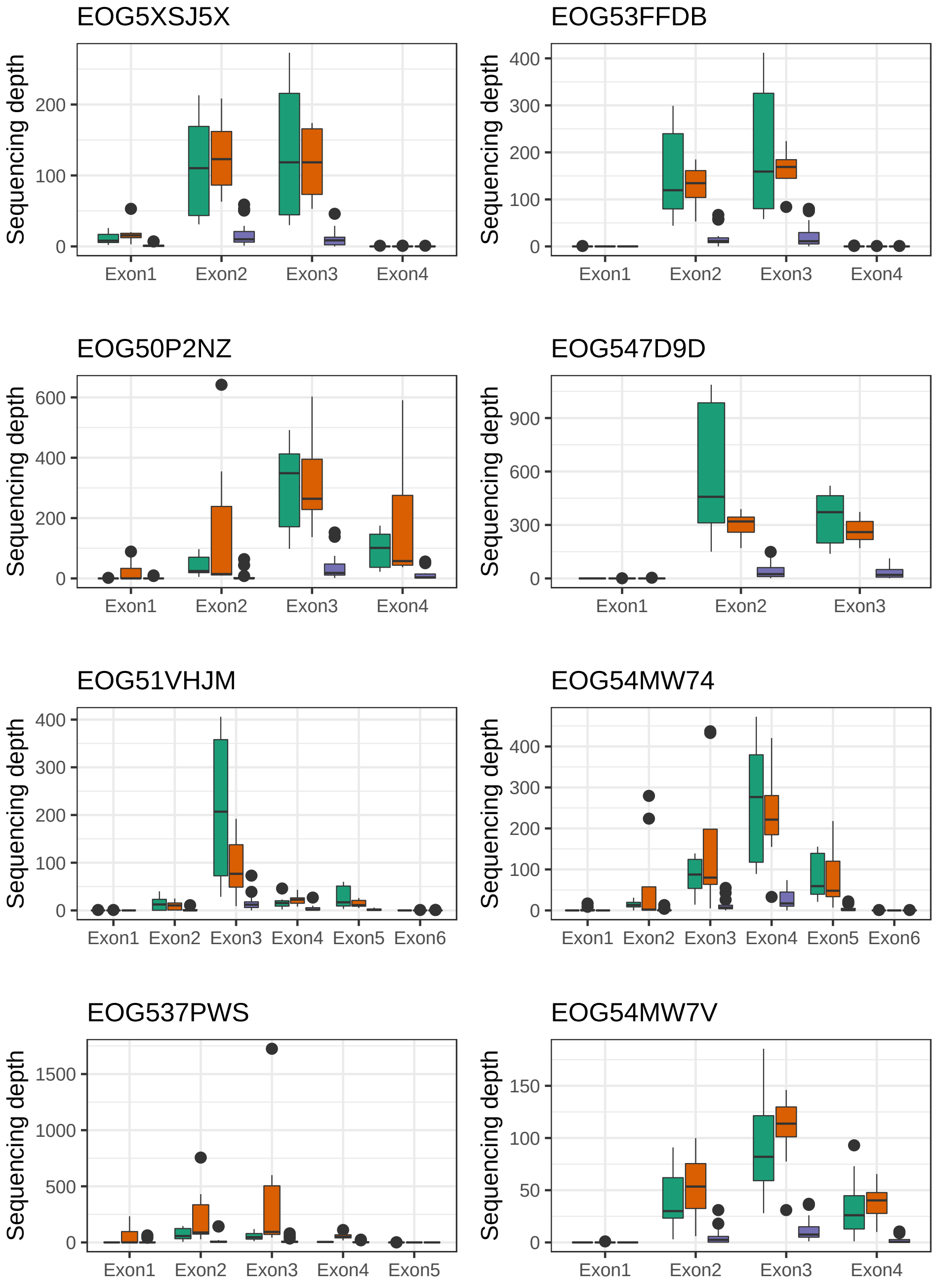
EOG5XSJ5X
Sequencing depth
0
100
200
Exon1
Exon2
Exon3
Exon4
EOG53FFDB
Sequencing depth
0
100
200
300
400
Exon1
Exon2
Exon3
Exon4
EOG50P2NZ
Sequencing depth
0
200
400
600
Exon1
Exon2
Exon3
Exon4
EOG547D9D
Sequencing depth
0
300
600
900
Exon1
Exon2
Exon3
EOG51VHJM
Sequencing depth
0
100
200
300
400
Exon1
Exon2
Exon3
Exon4
Exon5
Exon6
EOG54MW74
Sequencing depth
0
100
200
300
400
Exon1
Exon2
Exon3
Exon4
Exon5
Exon6
EOG537PWS
Sequencing depth
0
500
1000
1500
Exon1
Exon2
Exon3
Exon4
Exon5
EOG54MW7V
Sequencing depth
0
50
100
150
Exon1
Exon2
Exon3
Exon4

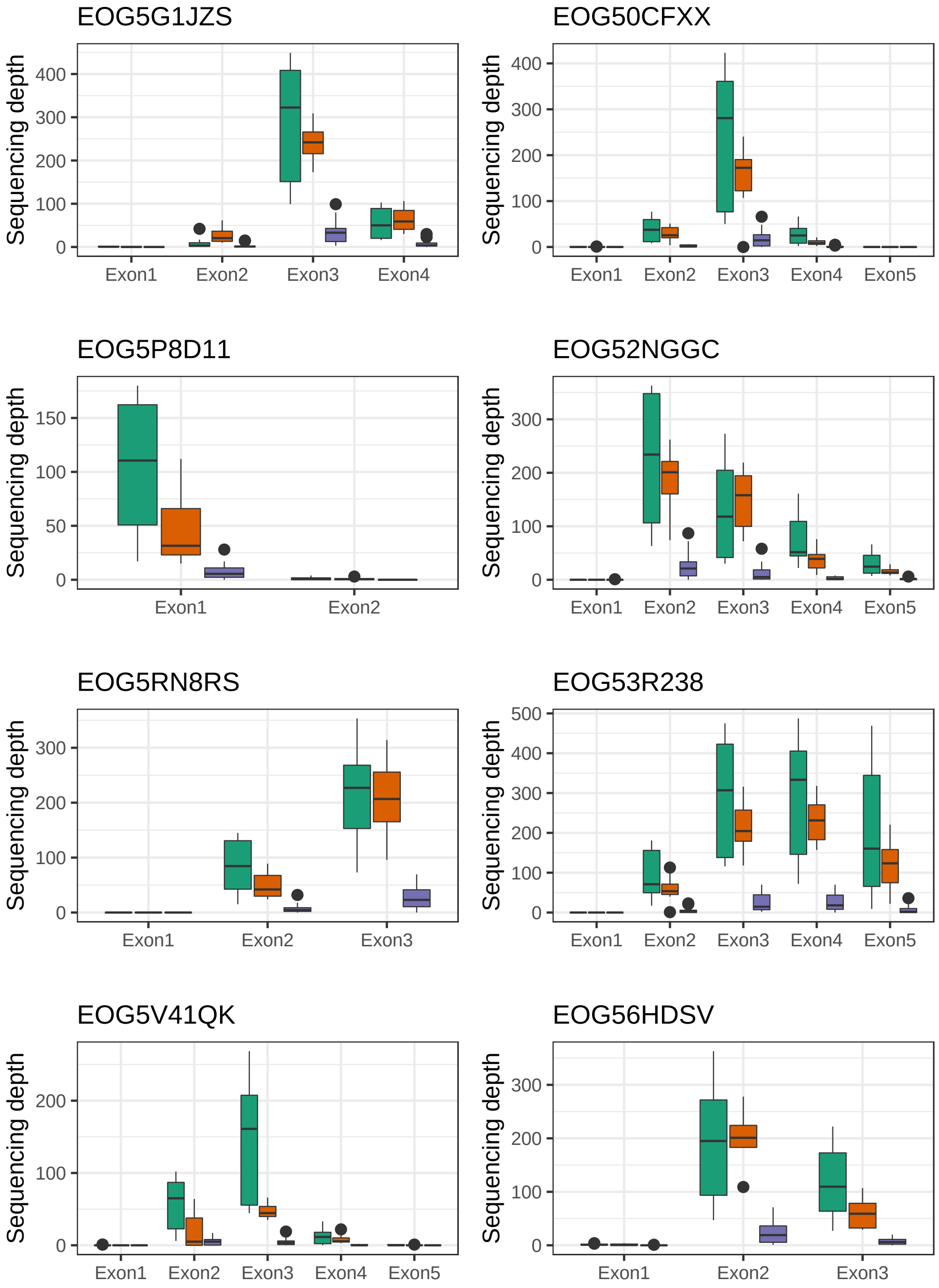
EOG5G1JZS
Sequencing depth
0
100
200
300
400
Exon1
Exon2
Exon3
Exon4
EOG50CFXX
Sequencing depth
0
100
200
300
400
Exon1
Exon2
Exon3
Exon4
Exon5
EOG5P8D11
Sequencing depth
0
50
100
150
Exon1
Exon2
EOG52NGGC
Sequencing depth
0
100
200
300
Exon1
Exon2
Exon3
Exon4
Exon5
EOG5RN8RS
Sequencing depth
0
100
200
300
Exon1
Exon2
Exon3
EOG53R238
Sequencing depth
0
100
200
300
400
500
Exon1
Exon2
Exon3
Exon4
Exon5
EOG5V41QK
Sequencing depth
0
100
200
Exon1
Exon2
Exon3
Exon4
Exon5
EOG56HDSV
Sequencing depth
0
100
200
300
Exon1
Exon2
Exon3

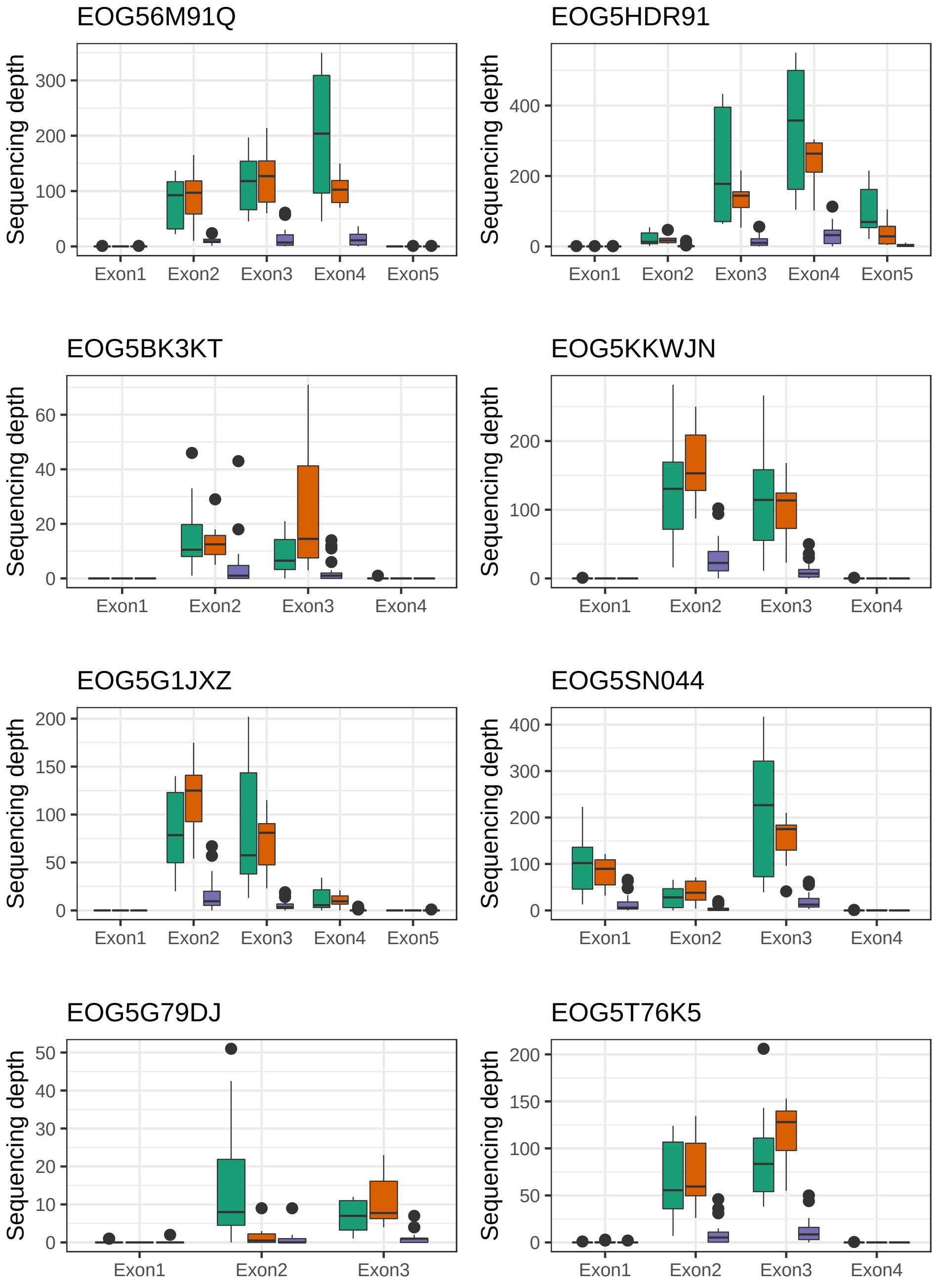
EOG56M91Q
Sequencing depth
0
100
200
300
Exon1
Exon2
Exon3
Exon4
Exon5
EOG5HDR91
Sequencing depth
0
200
400
Exon1
Exon2
Exon3
Exon4
Exon5
EOG5BK3KT
Sequencing depth
0
20
40
60
Exon1
Exon2
Exon3
Exon4
EOG5KKWJN
Sequencing depth
0
100
200
Exon1
Exon2
Exon3
Exon4
EOG5G1JXZ
Sequencing depth
0
50
100
150
200
Exon1
Exon2
Exon3
Exon4
Exon5
EOG5SN044
Sequencing depth
0
100
200
300
400
Exon1
Exon2
Exon3
Exon4
EOG5G79DJ
Sequencing depth
0
10
20
30
40
50
Exon1
Exon2
Exon3
EOG5T76K5
Sequencing depth
0
50
100
150
200
Exon1
Exon2
Exon3
Exon4

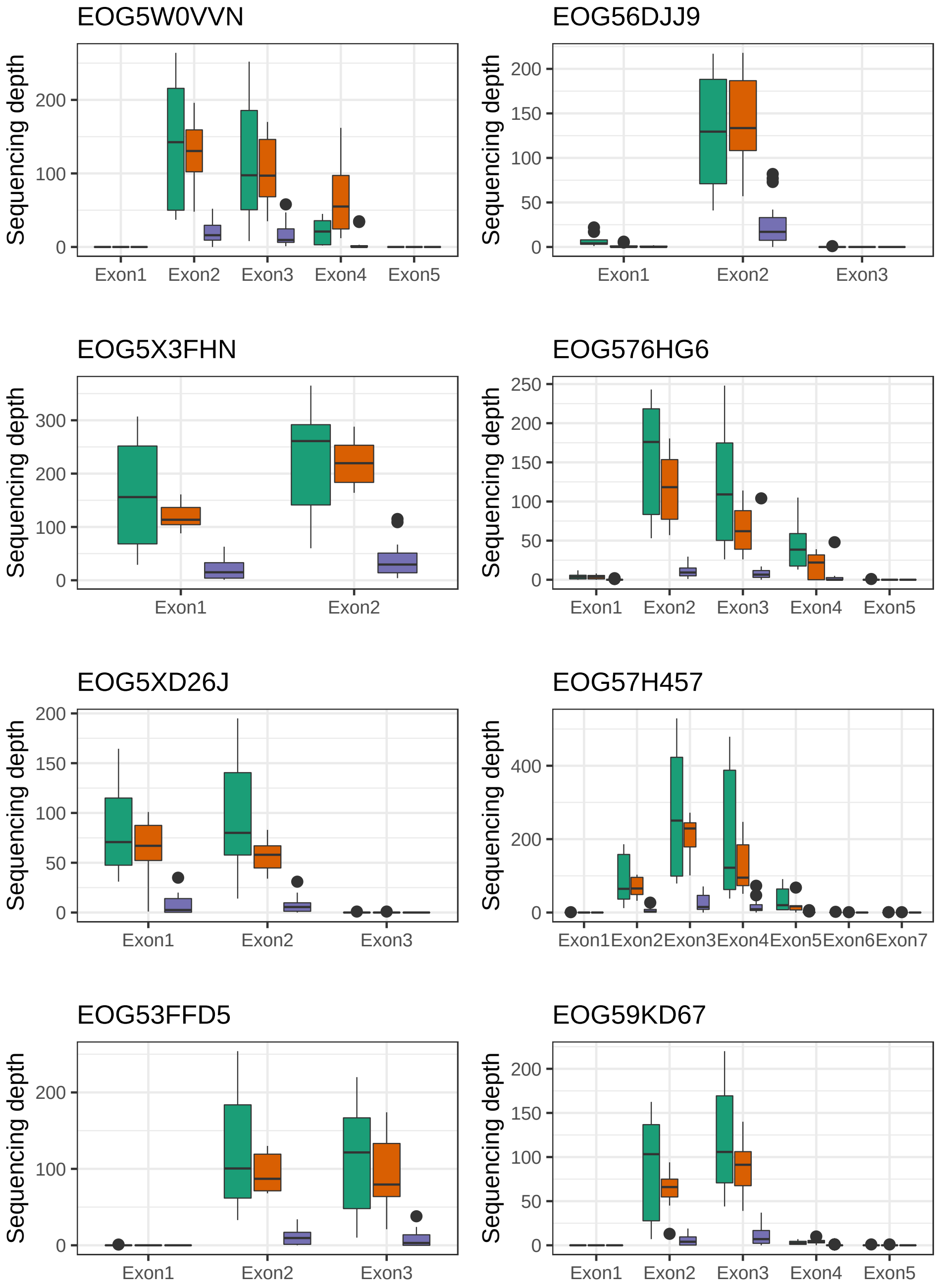
EOG5W0VVN
Sequencing depth
0
100
200
Exon1
Exon2
Exon3
Exon4
Exon5
EOG56DJJ9
Sequencing depth
0
50
100
150
200
Exon1
Exon2
Exon3
EOG5X3FHN
Sequencing depth
0
100
200
300
Exon1
Exon2
EOG576HG6
Sequencing depth
0
50
100
150
200
250
Exon1
Exon2
Exon3
Exon4
Exon5
EOG5XD26J
Sequencing depth
0
50
100
150
200
Exon1
Exon2
Exon3
EOG57H457
Sequencing depth
0
200
400
Exon1
Exon2
Exon3
Exon4
Exon5
Exon6
Exon7
EOG53FFD5
Sequencing depth
0
100
200
Exon1
Exon2
Exon3
EOG59KD67
Sequencing depth
0
50
100
150
200
Exon1
Exon2
Exon3
Exon4
Exon5

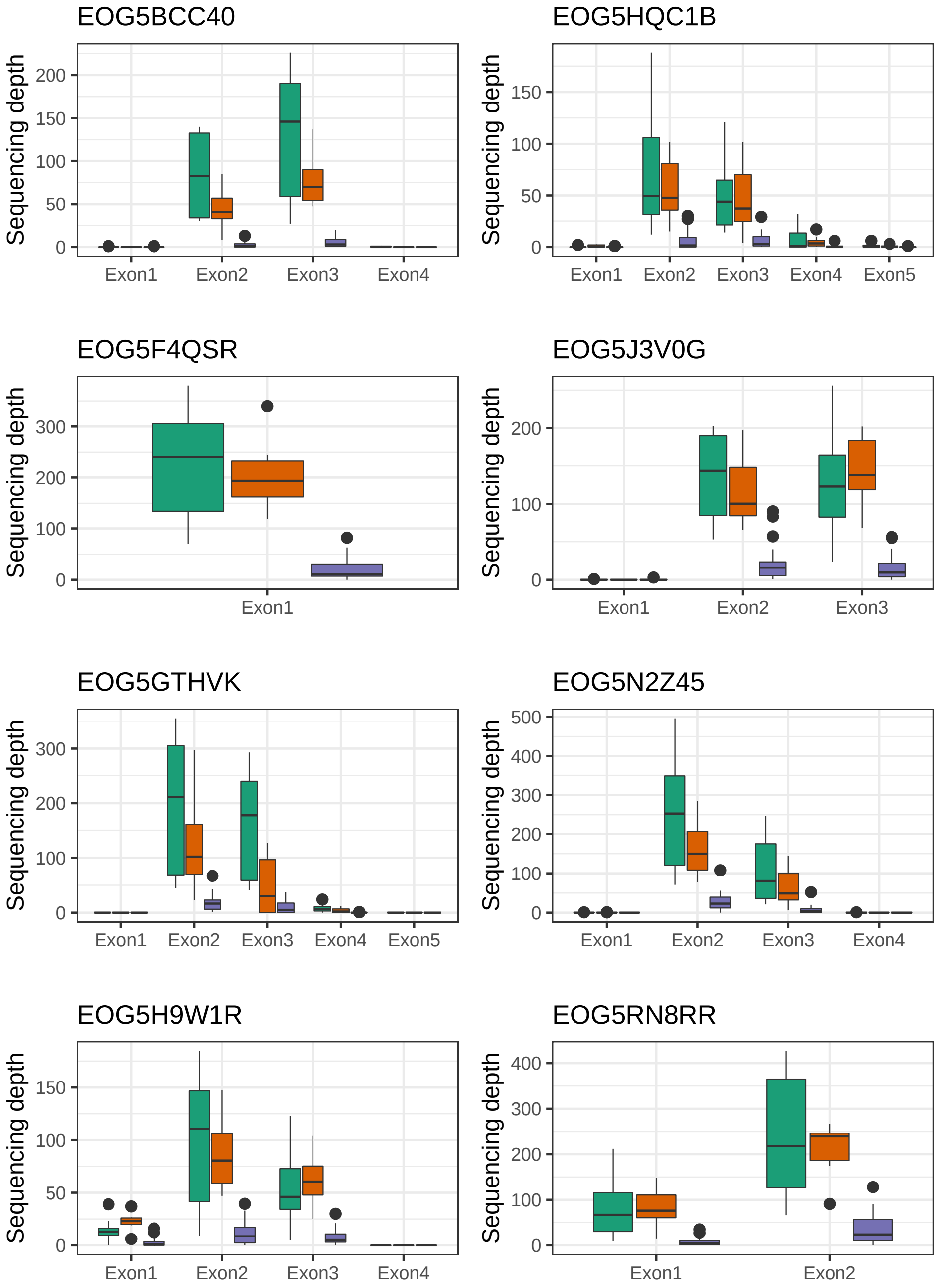

EOG5BCC40
EOG5HQC1B
EOG5F4QSR
EOG5J3V0G
EOG5GTHVK
EOG5N2Z45
EOG5H9W1R
EOG5RN8RR
Sequencing depth
Exon1
Exon2
Exon3
Exon4
Exon5

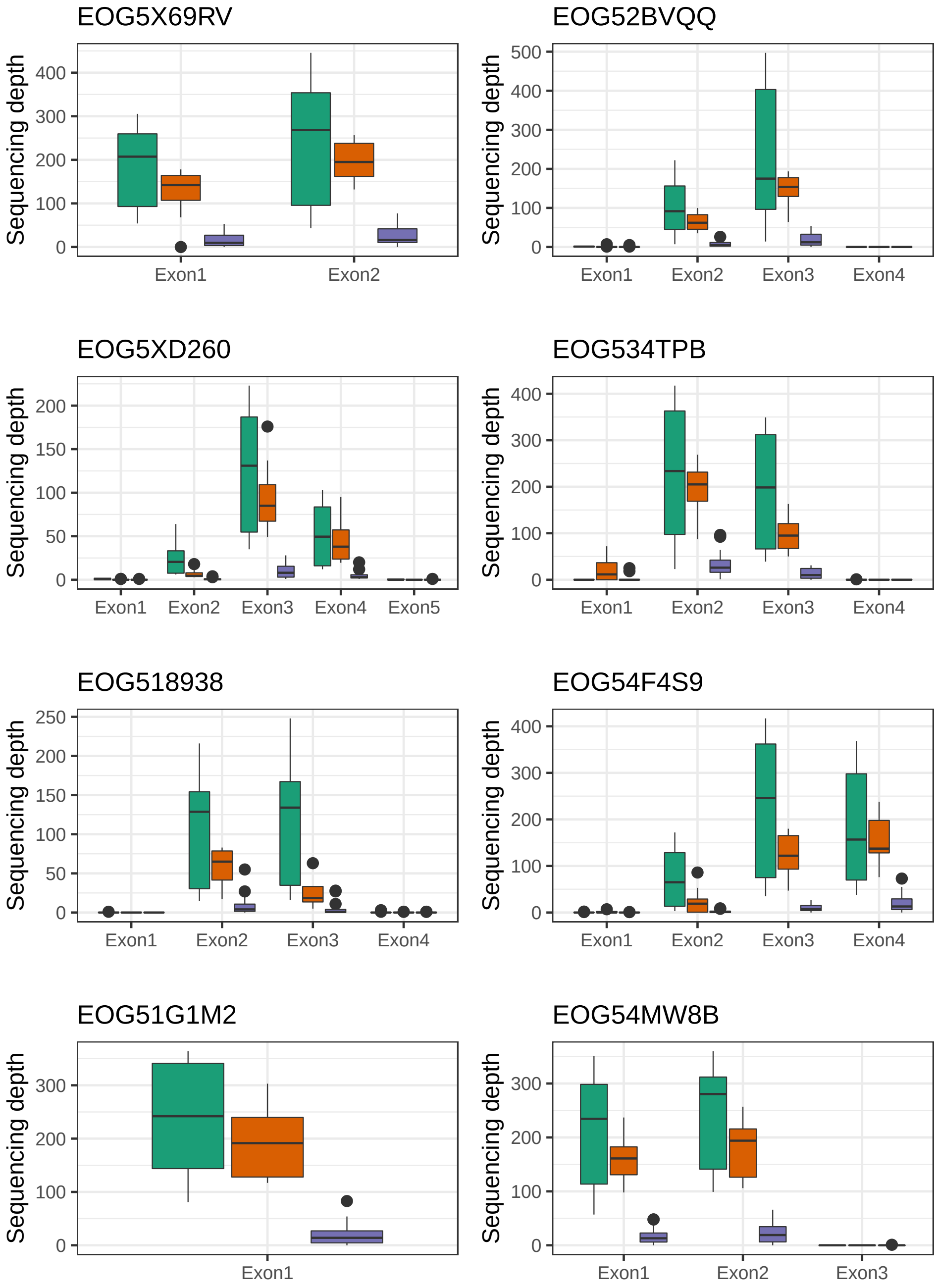
EOG5X69RV
EOG52BVQQ
EOG5XD260
EOG534TPB
EOG518938
EOG54F4S9
EOG51G1M2
EOG54MW8B
Sequencing depth
Exon1
Exon2
Exon3
Exon4
Exon5
0
50
100
150
200
250
300
400
500

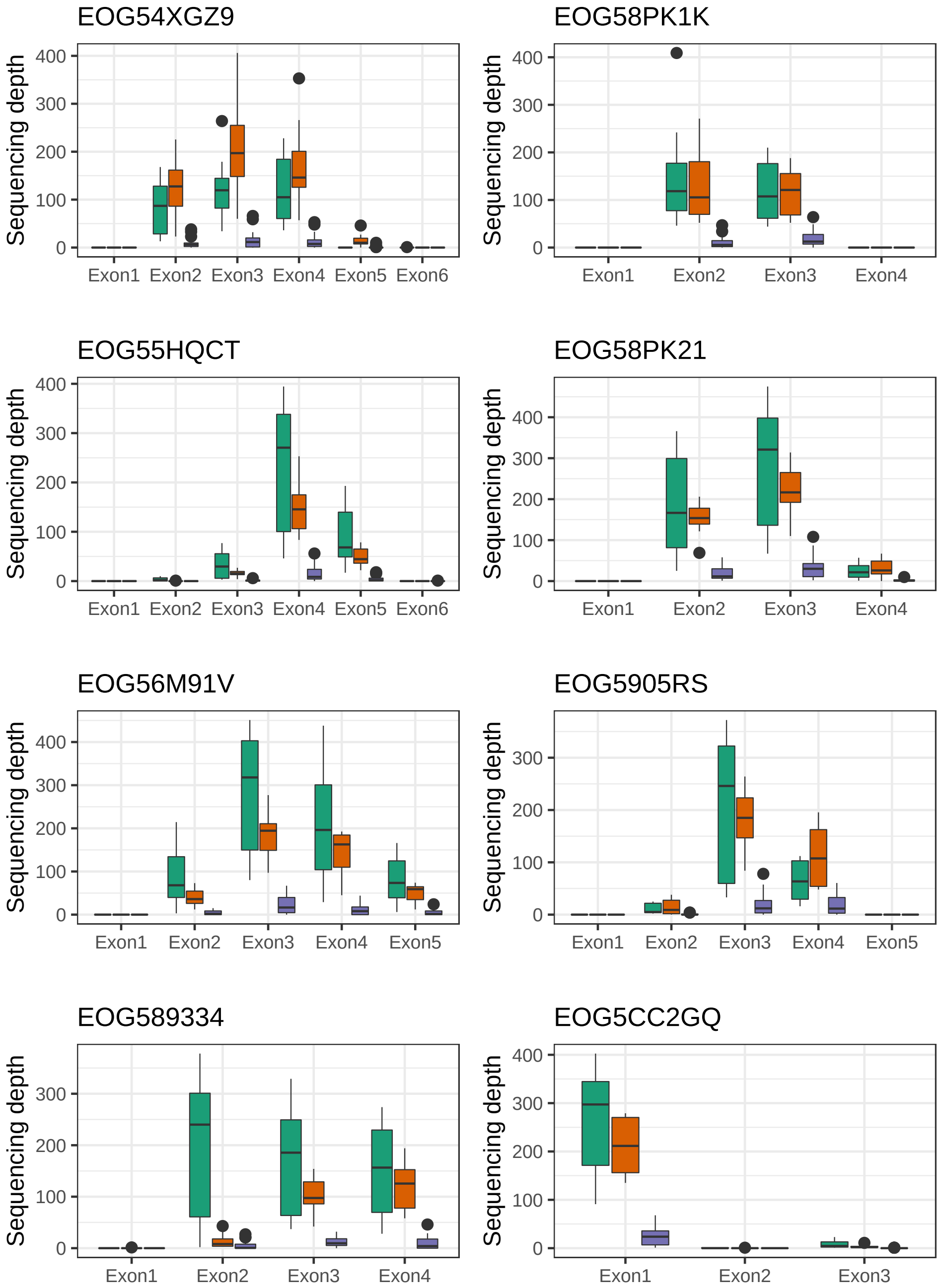
EOG54XGZ9
Sequencing depth
0
100
200
300
400
Exon1
Exon2
Exon3
Exon4
Exon5
Exon6
EOG58PK1K
Sequencing depth
0
100
200
300
400
Exon1
Exon2
Exon3
Exon4
EOG55HQCT
Sequencing depth
0
100
200
300
400
Exon1
Exon2
Exon3
Exon4
Exon5
Exon6
EOG58PK21
Sequencing depth
0
100
200
300
400
Exon1
Exon2
Exon3
Exon4
EOG56M91V
Sequencing depth
0
100
200
300
400
Exon1
Exon2
Exon3
Exon4
Exon5
EOG5905RS
Sequencing depth
0
100
200
300
Exon1
Exon2
Exon3
Exon4
Exon5
EOG589334
Sequencing depth
0
100
200
300
Exon1
Exon2
Exon3
Exon4
EOG5CC2GQ
Sequencing depth
0
100
200
300
400
Exon1
Exon2
Exon3

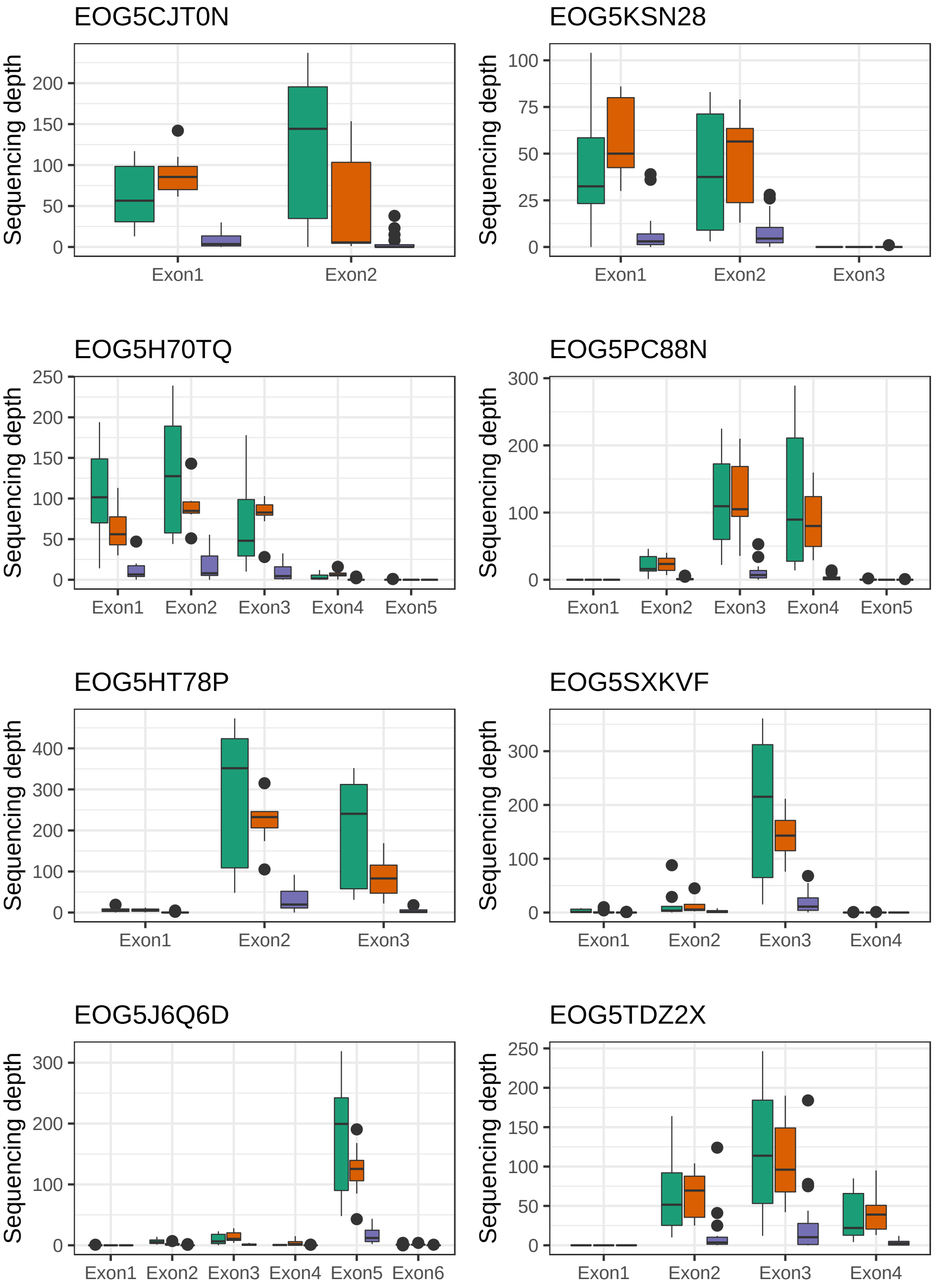
EOG5CJT0N
EOG5KSN28
EOG5H70TQ
EOG5PC88N
EOG5HT78P
EOG5SXKVF
EOG5J6Q6D
EOG5TDZ2X
Sequencing depth
Exon1
Exon2
Exon3
Exon4
Exon5
Exon6
0
25
50
75
100
150
200
250
300
400

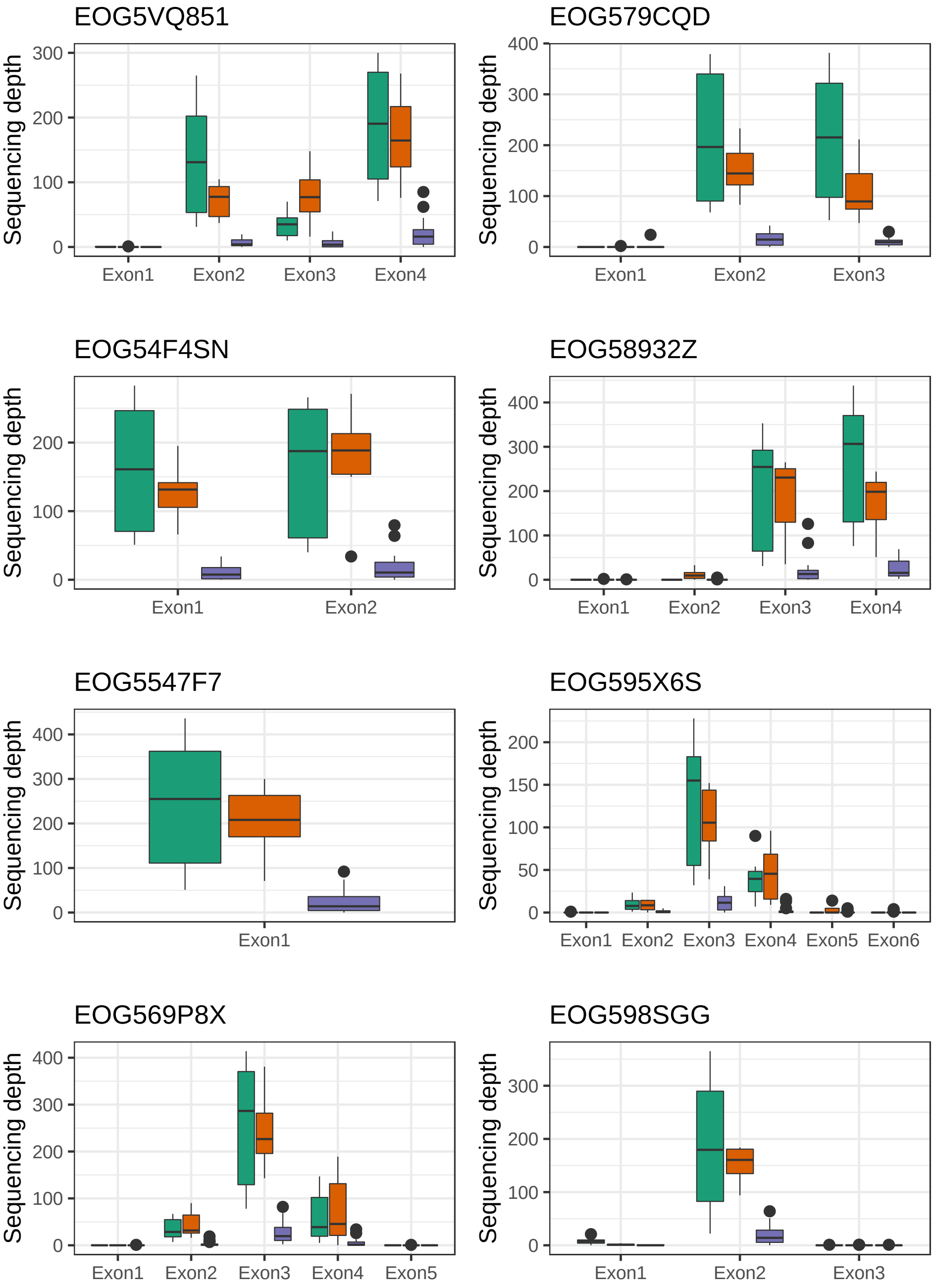
EOG5VQ851
Sequencing depth
0
100
200
300
Exon1
Exon2
Exon3
Exon4
EOG579CQD
Sequencing depth
0
100
200
300
400
Exon1
Exon2
Exon3
EOG54F4SN
Sequencing depth
0
100
200
Exon1
Exon2
EOG58932Z
Sequencing depth
0
100
200
300
400
Exon1
Exon2
Exon3
Exon4
EOG5547F7
Sequencing depth
0
100
200
300
400
Exon1
EOG595X6S
Sequencing depth
0
50
100
150
200
Exon1
Exon2
Exon3
Exon4
Exon5
Exon6
EOG569P8X
Sequencing depth
0
100
200
300
400
Exon1
Exon2
Exon3
Exon4
Exon5
EOG598SGG
Sequencing depth
0
100
200
300
Exon1
Exon2
Exon3

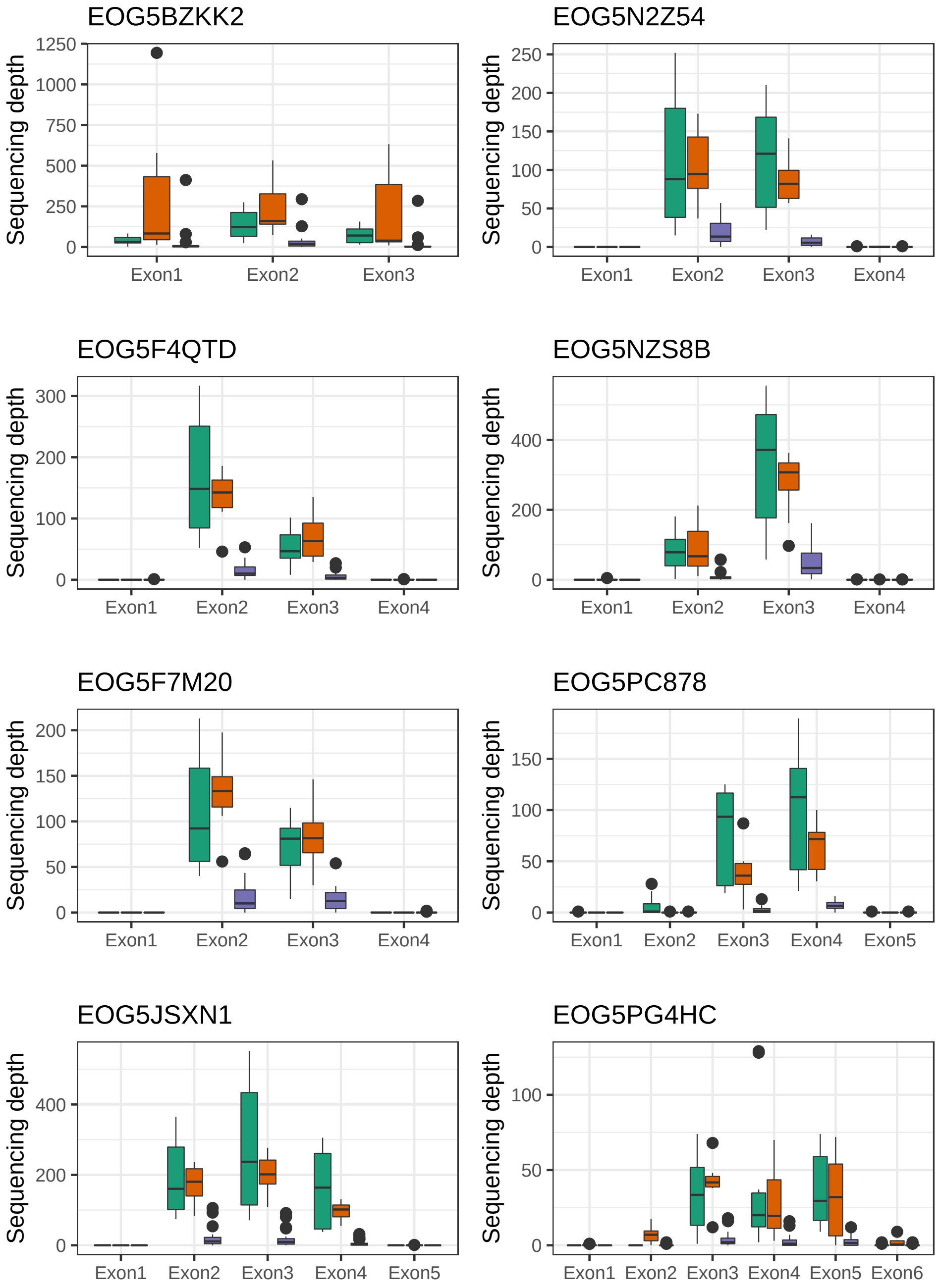
EOG5BZKK2
Sequencing depth
0
250
500
750
1000
1250
Exon1
Exon2
Exon3
EOG5N2Z54
Sequencing depth
0
50
100
150
200
250
Exon1
Exon2
Exon3
Exon4
EOG5F4QTD
Sequencing depth
0
100
200
300
Exon1
Exon2
Exon3
Exon4
EOG5NZS8B
Sequencing depth
0
200
400
Exon1
Exon2
Exon3
Exon4
EOG5F7M20
Sequencing depth
0
50
100
150
200
Exon1
Exon2
Exon3
Exon4
EOG5PC878
Sequencing depth
0
50
100
150
Exon1
Exon2
Exon3
Exon4
Exon5
EOG5JSXN1
Sequencing depth
0
200
400
Exon1
Exon2
Exon3
Exon4
Exon5
EOG5PG4HC
Sequencing depth
0
50
100
Exon1
Exon2
Exon3
Exon4
Exon5
Exon6

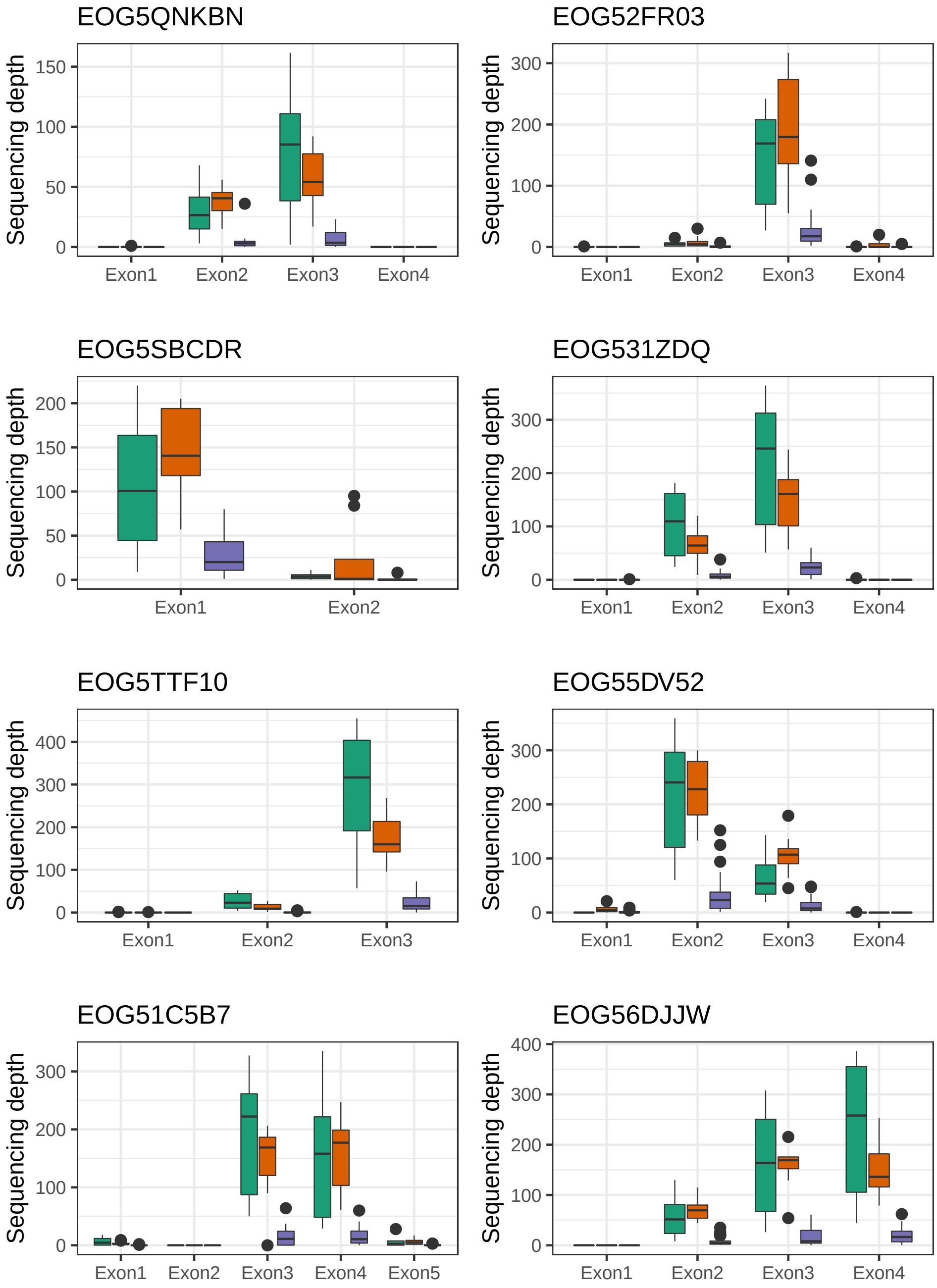
EOG5QNKBN
EOG52FR03
EOG5SBCDR
EOG531ZDQ
EOG5TTF10
EOG55DV52
EOG51C5B7
EOG56DJJW
Sequencing depth
Exon1
Exon2
Exon3
Exon4
Exon5
0
50
100
150
200
300
400

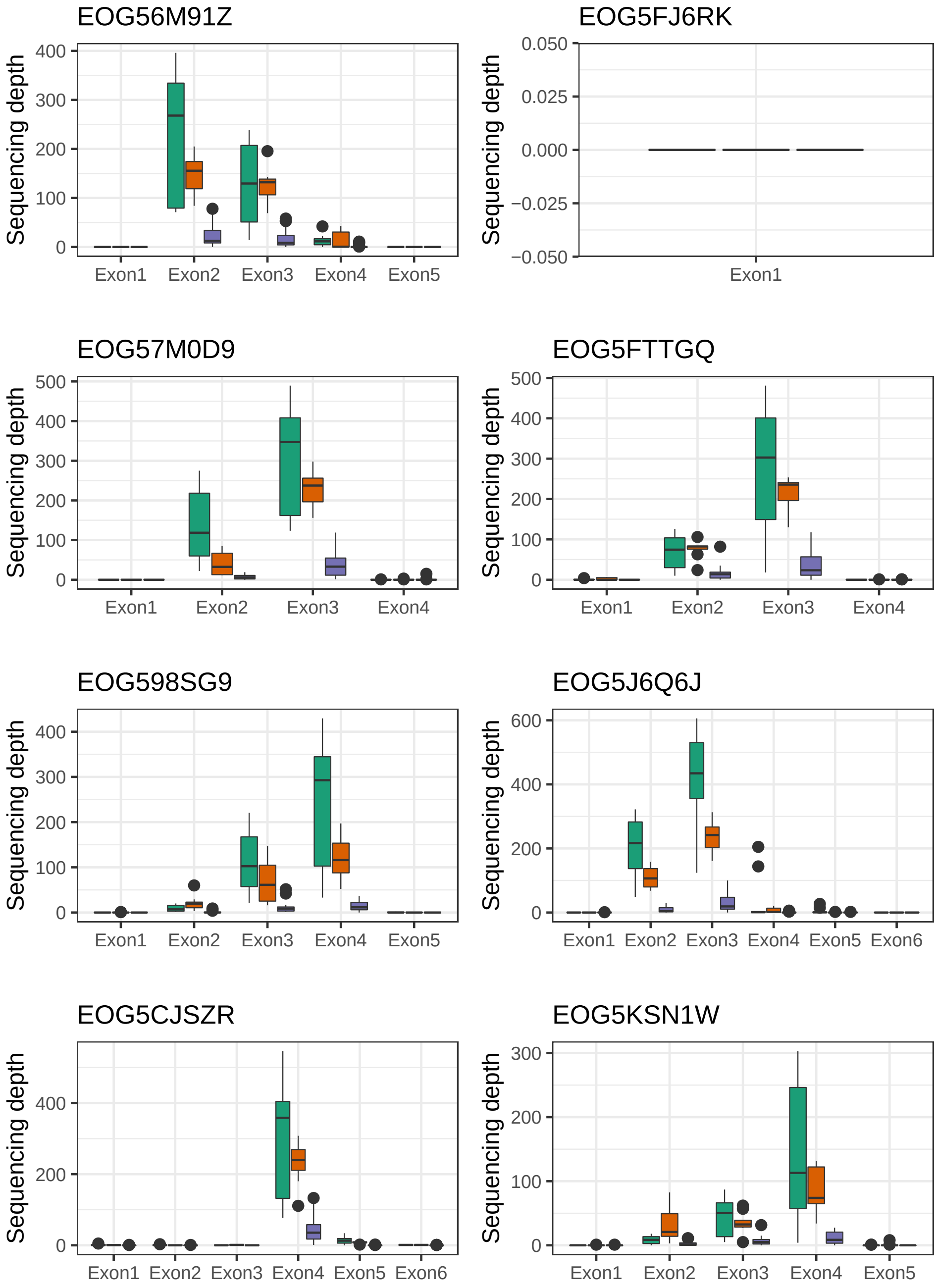
EOG56M91Z
EOG5FJ6RK
EOG57M0D9
EOG5FTTGQ
EOG598SG9
EOG5J6Q6J
EOG5CJSZR
EOG5KSN1W
Sequencing depth
Exon1
Exon2
Exon3
Exon4
Exon5
Exon6

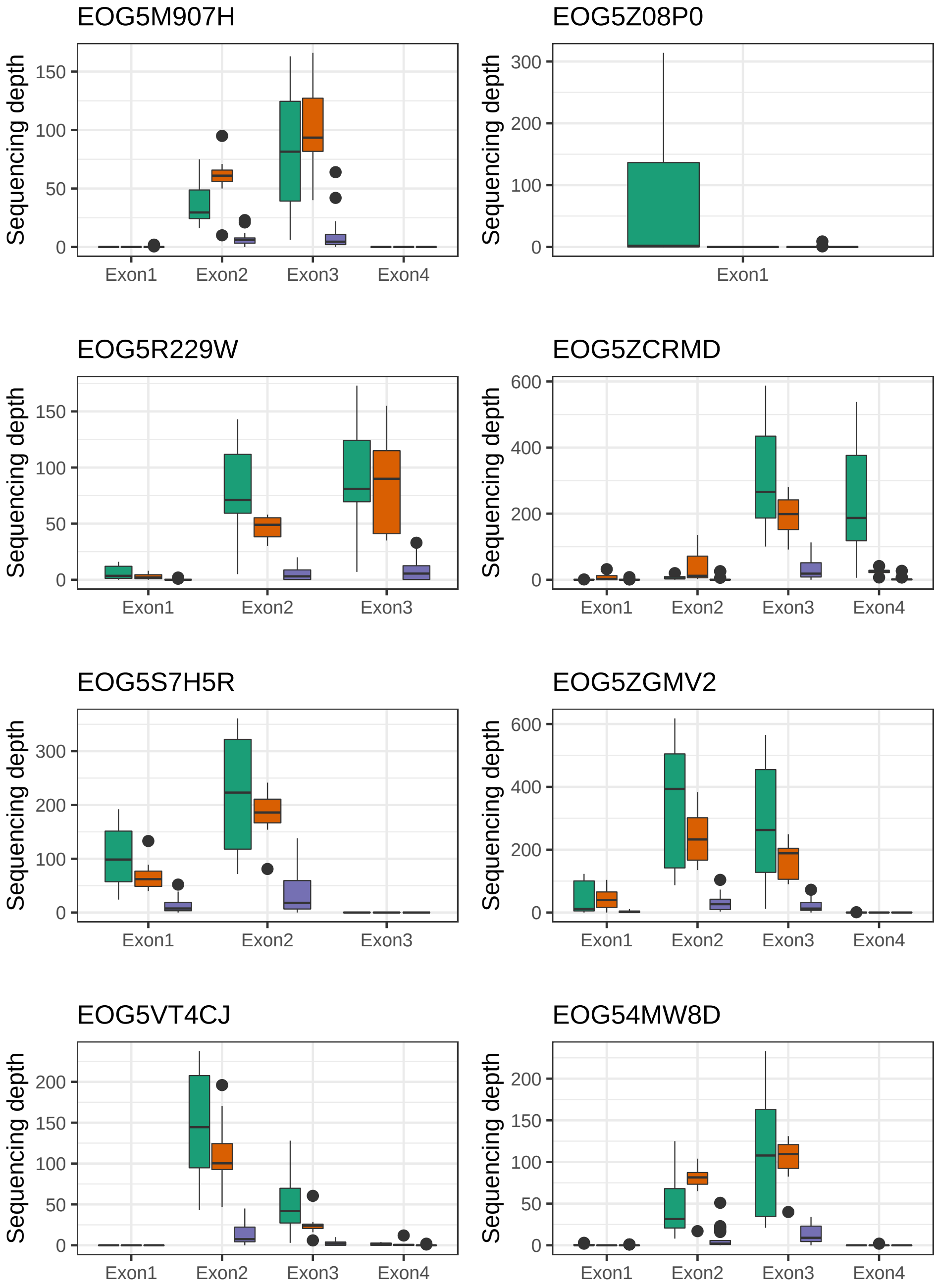
EOG5M907H
EOG5Z08P0
EOG5R229W
EOG5ZCRMD
EOG5S7H5R
EOG5ZGMV2
EOG5VT4CJ
EOG54MW8D
Sequencing depth
Exon1
Exon2
Exon3
Exon4

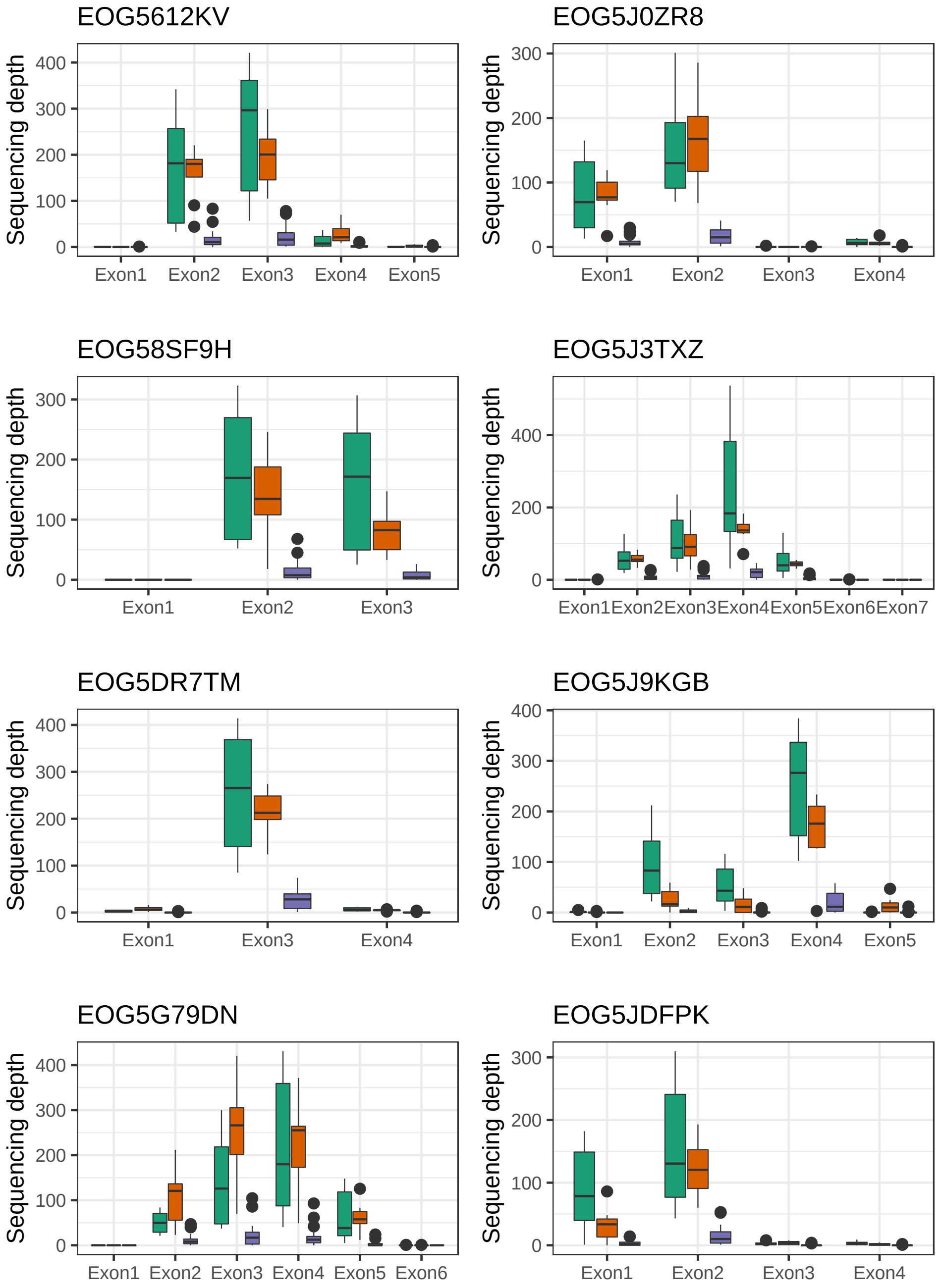
EOG5612KV
Sequencing depth
0
100
200
300
400
Exon1
Exon2
Exon3
Exon4
Exon5
EOG5J0ZR8
Sequencing depth
0
100
200
300
Exon1
Exon2
Exon3
Exon4
EOG58SF9H
Sequencing depth
0
100
200
300
Exon1
Exon2
Exon3
EOG5J3TXZ
Sequencing depth
0
200
400
Exon1
Exon2
Exon3
Exon4
Exon5
Exon6
Exon7
EOG5DR7TM
Sequencing depth
0
100
200
300
400
Exon1
Exon3
Exon4
EOG5J9KGB
Sequencing depth
0
100
200
300
400
Exon1
Exon2
Exon3
Exon4
Exon5
EOG5G79DN
Sequencing depth
0
100
200
300
400
Exon1
Exon2
Exon3
Exon4
Exon5
Exon6
EOG5JDFPK
Sequencing depth
0
100
200
300
Exon1
Exon2
Exon3
Exon4

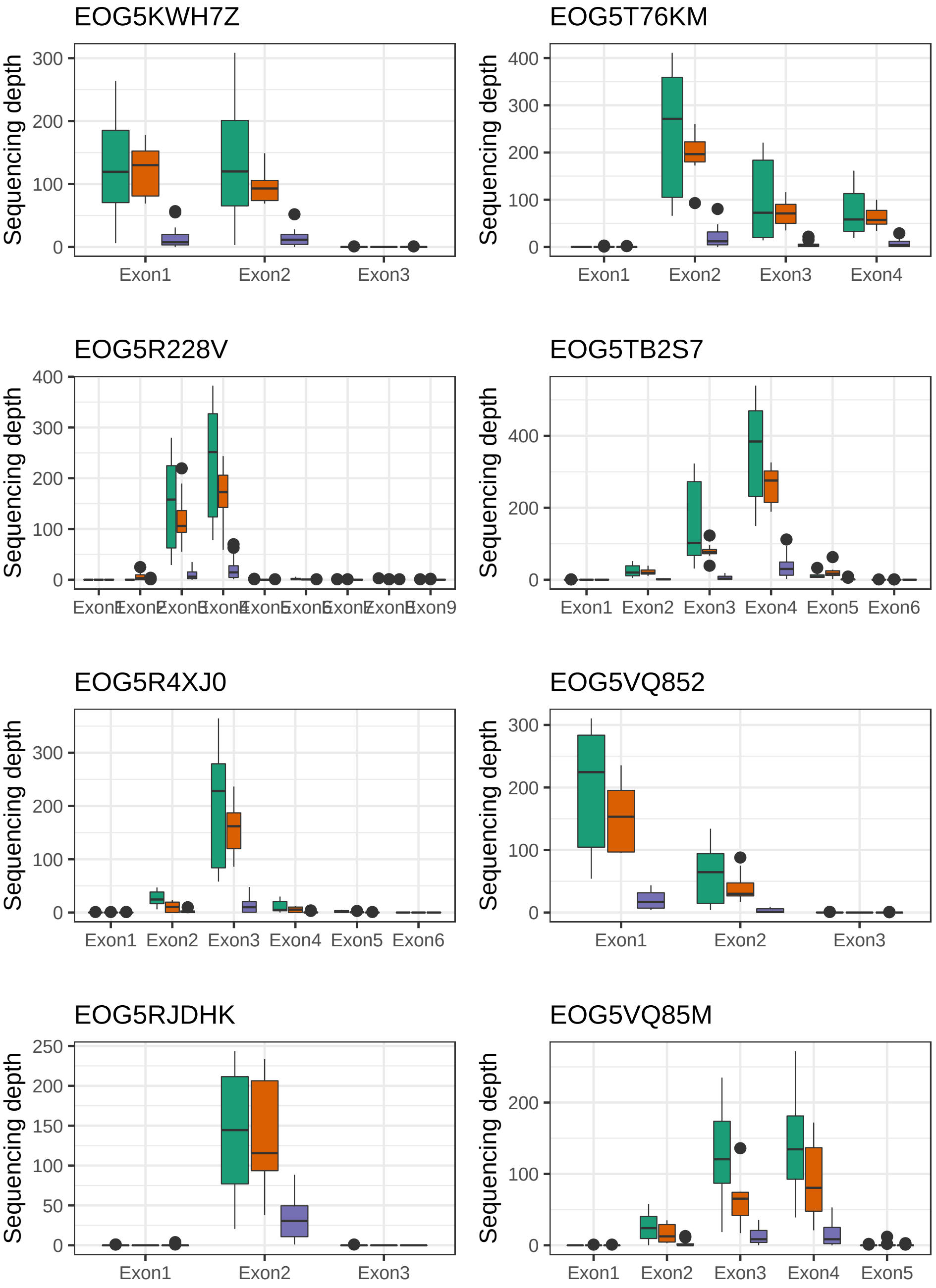
EOG5KWH7Z
Sequencing depth
0
100
200
300
Exon1
Exon2
Exon3
EOG5T76KM
Sequencing depth
0
100
200
300
400
Exon1
Exon2
Exon3
Exon4
EOG5R228V
Sequencing depth
0
100
200
300
400
Exon1
Exon2
Exon3
Exon4
Exon5
Exon6
Exon7
Exon8
Exon9
EOG5TB2S7
Sequencing depth
0
200
400
Exon1
Exon2
Exon3
Exon4
Exon5
Exon6
EOG5R4XJ0
Sequencing depth
0
100
200
300
Exon1
Exon2
Exon3
Exon4
Exon5
Exon6
EOG5VQ852
Sequencing depth
0
100
200
300
Exon1
Exon2
Exon3
EOG5RJDHK
Sequencing depth
0
50
100
150
200
250
Exon1
Exon2
Exon3
EOG5VQ85M
Sequencing depth
0
100
200
Exon1
Exon2
Exon3
Exon4
Exon5

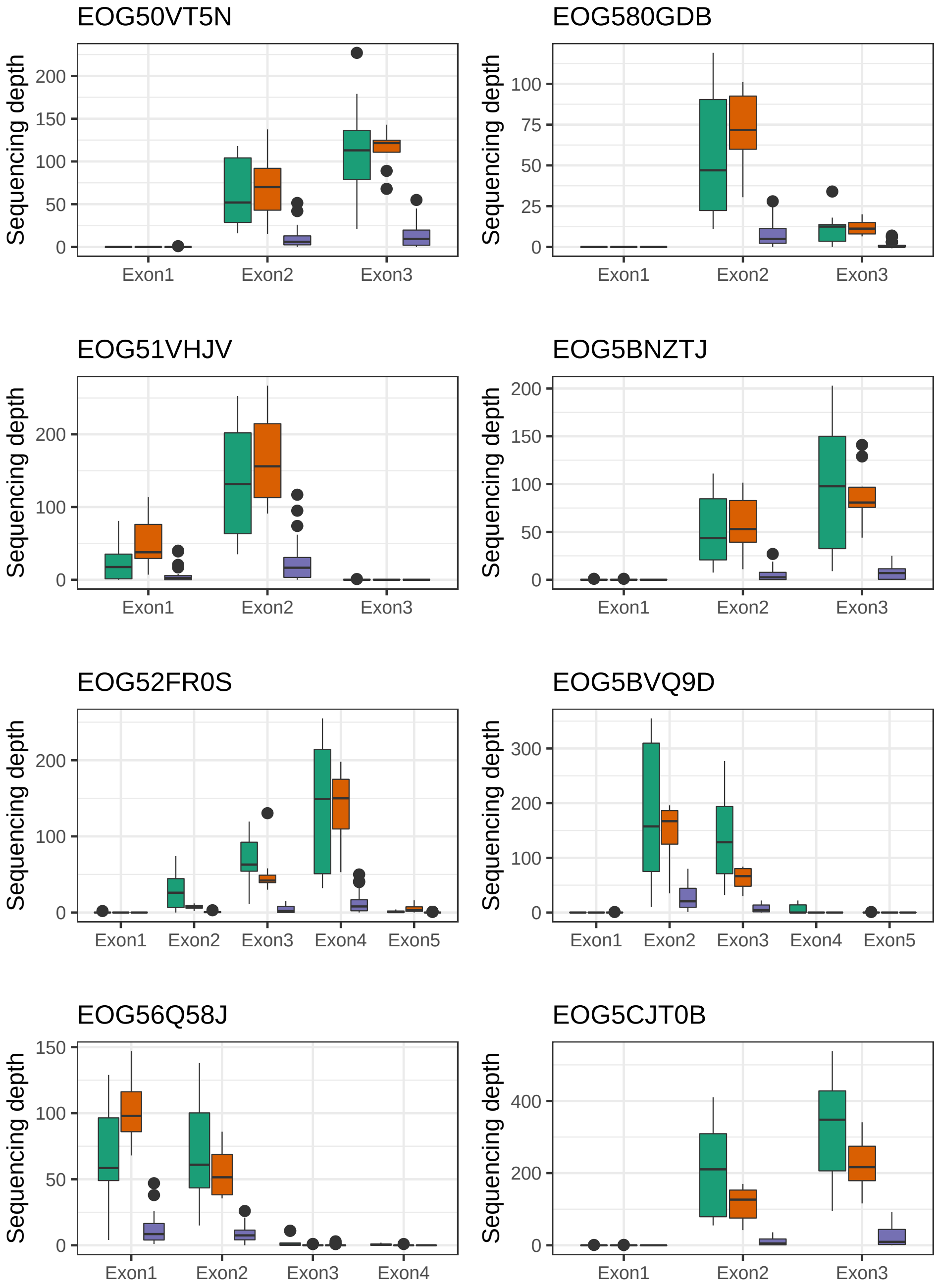
EOG50VT5N
EOG580GDB
EOG51VHJV
EOG5BNZTJ
EOG52FR0S
EOG5BVQ9D
EOG56Q58J
EOG5CJT0B
Sequencing depth
Exon1
Exon2
Exon3
Exon4
Exon5

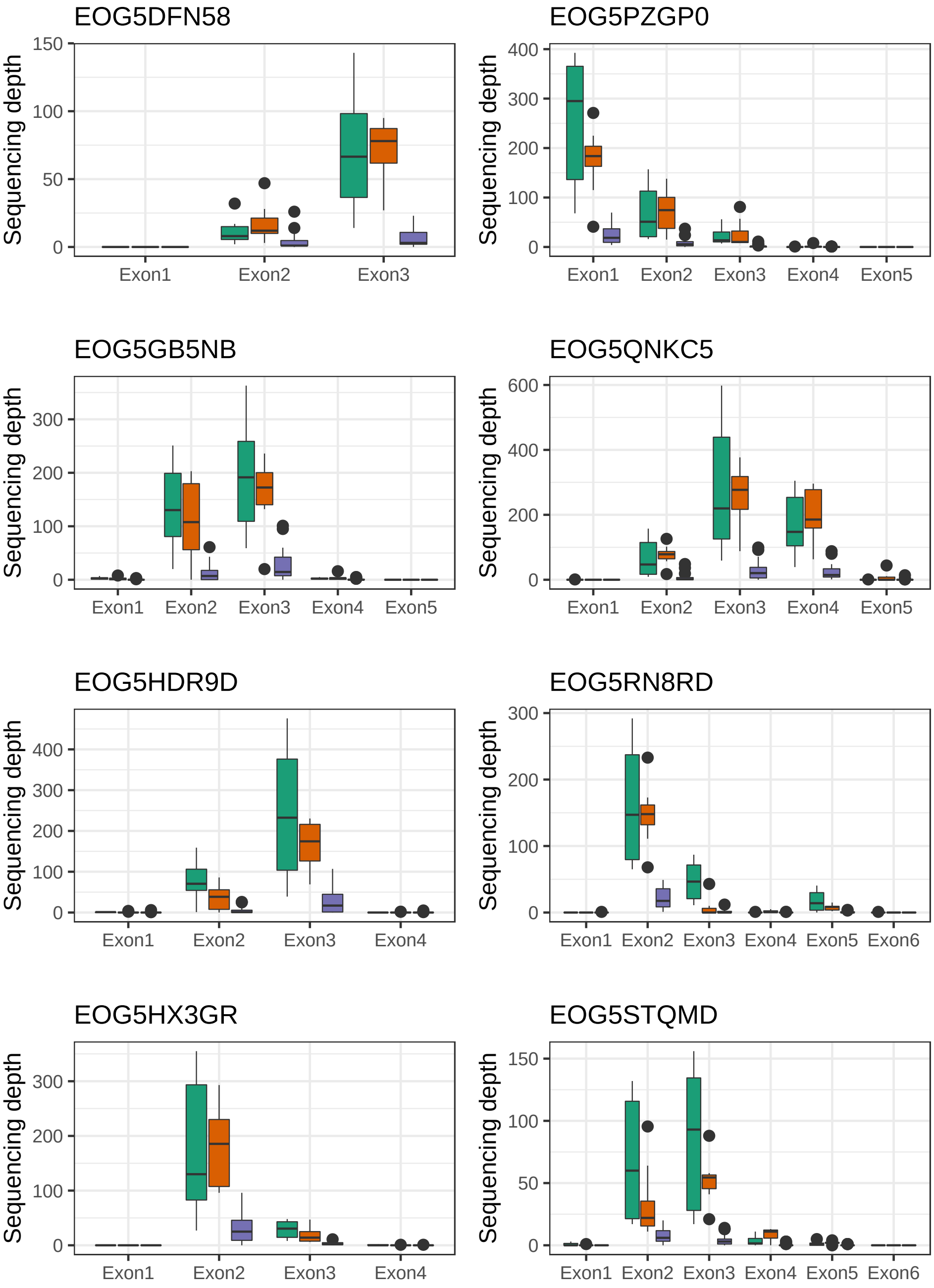
EOG5DFN58
Sequencing depth
0
50
100
150
Exon1
Exon2
Exon3
EOG5PZGP0
Sequencing depth
0
100
200
300
400
Exon1
Exon2
Exon3
Exon4
Exon5
EOG5GB5NB
Sequencing depth
0
100
200
300
Exon1
Exon2
Exon3
Exon4
Exon5
EOG5QNKC5
Sequencing depth
0
200
400
600
Exon1
Exon2
Exon3
Exon4
Exon5
EOG5HDR9D
Sequencing depth
0
100
200
300
400
Exon1
Exon2
Exon3
Exon4
EOG5RN8RD
Sequencing depth
0
100
200
300
Exon1
Exon2
Exon3
Exon4
Exon5
Exon6
EOG5HX3GR
Sequencing depth
0
100
200
300
Exon1
Exon2
Exon3
Exon4
EOG5STQMD
Sequencing depth
0
50
100
150
Exon1
Exon2
Exon3
Exon4
Exon5
Exon6

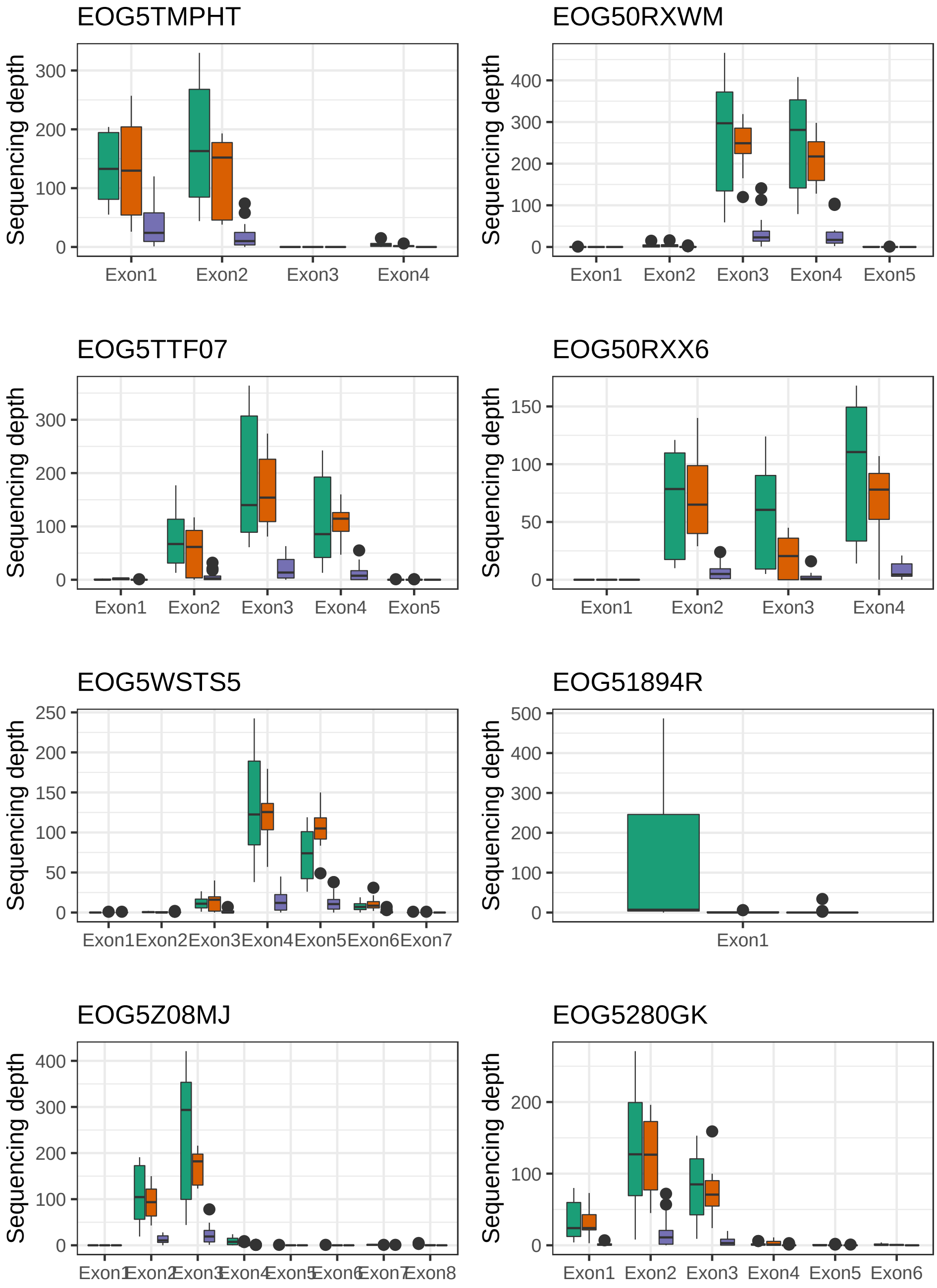

EOG5TMPHT
EOG50RXWM
EOG5TTF07
EOG50RXX6
EOG5WSTS5
EOG51894R
EOG5Z08MJ
EOG5280GK
Sequencing depth
Exon1
Exon2
Exon3
Exon4
Exon5
Exon6
Exon7
Exon8

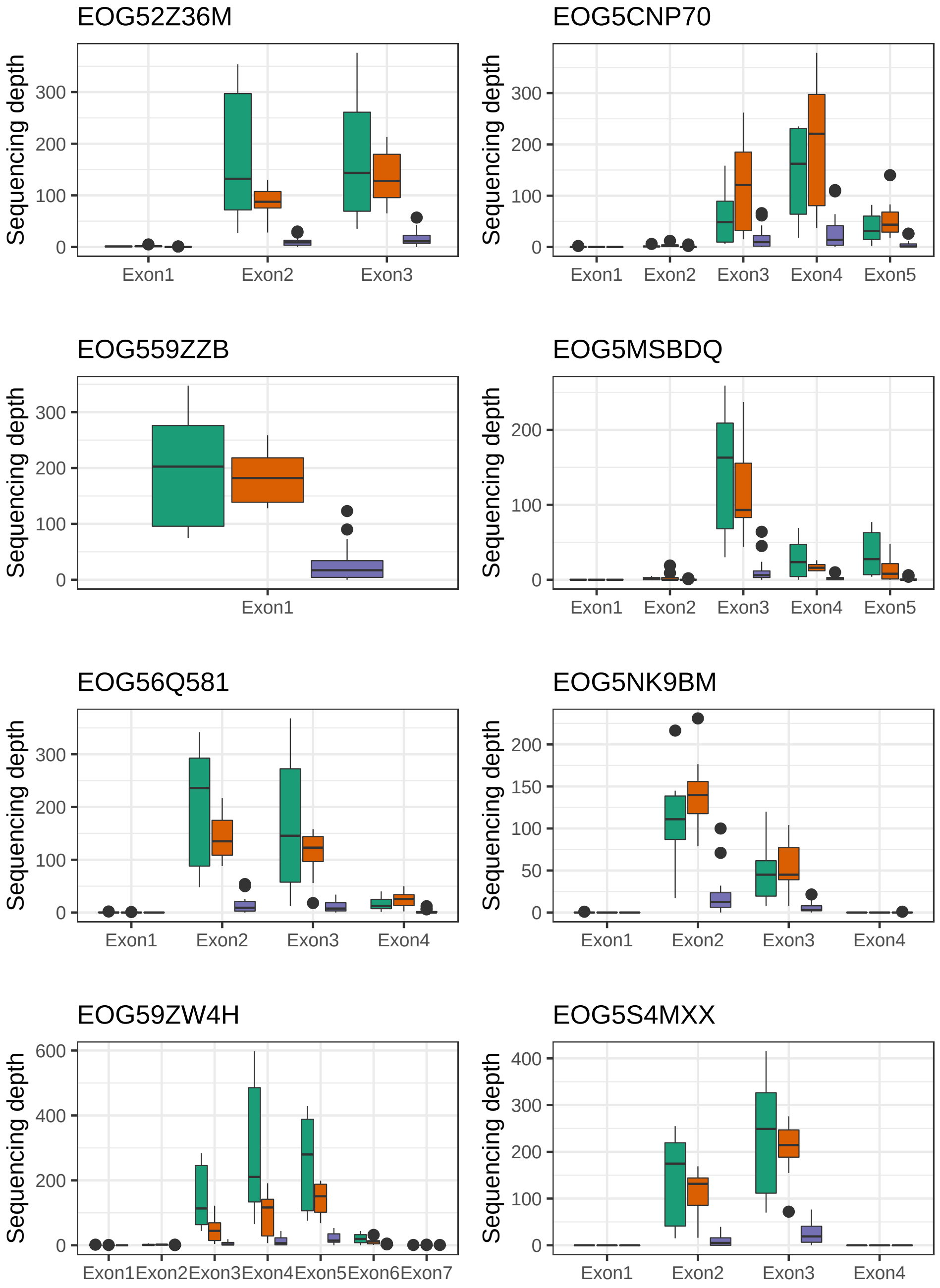

EOG52Z36M
EOG5CNP70
EOG559ZZB
EOG5MSBDQ
EOG56Q581
EOG5NK9BM
EOG59ZW4H
EOG5S4MXX
Sequencing depth
Exon1
Exon2
Exon3
Exon4
Exon5
Exon6
Exon7

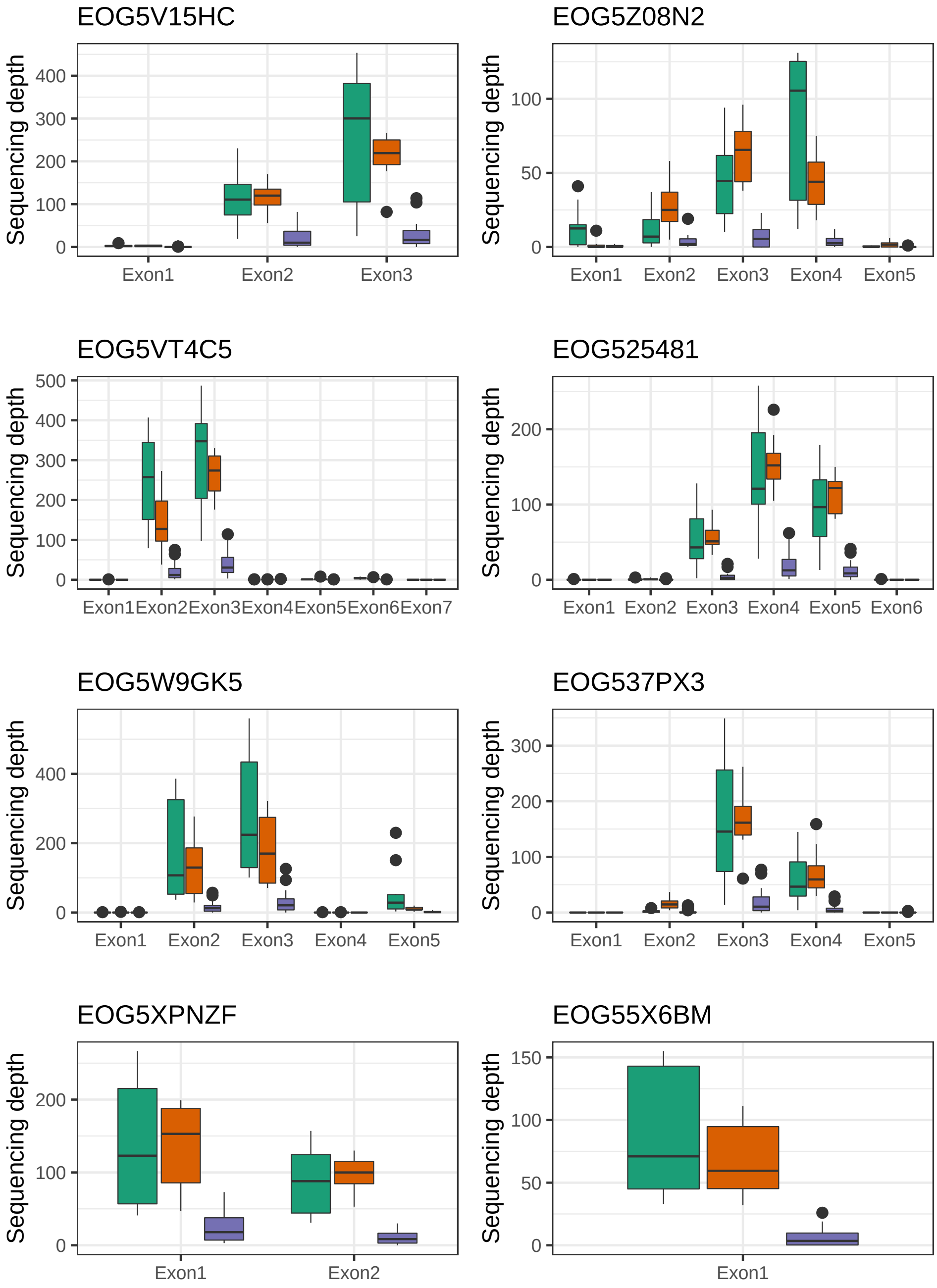
EOG5V15HC
Sequencing depth
0
100
200
300
400
Exon1
Exon2
Exon3
EOG5Z08N2
0
50
100
Exon1
Exon2
Exon3
Exon4
Exon5
EOG5VT4C5
0
100
200
300
400
500
Exon1
Exon2
Exon3
Exon4
Exon5
Exon6
Exon7
EOG525481
0
100
200
Exon1
Exon2
Exon3
Exon4
Exon5
Exon6
EOG5W9GK5
0
200
400
Exon1
Exon2
Exon3
Exon4
Exon5
EOG537PX3
0
100
200
300
Exon1
Exon2
Exon3
Exon4
Exon5
EOG5XPNZF
0
100
200
Exon1
Exon2
EOG55X6BM
0
50
100
150
Exon1

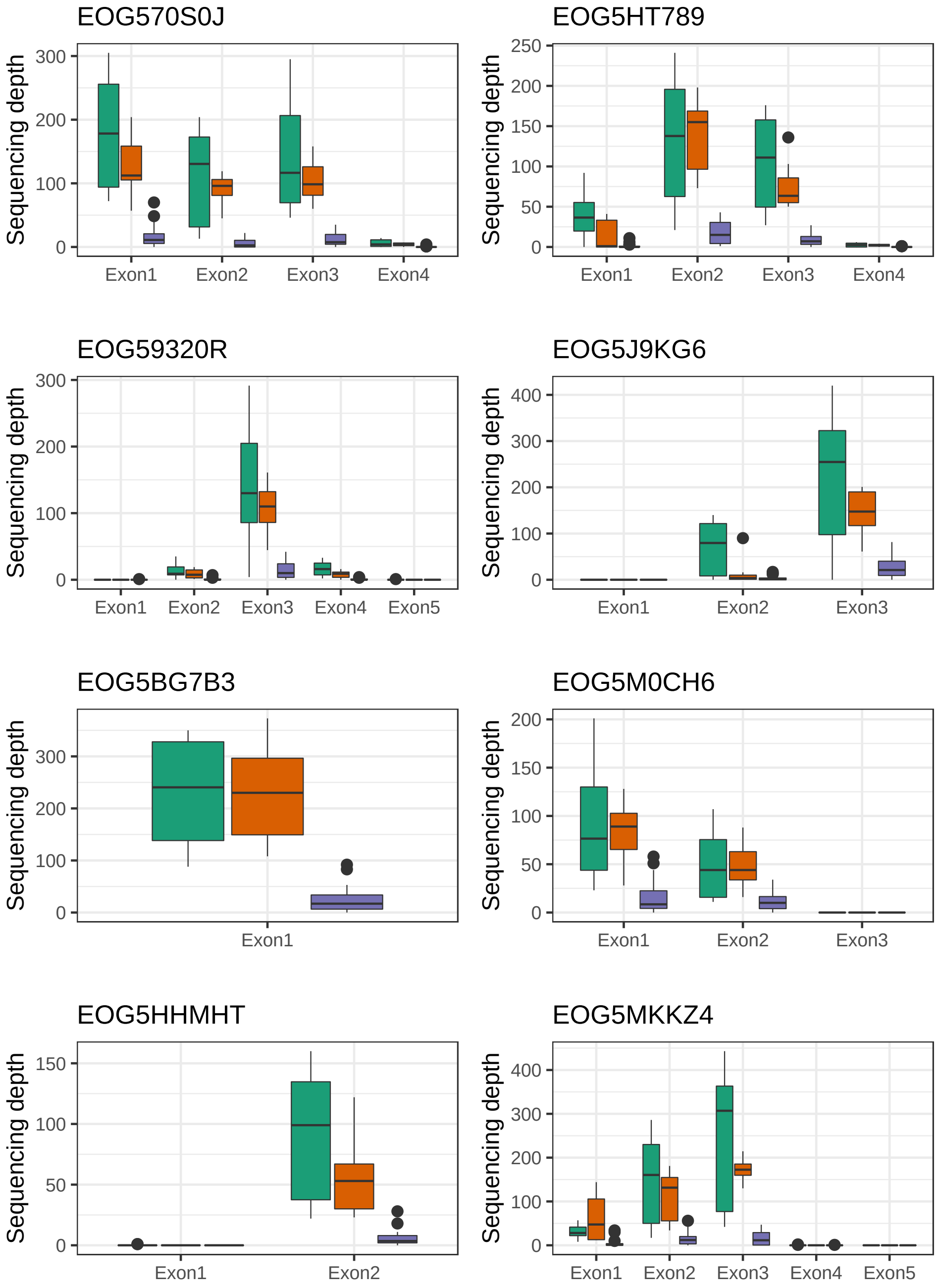

EOG570S0J
EOG5HT789
EOG59320R
EOG5J9KG6
EOG5BG7B3
EOG5M0CH6
EOG5HHMHT
EOG5MKKZ4
Sequencing depth
Exon1
Exon2
Exon3
Exon4
Exon5

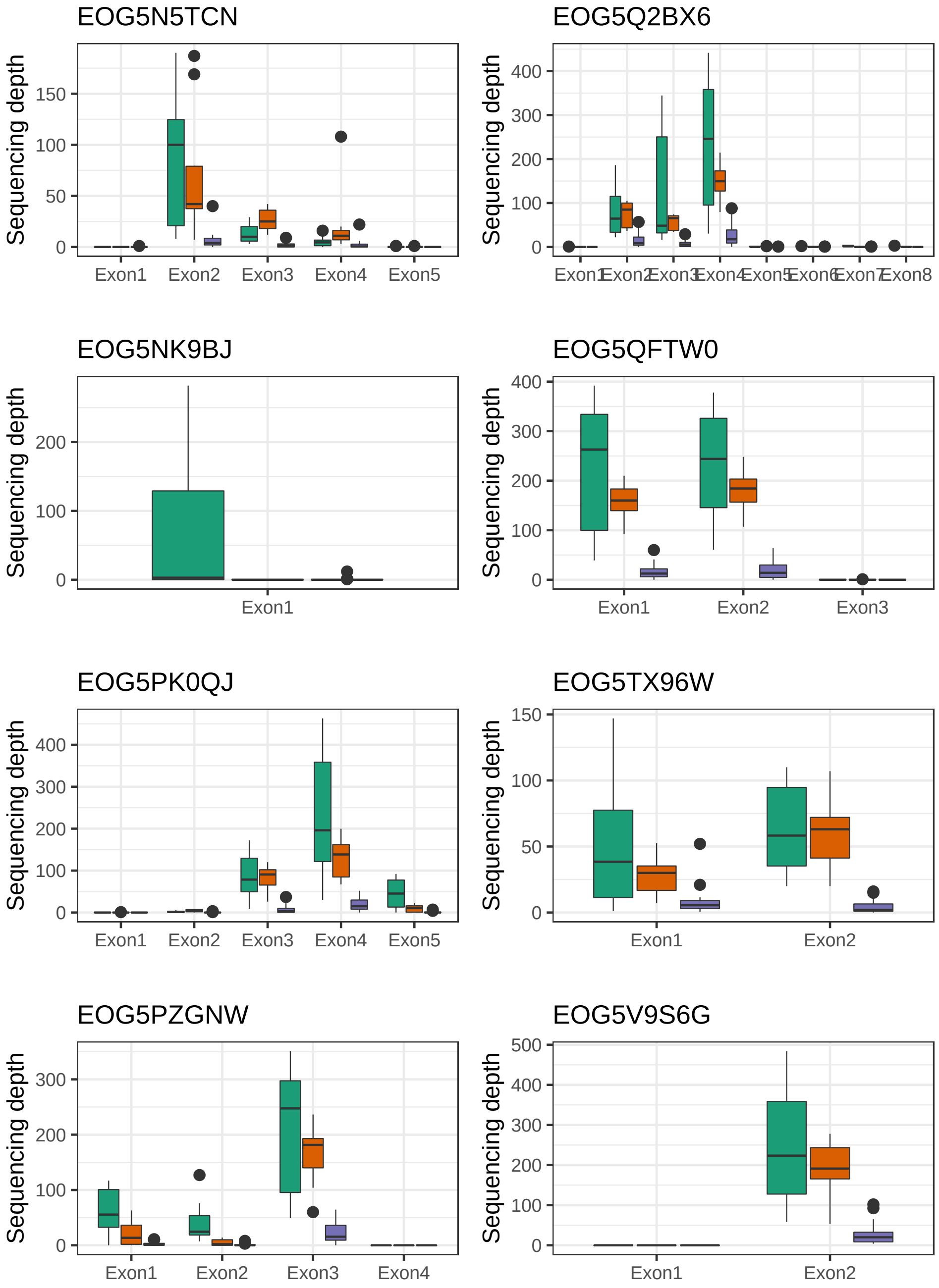
EOG5N5TCN
Sequencing depth
0
50
100
150
Exon1
Exon2
Exon3
Exon4
Exon5
EOG5Q2BX6
Sequencing depth
0
100
200
300
400
Exon1
Exon2
Exon3
Exon4
Exon5
Exon6
Exon7
Exon8
EOG5NK9BJ
Sequencing depth
0
100
200
Exon1
EOG5QFTW0
Sequencing depth
0
100
200
300
400
Exon1
Exon2
Exon3
EOG5PK0QJ
Sequencing depth
0
100
200
300
400
Exon1
Exon2
Exon3
Exon4
Exon5
EOG5TX96W
Sequencing depth
0
50
100
150
Exon1
Exon2
EOG5PZGNW
Sequencing depth
0
100
200
300
Exon1
Exon2
Exon3
Exon4
EOG5V9S6G
Sequencing depth
0
100
200
300
400
500
Exon1
Exon2

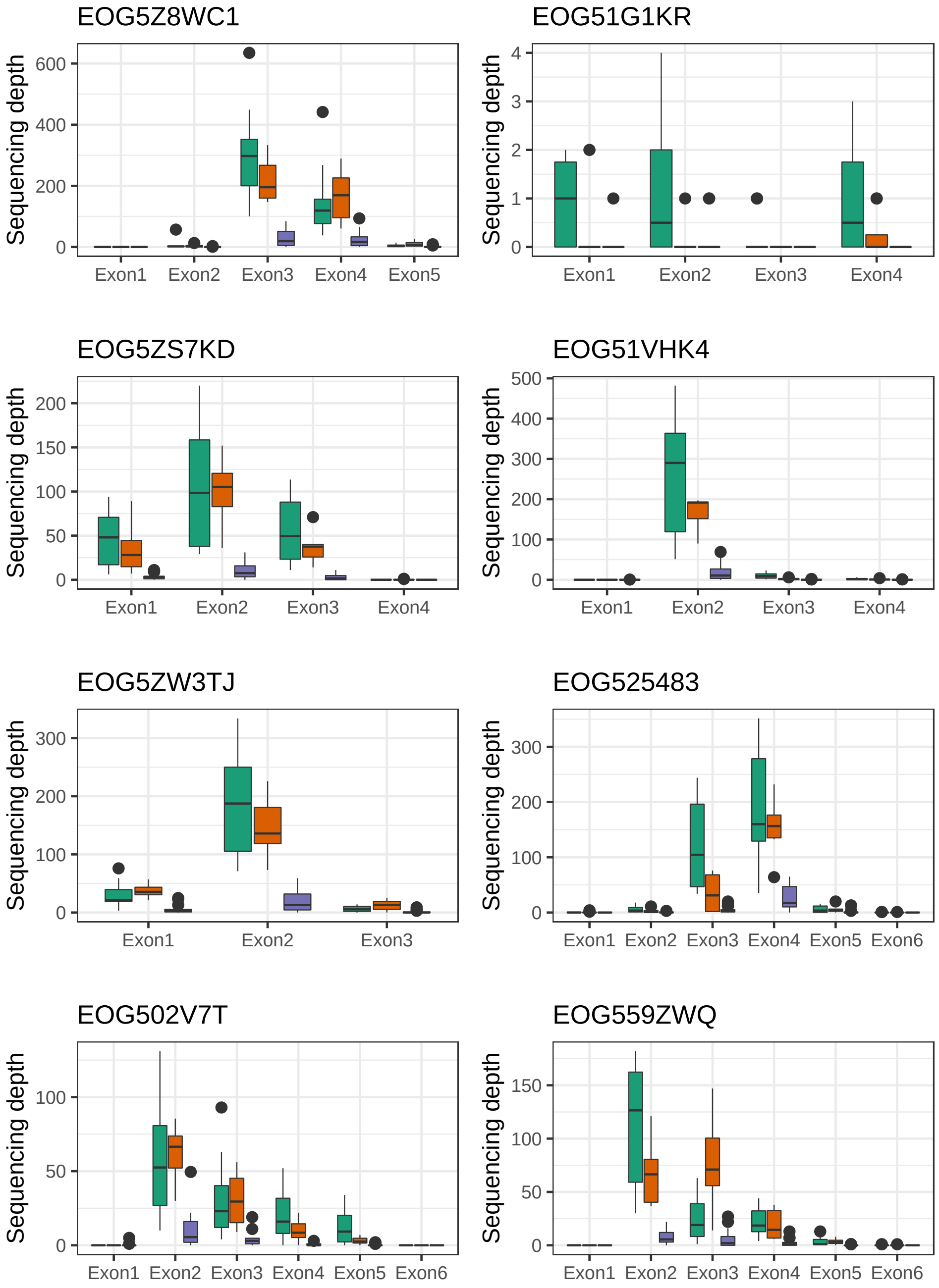
EOG5Z8WC1
EOG51G1KR
EOG5ZS7KD
EOG51VHK4
EOG5ZW3TJ
EOG525483
EOG502V7T
EOG559ZWQ
Sequencing depth
Exon1
Exon2
Exon3
Exon4
Exon5
Exon6
0
50
100
150
200
300
400
500
600
1
2
3
4

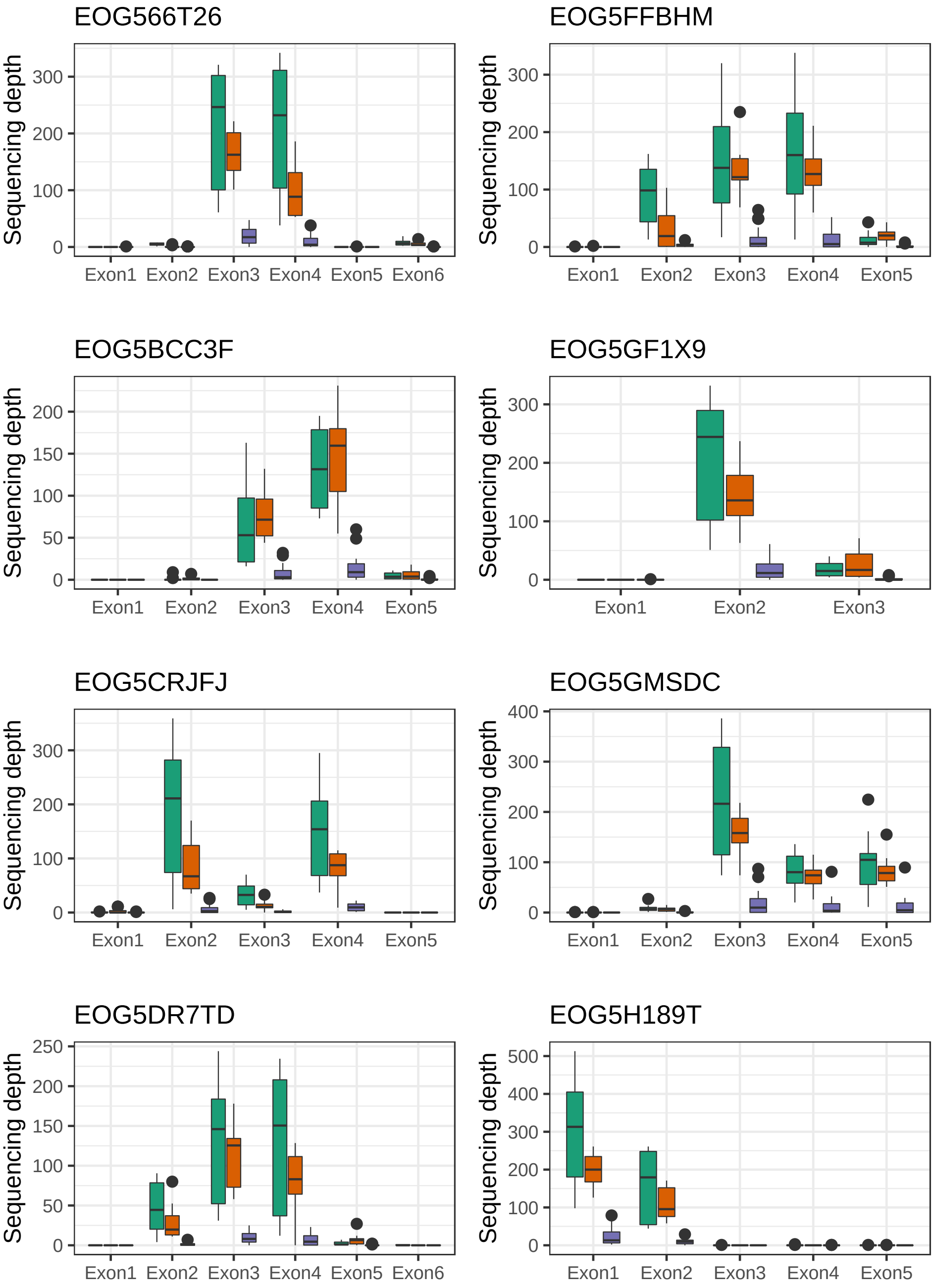
EOG566T26
Sequencing depth
0
100
200
300
Exon1
Exon2
Exon3
Exon4
Exon5
Exon6
EOG5FFBHM
Sequencing depth
0
100
200
300
Exon1
Exon2
Exon3
Exon4
Exon5
EOG5BCC3F
Sequencing depth
0
50
100
150
200
Exon1
Exon2
Exon3
Exon4
Exon5
EOG5GF1X9
Sequencing depth
0
100
200
300
Exon1
Exon2
Exon3
EOG5CRJFJ
Sequencing depth
0
100
200
300
Exon1
Exon2
Exon3
Exon4
Exon5
EOG5GMSDC
Sequencing depth
0
100
200
300
400
Exon1
Exon2
Exon3
Exon4
Exon5
EOG5DR7TD
Sequencing depth
0
50
100
150
200
250
Exon1
Exon2
Exon3
Exon4
Exon5
Exon6
EOG5H189T
Sequencing depth
0
100
200
300
400
500
Exon1
Exon2
Exon3
Exon4
Exon5

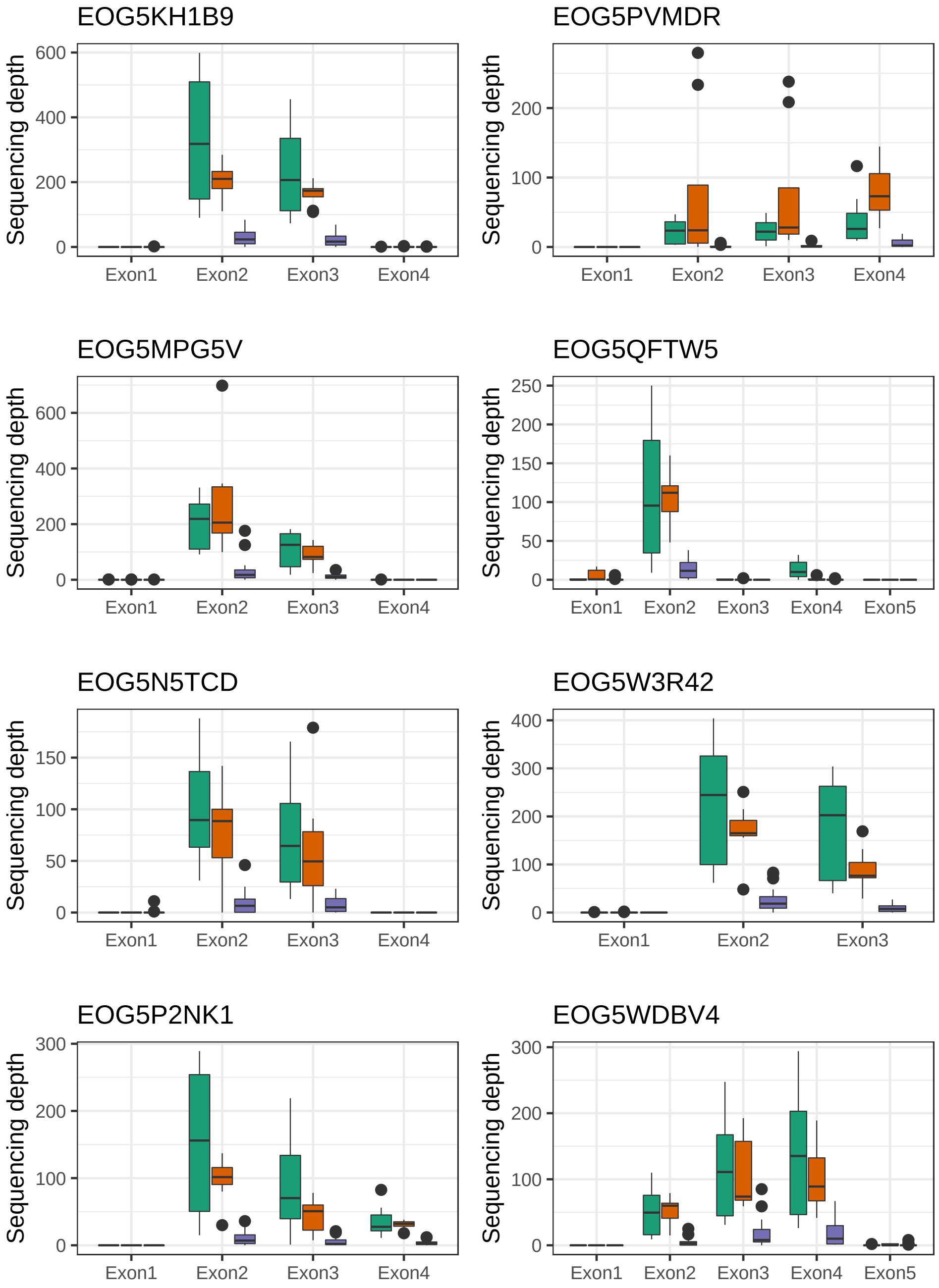
EOG5KH1B9
Sequencing depth
0
200
400
600
Exon1
Exon2
Exon3
Exon4
EOG5PVMDR
Sequencing depth
0
100
200
Exon1
Exon2
Exon3
Exon4
EOG5MPG5V
Sequencing depth
0
200
400
600
Exon1
Exon2
Exon3
Exon4
EOG5QFTW5
Sequencing depth
0
50
100
150
200
250
Exon1
Exon2
Exon3
Exon4
Exon5
EOG5N5TCD
Sequencing depth
0
50
100
150
Exon1
Exon2
Exon3
Exon4
EOG5W3R42
Sequencing depth
0
100
200
300
400
Exon1
Exon2
Exon3
EOG5P2NK1
Sequencing depth
0
100
200
300
Exon1
Exon2
Exon3
Exon4
EOG5WDBV4
Sequencing depth
0
100
200
300
Exon1
Exon2
Exon3
Exon4
Exon5

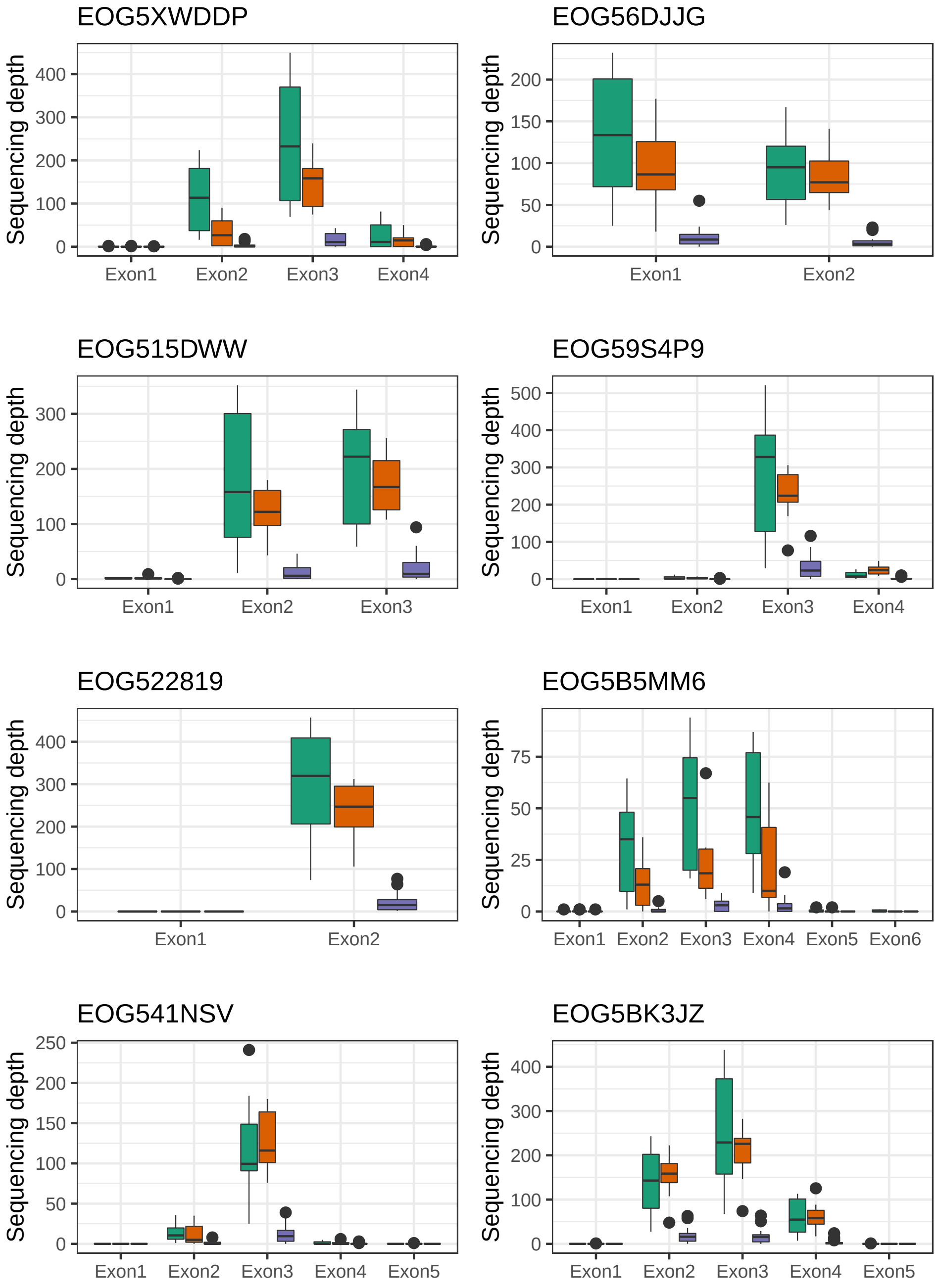
EOG5XWDDP
Sequencing depth
0
100
200
300
400
Exon1
Exon2
Exon3
Exon4
EOG56DJJG
Sequencing depth
0
50
100
150
200
Exon1
Exon2
EOG515DWW
Sequencing depth
0
100
200
300
Exon1
Exon2
Exon3
EOG59S4P9
Sequencing depth
0
100
200
300
400
500
Exon1
Exon2
Exon3
Exon4
EOG522819
Sequencing depth
0
100
200
300
400
Exon1
Exon2
EOG5B5MM6
Sequencing depth
0
25
50
75
Exon1
Exon2
Exon3
Exon4
Exon5
Exon6
EOG541NSV
Sequencing depth
0
50
100
150
200
250
Exon1
Exon2
Exon3
Exon4
Exon5
EOG5BK3JZ
Sequencing depth
0
100
200
300
400
Exon1
Exon2
Exon3
Exon4
Exon5

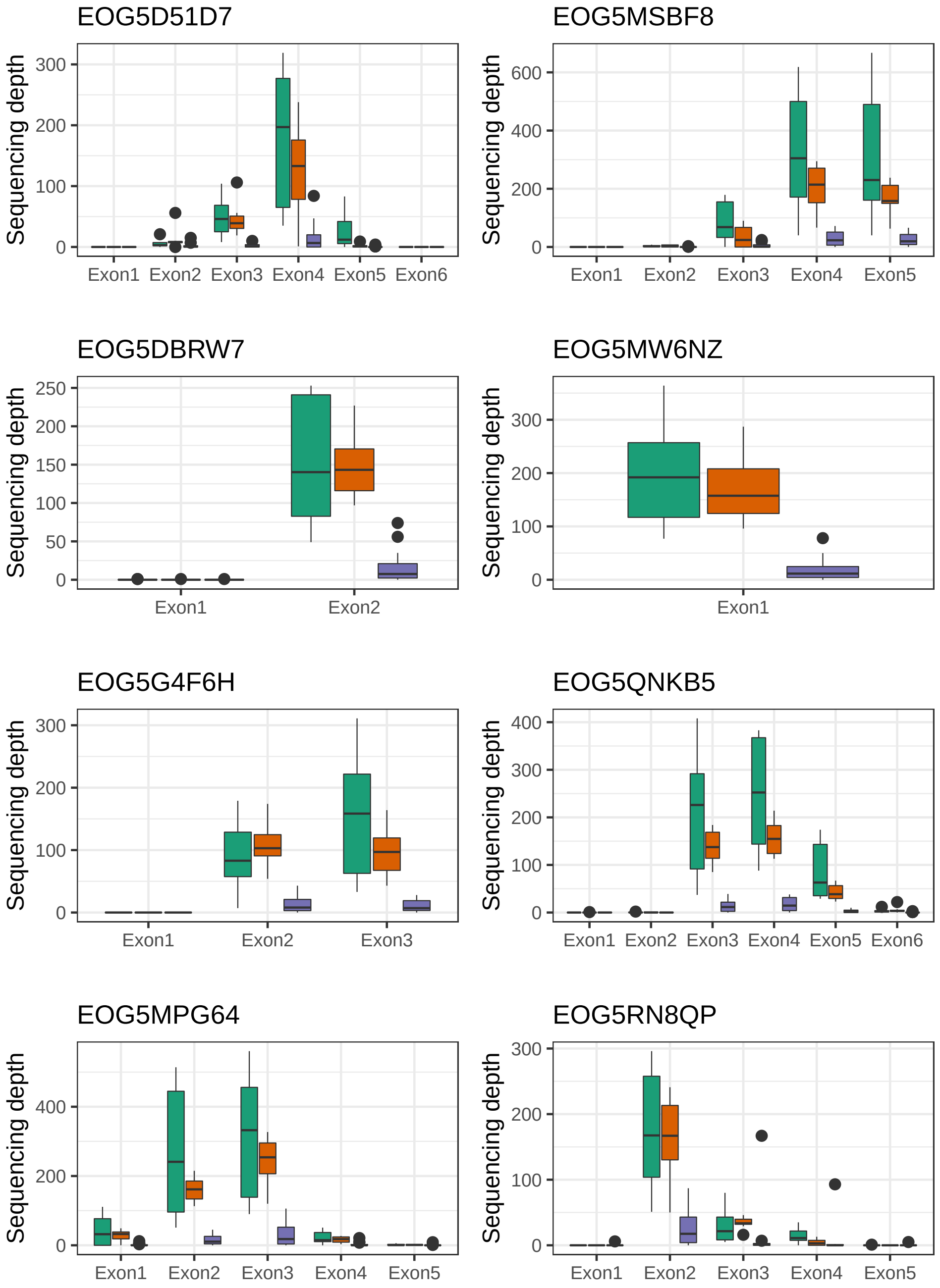
EOG5D51D7
Sequencing depth
Exon1
Exon2
Exon3
Exon4
Exon5
Exon6
EOG5MSBF8
Sequencing depth
Exon1
Exon2
Exon3
Exon4
Exon5
EOG5DBRW7
Sequencing depth
Exon1
Exon2
EOG5MW6NZ
Sequencing depth
Exon1
EOG5G4F6H
Sequencing depth
Exon1
Exon2
Exon3
EOG5QNKB5
Sequencing depth
Exon1
Exon2
Exon3
Exon4
Exon5
Exon6
EOG5MPG64
Sequencing depth
Exon1
Exon2
Exon3
Exon4
Exon5
EOG5RN8QP
Sequencing depth
Exon1
Exon2
Exon3
Exon4
Exon5

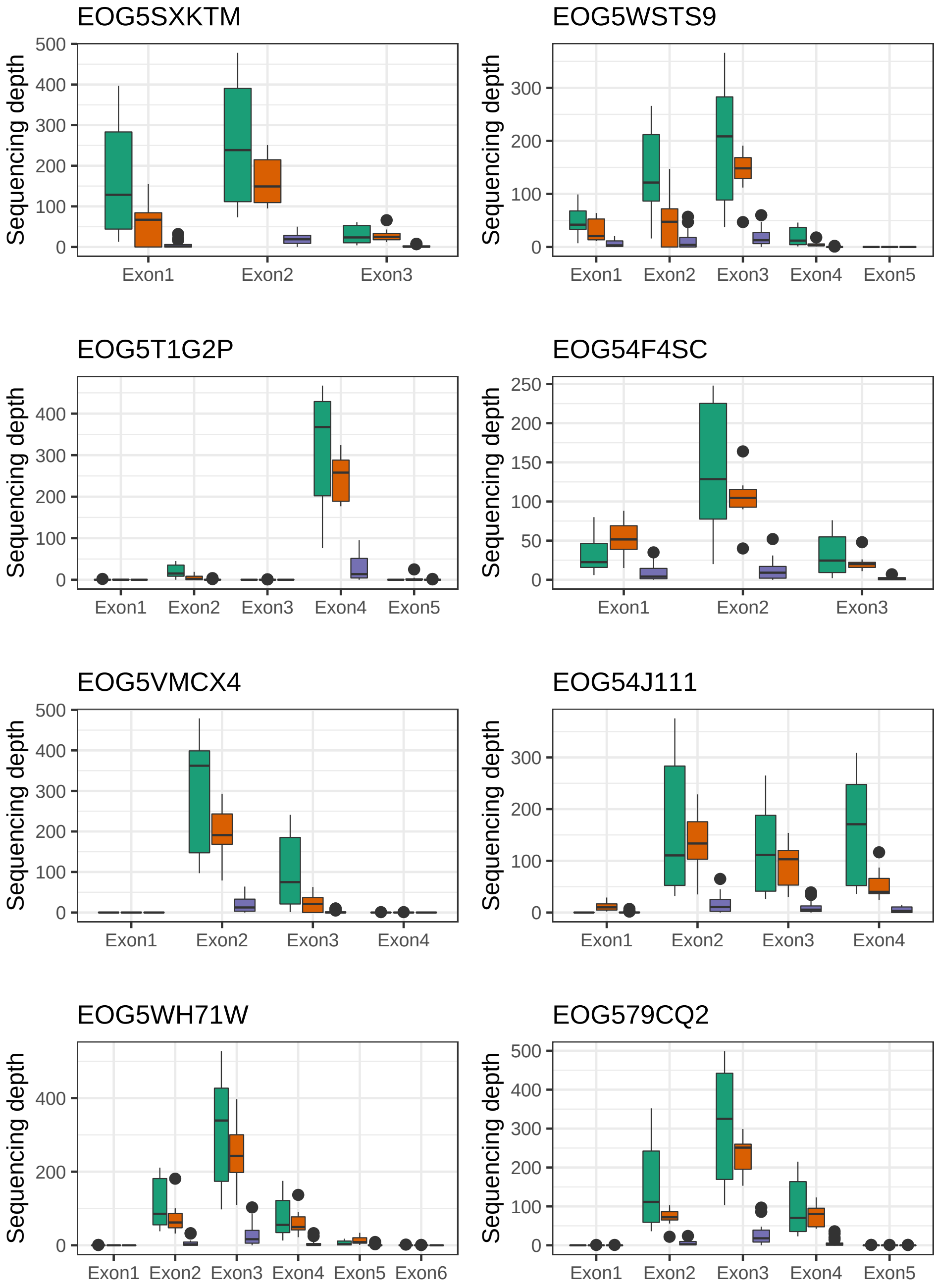
EOG5SXKTM
EOG5WSTS9
EOG5T1G2P
EOG54F4SC
EOG5VMCX4
EOG54J111
EOG5WH71W
EOG579CQ2
Sequencing depth
Exon1
Exon2
Exon3
Exon4
Exon5
Exon6

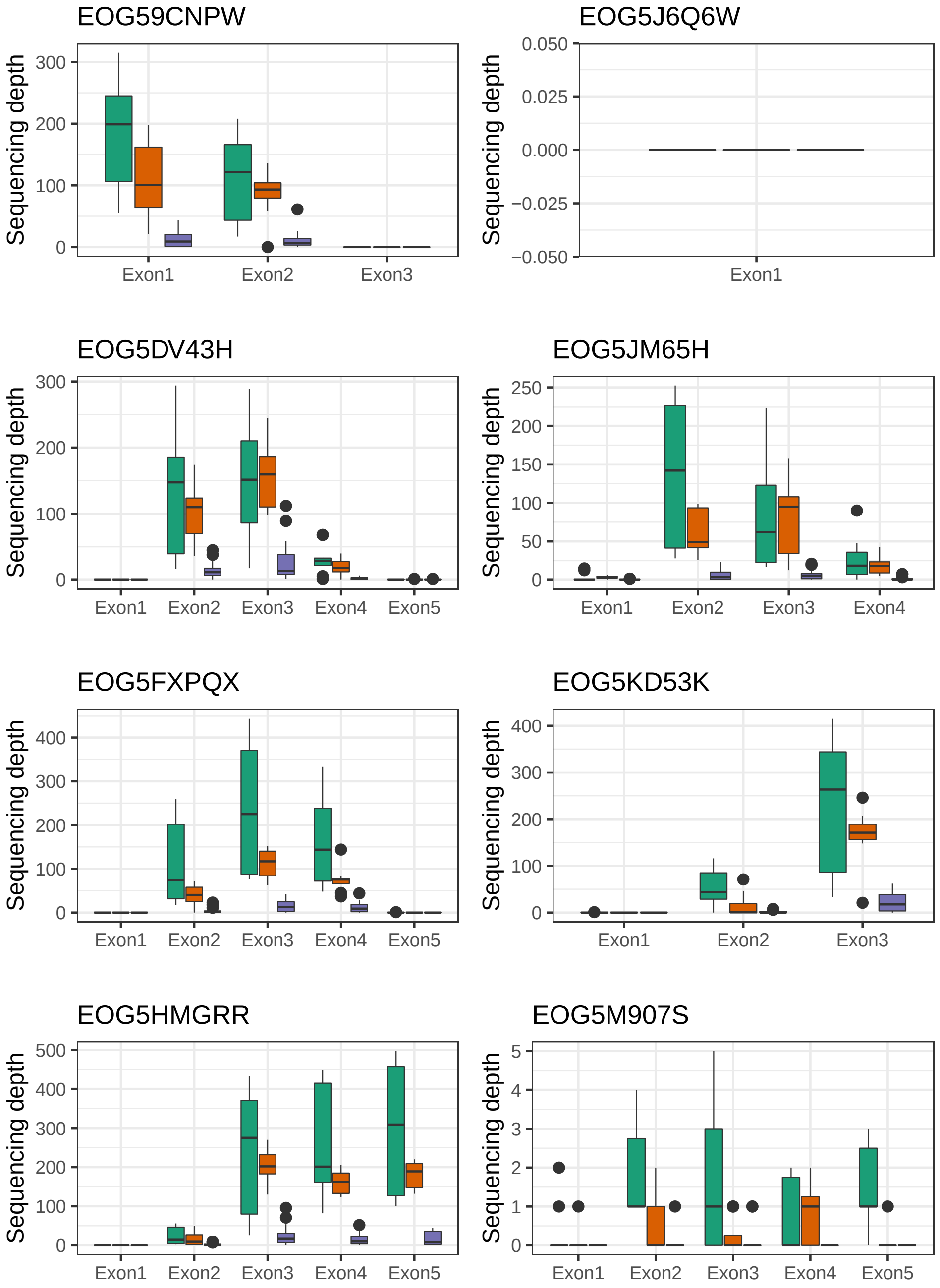
EOG59CNPW
Sequencing depth
0
100
200
300
Exon1
Exon2
Exon3
EOG5J6Q6W
Sequencing depth
0.050
0.025
0.000
−0.025
−0.050
Exon1
EOG5DV43H
Sequencing depth
0
100
200
300
Exon1
Exon2
Exon3
Exon4
Exon5
EOG5JM65H
Sequencing depth
0
50
100
150
200
250
Exon1
Exon2
Exon3
Exon4
EOG5FXPQX
Sequencing depth
0
100
200
300
400
Exon1
Exon2
Exon3
Exon4
Exon5
EOG5KD53K
Sequencing depth
0
100
200
300
400
Exon1
Exon2
Exon3
EOG5HMGRR
Sequencing depth
0
100
200
300
400
500
Exon1
Exon2
Exon3
Exon4
Exon5
EOG5M907S
Sequencing depth
0
1
2
3
4
5
Exon1
Exon2
Exon3
Exon4
Exon5

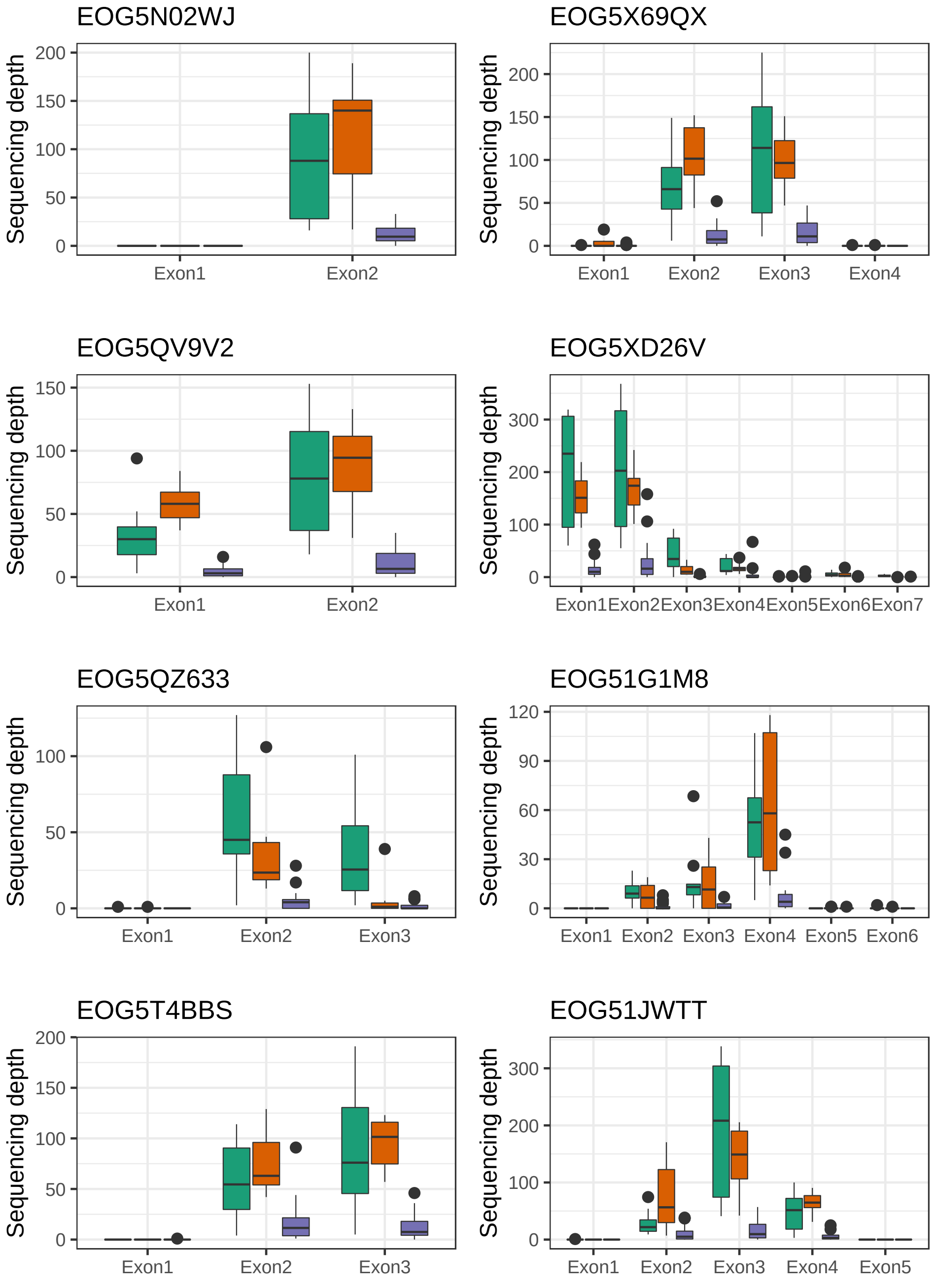

EOG5N02WJ
Sequencing depth
0
50
100
150
200
Exon1
Exon2
EOG5X69QX
Sequencing depth
0
50
100
150
200
Exon1
Exon2
Exon3
Exon4
EOG5QV9V2
Sequencing depth
0
50
100
150
Exon1
Exon2
EOG5XD26V
Sequencing depth
0
100
200
300
Exon1
Exon2
Exon3
Exon4
Exon5
Exon6
Exon7
EOG5QZ633
Sequencing depth
0
50
100
Exon1
Exon2
Exon3
EOG51G1M8
Sequencing depth
0
30
60
90
120
Exon1
Exon2
Exon3
Exon4
Exon5
Exon6
EOG5T4BBS
Sequencing depth
0
50
100
150
200
Exon1
Exon2
Exon3
EOG51JWTT
Sequencing depth
0
100
200
300
Exon1
Exon2
Exon3
Exon4
Exon5

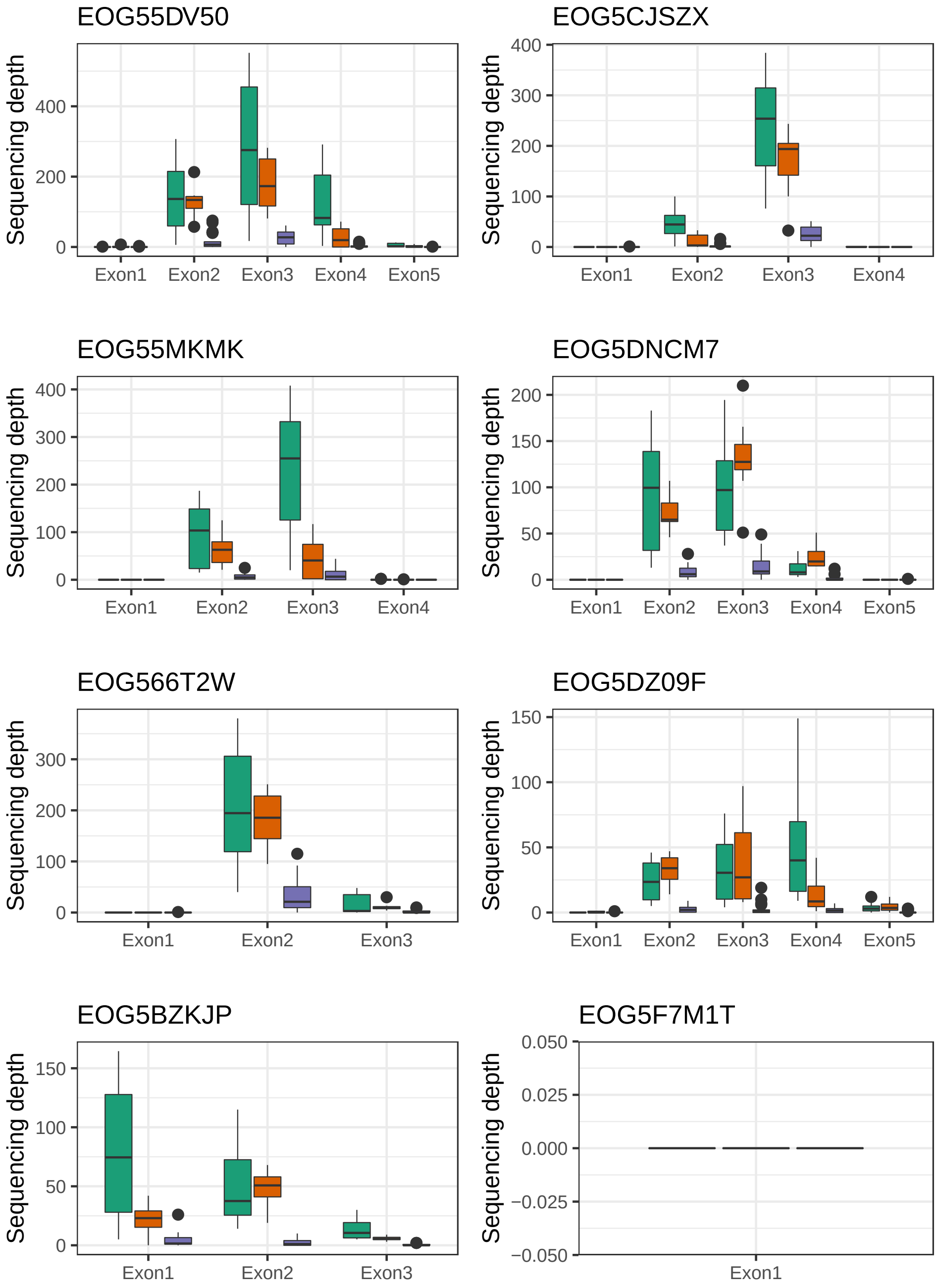

EOG55DV50
EOG5CJSZX
EOG55MKMK
EOG5DNCM7
EOG566T2W
EOG5DZ09F
EOG5BZKJP
EOG5F7M1T
Sequencing depth
Exon1
Exon2
Exon3
Exon4
Exon5

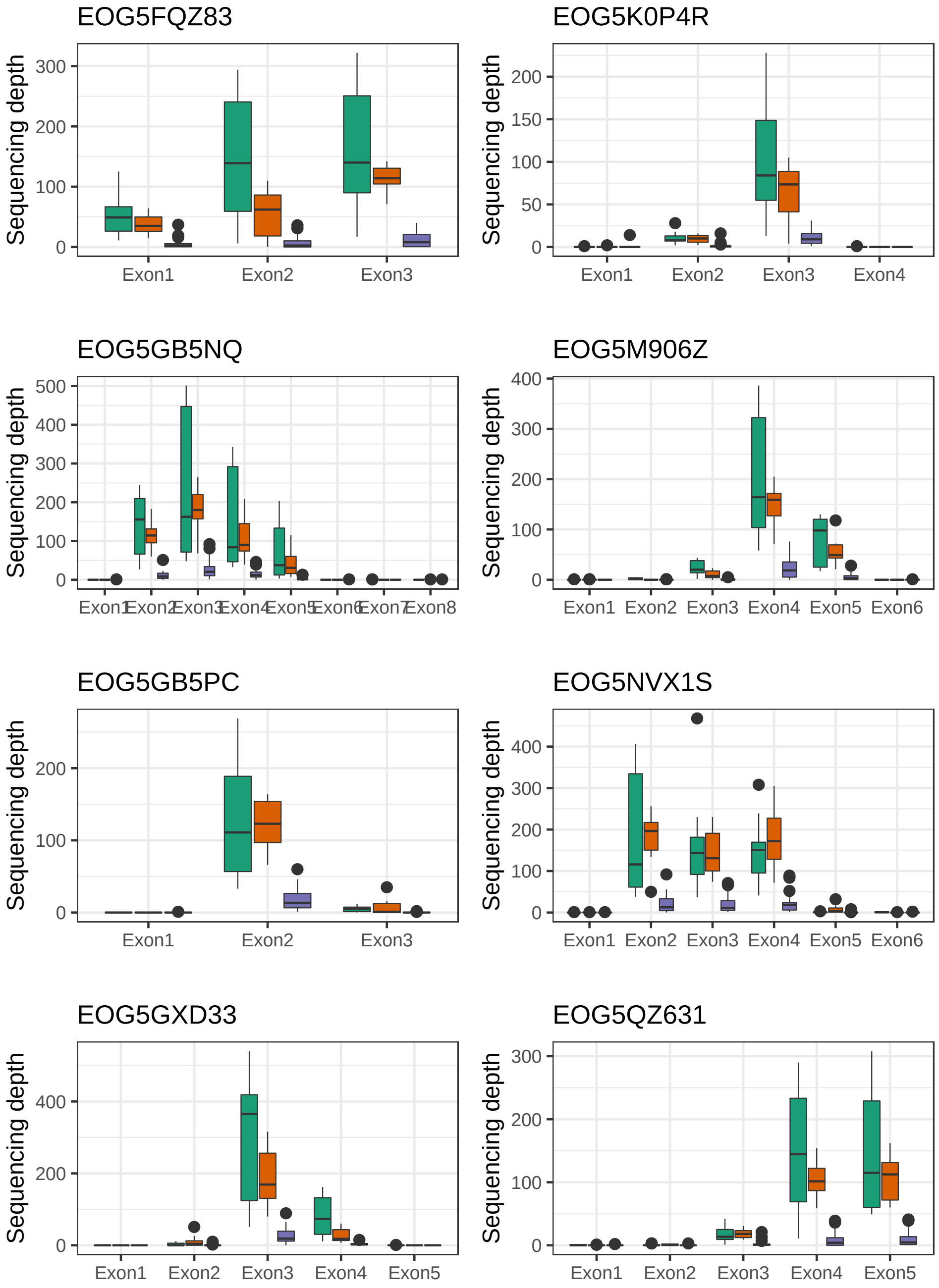
EOG5FQZ83
Sequencing depth
0
100
200
300
Exon1
Exon2
Exon3
EOG5K0P4R
Sequencing depth
0
50
100
150
200
Exon1
Exon2
Exon3
Exon4
EOG5GB5NQ
Sequencing depth
0
100
200
300
400
500
Exon1
Exon2
Exon3
Exon4
Exon5
Exon6
Exon7
Exon8
EOG5M906Z
Sequencing depth
0
100
200
300
400
Exon1
Exon2
Exon3
Exon4
Exon5
Exon6
EOG5GB5PC
Sequencing depth
0
100
200
Exon1
Exon2
Exon3
EOG5NVX1S
Sequencing depth
0
100
200
300
400
Exon1
Exon2
Exon3
Exon4
Exon5
Exon6
EOG5GXD33
Sequencing depth
0
200
400
Exon1
Exon2
Exon3
Exon4
Exon5
EOG5QZ631
Sequencing depth
0
100
200
300
Exon1
Exon2
Exon3
Exon4
Exon5

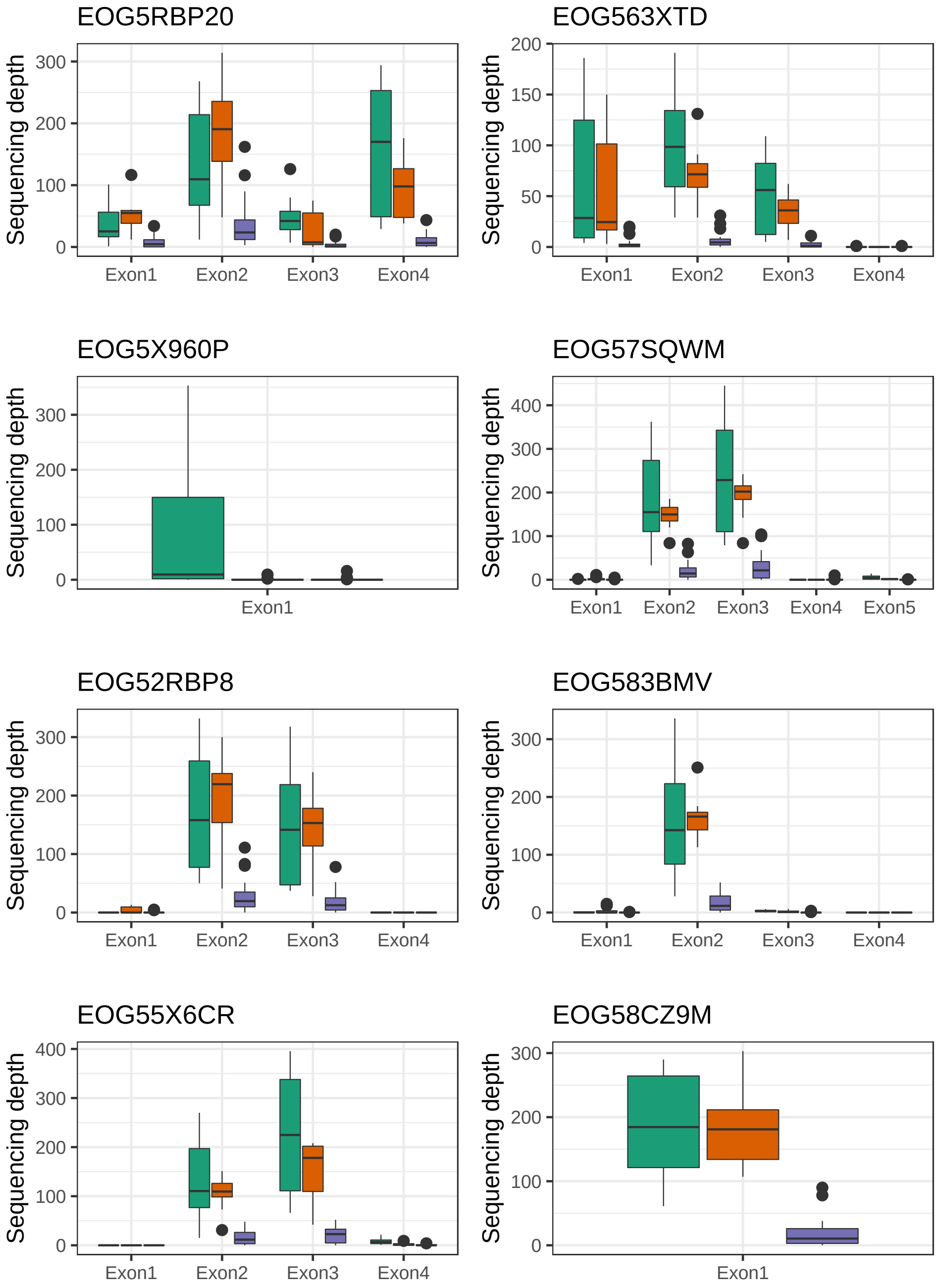
EOG5RBP20
EOG563XTD
EOG5X960P
EOG57SQWM
EOG52RBP8
EOG583BMV
EOG55X6CR
EOG58CZ9M
Sequencing depth
Exon1
Exon2
Exon3
Exon4
Exon5
0
50
100
150
200
300
400

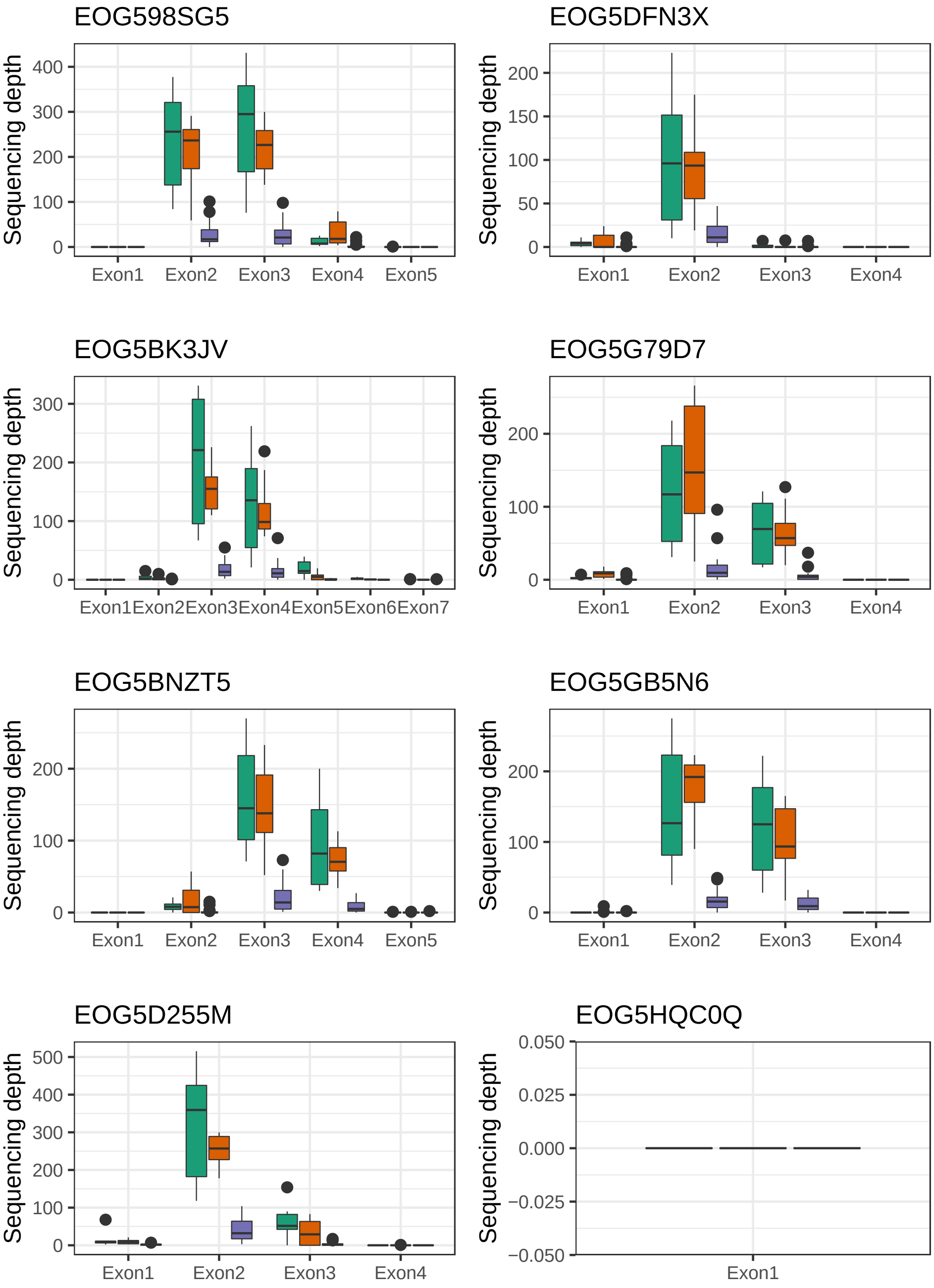
EOG598SG5
Sequencing depth
Exon1
Exon2
Exon3
Exon4
Exon5
EOG5DFN3X
Sequencing depth
Exon1
Exon2
Exon3
Exon4
EOG5BK3JV
Sequencing depth
Exon1
Exon2
Exon3
Exon4
Exon5
Exon6
Exon7
EOG5G79D7
Sequencing depth
Exon1
Exon2
Exon3
Exon4
EOG5BNZT5
Sequencing depth
Exon1
Exon2
Exon3
Exon4
Exon5
EOG5GB5N6
Sequencing depth
Exon1
Exon2
Exon3
Exon4
EOG5D255M
Sequencing depth
Exon1
Exon2
Exon3
Exon4
EOG5HQC0Q
Sequencing depth
Exon1

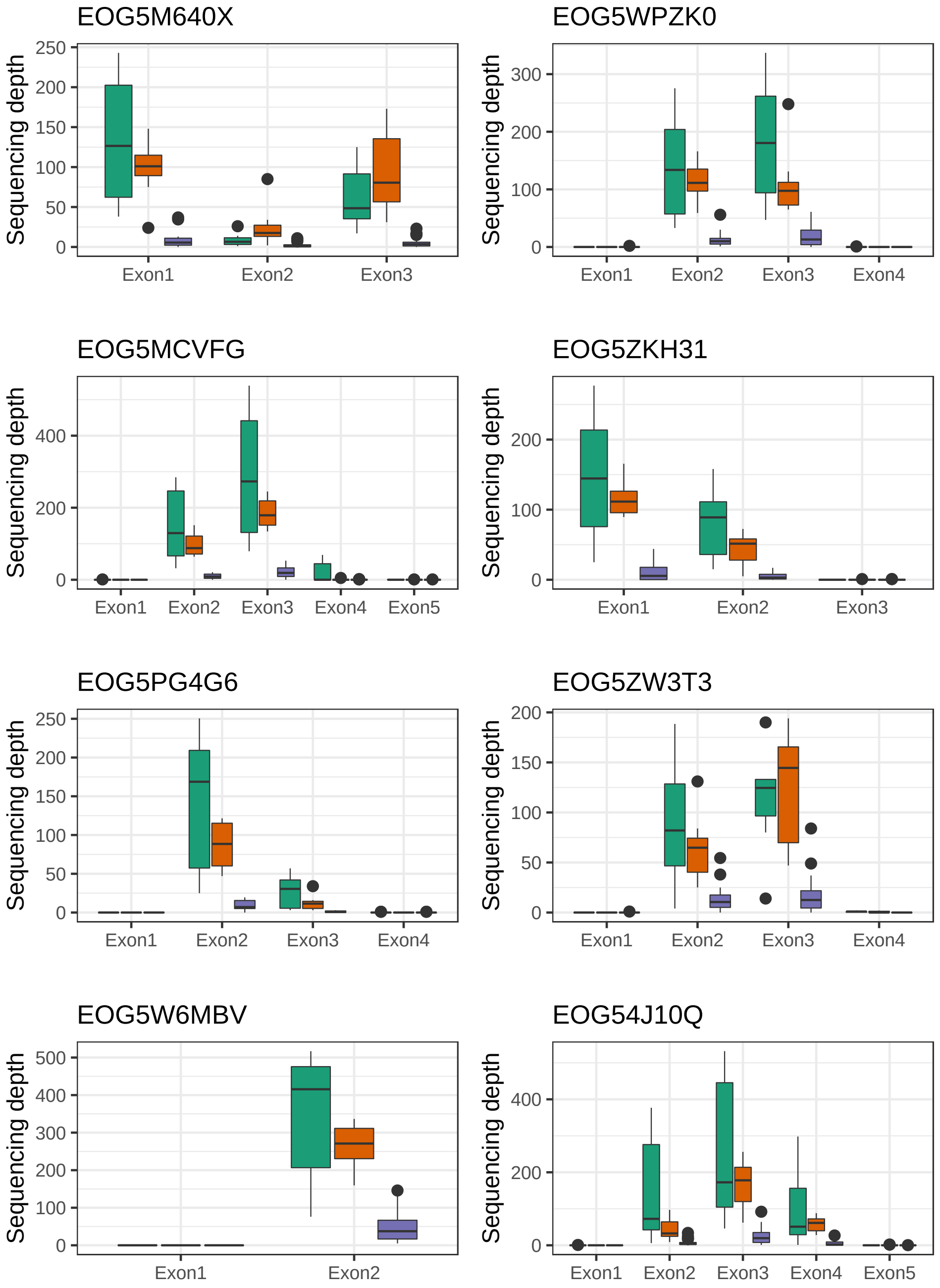
EOG5M640X
EOG5WPZK0
EOG5MCVFG
EOG5ZKH31
EOG5PG4G6
EOG5ZW3T3
EOG5W6MBV
EOG54J10Q
Sequencing depth
Exon1
Exon2
Exon3
Exon4
Exon5

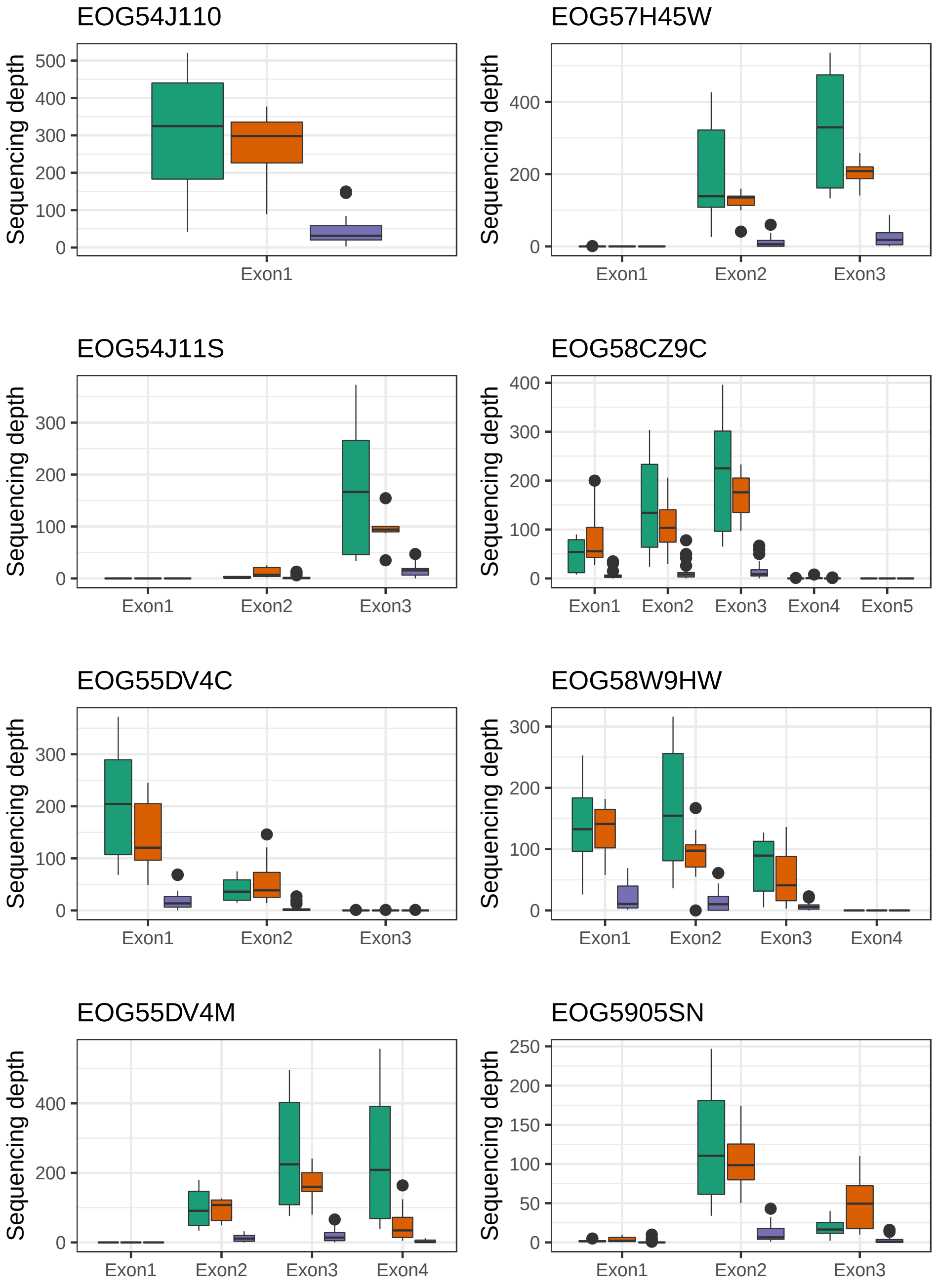
EOG54J110
Sequencing depth
0 100 200 300 400 500
Exon1
EOG57H45W
Sequencing depth
0 200 400
Exon1 Exon2 Exon3
EOG54J11S
Sequencing depth
0 100 200 300
Exon1 Exon2 Exon3
EOG58CZ9C
Sequencing depth
0 100 200 300 400
Exon1 Exon2 Exon3 Exon4 Exon5
EOG55DV4C
Sequencing depth
0 100 200 300
Exon1 Exon2 Exon3
EOG58W9HW
Sequencing depth
0 100 200 300
Exon1 Exon2 Exon3 Exon4
EOG55DV4M
Sequencing depth
0 200 400
Exon1 Exon2 Exon3 Exon4
EOG5905SN
Sequencing depth
0 50 100 150 200 250
Exon1 Exon2 Exon3

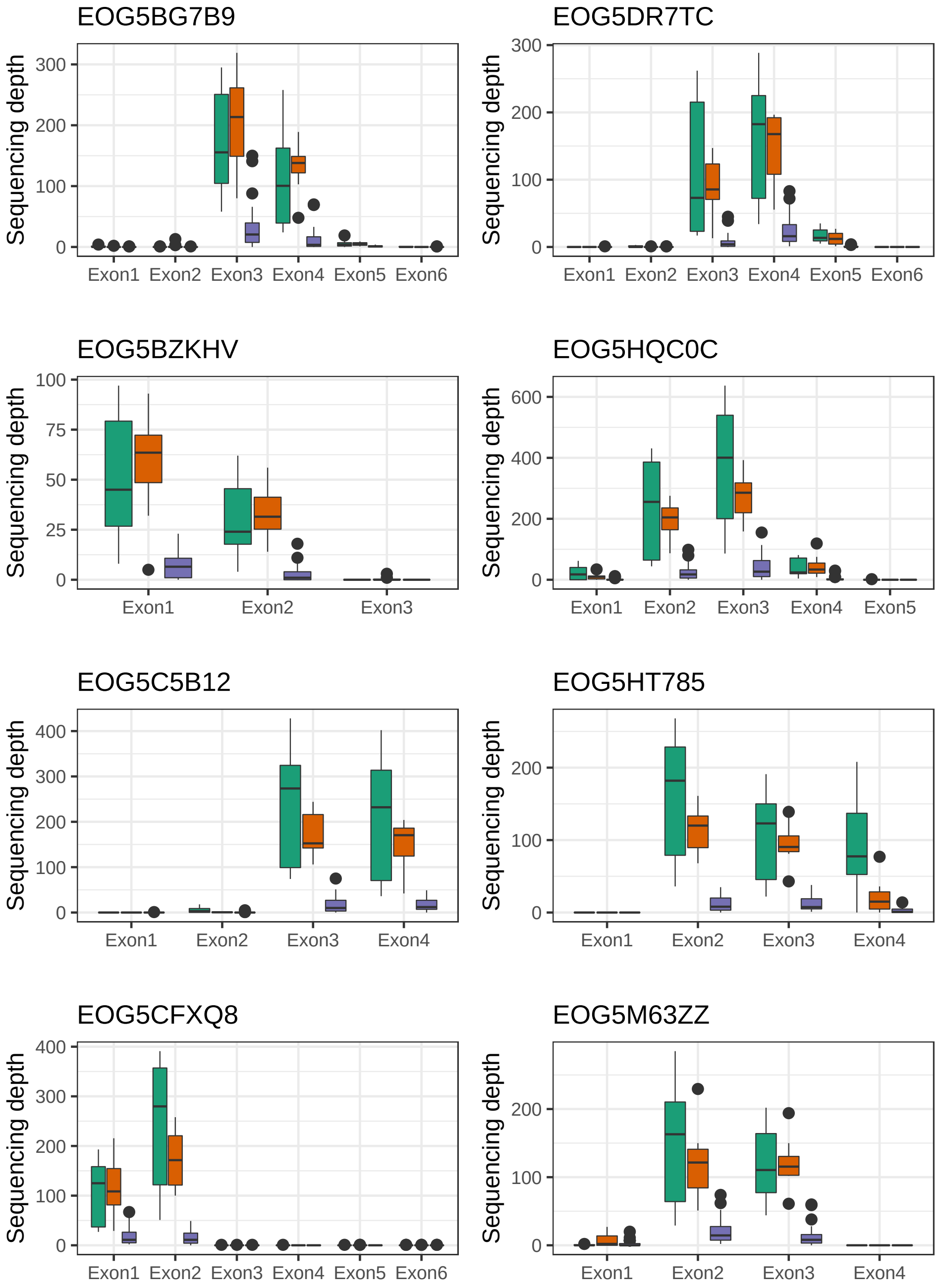
EOG5BG7B9
Sequencing depth
0
100
200
300
Exon1
Exon2
Exon3
Exon4
Exon5
Exon6
EOG5DR7TC
Sequencing depth
0
100
200
300
Exon1
Exon2
Exon3
Exon4
Exon5
Exon6
EOG5BZKHV
Sequencing depth
0
25
50
75
100
Exon1
Exon2
Exon3
EOG5HQC0C
Sequencing depth
0
200
400
600
Exon1
Exon2
Exon3
Exon4
Exon5
EOG5C5B12
Sequencing depth
0
100
200
300
400
Exon1
Exon2
Exon3
Exon4
EOG5HT785
Sequencing depth
0
100
200
Exon1
Exon2
Exon3
Exon4
EOG5CFXQ8
Sequencing depth
0
100
200
300
400
Exon1
Exon2
Exon3
Exon4
Exon5
Exon6
EOG5M63ZZ
Sequencing depth
0
100
200
Exon1
Exon2
Exon3
Exon4

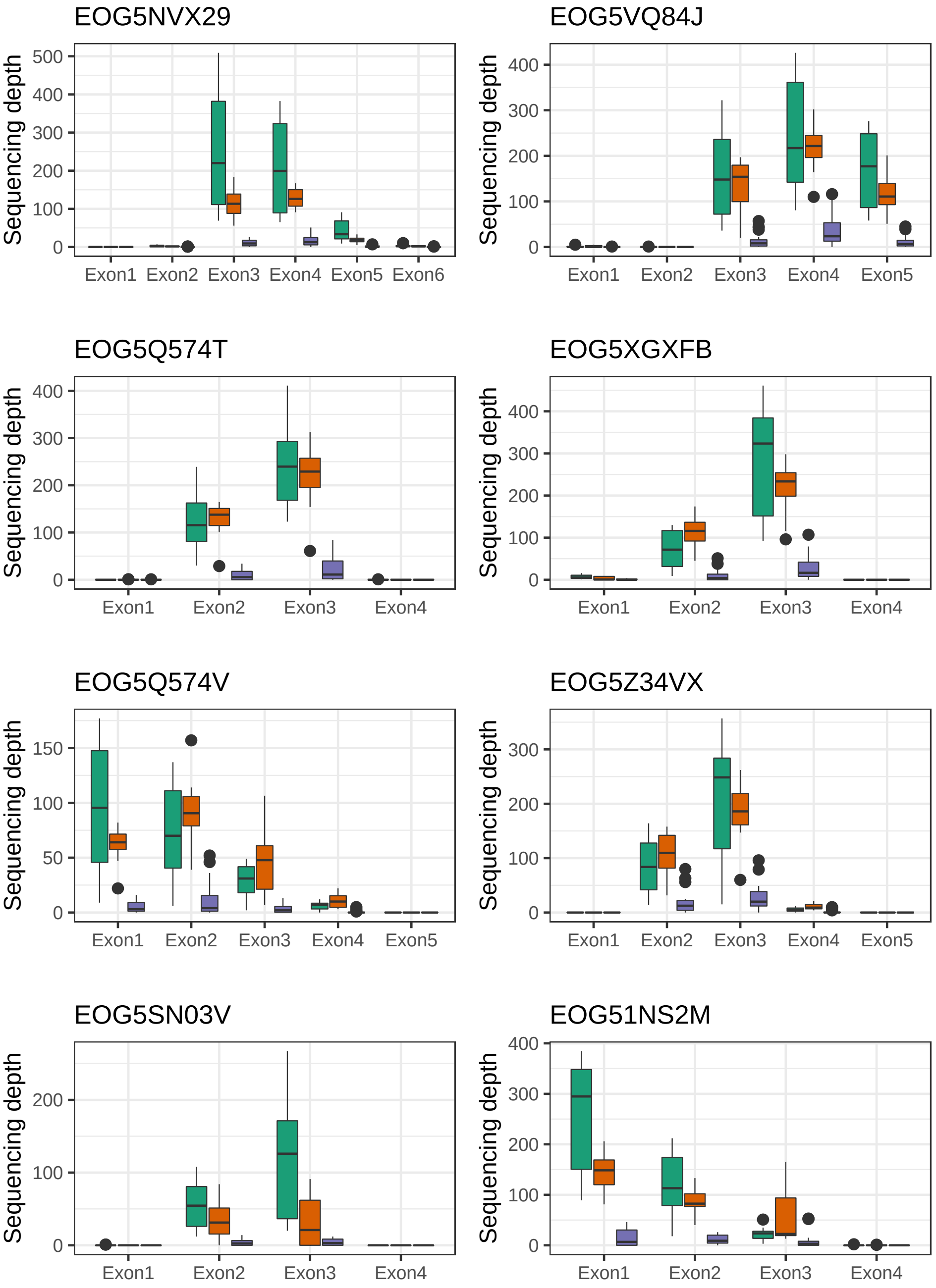

EOG5NVX29
Sequencing depth
0
100
200
300
400
500
Exon1
Exon2
Exon3
Exon4
Exon5
Exon6
EOG5VQ84J
Sequencing depth
0
100
200
300
400
Exon1
Exon2
Exon3
Exon4
Exon5
EOG5Q574T
Sequencing depth
0
100
200
300
400
Exon1
Exon2
Exon3
Exon4
EOG5XGXFB
Sequencing depth
0
100
200
300
400
Exon1
Exon2
Exon3
Exon4
EOG5Q574V
Sequencing depth
0
50
100
150
Exon1
Exon2
Exon3
Exon4
Exon5
EOG5Z34VX
Sequencing depth
0
100
200
300
Exon1
Exon2
Exon3
Exon4
Exon5
EOG5SN03V
Sequencing depth
0
100
200
Exon1
Exon2
Exon3
Exon4
EOG51NS2M
Sequencing depth
0
100
200
300
400
Exon1
Exon2
Exon3
Exon4

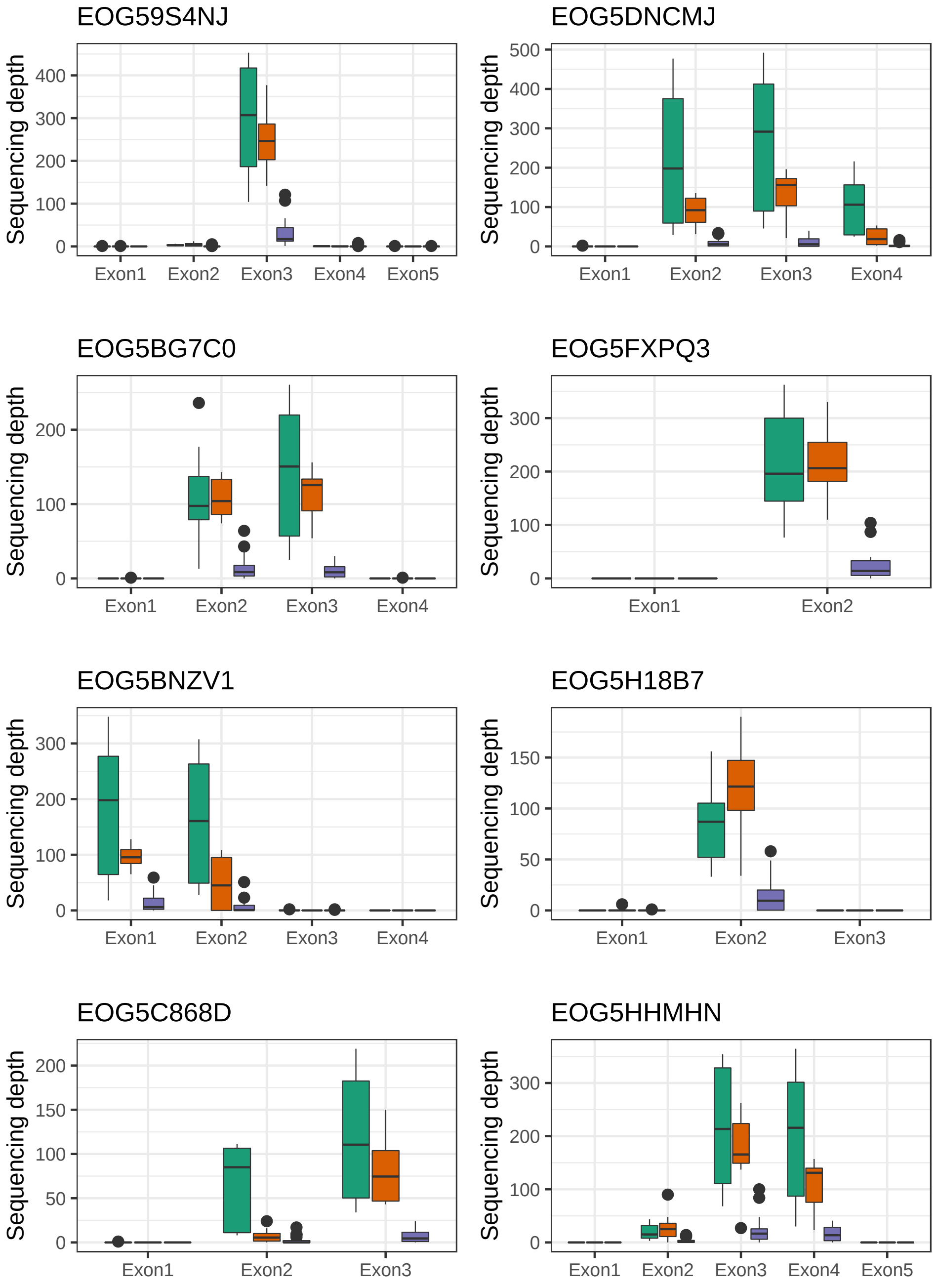
EOG59S4NJ
Sequencing depth
0
100
200
300
400
Exon1
Exon2
Exon3
Exon4
Exon5
EOG5DNCMJ
Sequencing depth
0
100
200
300
400
500
Exon1
Exon2
Exon3
Exon4
EOG5BG7C0
Sequencing depth
0
100
200
Exon1
Exon2
Exon3
Exon4
EOG5FXPQ3
Sequencing depth
0
100
200
300
Exon1
Exon2
EOG5BNZV1
Sequencing depth
0
100
200
300
Exon1
Exon2
Exon3
Exon4
EOG5H18B7
Sequencing depth
0
50
100
150
Exon1
Exon2
Exon3
EOG5C868D
Sequencing depth
0
50
100
150
200
Exon1
Exon2
Exon3
EOG5HHMHN
Sequencing depth
0
100
200
300
Exon1
Exon2
Exon3
Exon4
Exon5

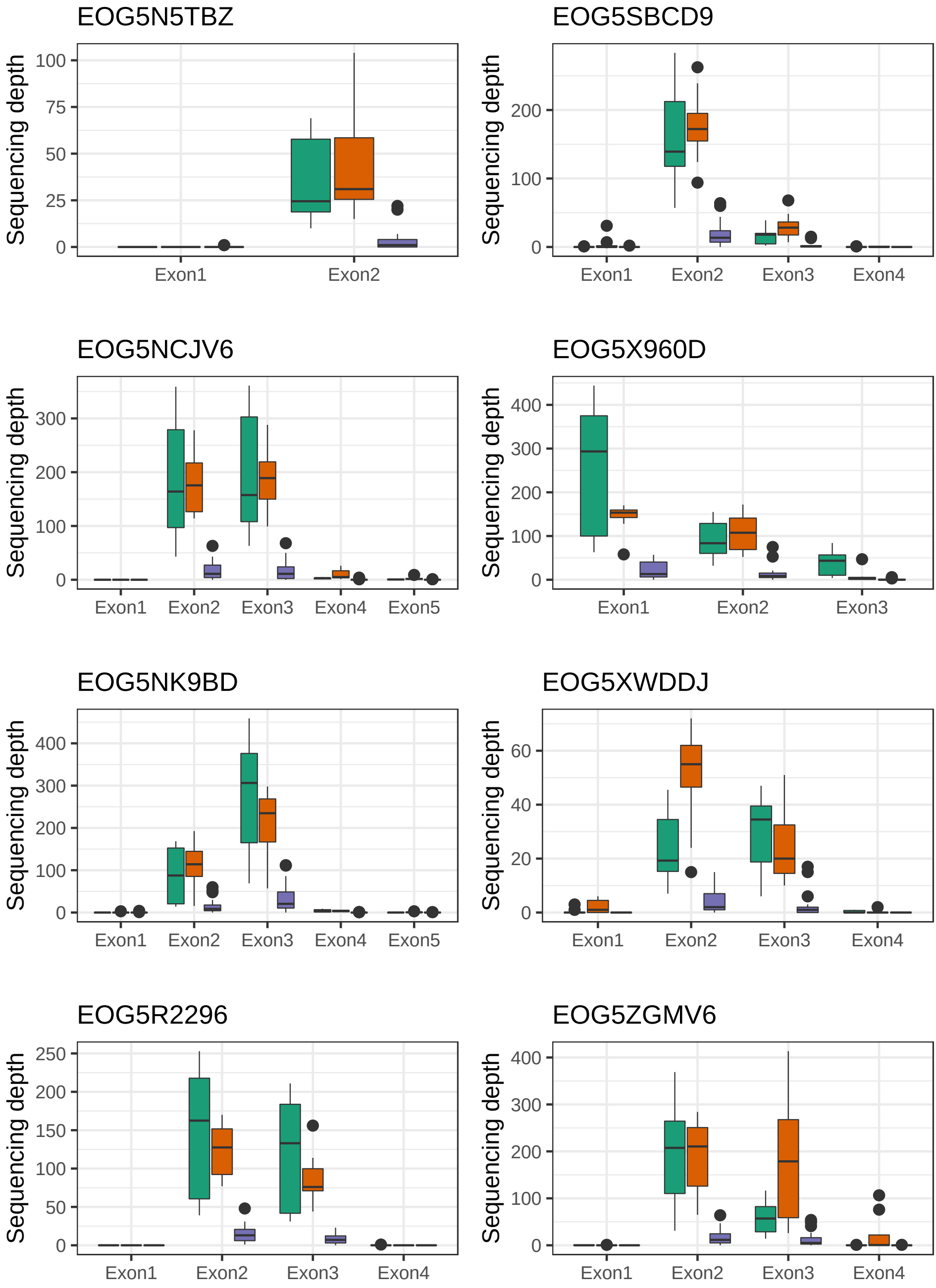
EOG5N5TBZ
Sequencing depth
100
75
50
25
0
Exon1
Exon2
EOG5SBCD9
Sequencing depth
200
100
0
Exon1
Exon2
Exon3
Exon4
EOG5NCJV6
Sequencing depth
300
200
100
0
Exon1
Exon2
Exon3
Exon4
Exon5
EOG5X960D
Sequencing depth
400
300
200
100
0
Exon1
Exon2
Exon3
EOG5NK9BD
Sequencing depth
400
300
200
100
0
Exon1
Exon2
Exon3
Exon4
Exon5
EOG5XWDDJ
Sequencing depth
60
40
20
0
Exon1
Exon2
Exon3
Exon4
EOG5R2296
Sequencing depth
250
200
150
100
50
0
Exon1
Exon2
Exon3
Exon4
EOG5ZGMV6
Sequencing depth
400
300
200
100
0
Exon1
Exon2
Exon3
Exon4

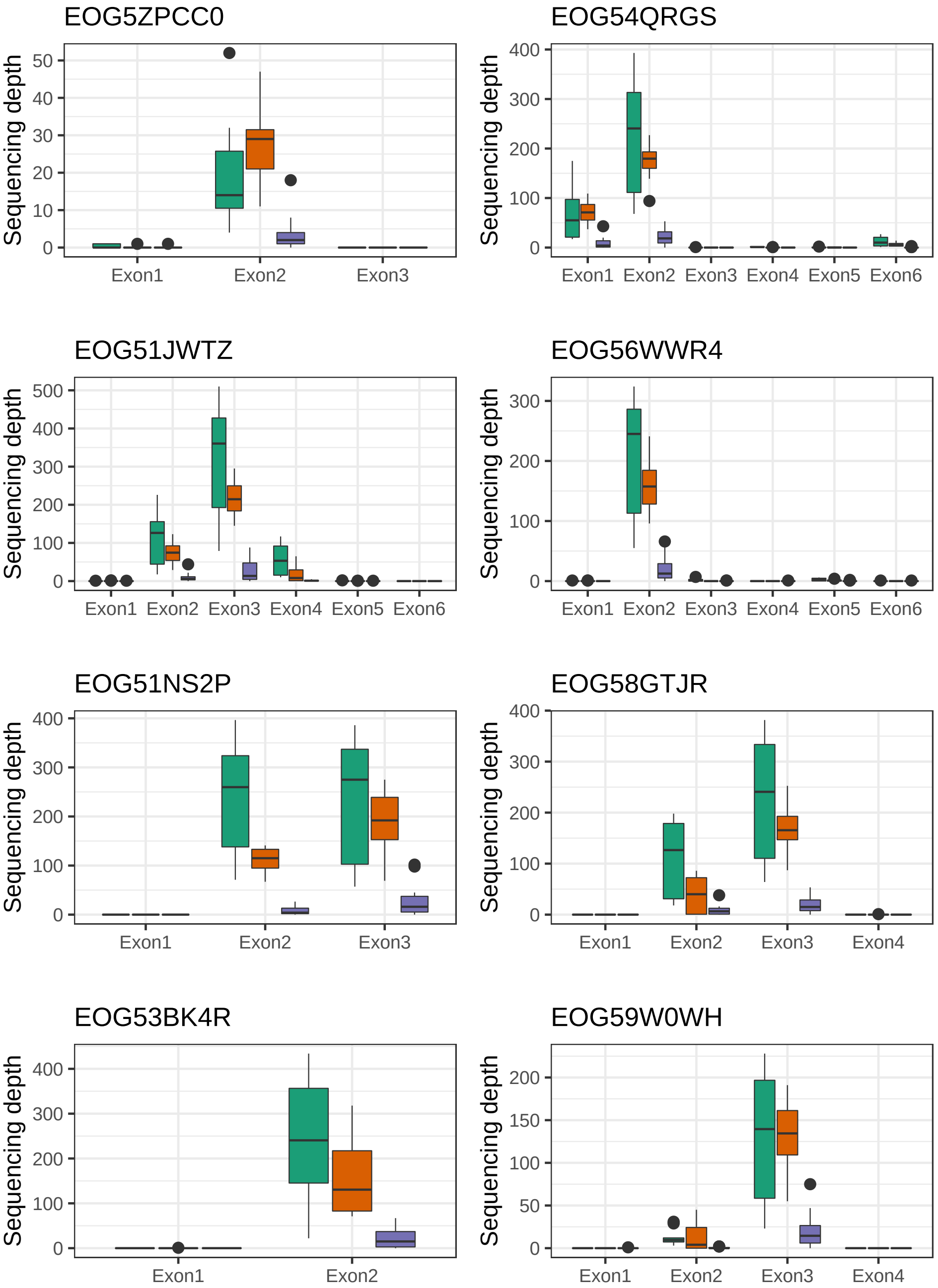
EOG5ZPCC0
Sequencing depth
0
10
20
30
40
50
Exon1
Exon2
Exon3
EOG54QRGS
Sequencing depth
0
100
200
300
400
Exon1
Exon2
Exon3
Exon4
Exon5
Exon6
EOG51JWTZ
Sequencing depth
0
100
200
300
400
500
Exon1
Exon2
Exon3
Exon4
Exon5
Exon6
EOG56WWR4
Sequencing depth
0
100
200
300
Exon1
Exon2
Exon3
Exon4
Exon5
Exon6
EOG51NS2P
Sequencing depth
0
100
200
300
400
Exon1
Exon2
Exon3
EOG58GTJR
Sequencing depth
0
100
200
300
400
Exon1
Exon2
Exon3
Exon4
EOG53BK4R
Sequencing depth
0
100
200
300
400
Exon1
Exon2
EOG59W0WH
Sequencing depth
0
50
100
150
200
Exon1
Exon2
Exon3
Exon4

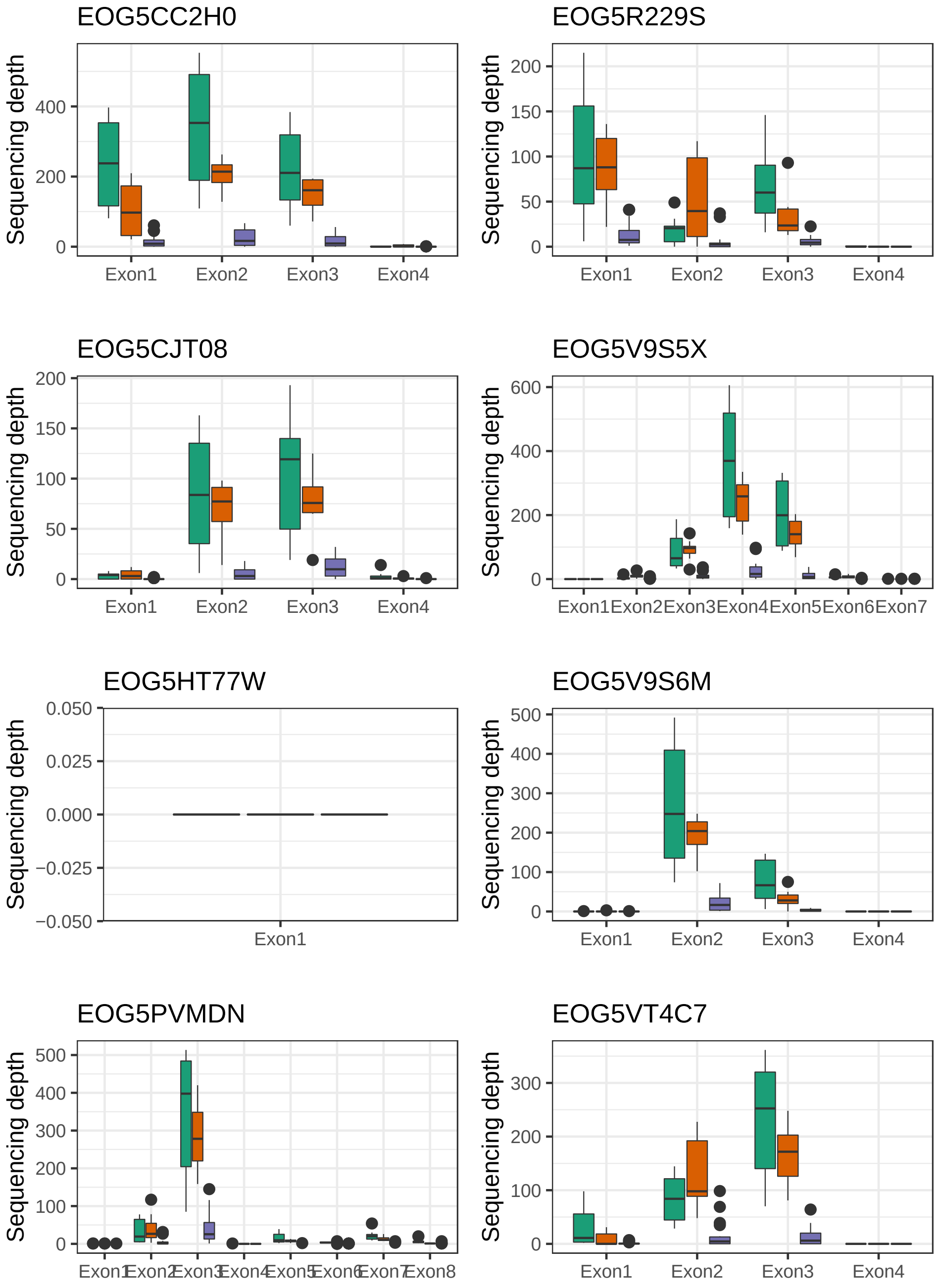
EOG5CC2H0
EOG5R229S
EOG5CJT08
EOG5V9S5X
EOG5HT77W
EOG5V9S6M
EOG5PVMDN
EOG5VT4C7
Sequencing depth
Exon1
Exon2
Exon3
Exon4
Exon5
Exon6
Exon7
Exon8

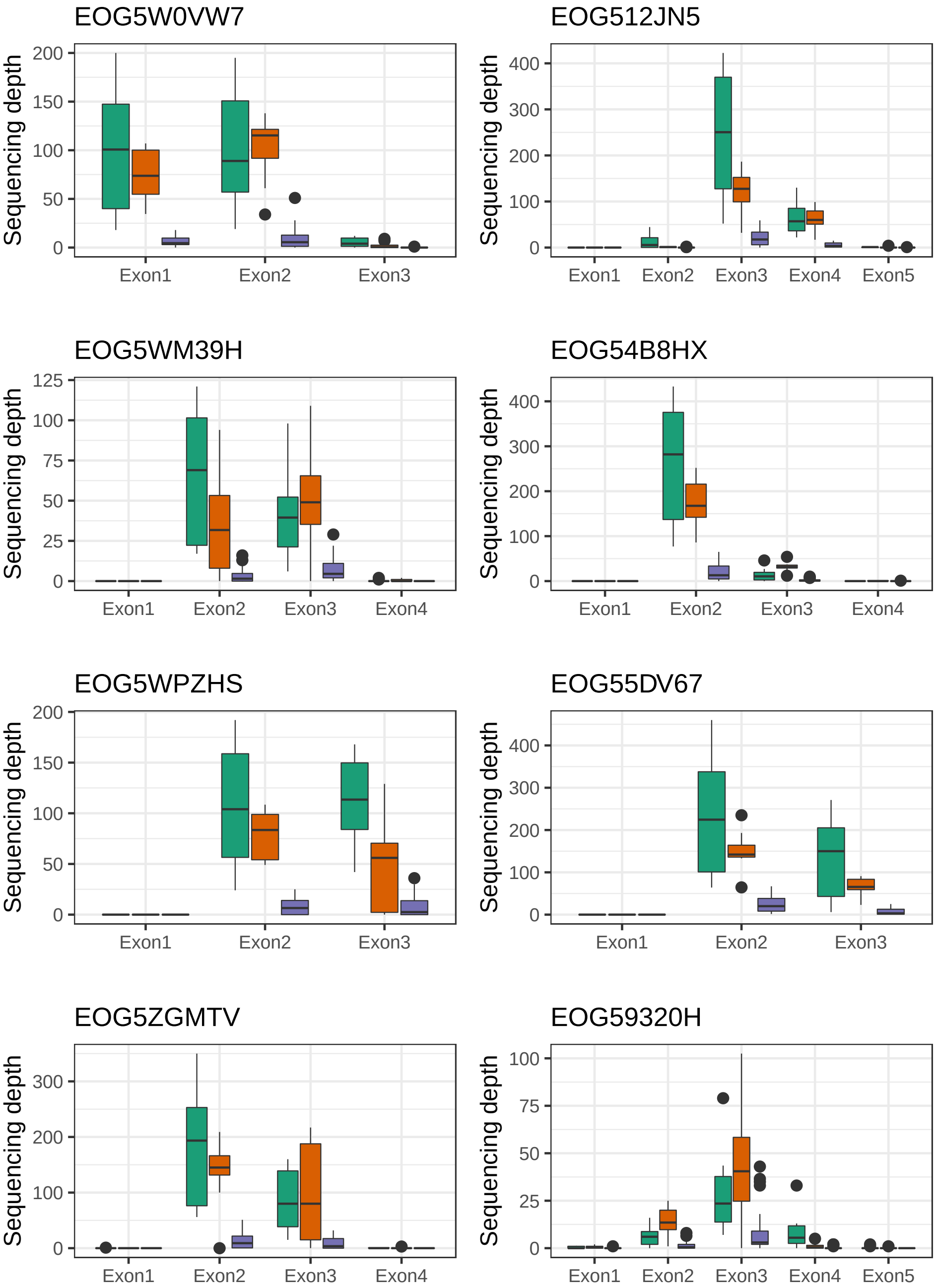
EOG5W0VW7
EOG512JN5
EOG5WM39H
EOG54B8HX
EOG5WPZHS
EOG55DV67
EOG5ZGMTV
EOG59320H
Sequencing depth
Exon1
Exon2
Exon3
Exon4
Exon5
0
25
50
75
100
125
150
200
300
400

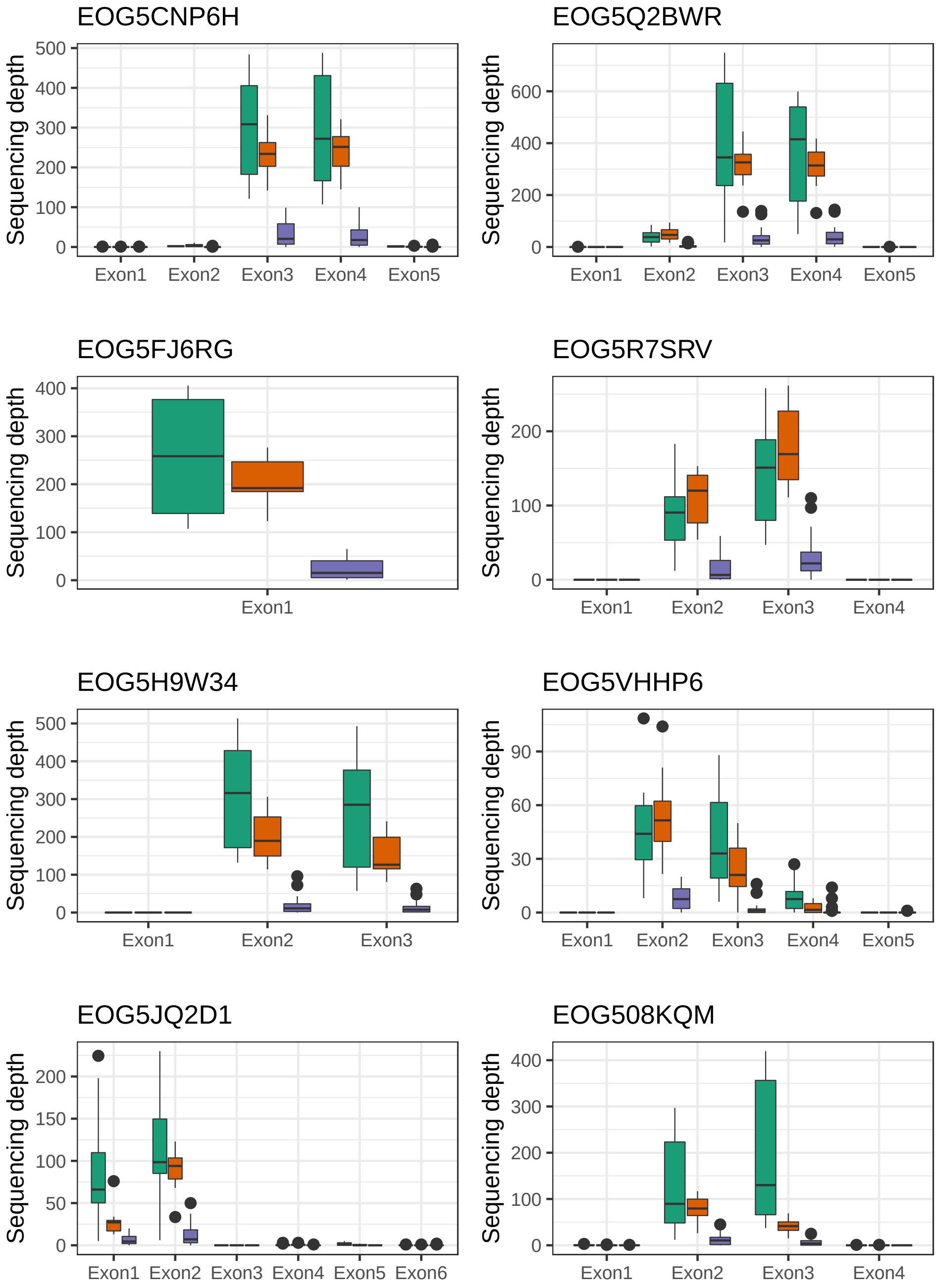
EOG5CNP6H
EOG5Q2BWR
EOG5FJ6RG
EOG5R7SRV
EOG5H9W34
EOG5VHHP6
EOG5JQ2D1
EOG508KQM
Sequencing depth
Exon1
Exon2
Exon3
Exon4
Exon5
Exon6

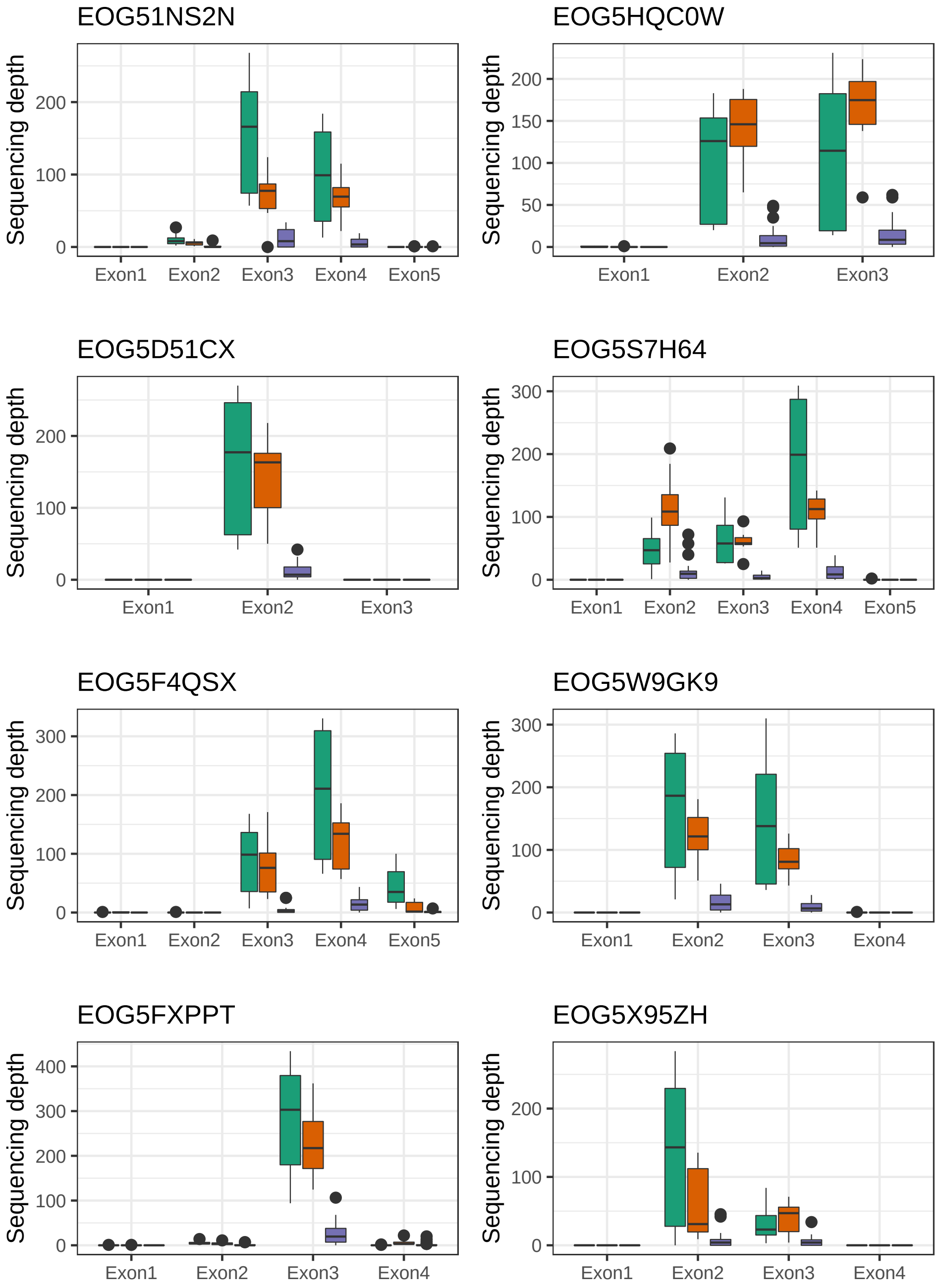
EOG51NS2N
EOG5HQC0W
EOG5D51CX
EOG5S7H64
EOG5F4QSX
EOG5W9GK9
EOG5FXPPT
EOG5X95ZH
Sequencing depth
Exon1
Exon2
Exon3
Exon4
Exon5
0
50
100
150
200
300
400

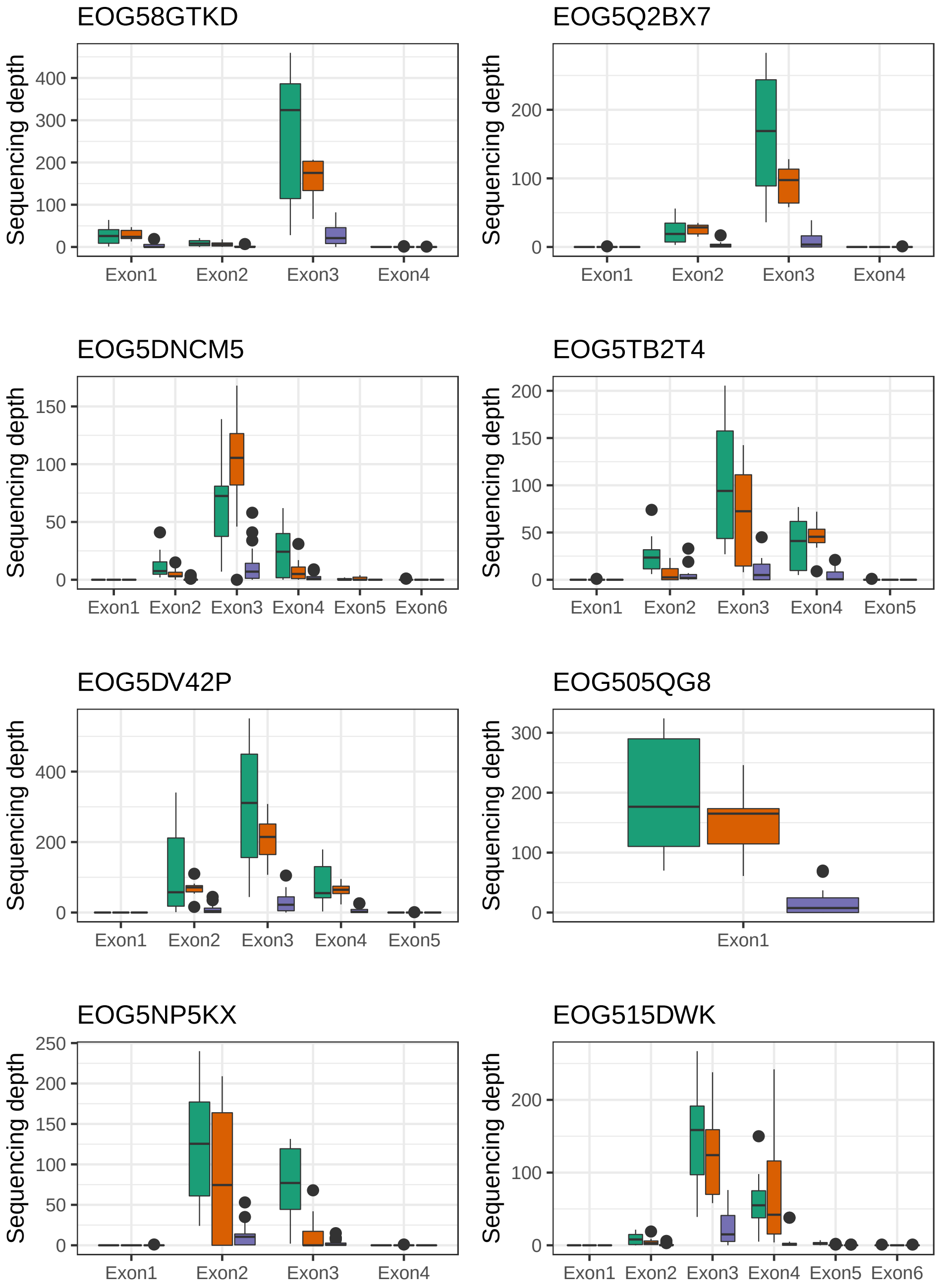
EOG58GTKD
Sequencing depth
0
100
200
300
400
Exon1
Exon2
Exon3
Exon4
EOG5Q2BX7
Sequencing depth
0
100
200
Exon1
Exon2
Exon3
Exon4
EOG5DNCM5
Sequencing depth
0
50
100
150
Exon1
Exon2
Exon3
Exon4
Exon5
Exon6
EOG5TB2T4
Sequencing depth
0
50
100
150
200
Exon1
Exon2
Exon3
Exon4
Exon5
EOG5DV42P
Sequencing depth
0
200
400
Exon1
Exon2
Exon3
Exon4
Exon5
EOG505QG8
Sequencing depth
0
100
200
300
Exon1
EOG5NP5KX
Sequencing depth
0
50
100
150
200
250
Exon1
Exon2
Exon3
Exon4
EOG515DWK
Sequencing depth
0
100
200
Exon1
Exon2
Exon3
Exon4
Exon5
Exon6

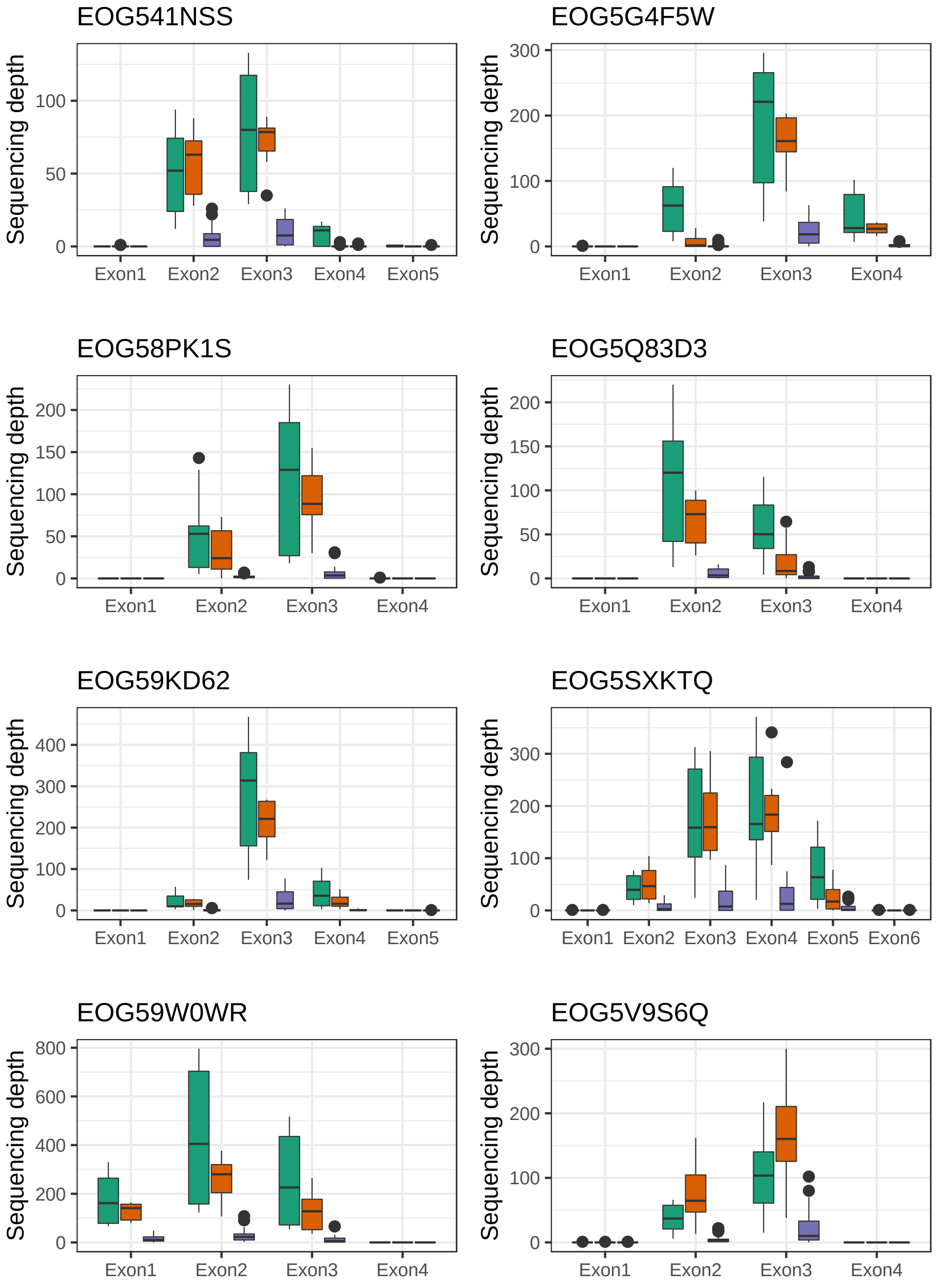
EOG541NSS
Sequencing depth
0
50
100
Exon1
Exon2
Exon3
Exon4
Exon5
EOG5G4F5W
Sequencing depth
0
100
200
300
Exon1
Exon2
Exon3
Exon4
EOG58PK1S
Sequencing depth
0
50
100
150
200
Exon1
Exon2
Exon3
Exon4
EOG5Q83D3
Sequencing depth
0
50
100
150
200
Exon1
Exon2
Exon3
Exon4
EOG59KD62
Sequencing depth
0
100
200
300
400
Exon1
Exon2
Exon3
Exon4
Exon5
EOG5SXKTQ
Sequencing depth
0
100
200
300
Exon1
Exon2
Exon3
Exon4
Exon5
Exon6
EOG59W0WR
Sequencing depth
0
200
400
600
800
Exon1
Exon2
Exon3
Exon4
EOG5V9S6Q
Sequencing depth
0
100
200
300
Exon1
Exon2
Exon3
Exon4

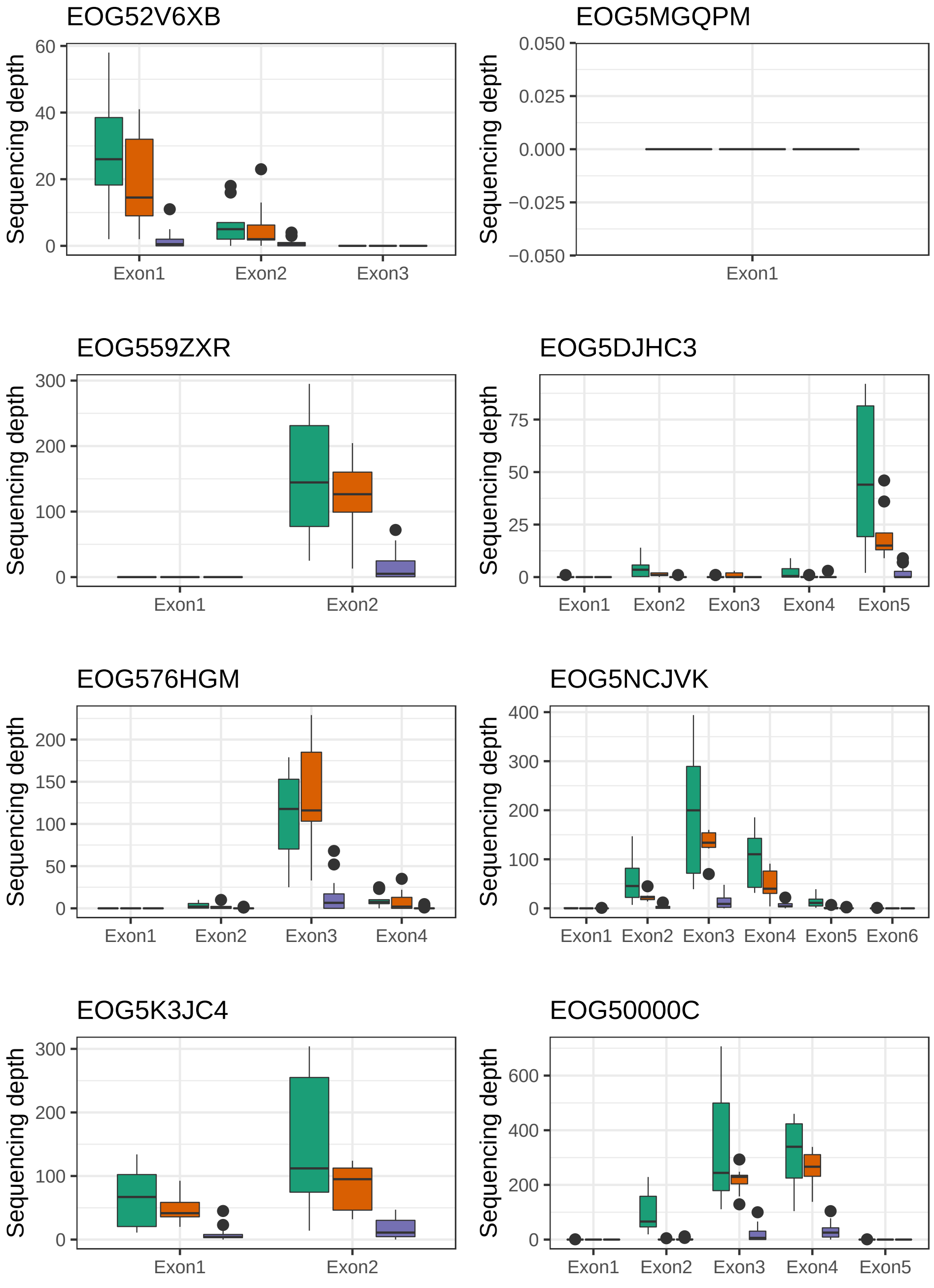
EOG52V6XB
Sequencing depth
0
20
40
60
Exon1
Exon2
Exon3
EOG5MGQPM
Sequencing depth
0.050
0.025
0.000
−0.025
−0.050
Exon1
EOG559ZXR
Sequencing depth
0
100
200
300
Exon1
Exon2
EOG5DJHC3
Sequencing depth
0
25
50
75
Exon1
Exon2
Exon3
Exon4
Exon5
EOG576HGM
Sequencing depth
0
50
100
150
200
Exon1
Exon2
Exon3
Exon4
EOG5NCJVK
Sequencing depth
0
100
200
300
400
Exon1
Exon2
Exon3
Exon4
Exon5
Exon6
EOG5K3JC4
Sequencing depth
0
100
200
300
Exon1
Exon2
EOG50000C
Sequencing depth
0
200
400
600
Exon1
Exon2
Exon3
Exon4
Exon5

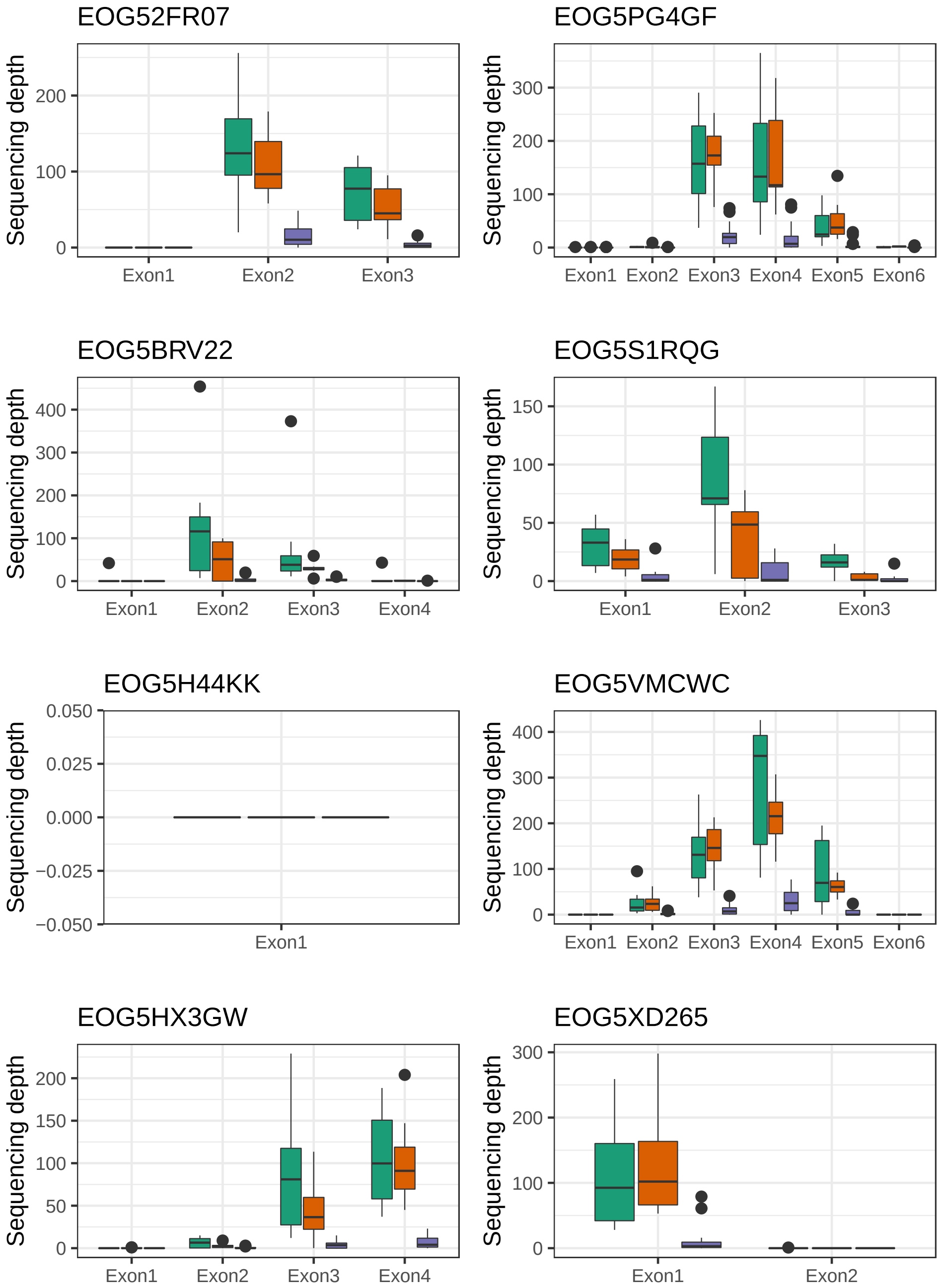
EOG52FR07
Sequencing depth
0
100
200
Exon1
Exon2
Exon3
EOG5PG4GF
Sequencing depth
0
100
200
300
Exon1
Exon2
Exon3
Exon4
Exon5
Exon6
EOG5BRV22
Sequencing depth
0
100
200
300
400
Exon1
Exon2
Exon3
Exon4
EOG5S1RQG
Sequencing depth
0
50
100
150
Exon1
Exon2
Exon3
EOG5H44KK
Sequencing depth
0.050
0.025
0.000
−0.025
−0.050
Exon1
EOG5VMCWC
Sequencing depth
0
100
200
300
400
Exon1
Exon2
Exon3
Exon4
Exon5
Exon6
EOG5HX3GW
Sequencing depth
0
50
100
150
200
Exon1
Exon2
Exon3
Exon4
EOG5XD265
Sequencing depth
0
100
200
300
Exon1
Exon2

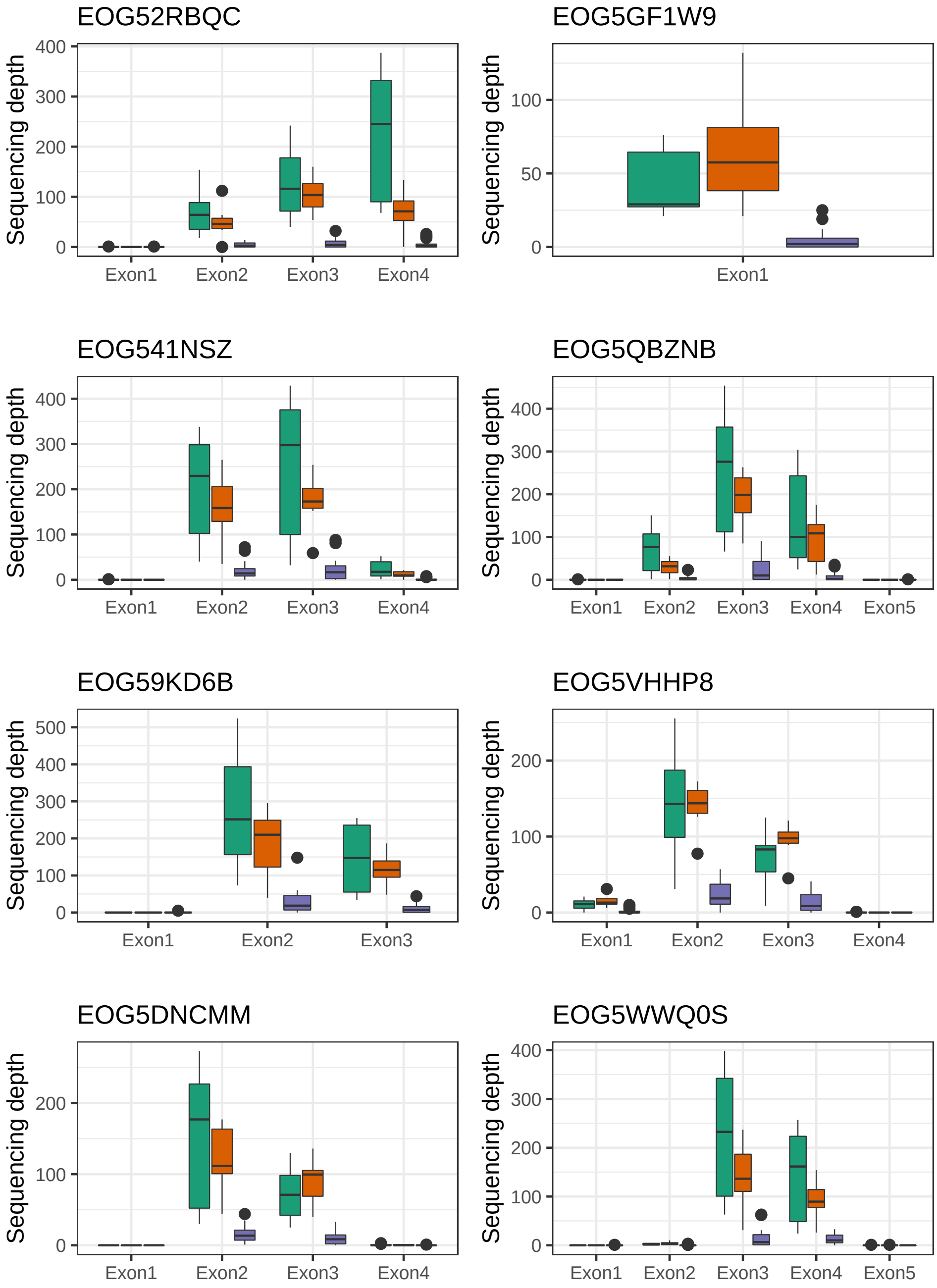
EOG52RBQC
EOG5GF1W9
EOG541NSZ
EOG5QBZNB
EOG59KD6B
EOG5VHHP8
EOG5DNCMM
EOG5WWQ0S
Sequencing depth
Exon1
Exon2
Exon3
Exon4
Exon5

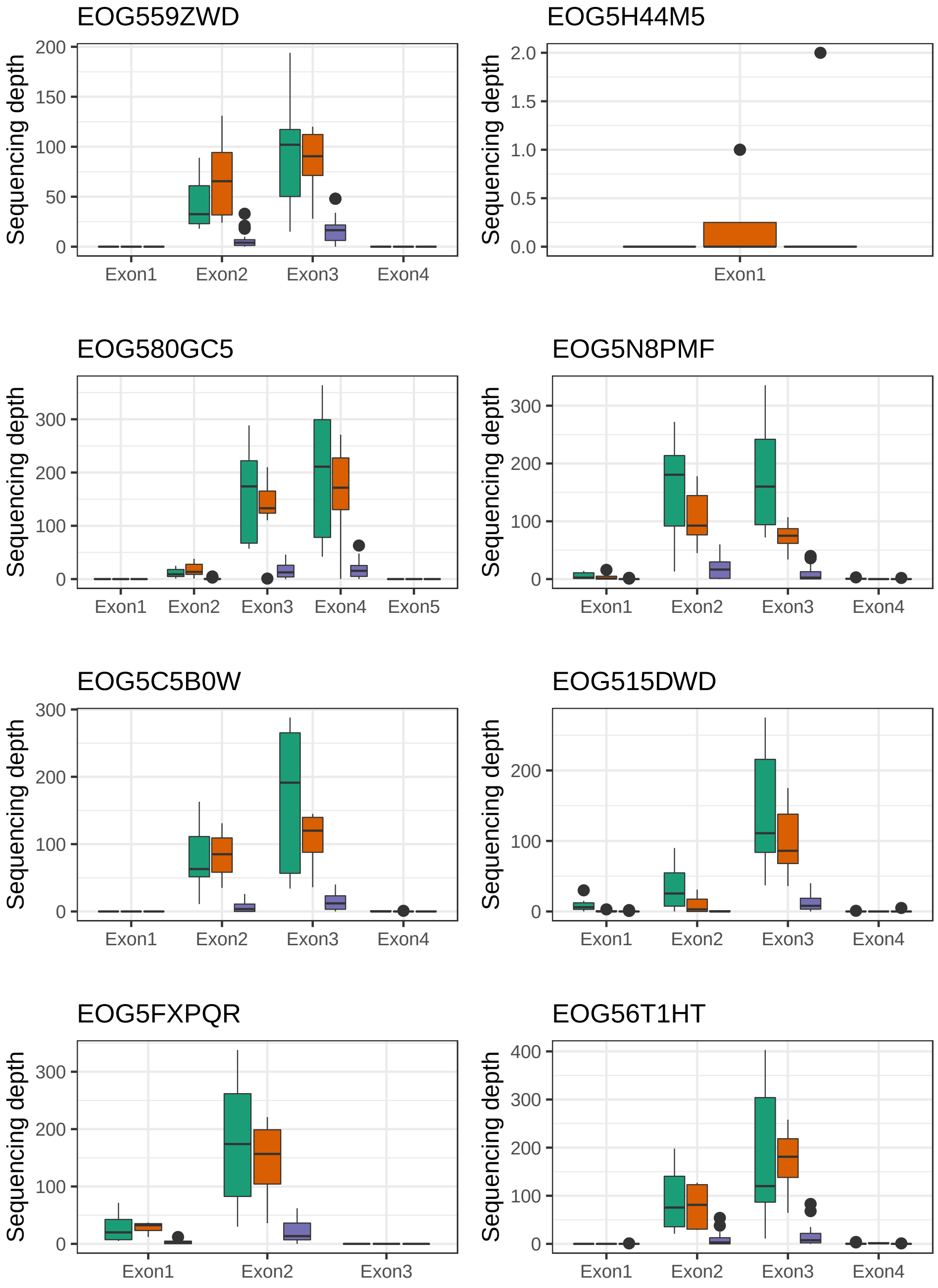
EOG559ZWD
Sequencing depth
0
50
100
150
200
Exon1
Exon2
Exon3
Exon4
EOG5H44M5
Sequencing depth
0.0
0.5
1.0
1.5
2.0
Exon1
EOG580GC5
Sequencing depth
0
100
200
300
Exon1
Exon2
Exon3
Exon4
Exon5
EOG5N8PMF
Sequencing depth
0
100
200
300
Exon1
Exon2
Exon3
Exon4
EOG5C5B0W
Sequencing depth
0
100
200
300
Exon1
Exon2
Exon3
Exon4
EOG515DWD
Sequencing depth
0
100
200
Exon1
Exon2
Exon3
Exon4
EOG5FXPQR
Sequencing depth
0
100
200
300
Exon1
Exon2
Exon3
EOG56T1HT
Sequencing depth
0
100
200
300
400
Exon1
Exon2
Exon3
Exon4

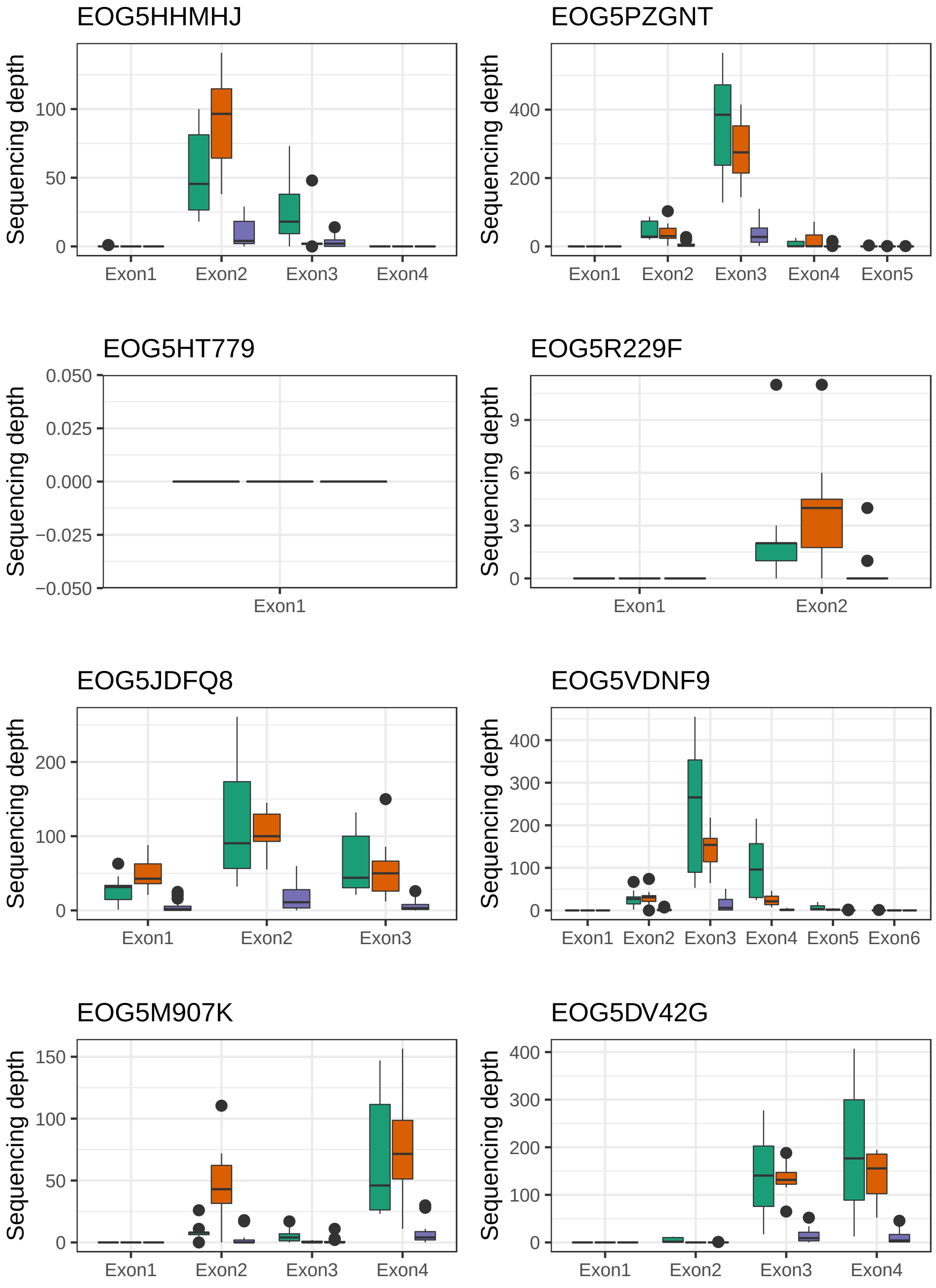
EOG5HHMHJ
Sequencing depth
0
50
100
Exon1
Exon2
Exon3
Exon4
EOG5PZGNT
Sequencing depth
0
200
400
Exon1
Exon2
Exon3
Exon4
Exon5
EOG5HT779
Sequencing depth
0.050
0.025
0.000
−0.025
−0.050
Exon1
EOG5R229F
Sequencing depth
0
3
6
9
Exon1
Exon2
EOG5JDFQ8
Sequencing depth
0
100
200
Exon1
Exon2
Exon3
EOG5VDNF9
Sequencing depth
0
100
200
300
400
Exon1
Exon2
Exon3
Exon4
Exon5
Exon6
EOG5M907K
Sequencing depth
0
50
100
150
Exon1
Exon2
Exon3
Exon4
EOG5DV42G
Sequencing depth
0
100
200
300
400
Exon1
Exon2
Exon3
Exon4

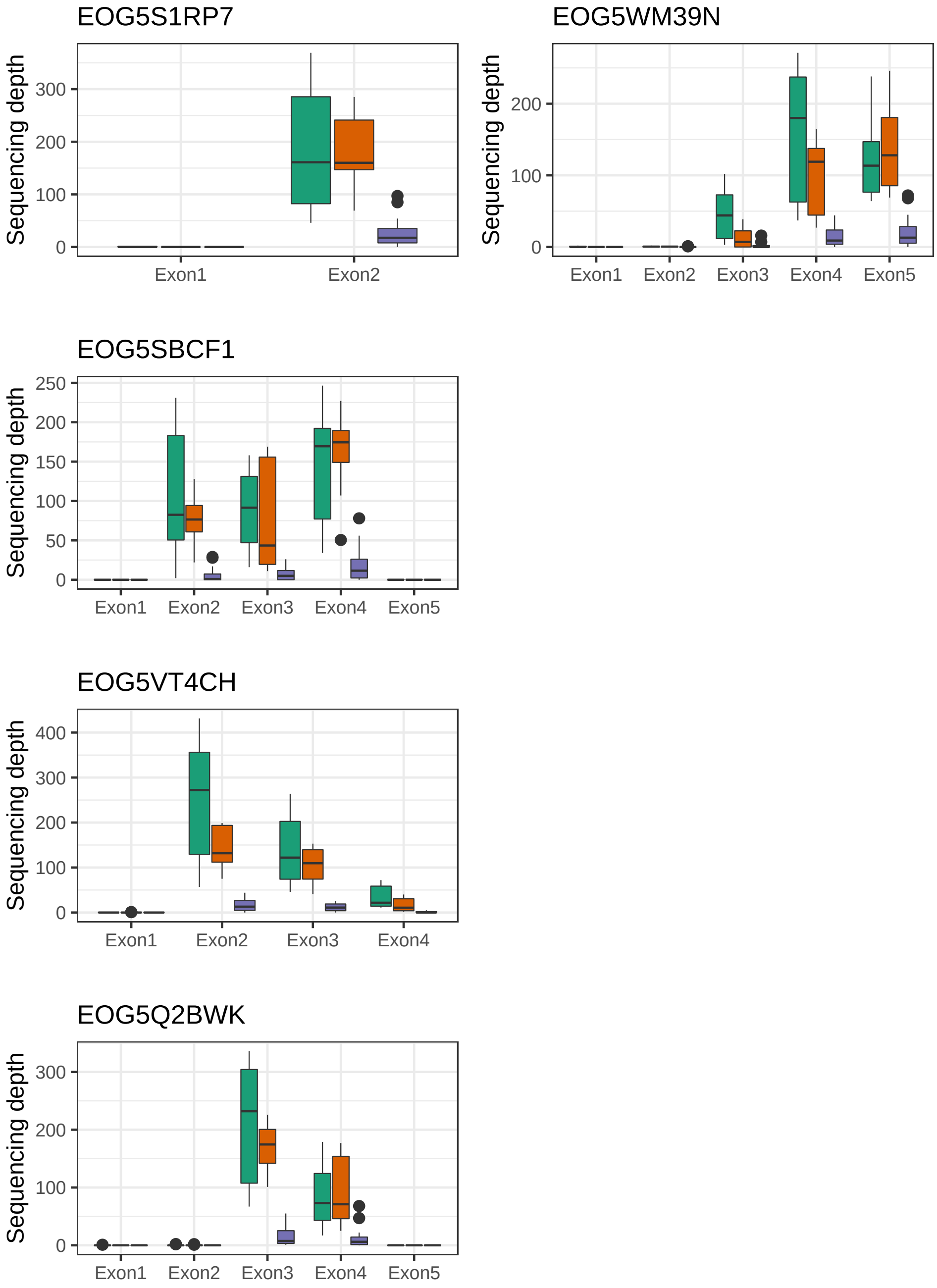
EOG5S1RP7
Sequencing depth
300
200
100
0
Exon1
Exon2
EOG5WM39N
Sequencing depth
200
100
0
Exon1
Exon2
Exon3
Exon4
Exon5
EOG5SBCF1
Sequencing depth
250
200
150
100
50
0
Exon1
Exon2
Exon3
Exon4
Exon5
EOG5VT4CH
Sequencing depth
400
300
200
100
0
Exon1
Exon2
Exon3
Exon4
EOG5Q2BWK
Sequencing depth
300
200
100
0
Exon1
Exon2
Exon3
Exon4
Exon5
