# Supplementary material for: Development and evaluation of a custom bait design based on 469 single-copy protein-coding genes for exon capture of isopods (Philosciidae: Haloniscus)
Source: PLoS One. 2021 Sep 17;16(9):e0256861. doi: 10.1371/journal.pone.0256861 (PMC8448321; doi:10.1371/journal.pone.0256861)
Supplement: S6 Fig — The ID of each OG is specified above plots, and points are coloured according to sequencing ID. (PDF) [file pone.0256861.s006.pdf]

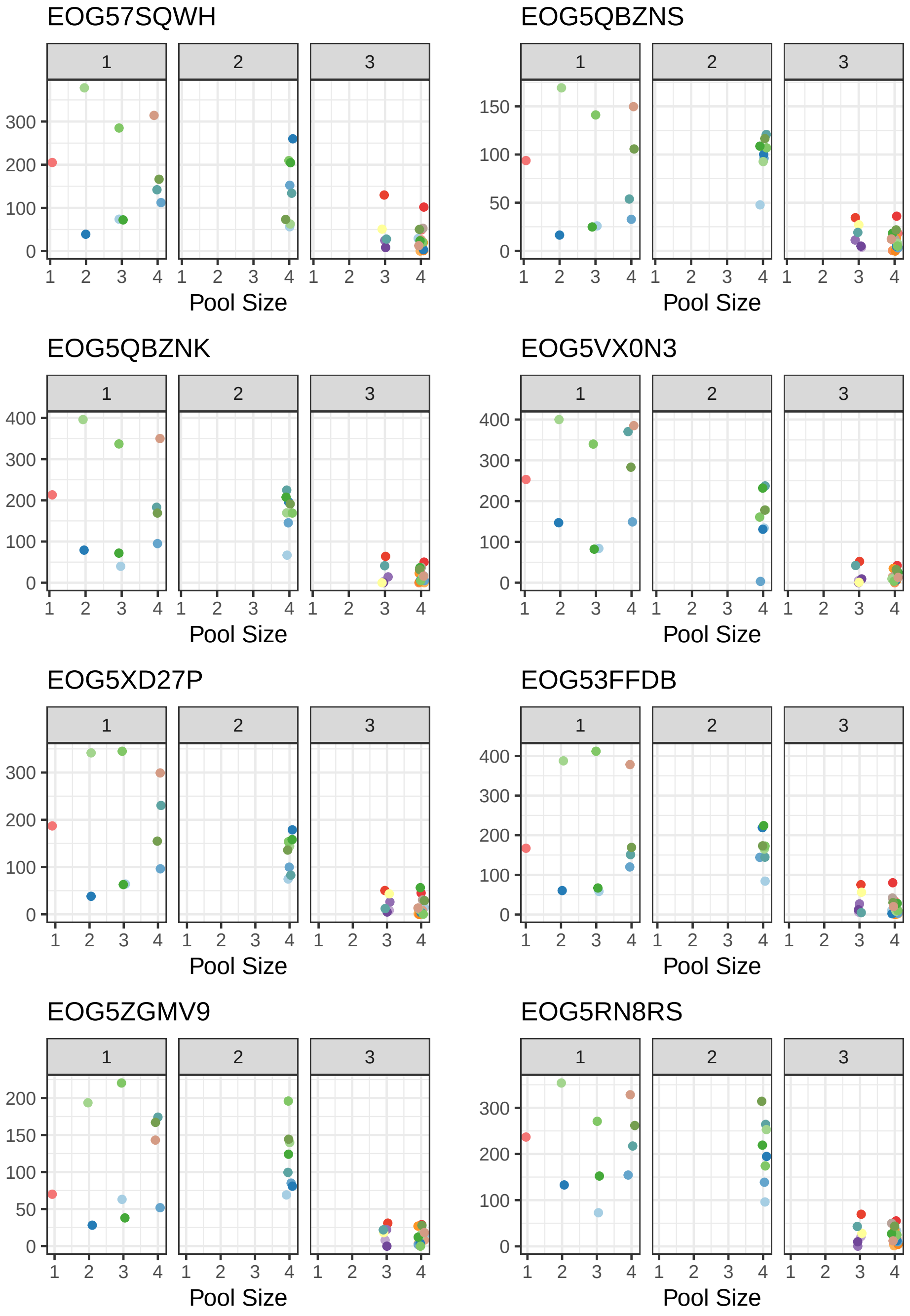
EOG57SQWH
1
2
3
300
200
100
0
1 2 3 4 1 2 3 4 1 2 3 4
Pool Size
EOG5QBZNS
1
2
3
150
100
50
0
1 2 3 4 1 2 3 4 1 2 3 4
Pool Size
EOG5QBZNK
1
2
3
400
300
200
100
0
1 2 3 4 1 2 3 4 1 2 3 4
Pool Size
EOG5VX0N3
1
2
3
400
300
200
100
0
1 2 3 4 1 2 3 4 1 2 3 4
Pool Size
EOG5XD27P
1
2
3
300
200
100
0
1 2 3 4 1 2 3 4 1 2 3 4
Pool Size
EOG53FFDB
1
2
3
400
300
200
100
0
1 2 3 4 1 2 3 4 1 2 3 4
Pool Size
EOG5ZGMV9
1
2
3
200
150
100
50
0
1 2 3 4 1 2 3 4 1 2 3 4
Pool Size
EOG5RN8RS
1
2
3
300
200
100
0
1 2 3 4 1 2 3 4 1 2 3 4
Pool Size

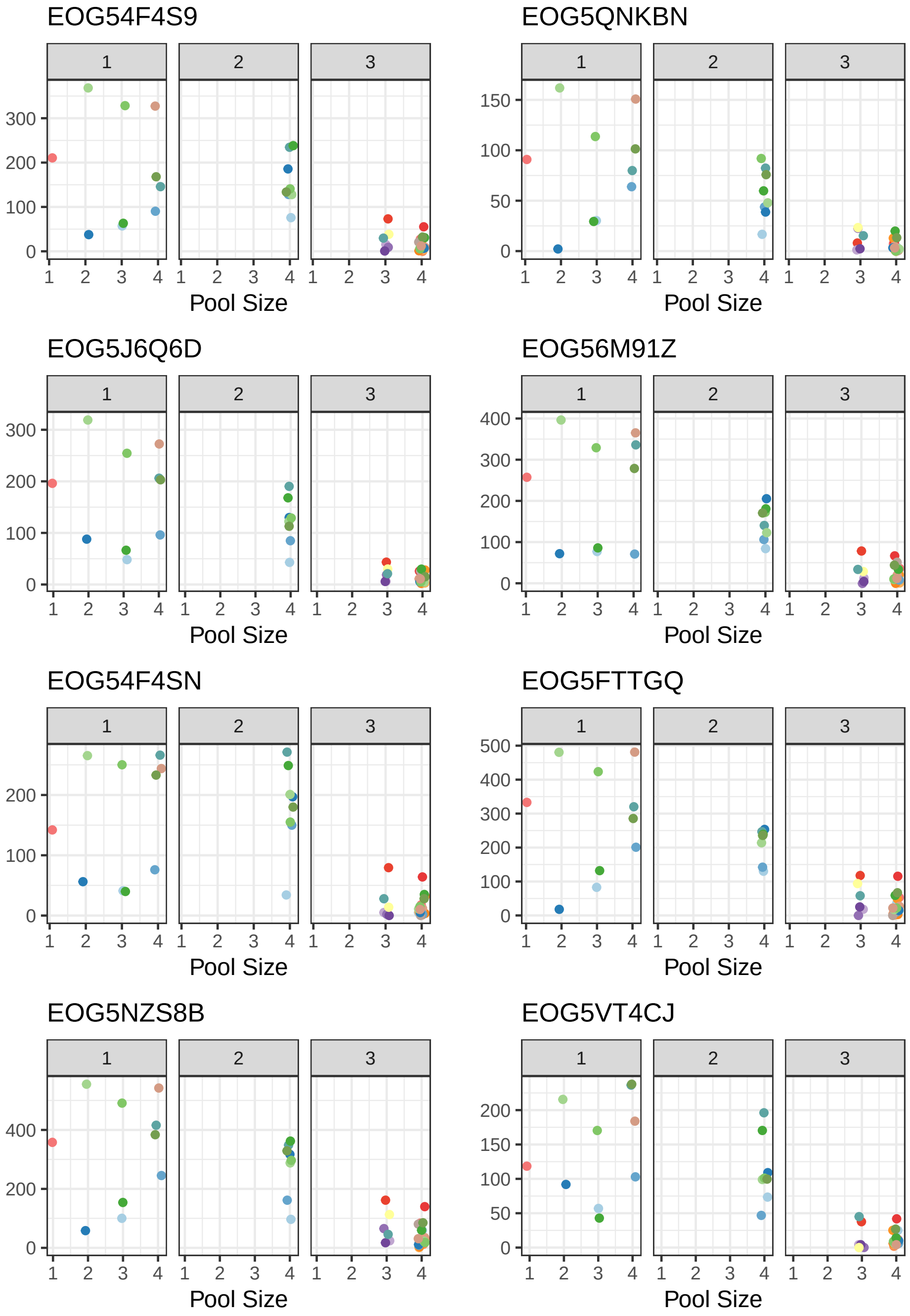
EOG54F4S9
1
2
3
0
100
200
300
Pool Size
EOG5QNKBN
1
2
3
0
50
100
150
Pool Size
EOG5J6Q6D
1
2
3
0
100
200
300
Pool Size
EOG56M91Z
1
2
3
0
100
200
300
400
Pool Size
EOG54F4SN
1
2
3
0
100
200
Pool Size
EOG5FTTGQ
1
2
3
0
100
200
300
400
500
Pool Size
EOG5NZS8B
1
2
3
0
200
400
Pool Size
EOG5VT4CJ
1
2
3
0
50
100
150
200
Pool Size

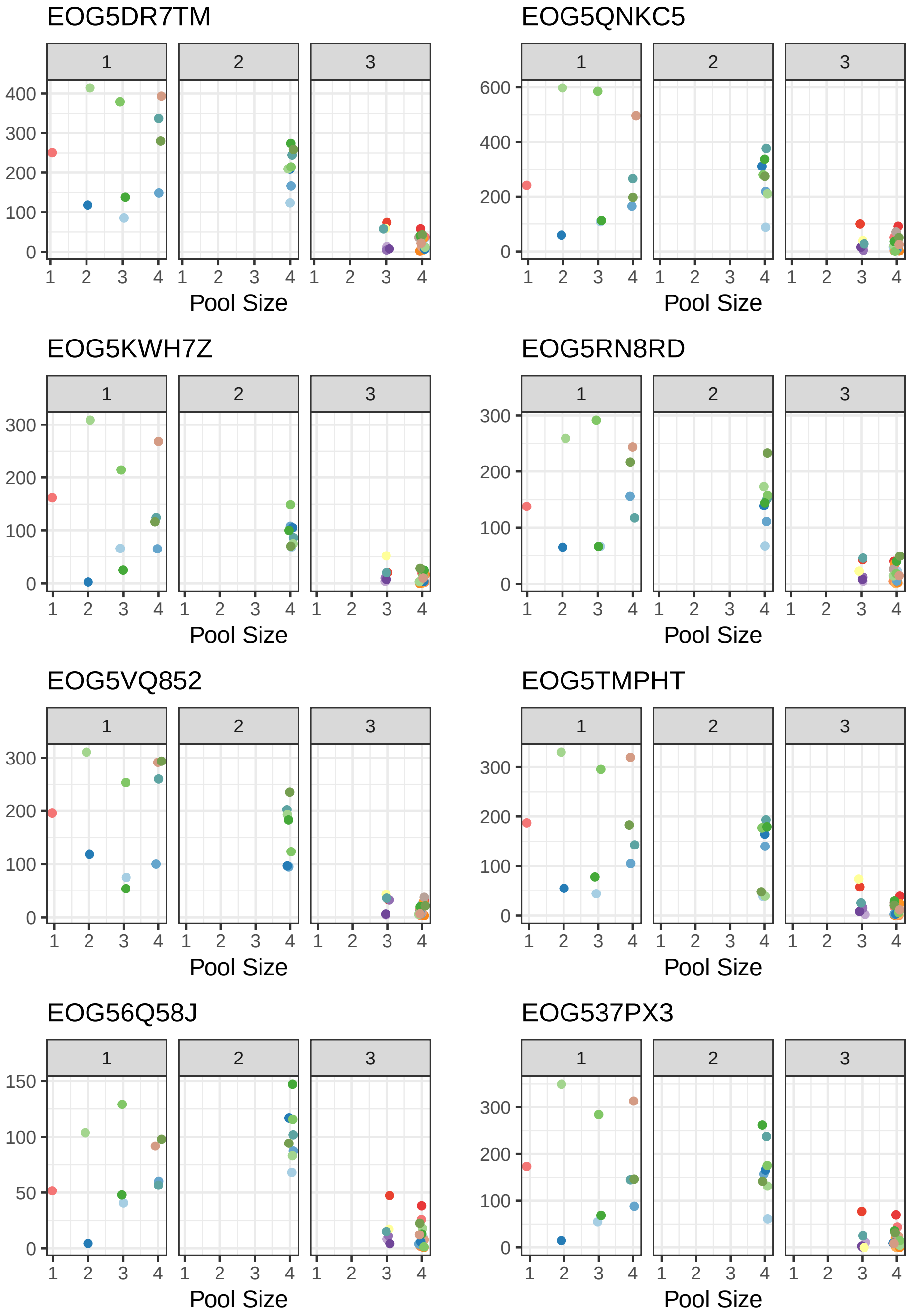
EOG5DR7TM
EOG5QNKC5
EOG5KWH7Z
EOG5RN8RD
EOG5VQ852
EOG5TMPHT
EOG56Q58J
EOG537PX3
1
2
3
Pool Size

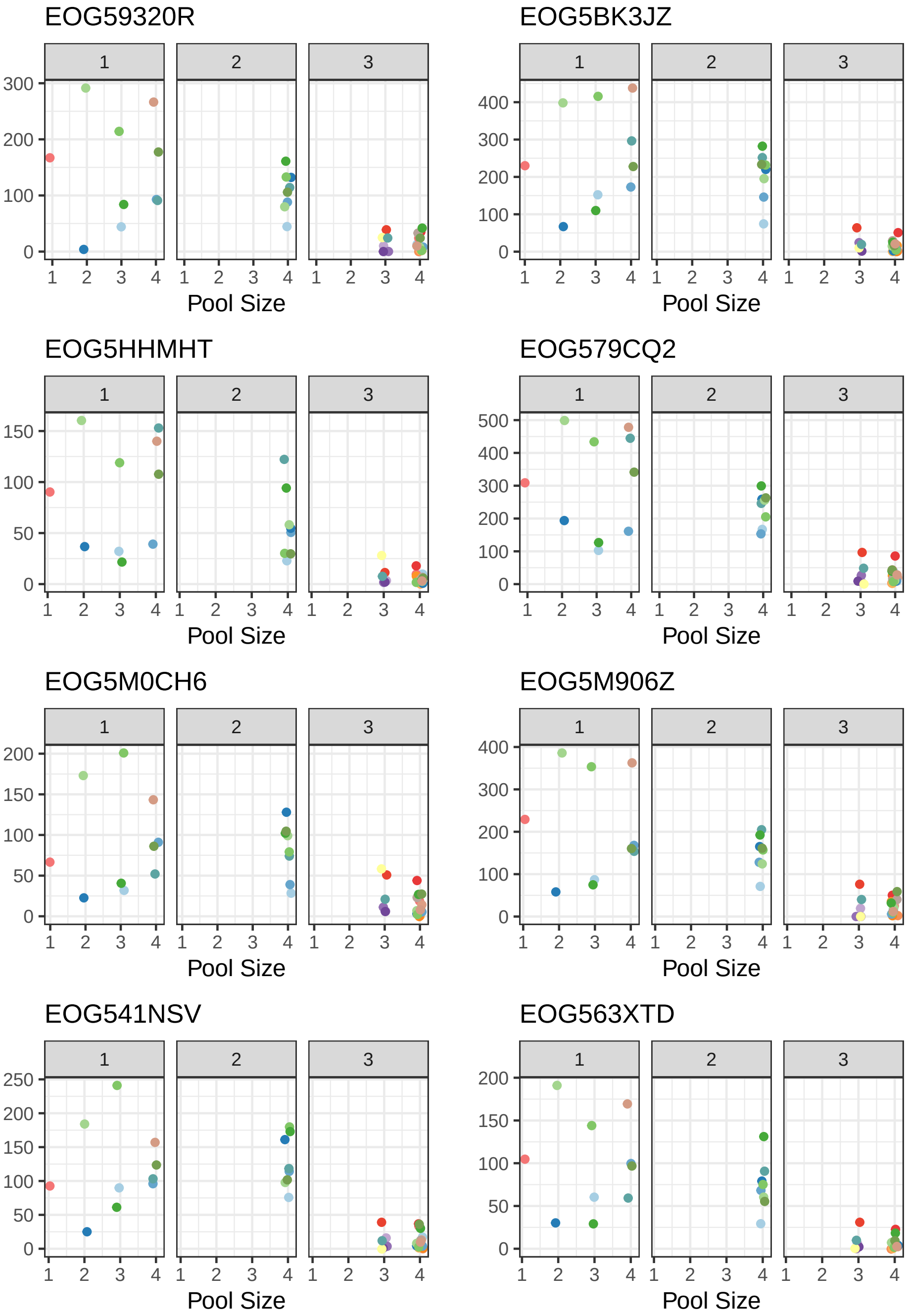
EOG59320R
EOG5BK3JZ
EOG5HHMHT
EOG579CQ2
EOG5M0CH6
EOG5M906Z
EOG541NSV
EOG563XTD
1
2
3
Pool Size

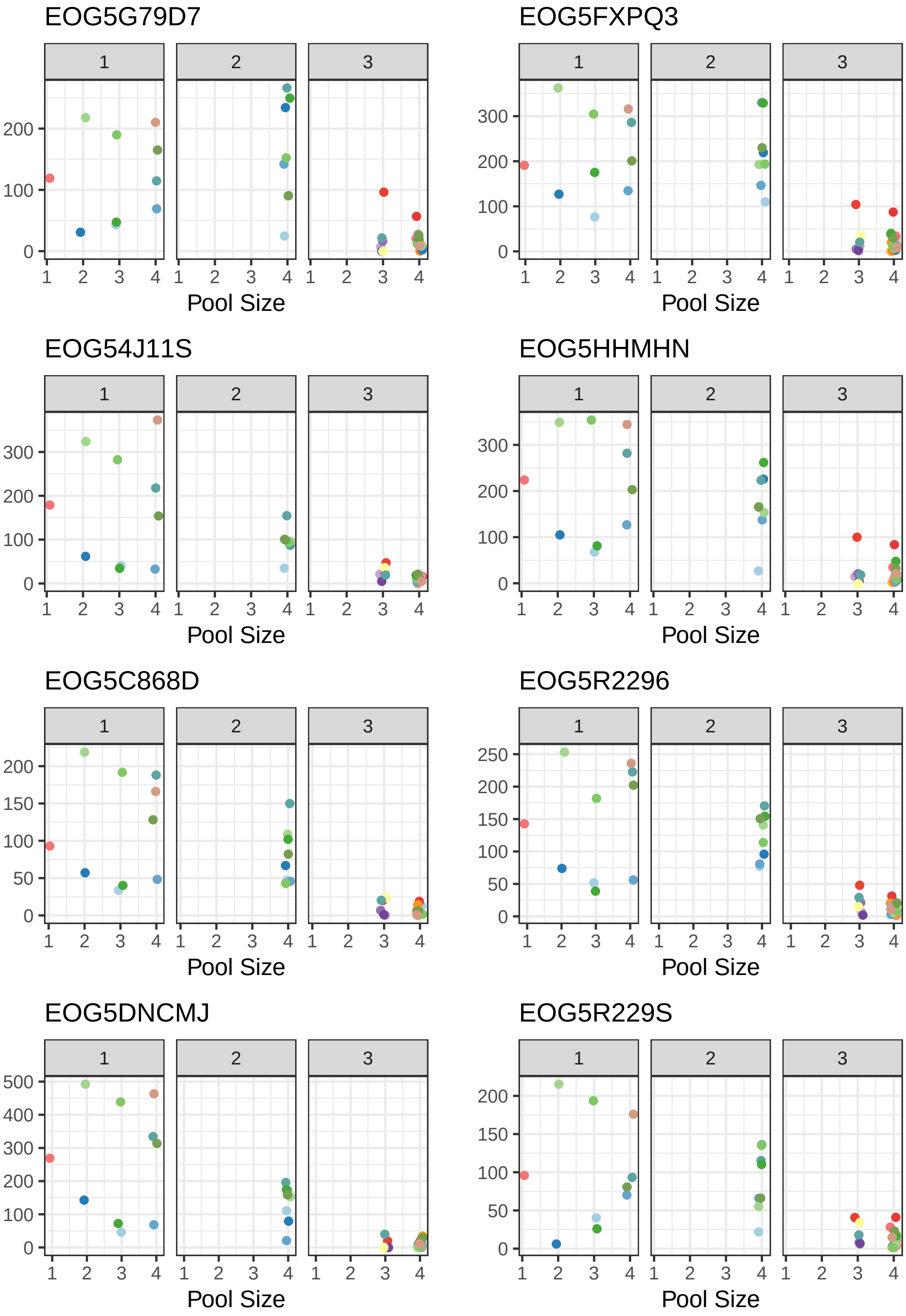
EOG5G79D7
EOG5FXPQ3
EOG54J11S
EOG5HHMHN
EOG5C868D
EOG5R2296
EOG5DNCMJ
EOG5R229S
1
2
3
Pool Size

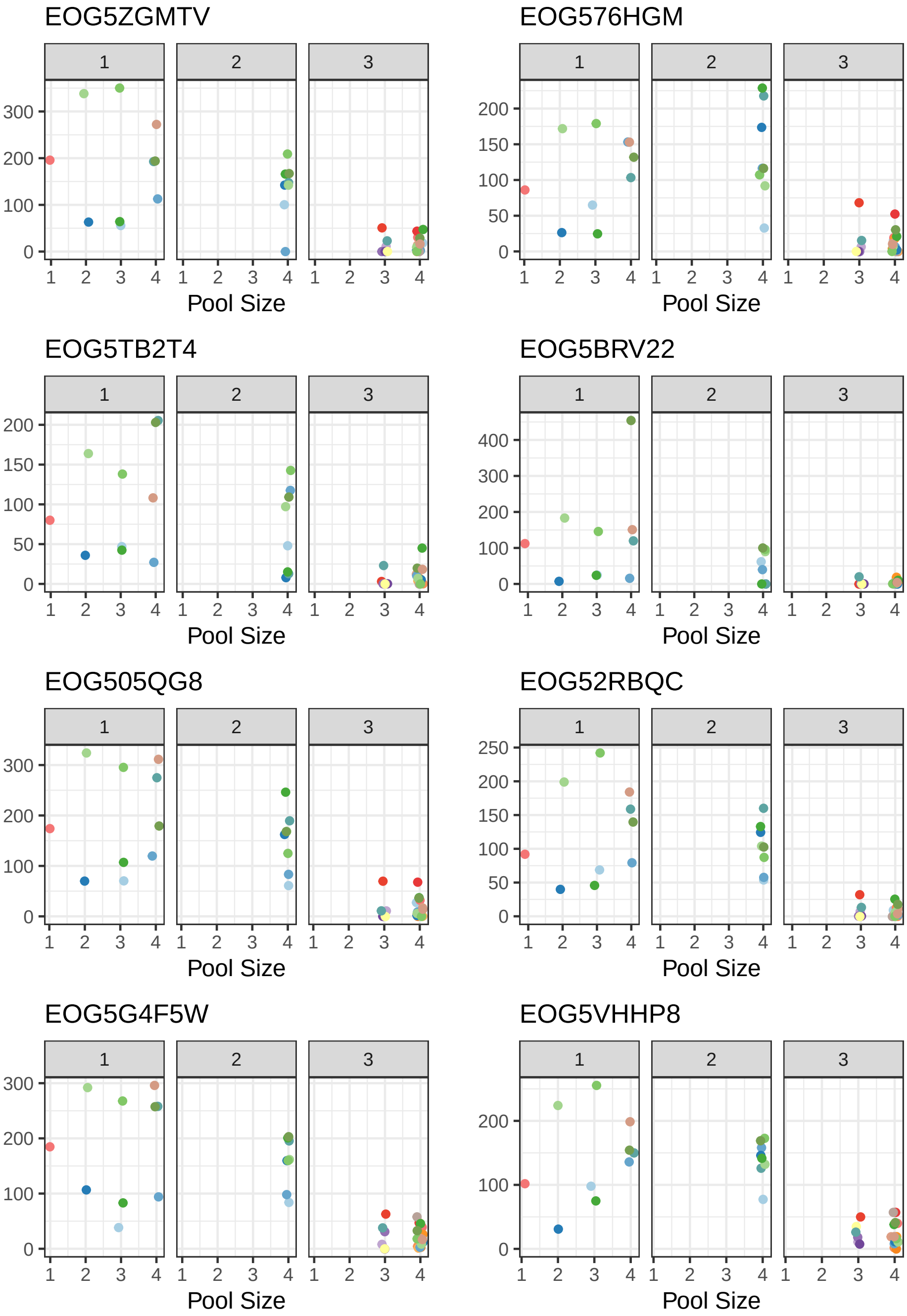

EOG5ZGMTV
1
2
3
300
200
100
0
1 2 3 4 1 2 3 4 1 2 3 4
Pool Size
EOG576HGM
1
2
3
200
150
100
50
0
1 2 3 4 1 2 3 4 1 2 3 4
Pool Size
EOG5TB2T4
1
2
3
200
150
100
50
0
1 2 3 4 1 2 3 4 1 2 3 4
Pool Size
EOG5BRV22
1
2
3
400
300
200
100
0
1 2 3 4 1 2 3 4 1 2 3 4
Pool Size
EOG505QG8
1
2
3
300
200
100
0
1 2 3 4 1 2 3 4 1 2 3 4
Pool Size
EOG52RBQC
1
2
3
250
200
150
100
50
0
1 2 3 4 1 2 3 4 1 2 3 4
Pool Size
EOG5G4F5W
1
2
3
300
200
100
0
1 2 3 4 1 2 3 4 1 2 3 4
Pool Size
EOG5VHHP8
1
2
3
200
100
0
1 2 3 4 1 2 3 4 1 2 3 4
Pool Size

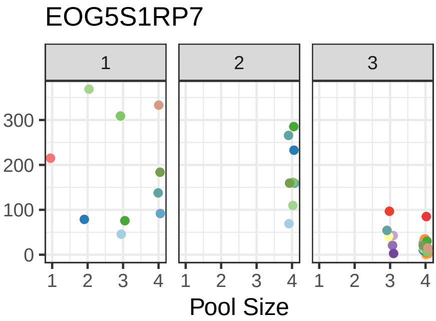
EOG5S1RP7
1
2
3
300
200
100
0
1 2 3 4 1 2 3 4 1 2 3 4
Pool Size
