# Supplementary material for: Development and evaluation of a custom bait design based on 469 single-copy protein-coding genes for exon capture of isopods (Philosciidae: Haloniscus)
Source: PLoS One. 2021 Sep 17;16(9):e0256861. doi: 10.1371/journal.pone.0256861 (PMC8448321; doi:10.1371/journal.pone.0256861)
Supplement: S7 Fig — The ID of each OG is specified above plots, and points are coloured according to sequencing ID. (PDF) [file pone.0256861.s007.pdf]

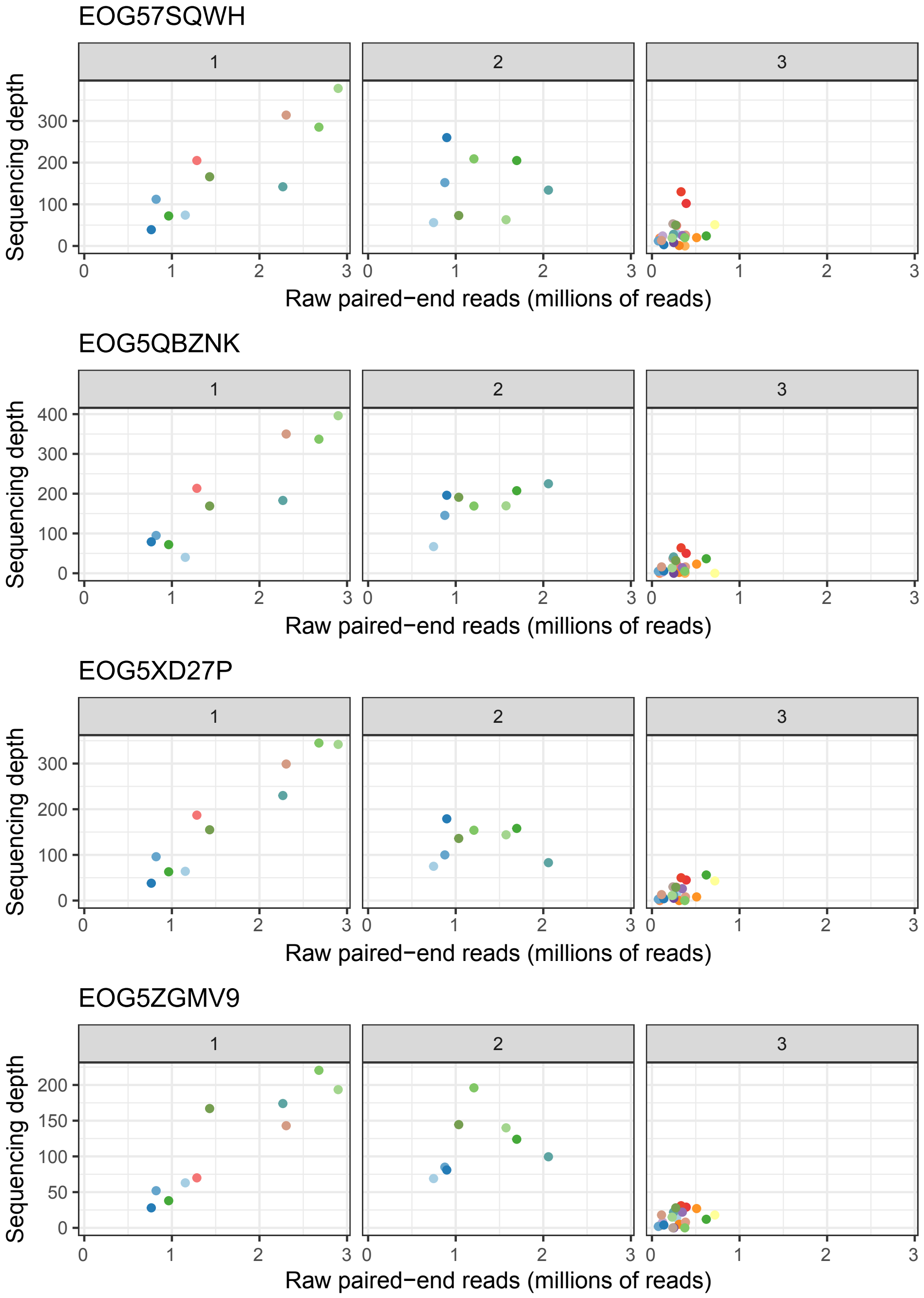
EOG57SQWH
1
2
3
Sequencing depth
Raw paired−end reads (millions of reads)
EOG5QBZNK
1
2
3
Sequencing depth
Raw paired−end reads (millions of reads)
EOG5XD27P
1
2
3
Sequencing depth
Raw paired−end reads (millions of reads)
EOG5ZGMV9
1
2
3
Sequencing depth
Raw paired−end reads (millions of reads)

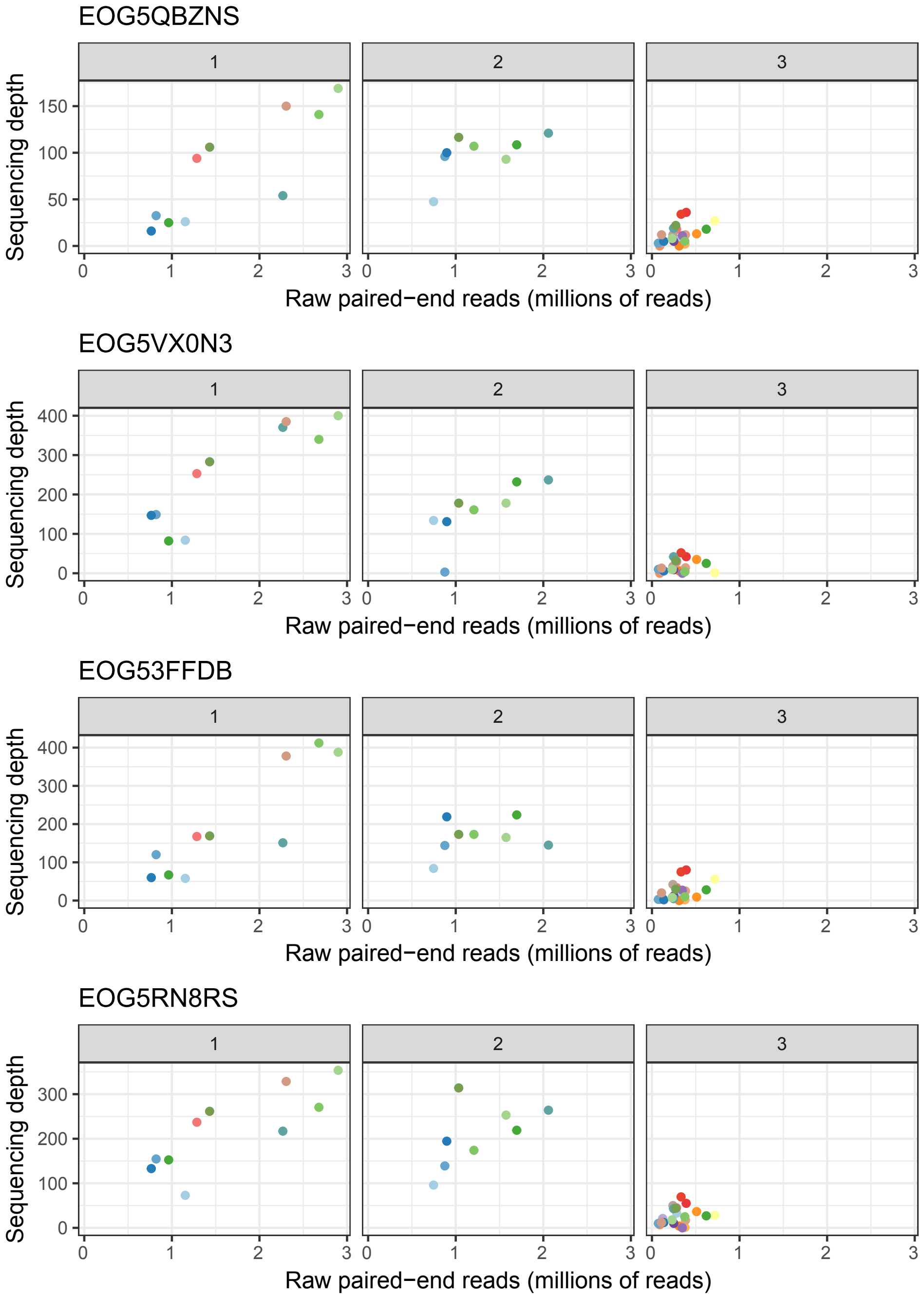
EOG5QBZNS
EOG5VX0N3
EOG53FFDB
EOG5RN8RS
1
2
3
Sequencing depth
Raw paired−end reads (millions of reads)

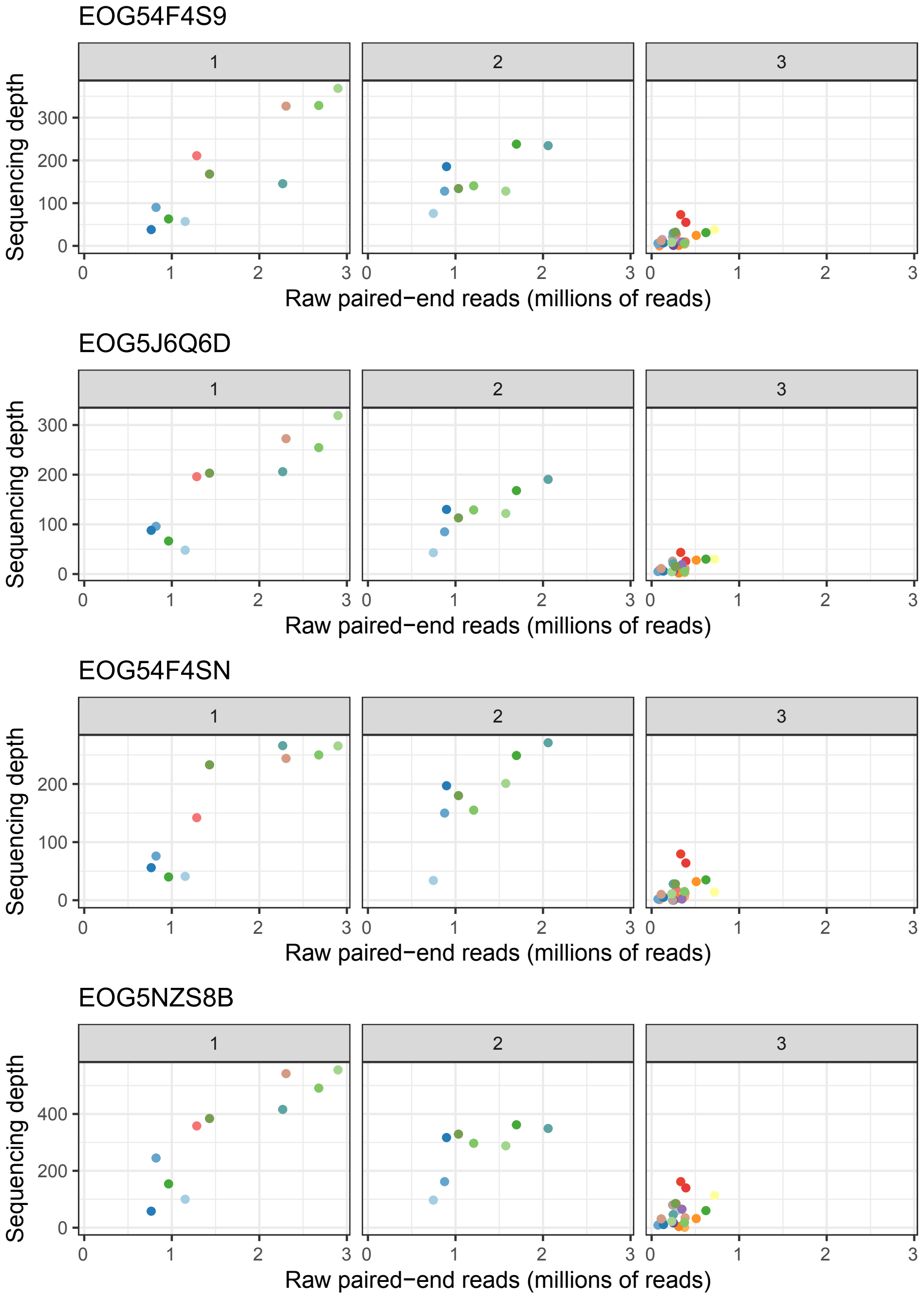
EOG54F4S9
EOG5J6Q6D
EOG54F4SN
EOG5NZS8B
1
2
3
Sequencing depth
Raw paired−end reads (millions of reads)
0
100
200
300
400

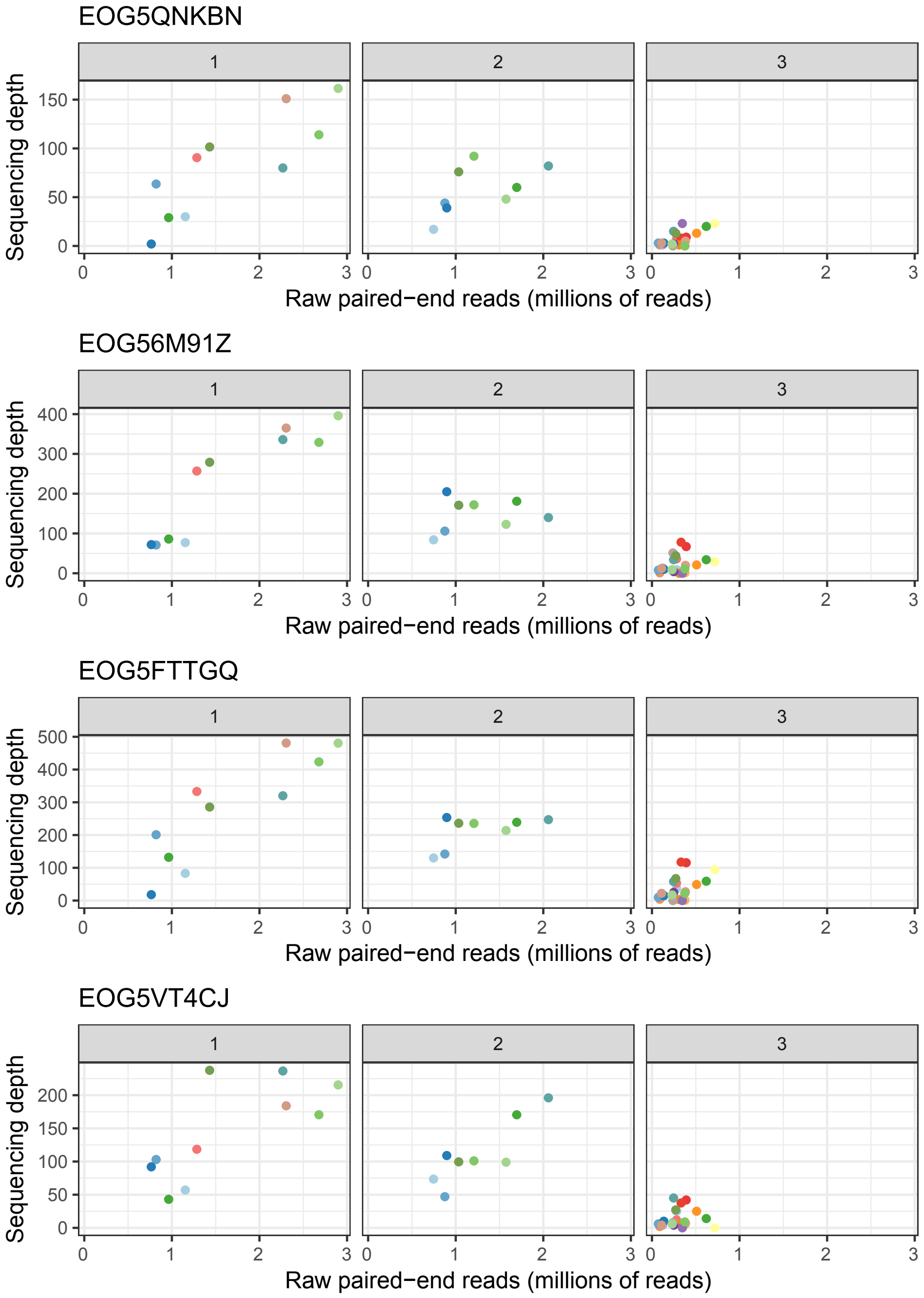
EOG5QNKBN
EOG56M91Z
EOG5FTTGQ
EOG5VT4CJ
1
2
3
Sequencing depth
Raw paired−end reads (millions of reads)

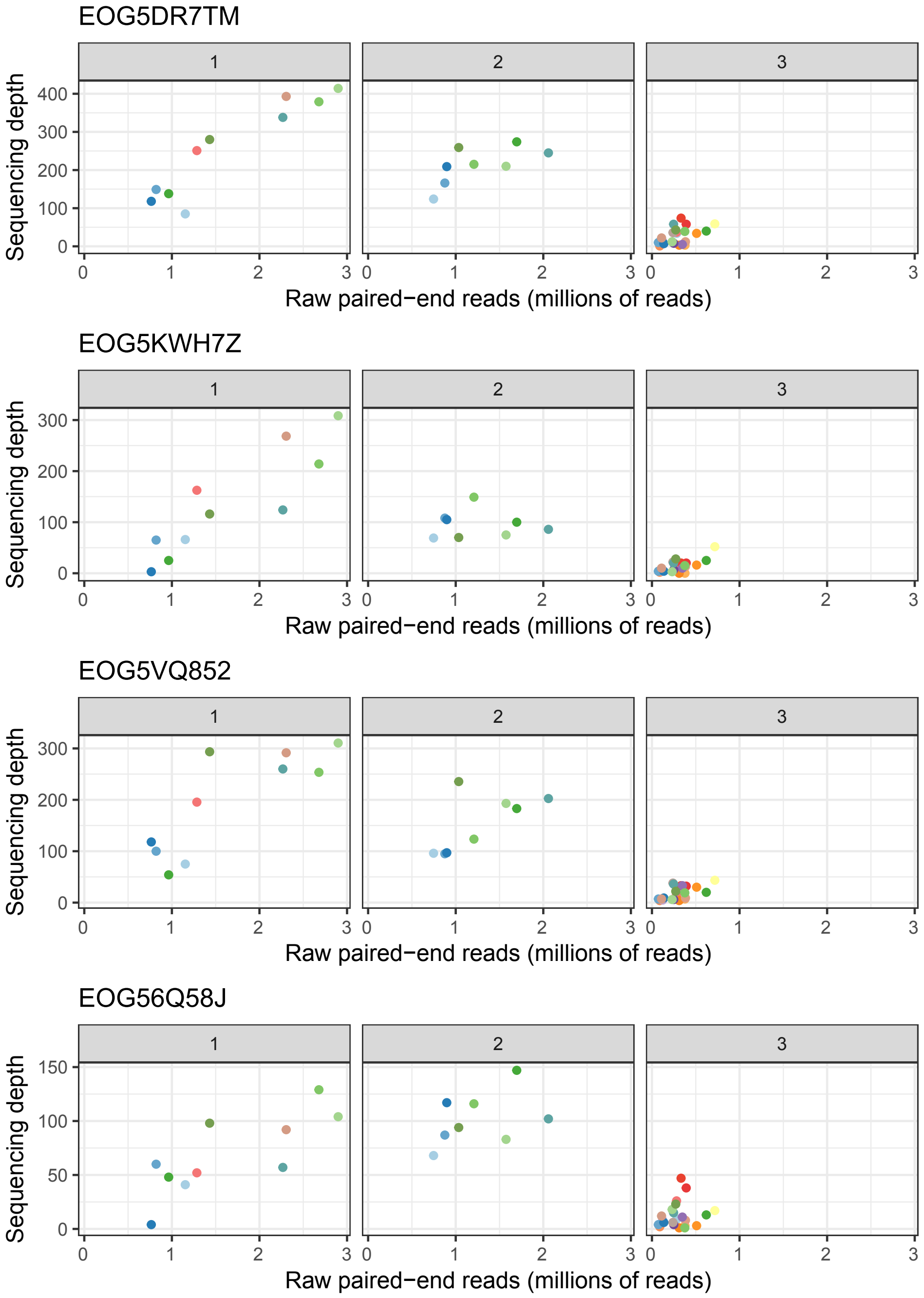
EOG5DR7TM
EOG5KWH7Z
EOG5VQ852
EOG56Q58J
1
2
3
Sequencing depth
Raw paired−end reads (millions of reads)

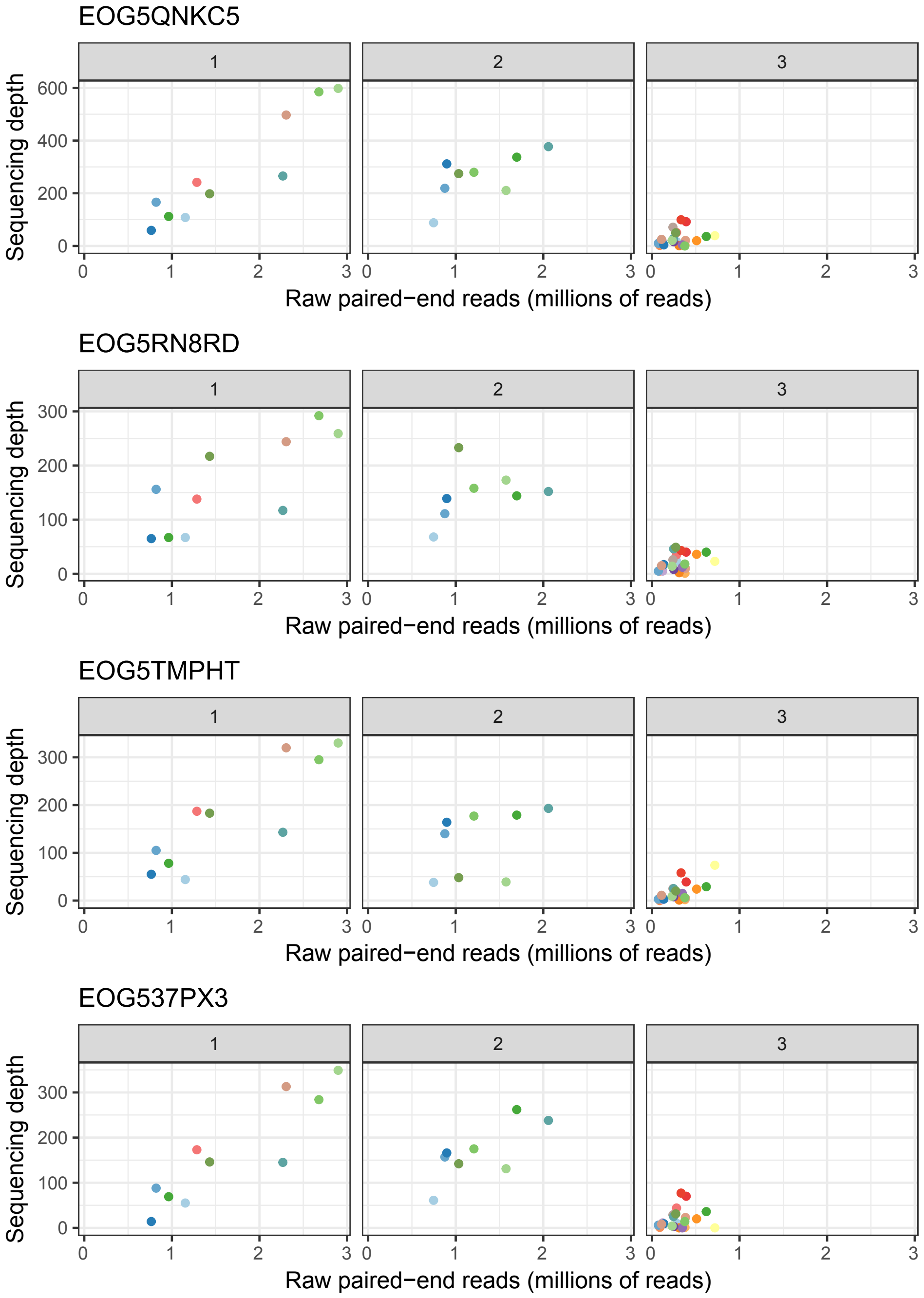
EOG5QNKC5
EOG5RN8RD
EOG5TMPHT
EOG537PX3
1
2
3
Sequencing depth
Raw paired−end reads (millions of reads)

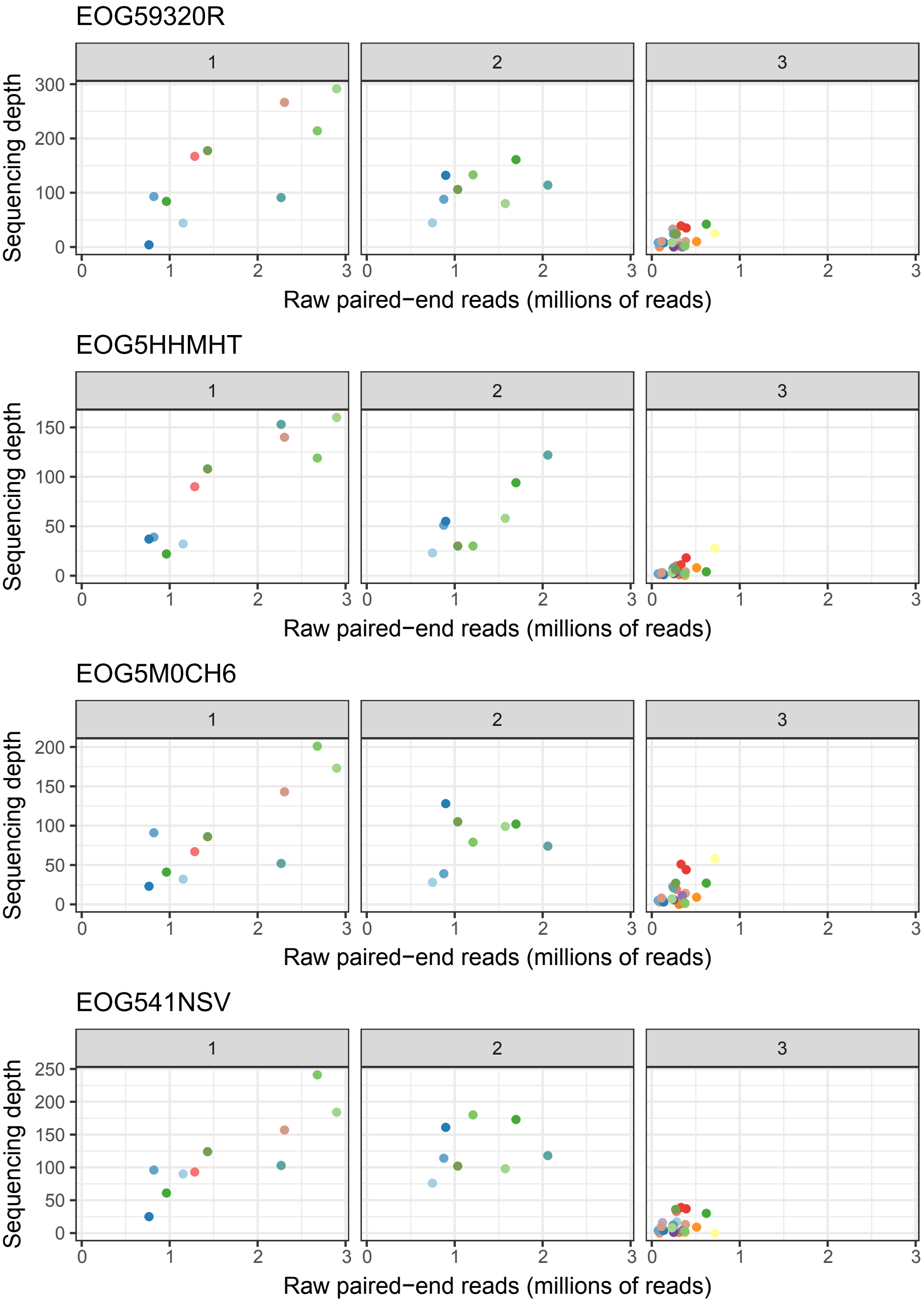
EOG59320R
1
2
3
Sequencing depth
0
100
200
300
0
1
2
3
Raw paired−end reads (millions of reads)
EOG5HHMHT
1
2
3
Sequencing depth
0
50
100
150
0
1
2
3
Raw paired−end reads (millions of reads)
EOG5M0CH6
1
2
3
Sequencing depth
0
50
100
150
200
0
1
2
3
Raw paired−end reads (millions of reads)
EOG541NSV
1
2
3
Sequencing depth
0
50
100
150
200
250
0
1
2
3
Raw paired−end reads (millions of reads)

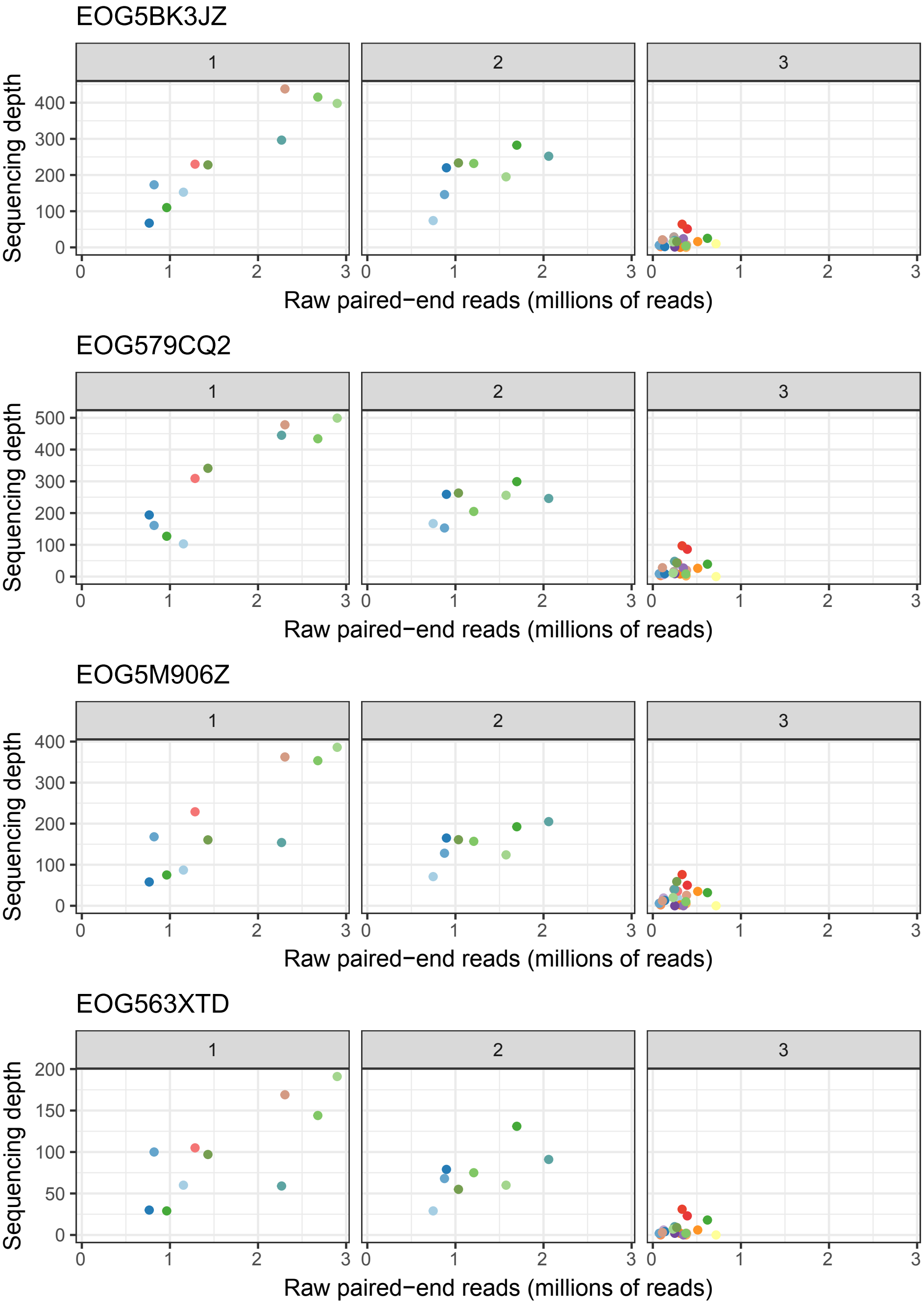
EOG5BK3JZ
1
2
3
Sequencing depth
0
100
200
300
400
0
1
2
3
Raw paired−end reads (millions of reads)
EOG579CQ2
1
2
3
Sequencing depth
0
100
200
300
400
500
Raw paired−end reads (millions of reads)
EOG5M906Z
1
2
3
Sequencing depth
0
100
200
300
400
Raw paired−end reads (millions of reads)
EOG563XTD
1
2
3
Sequencing depth
0
50
100
150
200
Raw paired−end reads (millions of reads)

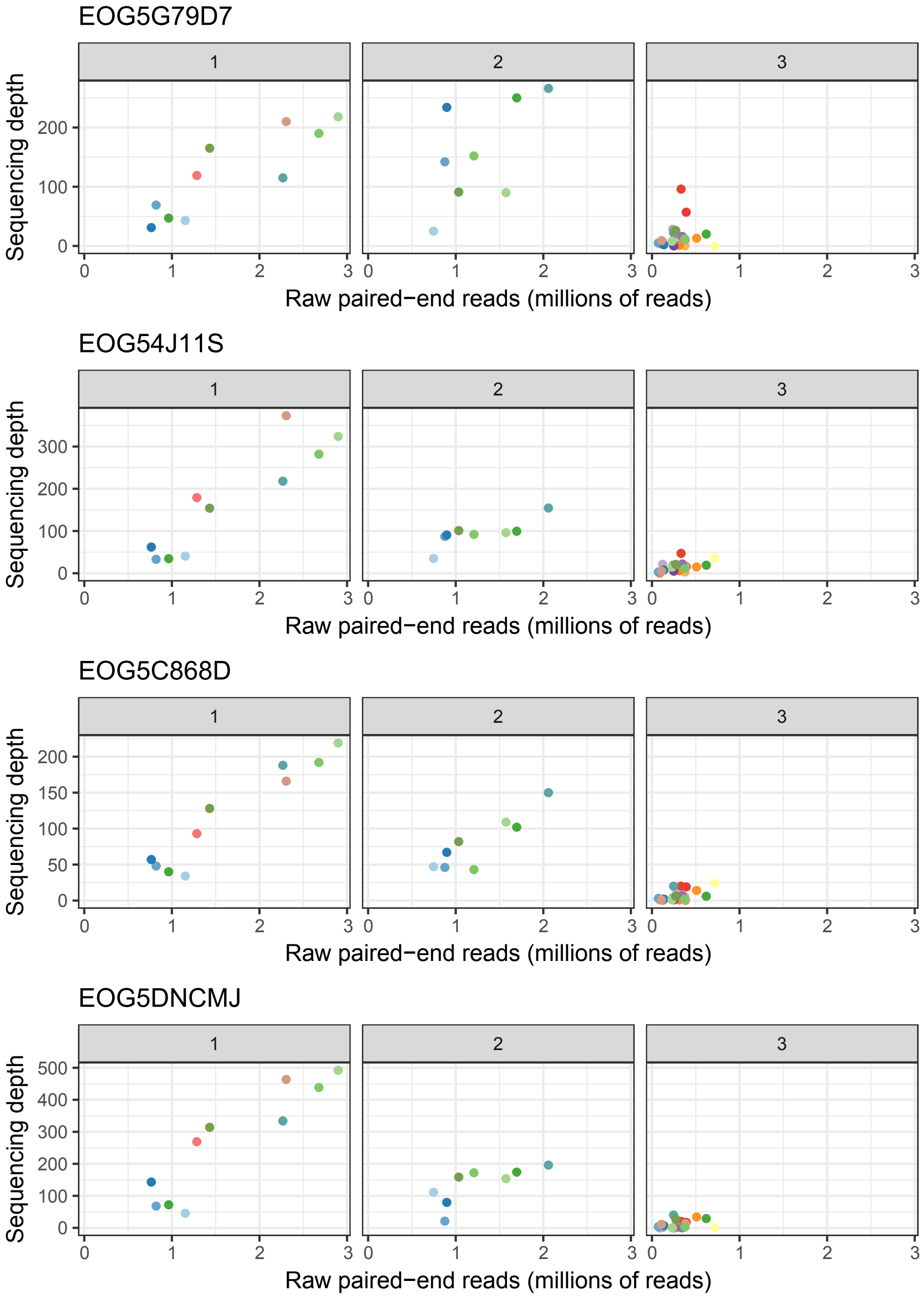

EOG5G79D7
EOG54J11S
EOG5C868D
EOG5DNCMJ
1
2
3
Sequencing depth
Raw paired−end reads (millions of reads)

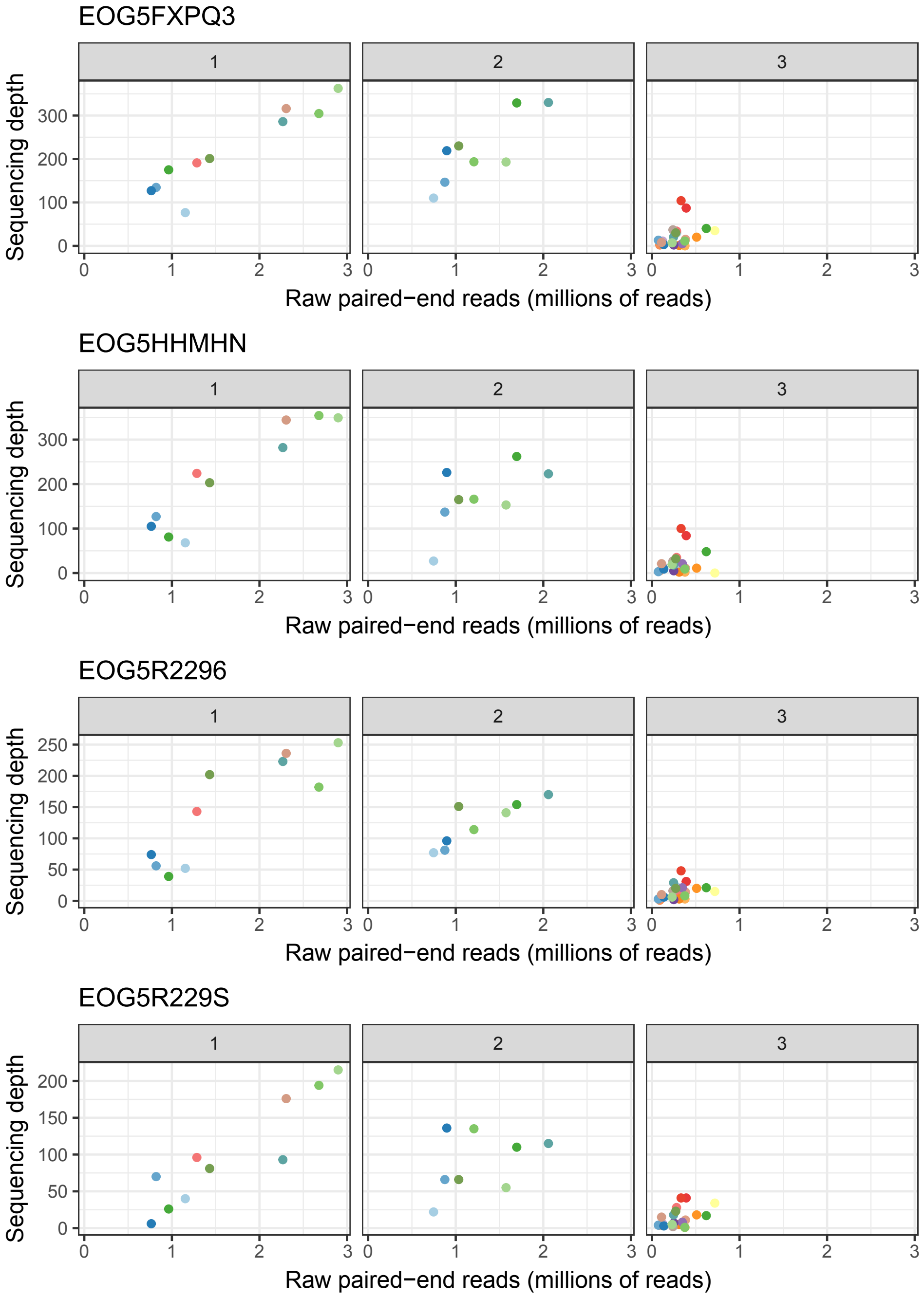
EOG5FXPQ3
EOG5HHMHN
EOG5R2296
EOG5R229S
1
2
3
Sequencing depth
Raw paired−end reads (millions of reads)

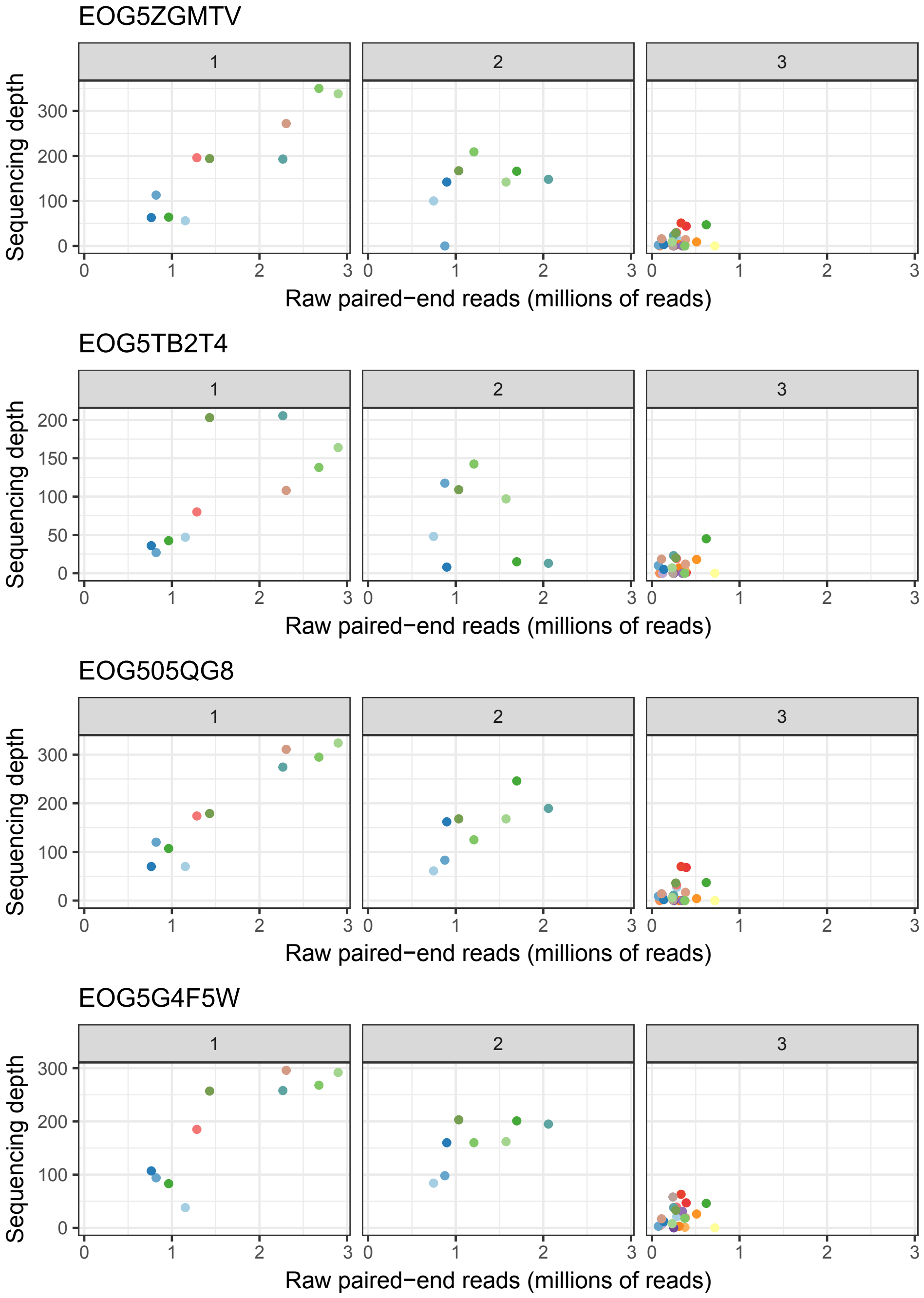
EOG5ZGMTV
EOG5TB2T4
EOG505QG8
EOG5G4F5W
1
2
3
Sequencing depth
Raw paired−end reads (millions of reads)
0
1
2
3
0
100
200
300
50
150

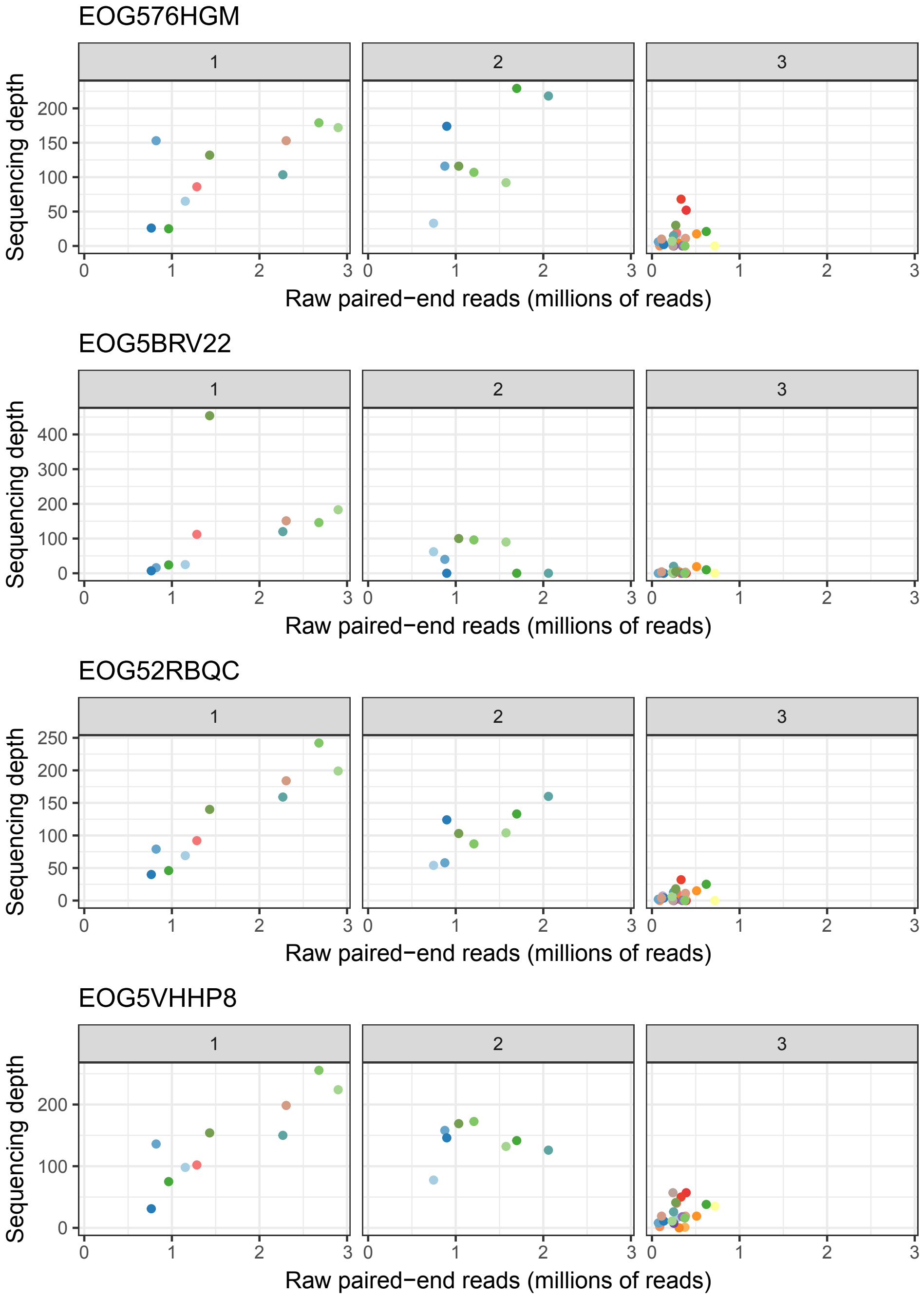
EOG576HGM
EOG5BRV22
EOG52RBQC
EOG5VHHP8
1
2
3
Sequencing depth
Raw paired−end reads (millions of reads)

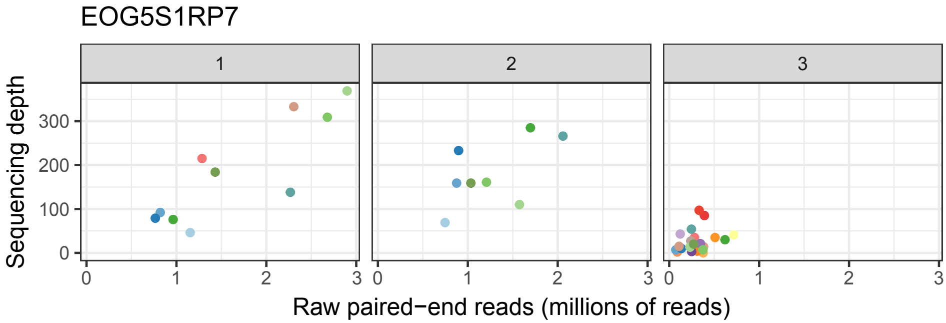

EOG5S1RP7
1
2
3
Sequencing depth
300
200
100
0
0
1
2
3
Raw paired−end reads (millions of reads)
