# Supplementary material for: Development and evaluation of a custom bait design based on 469 single-copy protein-coding genes for exon capture of isopods (Philosciidae: Haloniscus)
Source: PLoS One. 2021 Sep 17;16(9):e0256861. doi: 10.1371/journal.pone.0256861 (PMC8448321; doi:10.1371/journal.pone.0256861)
Supplement: S8 Fig — The ID of each OG is specified above plots, and points are coloured according to sequencing ID. (PDF) [file pone.0256861.s008.pdf]

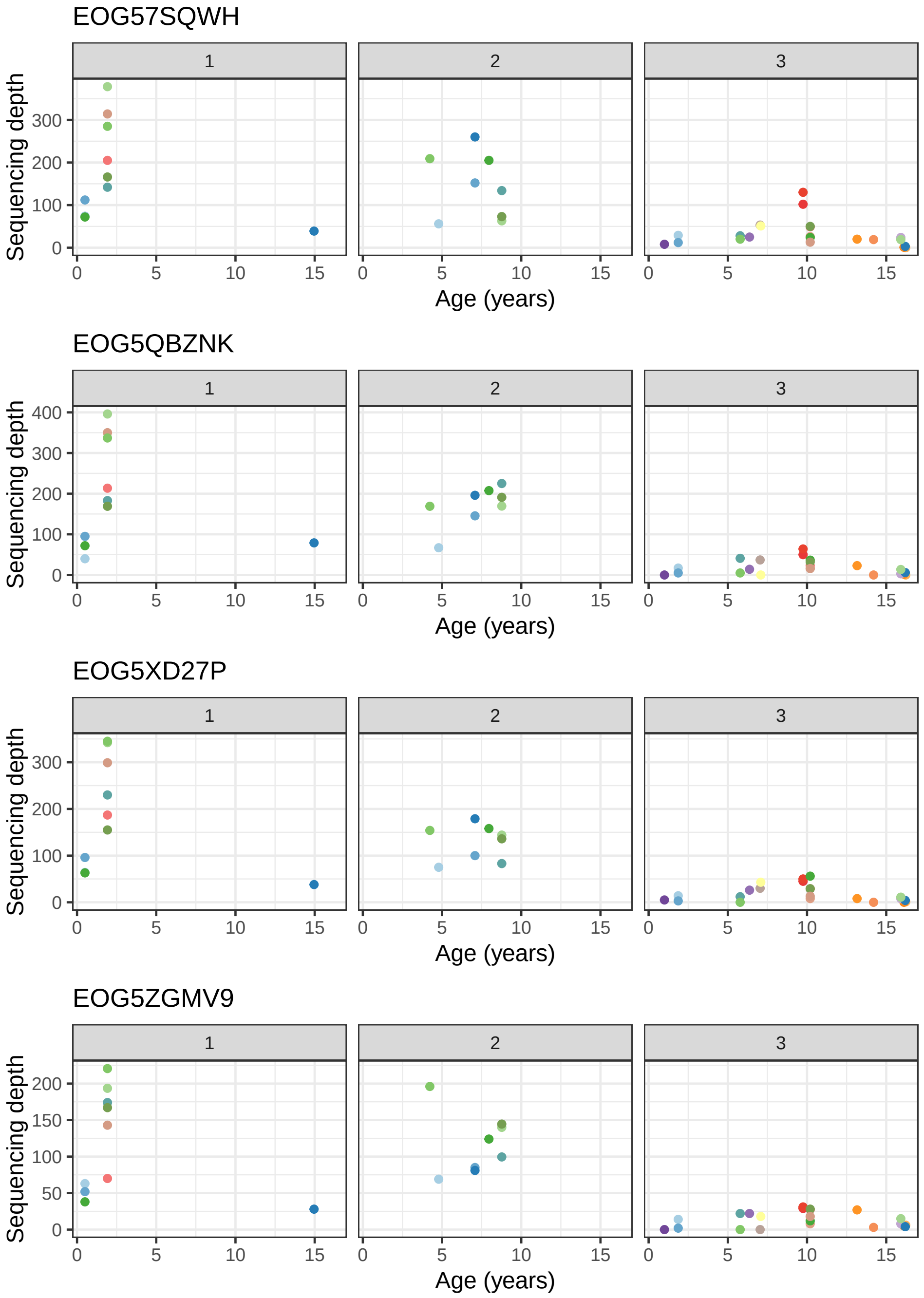
EOG57SQWH
EOG5QBZNK
EOG5XD27P
EOG5ZGMV9
1
2
3
Sequencing depth
Age (years)
0
5
10
15
0
50
100
150
200
300
400

EOG5QBZNS

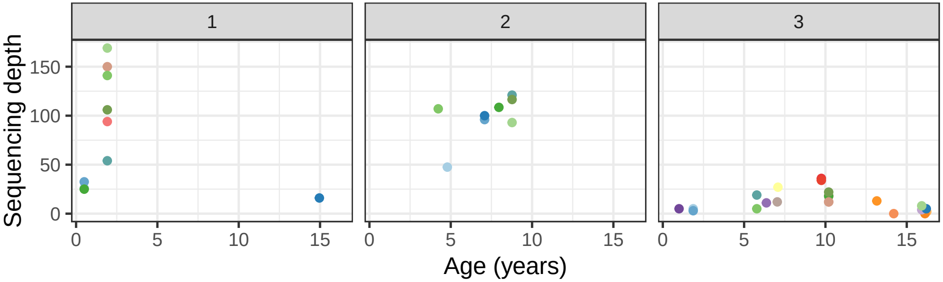

EOG5VX0N3

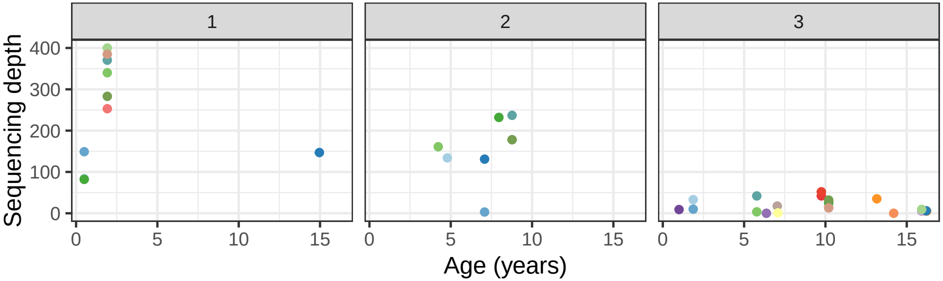

EOG53FFDB

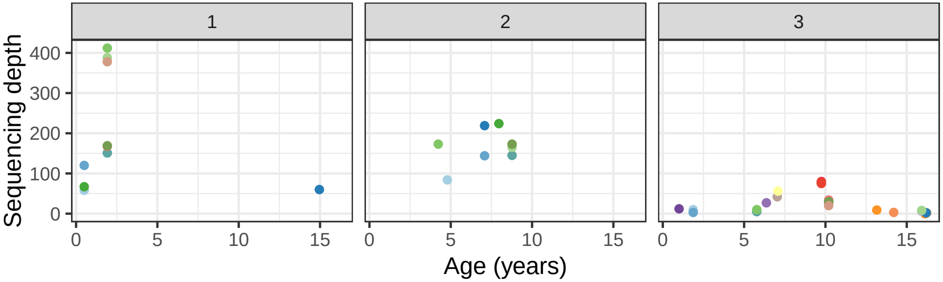

EOG5RN8RS

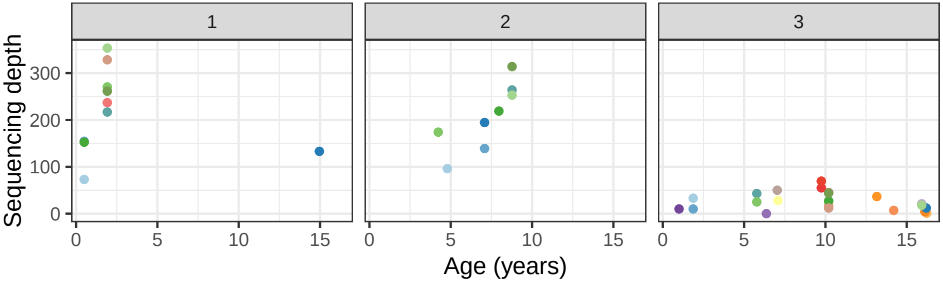

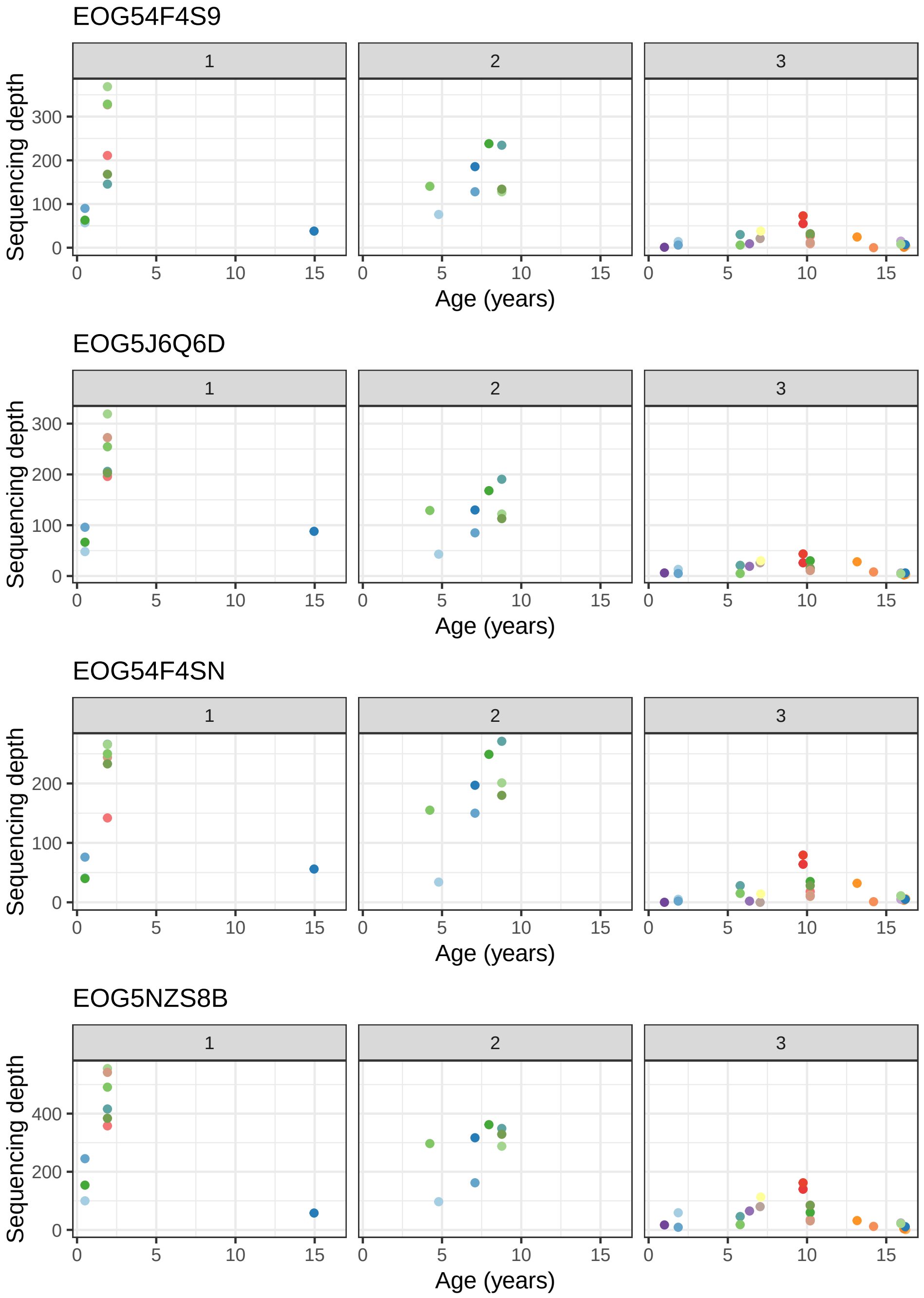
EOG54F4S9
EOG5J6Q6D
EOG54F4SN
EOG5NZS8B
1
2
3
Sequencing depth
Age (years)
0
5
10
15
100
200
300
400

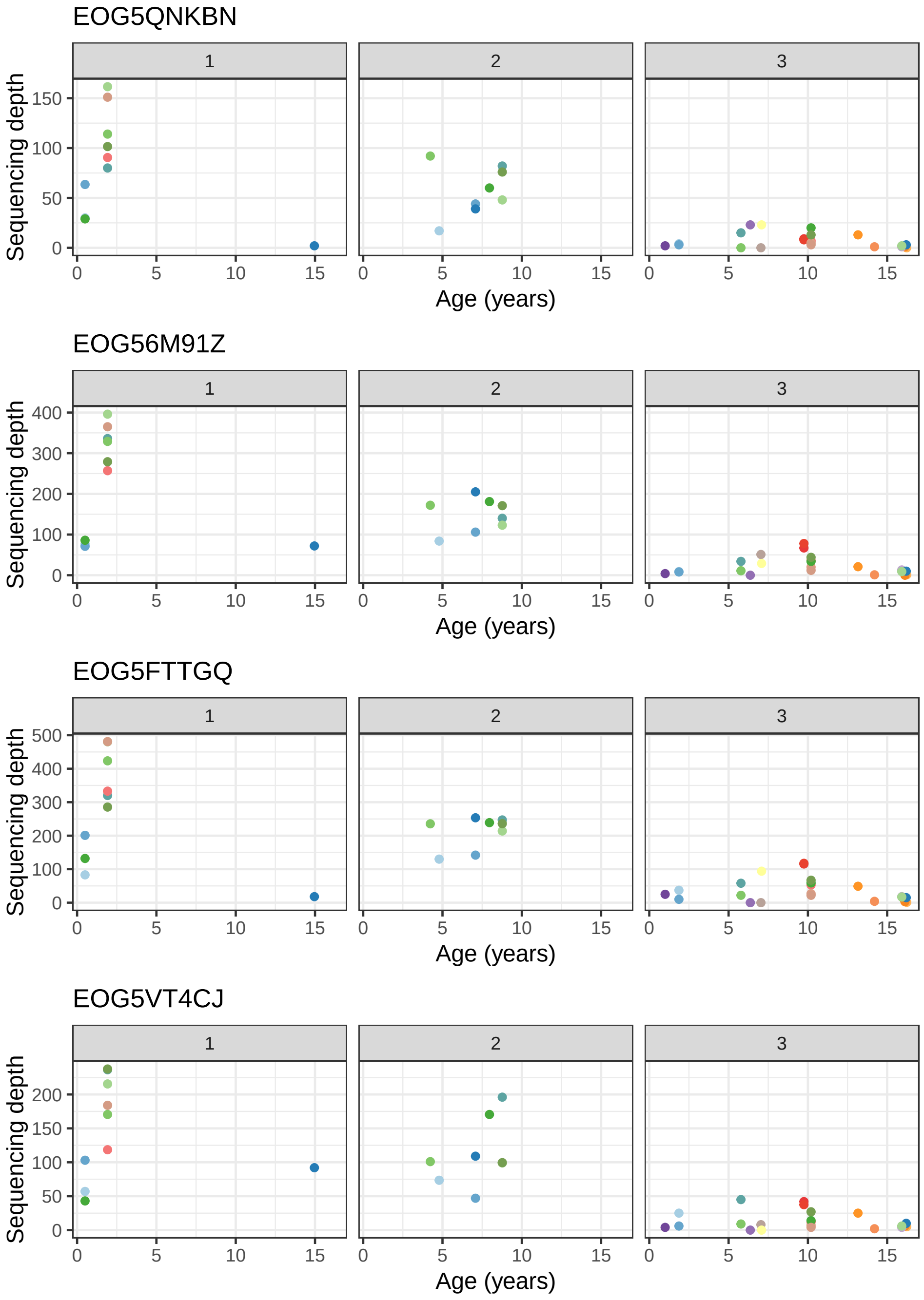
EOG5QNKBN
EOG56M91Z
EOG5FTTGQ
EOG5VT4CJ
1
2
3
Sequencing depth
Age (years)

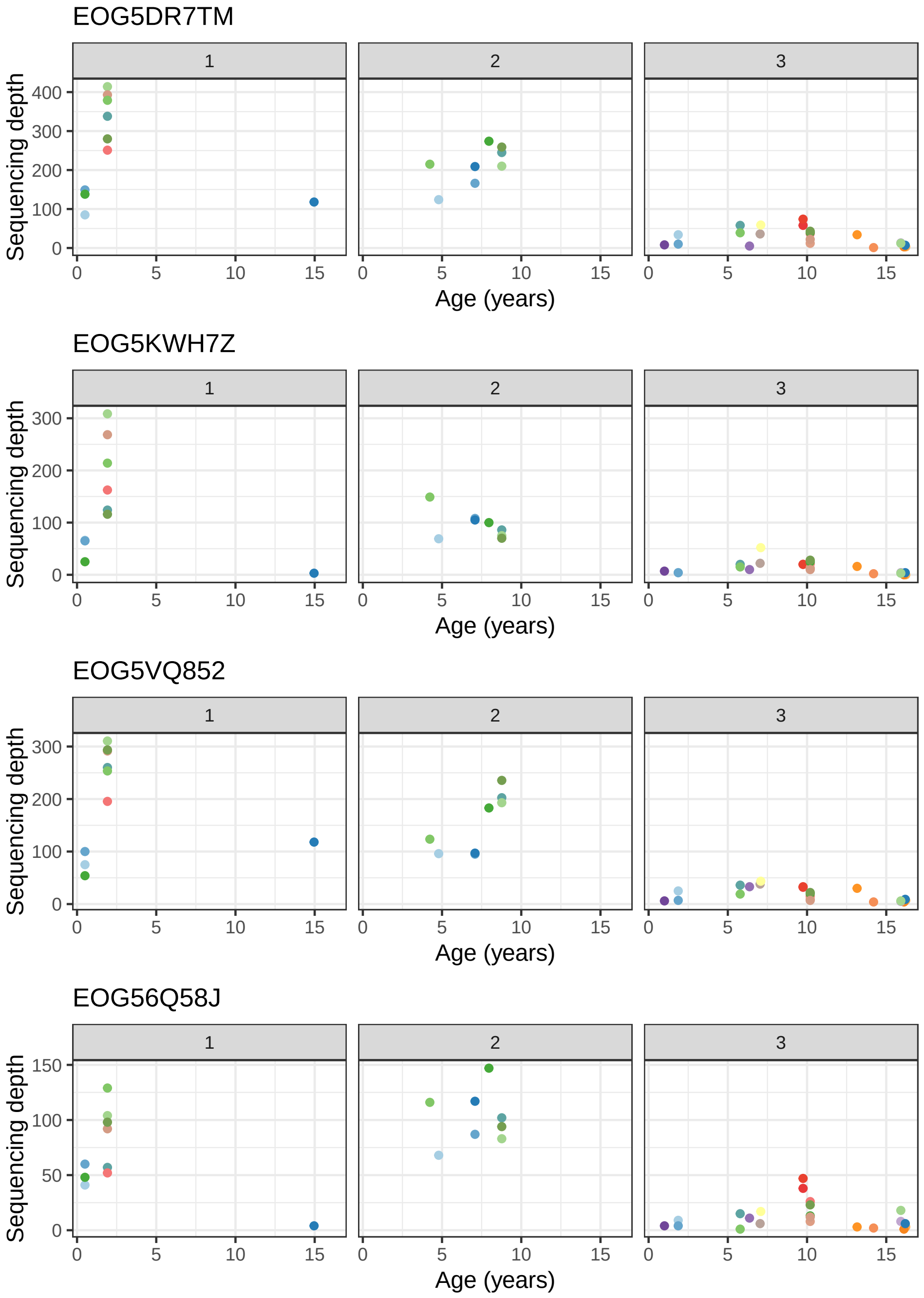
EOG5DR7TM
1
2
3
Sequencing depth
0
100
200
300
400
0
5
10
15
Age (years)
EOG5KWH7Z
1
2
3
Sequencing depth
0
100
200
300
0
5
10
15
Age (years)
EOG5VQ852
1
2
3
Sequencing depth
0
100
200
300
0
5
10
15
Age (years)
EOG56Q58J
1
2
3
Sequencing depth
0
50
100
150
0
5
10
15
Age (years)

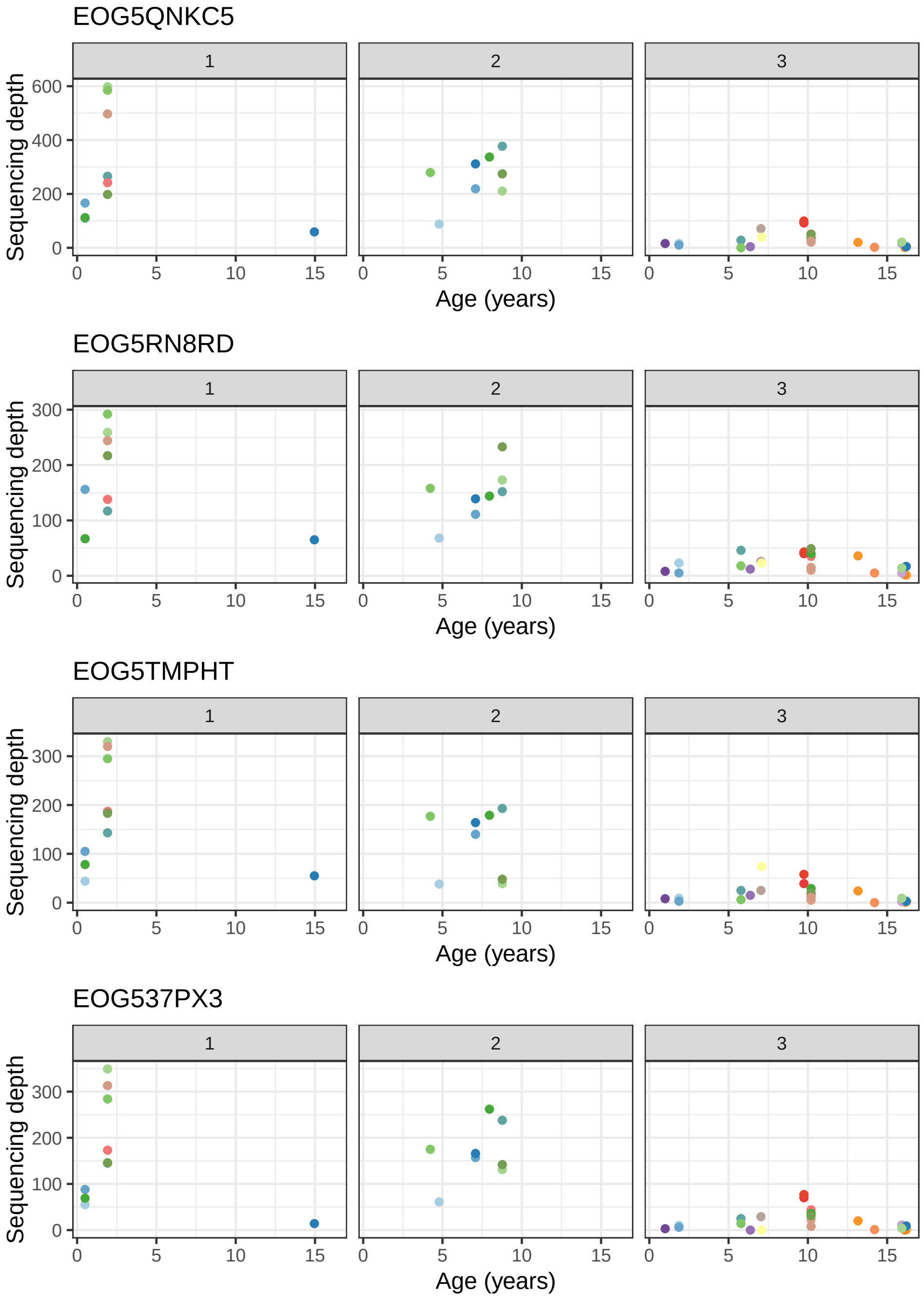
EOG5QNKC5
EOG5RN8RD
EOG5TMPHT
EOG537PX3
1
2
3
Sequencing depth
Age (years)
0
5
10
15
0
200
400
600
100
300

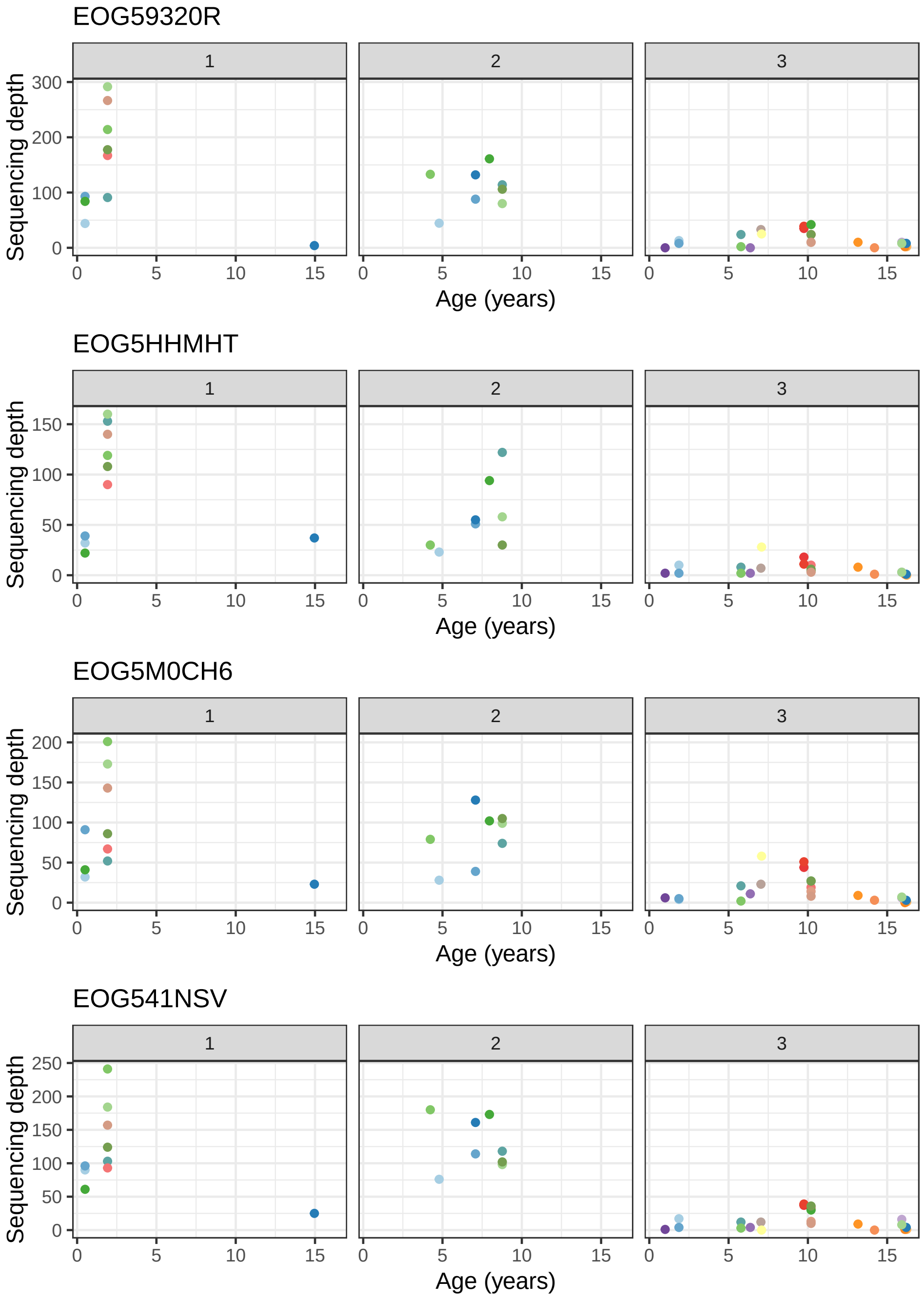
EOG59320R
1
2
3
Sequencing depth
300
200
100
0
0
5
10
15
Age (years)
EOG5HHMHT
1
2
3
Sequencing depth
150
100
50
0
0
5
10
15
Age (years)
EOG5M0CH6
1
2
3
Sequencing depth
200
150
100
50
0
0
5
10
15
Age (years)
EOG541NSV
1
2
3
Sequencing depth
250
200
150
100
50
0
0
5
10
15
Age (years)

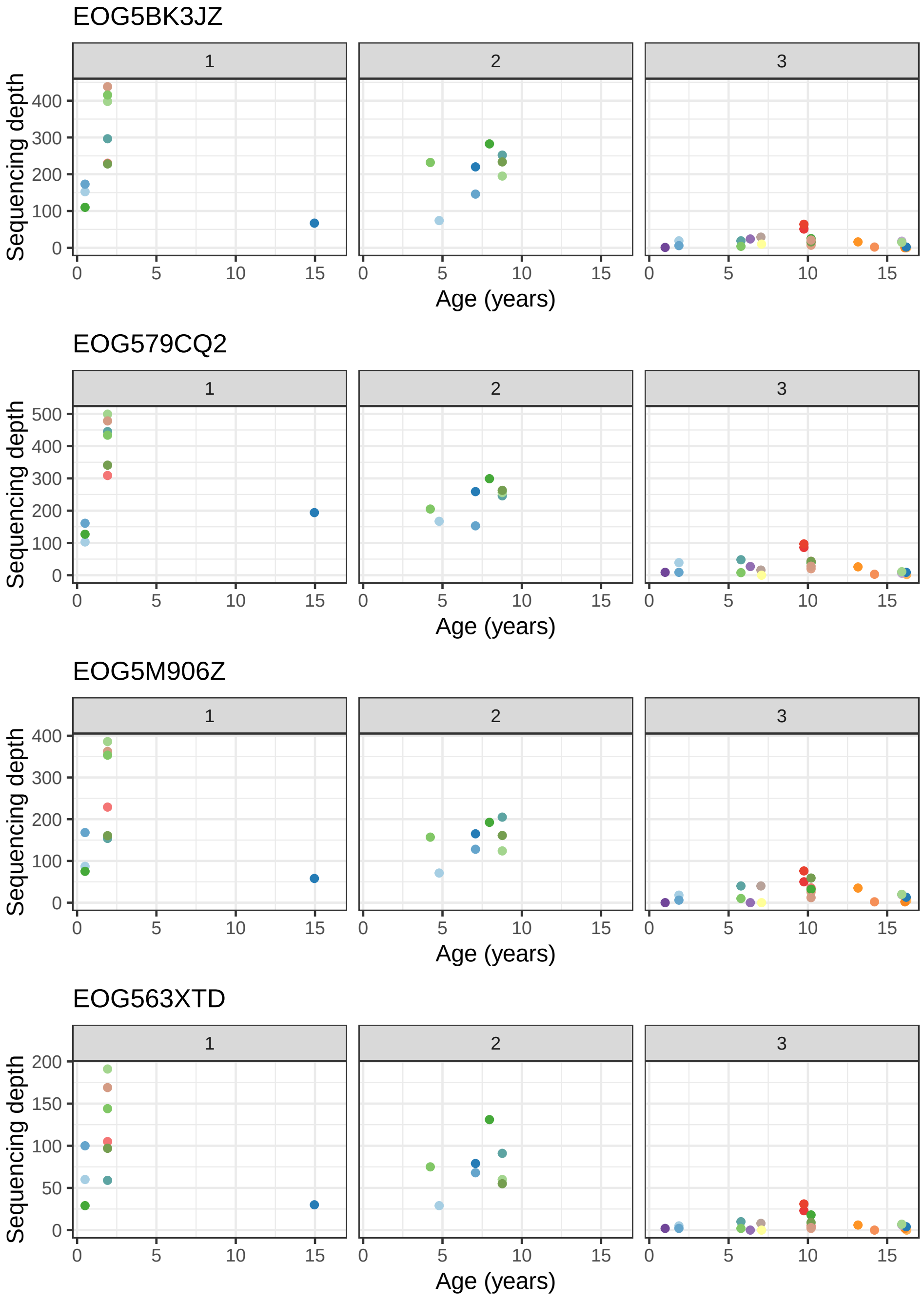
EOG5BK3JZ
EOG579CQ2
EOG5M906Z
EOG563XTD
1
2
3
Sequencing depth
Age (years)
0
5
10
15
0
100
200
300
400
500
0
50
100
150
200

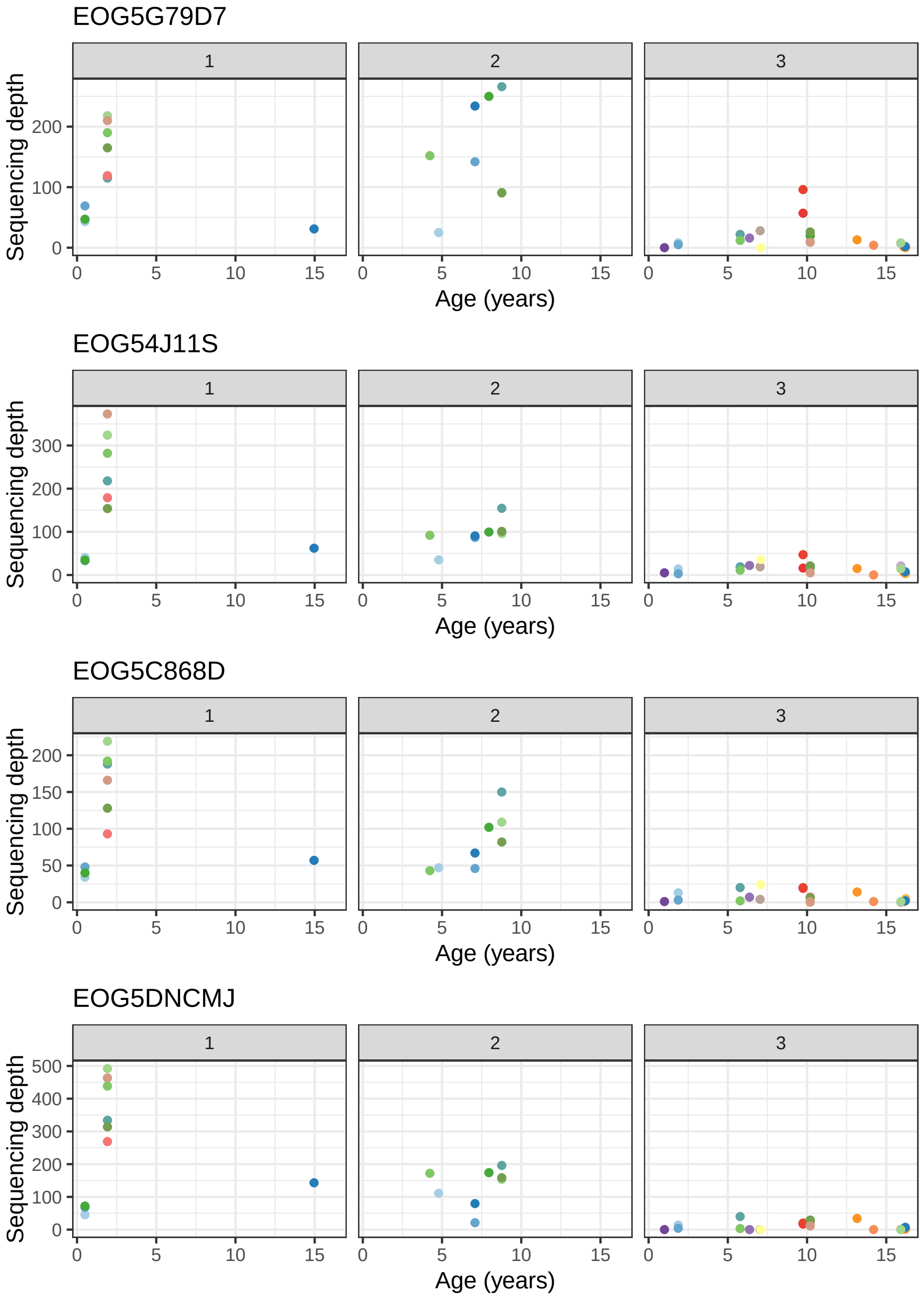

EOG5G79D7
EOG54J11S
EOG5C868D
EOG5DNCMJ
1
2
3
Sequencing depth
Age (years)
0
5
10
15
50
100
150
200
300
400
500

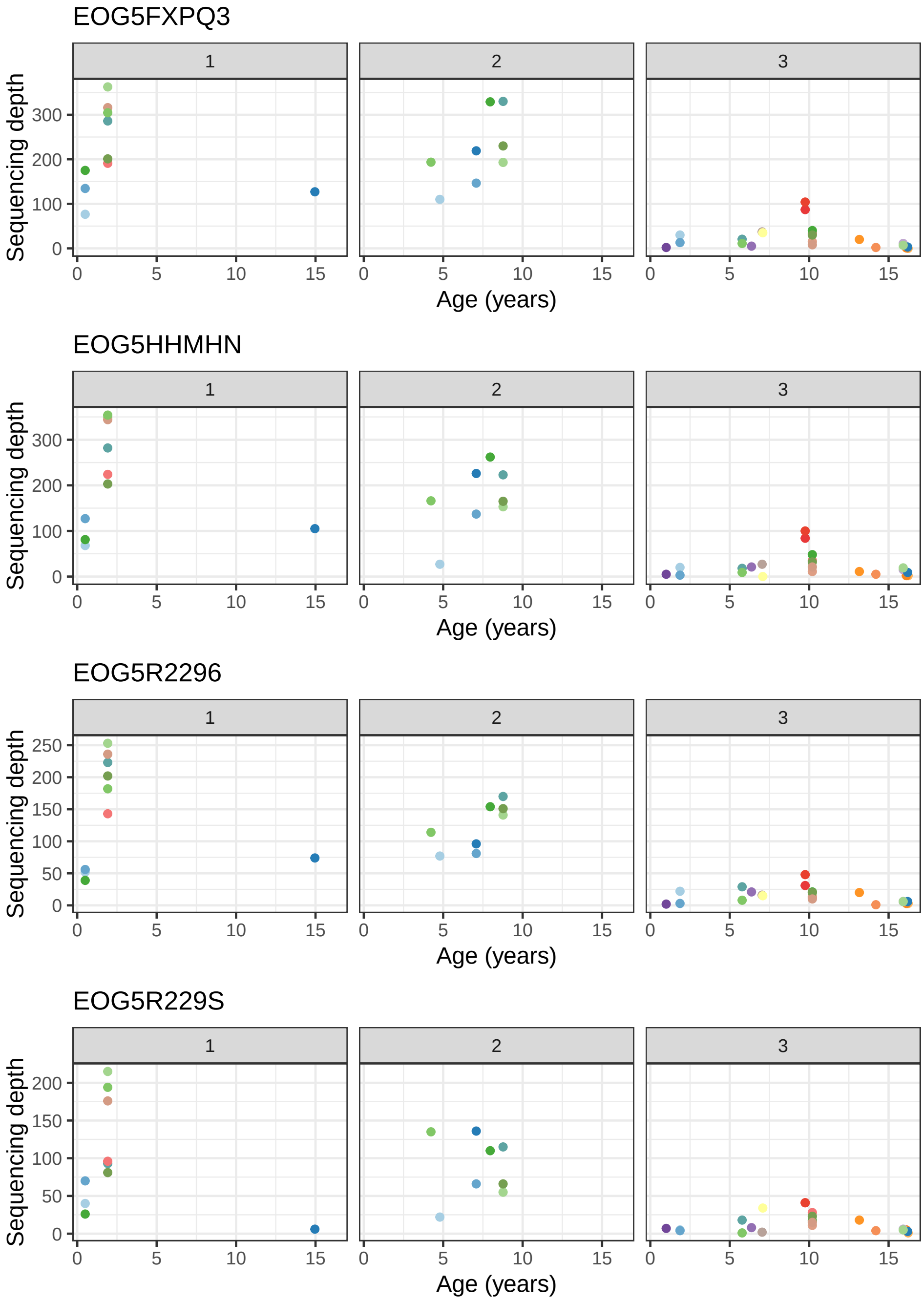
EOG5FXPQ3
1
2
3
Sequencing depth
0
100
200
300
0
5
10
15
Age (years)
EOG5HHMHN
1
2
3
Sequencing depth
0
100
200
300
0
5
10
15
Age (years)
EOG5R2296
1
2
3
Sequencing depth
0
50
100
150
200
250
0
5
10
15
Age (years)
EOG5R229S
1
2
3
Sequencing depth
0
50
100
150
200
0
5
10
15
Age (years)

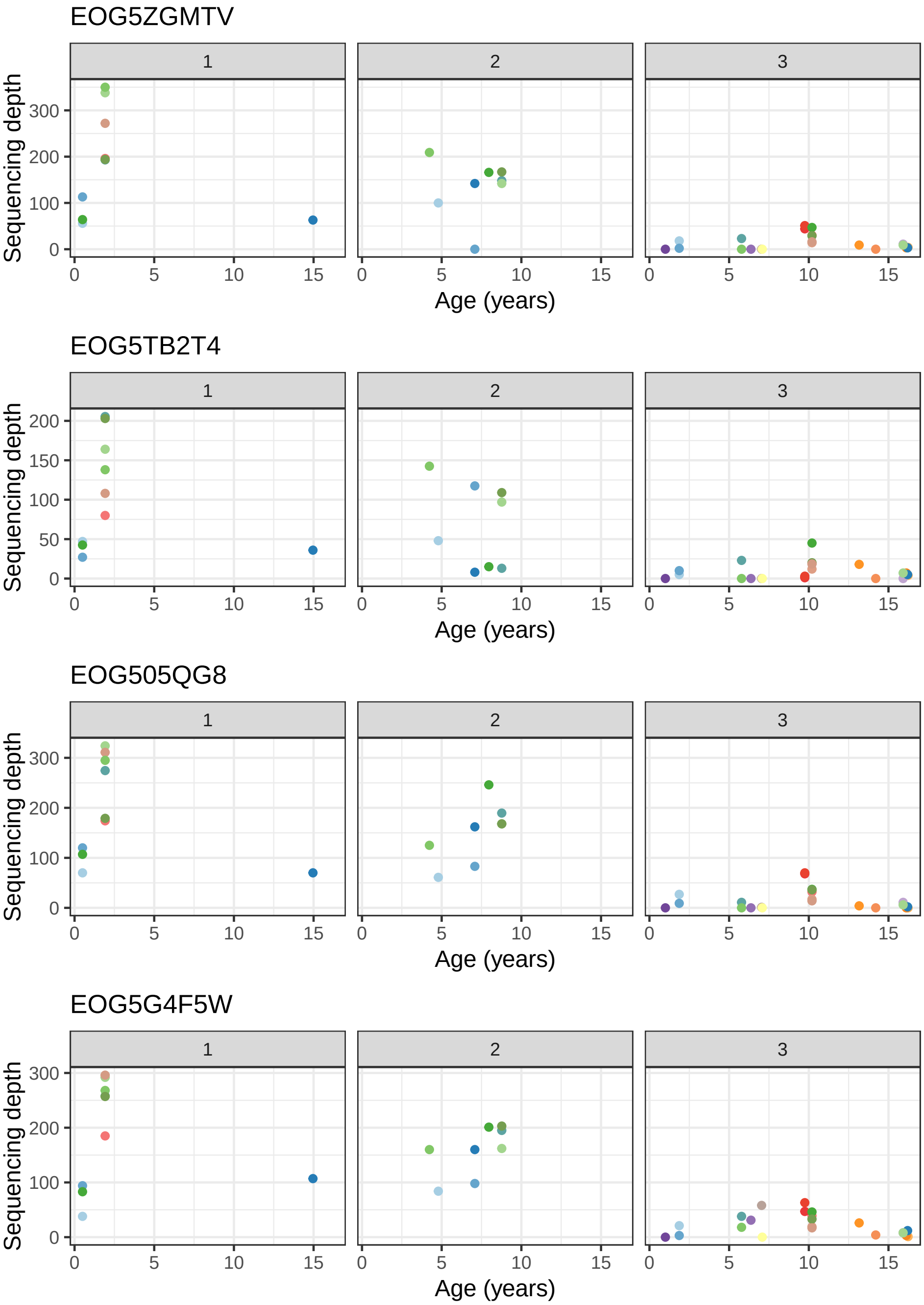
EOG5ZGMTV
EOG5TB2T4
EOG505QG8
EOG5G4F5W
1
2
3
Sequencing depth
Age (years)
0
5
10
15
50
100
150
200
300

EOG576HGM

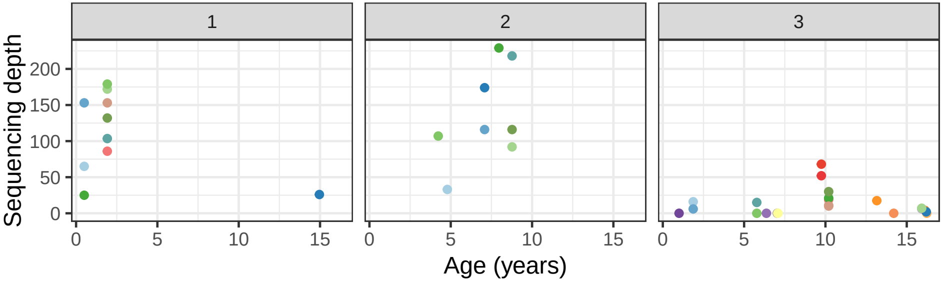

EOG5BRV22

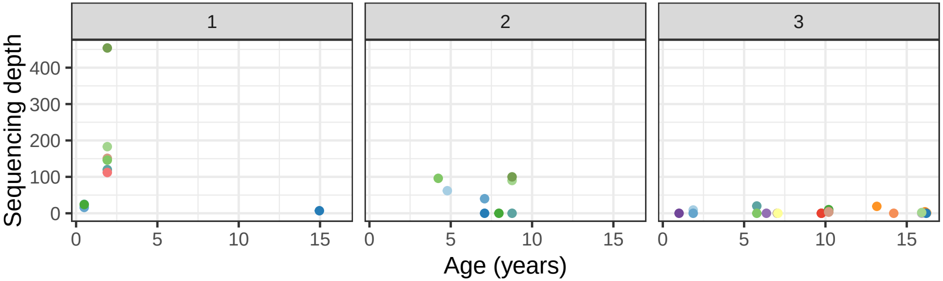

EOG52RBQC

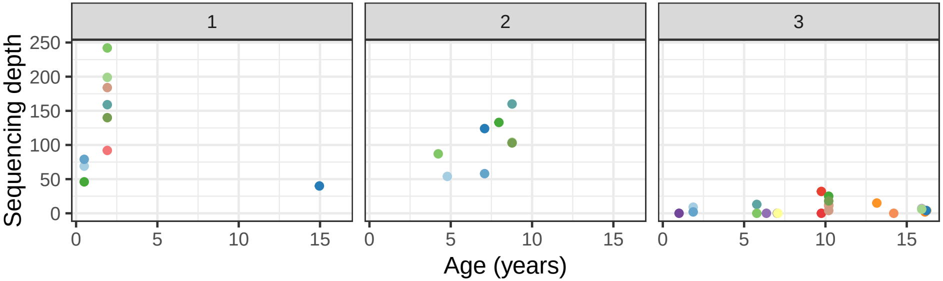

EOG5VHHP8

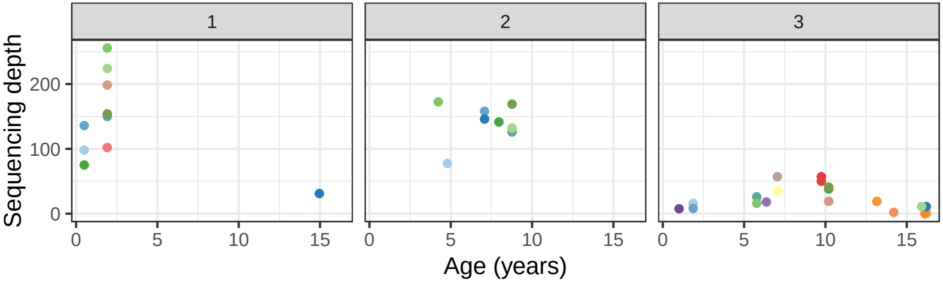

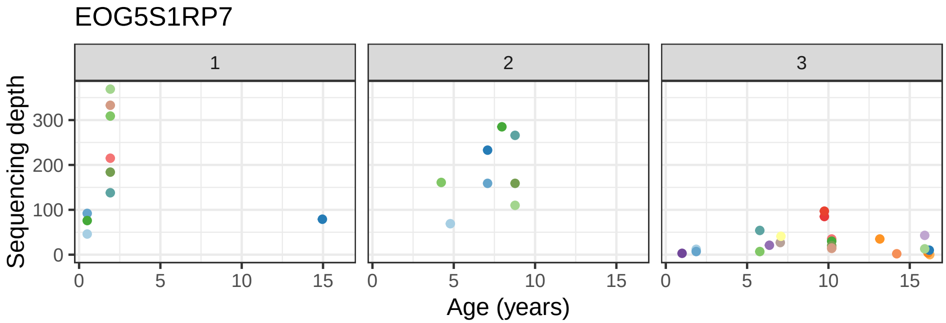
EOG5S1RP7
1
2
3
Sequencing depth
300
200
100
0
0
5
10
15
Age (years)
