# Supplementary material for: Development and evaluation of a custom bait design based on 469 single-copy protein-coding genes for exon capture of isopods (Philosciidae: Haloniscus)
Source: PLoS One. 2021 Sep 17;16(9):e0256861. doi: 10.1371/journal.pone.0256861 (PMC8448321; doi:10.1371/journal.pone.0256861)
Supplement: S1 File — (DOCX) [file pone.0256861.s012.docx]

**S1 File. Continued details for transcriptome sequencing and contig assembly methods.**

For the *Paraplatyarthrus* sp., *P. subterraneus* and *P. pruinosus* raw transcript reads, Tagcleaner v.0.12 [1] was used to trim SMARTer II adapters, and Trimmomatic v0.22 [2] was utilised to remove further adapters (Illumina sequencing adapters), along with long and short poly A and T tails with the module, ILLUMINACLP. Reads <30 bp post-trimming were discarded, resulting in paired and unpaired fastq files. Transcripts were then *de novo* assembled with Trinity v2012-06-18 [3,4] with default settings on a Dell PowerEdge R910 server using 512GB RAM.

For *Ceratothoa* sp. and *Armadillidium vulgare*, raw transcript reads were quality and adapter trimmed with Trimmomatic v0.32 [2] with ILLUMINACLIP. Reads were then assembled with IDBA-Tran v1.1.1 [5] with --mink 20 --maxk 60 --step 5.

**References**

1. Schmieder R, Lim YW, Rohwer F, Edwards R. TagCleaner: Identification and removal of tag sequences from genomic and metagenomics datasets. BMC Bioinformatics. 2010; 11: 341. doi: 10.1186/1471-2105-11-341
2. Bolger AM, Lohse M, Usadel B. Trimmomatic: a flexible trimmer for Illumina sequence data. Bioinformatics. 2014; 30: 2114–2120. doi: 10.1093/bioinformatics/btu170
3. Grabherr MG, Haas BJ, Yassour M, Levin JZ, Thompson DA, Amit I, et al. Full-length transcriptome assembly from RNA-Seq data without a reference genome. Nat Biotechnol. 2011; 15: 644–652. doi: 10.1038/nbt.1883
4. Haas BJ, Papanicolaou A, Yassour M, Grabherr M, Blood PD, Bowden J, et al. *De novo* transcript sequence reconstruction from RNA-seq using the Trinity platform for reference generation and analysis. Nat Protoc. 2013; 8: 1494–1512. doi: 10.1038/nprot.2013.084
5. Peng Y, Leung HCM, Yiu S-M, Lv M-J, Zhu X-G, Chin FYL. IDBA-tran: a more robust de novo de Bruijn graph assembler for transcriptomes with uneven expression levels. Bioinformatics. 2013; 29: i326–i334. doi:10.1093/bioinformatics/btt219
